# Full-Wave Electromagnetic Optimisation of Corrugated Metallic Reflectors Using a Multigrid Approach

**DOI:** 10.1038/s41598-017-18174-4

**Published:** 2018-01-19

**Authors:** Gökhan Karaova, Aşkın Altınoklu, Özgür Ergül

**Affiliations:** Department of Electrical and Electronics Engineering Middle East Technical University, Ankara, Turkey

## Abstract

A multigrid optimisation strategy is introduced to design passive metallic reflectors with corrugated shapes. The strategy is based on using genetic algorithms at multiple grids and shaping the metal sheets, starting from coarse details to fine tunings. This corresponds to a systematic expansion of the related optimisation space, which is explored more efficiently in comparison to a brute-force optimisation without using grid. By employing the multilevel fast multipole algorithm to analyse the electromagnetic problems corresponding to optimisation trials, we obtain accurately designed reflectors that provide focussing abilities with very high performances at single and multiple locations. The designed reflectors are also resistant to fabrication errors with less complex corrugations and simplified reflection mechanisms compared to those found by no-grid optimisation trials.

## Introduction

Electromagnetic design procedures often require finding optimal shapes and topologies that provide the desired electromagnetic characteristics and responses. Unsurprisingly, optimisation tools have become major components in the design of many electronic devices at radio, microwave, THz, and optical frequencies. In the literature, one can find a plethora of applications, including antennas^1–6^, reflecting surfaces^7,8^, frequency-selective surfaces^9,10^, metamaterials^11,12^, optical and photonic components^13–16^, where the structures are designed via optimisation to satisfy the desired absorption, radiation, reflection, scattering, and transmission properties. In some cases, it is possible to transform the original problem into a simplified form, such as a network of lumped elements, which can be easier to optimise at an analytical level^17^. In addition, topological optimisation involving relatively small perturbations can efficiently be handled by using gradient-based tools^3,4,18^, where the kernel solution methods are modified to incorporate the gradient operation on the electromagnetic interactions. On the other side, nature-inspired algorithms, such as particle swarm optimisation methods^19,20^ and genetic algorithms (GAs)^21,22^, provide a great freedom on the fitness functions, including those for multipurpose applications^5,23,24^. These heuristic algorithms can easily be combined externally with electromagnetic solvers, while, as a drawback, they need relatively large numbers of trials for satisfactory optimisation results.

Recently, we presented the optimisation of passive corrugated metallic sheets (reflectors) for desired electromagnetic reflection characteristics^25^. We showed that a GA implementation combined with an iterative fast solver based on the multilevel fast multipole algorithm (MLFMA)^26–28^ can provide effective designs, which have various focussing abilities when illuminated externally. The designed corrugated sheets can be used in diverse applications, including but not limited to optical sensing, communication, imaging, and energy harvesting. However, while we clearly showed their good enhancement properties, the designs were suffering from typical disadvantages of an heuristic optimisation, i.e., slow convergence and attraction to poor local maxima. The issues related to the slow convergence can partially be solved by using MLFMA and its complete integration with the GA module (e.g., via dynamic accuracy control)^6,25^ such that the number of generations can be extended as much as possible. On the other hand, attraction to poor local maxima seems to be a major issue since the desired corrugation applications need great flexibility on the shape of the reflectors, leading to huge optimisation spaces that are very difficult to explore. As shown in^25^, despite relatively good enhancement and focusing performances can be obtained, the designed reflectors typically have wildly oscillating corrugations. Besides the difficulty in the realisation of these structures, they are also observed to be sensitive to fabrication errors.

In this contribution, we introduce a multigrid optimisation strategy, which is well suited for the design of corrugated metallic sheets. We employ grids at different levels such that the reflectors are designed progressively from coarse details to fine details. From the optimisation point of view, this corresponds to expanding the optimisation space as the grid is refined. Hence, the final optimisation space involving extremely many possibilities representing all arbitrary movements of the discretisation nodes can effectively be explored. While, as in all heuristic algorithms, the developed implementation does not guarantee a globally optimal solution, we show that it provides the designs of much better reflectors with improved focusing abilities, in comparison to those obtained without using grid. In addition to better performances, the designed reflectors are also more suitable for fabrication to be used in the applications aforementioned above.

## Multigrid Optimisation

**Figure 1:**
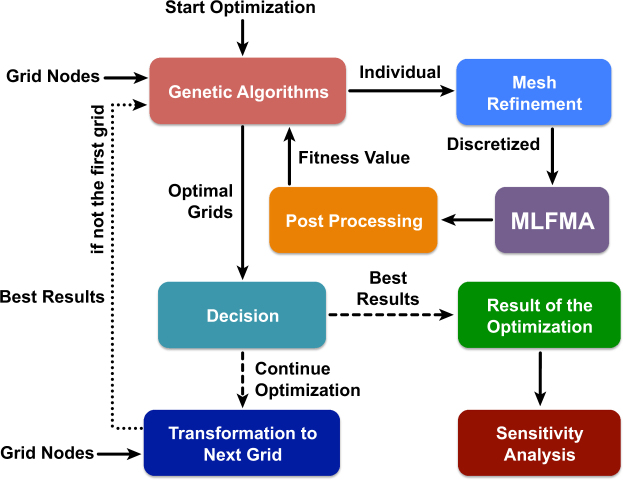
The designed multigrid optimisation mechanism. The dashed lines indicate optional paths based on the decision.

The proposed multigrid optimisation mechanism for metallic reflectors is depicted in Fig. 1. Our aim is to deform a metallic surface such that it behaves as an effective reflector that focusses the power at desired location(s) when illuminated by an external wave. We generally start with an initially flat surface located on the *x* − *y* plane (*z* = 0). Then, the deformations are allowed in a *z* ∈ [−*l*, *l*] range, leading to reflectors with maximum 2*l* profile thicknesses. We consider multigrid optimisation involving *G* grids, where *g* = 1, 2, 3, …, *G* are applied consecutively. The major steps of the mechanism for a grid *g* are as follows.

The grid nodes are given to the GA implementation, which has been developed for alternative optimisation purposes in diverse applications^29^. The chromosome length for each individual is *bN*_*g*_, where *b* is the number of bits to encode the position of a grid node and *N*_*g*_ is the number of grid nodes. The given profile range [−*l*,*l*] for the reflectors is divided into 2^*b*^ − 1 equal intervals. We note that the optimisation space involves ${2}^{b{N}_{g}}$ candidate solutions. Among these, a total of 2^*b*^ flat surfaces also exist, e.g., [000…000] represents a flat surface at *z* = −*l* and [111…111] represents a flat surface at *z* = *l*.

The GA implementation works on a number of individuals, each representing a candidate solution. For each individual, the fitness value is calculated via an electromagnetic simulation using MLFMA. For accurate numerical solutions, adaptive mesh refinement is applied on the deformed grids such that all discretisation triangles (specifically their edges) are smaller than a given threshold. The electromagnetic problems are formulated with the electric-field integral equation and discretized with the Rao-Wilton-Glisson functions on triangles^30^. A standard MLFMA is used to solve the electromagnetic scattering problems. Despite the number of unknowns can be relatively small (4000–15,000 for the examples in this paper), we prefer MLFMA since it allows for dynamic accuracy control (changing the solution accuracy as the optimisation steps progress^31^), leading to overall faster solutions in comparison to the direct method of moments (MOM) and matrix factorisation. We note that the MOM matrices for different individuals are mostly different from each other, making it difficult to reuse any matrix factorisation.

Once the expansion coefficients are found for the induced current density on a reflector represented by an individual, a post processing is applied to evaluate the value of the corresponding fitness function. For the applications in this paper, the fitness is the power density at the given optimisation location or its mean value if multiple locations are considered. The fitness value is used by the GA implementation to evaluate the success of the individual in comparison to others.

Optimisation on a grid continues for a given number of generations, followed by the selection of a number of best individuals. If it is decided to continue the main optimisation (can be decided based on the convergence characteristics or using the criteria *g* < *G* if *G* is fixed), nodal representations of these successful individuals are generated for the fine grid (*g* + 1). In this transformation, the location of a new node in the fine grid is found by interpolating the positions of the related nodes in the coarse grid. To make the interpolations easier, we generally refine a grid by dividing each triangle into four subtriangles. Then, any new node in the fine grid is located on a line between two nodes of the coarse grid. Transformation into a fine grid leads to new chromosomes with *bN*_*g*+1_ > *bN*_*g*_ bits, while the optimisation space is enlarged to ${2}^{b{N}_{g+1}} > {2}^{b{N}_{g}}$ possible solutions. At the same time, since successful individuals are selected from the previous grid, the starting positions are much better than those obtained by randomly sampling the new optimisation space.

After the optimisation for the last grid (*G*) is completed, the most successful individuals (reflector geometries) are selected and further exposed to sensitivity analysis, such as the one shown in this paper. The best reflector is selected as the optimisation result.

In order to show the effectiveness of the multigrid approach, we compare the results with those obtained via no-grid optimisation trials, where the discretisation nodes are directly used to deform the surfaces^25^.

## Results

As numerical examples, we consider metallic reflectors that are designed for focusing the electromagnetic power at single or multiple locations. Initially, a flat 4*λ* × 4*λ* metallic sheet (where *λ* is the wavelength) is located in free space on the *x*-*y* plane centred at the origin (*x*, *y*, *z*) = (0, 0, 0). The excitation is a 1 V/m plane wave propagating in the −*z* direction, i.e., with normal incidence on the original flat surface. To design reflectors, deformations are applied by shifting the grid nodes vertically in the [−0.2*λ*, 0.2*λ*] range, leading to a maximum profile thickness of 0.4*λ*. In the optimisation trials, pools of 40 individuals are used for 250 generations. Each grid node is represented by 10 bits. The multigrid approach is employed by using 2 × 2, 4 × 4, 8 × 8, 16 × 16, and 32 × 32 grids (50 generations for each grid *g*, where 1 ≤ *g* ≤ 5). We note that these numbers (2, 4, 8, 16, 32) indicate the number of intervals at each edge of the sheet, i.e., the number of grid nodes per edge is 3, 5, 9, 17, and 33, respectively, considering the four vertices of the sheet. Hence, the total number of grid nodes are 3^2^ = 9, 5^2^ = 25, 9^2^ = 81, 17^2^ = 289, and 33^2^ = 1089, respectively. For MLFMA solutions, *λ*/10 threshold is used, i.e., for each reflector shape represented by an individual (using any grid), adaptive mesh refinement is applied until all discretisation triangles are smaller than *λ*/10. The multigrid approach is compared to no-grid optimisation trials (with the same pool size and 250 generations), where a total of 4225 discretisation nodes are directly shifted. Mesh refinement is also applied, where necessary, for the MLFMA solutions of a no-grid optimisation.

**Figure 2:**
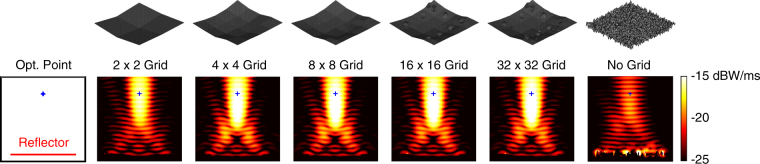
Optimisation results when the power density is maximised at (*x*, *y*, *z*) = (0, −0.5*λ*, 3.2*λ*) using 4*λ* × 4*λ* reflectors. The best reflectors at the ends of the multigrid optimisation stages are compared to the one obtained by the no-grid optimisation.

We first consider the maximisation of the power density at (*x*, *y*, *z*) = (0, −0.5*λ*, 3.2*λ*). Figure 2 depicts the power density (in dBW/m^2^) in the vicinity of the best reflectors at the ends of the multigrid optimisation stages, as well as the final result of the corresponding no-grid optimisation. In the plots (and also in the plots of the following examples), the power density is shown at the *y *= −0.5*λ* plane in a 5*λ* × 5*λ* frame with respect to *x* (horizontal) and *z* (vertical) locations. The reflectors are at the bottom, as also shown in an empty plate containing the optimisation point. Hence, we observe the power density distribution mainly in the reflection region. We note that the incident power of the unit plane wave (1 V/m) in free space is 2.65 × 10^−3^ W/m^2^. For each result in Fig. 2, the geometry of the corresponding reflector with the discretisation nodes is also shown. We observe that the power density is significantly increased at the optimisation location even when using the 2 × 2 grid. Then, the focusing becomes stronger as the grid is refined. It is remarkable that the general shape of the reflector is already formed when using the 8 × 8 grid. Specifically, for maximising the power at a symmetrically located position with 3.2*λ* distance from the reflector, the optimal geometry seems to be a concave shape. We note that this shape is reached by the GA implementation, without any external intervention. Using 16 × 16 and 32 × 32 grids (the last 100 generations) brings some fine tunings, despite the continuing freedom of the shifts in the [−0.2*λ*, 0.2*λ*] range. For comparisons, the rightmost plots depict the best reflector obtained via a no-grid optimisation and the corresponding reflector geometry. We observe that the design provided by the no-grid optimisation has a very complex shape, while its focusing ability is relatively poor in comparison to the result of the multigrid optimisation.

**Figure 3:**
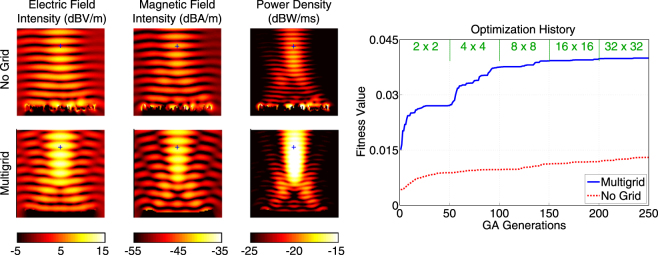
More detailed optimisation results when the power density is maximised at (*x*, *y*, *z*) = (0,−0.5*λ*, 3.2*λ*) using 4*λ* × 4*λ* reflectors. The best results obtained with multigrid and no-grid optimisation trials are depicted. The optimisation histories, i.e., the fitness values with respect to GA generations, are also shown. The fitness is defined as the power density values at the optimisation point.

Figure 3 presents a more detailed comparison of the multigrid and no-grid optimisation results for the single-point optimisation trials in Fig. 2. In addition to the power density distributions, the electric field intensity (in dBV/m) and the magnetic field intensity (in dBA/m) are plotted. The superiority of the reflector found by the multigrid optimisation is also visible with larger peak values in the field plots. As more quantitive data, Fig. 3 also contains the optimisation histories, i.e., the fitness values (power density values at the optimisation location) with respect to the GA generations. We observe that the multigrid approach leads to a fitness value of 2.71 × 10^−2^ W/m^2^ at the 50th generation using 2 × 2 grid. Then, the upgrade to the 4 × 4 grid boosts the value up to 3.75 × 10^−2^ W/m^2^. While each refinement of the grid improves the fitness, we also observe slower progress, especially after the 100th generation. The fitness value reaches 4.00 × 10^−2^ W/m^2^ at the end of the multigrid optimisation (250th generation). This corresponds to more than 15 times enhancement (with respect to the incident power). Without using grid, however, the power density is increased from 4.26 × 10^−3^ W/m^2^ to 1.30 × 10^−2^ W/m, corresponding to less than 5 times enhancement (with respect to the incident power), in 250 generations (with the same computational cost).

**Figure 4:**
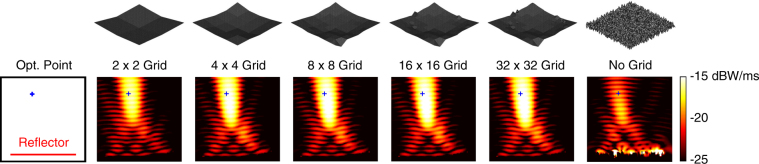
Optimisation results when the power density is maximised at (*x*, *y*, *z*) = (−0.5*λ*, −0.5*λ*, 3.2*λ*) using 4*λ* × 4*λ* reflectors. The best reflectors at the ends of the multigrid optimisation stages are compared to the one obtained by the no-grid optimisation.

**Figure 5:**
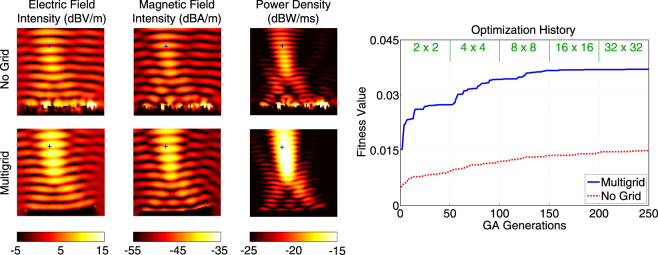
More detailed optimisation results when the power density is maximised at (*x*, *y*, *z*) = (−0.5*λ*,−0.5*λ*, 3.2*λ*) using 4*λ* × 4*λ* reflectors. The best results obtained with multigrid and no-grid optimisation trials are depicted. The optimisation histories, i.e., the fitness values with respect to GA generations, are also shown. The fitness is defined as the power density values at the optimisation point.

Next, we consider the maximisation of the power density at (*x*, *y*, *z*) = (−0.5*λ*,−0.5*λ*, 3.2*λ*). For this asymmetric case, we expect some drop in the performances of the reflectors, in comparison to the symmetric case (location) considered in Figs 2 and 3. Figure 4 presents the power density in the vicinity of the reflectors (similar to Fig. 2). In addition, we compare the electric and magnetic field intensity distributions in Fig. 5, which also contains the optimisation histories. Using the multigrid approach, we again observe a very good kick off with the 2 × 2 grid and significant improvements as the grid is refined to 4 × 4 and 8 × 8, followed by fine tunings with the 16 × 16 and 32 × 32 grids. A close investigation of the reflector geometries in Fig. 5 reveals that an asymmetric concave shape is formed to maximise the power density at the desired location. The final value of the fitness at the 250th generation is 3.70 × 10^−2^ W/m^2^, corresponding to approximately 14 times enhancement (with respect to the incident power). On the other hand, without using grid, the power density can be increased to 1.49 × 10^−2^ W/m^2^, corresponding to less than 6 times enhancement (with respect to the incident power), while the geometry of the reflector is again more complex.

**Figure 6:**
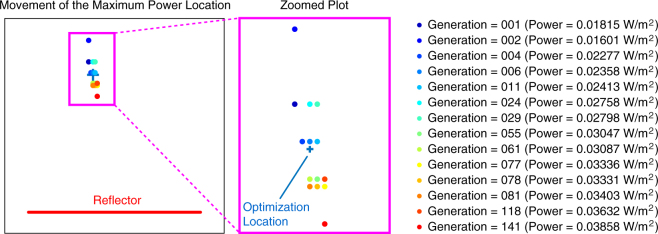
The movement of the maximum power location and the corresponding power density values for the multigrid optimisation in Figs 4 and 5.

Figure 6 presents the movement of the maximum power location for the multigrid optimisation presented in Figs 4 and 5. The circular points in both the overall and zoomed plots represent the location at which the power density is maximum for different critical generations (generations when the fitness value changes). The corresponding power density values are also listed. We note that the purpose of the optimisation is the maximise the power at the target location (shown with the cross sign), while there is no control over where the actual maximum occurs. We observe that the maximum power location is moving at around the optimisation point, while it finally reaches to a location at the bottom of the zoomed plot. The power density at this location is 3.86 × 10^−2^ W/m^2^, which is slightly larger than the power density at the optimisation location (3.70 × 10^−2^ W/m^2^).

Single-point optimisation trials can be considered to be relatively easy to achieve. In fact, the optimisation results converge into concave type reflectors, which are among the most fundamental geometries of optics. We note that, due to the relatively small sizes and profiles of the reflectors (4*λ* × 4*λ* × 0.4*λ*), many analytical models or approximations, such as ray optics, are still inapplicable for the design of these reflectors. On the other hand, a concave shape may be enforced in a parametric study, where the reflector is designed by trial and error, at the cost of the increased uncertainty in the optimality of the shapes. Therefore, to clearly demonstrate the effectiveness of the developed multigrid approach, we now focus on multipoint optimisation trials, where the power density is simultaneously maximised at multiple locations. It is remarkable that such an optimisation does not bring any complexity or extra computational cost when using the developed optimisation procedures. Only the fitness function is changed to the mean value of the power density at the desired locations. Once again, we clearly present the advantages of the multigrid strategy for efficient optimisation.

**Figure 7:**
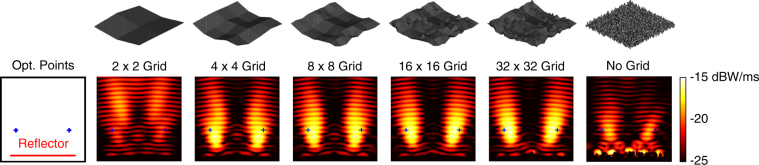
Optimisation results when the power density is maximised simultaneously at (*x*, *y*, *z*) = (−1.25*λ*,−0.5*λ*, 1.5*λ*) and (*x*, *y*, *z*) = (1.25*λ*,−0.5*λ*, 1.5*λ*) using 4*λ* × 4*λ* reflectors. The best reflectors at the ends of the multigrid optimisation stages are compared to the one obtained by the no-grid optimisation.

**Figure 8:**
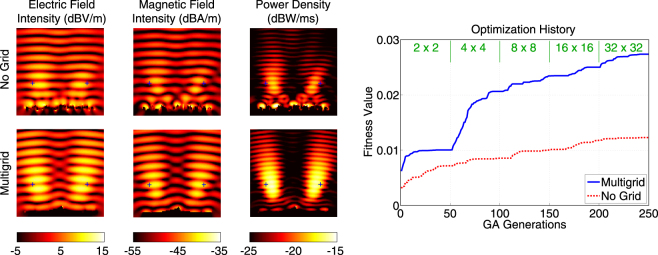
More detailed optimisation results when the power density is maximised simultaneously at (*x*, *y*, *z*) = (−1.25*λ*,−0.5*λ*, 1.5*λ*) and (*x*, *y*, *z*) = (1.25*λ*,−0.5*λ*, 1.5*λ*) using 4*λ* × 4*λ* reflectors. The best results obtained with multigrid and no-grid optimisation trials are depicted. The optimisation histories, i.e., the fitness values with respect to GA generations, are also shown. The fitness is defined as the mean of the power density values at the optimisation point.

Figures 7 and 8 present the optimisation results when the power density is maximised simultaneously at (*x*, *y*, *z*) = (−1.25*λ*,−0.5*λ*, 1.5*λ*) and (*x*, *y*, *z*) = (1.25*λ*,−0.5*λ*, 1.5*λ*). Investigating the power density results in Fig. 7, we observe that the 2 × 2 grid does not provide good focusing characteristics, as opposed to the single-point optimisation trials. Specifically, the power density cannot be maximised at the given locations by controlling only 3 × 3 = 9 grid nodes, as expected. A remarkable improvement occurs when the grid is refined to 4 × 4 (25 grid nodes), while the power enhancement becomes increasingly better with more refinements. Effective usage of the finer grids is obvious also in the geometry plots. For example, using the 16 × 16 grid does not bring just fine tunings over the 8 × 8 grid; significantly large new corrugations are observed. As also shown in Fig. 8, the multigrid approach leads to a very successful power distribution focused at the desired locations. We note that the optimisation target is to increase the mean value of the power density, i.e., we do not suppress the power density at other locations. The fitness values with respect to the GA generations in Fig. 8 clearly show the step-by-step improvement as the grid is refined. At the 25th generation, the fitness value reaches 2.74 × 10^−2^ W/m^2^, which indicates more than 10 times average enhancement (with respect to the incident power). The corresponding value obtain with a no-grid optimisation is only 1.23 × 10^−2^ W/m^2^ (less than 5 times enhancement with respect to the incident power).

**Figure 9:**
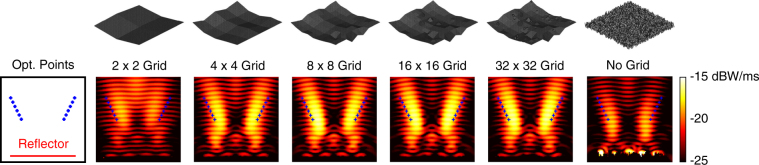
Optimisation results when the power density is maximised simultaneously at 12 different locations using 4*λ* × 4*λ* reflectors. The best reflectors at the ends of the multigrid optimisation stages are compared to the one obtained by the no-grid optimisation.

**Figure 10:**
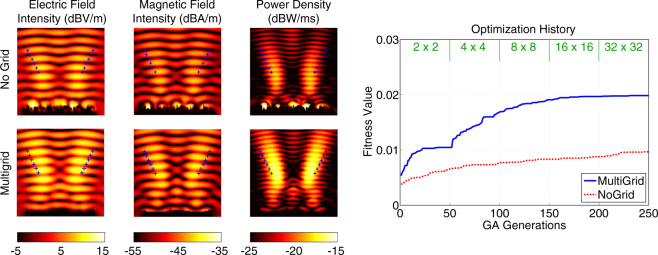
More detailed optimisation results when the power density is maximised simultaneously at 12 different locations using 4*λ* × 4*λ* reflectors. The best results obtained with multigrid and no-grid optimisation trials are depicted. The optimisation histories, i.e., the fitness values with respect to GA generations, are also shown. The fitness is defined as the mean of the power density values at the optimisation point.

Figures 9 and 10 present the results of the optimisation trials when the power density is maximised simultaneously at 12 points that are arranged as two symmetrically located inclined lines. As shown in Fig. 9, the power density is maximised at the desired locations when the grid is refined to 4 × 4 and 8 × 8. In this case, 16 × 16 and 32 × 32 grids seem to provide only fine tunings on the reflector, while this could not be known before using them. Once again, the final reflector design provided by the multigrid approach performs much better than the one obtained from a no-grid optimisation. As shown in Fig. 10, the fitness value can be increased successfully to 2.01 × 10^−2^ W/m^2^ (more than 7 times simultaneous enhancement) using the multigrid approach, while it reaches only 9.66 × 10^−3^ W/m^2^ (50% less) with the no-grid optimisation.

All simulations presented in this paper are performed in the MATLAB environment on cores of E5-2680v3 processors. The solution time strongly depends on the reflector geometry (mainly due to different numbers of iterations). For the results described above, it takes 200–600 seconds to complete a single MLFMA solution (when using full MLFMA without introducing any approximation due to the dynamic accuracy control). These durations, more or less, correspond to the processing time per generation since we solve all individuals in a generation simultaneously via embarrassing parallelisation. Hence, without using dynamic accuracy control, lookup table, and similar acceleration techniques, an optimisation requires less than two days. Depending on the optimisation problem and using the listed acceleration techniques, this time can be reduced to less than 24 hours. Since the number of unknowns is relatively small (4000–15,000 for the examples in this paper), the required memory for the simulations is also small, i.e., the peak is usually at around 500 MB.

Finally, Table 1 provides more quantitative data on all simulations presented above. We list the fitness values, i.e., the power density values at the optimisation location or the mean of the power density values when multiple locations are involved. For the multigrid trials, the obtained values at the end of each grid (50th, 100th, 150th, and 200th generations) are provided in addition to the final result (250th generation). The superiority of the multigrid approach in comparison to the no-grid approach (the last column) is clearly visible.

**Table 1:** Summary of the optimisation trials depicted in Figs 2–10.

Results	Multigrid Optimisation					No Grid
	50th Gen.	100th Gen.	150th Gen.	200th Gen.	250th Gen.	
Figs 2 and 3	2.71 × 10^−2^	3.75 × 10^−2^	3.93 × 10^−2^	3.98 × 10^−2^	4.00 × 10^−2^	1.30 × 10^−2^
Figs 4, 5, and 6	2.74 × 10^−2^	3.43 × 10^−2^	3.67 × 10^−2^	3.69 × 10^−2^	3.70 × 10^−2^	1.49 × 10^−2^
Figs 7 and 8	1.00 × 10^−2^	2.06 × 10^−2^	2.35 × 10^−2^	2.57 × 10^−2^	2.74 × 10^−2^	1.23 × 10^−2^
Figs 9 and 10	1.05 × 10^−2^	1.69 × 10^−2^	1.91 × 10^−2^	1.97 × 10^−2^	2.01 × 10^−2^	0.966 × 10^−2^

The fitness values (the power density values at the optimisation location or the mean of the power density values at the optimisation locations) are listed when the multigrid approach is used, in contrast to the no-grid case. For the multigrid approach, the obtained values at the end of each grid (50th, 100th, 150th, and 200th generations, in addition to the 250th generation) are also provided. The unit of the fitness value is W/m^2^. For comparisons, we recall that the incident power density is 0.265 × 10^−2^ W/m^2^.

For future computational and experimental studies, all reflector geometries found by the multigrid approach and presented above are available as text data in Supplementary Files.

## Discussion

As shown in the results, the proposed multigrid approach provides more successful optimisation of metallic reflectors, in comparison to the brute force approach using the discretisation nodes directly. All results involve 4*λ* × 4*λ* metallic sheets, while we have also practiced the same level of performances for other similar sizes. We note that the reflectors are relatively small, e.g., they are approximately 6 × 6 *μ*m at 200 THz (a common infrared frequency). The small sizes of the reflectors with respect to wavelength make it challenging to control the reflections to generate focus points. This increases the importance of the optimisation results, especially employing full-wave solutions without resorting to approximate methods.

The multigrid optimisation strategy not only provides better results but also leads to reflector geometries that are potentially easier to fabricate. We emphasise that the geometries found by no-grid optimisation trials are not merely noisy surfaces, i.e., they still provide remarkable enhancements at desired locations. On the other hand, they are difficult to realise in practice since they have complicated shapes and they are more sensitive to the fabrication errors since their focusing characteristics are based on complex interactions between widely oscillating corrugations. Using the multigrid approach to design a reflector, the main reflection mechanism is constructed by large surfaces, which are progressively reshaped and improved as the grid is refined. If the finer grids provide limited improvement on the focusing characteristics, they can also be discarded to simplify the fabrication procedures.

**Figure 11:**
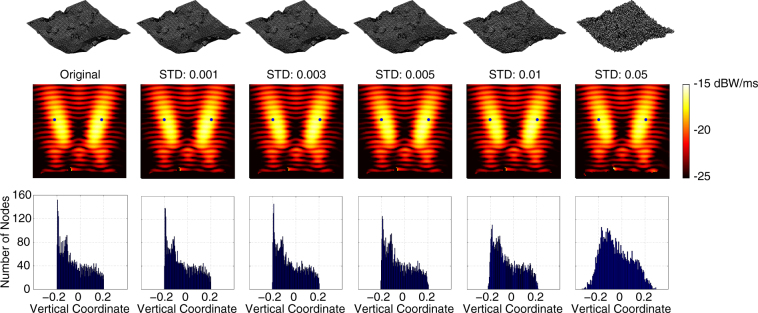
Sensitivity analysis on the result of a multigrid optimisation to maximise the power density simultaneously at two locations. Random vertical movements for the discretisation nodes are introduced.

The reflector geometries found by the multigrid optimisation strategy are typically resistant to fabrication errors. As an example, Fig. 11 presents the optimisation results when the power density is focused simultaneously at two separate points. After the optimal shape is found via the multigrid approach, the discretisation nodes are randomly moved vertically to simulate fabrication errors. In addition to the power density distributions and the corresponding shapes of the reflectors, histograms for the vertical positions of the discretisation nodes are also depicted. For the movements, Gaussian distributions with zero median and different standard deviations (STD) from 0.001 to 0.05 are applied. We observe that the reflector shape is significantly degenerated as STD increases. On the other hand, the performance of the reflector is very stable, maintaining the two focus points even for very noisy cases. A further investigation shows that, for these cases, the current density distributions on the reflector surfaces remain quite similar when the noises are introduced.

From the optimisation point of view, the multigrid approach can be seen as systematically expanding optimisation spaces, where potentially good regions are estimated via coarser scans and they are investigated in detail via finer searches. We note that the solutions found by a multigrid optimisation are already in the optimisation space of a no-grid optimisation (omitting minor differences due to the transverse locations of the nodes). Specifically, an ideal optimisation without using a grid should reach the optimal geometries (or similarly performing ones) found by the corresponding multigrid optimisation. Unfortunately, the optimisation spaces in these applications are so large that it becomes extremely difficult to approach a global solution. From this perspective, a multigrid optimisation also does not guarantee convergence to globally best geometries. On the other hand, it provides more reliable geometries that definitely perform better than those obtained via no-grid optimisation trials. Besides, concave geometries found for the single-point optimisation trials (despite that the optimisation trials are not guided externally to specific shapes or attributes like symmetry) demonstrate the reliability of the multigrid approach.

An important parameter in a multigrid optimisation is the number of grids. Basically, it is reasonable to start with a grid as coarse as possible. In the results of this paper, we use 2 × 2 grids (3 × 3 grid nodes), i.e., the coarsest possible grid to deform a sheet at all four edges. In some cases, such a grid already provides relatively successful results, while it performs poorly for others, especially involving multiple optimisation locations. In general, it is not known (or difficult to know) how many grid refinements should be applied in a multigrid optimisation. In the results of this paper, we use fixed *G* = 5 grids based on our experience with this type of reflectors. An automated approach can be based on investigating the convergence characteristics, while this may not be trivial. As also evident in the results, a convergence of the fitness using a grid does not indicate an overall convergence, i.e., an upgrade to a finer grid may boost the fitness. Then, the criteria can be the amount of the jump when a grid is refined. Among the results of this paper, only the two-point optimisation in Figs 7 and 8 demonstrates an improvement when the grid is refined to 32 × 32. This may suggest that a finer grid could also be tried for this case. For fair comparisons, however, we make all optimisation trials involve 50 generations per grid and maximum 250 generations, while an automated mechanism to determine the number of grids and the number of generations per grid is under investigation.

## Methods

In the numerical solutions of the scattering problems, a conventional MLFMA based on the expansion of far-zone interactions via plane waves^28^ is used. For dynamic accuracy control in an optimisation^31^, approximate versions of MLFMA (AMLFMA) using reduced number of harmonics are used. In the full version, all electromagnetic interactions are computed with maximum 1% error. Iterative solutions are performed by using the generalised minimal residual (GMRES) method or its flexible version (FGMRES). Convergence threshold for the iterations is set to 0.001. Since the metallic reflectors are modelled as perfect electric conductors with zero thicknesses, EFIE is used as the formulation in the frequency domain. The RWG functions are used for the discretisation since the considered problems are not affected from a low-frequency breakdown. At the same time, preconditioning is applied, where necessary, via multilayer solutions^32^.

EFIE and MLFMA are very well-known in the literature; but, for the completeness of the paper, we briefly present the formulation. Electromagnetic problems are considered in the frequency domain using the $\exp (\,-\,i\omega t)$ time convention. When discretized with the RWG functions, EFIE leads to matrix equations as

1 $${\bar{Z}}^{{\rm{EFIE}}}\cdot a={w}^{{\rm{EFIE}}},$$

where *a* represents current coefficients (to be found), while the matrix elements and the elements of the right-hand-side vector are derived as

2 $${\bar{Z}}^{{\rm{E}}{\rm{F}}{\rm{I}}{\rm{E}}}[m,n]=i\omega {\mu }_{0}{\int }_{{S}_{m}}dr{t}_{m}(r)\cdot {\int }_{{S}_{n}}dr^{\prime} {b}_{n}(r^{\prime} ){g}_{0}(r,r^{\prime} )+\frac{1}{i\omega {\varepsilon }_{0}}{\int }_{{S}_{m}}dr{\rm{\nabla }}\cdot {t}_{m}(r){\int }_{{S}_{n}}dr^{\prime} {g}_{0}(r,r^{\prime} ){\rm{\nabla }}^{\prime} \cdot {b}_{n}(r^{\prime} )$$

3 $${w}^{{\rm{EFIE}}}[m]=-{\int }_{{S}_{m}}dr{t}_{m}(r)\cdot {E}^{{\rm{inc}}}(r\mathrm{)}.$$

In the above, *E*^inc^ is the incident electric field intensity created by external sources (plane waves in this paper), *ε*_0_ and *μ*_0_ are the permittivity and permeability of the host medium (free space in this paper), ${\eta }_{0}=\sqrt{({\mu }_{0}/{\varepsilon }_{0})}$ is the intrinsic impedance, ${k}_{0}=2\pi /{\lambda }_{0}=\omega \sqrt{\,{\mu }_{0}{\varepsilon }_{0}}$ is the wavenumber, and ${g}_{0}(r,r^{\prime} )=\exp (i{k}_{0}|r-r^{\prime} |)/\mathrm{(4}\pi |r-r^{\prime} |)$ is the three-dimensional Green’s function. In addition, *b*_*n*_ and *t*_*m*_ represent the basis and testing functions for *n* = 1, 2, …, *N* and *m* = 1, 2, …, *N*, where *N* is the number of the RWG functions. When MLFMA is used, the Green’s function is expanded as^26–28^

4 $${g}_{0}(r,r^{\prime} )=\frac{i{k}_{0}}{{\mathrm{(4}\pi )}^{2}}\int {d}^{2}\hat{k}\,\exp \,(i{k}_{0}\hat{k}\cdot ({d}_{1}+{d}_{2})){\alpha }_{\tau }({k}_{0},\hat{k},D)$$

5 $$\{{\rm{\nabla }}{\rm{\nabla }}^{\prime} {g}_{0}\}(r,r^{\prime} )=\frac{i{k}_{0}}{{(4\pi )}^{2}}\int {d}^{2}\hat{k}\,{k}_{0}^{2}(\hat{k}\hat{k})\,\exp \,(i{k}_{0}\hat{k}\cdot ({d}_{1}+{d}_{2})){\alpha }_{\tau }({k}_{0},\hat{k},D),$$

where *α*_*τ*_ represents truncated translation operators (*τ*: truncation number) and *r* − *r*′ = *D* + *d* = *D* + *d*_1_ + *d*_2_ with the condition |*D*| > |*d*|. To perform far-zone interactions on-the-fly, multilevel tree structures are constructed by using cubic boxes. One-box-buffer scheme is used along with the excess bandwidth formula to decide the far-zone rules (translations, number of harmonics, angular sampling, etc). Standard Lagrange interpolation and anterpolation is used between levels.

Once current coefficients are found via the iterative solution of a matrix equation, the total electric and magnetic fields can be obtained at any observation point *r* in the near-zone as

6 $$E(r)={E}^{{\rm{inc}}}(r)+i{k}_{0}{\eta }_{0}\sum _{n=1}^{N}{a}_{n}\int dr^{\prime} {b}_{n}(r^{\prime} ){g}_{0}(r,r^{\prime} )-i\frac{{\eta }_{0}}{{k}_{0}}\sum _{n=1}^{N}{a}_{n}\int dr^{\prime} \nabla ^{\prime} \cdot {b}_{n}(r^{\prime} )\nabla ^{\prime} {g}_{0}(r,r^{\prime} )$$

7 $$H(r)={H}^{{\rm{inc}}}(r)+\sum _{n=1}^{N}{a}_{n}\int dr^{\prime} {b}_{n}(r^{\prime} )\times \nabla ^{\prime} {g}_{0}(r,r^{\prime} ),$$

where *H*^inc^ is the incident magnetic field intensity. Then the power density (the major quantity considered in the optimisation trials of this paper) is computed as

8 $$S(r)=|E(r)\times {H}^{\ast }(r)|,$$

where * is the complex conjugate operation.

The GA implementation used in this paper has been developed for various optimisation studies in different applications^29^. As described in^33^, we use one-to-one crossover, family elitism, and success-based mutations for improved optimisation convergence. For a reflector optimisation considered in this study, similar individuals are rarely considered other than those due to elitism. Therefore, the number of MLFMA simulations is proportional to the number of generations times the pool size. For the presented examples, this corresponds to as large as 250 × 40 = 10,000 simulations per optimisation. Due to relatively small sizes of the problems, MLFMA itself is not parallelized. However, we use an embarrassing parallelisation during an optimisation, i.e., multiple individuals are evaluated simultaneously on multiple CPU cores.

## Electronic supplementary material

Supplementary Information 
