# Publisher Correction: Full-Wave Electromagnetic Optimisation of Corrugated Metallic Reflectors Using a Multigrid Approach

**DOI:** 10.1038/s41598-018-25860-4

**Published:** 2018-05-08

**Authors:** Gökhan Karaova, Aşkın Altınoklu, Özgür Ergül

**Affiliations:** Department of Electrical and Electronics Engineering Middle East Technical University, Ankara, Turkey

Correction to: *Scientific Reports* 10.1038/s41598-017-18174-4, published online 19 January 2018

The original version of this Article contained an error in the title of the paper, where the word “Reflectors” was incorrectly given as “Reectors”. This has now been corrected in the PDF and HTML versions of the Article.

