# Supplementary material for: Full-Wave Electromagnetic Optimisation of Corrugated Metallic Reflectors Using a Multigrid Approach
Source: Sci Rep. 2018 Jan 19;8:1267. doi: 10.1038/s41598-017-18174-4 (PMC5775196; doi:10.1038/s41598-017-18174-4)
Supplement: Supplementary file 1 — Supplementary Information [file 41598_2017_18174_MOESM1_ESM.pdf]

Full-Wave Electromagnetic Optimisation of Corrugated Metallic Reflectors Using a Multigrid Approach

Gökhan Karaova, Aşkın Altınoklu, and Özgür Ergül

Department of Electrical and Electronics Engineering
Middle East Technical University, Ankara, Turkey

ozergul@metu.edu.tr

SUPPLEMENTARY MATERIAL

Design 1: Pages 2-221
Design 2: Pages 222-434
Design 3: Pages 435-691
Design 4: Pages 691-917

DESIGN 1

Node Index	Node Coordinates X(m)	Y(m)	Z(m)
1	0.00000000	0.00000000	0.19921799
2	0.00000000	4.00000000	0.19374389
3	4.00000000	0.00000000	0.15073314
4	4.00000000	4.00000000	0.19882698
5	0.00000000	2.00000000	-0.02639296
6	2.00000000	0.00000000	-0.12844575
7	2.00000000	2.00000000	-0.20000000
8	2.00000000	4.00000000	0.16911046
9	4.00000000	2.00000000	-0.01857283
10	0.00000000	1.00000000	-0.01427175
11	1.00000000	0.00000000	-0.05650049
12	1.00000000	1.00000000	-0.17302053
13	0.00000000	3.00000000	0.18435973
14	1.00000000	4.00000000	0.17145650
15	3.00000000	0.00000000	-0.02326491
16	4.00000000	1.00000000	0.05063539
17	3.00000000	3.00000000	-0.02561095
18	3.00000000	4.00000000	0.20000000
19	4.00000000	3.00000000	0.18240469
20	1.00000000	2.00000000	-0.19100684
21	1.00000000	3.00000000	0.00136852
22	2.00000000	1.00000000	-0.19921799
23	3.00000000	1.00000000	-0.19217986
24	2.00000000	3.00000000	-0.11867058
25	3.00000000	2.00000000	-0.19608993
26	0.00000000	0.50000000	0.08269795
27	0.50000000	0.00000000	0.19530792
28	0.50000000	0.50000000	-0.09990225
29	0.00000000	3.50000000	0.19843597
30	0.50000000	4.00000000	0.19843597
31	3.50000000	0.00000000	0.06549365
32	4.00000000	0.50000000	0.10029326
33	3.50000000	3.50000000	0.18553275
34	3.50000000	4.00000000	0.19921799
35	4.00000000	3.50000000	0.19061584
36	0.00000000	1.50000000	-0.02013685
37	0.00000000	2.50000000	0.02873900
38	0.50000000	2.00000000	-0.10694037
39	0.50000000	2.50000000	0.01270772
40	1.50000000	0.00000000	-0.09247312
41	2.50000000	0.00000000	-0.07565982
42	2.00000000	0.50000000	-0.16363636
43	2.50000000	0.50000000	-0.17341153
44	1.50000000	1.50000000	-0.18631476
45	2.50000000	2.50000000	-0.11358749
46	1.50000000	2.00000000	-0.19530792
47	2.00000000	1.50000000	-0.19960899
48	2.00000000	2.50000000	-0.18435973
49	2.50000000	2.00000000	-0.19804497
50	1.50000000	4.00000000	0.17028348
51	2.50000000	4.00000000	0.19687195
52	1.50000000	3.50000000	0.10420332
53	2.00000000	3.50000000	0.04398827
54	4.00000000	1.50000000	0.01622678
55	4.00000000	2.50000000	0.08191593

56	3.50000000	1.50000000	-0.10537634
57	3.50000000	2.00000000	-0.10733138
58	0.50000000	1.00000000	-0.09364614
59	0.50000000	1.50000000	-0.00254154
60	1.00000000	0.50000000	-0.11476051
61	1.50000000	0.50000000	-0.18396872
62	1.00000000	1.50000000	-0.18201369
63	1.50000000	1.00000000	-0.18592375
64	0.50000000	3.50000000	0.19061584
65	0.50000000	3.00000000	0.01857283
66	1.00000000	3.50000000	0.11241447
67	3.50000000	0.50000000	0.00019550
68	3.00000000	0.50000000	-0.02013685
69	3.50000000	1.00000000	-0.08308895
70	3.00000000	3.50000000	0.07057674
71	3.50000000	3.00000000	-0.03812317
72	2.50000000	3.00000000	-0.19139785
73	3.00000000	2.50000000	-0.12140762
74	2.50000000	3.50000000	0.08973607
75	3.50000000	2.50000000	-0.04750733
76	1.00000000	2.50000000	-0.09481916
77	1.50000000	2.50000000	-0.16637341
78	1.50000000	3.00000000	-0.18435973
79	2.50000000	1.00000000	-0.19569892
80	2.50000000	1.50000000	-0.19765396
81	3.00000000	1.50000000	-0.19413490
82	0.00000000	0.25000000	0.13079179
83	0.25000000	0.00000000	0.12414467
84	0.25000000	0.25000000	0.08973607
85	0.00000000	3.75000000	0.19139785
86	0.25000000	4.00000000	0.19608993
87	3.75000000	0.00000000	0.10811339
88	4.00000000	0.25000000	0.12570870
89	3.75000000	3.75000000	0.19217986
90	3.75000000	4.00000000	0.19921799
91	4.00000000	3.75000000	0.19491691
92	0.00000000	1.75000000	-0.02326491
93	0.00000000	2.25000000	0.00136852
94	0.25000000	2.00000000	-0.06666667
95	0.25000000	2.25000000	-0.00684262
96	1.75000000	0.00000000	-0.10420332
97	2.25000000	0.00000000	-0.10185728
98	2.00000000	0.25000000	-0.14604106
99	2.25000000	0.25000000	-0.15073314
100	1.75000000	1.75000000	-0.19335288
101	2.25000000	2.25000000	-0.15659824
102	1.75000000	2.00000000	-0.19765396
103	2.00000000	1.75000000	-0.20000000
104	2.00000000	2.25000000	-0.19217986
105	2.25000000	2.00000000	-0.19921799
106	1.75000000	4.00000000	0.16989247
107	2.25000000	4.00000000	0.18318671
108	1.75000000	3.75000000	0.13665689
109	2.00000000	3.75000000	0.10654936
110	4.00000000	1.75000000	-0.00136852
111	4.00000000	2.25000000	0.03186706
112	3.75000000	1.75000000	-0.06119257
113	3.75000000	2.00000000	-0.06275660
114	0.00000000	0.75000000	0.03421310

115	0.00000000	1.25000000	-0.01700880
116	0.25000000	1.00000000	-0.05376344
117	0.25000000	1.25000000	-0.05845552
118	0.75000000	0.00000000	0.00645161
119	1.25000000	0.00000000	-0.07448680
120	1.00000000	0.25000000	-0.08543500
121	1.25000000	0.25000000	-0.09208211
122	0.75000000	0.75000000	-0.08660802
123	1.25000000	1.25000000	-0.17966764
124	0.75000000	1.00000000	-0.13352884
125	1.00000000	0.75000000	-0.14369501
126	1.00000000	1.25000000	-0.17771261
127	1.25000000	1.00000000	-0.17966764
128	0.00000000	3.25000000	0.18670577
129	0.00000000	2.75000000	0.10654936
130	0.25000000	3.25000000	0.18123167
131	0.25000000	3.00000000	0.15151515
132	0.75000000	4.00000000	0.18475073
133	1.25000000	4.00000000	0.17106549
134	0.75000000	3.75000000	0.17497556
135	1.00000000	3.75000000	0.13978495
136	3.25000000	0.00000000	0.02130987
137	2.75000000	0.00000000	-0.04946237
138	3.25000000	0.25000000	-0.01114370
139	3.00000000	0.25000000	-0.06549365
140	4.00000000	0.75000000	0.07565982
141	4.00000000	1.25000000	0.03343109
142	3.75000000	0.75000000	0.02600196
143	3.75000000	1.00000000	-0.00997067
144	3.25000000	3.25000000	0.07996090
145	2.75000000	2.75000000	-0.06940371
146	3.00000000	3.25000000	0.03030303
147	3.25000000	3.00000000	0.05415445
148	2.75000000	3.00000000	-0.05806452
149	3.00000000	2.75000000	-0.07370479
150	3.25000000	4.00000000	0.19960899
151	2.75000000	4.00000000	0.19843597
152	3.00000000	3.75000000	0.14330401
153	2.75000000	3.75000000	0.13900293
154	4.00000000	3.25000000	0.18670577
155	4.00000000	2.75000000	0.13196481
156	3.75000000	3.00000000	0.15816227
157	3.75000000	2.75000000	0.07292278
158	0.75000000	2.00000000	-0.14877810
159	1.25000000	2.00000000	-0.19335288
160	0.75000000	1.75000000	-0.14682307
161	1.00000000	1.75000000	-0.18631476
162	1.00000000	2.25000000	-0.14291300
163	1.25000000	2.25000000	-0.17302053
164	0.75000000	2.75000000	0.00723363
165	1.25000000	3.25000000	0.05298143
166	0.75000000	3.00000000	0.06001955
167	1.00000000	3.25000000	0.05493646
168	1.00000000	2.75000000	-0.04672532
169	1.25000000	3.00000000	-0.01583578
170	2.00000000	0.75000000	-0.18123167
171	2.00000000	1.25000000	-0.19921799
172	1.75000000	0.75000000	-0.16324536
173	1.75000000	1.00000000	-0.19257087

174	2.25000000	1.00000000	-0.19726295
175	2.25000000	1.25000000	-0.19843597
176	2.75000000	0.75000000	-0.18279570
177	3.25000000	1.25000000	-0.14877810
178	3.00000000	0.75000000	-0.14995112
179	3.25000000	1.00000000	-0.13157380
180	2.75000000	1.00000000	-0.19413490
181	3.00000000	1.25000000	-0.19335288
182	2.00000000	2.75000000	-0.15151515
183	2.00000000	3.25000000	-0.03734115
184	2.25000000	3.00000000	-0.10459433
185	2.25000000	3.25000000	-0.02013685
186	1.75000000	2.75000000	-0.13665689
187	1.75000000	3.00000000	-0.07605083
188	2.75000000	2.00000000	-0.19726295
189	3.25000000	2.00000000	-0.15190616
190	3.00000000	2.25000000	-0.15894428
191	3.25000000	2.25000000	-0.11632454
192	2.75000000	1.75000000	-0.19687195
193	3.00000000	1.75000000	-0.19530792
194	0.25000000	0.50000000	0.04125122
195	0.25000000	0.75000000	-0.00566960
196	0.50000000	0.25000000	0.03460411
197	0.75000000	0.25000000	-0.02248289
198	0.50000000	0.75000000	-0.04672532
199	0.75000000	0.50000000	-0.05728250
200	0.25000000	3.75000000	0.19374389
201	0.25000000	3.50000000	0.18357771
202	0.50000000	3.75000000	0.18826979
203	3.75000000	0.25000000	0.08269795
204	3.50000000	0.25000000	0.03343109
205	3.75000000	0.50000000	0.05063539
206	3.50000000	3.75000000	0.19257087
207	3.75000000	3.50000000	0.18787879
208	3.25000000	3.50000000	0.13587488
209	3.50000000	3.25000000	0.15972630
210	3.25000000	3.75000000	0.14291300
211	3.75000000	3.25000000	0.16246334
212	0.25000000	1.75000000	-0.06353861
213	0.25000000	1.50000000	-0.06158358
214	0.25000000	2.50000000	0.02091887
215	0.25000000	2.75000000	0.07370479
216	0.50000000	2.25000000	-0.04711632
217	0.50000000	1.75000000	-0.10459433
218	0.75000000	2.25000000	-0.10068426
219	0.50000000	2.75000000	0.06588465
220	0.75000000	2.50000000	-0.04086022
221	1.75000000	0.25000000	-0.12805474
222	1.50000000	0.25000000	-0.11006843
223	2.50000000	0.25000000	-0.12453568
224	2.75000000	0.25000000	-0.09169110
225	2.25000000	0.50000000	-0.16832845
226	1.75000000	0.50000000	-0.14565005
227	2.25000000	0.75000000	-0.17966764
228	2.75000000	0.50000000	-0.14056696
229	2.50000000	0.75000000	-0.18475073
230	1.50000000	1.75000000	-0.19100684
231	1.75000000	1.50000000	-0.19296188
232	1.25000000	1.50000000	-0.18435973

233	1.50000000	1.25000000	-0.18631476
234	2.25000000	2.50000000	-0.14877810
235	2.50000000	2.25000000	-0.15581623
236	2.50000000	2.75000000	-0.10224829
237	2.75000000	2.50000000	-0.11788856
238	1.75000000	2.25000000	-0.18983382
239	1.25000000	1.75000000	-0.18866080
240	1.50000000	2.25000000	-0.17497556
241	2.25000000	1.75000000	-0.19882698
242	1.75000000	1.25000000	-0.19296188
243	2.25000000	1.50000000	-0.19843597
244	2.25000000	2.75000000	-0.13782991
245	1.75000000	2.50000000	-0.16950147
246	2.75000000	2.25000000	-0.16011730
247	2.50000000	1.75000000	-0.19804497
248	1.50000000	3.75000000	0.13743891
249	1.25000000	3.75000000	0.13939394
250	2.25000000	3.75000000	0.12062561
251	2.50000000	3.75000000	0.13743891
252	1.75000000	3.50000000	0.07409580
253	1.25000000	3.50000000	0.10615836
254	1.50000000	3.25000000	0.03538612
255	2.25000000	3.50000000	0.06119257
256	1.75000000	3.25000000	0.00527859
257	3.75000000	1.50000000	-0.04437928
258	3.75000000	1.25000000	-0.02717498
259	3.75000000	2.25000000	-0.01270772
260	3.75000000	2.50000000	0.02287390
261	3.50000000	1.75000000	-0.10615836
262	3.50000000	1.25000000	-0.08778104
263	3.25000000	1.50000000	-0.14995112
264	3.50000000	2.25000000	-0.07174976
265	3.25000000	1.75000000	-0.15073314
266	0.50000000	1.25000000	-0.09794721
267	0.75000000	1.25000000	-0.13782991
268	0.75000000	1.50000000	-0.14213099
269	1.25000000	0.50000000	-0.12101662
270	1.25000000	0.75000000	-0.15034213
271	1.50000000	0.75000000	-0.15659824
272	0.50000000	3.25000000	0.14838710
273	0.75000000	3.50000000	0.14330401
274	0.75000000	3.25000000	0.11319648
275	3.25000000	0.50000000	-0.05337243
276	3.50000000	0.75000000	-0.03460411
277	3.25000000	0.75000000	-0.08895406
278	2.75000000	3.25000000	-0.00215054
279	2.75000000	3.50000000	0.08191593
280	3.25000000	2.75000000	0.00606061
281	3.50000000	2.75000000	0.04868035
282	2.50000000	3.25000000	-0.00645161
283	3.25000000	2.50000000	-0.07917889
284	1.25000000	2.50000000	-0.12492669
285	1.25000000	2.75000000	-0.06392962
286	1.50000000	2.75000000	-0.09403715
287	2.50000000	1.25000000	-0.19648094
288	2.75000000	1.25000000	-0.19491691
289	2.75000000	1.50000000	-0.19608993
290	0.00000000	0.12500000	0.18944282
291	0.12500000	0.00000000	0.18475073

292	0.12500000	0.12500000	0.11671554
293	0.00000000	3.87500000	0.19257087
294	0.12500000	4.00000000	0.19491691
295	3.87500000	0.00000000	0.12961877
296	4.00000000	0.12500000	0.13822092
297	3.87500000	3.87500000	0.19569892
298	3.87500000	4.00000000	0.19921799
299	4.00000000	3.87500000	0.19687195
300	0.00000000	1.87500000	-0.02482893
301	0.00000000	2.12500000	-0.01231672
302	0.12500000	2.00000000	-0.04633431
303	0.12500000	2.12500000	-0.01661779
304	1.87500000	0.00000000	-0.11632454
305	2.12500000	0.00000000	-0.11515152
306	2.00000000	0.12500000	-0.13743891
307	2.12500000	0.12500000	-0.13939394
308	1.87500000	1.87500000	-0.19648094
309	2.12500000	2.12500000	-0.17849462
310	1.87500000	2.00000000	-0.19882698
311	2.00000000	1.87500000	-0.20000000
312	2.00000000	2.12500000	-0.19608993
313	2.12500000	2.00000000	-0.19960899
314	1.87500000	4.00000000	0.17262952
315	2.12500000	4.00000000	0.17614858
316	1.87500000	3.87500000	0.15307918
317	2.00000000	3.87500000	0.13782991
318	4.00000000	1.87500000	-0.00997067
319	4.00000000	2.12500000	0.00684262
320	3.87500000	1.87500000	-0.04007820
321	3.87500000	2.00000000	-0.04046921
322	0.00000000	0.87500000	0.00997067
323	0.00000000	1.12500000	-0.01544477
324	0.12500000	1.00000000	-0.03421310
325	0.12500000	1.12500000	-0.03655914
326	0.87500000	0.00000000	-0.02482893
327	1.12500000	0.00000000	-0.06549365
328	1.00000000	0.12500000	-0.07096774
329	1.12500000	0.12500000	-0.07409580
330	0.87500000	0.87500000	-0.12961877
331	1.12500000	1.12500000	-0.17653959
332	0.87500000	1.00000000	-0.15268817
333	1.00000000	0.87500000	-0.15855327
334	1.00000000	1.12500000	-0.17536657
335	1.12500000	1.00000000	-0.17653959
336	0.00000000	3.12500000	0.18553275
337	0.00000000	2.87500000	0.14565005
338	0.12500000	3.12500000	0.18279570
339	0.12500000	3.00000000	0.16793744
340	0.87500000	4.00000000	0.17810362
341	1.12500000	4.00000000	0.17458456
342	0.87500000	3.87500000	0.17341153
343	1.00000000	3.87500000	0.15581623
344	3.12500000	0.00000000	-0.00097752
345	2.87500000	0.00000000	-0.03655914
346	3.12500000	0.12500000	-0.01700880
347	3.00000000	0.12500000	-0.04437928
348	4.00000000	0.87500000	0.06314761
349	4.00000000	1.12500000	0.04203324
350	3.87500000	0.87500000	0.03851417

351	3.87500000	1.00000000	0.02052786
352	3.12500000	3.12500000	0.02717498
353	2.87500000	2.87500000	-0.04750733
354	3.00000000	3.12500000	0.00254154
355	3.12500000	3.00000000	0.01427175
356	2.87500000	3.00000000	-0.04164223
357	3.00000000	2.87500000	-0.04946237
358	3.12500000	4.00000000	0.20000000
359	2.87500000	4.00000000	0.19921799
360	3.00000000	3.87500000	0.17184751
361	2.87500000	3.87500000	0.16950147
362	4.00000000	3.12500000	0.18475073
363	4.00000000	2.87500000	0.15738025
364	3.87500000	3.00000000	0.17028348
365	3.87500000	2.87500000	0.12766373
366	0.87500000	2.00000000	-0.16989247
367	1.12500000	2.00000000	-0.19217986
368	0.87500000	1.87500000	-0.16911046
369	1.00000000	1.87500000	-0.18866080
370	1.00000000	2.12500000	-0.16676442
371	1.12500000	2.12500000	-0.18201369
372	0.87500000	2.87500000	0.00449658
373	1.12500000	3.12500000	0.02717498
374	0.87500000	3.00000000	0.03069404
375	1.00000000	3.12500000	-0.17223851
376	1.00000000	2.87500000	-0.02248289
377	1.12500000	3.00000000	-0.00723363
378	2.00000000	0.87500000	-0.19022483
379	2.00000000	1.12500000	-0.19921799
380	1.87500000	0.87500000	-0.18123167
381	1.87500000	1.00000000	-0.19608993
382	2.12500000	1.00000000	-0.19804497
383	2.12500000	1.12500000	-0.19882698
384	2.87500000	0.87500000	-0.18748778
385	3.12500000	1.12500000	-0.17067449
386	3.00000000	0.87500000	-0.17106549
387	3.12500000	1.00000000	-0.16207234
388	2.87500000	1.00000000	-0.09325513
389	3.00000000	1.12500000	-0.19296188
390	2.00000000	2.87500000	-0.13509286
391	2.00000000	3.12500000	-0.07800587
392	2.12500000	3.00000000	-0.11163245
393	2.12500000	3.12500000	-0.01935484
394	1.87500000	2.87500000	-0.12766373
395	1.87500000	3.00000000	-0.09716520
396	2.87500000	2.00000000	-0.19648094
397	3.12500000	2.00000000	-0.17380254
398	3.00000000	2.12500000	-0.17732160
399	3.12500000	2.12500000	-0.15620723
400	2.87500000	1.87500000	-0.19648094
401	3.00000000	1.87500000	-0.19569892
402	0.00000000	0.37500000	0.10694037
403	0.00000000	0.62500000	0.05845552
404	0.12500000	0.50000000	0.06197458
405	0.12500000	0.62500000	0.03851417
406	0.37500000	0.00000000	0.15972630
407	0.62500000	0.00000000	0.10107527
408	0.50000000	0.12500000	0.11515152
409	0.62500000	0.12500000	0.08660802

410	0.37500000	0.37500000	-0.05532747
411	0.62500000	0.62500000	-0.09325513
412	0.37500000	0.50000000	-0.02913001
413	0.50000000	0.37500000	-0.03264907
414	0.50000000	0.62500000	-0.02326491
415	0.62500000	0.50000000	-0.07839687
416	0.00000000	3.62500000	0.19491691
417	0.00000000	3.37500000	0.19257087
418	0.12500000	3.62500000	0.19608993
419	0.12500000	3.50000000	0.19100684
420	0.37500000	4.00000000	0.19726295
421	0.62500000	4.00000000	0.19178886
422	0.37500000	3.87500000	0.19608993
423	0.50000000	3.87500000	0.19335288
424	3.62500000	0.00000000	0.08660802
425	3.37500000	0.00000000	0.04359726
426	3.62500000	0.12500000	0.07409580
427	3.50000000	0.12500000	0.04946237
428	4.00000000	0.37500000	0.11319648
429	4.00000000	0.62500000	0.08778104
430	3.87500000	0.37500000	0.09169110
431	3.87500000	0.50000000	0.07565982
432	3.62500000	3.62500000	0.18905181
433	3.37500000	3.37500000	0.13274682
434	3.50000000	3.62500000	0.18905181
435	3.62500000	3.50000000	0.18670577
436	3.37500000	3.50000000	0.16089932
437	3.50000000	3.37500000	0.17262952
438	3.62500000	4.00000000	0.19921799
439	3.37500000	4.00000000	0.19960899
440	3.50000000	3.87500000	0.19569892
441	3.37500000	3.87500000	0.17106549
442	4.00000000	3.62500000	0.19296188
443	4.00000000	3.37500000	0.18866080
444	3.87500000	3.50000000	0.18944282
445	3.87500000	3.37500000	0.17653959
446	0.00000000	1.62500000	-0.07174976
447	0.00000000	1.37500000	-0.01857283
448	0.12500000	1.62500000	-0.04203324
449	0.12500000	1.50000000	-0.04086022
450	0.00000000	2.37500000	0.01505376
451	0.00000000	2.62500000	0.06783969
452	0.12500000	2.50000000	0.02482893
453	0.12500000	2.62500000	0.05141740
454	0.37500000	2.00000000	-0.08699902
455	0.62500000	2.00000000	-0.12766373
456	0.37500000	1.87500000	-0.08543500
457	0.50000000	2.12500000	-0.07683284
458	0.50000000	1.87500000	-0.10576735
459	0.62500000	2.12500000	-0.10381232
460	0.37500000	2.37500000	0.00293255
461	0.62500000	2.62500000	0.00997067
462	0.37500000	2.50000000	0.01700880
463	0.50000000	2.37500000	-0.01700880
464	0.50000000	2.62500000	0.03929619
465	0.62500000	2.50000000	-0.01388074
466	1.62500000	0.00000000	-0.09833822
467	1.37500000	0.00000000	-0.08347996
468	1.62500000	0.12500000	-0.11006843

469	1.50000000	0.12500000	-0.10146628
470	2.37500000	0.00000000	-0.08856305
471	2.62500000	0.00000000	-0.06236559
472	2.50000000	0.12500000	-0.09990225
473	2.62500000	0.12500000	-0.08347996
474	2.00000000	0.37500000	-0.15464321
475	2.00000000	0.62500000	-0.17223851
476	1.87500000	0.37500000	-0.14565005
477	2.12500000	0.50000000	-0.16598240
478	1.87500000	0.50000000	-0.15464321
479	2.12500000	0.62500000	-0.17145650
480	2.37500000	0.37500000	-0.16207234
481	2.62500000	0.62500000	-0.18123167
482	2.50000000	0.37500000	-0.14877810
483	2.37500000	0.50000000	-0.17106549
484	2.62500000	0.50000000	-0.15698925
485	2.50000000	0.62500000	-0.17888563
486	1.62500000	1.62500000	-0.18983382
487	1.37500000	1.37500000	-0.18318671
488	1.50000000	1.62500000	-0.18866080
489	1.62500000	1.50000000	-0.18944282
490	1.37500000	1.50000000	-0.18553275
491	1.50000000	1.37500000	-0.18631476
492	2.37500000	2.37500000	-0.13509286
493	2.62500000	2.62500000	-0.09130010
494	2.37500000	2.50000000	-0.13118280
495	2.50000000	2.37500000	-0.13470186
496	2.50000000	2.62500000	-0.10772239
497	2.62500000	2.50000000	-0.11554252
498	1.62500000	2.00000000	-0.19648094
499	1.37500000	2.00000000	-0.19413490
500	1.50000000	1.87500000	-0.19335288
501	1.62500000	2.12500000	-0.19257087
502	1.37500000	1.87500000	-0.19178886
503	1.50000000	2.12500000	-0.18514174
504	2.00000000	1.62500000	-0.20000000
505	2.00000000	1.37500000	-0.19921799
506	1.87500000	1.50000000	-0.19608993
507	2.12500000	1.62500000	-0.19921799
508	1.87500000	1.37500000	-0.19608993
509	2.12500000	1.50000000	-0.19921799
510	2.00000000	2.37500000	-0.18826979
511	2.00000000	2.62500000	-0.06783969
512	2.12500000	2.50000000	-0.16676442
513	1.87500000	2.37500000	-0.18709677
514	2.12500000	2.62500000	-0.16129032
515	1.87500000	2.50000000	-0.17693060
516	2.37500000	2.00000000	-0.19843597
517	2.62500000	2.00000000	-0.19765396
518	2.50000000	2.12500000	-0.17693060
519	2.37500000	1.87500000	-0.19843597
520	2.62500000	2.12500000	-0.17888563
521	2.50000000	1.87500000	-0.19804497
522	1.62500000	4.00000000	0.17028348
523	1.37500000	4.00000000	0.17067449
524	1.50000000	3.87500000	0.10381232
525	1.37500000	3.87500000	0.15503421
526	2.37500000	4.00000000	0.19022483
527	2.62500000	4.00000000	0.19765396

528	2.37500000	3.87500000	0.15894428
529	2.50000000	3.87500000	0.16715543
530	1.62500000	3.62500000	0.11436950
531	1.37500000	3.37500000	-0.12023460
532	1.50000000	3.62500000	0.12062561
533	1.62500000	3.50000000	-0.15464321
534	1.37500000	3.50000000	0.10537634
535	1.50000000	3.37500000	0.06979472
536	2.00000000	3.62500000	0.07526882
537	2.00000000	3.37500000	0.00332356
538	2.12500000	3.62500000	0.08230694
539	1.87500000	3.50000000	0.05923754
540	2.12500000	3.50000000	0.05259042
541	1.87500000	3.37500000	0.02482893
542	4.00000000	1.62500000	0.00762463
543	4.00000000	1.37500000	0.02482893
544	3.87500000	1.50000000	-0.01388074
545	3.87500000	1.37500000	-0.00566960
546	4.00000000	2.37500000	0.05689150
547	4.00000000	2.62500000	0.10694037
548	3.87500000	2.37500000	0.03460411
549	3.87500000	2.50000000	0.05219941
550	3.62500000	1.62500000	-0.08347996
551	3.37500000	1.37500000	-0.12688172
552	3.62500000	1.50000000	-0.07487781
553	3.50000000	1.62500000	-0.10576735
554	3.50000000	1.37500000	-0.09638319
555	3.37500000	1.50000000	-0.12766373
556	3.62500000	2.00000000	-0.08504399
557	3.37500000	2.00000000	-0.12961877
558	3.62500000	2.12500000	-0.05689150
559	3.50000000	1.87500000	-0.10654936
560	3.50000000	2.12500000	-0.08934506
561	3.37500000	1.87500000	-0.12922776
562	0.37500000	1.00000000	-0.07370479
563	0.62500000	1.00000000	-0.11358749
564	0.37500000	0.87500000	-0.04985337
565	0.50000000	0.87500000	-0.07018573
566	0.50000000	1.12500000	-0.09560117
567	0.62500000	1.12500000	-0.11593353
568	0.37500000	1.37500000	-0.03030303
569	0.62500000	1.62500000	-0.07487781
570	0.37500000	1.50000000	-0.03186706
571	0.50000000	1.62500000	-0.05337243
572	0.50000000	1.37500000	-0.05024438
573	0.62500000	1.50000000	-0.07214076
574	1.00000000	0.37500000	-0.09990225
575	1.00000000	0.62500000	-0.12922776
576	0.87500000	0.37500000	-0.06862170
577	0.87500000	0.50000000	-0.08621701
578	1.12500000	0.50000000	-0.11788856
579	1.12500000	0.62500000	-0.13235582
580	1.37500000	0.37500000	-0.13822092
581	1.62500000	0.62500000	-0.17380254
582	1.50000000	0.37500000	-0.14682307
583	1.62500000	0.50000000	-0.16480938
584	1.37500000	0.50000000	-0.15268817
585	1.50000000	0.62500000	-0.19530792
586	1.00000000	1.37500000	-0.17966764

587	1.00000000	1.62500000	-0.18435973
588	1.12500000	1.50000000	-0.18318671
589	1.12500000	1.62500000	-0.18553275
590	0.87500000	1.37500000	-0.16011730
591	0.87500000	1.50000000	-0.16207234
592	1.37500000	1.00000000	-0.18279570
593	1.62500000	1.00000000	-0.18944282
594	1.50000000	1.12500000	-0.18631476
595	1.62500000	1.12500000	-0.18944282
596	1.37500000	0.87500000	-0.16832845
597	1.50000000	0.87500000	-0.17106549
598	0.37500000	3.37500000	0.18592375
599	0.62500000	3.62500000	0.18279570
600	0.37500000	3.50000000	0.18709677
601	0.50000000	3.62500000	0.18944282
602	0.50000000	3.37500000	0.16950147
603	0.62500000	3.50000000	0.16715543
604	0.37500000	3.00000000	0.08504399
605	0.62500000	3.00000000	0.03929619
606	0.37500000	2.87500000	0.14604106
607	0.50000000	2.87500000	0.04203324
608	0.50000000	3.12500000	0.08347996
609	0.62500000	3.12500000	0.06588465
610	1.00000000	3.62500000	0.12609971
611	1.00000000	3.37500000	0.08387097
612	1.12500000	3.62500000	0.12609971
613	1.12500000	3.50000000	0.10928641
614	0.87500000	3.50000000	0.12805474
615	0.87500000	3.37500000	0.11280547
616	3.37500000	0.37500000	-0.00566960
617	3.62500000	0.62500000	0.01309873
618	3.50000000	0.37500000	0.01700880
619	3.62500000	0.50000000	0.02561095
620	3.37500000	0.50000000	-0.02678397
621	3.50000000	0.62500000	-0.01700880
622	3.00000000	0.37500000	-0.04281525
623	3.00000000	0.62500000	-0.08504399
624	2.87500000	0.37500000	-0.05571848
625	2.87500000	0.50000000	-0.08035191
626	3.12500000	0.50000000	-0.03655914
627	3.12500000	0.62500000	-0.05454545
628	3.62500000	1.00000000	-0.04633431
629	3.37500000	1.00000000	-0.10733138
630	3.62500000	1.12500000	-0.05493646
631	3.50000000	1.12500000	-0.08543500
632	3.50000000	0.87500000	-0.05884653
633	3.37500000	0.87500000	-0.08621701
634	3.00000000	3.37500000	0.05063539
635	3.00000000	3.62500000	0.10694037
636	3.12500000	3.50000000	0.15347019
637	3.12500000	3.62500000	0.10694037
638	2.87500000	3.37500000	0.03421310
639	2.87500000	3.50000000	0.07605083
640	3.37500000	3.00000000	0.00801564
641	3.62500000	3.00000000	0.06001955
642	3.50000000	3.12500000	0.06080156
643	3.62500000	3.12500000	0.06236559
644	3.37500000	2.87500000	-0.01583578
645	3.50000000	2.87500000	0.00527859

646	2.62500000	3.00000000	-0.12453568
647	2.37500000	3.00000000	-0.14799609
648	2.50000000	2.87500000	-0.14682307
649	2.37500000	2.87500000	-0.16441838
650	2.62500000	3.12500000	-0.09677419
651	2.50000000	3.12500000	-0.09872923
652	3.00000000	2.62500000	-0.09755621
653	3.00000000	2.37500000	-0.14017595
654	2.87500000	2.50000000	-0.11984360
655	2.87500000	2.37500000	-0.14095797
656	3.12500000	2.62500000	-0.05845552
657	3.12500000	2.50000000	-0.01270772
658	2.62500000	3.62500000	0.11436950
659	2.37500000	3.37500000	0.03460411
660	2.50000000	3.62500000	0.11358749
661	2.37500000	3.50000000	0.07526882
662	2.62500000	3.50000000	0.08582600
663	2.50000000	3.37500000	0.04164223
664	3.62500000	2.62500000	0.01270772
665	3.37500000	2.37500000	-0.08191593
666	3.62500000	2.50000000	-0.01231672
667	3.50000000	2.37500000	-0.05962854
668	3.50000000	2.62500000	0.00058651
669	3.37500000	2.50000000	-0.06314761
670	1.00000000	2.37500000	-0.11867058
671	1.00000000	2.62500000	-0.07057674
672	0.87500000	2.37500000	-0.09755621
673	0.87500000	2.50000000	-0.06783969
674	1.12500000	2.50000000	-0.11006843
675	1.12500000	2.62500000	-0.07917889
676	1.37500000	2.37500000	-0.16950147
677	1.62500000	2.62500000	-0.15151515
678	1.50000000	2.37500000	-0.17067449
679	1.62500000	2.50000000	-0.16793744
680	1.37500000	2.50000000	-0.14565005
681	1.50000000	2.62500000	-0.13040078
682	1.37500000	3.00000000	-0.10029326
683	1.62500000	3.00000000	-0.13040078
684	1.50000000	3.12500000	-0.07448680
685	1.62500000	3.12500000	-0.08934506
686	1.37500000	2.87500000	-0.12414467
687	1.50000000	2.87500000	-0.13939394
688	2.37500000	1.00000000	-0.19648094
689	2.62500000	1.00000000	-0.19491691
690	2.37500000	0.87500000	-0.18748778
691	2.50000000	0.87500000	-0.19022483
692	2.50000000	1.12500000	-0.19608993
693	2.62500000	1.12500000	-0.19530792
694	2.37500000	1.37500000	-0.19804497
695	2.62500000	1.62500000	-0.19726295
696	2.37500000	1.50000000	-0.19804497
697	2.50000000	1.62500000	-0.19804497
698	2.50000000	1.37500000	-0.19726295
699	2.62500000	1.50000000	-0.19687195
700	3.00000000	1.37500000	-0.19374389
701	3.00000000	1.62500000	-0.19452590
702	3.12500000	1.50000000	-0.17184751
703	3.12500000	1.62500000	-0.17262952
704	2.87500000	1.37500000	-0.19452590

705	2.87500000	1.50000000	-0.19530792
706	0.12500000	0.25000000	0.11045943
707	0.12500000	0.37500000	0.08582600
708	0.25000000	0.12500000	0.10694037
709	0.37500000	0.12500000	0.07956989
710	0.25000000	0.37500000	0.06549365
711	0.37500000	0.25000000	0.06236559
712	0.12500000	3.87500000	0.19374389
713	0.12500000	3.75000000	0.19257087
714	0.25000000	3.87500000	0.19491691
715	3.87500000	0.12500000	0.11710655
716	3.75000000	0.12500000	0.09560117
717	3.87500000	0.25000000	0.10420332
718	3.75000000	3.87500000	0.19569892
719	3.87500000	3.75000000	0.19374389
720	3.62500000	3.75000000	0.19257087
721	3.75000000	3.62500000	0.19022483
722	3.62500000	3.87500000	0.19569892
723	3.87500000	3.62500000	0.19139785
724	0.12500000	1.87500000	-0.04477028
725	0.12500000	1.75000000	-0.04320626
726	0.12500000	2.25000000	-0.00254154
727	0.12500000	2.37500000	0.01114370
728	0.25000000	2.12500000	-0.03655914
729	0.25000000	1.87500000	-0.06510264
730	0.37500000	2.12500000	-0.05689150
731	0.25000000	2.37500000	0.00723363
732	0.37500000	2.25000000	-0.02678397
733	1.87500000	0.12500000	-0.12492669
734	1.75000000	0.12500000	-0.11593353
735	2.25000000	0.12500000	-0.12649071
736	2.37500000	0.12500000	-0.11319648
737	2.12500000	0.25000000	-0.14838710
738	1.87500000	0.25000000	-0.13704790
739	2.12500000	0.37500000	-0.15698925
740	2.37500000	0.25000000	-0.13743891
741	2.25000000	0.37500000	-0.15933529
742	1.75000000	1.87500000	-0.19530792
743	1.87500000	1.75000000	-0.19648094
744	1.62500000	1.75000000	-0.19217986
745	1.75000000	1.62500000	-0.19335288
746	2.12500000	2.25000000	-0.17458456
747	2.25000000	2.12500000	-0.17771261
748	2.25000000	2.37500000	-0.15268817
749	2.37500000	2.25000000	-0.15620723
750	1.87500000	2.12500000	-0.19491691
751	1.62500000	1.87500000	-0.19413490
752	1.75000000	2.12500000	-0.19374389
753	2.12500000	1.87500000	-0.19960899
754	1.87500000	1.62500000	-0.19648094
755	2.12500000	1.75000000	-0.19960899
756	2.12500000	2.37500000	-0.17067449
757	1.87500000	2.25000000	-0.19100684
758	2.37500000	2.12500000	-0.17732160
759	2.25000000	1.87500000	-0.19921799
760	1.75000000	3.87500000	0.15347019
761	1.62500000	3.87500000	0.15386119
762	2.12500000	3.87500000	0.14486804
763	2.25000000	3.87500000	0.15190616

764	1.87500000	3.75000000	0.12179863
765	1.62500000	3.75000000	0.13704790
766	1.75000000	3.62500000	0.10537634
767	2.12500000	3.75000000	0.11358749
768	1.87500000	3.62500000	0.09051808
769	3.87500000	1.75000000	-0.03147605
770	3.87500000	1.62500000	-0.02287390
771	3.87500000	2.12500000	-0.01544477
772	3.87500000	2.25000000	0.00957967
773	3.75000000	1.87500000	-0.06236559
774	3.75000000	1.62500000	-0.06549365
775	3.62500000	1.75000000	-0.08426197
776	3.75000000	2.12500000	-0.03773216
777	3.62500000	1.87500000	-0.08465298
778	0.12500000	0.87500000	-0.00957967
779	0.12500000	0.75000000	0.01427175
780	0.12500000	1.25000000	-0.03773216
781	0.12500000	1.37500000	-0.03929619
782	0.25000000	1.12500000	-0.05610948
783	0.25000000	0.87500000	-0.02952102
784	0.37500000	1.12500000	-0.07565982
785	0.25000000	1.37500000	-0.06001955
786	0.37500000	1.25000000	-0.07800587
787	0.87500000	0.12500000	-0.03929619
788	0.75000000	0.12500000	-0.00801564
789	1.25000000	0.12500000	-0.08347996
790	1.37500000	0.12500000	-0.09208211
791	1.12500000	0.25000000	-0.08856305
792	0.87500000	0.25000000	-0.05376344
793	1.12500000	0.37500000	-0.10342131
794	1.37500000	0.25000000	-0.10107527
795	1.25000000	0.37500000	-0.10694037
796	0.75000000	0.87500000	-0.10967742
797	0.87500000	0.75000000	-0.11515152
798	0.62500000	0.75000000	-0.06666667
799	0.75000000	0.62500000	-0.07174976
800	1.12500000	1.25000000	-0.17888563
801	1.25000000	1.12500000	-0.17653959
802	1.25000000	1.37500000	-0.18201369
803	1.37500000	1.25000000	-0.18318671
804	0.87500000	1.12500000	-0.15581623
805	0.62500000	0.87500000	-0.09012708
806	0.75000000	1.12500000	-0.13548387
807	1.12500000	0.87500000	-0.16168133
808	0.87500000	0.62500000	-0.10029326
809	1.12500000	0.75000000	-0.14682307
810	1.12500000	1.37500000	-0.18123167
811	0.87500000	1.25000000	-0.15698925
812	1.37500000	1.12500000	-0.18318671
813	1.25000000	0.87500000	-0.16520039
814	0.12500000	3.25000000	0.18396872
815	0.12500000	3.37500000	0.18514174
816	0.12500000	2.87500000	0.12922776
817	0.12500000	2.75000000	0.09012708
818	0.25000000	3.12500000	0.16637341
819	0.25000000	3.37500000	0.18240469
820	0.37500000	3.25000000	0.16480938
821	0.25000000	2.87500000	0.11280547
822	0.37500000	3.12500000	0.14995112

823	0.75000000	3.87500000	0.18005865
824	0.62500000	3.87500000	0.18670577
825	1.12500000	3.87500000	0.15542522
826	1.25000000	3.87500000	0.15542522
827	0.87500000	3.75000000	0.15738025
828	0.62500000	3.75000000	0.18162268
829	0.75000000	3.62500000	0.15933529
830	1.12500000	3.75000000	0.13978495
831	0.87500000	3.62500000	0.14173998
832	3.25000000	0.12500000	0.00527859
833	3.37500000	0.12500000	0.02756598
834	2.87500000	0.12500000	-0.05767351
835	2.75000000	0.12500000	-0.07057674
836	3.12500000	0.25000000	-0.03812317
837	3.37500000	0.25000000	0.01114370
838	3.25000000	0.37500000	-0.03225806
839	2.87500000	0.25000000	-0.07878788
840	3.12500000	0.37500000	-0.05962854
841	3.87500000	0.75000000	0.05102639
842	3.87500000	0.62500000	0.06314761
843	3.87500000	1.12500000	0.01192571
844	3.87500000	1.25000000	0.00332356
845	3.75000000	0.87500000	0.00801564
846	3.75000000	0.62500000	0.03851417
847	3.62500000	0.75000000	-0.00410557
848	3.75000000	1.12500000	-0.01857283
849	3.62500000	0.87500000	-0.02209189
850	3.12500000	3.25000000	0.05532747
851	3.25000000	3.12500000	0.06705767
852	3.25000000	3.37500000	0.10811339
853	3.37500000	3.25000000	0.11984360
854	2.75000000	2.87500000	-0.06353861
855	2.87500000	2.75000000	-0.07135875
856	2.62500000	2.75000000	-0.08582600
857	2.75000000	2.62500000	-0.09364614
858	2.87500000	3.12500000	-0.01388074
859	3.12500000	3.37500000	0.08308895
860	2.87500000	3.25000000	0.01427175
861	3.12500000	2.87500000	-0.00957967
862	3.37500000	3.12500000	0.10694037
863	3.25000000	2.87500000	0.02991202
864	2.62500000	2.87500000	-0.08035191
865	2.75000000	3.12500000	-0.02991202
866	2.87500000	2.62500000	-0.09560117
867	3.12500000	2.75000000	-0.03382209
868	3.12500000	3.87500000	0.17145650
869	3.25000000	3.87500000	0.17145650
870	2.75000000	3.87500000	0.16871945
871	2.62500000	3.87500000	0.16793744
872	2.87500000	3.75000000	0.14134897
873	3.12500000	3.75000000	0.14330401
874	2.87500000	3.62500000	0.11280547
875	2.62500000	3.75000000	0.13822092
876	2.75000000	3.62500000	0.11045943
877	3.87500000	3.12500000	0.17262952
878	3.87500000	3.25000000	0.17458456
879	3.87500000	2.75000000	0.10263930
880	3.87500000	2.62500000	0.07761486
881	3.75000000	2.87500000	0.11554252

882	3.75000000	3.12500000	0.16050831
883	3.62500000	2.87500000	0.10342131
884	3.75000000	2.62500000	0.04789834
885	3.62500000	2.75000000	0.06080156
886	0.75000000	1.87500000	-0.14799609
887	0.87500000	2.12500000	-0.14604106
888	0.62500000	1.87500000	-0.12688172
889	0.75000000	2.12500000	-0.12492669
890	1.12500000	1.87500000	-0.18983382
891	1.25000000	2.12500000	-0.18318671
892	1.25000000	1.87500000	-0.19100684
893	1.37500000	2.12500000	-0.18396872
894	0.87500000	1.75000000	-0.16676442
895	0.62500000	1.75000000	-0.12570870
896	0.75000000	1.62500000	-0.14447703
897	1.12500000	1.75000000	-0.18748778
898	0.87500000	1.62500000	-0.16441838
899	1.12500000	2.25000000	-0.15777126
900	0.87500000	2.25000000	-0.12179863
901	1.12500000	2.37500000	-0.13391984
902	1.37500000	2.25000000	-0.17380254
903	1.25000000	2.37500000	-0.14877810
904	0.75000000	2.87500000	0.03382209
905	0.87500000	2.75000000	-0.01974585
906	0.62500000	2.75000000	0.03655914
907	0.75000000	2.62500000	-0.01661779
908	1.12500000	3.25000000	0.05415445
909	1.25000000	3.12500000	0.01857283
910	1.25000000	3.37500000	0.07956989
911	1.37500000	3.25000000	0.04437928
912	0.87500000	3.12500000	0.05728250
913	0.62500000	2.87500000	0.06314761
914	0.75000000	3.12500000	0.08660802
915	1.12500000	3.37500000	0.08035191
916	0.87500000	3.25000000	0.08387097
917	1.12500000	2.87500000	-0.03108504
918	0.87500000	2.62500000	-0.04359726
919	1.12500000	2.75000000	-0.05532747
920	1.37500000	3.12500000	0.00997067
921	1.25000000	2.87500000	-0.03968719
922	1.87500000	0.75000000	-0.17223851
923	2.12500000	0.87500000	-0.18944282
924	1.87500000	0.62500000	-0.16324536
925	2.12500000	0.75000000	-0.18044966
926	1.87500000	1.12500000	-0.19608993
927	2.12500000	1.25000000	-0.19882698
928	1.87500000	1.25000000	-0.19608993
929	2.12500000	1.37500000	-0.19882698
930	1.75000000	0.87500000	-0.17771261
931	1.75000000	0.62500000	-0.15464321
932	1.62500000	0.75000000	-0.16011730
933	1.75000000	1.12500000	-0.19296188
934	1.62500000	0.87500000	-0.17458456
935	2.25000000	1.12500000	-0.19804497
936	2.25000000	0.87500000	-0.18826979
937	2.37500000	1.12500000	-0.19687195
938	2.25000000	1.37500000	-0.19843597
939	2.37500000	1.25000000	-0.19765396
940	2.87500000	0.75000000	-0.16637341

941	2.75000000	0.87500000	-0.18826979
942	2.75000000	0.62500000	-0.16168133
943	2.62500000	0.75000000	-0.18357771
944	3.25000000	1.12500000	-0.14017595
945	3.12500000	1.25000000	-0.17106549
946	3.37500000	1.25000000	-0.11827957
947	3.25000000	1.37500000	-0.14956012
948	3.12500000	0.87500000	-0.14095797
949	2.87500000	0.62500000	-0.14525904
950	3.12500000	0.75000000	-0.11945259
951	3.37500000	1.12500000	-0.10967742
952	3.25000000	0.87500000	-0.11045943
953	2.87500000	1.12500000	-0.19374389
954	2.62500000	0.87500000	-0.18944282
955	2.75000000	1.12500000	-0.19452590
956	3.12500000	1.37500000	-0.17145650
957	2.87500000	1.25000000	-0.19413490
958	2.12500000	2.87500000	-0.12805474
959	1.87500000	2.75000000	-0.14408602
960	2.12500000	2.75000000	-0.14447703
961	1.87500000	2.62500000	-0.16050831
962	2.12500000	3.25000000	-0.02873900
963	1.87500000	3.12500000	-0.05650049
964	2.12500000	3.37500000	0.01192571
965	1.87500000	3.25000000	-0.01583578
966	2.25000000	3.12500000	-0.06236559
967	2.25000000	2.87500000	-0.12101662
968	2.37500000	3.12500000	-0.05532747
969	2.25000000	3.37500000	0.02052786
970	2.37500000	3.25000000	-0.01309873
971	1.75000000	2.87500000	-0.10615836
972	1.75000000	2.62500000	-0.15307918
973	1.62500000	2.75000000	-0.11515152
974	1.75000000	3.12500000	-0.03538612
975	1.62500000	2.87500000	-0.08504399
976	2.87500000	2.12500000	-0.17810362
977	2.75000000	1.87500000	-0.19726295
978	2.75000000	2.12500000	-0.17888563
979	2.62500000	1.87500000	-0.19765396
980	3.25000000	2.12500000	-0.13391984
981	3.12500000	1.87500000	-0.17341153
982	3.37500000	2.12500000	-0.11163245
983	3.25000000	1.87500000	-0.15151515
984	3.12500000	2.25000000	-0.13743891
985	2.87500000	2.25000000	-0.15933529
986	3.12500000	2.37500000	-0.11906158
987	3.37500000	2.25000000	-0.09403715
988	3.25000000	2.37500000	-0.09755621
989	2.87500000	1.75000000	-0.19608993
990	2.62500000	1.75000000	-0.19765396
991	2.75000000	1.62500000	-0.19648094
992	3.12500000	1.75000000	-0.17302053
993	2.87500000	1.62500000	-0.19569892
994	0.25000000	0.62500000	0.01779081
995	0.37500000	0.62500000	-0.00254154
996	0.37500000	0.75000000	-0.02600196
997	0.62500000	0.25000000	0.00606061
998	0.62500000	0.37500000	-0.01153470
999	0.75000000	0.37500000	-0.03968719

1000	0.25000000	3.62500000	0.18866080
1001	0.37500000	3.75000000	0.19100684
1002	0.37500000	3.62500000	0.18592375
1003	3.62500000	0.25000000	0.05806452
1004	3.75000000	0.37500000	0.06666667
1005	3.62500000	0.37500000	0.04203324
1006	3.37500000	3.62500000	0.16441838
1007	3.37500000	3.75000000	0.16793744
1008	3.62500000	3.37500000	0.17380254
1009	3.75000000	3.37500000	0.17536657
1010	3.25000000	3.62500000	0.13939394
1011	3.62500000	3.25000000	0.16129032
1012	0.25000000	1.62500000	-0.06236559
1013	0.37500000	1.75000000	-0.08426197
1014	0.37500000	1.62500000	-0.08308895
1015	0.25000000	2.62500000	0.04750733
1016	0.37500000	2.62500000	0.04359726
1017	0.37500000	2.75000000	0.06979472
1018	0.62500000	2.25000000	-0.07370479
1019	0.62500000	2.37500000	-0.04398827
1020	0.75000000	2.37500000	-0.07057674
1021	1.62500000	0.25000000	-0.11906158
1022	1.75000000	0.37500000	-0.13665689
1023	1.62500000	0.37500000	-0.12766373
1024	2.62500000	0.25000000	-0.10811339
1025	2.62500000	0.37500000	-0.13235582
1026	2.75000000	0.37500000	-0.11593353
1027	2.25000000	0.62500000	-0.17380254
1028	2.37500000	0.62500000	-0.17653959
1029	2.37500000	0.75000000	-0.18201369
1030	1.37500000	1.62500000	-0.18748778
1031	1.37500000	1.75000000	-0.18983382
1032	1.62500000	1.37500000	-0.18944282
1033	1.75000000	1.37500000	-0.19296188
1034	1.25000000	1.62500000	-0.18631476
1035	1.62500000	1.25000000	-0.18944282
1036	2.37500000	2.62500000	-0.12531769
1037	2.25000000	2.62500000	-0.14330401
1038	2.62500000	2.37500000	-0.13665689
1039	2.62500000	2.25000000	-0.15777126
1040	2.37500000	2.75000000	-0.11984360
1041	2.75000000	2.37500000	-0.13900293
1042	1.62500000	2.25000000	-0.18240469
1043	1.75000000	2.37500000	-0.17966764
1044	1.62500000	2.37500000	-0.17223851
1045	2.25000000	1.62500000	-0.19882698
1046	2.37500000	1.75000000	-0.19843597
1047	2.37500000	1.62500000	-0.19804497
1048	1.37500000	3.75000000	0.13861193
1049	1.37500000	3.62500000	0.12179863
1050	1.25000000	3.62500000	0.12297165
1051	2.37500000	3.75000000	0.12883675
1052	2.25000000	3.62500000	0.09090909
1053	2.37500000	3.62500000	0.09951124
1054	1.62500000	3.37500000	0.05493646
1055	1.75000000	3.37500000	0.03968719
1056	1.62500000	3.25000000	0.02052786
1057	3.75000000	1.37500000	-0.03577713
1058	3.62500000	1.37500000	-0.06588465

1059	3.62500000	1.25000000	-0.05767351
1060	3.75000000	2.37500000	0.00527859
1061	3.62500000	2.25000000	-0.04203324
1062	3.62500000	2.37500000	-0.02443793
1063	3.37500000	1.62500000	-0.12805474
1064	3.37500000	1.75000000	-0.12844575
1065	3.25000000	1.62500000	-0.15034213
1066	0.62500000	1.25000000	-0.11788856
1067	0.62500000	1.37500000	-0.11984360
1068	0.75000000	1.37500000	-0.14017595
1069	1.25000000	0.62500000	-0.13548387
1070	1.37500000	0.62500000	-0.13900293
1071	1.37500000	0.75000000	-0.15347019
1072	0.62500000	3.37500000	0.14565005
1073	0.62500000	3.25000000	0.13079179
1074	0.75000000	3.37500000	0.12805474
1075	3.37500000	0.62500000	-0.04398827
1076	3.25000000	0.62500000	-0.07096774
1077	3.37500000	0.75000000	-0.06197458
1078	2.75000000	3.37500000	0.04007820
1079	2.62500000	3.25000000	-0.00410557
1080	2.62500000	3.37500000	0.03773216
1081	3.37500000	2.75000000	0.02717498
1082	3.25000000	2.62500000	-0.03655914
1083	3.37500000	2.62500000	-0.01505376
1084	1.25000000	2.62500000	-0.09442815
1085	1.37500000	2.62500000	-0.10928641
1086	1.37500000	2.75000000	-0.07878788
1087	2.62500000	1.25000000	-0.19569892
1088	2.62500000	1.37500000	-0.19608993
1089	2.75000000	1.37500000	-0.19530792
1090	0.00000000	0.06250000	0.19433040
1091	0.06250000	0.00000000	0.19198436
1092	0.06250000	0.06250000	0.15796676
1093	0.00000000	3.93750000	0.19315738
1094	0.06250000	4.00000000	0.19433040
1095	3.93750000	0.00000000	0.14017595
1096	4.00000000	0.06250000	0.14447703
1097	3.93750000	3.93750000	0.19726295
1098	3.93750000	4.00000000	0.19902248
1099	4.00000000	3.93750000	0.19784946
1100	0.00000000	1.93750000	-0.02561095
1101	0.00000000	2.06250000	-0.01935484
1102	0.06250000	2.00000000	-0.03636364
1103	0.06250000	2.06250000	-0.02150538
1104	1.93750000	0.00000000	-0.12238514
1105	2.06250000	0.00000000	-0.12179863
1106	2.00000000	0.06250000	-0.13294233
1107	2.06250000	0.06250000	-0.13391984
1108	1.93750000	1.93750000	-0.19824047
1109	2.06250000	2.06250000	-0.18924731
1110	1.93750000	2.00000000	-0.19941349
1111	2.00000000	1.93750000	-0.20000000
1112	2.00000000	2.06250000	-0.19804497
1113	2.06250000	2.00000000	-0.19980450
1114	1.93750000	4.00000000	0.17086999
1115	2.06250000	4.00000000	0.17262952
1116	1.93750000	3.93750000	0.16109482
1117	2.00000000	3.93750000	0.15347019

1118	4.00000000	1.93750000	-0.01427175
1119	4.00000000	2.06250000	-0.00586510
1120	3.93750000	1.93750000	-0.02932551
1121	3.93750000	2.00000000	-0.02952102
1122	0.00000000	0.93750000	-0.00215054
1123	0.00000000	1.06250000	-0.01485826
1124	0.06250000	1.00000000	-0.02424242
1125	0.06250000	1.06250000	-0.02541544
1126	0.93750000	0.00000000	-0.04066471
1127	1.06250000	0.00000000	-0.06099707
1128	1.00000000	0.06250000	-0.06373412
1129	1.06250000	0.06250000	-0.06529814
1130	0.93750000	0.93750000	-0.15131965
1131	1.06250000	1.06250000	-0.17478006
1132	0.93750000	1.00000000	-0.16285435
1133	1.00000000	0.93750000	-0.16578690
1134	1.00000000	1.06250000	-0.17419355
1135	1.06250000	1.00000000	-0.17478006
1136	0.00000000	3.06250000	0.18494624
1137	0.00000000	2.93750000	0.16500489
1138	0.06250000	3.06250000	0.18357771
1139	0.06250000	3.00000000	0.17614858
1140	0.93750000	4.00000000	0.17478006
1141	1.06250000	4.00000000	0.17302053
1142	0.93750000	3.93750000	0.17243402
1143	1.00000000	3.93750000	0.16363636
1144	3.06250000	0.00000000	-0.01212121
1145	2.93750000	0.00000000	-0.02991202
1146	3.06250000	0.06250000	-0.02013685
1147	3.00000000	0.06250000	-0.03382209
1148	4.00000000	0.93750000	0.05689150
1149	4.00000000	1.06250000	0.04633431
1150	3.93750000	0.93750000	0.04457478
1151	3.93750000	1.00000000	0.03558162
1152	3.06250000	3.06250000	0.00078201
1153	2.93750000	2.93750000	-0.03655914
1154	3.00000000	3.06250000	-0.01153470
1155	3.06250000	3.00000000	-0.00566960
1156	2.93750000	3.00000000	-0.03362659
1157	3.00000000	2.93750000	-0.03753666
1158	3.06250000	4.00000000	0.20000000
1159	2.93750000	4.00000000	0.19960899
1160	3.00000000	3.93750000	0.18592375
1161	2.93750000	3.93750000	0.18475073
1162	4.00000000	3.06250000	0.18357771
1163	4.00000000	2.93750000	0.16989247
1164	3.93750000	3.00000000	0.17634409
1165	3.93750000	2.93750000	0.15503421
1166	0.93750000	2.00000000	-0.18044966
1167	1.06250000	2.00000000	-0.19159335
1168	0.93750000	1.93750000	-0.18005865
1169	1.00000000	1.93750000	-0.18983382
1170	1.00000000	2.06250000	-0.17888563
1171	1.06250000	2.06250000	-0.18651026
1172	0.93750000	2.93750000	0.00293255
1173	1.06250000	3.06250000	0.01427175
1174	0.93750000	3.00000000	0.01603128
1175	1.00000000	3.06250000	-0.08543500
1176	1.00000000	2.93750000	-0.01055718

1177	1.06250000	3.00000000	-0.00293255
1178	2.00000000	0.93750000	-0.19472141
1179	2.00000000	1.06250000	-0.19921799
1180	1.93750000	0.93750000	-0.19022483
1181	1.93750000	1.00000000	-0.19765396
1182	2.06250000	1.00000000	-0.19863148
1183	2.06250000	1.06250000	-0.19902248
1184	2.93750000	0.93750000	-0.18983382
1185	3.06250000	1.06250000	-0.18142717
1186	3.00000000	0.93750000	-0.18162268
1187	3.06250000	1.00000000	-0.17712610
1188	2.93750000	1.00000000	-0.14271750
1189	3.00000000	1.06250000	-0.19257087
1190	2.00000000	2.93750000	-0.12688172
1191	2.00000000	3.06250000	-0.09833822
1192	2.06250000	3.00000000	-0.11515152
1193	2.06250000	3.06250000	-0.06901271
1194	1.93750000	2.93750000	-0.12316716
1195	1.93750000	3.00000000	-0.10791789
1196	2.93750000	2.00000000	-0.19628543
1197	3.06250000	2.00000000	-0.18494624
1198	3.00000000	2.06250000	-0.18670577
1199	3.06250000	2.06250000	-0.17614858
1200	2.93750000	1.93750000	-0.19628543
1201	3.00000000	1.93750000	-0.19589443
1202	0.00000000	0.43750000	0.09481916
1203	0.00000000	0.56250000	0.07057674
1204	0.06250000	0.50000000	0.07233627
1205	0.06250000	0.56250000	0.06060606
1206	0.43750000	0.00000000	0.17751711
1207	0.56250000	0.00000000	0.14819159
1208	0.50000000	0.06250000	0.15522972
1209	0.56250000	0.06250000	0.14095797
1210	0.43750000	0.43750000	-0.07761486
1211	0.56250000	0.56250000	-0.09657869
1212	0.43750000	0.50000000	-0.06451613
1213	0.50000000	0.43750000	-0.06627566
1214	0.50000000	0.56250000	-0.06158358
1215	0.56250000	0.50000000	-0.08914956
1216	0.00000000	3.56250000	0.19667644
1217	0.00000000	3.43750000	0.19550342
1218	0.06250000	3.56250000	0.19726295
1219	0.06250000	3.50000000	0.19472141
1220	0.43750000	4.00000000	0.19784946
1221	0.56250000	4.00000000	0.19511241
1222	0.43750000	3.93750000	0.19726295
1223	0.50000000	3.93750000	0.19589443
1224	3.56250000	0.00000000	0.07605083
1225	3.43750000	0.00000000	0.05454545
1226	3.56250000	0.06250000	0.06979472
1227	3.50000000	0.06250000	0.05747801
1228	4.00000000	0.43750000	0.10674487
1229	4.00000000	0.56250000	0.09403715
1230	3.93750000	0.43750000	0.09599218
1231	3.93750000	0.50000000	0.08797654
1232	3.56250000	3.56250000	0.18729228
1233	3.43750000	3.43750000	0.15913978
1234	3.50000000	3.56250000	0.18729228
1235	3.56250000	3.50000000	0.18611926

1236	3.43750000	3.50000000	0.17321603
1237	3.50000000	3.43750000	0.17908113
1238	3.56250000	4.00000000	0.19921799
1239	3.43750000	4.00000000	0.19941349
1240	3.50000000	3.93750000	0.19745846
1241	3.43750000	3.93750000	0.18514174
1242	4.00000000	3.56250000	0.19178886
1243	4.00000000	3.43750000	0.18963832
1244	3.93750000	3.50000000	0.19002933
1245	3.93750000	3.43750000	0.18357771
1246	0.00000000	1.56250000	-0.04594330
1247	0.00000000	1.43750000	-0.01935484
1248	0.06250000	1.56250000	-0.03108504
1249	0.06250000	1.50000000	-0.03049853
1250	0.00000000	2.43750000	0.02189638
1251	0.00000000	2.56250000	0.04828935
1252	0.06250000	2.50000000	0.02678397
1253	0.06250000	2.56250000	0.04007820
1254	0.43750000	2.00000000	-0.09696970
1255	0.56250000	2.00000000	-0.11730205
1256	0.43750000	1.93750000	-0.09618768
1257	0.50000000	2.06250000	-0.09188661
1258	0.50000000	1.93750000	-0.10635386
1259	0.56250000	2.06250000	-0.10537634
1260	0.43750000	2.43750000	0.00782014
1261	0.56250000	2.56250000	0.01133920
1262	0.43750000	2.50000000	0.01485826
1263	0.50000000	2.43750000	-0.00215054
1264	0.50000000	2.56250000	0.02600196
1265	0.56250000	2.50000000	-0.00058651
1266	1.56250000	0.00000000	-0.09540567
1267	1.43750000	0.00000000	-0.08797654
1268	1.56250000	0.06250000	-0.10127077
1269	1.50000000	0.06250000	-0.09696970
1270	2.43750000	0.00000000	-0.08211144
1271	2.56250000	0.00000000	-0.06901271
1272	2.50000000	0.06250000	-0.08778104
1273	2.56250000	0.06250000	-0.07956989
1274	2.00000000	0.43750000	-0.15913978
1275	2.00000000	0.56250000	-0.16793744
1276	1.93750000	0.43750000	-0.15464321
1277	2.06250000	0.50000000	-0.16480938
1278	1.93750000	0.50000000	-0.15913978
1279	2.06250000	0.56250000	-0.16754643
1280	2.43750000	0.43750000	-0.16774194
1281	2.56250000	0.56250000	-0.17732160
1282	2.50000000	0.43750000	-0.16109482
1283	2.43750000	0.50000000	-0.17223851
1284	2.56250000	0.50000000	-0.16520039
1285	2.50000000	0.56250000	-0.17614858
1286	1.56250000	1.56250000	-0.18807429
1287	1.43750000	1.43750000	-0.18475073
1288	1.50000000	1.56250000	-0.18748778
1289	1.56250000	1.50000000	-0.18787879
1290	1.43750000	1.50000000	-0.18592375
1291	1.50000000	1.43750000	-0.18631476
1292	2.43750000	2.43750000	-0.12434018
1293	2.56250000	2.56250000	-0.10244379
1294	2.43750000	2.50000000	-0.12238514

1295	2.50000000	2.43750000	-0.12414467
1296	2.50000000	2.56250000	-0.11065494
1297	2.56250000	2.50000000	-0.11456500
1298	1.56250000	2.00000000	-0.19589443
1299	1.43750000	2.00000000	-0.19472141
1300	1.50000000	1.93750000	-0.19433040
1301	1.56250000	2.06250000	-0.19393939
1302	1.43750000	1.93750000	-0.19354839
1303	1.50000000	2.06250000	-0.19022483
1304	2.00000000	1.56250000	-0.19980450
1305	2.00000000	1.43750000	-0.19941349
1306	1.93750000	1.50000000	-0.19784946
1307	2.06250000	1.56250000	-0.19941349
1308	1.93750000	1.43750000	-0.19784946
1309	2.06250000	1.50000000	-0.19941349
1310	2.00000000	2.43750000	-0.18631476
1311	2.00000000	2.56250000	-0.12609971
1312	2.06250000	2.50000000	-0.17556207
1313	1.93750000	2.43750000	-0.18572825
1314	2.06250000	2.56250000	-0.17282502
1315	1.93750000	2.50000000	-0.18064516
1316	2.43750000	2.00000000	-0.19824047
1317	2.56250000	2.00000000	-0.19784946
1318	2.50000000	2.06250000	-0.18748778
1319	2.43750000	1.93750000	-0.19824047
1320	2.56250000	2.06250000	-0.18846530
1321	2.50000000	1.93750000	-0.19804497
1322	1.56250000	4.00000000	0.17028348
1323	1.43750000	4.00000000	0.17047898
1324	1.50000000	3.93750000	0.13704790
1325	1.43750000	3.93750000	0.16265885
1326	2.43750000	4.00000000	0.19354839
1327	2.56250000	4.00000000	0.19726295
1328	2.43750000	3.93750000	0.17790811
1329	2.50000000	3.93750000	0.18201369
1330	1.56250000	3.56250000	0.10928641
1331	1.43750000	3.43750000	-0.00801564
1332	1.50000000	3.56250000	0.11241447
1333	1.56250000	3.50000000	-0.02521994
1334	1.43750000	3.50000000	0.10478983
1335	1.50000000	3.43750000	0.08699902
1336	2.00000000	3.56250000	0.05962854
1337	2.00000000	3.43750000	0.02365591
1338	2.06250000	3.56250000	0.06314761
1339	1.93750000	3.50000000	0.05161290
1340	2.06250000	3.50000000	0.04828935
1341	1.93750000	3.43750000	0.03440860
1342	4.00000000	1.56250000	0.01192571
1343	4.00000000	1.43750000	0.02052786
1344	3.93750000	1.50000000	0.00117302
1345	3.93750000	1.43750000	0.00527859
1346	4.00000000	2.43750000	0.06940371
1347	4.00000000	2.56250000	0.09442815
1348	3.93750000	2.43750000	0.05826002
1349	3.93750000	2.50000000	0.06705767
1350	3.56250000	1.56250000	-0.09442815
1351	3.43750000	1.43750000	-0.11612903
1352	3.56250000	1.50000000	-0.09012708
1353	3.50000000	1.56250000	-0.10557185

1354	3.50000000	1.43750000	-0.10087977
1355	3.43750000	1.50000000	-0.11652004
1356	3.56250000	2.00000000	-0.09618768
1357	3.43750000	2.00000000	-0.11847507
1358	3.56250000	2.06250000	-0.08211144
1359	3.50000000	1.93750000	-0.10694037
1360	3.50000000	2.06250000	-0.09833822
1361	3.43750000	1.93750000	-0.11827957
1362	0.43750000	1.00000000	-0.08367546
1363	0.56250000	1.00000000	-0.10361681
1364	0.43750000	0.93750000	-0.07174976
1365	0.50000000	0.93750000	-0.08191593
1366	0.50000000	1.06250000	-0.09462366
1367	0.56250000	1.06250000	-0.10478983
1368	0.43750000	1.43750000	-0.01642229
1369	0.56250000	1.56250000	-0.03870968
1370	0.43750000	1.50000000	-0.01720430
1371	0.50000000	1.56250000	-0.02795699
1372	0.50000000	1.43750000	-0.02639296
1373	0.56250000	1.50000000	-0.03734115
1374	1.00000000	0.43750000	-0.10733138
1375	1.00000000	0.56250000	-0.12199413
1376	0.93750000	0.43750000	-0.09169110
1377	0.93750000	0.50000000	-0.10048876
1378	1.06250000	0.50000000	-0.11632454
1379	1.06250000	0.56250000	-0.12355816
1380	1.43750000	0.43750000	-0.16109482
1381	1.56250000	0.56250000	-0.17888563
1382	1.50000000	0.43750000	-0.16539589
1383	1.56250000	0.50000000	-0.17438905
1384	1.43750000	0.50000000	-0.16832845
1385	1.50000000	0.56250000	-0.18963832
1386	1.00000000	1.43750000	-0.18084066
1387	1.00000000	1.56250000	-0.18318671
1388	1.06250000	1.50000000	-0.18260020
1389	1.06250000	1.56250000	-0.18377322
1390	0.93750000	1.43750000	-0.17106549
1391	0.93750000	1.50000000	-0.17204301
1392	1.43750000	1.00000000	-0.18435973
1393	1.56250000	1.00000000	-0.18768328
1394	1.50000000	1.06250000	-0.18611926
1395	1.56250000	1.06250000	-0.18768328
1396	1.43750000	0.93750000	-0.17712610
1397	1.50000000	0.93750000	-0.17849462
1398	0.43750000	3.43750000	0.18826979
1399	0.56250000	3.56250000	0.18670577
1400	0.43750000	3.50000000	0.18885630
1401	0.50000000	3.56250000	0.19002933
1402	0.50000000	3.43750000	0.18005865
1403	0.56250000	3.50000000	0.17888563
1404	0.43750000	3.00000000	0.05180841
1405	0.56250000	3.00000000	0.02893451
1406	0.43750000	2.93750000	0.08230694
1407	0.50000000	2.93750000	0.03030303
1408	0.50000000	3.06250000	0.05102639
1409	0.56250000	3.06250000	0.04222874
1410	1.00000000	3.56250000	0.11925709
1411	1.00000000	3.43750000	0.09814272
1412	1.06250000	3.56250000	0.11925709

1413	1.06250000	3.50000000	0.11085044
1414	0.93750000	3.50000000	0.12023460
1415	0.93750000	3.43750000	0.11260997
1416	3.43750000	0.43750000	-0.00273705
1417	3.56250000	0.56250000	0.00664712
1418	3.50000000	0.43750000	0.00860215
1419	3.56250000	0.50000000	0.01290323
1420	3.43750000	0.50000000	-0.01329423
1421	3.50000000	0.56250000	-0.00840665
1422	3.00000000	0.43750000	-0.03147605
1423	3.00000000	0.56250000	-0.05259042
1424	2.93750000	0.43750000	-0.03792766
1425	2.93750000	0.50000000	-0.05024438
1426	3.06250000	0.50000000	-0.02834800
1427	3.06250000	0.56250000	-0.03734115
1428	3.56250000	1.00000000	-0.06471163
1429	3.43750000	1.00000000	-0.09521017
1430	3.56250000	1.06250000	-0.06901271
1431	3.50000000	1.06250000	-0.08426197
1432	3.50000000	0.93750000	-0.07096774
1433	3.43750000	0.93750000	-0.08465298
1434	3.00000000	3.43750000	0.06060606
1435	3.00000000	3.56250000	0.08875855
1436	3.06250000	3.50000000	0.11202346
1437	3.06250000	3.56250000	0.08875855
1438	2.93750000	3.43750000	0.05239492
1439	2.93750000	3.50000000	0.07331378
1440	3.43750000	3.00000000	-0.01505376
1441	3.56250000	3.00000000	0.01094819
1442	3.50000000	3.06250000	0.01133920
1443	3.56250000	3.06250000	0.01212121
1444	3.43750000	2.93750000	-0.02697947
1445	3.50000000	2.93750000	-0.01642229
1446	2.56250000	3.00000000	-0.15796676
1447	2.43750000	3.00000000	-0.16969697
1448	2.50000000	2.93750000	-0.16911046
1449	2.43750000	2.93750000	-0.17790811
1450	2.56250000	3.06250000	-0.14408602
1451	2.50000000	3.06250000	-0.14506354
1452	3.00000000	2.56250000	-0.10948192
1453	3.00000000	2.43750000	-0.13079179
1454	2.93750000	2.50000000	-0.12062561
1455	2.93750000	2.43750000	-0.13118280
1456	3.06250000	2.56250000	-0.08993157
1457	3.06250000	2.50000000	-0.06705767
1458	2.56250000	3.56250000	0.10205279
1459	2.43750000	3.43750000	0.06217009
1460	2.50000000	3.56250000	0.10166178
1461	2.43750000	3.50000000	0.08250244
1462	2.56250000	3.50000000	0.08778104
1463	2.50000000	3.43750000	0.06568915
1464	3.56250000	2.56250000	-0.01739980
1465	3.43750000	2.43750000	-0.06471163
1466	3.56250000	2.50000000	-0.02991202
1467	3.50000000	2.43750000	-0.05356794
1468	3.50000000	2.56250000	-0.02346041
1469	3.43750000	2.50000000	-0.05532747
1470	1.00000000	2.43750000	-0.10674487
1471	1.00000000	2.56250000	-0.08269795

1472	0.93750000	2.43750000	-0.09618768
1473	0.93750000	2.50000000	-0.08132942
1474	1.06250000	2.50000000	-0.10244379
1475	1.06250000	2.56250000	-0.08699902
1476	1.43750000	2.43750000	-0.16793744
1477	1.56250000	2.56250000	-0.15894428
1478	1.50000000	2.43750000	-0.16852395
1479	1.56250000	2.50000000	-0.16715543
1480	1.43750000	2.50000000	-0.15601173
1481	1.50000000	2.56250000	-0.14838710
1482	1.43750000	3.00000000	-0.14232649
1483	1.56250000	3.00000000	-0.15738025
1484	1.50000000	3.06250000	-0.12942326
1485	1.56250000	3.06250000	-0.13685239
1486	1.43750000	2.93750000	-0.15425220
1487	1.50000000	2.93750000	-0.16187683
1488	2.43750000	1.00000000	-0.19608993
1489	2.56250000	1.00000000	-0.19530792
1490	2.43750000	0.93750000	-0.19159335
1491	2.50000000	0.93750000	-0.19296188
1492	2.50000000	1.06250000	-0.19589443
1493	2.56250000	1.06250000	-0.19550342
1494	2.43750000	1.43750000	-0.19784946
1495	2.56250000	1.56250000	-0.19745846
1496	2.43750000	1.50000000	-0.19784946
1497	2.50000000	1.56250000	-0.19784946
1498	2.50000000	1.43750000	-0.19745846
1499	2.56250000	1.50000000	-0.19726295
1500	3.00000000	1.43750000	-0.19393939
1501	3.00000000	1.56250000	-0.19433040
1502	3.06250000	1.50000000	-0.18299120
1503	3.06250000	1.56250000	-0.18338221
1504	2.93750000	1.43750000	-0.19433040
1505	2.93750000	1.50000000	-0.19472141
1506	0.00000000	0.18750000	0.16011730
1507	0.00000000	0.31250000	0.11886608
1508	0.06250000	0.25000000	0.12062561
1509	0.06250000	0.31250000	0.10830890
1510	0.18750000	0.00000000	0.15444770
1511	0.31250000	0.00000000	0.14193548
1512	0.25000000	0.06250000	0.11554252
1513	0.31250000	0.06250000	0.10185728
1514	0.18750000	0.18750000	0.10322581
1515	0.31250000	0.31250000	0.01720430
1516	0.18750000	0.25000000	0.10009775
1517	0.25000000	0.18750000	0.09833822
1518	0.25000000	0.31250000	0.07761486
1519	0.31250000	0.25000000	0.07605083
1520	0.00000000	3.81250000	0.19198436
1521	0.00000000	3.68750000	0.19315738
1522	0.06250000	3.81250000	0.19257087
1523	0.06250000	3.75000000	0.19198436
1524	0.18750000	4.00000000	0.19550342
1525	0.31250000	4.00000000	0.19667644
1526	0.18750000	3.93750000	0.19491691
1527	0.25000000	3.93750000	0.19550342
1528	3.81250000	0.00000000	0.11886608
1529	3.68750000	0.00000000	0.09736070
1530	3.81250000	0.06250000	0.11260997

1531	3.75000000	0.06250000	0.10185728
1532	4.00000000	0.18750000	0.13196481
1533	4.00000000	0.31250000	0.11945259
1534	3.93750000	0.18750000	0.12140762
1535	3.93750000	0.25000000	0.11495601
1536	3.81250000	3.81250000	0.19393939
1537	3.68750000	3.68750000	0.19061584
1538	3.75000000	3.81250000	0.19393939
1539	3.81250000	3.75000000	0.19296188
1540	3.68750000	3.75000000	0.19237537
1541	3.75000000	3.68750000	0.19120235
1542	3.81250000	4.00000000	0.19921799
1543	3.68750000	4.00000000	0.19921799
1544	3.75000000	3.93750000	0.19745846
1545	3.68750000	3.93750000	0.19745846
1546	4.00000000	3.81250000	0.19589443
1547	4.00000000	3.68750000	0.19393939
1548	3.93750000	3.75000000	0.19433040
1549	3.93750000	3.68750000	0.19315738
1550	0.00000000	1.81250000	-0.02404692
1551	0.00000000	1.68750000	-0.04750733
1552	0.06250000	1.81250000	-0.03401760
1553	0.06250000	1.75000000	-0.03323558
1554	0.00000000	2.18750000	-0.00547410
1555	0.00000000	2.31250000	0.00821114
1556	0.06250000	2.25000000	-0.00058651
1557	0.06250000	2.31250000	0.00625611
1558	0.18750000	2.00000000	-0.05650049
1559	0.31250000	2.00000000	-0.07683284
1560	0.18750000	1.93750000	-0.05571848
1561	0.25000000	2.06250000	-0.05161290
1562	0.25000000	1.93750000	-0.06588465
1563	0.31250000	2.06250000	-0.06177908
1564	0.18750000	2.18750000	-0.01173021
1565	0.31250000	2.31250000	-0.00195503
1566	0.18750000	2.25000000	-0.00469208
1567	0.25000000	2.18750000	-0.02170088
1568	0.25000000	2.31250000	0.00019550
1569	0.31250000	2.25000000	-0.01681329
1570	1.81250000	0.00000000	-0.11026393
1571	1.68750000	0.00000000	-0.10127077
1572	1.81250000	0.06250000	-0.11456500
1573	1.75000000	0.06250000	-0.11006843
1574	2.18750000	0.00000000	-0.10850440
1575	2.31250000	0.00000000	-0.09521017
1576	2.25000000	0.06250000	-0.11417400
1577	2.31250000	0.06250000	-0.10752688
1578	2.00000000	0.18750000	-0.14173998
1579	2.00000000	0.31250000	-0.15034213
1580	1.93750000	0.18750000	-0.13548387
1581	2.06250000	0.25000000	-0.14721408
1582	1.93750000	0.25000000	-0.14154448
1583	2.06250000	0.31250000	-0.15151515
1584	2.18750000	0.18750000	-0.14506354
1585	2.31250000	0.31250000	-0.15640274
1586	2.25000000	0.18750000	-0.13861193
1587	2.18750000	0.25000000	-0.14956012
1588	2.31250000	0.25000000	-0.14408602
1589	2.25000000	0.31250000	-0.15503421

1590	1.81250000	1.81250000	-0.19491691
1591	1.68750000	1.68750000	-0.19159335
1592	1.75000000	1.81250000	-0.19433040
1593	1.81250000	1.75000000	-0.19491691
1594	1.68750000	1.75000000	-0.19276637
1595	1.75000000	1.68750000	-0.19335288
1596	2.18750000	2.18750000	-0.16754643
1597	2.31250000	2.31250000	-0.14584555
1598	2.18750000	2.25000000	-0.16559140
1599	2.25000000	2.18750000	-0.16715543
1600	2.25000000	2.31250000	-0.15464321
1601	2.31250000	2.25000000	-0.15640274
1602	1.81250000	2.00000000	-0.19824047
1603	1.68750000	2.00000000	-0.19706745
1604	1.75000000	1.93750000	-0.19648094
1605	1.81250000	2.06250000	-0.19628543
1606	1.68750000	1.93750000	-0.19589443
1607	1.75000000	2.06250000	-0.19569892
1608	2.00000000	1.81250000	-0.20000000
1609	2.00000000	1.68750000	-0.20000000
1610	1.93750000	1.75000000	-0.19824047
1611	2.06250000	1.81250000	-0.19980450
1612	1.93750000	1.68750000	-0.19824047
1613	2.06250000	1.75000000	-0.19980450
1614	2.00000000	2.18750000	-0.19413490
1615	2.00000000	2.31250000	-0.19022483
1616	2.06250000	2.25000000	-0.18338221
1617	1.93750000	2.18750000	-0.19354839
1618	2.06250000	2.31250000	-0.18142717
1619	1.93750000	2.25000000	-0.19159335
1620	2.18750000	2.00000000	-0.19941349
1621	2.31250000	2.00000000	-0.19882698
1622	2.25000000	2.06250000	-0.18846530
1623	2.18750000	1.93750000	-0.19941349
1624	2.31250000	2.06250000	-0.18826979
1625	2.25000000	1.93750000	-0.19921799
1626	1.81250000	4.00000000	0.17126100
1627	1.68750000	4.00000000	0.17008798
1628	1.75000000	3.93750000	0.16168133
1629	1.68750000	3.93750000	0.16187683
1630	2.18750000	4.00000000	0.17966764
1631	2.31250000	4.00000000	0.18670577
1632	2.18750000	3.93750000	0.16402737
1633	2.25000000	3.93750000	0.16754643
1634	1.81250000	3.81250000	0.14486804
1635	1.68750000	3.68750000	0.12551320
1636	1.75000000	3.81250000	0.14506354
1637	1.81250000	3.75000000	0.12922776
1638	1.68750000	3.75000000	0.13685239
1639	1.75000000	3.68750000	0.12101662
1640	2.00000000	3.81250000	0.12218964
1641	2.00000000	3.68750000	0.09090909
1642	2.06250000	3.81250000	0.12570870
1643	1.93750000	3.75000000	0.11417400
1644	2.06250000	3.75000000	0.11006843
1645	1.93750000	3.68750000	0.09853372
1646	4.00000000	1.81250000	-0.00566960
1647	4.00000000	1.68750000	0.00312805
1648	3.93750000	1.75000000	-0.01642229

1649	3.93750000	1.68750000	-0.01212121
1650	4.00000000	2.18750000	0.01935484
1651	4.00000000	2.31250000	0.04437928
1652	3.93750000	2.18750000	0.00821114
1653	3.93750000	2.25000000	0.02072336
1654	3.81250000	1.81250000	-0.05063539
1655	3.68750000	1.68750000	-0.07233627
1656	3.81250000	1.75000000	-0.04633431
1657	3.75000000	1.81250000	-0.06177908
1658	3.75000000	1.68750000	-0.06334311
1659	3.68750000	1.75000000	-0.07272727
1660	3.81250000	2.00000000	-0.05161290
1661	3.68750000	2.00000000	-0.07390029
1662	3.81250000	2.06250000	-0.03910068
1663	3.75000000	1.93750000	-0.06256109
1664	3.75000000	2.06250000	-0.05024438
1665	3.68750000	1.93750000	-0.07370479
1666	0.00000000	0.81250000	0.02209189
1667	0.00000000	0.68750000	0.04633431
1668	0.06250000	0.81250000	0.01231672
1669	0.06250000	0.75000000	0.02424242
1670	0.00000000	1.18750000	-0.01622678
1671	0.00000000	1.31250000	-0.01779081
1672	0.06250000	1.25000000	-0.02737048
1673	0.06250000	1.31250000	-0.02815249
1674	0.18750000	1.00000000	-0.04398827
1675	0.31250000	1.00000000	-0.06373412
1676	0.18750000	0.93750000	-0.03167155
1677	0.25000000	1.06250000	-0.05493646
1678	0.25000000	0.93750000	-0.04164223
1679	0.31250000	1.06250000	-0.06471163
1680	0.18750000	1.18750000	-0.04750733
1681	0.31250000	1.31250000	-0.04437928
1682	0.18750000	1.25000000	-0.04809384
1683	0.25000000	1.18750000	-0.05728250
1684	0.25000000	1.31250000	-0.05923754
1685	0.31250000	1.25000000	-0.06823069
1686	0.81250000	0.00000000	-0.00918866
1687	0.68750000	0.00000000	0.05376344
1688	0.81250000	0.06250000	-0.01642229
1689	0.75000000	0.06250000	-0.00078201
1690	1.18750000	0.00000000	-0.06999022
1691	1.31250000	0.00000000	-0.07898338
1692	1.25000000	0.06250000	-0.07898338
1693	1.31250000	0.06250000	-0.08328446
1694	1.00000000	0.18750000	-0.07820137
1695	1.00000000	0.31250000	-0.09266862
1696	0.93750000	0.18750000	-0.06236559
1697	1.06250000	0.25000000	-0.08699902
1698	0.93750000	0.25000000	-0.06959922
1699	1.06250000	0.31250000	-0.09442815
1700	1.18750000	0.18750000	-0.08308895
1701	1.31250000	0.31250000	-0.11515152
1702	1.25000000	0.18750000	-0.08778104
1703	1.18750000	0.25000000	-0.09032258
1704	1.31250000	0.25000000	-0.09657869
1705	1.25000000	0.31250000	-0.09951124
1706	0.81250000	0.81250000	-0.10811339
1707	0.68750000	0.68750000	-0.08993157

1708	0.75000000	0.81250000	-0.09814272
1709	0.81250000	0.75000000	-0.10087977
1710	0.68750000	0.75000000	-0.07663734
1711	0.75000000	0.68750000	-0.07917889
1712	1.18750000	1.18750000	-0.17810362
1713	1.31250000	1.31250000	-0.18142717
1714	1.18750000	1.25000000	-0.17927664
1715	1.25000000	1.18750000	-0.17810362
1716	1.25000000	1.31250000	-0.18084066
1717	1.31250000	1.25000000	-0.18142717
1718	0.81250000	1.00000000	-0.14310850
1719	0.68750000	1.00000000	-0.12355816
1720	0.75000000	0.93750000	-0.12160313
1721	0.81250000	1.06250000	-0.14467253
1722	0.68750000	0.93750000	-0.11182796
1723	0.75000000	1.06250000	-0.13450635
1724	1.00000000	0.81250000	-0.15112414
1725	1.00000000	0.68750000	-0.13646139
1726	0.93750000	0.75000000	-0.12942326
1727	1.06250000	0.81250000	-0.15268817
1728	0.93750000	0.68750000	-0.12199413
1729	1.06250000	0.75000000	-0.14525904
1730	1.00000000	1.18750000	-0.17653959
1731	1.00000000	1.31250000	-0.17869013
1732	1.06250000	1.25000000	-0.17829912
1733	0.93750000	1.18750000	-0.16676442
1734	1.06250000	1.31250000	-0.17947214
1735	0.93750000	1.25000000	-0.16735093
1736	1.18750000	1.00000000	-0.17810362
1737	1.31250000	1.00000000	-0.18123167
1738	1.25000000	1.06250000	-0.17810362
1739	1.18750000	0.93750000	-0.17067449
1740	1.31250000	1.06250000	-0.18142717
1741	1.25000000	0.93750000	-0.17243402
1742	0.00000000	3.18750000	0.18611926
1743	0.00000000	3.31250000	0.18963832
1744	0.06250000	3.25000000	0.18533724
1745	0.06250000	3.31250000	0.18592375
1746	0.00000000	2.81250000	0.12609971
1747	0.00000000	2.68750000	0.08719453
1748	0.06250000	2.81250000	0.11788856
1749	0.06250000	2.75000000	0.09833822
1750	0.18750000	3.18750000	0.18201369
1751	0.31250000	3.31250000	0.18357771
1752	0.18750000	3.25000000	0.18260020
1753	0.25000000	3.18750000	0.17380254
1754	0.25000000	3.31250000	0.18181818
1755	0.31250000	3.25000000	0.17302053
1756	0.18750000	3.00000000	0.15972630
1757	0.31250000	3.00000000	0.11827957
1758	0.18750000	2.93750000	0.14037146
1759	0.25000000	3.06250000	0.15894428
1760	0.25000000	2.93750000	0.13216031
1761	0.31250000	3.06250000	0.15073314
1762	0.81250000	4.00000000	0.18142717
1763	0.68750000	4.00000000	0.18826979
1764	0.75000000	3.93750000	0.18240469
1765	0.68750000	3.93750000	0.18572825
1766	1.18750000	4.00000000	0.17282502

1767	1.31250000	4.00000000	0.17086999
1768	1.18750000	3.93750000	0.16324536
1769	1.25000000	3.93750000	0.16324536
1770	0.81250000	3.81250000	0.17419355
1771	0.68750000	3.68750000	0.17888563
1772	0.75000000	3.81250000	0.17751711
1773	0.81250000	3.75000000	0.16617791
1774	0.68750000	3.75000000	0.17829912
1775	0.75000000	3.68750000	0.16715543
1776	1.00000000	3.81250000	0.14780059
1777	1.00000000	3.68750000	0.13294233
1778	1.06250000	3.81250000	0.14760508
1779	0.93750000	3.75000000	0.14858260
1780	1.06250000	3.75000000	0.13978495
1781	0.93750000	3.68750000	0.14076246
1782	3.18750000	0.00000000	0.01016618
1783	3.31250000	0.00000000	0.03245357
1784	3.25000000	0.06250000	0.01329423
1785	3.31250000	0.06250000	0.02443793
1786	2.81250000	0.00000000	-0.04301075
1787	2.68750000	0.00000000	-0.05591398
1788	2.81250000	0.06250000	-0.05356794
1789	2.75000000	0.06250000	-0.06001955
1790	3.18750000	0.18750000	-0.01407625
1791	3.31250000	0.31250000	-0.00840665
1792	3.25000000	0.18750000	-0.00293255
1793	3.18750000	0.25000000	-0.02463343
1794	3.31250000	0.25000000	0.00000000
1795	3.25000000	0.31250000	-0.02170088
1796	3.00000000	0.18750000	-0.05493646
1797	3.00000000	0.31250000	-0.05415445
1798	2.93750000	0.18750000	-0.06158358
1799	3.06250000	0.25000000	-0.05180841
1800	2.93750000	0.25000000	-0.07214076
1801	3.06250000	0.31250000	-0.06256109
1802	4.00000000	0.81250000	0.06940371
1803	4.00000000	0.68750000	0.08172043
1804	3.93750000	0.75000000	0.06334311
1805	3.93750000	0.68750000	0.06940371
1806	4.00000000	1.18750000	0.03773216
1807	4.00000000	1.31250000	0.02913001
1808	3.93750000	1.18750000	0.02267840
1809	3.93750000	1.25000000	0.01837732
1810	3.81250000	0.81250000	0.03225806
1811	3.68750000	0.68750000	0.01955034
1812	3.81250000	0.75000000	0.03851417
1813	3.75000000	0.81250000	0.01700880
1814	3.75000000	0.68750000	0.03225806
1815	3.68750000	0.75000000	0.01094819
1816	3.81250000	1.00000000	0.00527859
1817	3.68750000	1.00000000	-0.02815249
1818	3.81250000	1.06250000	0.00097752
1819	3.75000000	0.93750000	-0.00097752
1820	3.75000000	1.06250000	-0.01427175
1821	3.68750000	0.93750000	-0.01603128
1822	3.18750000	3.18750000	0.05356794
1823	3.31250000	3.31250000	0.10635386
1824	3.18750000	3.25000000	0.06764418
1825	3.25000000	3.18750000	0.07350929

1826	3.25000000	3.31250000	0.09403715
1827	3.31250000	3.25000000	0.09990225
1828	2.81250000	2.81250000	-0.05845552
1829	2.68750000	2.68750000	-0.08035191
1830	2.75000000	2.81250000	-0.06647116
1831	2.81250000	2.75000000	-0.07038123
1832	2.68750000	2.75000000	-0.07761486
1833	2.75000000	2.68750000	-0.08152493
1834	3.00000000	3.18750000	0.01642229
1835	3.00000000	3.31250000	0.04046921
1836	3.06250000	3.25000000	0.04281525
1837	2.93750000	3.18750000	0.00821114
1838	3.06250000	3.31250000	0.05669599
1839	2.93750000	3.25000000	0.02228739
1840	3.18750000	3.00000000	0.03421310
1841	3.31250000	3.00000000	0.03108504
1842	3.25000000	3.06250000	0.06060606
1843	3.18750000	2.93750000	0.02228739
1844	3.31250000	3.06250000	0.08054741
1845	3.25000000	2.93750000	0.04203324
1846	2.81250000	3.00000000	-0.04985337
1847	2.68750000	3.00000000	-0.09130010
1848	2.75000000	2.93750000	-0.06080156
1849	2.81250000	3.06250000	-0.03597263
1850	2.68750000	2.93750000	-0.06920821
1851	2.75000000	3.06250000	-0.04398827
1852	3.00000000	2.81250000	-0.06158358
1853	3.00000000	2.68750000	-0.08563050
1854	2.93750000	2.75000000	-0.07253177
1855	3.06250000	2.81250000	-0.04164223
1856	2.93750000	2.68750000	-0.08465298
1857	3.06250000	2.75000000	-0.05376344
1858	3.18750000	4.00000000	0.19980450
1859	3.31250000	4.00000000	0.19960899
1860	3.18750000	3.93750000	0.18553275
1861	3.25000000	3.93750000	0.18553275
1862	2.81250000	4.00000000	0.19882698
1863	2.68750000	4.00000000	0.19804497
1864	2.75000000	3.93750000	0.18357771
1865	2.68750000	3.93750000	0.18318671
1866	3.00000000	3.81250000	0.15757576
1867	3.00000000	3.68750000	0.12512219
1868	3.06250000	3.81250000	0.15738025
1869	2.93750000	3.75000000	0.14232649
1870	3.06250000	3.75000000	0.14330401
1871	2.93750000	3.68750000	0.12805474
1872	2.81250000	3.81250000	0.15425220
1873	2.68750000	3.68750000	0.12668622
1874	2.75000000	3.81250000	0.15386119
1875	2.81250000	3.75000000	0.14017595
1876	2.68750000	3.75000000	0.13861193
1877	2.75000000	3.68750000	0.12473118
1878	4.00000000	3.18750000	0.18572825
1879	4.00000000	3.31250000	0.18768328
1880	3.93750000	3.18750000	0.17966764
1881	3.93750000	3.25000000	0.18064516
1882	4.00000000	2.81250000	0.14467253
1883	4.00000000	2.68750000	0.11945259
1884	3.93750000	2.75000000	0.11730205

1885	3.93750000	2.68750000	0.10478983
1886	3.81250000	3.00000000	0.16422287
1887	3.68750000	3.00000000	0.10909091
1888	3.81250000	3.06250000	0.16539589
1889	3.75000000	2.93750000	0.13685239
1890	3.75000000	3.06250000	0.15933529
1891	3.68750000	2.93750000	0.13079179
1892	3.81250000	2.81250000	0.10029326
1893	3.68750000	2.68750000	0.04281525
1894	3.81250000	2.75000000	0.08778104
1895	3.75000000	2.81250000	0.09423265
1896	3.75000000	2.68750000	0.06041056
1897	3.68750000	2.75000000	0.06686217
1898	0.81250000	2.00000000	-0.15933529
1899	0.68750000	2.00000000	-0.13822092
1900	0.75000000	1.93750000	-0.14838710
1901	0.81250000	2.06250000	-0.14740958
1902	0.68750000	1.93750000	-0.13782991
1903	0.75000000	2.06250000	-0.13685239
1904	1.18750000	2.00000000	-0.19276637
1905	1.31250000	2.00000000	-0.19374389
1906	1.18750000	1.93750000	-0.19159335
1907	1.25000000	2.06250000	-0.18826979
1908	1.25000000	1.93750000	-0.19217986
1909	1.31250000	2.06250000	-0.18866080
1910	0.81250000	1.81250000	-0.15796676
1911	0.68750000	1.68750000	-0.11085044
1912	0.75000000	1.81250000	-0.14740958
1913	0.81250000	1.75000000	-0.15679374
1914	0.68750000	1.75000000	-0.13626588
1915	0.75000000	1.68750000	-0.14565005
1916	1.00000000	1.81250000	-0.18748778
1917	1.00000000	1.68750000	-0.18533724
1918	1.06250000	1.81250000	-0.18807429
1919	0.93750000	1.75000000	-0.17653959
1920	1.06250000	1.75000000	-0.18690127
1921	0.93750000	1.68750000	-0.17536657
1922	1.00000000	2.18750000	-0.15483871
1923	1.00000000	2.31250000	-0.13079179
1924	0.93750000	2.18750000	-0.14447703
1925	1.06250000	2.25000000	-0.15034213
1926	0.93750000	2.25000000	-0.13235582
1927	1.06250000	2.31250000	-0.13841642
1928	1.18750000	2.18750000	-0.17751711
1929	1.31250000	2.31250000	-0.17126100
1930	1.25000000	2.18750000	-0.17810362
1931	1.18750000	2.25000000	-0.16539589
1932	1.31250000	2.25000000	-0.17341153
1933	1.25000000	2.31250000	-0.16089932
1934	0.81250000	2.81250000	0.00586510
1935	0.68750000	2.68750000	0.00860215
1936	0.75000000	2.81250000	0.02052786
1937	0.81250000	2.75000000	-0.00625611
1938	0.68750000	2.75000000	0.02189638
1939	0.75000000	2.68750000	-0.00469208
1940	1.18750000	3.18750000	0.04007820
1941	1.31250000	3.31250000	-0.03362659
1942	1.18750000	3.25000000	0.05356794
1943	1.25000000	3.18750000	0.03577713

1944	1.25000000	3.31250000	0.06627566
1945	1.31250000	3.25000000	0.04868035
1946	0.81250000	3.00000000	0.04535679
1947	0.68750000	3.00000000	0.04965787
1948	0.75000000	2.93750000	0.04692082
1949	0.81250000	3.06250000	0.05865103
1950	0.68750000	2.93750000	0.06158358
1951	0.75000000	3.06250000	0.07331378
1952	1.00000000	3.18750000	-0.05865103
1953	1.00000000	3.31250000	0.06940371
1954	1.06250000	3.25000000	0.05454545
1955	0.93750000	3.18750000	0.05610948
1956	1.06250000	3.31250000	0.06764418
1957	0.93750000	3.25000000	0.06940371
1958	1.00000000	2.81250000	-0.03460411
1959	1.00000000	2.68750000	-0.05865103
1960	0.93750000	2.75000000	-0.03323558
1961	1.06250000	2.81250000	-0.03890518
1962	0.93750000	2.68750000	-0.04516129
1963	1.06250000	2.75000000	-0.05102639
1964	1.18750000	3.00000000	-0.01153470
1965	1.31250000	3.00000000	-0.05806452
1966	1.25000000	3.06250000	0.00136852
1967	1.18750000	2.93750000	-0.02346041
1968	1.31250000	3.06250000	-0.00293255
1969	1.25000000	2.93750000	-0.02776149
1970	2.00000000	0.81250000	-0.18572825
1971	2.00000000	0.68750000	-0.17673509
1972	1.93750000	0.75000000	-0.17673509
1973	2.06250000	0.81250000	-0.18533724
1974	1.93750000	0.68750000	-0.17223851
1975	2.06250000	0.75000000	-0.18084066
1976	2.00000000	1.18750000	-0.19921799
1977	2.00000000	1.31250000	-0.19921799
1978	1.93750000	1.18750000	-0.19765396
1979	2.06250000	1.25000000	-0.19902248
1980	1.93750000	1.25000000	-0.19765396
1981	2.06250000	1.31250000	-0.19902248
1982	1.81250000	0.81250000	-0.17223851
1983	1.68750000	0.68750000	-0.16852395
1984	1.81250000	0.75000000	-0.16774194
1985	1.75000000	0.81250000	-0.17047898
1986	1.75000000	0.68750000	-0.15894428
1987	1.68750000	0.75000000	-0.16168133
1988	1.81250000	1.00000000	-0.19433040
1989	1.68750000	1.00000000	-0.19100684
1990	1.81250000	1.06250000	-0.19433040
1991	1.75000000	0.93750000	-0.18514174
1992	1.75000000	1.06250000	-0.19276637
1993	1.68750000	0.93750000	-0.18357771
1994	2.18750000	1.00000000	-0.19765396
1995	2.31250000	1.00000000	-0.19687195
1996	2.18750000	0.93750000	-0.19335288
1997	2.25000000	1.06250000	-0.19765396
1998	2.25000000	0.93750000	-0.19276637
1999	2.31250000	1.06250000	-0.19706745
2000	2.18750000	1.18750000	-0.19863148
2001	2.31250000	1.31250000	-0.19824047
2002	2.18750000	1.25000000	-0.19863148

2003	2.25000000	1.18750000	-0.19824047
2004	2.25000000	1.31250000	-0.19843597
2005	2.31250000	1.25000000	-0.19804497
2006	2.81250000	0.81250000	-0.18514174
2007	2.68750000	0.68750000	-0.18201369
2008	2.81250000	0.75000000	-0.17458456
2009	2.75000000	0.81250000	-0.18553275
2010	2.75000000	0.68750000	-0.17223851
2011	2.68750000	0.75000000	-0.18318671
2012	3.18750000	1.18750000	-0.15972630
2013	3.31250000	1.31250000	-0.13782991
2014	3.25000000	1.18750000	-0.14447703
2015	3.18750000	1.25000000	-0.15992180
2016	3.31250000	1.25000000	-0.13352884
2017	3.25000000	1.31250000	-0.14916911
2018	3.00000000	0.81250000	-0.16050831
2019	3.00000000	0.68750000	-0.11749756
2020	2.93750000	0.75000000	-0.15816227
2021	3.06250000	0.81250000	-0.14545455
2022	2.93750000	0.68750000	-0.14760508
2023	3.06250000	0.75000000	-0.13470186
2024	3.18750000	1.00000000	-0.14682307
2025	3.31250000	1.00000000	-0.11945259
2026	3.25000000	1.06250000	-0.13587488
2027	3.18750000	0.93750000	-0.13626588
2028	3.31250000	1.06250000	-0.12062561
2029	3.25000000	0.93750000	-0.12101662
2030	2.81250000	1.00000000	-0.14369501
2031	2.68750000	1.00000000	-0.19452590
2032	2.75000000	0.93750000	-0.19120235
2033	2.81250000	1.06250000	-0.19393939
2034	2.68750000	0.93750000	-0.19178886
2035	2.75000000	1.06250000	-0.19433040
2036	3.00000000	1.18750000	-0.19315738
2037	3.00000000	1.31250000	-0.19354839
2038	3.06250000	1.25000000	-0.18220919
2039	2.93750000	1.18750000	-0.19354839
2040	3.06250000	1.31250000	-0.18240469
2041	2.93750000	1.25000000	-0.19374389
2042	2.00000000	2.81250000	-0.14330401
2043	2.00000000	2.68750000	-0.10967742
2044	2.06250000	2.81250000	-0.13978495
2045	1.93750000	2.75000000	-0.14780059
2046	2.06250000	2.75000000	-0.14799609
2047	1.93750000	2.68750000	-0.15601173
2048	2.00000000	3.18750000	-0.05767351
2049	2.00000000	3.31250000	-0.01700880
2050	2.06250000	3.25000000	-0.03304008
2051	1.93750000	3.18750000	-0.04692082
2052	2.06250000	3.31250000	-0.01270772
2053	1.93750000	3.25000000	-0.02658847
2054	2.18750000	3.00000000	-0.10811339
2055	2.31250000	3.00000000	-0.12629521
2056	2.18750000	2.93750000	-0.11632454
2057	2.25000000	3.06250000	-0.08347996
2058	2.25000000	2.93750000	-0.11280547
2059	2.31250000	3.06250000	-0.07996090
2060	2.18750000	3.18750000	-0.01974585
2061	2.31250000	3.31250000	0.00723363

2062	2.18750000	3.25000000	-0.02443793
2063	2.25000000	3.18750000	-0.04125122
2064	2.25000000	3.31250000	0.00019550
2065	2.31250000	3.25000000	-0.01661779
2066	1.81250000	2.81250000	-0.13216031
2067	1.68750000	2.68750000	-0.14408602
2068	1.81250000	2.75000000	-0.14037146
2069	1.75000000	2.81250000	-0.12140762
2070	1.75000000	2.68750000	-0.14486804
2071	1.68750000	2.75000000	-0.12590420
2072	1.81250000	3.00000000	-0.08660802
2073	1.68750000	3.00000000	-0.10322581
2074	1.81250000	3.06250000	-0.06627566
2075	1.75000000	2.93750000	-0.09110459
2076	1.75000000	3.06250000	-0.05571848
2077	1.68750000	2.93750000	-0.08054741
2078	2.81250000	2.00000000	-0.19687195
2079	2.68750000	2.00000000	-0.19745846
2080	2.81250000	2.06250000	-0.18768328
2081	2.75000000	1.93750000	-0.19726295
2082	2.75000000	2.06250000	-0.18807429
2083	2.68750000	1.93750000	-0.19745846
2084	3.18750000	2.00000000	-0.16285435
2085	3.31250000	2.00000000	-0.14076246
2086	3.25000000	2.06250000	-0.14291300
2087	3.18750000	1.93750000	-0.16265885
2088	3.31250000	2.06250000	-0.13176931
2089	3.25000000	1.93750000	-0.15171065
2090	3.00000000	2.18750000	-0.16813294
2091	3.00000000	2.31250000	-0.14956012
2092	2.93750000	2.18750000	-0.16852395
2093	3.06250000	2.25000000	-0.14819159
2094	2.93750000	2.25000000	-0.15913978
2095	3.06250000	2.31250000	-0.13900293
2096	3.18750000	2.18750000	-0.13626588
2097	3.31250000	2.31250000	-0.09912023
2098	3.25000000	2.18750000	-0.12512219
2099	3.18750000	2.25000000	-0.12688172
2100	3.31250000	2.25000000	-0.10518084
2101	3.25000000	2.31250000	-0.10694037
2102	2.81250000	1.81250000	-0.19667644
2103	2.68750000	1.68750000	-0.19706745
2104	2.75000000	1.81250000	-0.19706745
2105	2.81250000	1.75000000	-0.19648094
2106	2.68750000	1.75000000	-0.19726295
2107	2.75000000	1.68750000	-0.19667644
2108	3.00000000	1.81250000	-0.19550342
2109	3.00000000	1.68750000	-0.19491691
2110	3.06250000	1.81250000	-0.18435973
2111	2.93750000	1.75000000	-0.19569892
2112	3.06250000	1.75000000	-0.18416422
2113	2.93750000	1.68750000	-0.19550342
2114	0.18750000	0.50000000	0.05161290
2115	0.31250000	0.50000000	0.00606061
2116	0.18750000	0.43750000	0.06353861
2117	0.25000000	0.43750000	0.05337243
2118	0.25000000	0.56250000	0.02952102
2119	0.31250000	0.56250000	0.01935484
2120	0.18750000	0.68750000	0.01642229

2121	0.31250000	0.81250000	-0.02776149
2122	0.18750000	0.75000000	0.00430108
2123	0.25000000	0.81250000	-0.01759531
2124	0.25000000	0.68750000	0.00606061
2125	0.31250000	0.75000000	-0.01583578
2126	0.50000000	0.18750000	0.07487781
2127	0.50000000	0.31250000	0.00097752
2128	0.43750000	0.18750000	0.05708700
2129	0.43750000	0.25000000	0.04848485
2130	0.56250000	0.25000000	0.02033236
2131	0.56250000	0.31250000	0.01153470
2132	0.68750000	0.18750000	0.03206256
2133	0.81250000	0.31250000	-0.04555230
2134	0.75000000	0.18750000	-0.01524927
2135	0.81250000	0.25000000	-0.03812317
2136	0.68750000	0.25000000	-0.00821114
2137	0.75000000	0.31250000	-0.03108504
2138	0.50000000	0.68750000	-0.03499511
2139	0.50000000	0.81250000	-0.05845552
2140	0.56250000	0.75000000	-0.05669599
2141	0.56250000	0.81250000	-0.06842620
2142	0.43750000	0.68750000	-0.02463343
2143	0.43750000	0.75000000	-0.03636364
2144	0.68750000	0.50000000	-0.06783969
2145	0.81250000	0.50000000	-0.07174976
2146	0.75000000	0.56250000	-0.06451613
2147	0.81250000	0.56250000	-0.07878788
2148	0.68750000	0.43750000	-0.03440860
2149	0.75000000	0.43750000	-0.04848485
2150	0.18750000	3.68750000	0.19491691
2151	0.31250000	3.81250000	0.19491691
2152	0.18750000	3.75000000	0.19315738
2153	0.25000000	3.81250000	0.19433040
2154	0.25000000	3.68750000	0.19120235
2155	0.31250000	3.75000000	0.19237537
2156	0.18750000	3.50000000	0.18729228
2157	0.31250000	3.50000000	0.18533724
2158	0.18750000	3.43750000	0.18435973
2159	0.25000000	3.43750000	0.18299120
2160	0.25000000	3.56250000	0.18611926
2161	0.31250000	3.56250000	0.18475073
2162	0.50000000	3.81250000	0.19081134
2163	0.50000000	3.68750000	0.18885630
2164	0.56250000	3.81250000	0.18748778
2165	0.56250000	3.75000000	0.18494624
2166	0.43750000	3.75000000	0.18963832
2167	0.43750000	3.68750000	0.18709677
2168	3.68750000	0.18750000	0.07839687
2169	3.81250000	0.31250000	0.08719453
2170	3.75000000	0.18750000	0.08914956
2171	3.81250000	0.25000000	0.09345064
2172	3.68750000	0.25000000	0.07038123
2173	3.75000000	0.31250000	0.07468231
2174	3.50000000	0.18750000	0.04144673
2175	3.50000000	0.31250000	0.02521994
2176	3.43750000	0.18750000	0.03049853
2177	3.43750000	0.25000000	0.02228739
2178	3.56250000	0.25000000	0.04574780
2179	3.56250000	0.31250000	0.03773216

2180	3.81250000	0.50000000	0.06314761
2181	3.68750000	0.50000000	0.03812317
2182	3.81250000	0.56250000	0.05689150
2183	3.75000000	0.56250000	0.04457478
2184	3.75000000	0.43750000	0.05865103
2185	3.68750000	0.43750000	0.04633431
2186	3.50000000	3.68750000	0.19081134
2187	3.50000000	3.81250000	0.19413490
2188	3.56250000	3.75000000	0.19257087
2189	3.56250000	3.81250000	0.19413490
2190	3.43750000	3.68750000	0.17849462
2191	3.43750000	3.75000000	0.18025415
2192	3.68750000	3.50000000	0.18729228
2193	3.81250000	3.50000000	0.18866080
2194	3.75000000	3.56250000	0.18905181
2195	3.81250000	3.56250000	0.18963832
2196	3.68750000	3.43750000	0.18084066
2197	3.75000000	3.43750000	0.18162268
2198	3.31250000	3.50000000	0.14838710
2199	3.18750000	3.50000000	0.14467253
2200	3.25000000	3.43750000	0.12199413
2201	3.18750000	3.43750000	0.10948192
2202	3.31250000	3.56250000	0.15014663
2203	3.25000000	3.56250000	0.13763441
2204	3.50000000	3.31250000	0.16617791
2205	3.50000000	3.18750000	0.11026393
2206	3.43750000	3.25000000	0.13978495
2207	3.43750000	3.18750000	0.13333333
2208	3.56250000	3.31250000	0.16676442
2209	3.56250000	3.25000000	0.16050831
2210	3.31250000	3.81250000	0.15698925
2211	3.18750000	3.68750000	0.12492669
2212	3.25000000	3.81250000	0.15718475
2213	3.18750000	3.75000000	0.14310850
2214	3.31250000	3.75000000	0.15542522
2215	3.25000000	3.68750000	0.14115347
2216	3.81250000	3.31250000	0.16950147
2217	3.68750000	3.18750000	0.11241447
2218	3.81250000	3.25000000	0.16852395
2219	3.75000000	3.18750000	0.16148583
2220	3.75000000	3.31250000	0.16891496
2221	3.68750000	3.25000000	0.16187683
2222	0.18750000	1.68750000	-0.05278592
2223	0.31250000	1.81250000	-0.07448680
2224	0.18750000	1.75000000	-0.05337243
2225	0.25000000	1.81250000	-0.06432063
2226	0.25000000	1.68750000	-0.06295210
2227	0.31250000	1.75000000	-0.07390029
2228	0.18750000	1.50000000	-0.05122190
2229	0.31250000	1.50000000	-0.04672532
2230	0.18750000	1.43750000	-0.05043988
2231	0.25000000	1.43750000	-0.06080156
2232	0.25000000	1.56250000	-0.06197458
2233	0.31250000	1.56250000	-0.07233627
2234	0.18750000	2.50000000	0.02287390
2235	0.31250000	2.50000000	0.01896383
2236	0.18750000	2.43750000	0.01603128
2237	0.25000000	2.43750000	0.01407625
2238	0.25000000	2.56250000	0.03421310

2239	0.31250000	2.56250000	0.03225806
2240	0.18750000	2.68750000	0.06256109
2241	0.31250000	2.81250000	0.10987292
2242	0.18750000	2.75000000	0.08191593
2243	0.25000000	2.81250000	0.09325513
2244	0.25000000	2.68750000	0.06060606
2245	0.31250000	2.75000000	0.07174976
2246	0.50000000	2.18750000	-0.06197458
2247	0.50000000	2.31250000	-0.03206256
2248	0.43750000	2.18750000	-0.05200391
2249	0.43750000	2.25000000	-0.03695015
2250	0.56250000	2.25000000	-0.06041056
2251	0.56250000	2.31250000	-0.04555230
2252	0.50000000	1.81250000	-0.10518084
2253	0.50000000	1.68750000	-0.07898338
2254	0.56250000	1.81250000	-0.11573803
2255	0.56250000	1.75000000	-0.11515152
2256	0.43750000	1.75000000	-0.09442815
2257	0.43750000	1.68750000	-0.09384164
2258	0.68750000	2.18750000	-0.10224829
2259	0.81250000	2.31250000	-0.09912023
2260	0.75000000	2.18750000	-0.11280547
2261	0.81250000	2.25000000	-0.11124145
2262	0.68750000	2.25000000	-0.08719453
2263	0.75000000	2.31250000	-0.08563050
2264	0.50000000	2.68750000	0.05259042
2265	0.50000000	2.81250000	0.05395894
2266	0.56250000	2.75000000	0.05122190
2267	0.56250000	2.81250000	0.06451613
2268	0.43750000	2.68750000	0.05474096
2269	0.43750000	2.75000000	0.06783969
2270	0.68750000	2.50000000	-0.02737048
2271	0.81250000	2.50000000	-0.05434995
2272	0.75000000	2.56250000	-0.02873900
2273	0.81250000	2.56250000	-0.04222874
2274	0.68750000	2.43750000	-0.04242424
2275	0.75000000	2.43750000	-0.05571848
2276	1.68750000	0.18750000	-0.11906158
2277	1.81250000	0.31250000	-0.13685239
2278	1.75000000	0.18750000	-0.12199413
2279	1.81250000	0.25000000	-0.13255132
2280	1.68750000	0.25000000	-0.12355816
2281	1.75000000	0.31250000	-0.13235582
2282	1.50000000	0.18750000	-0.10576735
2283	1.50000000	0.31250000	-0.12844575
2284	1.43750000	0.18750000	-0.10107527
2285	1.43750000	0.25000000	-0.10557185
2286	1.56250000	0.25000000	-0.11456500
2287	1.56250000	0.31250000	-0.11886608
2288	2.50000000	0.18750000	-0.11221896
2289	2.50000000	0.31250000	-0.13665689
2290	2.43750000	0.18750000	-0.11886608
2291	2.43750000	0.25000000	-0.13098729
2292	2.56250000	0.25000000	-0.11632454
2293	2.56250000	0.31250000	-0.12844575
2294	2.68750000	0.18750000	-0.08758553
2295	2.81250000	0.31250000	-0.07370479
2296	2.75000000	0.18750000	-0.08113392
2297	2.81250000	0.25000000	-0.08523949

2298	2.68750000	0.25000000	-0.09990225
2299	2.75000000	0.31250000	-0.10381232
2300	2.18750000	0.50000000	-0.16715543
2301	2.31250000	0.50000000	-0.16969697
2302	2.18750000	0.43750000	-0.16265885
2303	2.25000000	0.43750000	-0.16383187
2304	2.25000000	0.56250000	-0.17106549
2305	2.31250000	0.56250000	-0.17243402
2306	1.81250000	0.50000000	-0.15014663
2307	1.68750000	0.50000000	-0.15522972
2308	1.81250000	0.56250000	-0.15444770
2309	1.75000000	0.56250000	-0.15014663
2310	1.75000000	0.43750000	-0.14115347
2311	1.68750000	0.43750000	-0.13665689
2312	2.18750000	0.68750000	-0.17556207
2313	2.31250000	0.81250000	-0.18357771
2314	2.18750000	0.75000000	-0.18005865
2315	2.25000000	0.81250000	-0.18396872
2316	2.25000000	0.68750000	-0.17673509
2317	2.31250000	0.75000000	-0.18084066
2318	2.68750000	0.50000000	-0.14877810
2319	2.81250000	0.50000000	-0.11045943
2320	2.75000000	0.56250000	-0.15112414
2321	2.81250000	0.56250000	-0.14291300
2322	2.68750000	0.43750000	-0.13646139
2323	2.75000000	0.43750000	-0.12825024
2324	2.50000000	0.68750000	-0.18181818
2325	2.50000000	0.81250000	-0.18748778
2326	2.56250000	0.75000000	-0.18416422
2327	2.56250000	0.81250000	-0.18709677
2328	2.43750000	0.68750000	-0.18064516
2329	2.43750000	0.75000000	-0.18338221
2330	1.50000000	1.68750000	-0.18983382
2331	1.50000000	1.81250000	-0.19217986
2332	1.56250000	1.75000000	-0.19159335
2333	1.56250000	1.81250000	-0.19257087
2334	1.43750000	1.68750000	-0.18924731
2335	1.43750000	1.75000000	-0.19042033
2336	1.68750000	1.50000000	-0.19120235
2337	1.81250000	1.50000000	-0.19452590
2338	1.75000000	1.56250000	-0.19315738
2339	1.81250000	1.56250000	-0.19472141
2340	1.68750000	1.43750000	-0.19120235
2341	1.75000000	1.43750000	-0.19296188
2342	1.31250000	1.50000000	-0.18494624
2343	1.18750000	1.50000000	-0.18377322
2344	1.25000000	1.43750000	-0.18318671
2345	1.18750000	1.43750000	-0.18279570
2346	1.31250000	1.56250000	-0.18592375
2347	1.25000000	1.56250000	-0.18533724
2348	1.50000000	1.31250000	-0.18631476
2349	1.50000000	1.18750000	-0.18631476
2350	1.43750000	1.25000000	-0.18475073
2351	1.43750000	1.18750000	-0.18475073
2352	1.56250000	1.31250000	-0.18787879
2353	1.56250000	1.25000000	-0.18787879
2354	2.31250000	2.50000000	-0.13998045
2355	2.18750000	2.50000000	-0.15777126
2356	2.25000000	2.43750000	-0.15073314

2357	2.18750000	2.43750000	-0.15972630
2358	2.31250000	2.56250000	-0.13704790
2359	2.25000000	2.56250000	-0.14604106
2360	2.50000000	2.31250000	-0.14525904
2361	2.50000000	2.18750000	-0.16637341
2362	2.43750000	2.25000000	-0.15601173
2363	2.43750000	2.18750000	-0.16656891
2364	2.56250000	2.31250000	-0.14623656
2365	2.56250000	2.25000000	-0.15679374
2366	2.50000000	2.68750000	-0.10498534
2367	2.50000000	2.81250000	-0.12453568
2368	2.56250000	2.75000000	-0.09403715
2369	2.56250000	2.81250000	-0.09130010
2370	2.43750000	2.68750000	-0.11378299
2371	2.43750000	2.75000000	-0.11104594
2372	2.68750000	2.50000000	-0.11671554
2373	2.81250000	2.50000000	-0.11886608
2374	2.75000000	2.56250000	-0.10576735
2375	2.81250000	2.56250000	-0.10674487
2376	2.68750000	2.43750000	-0.12727273
2377	2.75000000	2.43750000	-0.12844575
2378	1.68750000	2.18750000	-0.19120235
2379	1.81250000	2.31250000	-0.18846530
2380	1.75000000	2.18750000	-0.19178886
2381	1.81250000	2.25000000	-0.19042033
2382	1.68750000	2.25000000	-0.18611926
2383	1.75000000	2.31250000	-0.18475073
2384	1.31250000	1.81250000	-0.19022483
2385	1.18750000	1.68750000	-0.18709677
2386	1.25000000	1.81250000	-0.18983382
2387	1.18750000	1.75000000	-0.18807429
2388	1.31250000	1.75000000	-0.18924731
2389	1.25000000	1.68750000	-0.18748778
2390	1.50000000	2.18750000	-0.18005865
2391	1.50000000	2.31250000	-0.17282502
2392	1.43750000	2.18750000	-0.17947214
2393	1.43750000	2.25000000	-0.17438905
2394	1.56250000	2.25000000	-0.17869013
2395	1.56250000	2.31250000	-0.17360704
2396	2.18750000	1.68750000	-0.19902248
2397	2.31250000	1.81250000	-0.19863148
2398	2.18750000	1.75000000	-0.19921799
2399	2.25000000	1.81250000	-0.19902248
2400	2.25000000	1.68750000	-0.19882698
2401	2.31250000	1.75000000	-0.19863148
2402	1.81250000	1.31250000	-0.19452590
2403	1.68750000	1.18750000	-0.19120235
2404	1.81250000	1.25000000	-0.19452590
2405	1.75000000	1.18750000	-0.19296188
2406	1.75000000	1.31250000	-0.19296188
2407	1.68750000	1.25000000	-0.19120235
2408	2.18750000	1.50000000	-0.19882698
2409	2.31250000	1.50000000	-0.19824047
2410	2.18750000	1.43750000	-0.19863148
2411	2.25000000	1.43750000	-0.19843597
2412	2.25000000	1.56250000	-0.19863148
2413	2.31250000	1.56250000	-0.19824047
2414	2.18750000	2.68750000	-0.14956012
2415	2.31250000	2.81250000	-0.15112414

2416	2.18750000	2.75000000	-0.14115347
2417	2.25000000	2.81250000	-0.12942326
2418	2.25000000	2.68750000	-0.14056696
2419	2.31250000	2.75000000	-0.12883675
2420	1.81250000	2.50000000	-0.17321603
2421	1.68750000	2.50000000	-0.16871945
2422	1.81250000	2.56250000	-0.16500489
2423	1.75000000	2.56250000	-0.16129032
2424	1.75000000	2.43750000	-0.17458456
2425	1.68750000	2.43750000	-0.17086999
2426	2.68750000	2.18750000	-0.16950147
2427	2.81250000	2.31250000	-0.15053763
2428	2.75000000	2.18750000	-0.16950147
2429	2.81250000	2.25000000	-0.15972630
2430	2.68750000	2.25000000	-0.15894428
2431	2.75000000	2.31250000	-0.14956012
2432	2.50000000	1.81250000	-0.19804497
2433	2.50000000	1.68750000	-0.19804497
2434	2.56250000	1.81250000	-0.19784946
2435	2.56250000	1.75000000	-0.19784946
2436	2.43750000	1.75000000	-0.19824047
2437	2.43750000	1.68750000	-0.19804497
2438	1.50000000	3.81250000	0.12062561
2439	1.50000000	3.68750000	0.12903226
2440	1.56250000	3.81250000	0.14565005
2441	1.56250000	3.75000000	0.13724340
2442	1.43750000	3.75000000	0.13802542
2443	1.43750000	3.68750000	0.12961877
2444	1.31250000	3.81250000	0.14721408
2445	1.18750000	3.68750000	0.13274682
2446	1.25000000	3.81250000	0.14740958
2447	1.18750000	3.75000000	0.13958944
2448	1.31250000	3.75000000	0.13900293
2449	1.25000000	3.68750000	0.13118280
2450	2.31250000	3.81250000	0.13978495
2451	2.18750000	3.68750000	0.10146628
2452	2.25000000	3.81250000	0.13626588
2453	2.18750000	3.75000000	0.11710655
2454	2.31250000	3.75000000	0.12473118
2455	2.25000000	3.68750000	0.10576735
2456	2.50000000	3.81250000	0.15229717
2457	2.50000000	3.68750000	0.12551320
2458	2.56250000	3.81250000	0.15268817
2459	2.56250000	3.75000000	0.13782991
2460	2.43750000	3.75000000	0.13313783
2461	2.43750000	3.68750000	0.11847507
2462	1.68750000	3.50000000	-0.04027370
2463	1.81250000	3.50000000	0.06666667
2464	1.75000000	3.56250000	0.08973607
2465	1.81250000	3.56250000	0.08230694
2466	1.68750000	3.43750000	0.06451613
2467	1.75000000	3.43750000	0.05689150
2468	1.31250000	3.50000000	0.10576735
2469	1.18750000	3.50000000	0.10772239
2470	1.25000000	3.43750000	0.09286413
2471	1.18750000	3.43750000	0.09325513
2472	1.31250000	3.56250000	0.11397849
2473	1.25000000	3.56250000	0.11456500
2474	1.50000000	3.31250000	0.05259042

2475	1.50000000	3.18750000	-0.01955034
2476	1.43750000	3.25000000	0.03988270
2477	1.43750000	3.18750000	0.02267840
2478	1.56250000	3.31250000	0.04516129
2479	1.56250000	3.25000000	0.02795699
2480	2.18750000	3.50000000	0.05689150
2481	2.31250000	3.50000000	0.06823069
2482	2.18750000	3.43750000	0.03655914
2483	2.25000000	3.43750000	0.04086022
2484	2.25000000	3.56250000	0.07605083
2485	2.31250000	3.56250000	0.08035191
2486	1.81250000	3.31250000	0.01505376
2487	1.68750000	3.18750000	-0.04203324
2488	1.81250000	3.25000000	-0.00527859
2489	1.75000000	3.18750000	-0.01505376
2490	1.75000000	3.31250000	0.02248289
2491	1.68750000	3.25000000	0.01290323
2492	3.81250000	1.50000000	-0.02913001
2493	3.68750000	1.50000000	-0.05962854
2494	3.81250000	1.56250000	-0.03362659
2495	3.75000000	1.56250000	-0.05493646
2496	3.75000000	1.43750000	-0.04007820
2497	3.68750000	1.43750000	-0.05513196
2498	3.81250000	1.31250000	-0.01642229
2499	3.68750000	1.18750000	-0.04105572
2500	3.81250000	1.25000000	-0.01192571
2501	3.75000000	1.18750000	-0.02287390
2502	3.75000000	1.31250000	-0.03147605
2503	3.68750000	1.25000000	-0.04242424
2504	3.81250000	2.31250000	0.01094819
2505	3.68750000	2.18750000	-0.03479961
2506	3.81250000	2.25000000	-0.00156403
2507	3.75000000	2.18750000	-0.02521994
2508	3.75000000	2.31250000	-0.00371457
2509	3.68750000	2.25000000	-0.02737048
2510	3.81250000	2.50000000	0.03753666
2511	3.68750000	2.50000000	0.00527859
2512	3.81250000	2.56250000	0.05024438
2513	3.75000000	2.56250000	0.03538612
2514	3.75000000	2.43750000	0.01407625
2515	3.68750000	2.43750000	-0.00078201
2516	3.50000000	1.68750000	-0.10596285
2517	3.50000000	1.81250000	-0.10635386
2518	3.56250000	1.75000000	-0.09521017
2519	3.56250000	1.81250000	-0.09540567
2520	3.43750000	1.68750000	-0.11710655
2521	3.43750000	1.75000000	-0.11730205
2522	3.50000000	1.31250000	-0.09208211
2523	3.50000000	1.18750000	-0.08660802
2524	3.43750000	1.25000000	-0.10303030
2525	3.43750000	1.18750000	-0.09872923
2526	3.56250000	1.31250000	-0.07683284
2527	3.56250000	1.25000000	-0.07272727
2528	3.31250000	1.50000000	-0.13880743
2529	3.18750000	1.50000000	-0.16089932
2530	3.25000000	1.43750000	-0.14975562
2531	3.18750000	1.43750000	-0.16070381
2532	3.31250000	1.56250000	-0.13900293
2533	3.25000000	1.56250000	-0.15014663

2534	3.50000000	2.18750000	-0.08054741
2535	3.50000000	2.31250000	-0.06568915
2536	3.43750000	2.18750000	-0.09169110
2537	3.43750000	2.25000000	-0.08289345
2538	3.56250000	2.25000000	-0.05689150
2539	3.56250000	2.31250000	-0.04809384
2540	3.31250000	1.81250000	-0.13998045
2541	3.18750000	1.68750000	-0.16168133
2542	3.25000000	1.81250000	-0.15112414
2543	3.18750000	1.75000000	-0.16187683
2544	3.31250000	1.75000000	-0.13958944
2545	3.25000000	1.68750000	-0.15053763
2546	0.50000000	1.18750000	-0.09677419
2547	0.50000000	1.31250000	-0.07409580
2548	0.43750000	1.18750000	-0.08680352
2549	0.43750000	1.25000000	-0.08797654
2550	0.56250000	1.25000000	-0.10791789
2551	0.56250000	1.31250000	-0.10889541
2552	0.68750000	1.18750000	-0.12688172
2553	0.81250000	1.31250000	-0.14897361
2554	0.75000000	1.18750000	-0.13665689
2555	0.81250000	1.25000000	-0.14740958
2556	0.68750000	1.25000000	-0.12785924
2557	0.75000000	1.31250000	-0.13900293
2558	0.68750000	1.50000000	-0.10713587
2559	0.81250000	1.50000000	-0.15210166
2560	0.75000000	1.56250000	-0.14330401
2561	0.81250000	1.56250000	-0.15327468
2562	0.68750000	1.43750000	-0.13098729
2563	0.75000000	1.43750000	-0.14115347
2564	1.18750000	0.50000000	-0.11945259
2565	1.31250000	0.50000000	-0.13685239
2566	1.18750000	0.43750000	-0.11221896
2567	1.25000000	0.43750000	-0.11397849
2568	1.25000000	0.56250000	-0.12825024
2569	1.31250000	0.56250000	-0.13000978
2570	1.18750000	0.68750000	-0.14134897
2571	1.31250000	0.81250000	-0.15933529
2572	1.18750000	0.75000000	-0.14858260
2573	1.25000000	0.81250000	-0.15777126
2574	1.25000000	0.68750000	-0.14291300
2575	1.31250000	0.75000000	-0.15190616
2576	1.50000000	0.68750000	-0.17595308
2577	1.50000000	0.81250000	-0.16383187
2578	1.56250000	0.75000000	-0.15835777
2579	1.56250000	0.81250000	-0.16559140
2580	1.43750000	0.68750000	-0.14780059
2581	1.43750000	0.75000000	-0.15503421
2582	0.50000000	3.31250000	0.15894428
2583	0.50000000	3.18750000	0.11593353
2584	0.43750000	3.25000000	0.15659824
2585	0.43750000	3.18750000	0.14916911
2586	0.56250000	3.31250000	0.14701857
2587	0.56250000	3.25000000	0.13958944
2588	0.68750000	3.50000000	0.15522972
2589	0.81250000	3.50000000	0.13567937
2590	0.75000000	3.56250000	0.15131965
2591	0.81250000	3.56250000	0.14252199
2592	0.68750000	3.43750000	0.14447703

2593	0.75000000	3.43750000	0.13567937
2594	0.68750000	3.18750000	0.08954057
2595	0.81250000	3.31250000	0.11300098
2596	0.75000000	3.18750000	0.09990225
2597	0.81250000	3.25000000	0.09853372
2598	0.68750000	3.25000000	0.12199413
2599	0.75000000	3.31250000	0.12062561
2600	3.31250000	0.50000000	-0.04007820
2601	3.18750000	0.50000000	-0.04496579
2602	3.25000000	0.43750000	-0.04281525
2603	3.18750000	0.43750000	-0.05650049
2604	3.31250000	0.56250000	-0.04868035
2605	3.25000000	0.56250000	-0.06217009
2606	3.50000000	0.68750000	-0.02580645
2607	3.50000000	0.81250000	-0.04672532
2608	3.56250000	0.75000000	-0.01935484
2609	3.56250000	0.81250000	-0.02834800
2610	3.43750000	0.68750000	-0.03929619
2611	3.43750000	0.75000000	-0.04828935
2612	3.18750000	0.68750000	-0.07174976
2613	3.31250000	0.81250000	-0.08758553
2614	3.18750000	0.75000000	-0.10420332
2615	3.25000000	0.81250000	-0.09970674
2616	3.25000000	0.68750000	-0.07996090
2617	3.31250000	0.75000000	-0.07546432
2618	2.81250000	3.31250000	0.01603128
2619	2.68750000	3.18750000	-0.04946237
2620	2.81250000	3.25000000	0.00606061
2621	2.75000000	3.18750000	-0.01603128
2622	2.75000000	3.31250000	0.01896383
2623	2.68750000	3.25000000	-0.00312805
2624	2.81250000	3.50000000	0.07898338
2625	2.68750000	3.50000000	0.08387097
2626	2.81250000	3.56250000	0.09736070
2627	2.75000000	3.56250000	0.09618768
2628	2.75000000	3.43750000	0.06099707
2629	2.68750000	3.43750000	0.05982405
2630	3.31250000	2.81250000	-0.00488759
2631	3.18750000	2.68750000	-0.02619746
2632	3.25000000	2.81250000	0.01798631
2633	3.18750000	2.75000000	-0.01388074
2634	3.31250000	2.75000000	0.01661779
2635	3.25000000	2.68750000	-0.01524927
2636	3.50000000	2.81250000	0.02697947
2637	3.50000000	2.68750000	0.02463343
2638	3.56250000	2.81250000	0.07605083
2639	3.56250000	2.75000000	0.05474096
2640	3.43750000	2.75000000	0.03792766
2641	3.43750000	2.68750000	0.01681329
2642	2.50000000	3.18750000	-0.05259042
2643	2.50000000	3.31250000	0.01759531
2644	2.43750000	3.18750000	-0.03088954
2645	2.43750000	3.25000000	-0.00977517
2646	2.56250000	3.25000000	-0.00527859
2647	2.56250000	3.31250000	0.01564027
2648	3.18750000	2.50000000	-0.04594330
2649	3.31250000	2.50000000	-0.07116325
2650	3.18750000	2.43750000	-0.09912023
2651	3.25000000	2.43750000	-0.08836755

2652	3.25000000	2.56250000	-0.05786901
2653	3.31250000	2.56250000	-0.04711632
2654	1.18750000	2.50000000	-0.11749756
2655	1.31250000	2.50000000	-0.13528837
2656	1.18750000	2.43750000	-0.12942326
2657	1.25000000	2.43750000	-0.13685239
2658	1.25000000	2.56250000	-0.10967742
2659	1.31250000	2.56250000	-0.11710655
2660	1.18750000	2.68750000	-0.07155425
2661	1.31250000	2.81250000	-0.09403715
2662	1.18750000	2.75000000	-0.05962854
2663	1.25000000	2.81250000	-0.05180841
2664	1.25000000	2.68750000	-0.07917889
2665	1.31250000	2.75000000	-0.07135875
2666	1.50000000	2.68750000	-0.11221896
2667	1.50000000	2.81250000	-0.11671554
2668	1.56250000	2.75000000	-0.10459433
2669	1.56250000	2.81250000	-0.08954057
2670	1.43750000	2.68750000	-0.10166178
2671	1.43750000	2.75000000	-0.08641251
2672	2.50000000	1.18750000	-0.19628543
2673	2.50000000	1.31250000	-0.19687195
2674	2.43750000	1.18750000	-0.19667644
2675	2.43750000	1.25000000	-0.19706745
2676	2.56250000	1.25000000	-0.19608993
2677	2.56250000	1.31250000	-0.19628543
2678	2.68750000	1.18750000	-0.19511241
2679	2.81250000	1.31250000	-0.19472141
2680	2.75000000	1.18750000	-0.19472141
2681	2.81250000	1.25000000	-0.19452590
2682	2.68750000	1.25000000	-0.19530792
2683	2.75000000	1.31250000	-0.19511241
2684	2.68750000	1.50000000	-0.19648094
2685	2.81250000	1.50000000	-0.19569892
2686	2.75000000	1.56250000	-0.19628543
2687	2.81250000	1.56250000	-0.19589443
2688	2.68750000	1.43750000	-0.19608993
2689	2.75000000	1.43750000	-0.19569892
2690	0.06250000	0.12500000	0.15307918
2691	0.06250000	0.18750000	0.14995112
2692	0.12500000	0.06250000	0.15073314
2693	0.18750000	0.06250000	0.14584555
2694	0.12500000	0.18750000	0.11358749
2695	0.18750000	0.12500000	0.11182796
2696	0.06250000	3.93750000	0.19374389
2697	0.06250000	3.87500000	0.19315738
2698	0.12500000	3.93750000	0.19433040
2699	3.93750000	0.06250000	0.13391984
2700	3.87500000	0.06250000	0.12336266
2701	3.93750000	0.12500000	0.12766373
2702	3.87500000	3.93750000	0.19745846
2703	3.93750000	3.87500000	0.19628543
2704	3.81250000	3.87500000	0.19569892
2705	3.87500000	3.81250000	0.19472141
2706	3.81250000	3.93750000	0.19745846
2707	3.93750000	3.81250000	0.19530792
2708	0.06250000	1.93750000	-0.03558162
2709	0.06250000	1.87500000	-0.03479961
2710	0.06250000	2.12500000	-0.01446725

2711	0.06250000	2.18750000	-0.00742913
2712	0.12500000	2.06250000	-0.03147605
2713	0.12500000	1.93750000	-0.04555230
2714	0.18750000	2.06250000	-0.04144673
2715	0.12500000	2.18750000	-0.00957967
2716	0.18750000	2.12500000	-0.02658847
2717	1.93750000	0.06250000	-0.12688172
2718	1.87500000	0.06250000	-0.12062561
2719	2.12500000	0.06250000	-0.12727273
2720	2.18750000	0.06250000	-0.12082111
2721	2.06250000	0.12500000	-0.13841642
2722	1.93750000	0.12500000	-0.13118280
2723	2.06250000	0.18750000	-0.14291300
2724	2.18750000	0.12500000	-0.13294233
2725	2.12500000	0.18750000	-0.14389052
2726	1.87500000	1.93750000	-0.19765396
2727	1.93750000	1.87500000	-0.19824047
2728	1.81250000	1.87500000	-0.19589443
2729	1.87500000	1.81250000	-0.19648094
2730	2.06250000	2.12500000	-0.18729228
2731	2.12500000	2.06250000	-0.18905181
2732	2.12500000	2.18750000	-0.17653959
2733	2.18750000	2.12500000	-0.17810362
2734	1.93750000	2.06250000	-0.19745846
2735	1.81250000	1.93750000	-0.19706745
2736	1.87500000	2.06250000	-0.19687195
2737	2.06250000	1.93750000	-0.19980450
2738	1.93750000	1.81250000	-0.19824047
2739	2.06250000	1.87500000	-0.19980450
2740	2.06250000	2.18750000	-0.18533724
2741	1.93750000	2.12500000	-0.19550342
2742	2.18750000	2.06250000	-0.18866080
2743	2.12500000	1.93750000	-0.19960899
2744	1.87500000	3.93750000	0.16285435
2745	1.81250000	3.93750000	0.16304985
2746	2.06250000	3.93750000	0.15698925
2747	2.12500000	3.93750000	0.16050831
2748	1.93750000	3.87500000	0.14545455
2749	1.81250000	3.87500000	0.15327468
2750	1.87500000	3.81250000	0.13743891
2751	2.06250000	3.87500000	0.14134897
2752	1.93750000	3.81250000	0.12981427
2753	3.93750000	1.87500000	-0.02502444
2754	3.93750000	1.81250000	-0.02072336
2755	3.93750000	2.06250000	-0.01681329
2756	3.93750000	2.12500000	-0.00430108
2757	3.87500000	1.93750000	-0.04027370
2758	3.87500000	1.81250000	-0.03577713
2759	3.81250000	1.87500000	-0.05122190
2760	3.87500000	2.06250000	-0.02795699
2761	3.81250000	1.93750000	-0.05141740
2762	0.06250000	0.93750000	-0.01212121
2763	0.06250000	0.87500000	0.00019550
2764	0.06250000	1.12500000	-0.02600196
2765	0.06250000	1.18750000	-0.02658847
2766	0.12500000	1.06250000	-0.03538612
2767	0.12500000	0.93750000	-0.02189638
2768	0.18750000	1.06250000	-0.04516129
2769	0.12500000	1.18750000	-0.03714565

2770	0.18750000	1.12500000	-0.04633431
2771	0.93750000	0.06250000	-0.04789834
2772	0.87500000	0.06250000	-0.03206256
2773	1.12500000	0.06250000	-0.06979472
2774	1.18750000	0.06250000	-0.07448680
2775	1.06250000	0.12500000	-0.07253177
2776	0.93750000	0.12500000	-0.05513196
2777	1.06250000	0.18750000	-0.07976540
2778	1.18750000	0.12500000	-0.07878788
2779	1.12500000	0.18750000	-0.08132942
2780	0.87500000	0.93750000	-0.14115347
2781	0.93750000	0.87500000	-0.14408602
2782	0.81250000	0.87500000	-0.11964809
2783	0.87500000	0.81250000	-0.12238514
2784	1.06250000	1.12500000	-0.17595308
2785	1.12500000	1.06250000	-0.17653959
2786	1.12500000	1.18750000	-0.17771261
2787	1.18750000	1.12500000	-0.17653959
2788	0.93750000	1.06250000	-0.16402737
2789	0.81250000	0.93750000	-0.13118280
2790	0.87500000	1.06250000	-0.15425220
2791	1.06250000	0.93750000	-0.16754643
2792	0.93750000	0.81250000	-0.13685239
2793	1.06250000	0.87500000	-0.16011730
2794	1.06250000	1.18750000	-0.17712610
2795	0.93750000	1.12500000	-0.16559140
2796	1.18750000	1.06250000	-0.17653959
2797	1.12500000	0.93750000	-0.16911046
2798	0.06250000	3.12500000	0.18416422
2799	0.06250000	3.18750000	0.18475073
2800	0.06250000	2.93750000	0.15679374
2801	0.06250000	2.87500000	0.13743891
2802	0.12500000	3.06250000	0.17536657
2803	0.12500000	3.18750000	0.18338221
2804	0.18750000	3.12500000	0.17458456
2805	0.12500000	2.93750000	0.14858260
2806	0.18750000	3.06250000	0.16715543
2807	0.87500000	3.93750000	0.17575758
2808	0.81250000	3.93750000	0.17908113
2809	1.06250000	3.93750000	0.16520039
2810	1.12500000	3.93750000	0.16500489
2811	0.93750000	3.87500000	0.16461388
2812	0.81250000	3.87500000	0.17673509
2813	0.87500000	3.81250000	0.16539589
2814	1.06250000	3.87500000	0.15562072
2815	0.93750000	3.81250000	0.15659824
2816	3.12500000	0.06250000	-0.00899316
2817	3.18750000	0.06250000	0.00215054
2818	2.93750000	0.06250000	-0.04046921
2819	2.87500000	0.06250000	-0.04711632
2820	3.06250000	0.12500000	-0.03069404
2821	3.18750000	0.12500000	-0.00586510
2822	3.12500000	0.18750000	-0.02756598
2823	2.93750000	0.12500000	-0.05102639
2824	3.06250000	0.18750000	-0.04125122
2825	3.93750000	0.87500000	0.05083089
2826	3.93750000	0.81250000	0.05708700
2827	3.93750000	1.06250000	0.03128055
2828	3.93750000	1.12500000	0.02697947

2829	3.87500000	0.93750000	0.02952102
2830	3.87500000	0.81250000	0.04477028
2831	3.81250000	0.87500000	0.02326491
2832	3.87500000	1.06250000	0.01622678
2833	3.81250000	0.93750000	0.01427175
2834	3.06250000	3.12500000	0.01485826
2835	3.12500000	3.06250000	0.02072336
2836	3.12500000	3.18750000	0.04125122
2837	3.18750000	3.12500000	0.04711632
2838	2.87500000	2.93750000	-0.04457478
2839	2.93750000	2.87500000	-0.04848485
2840	2.81250000	2.87500000	-0.05552297
2841	2.87500000	2.81250000	-0.05943304
2842	2.93750000	3.06250000	-0.01955034
2843	3.06250000	3.18750000	0.02893451
2844	2.93750000	3.12500000	-0.00566960
2845	3.06250000	2.93750000	-0.01759531
2846	3.18750000	3.06250000	0.04066471
2847	3.12500000	2.93750000	0.00234604
2848	2.81250000	2.93750000	-0.05259042
2849	2.87500000	3.06250000	-0.02776149
2850	2.93750000	2.81250000	-0.06041056
2851	3.06250000	2.87500000	-0.02952102
2852	3.06250000	3.93750000	0.18592375
2853	3.12500000	3.93750000	0.18572825
2854	2.87500000	3.93750000	0.18435973
2855	2.81250000	3.93750000	0.18396872
2856	2.93750000	3.87500000	0.17067449
2857	3.06250000	3.87500000	0.17165200
2858	2.93750000	3.81250000	0.15659824
2859	2.81250000	3.87500000	0.16911046
2860	2.87500000	3.81250000	0.15542522
2861	3.93750000	3.06250000	0.17751711
2862	3.93750000	3.12500000	0.17869013
2863	3.93750000	2.87500000	0.14252199
2864	3.93750000	2.81250000	0.13000978
2865	3.87500000	2.93750000	0.14897361
2866	3.87500000	3.06250000	0.17145650
2867	3.81250000	2.93750000	0.14291300
2868	3.87500000	2.81250000	0.11515152
2869	3.81250000	2.87500000	0.12160313
2870	0.87500000	1.93750000	-0.16950147
2871	0.93750000	2.06250000	-0.16832845
2872	0.81250000	1.93750000	-0.15894428
2873	0.87500000	2.06250000	-0.15796676
2874	1.06250000	1.93750000	-0.19042033
2875	1.12500000	2.06250000	-0.18709677
2876	1.12500000	1.93750000	-0.19100684
2877	1.18750000	2.06250000	-0.18768328
2878	0.93750000	1.87500000	-0.17888563
2879	0.81250000	1.87500000	-0.15855327
2880	0.87500000	1.81250000	-0.16793744
2881	1.06250000	1.87500000	-0.18924731
2882	0.93750000	1.81250000	-0.17771261
2883	1.06250000	2.12500000	-0.17438905
2884	0.93750000	2.12500000	-0.15640274
2885	1.06250000	2.18750000	-0.16226784
2886	1.18750000	2.12500000	-0.18260020
2887	1.12500000	2.18750000	-0.16989247

2888	0.87500000	2.93750000	0.01759531
2889	0.93750000	2.87500000	-0.00899316
2890	0.81250000	2.87500000	0.01915934
2891	0.87500000	2.81250000	-0.00762463
2892	1.06250000	3.12500000	-0.07253177
2893	1.12500000	3.06250000	0.00997067
2894	1.12500000	3.18750000	0.04066471
2895	1.18750000	3.12500000	0.02287390
2896	0.93750000	3.06250000	-0.07077224
2897	0.81250000	2.93750000	0.03225806
2898	0.87500000	3.06250000	0.04398827
2899	1.06250000	3.18750000	-0.05904203
2900	0.93750000	3.12500000	-0.05747801
2901	1.06250000	2.93750000	-0.01485826
2902	0.93750000	2.81250000	-0.02111437
2903	1.06250000	2.87500000	-0.02678397
2904	1.18750000	3.06250000	0.00566960
2905	1.12500000	2.93750000	-0.01915934
2906	1.93750000	0.87500000	-0.18572825
2907	2.06250000	0.93750000	-0.19413490
2908	1.93750000	0.81250000	-0.18123167
2909	2.06250000	0.87500000	-0.18983382
2910	1.93750000	1.06250000	-0.19765396
2911	2.06250000	1.12500000	-0.19902248
2912	1.93750000	1.12500000	-0.19765396
2913	2.06250000	1.18750000	-0.19902248
2914	1.87500000	0.93750000	-0.18866080
2915	1.87500000	0.81250000	-0.17673509
2916	1.81250000	0.87500000	-0.17947214
2917	1.87500000	1.06250000	-0.19608993
2918	1.81250000	0.93750000	-0.18690127
2919	2.12500000	1.06250000	-0.19843597
2920	2.12500000	0.93750000	-0.19374389
2921	2.18750000	1.06250000	-0.19804497
2922	2.12500000	1.18750000	-0.19882698
2923	2.18750000	1.12500000	-0.19843597
2924	2.93750000	0.87500000	-0.17927664
2925	2.87500000	0.93750000	-0.14037146
2926	2.87500000	0.81250000	-0.17693060
2927	2.81250000	0.87500000	-0.18787879
2928	3.12500000	1.06250000	-0.16637341
2929	3.06250000	1.12500000	-0.18181818
2930	3.18750000	1.12500000	-0.15542522
2931	3.12500000	1.18750000	-0.17086999
2932	3.06250000	0.93750000	-0.16656891
2933	2.93750000	0.81250000	-0.16871945
2934	3.06250000	0.87500000	-0.15601173
2935	3.18750000	1.06250000	-0.15112414
2936	3.12500000	0.93750000	-0.15151515
2937	2.93750000	1.06250000	-0.14310850
2938	2.81250000	0.93750000	-0.14076246
2939	2.87500000	1.06250000	-0.14349951
2940	3.06250000	1.18750000	-0.18201369
2941	2.93750000	1.12500000	-0.19335288
2942	2.06250000	2.93750000	-0.12336266
2943	1.93750000	2.87500000	-0.13137830
2944	2.06250000	2.87500000	-0.13157380
2945	1.93750000	2.81250000	-0.13958944
2946	2.06250000	3.12500000	-0.04868035

2947	1.93750000	3.06250000	-0.08758553
2948	2.06250000	3.18750000	-0.05337243
2949	1.93750000	3.12500000	-0.06725318
2950	2.12500000	3.06250000	-0.06549365
2951	2.12500000	2.93750000	-0.11984360
2952	2.18750000	3.06250000	-0.08699902
2953	2.12500000	3.18750000	-0.02404692
2954	2.18750000	3.12500000	-0.04086022
2955	1.87500000	2.93750000	-0.11241447
2956	1.87500000	2.81250000	-0.13587488
2957	1.81250000	2.87500000	-0.11691105
2958	1.87500000	3.06250000	-0.07683284
2959	1.81250000	2.93750000	-0.10166178
2960	2.93750000	2.06250000	-0.18690127
2961	2.87500000	1.93750000	-0.19648094
2962	2.87500000	2.06250000	-0.18729228
2963	2.81250000	1.93750000	-0.19687195
2964	3.12500000	2.06250000	-0.16500489
2965	3.06250000	1.93750000	-0.18475073
2966	3.18750000	2.06250000	-0.15386119
2967	3.12500000	1.93750000	-0.17360704
2968	3.06250000	2.12500000	-0.16676442
2969	2.93750000	2.12500000	-0.17771261
2970	3.06250000	2.18750000	-0.15738025
2971	3.18750000	2.12500000	-0.14506354
2972	3.12500000	2.18750000	-0.14682307
2973	2.93750000	1.87500000	-0.19608993
2974	2.81250000	1.87500000	-0.19687195
2975	2.87500000	1.81250000	-0.19628543
2976	3.06250000	1.87500000	-0.18455523
2977	2.93750000	1.81250000	-0.19589443
2978	0.06250000	0.43750000	0.08445748
2979	0.06250000	0.37500000	0.09638319
2980	0.06250000	0.62500000	0.04848485
2981	0.06250000	0.68750000	0.03636364
2982	0.12500000	0.56250000	0.05024438
2983	0.12500000	0.43750000	0.07390029
2984	0.18750000	0.56250000	0.03988270
2985	0.12500000	0.68750000	0.02639296
2986	0.18750000	0.62500000	0.02815249
2987	0.43750000	0.06250000	0.13743891
2988	0.37500000	0.06250000	0.11964809
2989	0.62500000	0.06250000	0.09384164
2990	0.68750000	0.06250000	0.04652981
2991	0.56250000	0.12500000	0.10087977
2992	0.43750000	0.12500000	0.09736070
2993	0.56250000	0.18750000	0.06060606
2994	0.68750000	0.12500000	0.03929619
2995	0.62500000	0.18750000	0.04633431
2996	0.37500000	0.43750000	-0.04222874
2997	0.43750000	0.37500000	-0.04398827
2998	0.31250000	0.37500000	0.00508309
2999	0.37500000	0.31250000	0.00351906
3000	0.56250000	0.62500000	-0.05826002
3001	0.62500000	0.56250000	-0.08582600
3002	0.62500000	0.68750000	-0.07996090
3003	0.68750000	0.62500000	-0.08250244
3004	0.43750000	0.56250000	-0.02619746
3005	0.31250000	0.43750000	0.01818182

3006	0.37500000	0.56250000	-0.01583578
3007	0.56250000	0.43750000	-0.05552297
3008	0.43750000	0.31250000	0.01485826
3009	0.56250000	0.37500000	-0.02209189
3010	0.56250000	0.68750000	-0.04496579
3011	0.43750000	0.62500000	-0.01290323
3012	0.68750000	0.56250000	-0.07507331
3013	0.62500000	0.43750000	-0.04496579
3014	0.06250000	3.62500000	0.19550342
3015	0.06250000	3.68750000	0.19374389
3016	0.06250000	3.43750000	0.19178886
3017	0.06250000	3.37500000	0.18885630
3018	0.12500000	3.56250000	0.19354839
3019	0.12500000	3.68750000	0.19433040
3020	0.18750000	3.62500000	0.19237537
3021	0.12500000	3.43750000	0.18807429
3022	0.18750000	3.56250000	0.18983382
3023	0.37500000	3.93750000	0.19667644
3024	0.31250000	3.93750000	0.19608993
3025	0.56250000	3.93750000	0.19257087
3026	0.62500000	3.93750000	0.18924731
3027	0.43750000	3.87500000	0.19472141
3028	0.31250000	3.87500000	0.19550342
3029	0.37500000	3.81250000	0.19354839
3030	0.56250000	3.87500000	0.19002933
3031	0.43750000	3.81250000	0.19217986
3032	3.62500000	0.06250000	0.08035191
3033	3.68750000	0.06250000	0.09110459
3034	3.43750000	0.06250000	0.04652981
3035	3.37500000	0.06250000	0.03558162
3036	3.56250000	0.12500000	0.06177908
3037	3.68750000	0.12500000	0.08484848
3038	3.62500000	0.18750000	0.06608016
3039	3.43750000	0.12500000	0.03851417
3040	3.56250000	0.18750000	0.05376344
3041	3.93750000	0.37500000	0.10244379
3042	3.93750000	0.31250000	0.10869990
3043	3.93750000	0.56250000	0.08172043
3044	3.93750000	0.62500000	0.07546432
3045	3.87500000	0.43750000	0.08367546
3046	3.87500000	0.31250000	0.09794721
3047	3.81250000	0.37500000	0.07917889
3048	3.87500000	0.56250000	0.06940371
3049	3.81250000	0.43750000	0.07116325
3050	3.56250000	3.62500000	0.18905181
3051	3.62500000	3.56250000	0.18787879
3052	3.62500000	3.68750000	0.19081134
3053	3.68750000	3.62500000	0.18963832
3054	3.37500000	3.43750000	0.14682307
3055	3.43750000	3.37500000	0.15268817
3056	3.31250000	3.37500000	0.12043011
3057	3.37500000	3.31250000	0.12629521
3058	3.43750000	3.56250000	0.17497556
3059	3.56250000	3.68750000	0.19081134
3060	3.43750000	3.62500000	0.17673509
3061	3.56250000	3.43750000	0.17966764
3062	3.68750000	3.56250000	0.18846530
3063	3.62500000	3.43750000	0.18025415
3064	3.31250000	3.43750000	0.13450635

3065	3.37500000	3.56250000	0.16265885
3066	3.43750000	3.31250000	0.14623656
3067	3.56250000	3.37500000	0.17321603
3068	3.56250000	3.93750000	0.19745846
3069	3.62500000	3.93750000	0.19745846
3070	3.37500000	3.93750000	0.18533724
3071	3.31250000	3.93750000	0.18553275
3072	3.43750000	3.87500000	0.18338221
3073	3.56250000	3.87500000	0.19569892
3074	3.43750000	3.81250000	0.18181818
3075	3.31250000	3.87500000	0.17126100
3076	3.37500000	3.81250000	0.16950147
3077	3.93750000	3.56250000	0.19120235
3078	3.93750000	3.62500000	0.19217986
3079	3.93750000	3.37500000	0.18260020
3080	3.93750000	3.31250000	0.18162268
3081	3.87500000	3.43750000	0.18299120
3082	3.87500000	3.56250000	0.19042033
3083	3.81250000	3.43750000	0.18240469
3084	3.87500000	3.31250000	0.17556207
3085	3.81250000	3.37500000	0.17595308
3086	0.06250000	1.62500000	-0.05689150
3087	0.06250000	1.68750000	-0.05747801
3088	0.06250000	1.43750000	-0.02971652
3089	0.06250000	1.37500000	-0.02893451
3090	0.12500000	1.56250000	-0.04144673
3091	0.12500000	1.68750000	-0.04261975
3092	0.18750000	1.62500000	-0.05219941
3093	0.12500000	1.43750000	-0.04007820
3094	0.18750000	1.56250000	-0.05161290
3095	0.06250000	2.43750000	0.01994135
3096	0.06250000	2.37500000	0.01309873
3097	0.06250000	2.62500000	0.05962854
3098	0.06250000	2.68750000	0.07898338
3099	0.12500000	2.56250000	0.03812317
3100	0.12500000	2.43750000	0.01798631
3101	0.18750000	2.56250000	0.03616813
3102	0.12500000	2.68750000	0.07077224
3103	0.18750000	2.62500000	0.04946237
3104	0.37500000	1.93750000	-0.08621701
3105	0.43750000	2.06250000	-0.08191593
3106	0.31250000	1.93750000	-0.07605083
3107	0.37500000	2.06250000	-0.07194526
3108	0.56250000	1.93750000	-0.11671554
3109	0.62500000	2.06250000	-0.11573803
3110	0.62500000	1.93750000	-0.12727273
3111	0.68750000	2.06250000	-0.12629521
3112	0.43750000	1.87500000	-0.09560117
3113	0.31250000	1.87500000	-0.07526882
3114	0.37500000	1.81250000	-0.08484848
3115	0.56250000	2.12500000	-0.09032258
3116	0.43750000	2.12500000	-0.06686217
3117	0.56250000	2.18750000	-0.07526882
3118	0.56250000	1.87500000	-0.11632454
3119	0.43750000	1.81250000	-0.09501466
3120	0.68750000	2.12500000	-0.11436950
3121	0.62500000	2.18750000	-0.08875855
3122	0.37500000	2.43750000	0.00997067
3123	0.43750000	2.37500000	-0.00703812

3124	0.31250000	2.37500000	0.00508309
3125	0.37500000	2.31250000	-0.01192571
3126	0.56250000	2.62500000	0.02463343
3127	0.62500000	2.56250000	-0.00195503
3128	0.62500000	2.68750000	0.02326491
3129	0.68750000	2.62500000	-0.00332356
3130	0.43750000	2.56250000	0.02815249
3131	0.31250000	2.43750000	0.01212121
3132	0.37500000	2.56250000	0.03030303
3133	0.56250000	2.43750000	-0.01544477
3134	0.43750000	2.31250000	-0.02189638
3135	0.56250000	2.37500000	-0.03049853
3136	0.56250000	2.68750000	0.03792766
3137	0.43750000	2.62500000	0.04144673
3138	0.68750000	2.56250000	-0.01524927
3139	0.62500000	2.43750000	-0.02893451
3140	1.62500000	0.06250000	-0.10420332
3141	1.68750000	0.06250000	-0.10713587
3142	1.43750000	0.06250000	-0.09247312
3143	1.37500000	0.06250000	-0.08778104
3144	1.56250000	0.12500000	-0.10576735
3145	1.68750000	0.12500000	-0.11300098
3146	1.62500000	0.18750000	-0.11456500
3147	1.43750000	0.12500000	-0.09677419
3148	1.56250000	0.18750000	-0.11026393
3149	2.43750000	0.06250000	-0.09423265
3150	2.37500000	0.06250000	-0.10087977
3151	2.62500000	0.06250000	-0.07292278
3152	2.68750000	0.06250000	-0.06647116
3153	2.56250000	0.12500000	-0.09169110
3154	2.43750000	0.12500000	-0.10654936
3155	2.56250000	0.18750000	-0.10400782
3156	2.68750000	0.12500000	-0.07702835
3157	2.62500000	0.18750000	-0.09579668
3158	1.93750000	0.37500000	-0.15014663
3159	2.06250000	0.43750000	-0.16031281
3160	1.93750000	0.31250000	-0.14584555
3161	2.06250000	0.37500000	-0.15581623
3162	1.93750000	0.56250000	-0.16344086
3163	2.06250000	0.62500000	-0.17184751
3164	1.93750000	0.62500000	-0.16774194
3165	2.06250000	0.68750000	-0.17634409
3166	1.87500000	0.43750000	-0.15014663
3167	1.87500000	0.31250000	-0.14134897
3168	1.81250000	0.37500000	-0.14115347
3169	2.12500000	0.56250000	-0.16871945
3170	2.12500000	0.43750000	-0.16148583
3171	2.18750000	0.56250000	-0.16989247
3172	1.87500000	0.56250000	-0.15894428
3173	1.81250000	0.43750000	-0.14565005
3174	2.12500000	0.68750000	-0.17595308
3175	2.18750000	0.62500000	-0.17262952
3176	2.43750000	0.37500000	-0.15542522
3177	2.37500000	0.43750000	-0.16656891
3178	2.37500000	0.31250000	-0.14975562
3179	2.31250000	0.37500000	-0.16070381
3180	2.62500000	0.56250000	-0.16911046
3181	2.56250000	0.62500000	-0.18005865
3182	2.68750000	0.62500000	-0.17145650

3183	2.62500000	0.68750000	-0.18240469
3184	2.56250000	0.43750000	-0.15288368
3185	2.43750000	0.31250000	-0.14310850
3186	2.56250000	0.37500000	-0.14056696
3187	2.43750000	0.56250000	-0.17497556
3188	2.31250000	0.43750000	-0.16520039
3189	2.37500000	0.56250000	-0.17380254
3190	2.68750000	0.56250000	-0.15933529
3191	2.62500000	0.43750000	-0.14467253
3192	2.56250000	0.68750000	-0.18123167
3193	2.43750000	0.62500000	-0.17771261
3194	1.56250000	1.62500000	-0.18924731
3195	1.62500000	1.56250000	-0.18963832
3196	1.62500000	1.68750000	-0.19100684
3197	1.68750000	1.62500000	-0.19159335
3198	1.37500000	1.43750000	-0.18435973
3199	1.43750000	1.37500000	-0.18475073
3200	1.31250000	1.37500000	-0.18260020
3201	1.37500000	1.31250000	-0.18318671
3202	1.43750000	1.56250000	-0.18709677
3203	1.56250000	1.68750000	-0.19042033
3204	1.43750000	1.62500000	-0.18807429
3205	1.56250000	1.43750000	-0.18787879
3206	1.68750000	1.56250000	-0.19139785
3207	1.62500000	1.43750000	-0.18944282
3208	1.31250000	1.43750000	-0.18377322
3209	1.37500000	1.56250000	-0.18651026
3210	1.43750000	1.31250000	-0.18475073
3211	1.56250000	1.37500000	-0.18787879
3212	2.37500000	2.43750000	-0.13313783
3213	2.43750000	2.37500000	-0.13489736
3214	2.31250000	2.37500000	-0.14389052
3215	2.37500000	2.31250000	-0.14565005
3216	2.56250000	2.62500000	-0.09951124
3217	2.62500000	2.56250000	-0.10342131
3218	2.62500000	2.68750000	-0.08856305
3219	2.68750000	2.62500000	-0.09247312
3220	2.43750000	2.56250000	-0.11945259
3221	2.31250000	2.43750000	-0.14193548
3222	2.37500000	2.56250000	-0.12825024
3223	2.56250000	2.43750000	-0.12512219
3224	2.43750000	2.31250000	-0.14545455
3225	2.56250000	2.37500000	-0.13567937
3226	2.56250000	2.68750000	-0.09677419
3227	2.43750000	2.62500000	-0.11652004
3228	2.68750000	2.56250000	-0.10459433
3229	2.62500000	2.43750000	-0.12609971
3230	1.56250000	1.93750000	-0.19491691
3231	1.62500000	2.06250000	-0.19452590
3232	1.62500000	1.93750000	-0.19530792
3233	1.68750000	2.06250000	-0.19511241
3234	1.37500000	1.93750000	-0.19296188
3235	1.43750000	2.06250000	-0.18963832
3236	1.31250000	1.93750000	-0.19257087
3237	1.37500000	2.06250000	-0.18905181
3238	1.43750000	1.87500000	-0.19257087
3239	1.56250000	1.87500000	-0.19374389
3240	1.43750000	1.81250000	-0.19159335
3241	1.56250000	2.12500000	-0.18885630

3242	1.68750000	2.12500000	-0.19315738
3243	1.62500000	2.18750000	-0.18748778
3244	1.31250000	1.87500000	-0.19139785
3245	1.37500000	1.81250000	-0.19081134
3246	1.43750000	2.12500000	-0.18455523
3247	1.56250000	2.18750000	-0.18377322
3248	1.93750000	1.56250000	-0.19804497
3249	2.06250000	1.62500000	-0.19960899
3250	1.93750000	1.62500000	-0.19824047
3251	2.06250000	1.68750000	-0.19980450
3252	1.93750000	1.37500000	-0.19765396
3253	2.06250000	1.43750000	-0.19921799
3254	1.93750000	1.31250000	-0.19765396
3255	2.06250000	1.37500000	-0.19902248
3256	1.87500000	1.43750000	-0.19608993
3257	1.87500000	1.56250000	-0.19628543
3258	1.81250000	1.43750000	-0.19452590
3259	2.12500000	1.56250000	-0.19921799
3260	2.12500000	1.68750000	-0.19941349
3261	2.18750000	1.62500000	-0.19902248
3262	1.87500000	1.31250000	-0.19608993
3263	1.81250000	1.37500000	-0.19452590
3264	2.12500000	1.43750000	-0.19902248
3265	2.18750000	1.56250000	-0.19902248
3266	2.06250000	2.43750000	-0.17751711
3267	1.93750000	2.37500000	-0.18768328
3268	2.06250000	2.37500000	-0.17947214
3269	1.93750000	2.31250000	-0.18963832
3270	2.06250000	2.62500000	-0.11456500
3271	1.93750000	2.56250000	-0.12238514
3272	2.06250000	2.68750000	-0.10615836
3273	1.93750000	2.62500000	-0.11417400
3274	2.12500000	2.56250000	-0.16402737
3275	2.12500000	2.43750000	-0.16871945
3276	2.18750000	2.56250000	-0.15503421
3277	1.87500000	2.43750000	-0.18201369
3278	1.87500000	2.31250000	-0.18905181
3279	1.81250000	2.37500000	-0.18338221
3280	2.12500000	2.68750000	-0.15288368
3281	2.18750000	2.62500000	-0.15229717
3282	1.87500000	2.56250000	-0.16871945
3283	1.81250000	2.43750000	-0.17829912
3284	2.43750000	2.06250000	-0.18768328
3285	2.37500000	1.93750000	-0.19843597
3286	2.37500000	2.06250000	-0.18787879
3287	2.31250000	1.93750000	-0.19882698
3288	2.62500000	2.06250000	-0.18826979
3289	2.56250000	1.93750000	-0.19784946
3290	2.68750000	2.06250000	-0.18826979
3291	2.62500000	1.93750000	-0.19765396
3292	2.56250000	2.12500000	-0.17790811
3293	2.43750000	2.12500000	-0.17712610
3294	2.56250000	2.18750000	-0.16735093
3295	2.43750000	1.87500000	-0.19824047
3296	2.31250000	1.87500000	-0.19882698
3297	2.37500000	1.81250000	-0.19843597
3298	2.68750000	2.12500000	-0.17888563
3299	2.62500000	2.18750000	-0.16832845
3300	2.56250000	1.87500000	-0.19784946

3301	2.43750000	1.81250000	-0.19824047
3302	1.56250000	3.93750000	0.13704790
3303	1.62500000	3.93750000	0.16207234
3304	1.37500000	3.93750000	0.16285435
3305	1.31250000	3.93750000	0.16304985
3306	1.43750000	3.87500000	0.12942326
3307	1.56250000	3.87500000	0.12883675
3308	1.43750000	3.81250000	0.12121212
3309	1.31250000	3.87500000	0.15522972
3310	1.37500000	3.81250000	0.14682307
3311	2.37500000	3.93750000	0.17458456
3312	2.31250000	3.93750000	0.17106549
3313	2.56250000	3.93750000	0.18240469
3314	2.62500000	3.93750000	0.18279570
3315	2.43750000	3.87500000	0.16304985
3316	2.31250000	3.87500000	0.15542522
3317	2.37500000	3.81250000	0.14389052
3318	2.56250000	3.87500000	0.16754643
3319	2.43750000	3.81250000	0.14799609
3320	1.56250000	3.62500000	0.11749756
3321	1.62500000	3.56250000	-0.02013685
3322	1.62500000	3.68750000	0.12570870
3323	1.68750000	3.62500000	0.10987292
3324	1.37500000	3.43750000	-0.00742913
3325	1.43750000	3.37500000	-0.02521994
3326	1.31250000	3.37500000	-0.02033236
3327	1.37500000	3.31250000	-0.03792766
3328	1.43750000	3.56250000	0.11300098
3329	1.56250000	3.68750000	0.12883675
3330	1.43750000	3.62500000	0.12121212
3331	1.56250000	3.43750000	-0.04242424
3332	1.68750000	3.56250000	-0.02463343
3333	1.62500000	3.43750000	-0.04985337
3334	1.31250000	3.43750000	0.09247312
3335	1.37500000	3.56250000	0.11358749
3336	1.43750000	3.31250000	0.05708700
3337	1.56250000	3.37500000	0.06236559
3338	2.06250000	3.62500000	0.07878788
3339	1.93750000	3.56250000	0.06725318
3340	2.06250000	3.68750000	0.09442815
3341	1.93750000	3.62500000	0.08289345
3342	2.06250000	3.43750000	0.02795699
3343	1.93750000	3.37500000	0.01407625
3344	2.06250000	3.37500000	0.00762463
3345	1.93750000	3.31250000	-0.00625611
3346	2.12500000	3.56250000	0.06744868
3347	2.12500000	3.68750000	0.09794721
3348	2.18750000	3.62500000	0.08660802
3349	1.87500000	3.43750000	0.04203324
3350	1.87500000	3.56250000	0.07487781
3351	1.81250000	3.43750000	0.04946237
3352	2.12500000	3.43750000	0.03225806
3353	2.18750000	3.56250000	0.07174976
3354	1.87500000	3.31250000	0.00449658
3355	1.81250000	3.37500000	0.03225806
3356	3.93750000	1.56250000	-0.00312805
3357	3.93750000	1.62500000	-0.00762463
3358	3.93750000	1.37500000	0.00957967
3359	3.93750000	1.31250000	0.01407625

3360	3.87500000	1.43750000	-0.00977517
3361	3.87500000	1.56250000	-0.01837732
3362	3.81250000	1.43750000	-0.02482893
3363	3.87500000	1.31250000	-0.00117302
3364	3.81250000	1.37500000	-0.02072336
3365	3.93750000	2.37500000	0.04574780
3366	3.93750000	2.31250000	0.03323558
3367	3.93750000	2.56250000	0.07956989
3368	3.93750000	2.62500000	0.09227761
3369	3.87500000	2.43750000	0.04340176
3370	3.87500000	2.31250000	0.02209189
3371	3.81250000	2.37500000	0.01994135
3372	3.87500000	2.56250000	0.06490714
3373	3.81250000	2.43750000	0.02873900
3374	3.62500000	1.56250000	-0.07917889
3375	3.56250000	1.62500000	-0.09462366
3376	3.68750000	1.62500000	-0.07448680
3377	3.62500000	1.68750000	-0.08387097
3378	3.43750000	1.37500000	-0.11163245
3379	3.37500000	1.43750000	-0.12727273
3380	3.37500000	1.31250000	-0.12258065
3381	3.31250000	1.37500000	-0.13822092
3382	3.56250000	1.43750000	-0.08563050
3383	3.68750000	1.56250000	-0.07018573
3384	3.62500000	1.43750000	-0.07038123
3385	3.43750000	1.56250000	-0.11671554
3386	3.56250000	1.68750000	-0.09501466
3387	3.43750000	1.62500000	-0.11691105
3388	3.43750000	1.31250000	-0.10733138
3389	3.56250000	1.37500000	-0.08113392
3390	3.31250000	1.43750000	-0.13861193
3391	3.37500000	1.56250000	-0.12785924
3392	3.62500000	2.06250000	-0.07096774
3393	3.56250000	1.93750000	-0.09579668
3394	3.68750000	2.06250000	-0.06138807
3395	3.62500000	1.93750000	-0.08484848
3396	3.43750000	2.06250000	-0.10948192
3397	3.37500000	1.93750000	-0.12942326
3398	3.37500000	2.06250000	-0.12062561
3399	3.31250000	1.93750000	-0.14056696
3400	3.56250000	2.12500000	-0.07311828
3401	3.68750000	2.12500000	-0.04731183
3402	3.62500000	2.18750000	-0.04946237
3403	3.43750000	1.87500000	-0.11788856
3404	3.56250000	1.87500000	-0.09560117
3405	3.43750000	1.81250000	-0.11749756
3406	3.43750000	2.12500000	-0.10048876
3407	3.56250000	2.18750000	-0.06568915
3408	3.31250000	1.87500000	-0.14037146
3409	3.37500000	1.81250000	-0.12883675
3410	0.37500000	0.93750000	-0.06177908
3411	0.43750000	1.06250000	-0.08465298
3412	0.31250000	0.93750000	-0.05161290
3413	0.37500000	1.06250000	-0.07468231
3414	0.56250000	0.93750000	-0.09188661
3415	0.62500000	1.06250000	-0.11476051
3416	0.62500000	0.93750000	-0.10185728
3417	0.68750000	1.06250000	-0.12453568
3418	0.43750000	0.87500000	-0.06001955

3419	0.31250000	0.87500000	-0.03968719
3420	0.37500000	0.81250000	-0.03792766
3421	0.56250000	0.87500000	-0.08015640
3422	0.43750000	0.81250000	-0.04809384
3423	0.56250000	1.12500000	-0.10576735
3424	0.43750000	1.12500000	-0.08563050
3425	0.56250000	1.18750000	-0.10674487
3426	0.68750000	1.12500000	-0.12570870
3427	0.62500000	1.18750000	-0.11691105
3428	0.37500000	1.43750000	-0.03108504
3429	0.43750000	1.37500000	-0.04027370
3430	0.31250000	1.37500000	-0.04516129
3431	0.37500000	1.31250000	-0.05415445
3432	0.56250000	1.62500000	-0.06412512
3433	0.62500000	1.56250000	-0.07350929
3434	0.62500000	1.68750000	-0.10029326
3435	0.68750000	1.62500000	-0.10967742
3436	0.43750000	1.56250000	-0.04261975
3437	0.31250000	1.43750000	-0.04594330
3438	0.37500000	1.56250000	-0.05747801
3439	0.56250000	1.68750000	-0.08954057
3440	0.43750000	1.62500000	-0.06823069
3441	0.56250000	1.43750000	-0.06119257
3442	0.43750000	1.31250000	-0.06412512
3443	0.56250000	1.37500000	-0.08504399
3444	0.68750000	1.56250000	-0.10830890
3445	0.62500000	1.43750000	-0.09599218
3446	0.93750000	0.37500000	-0.08426197
3447	1.06250000	0.43750000	-0.10889541
3448	0.93750000	0.31250000	-0.07683284
3449	1.06250000	0.37500000	-0.10166178
3450	0.93750000	0.56250000	-0.10772239
3451	1.06250000	0.62500000	-0.13079179
3452	0.93750000	0.62500000	-0.11476051
3453	1.06250000	0.68750000	-0.13802542
3454	0.87500000	0.43750000	-0.07741935
3455	0.87500000	0.31250000	-0.06119257
3456	0.81250000	0.37500000	-0.05415445
3457	0.87500000	0.56250000	-0.09325513
3458	0.81250000	0.43750000	-0.06295210
3459	1.12500000	0.56250000	-0.12512219
3460	1.12500000	0.43750000	-0.11065494
3461	1.18750000	0.56250000	-0.12668622
3462	1.12500000	0.68750000	-0.13958944
3463	1.18750000	0.62500000	-0.13391984
3464	1.43750000	0.37500000	-0.14252199
3465	1.37500000	0.43750000	-0.14545455
3466	1.37500000	0.31250000	-0.11964809
3467	1.31250000	0.37500000	-0.12258065
3468	1.62500000	0.56250000	-0.16930596
3469	1.56250000	0.62500000	-0.18455523
3470	1.68750000	0.62500000	-0.16422287
3471	1.62500000	0.68750000	-0.16695992
3472	1.56250000	0.43750000	-0.15581623
3473	1.43750000	0.31250000	-0.12394917
3474	1.56250000	0.37500000	-0.13724340
3475	1.68750000	0.56250000	-0.15972630
3476	1.62500000	0.43750000	-0.14623656
3477	1.43750000	0.56250000	-0.17399804

3478	1.31250000	0.43750000	-0.12981427
3479	1.37500000	0.56250000	-0.14584555
3480	1.56250000	0.68750000	-0.17771261
3481	1.43750000	0.62500000	-0.16715543
3482	1.06250000	1.43750000	-0.18142717
3483	0.93750000	1.37500000	-0.16989247
3484	1.06250000	1.37500000	-0.18044966
3485	0.93750000	1.31250000	-0.16832845
3486	1.06250000	1.62500000	-0.18494624
3487	0.93750000	1.56250000	-0.17321603
3488	1.06250000	1.68750000	-0.18592375
3489	0.93750000	1.62500000	-0.17438905
3490	1.12500000	1.56250000	-0.18435973
3491	1.12500000	1.43750000	-0.18220919
3492	1.18750000	1.56250000	-0.18475073
3493	1.12500000	1.68750000	-0.18651026
3494	1.18750000	1.62500000	-0.18592375
3495	0.87500000	1.43750000	-0.16109482
3496	0.87500000	1.31250000	-0.15855327
3497	0.81250000	1.37500000	-0.15014663
3498	0.87500000	1.56250000	-0.16324536
3499	0.81250000	1.43750000	-0.15112414
3500	1.43750000	1.06250000	-0.18455523
3501	1.37500000	0.93750000	-0.17556207
3502	1.37500000	1.06250000	-0.18299120
3503	1.31250000	0.93750000	-0.17399804
3504	1.62500000	1.06250000	-0.18944282
3505	1.56250000	0.93750000	-0.18025415
3506	1.68750000	1.06250000	-0.19120235
3507	1.62500000	0.93750000	-0.18201369
3508	1.56250000	1.12500000	-0.18787879
3509	1.43750000	1.12500000	-0.18475073
3510	1.56250000	1.18750000	-0.18787879
3511	1.68750000	1.12500000	-0.19120235
3512	1.62500000	1.18750000	-0.18944282
3513	1.43750000	0.87500000	-0.16969697
3514	1.31250000	0.87500000	-0.16676442
3515	1.37500000	0.81250000	-0.16089932
3516	1.56250000	0.87500000	-0.17282502
3517	1.43750000	0.81250000	-0.16226784
3518	0.37500000	3.43750000	0.18651026
3519	0.43750000	3.37500000	0.17771261
3520	0.31250000	3.37500000	0.18416422
3521	0.37500000	3.31250000	0.17536657
3522	0.56250000	3.62500000	0.18611926
3523	0.62500000	3.56250000	0.17497556
3524	0.62500000	3.68750000	0.18220919
3525	0.68750000	3.62500000	0.17106549
3526	0.43750000	3.56250000	0.18826979
3527	0.31250000	3.43750000	0.18475073
3528	0.37500000	3.56250000	0.18651026
3529	0.56250000	3.68750000	0.18553275
3530	0.43750000	3.62500000	0.18768328
3531	0.56250000	3.43750000	0.16832845
3532	0.43750000	3.31250000	0.16715543
3533	0.56250000	3.37500000	0.15757576
3534	0.68750000	3.56250000	0.16324536
3535	0.62500000	3.43750000	0.15640274
3536	0.37500000	2.93750000	0.11554252

3537	0.43750000	3.06250000	0.08426197
3538	0.31250000	2.93750000	0.09892473
3539	0.37500000	3.06250000	0.11749756
3540	0.56250000	2.93750000	0.04066471
3541	0.62500000	3.06250000	0.05259042
3542	0.62500000	2.93750000	0.05122190
3543	0.68750000	3.06250000	0.06295210
3544	0.43750000	2.87500000	0.09403715
3545	0.31250000	2.87500000	0.12942326
3546	0.37500000	2.81250000	0.10791789
3547	0.56250000	2.87500000	0.05259042
3548	0.43750000	2.81250000	0.05591398
3549	0.56250000	3.12500000	0.07468231
3550	0.43750000	3.12500000	0.11671554
3551	0.56250000	3.18750000	0.10713587
3552	0.68750000	3.12500000	0.07624633
3553	0.62500000	3.18750000	0.09833822
3554	1.06250000	3.62500000	0.12609971
3555	0.93750000	3.56250000	0.12707722
3556	1.06250000	3.68750000	0.13294233
3557	0.93750000	3.62500000	0.13391984
3558	1.06250000	3.43750000	0.09657869
3559	0.93750000	3.37500000	0.09833822
3560	1.06250000	3.37500000	0.08211144
3561	0.93750000	3.31250000	0.08387097
3562	1.12500000	3.56250000	0.11769306
3563	1.12500000	3.68750000	0.13294233
3564	1.18750000	3.62500000	0.12453568
3565	1.12500000	3.43750000	0.09481916
3566	1.18750000	3.56250000	0.11612903
3567	0.87500000	3.43750000	0.12043011
3568	0.87500000	3.56250000	0.13489736
3569	0.81250000	3.43750000	0.12805474
3570	0.87500000	3.31250000	0.09833822
3571	0.81250000	3.37500000	0.12043011
3572	3.43750000	0.37500000	0.00566960
3573	3.37500000	0.43750000	-0.01622678
3574	3.37500000	0.31250000	0.00273705
3575	3.31250000	0.37500000	-0.01896383
3576	3.62500000	0.56250000	0.01935484
3577	3.56250000	0.62500000	-0.00195503
3578	3.68750000	0.62500000	0.02580645
3579	3.62500000	0.68750000	0.00449658
3580	3.56250000	0.43750000	0.02130987
3581	3.43750000	0.31250000	0.01407625
3582	3.56250000	0.37500000	0.02952102
3583	3.68750000	0.56250000	0.03206256
3584	3.62500000	0.43750000	0.03382209
3585	3.43750000	0.56250000	-0.02189638
3586	3.31250000	0.43750000	-0.02952102
3587	3.37500000	0.56250000	-0.03538612
3588	3.56250000	0.68750000	-0.01055718
3589	3.43750000	0.62500000	-0.03049853
3590	2.93750000	0.37500000	-0.04926686
3591	3.06250000	0.43750000	-0.03968719
3592	2.93750000	0.31250000	-0.06080156
3593	3.06250000	0.37500000	-0.05122190
3594	2.93750000	0.56250000	-0.08269795
3595	3.06250000	0.62500000	-0.06979472

3596	2.93750000	0.62500000	-0.11515152
3597	3.06250000	0.68750000	-0.10224829
3598	2.87500000	0.43750000	-0.06803519
3599	2.87500000	0.31250000	-0.06725318
3600	2.81250000	0.37500000	-0.08582600
3601	2.87500000	0.56250000	-0.11280547
3602	2.81250000	0.43750000	-0.09814272
3603	3.12500000	0.56250000	-0.04555230
3604	3.12500000	0.43750000	-0.04809384
3605	3.18750000	0.56250000	-0.05376344
3606	3.12500000	0.68750000	-0.08699902
3607	3.18750000	0.62500000	-0.06275660
3608	3.62500000	1.06250000	-0.05063539
3609	3.56250000	0.93750000	-0.05259042
3610	3.68750000	1.06250000	-0.03245357
3611	3.62500000	0.93750000	-0.03421310
3612	3.43750000	1.06250000	-0.09638319
3613	3.37500000	0.93750000	-0.09677419
3614	3.37500000	1.06250000	-0.10850440
3615	3.31250000	0.93750000	-0.10889541
3616	3.56250000	1.12500000	-0.07018573
3617	3.68750000	1.12500000	-0.03675464
3618	3.62500000	1.18750000	-0.05630499
3619	3.43750000	1.12500000	-0.09755621
3620	3.56250000	1.18750000	-0.07155425
3621	3.43750000	0.87500000	-0.07253177
3622	3.56250000	0.87500000	-0.04046921
3623	3.43750000	0.81250000	-0.06041056
3624	3.31250000	0.87500000	-0.09833822
3625	3.37500000	0.81250000	-0.07409580
3626	3.06250000	3.43750000	0.10205279
3627	2.93750000	3.37500000	0.04242424
3628	3.06250000	3.37500000	0.06686217
3629	2.93750000	3.31250000	0.03245357
3630	3.06250000	3.62500000	0.10694037
3631	2.93750000	3.56250000	0.09149560
3632	3.06250000	3.68750000	0.12512219
3633	2.93750000	3.62500000	0.10987292
3634	3.12500000	3.56250000	0.13020528
3635	3.12500000	3.43750000	0.11827957
3636	3.18750000	3.56250000	0.14643206
3637	3.12500000	3.68750000	0.12512219
3638	3.18750000	3.62500000	0.12316716
3639	2.87500000	3.43750000	0.05513196
3640	2.87500000	3.31250000	0.02424242
3641	2.81250000	3.37500000	0.03714565
3642	2.87500000	3.56250000	0.09442815
3643	2.81250000	3.43750000	0.05806452
3644	3.43750000	3.06250000	0.03440860
3645	3.37500000	2.93750000	-0.00391007
3646	3.37500000	3.06250000	0.05747801
3647	3.31250000	2.93750000	0.01896383
3648	3.62500000	3.06250000	0.06119257
3649	3.56250000	2.93750000	0.03264907
3650	3.68750000	3.06250000	0.11026393
3651	3.62500000	2.93750000	0.08172043
3652	3.56250000	3.12500000	0.06158358
3653	3.43750000	3.12500000	0.08387097
3654	3.56250000	3.18750000	0.11104594

3655	3.68750000	3.12500000	0.11143695
3656	3.62500000	3.18750000	0.11182796
3657	3.43750000	2.87500000	-0.00527859
3658	3.31250000	2.87500000	0.00703812
3659	3.37500000	2.81250000	0.00566960
3660	3.56250000	2.87500000	0.05434995
3661	3.43750000	2.81250000	0.01622678
3662	2.56250000	2.93750000	-0.13567937
3663	2.62500000	3.06250000	-0.11065494
3664	2.62500000	2.93750000	-0.10244379
3665	2.68750000	3.06250000	-0.07722385
3666	2.37500000	2.93750000	-0.15620723
3667	2.43750000	3.06250000	-0.12336266
3668	2.31250000	2.93750000	-0.13450635
3669	2.37500000	3.06250000	-0.10166178
3670	2.43750000	2.87500000	-0.15562072
3671	2.56250000	2.87500000	-0.11358749
3672	2.43750000	2.81250000	-0.13333333
3673	2.31250000	2.87500000	-0.14271750
3674	2.37500000	2.81250000	-0.14213099
3675	2.56250000	3.12500000	-0.09775171
3676	2.68750000	3.12500000	-0.06334311
3677	2.62500000	3.18750000	-0.05043988
3678	2.43750000	3.12500000	-0.07702835
3679	2.56250000	3.18750000	-0.05141740
3680	2.93750000	2.56250000	-0.10869990
3681	3.06250000	2.62500000	-0.07800587
3682	2.93750000	2.62500000	-0.09657869
3683	3.06250000	2.68750000	-0.06568915
3684	2.93750000	2.37500000	-0.14056696
3685	3.06250000	2.43750000	-0.07644184
3686	2.93750000	2.31250000	-0.14975562
3687	3.06250000	2.37500000	-0.12961877
3688	2.87500000	2.43750000	-0.13040078
3689	2.87500000	2.56250000	-0.10772239
3690	2.81250000	2.43750000	-0.12942326
3691	2.87500000	2.31250000	-0.15014663
3692	2.81250000	2.37500000	-0.13998045
3693	3.12500000	2.56250000	-0.03558162
3694	3.12500000	2.68750000	-0.04613881
3695	3.18750000	2.62500000	-0.04750733
3696	3.12500000	2.43750000	-0.06588465
3697	3.18750000	2.56250000	-0.02463343
3698	2.56250000	3.62500000	0.11397849
3699	2.62500000	3.56250000	0.10009775
3700	2.62500000	3.68750000	0.12629521
3701	2.68750000	3.62500000	0.11241447
3702	2.37500000	3.43750000	0.05493646
3703	2.43750000	3.37500000	0.03812317
3704	2.31250000	3.37500000	0.02756598
3705	2.37500000	3.31250000	0.01075269
3706	2.43750000	3.56250000	0.09442815
3707	2.56250000	3.68750000	0.12590420
3708	2.43750000	3.62500000	0.10654936
3709	2.31250000	3.43750000	0.04789834
3710	2.37500000	3.56250000	0.08739003
3711	2.56250000	3.43750000	0.06373412
3712	2.68750000	3.56250000	0.09814272
3713	2.62500000	3.43750000	0.06177908

3714	2.43750000	3.31250000	0.01427175
3715	2.56250000	3.37500000	0.03968719
3716	3.62500000	2.56250000	0.00019550
3717	3.56250000	2.62500000	0.00664712
3718	3.68750000	2.62500000	0.03030303
3719	3.62500000	2.68750000	0.03675464
3720	3.43750000	2.37500000	-0.07077224
3721	3.37500000	2.43750000	-0.07253177
3722	3.37500000	2.31250000	-0.08797654
3723	3.31250000	2.37500000	-0.08973607
3724	3.56250000	2.43750000	-0.03597263
3725	3.68750000	2.56250000	0.01779081
3726	3.62500000	2.43750000	-0.01837732
3727	3.43750000	2.31250000	-0.07683284
3728	3.56250000	2.37500000	-0.04203324
3729	3.43750000	2.56250000	-0.03128055
3730	3.56250000	2.68750000	0.03069404
3731	3.43750000	2.62500000	-0.00723363
3732	3.31250000	2.43750000	-0.08035191
3733	3.37500000	2.56250000	-0.03910068
3734	0.93750000	2.37500000	-0.10811339
3735	1.06250000	2.43750000	-0.11436950
3736	0.93750000	2.31250000	-0.12023460
3737	1.06250000	2.37500000	-0.12629521
3738	0.93750000	2.56250000	-0.06920821
3739	1.06250000	2.62500000	-0.07487781
3740	0.93750000	2.62500000	-0.05708700
3741	1.06250000	2.68750000	-0.06295210
3742	0.87500000	2.43750000	-0.08269795
3743	0.87500000	2.31250000	-0.10967742
3744	0.81250000	2.37500000	-0.08406647
3745	0.87500000	2.56250000	-0.05571848
3746	0.81250000	2.43750000	-0.06920821
3747	1.12500000	2.56250000	-0.09462366
3748	1.12500000	2.43750000	-0.12199413
3749	1.18750000	2.56250000	-0.10224829
3750	1.12500000	2.68750000	-0.06725318
3751	1.18750000	2.62500000	-0.08680352
3752	1.43750000	2.37500000	-0.17008798
3753	1.37500000	2.43750000	-0.15757576
3754	1.37500000	2.31250000	-0.17165200
3755	1.31250000	2.37500000	-0.15913978
3756	1.62500000	2.56250000	-0.15972630
3757	1.56250000	2.62500000	-0.14095797
3758	1.68750000	2.62500000	-0.15229717
3759	1.62500000	2.68750000	-0.13333333
3760	1.56250000	2.43750000	-0.16930596
3761	1.43750000	2.31250000	-0.17223851
3762	1.56250000	2.37500000	-0.17145650
3763	1.68750000	2.56250000	-0.16050831
3764	1.62500000	2.43750000	-0.17008798
3765	1.43750000	2.56250000	-0.13802542
3766	1.31250000	2.43750000	-0.14721408
3767	1.37500000	2.56250000	-0.12746823
3768	1.56250000	2.68750000	-0.12277615
3769	1.43750000	2.62500000	-0.11984360
3770	1.43750000	3.06250000	-0.08739003
3771	1.37500000	2.93750000	-0.11221896
3772	1.37500000	3.06250000	-0.04516129

3773	1.31250000	2.93750000	-0.06999022
3774	1.62500000	3.06250000	-0.10987292
3775	1.56250000	2.93750000	-0.13489736
3776	1.68750000	3.06250000	-0.08289345
3777	1.62500000	2.93750000	-0.10772239
3778	1.56250000	3.12500000	-0.08191593
3779	1.43750000	3.12500000	-0.03225806
3780	1.56250000	3.18750000	-0.02697947
3781	1.68750000	3.12500000	-0.06236559
3782	1.62500000	3.18750000	-0.03440860
3783	1.43750000	2.87500000	-0.13176931
3784	1.31250000	2.87500000	-0.08191593
3785	1.37500000	2.81250000	-0.10146628
3786	1.56250000	2.87500000	-0.11221896
3787	1.43750000	2.81250000	-0.10909091
3788	2.37500000	0.93750000	-0.19198436
3789	2.43750000	1.06250000	-0.19628543
3790	2.31250000	0.93750000	-0.19237537
3791	2.37500000	1.06250000	-0.19667644
3792	2.56250000	0.93750000	-0.19257087
3793	2.62500000	1.06250000	-0.19511241
3794	2.62500000	0.93750000	-0.19217986
3795	2.68750000	1.06250000	-0.19472141
3796	2.43750000	0.87500000	-0.18885630
3797	2.31250000	0.87500000	-0.18787879
3798	2.37500000	0.81250000	-0.18475073
3799	2.56250000	0.87500000	-0.18983382
3800	2.43750000	0.81250000	-0.18611926
3801	2.56250000	1.12500000	-0.19569892
3802	2.43750000	1.12500000	-0.19648094
3803	2.56250000	1.18750000	-0.19589443
3804	2.68750000	1.12500000	-0.19491691
3805	2.62500000	1.18750000	-0.19550342
3806	2.37500000	1.43750000	-0.19804497
3807	2.43750000	1.37500000	-0.19765396
3808	2.31250000	1.37500000	-0.19824047
3809	2.37500000	1.31250000	-0.19784946
3810	2.56250000	1.62500000	-0.19765396
3811	2.62500000	1.56250000	-0.19706745
3812	2.62500000	1.68750000	-0.19745846
3813	2.68750000	1.62500000	-0.19687195
3814	2.43750000	1.56250000	-0.19804497
3815	2.31250000	1.43750000	-0.19824047
3816	2.37500000	1.56250000	-0.19804497
3817	2.56250000	1.68750000	-0.19784946
3818	2.43750000	1.62500000	-0.19804497
3819	2.56250000	1.43750000	-0.19706745
3820	2.43750000	1.31250000	-0.19745846
3821	2.56250000	1.37500000	-0.19667644
3822	2.68750000	1.56250000	-0.19667644
3823	2.62500000	1.43750000	-0.19648094
3824	3.06250000	1.43750000	-0.18279570
3825	2.93750000	1.37500000	-0.19413490
3826	3.06250000	1.37500000	-0.18260020
3827	2.93750000	1.31250000	-0.19393939
3828	3.06250000	1.62500000	-0.18357771
3829	2.93750000	1.56250000	-0.19491691
3830	3.06250000	1.68750000	-0.18377322
3831	2.93750000	1.62500000	-0.19511241

3832	3.12500000	1.56250000	-0.17223851
3833	3.12500000	1.43750000	-0.17165200
3834	3.18750000	1.56250000	-0.16109482
3835	3.12500000	1.68750000	-0.17282502
3836	3.18750000	1.62500000	-0.16148583
3837	2.87500000	1.43750000	-0.19491691
3838	2.87500000	1.31250000	-0.19433040
3839	2.81250000	1.37500000	-0.19491691
3840	2.87500000	1.56250000	-0.19550342
3841	2.81250000	1.43750000	-0.19530792
3842	0.12500000	0.31250000	0.09814272
3843	0.18750000	0.31250000	0.08797654
3844	0.18750000	0.37500000	0.07565982
3845	0.31250000	0.12500000	0.09325513
3846	0.31250000	0.18750000	0.08465298
3847	0.37500000	0.18750000	0.07096774
3848	0.12500000	3.81250000	0.19315738
3849	0.18750000	3.87500000	0.19433040
3850	0.18750000	3.81250000	0.19374389
3851	3.81250000	0.12500000	0.10635386
3852	3.87500000	0.18750000	0.11065494
3853	3.81250000	0.18750000	0.09990225
3854	3.68750000	3.81250000	0.19413490
3855	3.68750000	3.87500000	0.19569892
3856	3.81250000	3.68750000	0.19198436
3857	3.87500000	3.68750000	0.19257087
3858	3.62500000	3.81250000	0.19413490
3859	3.81250000	3.62500000	0.19081134
3860	0.12500000	1.81250000	-0.04398827
3861	0.18750000	1.87500000	-0.05493646
3862	0.18750000	1.81250000	-0.05415445
3863	0.12500000	2.31250000	0.00430108
3864	0.18750000	2.31250000	0.00234604
3865	0.18750000	2.37500000	0.00918866
3866	0.31250000	2.12500000	-0.04672532
3867	0.31250000	2.18750000	-0.03167155
3868	0.37500000	2.18750000	-0.04183773
3869	1.81250000	0.12500000	-0.12043011
3870	1.87500000	0.18750000	-0.13098729
3871	1.81250000	0.18750000	-0.12649071
3872	2.31250000	0.12500000	-0.11984360
3873	2.31250000	0.18750000	-0.13196481
3874	2.37500000	0.18750000	-0.12531769
3875	2.12500000	0.31250000	-0.15268817
3876	2.18750000	0.31250000	-0.15386119
3877	2.18750000	0.37500000	-0.15816227
3878	1.68750000	1.81250000	-0.19374389
3879	1.68750000	1.87500000	-0.19472141
3880	1.81250000	1.68750000	-0.19491691
3881	1.87500000	1.68750000	-0.19648094
3882	1.62500000	1.81250000	-0.19315738
3883	1.81250000	1.62500000	-0.19491691
3884	2.18750000	2.31250000	-0.16363636
3885	2.12500000	2.31250000	-0.17262952
3886	2.31250000	2.18750000	-0.16695992
3887	2.31250000	2.12500000	-0.17751711
3888	2.18750000	2.37500000	-0.16168133
3889	2.37500000	2.18750000	-0.16676442
3890	1.81250000	2.12500000	-0.19433040

3891	1.87500000	2.18750000	-0.19296188
3892	1.81250000	2.18750000	-0.19237537
3893	2.12500000	1.81250000	-0.19960899
3894	2.18750000	1.87500000	-0.19941349
3895	2.18750000	1.81250000	-0.19941349
3896	1.68750000	3.87500000	0.15366569
3897	1.68750000	3.81250000	0.14525904
3898	1.62500000	3.81250000	0.14545455
3899	2.18750000	3.87500000	0.14838710
3900	2.12500000	3.81250000	0.12922776
3901	2.18750000	3.81250000	0.13274682
3902	1.81250000	3.68750000	0.11358749
3903	1.87500000	3.68750000	0.10615836
3904	1.81250000	3.62500000	0.09794721
3905	3.87500000	1.68750000	-0.02717498
3906	3.81250000	1.68750000	-0.04848485
3907	3.81250000	1.62500000	-0.04418377
3908	3.87500000	2.18750000	-0.00293255
3909	3.81250000	2.12500000	-0.02658847
3910	3.81250000	2.18750000	-0.01407625
3911	3.68750000	1.81250000	-0.07331378
3912	3.68750000	1.87500000	-0.07350929
3913	3.62500000	1.81250000	-0.08445748
3914	0.12500000	0.81250000	0.00234604
3915	0.18750000	0.87500000	-0.01955034
3916	0.18750000	0.81250000	-0.00762463
3917	0.12500000	1.31250000	-0.03851417
3918	0.18750000	1.31250000	-0.04887586
3919	0.18750000	1.37500000	-0.04965787
3920	0.31250000	1.12500000	-0.06588465
3921	0.31250000	1.18750000	-0.06705767
3922	0.37500000	1.18750000	-0.07683284
3923	0.81250000	0.12500000	-0.02365591
3924	0.87500000	0.18750000	-0.04652981
3925	0.81250000	0.18750000	-0.03088954
3926	1.31250000	0.12500000	-0.08778104
3927	1.31250000	0.18750000	-0.09227761
3928	1.37500000	0.18750000	-0.09657869
3929	1.12500000	0.31250000	-0.09599218
3930	1.18750000	0.31250000	-0.09775171
3931	1.18750000	0.37500000	-0.10518084
3932	0.68750000	0.81250000	-0.08817204
3933	0.68750000	0.87500000	-0.09990225
3934	0.81250000	0.68750000	-0.09345064
3935	0.87500000	0.68750000	-0.10772239
3936	0.62500000	0.81250000	-0.07839687
3937	0.81250000	0.62500000	-0.08602151
3938	1.18750000	1.31250000	-0.18044966
3939	1.12500000	1.31250000	-0.18005865
3940	1.31250000	1.18750000	-0.17986315
3941	1.31250000	1.12500000	-0.17986315
3942	1.18750000	1.37500000	-0.18162268
3943	1.37500000	1.18750000	-0.18318671
3944	0.81250000	1.12500000	-0.14565005
3945	0.87500000	1.18750000	-0.15640274
3946	0.81250000	1.18750000	-0.14623656
3947	1.12500000	0.81250000	-0.15425220
3948	1.18750000	0.87500000	-0.16344086
3949	1.18750000	0.81250000	-0.15601173

3950	0.12500000	3.31250000	0.18455523
3951	0.18750000	3.31250000	0.18318671
3952	0.18750000	3.37500000	0.18377322
3953	0.12500000	2.81250000	0.10967742
3954	0.18750000	2.87500000	0.12101662
3955	0.18750000	2.81250000	0.10146628
3956	0.31250000	3.18750000	0.16559140
3957	0.31250000	3.12500000	0.15816227
3958	0.37500000	3.18750000	0.15738025
3959	0.68750000	3.87500000	0.18338221
3960	0.68750000	3.81250000	0.18084066
3961	0.62500000	3.81250000	0.18416422
3962	1.18750000	3.87500000	0.15542522
3963	1.12500000	3.81250000	0.14760508
3964	1.18750000	3.81250000	0.14760508
3965	0.81250000	3.68750000	0.15835777
3966	0.87500000	3.68750000	0.14956012
3967	0.81250000	3.62500000	0.15053763
3968	3.31250000	0.12500000	0.01642229
3969	3.31250000	0.18750000	0.00821114
3970	3.37500000	0.18750000	0.01935484
3971	2.81250000	0.12500000	-0.06412512
3972	2.87500000	0.18750000	-0.06823069
3973	2.81250000	0.18750000	-0.07468231
3974	3.18750000	0.31250000	-0.03519062
3975	3.12500000	0.31250000	-0.04887586
3976	3.18750000	0.37500000	-0.04594330
3977	3.87500000	0.68750000	0.05708700
3978	3.81250000	0.68750000	0.04477028
3979	3.81250000	0.62500000	0.05083089
3980	3.87500000	1.18750000	0.00762463
3981	3.81250000	1.12500000	-0.00332356
3982	3.81250000	1.18750000	-0.00762463
3983	3.68750000	0.81250000	0.00195503
3984	3.68750000	0.87500000	-0.00703812
3985	3.62500000	0.81250000	-0.01309873
3986	3.18750000	3.31250000	0.08172043
3987	3.12500000	3.31250000	0.06920821
3988	3.31250000	3.18750000	0.09345064
3989	3.31250000	3.12500000	0.08699902
3990	3.18750000	3.37500000	0.09560117
3991	3.37500000	3.18750000	0.11339198
3992	2.68750000	2.81250000	-0.07468231
3993	2.68750000	2.87500000	-0.07194526
3994	2.81250000	2.68750000	-0.08250244
3995	2.87500000	2.68750000	-0.08347996
3996	2.62500000	2.81250000	-0.08308895
3997	2.81250000	2.62500000	-0.09462366
3998	2.87500000	3.18750000	0.00019550
3999	2.81250000	3.12500000	-0.02189638
4000	2.81250000	3.18750000	-0.00782014
4001	3.18750000	2.87500000	0.01016618
4002	3.12500000	2.81250000	-0.02170088
4003	3.18750000	2.81250000	-0.00195503
4004	3.18750000	3.87500000	0.17145650
4005	3.12500000	3.81250000	0.15738025
4006	3.18750000	3.81250000	0.15738025
4007	2.68750000	3.87500000	0.16832845
4008	2.68750000	3.81250000	0.15347019

4009	2.62500000	3.81250000	0.15307918
4010	2.87500000	3.68750000	0.12707722
4011	2.81250000	3.68750000	0.12590420
4012	2.81250000	3.62500000	0.11163245
4013	3.87500000	3.18750000	0.17360704
4014	3.81250000	3.12500000	0.16656891
4015	3.81250000	3.18750000	0.16754643
4016	3.87500000	2.68750000	0.09012708
4017	3.81250000	2.68750000	0.07526882
4018	3.81250000	2.62500000	0.06275660
4019	3.68750000	2.87500000	0.10948192
4020	3.68750000	2.81250000	0.08817204
4021	3.62500000	2.81250000	0.08211144
4022	0.68750000	1.87500000	-0.13743891
4023	0.68750000	1.81250000	-0.13685239
4024	0.81250000	2.12500000	-0.13548387
4025	0.87500000	2.18750000	-0.13391984
4026	0.62500000	1.81250000	-0.12629521
4027	0.81250000	2.18750000	-0.12336266
4028	1.18750000	1.87500000	-0.19042033
4029	1.12500000	1.81250000	-0.18866080
4030	1.31250000	2.12500000	-0.18357771
4031	1.31250000	2.18750000	-0.17849462
4032	1.18750000	1.81250000	-0.18924731
4033	1.37500000	2.18750000	-0.17888563
4034	0.81250000	1.68750000	-0.15562072
4035	0.87500000	1.68750000	-0.16559140
4036	0.81250000	1.62500000	-0.15444770
4037	1.12500000	2.31250000	-0.14584555
4038	1.18750000	2.31250000	-0.15327468
4039	1.18750000	2.37500000	-0.14134897
4040	0.68750000	2.81250000	0.03519062
4041	0.68750000	2.87500000	0.04848485
4042	0.81250000	2.68750000	-0.01818182
4043	0.87500000	2.68750000	-0.03167155
4044	0.62500000	2.81250000	0.04985337
4045	0.81250000	2.62500000	-0.03010753
4046	1.18750000	3.31250000	0.06686217
4047	1.12500000	3.31250000	0.06725318
4048	1.31250000	3.18750000	0.03147605
4049	1.31250000	3.12500000	0.01427175
4050	1.18750000	3.37500000	0.07996090
4051	1.37500000	3.18750000	0.02717498
4052	0.81250000	3.12500000	0.07194526
4053	0.87500000	3.18750000	0.07057674
4054	0.81250000	3.18750000	0.08523949
4055	1.12500000	2.81250000	-0.04320626
4056	1.18750000	2.87500000	-0.03538612
4057	1.18750000	2.81250000	-0.04750733
4058	1.87500000	0.68750000	-0.16774194
4059	1.81250000	0.68750000	-0.16344086
4060	2.12500000	0.81250000	-0.18494624
4061	2.18750000	0.87500000	-0.18885630
4062	1.81250000	0.62500000	-0.15894428
4063	2.18750000	0.81250000	-0.18435973
4064	1.87500000	1.18750000	-0.19608993
4065	1.81250000	1.12500000	-0.19452590
4066	2.12500000	1.31250000	-0.19882698
4067	2.18750000	1.31250000	-0.19863148

4068	1.81250000	1.18750000	-0.19452590
4069	2.18750000	1.37500000	-0.19863148
4070	1.68750000	0.81250000	-0.16891496
4071	1.68750000	0.87500000	-0.17614858
4072	1.62500000	0.81250000	-0.16735093
4073	2.31250000	1.12500000	-0.19745846
4074	2.31250000	1.18750000	-0.19784946
4075	2.37500000	1.18750000	-0.19726295
4076	2.81250000	0.68750000	-0.16402737
4077	2.87500000	0.68750000	-0.15581623
4078	2.68750000	0.81250000	-0.18592375
4079	2.68750000	0.87500000	-0.18885630
4080	2.81250000	0.62500000	-0.15347019
4081	2.62500000	0.81250000	-0.18651026
4082	3.31250000	1.18750000	-0.12922776
4083	3.31250000	1.12500000	-0.12492669
4084	3.18750000	1.31250000	-0.16031281
4085	3.12500000	1.31250000	-0.17126100
4086	3.37500000	1.18750000	-0.11397849
4087	3.18750000	1.37500000	-0.16050831
4088	3.12500000	0.81250000	-0.13020528
4089	3.18750000	0.87500000	-0.12570870
4090	3.18750000	0.81250000	-0.11495601
4091	2.81250000	1.12500000	-0.19413490
4092	2.87500000	1.18750000	-0.19393939
4093	2.81250000	1.18750000	-0.19433040
4094	2.12500000	2.81250000	-0.13626588
4095	2.18750000	2.87500000	-0.12453568
4096	1.87500000	2.68750000	-0.15229717
4097	1.81250000	2.68750000	-0.14858260
4098	2.18750000	2.81250000	-0.13274682
4099	1.81250000	2.62500000	-0.15679374
4100	2.12500000	3.31250000	-0.00840665
4101	2.18750000	3.31250000	-0.00410557
4102	1.87500000	3.18750000	-0.03616813
4103	1.81250000	3.12500000	-0.04594330
4104	2.18750000	3.37500000	0.01622678
4105	1.81250000	3.18750000	-0.02561095
4106	2.31250000	3.12500000	-0.05884653
4107	2.31250000	3.18750000	-0.03773216
4108	2.37500000	3.18750000	-0.03421310
4109	1.68750000	2.81250000	-0.11065494
4110	1.68750000	2.87500000	-0.09560117
4111	1.62500000	2.81250000	-0.10009775
4112	2.81250000	2.12500000	-0.17849462
4113	2.87500000	2.18750000	-0.16871945
4114	2.68750000	1.87500000	-0.19745846
4115	2.68750000	1.81250000	-0.19745846
4116	2.81250000	2.18750000	-0.16911046
4117	2.62500000	1.81250000	-0.19765396
4118	3.31250000	2.12500000	-0.12277615
4119	3.31250000	2.18750000	-0.11397849
4120	3.18750000	1.87500000	-0.16246334
4121	3.12500000	1.81250000	-0.17321603
4122	3.37500000	2.18750000	-0.10283480
4123	3.18750000	1.81250000	-0.16226784
4124	3.12500000	2.31250000	-0.12825024
4125	3.18750000	2.31250000	-0.11749756
4126	3.18750000	2.37500000	-0.10830890

4127	2.81250000	1.68750000	-0.19628543
4128	2.87500000	1.68750000	-0.19589443
4129	2.81250000	1.62500000	-0.19608993
4130	0.31250000	0.62500000	0.00762463
4131	0.31250000	0.68750000	-0.00410557
4132	0.37500000	0.68750000	-0.01427175
4133	0.62500000	0.31250000	-0.00273705
4134	0.68750000	0.31250000	-0.01681329
4135	0.68750000	0.37500000	-0.02561095
4136	0.31250000	3.68750000	0.18983382
4137	0.31250000	3.62500000	0.18729228
4138	0.37500000	3.68750000	0.18846530
4139	3.68750000	0.31250000	0.06236559
4140	3.62500000	0.31250000	0.05004888
4141	3.68750000	0.37500000	0.05434995
4142	3.37500000	3.68750000	0.16617791
4143	3.31250000	3.62500000	0.15190616
4144	3.31250000	3.68750000	0.15366569
4145	3.68750000	3.37500000	0.17458456
4146	3.62500000	3.31250000	0.16754643
4147	3.68750000	3.31250000	0.16832845
4148	0.31250000	1.68750000	-0.07331378
4149	0.31250000	1.62500000	-0.07272727
4150	0.37500000	1.68750000	-0.08367546
4151	0.31250000	2.62500000	0.04555230
4152	0.31250000	2.68750000	0.05865103
4153	0.37500000	2.68750000	0.05669599
4154	0.62500000	2.31250000	-0.05884653
4155	0.68750000	2.31250000	-0.07214076
4156	0.68750000	2.37500000	-0.05728250
4157	1.68750000	0.31250000	-0.12785924
4158	1.62500000	0.31250000	-0.12336266
4159	1.68750000	0.37500000	-0.13216031
4160	2.62500000	0.31250000	-0.12023460
4161	2.68750000	0.31250000	-0.11202346
4162	2.68750000	0.37500000	-0.12414467
4163	2.31250000	0.62500000	-0.17517107
4164	2.31250000	0.68750000	-0.17790811
4165	2.37500000	0.68750000	-0.17927664
4166	1.37500000	1.68750000	-0.18866080
4167	1.31250000	1.62500000	-0.18690127
4168	1.31250000	1.68750000	-0.18807429
4169	1.68750000	1.37500000	-0.19120235
4170	1.62500000	1.31250000	-0.18944282
4171	1.68750000	1.31250000	-0.19120235
4172	2.31250000	2.62500000	-0.13431085
4173	2.37500000	2.68750000	-0.12258065
4174	2.31250000	2.68750000	-0.13157380
4175	2.62500000	2.31250000	-0.14721408
4176	2.68750000	2.37500000	-0.13782991
4177	2.68750000	2.31250000	-0.14838710
4178	1.68750000	2.31250000	-0.18103617
4179	1.62500000	2.31250000	-0.17732160
4180	1.68750000	2.37500000	-0.17595308
4181	2.31250000	1.68750000	-0.19863148
4182	2.31250000	1.62500000	-0.19843597
4183	2.37500000	1.68750000	-0.19824047
4184	1.37500000	3.68750000	0.13020528
4185	1.31250000	3.68750000	0.13079179

4186	1.31250000	3.62500000	0.12238514
4187	2.31250000	3.68750000	0.10987292
4188	2.37500000	3.68750000	0.11417400
4189	2.31250000	3.62500000	0.09521017
4190	1.68750000	3.37500000	0.04731183
4191	1.62500000	3.31250000	0.03773216
4192	1.68750000	3.31250000	0.03010753
4193	3.68750000	1.37500000	-0.05083089
4194	3.68750000	1.31250000	-0.04672532
4195	3.62500000	1.31250000	-0.06177908
4196	3.68750000	2.31250000	-0.01837732
4197	3.68750000	2.37500000	-0.00957967
4198	3.62500000	2.31250000	-0.03323558
4199	3.37500000	1.68750000	-0.12825024
4200	3.31250000	1.62500000	-0.13919844
4201	3.31250000	1.68750000	-0.13939394
4202	0.62500000	1.31250000	-0.11886608
4203	0.68750000	1.31250000	-0.12903226
4204	0.68750000	1.37500000	-0.13000978
4205	1.31250000	0.62500000	-0.13724340
4206	1.31250000	0.68750000	-0.14447703
4207	1.37500000	0.68750000	-0.14623656
4208	0.62500000	3.31250000	0.13822092
4209	0.68750000	3.37500000	0.13685239
4210	0.68750000	3.31250000	0.12942326
4211	3.31250000	0.62500000	-0.05747801
4212	3.37500000	0.68750000	-0.05298143
4213	3.31250000	0.68750000	-0.06647116
4214	2.68750000	3.31250000	0.01798631
4215	2.68750000	3.37500000	0.03890518
4216	2.62500000	3.31250000	0.01681329
4217	3.31250000	2.68750000	-0.00469208
4218	3.37500000	2.68750000	0.00606061
4219	3.31250000	2.62500000	-0.02580645
4220	1.31250000	2.62500000	-0.10185728
4221	1.31250000	2.68750000	-0.08660802
4222	1.37500000	2.68750000	-0.09403715
4223	2.62500000	1.31250000	-0.19589443
4224	2.68750000	1.31250000	-0.19550342
4225	2.68750000	1.37500000	-0.19569892
4226	1.00000000	3.03125000	-0.04203324
4227	2.03125000	3.03125000	-0.09384164
4228	0.53125000	0.03125000	0.16813294
4229	1.46875000	3.46875000	0.04809384
4230	1.53125000	3.50000000	0.03949169
4231	0.46875000	2.96875000	0.05043988
4232	3.53125000	3.03125000	-0.01300098
4233	2.53125000	3.03125000	-0.16774194
4234	1.53125000	3.03125000	-0.16060606
4235	0.28125000	0.28125000	0.05347019
4236	1.28125000	3.28125000	0.00967742
4237	1.00000000	3.21875000	-0.00185728
4238	0.71875000	0.21875000	0.00478983
4239	3.71875000	3.21875000	0.13743891
4240	1.71875000	3.50000000	0.01691105
4241	1.71875000	3.21875000	-0.01837732
4242	2.71875000	3.21875000	-0.02580645
4243	1.09375000	3.12500000	-0.02267840
4244	0.90625000	3.03125000	-0.02003910

4245	1.00000000	3.09375000	-0.12883675
4246	1.00000000	3.15625000	-0.11544477
4247	1.03125000	3.12500000	-0.12238514
4248	0.96875000	3.09375000	-0.12150538
4249	1.03125000	3.15625000	-0.11564027
4250	0.96875000	3.12500000	-0.11485826
4251	2.90625000	1.03125000	-0.11818182
4252	2.84375000	0.96875000	-0.11700880
4253	2.96875000	1.09375000	-0.16803519
4254	2.09375000	3.09375000	-0.04418377
4255	0.65625000	0.03125000	0.07380254
4256	0.53125000	0.15625000	0.08787879
4257	0.59375000	0.09375000	0.11378299
4258	0.65625000	0.15625000	0.05933529
4259	0.34375000	0.34375000	-0.01906158
4260	0.34375000	0.46875000	-0.00547410
4261	0.46875000	0.34375000	-0.00889541
4262	1.96875000	2.59375000	-0.09511241
4263	1.90625000	2.53125000	-0.14965787
4264	1.62500000	3.59375000	0.04711632
4265	1.40625000	3.40625000	-0.06412512
4266	1.34375000	3.34375000	-0.07693060
4267	1.37500000	3.40625000	-0.06383187
4268	1.40625000	3.37500000	-0.07272727
4269	1.34375000	3.37500000	-0.07028348
4270	1.37500000	3.34375000	-0.07908113
4271	1.59375000	3.50000000	-0.08993157
4272	1.65625000	3.50000000	-0.09745846
4273	1.62500000	3.53125000	-0.08739003
4274	1.59375000	3.46875000	-0.09853372
4275	1.65625000	3.53125000	-0.08963832
4276	1.62500000	3.46875000	-0.10224829
4277	1.37500000	3.46875000	0.04897361
4278	1.46875000	3.37500000	0.02228739
4279	1.53125000	3.40625000	0.01368524
4280	0.40625000	2.90625000	0.11417400
4281	3.03125000	3.40625000	0.07634409
4282	3.09375000	3.46875000	0.12776149
4283	3.65625000	3.03125000	0.08514174
4284	3.53125000	3.15625000	0.08592375
4285	3.59375000	3.09375000	0.03724340
4286	3.65625000	3.15625000	0.08739003
4287	2.65625000	3.03125000	-0.10087977
4288	2.59375000	3.09375000	-0.12043011
4289	2.65625000	3.15625000	-0.07311828
4290	2.53125000	3.15625000	-0.07507331
4291	3.03125000	2.40625000	-0.10830890
4292	3.09375000	2.46875000	-0.04457478
4293	1.65625000	3.03125000	-0.10664712
4294	1.53125000	3.15625000	-0.05073314
4295	1.59375000	3.09375000	-0.11309873
4296	1.65625000	3.15625000	-0.06568915
4297	0.28125000	0.40625000	0.04183773
4298	0.40625000	0.28125000	0.03861193
4299	1.71875000	3.59375000	0.04037146
4300	0.71875000	0.09375000	0.01925709
4301	2.71875000	3.09375000	-0.05356794
4302	3.71875000	3.09375000	0.13538612
4303	1.09375000	3.21875000	-0.00244379

4304	1.28125000	3.37500000	0.02961877
4305	1.37500000	3.28125000	0.00322581
4306	0.90625000	3.12500000	-0.00009775
4307	2.78125000	0.90625000	-0.16451613
4308	1.71875000	3.09375000	-0.05913978
4309	0.59375000	0.21875000	0.03333333
4310	3.59375000	3.21875000	0.13616813
4311	1.62500000	3.40625000	0.00254154
4312	1.59375000	3.21875000	-0.00322581
4313	2.59375000	3.21875000	-0.02776149
4314	1.03125000	3.06250000	-0.03558162
4315	1.06250000	3.09375000	-0.02913001
4316	0.96875000	3.03125000	-0.03470186
4317	0.93750000	3.03125000	-0.02737048
4318	2.96875000	1.03125000	-0.16764418
4319	2.03125000	3.09375000	-0.07350929
4320	2.09375000	3.03125000	-0.09032258
4321	0.59375000	0.03125000	0.12101662
4322	0.53125000	0.09375000	0.12805474
4323	1.96875000	2.53125000	-0.15337243
4324	1.56250000	3.53125000	0.04203324
4325	1.59375000	3.56250000	0.04457478
4326	1.43750000	3.46875000	0.04838710
4327	1.46875000	3.43750000	0.03949169
4328	1.53125000	3.46875000	0.03088954
4329	1.40625000	3.46875000	0.04868035
4330	1.46875000	3.40625000	0.03088954
4331	1.53125000	3.43750000	0.02228739
4332	0.40625000	2.96875000	0.08367546
4333	0.46875000	2.90625000	0.06217009
4334	3.03125000	3.46875000	0.08631476
4335	3.59375000	3.03125000	0.03607038
4336	3.53125000	3.09375000	0.03646139
4337	2.59375000	3.03125000	-0.13431085
4338	2.53125000	3.09375000	-0.12140762
4339	3.03125000	2.46875000	-0.09892473
4340	1.59375000	3.03125000	-0.13362659
4341	1.53125000	3.09375000	-0.10566960
4342	0.28125000	0.34375000	0.04134897
4343	0.34375000	0.28125000	0.03978495
4344	0.71875000	0.03125000	0.02649071
4345	2.71875000	3.03125000	-0.06764418
4346	3.71875000	3.03125000	0.13421310
4347	1.28125000	3.31250000	0.01632454
4348	1.31250000	3.28125000	0.00752688
4349	1.28125000	3.34375000	0.02297165
4350	1.34375000	3.28125000	0.00537634
4351	1.03125000	3.21875000	-0.00205279
4352	0.96875000	3.18750000	-0.00127077
4353	1.06250000	3.21875000	-0.00224829
4354	0.93750000	3.15625000	-0.00068426
4355	2.78125000	0.96875000	-0.16744868
4356	1.71875000	3.03125000	-0.07947214
4357	0.28125000	0.46875000	0.02971652
4358	0.53125000	0.21875000	0.04760508
4359	0.46875000	0.28125000	0.02473118
4360	0.71875000	0.15625000	0.01202346
4361	0.65625000	0.21875000	0.01906158
4362	3.53125000	3.21875000	0.13538612

4363	3.71875000	3.15625000	0.13646139
4364	3.65625000	3.21875000	0.13685239
4365	1.71875000	3.53125000	0.02473118
4366	1.68750000	3.46875000	0.01212121
4367	1.71875000	3.56250000	0.03255132
4368	1.65625000	3.43750000	0.00733138
4369	1.53125000	3.21875000	0.00420332
4370	1.71875000	3.15625000	-0.03870968
4371	1.65625000	3.21875000	-0.01075269
4372	2.71875000	3.15625000	-0.03968719
4373	2.65625000	3.21875000	-0.02678397
4374	2.53125000	3.21875000	-0.02893451
4375	1.09375000	3.15625000	-0.01593353
4376	1.09375000	3.18750000	-0.00918866
4377	0.90625000	3.06250000	-0.01339198
4378	0.90625000	3.09375000	-0.00674487
4379	2.84375000	0.90625000	-0.16412512
4380	2.90625000	1.09375000	-0.16842620
4381	0.65625000	0.09375000	0.06656891
4382	0.59375000	0.15625000	0.07360704
4383	0.34375000	0.40625000	-0.01857283
4384	0.40625000	0.34375000	-0.02023460
4385	1.90625000	2.59375000	-0.14144673
4386	1.65625000	3.59375000	0.04486804
4387	1.68750000	3.59375000	0.04261975
4388	1.34375000	3.43750000	0.04252199
4389	1.43750000	3.34375000	0.01593353
4390	1.31250000	3.40625000	0.03607038
4391	1.40625000	3.31250000	0.00957967
4392	1.56250000	3.40625000	0.00997067
4393	1.59375000	3.40625000	0.00625611
4394	3.09375000	3.40625000	0.09257087
4395	3.65625000	3.09375000	0.08631476
4396	3.59375000	3.15625000	0.08670577
4397	2.65625000	3.09375000	-0.08699902
4398	2.59375000	3.15625000	-0.07409580
4399	3.09375000	2.40625000	-0.09775171
4400	1.65625000	3.09375000	-0.08611926
4401	1.59375000	3.15625000	-0.05816227

End of Node Coordinates

Connectivity Matrix of Nodes

Triangular Index	Node 1	Node 2	Node 3
1	1	1091	1092
2	291	2692	1091
3	292	1092	2692
4	1091	2692	1092
5	83	1512	1510
6	708	2693	1512
7	291	1510	2693
8	1512	2693	1510
9	84	1514	1517
10	292	2695	1514
11	708	1517	2695
12	1514	2695	1517
13	291	2693	2692

14	708	2695	2693
15	292	2692	2695
16	2693	2695	2692
17	27	1208	1206
18	408	2987	1208
19	406	1206	2987
20	1208	2987	1206
21	196	2128	2126
22	709	2992	2128
23	408	2126	2992
24	2128	2992	2126
25	83	1511	1513
26	406	2988	1511
27	709	1513	2988
28	1511	2988	1513
29	408	2992	2987
30	709	2988	2992
31	406	2987	2988
32	2992	2988	2987
33	28	1210	1213
34	410	2997	1210
35	413	1213	2997
36	1210	2997	1213
37	83	1513	1512
38	709	3845	1513
39	708	1512	3845
40	1513	3845	1512
41	196	2129	2128
42	711	3847	2129
43	709	2128	3847
44	2129	3847	2128
45	84	1517	1519
46	708	3846	1517
47	711	1519	3846
48	1517	3846	1519
49	709	3847	3845
50	711	3846	3847
51	708	3845	3846
52	3847	3846	3845
53	11	1128	1126
54	328	2771	1128
55	326	1126	2771
56	1128	2771	1126
57	120	1696	1694
58	787	2776	1696
59	328	1694	2776
60	1696	2776	1694
61	118	1686	1688
62	326	2772	1686
63	787	1688	2772
64	1686	2772	1688
65	328	2776	2771
66	787	2772	2776
67	326	2771	2772
68	2776	2772	2771
69	60	1376	1374
70	576	3446	1376
71	574	1374	3446
72	1376	3446	1374

73	197	2135	2133
74	792	3455	2135
75	576	2133	3455
76	2135	3455	2133
77	120	1695	1698
78	574	3448	1695
79	792	1698	3448
80	1695	3448	1698
81	576	3455	3446
82	792	3448	3455
83	574	3446	3448
84	3455	3448	3446
85	120	1698	1696
86	792	3924	1698
87	787	1696	3924
88	1698	3924	1696
89	197	2134	2135
90	788	3925	2134
91	792	2135	3925
92	2134	3925	2135
93	118	1688	1689
94	787	3923	1688
95	788	1689	3923
96	1688	3923	1689
97	792	3925	3924
98	788	3923	3925
99	787	3924	3923
100	3925	3923	3924
101	12	1130	1133
102	330	2781	1130
103	333	1133	2781
104	1130	2781	1133
105	122	1709	1706
106	797	2783	1709
107	330	1706	2783
108	1709	2783	1706
109	125	1724	1726
110	333	2792	1724
111	797	1726	2792
112	1724	2792	1726
113	330	2783	2781
114	797	2792	2783
115	333	2781	2792
116	2783	2792	2781
117	28	1215	1211
118	415	3001	1215
119	411	1211	3001
120	1215	3001	1211
121	199	2146	2144
122	799	3012	2146
123	415	2144	3012
124	2146	3012	2144
125	122	1707	1711
126	411	3003	1707
127	799	1711	3003
128	1707	3003	1711
129	415	3012	3001
130	799	3003	3012
131	411	3001	3003

132	3012	3003	3001
133	60	1375	1377
134	575	3450	1375
135	577	1377	3450
136	1375	3450	1377
137	125	1728	1725
138	808	3452	1728
139	575	1725	3452
140	1728	3452	1725
141	199	2145	2147
142	577	3457	2145
143	808	2147	3457
144	2145	3457	2147
145	575	3452	3450
146	808	3457	3452
147	577	3450	3457
148	3452	3457	3450
149	122	1711	1709
150	799	3934	1711
151	797	1709	3934
152	1711	3934	1709
153	199	2147	2146
154	808	3937	2147
155	799	2146	3937
156	2147	3937	2146
157	125	1726	1728
158	797	3935	1726
159	808	1728	3935
160	1726	3935	1728
161	799	3937	3934
162	808	3935	3937
163	797	3934	3935
164	3937	3935	3934
165	60	1377	1376
166	577	3454	1377
167	576	1376	3454
168	1377	3454	1376
169	199	2149	2145
170	999	3458	2149
171	577	2145	3458
172	2149	3458	2145
173	197	2133	2137
174	576	3456	2133
175	999	2137	3456
176	2133	3456	2137
177	577	3458	3454
178	999	3456	3458
179	576	3454	3456
180	3458	3456	3454
181	28	1213	1215
182	413	3007	1213
183	415	1215	3007
184	1213	3007	1215
185	196	2131	2127
186	998	3009	2131
187	413	2127	3009
188	2131	3009	2127
189	199	2144	2148
190	415	3013	2144

191	998	2148	3013
192	2144	3013	2148
193	413	3009	3007
194	998	3013	3009
195	415	3007	3013
196	3009	3013	3007
197	197	2137	2136
198	999	4134	2137
199	997	2136	4134
200	2137	4134	2136
201	199	2148	2149
202	998	4135	2148
203	999	2149	4135
204	2148	4135	2149
205	196	2130	2131
206	997	4133	2130
207	998	2131	4133
208	2130	4133	2131
209	999	4135	4134
210	998	4133	4135
211	997	4134	4133
212	4135	4133	4134
213	6	1106	1104
214	306	2717	1106
215	304	1104	2717
216	1106	2717	1104
217	98	1580	1578
218	733	2722	1580
219	306	1578	2722
220	1580	2722	1578
221	96	1570	1572
222	304	2718	1570
223	733	1572	2718
224	1570	2718	1572
225	306	2722	2717
226	733	2718	2722
227	304	2717	2718
228	2722	2718	2717
229	42	1276	1274
230	476	3158	1276
231	474	1274	3158
232	1276	3158	1274
233	221	2279	2277
234	738	3167	2279
235	476	2277	3167
236	2279	3167	2277
237	98	1579	1582
238	474	3160	1579
239	738	1582	3160
240	1579	3160	1582
241	476	3167	3158
242	738	3160	3167
243	474	3158	3160
244	3167	3160	3158
245	40	1266	1268
246	466	3140	1266
247	468	1268	3140
248	1266	3140	1268
249	96	1573	1571

250	734	3141	1573
251	466	1571	3141
252	1573	3141	1571
253	221	2276	2278
254	468	3145	2276
255	734	2278	3145
256	2276	3145	2278
257	466	3141	3140
258	734	3145	3141
259	468	3140	3145
260	3141	3145	3140
261	98	1582	1580
262	738	3870	1582
263	733	1580	3870
264	1582	3870	1580
265	221	2278	2279
266	734	3871	2278
267	738	2279	3871
268	2278	3871	2279
269	96	1572	1573
270	733	3869	1572
271	734	1573	3869
272	1572	3869	1573
273	738	3871	3870
274	734	3869	3871
275	733	3870	3869
276	3871	3869	3870
277	22	1180	1178
278	380	2906	1180
279	378	1178	2906
280	1180	2906	1178
281	172	1984	1982
282	922	2915	1984
283	380	1982	2915
284	1984	2915	1982
285	170	1970	1972
286	378	2908	1970
287	922	1972	2908
288	1970	2908	1972
289	380	2915	2906
290	922	2908	2915
291	378	2906	2908
292	2915	2908	2906
293	61	1383	1381
294	583	3468	1383
295	581	1381	3468
296	1383	3468	1381
297	226	2309	2307
298	931	3475	2309
299	583	2307	3475
300	2309	3475	2307
301	172	1983	1986
302	581	3470	1983
303	931	1986	3470
304	1983	3470	1986
305	583	3475	3468
306	931	3470	3475
307	581	3468	3470
308	3475	3470	3468

309	42	1275	1278
310	475	3162	1275
311	478	1278	3162
312	1275	3162	1278
313	170	1974	1971
314	924	3164	1974
315	475	1971	3164
316	1974	3164	1971
317	226	2306	2308
318	478	3172	2306
319	924	2308	3172
320	2306	3172	2308
321	475	3164	3162
322	924	3172	3164
323	478	3162	3172
324	3164	3172	3162
325	172	1986	1984
326	931	4059	1986
327	922	1984	4059
328	1986	4059	1984
329	226	2308	2309
330	924	4062	2308
331	931	2309	4062
332	2308	4062	2309
333	170	1972	1974
334	922	4058	1972
335	924	1974	4058
336	1972	4058	1974
337	931	4062	4059
338	924	4058	4062
339	922	4059	4058
340	4062	4058	4059
341	11	1127	1129
342	327	2773	1127
343	329	1129	2773
344	1127	2773	1129
345	119	1692	1690
346	789	2774	1692
347	327	1690	2774
348	1692	2774	1690
349	121	1700	1702
350	329	2778	1700
351	789	1702	2778
352	1700	2778	1702
353	327	2774	2773
354	789	2778	2774
355	329	2773	2778
356	2774	2778	2773
357	40	1269	1267
358	469	3142	1269
359	467	1267	3142
360	1269	3142	1267
361	222	2284	2282
362	790	3147	2284
363	469	2282	3147
364	2284	3147	2282
365	119	1691	1693
366	467	3143	1691
367	790	1693	3143

368	1691	3143	1693
369	469	3147	3142
370	790	3143	3147
371	467	3142	3143
372	3147	3143	3142
373	61	1380	1382
374	580	3464	1380
375	582	1382	3464
376	1380	3464	1382
377	121	1704	1701
378	794	3466	1704
379	580	1701	3466
380	1704	3466	1701
381	222	2283	2285
382	582	3473	2283
383	794	2285	3473
384	2283	3473	2285
385	580	3466	3464
386	794	3473	3466
387	582	3464	3473
388	3466	3473	3464
389	119	1693	1692
390	790	3926	1693
391	789	1692	3926
392	1693	3926	1692
393	222	2285	2284
394	794	3928	2285
395	790	2284	3928
396	2285	3928	2284
397	121	1702	1704
398	789	3927	1702
399	794	1704	3927
400	1702	3927	1704
401	790	3928	3926
402	794	3927	3928
403	789	3926	3927
404	3928	3927	3926
405	42	1278	1276
406	478	3166	1278
407	476	1276	3166
408	1278	3166	1276
409	226	2310	2306
410	1022	3173	2310
411	478	2306	3173
412	2310	3173	2306
413	221	2277	2281
414	476	3168	2277
415	1022	2281	3168
416	2277	3168	2281
417	478	3173	3166
418	1022	3168	3173
419	476	3166	3168
420	3173	3168	3166
421	61	1382	1383
422	582	3472	1382
423	583	1383	3472
424	1382	3472	1383
425	222	2287	2283
426	1023	3474	2287

427	582	2283	3474
428	2287	3474	2283
429	226	2307	2311
430	583	3476	2307
431	1023	2311	3476
432	2307	3476	2311
433	582	3474	3472
434	1023	3476	3474
435	583	3472	3476
436	3474	3476	3472
437	40	1268	1269
438	468	3144	1268
439	469	1269	3144
440	1268	3144	1269
441	221	2280	2276
442	1021	3146	2280
443	468	2276	3146
444	2280	3146	2276
445	222	2282	2286
446	469	3148	2282
447	1021	2286	3148
448	2282	3148	2286
449	468	3146	3144
450	1021	3148	3146
451	469	3144	3148
452	3146	3148	3144
453	226	2311	2310
454	1023	4159	2311
455	1022	2310	4159
456	2311	4159	2310
457	222	2286	2287
458	1021	4158	2286
459	1023	2287	4158
460	2286	4158	2287
461	221	2281	2280
462	1022	4157	2281
463	1021	2280	4157
464	2281	4157	2280
465	1023	4158	4159
466	1021	4157	4158
467	1022	4159	4157
468	4158	4157	4159
469	7	1108	1111
470	308	2727	1108
471	311	1111	2727
472	1108	2727	1111
473	100	1593	1590
474	743	2729	1593
475	308	1590	2729
476	1593	2729	1590
477	103	1608	1610
478	311	2738	1608
479	743	1610	2738
480	1608	2738	1610
481	308	2729	2727
482	743	2738	2729
483	311	2727	2738
484	2729	2738	2727
485	44	1289	1286

486	489	3195	1289
487	486	1286	3195
488	1289	3195	1286
489	231	2338	2336
490	745	3206	2338
491	489	2336	3206
492	2338	3206	2336
493	100	1591	1595
494	486	3197	1591
495	745	1595	3197
496	1591	3197	1595
497	489	3206	3195
498	745	3197	3206
499	486	3195	3197
500	3206	3197	3195
501	47	1304	1306
502	504	3248	1304
503	506	1306	3248
504	1304	3248	1306
505	103	1612	1609
506	754	3250	1612
507	504	1609	3250
508	1612	3250	1609
509	231	2337	2339
510	506	3257	2337
511	754	2339	3257
512	2337	3257	2339
513	504	3250	3248
514	754	3257	3250
515	506	3248	3257
516	3250	3257	3248
517	100	1595	1593
518	745	3880	1595
519	743	1593	3880
520	1595	3880	1593
521	231	2339	2338
522	754	3883	2339
523	745	2338	3883
524	2339	3883	2338
525	103	1610	1612
526	743	3881	1610
527	754	1612	3881
528	1610	3881	1612
529	745	3883	3880
530	754	3881	3883
531	743	3880	3881
532	3883	3881	3880
533	12	1135	1131
534	335	2785	1135
535	331	1131	2785
536	1135	2785	1131
537	127	1738	1736
538	801	2796	1738
539	335	1736	2796
540	1738	2796	1736
541	123	1712	1715
542	331	2787	1712
543	801	1715	2787
544	1712	2787	1715

545	335	2796	2785
546	801	2787	2796
547	331	2785	2787
548	2796	2787	2785
549	63	1394	1392
550	594	3500	1394
551	592	1392	3500
552	1394	3500	1392
553	233	2351	2349
554	812	3509	2351
555	594	2349	3509
556	2351	3509	2349
557	127	1737	1740
558	592	3502	1737
559	812	1740	3502
560	1737	3502	1740
561	594	3509	3500
562	812	3502	3509
563	592	3500	3502
564	3509	3502	3500
565	44	1287	1291
566	487	3199	1287
567	491	1291	3199
568	1287	3199	1291
569	123	1717	1713
570	803	3201	1717
571	487	1713	3201
572	1717	3201	1713
573	233	2348	2350
574	491	3210	2348
575	803	2350	3210
576	2348	3210	2350
577	487	3201	3199
578	803	3210	3201
579	491	3199	3210
580	3201	3210	3199
581	127	1740	1738
582	812	3941	1740
583	801	1738	3941
584	1740	3941	1738
585	233	2350	2351
586	803	3943	2350
587	812	2351	3943
588	2350	3943	2351
589	123	1715	1717
590	801	3940	1715
591	803	1717	3940
592	1715	3940	1717
593	812	3943	3941
594	803	3940	3943
595	801	3941	3940
596	3943	3940	3941
597	22	1179	1181
598	379	2910	1179
599	381	1181	2910
600	1179	2910	1181
601	171	1978	1976
602	926	2912	1978
603	379	1976	2912

604	1978	2912	1976
605	173	1988	1990
606	381	2917	1988
607	926	1990	2917
608	1988	2917	1990
609	379	2912	2910
610	926	2917	2912
611	381	2910	2917
612	2912	2917	2910
613	47	1308	1305
614	508	3252	1308
615	505	1305	3252
616	1308	3252	1305
617	242	2404	2402
618	928	3262	2404
619	508	2402	3262
620	2404	3262	2402
621	171	1977	1980
622	505	3254	1977
623	928	1980	3254
624	1977	3254	1980
625	508	3262	3252
626	928	3254	3262
627	505	3252	3254
628	3262	3254	3252
629	63	1393	1395
630	593	3504	1393
631	595	1395	3504
632	1393	3504	1395
633	173	1992	1989
634	933	3506	1992
635	593	1989	3506
636	1992	3506	1989
637	242	2403	2405
638	595	3511	2403
639	933	2405	3511
640	2403	3511	2405
641	593	3506	3504
642	933	3511	3506
643	595	3504	3511
644	3506	3511	3504
645	171	1980	1978
646	928	4064	1980
647	926	1978	4064
648	1980	4064	1978
649	242	2405	2404
650	933	4068	2405
651	928	2404	4068
652	2405	4068	2404
653	173	1990	1992
654	926	4065	1990
655	933	1992	4065
656	1990	4065	1992
657	928	4068	4064
658	933	4065	4068
659	926	4064	4065
660	4068	4065	4064
661	44	1291	1289
662	491	3205	1291

663	489	1289	3205
664	1291	3205	1289
665	233	2352	2348
666	1032	3211	2352
667	491	2348	3211
668	2352	3211	2348
669	231	2336	2340
670	489	3207	2336
671	1032	2340	3207
672	2336	3207	2340
673	491	3211	3205
674	1032	3207	3211
675	489	3205	3207
676	3211	3207	3205
677	63	1395	1394
678	595	3508	1395
679	594	1394	3508
680	1395	3508	1394
681	242	2407	2403
682	1035	3512	2407
683	595	2403	3512
684	2407	3512	2403
685	233	2349	2353
686	594	3510	2349
687	1035	2353	3510
688	2349	3510	2353
689	595	3512	3508
690	1035	3510	3512
691	594	3508	3510
692	3512	3510	3508
693	47	1306	1308
694	506	3256	1306
695	508	1308	3256
696	1306	3256	1308
697	231	2341	2337
698	1033	3258	2341
699	506	2337	3258
700	2341	3258	2337
701	242	2402	2406
702	508	3263	2402
703	1033	2406	3263
704	2402	3263	2406
705	506	3258	3256
706	1033	3263	3258
707	508	3256	3263
708	3258	3263	3256
709	233	2353	2352
710	1035	4170	2353
711	1032	2352	4170
712	2353	4170	2352
713	242	2406	2407
714	1033	4171	2406
715	1035	2407	4171
716	2406	4171	2407
717	231	2340	2341
718	1032	4169	2340
719	1033	2341	4169
720	2340	4169	2341
721	1035	4171	4170

722	1033	4169	4171
723	1032	4170	4169
724	4171	4169	4170
725	11	1129	1128
726	329	2775	1129
727	328	1128	2775
728	1129	2775	1128
729	121	1703	1700
730	791	2779	1703
731	329	1700	2779
732	1703	2779	1700
733	120	1694	1697
734	328	2777	1694
735	791	1697	2777
736	1694	2777	1697
737	329	2779	2775
738	791	2777	2779
739	328	2775	2777
740	2779	2777	2775
741	61	1384	1380
742	584	3465	1384
743	580	1380	3465
744	1384	3465	1380
745	269	2567	2565
746	795	3478	2567
747	584	2565	3478
748	2567	3478	2565
749	121	1701	1705
750	580	3467	1701
751	795	1705	3467
752	1701	3467	1705
753	584	3478	3465
754	795	3467	3478
755	580	3465	3467
756	3478	3467	3465
757	60	1374	1378
758	574	3447	1374
759	578	1378	3447
760	1374	3447	1378
761	120	1699	1695
762	793	3449	1699
763	574	1695	3449
764	1699	3449	1695
765	269	2564	2566
766	578	3460	2564
767	793	2566	3460
768	2564	3460	2566
769	574	3449	3447
770	793	3460	3449
771	578	3447	3460
772	3449	3460	3447
773	121	1705	1703
774	795	3930	1705
775	791	1703	3930
776	1705	3930	1703
777	269	2566	2567
778	793	3931	2566
779	795	2567	3931
780	2566	3931	2567

781	120	1697	1699
782	791	3929	1697
783	793	1699	3929
784	1697	3929	1699
785	795	3931	3930
786	793	3929	3931
787	791	3930	3929
788	3931	3929	3930
789	22	1181	1180
790	381	2914	1181
791	380	1180	2914
792	1181	2914	1180
793	173	1991	1988
794	930	2918	1991
795	381	1988	2918
796	1991	2918	1988
797	172	1982	1985
798	380	2916	1982
799	930	1985	2916
800	1982	2916	1985
801	381	2918	2914
802	930	2916	2918
803	380	2914	2916
804	2918	2916	2914
805	63	1397	1393
806	597	3505	1397
807	593	1393	3505
808	1397	3505	1393
809	271	2579	2577
810	934	3516	2579
811	597	2577	3516
812	2579	3516	2577
813	173	1989	1993
814	593	3507	1989
815	934	1993	3507
816	1989	3507	1993
817	597	3516	3505
818	934	3507	3516
819	593	3505	3507
820	3516	3507	3505
821	61	1381	1385
822	581	3469	1381
823	585	1385	3469
824	1381	3469	1385
825	172	1987	1983
826	932	3471	1987
827	581	1983	3471
828	1987	3471	1983
829	271	2576	2578
830	585	3480	2576
831	932	2578	3480
832	2576	3480	2578
833	581	3471	3469
834	932	3480	3471
835	585	3469	3480
836	3471	3480	3469
837	173	1993	1991
838	934	4071	1993
839	930	1991	4071

840	1993	4071	1991
841	271	2578	2579
842	932	4072	2578
843	934	2579	4072
844	2578	4072	2579
845	172	1985	1987
846	930	4070	1985
847	932	1987	4070
848	1985	4070	1987
849	934	4072	4071
850	932	4070	4072
851	930	4071	4070
852	4072	4070	4071
853	12	1133	1135
854	333	2791	1133
855	335	1135	2791
856	1133	2791	1135
857	125	1727	1724
858	807	2793	1727
859	333	1724	2793
860	1727	2793	1724
861	127	1736	1739
862	335	2797	1736
863	807	1739	2797
864	1736	2797	1739
865	333	2793	2791
866	807	2797	2793
867	335	2791	2797
868	2793	2797	2791
869	60	1379	1375
870	579	3451	1379
871	575	1375	3451
872	1379	3451	1375
873	270	2572	2570
874	809	3462	2572
875	579	2570	3462
876	2572	3462	2570
877	125	1725	1729
878	575	3453	1725
879	809	1729	3453
880	1725	3453	1729
881	579	3462	3451
882	809	3453	3462
883	575	3451	3453
884	3462	3453	3451
885	63	1392	1396
886	592	3501	1392
887	596	1396	3501
888	1392	3501	1396
889	127	1741	1737
890	813	3503	1741
891	592	1737	3503
892	1741	3503	1737
893	270	2571	2573
894	596	3514	2571
895	813	2573	3514
896	2571	3514	2573
897	592	3503	3501
898	813	3514	3503

899	596	3501	3514
900	3503	3514	3501
901	125	1729	1727
902	809	3947	1729
903	807	1727	3947
904	1729	3947	1727
905	270	2573	2572
906	813	3949	2573
907	809	2572	3949
908	2573	3949	2572
909	127	1739	1741
910	807	3948	1739
911	813	1741	3948
912	1739	3948	1741
913	809	3949	3947
914	813	3948	3949
915	807	3947	3948
916	3949	3948	3947
917	61	1385	1384
918	585	3477	1385
919	584	1384	3477
920	1385	3477	1384
921	271	2580	2576
922	1070	3481	2580
923	585	2576	3481
924	2580	3481	2576
925	269	2565	2569
926	584	3479	2565
927	1070	2569	3479
928	2565	3479	2569
929	585	3481	3477
930	1070	3479	3481
931	584	3477	3479
932	3481	3479	3477
933	63	1396	1397
934	596	3513	1396
935	597	1397	3513
936	1396	3513	1397
937	270	2575	2571
938	1071	3515	2575
939	596	2571	3515
940	2575	3515	2571
941	271	2577	2581
942	597	3517	2577
943	1071	2581	3517
944	2577	3517	2581
945	596	3515	3513
946	1071	3517	3515
947	597	3513	3517
948	3515	3517	3513
949	60	1378	1379
950	578	3459	1378
951	579	1379	3459
952	1378	3459	1379
953	269	2568	2564
954	1069	3461	2568
955	578	2564	3461
956	2568	3461	2564
957	270	2570	2574

958	579	3463	2570
959	1069	2574	3463
960	2570	3463	2574
961	578	3461	3459
962	1069	3463	3461
963	579	3459	3463
964	3461	3463	3459
965	271	2581	2580
966	1071	4207	2581
967	1070	2580	4207
968	2581	4207	2580
969	270	2574	2575
970	1069	4206	2574
971	1071	2575	4206
972	2574	4206	2575
973	269	2569	2568
974	1070	4205	2569
975	1069	2568	4205
976	2569	4205	2568
977	1071	4206	4207
978	1069	4205	4206
979	1070	4207	4205
980	4206	4205	4207
981	3	1096	1095
982	296	2699	1096
983	295	1095	2699
984	1096	2699	1095
985	88	1534	1532
986	715	2701	1534
987	296	1532	2701
988	1534	2701	1532
989	87	1528	1530
990	295	2700	1528
991	715	1530	2700
992	1528	2700	1530
993	296	2701	2699
994	715	2700	2701
995	295	2699	2700
996	2701	2700	2699
997	32	1230	1228
998	430	3041	1230
999	428	1228	3041
1000	1230	3041	1228
1001	203	2171	2169
1002	717	3046	2171
1003	430	2169	3046
1004	2171	3046	2169
1005	88	1533	1535
1006	428	3042	1533
1007	717	1535	3042
1008	1533	3042	1535
1009	430	3046	3041
1010	717	3042	3046
1011	428	3041	3042
1012	3046	3042	3041
1013	31	1224	1226
1014	424	3032	1224
1015	426	1226	3032
1016	1224	3032	1226

1017	87	1531	1529
1018	716	3033	1531
1019	424	1529	3033
1020	1531	3033	1529
1021	203	2168	2170
1022	426	3037	2168
1023	716	2170	3037
1024	2168	3037	2170
1025	424	3033	3032
1026	716	3037	3033
1027	426	3032	3037
1028	3033	3037	3032
1029	88	1535	1534
1030	717	3852	1535
1031	715	1534	3852
1032	1535	3852	1534
1033	203	2170	2171
1034	716	3853	2170
1035	717	2171	3853
1036	2170	3853	2171
1037	87	1530	1531
1038	715	3851	1530
1039	716	1531	3851
1040	1530	3851	1531
1041	717	3853	3852
1042	716	3851	3853
1043	715	3852	3851
1044	3853	3851	3852
1045	16	1150	1148
1046	350	2825	1150
1047	348	1148	2825
1048	1150	2825	1148
1049	142	1812	1810
1050	841	2830	1812
1051	350	1810	2830
1052	1812	2830	1810
1053	140	1802	1804
1054	348	2826	1802
1055	841	1804	2826
1056	1802	2826	1804
1057	350	2830	2825
1058	841	2826	2830
1059	348	2825	2826
1060	2830	2826	2825
1061	67	1419	1417
1062	619	3576	1419
1063	617	1417	3576
1064	1419	3576	1417
1065	205	2183	2181
1066	846	3583	2183
1067	619	2181	3583
1068	2183	3583	2181
1069	142	1811	1814
1070	617	3578	1811
1071	846	1814	3578
1072	1811	3578	1814
1073	619	3583	3576
1074	846	3578	3583
1075	617	3576	3578

1076	3583	3578	3576
1077	32	1229	1231
1078	429	3043	1229
1079	431	1231	3043
1080	1229	3043	1231
1081	140	1805	1803
1082	842	3044	1805
1083	429	1803	3044
1084	1805	3044	1803
1085	205	2180	2182
1086	431	3048	2180
1087	842	2182	3048
1088	2180	3048	2182
1089	429	3044	3043
1090	842	3048	3044
1091	431	3043	3048
1092	3044	3048	3043
1093	142	1814	1812
1094	846	3978	1814
1095	841	1812	3978
1096	1814	3978	1812
1097	205	2182	2183
1098	842	3979	2182
1099	846	2183	3979
1100	2182	3979	2183
1101	140	1804	1805
1102	841	3977	1804
1103	842	1805	3977
1104	1804	3977	1805
1105	846	3979	3978
1106	842	3977	3979
1107	841	3978	3977
1108	3979	3977	3978
1109	15	1144	1146
1110	344	2816	1144
1111	346	1146	2816
1112	1144	2816	1146
1113	136	1784	1782
1114	832	2817	1784
1115	344	1782	2817
1116	1784	2817	1782
1117	138	1790	1792
1118	346	2821	1790
1119	832	1792	2821
1120	1790	2821	1792
1121	344	2817	2816
1122	832	2821	2817
1123	346	2816	2821
1124	2817	2821	2816
1125	31	1227	1225
1126	427	3034	1227
1127	425	1225	3034
1128	1227	3034	1225
1129	204	2176	2174
1130	833	3039	2176
1131	427	2174	3039
1132	2176	3039	2174
1133	136	1783	1785
1134	425	3035	1783

1135	833	1785	3035
1136	1783	3035	1785
1137	427	3039	3034
1138	833	3035	3039
1139	425	3034	3035
1140	3039	3035	3034
1141	67	1416	1418
1142	616	3572	1416
1143	618	1418	3572
1144	1416	3572	1418
1145	138	1794	1791
1146	837	3574	1794
1147	616	1791	3574
1148	1794	3574	1791
1149	204	2175	2177
1150	618	3581	2175
1151	837	2177	3581
1152	2175	3581	2177
1153	616	3574	3572
1154	837	3581	3574
1155	618	3572	3581
1156	3574	3581	3572
1157	136	1785	1784
1158	833	3968	1785
1159	832	1784	3968
1160	1785	3968	1784
1161	204	2177	2176
1162	837	3970	2177
1163	833	2176	3970
1164	2177	3970	2176
1165	138	1792	1794
1166	832	3969	1792
1167	837	1794	3969
1168	1792	3969	1794
1169	833	3970	3968
1170	837	3969	3970
1171	832	3968	3969
1172	3970	3969	3968
1173	32	1231	1230
1174	431	3045	1231
1175	430	1230	3045
1176	1231	3045	1230
1177	205	2184	2180
1178	1004	3049	2184
1179	431	2180	3049
1180	2184	3049	2180
1181	203	2169	2173
1182	430	3047	2169
1183	1004	2173	3047
1184	2169	3047	2173
1185	431	3049	3045
1186	1004	3047	3049
1187	430	3045	3047
1188	3049	3047	3045
1189	67	1418	1419
1190	618	3580	1418
1191	619	1419	3580
1192	1418	3580	1419
1193	204	2179	2175

1194	1005	3582	2179
1195	618	2175	3582
1196	2179	3582	2175
1197	205	2181	2185
1198	619	3584	2181
1199	1005	2185	3584
1200	2181	3584	2185
1201	618	3582	3580
1202	1005	3584	3582
1203	619	3580	3584
1204	3582	3584	3580
1205	31	1226	1227
1206	426	3036	1226
1207	427	1227	3036
1208	1226	3036	1227
1209	203	2172	2168
1210	1003	3038	2172
1211	426	2168	3038
1212	2172	3038	2168
1213	204	2174	2178
1214	427	3040	2174
1215	1003	2178	3040
1216	2174	3040	2178
1217	426	3038	3036
1218	1003	3040	3038
1219	427	3036	3040
1220	3038	3040	3036
1221	205	2185	2184
1222	1005	4141	2185
1223	1004	2184	4141
1224	2185	4141	2184
1225	204	2178	2179
1226	1003	4140	2178
1227	1005	2179	4140
1228	2178	4140	2179
1229	203	2173	2172
1230	1004	4139	2173
1231	1003	2172	4139
1232	2173	4139	2172
1233	1005	4140	4141
1234	1003	4139	4140
1235	1004	4141	4139
1236	4140	4139	4141
1237	9	1120	1118
1238	320	2753	1120
1239	318	1118	2753
1240	1120	2753	1118
1241	112	1656	1654
1242	769	2758	1656
1243	320	1654	2758
1244	1656	2758	1654
1245	110	1646	1648
1246	318	2754	1646
1247	769	1648	2754
1248	1646	2754	1648
1249	320	2758	2753
1250	769	2754	2758
1251	318	2753	2754
1252	2758	2754	2753

1253	56	1352	1350
1254	552	3374	1352
1255	550	1350	3374
1256	1352	3374	1350
1257	257	2495	2493
1258	774	3383	2495
1259	552	2493	3383
1260	2495	3383	2493
1261	112	1655	1658
1262	550	3376	1655
1263	774	1658	3376
1264	1655	3376	1658
1265	552	3383	3374
1266	774	3376	3383
1267	550	3374	3376
1268	3383	3376	3374
1269	54	1342	1344
1270	542	3356	1342
1271	544	1344	3356
1272	1342	3356	1344
1273	110	1649	1647
1274	770	3357	1649
1275	542	1647	3357
1276	1649	3357	1647
1277	257	2492	2494
1278	544	3361	2492
1279	770	2494	3361
1280	2492	3361	2494
1281	542	3357	3356
1282	770	3361	3357
1283	544	3356	3361
1284	3357	3361	3356
1285	112	1658	1656
1286	774	3906	1658
1287	769	1656	3906
1288	1658	3906	1656
1289	257	2494	2495
1290	770	3907	2494
1291	774	2495	3907
1292	2494	3907	2495
1293	110	1648	1649
1294	769	3905	1648
1295	770	1649	3905
1296	1648	3905	1649
1297	774	3907	3906
1298	770	3905	3907
1299	769	3906	3905
1300	3907	3905	3906
1301	23	1187	1185
1302	387	2928	1187
1303	385	1185	2928
1304	1187	2928	1185
1305	179	2026	2024
1306	944	2935	2026
1307	387	2024	2935
1308	2026	2935	2024
1309	177	2012	2014
1310	385	2930	2012
1311	944	2014	2930

1312	2012	2930	2014
1313	387	2935	2928
1314	944	2930	2935
1315	385	2928	2930
1316	2935	2930	2928
1317	69	1431	1429
1318	631	3612	1431
1319	629	1429	3612
1320	1431	3612	1429
1321	262	2525	2523
1322	951	3619	2525
1323	631	2523	3619
1324	2525	3619	2523
1325	179	2025	2028
1326	629	3614	2025
1327	951	2028	3614
1328	2025	3614	2028
1329	631	3619	3612
1330	951	3614	3619
1331	629	3612	3614
1332	3619	3614	3612
1333	56	1351	1354
1334	551	3378	1351
1335	554	1354	3378
1336	1351	3378	1354
1337	177	2016	2013
1338	946	3380	2016
1339	551	2013	3380
1340	2016	3380	2013
1341	262	2522	2524
1342	554	3388	2522
1343	946	2524	3388
1344	2522	3388	2524
1345	551	3380	3378
1346	946	3388	3380
1347	554	3378	3388
1348	3380	3388	3378
1349	179	2028	2026
1350	951	4083	2028
1351	944	2026	4083
1352	2028	4083	2026
1353	262	2524	2525
1354	946	4086	2524
1355	951	2525	4086
1356	2524	4086	2525
1357	177	2014	2016
1358	944	4082	2014
1359	946	2016	4082
1360	2014	4082	2016
1361	951	4086	4083
1362	946	4082	4086
1363	944	4083	4082
1364	4086	4082	4083
1365	16	1149	1151
1366	349	2827	1149
1367	351	1151	2827
1368	1149	2827	1151
1369	141	1808	1806
1370	843	2828	1808

1371	349	1806	2828
1372	1808	2828	1806
1373	143	1816	1818
1374	351	2832	1816
1375	843	1818	2832
1376	1816	2832	1818
1377	349	2828	2827
1378	843	2832	2828
1379	351	2827	2832
1380	2828	2832	2827
1381	54	1345	1343
1382	545	3358	1345
1383	543	1343	3358
1384	1345	3358	1343
1385	258	2500	2498
1386	844	3363	2500
1387	545	2498	3363
1388	2500	3363	2498
1389	141	1807	1809
1390	543	3359	1807
1391	844	1809	3359
1392	1807	3359	1809
1393	545	3363	3358
1394	844	3359	3363
1395	543	3358	3359
1396	3363	3359	3358
1397	69	1428	1430
1398	628	3608	1428
1399	630	1430	3608
1400	1428	3608	1430
1401	143	1820	1817
1402	848	3610	1820
1403	628	1817	3610
1404	1820	3610	1817
1405	258	2499	2501
1406	630	3617	2499
1407	848	2501	3617
1408	2499	3617	2501
1409	628	3610	3608
1410	848	3617	3610
1411	630	3608	3617
1412	3610	3617	3608
1413	141	1809	1808
1414	844	3980	1809
1415	843	1808	3980
1416	1809	3980	1808
1417	258	2501	2500
1418	848	3982	2501
1419	844	2500	3982
1420	2501	3982	2500
1421	143	1818	1820
1422	843	3981	1818
1423	848	1820	3981
1424	1818	3981	1820
1425	844	3982	3980
1426	848	3981	3982
1427	843	3980	3981
1428	3982	3981	3980
1429	56	1354	1352

1430	554	3382	1354
1431	552	1352	3382
1432	1354	3382	1352
1433	262	2526	2522
1434	1058	3389	2526
1435	554	2522	3389
1436	2526	3389	2522
1437	257	2493	2497
1438	552	3384	2493
1439	1058	2497	3384
1440	2493	3384	2497
1441	554	3389	3382
1442	1058	3384	3389
1443	552	3382	3384
1444	3389	3384	3382
1445	69	1430	1431
1446	630	3616	1430
1447	631	1431	3616
1448	1430	3616	1431
1449	258	2503	2499
1450	1059	3618	2503
1451	630	2499	3618
1452	2503	3618	2499
1453	262	2523	2527
1454	631	3620	2523
1455	1059	2527	3620
1456	2523	3620	2527
1457	630	3618	3616
1458	1059	3620	3618
1459	631	3616	3620
1460	3618	3620	3616
1461	54	1344	1345
1462	544	3360	1344
1463	545	1345	3360
1464	1344	3360	1345
1465	257	2496	2492
1466	1057	3362	2496
1467	544	2492	3362
1468	2496	3362	2492
1469	258	2498	2502
1470	545	3364	2498
1471	1057	2502	3364
1472	2498	3364	2502
1473	544	3362	3360
1474	1057	3364	3362
1475	545	3360	3364
1476	3362	3364	3360
1477	262	2527	2526
1478	1059	4195	2527
1479	1058	2526	4195
1480	2527	4195	2526
1481	258	2502	2503
1482	1057	4194	2502
1483	1059	2503	4194
1484	2502	4194	2503
1485	257	2497	2496
1486	1058	4193	2497
1487	1057	2496	4193
1488	2497	4193	2496

1489	1059	4194	4195
1490	1057	4193	4194
1491	1058	4195	4193
1492	4194	4193	4195
1493	6	1105	1107
1494	305	2719	1105
1495	307	1107	2719
1496	1105	2719	1107
1497	97	1576	1574
1498	735	2720	1576
1499	305	1574	2720
1500	1576	2720	1574
1501	99	1584	1586
1502	307	2724	1584
1503	735	1586	2724
1504	1584	2724	1586
1505	305	2720	2719
1506	735	2724	2720
1507	307	2719	2724
1508	2720	2724	2719
1509	41	1272	1270
1510	472	3149	1272
1511	470	1270	3149
1512	1272	3149	1270
1513	223	2290	2288
1514	736	3154	2290
1515	472	2288	3154
1516	2290	3154	2288
1517	97	1575	1577
1518	470	3150	1575
1519	736	1577	3150
1520	1575	3150	1577
1521	472	3154	3149
1522	736	3150	3154
1523	470	3149	3150
1524	3154	3150	3149
1525	43	1280	1282
1526	480	3176	1280
1527	482	1282	3176
1528	1280	3176	1282
1529	99	1588	1585
1530	740	3178	1588
1531	480	1585	3178
1532	1588	3178	1585
1533	223	2289	2291
1534	482	3185	2289
1535	740	2291	3185
1536	2289	3185	2291
1537	480	3178	3176
1538	740	3185	3178
1539	482	3176	3185
1540	3178	3185	3176
1541	97	1577	1576
1542	736	3872	1577
1543	735	1576	3872
1544	1577	3872	1576
1545	223	2291	2290
1546	740	3874	2291
1547	736	2290	3874

1548	2291	3874	2290
1549	99	1586	1588
1550	735	3873	1586
1551	740	1588	3873
1552	1586	3873	1588
1553	736	3874	3872
1554	740	3873	3874
1555	735	3872	3873
1556	3874	3873	3872
1557	15	1147	1145
1558	347	2818	1147
1559	345	1145	2818
1560	1147	2818	1145
1561	139	1798	1796
1562	834	2823	1798
1563	347	1796	2823
1564	1798	2823	1796
1565	137	1786	1788
1566	345	2819	1786
1567	834	1788	2819
1568	1786	2819	1788
1569	347	2823	2818
1570	834	2819	2823
1571	345	2818	2819
1572	2823	2819	2818
1573	68	1424	1422
1574	624	3590	1424
1575	622	1422	3590
1576	1424	3590	1422
1577	224	2297	2295
1578	839	3599	2297
1579	624	2295	3599
1580	2297	3599	2295
1581	139	1797	1800
1582	622	3592	1797
1583	839	1800	3592
1584	1797	3592	1800
1585	624	3599	3590
1586	839	3592	3599
1587	622	3590	3592
1588	3599	3592	3590
1589	41	1271	1273
1590	471	3151	1271
1591	473	1273	3151
1592	1271	3151	1273
1593	137	1789	1787
1594	835	3152	1789
1595	471	1787	3152
1596	1789	3152	1787
1597	224	2294	2296
1598	473	3156	2294
1599	835	2296	3156
1600	2294	3156	2296
1601	471	3152	3151
1602	835	3156	3152
1603	473	3151	3156
1604	3152	3156	3151
1605	139	1800	1798
1606	839	3972	1800

1607	834	1798	3972
1608	1800	3972	1798
1609	224	2296	2297
1610	835	3973	2296
1611	839	2297	3973
1612	2296	3973	2297
1613	137	1788	1789
1614	834	3971	1788
1615	835	1789	3971
1616	1788	3971	1789
1617	839	3973	3972
1618	835	3971	3973
1619	834	3972	3971
1620	3973	3971	3972
1621	23	1184	1186
1622	384	2924	1184
1623	386	1186	2924
1624	1184	2924	1186
1625	176	2008	2006
1626	940	2926	2008
1627	384	2006	2926
1628	2008	2926	2006
1629	178	2018	2020
1630	386	2933	2018
1631	940	2020	2933
1632	2018	2933	2020
1633	384	2926	2924
1634	940	2933	2926
1635	386	2924	2933
1636	2926	2933	2924
1637	43	1284	1281
1638	484	3180	1284
1639	481	1281	3180
1640	1284	3180	1281
1641	228	2320	2318
1642	942	3190	2320
1643	484	2318	3190
1644	2320	3190	2318
1645	176	2007	2010
1646	481	3182	2007
1647	942	2010	3182
1648	2007	3182	2010
1649	484	3190	3180
1650	942	3182	3190
1651	481	3180	3182
1652	3190	3182	3180
1653	68	1423	1425
1654	623	3594	1423
1655	625	1425	3594
1656	1423	3594	1425
1657	178	2022	2019
1658	949	3596	2022
1659	623	2019	3596
1660	2022	3596	2019
1661	228	2319	2321
1662	625	3601	2319
1663	949	2321	3601
1664	2319	3601	2321
1665	623	3596	3594

1666	949	3601	3596
1667	625	3594	3601
1668	3596	3601	3594
1669	176	2010	2008
1670	942	4076	2010
1671	940	2008	4076
1672	2010	4076	2008
1673	228	2321	2320
1674	949	4080	2321
1675	942	2320	4080
1676	2321	4080	2320
1677	178	2020	2022
1678	940	4077	2020
1679	949	2022	4077
1680	2020	4077	2022
1681	942	4080	4076
1682	949	4077	4080
1683	940	4076	4077
1684	4080	4077	4076
1685	41	1273	1272
1686	473	3153	1273
1687	472	1272	3153
1688	1273	3153	1272
1689	224	2298	2294
1690	1024	3157	2298
1691	473	2294	3157
1692	2298	3157	2294
1693	223	2288	2292
1694	472	3155	2288
1695	1024	2292	3155
1696	2288	3155	2292
1697	473	3157	3153
1698	1024	3155	3157
1699	472	3153	3155
1700	3157	3155	3153
1701	68	1425	1424
1702	625	3598	1425
1703	624	1424	3598
1704	1425	3598	1424
1705	228	2323	2319
1706	1026	3602	2323
1707	625	2319	3602
1708	2323	3602	2319
1709	224	2295	2299
1710	624	3600	2295
1711	1026	2299	3600
1712	2295	3600	2299
1713	625	3602	3598
1714	1026	3600	3602
1715	624	3598	3600
1716	3602	3600	3598
1717	43	1282	1284
1718	482	3184	1282
1719	484	1284	3184
1720	1282	3184	1284
1721	223	2293	2289
1722	1025	3186	2293
1723	482	2289	3186
1724	2293	3186	2289

1725	228	2318	2322
1726	484	3191	2318
1727	1025	2322	3191
1728	2318	3191	2322
1729	482	3186	3184
1730	1025	3191	3186
1731	484	3184	3191
1732	3186	3191	3184
1733	224	2299	2298
1734	1026	4161	2299
1735	1024	2298	4161
1736	2299	4161	2298
1737	228	2322	2323
1738	1025	4162	2322
1739	1026	2323	4162
1740	2322	4162	2323
1741	223	2292	2293
1742	1024	4160	2292
1743	1025	2293	4160
1744	2292	4160	2293
1745	1026	4162	4161
1746	1025	4160	4162
1747	1024	4161	4160
1748	4162	4160	4161
1749	16	1151	1150
1750	351	2829	1151
1751	350	1150	2829
1752	1151	2829	1150
1753	143	1819	1816
1754	845	2833	1819
1755	351	1816	2833
1756	1819	2833	1816
1757	142	1810	1813
1758	350	2831	1810
1759	845	1813	2831
1760	1810	2831	1813
1761	351	2833	2829
1762	845	2831	2833
1763	350	2829	2831
1764	2833	2831	2829
1765	69	1432	1428
1766	632	3609	1432
1767	628	1428	3609
1768	1432	3609	1428
1769	276	2609	2607
1770	849	3622	2609
1771	632	2607	3622
1772	2609	3622	2607
1773	143	1817	1821
1774	628	3611	1817
1775	849	1821	3611
1776	1817	3611	1821
1777	632	3622	3609
1778	849	3611	3622
1779	628	3609	3611
1780	3622	3611	3609
1781	67	1417	1421
1782	617	3577	1417
1783	621	1421	3577

1784	1417	3577	1421
1785	142	1815	1811
1786	847	3579	1815
1787	617	1811	3579
1788	1815	3579	1811
1789	276	2606	2608
1790	621	3588	2606
1791	847	2608	3588
1792	2606	3588	2608
1793	617	3579	3577
1794	847	3588	3579
1795	621	3577	3588
1796	3579	3588	3577
1797	143	1821	1819
1798	849	3984	1821
1799	845	1819	3984
1800	1821	3984	1819
1801	276	2608	2609
1802	847	3985	2608
1803	849	2609	3985
1804	2608	3985	2609
1805	142	1813	1815
1806	845	3983	1813
1807	847	1815	3983
1808	1813	3983	1815
1809	849	3985	3984
1810	847	3983	3985
1811	845	3984	3983
1812	3985	3983	3984
1813	23	1186	1187
1814	386	2932	1186
1815	387	1187	2932
1816	1186	2932	1187
1817	178	2021	2018
1818	948	2934	2021
1819	386	2018	2934
1820	2021	2934	2018
1821	179	2024	2027
1822	387	2936	2024
1823	948	2027	2936
1824	2024	2936	2027
1825	386	2934	2932
1826	948	2936	2934
1827	387	2932	2936
1828	2934	2936	2932
1829	68	1427	1423
1830	627	3595	1427
1831	623	1423	3595
1832	1427	3595	1423
1833	277	2614	2612
1834	950	3606	2614
1835	627	2612	3606
1836	2614	3606	2612
1837	178	2019	2023
1838	623	3597	2019
1839	950	2023	3597
1840	2019	3597	2023
1841	627	3606	3595
1842	950	3597	3606

1843	623	3595	3597
1844	3606	3597	3595
1845	69	1429	1433
1846	629	3613	1429
1847	633	1433	3613
1848	1429	3613	1433
1849	179	2029	2025
1850	952	3615	2029
1851	629	2025	3615
1852	2029	3615	2025
1853	277	2613	2615
1854	633	3624	2613
1855	952	2615	3624
1856	2613	3624	2615
1857	629	3615	3613
1858	952	3624	3615
1859	633	3613	3624
1860	3615	3624	3613
1861	178	2023	2021
1862	950	4088	2023
1863	948	2021	4088
1864	2023	4088	2021
1865	277	2615	2614
1866	952	4090	2615
1867	950	2614	4090
1868	2615	4090	2614
1869	179	2027	2029
1870	948	4089	2027
1871	952	2029	4089
1872	2027	4089	2029
1873	950	4090	4088
1874	952	4089	4090
1875	948	4088	4089
1876	4090	4089	4088
1877	15	1146	1147
1878	346	2820	1146
1879	347	1147	2820
1880	1146	2820	1147
1881	138	1793	1790
1882	836	2822	1793
1883	346	1790	2822
1884	1793	2822	1790
1885	139	1796	1799
1886	347	2824	1796
1887	836	1799	2824
1888	1796	2824	1799
1889	346	2822	2820
1890	836	2824	2822
1891	347	2820	2824
1892	2822	2824	2820
1893	67	1420	1416
1894	620	3573	1420
1895	616	1416	3573
1896	1420	3573	1416
1897	275	2602	2600
1898	838	3586	2602
1899	620	2600	3586
1900	2602	3586	2600
1901	138	1791	1795

1902	616	3575	1791
1903	838	1795	3575
1904	1791	3575	1795
1905	620	3586	3573
1906	838	3575	3586
1907	616	3573	3575
1908	3586	3575	3573
1909	68	1422	1426
1910	622	3591	1422
1911	626	1426	3591
1912	1422	3591	1426
1913	139	1801	1797
1914	840	3593	1801
1915	622	1797	3593
1916	1801	3593	1797
1917	275	2601	2603
1918	626	3604	2601
1919	840	2603	3604
1920	2601	3604	2603
1921	622	3593	3591
1922	840	3604	3593
1923	626	3591	3604
1924	3593	3604	3591
1925	138	1795	1793
1926	838	3974	1795
1927	836	1793	3974
1928	1795	3974	1793
1929	275	2603	2602
1930	840	3976	2603
1931	838	2602	3976
1932	2603	3976	2602
1933	139	1799	1801
1934	836	3975	1799
1935	840	1801	3975
1936	1799	3975	1801
1937	838	3976	3974
1938	840	3975	3976
1939	836	3974	3975
1940	3976	3975	3974
1941	69	1433	1432
1942	633	3621	1433
1943	632	1432	3621
1944	1433	3621	1432
1945	277	2617	2613
1946	1077	3625	2617
1947	633	2613	3625
1948	2617	3625	2613
1949	276	2607	2611
1950	632	3623	2607
1951	1077	2611	3623
1952	2607	3623	2611
1953	633	3625	3621
1954	1077	3623	3625
1955	632	3621	3623
1956	3625	3623	3621
1957	68	1426	1427
1958	626	3603	1426
1959	627	1427	3603
1960	1426	3603	1427

1961	275	2605	2601
1962	1076	3605	2605
1963	626	2601	3605
1964	2605	3605	2601
1965	277	2612	2616
1966	627	3607	2612
1967	1076	2616	3607
1968	2612	3607	2616
1969	626	3605	3603
1970	1076	3607	3605
1971	627	3603	3607
1972	3605	3607	3603
1973	67	1421	1420
1974	621	3585	1421
1975	620	1420	3585
1976	1421	3585	1420
1977	276	2610	2606
1978	1075	3589	2610
1979	621	2606	3589
1980	2610	3589	2606
1981	275	2600	2604
1982	620	3587	2600
1983	1075	2604	3587
1984	2600	3587	2604
1985	621	3589	3585
1986	1075	3587	3589
1987	620	3585	3587
1988	3589	3587	3585
1989	277	2616	2617
1990	1076	4213	2616
1991	1077	2617	4213
1992	2616	4213	2617
1993	275	2604	2605
1994	1075	4211	2604
1995	1076	2605	4211
1996	2604	4211	2605
1997	276	2611	2610
1998	1077	4212	2611
1999	1075	2610	4212
2000	2611	4212	2610
2001	1076	4211	4213
2002	1075	4212	4211
2003	1077	4213	4212
2004	4211	4212	4213
2005	4	1097	1099
2006	297	2703	1097
2007	299	1099	2703
2008	1097	2703	1099
2009	89	1539	1536
2010	719	2705	1539
2011	297	1536	2705
2012	1539	2705	1536
2013	91	1546	1548
2014	299	2707	1546
2015	719	1548	2707
2016	1546	2707	1548
2017	297	2705	2703
2018	719	2707	2705
2019	299	2703	2707

2020	2705	2707	2703
2021	33	1235	1232
2022	435	3051	1235
2023	432	1232	3051
2024	1235	3051	1232
2025	207	2194	2192
2026	721	3062	2194
2027	435	2192	3062
2028	2194	3062	2192
2029	89	1537	1541
2030	432	3053	1537
2031	721	1541	3053
2032	1537	3053	1541
2033	435	3062	3051
2034	721	3053	3062
2035	432	3051	3053
2036	3062	3053	3051
2037	35	1242	1244
2038	442	3077	1242
2039	444	1244	3077
2040	1242	3077	1244
2041	91	1549	1547
2042	723	3078	1549
2043	442	1547	3078
2044	1549	3078	1547
2045	207	2193	2195
2046	444	3082	2193
2047	723	2195	3082
2048	2193	3082	2195
2049	442	3078	3077
2050	723	3082	3078
2051	444	3077	3082
2052	3078	3082	3077
2053	89	1541	1539
2054	721	3856	1541
2055	719	1539	3856
2056	1541	3856	1539
2057	207	2195	2194
2058	723	3859	2195
2059	721	2194	3859
2060	2195	3859	2194
2061	91	1548	1549
2062	719	3857	1548
2063	723	1549	3857
2064	1548	3857	1549
2065	721	3859	3856
2066	723	3857	3859
2067	719	3856	3857
2068	3859	3857	3856
2069	17	1155	1152
2070	355	2835	1155
2071	352	1152	2835
2072	1155	2835	1152
2073	147	1842	1840
2074	851	2846	1842
2075	355	1840	2846
2076	1842	2846	1840
2077	144	1822	1825
2078	352	2837	1822

2079	851	1825	2837
2080	1822	2837	1825
2081	355	2846	2835
2082	851	2837	2846
2083	352	2835	2837
2084	2846	2837	2835
2085	71	1442	1440
2086	642	3644	1442
2087	640	1440	3644
2088	1442	3644	1440
2089	209	2207	2205
2090	862	3653	2207
2091	642	2205	3653
2092	2207	3653	2205
2093	147	1841	1844
2094	640	3646	1841
2095	862	1844	3646
2096	1841	3646	1844
2097	642	3653	3644
2098	862	3646	3653
2099	640	3644	3646
2100	3653	3646	3644
2101	33	1233	1237
2102	433	3055	1233
2103	437	1237	3055
2104	1233	3055	1237
2105	144	1827	1823
2106	853	3057	1827
2107	433	1823	3057
2108	1827	3057	1823
2109	209	2204	2206
2110	437	3066	2204
2111	853	2206	3066
2112	2204	3066	2206
2113	433	3057	3055
2114	853	3066	3057
2115	437	3055	3066
2116	3057	3066	3055
2117	147	1844	1842
2118	862	3989	1844
2119	851	1842	3989
2120	1844	3989	1842
2121	209	2206	2207
2122	853	3991	2206
2123	862	2207	3991
2124	2206	3991	2207
2125	144	1825	1827
2126	851	3988	1825
2127	853	1827	3988
2128	1825	3988	1827
2129	862	3991	3989
2130	853	3988	3991
2131	851	3989	3988
2132	3991	3988	3989
2133	19	1162	1164
2134	362	2861	1162
2135	364	1164	2861
2136	1162	2861	1164
2137	154	1880	1878

2138	877	2862	1880
2139	362	1878	2862
2140	1880	2862	1878
2141	156	1886	1888
2142	364	2866	1886
2143	877	1888	2866
2144	1886	2866	1888
2145	362	2862	2861
2146	877	2866	2862
2147	364	2861	2866
2148	2862	2866	2861
2149	35	1245	1243
2150	445	3079	1245
2151	443	1243	3079
2152	1245	3079	1243
2153	211	2218	2216
2154	878	3084	2218
2155	445	2216	3084
2156	2218	3084	2216
2157	154	1879	1881
2158	443	3080	1879
2159	878	1881	3080
2160	1879	3080	1881
2161	445	3084	3079
2162	878	3080	3084
2163	443	3079	3080
2164	3084	3080	3079
2165	154	1881	1880
2166	878	4013	1881
2167	877	1880	4013
2168	1881	4013	1880
2169	211	2219	2218
2170	882	4015	2219
2171	878	2218	4015
2172	2219	4015	2218
2173	156	1888	1890
2174	877	4014	1888
2175	882	1890	4014
2176	1888	4014	1890
2177	878	4015	4013
2178	882	4014	4015
2179	877	4013	4014
2180	4015	4014	4013
2181	33	1237	1235
2182	437	3061	1237
2183	435	1235	3061
2184	1237	3061	1235
2185	209	2208	2204
2186	1008	3067	2208
2187	437	2204	3067
2188	2208	3067	2204
2189	207	2192	2196
2190	435	3063	2192
2191	1008	2196	3063
2192	2192	3063	2196
2193	437	3067	3061
2194	1008	3063	3067
2195	435	3061	3063
2196	3067	3063	3061

2197	35	1244	1245
2198	444	3081	1244
2199	445	1245	3081
2200	1244	3081	1245
2201	207	2197	2193
2202	1009	3083	2197
2203	444	2193	3083
2204	2197	3083	2193
2205	211	2216	2220
2206	445	3085	2216
2207	1009	2220	3085
2208	2216	3085	2220
2209	444	3083	3081
2210	1009	3085	3083
2211	445	3081	3085
2212	3083	3085	3081
2213	209	2209	2208
2214	1011	4146	2209
2215	1008	2208	4146
2216	2209	4146	2208
2217	211	2220	2221
2218	1009	4147	2220
2219	1011	2221	4147
2220	2220	4147	2221
2221	207	2196	2197
2222	1008	4145	2196
2223	1009	2197	4145
2224	2196	4145	2197
2225	1011	4147	4146
2226	1009	4145	4147
2227	1008	4146	4145
2228	4147	4145	4146
2229	7	1113	1109
2230	313	2731	1113
2231	309	1109	2731
2232	1113	2731	1109
2233	105	1622	1620
2234	747	2742	1622
2235	313	1620	2742
2236	1622	2742	1620
2237	101	1596	1599
2238	309	2733	1596
2239	747	1599	2733
2240	1596	2733	1599
2241	313	2742	2731
2242	747	2733	2742
2243	309	2731	2733
2244	2742	2733	2731
2245	49	1318	1316
2246	518	3284	1318
2247	516	1316	3284
2248	1318	3284	1316
2249	235	2363	2361
2250	758	3293	2363
2251	518	2361	3293
2252	2363	3293	2361
2253	105	1621	1624
2254	516	3286	1621
2255	758	1624	3286

2256	1621	3286	1624
2257	518	3293	3284
2258	758	3286	3293
2259	516	3284	3286
2260	3293	3286	3284
2261	45	1292	1295
2262	492	3213	1292
2263	495	1295	3213
2264	1292	3213	1295
2265	101	1601	1597
2266	749	3215	1601
2267	492	1597	3215
2268	1601	3215	1597
2269	235	2360	2362
2270	495	3224	2360
2271	749	2362	3224
2272	2360	3224	2362
2273	492	3215	3213
2274	749	3224	3215
2275	495	3213	3224
2276	3215	3224	3213
2277	105	1624	1622
2278	758	3887	1624
2279	747	1622	3887
2280	1624	3887	1622
2281	235	2362	2363
2282	749	3889	2362
2283	758	2363	3889
2284	2362	3889	2363
2285	101	1599	1601
2286	747	3886	1599
2287	749	1601	3886
2288	1599	3886	1601
2289	758	3889	3887
2290	749	3886	3889
2291	747	3887	3886
2292	3889	3886	3887
2293	25	1198	1196
2294	398	2960	1198
2295	396	1196	2960
2296	1198	2960	1196
2297	190	2092	2090
2298	976	2969	2092
2299	398	2090	2969
2300	2092	2969	2090
2301	188	2078	2080
2302	396	2962	2078
2303	976	2080	2962
2304	2078	2962	2080
2305	398	2969	2960
2306	976	2962	2969
2307	396	2960	2962
2308	2969	2962	2960
2309	73	1455	1453
2310	655	3684	1455
2311	653	1453	3684
2312	1455	3684	1453
2313	246	2429	2427
2314	985	3691	2429

2315	655	2427	3691
2316	2429	3691	2427
2317	190	2091	2094
2318	653	3686	2091
2319	985	2094	3686
2320	2091	3686	2094
2321	655	3691	3684
2322	985	3686	3691
2323	653	3684	3686
2324	3691	3686	3684
2325	49	1317	1320
2326	517	3288	1317
2327	520	1320	3288
2328	1317	3288	1320
2329	188	2082	2079
2330	978	3290	2082
2331	517	2079	3290
2332	2082	3290	2079
2333	246	2426	2428
2334	520	3298	2426
2335	978	2428	3298
2336	2426	3298	2428
2337	517	3290	3288
2338	978	3298	3290
2339	520	3288	3298
2340	3290	3298	3288
2341	190	2094	2092
2342	985	4113	2094
2343	976	2092	4113
2344	2094	4113	2092
2345	246	2428	2429
2346	978	4116	2428
2347	985	2429	4116
2348	2428	4116	2429
2349	188	2080	2082
2350	976	4112	2080
2351	978	2082	4112
2352	2080	4112	2082
2353	985	4116	4113
2354	978	4112	4116
2355	976	4113	4112
2356	4116	4112	4113
2357	17	1153	1157
2358	353	2839	1153
2359	357	1157	2839
2360	1153	2839	1157
2361	145	1831	1828
2362	855	2841	1831
2363	353	1828	2841
2364	1831	2841	1828
2365	149	1852	1854
2366	357	2850	1852
2367	855	1854	2850
2368	1852	2850	1854
2369	353	2841	2839
2370	855	2850	2841
2371	357	2839	2850
2372	2841	2850	2839
2373	45	1297	1293

2374	497	3217	1297
2375	493	1293	3217
2376	1297	3217	1293
2377	237	2374	2372
2378	857	3228	2374
2379	497	2372	3228
2380	2374	3228	2372
2381	145	1829	1833
2382	493	3219	1829
2383	857	1833	3219
2384	1829	3219	1833
2385	497	3228	3217
2386	857	3219	3228
2387	493	3217	3219
2388	3228	3219	3217
2389	73	1452	1454
2390	652	3680	1452
2391	654	1454	3680
2392	1452	3680	1454
2393	149	1856	1853
2394	866	3682	1856
2395	652	1853	3682
2396	1856	3682	1853
2397	237	2373	2375
2398	654	3689	2373
2399	866	2375	3689
2400	2373	3689	2375
2401	652	3682	3680
2402	866	3689	3682
2403	654	3680	3689
2404	3682	3689	3680
2405	145	1833	1831
2406	857	3994	1833
2407	855	1831	3994
2408	1833	3994	1831
2409	237	2375	2374
2410	866	3997	2375
2411	857	2374	3997
2412	2375	3997	2374
2413	149	1854	1856
2414	855	3995	1854
2415	866	1856	3995
2416	1854	3995	1856
2417	857	3997	3994
2418	866	3995	3997
2419	855	3994	3995
2420	3997	3995	3994
2421	49	1320	1318
2422	520	3292	1320
2423	518	1318	3292
2424	1320	3292	1318
2425	246	2430	2426
2426	1039	3299	2430
2427	520	2426	3299
2428	2430	3299	2426
2429	235	2361	2365
2430	518	3294	2361
2431	1039	2365	3294
2432	2361	3294	2365

2433	520	3299	3292
2434	1039	3294	3299
2435	518	3292	3294
2436	3299	3294	3292
2437	73	1454	1455
2438	654	3688	1454
2439	655	1455	3688
2440	1454	3688	1455
2441	237	2377	2373
2442	1041	3690	2377
2443	654	2373	3690
2444	2377	3690	2373
2445	246	2427	2431
2446	655	3692	2427
2447	1041	2431	3692
2448	2427	3692	2431
2449	654	3690	3688
2450	1041	3692	3690
2451	655	3688	3692
2452	3690	3692	3688
2453	45	1295	1297
2454	495	3223	1295
2455	497	1297	3223
2456	1295	3223	1297
2457	235	2364	2360
2458	1038	3225	2364
2459	495	2360	3225
2460	2364	3225	2360
2461	237	2372	2376
2462	497	3229	2372
2463	1038	2376	3229
2464	2372	3229	2376
2465	495	3225	3223
2466	1038	3229	3225
2467	497	3223	3229
2468	3225	3229	3223
2469	246	2431	2430
2470	1041	4177	2431
2471	1039	2430	4177
2472	2431	4177	2430
2473	237	2376	2377
2474	1038	4176	2376
2475	1041	2377	4176
2476	2376	4176	2377
2477	235	2365	2364
2478	1039	4175	2365
2479	1038	2364	4175
2480	2365	4175	2364
2481	1041	4176	4177
2482	1038	4175	4176
2483	1039	4177	4175
2484	4176	4175	4177
2485	9	1119	1121
2486	319	2755	1119
2487	321	1121	2755
2488	1119	2755	1121
2489	111	1652	1650
2490	771	2756	1652
2491	319	1650	2756

2492	1652	2756	1650
2493	113	1660	1662
2494	321	2760	1660
2495	771	1662	2760
2496	1660	2760	1662
2497	319	2756	2755
2498	771	2760	2756
2499	321	2755	2760
2500	2756	2760	2755
2501	55	1348	1346
2502	548	3365	1348
2503	546	1346	3365
2504	1348	3365	1346
2505	259	2506	2504
2506	772	3370	2506
2507	548	2504	3370
2508	2506	3370	2504
2509	111	1651	1653
2510	546	3366	1651
2511	772	1653	3366
2512	1651	3366	1653
2513	548	3370	3365
2514	772	3366	3370
2515	546	3365	3366
2516	3370	3366	3365
2517	57	1356	1358
2518	556	3392	1356
2519	558	1358	3392
2520	1356	3392	1358
2521	113	1664	1661
2522	776	3394	1664
2523	556	1661	3394
2524	1664	3394	1661
2525	259	2505	2507
2526	558	3401	2505
2527	776	2507	3401
2528	2505	3401	2507
2529	556	3394	3392
2530	776	3401	3394
2531	558	3392	3401
2532	3394	3401	3392
2533	111	1653	1652
2534	772	3908	1653
2535	771	1652	3908
2536	1653	3908	1652
2537	259	2507	2506
2538	776	3910	2507
2539	772	2506	3910
2540	2507	3910	2506
2541	113	1662	1664
2542	771	3909	1662
2543	776	1664	3909
2544	1662	3909	1664
2545	772	3910	3908
2546	776	3909	3910
2547	771	3908	3909
2548	3910	3909	3908
2549	19	1165	1163
2550	365	2863	1165

2551	363	1163	2863
2552	1165	2863	1163
2553	157	1894	1892
2554	879	2868	1894
2555	365	1892	2868
2556	1894	2868	1892
2557	155	1882	1884
2558	363	2864	1882
2559	879	1884	2864
2560	1882	2864	1884
2561	365	2868	2863
2562	879	2864	2868
2563	363	2863	2864
2564	2868	2864	2863
2565	75	1466	1464
2566	666	3716	1466
2567	664	1464	3716
2568	1466	3716	1464
2569	260	2513	2511
2570	884	3725	2513
2571	666	2511	3725
2572	2513	3725	2511
2573	157	1893	1896
2574	664	3718	1893
2575	884	1896	3718
2576	1893	3718	1896
2577	666	3725	3716
2578	884	3718	3725
2579	664	3716	3718
2580	3725	3718	3716
2581	55	1347	1349
2582	547	3367	1347
2583	549	1349	3367
2584	1347	3367	1349
2585	155	1885	1883
2586	880	3368	1885
2587	547	1883	3368
2588	1885	3368	1883
2589	260	2510	2512
2590	549	3372	2510
2591	880	2512	3372
2592	2510	3372	2512
2593	547	3368	3367
2594	880	3372	3368
2595	549	3367	3372
2596	3368	3372	3367
2597	157	1896	1894
2598	884	4017	1896
2599	879	1894	4017
2600	1896	4017	1894
2601	260	2512	2513
2602	880	4018	2512
2603	884	2513	4018
2604	2512	4018	2513
2605	155	1884	1885
2606	879	4016	1884
2607	880	1885	4016
2608	1884	4016	1885
2609	884	4018	4017

2610	880	4016	4018
2611	879	4017	4016
2612	4018	4016	4017
2613	25	1197	1199
2614	397	2964	1197
2615	399	1199	2964
2616	1197	2964	1199
2617	189	2086	2084
2618	980	2966	2086
2619	397	2084	2966
2620	2086	2966	2084
2621	191	2096	2098
2622	399	2971	2096
2623	980	2098	2971
2624	2096	2971	2098
2625	397	2966	2964
2626	980	2971	2966
2627	399	2964	2971
2628	2966	2971	2964
2629	57	1360	1357
2630	560	3396	1360
2631	557	1357	3396
2632	1360	3396	1357
2633	264	2536	2534
2634	982	3406	2536
2635	560	2534	3406
2636	2536	3406	2534
2637	189	2085	2088
2638	557	3398	2085
2639	982	2088	3398
2640	2085	3398	2088
2641	560	3406	3396
2642	982	3398	3406
2643	557	3396	3398
2644	3406	3398	3396
2645	75	1465	1467
2646	665	3720	1465
2647	667	1467	3720
2648	1465	3720	1467
2649	191	2100	2097
2650	987	3722	2100
2651	665	2097	3722
2652	2100	3722	2097
2653	264	2535	2537
2654	667	3727	2535
2655	987	2537	3727
2656	2535	3727	2537
2657	665	3722	3720
2658	987	3727	3722
2659	667	3720	3727
2660	3722	3727	3720
2661	189	2088	2086
2662	982	4118	2088
2663	980	2086	4118
2664	2088	4118	2086
2665	264	2537	2536
2666	987	4122	2537
2667	982	2536	4122
2668	2537	4122	2536

2669	191	2098	2100
2670	980	4119	2098
2671	987	2100	4119
2672	2098	4119	2100
2673	982	4122	4118
2674	987	4119	4122
2675	980	4118	4119
2676	4122	4119	4118
2677	55	1349	1348
2678	549	3369	1349
2679	548	1348	3369
2680	1349	3369	1348
2681	260	2514	2510
2682	1060	3373	2514
2683	549	2510	3373
2684	2514	3373	2510
2685	259	2504	2508
2686	548	3371	2504
2687	1060	2508	3371
2688	2504	3371	2508
2689	549	3373	3369
2690	1060	3371	3373
2691	548	3369	3371
2692	3373	3371	3369
2693	75	1467	1466
2694	667	3724	1467
2695	666	1466	3724
2696	1467	3724	1466
2697	264	2539	2535
2698	1062	3728	2539
2699	667	2535	3728
2700	2539	3728	2535
2701	260	2511	2515
2702	666	3726	2511
2703	1062	2515	3726
2704	2511	3726	2515
2705	667	3728	3724
2706	1062	3726	3728
2707	666	3724	3726
2708	3728	3726	3724
2709	57	1358	1360
2710	558	3400	1358
2711	560	1360	3400
2712	1358	3400	1360
2713	259	2509	2505
2714	1061	3402	2509
2715	558	2505	3402
2716	2509	3402	2505
2717	264	2534	2538
2718	560	3407	2534
2719	1061	2538	3407
2720	2534	3407	2538
2721	558	3402	3400
2722	1061	3407	3402
2723	560	3400	3407
2724	3402	3407	3400
2725	260	2515	2514
2726	1062	4197	2515
2727	1060	2514	4197

2728	2515	4197	2514
2729	264	2538	2539
2730	1061	4198	2538
2731	1062	2539	4198
2732	2538	4198	2539
2733	259	2508	2509
2734	1060	4196	2508
2735	1061	2509	4196
2736	2508	4196	2509
2737	1062	4198	4197
2738	1061	4196	4198
2739	1060	4197	4196
2740	4198	4196	4197
2741	17	1157	1155
2742	357	2845	1157
2743	355	1155	2845
2744	1157	2845	1155
2745	149	1855	1852
2746	861	2851	1855
2747	357	1852	2851
2748	1855	2851	1852
2749	147	1840	1843
2750	355	2847	1840
2751	861	1843	2847
2752	1840	2847	1843
2753	357	2851	2845
2754	861	2847	2851
2755	355	2845	2847
2756	2851	2847	2845
2757	73	1456	1452
2758	656	3681	1456
2759	652	1452	3681
2760	1456	3681	1452
2761	280	2633	2631
2762	867	3694	2633
2763	656	2631	3694
2764	2633	3694	2631
2765	149	1853	1857
2766	652	3683	1853
2767	867	1857	3683
2768	1853	3683	1857
2769	656	3694	3681
2770	867	3683	3694
2771	652	3681	3683
2772	3694	3683	3681
2773	71	1440	1444
2774	640	3645	1440
2775	644	1444	3645
2776	1440	3645	1444
2777	147	1845	1841
2778	863	3647	1845
2779	640	1841	3647
2780	1845	3647	1841
2781	280	2630	2632
2782	644	3658	2630
2783	863	2632	3658
2784	2630	3658	2632
2785	640	3647	3645
2786	863	3658	3647

2787	644	3645	3658
2788	3647	3658	3645
2789	149	1857	1855
2790	867	4002	1857
2791	861	1855	4002
2792	1857	4002	1855
2793	280	2632	2633
2794	863	4003	2632
2795	867	2633	4003
2796	2632	4003	2633
2797	147	1843	1845
2798	861	4001	1843
2799	863	1845	4001
2800	1843	4001	1845
2801	867	4003	4002
2802	863	4001	4003
2803	861	4002	4001
2804	4003	4001	4002
2805	25	1199	1198
2806	399	2968	1199
2807	398	1198	2968
2808	1199	2968	1198
2809	191	2099	2096
2810	984	2972	2099
2811	399	2096	2972
2812	2099	2972	2096
2813	190	2090	2093
2814	398	2970	2090
2815	984	2093	2970
2816	2090	2970	2093
2817	399	2972	2968
2818	984	2970	2972
2819	398	2968	2970
2820	2972	2970	2968
2821	75	1469	1465
2822	669	3721	1469
2823	665	1465	3721
2824	1469	3721	1465
2825	283	2651	2649
2826	988	3732	2651
2827	669	2649	3732
2828	2651	3732	2649
2829	191	2097	2101
2830	665	3723	2097
2831	988	2101	3723
2832	2097	3723	2101
2833	669	3732	3721
2834	988	3723	3732
2835	665	3721	3723
2836	3732	3723	3721
2837	190	2095	2091
2838	986	3687	2095
2839	653	2091	3687
2840	2095	3687	2091
2841	283	2648	2650
2842	657	3696	2648
2843	986	2650	3696
2844	2648	3696	2650
2845	191	2101	2099

2846	988	4125	2101
2847	984	2099	4125
2848	2101	4125	2099
2849	283	2650	2651
2850	986	4126	2650
2851	988	2651	4126
2852	2650	4126	2651
2853	190	2093	2095
2854	984	4124	2093
2855	986	2095	4124
2856	2093	4124	2095
2857	988	4126	4125
2858	986	4124	4126
2859	984	4125	4124
2860	4126	4124	4125
2861	19	1164	1165
2862	364	2865	1164
2863	365	1165	2865
2864	1164	2865	1165
2865	156	1889	1886
2866	881	2867	1889
2867	364	1886	2867
2868	1889	2867	1886
2869	157	1892	1895
2870	365	2869	1892
2871	881	1895	2869
2872	1892	2869	1895
2873	364	2867	2865
2874	881	2869	2867
2875	365	2865	2869
2876	2867	2869	2865
2877	71	1445	1441
2878	645	3649	1445
2879	641	1441	3649
2880	1445	3649	1441
2881	281	2638	2636
2882	883	3660	2638
2883	645	2636	3660
2884	2638	3660	2636
2885	156	1887	1891
2886	641	3651	1887
2887	883	1891	3651
2888	1887	3651	1891
2889	645	3660	3649
2890	883	3651	3660
2891	641	3649	3651
2892	3660	3651	3649
2893	75	1464	1468
2894	664	3717	1464
2895	668	1468	3717
2896	1464	3717	1468
2897	157	1897	1893
2898	885	3719	1897
2899	664	1893	3719
2900	1897	3719	1893
2901	281	2637	2639
2902	668	3730	2637
2903	885	2639	3730
2904	2637	3730	2639

2905	664	3719	3717
2906	885	3730	3719
2907	668	3717	3730
2908	3719	3730	3717
2909	156	1891	1889
2910	883	4019	1891
2911	881	1889	4019
2912	1891	4019	1889
2913	281	2639	2638
2914	885	4021	2639
2915	883	2638	4021
2916	2639	4021	2638
2917	157	1895	1897
2918	881	4020	1895
2919	885	1897	4020
2920	1895	4020	1897
2921	883	4021	4019
2922	885	4020	4021
2923	881	4019	4020
2924	4021	4020	4019
2925	73	1457	1456
2926	657	3693	1457
2927	656	1456	3693
2928	1457	3693	1456
2929	283	2652	2648
2930	1082	3697	2652
2931	657	2648	3697
2932	2652	3697	2648
2933	280	2631	2635
2934	656	3695	2631
2935	1082	2635	3695
2936	2631	3695	2635
2937	657	3697	3693
2938	1082	3695	3697
2939	656	3693	3695
2940	3697	3695	3693
2941	75	1468	1469
2942	668	3729	1468
2943	669	1469	3729
2944	1468	3729	1469
2945	281	2641	2637
2946	1083	3731	2641
2947	668	2637	3731
2948	2641	3731	2637
2949	283	2649	2653
2950	669	3733	2649
2951	1083	2653	3733
2952	2649	3733	2653
2953	668	3731	3729
2954	1083	3733	3731
2955	669	3729	3733
2956	3731	3733	3729
2957	71	1444	1445
2958	644	3657	1444
2959	645	1445	3657
2960	1444	3657	1445
2961	280	2634	2630
2962	1081	3659	2634
2963	644	2630	3659

2964	2634	3659	2630
2965	281	2636	2640
2966	645	3661	2636
2967	1081	2640	3661
2968	2636	3661	2640
2969	644	3659	3657
2970	1081	3661	3659
2971	645	3657	3661
2972	3659	3661	3657
2973	283	2653	2652
2974	1083	4219	2653
2975	1082	2652	4219
2976	2653	4219	2652
2977	281	2640	2641
2978	1081	4218	2640
2979	1083	2641	4218
2980	2640	4218	2641
2981	280	2635	2634
2982	1082	4217	2635
2983	1081	2634	4217
2984	2635	4217	2634
2985	1083	4218	4219
2986	1081	4217	4218
2987	1082	4219	4217
2988	4218	4217	4219
2989	6	1107	1106
2990	307	2721	1107
2991	306	1106	2721
2992	1107	2721	1106
2993	99	1587	1584
2994	737	2725	1587
2995	307	1584	2725
2996	1587	2725	1584
2997	98	1578	1581
2998	306	2723	1578
2999	737	1581	2723
3000	1578	2723	1581
3001	307	2725	2721
3002	737	2723	2725
3003	306	2721	2723
3004	2725	2723	2721
3005	43	1283	1280
3006	483	3177	1283
3007	480	1280	3177
3008	1283	3177	1280
3009	225	2303	2301
3010	741	3188	2303
3011	483	2301	3188
3012	2303	3188	2301
3013	99	1585	1589
3014	480	3179	1585
3015	741	1589	3179
3016	1585	3179	1589
3017	483	3188	3177
3018	741	3179	3188
3019	480	3177	3179
3020	3188	3179	3177
3021	42	1274	1277
3022	474	3159	1274

3023	477	1277	3159
3024	1274	3159	1277
3025	98	1583	1579
3026	739	3161	1583
3027	474	1579	3161
3028	1583	3161	1579
3029	225	2300	2302
3030	477	3170	2300
3031	739	2302	3170
3032	2300	3170	2302
3033	474	3161	3159
3034	739	3170	3161
3035	477	3159	3170
3036	3161	3170	3159
3037	99	1589	1587
3038	741	3876	1589
3039	737	1587	3876
3040	1589	3876	1587
3041	225	2302	2303
3042	739	3877	2302
3043	741	2303	3877
3044	2302	3877	2303
3045	98	1581	1583
3046	737	3875	1581
3047	739	1583	3875
3048	1581	3875	1583
3049	741	3877	3876
3050	739	3875	3877
3051	737	3876	3875
3052	3877	3875	3876
3053	23	1188	1184
3054	388	2925	1188
3055	384	1184	2925
3056	1188	2925	1184
3057	176	2006	2009
3058	384	2927	2006
3059	941	2009	2927
3060	2006	2927	2009
3061	79	1491	1489
3062	691	3792	1491
3063	689	1489	3792
3064	1491	3792	1489
3065	229	2327	2325
3066	954	3799	2327
3067	691	2325	3799
3068	2327	3799	2325
3069	180	2031	2034
3070	689	3794	2031
3071	954	2034	3794
3072	2031	3794	2034
3073	691	3799	3792
3074	954	3794	3799
3075	689	3792	3794
3076	3799	3794	3792
3077	43	1281	1285
3078	481	3181	1281
3079	485	1285	3181
3080	1281	3181	1285
3081	176	2011	2007

3082	943	3183	2011
3083	481	2007	3183
3084	2011	3183	2007
3085	229	2324	2326
3086	485	3192	2324
3087	943	2326	3192
3088	2324	3192	2326
3089	481	3183	3181
3090	943	3192	3183
3091	485	3181	3192
3092	3183	3192	3181
3093	180	2034	2032
3094	954	4079	2034
3095	941	2032	4079
3096	2034	4079	2032
3097	229	2326	2327
3098	943	4081	2326
3099	954	2327	4081
3100	2326	4081	2327
3101	176	2009	2011
3102	941	4078	2009
3103	943	2011	4078
3104	2009	4078	2011
3105	954	4081	4079
3106	943	4078	4081
3107	941	4079	4078
3108	4081	4078	4079
3109	22	1178	1182
3110	378	2907	1178
3111	382	1182	2907
3112	1178	2907	1182
3113	170	1973	1970
3114	923	2909	1973
3115	378	1970	2909
3116	1973	2909	1970
3117	174	1994	1996
3118	382	2920	1994
3119	923	1996	2920
3120	1994	2920	1996
3121	378	2909	2907
3122	923	2920	2909
3123	382	2907	2920
3124	2909	2920	2907
3125	42	1279	1275
3126	479	3163	1279
3127	475	1275	3163
3128	1279	3163	1275
3129	227	2314	2312
3130	925	3174	2314
3131	479	2312	3174
3132	2314	3174	2312
3133	170	1971	1975
3134	475	3165	1971
3135	925	1975	3165
3136	1971	3165	1975
3137	479	3174	3163
3138	925	3165	3174
3139	475	3163	3165
3140	3174	3165	3163

3141	79	1488	1490
3142	688	3788	1488
3143	690	1490	3788
3144	1488	3788	1490
3145	174	1998	1995
3146	936	3790	1998
3147	688	1995	3790
3148	1998	3790	1995
3149	227	2313	2315
3150	690	3797	2313
3151	936	2315	3797
3152	2313	3797	2315
3153	688	3790	3788
3154	936	3797	3790
3155	690	3788	3797
3156	3790	3797	3788
3157	170	1975	1973
3158	925	4060	1975
3159	923	1973	4060
3160	1975	4060	1973
3161	227	2315	2314
3162	936	4063	2315
3163	925	2314	4063
3164	2315	4063	2314
3165	174	1996	1998
3166	923	4061	1996
3167	936	1998	4061
3168	1996	4061	1998
3169	925	4063	4060
3170	936	4061	4063
3171	923	4060	4061
3172	4063	4061	4060
3173	43	1285	1283
3174	485	3187	1285
3175	483	1283	3187
3176	1285	3187	1283
3177	229	2328	2324
3178	1028	3193	2328
3179	485	2324	3193
3180	2328	3193	2324
3181	225	2301	2305
3182	483	3189	2301
3183	1028	2305	3189
3184	2301	3189	2305
3185	485	3193	3187
3186	1028	3189	3193
3187	483	3187	3189
3188	3193	3189	3187
3189	79	1490	1491
3190	690	3796	1490
3191	691	1491	3796
3192	1490	3796	1491
3193	227	2317	2313
3194	1029	3798	2317
3195	690	2313	3798
3196	2317	3798	2313
3197	229	2325	2329
3198	691	3800	2325
3199	1029	2329	3800

3200	2325	3800	2329
3201	690	3798	3796
3202	1029	3800	3798
3203	691	3796	3800
3204	3798	3800	3796
3205	42	1277	1279
3206	477	3169	1277
3207	479	1279	3169
3208	1277	3169	1279
3209	225	2304	2300
3210	1027	3171	2304
3211	477	2300	3171
3212	2304	3171	2300
3213	227	2312	2316
3214	479	3175	2312
3215	1027	2316	3175
3216	2312	3175	2316
3217	477	3171	3169
3218	1027	3175	3171
3219	479	3169	3175
3220	3171	3175	3169
3221	229	2329	2328
3222	1029	4165	2329
3223	1028	2328	4165
3224	2329	4165	2328
3225	227	2316	2317
3226	1027	4164	2316
3227	1029	2317	4164
3228	2316	4164	2317
3229	225	2305	2304
3230	1028	4163	2305
3231	1027	2304	4163
3232	2305	4163	2304
3233	1029	4164	4165
3234	1027	4163	4164
3235	1028	4165	4163
3236	4164	4163	4165
3237	9	1121	1120
3238	321	2757	1121
3239	320	1120	2757
3240	1121	2757	1120
3241	113	1663	1660
3242	773	2761	1663
3243	321	1660	2761
3244	1663	2761	1660
3245	112	1654	1657
3246	320	2759	1654
3247	773	1657	2759
3248	1654	2759	1657
3249	321	2761	2757
3250	773	2759	2761
3251	320	2757	2759
3252	2761	2759	2757
3253	57	1359	1356
3254	559	3393	1359
3255	556	1356	3393
3256	1359	3393	1356
3257	261	2519	2517
3258	777	3404	2519

3259	559	2517	3404
3260	2519	3404	2517
3261	113	1661	1665
3262	556	3395	1661
3263	777	1665	3395
3264	1661	3395	1665
3265	559	3404	3393
3266	777	3395	3404
3267	556	3393	3395
3268	3404	3395	3393
3269	56	1350	1353
3270	550	3375	1350
3271	553	1353	3375
3272	1350	3375	1353
3273	112	1659	1655
3274	775	3377	1659
3275	550	1655	3377
3276	1659	3377	1655
3277	261	2516	2518
3278	553	3386	2516
3279	775	2518	3386
3280	2516	3386	2518
3281	550	3377	3375
3282	775	3386	3377
3283	553	3375	3386
3284	3377	3386	3375
3285	113	1665	1663
3286	777	3912	1665
3287	773	1663	3912
3288	1665	3912	1663
3289	261	2518	2519
3290	775	3913	2518
3291	777	2519	3913
3292	2518	3913	2519
3293	112	1657	1659
3294	773	3911	1657
3295	775	1659	3911
3296	1657	3911	1659
3297	777	3913	3912
3298	775	3911	3913
3299	773	3912	3911
3300	3913	3911	3912
3301	25	1201	1197
3302	401	2965	1201
3303	397	1197	2965
3304	1201	2965	1197
3305	193	2110	2108
3306	981	2976	2110
3307	401	2108	2976
3308	2110	2976	2108
3309	189	2084	2087
3310	397	2967	2084
3311	981	2087	2967
3312	2084	2967	2087
3313	401	2976	2965
3314	981	2967	2976
3315	397	2965	2967
3316	2976	2967	2965
3317	81	1503	1501

3318	703	3828	1503
3319	701	1501	3828
3320	1503	3828	1501
3321	265	2543	2541
3322	992	3835	2543
3323	703	2541	3835
3324	2543	3835	2541
3325	193	2109	2112
3326	701	3830	2109
3327	992	2112	3830
3328	2109	3830	2112
3329	703	3835	3828
3330	992	3830	3835
3331	701	3828	3830
3332	3835	3830	3828
3333	57	1357	1361
3334	557	3397	1357
3335	561	1361	3397
3336	1357	3397	1361
3337	189	2089	2085
3338	983	3399	2089
3339	557	2085	3399
3340	2089	3399	2085
3341	265	2540	2542
3342	561	3408	2540
3343	983	2542	3408
3344	2540	3408	2542
3345	557	3399	3397
3346	983	3408	3399
3347	561	3397	3408
3348	3399	3408	3397
3349	193	2112	2110
3350	992	4121	2112
3351	981	2110	4121
3352	2112	4121	2110
3353	265	2542	2543
3354	983	4123	2542
3355	992	2543	4123
3356	2542	4123	2543
3357	189	2087	2089
3358	981	4120	2087
3359	983	2089	4120
3360	2087	4120	2089
3361	992	4123	4121
3362	983	4120	4123
3363	981	4121	4120
3364	4123	4120	4121
3365	23	1185	1189
3366	385	2929	1185
3367	389	1189	2929
3368	1185	2929	1189
3369	177	2015	2012
3370	945	2931	2015
3371	385	2012	2931
3372	2015	2931	2012
3373	181	2036	2038
3374	389	2940	2036
3375	945	2038	2940
3376	2036	2940	2038

3377	385	2931	2929
3378	945	2940	2931
3379	389	2929	2940
3380	2931	2940	2929
3381	56	1355	1351
3382	555	3379	1355
3383	551	1351	3379
3384	1355	3379	1351
3385	263	2530	2528
3386	947	3390	2530
3387	555	2528	3390
3388	2530	3390	2528
3389	177	2013	2017
3390	551	3381	2013
3391	947	2017	3381
3392	2013	3381	2017
3393	555	3390	3379
3394	947	3381	3390
3395	551	3379	3381
3396	3390	3381	3379
3397	81	1500	1502
3398	700	3824	1500
3399	702	1502	3824
3400	1500	3824	1502
3401	181	2040	2037
3402	956	3826	2040
3403	700	2037	3826
3404	2040	3826	2037
3405	263	2529	2531
3406	702	3833	2529
3407	956	2531	3833
3408	2529	3833	2531
3409	700	3826	3824
3410	956	3833	3826
3411	702	3824	3833
3412	3826	3833	3824
3413	177	2017	2015
3414	947	4084	2017
3415	945	2015	4084
3416	2017	4084	2015
3417	263	2531	2530
3418	956	4087	2531
3419	947	2530	4087
3420	2531	4087	2530
3421	181	2038	2040
3422	945	4085	2038
3423	956	2040	4085
3424	2038	4085	2040
3425	947	4087	4084
3426	956	4085	4087
3427	945	4084	4085
3428	4087	4085	4084
3429	57	1361	1359
3430	561	3403	1361
3431	559	1359	3403
3432	1361	3403	1359
3433	265	2544	2540
3434	1064	3409	2544
3435	561	2540	3409

3436	2544	3409	2540
3437	261	2517	2521
3438	559	3405	2517
3439	1064	2521	3405
3440	2517	3405	2521
3441	561	3409	3403
3442	1064	3405	3409
3443	559	3403	3405
3444	3409	3405	3403
3445	81	1502	1503
3446	702	3832	1502
3447	703	1503	3832
3448	1502	3832	1503
3449	263	2533	2529
3450	1065	3834	2533
3451	702	2529	3834
3452	2533	3834	2529
3453	265	2541	2545
3454	703	3836	2541
3455	1065	2545	3836
3456	2541	3836	2545
3457	702	3834	3832
3458	1065	3836	3834
3459	703	3832	3836
3460	3834	3836	3832
3461	56	1353	1355
3462	553	3385	1353
3463	555	1355	3385
3464	1353	3385	1355
3465	261	2520	2516
3466	1063	3387	2520
3467	553	2516	3387
3468	2520	3387	2516
3469	263	2528	2532
3470	555	3391	2528
3471	1063	2532	3391
3472	2528	3391	2532
3473	553	3387	3385
3474	1063	3391	3387
3475	555	3385	3391
3476	3387	3391	3385
3477	265	2545	2544
3478	1065	4201	2545
3479	1064	2544	4201
3480	2545	4201	2544
3481	263	2532	2533
3482	1063	4200	2532
3483	1065	2533	4200
3484	2532	4200	2533
3485	261	2521	2520
3486	1064	4199	2521
3487	1063	2520	4199
3488	2521	4199	2520
3489	1065	4200	4201
3490	1063	4199	4200
3491	1064	4201	4199
3492	4200	4199	4201
3493	7	1111	1113
3494	311	2737	1111

3495	313	1113	2737
3496	1111	2737	1113
3497	103	1611	1608
3498	753	2739	1611
3499	311	1608	2739
3500	1611	2739	1608
3501	105	1620	1623
3502	313	2743	1620
3503	753	1623	2743
3504	1620	2743	1623
3505	311	2739	2737
3506	753	2743	2739
3507	313	2737	2743
3508	2739	2743	2737
3509	47	1307	1304
3510	507	3249	1307
3511	504	1304	3249
3512	1307	3249	1304
3513	241	2398	2396
3514	755	3260	2398
3515	507	2396	3260
3516	2398	3260	2396
3517	103	1609	1613
3518	504	3251	1609
3519	755	1613	3251
3520	1609	3251	1613
3521	507	3260	3249
3522	755	3251	3260
3523	504	3249	3251
3524	3260	3251	3249
3525	49	1316	1319
3526	516	3285	1316
3527	519	1319	3285
3528	1316	3285	1319
3529	105	1625	1621
3530	759	3287	1625
3531	516	1621	3287
3532	1625	3287	1621
3533	241	2397	2399
3534	519	3296	2397
3535	759	2399	3296
3536	2397	3296	2399
3537	516	3287	3285
3538	759	3296	3287
3539	519	3285	3296
3540	3287	3296	3285
3541	103	1613	1611
3542	755	3893	1613
3543	753	1611	3893
3544	1613	3893	1611
3545	241	2399	2398
3546	759	3895	2399
3547	755	2398	3895
3548	2399	3895	2398
3549	105	1623	1625
3550	753	3894	1623
3551	759	1625	3894
3552	1623	3894	1625
3553	755	3895	3893

3554	759	3894	3895
3555	753	3893	3894
3556	3895	3894	3893
3557	22	1183	1179
3558	383	2911	1183
3559	379	1179	2911
3560	1183	2911	1179
3561	175	2002	2000
3562	927	2922	2002
3563	383	2000	2922
3564	2002	2922	2000
3565	171	1976	1979
3566	379	2913	1976
3567	927	1979	2913
3568	1976	2913	1979
3569	383	2922	2911
3570	927	2913	2922
3571	379	2911	2913
3572	2922	2913	2911
3573	80	1496	1494
3574	696	3806	1496
3575	694	1494	3806
3576	1496	3806	1494
3577	243	2411	2409
3578	938	3815	2411
3579	696	2409	3815
3580	2411	3815	2409
3581	175	2001	2004
3582	694	3808	2001
3583	938	2004	3808
3584	2001	3808	2004
3585	696	3815	3806
3586	938	3808	3815
3587	694	3806	3808
3588	3815	3808	3806
3589	47	1305	1309
3590	505	3253	1305
3591	509	1309	3253
3592	1305	3253	1309
3593	171	1981	1977
3594	929	3255	1981
3595	505	1977	3255
3596	1981	3255	1977
3597	243	2408	2410
3598	509	3264	2408
3599	929	2410	3264
3600	2408	3264	2410
3601	505	3255	3253
3602	929	3264	3255
3603	509	3253	3264
3604	3255	3264	3253
3605	175	2004	2002
3606	938	4067	2004
3607	927	2002	4067
3608	2004	4067	2002
3609	243	2410	2411
3610	929	4069	2410
3611	938	2411	4069
3612	2410	4069	2411

3613	171	1979	1981
3614	927	4066	1979
3615	929	1981	4066
3616	1979	4066	1981
3617	938	4069	4067
3618	929	4066	4069
3619	927	4067	4066
3620	4069	4066	4067
3621	25	1196	1200
3622	396	2961	1196
3623	400	1200	2961
3624	1196	2961	1200
3625	188	2081	2078
3626	977	2963	2081
3627	396	2078	2963
3628	2081	2963	2078
3629	192	2102	2104
3630	400	2974	2102
3631	977	2104	2974
3632	2102	2974	2104
3633	396	2963	2961
3634	977	2974	2963
3635	400	2961	2974
3636	2963	2974	2961
3637	49	1321	1317
3638	521	3289	1321
3639	517	1317	3289
3640	1321	3289	1317
3641	247	2434	2432
3642	979	3300	2434
3643	521	2432	3300
3644	2434	3300	2432
3645	188	2079	2083
3646	517	3291	2079
3647	979	2083	3291
3648	2079	3291	2083
3649	521	3300	3289
3650	979	3291	3300
3651	517	3289	3291
3652	3300	3291	3289
3653	80	1495	1497
3654	695	3810	1495
3655	697	1497	3810
3656	1495	3810	1497
3657	192	2106	2103
3658	990	3812	2106
3659	695	2103	3812
3660	2106	3812	2103
3661	247	2433	2435
3662	697	3817	2433
3663	990	2435	3817
3664	2433	3817	2435
3665	695	3812	3810
3666	990	3817	3812
3667	697	3810	3817
3668	3812	3817	3810
3669	188	2083	2081
3670	979	4114	2083
3671	977	2081	4114

3672	2083	4114	2081
3673	247	2435	2434
3674	990	4117	2435
3675	979	2434	4117
3676	2435	4117	2434
3677	192	2104	2106
3678	977	4115	2104
3679	990	2106	4115
3680	2104	4115	2106
3681	979	4117	4114
3682	990	4115	4117
3683	977	4114	4115
3684	4117	4115	4114
3685	47	1309	1307
3686	509	3259	1309
3687	507	1307	3259
3688	1309	3259	1307
3689	243	2412	2408
3690	1045	3265	2412
3691	509	2408	3265
3692	2412	3265	2408
3693	241	2396	2400
3694	507	3261	2396
3695	1045	2400	3261
3696	2396	3261	2400
3697	509	3265	3259
3698	1045	3261	3265
3699	507	3259	3261
3700	3265	3261	3259
3701	80	1497	1496
3702	697	3814	1497
3703	696	1496	3814
3704	1497	3814	1496
3705	247	2437	2433
3706	1047	3818	2437
3707	697	2433	3818
3708	2437	3818	2433
3709	243	2409	2413
3710	696	3816	2409
3711	1047	2413	3816
3712	2409	3816	2413
3713	697	3818	3814
3714	1047	3816	3818
3715	696	3814	3816
3716	3818	3816	3814
3717	49	1319	1321
3718	519	3295	1319
3719	521	1321	3295
3720	1319	3295	1321
3721	241	2401	2397
3722	1046	3297	2401
3723	519	2397	3297
3724	2401	3297	2397
3725	247	2432	2436
3726	521	3301	2432
3727	1046	2436	3301
3728	2432	3301	2436
3729	519	3297	3295
3730	1046	3301	3297

3731	521	3295	3301
3732	3297	3301	3295
3733	243	2413	2412
3734	1047	4182	2413
3735	1045	2412	4182
3736	2413	4182	2412
3737	247	2436	2437
3738	1046	4183	2436
3739	1047	2437	4183
3740	2436	4183	2437
3741	241	2400	2401
3742	1045	4181	2400
3743	1046	2401	4181
3744	2400	4181	2401
3745	1047	4183	4182
3746	1046	4181	4183
3747	1045	4182	4181
3748	4183	4181	4182
3749	181	2039	2036
3750	953	2941	2039
3751	389	2036	2941
3752	2039	2941	2036
3753	180	2030	2033
3754	388	2939	2030
3755	953	2033	2939
3756	2030	2939	2033
3757	81	1504	1500
3758	704	3825	1504
3759	700	1500	3825
3760	1504	3825	1500
3761	288	2681	2679
3762	957	3838	2681
3763	704	2679	3838
3764	2681	3838	2679
3765	181	2037	2041
3766	700	3827	2037
3767	957	2041	3827
3768	2037	3827	2041
3769	704	3838	3825
3770	957	3827	3838
3771	700	3825	3827
3772	3838	3827	3825
3773	79	1489	1493
3774	689	3793	1489
3775	693	1493	3793
3776	1489	3793	1493
3777	180	2035	2031
3778	955	3795	2035
3779	689	2031	3795
3780	2035	3795	2031
3781	288	2678	2680
3782	693	3804	2678
3783	955	2680	3804
3784	2678	3804	2680
3785	689	3795	3793
3786	955	3804	3795
3787	693	3793	3804
3788	3795	3804	3793
3789	181	2041	2039

3790	957	4092	2041
3791	953	2039	4092
3792	2041	4092	2039
3793	288	2680	2681
3794	955	4093	2680
3795	957	2681	4093
3796	2680	4093	2681
3797	180	2033	2035
3798	953	4091	2033
3799	955	2035	4091
3800	2033	4091	2035
3801	957	4093	4092
3802	955	4091	4093
3803	953	4092	4091
3804	4093	4091	4092
3805	25	1200	1201
3806	400	2973	1200
3807	401	1201	2973
3808	1200	2973	1201
3809	192	2105	2102
3810	989	2975	2105
3811	400	2102	2975
3812	2105	2975	2102
3813	193	2108	2111
3814	401	2977	2108
3815	989	2111	2977
3816	2108	2977	2111
3817	400	2975	2973
3818	989	2977	2975
3819	401	2973	2977
3820	2975	2977	2973
3821	80	1499	1495
3822	699	3811	1499
3823	695	1495	3811
3824	1499	3811	1495
3825	289	2686	2684
3826	991	3822	2686
3827	699	2684	3822
3828	2686	3822	2684
3829	192	2103	2107
3830	695	3813	2103
3831	991	2107	3813
3832	2103	3813	2107
3833	699	3822	3811
3834	991	3813	3822
3835	695	3811	3813
3836	3822	3813	3811
3837	81	1501	1505
3838	701	3829	1501
3839	705	1505	3829
3840	1501	3829	1505
3841	193	2113	2109
3842	993	3831	2113
3843	701	2109	3831
3844	2113	3831	2109
3845	289	2685	2687
3846	705	3840	2685
3847	993	2687	3840
3848	2685	3840	2687

3849	701	3831	3829
3850	993	3840	3831
3851	705	3829	3840
3852	3831	3840	3829
3853	192	2107	2105
3854	991	4127	2107
3855	989	2105	4127
3856	2107	4127	2105
3857	289	2687	2686
3858	993	4129	2687
3859	991	2686	4129
3860	2687	4129	2686
3861	193	2111	2113
3862	989	4128	2111
3863	993	2113	4128
3864	2111	4128	2113
3865	991	4129	4127
3866	993	4128	4129
3867	989	4127	4128
3868	4129	4128	4127
3869	22	1182	1183
3870	382	2919	1182
3871	383	1183	2919
3872	1182	2919	1183
3873	174	1997	1994
3874	935	2921	1997
3875	382	1994	2921
3876	1997	2921	1994
3877	175	2000	2003
3878	383	2923	2000
3879	935	2003	2923
3880	2000	2923	2003
3881	382	2921	2919
3882	935	2923	2921
3883	383	2919	2923
3884	2921	2923	2919
3885	79	1492	1488
3886	692	3789	1492
3887	688	1488	3789
3888	1492	3789	1488
3889	287	2674	2672
3890	937	3802	2674
3891	692	2672	3802
3892	2674	3802	2672
3893	174	1995	1999
3894	688	3791	1995
3895	937	1999	3791
3896	1995	3791	1999
3897	692	3802	3789
3898	937	3791	3802
3899	688	3789	3791
3900	3802	3791	3789
3901	80	1494	1498
3902	694	3807	1494
3903	698	1498	3807
3904	1494	3807	1498
3905	175	2005	2001
3906	939	3809	2005
3907	694	2001	3809

3908	2005	3809	2001
3909	287	2673	2675
3910	698	3820	2673
3911	939	2675	3820
3912	2673	3820	2675
3913	694	3809	3807
3914	939	3820	3809
3915	698	3807	3820
3916	3809	3820	3807
3917	174	1999	1997
3918	937	4073	1999
3919	935	1997	4073
3920	1999	4073	1997
3921	287	2675	2674
3922	939	4075	2675
3923	937	2674	4075
3924	2675	4075	2674
3925	175	2003	2005
3926	935	4074	2003
3927	939	2005	4074
3928	2003	4074	2005
3929	937	4075	4073
3930	939	4074	4075
3931	935	4073	4074
3932	4075	4074	4073
3933	81	1505	1504
3934	705	3837	1505
3935	704	1504	3837
3936	1505	3837	1504
3937	289	2689	2685
3938	1089	3841	2689
3939	705	2685	3841
3940	2689	3841	2685
3941	288	2679	2683
3942	704	3839	2679
3943	1089	2683	3839
3944	2679	3839	2683
3945	705	3841	3837
3946	1089	3839	3841
3947	704	3837	3839
3948	3841	3839	3837
3949	80	1498	1499
3950	698	3819	1498
3951	699	1499	3819
3952	1498	3819	1499
3953	287	2677	2673
3954	1088	3821	2677
3955	698	2673	3821
3956	2677	3821	2673
3957	289	2684	2688
3958	699	3823	2684
3959	1088	2688	3823
3960	2684	3823	2688
3961	698	3821	3819
3962	1088	3823	3821
3963	699	3819	3823
3964	3821	3823	3819
3965	79	1493	1492
3966	693	3801	1493

3967	692	1492	3801
3968	1493	3801	1492
3969	288	2682	2678
3970	1087	3805	2682
3971	693	2678	3805
3972	2682	3805	2678
3973	287	2672	2676
3974	692	3803	2672
3975	1087	2676	3803
3976	2672	3803	2676
3977	693	3805	3801
3978	1087	3803	3805
3979	692	3801	3803
3980	3805	3803	3801
3981	289	2688	2689
3982	1088	4225	2688
3983	1089	2689	4225
3984	2688	4225	2689
3985	287	2676	2677
3986	1087	4223	2676
3987	1088	2677	4223
3988	2676	4223	2677
3989	288	2683	2682
3990	1089	4224	2683
3991	1087	2682	4224
3992	2683	4224	2682
3993	1088	4223	4225
3994	1087	4224	4223
3995	1089	4225	4224
3996	4223	4224	4225
3997	1	1092	1090
3998	292	2690	1092
3999	290	1090	2690
4000	1092	2690	1090
4001	84	1516	1514
4002	706	2694	1516
4003	292	1514	2694
4004	1516	2694	1514
4005	82	1506	1508
4006	290	2691	1506
4007	706	1508	2691
4008	1506	2691	1508
4009	292	2694	2690
4010	706	2691	2694
4011	290	2690	2691
4012	2694	2691	2690
4013	28	1212	1210
4014	412	2996	1212
4015	410	1210	2996
4016	1212	2996	1210
4017	26	1202	1204
4018	402	2978	1202
4019	404	1204	2978
4020	1202	2978	1204
4021	82	1509	1507
4022	707	2979	1509
4023	402	1507	2979
4024	1509	2979	1507
4025	194	2114	2116

4026	404	2983	2114
4027	707	2116	2983
4028	2114	2983	2116
4029	402	2979	2978
4030	707	2983	2979
4031	404	2978	2983
4032	2979	2983	2978
4033	84	1518	1516
4034	710	3843	1518
4035	706	1516	3843
4036	1518	3843	1516
4037	194	2116	2117
4038	707	3844	2116
4039	710	2117	3844
4040	2116	3844	2117
4041	82	1508	1509
4042	706	3842	1508
4043	707	1509	3842
4044	1508	3842	1509
4045	710	3844	3843
4046	707	3842	3844
4047	706	3843	3842
4048	3844	3842	3843
4049	12	1132	1130
4050	332	2780	1132
4051	330	1130	2780
4052	1132	2780	1130
4053	124	1720	1718
4054	796	2789	1720
4055	332	1718	2789
4056	1720	2789	1718
4057	122	1706	1708
4058	330	2782	1706
4059	796	1708	2782
4060	1706	2782	1708
4061	332	2789	2780
4062	796	2782	2789
4063	330	2780	2782
4064	2789	2782	2780
4065	58	1365	1363
4066	565	3414	1365
4067	563	1363	3414
4068	1365	3414	1363
4069	198	2141	2139
4070	805	3421	2141
4071	565	2139	3421
4072	2141	3421	2139
4073	124	1719	1722
4074	563	3416	1719
4075	805	1722	3416
4076	1719	3416	1722
4077	565	3421	3414
4078	805	3416	3421
4079	563	3414	3416
4080	3421	3416	3414
4081	28	1211	1214
4082	411	3000	1211
4083	414	1214	3000
4084	1211	3000	1214

4085	122	1710	1707
4086	798	3002	1710
4087	411	1707	3002
4088	1710	3002	1707
4089	198	2138	2140
4090	414	3010	2138
4091	798	2140	3010
4092	2138	3010	2140
4093	411	3002	3000
4094	798	3010	3002
4095	414	3000	3010
4096	3002	3010	3000
4097	124	1722	1720
4098	805	3933	1722
4099	796	1720	3933
4100	1722	3933	1720
4101	198	2140	2141
4102	798	3936	2140
4103	805	2141	3936
4104	2140	3936	2141
4105	122	1708	1710
4106	796	3932	1708
4107	798	1710	3932
4108	1708	3932	1710
4109	805	3936	3933
4110	798	3932	3936
4111	796	3933	3932
4112	3936	3932	3933
4113	10	1122	1124
4114	322	2762	1122
4115	324	1124	2762
4116	1122	2762	1124
4117	114	1668	1666
4118	778	2763	1668
4119	322	1666	2763
4120	1668	2763	1666
4121	116	1674	1676
4122	324	2767	1674
4123	778	1676	2767
4124	1674	2767	1676
4125	322	2763	2762
4126	778	2767	2763
4127	324	2762	2767
4128	2763	2767	2762
4129	26	1205	1203
4130	405	2980	1205
4131	403	1203	2980
4132	1205	2980	1203
4133	195	2122	2120
4134	779	2985	2122
4135	405	2120	2985
4136	2122	2985	2120
4137	114	1667	1669
4138	403	2981	1667
4139	779	1669	2981
4140	1667	2981	1669
4141	405	2985	2980
4142	779	2981	2985
4143	403	2980	2981

4144	2985	2981	2980
4145	58	1362	1364
4146	562	3410	1362
4147	564	1364	3410
4148	1362	3410	1364
4149	116	1678	1675
4150	783	3412	1678
4151	562	1675	3412
4152	1678	3412	1675
4153	195	2121	2123
4154	564	3419	2121
4155	783	2123	3419
4156	2121	3419	2123
4157	562	3412	3410
4158	783	3419	3412
4159	564	3410	3419
4160	3412	3419	3410
4161	114	1669	1668
4162	779	3914	1669
4163	778	1668	3914
4164	1669	3914	1668
4165	195	2123	2122
4166	783	3916	2123
4167	779	2122	3916
4168	2123	3916	2122
4169	116	1676	1678
4170	778	3915	1676
4171	783	1678	3915
4172	1676	3915	1678
4173	779	3916	3914
4174	783	3915	3916
4175	778	3914	3915
4176	3916	3915	3914
4177	28	1214	1212
4178	414	3004	1214
4179	412	1212	3004
4180	1214	3004	1212
4181	198	2142	2138
4182	995	3011	2142
4183	414	2138	3011
4184	2142	3011	2138
4185	194	2115	2119
4186	412	3006	2115
4187	995	2119	3006
4188	2115	3006	2119
4189	414	3011	3004
4190	995	3006	3011
4191	412	3004	3006
4192	3011	3006	3004
4193	58	1364	1365
4194	564	3418	1364
4195	565	1365	3418
4196	1364	3418	1365
4197	195	2125	2121
4198	996	3420	2125
4199	564	2121	3420
4200	2125	3420	2121
4201	198	2139	2143
4202	565	3422	2139

4203	996	2143	3422
4204	2139	3422	2143
4205	564	3420	3418
4206	996	3422	3420
4207	565	3418	3422
4208	3420	3422	3418
4209	26	1204	1205
4210	404	2982	1204
4211	405	1205	2982
4212	1204	2982	1205
4213	194	2118	2114
4214	994	2984	2118
4215	404	2114	2984
4216	2118	2984	2114
4217	195	2120	2124
4218	405	2986	2120
4219	994	2124	2986
4220	2120	2986	2124
4221	404	2984	2982
4222	994	2986	2984
4223	405	2982	2986
4224	2984	2986	2982
4225	198	2143	2142
4226	996	4132	2143
4227	995	2142	4132
4228	2143	4132	2142
4229	195	2124	2125
4230	994	4131	2124
4231	996	2125	4131
4232	2124	4131	2125
4233	194	2119	2118
4234	995	4130	2119
4235	994	2118	4130
4236	2119	4130	2118
4237	996	4131	4132
4238	994	4130	4131
4239	995	4132	4130
4240	4131	4130	4132
4241	7	1110	1108
4242	310	2726	1110
4243	308	1108	2726
4244	1110	2726	1108
4245	102	1604	1602
4246	742	2735	1604
4247	310	1602	2735
4248	1604	2735	1602
4249	100	1590	1592
4250	308	2728	1590
4251	742	1592	2728
4252	1590	2728	1592
4253	310	2735	2726
4254	742	2728	2735
4255	308	2726	2728
4256	2735	2728	2726
4257	46	1300	1298
4258	500	3230	1300
4259	498	1298	3230
4260	1300	3230	1298
4261	230	2333	2331

4262	751	3239	2333
4263	500	2331	3239
4264	2333	3239	2331
4265	102	1603	1606
4266	498	3232	1603
4267	751	1606	3232
4268	1603	3232	1606
4269	500	3239	3230
4270	751	3232	3239
4271	498	3230	3232
4272	3239	3232	3230
4273	44	1286	1288
4274	486	3194	1286
4275	488	1288	3194
4276	1286	3194	1288
4277	100	1594	1591
4278	744	3196	1594
4279	486	1591	3196
4280	1594	3196	1591
4281	230	2330	2332
4282	488	3203	2330
4283	744	2332	3203
4284	2330	3203	2332
4285	486	3196	3194
4286	744	3203	3196
4287	488	3194	3203
4288	3196	3203	3194
4289	102	1606	1604
4290	751	3879	1606
4291	742	1604	3879
4292	1606	3879	1604
4293	230	2332	2333
4294	744	3882	2332
4295	751	2333	3882
4296	2332	3882	2333
4297	100	1592	1594
4298	742	3878	1592
4299	744	1594	3878
4300	1592	3878	1594
4301	751	3882	3879
4302	744	3878	3882
4303	742	3879	3878
4304	3882	3878	3879
4305	20	1169	1167
4306	369	2874	1169
4307	367	1167	2874
4308	1169	2874	1167
4309	161	1918	1916
4310	890	2881	1918
4311	369	1916	2881
4312	1918	2881	1916
4313	159	1904	1906
4314	367	2876	1904
4315	890	1906	2876
4316	1904	2876	1906
4317	369	2881	2874
4318	890	2876	2881
4319	367	2874	2876
4320	2881	2876	2874

4321	62	1389	1387
4322	589	3486	1389
4323	587	1387	3486
4324	1389	3486	1387
4325	239	2387	2385
4326	897	3493	2387
4327	589	2385	3493
4328	2387	3493	2385
4329	161	1917	1920
4330	587	3488	1917
4331	897	1920	3488
4332	1917	3488	1920
4333	589	3493	3486
4334	897	3488	3493
4335	587	3486	3488
4336	3493	3488	3486
4337	46	1299	1302
4338	499	3234	1299
4339	502	1302	3234
4340	1299	3234	1302
4341	159	1908	1905
4342	892	3236	1908
4343	499	1905	3236
4344	1908	3236	1905
4345	239	2384	2386
4346	502	3244	2384
4347	892	2386	3244
4348	2384	3244	2386
4349	499	3236	3234
4350	892	3244	3236
4351	502	3234	3244
4352	3236	3244	3234
4353	161	1920	1918
4354	897	4029	1920
4355	890	1918	4029
4356	1920	4029	1918
4357	239	2386	2387
4358	892	4032	2386
4359	897	2387	4032
4360	2386	4032	2387
4361	159	1906	1908
4362	890	4028	1906
4363	892	1908	4028
4364	1906	4028	1908
4365	897	4032	4029
4366	892	4028	4032
4367	890	4029	4028
4368	4032	4028	4029
4369	12	1131	1134
4370	331	2784	1131
4371	334	1134	2784
4372	1131	2784	1134
4373	123	1714	1712
4374	800	2786	1714
4375	331	1712	2786
4376	1714	2786	1712
4377	126	1730	1732
4378	334	2794	1730
4379	800	1732	2794

4380	1730	2794	1732
4381	331	2786	2784
4382	800	2794	2786
4383	334	2784	2794
4384	2786	2794	2784
4385	44	1290	1287
4386	490	3198	1290
4387	487	1287	3198
4388	1290	3198	1287
4389	232	2344	2342
4390	802	3208	2344
4391	490	2342	3208
4392	2344	3208	2342
4393	123	1713	1716
4394	487	3200	1713
4395	802	1716	3200
4396	1713	3200	1716
4397	490	3208	3198
4398	802	3200	3208
4399	487	3198	3200
4400	3208	3200	3198
4401	62	1386	1388
4402	586	3482	1386
4403	588	1388	3482
4404	1386	3482	1388
4405	126	1734	1731
4406	810	3484	1734
4407	586	1731	3484
4408	1734	3484	1731
4409	232	2343	2345
4410	588	3491	2343
4411	810	2345	3491
4412	2343	3491	2345
4413	586	3484	3482
4414	810	3491	3484
4415	588	3482	3491
4416	3484	3491	3482
4417	123	1716	1714
4418	802	3938	1716
4419	800	1714	3938
4420	1716	3938	1714
4421	232	2345	2344
4422	810	3942	2345
4423	802	2344	3942
4424	2345	3942	2344
4425	126	1732	1734
4426	800	3939	1732
4427	810	1734	3939
4428	1732	3939	1734
4429	802	3942	3938
4430	810	3939	3942
4431	800	3938	3939
4432	3942	3939	3938
4433	46	1302	1300
4434	502	3238	1302
4435	500	1300	3238
4436	1302	3238	1300
4437	239	2388	2384
4438	1031	3245	2388

4439	502	2384	3245
4440	2388	3245	2384
4441	230	2331	2335
4442	500	3240	2331
4443	1031	2335	3240
4444	2331	3240	2335
4445	502	3245	3238
4446	1031	3240	3245
4447	500	3238	3240
4448	3245	3240	3238
4449	62	1388	1389
4450	588	3490	1388
4451	589	1389	3490
4452	1388	3490	1389
4453	232	2347	2343
4454	1034	3492	2347
4455	588	2343	3492
4456	2347	3492	2343
4457	239	2385	2389
4458	589	3494	2385
4459	1034	2389	3494
4460	2385	3494	2389
4461	588	3492	3490
4462	1034	3494	3492
4463	589	3490	3494
4464	3492	3494	3490
4465	44	1288	1290
4466	488	3202	1288
4467	490	1290	3202
4468	1288	3202	1290
4469	230	2334	2330
4470	1030	3204	2334
4471	488	2330	3204
4472	2334	3204	2330
4473	232	2342	2346
4474	490	3209	2342
4475	1030	2346	3209
4476	2342	3209	2346
4477	488	3204	3202
4478	1030	3209	3204
4479	490	3202	3209
4480	3204	3209	3202
4481	239	2389	2388
4482	1034	4168	2389
4483	1031	2388	4168
4484	2389	4168	2388
4485	232	2346	2347
4486	1030	4167	2346
4487	1034	2347	4167
4488	2346	4167	2347
4489	230	2335	2334
4490	1031	4166	2335
4491	1030	2334	4166
4492	2335	4166	2334
4493	1034	4167	4168
4494	1030	4166	4167
4495	1031	4168	4166
4496	4167	4166	4168
4497	5	1100	1102

4498	300	2708	1100
4499	302	1102	2708
4500	1100	2708	1102
4501	92	1552	1550
4502	724	2709	1552
4503	300	1550	2709
4504	1552	2709	1550
4505	94	1558	1560
4506	302	2713	1558
4507	724	1560	2713
4508	1558	2713	1560
4509	300	2709	2708
4510	724	2713	2709
4511	302	2708	2713
4512	2709	2713	2708
4513	36	1248	1246
4514	448	3086	1248
4515	446	1246	3086
4516	1248	3086	1246
4517	212	2224	2222
4518	725	3091	2224
4519	448	2222	3091
4520	2224	3091	2222
4521	92	1551	1553
4522	446	3087	1551
4523	725	1553	3087
4524	1551	3087	1553
4525	448	3091	3086
4526	725	3087	3091
4527	446	3086	3087
4528	3091	3087	3086
4529	38	1254	1256
4530	454	3104	1254
4531	456	1256	3104
4532	1254	3104	1256
4533	94	1562	1559
4534	729	3106	1562
4535	454	1559	3106
4536	1562	3106	1559
4537	212	2223	2225
4538	456	3113	2223
4539	729	2225	3113
4540	2223	3113	2225
4541	454	3106	3104
4542	729	3113	3106
4543	456	3104	3113
4544	3106	3113	3104
4545	92	1553	1552
4546	725	3860	1553
4547	724	1552	3860
4548	1553	3860	1552
4549	212	2225	2224
4550	729	3862	2225
4551	725	2224	3862
4552	2225	3862	2224
4553	94	1560	1562
4554	724	3861	1560
4555	729	1562	3861
4556	1560	3861	1562

4557	725	3862	3860
4558	729	3861	3862
4559	724	3860	3861
4560	3862	3861	3860
4561	10	1125	1123
4562	325	2764	1125
4563	323	1123	2764
4564	1125	2764	1123
4565	117	1682	1680
4566	780	2769	1682
4567	325	1680	2769
4568	1682	2769	1680
4569	115	1670	1672
4570	323	2765	1670
4571	780	1672	2765
4572	1670	2765	1672
4573	325	2769	2764
4574	780	2765	2769
4575	323	2764	2765
4576	2769	2765	2764
4577	59	1370	1368
4578	570	3428	1370
4579	568	1368	3428
4580	1370	3428	1368
4581	213	2231	2229
4582	785	3437	2231
4583	570	2229	3437
4584	2231	3437	2229
4585	117	1681	1684
4586	568	3430	1681
4587	785	1684	3430
4588	1681	3430	1684
4589	570	3437	3428
4590	785	3430	3437
4591	568	3428	3430
4592	3437	3430	3428
4593	36	1247	1249
4594	447	3088	1247
4595	449	1249	3088
4596	1247	3088	1249
4597	115	1673	1671
4598	781	3089	1673
4599	447	1671	3089
4600	1673	3089	1671
4601	213	2228	2230
4602	449	3093	2228
4603	781	2230	3093
4604	2228	3093	2230
4605	447	3089	3088
4606	781	3093	3089
4607	449	3088	3093
4608	3089	3093	3088
4609	117	1684	1682
4610	785	3918	1684
4611	780	1682	3918
4612	1684	3918	1682
4613	213	2230	2231
4614	781	3919	2230
4615	785	2231	3919

4616	2230	3919	2231
4617	115	1672	1673
4618	780	3917	1672
4619	781	1673	3917
4620	1672	3917	1673
4621	785	3919	3918
4622	781	3917	3919
4623	780	3918	3917
4624	3919	3917	3918
4625	20	1166	1168
4626	366	2870	1166
4627	368	1168	2870
4628	1166	2870	1168
4629	158	1900	1898
4630	886	2872	1900
4631	366	1898	2872
4632	1900	2872	1898
4633	160	1910	1912
4634	368	2879	1910
4635	886	1912	2879
4636	1910	2879	1912
4637	366	2872	2870
4638	886	2879	2872
4639	368	2870	2879
4640	2872	2879	2870
4641	38	1258	1255
4642	458	3108	1258
4643	455	1255	3108
4644	1258	3108	1255
4645	217	2254	2252
4646	888	3118	2254
4647	458	2252	3118
4648	2254	3118	2252
4649	158	1899	1902
4650	455	3110	1899
4651	888	1902	3110
4652	1899	3110	1902
4653	458	3118	3108
4654	888	3110	3118
4655	455	3108	3110
4656	3118	3110	3108
4657	59	1369	1371
4658	569	3432	1369
4659	571	1371	3432
4660	1369	3432	1371
4661	160	1914	1911
4662	895	3434	1914
4663	569	1911	3434
4664	1914	3434	1911
4665	217	2253	2255
4666	571	3439	2253
4667	895	2255	3439
4668	2253	3439	2255
4669	569	3434	3432
4670	895	3439	3434
4671	571	3432	3439
4672	3434	3439	3432
4673	158	1902	1900
4674	888	4022	1902

4675	886	1900	4022
4676	1902	4022	1900
4677	217	2255	2254
4678	895	4026	2255
4679	888	2254	4026
4680	2255	4026	2254
4681	160	1912	1914
4682	886	4023	1912
4683	895	1914	4023
4684	1912	4023	1914
4685	888	4026	4022
4686	895	4023	4026
4687	886	4022	4023
4688	4026	4023	4022
4689	36	1249	1248
4690	449	3090	1249
4691	448	1248	3090
4692	1249	3090	1248
4693	213	2232	2228
4694	1012	3094	2232
4695	449	2228	3094
4696	2232	3094	2228
4697	212	2222	2226
4698	448	3092	2222
4699	1012	2226	3092
4700	2222	3092	2226
4701	449	3094	3090
4702	1012	3092	3094
4703	448	3090	3092
4704	3094	3092	3090
4705	59	1371	1370
4706	571	3436	1371
4707	570	1370	3436
4708	1371	3436	1370
4709	217	2257	2253
4710	1014	3440	2257
4711	571	2253	3440
4712	2257	3440	2253
4713	213	2229	2233
4714	570	3438	2229
4715	1014	2233	3438
4716	2229	3438	2233
4717	571	3440	3436
4718	1014	3438	3440
4719	570	3436	3438
4720	3440	3438	3436
4721	38	1256	1258
4722	456	3112	1256
4723	458	1258	3112
4724	1256	3112	1258
4725	212	2227	2223
4726	1013	3114	2227
4727	456	2223	3114
4728	2227	3114	2223
4729	217	2252	2256
4730	458	3119	2252
4731	1013	2256	3119
4732	2252	3119	2256
4733	456	3114	3112

4734	1013	3119	3114
4735	458	3112	3119
4736	3114	3119	3112
4737	213	2233	2232
4738	1014	4149	2233
4739	1012	2232	4149
4740	2233	4149	2232
4741	217	2256	2257
4742	1013	4150	2256
4743	1014	2257	4150
4744	2256	4150	2257
4745	212	2226	2227
4746	1012	4148	2226
4747	1013	2227	4148
4748	2226	4148	2227
4749	1014	4150	4149
4750	1013	4148	4150
4751	1012	4149	4148
4752	4150	4148	4149
4753	12	1134	1132
4754	334	2788	1134
4755	332	1132	2788
4756	1134	2788	1132
4757	126	1733	1730
4758	804	2795	1733
4759	334	1730	2795
4760	1733	2795	1730
4761	124	1718	1721
4762	332	2790	1718
4763	804	1721	2790
4764	1718	2790	1721
4765	334	2795	2788
4766	804	2790	2795
4767	332	2788	2790
4768	2795	2790	2788
4769	62	1390	1386
4770	590	3483	1390
4771	586	1386	3483
4772	1390	3483	1386
4773	267	2555	2553
4774	811	3496	2555
4775	590	2553	3496
4776	2555	3496	2553
4777	126	1731	1735
4778	586	3485	1731
4779	811	1735	3485
4780	1731	3485	1735
4781	590	3496	3483
4782	811	3485	3496
4783	586	3483	3485
4784	3496	3485	3483
4785	58	1363	1367
4786	563	3415	1363
4787	567	1367	3415
4788	1363	3415	1367
4789	124	1723	1719
4790	806	3417	1723
4791	563	1719	3417
4792	1723	3417	1719

4793	267	2552	2554
4794	567	3426	2552
4795	806	2554	3426
4796	2552	3426	2554
4797	563	3417	3415
4798	806	3426	3417
4799	567	3415	3426
4800	3417	3426	3415
4801	126	1735	1733
4802	811	3945	1735
4803	804	1733	3945
4804	1735	3945	1733
4805	267	2554	2555
4806	806	3946	2554
4807	811	2555	3946
4808	2554	3946	2555
4809	124	1721	1723
4810	804	3944	1721
4811	806	1723	3944
4812	1721	3944	1723
4813	811	3946	3945
4814	806	3944	3946
4815	804	3945	3944
4816	3946	3944	3945
4817	20	1168	1169
4818	368	2878	1168
4819	369	1169	2878
4820	1168	2878	1169
4821	160	1913	1910
4822	894	2880	1913
4823	368	1910	2880
4824	1913	2880	1910
4825	161	1916	1919
4826	369	2882	1916
4827	894	1919	2882
4828	1916	2882	1919
4829	368	2880	2878
4830	894	2882	2880
4831	369	2878	2882
4832	2880	2882	2878
4833	59	1373	1369
4834	573	3433	1373
4835	569	1369	3433
4836	1373	3433	1369
4837	268	2560	2558
4838	896	3444	2560
4839	573	2558	3444
4840	2560	3444	2558
4841	160	1911	1915
4842	569	3435	1911
4843	896	1915	3435
4844	1911	3435	1915
4845	573	3444	3433
4846	896	3435	3444
4847	569	3433	3435
4848	3444	3435	3433
4849	62	1387	1391
4850	587	3487	1387
4851	591	1391	3487

4852	1387	3487	1391
4853	161	1921	1917
4854	898	3489	1921
4855	587	1917	3489
4856	1921	3489	1917
4857	268	2559	2561
4858	591	3498	2559
4859	898	2561	3498
4860	2559	3498	2561
4861	587	3489	3487
4862	898	3498	3489
4863	591	3487	3498
4864	3489	3498	3487
4865	160	1915	1913
4866	896	4034	1915
4867	894	1913	4034
4868	1915	4034	1913
4869	268	2561	2560
4870	898	4036	2561
4871	896	2560	4036
4872	2561	4036	2560
4873	161	1919	1921
4874	894	4035	1919
4875	898	1921	4035
4876	1919	4035	1921
4877	896	4036	4034
4878	898	4035	4036
4879	894	4034	4035
4880	4036	4035	4034
4881	10	1124	1125
4882	324	2766	1124
4883	325	1125	2766
4884	1124	2766	1125
4885	116	1677	1674
4886	782	2768	1677
4887	324	1674	2768
4888	1677	2768	1674
4889	117	1680	1683
4890	325	2770	1680
4891	782	1683	2770
4892	1680	2770	1683
4893	324	2768	2766
4894	782	2770	2768
4895	325	2766	2770
4896	2768	2770	2766
4897	58	1366	1362
4898	566	3411	1366
4899	562	1362	3411
4900	1366	3411	1362
4901	266	2548	2546
4902	784	3424	2548
4903	566	2546	3424
4904	2548	3424	2546
4905	116	1675	1679
4906	562	3413	1675
4907	784	1679	3413
4908	1675	3413	1679
4909	566	3424	3411
4910	784	3413	3424

4911	562	3411	3413
4912	3424	3413	3411
4913	59	1368	1372
4914	568	3429	1368
4915	572	1372	3429
4916	1368	3429	1372
4917	117	1685	1681
4918	786	3431	1685
4919	568	1681	3431
4920	1685	3431	1681
4921	266	2547	2549
4922	572	3442	2547
4923	786	2549	3442
4924	2547	3442	2549
4925	568	3431	3429
4926	786	3442	3431
4927	572	3429	3442
4928	3431	3442	3429
4929	116	1679	1677
4930	784	3920	1679
4931	782	1677	3920
4932	1679	3920	1677
4933	266	2549	2548
4934	786	3922	2549
4935	784	2548	3922
4936	2549	3922	2548
4937	117	1683	1685
4938	782	3921	1683
4939	786	1685	3921
4940	1683	3921	1685
4941	784	3922	3920
4942	786	3921	3922
4943	782	3920	3921
4944	3922	3921	3920
4945	62	1391	1390
4946	591	3495	1391
4947	590	1390	3495
4948	1391	3495	1390
4949	268	2563	2559
4950	1068	3499	2563
4951	591	2559	3499
4952	2563	3499	2559
4953	267	2553	2557
4954	590	3497	2553
4955	1068	2557	3497
4956	2553	3497	2557
4957	591	3499	3495
4958	1068	3497	3499
4959	590	3495	3497
4960	3499	3497	3495
4961	59	1372	1373
4962	572	3441	1372
4963	573	1373	3441
4964	1372	3441	1373
4965	266	2551	2547
4966	1067	3443	2551
4967	572	2547	3443
4968	2551	3443	2547
4969	268	2558	2562

4970	573	3445	2558
4971	1067	2562	3445
4972	2558	3445	2562
4973	572	3443	3441
4974	1067	3445	3443
4975	573	3441	3445
4976	3443	3445	3441
4977	58	1367	1366
4978	567	3423	1367
4979	566	1366	3423
4980	1367	3423	1366
4981	267	2556	2552
4982	1066	3427	2556
4983	567	2552	3427
4984	2556	3427	2552
4985	266	2546	2550
4986	566	3425	2546
4987	1066	2550	3425
4988	2546	3425	2550
4989	567	3427	3423
4990	1066	3425	3427
4991	566	3423	3425
4992	3427	3425	3423
4993	268	2562	2563
4994	1067	4204	2562
4995	1068	2563	4204
4996	2562	4204	2563
4997	266	2550	2551
4998	1066	4202	2550
4999	1067	2551	4202
5000	2550	4202	2551
5001	267	2557	2556
5002	1068	4203	2557
5003	1066	2556	4203
5004	2557	4203	2556
5005	1067	4202	4204
5006	1066	4203	4202
5007	1068	4204	4203
5008	4202	4203	4204
5009	4	1098	1097
5010	298	2702	1098
5011	297	1097	2702
5012	1098	2702	1097
5013	90	1544	1542
5014	718	2706	1544
5015	298	1542	2706
5016	1544	2706	1542
5017	89	1536	1538
5018	297	2704	1536
5019	718	1538	2704
5020	1536	2704	1538
5021	298	2706	2702
5022	718	2704	2706
5023	297	2702	2704
5024	2706	2704	2702
5025	34	1240	1238
5026	440	3068	1240
5027	438	1238	3068
5028	1240	3068	1238

5029	206	2189	2187
5030	722	3073	2189
5031	440	2187	3073
5032	2189	3073	2187
5033	90	1543	1545
5034	438	3069	1543
5035	722	1545	3069
5036	1543	3069	1545
5037	440	3073	3068
5038	722	3069	3073
5039	438	3068	3069
5040	3073	3069	3068
5041	33	1232	1234
5042	432	3050	1232
5043	434	1234	3050
5044	1232	3050	1234
5045	89	1540	1537
5046	720	3052	1540
5047	432	1537	3052
5048	1540	3052	1537
5049	206	2186	2188
5050	434	3059	2186
5051	720	2188	3059
5052	2186	3059	2188
5053	432	3052	3050
5054	720	3059	3052
5055	434	3050	3059
5056	3052	3059	3050
5057	90	1545	1544
5058	722	3855	1545
5059	718	1544	3855
5060	1545	3855	1544
5061	206	2188	2189
5062	720	3858	2188
5063	722	2189	3858
5064	2188	3858	2189
5065	89	1538	1540
5066	718	3854	1538
5067	720	1540	3854
5068	1538	3854	1540
5069	722	3858	3855
5070	720	3854	3858
5071	718	3855	3854
5072	3858	3854	3855
5073	18	1160	1158
5074	360	2852	1160
5075	358	1158	2852
5076	1160	2852	1158
5077	152	1868	1866
5078	868	2857	1868
5079	360	1866	2857
5080	1868	2857	1866
5081	150	1858	1860
5082	358	2853	1858
5083	868	1860	2853
5084	1858	2853	1860
5085	360	2857	2852
5086	868	2853	2857
5087	358	2852	2853

5088	2857	2853	2852
5089	70	1437	1435
5090	637	3630	1437
5091	635	1435	3630
5092	1437	3630	1435
5093	210	2213	2211
5094	873	3637	2213
5095	637	2211	3637
5096	2213	3637	2211
5097	152	1867	1870
5098	635	3632	1867
5099	873	1870	3632
5100	1867	3632	1870
5101	637	3637	3630
5102	873	3632	3637
5103	635	3630	3632
5104	3637	3632	3630
5105	34	1239	1241
5106	439	3070	1239
5107	441	1241	3070
5108	1239	3070	1241
5109	150	1861	1859
5110	869	3071	1861
5111	439	1859	3071
5112	1861	3071	1859
5113	210	2210	2212
5114	441	3075	2210
5115	869	2212	3075
5116	2210	3075	2212
5117	439	3071	3070
5118	869	3075	3071
5119	441	3070	3075
5120	3071	3075	3070
5121	152	1870	1868
5122	873	4005	1870
5123	868	1868	4005
5124	1870	4005	1868
5125	210	2212	2213
5126	869	4006	2212
5127	873	2213	4006
5128	2212	4006	2213
5129	150	1860	1861
5130	868	4004	1860
5131	869	1861	4004
5132	1860	4004	1861
5133	873	4006	4005
5134	869	4004	4006
5135	868	4005	4004
5136	4006	4004	4005
5137	17	1152	1154
5138	352	2834	1152
5139	354	1154	2834
5140	1152	2834	1154
5141	144	1824	1822
5142	850	2836	1824
5143	352	1822	2836
5144	1824	2836	1822
5145	146	1834	1836
5146	354	2843	1834

5147	850	1836	2843
5148	1834	2843	1836
5149	352	2836	2834
5150	850	2843	2836
5151	354	2834	2843
5152	2836	2843	2834
5153	33	1236	1233
5154	436	3054	1236
5155	433	1233	3054
5156	1236	3054	1233
5157	208	2200	2198
5158	852	3064	2200
5159	436	2198	3064
5160	2200	3064	2198
5161	144	1823	1826
5162	433	3056	1823
5163	852	1826	3056
5164	1823	3056	1826
5165	436	3064	3054
5166	852	3056	3064
5167	433	3054	3056
5168	3064	3056	3054
5169	146	1838	1835
5170	859	3628	1838
5171	634	1835	3628
5172	1838	3628	1835
5173	208	2199	2201
5174	636	3635	2199
5175	859	2201	3635
5176	2199	3635	2201
5177	144	1826	1824
5178	852	3986	1826
5179	850	1824	3986
5180	1826	3986	1824
5181	208	2201	2200
5182	859	3990	2201
5183	852	2200	3990
5184	2201	3990	2200
5185	146	1836	1838
5186	850	3987	1836
5187	859	1838	3987
5188	1836	3987	1838
5189	852	3990	3986
5190	859	3987	3990
5191	850	3986	3987
5192	3990	3987	3986
5193	34	1241	1240
5194	441	3072	1241
5195	440	1240	3072
5196	1241	3072	1240
5197	210	2214	2210
5198	1007	3076	2214
5199	441	2210	3076
5200	2214	3076	2210
5201	206	2187	2191
5202	440	3074	2187
5203	1007	2191	3074
5204	2187	3074	2191
5205	441	3076	3072

5206	1007	3074	3076
5207	440	3072	3074
5208	3076	3074	3072
5209	70	1436	1437
5210	636	3634	1436
5211	637	1437	3634
5212	1436	3634	1437
5213	208	2203	2199
5214	1010	3636	2203
5215	636	2199	3636
5216	2203	3636	2199
5217	210	2211	2215
5218	637	3638	2211
5219	1010	2215	3638
5220	2211	3638	2215
5221	636	3636	3634
5222	1010	3638	3636
5223	637	3634	3638
5224	3636	3638	3634
5225	33	1234	1236
5226	434	3058	1234
5227	436	1236	3058
5228	1234	3058	1236
5229	206	2190	2186
5230	1006	3060	2190
5231	434	2186	3060
5232	2190	3060	2186
5233	208	2198	2202
5234	436	3065	2198
5235	1006	2202	3065
5236	2198	3065	2202
5237	434	3060	3058
5238	1006	3065	3060
5239	436	3058	3065
5240	3060	3065	3058
5241	210	2215	2214
5242	1010	4144	2215
5243	1007	2214	4144
5244	2215	4144	2214
5245	208	2202	2203
5246	1006	4143	2202
5247	1010	2203	4143
5248	2202	4143	2203
5249	206	2191	2190
5250	1007	4142	2191
5251	1006	2190	4142
5252	2191	4142	2190
5253	1010	4143	4144
5254	1006	4142	4143
5255	1007	4144	4142
5256	4143	4142	4144
5257	8	1117	1115
5258	317	2746	1117
5259	315	1115	2746
5260	1117	2746	1115
5261	109	1642	1640
5262	762	2751	1642
5263	317	1640	2751
5264	1642	2751	1640

5265	107	1630	1632
5266	315	2747	1630
5267	762	1632	2747
5268	1630	2747	1632
5269	317	2751	2746
5270	762	2747	2751
5271	315	2746	2747
5272	2751	2747	2746
5273	53	1338	1336
5274	538	3338	1338
5275	536	1336	3338
5276	1338	3338	1336
5277	250	2453	2451
5278	767	3347	2453
5279	538	2451	3347
5280	2453	3347	2451
5281	109	1641	1644
5282	536	3340	1641
5283	767	1644	3340
5284	1641	3340	1644
5285	538	3347	3338
5286	767	3340	3347
5287	536	3338	3340
5288	3347	3340	3338
5289	51	1326	1328
5290	526	3311	1326
5291	528	1328	3311
5292	1326	3311	1328
5293	107	1633	1631
5294	763	3312	1633
5295	526	1631	3312
5296	1633	3312	1631
5297	250	2450	2452
5298	528	3316	2450
5299	763	2452	3316
5300	2450	3316	2452
5301	526	3312	3311
5302	763	3316	3312
5303	528	3311	3316
5304	3312	3316	3311
5305	109	1644	1642
5306	767	3900	1644
5307	762	1642	3900
5308	1644	3900	1642
5309	250	2452	2453
5310	763	3901	2452
5311	767	2453	3901
5312	2452	3901	2453
5313	107	1632	1633
5314	762	3899	1632
5315	763	1633	3899
5316	1632	3899	1633
5317	767	3901	3900
5318	763	3899	3901
5319	762	3900	3899
5320	3901	3899	3900
5321	185	2062	2060
5322	962	2953	2062
5323	393	2060	2953

5324	2062	2953	2060
5325	183	2048	2050
5326	391	2948	2048
5327	962	2050	2948
5328	2048	2948	2050
5329	393	2953	2946
5330	962	2948	2953
5331	391	2946	2948
5332	2953	2948	2946
5333	74	1461	1459
5334	661	3702	1461
5335	659	1459	3702
5336	1461	3702	1459
5337	255	2483	2481
5338	969	3709	2483
5339	661	2481	3709
5340	2483	3709	2481
5341	185	2061	2064
5342	659	3704	2061
5343	969	2064	3704
5344	2061	3704	2064
5345	661	3709	3702
5346	969	3704	3709
5347	659	3702	3704
5348	3709	3704	3702
5349	53	1337	1340
5350	537	3342	1337
5351	540	1340	3342
5352	1337	3342	1340
5353	183	2052	2049
5354	964	3344	2052
5355	537	2049	3344
5356	2052	3344	2049
5357	255	2480	2482
5358	540	3352	2480
5359	964	2482	3352
5360	2480	3352	2482
5361	537	3344	3342
5362	964	3352	3344
5363	540	3342	3352
5364	3344	3352	3342
5365	185	2064	2062
5366	969	4101	2064
5367	962	2062	4101
5368	2064	4101	2062
5369	255	2482	2483
5370	964	4104	2482
5371	969	2483	4104
5372	2482	4104	2483
5373	183	2050	2052
5374	962	4100	2050
5375	964	2052	4100
5376	2050	4100	2052
5377	969	4104	4101
5378	964	4100	4104
5379	962	4101	4100
5380	4104	4100	4101
5381	18	1159	1161
5382	359	2854	1159

5383	361	1161	2854
5384	1159	2854	1161
5385	151	1864	1862
5386	870	2855	1864
5387	359	1862	2855
5388	1864	2855	1862
5389	153	1872	1874
5390	361	2859	1872
5391	870	1874	2859
5392	1872	2859	1874
5393	359	2855	2854
5394	870	2859	2855
5395	361	2854	2859
5396	2855	2859	2854
5397	51	1329	1327
5398	529	3313	1329
5399	527	1327	3313
5400	1329	3313	1327
5401	251	2458	2456
5402	871	3318	2458
5403	529	2456	3318
5404	2458	3318	2456
5405	151	1863	1865
5406	527	3314	1863
5407	871	1865	3314
5408	1863	3314	1865
5409	529	3318	3313
5410	871	3314	3318
5411	527	3313	3314
5412	3318	3314	3313
5413	74	1458	1460
5414	658	3698	1458
5415	660	1460	3698
5416	1458	3698	1460
5417	153	1876	1873
5418	875	3700	1876
5419	658	1873	3700
5420	1876	3700	1873
5421	251	2457	2459
5422	660	3707	2457
5423	875	2459	3707
5424	2457	3707	2459
5425	658	3700	3698
5426	875	3707	3700
5427	660	3698	3707
5428	3700	3707	3698
5429	151	1865	1864
5430	871	4007	1865
5431	870	1864	4007
5432	1865	4007	1864
5433	251	2459	2458
5434	875	4009	2459
5435	871	2458	4009
5436	2459	4009	2458
5437	153	1874	1876
5438	870	4008	1874
5439	875	1876	4008
5440	1874	4008	1876
5441	871	4009	4007

5442	875	4008	4009
5443	870	4007	4008
5444	4009	4008	4007
5445	53	1340	1338
5446	540	3346	1340
5447	538	1338	3346
5448	1340	3346	1338
5449	255	2484	2480
5450	1052	3353	2484
5451	540	2480	3353
5452	2484	3353	2480
5453	250	2451	2455
5454	538	3348	2451
5455	1052	2455	3348
5456	2451	3348	2455
5457	540	3353	3346
5458	1052	3348	3353
5459	538	3346	3348
5460	3353	3348	3346
5461	74	1460	1461
5462	660	3706	1460
5463	661	1461	3706
5464	1460	3706	1461
5465	251	2461	2457
5466	1053	3708	2461
5467	660	2457	3708
5468	2461	3708	2457
5469	255	2481	2485
5470	661	3710	2481
5471	1053	2485	3710
5472	2481	3710	2485
5473	660	3708	3706
5474	1053	3710	3708
5475	661	3706	3710
5476	3708	3710	3706
5477	51	1328	1329
5478	528	3315	1328
5479	529	1329	3315
5480	1328	3315	1329
5481	250	2454	2450
5482	1051	3317	2454
5483	528	2450	3317
5484	2454	3317	2450
5485	251	2456	2460
5486	529	3319	2456
5487	1051	2460	3319
5488	2456	3319	2460
5489	528	3317	3315
5490	1051	3319	3317
5491	529	3315	3319
5492	3317	3319	3315
5493	255	2485	2484
5494	1053	4189	2485
5495	1052	2484	4189
5496	2485	4189	2484
5497	251	2460	2461
5498	1051	4188	2460
5499	1053	2461	4188
5500	2460	4188	2461

5501	250	2455	2454
5502	1052	4187	2455
5503	1051	2454	4187
5504	2455	4187	2454
5505	1053	4188	4189
5506	1051	4187	4188
5507	1052	4189	4187
5508	4188	4187	4189
5509	7	1109	1112
5510	309	2730	1109
5511	312	1112	2730
5512	1109	2730	1112
5513	101	1598	1596
5514	746	2732	1598
5515	309	1596	2732
5516	1598	2732	1596
5517	104	1614	1616
5518	312	2740	1614
5519	746	1616	2740
5520	1614	2740	1616
5521	309	2732	2730
5522	746	2740	2732
5523	312	2730	2740
5524	2732	2740	2730
5525	45	1294	1292
5526	494	3212	1294
5527	492	1292	3212
5528	1294	3212	1292
5529	234	2356	2354
5530	748	3221	2356
5531	494	2354	3221
5532	2356	3221	2354
5533	101	1597	1600
5534	492	3214	1597
5535	748	1600	3214
5536	1597	3214	1600
5537	494	3221	3212
5538	748	3214	3221
5539	492	3212	3214
5540	3221	3214	3212
5541	48	1310	1312
5542	510	3266	1310
5543	512	1312	3266
5544	1310	3266	1312
5545	104	1618	1615
5546	756	3268	1618
5547	510	1615	3268
5548	1618	3268	1615
5549	234	2355	2357
5550	512	3275	2355
5551	756	2357	3275
5552	2355	3275	2357
5553	510	3268	3266
5554	756	3275	3268
5555	512	3266	3275
5556	3268	3275	3266
5557	101	1600	1598
5558	748	3884	1600
5559	746	1598	3884

5560	1600	3884	1598
5561	234	2357	2356
5562	756	3888	2357
5563	748	2356	3888
5564	2357	3888	2356
5565	104	1616	1618
5566	746	3885	1616
5567	756	1618	3885
5568	1616	3885	1618
5569	748	3888	3884
5570	756	3885	3888
5571	746	3884	3885
5572	3888	3885	3884
5573	17	1156	1153
5574	356	2838	1156
5575	353	1153	2838
5576	1156	2838	1153
5577	148	1848	1846
5578	854	2848	1848
5579	356	1846	2848
5580	1848	2848	1846
5581	145	1828	1830
5582	353	2840	1828
5583	854	1830	2840
5584	1828	2840	1830
5585	356	2848	2838
5586	854	2840	2848
5587	353	2838	2840
5588	2848	2840	2838
5589	72	1448	1446
5590	648	3662	1448
5591	646	1446	3662
5592	1448	3662	1446
5593	236	2369	2367
5594	864	3671	2369
5595	648	2367	3671
5596	2369	3671	2367
5597	148	1847	1850
5598	646	3664	1847
5599	864	1850	3664
5600	1847	3664	1850
5601	648	3671	3662
5602	864	3664	3671
5603	646	3662	3664
5604	3671	3664	3662
5605	45	1293	1296
5606	493	3216	1293
5607	496	1296	3216
5608	1293	3216	1296
5609	145	1832	1829
5610	856	3218	1832
5611	493	1829	3218
5612	1832	3218	1829
5613	236	2366	2368
5614	496	3226	2366
5615	856	2368	3226
5616	2366	3226	2368
5617	493	3218	3216
5618	856	3226	3218

5619	496	3216	3226
5620	3218	3226	3216
5621	148	1850	1848
5622	864	3993	1850
5623	854	1848	3993
5624	1850	3993	1848
5625	236	2368	2369
5626	856	3996	2368
5627	864	2369	3996
5628	2368	3996	2369
5629	145	1830	1832
5630	854	3992	1830
5631	856	1832	3992
5632	1830	3992	1832
5633	864	3996	3993
5634	856	3992	3996
5635	854	3993	3992
5636	3996	3992	3993
5637	24	1190	1192
5638	390	2942	1190
5639	392	1192	2942
5640	1190	2942	1192
5641	182	2044	2042
5642	958	2944	2044
5643	390	2042	2944
5644	2044	2944	2042
5645	184	2054	2056
5646	392	2951	2054
5647	958	2056	2951
5648	2054	2951	2056
5649	390	2944	2942
5650	958	2951	2944
5651	392	2942	2951
5652	2944	2951	2942
5653	48	1314	1311
5654	514	3270	1314
5655	511	1311	3270
5656	1314	3270	1311
5657	244	2416	2414
5658	960	3280	2416
5659	514	2414	3280
5660	2416	3280	2414
5661	182	2043	2046
5662	511	3272	2043
5663	960	2046	3272
5664	2043	3272	2046
5665	514	3280	3270
5666	960	3272	3280
5667	511	3270	3272
5668	3280	3272	3270
5669	72	1447	1449
5670	647	3666	1447
5671	649	1449	3666
5672	1447	3666	1449
5673	184	2058	2055
5674	967	3668	2058
5675	647	2055	3668
5676	2058	3668	2055
5677	244	2415	2417

5678	649	3673	2415
5679	967	2417	3673
5680	2415	3673	2417
5681	647	3668	3666
5682	967	3673	3668
5683	649	3666	3673
5684	3668	3673	3666
5685	182	2046	2044
5686	960	4094	2046
5687	958	2044	4094
5688	2046	4094	2044
5689	244	2417	2416
5690	967	4098	2417
5691	960	2416	4098
5692	2417	4098	2416
5693	184	2056	2058
5694	958	4095	2056
5695	967	2058	4095
5696	2056	4095	2058
5697	960	4098	4094
5698	967	4095	4098
5699	958	4094	4095
5700	4098	4095	4094
5701	45	1296	1294
5702	496	3220	1296
5703	494	1294	3220
5704	1296	3220	1294
5705	236	2370	2366
5706	1036	3227	2370
5707	496	2366	3227
5708	2370	3227	2366
5709	234	2354	2358
5710	494	3222	2354
5711	1036	2358	3222
5712	2354	3222	2358
5713	496	3227	3220
5714	1036	3222	3227
5715	494	3220	3222
5716	3227	3222	3220
5717	72	1449	1448
5718	649	3670	1449
5719	648	1448	3670
5720	1449	3670	1448
5721	244	2419	2415
5722	1040	3674	2419
5723	649	2415	3674
5724	2419	3674	2415
5725	236	2367	2371
5726	648	3672	2367
5727	1040	2371	3672
5728	2367	3672	2371
5729	649	3674	3670
5730	1040	3672	3674
5731	648	3670	3672
5732	3674	3672	3670
5733	48	1312	1314
5734	512	3274	1312
5735	514	1314	3274
5736	1312	3274	1314

5737	234	2359	2355
5738	1037	3276	2359
5739	512	2355	3276
5740	2359	3276	2355
5741	244	2414	2418
5742	514	3281	2414
5743	1037	2418	3281
5744	2414	3281	2418
5745	512	3276	3274
5746	1037	3281	3276
5747	514	3274	3281
5748	3276	3281	3274
5749	236	2371	2370
5750	1040	4173	2371
5751	1036	2370	4173
5752	2371	4173	2370
5753	244	2418	2419
5754	1037	4174	2418
5755	1040	2419	4174
5756	2418	4174	2419
5757	234	2358	2359
5758	1036	4172	2358
5759	1037	2359	4172
5760	2358	4172	2359
5761	1040	4174	4173
5762	1037	4172	4174
5763	1036	4173	4172
5764	4174	4172	4173
5765	18	1161	1160
5766	361	2856	1161
5767	360	1160	2856
5768	1161	2856	1160
5769	153	1875	1872
5770	872	2860	1875
5771	361	1872	2860
5772	1875	2860	1872
5773	152	1866	1869
5774	360	2858	1866
5775	872	1869	2858
5776	1866	2858	1869
5777	361	2860	2856
5778	872	2858	2860
5779	360	2856	2858
5780	2860	2858	2856
5781	74	1462	1458
5782	662	3699	1462
5783	658	1458	3699
5784	1462	3699	1458
5785	279	2627	2625
5786	876	3712	2627
5787	662	2625	3712
5788	2627	3712	2625
5789	153	1873	1877
5790	658	3701	1873
5791	876	1877	3701
5792	1873	3701	1877
5793	662	3712	3699
5794	876	3701	3712
5795	658	3699	3701

5796	3712	3701	3699
5797	70	1435	1439
5798	635	3631	1435
5799	639	1439	3631
5800	1435	3631	1439
5801	152	1871	1867
5802	874	3633	1871
5803	635	1867	3633
5804	1871	3633	1867
5805	279	2624	2626
5806	639	3642	2624
5807	874	2626	3642
5808	2624	3642	2626
5809	635	3633	3631
5810	874	3642	3633
5811	639	3631	3642
5812	3633	3642	3631
5813	153	1877	1875
5814	876	4011	1877
5815	872	1875	4011
5816	1877	4011	1875
5817	279	2626	2627
5818	874	4012	2626
5819	876	2627	4012
5820	2626	4012	2627
5821	152	1869	1871
5822	872	4010	1869
5823	874	1871	4010
5824	1869	4010	1871
5825	876	4012	4011
5826	874	4010	4012
5827	872	4011	4010
5828	4012	4010	4011
5829	184	2057	2054
5830	966	2952	2057
5831	392	2054	2952
5832	2057	2952	2054
5833	185	2060	2063
5834	393	2954	2060
5835	966	2063	2954
5836	2060	2954	2063
5837	392	2952	2950
5838	966	2954	2952
5839	393	2950	2954
5840	2952	2954	2950
5841	72	1451	1447
5842	651	3667	1451
5843	647	1447	3667
5844	1451	3667	1447
5845	282	2644	2642
5846	968	3678	2644
5847	651	2642	3678
5848	2644	3678	2642
5849	184	2055	2059
5850	647	3669	2055
5851	968	2059	3669
5852	2055	3669	2059
5853	651	3678	3667
5854	968	3669	3678

5855	647	3667	3669
5856	3678	3669	3667
5857	74	1459	1463
5858	659	3703	1459
5859	663	1463	3703
5860	1459	3703	1463
5861	185	2065	2061
5862	970	3705	2065
5863	659	2061	3705
5864	2065	3705	2061
5865	282	2643	2645
5866	663	3714	2643
5867	970	2645	3714
5868	2643	3714	2645
5869	659	3705	3703
5870	970	3714	3705
5871	663	3703	3714
5872	3705	3714	3703
5873	184	2059	2057
5874	968	4106	2059
5875	966	2057	4106
5876	2059	4106	2057
5877	282	2645	2644
5878	970	4108	2645
5879	968	2644	4108
5880	2645	4108	2644
5881	185	2063	2065
5882	966	4107	2063
5883	970	2065	4107
5884	2063	4107	2065
5885	968	4108	4106
5886	970	4107	4108
5887	966	4106	4107
5888	4108	4107	4106
5889	17	1154	1156
5890	354	2842	1154
5891	356	1156	2842
5892	1154	2842	1156
5893	146	1837	1834
5894	858	2844	1837
5895	354	1834	2844
5896	1837	2844	1834
5897	148	1846	1849
5898	356	2849	1846
5899	858	1849	2849
5900	1846	2849	1849
5901	354	2844	2842
5902	858	2849	2844
5903	356	2842	2849
5904	2844	2849	2842
5905	70	1438	1434
5906	638	3627	1438
5907	634	1434	3627
5908	1438	3627	1434
5909	278	2620	2618
5910	860	3640	2620
5911	638	2618	3640
5912	2620	3640	2618
5913	146	1835	1839

5914	634	3629	1835
5915	860	1839	3629
5916	1835	3629	1839
5917	638	3640	3627
5918	860	3629	3640
5919	634	3627	3629
5920	3640	3629	3627
5921	146	1839	1837
5922	860	3998	1839
5923	858	1837	3998
5924	1839	3998	1837
5925	278	2621	2620
5926	865	4000	2621
5927	860	2620	4000
5928	2621	4000	2620
5929	148	1849	1851
5930	858	3999	1849
5931	865	1851	3999
5932	1849	3999	1851
5933	860	4000	3998
5934	865	3999	4000
5935	858	3998	3999
5936	4000	3999	3998
5937	74	1463	1462
5938	663	3711	1463
5939	662	1462	3711
5940	1463	3711	1462
5941	282	2647	2643
5942	1080	3715	2647
5943	663	2643	3715
5944	2647	3715	2643
5945	279	2625	2629
5946	662	3713	2625
5947	1080	2629	3713
5948	2625	3713	2629
5949	663	3715	3711
5950	1080	3713	3715
5951	662	3711	3713
5952	3715	3713	3711
5953	70	1439	1438
5954	639	3639	1439
5955	638	1438	3639
5956	1439	3639	1438
5957	279	2628	2624
5958	1078	3643	2628
5959	639	2624	3643
5960	2628	3643	2624
5961	278	2618	2622
5962	638	3641	2618
5963	1078	2622	3641
5964	2618	3641	2622
5965	639	3643	3639
5966	1078	3641	3643
5967	638	3639	3641
5968	3643	3641	3639
5969	282	2646	2647
5970	1079	4216	2646
5971	1080	2647	4216
5972	2646	4216	2647

5973	278	2622	2623
5974	1078	4214	2622
5975	1079	2623	4214
5976	2622	4214	2623
5977	279	2629	2628
5978	1080	4215	2629
5979	1078	2628	4215
5980	2629	4215	2628
5981	1079	4214	4216
5982	1078	4215	4214
5983	1080	4216	4215
5984	4214	4215	4216
5985	2	1093	1094
5986	293	2696	1093
5987	294	1094	2696
5988	1093	2696	1094
5989	85	1522	1520
5990	712	2697	1522
5991	293	1520	2697
5992	1522	2697	1520
5993	86	1524	1526
5994	294	2698	1524
5995	712	1526	2698
5996	1524	2698	1526
5997	293	2697	2696
5998	712	2698	2697
5999	294	2696	2698
6000	2697	2698	2696
6001	29	1218	1216
6002	418	3014	1218
6003	416	1216	3014
6004	1218	3014	1216
6005	200	2152	2150
6006	713	3019	2152
6007	418	2150	3019
6008	2152	3019	2150
6009	85	1521	1523
6010	416	3015	1521
6011	713	1523	3015
6012	1521	3015	1523
6013	418	3019	3014
6014	713	3015	3019
6015	416	3014	3015
6016	3019	3015	3014
6017	30	1220	1222
6018	420	3023	1220
6019	422	1222	3023
6020	1220	3023	1222
6021	86	1527	1525
6022	714	3024	1527
6023	420	1525	3024
6024	1527	3024	1525
6025	200	2151	2153
6026	422	3028	2151
6027	714	2153	3028
6028	2151	3028	2153
6029	420	3024	3023
6030	714	3028	3024
6031	422	3023	3028

6032	3024	3028	3023
6033	85	1523	1522
6034	713	3848	1523
6035	712	1522	3848
6036	1523	3848	1522
6037	200	2153	2152
6038	714	3850	2153
6039	713	2152	3850
6040	2153	3850	2152
6041	86	1526	1527
6042	712	3849	1526
6043	714	1527	3849
6044	1526	3849	1527
6045	713	3850	3848
6046	714	3849	3850
6047	712	3848	3849
6048	3850	3849	3848
6049	13	1138	1136
6050	338	2798	1138
6051	336	1136	2798
6052	1138	2798	1136
6053	130	1752	1750
6054	814	2803	1752
6055	338	1750	2803
6056	1752	2803	1750
6057	128	1742	1744
6058	336	2799	1742
6059	814	1744	2799
6060	1742	2799	1744
6061	338	2803	2798
6062	814	2799	2803
6063	336	2798	2799
6064	2803	2799	2798
6065	64	1400	1398
6066	600	3518	1400
6067	598	1398	3518
6068	1400	3518	1398
6069	201	2159	2157
6070	819	3527	2159
6071	600	2157	3527
6072	2159	3527	2157
6073	130	1751	1754
6074	598	3520	1751
6075	819	1754	3520
6076	1751	3520	1754
6077	600	3527	3518
6078	819	3520	3527
6079	598	3518	3520
6080	3527	3520	3518
6081	29	1217	1219
6082	417	3016	1217
6083	419	1219	3016
6084	1217	3016	1219
6085	128	1745	1743
6086	815	3017	1745
6087	417	1743	3017
6088	1745	3017	1743
6089	201	2156	2158
6090	419	3021	2156

6091	815	2158	3021
6092	2156	3021	2158
6093	417	3017	3016
6094	815	3021	3017
6095	419	3016	3021
6096	3017	3021	3016
6097	130	1754	1752
6098	819	3951	1754
6099	814	1752	3951
6100	1754	3951	1752
6101	201	2158	2159
6102	815	3952	2158
6103	819	2159	3952
6104	2158	3952	2159
6105	128	1744	1745
6106	814	3950	1744
6107	815	1745	3950
6108	1744	3950	1745
6109	819	3952	3951
6110	815	3950	3952
6111	814	3951	3950
6112	3952	3950	3951
6113	14	1140	1142
6114	340	2807	1140
6115	342	1142	2807
6116	1140	2807	1142
6117	132	1764	1762
6118	823	2808	1764
6119	340	1762	2808
6120	1764	2808	1762
6121	134	1770	1772
6122	342	2812	1770
6123	823	1772	2812
6124	1770	2812	1772
6125	340	2808	2807
6126	823	2812	2808
6127	342	2807	2812
6128	2808	2812	2807
6129	30	1223	1221
6130	423	3025	1223
6131	421	1221	3025
6132	1223	3025	1221
6133	202	2164	2162
6134	824	3030	2164
6135	423	2162	3030
6136	2164	3030	2162
6137	132	1763	1765
6138	421	3026	1763
6139	824	1765	3026
6140	1763	3026	1765
6141	423	3030	3025
6142	824	3026	3030
6143	421	3025	3026
6144	3030	3026	3025
6145	64	1399	1401
6146	599	3522	1399
6147	601	1401	3522
6148	1399	3522	1401
6149	134	1774	1771

6150	828	3524	1774
6151	599	1771	3524
6152	1774	3524	1771
6153	202	2163	2165
6154	601	3529	2163
6155	828	2165	3529
6156	2163	3529	2165
6157	599	3524	3522
6158	828	3529	3524
6159	601	3522	3529
6160	3524	3529	3522
6161	132	1765	1764
6162	824	3959	1765
6163	823	1764	3959
6164	1765	3959	1764
6165	202	2165	2164
6166	828	3961	2165
6167	824	2164	3961
6168	2165	3961	2164
6169	134	1772	1774
6170	823	3960	1772
6171	828	1774	3960
6172	1772	3960	1774
6173	824	3961	3959
6174	828	3960	3961
6175	823	3959	3960
6176	3961	3960	3959
6177	29	1219	1218
6178	419	3018	1219
6179	418	1218	3018
6180	1219	3018	1218
6181	201	2160	2156
6182	1000	3022	2160
6183	419	2156	3022
6184	2160	3022	2156
6185	200	2150	2154
6186	418	3020	2150
6187	1000	2154	3020
6188	2150	3020	2154
6189	419	3022	3018
6190	1000	3020	3022
6191	418	3018	3020
6192	3022	3020	3018
6193	64	1401	1400
6194	601	3526	1401
6195	600	1400	3526
6196	1401	3526	1400
6197	202	2167	2163
6198	1002	3530	2167
6199	601	2163	3530
6200	2167	3530	2163
6201	201	2157	2161
6202	600	3528	2157
6203	1002	2161	3528
6204	2157	3528	2161
6205	601	3530	3526
6206	1002	3528	3530
6207	600	3526	3528
6208	3530	3528	3526

6209	30	1222	1223
6210	422	3027	1222
6211	423	1223	3027
6212	1222	3027	1223
6213	200	2155	2151
6214	1001	3029	2155
6215	422	2151	3029
6216	2155	3029	2151
6217	202	2162	2166
6218	423	3031	2162
6219	1001	2166	3031
6220	2162	3031	2166
6221	422	3029	3027
6222	1001	3031	3029
6223	423	3027	3031
6224	3029	3031	3027
6225	201	2161	2160
6226	1002	4137	2161
6227	1000	2160	4137
6228	2161	4137	2160
6229	202	2166	2167
6230	1001	4138	2166
6231	1002	2167	4138
6232	2166	4138	2167
6233	200	2154	2155
6234	1000	4136	2154
6235	1001	2155	4136
6236	2154	4136	2155
6237	1002	4138	4137
6238	1001	4136	4138
6239	1000	4137	4136
6240	4138	4136	4137
6241	5	1103	1101
6242	303	2710	1103
6243	301	1101	2710
6244	1103	2710	1101
6245	95	1566	1564
6246	726	2715	1566
6247	303	1564	2715
6248	1566	2715	1564
6249	93	1554	1556
6250	301	2711	1554
6251	726	1556	2711
6252	1554	2711	1556
6253	303	2715	2710
6254	726	2711	2715
6255	301	2710	2711
6256	2715	2711	2710
6257	39	1262	1260
6258	462	3122	1262
6259	460	1260	3122
6260	1262	3122	1260
6261	214	2237	2235
6262	731	3131	2237
6263	462	2235	3131
6264	2237	3131	2235
6265	95	1565	1568
6266	460	3124	1565
6267	731	1568	3124

6268	1565	3124	1568
6269	462	3131	3122
6270	731	3124	3131
6271	460	3122	3124
6272	3131	3124	3122
6273	37	1250	1252
6274	450	3095	1250
6275	452	1252	3095
6276	1250	3095	1252
6277	93	1557	1555
6278	727	3096	1557
6279	450	1555	3096
6280	1557	3096	1555
6281	214	2234	2236
6282	452	3100	2234
6283	727	2236	3100
6284	2234	3100	2236
6285	450	3096	3095
6286	727	3100	3096
6287	452	3095	3100
6288	3096	3100	3095
6289	95	1568	1566
6290	731	3864	1568
6291	726	1566	3864
6292	1568	3864	1566
6293	214	2236	2237
6294	727	3865	2236
6295	731	2237	3865
6296	2236	3865	2237
6297	93	1556	1557
6298	726	3863	1556
6299	727	1557	3863
6300	1556	3863	1557
6301	731	3865	3864
6302	727	3863	3865
6303	726	3864	3863
6304	3865	3863	3864
6305	21	1174	1172
6306	374	2888	1174
6307	372	1172	2888
6308	1174	2888	1172
6309	166	1948	1946
6310	904	2897	1948
6311	374	1946	2897
6312	1948	2897	1946
6313	164	1934	1936
6314	372	2890	1934
6315	904	1936	2890
6316	1934	2890	1936
6317	374	2897	2888
6318	904	2890	2897
6319	372	2888	2890
6320	2897	2890	2888
6321	65	1407	1405
6322	607	3540	1407
6323	605	1405	3540
6324	1407	3540	1405
6325	219	2267	2265
6326	913	3547	2267

6327	607	2265	3547
6328	2267	3547	2265
6329	166	1947	1950
6330	605	3542	1947
6331	913	1950	3542
6332	1947	3542	1950
6333	607	3547	3540
6334	913	3542	3547
6335	605	3540	3542
6336	3547	3542	3540
6337	39	1261	1264
6338	461	3126	1261
6339	464	1264	3126
6340	1261	3126	1264
6341	164	1938	1935
6342	906	3128	1938
6343	461	1935	3128
6344	1938	3128	1935
6345	219	2264	2266
6346	464	3136	2264
6347	906	2266	3136
6348	2264	3136	2266
6349	461	3128	3126
6350	906	3136	3128
6351	464	3126	3136
6352	3128	3136	3126
6353	166	1950	1948
6354	913	4041	1950
6355	904	1948	4041
6356	1950	4041	1948
6357	219	2266	2267
6358	906	4044	2266
6359	913	2267	4044
6360	2266	4044	2267
6361	164	1936	1938
6362	904	4040	1936
6363	906	1938	4040
6364	1936	4040	1938
6365	913	4044	4041
6366	906	4040	4044
6367	904	4041	4040
6368	4044	4040	4041
6369	13	1137	1139
6370	337	2800	1137
6371	339	1139	2800
6372	1137	2800	1139
6373	129	1748	1746
6374	816	2801	1748
6375	337	1746	2801
6376	1748	2801	1746
6377	131	1756	1758
6378	339	2805	1756
6379	816	1758	2805
6380	1756	2805	1758
6381	337	2801	2800
6382	816	2805	2801
6383	339	2800	2805
6384	2801	2805	2800
6385	37	1253	1251

6386	453	3097	1253
6387	451	1251	3097
6388	1253	3097	1251
6389	215	2242	2240
6390	817	3102	2242
6391	453	2240	3102
6392	2242	3102	2240
6393	129	1747	1749
6394	451	3098	1747
6395	817	1749	3098
6396	1747	3098	1749
6397	453	3102	3097
6398	817	3098	3102
6399	451	3097	3098
6400	3102	3098	3097
6401	131	1760	1757
6402	821	3538	1760
6403	604	1757	3538
6404	1760	3538	1757
6405	215	2241	2243
6406	606	3545	2241
6407	821	2243	3545
6408	2241	3545	2243
6409	604	3538	3536
6410	821	3545	3538
6411	606	3536	3545
6412	3538	3545	3536
6413	129	1749	1748
6414	817	3953	1749
6415	816	1748	3953
6416	1749	3953	1748
6417	215	2243	2242
6418	821	3955	2243
6419	817	2242	3955
6420	2243	3955	2242
6421	131	1758	1760
6422	816	3954	1758
6423	821	1760	3954
6424	1758	3954	1760
6425	817	3955	3953
6426	821	3954	3955
6427	816	3953	3954
6428	3955	3954	3953
6429	39	1264	1262
6430	464	3130	1264
6431	462	1262	3130
6432	1264	3130	1262
6433	219	2268	2264
6434	1016	3137	2268
6435	464	2264	3137
6436	2268	3137	2264
6437	214	2235	2239
6438	462	3132	2235
6439	1016	2239	3132
6440	2235	3132	2239
6441	464	3137	3130
6442	1016	3132	3137
6443	462	3130	3132
6444	3137	3132	3130

6445	215	2245	2241
6446	1017	3546	2245
6447	606	2241	3546
6448	2245	3546	2241
6449	219	2265	2269
6450	607	3548	2265
6451	1017	2269	3548
6452	2265	3548	2269
6453	606	3546	3544
6454	1017	3548	3546
6455	607	3544	3548
6456	3546	3548	3544
6457	37	1252	1253
6458	452	3099	1252
6459	453	1253	3099
6460	1252	3099	1253
6461	214	2238	2234
6462	1015	3101	2238
6463	452	2234	3101
6464	2238	3101	2234
6465	215	2240	2244
6466	453	3103	2240
6467	1015	2244	3103
6468	2240	3103	2244
6469	452	3101	3099
6470	1015	3103	3101
6471	453	3099	3103
6472	3101	3103	3099
6473	219	2269	2268
6474	1017	4153	2269
6475	1016	2268	4153
6476	2269	4153	2268
6477	215	2244	2245
6478	1015	4152	2244
6479	1017	2245	4152
6480	2244	4152	2245
6481	214	2239	2238
6482	1016	4151	2239
6483	1015	2238	4151
6484	2239	4151	2238
6485	1017	4152	4153
6486	1015	4151	4152
6487	1016	4153	4151
6488	4152	4151	4153
6489	8	1114	1116
6490	314	2744	1114
6491	316	1116	2744
6492	1114	2744	1116
6493	106	1628	1626
6494	760	2745	1628
6495	314	1626	2745
6496	1628	2745	1626
6497	108	1634	1636
6498	316	2749	1634
6499	760	1636	2749
6500	1634	2749	1636
6501	314	2745	2744
6502	760	2749	2745
6503	316	2744	2749

6504	2745	2749	2744
6505	50	1324	1322
6506	524	3302	1324
6507	522	1322	3302
6508	1324	3302	1322
6509	248	2440	2438
6510	761	3307	2440
6511	524	2438	3307
6512	2440	3307	2438
6513	106	1627	1629
6514	522	3303	1627
6515	761	1629	3303
6516	1627	3303	1629
6517	524	3307	3302
6518	761	3303	3307
6519	522	3302	3303
6520	3307	3303	3302
6521	52	1330	1332
6522	530	3320	1330
6523	532	1332	3320
6524	1330	3320	1332
6525	108	1638	1635
6526	765	3322	1638
6527	530	1635	3322
6528	1638	3322	1635
6529	248	2439	2441
6530	532	3329	2439
6531	765	2441	3329
6532	2439	3329	2441
6533	530	3322	3320
6534	765	3329	3322
6535	532	3320	3329
6536	3322	3329	3320
6537	106	1629	1628
6538	761	3896	1629
6539	760	1628	3896
6540	1629	3896	1628
6541	248	2441	2440
6542	765	3898	2441
6543	761	2440	3898
6544	2441	3898	2440
6545	108	1636	1638
6546	760	3897	1636
6547	765	1638	3897
6548	1636	3897	1638
6549	761	3898	3896
6550	765	3897	3898
6551	760	3896	3897
6552	3898	3897	3896
6553	14	1143	1141
6554	343	2809	1143
6555	341	1141	2809
6556	1143	2809	1141
6557	135	1778	1776
6558	825	2814	1778
6559	343	1776	2814
6560	1778	2814	1776
6561	133	1766	1768
6562	341	2810	1766

6563	825	1768	2810
6564	1766	2810	1768
6565	343	2814	2809
6566	825	2810	2814
6567	341	2809	2810
6568	2814	2810	2809
6569	66	1412	1410
6570	612	3554	1412
6571	610	1410	3554
6572	1412	3554	1410
6573	249	2447	2445
6574	830	3563	2447
6575	612	2445	3563
6576	2447	3563	2445
6577	135	1777	1780
6578	610	3556	1777
6579	830	1780	3556
6580	1777	3556	1780
6581	612	3563	3554
6582	830	3556	3563
6583	610	3554	3556
6584	3563	3556	3554
6585	50	1323	1325
6586	523	3304	1323
6587	525	1325	3304
6588	1323	3304	1325
6589	133	1769	1767
6590	826	3305	1769
6591	523	1767	3305
6592	1769	3305	1767
6593	249	2444	2446
6594	525	3309	2444
6595	826	2446	3309
6596	2444	3309	2446
6597	523	3305	3304
6598	826	3309	3305
6599	525	3304	3309
6600	3305	3309	3304
6601	135	1780	1778
6602	830	3963	1780
6603	825	1778	3963
6604	1780	3963	1778
6605	249	2446	2447
6606	826	3964	2446
6607	830	2447	3964
6608	2446	3964	2447
6609	133	1768	1769
6610	825	3962	1768
6611	826	1769	3962
6612	1768	3962	1769
6613	830	3964	3963
6614	826	3962	3964
6615	825	3963	3962
6616	3964	3962	3963
6617	165	1942	1940
6618	908	2894	1942
6619	373	1940	2894
6620	1942	2894	1940
6621	253	2470	2468

6622	910	3334	2470
6623	534	2468	3334
6624	2470	3334	2468
6625	66	1411	1413
6626	611	3558	1411
6627	613	1413	3558
6628	1411	3558	1413
6629	167	1956	1953
6630	915	3560	1956
6631	611	1953	3560
6632	1956	3560	1953
6633	253	2469	2471
6634	613	3565	2469
6635	915	2471	3565
6636	2469	3565	2471
6637	611	3560	3558
6638	915	3565	3560
6639	613	3558	3565
6640	3560	3565	3558
6641	165	1944	1942
6642	910	4046	1944
6643	908	1942	4046
6644	1944	4046	1942
6645	253	2471	2470
6646	915	4050	2471
6647	910	2470	4050
6648	2471	4050	2470
6649	167	1954	1956
6650	908	4047	1954
6651	915	1956	4047
6652	1954	4047	1956
6653	910	4050	4046
6654	915	4047	4050
6655	908	4046	4047
6656	4050	4047	4046
6657	50	1325	1324
6658	525	3306	1325
6659	524	1324	3306
6660	1325	3306	1324
6661	249	2448	2444
6662	1048	3310	2448
6663	525	2444	3310
6664	2448	3310	2444
6665	248	2438	2442
6666	524	3308	2438
6667	1048	2442	3308
6668	2438	3308	2442
6669	525	3310	3306
6670	1048	3308	3310
6671	524	3306	3308
6672	3310	3308	3306
6673	66	1413	1412
6674	613	3562	1413
6675	612	1412	3562
6676	1413	3562	1412
6677	253	2473	2469
6678	1050	3566	2473
6679	613	2469	3566
6680	2473	3566	2469

6681	249	2445	2449
6682	612	3564	2445
6683	1050	2449	3564
6684	2445	3564	2449
6685	613	3566	3562
6686	1050	3564	3566
6687	612	3562	3564
6688	3566	3564	3562
6689	52	1332	1334
6690	532	3328	1332
6691	534	1334	3328
6692	1332	3328	1334
6693	248	2443	2439
6694	1049	3330	2443
6695	532	2439	3330
6696	2443	3330	2439
6697	253	2468	2472
6698	534	3335	2468
6699	1049	2472	3335
6700	2468	3335	2472
6701	532	3330	3328
6702	1049	3335	3330
6703	534	3328	3335
6704	3330	3335	3328
6705	249	2449	2448
6706	1050	4185	2449
6707	1048	2448	4185
6708	2449	4185	2448
6709	253	2472	2473
6710	1049	4186	2472
6711	1050	2473	4186
6712	2472	4186	2473
6713	248	2442	2443
6714	1048	4184	2442
6715	1049	2443	4184
6716	2442	4184	2443
6717	1050	4186	4185
6718	1049	4184	4186
6719	1048	4185	4184
6720	4186	4184	4185
6721	13	1139	1138
6722	339	2802	1139
6723	338	1138	2802
6724	1139	2802	1138
6725	131	1759	1756
6726	818	2806	1759
6727	339	1756	2806
6728	1759	2806	1756
6729	130	1750	1753
6730	338	2804	1750
6731	818	1753	2804
6732	1750	2804	1753
6733	339	2806	2802
6734	818	2804	2806
6735	338	2802	2804
6736	2806	2804	2802
6737	65	1408	1404
6738	608	3537	1408
6739	604	1404	3537

6740	1408	3537	1404
6741	272	2585	2583
6742	822	3550	2585
6743	608	2583	3550
6744	2585	3550	2583
6745	131	1757	1761
6746	604	3539	1757
6747	822	1761	3539
6748	1757	3539	1761
6749	608	3550	3537
6750	822	3539	3550
6751	604	3537	3539
6752	3550	3539	3537
6753	64	1398	1402
6754	598	3519	1398
6755	602	1402	3519
6756	1398	3519	1402
6757	130	1755	1751
6758	820	3521	1755
6759	598	1751	3521
6760	1755	3521	1751
6761	272	2582	2584
6762	602	3532	2582
6763	820	2584	3532
6764	2582	3532	2584
6765	598	3521	3519
6766	820	3532	3521
6767	602	3519	3532
6768	3521	3532	3519
6769	131	1761	1759
6770	822	3957	1761
6771	818	1759	3957
6772	1761	3957	1759
6773	272	2584	2585
6774	820	3958	2584
6775	822	2585	3958
6776	2584	3958	2585
6777	130	1753	1755
6778	818	3956	1753
6779	820	1755	3956
6780	1753	3956	1755
6781	822	3958	3957
6782	820	3956	3958
6783	818	3957	3956
6784	3958	3956	3957
6785	166	1946	1949
6786	374	2898	1946
6787	912	1949	2898
6788	1946	2898	1949
6789	66	1415	1411
6790	615	3559	1415
6791	611	1411	3559
6792	1415	3559	1411
6793	274	2597	2595
6794	916	3570	2597
6795	615	2595	3570
6796	2597	3570	2595
6797	167	1953	1957
6798	611	3561	1953

6799	916	1957	3561
6800	1953	3561	1957
6801	615	3570	3559
6802	916	3561	3570
6803	611	3559	3561
6804	3570	3561	3559
6805	65	1405	1409
6806	605	3541	1405
6807	609	1409	3541
6808	1405	3541	1409
6809	166	1951	1947
6810	914	3543	1951
6811	605	1947	3543
6812	1951	3543	1947
6813	274	2594	2596
6814	609	3552	2594
6815	914	2596	3552
6816	2594	3552	2596
6817	605	3543	3541
6818	914	3552	3543
6819	609	3541	3552
6820	3543	3552	3541
6821	167	1957	1955
6822	916	4053	1957
6823	912	1955	4053
6824	1957	4053	1955
6825	274	2596	2597
6826	914	4054	2596
6827	916	2597	4054
6828	2596	4054	2597
6829	166	1949	1951
6830	912	4052	1949
6831	914	1951	4052
6832	1949	4052	1951
6833	916	4054	4053
6834	914	4052	4054
6835	912	4053	4052
6836	4054	4052	4053
6837	14	1142	1143
6838	342	2811	1142
6839	343	1143	2811
6840	1142	2811	1143
6841	134	1773	1770
6842	827	2813	1773
6843	342	1770	2813
6844	1773	2813	1770
6845	135	1776	1779
6846	343	2815	1776
6847	827	1779	2815
6848	1776	2815	1779
6849	342	2813	2811
6850	827	2815	2813
6851	343	2811	2815
6852	2813	2815	2811
6853	64	1403	1399
6854	603	3523	1403
6855	599	1399	3523
6856	1403	3523	1399
6857	273	2590	2588

6858	829	3534	2590
6859	603	2588	3534
6860	2590	3534	2588
6861	134	1771	1775
6862	599	3525	1771
6863	829	1775	3525
6864	1771	3525	1775
6865	603	3534	3523
6866	829	3525	3534
6867	599	3523	3525
6868	3534	3525	3523
6869	66	1410	1414
6870	610	3555	1410
6871	614	1414	3555
6872	1410	3555	1414
6873	135	1781	1777
6874	831	3557	1781
6875	610	1777	3557
6876	1781	3557	1777
6877	273	2589	2591
6878	614	3568	2589
6879	831	2591	3568
6880	2589	3568	2591
6881	610	3557	3555
6882	831	3568	3557
6883	614	3555	3568
6884	3557	3568	3555
6885	134	1775	1773
6886	829	3965	1775
6887	827	1773	3965
6888	1775	3965	1773
6889	273	2591	2590
6890	831	3967	2591
6891	829	2590	3967
6892	2591	3967	2590
6893	135	1779	1781
6894	827	3966	1779
6895	831	1781	3966
6896	1779	3966	1781
6897	829	3967	3965
6898	831	3966	3967
6899	827	3965	3966
6900	3967	3966	3965
6901	65	1409	1408
6902	609	3549	1409
6903	608	1408	3549
6904	1409	3549	1408
6905	274	2598	2594
6906	1073	3553	2598
6907	609	2594	3553
6908	2598	3553	2594
6909	272	2583	2587
6910	608	3551	2583
6911	1073	2587	3551
6912	2583	3551	2587
6913	609	3553	3549
6914	1073	3551	3553
6915	608	3549	3551
6916	3553	3551	3549

6917	66	1414	1415
6918	614	3567	1414
6919	615	1415	3567
6920	1414	3567	1415
6921	273	2593	2589
6922	1074	3569	2593
6923	614	2589	3569
6924	2593	3569	2589
6925	274	2595	2599
6926	615	3571	2595
6927	1074	2599	3571
6928	2595	3571	2599
6929	614	3569	3567
6930	1074	3571	3569
6931	615	3567	3571
6932	3569	3571	3567
6933	64	1402	1403
6934	602	3531	1402
6935	603	1403	3531
6936	1402	3531	1403
6937	272	2586	2582
6938	1072	3533	2586
6939	602	2582	3533
6940	2586	3533	2582
6941	273	2588	2592
6942	603	3535	2588
6943	1072	2592	3535
6944	2588	3535	2592
6945	602	3533	3531
6946	1072	3535	3533
6947	603	3531	3535
6948	3533	3535	3531
6949	274	2599	2598
6950	1074	4210	2599
6951	1073	2598	4210
6952	2599	4210	2598
6953	273	2592	2593
6954	1072	4209	2592
6955	1074	2593	4209
6956	2592	4209	2593
6957	272	2587	2586
6958	1073	4208	2587
6959	1072	2586	4208
6960	2587	4208	2586
6961	1074	4209	4210
6962	1072	4208	4209
6963	1073	4210	4208
6964	4209	4208	4210
6965	7	1112	1110
6966	312	2734	1112
6967	310	1110	2734
6968	1112	2734	1110
6969	104	1617	1614
6970	750	2741	1617
6971	312	1614	2741
6972	1617	2741	1614
6973	102	1602	1605
6974	310	2736	1602
6975	750	1605	2736

6976	1602	2736	1605
6977	312	2741	2734
6978	750	2736	2741
6979	310	2734	2736
6980	2741	2736	2734
6981	48	1313	1310
6982	513	3267	1313
6983	510	1310	3267
6984	1313	3267	1310
6985	238	2381	2379
6986	757	3278	2381
6987	513	2379	3278
6988	2381	3278	2379
6989	104	1615	1619
6990	510	3269	1615
6991	757	1619	3269
6992	1615	3269	1619
6993	513	3278	3267
6994	757	3269	3278
6995	510	3267	3269
6996	3278	3269	3267
6997	46	1298	1301
6998	498	3231	1298
6999	501	1301	3231
7000	1298	3231	1301
7001	102	1607	1603
7002	752	3233	1607
7003	498	1603	3233
7004	1607	3233	1603
7005	238	2378	2380
7006	501	3242	2378
7007	752	2380	3242
7008	2378	3242	2380
7009	498	3233	3231
7010	752	3242	3233
7011	501	3231	3242
7012	3233	3242	3231
7013	104	1619	1617
7014	757	3891	1619
7015	750	1617	3891
7016	1619	3891	1617
7017	238	2380	2381
7018	752	3892	2380
7019	757	2381	3892
7020	2380	3892	2381
7021	102	1605	1607
7022	750	3890	1605
7023	752	1607	3890
7024	1605	3890	1607
7025	757	3892	3891
7026	752	3890	3892
7027	750	3891	3890
7028	3892	3890	3891
7029	24	1194	1190
7030	394	2943	1194
7031	390	1190	2943
7032	1194	2943	1190
7033	186	2068	2066
7034	959	2956	2068

7035	394	2066	2956
7036	2068	2956	2066
7037	182	2042	2045
7038	390	2945	2042
7039	959	2045	2945
7040	2042	2945	2045
7041	394	2956	2943
7042	959	2945	2956
7043	390	2943	2945
7044	2956	2945	2943
7045	77	1479	1477
7046	679	3756	1479
7047	677	1477	3756
7048	1479	3756	1477
7049	245	2423	2421
7050	972	3763	2423
7051	679	2421	3763
7052	2423	3763	2421
7053	186	2067	2070
7054	677	3758	2067
7055	972	2070	3758
7056	2067	3758	2070
7057	679	3763	3756
7058	972	3758	3763
7059	677	3756	3758
7060	3763	3758	3756
7061	182	2047	2043
7062	961	3273	2047
7063	511	2043	3273
7064	2047	3273	2043
7065	245	2420	2422
7066	515	3282	2420
7067	961	2422	3282
7068	2420	3282	2422
7069	186	2070	2068
7070	972	4097	2070
7071	959	2068	4097
7072	2070	4097	2068
7073	245	2422	2423
7074	961	4099	2422
7075	972	2423	4099
7076	2422	4099	2423
7077	182	2045	2047
7078	959	4096	2045
7079	961	2047	4096
7080	2045	4096	2047
7081	972	4099	4097
7082	961	4096	4099
7083	959	4097	4096
7084	4099	4096	4097
7085	20	1167	1171
7086	367	2875	1167
7087	371	1171	2875
7088	1167	2875	1171
7089	159	1907	1904
7090	891	2877	1907
7091	367	1904	2877
7092	1907	2877	1904
7093	163	1928	1930

7094	371	2886	1928
7095	891	1930	2886
7096	1928	2886	1930
7097	367	2877	2875
7098	891	2886	2877
7099	371	2875	2886
7100	2877	2886	2875
7101	46	1303	1299
7102	503	3235	1303
7103	499	1299	3235
7104	1303	3235	1299
7105	240	2392	2390
7106	893	3246	2392
7107	503	2390	3246
7108	2392	3246	2390
7109	159	1905	1909
7110	499	3237	1905
7111	893	1909	3237
7112	1905	3237	1909
7113	503	3246	3235
7114	893	3237	3246
7115	499	3235	3237
7116	3246	3237	3235
7117	77	1476	1478
7118	676	3752	1476
7119	678	1478	3752
7120	1476	3752	1478
7121	163	1932	1929
7122	902	3754	1932
7123	676	1929	3754
7124	1932	3754	1929
7125	240	2391	2393
7126	678	3761	2391
7127	902	2393	3761
7128	2391	3761	2393
7129	676	3754	3752
7130	902	3761	3754
7131	678	3752	3761
7132	3754	3761	3752
7133	159	1909	1907
7134	893	4030	1909
7135	891	1907	4030
7136	1909	4030	1907
7137	240	2393	2392
7138	902	4033	2393
7139	893	2392	4033
7140	2393	4033	2392
7141	163	1930	1932
7142	891	4031	1930
7143	902	1932	4031
7144	1930	4031	1932
7145	893	4033	4030
7146	902	4031	4033
7147	891	4030	4031
7148	4033	4031	4030
7149	48	1315	1313
7150	515	3277	1315
7151	513	1313	3277
7152	1315	3277	1313

7153	245	2424	2420
7154	1043	3283	2424
7155	515	2420	3283
7156	2424	3283	2420
7157	238	2379	2383
7158	513	3279	2379
7159	1043	2383	3279
7160	2379	3279	2383
7161	515	3283	3277
7162	1043	3279	3283
7163	513	3277	3279
7164	3283	3279	3277
7165	77	1478	1479
7166	678	3760	1478
7167	679	1479	3760
7168	1478	3760	1479
7169	240	2395	2391
7170	1044	3762	2395
7171	678	2391	3762
7172	2395	3762	2391
7173	245	2421	2425
7174	679	3764	2421
7175	1044	2425	3764
7176	2421	3764	2425
7177	678	3762	3760
7178	1044	3764	3762
7179	679	3760	3764
7180	3762	3764	3760
7181	46	1301	1303
7182	501	3241	1301
7183	503	1303	3241
7184	1301	3241	1303
7185	238	2382	2378
7186	1042	3243	2382
7187	501	2378	3243
7188	2382	3243	2378
7189	240	2390	2394
7190	503	3247	2390
7191	1042	2394	3247
7192	2390	3247	2394
7193	501	3243	3241
7194	1042	3247	3243
7195	503	3241	3247
7196	3243	3247	3241
7197	245	2425	2424
7198	1044	4180	2425
7199	1043	2424	4180
7200	2425	4180	2424
7201	240	2394	2395
7202	1042	4179	2394
7203	1044	2395	4179
7204	2394	4179	2395
7205	238	2383	2382
7206	1043	4178	2383
7207	1042	2382	4178
7208	2383	4178	2382
7209	1044	4179	4180
7210	1042	4178	4179
7211	1043	4180	4178

7212	4179	4178	4180
7213	8	1116	1117
7214	316	2748	1116
7215	317	1117	2748
7216	1116	2748	1117
7217	108	1637	1634
7218	764	2750	1637
7219	316	1634	2750
7220	1637	2750	1634
7221	109	1640	1643
7222	317	2752	1640
7223	764	1643	2752
7224	1640	2752	1643
7225	316	2750	2748
7226	764	2752	2750
7227	317	2748	2752
7228	2750	2752	2748
7229	108	1635	1639
7230	530	3323	1635
7231	766	1639	3323
7232	1635	3323	1639
7233	53	1336	1339
7234	536	3339	1336
7235	539	1339	3339
7236	1336	3339	1339
7237	109	1645	1641
7238	768	3341	1645
7239	536	1641	3341
7240	1645	3341	1641
7241	252	2463	2465
7242	539	3350	2463
7243	768	2465	3350
7244	2463	3350	2465
7245	536	3341	3339
7246	768	3350	3341
7247	539	3339	3350
7248	3341	3350	3339
7249	108	1639	1637
7250	766	3902	1639
7251	764	1637	3902
7252	1639	3902	1637
7253	252	2465	2464
7254	768	3904	2465
7255	766	2464	3904
7256	2465	3904	2464
7257	109	1643	1645
7258	764	3903	1643
7259	768	1645	3903
7260	1643	3903	1645
7261	766	3904	3902
7262	768	3903	3904
7263	764	3902	3903
7264	3904	3903	3902
7265	21	1177	1173
7266	377	2893	1177
7267	373	1173	2893
7268	1177	2893	1173
7269	169	1966	1964
7270	909	2904	1966

7271	377	1964	2904
7272	1966	2904	1964
7273	165	1940	1943
7274	373	2895	1940
7275	909	1943	2895
7276	1940	2895	1943
7277	377	2904	2893
7278	909	2895	2904
7279	373	2893	2895
7280	2904	2895	2893
7281	78	1484	1482
7282	684	3770	1484
7283	682	1482	3770
7284	1484	3770	1482
7285	254	2477	2475
7286	920	3779	2477
7287	684	2475	3779
7288	2477	3779	2475
7289	169	1965	1968
7290	682	3772	1965
7291	920	1968	3772
7292	1965	3772	1968
7293	684	3779	3770
7294	920	3772	3779
7295	682	3770	3772
7296	3779	3772	3770
7297	254	2474	2476
7298	535	3336	2474
7299	911	2476	3336
7300	2474	3336	2476
7301	169	1968	1966
7302	920	4049	1968
7303	909	1966	4049
7304	1968	4049	1966
7305	254	2476	2477
7306	911	4051	2476
7307	920	2477	4051
7308	2476	4051	2477
7309	165	1943	1945
7310	909	4048	1943
7311	911	1945	4048
7312	1943	4048	1945
7313	920	4051	4049
7314	911	4048	4051
7315	909	4049	4048
7316	4051	4048	4049
7317	24	1191	1195
7318	391	2947	1191
7319	395	1195	2947
7320	1191	2947	1195
7321	183	2051	2048
7322	963	2949	2051
7323	391	2048	2949
7324	2051	2949	2048
7325	187	2072	2074
7326	395	2958	2072
7327	963	2074	2958
7328	2072	2958	2074
7329	391	2949	2947

7330	963	2958	2949
7331	395	2947	2958
7332	2949	2958	2947
7333	53	1341	1337
7334	541	3343	1341
7335	537	1337	3343
7336	1341	3343	1337
7337	256	2488	2486
7338	965	3354	2488
7339	541	2486	3354
7340	2488	3354	2486
7341	183	2049	2053
7342	537	3345	2049
7343	965	2053	3345
7344	2049	3345	2053
7345	541	3354	3343
7346	965	3345	3354
7347	537	3343	3345
7348	3354	3345	3343
7349	183	2053	2051
7350	965	4102	2053
7351	963	2051	4102
7352	2053	4102	2051
7353	256	2489	2488
7354	974	4105	2489
7355	965	2488	4105
7356	2489	4105	2488
7357	187	2074	2076
7358	963	4103	2074
7359	974	2076	4103
7360	2074	4103	2076
7361	965	4105	4102
7362	974	4103	4105
7363	963	4102	4103
7364	4105	4103	4102
7365	254	2478	2474
7366	1054	3337	2478
7367	535	2474	3337
7368	2478	3337	2474
7369	53	1339	1341
7370	539	3349	1339
7371	541	1341	3349
7372	1339	3349	1341
7373	252	2467	2463
7374	1055	3351	2467
7375	539	2463	3351
7376	2467	3351	2463
7377	256	2486	2490
7378	541	3355	2486
7379	1055	2490	3355
7380	2486	3355	2490
7381	539	3351	3349
7382	1055	3355	3351
7383	541	3349	3355
7384	3351	3355	3349
7385	254	2479	2478
7386	1056	4191	2479
7387	1054	2478	4191
7388	2479	4191	2478

7389	256	2490	2491
7390	1055	4192	2490
7391	1056	2491	4192
7392	2490	4192	2491
7393	252	2466	2467
7394	1054	4190	2466
7395	1055	2467	4190
7396	2466	4190	2467
7397	1056	4192	4191
7398	1055	4190	4192
7399	1054	4191	4190
7400	4192	4190	4191
7401	5	1102	1103
7402	302	2712	1102
7403	303	1103	2712
7404	1102	2712	1103
7405	94	1561	1558
7406	728	2714	1561
7407	302	1558	2714
7408	1561	2714	1558
7409	95	1564	1567
7410	303	2716	1564
7411	728	1567	2716
7412	1564	2716	1567
7413	302	2714	2712
7414	728	2716	2714
7415	303	2712	2716
7416	2714	2716	2712
7417	38	1257	1254
7418	457	3105	1257
7419	454	1254	3105
7420	1257	3105	1254
7421	216	2248	2246
7422	730	3116	2248
7423	457	2246	3116
7424	2248	3116	2246
7425	94	1559	1563
7426	454	3107	1559
7427	730	1563	3107
7428	1559	3107	1563
7429	457	3116	3105
7430	730	3107	3116
7431	454	3105	3107
7432	3116	3107	3105
7433	39	1260	1263
7434	460	3123	1260
7435	463	1263	3123
7436	1260	3123	1263
7437	95	1569	1565
7438	732	3125	1569
7439	460	1565	3125
7440	1569	3125	1565
7441	216	2247	2249
7442	463	3134	2247
7443	732	2249	3134
7444	2247	3134	2249
7445	460	3125	3123
7446	732	3134	3125
7447	463	3123	3134

7448	3125	3134	3123
7449	94	1563	1561
7450	730	3866	1563
7451	728	1561	3866
7452	1563	3866	1561
7453	216	2249	2248
7454	732	3868	2249
7455	730	2248	3868
7456	2249	3868	2248
7457	95	1567	1569
7458	728	3867	1567
7459	732	1569	3867
7460	1567	3867	1569
7461	730	3868	3866
7462	732	3867	3868
7463	728	3866	3867
7464	3868	3867	3866
7465	20	1170	1166
7466	370	2871	1170
7467	366	1166	2871
7468	1170	2871	1166
7469	162	1924	1922
7470	887	2884	1924
7471	370	1922	2884
7472	1924	2884	1922
7473	158	1898	1901
7474	366	2873	1898
7475	887	1901	2873
7476	1898	2873	1901
7477	370	2884	2871
7478	887	2873	2884
7479	366	2871	2873
7480	2884	2873	2871
7481	76	1472	1470
7482	672	3734	1472
7483	670	1470	3734
7484	1472	3734	1470
7485	218	2261	2259
7486	900	3743	2261
7487	672	2259	3743
7488	2261	3743	2259
7489	162	1923	1926
7490	670	3736	1923
7491	900	1926	3736
7492	1923	3736	1926
7493	672	3743	3734
7494	900	3736	3743
7495	670	3734	3736
7496	3743	3736	3734
7497	38	1255	1259
7498	455	3109	1255
7499	459	1259	3109
7500	1255	3109	1259
7501	158	1903	1899
7502	889	3111	1903
7503	455	1899	3111
7504	1903	3111	1899
7505	218	2258	2260
7506	459	3120	2258

7507	889	2260	3120
7508	2258	3120	2260
7509	455	3111	3109
7510	889	3120	3111
7511	459	3109	3120
7512	3111	3120	3109
7513	162	1926	1924
7514	900	4025	1926
7515	887	1924	4025
7516	1926	4025	1924
7517	218	2260	2261
7518	889	4027	2260
7519	900	2261	4027
7520	2260	4027	2261
7521	158	1901	1903
7522	887	4024	1901
7523	889	1903	4024
7524	1901	4024	1903
7525	900	4027	4025
7526	889	4024	4027
7527	887	4025	4024
7528	4027	4024	4025
7529	21	1172	1176
7530	372	2889	1172
7531	376	1176	2889
7532	1172	2889	1176
7533	164	1937	1934
7534	905	2891	1937
7535	372	1934	2891
7536	1937	2891	1934
7537	168	1958	1960
7538	376	2902	1958
7539	905	1960	2902
7540	1958	2902	1960
7541	372	2891	2889
7542	905	2902	2891
7543	376	2889	2902
7544	2891	2902	2889
7545	39	1265	1261
7546	465	3127	1265
7547	461	1261	3127
7548	1265	3127	1261
7549	220	2272	2270
7550	907	3138	2272
7551	465	2270	3138
7552	2272	3138	2270
7553	164	1935	1939
7554	461	3129	1935
7555	907	1939	3129
7556	1935	3129	1939
7557	465	3138	3127
7558	907	3129	3138
7559	461	3127	3129
7560	3138	3129	3127
7561	76	1471	1473
7562	671	3738	1471
7563	673	1473	3738
7564	1471	3738	1473
7565	168	1962	1959

7566	918	3740	1962
7567	671	1959	3740
7568	1962	3740	1959
7569	220	2271	2273
7570	673	3745	2271
7571	918	2273	3745
7572	2271	3745	2273
7573	671	3740	3738
7574	918	3745	3740
7575	673	3738	3745
7576	3740	3745	3738
7577	164	1939	1937
7578	907	4042	1939
7579	905	1937	4042
7580	1939	4042	1937
7581	220	2273	2272
7582	918	4045	2273
7583	907	2272	4045
7584	2273	4045	2272
7585	168	1960	1962
7586	905	4043	1960
7587	918	1962	4043
7588	1960	4043	1962
7589	907	4045	4042
7590	918	4043	4045
7591	905	4042	4043
7592	4045	4043	4042
7593	38	1259	1257
7594	459	3115	1259
7595	457	1257	3115
7596	1259	3115	1257
7597	218	2262	2258
7598	1018	3121	2262
7599	459	2258	3121
7600	2262	3121	2258
7601	216	2246	2250
7602	457	3117	2246
7603	1018	2250	3117
7604	2246	3117	2250
7605	459	3121	3115
7606	1018	3117	3121
7607	457	3115	3117
7608	3121	3117	3115
7609	76	1473	1472
7610	673	3742	1473
7611	672	1472	3742
7612	1473	3742	1472
7613	220	2275	2271
7614	1020	3746	2275
7615	673	2271	3746
7616	2275	3746	2271
7617	218	2259	2263
7618	672	3744	2259
7619	1020	2263	3744
7620	2259	3744	2263
7621	673	3746	3742
7622	1020	3744	3746
7623	672	3742	3744
7624	3746	3744	3742

7625	39	1263	1265
7626	463	3133	1263
7627	465	1265	3133
7628	1263	3133	1265
7629	216	2251	2247
7630	1019	3135	2251
7631	463	2247	3135
7632	2251	3135	2247
7633	220	2270	2274
7634	465	3139	2270
7635	1019	2274	3139
7636	2270	3139	2274
7637	463	3135	3133
7638	1019	3139	3135
7639	465	3133	3139
7640	3135	3139	3133
7641	218	2263	2262
7642	1020	4155	2263
7643	1018	2262	4155
7644	2263	4155	2262
7645	220	2274	2275
7646	1019	4156	2274
7647	1020	2275	4156
7648	2274	4156	2275
7649	216	2250	2251
7650	1018	4154	2250
7651	1019	2251	4154
7652	2250	4154	2251
7653	1020	4156	4155
7654	1019	4154	4156
7655	1018	4155	4154
7656	4156	4154	4155
7657	24	1195	1194
7658	395	2955	1195
7659	394	1194	2955
7660	1195	2955	1194
7661	187	2075	2072
7662	971	2959	2075
7663	395	2072	2959
7664	2075	2959	2072
7665	186	2066	2069
7666	394	2957	2066
7667	971	2069	2957
7668	2066	2957	2069
7669	395	2959	2955
7670	971	2957	2959
7671	394	2955	2957
7672	2959	2957	2955
7673	78	1487	1483
7674	687	3775	1487
7675	683	1483	3775
7676	1487	3775	1483
7677	286	2669	2667
7678	975	3786	2669
7679	687	2667	3786
7680	2669	3786	2667
7681	187	2073	2077
7682	683	3777	2073
7683	975	2077	3777

7684	2073	3777	2077
7685	687	3786	3775
7686	975	3777	3786
7687	683	3775	3777
7688	3786	3777	3775
7689	77	1477	1481
7690	677	3757	1477
7691	681	1481	3757
7692	1477	3757	1481
7693	186	2071	2067
7694	973	3759	2071
7695	677	2067	3759
7696	2071	3759	2067
7697	286	2666	2668
7698	681	3768	2666
7699	973	2668	3768
7700	2666	3768	2668
7701	677	3759	3757
7702	973	3768	3759
7703	681	3757	3768
7704	3759	3768	3757
7705	187	2077	2075
7706	975	4110	2077
7707	971	2075	4110
7708	2077	4110	2075
7709	286	2668	2669
7710	973	4111	2668
7711	975	2669	4111
7712	2668	4111	2669
7713	186	2069	2071
7714	971	4109	2069
7715	973	2071	4109
7716	2069	4109	2071
7717	975	4111	4110
7718	973	4109	4111
7719	971	4110	4109
7720	4111	4109	4110
7721	21	1176	1177
7722	376	2901	1176
7723	377	1177	2901
7724	1176	2901	1177
7725	168	1961	1958
7726	917	2903	1961
7727	376	1958	2903
7728	1961	2903	1958
7729	169	1964	1967
7730	377	2905	1964
7731	917	1967	2905
7732	1964	2905	1967
7733	376	2903	2901
7734	917	2905	2903
7735	377	2901	2905
7736	2903	2905	2901
7737	76	1475	1471
7738	675	3739	1475
7739	671	1471	3739
7740	1475	3739	1471
7741	285	2662	2660
7742	919	3750	2662

7743	675	2660	3750
7744	2662	3750	2660
7745	168	1959	1963
7746	671	3741	1959
7747	919	1963	3741
7748	1959	3741	1963
7749	675	3750	3739
7750	919	3741	3750
7751	671	3739	3741
7752	3750	3741	3739
7753	78	1482	1486
7754	682	3771	1482
7755	686	1486	3771
7756	1482	3771	1486
7757	169	1969	1965
7758	921	3773	1969
7759	682	1965	3773
7760	1969	3773	1965
7761	285	2661	2663
7762	686	3784	2661
7763	921	2663	3784
7764	2661	3784	2663
7765	682	3773	3771
7766	921	3784	3773
7767	686	3771	3784
7768	3773	3784	3771
7769	168	1963	1961
7770	919	4055	1963
7771	917	1961	4055
7772	1963	4055	1961
7773	285	2663	2662
7774	921	4057	2663
7775	919	2662	4057
7776	2663	4057	2662
7777	169	1967	1969
7778	917	4056	1967
7779	921	1969	4056
7780	1967	4056	1969
7781	919	4057	4055
7782	921	4056	4057
7783	917	4055	4056
7784	4057	4056	4055
7785	20	1171	1170
7786	371	2883	1171
7787	370	1170	2883
7788	1171	2883	1170
7789	163	1931	1928
7790	899	2887	1931
7791	371	1928	2887
7792	1931	2887	1928
7793	162	1922	1925
7794	370	2885	1922
7795	899	1925	2885
7796	1922	2885	1925
7797	371	2887	2883
7798	899	2885	2887
7799	370	2883	2885
7800	2887	2885	2883
7801	77	1480	1476

7802	680	3753	1480
7803	676	1476	3753
7804	1480	3753	1476
7805	284	2657	2655
7806	903	3766	2657
7807	680	2655	3766
7808	2657	3766	2655
7809	163	1929	1933
7810	676	3755	1929
7811	903	1933	3755
7812	1929	3755	1933
7813	680	3766	3753
7814	903	3755	3766
7815	676	3753	3755
7816	3766	3755	3753
7817	76	1470	1474
7818	670	3735	1470
7819	674	1474	3735
7820	1470	3735	1474
7821	162	1927	1923
7822	901	3737	1927
7823	670	1923	3737
7824	1927	3737	1923
7825	284	2654	2656
7826	674	3748	2654
7827	901	2656	3748
7828	2654	3748	2656
7829	670	3737	3735
7830	901	3748	3737
7831	674	3735	3748
7832	3737	3748	3735
7833	163	1933	1931
7834	903	4038	1933
7835	899	1931	4038
7836	1933	4038	1931
7837	284	2656	2657
7838	901	4039	2656
7839	903	2657	4039
7840	2656	4039	2657
7841	162	1925	1927
7842	899	4037	1925
7843	901	1927	4037
7844	1925	4037	1927
7845	903	4039	4038
7846	901	4037	4039
7847	899	4038	4037
7848	4039	4037	4038
7849	78	1486	1487
7850	686	3783	1486
7851	687	1487	3783
7852	1486	3783	1487
7853	285	2665	2661
7854	1086	3785	2665
7855	686	2661	3785
7856	2665	3785	2661
7857	286	2667	2671
7858	687	3787	2667
7859	1086	2671	3787
7860	2667	3787	2671

7861	686	3785	3783
7862	1086	3787	3785
7863	687	3783	3787
7864	3785	3787	3783
7865	76	1474	1475
7866	674	3747	1474
7867	675	1475	3747
7868	1474	3747	1475
7869	284	2658	2654
7870	1084	3749	2658
7871	674	2654	3749
7872	2658	3749	2654
7873	285	2660	2664
7874	675	3751	2660
7875	1084	2664	3751
7876	2660	3751	2664
7877	674	3749	3747
7878	1084	3751	3749
7879	675	3747	3751
7880	3749	3751	3747
7881	77	1481	1480
7882	681	3765	1481
7883	680	1480	3765
7884	1481	3765	1480
7885	286	2670	2666
7886	1085	3769	2670
7887	681	2666	3769
7888	2670	3769	2666
7889	284	2655	2659
7890	680	3767	2655
7891	1085	2659	3767
7892	2655	3767	2659
7893	681	3769	3765
7894	1085	3767	3769
7895	680	3765	3767
7896	3769	3767	3765
7897	285	2664	2665
7898	1084	4221	2664
7899	1086	2665	4221
7900	2664	4221	2665
7901	284	2659	2658
7902	1085	4220	2659
7903	1084	2658	4220
7904	2659	4220	2658
7905	286	2671	2670
7906	1086	4222	2671
7907	1085	2670	4222
7908	2671	4222	2670
7909	1084	4220	4221
7910	1085	4222	4220
7911	1086	4221	4222
7912	4220	4222	4221
7913	1519	1515	4235
7914	1519	4235	84
7915	711	2999	4343
7916	711	4343	1519
7917	2999	4259	1515
7918	2999	410	4259
7919	1515	4343	2999

7920	1515	1519	4343
7921	196	4359	2129
7922	196	2127	4359
7923	2127	4261	3008
7924	2127	413	4261
7925	2129	3008	4298
7926	2129	4298	711
7927	3008	2129	4359
7928	3008	4359	2127
7929	410	2999	4384
7930	410	4384	2997
7931	2999	4298	3008
7932	2999	711	4298
7933	2997	3008	4261
7934	2997	4261	413
7935	3008	4384	2999
7936	3008	2997	4384
7937	1207	1209	4228
7938	1207	4228	27
7939	407	2989	4321
7940	407	4321	1207
7941	2989	4257	1209
7942	2989	409	4257
7943	1209	4321	2989
7944	1209	1207	4321
7945	118	1689	4344
7946	118	4344	1687
7947	1689	4300	2990
7948	1689	788	4300
7949	1687	2990	4255
7950	1687	4255	407
7951	2990	4344	1689
7952	2990	1687	4344
7953	2134	4238	2132
7954	2134	197	4238
7955	2994	2132	4258
7956	2994	4258	409
7957	788	4360	2994
7958	788	2134	4360
7959	2132	2994	4360
7960	2132	4360	2134
7961	2989	4255	2990
7962	2989	407	4255
7963	2994	2990	4300
7964	2994	4300	788
7965	409	4381	2994
7966	409	2989	4381
7967	2990	2994	4381
7968	2990	4381	2989
7969	1208	4228	1209
7970	1208	27	4228
7971	2991	1209	4257
7972	2991	4257	409
7973	408	4322	2991
7974	408	1208	4322
7975	1209	2991	4322
7976	1209	4322	1208
7977	2136	2132	4238
7978	2136	4238	197

7979	997	2995	4361
7980	997	4361	2136
7981	2995	4258	2132
7982	2995	409	4258
7983	2132	4361	2995
7984	2132	2136	4361
7985	196	4358	2130
7986	196	2126	4358
7987	2126	4256	2993
7988	2126	408	4256
7989	2130	2993	4309
7990	2130	4309	997
7991	2993	2130	4358
7992	2993	4358	2126
7993	409	2995	4382
7994	409	4382	2991
7995	2995	4309	2993
7996	2995	997	4309
7997	2991	2993	4256
7998	2991	4256	408
7999	2993	4382	2995
8000	2993	2991	4382
8001	1441	1443	4232
8002	1441	4232	71
8003	641	3648	4335
8004	641	4335	1441
8005	3648	4285	1443
8006	3648	643	4285
8007	1443	4335	3648
8008	1443	1441	4335
8009	156	1890	4346
8010	156	4346	1887
8011	1890	4302	3650
8012	1890	882	4302
8013	1887	3650	4283
8014	1887	4283	641
8015	3650	4346	1890
8016	3650	1887	4346
8017	2219	4239	2217
8018	2219	211	4239
8019	3655	2217	4286
8020	3655	4286	643
8021	882	4363	3655
8022	882	2219	4363
8023	2217	3655	4363
8024	2217	4363	2219
8025	3648	4283	3650
8026	3648	641	4283
8027	3655	3650	4302
8028	3655	4302	882
8029	643	4395	3655
8030	643	3648	4395
8031	3650	3655	4395
8032	3650	4395	3648
8033	1442	4232	1443
8034	1442	71	4232
8035	3652	1443	4285
8036	3652	4285	643
8037	642	4336	3652

8038	642	1442	4336
8039	1443	3652	4336
8040	1443	4336	1442
8041	2221	2217	4239
8042	2221	4239	211
8043	1011	3656	4364
8044	1011	4364	2221
8045	3656	4286	2217
8046	3656	643	4286
8047	2217	4364	3656
8048	2217	2221	4364
8049	209	4362	2209
8050	209	2205	4362
8051	2205	4284	3654
8052	2205	642	4284
8053	2209	3654	4310
8054	2209	4310	1011
8055	3654	2209	4362
8056	3654	4362	2205
8057	643	3656	4396
8058	643	4396	3652
8059	3656	4310	3654
8060	3656	1011	4310
8061	3652	3654	4284
8062	3652	4284	642
8063	3654	4396	3656
8064	3654	3652	4396
8065	73	4339	1457
8066	73	1453	4339
8067	1453	4291	3685
8068	1453	653	4291
8069	1457	3685	4292
8070	1457	4292	657
8071	3685	1457	4339
8072	3685	4339	1453
8073	3687	3685	4291
8074	3687	4291	653
8075	986	3696	4399
8076	986	4399	3687
8077	3696	4292	3685
8078	3696	657	4292
8079	3685	4399	3696
8080	3685	3687	4399
8081	180	2032	4355
8082	180	4355	2030
8083	2032	4307	2938
8084	2032	941	4307
8085	2030	2938	4252
8086	2030	4252	388
8087	2938	4355	2032
8088	2938	2030	4355
8089	2925	4252	2938
8090	2925	388	4252
8091	2927	2938	4307
8092	2927	4307	941
8093	384	4379	2927
8094	384	2925	4379
8095	2938	2927	4379
8096	2938	4379	2925

8097	23	1189	4318
8098	23	4318	1188
8099	1189	4253	2937
8100	1189	389	4253
8101	1188	2937	4251
8102	1188	4251	388
8103	2937	4318	1189
8104	2937	1188	4318
8105	2941	2937	4253
8106	2941	4253	389
8107	953	4380	2941
8108	953	2939	4380
8109	2939	4251	2937
8110	2939	388	4251
8111	2937	2941	4380
8112	2937	4380	2939
8113	194	2117	4357
8114	194	4357	2115
8115	2117	4297	3005
8116	2117	710	4297
8117	2115	3005	4260
8118	2115	4260	412
8119	3005	4357	2117
8120	3005	2115	4357
8121	1518	4235	1515
8122	1518	84	4235
8123	2998	1515	4259
8124	2998	4259	410
8125	710	4342	2998
8126	710	1518	4342
8127	1515	2998	4342
8128	1515	4342	1518
8129	2996	4260	3005
8130	2996	412	4260
8131	2998	3005	4297
8132	2998	4297	710
8133	410	4383	2998
8134	410	2996	4383
8135	3005	2998	4383
8136	3005	4383	2996
8137	70	4334	1436
8138	70	1434	4334
8139	1434	4281	3626
8140	1434	634	4281
8141	1436	3626	4282
8142	1436	4282	636
8143	3626	1436	4334
8144	3626	4334	1434
8145	3628	3626	4281
8146	3628	4281	634
8147	859	3635	4394
8148	859	4394	3628
8149	3635	4282	3626
8150	3635	636	4282
8151	3626	4394	3635
8152	3626	3628	4394
8153	1191	4227	1193
8154	1191	24	4227
8155	2946	1193	4254

8156	2946	4254	393
8157	391	4319	2946
8158	391	1191	4319
8159	1193	2946	4319
8160	1193	4319	1191
8161	1192	1193	4227
8162	1192	4227	24
8163	392	2950	4320
8164	392	4320	1192
8165	2950	4254	1193
8166	2950	393	4254
8167	1193	4320	2950
8168	1193	1192	4320
8169	1446	1450	4233
8170	1446	4233	72
8171	646	3663	4337
8172	646	4337	1446
8173	3663	4288	1450
8174	3663	650	4288
8175	1450	4337	3663
8176	1450	1446	4337
8177	148	1851	4345
8178	148	4345	1847
8179	1851	4301	3665
8180	1851	865	4301
8181	1847	3665	4287
8182	1847	4287	646
8183	3665	4345	1851
8184	3665	1847	4345
8185	2621	4242	2619
8186	2621	278	4242
8187	3676	2619	4289
8188	3676	4289	650
8189	865	4372	3676
8190	865	2621	4372
8191	2619	3676	4372
8192	2619	4372	2621
8193	3663	4287	3665
8194	3663	646	4287
8195	3676	3665	4301
8196	3676	4301	865
8197	650	4397	3676
8198	650	3663	4397
8199	3665	3676	4397
8200	3665	4397	3663
8201	1451	4233	1450
8202	1451	72	4233
8203	3675	1450	4288
8204	3675	4288	650
8205	651	4338	3675
8206	651	1451	4338
8207	1450	3675	4338
8208	1450	4338	1451
8209	2623	2619	4242
8210	2623	4242	278
8211	1079	3677	4373
8212	1079	4373	2623
8213	3677	4289	2619
8214	3677	650	4289

8215	2619	4373	3677
8216	2619	2623	4373
8217	282	4374	2646
8218	282	2642	4374
8219	2642	4290	3679
8220	2642	651	4290
8221	2646	3679	4313
8222	2646	4313	1079
8223	3679	2646	4374
8224	3679	4374	2642
8225	650	3677	4398
8226	650	4398	3675
8227	3677	4313	3679
8228	3677	1079	4313
8229	3675	3679	4290
8230	3675	4290	651
8231	3679	4398	3677
8232	3679	3675	4398
8233	1404	1406	4231
8234	1404	4231	65
8235	604	3536	4332
8236	604	4332	1404
8237	3536	4280	1406
8238	3536	606	4280
8239	1406	4332	3536
8240	1406	1404	4332
8241	1407	4231	1406
8242	1407	65	4231
8243	3544	1406	4280
8244	3544	4280	606
8245	607	4333	3544
8246	607	1407	4333
8247	1406	3544	4333
8248	1406	4333	1407
8249	1175	4226	4314
8250	4226	21	4314
8251	4314	21	1173
8252	2892	4315	4243
8253	4243	4315	1173
8254	4243	1173	373
8255	375	4245	4247
8256	4245	1175	4247
8257	4247	1175	2892
8258	1173	4315	4314
8259	4314	4315	2892
8260	4314	2892	1175
8261	1952	4351	4237
8262	4237	4351	167
8263	4351	1954	167
8264	375	4249	4246
8265	4246	4249	1952
8266	4249	2899	1952
8267	2899	4303	4353
8268	4303	1954	4353
8269	4303	908	1954
8270	1954	4351	4353
8271	4351	2899	4353
8272	4351	1952	2899
8273	2892	4243	4375

8274	4243	373	4375
8275	4375	373	2894
8276	2899	4376	4303
8277	4303	4376	2894
8278	4303	2894	908
8279	375	4247	4249
8280	4247	2892	4249
8281	4249	2892	2899
8282	2894	4376	4375
8283	4375	4376	2899
8284	4375	2899	2892
8285	1331	4229	4326
8286	4229	1334	4326
8287	4229	52	1334
8288	3324	4329	4277
8289	4277	4329	534
8290	4329	1334	534
8291	531	4265	4267
8292	4265	3324	4267
8293	4265	1331	3324
8294	1334	4329	4326
8295	4326	4329	1331
8296	4329	3324	1331
8297	1941	4347	4236
8298	4236	4347	1944
8299	4236	1944	165
8300	531	4269	4266
8301	4266	4269	3326
8302	4266	3326	1941
8303	3326	4304	4349
8304	4304	910	4349
8305	4349	910	1944
8306	1944	4347	4349
8307	4347	1941	4349
8308	4349	1941	3326
8309	3324	4277	4388
8310	4277	3334	4388
8311	4277	534	3334
8312	3326	4390	4304
8313	4304	4390	910
8314	4390	3334	910
8315	531	4267	4269
8316	4267	3326	4269
8317	4267	3324	3326
8318	3334	4390	4388
8319	4388	4390	3324
8320	4390	3326	3324
8321	1175	4316	4226
8322	4226	4316	21
8323	4316	1174	21
8324	375	4248	4245
8325	4245	4248	1175
8326	4248	2896	1175
8327	2896	4244	4317
8328	4244	1174	4317
8329	4244	374	1174
8330	1174	4316	4317
8331	4316	2896	4317
8332	4316	1175	2896

8333	1952	4237	4352
8334	4237	167	4352
8335	4352	167	1955
8336	2900	4354	4306
8337	4306	4354	1955
8338	4306	1955	912
8339	375	4246	4250
8340	4246	1952	4250
8341	4250	1952	2900
8342	1955	4354	4352
8343	4352	4354	2900
8344	4352	2900	1952
8345	375	4250	4248
8346	4250	2900	4248
8347	4248	2900	2896
8348	2900	4306	4378
8349	4306	912	4378
8350	4378	912	2898
8351	2896	4377	4244
8352	4244	4377	2898
8353	4244	2898	374
8354	2898	4377	4378
8355	4378	4377	2896
8356	4378	2896	2900
8357	48	4323	1315
8358	48	1311	4323
8359	1311	4262	3271
8360	1311	511	4262
8361	1315	3271	4263
8362	1315	4263	515
8363	3271	1315	4323
8364	3271	4323	1311
8365	3273	3271	4262
8366	3273	4262	511
8367	961	3282	4385
8368	961	4385	3273
8369	3282	4263	3271
8370	3282	515	4263
8371	3271	4385	3282
8372	3271	3273	4385
8373	1333	4324	4230
8374	4230	4324	52
8375	4324	1330	52
8376	533	4273	4271
8377	4271	4273	1333
8378	4273	3321	1333
8379	3321	4264	4325
8380	4264	1330	4325
8381	4264	530	1330
8382	1330	4324	4325
8383	4324	3321	4325
8384	4324	1333	3321
8385	2462	4240	4365
8386	4240	252	4365
8387	4365	252	2464
8388	3332	4367	4299
8389	4299	4367	2464
8390	4299	2464	766
8391	533	4272	4275

8392	4272	2462	4275
8393	4275	2462	3332
8394	2464	4367	4365
8395	4365	4367	3332
8396	4365	3332	2462
8397	533	4275	4273
8398	4275	3321	4273
8399	4275	3332	3321
8400	3332	4299	4387
8401	4299	3323	4387
8402	4299	766	3323
8403	3321	4386	4264
8404	4264	4386	530
8405	4386	3323	530
8406	3323	4386	4387
8407	4387	4386	3332
8408	4386	3321	3332
8409	1331	4327	4229
8410	4229	4327	1335
8411	4229	1335	52
8412	531	4268	4265
8413	4265	4268	3325
8414	4265	3325	1331
8415	3325	4278	4330
8416	4278	535	4330
8417	4330	535	1335
8418	1335	4327	4330
8419	4327	1331	4330
8420	4330	1331	3325
8421	1941	4236	4348
8422	4236	1945	4348
8423	4236	165	1945
8424	3327	4350	4305
8425	4305	4350	911
8426	4350	1945	911
8427	531	4266	4270
8428	4266	3327	4270
8429	4266	1941	3327
8430	1945	4350	4348
8431	4348	4350	1941
8432	4350	3327	1941
8433	531	4270	4268
8434	4270	3327	4268
8435	4268	3327	3325
8436	3327	4305	4391
8437	4305	911	4391
8438	4391	911	3336
8439	3325	4389	4278
8440	4278	4389	3336
8441	4278	3336	535
8442	3336	4389	4391
8443	4391	4389	3325
8444	4391	3325	3327
8445	1483	1485	4234
8446	1483	4234	78
8447	683	3774	4340
8448	683	4340	1483
8449	3774	4295	1485
8450	3774	685	4295

8451	1485	4340	3774
8452	1485	1483	4340
8453	187	2076	4356
8454	187	4356	2073
8455	2076	4308	3776
8456	2076	974	4308
8457	2073	3776	4293
8458	2073	4293	683
8459	3776	4356	2076
8460	3776	2073	4356
8461	2489	4241	2487
8462	2489	256	4241
8463	3781	2487	4296
8464	3781	4296	685
8465	974	4370	3781
8466	974	2489	4370
8467	2487	3781	4370
8468	2487	4370	2489
8469	3774	4293	3776
8470	3774	683	4293
8471	3781	3776	4308
8472	3781	4308	974
8473	685	4400	3781
8474	685	3774	4400
8475	3776	3781	4400
8476	3776	4400	3774
8477	1333	4230	4328
8478	4230	1335	4328
8479	4230	52	1335
8480	3331	4331	4279
8481	4279	4331	535
8482	4331	1335	535
8483	533	4271	4274
8484	4271	3331	4274
8485	4271	1333	3331
8486	1335	4331	4328
8487	4328	4331	1333
8488	4331	3331	1333
8489	2462	4366	4240
8490	4240	4366	2466
8491	4240	2466	252
8492	533	4276	4272
8493	4272	4276	3333
8494	4272	3333	2462
8495	3333	4311	4368
8496	4311	1054	4368
8497	4368	1054	2466
8498	2466	4366	4368
8499	4366	2462	4368
8500	4368	2462	3333
8501	3331	4279	4392
8502	4279	3337	4392
8503	4279	535	3337
8504	3333	4393	4311
8505	4311	4393	1054
8506	4393	3337	1054
8507	533	4274	4276
8508	4274	3333	4276
8509	4274	3331	3333

8510	3337	4393	4392
8511	4392	4393	3331
8512	4393	3333	3331
8513	1484	4234	1485
8514	1484	78	4234
8515	3778	1485	4295
8516	3778	4295	685
8517	684	4341	3778
8518	684	1484	4341
8519	1485	3778	4341
8520	1485	4341	1484
8521	2491	2487	4241
8522	2491	4241	256
8523	1056	3782	4371
8524	1056	4371	2491
8525	3782	4296	2487
8526	3782	685	4296
8527	2487	4371	3782
8528	2487	2491	4371
8529	254	4369	2479
8530	254	2475	4369
8531	2475	4294	3780
8532	2475	684	4294
8533	2479	3780	4312
8534	2479	4312	1056
8535	3780	2479	4369
8536	3780	4369	2475
8537	685	3782	4401
8538	685	4401	3778
8539	3782	4312	3780
8540	3782	1056	4312
8541	3778	3780	4294
8542	3778	4294	684
8543	3780	4401	3782
8544	3780	3778	4401

End of Connectivity Matrix of Nodes

DESIGN 2

Node Coordinates

Node Index	X(m)	Y(m)	Z(m)
1	0.00000000	0.00000000	0.00019550
2	0.00000000	4.00000000	0.18670577
3	4.00000000	0.00000000	0.19960899
4	4.00000000	4.00000000	0.17497556
5	0.00000000	2.00000000	-0.13391984
6	2.00000000	0.00000000	-0.11749756
7	2.00000000	2.00000000	-0.19960899
8	2.00000000	4.00000000	0.11593353
9	4.00000000	2.00000000	0.14134897
10	0.00000000	1.00000000	-0.12258065
11	1.00000000	0.00000000	-0.05728250
12	1.00000000	1.00000000	-0.19530792
13	0.00000000	3.00000000	-0.00332356
14	1.00000000	4.00000000	0.18592375
15	3.00000000	0.00000000	0.05454545
16	4.00000000	1.00000000	0.18318671
17	3.00000000	3.00000000	0.10029326
18	3.00000000	4.00000000	0.19921799
19	4.00000000	3.00000000	0.17536657
20	1.00000000	2.00000000	-0.19843597
21	1.00000000	3.00000000	-0.07761486
22	2.00000000	1.00000000	-0.19921799
23	3.00000000	1.00000000	-0.09716520
24	2.00000000	3.00000000	-0.15386119
25	3.00000000	2.00000000	-0.09951124
26	0.00000000	0.50000000	-0.06901271
27	0.50000000	0.00000000	-0.06510264
28	0.50000000	0.50000000	-0.08582600
29	0.00000000	3.50000000	0.09169110
30	0.50000000	4.00000000	0.18475073
31	3.50000000	0.00000000	0.12727273
32	4.00000000	0.50000000	0.19139785
33	3.50000000	3.50000000	0.19413490
34	3.50000000	4.00000000	-0.11945259
35	4.00000000	3.50000000	0.17536657
36	0.00000000	1.50000000	-0.14760508
37	0.00000000	2.50000000	-0.06549365
38	0.50000000	2.00000000	-0.16637341
39	0.50000000	2.50000000	-0.14056696
40	1.50000000	0.00000000	-0.11124145
41	2.50000000	0.00000000	-0.03147605
42	2.00000000	0.50000000	-0.17419355
43	2.50000000	0.50000000	-0.10889541
44	1.50000000	1.50000000	-0.19765396
45	2.50000000	2.50000000	-0.09833822
46	1.50000000	2.00000000	-0.19921799
47	2.00000000	1.50000000	-0.19921799
48	2.00000000	2.50000000	-0.17693060
49	2.50000000	2.00000000	-0.19960899
50	1.50000000	4.00000000	0.19648094
51	2.50000000	4.00000000	0.15659824
52	1.50000000	3.50000000	0.01309873
53	2.00000000	3.50000000	-0.00019550
54	4.00000000	1.50000000	0.11436950
55	4.00000000	2.50000000	0.18435973
56	3.50000000	1.50000000	0.03499511

57	3.50000000	2.00000000	0.00879765
58	0.50000000	1.00000000	-0.17849462
59	0.50000000	1.50000000	-0.18005865
60	1.00000000	0.50000000	-0.17614858
61	1.50000000	0.50000000	-0.17810362
62	1.00000000	1.50000000	-0.19687195
63	1.50000000	1.00000000	-0.19726295
64	0.50000000	3.50000000	0.09130010
65	0.50000000	3.00000000	-0.04046921
66	1.00000000	3.50000000	0.05415445
67	3.50000000	0.50000000	0.11906158
68	3.00000000	0.50000000	-0.02130987
69	3.50000000	1.00000000	0.04320626
70	3.00000000	3.50000000	0.14956012
71	3.50000000	3.00000000	0.13782991
72	2.50000000	3.00000000	-0.04398827
73	3.00000000	2.50000000	0.02521994
74	2.50000000	3.50000000	0.09951124
75	3.50000000	2.50000000	0.03812317
76	1.00000000	2.50000000	-0.13782991
77	1.50000000	2.50000000	-0.17614858
78	1.50000000	3.00000000	-0.11593353
79	2.50000000	1.00000000	-0.14799609
80	2.50000000	1.50000000	-0.14916911
81	3.00000000	1.50000000	-0.09833822
82	0.00000000	0.25000000	-0.01544477
83	0.25000000	0.00000000	-0.01348974
84	0.25000000	0.25000000	-0.02365591
85	0.00000000	3.75000000	0.13939394
86	0.25000000	4.00000000	0.18592375
87	3.75000000	0.00000000	0.16363636
88	4.00000000	0.25000000	0.19569892
89	3.75000000	3.75000000	0.18475073
90	3.75000000	4.00000000	0.02795699
91	4.00000000	3.75000000	0.17536657
92	0.00000000	1.75000000	-0.13978495
93	0.00000000	2.25000000	0.00058651
94	0.25000000	2.00000000	-0.14956012
95	0.25000000	2.25000000	-0.12453568
96	1.75000000	0.00000000	0.08582600
97	2.25000000	0.00000000	-0.05884653
98	2.00000000	0.25000000	-0.14565005
99	2.25000000	0.25000000	-0.11319648
100	1.75000000	1.75000000	-0.19843597
101	2.25000000	2.25000000	-0.14877810
102	1.75000000	2.00000000	-0.19921799
103	2.00000000	1.75000000	-0.19921799
104	2.00000000	2.25000000	-0.18826979
105	2.25000000	2.00000000	-0.19960899
106	1.75000000	4.00000000	0.15620723
107	2.25000000	4.00000000	0.13626588
108	1.75000000	3.75000000	0.06471163
109	2.00000000	3.75000000	0.05806452
110	4.00000000	1.75000000	0.12805474
111	4.00000000	2.25000000	0.16285435
112	3.75000000	1.75000000	0.08817204
113	3.75000000	2.00000000	0.07487781
114	0.00000000	0.75000000	-0.09560117
115	0.00000000	1.25000000	-0.13509286

116	0.25000000	1.00000000	-0.15034213
117	0.25000000	1.25000000	-0.15112414
118	0.75000000	0.00000000	-0.06119257
119	1.25000000	0.00000000	-0.08426197
120	1.00000000	0.25000000	-0.11671554
121	1.25000000	0.25000000	-0.11749756
122	0.75000000	0.75000000	-0.14056696
123	1.25000000	1.25000000	-0.19648094
124	0.75000000	1.00000000	-0.18670577
125	1.00000000	0.75000000	-0.18553275
126	1.00000000	1.25000000	-0.19608993
127	1.25000000	1.00000000	-0.19608993
128	0.00000000	3.25000000	0.04437928
129	0.00000000	2.75000000	-0.03460411
130	0.25000000	3.25000000	0.04398827
131	0.25000000	3.00000000	-0.02170088
132	0.75000000	4.00000000	0.18553275
133	1.25000000	4.00000000	0.19139785
134	0.75000000	3.75000000	0.18866080
135	1.00000000	3.75000000	0.12023460
136	3.25000000	0.00000000	0.09090909
137	2.75000000	0.00000000	0.01153470
138	3.25000000	0.25000000	0.08699902
139	3.00000000	0.25000000	0.01661779
140	4.00000000	0.75000000	0.18748778
141	4.00000000	1.25000000	0.14877810
142	3.75000000	0.75000000	0.15112414
143	3.75000000	1.00000000	0.11319648
144	3.25000000	3.25000000	0.14721408
145	2.75000000	2.75000000	0.00097752
146	3.00000000	3.25000000	0.12492669
147	3.25000000	3.00000000	0.11906158
148	2.75000000	3.00000000	0.02795699
149	3.00000000	2.75000000	0.06275660
150	3.25000000	4.00000000	0.04007820
151	2.75000000	4.00000000	0.17810362
152	3.00000000	3.75000000	0.17458456
153	2.75000000	3.75000000	0.14956012
154	4.00000000	3.25000000	0.17536657
155	4.00000000	2.75000000	0.18005865
156	3.75000000	3.00000000	0.15659824
157	3.75000000	2.75000000	0.10694037
158	0.75000000	2.00000000	-0.18240469
159	1.25000000	2.00000000	-0.19882698
160	0.75000000	1.75000000	-0.18944282
161	1.00000000	1.75000000	-0.19765396
162	1.00000000	2.25000000	-0.16832845
163	1.25000000	2.25000000	-0.18748778
164	0.75000000	2.75000000	-0.10889541
165	1.25000000	3.25000000	-0.03225806
166	0.75000000	3.00000000	-0.05923754
167	1.00000000	3.25000000	-0.01192571
168	1.00000000	2.75000000	-0.10772239
169	1.25000000	3.00000000	-0.09677419
170	2.00000000	0.75000000	-0.18670577
171	2.00000000	1.25000000	-0.19921799
172	1.75000000	0.75000000	-0.18866080
173	1.75000000	1.00000000	-0.19804497
174	2.25000000	1.00000000	-0.17341153

175	2.25000000	1.25000000	-0.17419355
176	2.75000000	0.75000000	-0.15307918
177	3.25000000	1.25000000	-0.03108504
178	3.00000000	0.75000000	-0.05923754
179	3.25000000	1.00000000	-0.02678397
180	2.75000000	1.00000000	-0.12258065
181	3.00000000	1.25000000	-0.17223851
182	2.00000000	2.75000000	-0.16520039
183	2.00000000	3.25000000	-0.02678397
184	2.25000000	3.00000000	-0.09872923
185	2.25000000	3.25000000	-0.02717498
186	1.75000000	2.75000000	-0.16480938
187	1.75000000	3.00000000	-0.13470186
188	2.75000000	2.00000000	-0.14956012
189	3.25000000	2.00000000	-0.04516129
190	3.00000000	2.25000000	-0.03695015
191	3.25000000	2.25000000	-0.03069404
192	2.75000000	1.75000000	-0.12414467
193	3.00000000	1.75000000	-0.09872923
194	0.25000000	0.50000000	-0.07722385
195	0.25000000	0.75000000	-0.12375367
196	0.50000000	0.25000000	-0.07526882
197	0.75000000	0.25000000	-0.12062561
198	0.50000000	0.75000000	-0.13196481
199	0.75000000	0.50000000	-0.13079179
200	0.25000000	3.75000000	0.13822092
201	0.25000000	3.50000000	0.09169110
202	0.50000000	3.75000000	0.13782991
203	3.75000000	0.25000000	0.15933529
204	3.50000000	0.25000000	0.12336266
205	3.75000000	0.50000000	0.15542522
206	3.50000000	3.75000000	0.03734115
207	3.75000000	3.50000000	0.18475073
208	3.25000000	3.50000000	0.17184751
209	3.50000000	3.25000000	0.16598240
210	3.25000000	3.75000000	0.01505376
211	3.75000000	3.25000000	0.15659824
212	0.25000000	1.75000000	-0.15698925
213	0.25000000	1.50000000	-0.16363636
214	0.25000000	2.50000000	-0.10303030
215	0.25000000	2.75000000	-0.05298143
216	0.50000000	2.25000000	-0.15347019
217	0.50000000	1.75000000	-0.17302053
218	0.75000000	2.25000000	-0.15190616
219	0.50000000	2.75000000	-0.09051808
220	0.75000000	2.50000000	-0.13939394
221	1.75000000	0.25000000	-0.14291300
222	1.50000000	0.25000000	-0.14447703
223	2.50000000	0.25000000	-0.07018573
224	2.75000000	0.25000000	-0.02639296
225	2.25000000	0.50000000	-0.14134897
226	1.75000000	0.50000000	-0.17614858
227	2.25000000	0.75000000	-0.16089932
228	2.75000000	0.50000000	-0.06510264
229	2.50000000	0.75000000	-0.12844575
230	1.50000000	1.75000000	-0.19843597
231	1.75000000	1.50000000	-0.19843597
232	1.25000000	1.50000000	-0.19726295
233	1.50000000	1.25000000	-0.19726295

234	2.25000000	2.50000000	-0.13743891
235	2.50000000	2.25000000	-0.14877810
236	2.50000000	2.75000000	-0.07096774
237	2.75000000	2.50000000	-0.03655914
238	1.75000000	2.25000000	-0.18787879
239	1.25000000	1.75000000	-0.19804497
240	1.50000000	2.25000000	-0.18748778
241	2.25000000	1.75000000	-0.19921799
242	1.75000000	1.25000000	-0.19804497
243	2.25000000	1.50000000	-0.17419355
244	2.25000000	2.75000000	-0.11045943
245	1.75000000	2.50000000	-0.17653959
246	2.75000000	2.25000000	-0.08699902
247	2.50000000	1.75000000	-0.17419355
248	1.50000000	3.75000000	0.10498534
249	1.25000000	3.75000000	0.12531769
250	2.25000000	3.75000000	0.07800587
251	2.50000000	3.75000000	0.12805474
252	1.75000000	3.50000000	0.00645161
253	1.25000000	3.50000000	0.03382209
254	1.50000000	3.25000000	-0.05141740
255	2.25000000	3.50000000	0.04985337
256	1.75000000	3.25000000	-0.05806452
257	3.75000000	1.50000000	0.07487781
258	3.75000000	1.25000000	0.07878788
259	3.75000000	2.25000000	0.09677419
260	3.75000000	2.50000000	0.11124145
261	3.50000000	1.75000000	0.02209189
262	3.50000000	1.25000000	0.03929619
263	3.25000000	1.50000000	-0.03147605
264	3.50000000	2.25000000	0.02365591
265	3.25000000	1.75000000	-0.04477028
266	0.50000000	1.25000000	-0.17927664
267	0.75000000	1.25000000	-0.18748778
268	0.75000000	1.50000000	-0.18826979
269	1.25000000	0.50000000	-0.17693060
270	1.25000000	0.75000000	-0.18670577
271	1.50000000	0.75000000	-0.18748778
272	0.50000000	3.25000000	0.02561095
273	0.75000000	3.50000000	0.07292278
274	0.75000000	3.25000000	0.00684262
275	3.25000000	0.50000000	0.04907136
276	3.50000000	0.75000000	0.08113392
277	3.25000000	0.75000000	0.01114370
278	2.75000000	3.25000000	0.05298143
279	2.75000000	3.50000000	0.12453568
280	3.25000000	2.75000000	0.08152493
281	3.50000000	2.75000000	0.08817204
282	2.50000000	3.25000000	0.02756598
283	3.25000000	2.50000000	0.03186706
284	1.25000000	2.50000000	-0.15698925
285	1.25000000	2.75000000	-0.12688172
286	1.50000000	2.75000000	-0.14604106
287	2.50000000	1.25000000	-0.14838710
288	2.75000000	1.25000000	-0.12297165
289	2.75000000	1.50000000	-0.12375367
290	0.00000000	0.12500000	0.00527859
291	0.12500000	0.00000000	0.00645161
292	0.12500000	0.12500000	0.00136852

293	0.00000000	3.87500000	0.16324536
294	0.12500000	4.00000000	0.18631476
295	3.87500000	0.00000000	0.18162268
296	4.00000000	0.12500000	0.19765396
297	3.87500000	3.87500000	0.18005865
298	3.87500000	4.00000000	0.10146628
299	4.00000000	3.87500000	0.17536657
300	0.00000000	1.87500000	-0.13665689
301	0.00000000	2.12500000	-0.06666667
302	0.12500000	2.00000000	-0.14173998
303	0.12500000	2.12500000	-0.12922776
304	1.87500000	0.00000000	-0.01583578
305	2.12500000	0.00000000	-0.08817204
306	2.00000000	0.12500000	-0.13157380
307	2.12500000	0.12500000	-0.11515152
308	1.87500000	1.87500000	-0.19921799
309	2.12500000	2.12500000	-0.17419355
310	1.87500000	2.00000000	-0.19921799
311	2.00000000	1.87500000	-0.19921799
312	2.00000000	2.12500000	-0.19413490
313	2.12500000	2.00000000	-0.19960899
314	1.87500000	4.00000000	0.13587488
315	2.12500000	4.00000000	0.12609971
316	1.87500000	3.87500000	0.09012708
317	2.00000000	3.87500000	0.08699902
318	4.00000000	1.87500000	0.13470186
319	4.00000000	2.12500000	0.15229717
320	3.87500000	1.87500000	0.11476051
321	3.87500000	2.00000000	0.10811339
322	0.00000000	0.87500000	-0.10889541
323	0.00000000	1.12500000	-0.12883675
324	0.12500000	1.00000000	-0.13626588
325	0.12500000	1.12500000	-0.13665689
326	0.87500000	0.00000000	-0.05923754
327	1.12500000	0.00000000	-0.07057674
328	1.00000000	0.12500000	-0.08699902
329	1.12500000	0.12500000	-0.08739003
330	0.87500000	0.87500000	-0.16793744
331	1.12500000	1.12500000	-0.19608993
332	0.87500000	1.00000000	-0.19100684
333	1.00000000	0.87500000	-0.19022483
334	1.00000000	1.12500000	-0.19569892
335	1.12500000	1.00000000	-0.19569892
336	0.00000000	3.12500000	0.02052786
337	0.00000000	2.87500000	-0.01896383
338	0.12500000	3.12500000	0.02052786
339	0.12500000	3.00000000	-0.01231672
340	0.87500000	4.00000000	0.18592375
341	1.12500000	4.00000000	0.18866080
342	0.87500000	3.87500000	0.18748778
343	1.00000000	3.87500000	0.15307918
344	3.12500000	0.00000000	0.07292278
345	2.87500000	0.00000000	0.03304008
346	3.12500000	0.12500000	0.07096774
347	3.00000000	0.12500000	0.03577713
348	4.00000000	0.87500000	0.18553275
349	4.00000000	1.12500000	0.16598240
350	3.87500000	0.87500000	0.16715543
351	3.87500000	1.00000000	0.14838710

352	3.12500000	3.12500000	0.12375367
353	2.87500000	2.87500000	0.05063539
354	3.00000000	3.12500000	0.11280547
355	3.12500000	3.00000000	0.10967742
356	2.87500000	3.00000000	0.06432063
357	3.00000000	2.87500000	0.08152493
358	3.12500000	4.00000000	0.11984360
359	2.87500000	4.00000000	0.18866080
360	3.00000000	3.87500000	0.18670577
361	2.87500000	3.87500000	0.17458456
362	4.00000000	3.12500000	0.17536657
363	4.00000000	2.87500000	0.17771261
364	3.87500000	3.00000000	0.16598240
365	3.87500000	2.87500000	0.14134897
366	0.87500000	2.00000000	-0.19022483
367	1.12500000	2.00000000	-0.19882698
368	0.87500000	1.87500000	-0.19413490
369	1.00000000	1.87500000	-0.19804497
370	1.00000000	2.12500000	-0.18357771
371	1.12500000	2.12500000	-0.19296188
372	0.87500000	2.87500000	-0.09325513
373	1.12500000	3.12500000	-0.05493646
374	0.87500000	3.00000000	-0.06823069
375	1.00000000	3.12500000	-0.04477028
376	1.00000000	2.87500000	-0.09247312
377	1.12500000	3.00000000	-0.08739003
378	2.00000000	0.87500000	-0.19296188
379	2.00000000	1.12500000	-0.19921799
380	1.87500000	0.87500000	-0.19374389
381	1.87500000	1.00000000	-0.19843597
382	2.12500000	1.00000000	-0.18631476
383	2.12500000	1.12500000	-0.18670577
384	2.87500000	0.87500000	-0.12492669
385	3.12500000	1.12500000	-0.06392962
386	3.00000000	0.87500000	-0.07800587
387	3.12500000	1.00000000	-0.06197458
388	2.87500000	1.00000000	-0.10967742
389	3.00000000	1.12500000	-0.13470186
390	2.00000000	2.87500000	-0.15933529
391	2.00000000	3.12500000	-0.09012708
392	2.12500000	3.00000000	-0.12649071
393	2.12500000	3.12500000	-0.09051808
394	1.87500000	2.87500000	-0.15933529
395	1.87500000	3.00000000	-0.14408602
396	2.87500000	2.00000000	-0.12453568
397	3.12500000	2.00000000	-0.07214076
398	3.00000000	2.12500000	-0.06823069
399	3.12500000	2.12500000	-0.06510264
400	2.87500000	1.87500000	-0.11163245
401	3.00000000	1.87500000	-0.09912023
402	0.00000000	0.37500000	-0.04203324
403	0.00000000	0.62500000	-0.08230694
404	0.12500000	0.50000000	-0.07292278
405	0.12500000	0.62500000	-0.09638319
406	0.37500000	0.00000000	-0.03929619
407	0.62500000	0.00000000	-0.06314761
408	0.50000000	0.12500000	-0.07018573
409	0.62500000	0.12500000	-0.09286413
410	0.37500000	0.37500000	-0.05454545

411	0.62500000	0.62500000	-0.11319648
412	0.37500000	0.50000000	-0.08152493
413	0.50000000	0.37500000	-0.08035191
414	0.50000000	0.62500000	-0.10889541
415	0.62500000	0.50000000	-0.10811339
416	0.00000000	3.62500000	0.11554252
417	0.00000000	3.37500000	0.06823069
418	0.12500000	3.62500000	0.11515152
419	0.12500000	3.50000000	0.09169110
420	0.37500000	4.00000000	0.18553275
421	0.62500000	4.00000000	0.18514174
422	0.37500000	3.87500000	0.16168133
423	0.50000000	3.87500000	0.16129032
424	3.62500000	0.00000000	0.14565005
425	3.37500000	0.00000000	0.10928641
426	3.62500000	0.12500000	0.14330401
427	3.50000000	0.12500000	0.12531769
428	4.00000000	0.37500000	0.19374389
429	4.00000000	0.62500000	0.18944282
430	3.87500000	0.37500000	0.17536657
431	3.87500000	0.50000000	0.17341153
432	3.62500000	3.62500000	0.18944282
433	3.37500000	3.37500000	0.17067449
434	3.50000000	3.62500000	0.11593353
435	3.62500000	3.50000000	0.18944282
436	3.37500000	3.50000000	0.18279570
437	3.50000000	3.37500000	0.18005865
438	3.62500000	4.00000000	-0.04555230
439	3.37500000	4.00000000	-0.03968719
440	3.50000000	3.87500000	-0.04086022
441	3.37500000	3.87500000	-0.05219941
442	4.00000000	3.62500000	0.17536657
443	4.00000000	3.37500000	0.17536657
444	3.87500000	3.50000000	0.18005865
445	3.87500000	3.37500000	0.16598240
446	0.00000000	1.62500000	-0.14369501
447	0.00000000	1.37500000	-0.14134897
448	0.12500000	1.62500000	-0.15229717
449	0.12500000	1.50000000	-0.15581623
450	0.00000000	2.37500000	-0.03264907
451	0.00000000	2.62500000	-0.04985337
452	0.12500000	2.50000000	-0.08426197
453	0.12500000	2.62500000	-0.05923754
454	0.37500000	2.00000000	-0.15777126
455	0.62500000	2.00000000	-0.17419355
456	0.37500000	1.87500000	-0.16168133
457	0.50000000	2.12500000	-0.15972630
458	0.50000000	1.87500000	-0.16950147
459	0.62500000	2.12500000	-0.15894428
460	0.37500000	2.37500000	-0.13235582
461	0.62500000	2.62500000	-0.12453568
462	0.37500000	2.50000000	-0.12179863
463	0.50000000	2.37500000	-0.14682307
464	0.50000000	2.62500000	-0.11554252
465	0.62500000	2.50000000	-0.12649071
466	1.62500000	0.00000000	-0.05884653
467	1.37500000	0.00000000	0.07761486
468	1.62500000	0.12500000	-0.12688172
469	1.50000000	0.12500000	-0.12766373

470	2.37500000	0.00000000	-0.04516129
471	2.62500000	0.00000000	-0.00997067
472	2.50000000	0.12500000	-0.05063539
473	2.62500000	0.12500000	-0.02873900
474	2.00000000	0.37500000	-0.15972630
475	2.00000000	0.62500000	-0.18044966
476	1.87500000	0.37500000	-0.15855327
477	2.12500000	0.50000000	-0.15777126
478	1.87500000	0.50000000	-0.17497556
479	2.12500000	0.62500000	-0.16754643
480	2.37500000	0.37500000	-0.11085044
481	2.62500000	0.62500000	-0.13079179
482	2.50000000	0.37500000	-0.08934506
483	2.37500000	0.50000000	-0.12492669
484	2.62500000	0.50000000	-0.08699902
485	2.50000000	0.62500000	-0.11867058
486	1.62500000	1.62500000	-0.19804497
487	1.37500000	1.37500000	-0.19726295
488	1.50000000	1.62500000	-0.19804497
489	1.62500000	1.50000000	-0.19804497
490	1.37500000	1.50000000	-0.19726295
491	1.50000000	1.37500000	-0.19726295
492	2.37500000	2.37500000	-0.12336266
493	2.62500000	2.62500000	-0.04868035
494	2.37500000	2.50000000	-0.11788856
495	2.50000000	2.37500000	-0.12336266
496	2.50000000	2.62500000	-0.08465298
497	2.62500000	2.50000000	-0.06744868
498	1.62500000	2.00000000	-0.19921799
499	1.37500000	2.00000000	-0.19921799
500	1.50000000	1.87500000	-0.19882698
501	1.62500000	2.12500000	-0.19335288
502	1.37500000	1.87500000	-0.19843597
503	1.50000000	2.12500000	-0.19335288
504	2.00000000	1.62500000	-0.19921799
505	2.00000000	1.37500000	-0.19921799
506	1.87500000	1.50000000	-0.19882698
507	2.12500000	1.62500000	-0.19921799
508	1.87500000	1.37500000	-0.19843597
509	2.12500000	1.50000000	-0.18670577
510	2.00000000	2.37500000	-0.18279570
511	2.00000000	2.62500000	-0.17106549
512	2.12500000	2.50000000	-0.15698925
513	1.87500000	2.37500000	-0.18240469
514	2.12500000	2.62500000	-0.14369501
515	1.87500000	2.50000000	-0.17693060
516	2.37500000	2.00000000	-0.19960899
517	2.62500000	2.00000000	-0.17458456
518	2.50000000	2.12500000	-0.17419355
519	2.37500000	1.87500000	-0.19921799
520	2.62500000	2.12500000	-0.14330401
521	2.50000000	1.87500000	-0.18670577
522	1.62500000	4.00000000	0.17614858
523	1.37500000	4.00000000	0.19374389
524	1.50000000	3.87500000	0.15073314
525	1.37500000	3.87500000	0.16089932
526	2.37500000	4.00000000	0.14643206
527	2.62500000	4.00000000	0.16754643
528	2.37500000	3.87500000	0.11749756

529	2.50000000	3.87500000	0.14252199
530	1.62500000	3.62500000	0.03890518
531	1.37500000	3.37500000	-0.00957967
532	1.50000000	3.62500000	0.05923754
533	1.62500000	3.50000000	0.00997067
534	1.37500000	3.50000000	0.02365591
535	1.50000000	3.37500000	-0.01896383
536	2.00000000	3.62500000	0.02873900
537	2.00000000	3.37500000	-0.01348974
538	2.12500000	3.62500000	0.03890518
539	1.87500000	3.50000000	0.00332356
540	2.12500000	3.50000000	0.02482893
541	1.87500000	3.37500000	-0.02913001
542	4.00000000	1.62500000	0.12140762
543	4.00000000	1.37500000	0.13157380
544	3.87500000	1.50000000	0.09442815
545	3.87500000	1.37500000	0.09677419
546	4.00000000	2.37500000	0.17380254
547	4.00000000	2.62500000	0.18240469
548	3.87500000	2.37500000	0.14056696
549	3.87500000	2.50000000	0.14799609
550	3.62500000	1.62500000	0.06158358
551	3.37500000	1.37500000	0.00215054
552	3.62500000	1.50000000	0.05493646
553	3.50000000	1.62500000	0.02834800
554	3.50000000	1.37500000	0.03734115
555	3.37500000	1.50000000	0.00175953
556	3.62500000	2.00000000	0.04164223
557	3.37500000	2.00000000	-0.01818182
558	3.62500000	2.12500000	0.05298143
559	3.50000000	1.87500000	0.01544477
560	3.50000000	2.12500000	0.01622678
561	3.37500000	1.87500000	-0.01779081
562	0.37500000	1.00000000	-0.16441838
563	0.62500000	1.00000000	-0.18279570
564	0.37500000	0.87500000	-0.15112414
565	0.50000000	0.87500000	-0.15503421
566	0.50000000	1.12500000	-0.17888563
567	0.62500000	1.12500000	-0.18279570
568	0.37500000	1.37500000	-0.16559140
569	0.62500000	1.62500000	-0.18475073
570	0.37500000	1.50000000	-0.17184751
571	0.50000000	1.62500000	-0.17653959
572	0.50000000	1.37500000	-0.17966764
573	0.62500000	1.50000000	-0.18396872
574	1.00000000	0.37500000	-0.14643206
575	1.00000000	0.62500000	-0.18084066
576	0.87500000	0.37500000	-0.14838710
577	0.87500000	0.50000000	-0.15347019
578	1.12500000	0.50000000	-0.17653959
579	1.12500000	0.62500000	-0.18123167
580	1.37500000	0.37500000	-0.14760508
581	1.62500000	0.62500000	-0.18318671
582	1.50000000	0.37500000	-0.16129032
583	1.62500000	0.50000000	-0.17693060
584	1.37500000	0.50000000	-0.17732160
585	1.50000000	0.62500000	-0.18279570
586	1.00000000	1.37500000	-0.19648094
587	1.00000000	1.62500000	-0.19726295

588	1.12500000	1.50000000	-0.19726295
589	1.12500000	1.62500000	-0.19765396
590	0.87500000	1.37500000	-0.19217986
591	0.87500000	1.50000000	-0.19257087
592	1.37500000	1.00000000	-0.19648094
593	1.62500000	1.00000000	-0.19765396
594	1.50000000	1.12500000	-0.19726295
595	1.62500000	1.12500000	-0.19765396
596	1.37500000	0.87500000	-0.19178886
597	1.50000000	0.87500000	-0.19217986
598	0.37500000	3.37500000	0.06783969
599	0.62500000	3.62500000	0.13978495
600	0.37500000	3.50000000	0.09169110
601	0.50000000	3.62500000	0.11476051
602	0.50000000	3.37500000	0.05845552
603	0.62500000	3.50000000	0.08191593
604	0.37500000	3.00000000	-0.03108504
605	0.62500000	3.00000000	-0.04985337
606	0.37500000	2.87500000	-0.04672532
607	0.50000000	2.87500000	-0.06549365
608	0.50000000	3.12500000	-0.00723363
609	0.62500000	3.12500000	-0.01661779
610	1.00000000	3.62500000	0.08739003
611	1.00000000	3.37500000	0.02130987
612	1.12500000	3.62500000	0.08973607
613	1.12500000	3.50000000	0.04398827
614	0.87500000	3.50000000	0.06353861
615	0.87500000	3.37500000	0.03030303
616	3.37500000	0.37500000	0.10303030
617	3.62500000	0.62500000	0.13509286
618	3.50000000	0.37500000	0.12140762
619	3.62500000	0.50000000	0.13743891
620	3.37500000	0.50000000	0.08387097
621	3.50000000	0.62500000	0.10029326
622	3.00000000	0.37500000	-0.00215054
623	3.00000000	0.62500000	-0.04007820
624	2.87500000	0.37500000	-0.02365591
625	2.87500000	0.50000000	-0.04320626
626	3.12500000	0.50000000	0.01388074
627	3.12500000	0.62500000	-0.00488759
628	3.62500000	1.00000000	0.07800587
629	3.37500000	1.00000000	0.00840665
630	3.62500000	1.12500000	0.06119257
631	3.50000000	1.12500000	0.04125122
632	3.50000000	0.87500000	0.06236559
633	3.37500000	0.87500000	0.02717498
634	3.00000000	3.37500000	0.13743891
635	3.00000000	3.62500000	0.16207234
636	3.12500000	3.50000000	0.16089932
637	3.12500000	3.62500000	0.08230694
638	2.87500000	3.37500000	0.10146628
639	2.87500000	3.50000000	0.13704790
640	3.37500000	3.00000000	0.12844575
641	3.62500000	3.00000000	0.14721408
642	3.50000000	3.12500000	0.15190616
643	3.62500000	3.12500000	0.14721408
644	3.37500000	2.87500000	0.10967742
645	3.50000000	2.87500000	0.11319648
646	2.62500000	3.00000000	-0.00801564

647	2.37500000	3.00000000	-0.07135875
648	2.50000000	2.87500000	-0.05767351
649	2.37500000	2.87500000	-0.07722385
650	2.62500000	3.12500000	0.00449658
651	2.50000000	3.12500000	-0.00801564
652	3.00000000	2.62500000	0.04398827
653	3.00000000	2.37500000	-0.00566960
654	2.87500000	2.50000000	-0.00566960
655	2.87500000	2.37500000	-0.03069404
656	3.12500000	2.62500000	0.05337243
657	3.12500000	2.50000000	0.02873900
658	2.62500000	3.62500000	0.12453568
659	2.37500000	3.37500000	0.03616813
660	2.50000000	3.62500000	0.11397849
661	2.37500000	3.50000000	0.07487781
662	2.62500000	3.50000000	0.11202346
663	2.50000000	3.37500000	0.06353861
664	3.62500000	2.62500000	0.07253177
665	3.37500000	2.37500000	0.00371457
666	3.62500000	2.50000000	0.07487781
667	3.50000000	2.37500000	0.03069404
668	3.50000000	2.62500000	0.06314761
669	3.37500000	2.50000000	0.03499511
670	1.00000000	2.37500000	-0.15307918
671	1.00000000	2.62500000	-0.12258065
672	0.87500000	2.37500000	-0.14486804
673	0.87500000	2.50000000	-0.13861193
674	1.12500000	2.50000000	-0.14721408
675	1.12500000	2.62500000	-0.13235582
676	1.37500000	2.37500000	-0.18162268
677	1.62500000	2.62500000	-0.17028348
678	1.50000000	2.37500000	-0.18162268
679	1.62500000	2.50000000	-0.17614858
680	1.37500000	2.50000000	-0.16637341
681	1.50000000	2.62500000	-0.16089932
682	1.37500000	3.00000000	-0.10615836
683	1.62500000	3.00000000	-0.12531769
684	1.50000000	3.12500000	-0.08347996
685	1.62500000	3.12500000	-0.08699902
686	1.37500000	2.87500000	-0.12140762
687	1.50000000	2.87500000	-0.13079179
688	2.37500000	1.00000000	-0.16050831
689	2.62500000	1.00000000	-0.13548387
690	2.37500000	0.87500000	-0.15464321
691	2.50000000	0.87500000	-0.13822092
692	2.50000000	1.12500000	-0.14799609
693	2.62500000	1.12500000	-0.13548387
694	2.37500000	1.37500000	-0.16168133
695	2.62500000	1.62500000	-0.13665689
696	2.37500000	1.50000000	-0.16168133
697	2.50000000	1.62500000	-0.16168133
698	2.50000000	1.37500000	-0.14877810
699	2.62500000	1.50000000	-0.13626588
700	3.00000000	1.37500000	-0.13509286
701	3.00000000	1.62500000	-0.09833822
702	3.12500000	1.50000000	-0.06471163
703	3.12500000	1.62500000	-0.07135875
704	2.87500000	1.37500000	-0.11045943
705	2.87500000	1.50000000	-0.11085044

706	0.12500000	0.25000000	-0.01935484
707	0.12500000	0.37500000	-0.04633431
708	0.25000000	0.12500000	-0.01857283
709	0.37500000	0.12500000	-0.04437928
710	0.25000000	0.37500000	-0.05024438
711	0.37500000	0.25000000	-0.04946237
712	0.12500000	3.87500000	0.16285435
713	0.12500000	3.75000000	0.13900293
714	0.25000000	3.87500000	0.16207234
715	3.87500000	0.12500000	0.17966764
716	3.75000000	0.12500000	0.16168133
717	3.87500000	0.25000000	0.17771261
718	3.75000000	3.87500000	0.10654936
719	3.87500000	3.75000000	0.18005865
720	3.62500000	3.75000000	0.11124145
721	3.75000000	3.62500000	0.18475073
722	3.62500000	3.87500000	0.03264907
723	3.87500000	3.62500000	0.18005865
724	0.12500000	1.87500000	-0.14447703
725	0.12500000	1.75000000	-0.14838710
726	0.12500000	2.25000000	-0.06197458
727	0.12500000	2.37500000	-0.05102639
728	0.25000000	2.12500000	-0.13704790
729	0.25000000	1.87500000	-0.15307918
730	0.37500000	2.12500000	-0.15151515
731	0.25000000	2.37500000	-0.11358749
732	0.37500000	2.25000000	-0.13900293
733	1.87500000	0.12500000	-0.02991202
734	1.75000000	0.12500000	-0.02873900
735	2.25000000	0.12500000	-0.08621701
736	2.37500000	0.12500000	-0.06432063
737	2.12500000	0.25000000	-0.12922776
738	1.87500000	0.25000000	-0.14408602
739	2.12500000	0.37500000	-0.14330401
740	2.37500000	0.25000000	-0.09169110
741	2.25000000	0.37500000	-0.12727273
742	1.75000000	1.87500000	-0.19882698
743	1.87500000	1.75000000	-0.19882698
744	1.62500000	1.75000000	-0.19843597
745	1.75000000	1.62500000	-0.19843597
746	2.12500000	2.25000000	-0.16871945
747	2.25000000	2.12500000	-0.17419355
748	2.25000000	2.37500000	-0.14330401
749	2.37500000	2.25000000	-0.14877810
750	1.87500000	2.12500000	-0.19374389
751	1.62500000	1.87500000	-0.19882698
752	1.75000000	2.12500000	-0.19335288
753	2.12500000	1.87500000	-0.19921799
754	1.87500000	1.62500000	-0.19882698
755	2.12500000	1.75000000	-0.19921799
756	2.12500000	2.37500000	-0.16285435
757	1.87500000	2.25000000	-0.18826979
758	2.37500000	2.12500000	-0.17419355
759	2.25000000	1.87500000	-0.19921799
760	1.75000000	3.87500000	0.11045943
761	1.62500000	3.87500000	0.13040078
762	2.12500000	3.87500000	0.09716520
763	2.25000000	3.87500000	0.10733138
764	1.87500000	3.75000000	0.06119257

765	1.62500000	3.75000000	0.08465298
766	1.75000000	3.62500000	0.03577713
767	2.12500000	3.75000000	0.06823069
768	1.87500000	3.62500000	0.03225806
769	3.87500000	1.75000000	0.10811339
770	3.87500000	1.62500000	0.10146628
771	3.87500000	2.12500000	0.11906158
772	3.87500000	2.25000000	0.13000978
773	3.75000000	1.87500000	0.08152493
774	3.75000000	1.62500000	0.08152493
775	3.62500000	1.75000000	0.05532747
776	3.75000000	2.12500000	0.08582600
777	3.62500000	1.87500000	0.04828935
778	0.12500000	0.87500000	-0.12297165
779	0.12500000	0.75000000	-0.10967742
780	0.12500000	1.25000000	-0.14330401
781	0.12500000	1.37500000	-0.14916911
782	0.25000000	1.12500000	-0.15073314
783	0.25000000	0.87500000	-0.13704790
784	0.37500000	1.12500000	-0.16480938
785	0.25000000	1.37500000	-0.15738025
786	0.37500000	1.25000000	-0.16520039
787	0.87500000	0.12500000	-0.08895406
788	0.75000000	0.12500000	-0.09090909
789	1.25000000	0.12500000	-0.10068426
790	1.37500000	0.12500000	-0.11436950
791	1.12500000	0.25000000	-0.11710655
792	0.87500000	0.25000000	-0.11867058
793	1.12500000	0.37500000	-0.14682307
794	1.37500000	0.25000000	-0.13079179
795	1.25000000	0.37500000	-0.14721408
796	0.75000000	0.87500000	-0.16363636
797	0.87500000	0.75000000	-0.16285435
798	0.62500000	0.75000000	-0.13626588
799	0.75000000	0.62500000	-0.13548387
800	1.12500000	1.25000000	-0.19608993
801	1.25000000	1.12500000	-0.19608993
802	1.25000000	1.37500000	-0.19687195
803	1.37500000	1.25000000	-0.19687195
804	0.87500000	1.12500000	-0.19139785
805	0.62500000	0.87500000	-0.15933529
806	0.75000000	1.12500000	-0.18709677
807	1.12500000	0.87500000	-0.19100684
808	0.87500000	0.62500000	-0.15816227
809	1.12500000	0.75000000	-0.18592375
810	1.12500000	1.37500000	-0.19648094
811	0.87500000	1.25000000	-0.19178886
812	1.37500000	1.12500000	-0.19648094
813	1.25000000	0.87500000	-0.19139785
814	0.12500000	3.25000000	0.04437928
815	0.12500000	3.37500000	0.06823069
816	0.12500000	2.87500000	-0.02717498
817	0.12500000	2.75000000	-0.13431085
818	0.25000000	3.12500000	0.10967742
819	0.25000000	3.37500000	0.07096774
820	0.37500000	3.25000000	0.04907136
821	0.25000000	2.87500000	-0.00997067
822	0.37500000	3.12500000	0.00215054
823	0.75000000	3.87500000	0.18709677

824	0.62500000	3.87500000	0.16168133
825	1.12500000	3.87500000	0.15581623
826	1.25000000	3.87500000	0.15855327
827	0.87500000	3.75000000	0.15464321
828	0.62500000	3.75000000	0.16324536
829	0.75000000	3.62500000	0.13079179
830	1.12500000	3.75000000	0.12297165
831	0.87500000	3.62500000	0.09638319
832	3.25000000	0.12500000	0.08895406
833	3.37500000	0.12500000	0.10733138
834	2.87500000	0.12500000	0.01427175
835	2.75000000	0.12500000	-0.00762463
836	3.12500000	0.25000000	0.05180841
837	3.37500000	0.25000000	0.10537634
838	3.25000000	0.37500000	0.06823069
839	2.87500000	0.25000000	-0.00488759
840	3.12500000	0.37500000	0.03264907
841	3.87500000	0.75000000	0.16950147
842	3.87500000	0.62500000	0.17145650
843	3.87500000	1.12500000	0.13118280
844	3.87500000	1.25000000	0.11397849
845	3.75000000	0.87500000	0.13196481
846	3.75000000	0.62500000	0.15347019
847	3.62500000	0.75000000	0.11632454
848	3.75000000	1.12500000	0.09599218
849	3.62500000	0.87500000	0.09716520
850	3.12500000	3.25000000	0.13587488
851	3.25000000	3.12500000	0.13313783
852	3.25000000	3.37500000	0.15933529
853	3.37500000	3.25000000	0.15659824
854	2.75000000	2.87500000	0.01466276
855	2.87500000	2.75000000	0.03186706
856	2.62500000	2.75000000	-0.03499511
857	2.75000000	2.62500000	-0.01779081
858	2.87500000	3.12500000	0.07644184
859	3.12500000	3.37500000	0.14838710
860	2.87500000	3.25000000	0.08895406
861	3.12500000	2.87500000	0.09090909
862	3.37500000	3.12500000	0.14252199
863	3.25000000	2.87500000	0.10029326
864	2.62500000	2.87500000	-0.02130987
865	2.75000000	3.12500000	0.04046921
866	2.87500000	2.62500000	0.01309873
867	3.12500000	2.75000000	0.07214076
868	3.12500000	3.87500000	0.10733138
869	3.25000000	3.87500000	0.02756598
870	2.75000000	3.87500000	0.16402737
871	2.62500000	3.87500000	0.15307918
872	2.87500000	3.75000000	0.16207234
873	3.12500000	3.75000000	0.09481916
874	2.87500000	3.62500000	0.14956012
875	2.62500000	3.75000000	0.13900293
876	2.75000000	3.62500000	0.13704790
877	3.87500000	3.12500000	0.16598240
878	3.87500000	3.25000000	0.16598240
879	3.87500000	2.75000000	0.14369501
880	3.87500000	2.62500000	0.14565005
881	3.75000000	2.87500000	0.13196481
882	3.75000000	3.12500000	0.15659824

883	3.62500000	2.87500000	0.12258065
884	3.75000000	2.62500000	0.10928641
885	3.62500000	2.75000000	0.09755621
886	0.75000000	1.87500000	-0.18592375
887	0.87500000	2.12500000	-0.17536657
888	0.62500000	1.87500000	-0.17771261
889	0.75000000	2.12500000	-0.16715543
890	1.12500000	1.87500000	-0.19804497
891	1.25000000	2.12500000	-0.19335288
892	1.25000000	1.87500000	-0.19843597
893	1.37500000	2.12500000	-0.19335288
894	0.87500000	1.75000000	-0.19335288
895	0.62500000	1.75000000	-0.18123167
896	0.75000000	1.62500000	-0.18905181
897	1.12500000	1.75000000	-0.19804497
898	0.87500000	1.62500000	-0.19296188
899	1.12500000	2.25000000	-0.17771261
900	0.87500000	2.25000000	-0.16011730
901	1.12500000	2.37500000	-0.16246334
902	1.37500000	2.25000000	-0.18748778
903	1.25000000	2.37500000	-0.17223851
904	0.75000000	2.87500000	-0.08426197
905	0.87500000	2.75000000	-0.10811339
906	0.62500000	2.75000000	-0.09951124
907	0.75000000	2.62500000	-0.12414467
908	1.12500000	3.25000000	-0.02209189
909	1.25000000	3.12500000	-0.06432063
910	1.25000000	3.37500000	0.00097752
911	1.37500000	3.25000000	-0.04164223
912	0.87500000	3.12500000	-0.03538612
913	0.62500000	2.87500000	-0.07487781
914	0.75000000	3.12500000	-0.02600196
915	1.12500000	3.37500000	0.01114370
916	0.87500000	3.25000000	-0.00254154
917	1.12500000	2.87500000	-0.10224829
918	0.87500000	2.62500000	-0.12336266
919	1.12500000	2.75000000	-0.11710655
920	1.37500000	3.12500000	-0.07409580
921	1.25000000	2.87500000	-0.11202346
922	1.87500000	0.75000000	-0.18748778
923	2.12500000	0.87500000	-0.18005865
924	1.87500000	0.62500000	-0.18123167
925	2.12500000	0.75000000	-0.17380254
926	1.87500000	1.12500000	-0.19843597
927	2.12500000	1.25000000	-0.18670577
928	1.87500000	1.25000000	-0.19843597
929	2.12500000	1.37500000	-0.18670577
930	1.75000000	0.87500000	-0.19335288
931	1.75000000	0.62500000	-0.18240469
932	1.62500000	0.75000000	-0.18787879
933	1.75000000	1.12500000	-0.19804497
934	1.62500000	0.87500000	-0.19296188
935	2.25000000	1.12500000	-0.17380254
936	2.25000000	0.87500000	-0.19217986
937	2.37500000	1.12500000	-0.16089932
938	2.25000000	1.37500000	-0.17419355
939	2.37500000	1.25000000	-0.16129032
940	2.87500000	0.75000000	-0.10615836
941	2.75000000	0.87500000	-0.13782991

942	2.75000000	0.62500000	-0.10889541
943	2.62500000	0.75000000	-0.14056696
944	3.25000000	1.12500000	-0.02873900
945	3.12500000	1.25000000	-0.10146628
946	3.37500000	1.25000000	0.00410557
947	3.25000000	1.37500000	-0.03108504
948	3.12500000	0.87500000	-0.04281525
949	2.87500000	0.62500000	-0.06236559
950	3.12500000	0.75000000	-0.02404692
951	3.37500000	1.12500000	0.00645161
952	3.25000000	0.87500000	-0.00762463
953	2.87500000	1.12500000	-0.14721408
954	2.62500000	0.87500000	-0.12531769
955	2.75000000	1.12500000	-0.12258065
956	3.12500000	1.37500000	-0.10185728
957	2.87500000	1.25000000	-0.14760508
958	2.12500000	2.87500000	-0.13196481
959	1.87500000	2.75000000	-0.16520039
960	2.12500000	2.75000000	-0.13782991
961	1.87500000	2.62500000	-0.17106549
962	2.12500000	3.25000000	-0.02678397
963	1.87500000	3.12500000	-0.08074291
964	2.12500000	3.37500000	0.01153470
965	1.87500000	3.25000000	-0.04242424
966	2.25000000	3.12500000	-0.06275660
967	2.25000000	2.87500000	-0.10459433
968	2.37500000	3.12500000	-0.03538612
969	2.25000000	3.37500000	0.01153470
970	2.37500000	3.25000000	0.00019550
971	1.75000000	2.87500000	-0.14995112
972	1.75000000	2.62500000	-0.17067449
973	1.62500000	2.75000000	-0.15542522
974	1.75000000	3.12500000	-0.09638319
975	1.62500000	2.87500000	-0.14017595
976	2.87500000	2.12500000	-0.09325513
977	2.75000000	1.87500000	-0.13665689
978	2.75000000	2.12500000	-0.11827957
979	2.62500000	1.87500000	-0.16168133
980	3.25000000	2.12500000	-0.03773216
981	3.12500000	1.87500000	-0.07174976
982	3.37500000	2.12500000	-0.01075269
983	3.25000000	1.87500000	-0.04477028
984	3.12500000	2.25000000	-0.03382209
985	2.87500000	2.25000000	-0.06197458
986	3.12500000	2.37500000	-0.00254154
987	3.37500000	2.25000000	-0.00371457
988	3.25000000	2.37500000	0.00058651
989	2.87500000	1.75000000	-0.11124145
990	2.62500000	1.75000000	-0.14916911
991	2.75000000	1.62500000	-0.12375367
992	3.12500000	1.75000000	-0.07174976
993	2.87500000	1.62500000	-0.11124145
994	0.25000000	0.62500000	-0.10029326
995	0.37500000	0.62500000	-0.10459433
996	0.37500000	0.75000000	-0.12766373
997	0.62500000	0.25000000	-0.09794721
998	0.62500000	0.37500000	-0.10303030
999	0.75000000	0.37500000	-0.12570870
1000	0.25000000	3.62500000	0.11515152

1001	0.37500000	3.75000000	0.13822092
1002	0.37500000	3.62500000	0.11476051
1003	3.62500000	0.25000000	0.14134897
1004	3.75000000	0.37500000	0.15738025
1005	3.62500000	0.37500000	0.13939394
1006	3.37500000	3.62500000	0.10459433
1007	3.37500000	3.75000000	0.02600196
1008	3.62500000	3.37500000	0.17536657
1009	3.75000000	3.37500000	0.17067449
1010	3.25000000	3.62500000	0.09364614
1011	3.62500000	3.25000000	0.16129032
1012	0.25000000	1.62500000	-0.16011730
1013	0.37500000	1.75000000	-0.16480938
1014	0.37500000	1.62500000	-0.16832845
1015	0.25000000	2.62500000	-0.07800587
1016	0.37500000	2.62500000	-0.09677419
1017	0.37500000	2.75000000	-0.07174976
1018	0.62500000	2.25000000	-0.15268817
1019	0.62500000	2.37500000	-0.14643206
1020	0.75000000	2.37500000	-0.14565005
1021	1.62500000	0.25000000	-0.14369501
1022	1.75000000	0.37500000	-0.15933529
1023	1.62500000	0.37500000	-0.16050831
1024	2.62500000	0.25000000	-0.04828935
1025	2.62500000	0.37500000	-0.06744868
1026	2.75000000	0.37500000	-0.04594330
1027	2.25000000	0.62500000	-0.15112414
1028	2.37500000	0.62500000	-0.13470186
1029	2.37500000	0.75000000	-0.14447703
1030	1.37500000	1.62500000	-0.19804497
1031	1.37500000	1.75000000	-0.19804497
1032	1.62500000	1.37500000	-0.19804497
1033	1.75000000	1.37500000	-0.19804497
1034	1.25000000	1.62500000	-0.19765396
1035	1.62500000	1.25000000	-0.19765396
1036	2.37500000	2.62500000	-0.10420332
1037	2.25000000	2.62500000	-0.12375367
1038	2.62500000	2.37500000	-0.09247312
1039	2.62500000	2.25000000	-0.11788856
1040	2.37500000	2.75000000	-0.09051808
1041	2.75000000	2.37500000	-0.06158358
1042	1.62500000	2.25000000	-0.18748778
1043	1.75000000	2.37500000	-0.18201369
1044	1.62500000	2.37500000	-0.18201369
1045	2.25000000	1.62500000	-0.18670577
1046	2.37500000	1.75000000	-0.18670577
1047	2.37500000	1.62500000	-0.17419355
1048	1.37500000	3.75000000	0.11515152
1049	1.37500000	3.62500000	0.06940371
1050	1.25000000	3.62500000	0.07956989
1051	2.37500000	3.75000000	0.10303030
1052	2.25000000	3.62500000	0.06392962
1053	2.37500000	3.62500000	0.08895406
1054	1.62500000	3.37500000	-0.02248289
1055	1.75000000	3.37500000	-0.02561095
1056	1.62500000	3.25000000	-0.05454545
1057	3.75000000	1.37500000	0.07683284
1058	3.62500000	1.37500000	0.05728250
1059	3.62500000	1.25000000	0.05923754

1060	3.75000000	2.37500000	0.10420332
1061	3.62500000	2.25000000	0.06041056
1062	3.62500000	2.37500000	0.06744868
1063	3.37500000	1.62500000	-0.00449658
1064	3.37500000	1.75000000	-0.01153470
1065	3.25000000	1.62500000	-0.03812317
1066	0.62500000	1.25000000	-0.18357771
1067	0.62500000	1.37500000	-0.18357771
1068	0.75000000	1.37500000	-0.18787879
1069	1.25000000	0.62500000	-0.18162268
1070	1.37500000	0.62500000	-0.18201369
1071	1.37500000	0.75000000	-0.18709677
1072	0.62500000	3.37500000	0.04946237
1073	0.62500000	3.25000000	0.01622678
1074	0.75000000	3.37500000	0.03968719
1075	3.37500000	0.62500000	0.06510264
1076	3.25000000	0.62500000	0.02991202
1077	3.37500000	0.75000000	0.04633431
1078	2.75000000	3.37500000	0.08856305
1079	2.62500000	3.25000000	0.04046921
1080	2.62500000	3.37500000	0.07605083
1081	3.37500000	2.75000000	0.08504399
1082	3.25000000	2.62500000	0.05689150
1083	3.37500000	2.62500000	0.06001955
1084	1.25000000	2.62500000	-0.14173998
1085	1.37500000	2.62500000	-0.15151515
1086	1.37500000	2.75000000	-0.13626588
1087	2.62500000	1.25000000	-0.13548387
1088	2.62500000	1.37500000	-0.13587488
1089	2.75000000	1.37500000	-0.12336266
1090	0.00000000	0.06250000	0.00273705
1091	0.06250000	0.00000000	0.00332356
1092	0.06250000	0.06250000	0.00078201
1093	0.00000000	3.93750000	0.17497556
1094	0.06250000	4.00000000	0.18651026
1095	3.93750000	0.00000000	0.19061584
1096	4.00000000	0.06250000	0.19863148
1097	3.93750000	3.93750000	0.17751711
1098	3.93750000	4.00000000	0.13822092
1099	4.00000000	3.93750000	0.17517107
1100	0.00000000	1.93750000	-0.13528837
1101	0.00000000	2.06250000	-0.10029326
1102	0.06250000	2.00000000	-0.13782991
1103	0.06250000	2.06250000	-0.13157380
1104	1.93750000	0.00000000	-0.06666667
1105	2.06250000	0.00000000	-0.10283480
1106	2.00000000	0.06250000	-0.12453568
1107	2.06250000	0.06250000	-0.11632454
1108	1.93750000	1.93750000	-0.19941349
1109	2.06250000	2.06250000	-0.18690127
1110	1.93750000	2.00000000	-0.19941349
1111	2.00000000	1.93750000	-0.19941349
1112	2.00000000	2.06250000	-0.19687195
1113	2.06250000	2.00000000	-0.19960899
1114	1.93750000	4.00000000	0.12590420
1115	2.06250000	4.00000000	0.12101662
1116	1.93750000	3.93750000	0.10303030
1117	2.00000000	3.93750000	0.10146628
1118	4.00000000	1.93750000	0.13802542

1119	4.00000000	2.06250000	0.14682307
1120	3.93750000	1.93750000	0.12805474
1121	3.93750000	2.00000000	0.12473118
1122	0.00000000	0.93750000	-0.11573803
1123	0.00000000	1.06250000	-0.12570870
1124	0.06250000	1.00000000	-0.12942326
1125	0.06250000	1.06250000	-0.12961877
1126	0.93750000	0.00000000	-0.05826002
1127	1.06250000	0.00000000	-0.06392962
1128	1.00000000	0.06250000	-0.07214076
1129	1.06250000	0.06250000	-0.07233627
1130	0.93750000	0.93750000	-0.18162268
1131	1.06250000	1.06250000	-0.19569892
1132	0.93750000	1.00000000	-0.19315738
1133	1.00000000	0.93750000	-0.19276637
1134	1.00000000	1.06250000	-0.19550342
1135	1.06250000	1.00000000	-0.19550342
1136	0.00000000	3.06250000	0.00860215
1137	0.00000000	2.93750000	-0.01114370
1138	0.06250000	3.06250000	0.00860215
1139	0.06250000	3.00000000	-0.00782014
1140	0.93750000	4.00000000	0.18592375
1141	1.06250000	4.00000000	0.18729228
1142	0.93750000	3.93750000	0.18670577
1143	1.00000000	3.93750000	0.16950147
1144	3.06250000	0.00000000	0.06373412
1145	2.93750000	0.00000000	0.04379277
1146	3.06250000	0.06250000	0.06275660
1147	3.00000000	0.06250000	0.04516129
1148	4.00000000	0.93750000	0.18435973
1149	4.00000000	1.06250000	0.17458456
1150	3.93750000	0.93750000	0.17517107
1151	3.93750000	1.00000000	0.16578690
1152	3.06250000	3.06250000	0.11202346
1153	2.93750000	2.93750000	0.07546432
1154	3.00000000	3.06250000	0.10654936
1155	3.06250000	3.00000000	0.10498534
1156	2.93750000	3.00000000	0.08230694
1157	3.00000000	2.93750000	0.09090909
1158	3.06250000	4.00000000	0.15953079
1159	2.93750000	4.00000000	0.19393939
1160	3.00000000	3.93750000	0.19296188
1161	2.93750000	3.93750000	0.18690127
1162	4.00000000	3.06250000	0.17536657
1163	4.00000000	2.93750000	0.17653959
1164	3.93750000	3.00000000	0.17067449
1165	3.93750000	2.93750000	0.15835777
1166	0.93750000	2.00000000	-0.19433040
1167	1.06250000	2.00000000	-0.19863148
1168	0.93750000	1.93750000	-0.19628543
1169	1.00000000	1.93750000	-0.19824047
1170	1.00000000	2.06250000	-0.19100684
1171	1.06250000	2.06250000	-0.19569892
1172	0.93750000	2.93750000	-0.08543500
1173	1.06250000	3.06250000	-0.06627566
1174	0.93750000	3.00000000	-0.07292278
1175	1.00000000	3.06250000	-0.06119257
1176	1.00000000	2.93750000	-0.08504399
1177	1.06250000	3.00000000	-0.08250244

1178	2.00000000	0.93750000	-0.19608993
1179	2.00000000	1.06250000	-0.19921799
1180	1.93750000	0.93750000	-0.19648094
1181	1.93750000	1.00000000	-0.19882698
1182	2.06250000	1.00000000	-0.19276637
1183	2.06250000	1.06250000	-0.19296188
1184	2.93750000	0.93750000	-0.11104594
1185	3.06250000	1.06250000	-0.08054741
1186	3.00000000	0.93750000	-0.08758553
1187	3.06250000	1.00000000	-0.07956989
1188	2.93750000	1.00000000	-0.10342131
1189	3.00000000	1.06250000	-0.11593353
1190	2.00000000	2.93750000	-0.15659824
1191	2.00000000	3.06250000	-0.12199413
1192	2.06250000	3.00000000	-0.14017595
1193	2.06250000	3.06250000	-0.12218964
1194	1.93750000	2.93750000	-0.15659824
1195	1.93750000	3.00000000	-0.14897361
1196	2.93750000	2.00000000	-0.11202346
1197	3.06250000	2.00000000	-0.08582600
1198	3.00000000	2.06250000	-0.08387097
1199	3.06250000	2.06250000	-0.08230694
1200	2.93750000	1.93750000	-0.10557185
1201	3.00000000	1.93750000	-0.09931574
1202	0.00000000	0.43750000	-0.05552297
1203	0.00000000	0.56250000	-0.07565982
1204	0.06250000	0.50000000	-0.07096774
1205	0.06250000	0.56250000	-0.08269795
1206	0.43750000	0.00000000	-0.05219941
1207	0.56250000	0.00000000	-0.06412512
1208	0.50000000	0.06250000	-0.06764418
1209	0.56250000	0.06250000	-0.07898338
1210	0.43750000	0.43750000	-0.07018573
1211	0.56250000	0.56250000	-0.09951124
1212	0.43750000	0.50000000	-0.08367546
1213	0.50000000	0.43750000	-0.08308895
1214	0.50000000	0.56250000	-0.09736070
1215	0.56250000	0.50000000	-0.09696970
1216	0.00000000	3.56250000	0.10361681
1217	0.00000000	3.43750000	0.07996090
1218	0.06250000	3.56250000	0.10342131
1219	0.06250000	3.50000000	0.09169110
1220	0.43750000	4.00000000	0.18514174
1221	0.56250000	4.00000000	0.18494624
1222	0.43750000	3.93750000	0.17321603
1223	0.50000000	3.93750000	0.17302053
1224	3.56250000	0.00000000	0.13646139
1225	3.43750000	0.00000000	0.11827957
1226	3.56250000	0.06250000	0.13528837
1227	3.50000000	0.06250000	0.12629521
1228	4.00000000	0.43750000	0.19257087
1229	4.00000000	0.56250000	0.19042033
1230	3.93750000	0.43750000	0.18338221
1231	3.93750000	0.50000000	0.18240469
1232	3.56250000	3.56250000	0.19178886
1233	3.43750000	3.43750000	0.18240469
1234	3.50000000	3.56250000	0.15503421
1235	3.56250000	3.50000000	0.19178886
1236	3.43750000	3.50000000	0.18846530

1237	3.50000000	3.43750000	0.18709677
1238	3.56250000	4.00000000	-0.08250244
1239	3.43750000	4.00000000	-0.07956989
1240	3.50000000	3.93750000	-0.08015640
1241	3.43750000	3.93750000	-0.08582600
1242	4.00000000	3.56250000	0.17536657
1243	4.00000000	3.43750000	0.17536657
1244	3.93750000	3.50000000	0.17771261
1245	3.93750000	3.43750000	0.17067449
1246	0.00000000	1.56250000	-0.14565005
1247	0.00000000	1.43750000	-0.14447703
1248	0.06250000	1.56250000	-0.14995112
1249	0.06250000	1.50000000	-0.15171065
1250	0.00000000	2.43750000	-0.04907136
1251	0.00000000	2.56250000	-0.05767351
1252	0.06250000	2.50000000	-0.07487781
1253	0.06250000	2.56250000	-0.06236559
1254	0.43750000	2.00000000	-0.16207234
1255	0.56250000	2.00000000	-0.17028348
1256	0.43750000	1.93750000	-0.16402737
1257	0.50000000	2.06250000	-0.16304985
1258	0.50000000	1.93750000	-0.16793744
1259	0.56250000	2.06250000	-0.16265885
1260	0.43750000	2.43750000	-0.13646139
1261	0.56250000	2.56250000	-0.13255132
1262	0.43750000	2.50000000	-0.13118280
1263	0.50000000	2.43750000	-0.14369501
1264	0.50000000	2.56250000	-0.12805474
1265	0.56250000	2.50000000	-0.13352884
1266	1.56250000	0.00000000	-0.08504399
1267	1.43750000	0.00000000	-0.01681329
1268	1.56250000	0.06250000	-0.11906158
1269	1.50000000	0.06250000	-0.11945259
1270	2.43750000	0.00000000	-0.03831867
1271	2.56250000	0.00000000	-0.02072336
1272	2.50000000	0.06250000	-0.04105572
1273	2.56250000	0.06250000	-0.03010753
1274	2.00000000	0.43750000	-0.16695992
1275	2.00000000	0.56250000	-0.17732160
1276	1.93750000	0.43750000	-0.16637341
1277	2.06250000	0.50000000	-0.16598240
1278	1.93750000	0.50000000	-0.17458456
1279	2.06250000	0.56250000	-0.17086999
1280	2.43750000	0.43750000	-0.10987292
1281	2.56250000	0.56250000	-0.11984360
1282	2.50000000	0.43750000	-0.09912023
1283	2.43750000	0.50000000	-0.11691105
1284	2.56250000	0.50000000	-0.09794721
1285	2.50000000	0.56250000	-0.11378299
1286	1.56250000	1.56250000	-0.19784946
1287	1.43750000	1.43750000	-0.19745846
1288	1.50000000	1.56250000	-0.19784946
1289	1.56250000	1.50000000	-0.19784946
1290	1.43750000	1.50000000	-0.19745846
1291	1.50000000	1.43750000	-0.19745846
1292	2.43750000	2.43750000	-0.11085044
1293	2.56250000	2.56250000	-0.07350929
1294	2.43750000	2.50000000	-0.10811339
1295	2.50000000	2.43750000	-0.11085044

1296	2.50000000	2.56250000	-0.09149560
1297	2.56250000	2.50000000	-0.08289345
1298	1.56250000	2.00000000	-0.19921799
1299	1.43750000	2.00000000	-0.19921799
1300	1.50000000	1.93750000	-0.19902248
1301	1.56250000	2.06250000	-0.19628543
1302	1.43750000	1.93750000	-0.19882698
1303	1.50000000	2.06250000	-0.19628543
1304	2.00000000	1.56250000	-0.19921799
1305	2.00000000	1.43750000	-0.19921799
1306	1.93750000	1.50000000	-0.19902248
1307	2.06250000	1.56250000	-0.19921799
1308	1.93750000	1.43750000	-0.19882698
1309	2.06250000	1.50000000	-0.19296188
1310	2.00000000	2.43750000	-0.17986315
1311	2.00000000	2.56250000	-0.17399804
1312	2.06250000	2.50000000	-0.16695992
1313	1.93750000	2.43750000	-0.17966764
1314	2.06250000	2.56250000	-0.16031281
1315	1.93750000	2.50000000	-0.17693060
1316	2.43750000	2.00000000	-0.19960899
1317	2.56250000	2.00000000	-0.18709677
1318	2.50000000	2.06250000	-0.18690127
1319	2.43750000	1.93750000	-0.19941349
1320	2.56250000	2.06250000	-0.17145650
1321	2.50000000	1.93750000	-0.19315738
1322	1.56250000	4.00000000	0.18631476
1323	1.43750000	4.00000000	0.19511241
1324	1.50000000	3.93750000	0.17360704
1325	1.43750000	3.93750000	0.17869013
1326	2.43750000	4.00000000	0.15151515
1327	2.56250000	4.00000000	0.16207234
1328	2.43750000	3.93750000	0.13704790
1329	2.50000000	3.93750000	0.14956012
1330	1.56250000	3.56250000	0.02600196
1331	1.43750000	3.43750000	0.00175953
1332	1.50000000	3.56250000	0.03616813
1333	1.56250000	3.50000000	0.01153470
1334	1.43750000	3.50000000	0.01837732
1335	1.50000000	3.43750000	-0.00293255
1336	2.00000000	3.56250000	0.01427175
1337	2.00000000	3.43750000	-0.00684262
1338	2.06250000	3.56250000	0.01935484
1339	1.93750000	3.50000000	0.00156403
1340	2.06250000	3.50000000	0.01231672
1341	1.93750000	3.43750000	-0.01466276
1342	4.00000000	1.56250000	0.11788856
1343	4.00000000	1.43750000	0.12297165
1344	3.93750000	1.50000000	0.10439883
1345	3.93750000	1.43750000	0.10557185
1346	4.00000000	2.43750000	0.17908113
1347	4.00000000	2.56250000	0.18338221
1348	3.93750000	2.43750000	0.16246334
1349	3.93750000	2.50000000	0.16617791
1350	3.56250000	1.56250000	0.04828935
1351	3.43750000	1.43750000	0.01857283
1352	3.56250000	1.50000000	0.04496579
1353	3.50000000	1.56250000	0.03167155
1354	3.50000000	1.43750000	0.03616813

1355	3.43750000	1.50000000	0.01837732
1356	3.56250000	2.00000000	0.02521994
1357	3.43750000	2.00000000	-0.00469208
1358	3.56250000	2.06250000	0.03088954
1359	3.50000000	1.93750000	0.01212121
1360	3.50000000	2.06250000	0.01251222
1361	3.43750000	1.93750000	-0.00449658
1362	0.43750000	1.00000000	-0.17145650
1363	0.56250000	1.00000000	-0.18064516
1364	0.43750000	0.93750000	-0.16480938
1365	0.50000000	0.93750000	-0.16676442
1366	0.50000000	1.06250000	-0.17869013
1367	0.56250000	1.06250000	-0.18064516
1368	0.43750000	1.43750000	-0.17282502
1369	0.56250000	1.56250000	-0.18240469
1370	0.43750000	1.50000000	-0.17595308
1371	0.50000000	1.56250000	-0.17829912
1372	0.50000000	1.43750000	-0.17986315
1373	0.56250000	1.50000000	-0.18201369
1374	1.00000000	0.43750000	-0.16129032
1375	1.00000000	0.56250000	-0.17849462
1376	0.93750000	0.43750000	-0.16226784
1377	0.93750000	0.50000000	-0.16480938
1378	1.06250000	0.50000000	-0.17634409
1379	1.06250000	0.56250000	-0.17869013
1380	1.43750000	0.43750000	-0.16285435
1381	1.56250000	0.56250000	-0.18064516
1382	1.50000000	0.43750000	-0.16969697
1383	1.56250000	0.50000000	-0.17751711
1384	1.43750000	0.50000000	-0.17771261
1385	1.50000000	0.56250000	-0.18044966
1386	1.00000000	1.43750000	-0.19667644
1387	1.00000000	1.56250000	-0.19706745
1388	1.06250000	1.50000000	-0.19706745
1389	1.06250000	1.56250000	-0.19726295
1390	0.93750000	1.43750000	-0.19452590
1391	0.93750000	1.50000000	-0.19472141
1392	1.43750000	1.00000000	-0.19687195
1393	1.56250000	1.00000000	-0.19745846
1394	1.50000000	1.06250000	-0.19726295
1395	1.56250000	1.06250000	-0.19745846
1396	1.43750000	0.93750000	-0.19452590
1397	1.50000000	0.93750000	-0.19472141
1398	0.43750000	3.43750000	0.07956989
1399	0.56250000	3.56250000	0.11554252
1400	0.43750000	3.50000000	0.09149560
1401	0.50000000	3.56250000	0.10303030
1402	0.50000000	3.43750000	0.07487781
1403	0.56250000	3.50000000	0.08660802
1404	0.43750000	3.00000000	-0.03577713
1405	0.56250000	3.00000000	-0.04516129
1406	0.43750000	2.93750000	-0.04359726
1407	0.50000000	2.93750000	-0.05298143
1408	0.50000000	3.06250000	-0.02385142
1409	0.56250000	3.06250000	-0.02854350
1410	1.00000000	3.56250000	0.07077224
1411	1.00000000	3.43750000	0.03773216
1412	1.06250000	3.56250000	0.07194526
1413	1.06250000	3.50000000	0.04907136

1414	0.93750000	3.50000000	0.05884653
1415	0.93750000	3.43750000	0.04222874
1416	3.43750000	0.43750000	0.11104594
1417	3.56250000	0.56250000	0.12707722
1418	3.50000000	0.43750000	0.12023460
1419	3.56250000	0.50000000	0.12825024
1420	3.43750000	0.50000000	0.10146628
1421	3.50000000	0.56250000	0.10967742
1422	3.00000000	0.43750000	-0.01173021
1423	3.00000000	0.56250000	-0.03069404
1424	2.93750000	0.43750000	-0.02248289
1425	2.93750000	0.50000000	-0.03225806
1426	3.06250000	0.50000000	-0.00371457
1427	3.06250000	0.56250000	-0.01309873
1428	3.56250000	1.00000000	0.06060606
1429	3.43750000	1.00000000	0.02580645
1430	3.56250000	1.06250000	0.05219941
1431	3.50000000	1.06250000	0.04222874
1432	3.50000000	0.93750000	0.05278592
1433	3.43750000	0.93750000	0.03519062
1434	3.00000000	3.43750000	0.14349951
1435	3.00000000	3.56250000	0.15581623
1436	3.06250000	3.50000000	0.15522972
1437	3.06250000	3.56250000	0.11593353
1438	2.93750000	3.43750000	0.12551320
1439	2.93750000	3.50000000	0.14330401
1440	3.43750000	3.00000000	0.13313783
1441	3.56250000	3.00000000	0.14252199
1442	3.50000000	3.06250000	0.14486804
1443	3.56250000	3.06250000	0.14252199
1444	3.43750000	2.93750000	0.12375367
1445	3.50000000	2.93750000	0.12551320
1446	2.56250000	3.00000000	-0.02600196
1447	2.43750000	3.00000000	-0.05767351
1448	2.50000000	2.93750000	-0.05083089
1449	2.43750000	2.93750000	-0.06060606
1450	2.56250000	3.06250000	-0.01974585
1451	2.50000000	3.06250000	-0.02600196
1452	3.00000000	2.56250000	0.03460411
1453	3.00000000	2.43750000	0.00977517
1454	2.93750000	2.50000000	0.00977517
1455	2.93750000	2.43750000	-0.00273705
1456	3.06250000	2.56250000	0.03929619
1457	3.06250000	2.50000000	0.02697947
1458	2.56250000	3.56250000	0.11202346
1459	2.43750000	3.43750000	0.06783969
1460	2.50000000	3.56250000	0.10674487
1461	2.43750000	3.50000000	0.08719453
1462	2.56250000	3.50000000	0.10576735
1463	2.50000000	3.43750000	0.08152493
1464	3.56250000	2.56250000	0.05532747
1465	3.43750000	2.43750000	0.02091887
1466	3.56250000	2.50000000	0.05650049
1467	3.50000000	2.43750000	0.03440860
1468	3.50000000	2.56250000	0.05063539
1469	3.43750000	2.50000000	0.03655914
1470	1.00000000	2.43750000	-0.14545455
1471	1.00000000	2.56250000	-0.13020528
1472	0.93750000	2.43750000	-0.14134897

1473	0.93750000	2.50000000	-0.13822092
1474	1.06250000	2.50000000	-0.14252199
1475	1.06250000	2.56250000	-0.13509286
1476	1.43750000	2.43750000	-0.17888563
1477	1.56250000	2.56250000	-0.17321603
1478	1.50000000	2.43750000	-0.17888563
1479	1.56250000	2.50000000	-0.17614858
1480	1.43750000	2.50000000	-0.17126100
1481	1.50000000	2.56250000	-0.16852395
1482	1.43750000	3.00000000	-0.11104594
1483	1.56250000	3.00000000	-0.12062561
1484	1.50000000	3.06250000	-0.09970674
1485	1.56250000	3.06250000	-0.10146628
1486	1.43750000	2.93750000	-0.11867058
1487	1.50000000	2.93750000	-0.12336266
1488	2.43750000	1.00000000	-0.15425220
1489	2.56250000	1.00000000	-0.14173998
1490	2.43750000	0.93750000	-0.15131965
1491	2.50000000	0.93750000	-0.14310850
1492	2.50000000	1.06250000	-0.14799609
1493	2.56250000	1.06250000	-0.14173998
1494	2.43750000	1.43750000	-0.15542522
1495	2.56250000	1.56250000	-0.14291300
1496	2.43750000	1.50000000	-0.15542522
1497	2.50000000	1.56250000	-0.15542522
1498	2.50000000	1.43750000	-0.14897361
1499	2.56250000	1.50000000	-0.14271750
1500	3.00000000	1.43750000	-0.11671554
1501	3.00000000	1.56250000	-0.09833822
1502	3.06250000	1.50000000	-0.08152493
1503	3.06250000	1.56250000	-0.08484848
1504	2.93750000	1.43750000	-0.10439883
1505	2.93750000	1.50000000	-0.10459433
1506	0.00000000	0.18750000	-0.00508309
1507	0.00000000	0.31250000	-0.02873900
1508	0.06250000	0.25000000	-0.01739980
1509	0.06250000	0.31250000	-0.03088954
1510	0.18750000	0.00000000	-0.00351906
1511	0.31250000	0.00000000	-0.02639296
1512	0.25000000	0.06250000	-0.01603128
1513	0.31250000	0.06250000	-0.02893451
1514	0.18750000	0.18750000	-0.01114370
1515	0.31250000	0.31250000	-0.03910068
1516	0.18750000	0.25000000	-0.02150538
1517	0.25000000	0.18750000	-0.02111437
1518	0.25000000	0.31250000	-0.03695015
1519	0.31250000	0.25000000	-0.03655914
1520	0.00000000	3.81250000	0.15131965
1521	0.00000000	3.68750000	0.12746823
1522	0.06250000	3.81250000	0.15112414
1523	0.06250000	3.75000000	0.13919844
1524	0.18750000	4.00000000	0.18611926
1525	0.31250000	4.00000000	0.18572825
1526	0.18750000	3.93750000	0.17438905
1527	0.25000000	3.93750000	0.17399804
1528	3.81250000	0.00000000	0.17262952
1529	3.68750000	0.00000000	0.15464321
1530	3.81250000	0.06250000	0.17165200
1531	3.75000000	0.06250000	0.16265885

1532	4.00000000	0.18750000	0.19667644
1533	4.00000000	0.31250000	0.19472141
1534	3.93750000	0.18750000	0.18768328
1535	3.93750000	0.25000000	0.18670577
1536	3.81250000	3.81250000	0.18240469
1537	3.68750000	3.68750000	0.18709677
1538	3.75000000	3.81250000	0.14565005
1539	3.81250000	3.75000000	0.18240469
1540	3.68750000	3.75000000	0.14799609
1541	3.75000000	3.68750000	0.18475073
1542	3.81250000	4.00000000	0.06471163
1543	3.68750000	4.00000000	-0.00879765
1544	3.75000000	3.93750000	0.06725318
1545	3.68750000	3.93750000	0.03030303
1546	4.00000000	3.81250000	0.17536657
1547	4.00000000	3.68750000	0.17536657
1548	3.93750000	3.75000000	0.17771261
1549	3.93750000	3.68750000	0.17771261
1550	0.00000000	1.81250000	-0.13822092
1551	0.00000000	1.68750000	-0.14173998
1552	0.06250000	1.81250000	-0.14213099
1553	0.06250000	1.75000000	-0.14408602
1554	0.00000000	2.18750000	-0.03304008
1555	0.00000000	2.31250000	-0.01603128
1556	0.06250000	2.25000000	-0.03069404
1557	0.06250000	2.31250000	-0.02521994
1558	0.18750000	2.00000000	-0.14565005
1559	0.31250000	2.00000000	-0.15366569
1560	0.18750000	1.93750000	-0.14701857
1561	0.25000000	2.06250000	-0.14330401
1562	0.25000000	1.93750000	-0.15131965
1563	0.31250000	2.06250000	-0.15053763
1564	0.18750000	2.18750000	-0.12688172
1565	0.31250000	2.31250000	-0.12844575
1566	0.18750000	2.25000000	-0.09325513
1567	0.25000000	2.18750000	-0.13079179
1568	0.25000000	2.31250000	-0.11906158
1569	0.31250000	2.25000000	-0.13176931
1570	1.81250000	0.00000000	0.03499511
1571	1.68750000	0.00000000	0.01348974
1572	1.81250000	0.06250000	0.02795699
1573	1.75000000	0.06250000	0.02854350
1574	2.18750000	0.00000000	-0.07350929
1575	2.31250000	0.00000000	-0.05200391
1576	2.25000000	0.06250000	-0.07253177
1577	2.31250000	0.06250000	-0.06158358
1578	2.00000000	0.18750000	-0.13861193
1579	2.00000000	0.31250000	-0.15268817
1580	1.93750000	0.18750000	-0.08778104
1581	2.06250000	0.25000000	-0.13743891
1582	1.93750000	0.25000000	-0.14486804
1583	2.06250000	0.31250000	-0.14447703
1584	2.18750000	0.18750000	-0.11417400
1585	2.31250000	0.31250000	-0.11202346
1586	2.25000000	0.18750000	-0.09970674
1587	2.18750000	0.25000000	-0.12121212
1588	2.31250000	0.25000000	-0.10244379
1589	2.25000000	0.31250000	-0.12023460
1590	1.81250000	1.81250000	-0.19882698

1591	1.68750000	1.68750000	-0.19824047
1592	1.75000000	1.81250000	-0.19863148
1593	1.81250000	1.75000000	-0.19863148
1594	1.68750000	1.75000000	-0.19843597
1595	1.75000000	1.68750000	-0.19843597
1596	2.18750000	2.18750000	-0.16148583
1597	2.31250000	2.31250000	-0.13607038
1598	2.18750000	2.25000000	-0.15874878
1599	2.25000000	2.18750000	-0.16148583
1600	2.25000000	2.31250000	-0.14604106
1601	2.31250000	2.25000000	-0.14877810
1602	1.81250000	2.00000000	-0.19921799
1603	1.68750000	2.00000000	-0.19921799
1604	1.75000000	1.93750000	-0.19902248
1605	1.81250000	2.06250000	-0.19648094
1606	1.68750000	1.93750000	-0.19902248
1607	1.75000000	2.06250000	-0.19628543
1608	2.00000000	1.81250000	-0.19921799
1609	2.00000000	1.68750000	-0.19921799
1610	1.93750000	1.75000000	-0.19902248
1611	2.06250000	1.81250000	-0.19921799
1612	1.93750000	1.68750000	-0.19902248
1613	2.06250000	1.75000000	-0.19921799
1614	2.00000000	2.18750000	-0.19120235
1615	2.00000000	2.31250000	-0.18553275
1616	2.06250000	2.25000000	-0.17849462
1617	1.93750000	2.18750000	-0.19100684
1618	2.06250000	2.31250000	-0.17556207
1619	1.93750000	2.25000000	-0.18826979
1620	2.18750000	2.00000000	-0.19960899
1621	2.31250000	2.00000000	-0.19960899
1622	2.25000000	2.06250000	-0.18690127
1623	2.18750000	1.93750000	-0.19941349
1624	2.31250000	2.06250000	-0.18690127
1625	2.25000000	1.93750000	-0.19941349
1626	1.81250000	4.00000000	0.14604106
1627	1.68750000	4.00000000	0.16617791
1628	1.75000000	3.93750000	0.13333333
1629	1.68750000	3.93750000	0.14330401
1630	2.18750000	4.00000000	0.13118280
1631	2.31250000	4.00000000	0.14134897
1632	2.18750000	3.93750000	0.11671554
1633	2.25000000	3.93750000	0.12179863
1634	1.81250000	3.81250000	0.07741935
1635	1.68750000	3.68750000	0.05180841
1636	1.75000000	3.81250000	0.08758553
1637	1.81250000	3.75000000	0.06295210
1638	1.68750000	3.75000000	0.07468231
1639	1.75000000	3.68750000	0.05024438
1640	2.00000000	3.81250000	0.07253177
1641	2.00000000	3.68750000	0.04340176
1642	2.06250000	3.81250000	0.07761486
1643	1.93750000	3.75000000	0.05962854
1644	2.06250000	3.75000000	0.06314761
1645	1.93750000	3.68750000	0.04516129
1646	4.00000000	1.81250000	0.13137830
1647	4.00000000	1.68750000	0.12473118
1648	3.93750000	1.75000000	0.11808407
1649	3.93750000	1.68750000	0.11476051

1650	4.00000000	2.18750000	0.15757576
1651	4.00000000	2.31250000	0.16832845
1652	3.93750000	2.18750000	0.14095797
1653	3.93750000	2.25000000	0.14643206
1654	3.81250000	1.81250000	0.10146628
1655	3.68750000	1.68750000	0.07487781
1656	3.81250000	1.75000000	0.09814272
1657	3.75000000	1.81250000	0.08484848
1658	3.75000000	1.68750000	0.08484848
1659	3.68750000	1.75000000	0.07174976
1660	3.81250000	2.00000000	0.09149560
1661	3.68750000	2.00000000	0.05826002
1662	3.81250000	2.06250000	0.09696970
1663	3.75000000	1.93750000	0.07820137
1664	3.75000000	2.06250000	0.08035191
1665	3.68750000	1.93750000	0.06158358
1666	0.00000000	0.81250000	-0.10224829
1667	0.00000000	0.68750000	-0.08895406
1668	0.06250000	0.81250000	-0.10928641
1669	0.06250000	0.75000000	-0.10263930
1670	0.00000000	1.18750000	-0.13196481
1671	0.00000000	1.31250000	-0.13822092
1672	0.06250000	1.25000000	-0.13919844
1673	0.06250000	1.31250000	-0.14213099
1674	0.18750000	1.00000000	-0.14330401
1675	0.31250000	1.00000000	-0.15738025
1676	0.18750000	0.93750000	-0.13665689
1677	0.25000000	1.06250000	-0.15053763
1678	0.25000000	0.93750000	-0.14369501
1679	0.31250000	1.06250000	-0.15757576
1680	0.18750000	1.18750000	-0.14389052
1681	0.31250000	1.31250000	-0.15835777
1682	0.18750000	1.25000000	-0.14721408
1683	0.25000000	1.18750000	-0.15092864
1684	0.25000000	1.31250000	-0.15425220
1685	0.31250000	1.25000000	-0.15816227
1686	0.81250000	0.00000000	-0.06021505
1687	0.68750000	0.00000000	-0.06217009
1688	0.81250000	0.06250000	-0.07507331
1689	0.75000000	0.06250000	-0.07605083
1690	1.18750000	0.00000000	-0.07741935
1691	1.31250000	0.00000000	-0.00332356
1692	1.25000000	0.06250000	-0.09247312
1693	1.31250000	0.06250000	-0.09931574
1694	1.00000000	0.18750000	-0.10185728
1695	1.00000000	0.31250000	-0.13157380
1696	0.93750000	0.18750000	-0.10283480
1697	1.06250000	0.25000000	-0.11691105
1698	0.93750000	0.25000000	-0.11769306
1699	1.06250000	0.31250000	-0.13176931
1700	1.18750000	0.18750000	-0.10244379
1701	1.31250000	0.31250000	-0.13255132
1702	1.25000000	0.18750000	-0.10909091
1703	1.18750000	0.25000000	-0.11730205
1704	1.31250000	0.25000000	-0.12414467
1705	1.25000000	0.31250000	-0.13235582
1706	0.81250000	0.81250000	-0.15425220
1707	0.68750000	0.68750000	-0.12688172
1708	0.75000000	0.81250000	-0.15210166

1709	0.81250000	0.75000000	-0.15171065
1710	0.68750000	0.75000000	-0.13841642
1711	0.75000000	0.68750000	-0.13802542
1712	1.18750000	1.18750000	-0.19628543
1713	1.31250000	1.31250000	-0.19687195
1714	1.18750000	1.25000000	-0.19628543
1715	1.25000000	1.18750000	-0.19628543
1716	1.25000000	1.31250000	-0.19667644
1717	1.31250000	1.25000000	-0.19667644
1718	0.81250000	1.00000000	-0.18885630
1719	0.68750000	1.00000000	-0.18475073
1720	0.75000000	0.93750000	-0.17517107
1721	0.81250000	1.06250000	-0.18905181
1722	0.68750000	0.93750000	-0.17302053
1723	0.75000000	1.06250000	-0.18690127
1724	1.00000000	0.81250000	-0.18787879
1725	1.00000000	0.68750000	-0.18318671
1726	0.93750000	0.75000000	-0.17419355
1727	1.06250000	0.81250000	-0.18826979
1728	0.93750000	0.68750000	-0.17184751
1729	1.06250000	0.75000000	-0.18572825
1730	1.00000000	1.18750000	-0.19589443
1731	1.00000000	1.31250000	-0.19628543
1732	1.06250000	1.25000000	-0.19608993
1733	0.93750000	1.18750000	-0.19374389
1734	1.06250000	1.31250000	-0.19628543
1735	0.93750000	1.25000000	-0.19393939
1736	1.18750000	1.00000000	-0.19589443
1737	1.31250000	1.00000000	-0.19628543
1738	1.25000000	1.06250000	-0.19608993
1739	1.18750000	0.93750000	-0.19354839
1740	1.31250000	1.06250000	-0.19628543
1741	1.25000000	0.93750000	-0.19374389
1742	0.00000000	3.18750000	0.03245357
1743	0.00000000	3.31250000	0.05630499
1744	0.06250000	3.25000000	0.04437928
1745	0.06250000	3.31250000	0.05630499
1746	0.00000000	2.81250000	-0.02678397
1747	0.00000000	2.68750000	-0.04222874
1748	0.06250000	2.81250000	-0.03088954
1749	0.06250000	2.75000000	-0.08445748
1750	0.18750000	3.18750000	0.03225806
1751	0.31250000	3.31250000	0.05591398
1752	0.18750000	3.25000000	0.04418377
1753	0.25000000	3.18750000	0.07683284
1754	0.25000000	3.31250000	0.05747801
1755	0.31250000	3.25000000	0.04652981
1756	0.18750000	3.00000000	-0.01700880
1757	0.31250000	3.00000000	-0.02639296
1758	0.18750000	2.93750000	-0.02443793
1759	0.25000000	3.06250000	0.04398827
1760	0.25000000	2.93750000	-0.01583578
1761	0.31250000	3.06250000	-0.00977517
1762	0.81250000	4.00000000	0.18572825
1763	0.68750000	4.00000000	0.18533724
1764	0.75000000	3.93750000	0.18631476
1765	0.68750000	3.93750000	0.17360704
1766	1.18750000	4.00000000	0.19002933
1767	1.31250000	4.00000000	0.19257087

1768	1.18750000	3.93750000	0.17360704
1769	1.25000000	3.93750000	0.17497556
1770	0.81250000	3.81250000	0.18807429
1771	0.68750000	3.68750000	0.16422287
1772	0.75000000	3.81250000	0.18787879
1773	0.81250000	3.75000000	0.17165200
1774	0.68750000	3.75000000	0.17595308
1775	0.75000000	3.68750000	0.15972630
1776	1.00000000	3.81250000	0.13665689
1777	1.00000000	3.68750000	0.10381232
1778	1.06250000	3.81250000	0.13802542
1779	0.93750000	3.75000000	0.13743891
1780	1.06250000	3.75000000	0.12160313
1781	0.93750000	3.68750000	0.10830890
1782	3.18750000	0.00000000	0.08191593
1783	3.31250000	0.00000000	0.10009775
1784	3.25000000	0.06250000	0.08993157
1785	3.31250000	0.06250000	0.09912023
1786	2.81250000	0.00000000	0.02228739
1787	2.68750000	0.00000000	0.00078201
1788	2.81250000	0.06250000	0.01290323
1789	2.75000000	0.06250000	0.00195503
1790	3.18750000	0.18750000	0.07898338
1791	3.31250000	0.31250000	0.09501466
1792	3.25000000	0.18750000	0.08797654
1793	3.18750000	0.25000000	0.06940371
1794	3.31250000	0.25000000	0.09618768
1795	3.25000000	0.31250000	0.07761486
1796	3.00000000	0.18750000	0.02619746
1797	3.00000000	0.31250000	0.00723363
1798	2.93750000	0.18750000	0.01544477
1799	3.06250000	0.25000000	0.03421310
1800	2.93750000	0.25000000	0.00586510
1801	3.06250000	0.31250000	0.02463343
1802	4.00000000	0.81250000	0.18651026
1803	4.00000000	0.68750000	0.18846530
1804	3.93750000	0.75000000	0.17849462
1805	3.93750000	0.68750000	0.17947214
1806	4.00000000	1.18750000	0.15738025
1807	4.00000000	1.31250000	0.14017595
1808	3.93750000	1.18750000	0.13998045
1809	3.93750000	1.25000000	0.13137830
1810	3.81250000	0.81250000	0.15913978
1811	3.68750000	0.68750000	0.14310850
1812	3.81250000	0.75000000	0.16031281
1813	3.75000000	0.81250000	0.14154448
1814	3.75000000	0.68750000	0.15229717
1815	3.68750000	0.75000000	0.13372434
1816	3.81250000	1.00000000	0.13079179
1817	3.68750000	1.00000000	0.09560117
1818	3.81250000	1.06250000	0.12218964
1819	3.75000000	0.93750000	0.12258065
1820	3.75000000	1.06250000	0.10459433
1821	3.68750000	0.93750000	0.10518084
1822	3.18750000	3.18750000	0.13548387
1823	3.31250000	3.31250000	0.15894428
1824	3.18750000	3.25000000	0.14154448
1825	3.25000000	3.18750000	0.14017595
1826	3.25000000	3.31250000	0.15327468

1827	3.31250000	3.25000000	0.15190616
1828	2.81250000	2.81250000	0.02580645
1829	2.68750000	2.68750000	-0.02385142
1830	2.75000000	2.81250000	0.00782014
1831	2.81250000	2.75000000	0.01642229
1832	2.68750000	2.75000000	-0.01700880
1833	2.75000000	2.68750000	-0.00840665
1834	3.00000000	3.18750000	0.11886608
1835	3.00000000	3.31250000	0.13118280
1836	3.06250000	3.25000000	0.13040078
1837	2.93750000	3.18750000	0.10068426
1838	3.06250000	3.31250000	0.13665689
1839	2.93750000	3.25000000	0.10694037
1840	3.18750000	3.00000000	0.11436950
1841	3.31250000	3.00000000	0.12375367
1842	3.25000000	3.06250000	0.12609971
1843	3.18750000	2.93750000	0.10498534
1844	3.31250000	3.06250000	0.13079179
1845	3.25000000	2.93750000	0.10967742
1846	2.81250000	3.00000000	0.04613881
1847	2.68750000	3.00000000	0.00997067
1848	2.75000000	2.93750000	0.02130987
1849	2.81250000	3.06250000	0.05219941
1850	2.68750000	2.93750000	0.00332356
1851	2.75000000	3.06250000	0.03421310
1852	3.00000000	2.81250000	0.07214076
1853	3.00000000	2.68750000	0.05337243
1854	2.93750000	2.75000000	0.04731183
1855	3.06250000	2.81250000	0.07683284
1856	2.93750000	2.68750000	0.03792766
1857	3.06250000	2.75000000	0.06744868
1858	3.18750000	4.00000000	0.07996090
1859	3.31250000	4.00000000	0.00019550
1860	3.18750000	3.93750000	0.07370479
1861	3.25000000	3.93750000	0.03382209
1862	2.81250000	4.00000000	0.18338221
1863	2.68750000	4.00000000	0.17282502
1864	2.75000000	3.93750000	0.17106549
1865	2.68750000	3.93750000	0.16559140
1866	3.00000000	3.81250000	0.18064516
1867	3.00000000	3.68750000	0.16832845
1868	3.06250000	3.81250000	0.14095797
1869	2.93750000	3.75000000	0.16832845
1870	3.06250000	3.75000000	0.13470186
1871	2.93750000	3.68750000	0.16207234
1872	2.81250000	3.81250000	0.16207234
1873	2.68750000	3.68750000	0.13704790
1874	2.75000000	3.81250000	0.15679374
1875	2.81250000	3.75000000	0.15581623
1876	2.68750000	3.75000000	0.14428152
1877	2.75000000	3.68750000	0.14330401
1878	4.00000000	3.18750000	0.17536657
1879	4.00000000	3.31250000	0.17536657
1880	3.93750000	3.18750000	0.17067449
1881	3.93750000	3.25000000	0.17067449
1882	4.00000000	2.81250000	0.17888563
1883	4.00000000	2.68750000	0.18123167
1884	3.93750000	2.75000000	0.16187683
1885	3.93750000	2.68750000	0.16285435

1886	3.81250000	3.00000000	0.16129032
1887	3.68750000	3.00000000	0.15190616
1888	3.81250000	3.06250000	0.16129032
1889	3.75000000	2.93750000	0.14428152
1890	3.75000000	3.06250000	0.15659824
1891	3.68750000	2.93750000	0.13958944
1892	3.81250000	2.81250000	0.12414467
1893	3.68750000	2.68750000	0.08973607
1894	3.81250000	2.75000000	0.12531769
1895	3.75000000	2.81250000	0.11945259
1896	3.75000000	2.68750000	0.10811339
1897	3.68750000	2.75000000	0.10224829
1898	0.81250000	2.00000000	-0.18631476
1899	0.68750000	2.00000000	-0.17829912
1900	0.75000000	1.93750000	-0.18416422
1901	0.81250000	2.06250000	-0.17888563
1902	0.68750000	1.93750000	-0.18005865
1903	0.75000000	2.06250000	-0.17478006
1904	1.18750000	2.00000000	-0.19882698
1905	1.31250000	2.00000000	-0.19902248
1906	1.18750000	1.93750000	-0.19843597
1907	1.25000000	2.06250000	-0.19608993
1908	1.25000000	1.93750000	-0.19863148
1909	1.31250000	2.06250000	-0.19608993
1910	0.81250000	1.81250000	-0.19178886
1911	0.68750000	1.68750000	-0.18709677
1912	0.75000000	1.81250000	-0.18768328
1913	0.81250000	1.75000000	-0.19139785
1914	0.68750000	1.75000000	-0.18533724
1915	0.75000000	1.68750000	-0.18924731
1916	1.00000000	1.81250000	-0.19784946
1917	1.00000000	1.68750000	-0.19745846
1918	1.06250000	1.81250000	-0.19784946
1919	0.93750000	1.75000000	-0.19550342
1920	1.06250000	1.75000000	-0.19784946
1921	0.93750000	1.68750000	-0.19530792
1922	1.00000000	2.18750000	-0.17595308
1923	1.00000000	2.31250000	-0.16070381
1924	0.93750000	2.18750000	-0.17184751
1925	1.06250000	2.25000000	-0.17302053
1926	0.93750000	2.25000000	-0.16422287
1927	1.06250000	2.31250000	-0.16539589
1928	1.18750000	2.18750000	-0.19022483
1929	1.31250000	2.31250000	-0.18455523
1930	1.25000000	2.18750000	-0.19042033
1931	1.18750000	2.25000000	-0.18260020
1932	1.31250000	2.25000000	-0.18748778
1933	1.25000000	2.31250000	-0.17986315
1934	0.81250000	2.81250000	-0.10107527
1935	0.68750000	2.68750000	-0.11671554
1936	0.75000000	2.81250000	-0.09657869
1937	0.81250000	2.75000000	-0.10850440
1938	0.68750000	2.75000000	-0.10420332
1939	0.75000000	2.68750000	-0.11652004
1940	1.18750000	3.18750000	-0.04359726
1941	1.31250000	3.31250000	-0.02091887
1942	1.18750000	3.25000000	-0.02717498
1943	1.25000000	3.18750000	-0.04828935
1944	1.25000000	3.31250000	-0.01564027

1945	1.31250000	3.25000000	-0.03695015
1946	0.81250000	3.00000000	-0.06373412
1947	0.68750000	3.00000000	-0.05454545
1948	0.75000000	2.93750000	-0.07174976
1949	0.81250000	3.06250000	-0.04731183
1950	0.68750000	2.93750000	-0.06705767
1951	0.75000000	3.06250000	-0.04261975
1952	1.00000000	3.18750000	-0.02834800
1953	1.00000000	3.31250000	0.00469208
1954	1.06250000	3.25000000	-0.01700880
1955	0.93750000	3.18750000	-0.02365591
1956	1.06250000	3.31250000	-0.00039101
1957	0.93750000	3.25000000	-0.00723363
1958	1.00000000	2.81250000	-0.10009775
1959	1.00000000	2.68750000	-0.11515152
1960	0.93750000	2.75000000	-0.10791789
1961	1.06250000	2.81250000	-0.10498534
1962	0.93750000	2.68750000	-0.11554252
1963	1.06250000	2.75000000	-0.11241447
1964	1.18750000	3.00000000	-0.09208211
1965	1.31250000	3.00000000	-0.10146628
1966	1.25000000	3.06250000	-0.08054741
1967	1.18750000	2.93750000	-0.09951124
1968	1.31250000	3.06250000	-0.08543500
1969	1.25000000	2.93750000	-0.10439883
1970	2.00000000	0.81250000	-0.18983382
1971	2.00000000	0.68750000	-0.18357771
1972	1.93750000	0.75000000	-0.18709677
1973	2.06250000	0.81250000	-0.18338221
1974	1.93750000	0.68750000	-0.18396872
1975	2.06250000	0.75000000	-0.18025415
1976	2.00000000	1.18750000	-0.19921799
1977	2.00000000	1.31250000	-0.19921799
1978	1.93750000	1.18750000	-0.19882698
1979	2.06250000	1.25000000	-0.19296188
1980	1.93750000	1.25000000	-0.19882698
1981	2.06250000	1.31250000	-0.19296188
1982	1.81250000	0.81250000	-0.19120235
1983	1.68750000	0.68750000	-0.18592375
1984	1.81250000	0.75000000	-0.18807429
1985	1.75000000	0.81250000	-0.19100684
1986	1.75000000	0.68750000	-0.18553275
1987	1.68750000	0.75000000	-0.18826979
1988	1.81250000	1.00000000	-0.19824047
1989	1.68750000	1.00000000	-0.19784946
1990	1.81250000	1.06250000	-0.19824047
1991	1.75000000	0.93750000	-0.19569892
1992	1.75000000	1.06250000	-0.19804497
1993	1.68750000	0.93750000	-0.19550342
1994	2.18750000	1.00000000	-0.17986315
1995	2.31250000	1.00000000	-0.16695992
1996	2.18750000	0.93750000	-0.17673509
1997	2.25000000	1.06250000	-0.17360704
1998	2.25000000	0.93750000	-0.18279570
1999	2.31250000	1.06250000	-0.16715543
2000	2.18750000	1.18750000	-0.18044966
2001	2.31250000	1.31250000	-0.16793744
2002	2.18750000	1.25000000	-0.18044966
2003	2.25000000	1.18750000	-0.17399804

2004	2.25000000	1.31250000	-0.17419355
2005	2.31250000	1.25000000	-0.16774194
2006	2.81250000	0.81250000	-0.13900293
2007	2.68750000	0.68750000	-0.14193548
2008	2.81250000	0.75000000	-0.12961877
2009	2.75000000	0.81250000	-0.14545455
2010	2.75000000	0.68750000	-0.13098729
2011	2.68750000	0.75000000	-0.14682307
2012	3.18750000	1.18750000	-0.04750733
2013	3.31250000	1.31250000	-0.01446725
2014	3.25000000	1.18750000	-0.02991202
2015	3.18750000	1.25000000	-0.06627566
2016	3.31250000	1.25000000	-0.01348974
2017	3.25000000	1.31250000	-0.03108504
2018	3.00000000	0.81250000	-0.06862170
2019	3.00000000	0.68750000	-0.04965787
2020	2.93750000	0.75000000	-0.08269795
2021	3.06250000	0.81250000	-0.05102639
2022	2.93750000	0.68750000	-0.06080156
2023	3.06250000	0.75000000	-0.04164223
2024	3.18750000	1.00000000	-0.04437928
2025	3.31250000	1.00000000	-0.00918866
2026	3.25000000	1.06250000	-0.02776149
2027	3.18750000	0.93750000	-0.03479961
2028	3.31250000	1.06250000	-0.01016618
2029	3.25000000	0.93750000	-0.01720430
2030	2.81250000	1.00000000	-0.11612903
2031	2.68750000	1.00000000	-0.12903226
2032	2.75000000	0.93750000	-0.13020528
2033	2.81250000	1.06250000	-0.13489736
2034	2.68750000	0.93750000	-0.12394917
2035	2.75000000	1.06250000	-0.12258065
2036	3.00000000	1.18750000	-0.15347019
2037	3.00000000	1.31250000	-0.15366569
2038	3.06250000	1.25000000	-0.13685239
2039	2.93750000	1.18750000	-0.15972630
2040	3.06250000	1.31250000	-0.13704790
2041	2.93750000	1.25000000	-0.15992180
2042	2.00000000	2.81250000	-0.16226784
2043	2.00000000	2.68750000	-0.16813294
2044	2.06250000	2.81250000	-0.14858260
2045	1.93750000	2.75000000	-0.16520039
2046	2.06250000	2.75000000	-0.15151515
2047	1.93750000	2.68750000	-0.16813294
2048	2.00000000	3.18750000	-0.05845552
2049	2.00000000	3.31250000	-0.02013685
2050	2.06250000	3.25000000	-0.02678397
2051	1.93750000	3.18750000	-0.05376344
2052	2.06250000	3.31250000	-0.00762463
2053	1.93750000	3.25000000	-0.03460411
2054	2.18750000	3.00000000	-0.11260997
2055	2.31250000	3.00000000	-0.08504399
2056	2.18750000	2.93750000	-0.11534702
2057	2.25000000	3.06250000	-0.08074291
2058	2.25000000	2.93750000	-0.10166178
2059	2.31250000	3.06250000	-0.06705767
2060	2.18750000	3.18750000	-0.05884653
2061	2.31250000	3.31250000	0.00449658
2062	2.18750000	3.25000000	-0.02697947

2063	2.25000000	3.18750000	-0.04496579
2064	2.25000000	3.31250000	-0.00782014
2065	2.31250000	3.25000000	-0.01348974
2066	1.81250000	2.81250000	-0.16207234
2067	1.68750000	2.68750000	-0.16754643
2068	1.81250000	2.75000000	-0.16500489
2069	1.75000000	2.81250000	-0.15738025
2070	1.75000000	2.68750000	-0.16774194
2071	1.68750000	2.75000000	-0.16011730
2072	1.81250000	3.00000000	-0.13939394
2073	1.68750000	3.00000000	-0.13000978
2074	1.81250000	3.06250000	-0.10772239
2075	1.75000000	2.93750000	-0.14232649
2076	1.75000000	3.06250000	-0.11554252
2077	1.68750000	2.93750000	-0.13743891
2078	2.81250000	2.00000000	-0.13704790
2079	2.68750000	2.00000000	-0.16207234
2080	2.81250000	2.06250000	-0.12140762
2081	2.75000000	1.93750000	-0.14310850
2082	2.75000000	2.06250000	-0.13391984
2083	2.68750000	1.93750000	-0.15562072
2084	3.18750000	2.00000000	-0.05865103
2085	3.31250000	2.00000000	-0.03167155
2086	3.25000000	2.06250000	-0.04144673
2087	3.18750000	1.93750000	-0.05845552
2088	3.31250000	2.06250000	-0.02795699
2089	3.25000000	1.93750000	-0.04496579
2090	3.00000000	2.18750000	-0.05259042
2091	3.00000000	2.31250000	-0.02130987
2092	2.93750000	2.18750000	-0.06510264
2093	3.06250000	2.25000000	-0.03538612
2094	2.93750000	2.25000000	-0.04946237
2095	3.06250000	2.31250000	-0.01974585
2096	3.18750000	2.18750000	-0.04789834
2097	3.31250000	2.31250000	-0.01348974
2098	3.25000000	2.18750000	-0.03421310
2099	3.18750000	2.25000000	-0.03225806
2100	3.31250000	2.25000000	-0.01720430
2101	3.25000000	2.31250000	-0.01505376
2102	2.81250000	1.81250000	-0.11788856
2103	2.68750000	1.68750000	-0.13040078
2104	2.75000000	1.81250000	-0.13040078
2105	2.81250000	1.75000000	-0.11769306
2106	2.68750000	1.75000000	-0.13665689
2107	2.75000000	1.68750000	-0.12394917
2108	3.00000000	1.81250000	-0.09892473
2109	3.00000000	1.68750000	-0.09853372
2110	3.06250000	1.81250000	-0.08523949
2111	2.93750000	1.75000000	-0.10498534
2112	3.06250000	1.75000000	-0.08523949
2113	2.93750000	1.68750000	-0.10498534
2114	0.18750000	0.50000000	-0.07507331
2115	0.31250000	0.50000000	-0.07937439
2116	0.18750000	0.43750000	-0.06177908
2117	0.25000000	0.43750000	-0.06373412
2118	0.25000000	0.56250000	-0.08875855
2119	0.31250000	0.56250000	-0.09090909
2120	0.18750000	0.68750000	-0.11006843
2121	0.31250000	0.81250000	-0.13743891

2122	0.18750000	0.75000000	-0.11671554
2123	0.25000000	0.81250000	-0.13040078
2124	0.25000000	0.68750000	-0.11202346
2125	0.31250000	0.75000000	-0.12570870
2126	0.50000000	0.18750000	-0.07272727
2127	0.50000000	0.31250000	-0.07781036
2128	0.43750000	0.18750000	-0.05982405
2129	0.43750000	0.25000000	-0.06236559
2130	0.56250000	0.25000000	-0.08660802
2131	0.56250000	0.31250000	-0.08914956
2132	0.68750000	0.18750000	-0.10674487
2133	0.81250000	0.31250000	-0.13450635
2134	0.75000000	0.18750000	-0.10576735
2135	0.81250000	0.25000000	-0.11964809
2136	0.68750000	0.25000000	-0.10928641
2137	0.75000000	0.31250000	-0.12316716
2138	0.50000000	0.68750000	-0.12043011
2139	0.50000000	0.81250000	-0.14349951
2140	0.56250000	0.75000000	-0.13411535
2141	0.56250000	0.81250000	-0.14565005
2142	0.43750000	0.68750000	-0.11827957
2143	0.43750000	0.75000000	-0.12981427
2144	0.68750000	0.50000000	-0.11945259
2145	0.81250000	0.50000000	-0.14213099
2146	0.75000000	0.56250000	-0.13313783
2147	0.81250000	0.56250000	-0.14447703
2148	0.68750000	0.43750000	-0.11691105
2149	0.75000000	0.43750000	-0.12825024
2150	0.18750000	3.68750000	0.12668622
2151	0.31250000	3.81250000	0.14995112
2152	0.18750000	3.75000000	0.13861193
2153	0.25000000	3.81250000	0.15014663
2154	0.25000000	3.68750000	0.12668622
2155	0.31250000	3.75000000	0.13822092
2156	0.18750000	3.50000000	0.09169110
2157	0.31250000	3.50000000	0.09169110
2158	0.18750000	3.43750000	0.07996090
2159	0.25000000	3.43750000	0.08132942
2160	0.25000000	3.56250000	0.10342131
2161	0.31250000	3.56250000	0.10322581
2162	0.50000000	3.81250000	0.14956012
2163	0.50000000	3.68750000	0.12629521
2164	0.56250000	3.81250000	0.14975562
2165	0.56250000	3.75000000	0.15053763
2166	0.43750000	3.75000000	0.13802542
2167	0.43750000	3.68750000	0.12629521
2168	3.68750000	0.18750000	0.15131965
2169	3.81250000	0.31250000	0.16735093
2170	3.75000000	0.18750000	0.16050831
2171	3.81250000	0.25000000	0.16852395
2172	3.68750000	0.25000000	0.15034213
2173	3.75000000	0.31250000	0.15835777
2174	3.50000000	0.18750000	0.12434018
2175	3.50000000	0.31250000	0.12238514
2176	3.43750000	0.18750000	0.11534702
2177	3.43750000	0.25000000	0.11436950
2178	3.56250000	0.25000000	0.13235582
2179	3.56250000	0.31250000	0.13137830
2180	3.81250000	0.50000000	0.16441838

2181	3.68750000	0.50000000	0.14643206
2182	3.81250000	0.56250000	0.16344086
2183	3.75000000	0.56250000	0.15444770
2184	3.75000000	0.43750000	0.15640274
2185	3.68750000	0.43750000	0.14740958
2186	3.50000000	3.68750000	0.07663734
2187	3.50000000	3.81250000	-0.00175953
2188	3.56250000	3.75000000	0.07429130
2189	3.56250000	3.81250000	0.03499511
2190	3.43750000	3.68750000	0.07096774
2191	3.43750000	3.75000000	0.03167155
2192	3.68750000	3.50000000	0.18709677
2193	3.81250000	3.50000000	0.18240469
2194	3.75000000	3.56250000	0.18475073
2195	3.81250000	3.56250000	0.18240469
2196	3.68750000	3.43750000	0.18005865
2197	3.75000000	3.43750000	0.17771261
2198	3.31250000	3.50000000	0.17732160
2199	3.18750000	3.50000000	0.16637341
2200	3.25000000	3.43750000	0.16559140
2201	3.18750000	3.43750000	0.16011730
2202	3.31250000	3.56250000	0.13822092
2203	3.25000000	3.56250000	0.13274682
2204	3.50000000	3.31250000	0.17302053
2205	3.50000000	3.18750000	0.15894428
2206	3.43750000	3.25000000	0.16129032
2207	3.43750000	3.18750000	0.15425220
2208	3.56250000	3.31250000	0.17067449
2209	3.56250000	3.25000000	0.16363636
2210	3.31250000	3.81250000	-0.01857283
2211	3.18750000	3.68750000	0.04868035
2212	3.25000000	3.81250000	0.02130987
2213	3.18750000	3.75000000	0.05493646
2214	3.31250000	3.75000000	0.02052786
2215	3.25000000	3.68750000	0.05434995
2216	3.81250000	3.31250000	0.16129032
2217	3.68750000	3.18750000	0.15190616
2218	3.81250000	3.25000000	0.16129032
2219	3.75000000	3.18750000	0.15659824
2220	3.75000000	3.31250000	0.16363636
2221	3.68750000	3.25000000	0.15894428
2222	0.18750000	1.68750000	-0.15464321
2223	0.31250000	1.81250000	-0.15933529
2224	0.18750000	1.75000000	-0.15268817
2225	0.25000000	1.81250000	-0.15503421
2226	0.25000000	1.68750000	-0.15855327
2227	0.31250000	1.75000000	-0.16089932
2228	0.18750000	1.50000000	-0.15972630
2229	0.31250000	1.50000000	-0.16774194
2230	0.18750000	1.43750000	-0.15640274
2231	0.25000000	1.43750000	-0.16050831
2232	0.25000000	1.56250000	-0.16187683
2233	0.31250000	1.56250000	-0.16598240
2234	0.18750000	2.50000000	-0.09364614
2235	0.31250000	2.50000000	-0.11241447
2236	0.18750000	2.43750000	-0.07702835
2237	0.25000000	2.43750000	-0.10830890
2238	0.25000000	2.56250000	-0.09051808
2239	0.31250000	2.56250000	-0.09990225

2240	0.18750000	2.68750000	-0.05610948
2241	0.31250000	2.81250000	-0.04985337
2242	0.18750000	2.75000000	-0.09364614
2243	0.25000000	2.81250000	-0.03147605
2244	0.25000000	2.68750000	-0.06549365
2245	0.31250000	2.75000000	-0.06236559
2246	0.50000000	2.18750000	-0.15659824
2247	0.50000000	2.31250000	-0.15014663
2248	0.43750000	2.18750000	-0.15249267
2249	0.43750000	2.25000000	-0.14623656
2250	0.56250000	2.25000000	-0.15307918
2251	0.56250000	2.31250000	-0.14995112
2252	0.50000000	1.81250000	-0.17126100
2253	0.50000000	1.68750000	-0.17478006
2254	0.56250000	1.81250000	-0.17536657
2255	0.56250000	1.75000000	-0.17712610
2256	0.43750000	1.75000000	-0.16891496
2257	0.43750000	1.68750000	-0.17067449
2258	0.68750000	2.18750000	-0.15542522
2259	0.81250000	2.31250000	-0.14838710
2260	0.75000000	2.18750000	-0.15953079
2261	0.81250000	2.25000000	-0.15601173
2262	0.68750000	2.25000000	-0.15229717
2263	0.75000000	2.31250000	-0.14877810
2264	0.50000000	2.68750000	-0.10303030
2265	0.50000000	2.81250000	-0.07800587
2266	0.56250000	2.75000000	-0.09501466
2267	0.56250000	2.81250000	-0.08269795
2268	0.43750000	2.68750000	-0.09364614
2269	0.43750000	2.75000000	-0.08113392
2270	0.68750000	2.50000000	-0.13294233
2271	0.81250000	2.50000000	-0.13900293
2272	0.75000000	2.56250000	-0.13176931
2273	0.81250000	2.56250000	-0.13137830
2274	0.68750000	2.43750000	-0.14291300
2275	0.75000000	2.43750000	-0.14252199
2276	1.68750000	0.18750000	-0.13489736
2277	1.81250000	0.31250000	-0.15073314
2278	1.75000000	0.18750000	-0.08582600
2279	1.81250000	0.25000000	-0.14349951
2280	1.68750000	0.25000000	-0.14330401
2281	1.75000000	0.31250000	-0.15112414
2282	1.50000000	0.18750000	-0.13607038
2283	1.50000000	0.31250000	-0.15288368
2284	1.43750000	0.18750000	-0.12942326
2285	1.43750000	0.25000000	-0.13763441
2286	1.56250000	0.25000000	-0.14408602
2287	1.56250000	0.31250000	-0.15249267
2288	2.50000000	0.18750000	-0.06041056
2289	2.50000000	0.31250000	-0.07976540
2290	2.43750000	0.18750000	-0.06725318
2291	2.43750000	0.25000000	-0.08093842
2292	2.56250000	0.25000000	-0.05923754
2293	2.56250000	0.31250000	-0.06881720
2294	2.68750000	0.18750000	-0.02756598
2295	2.81250000	0.31250000	-0.02502444
2296	2.75000000	0.18750000	-0.01700880
2297	2.81250000	0.25000000	-0.01564027
2298	2.68750000	0.25000000	-0.03734115

2299	2.75000000	0.31250000	-0.03616813
2300	2.18750000	0.50000000	-0.14956012
2301	2.31250000	0.50000000	-0.13313783
2302	2.18750000	0.43750000	-0.14232649
2303	2.25000000	0.43750000	-0.13431085
2304	2.25000000	0.56250000	-0.14623656
2305	2.31250000	0.56250000	-0.13802542
2306	1.81250000	0.50000000	-0.17556207
2307	1.68750000	0.50000000	-0.17653959
2308	1.81250000	0.56250000	-0.17869013
2309	1.75000000	0.56250000	-0.17927664
2310	1.75000000	0.43750000	-0.16774194
2311	1.68750000	0.43750000	-0.16832845
2312	2.18750000	0.68750000	-0.16422287
2313	2.31250000	0.81250000	-0.15777126
2314	2.18750000	0.75000000	-0.16735093
2315	2.25000000	0.81250000	-0.17653959
2316	2.25000000	0.68750000	-0.15601173
2317	2.31250000	0.75000000	-0.15268817
2318	2.68750000	0.50000000	-0.07605083
2319	2.81250000	0.50000000	-0.05415445
2320	2.75000000	0.56250000	-0.08699902
2321	2.81250000	0.56250000	-0.06373412
2322	2.68750000	0.43750000	-0.06627566
2323	2.75000000	0.43750000	-0.05552297
2324	2.50000000	0.68750000	-0.12355816
2325	2.50000000	0.81250000	-0.13333333
2326	2.56250000	0.75000000	-0.13450635
2327	2.56250000	0.81250000	-0.12688172
2328	2.43750000	0.68750000	-0.13157380
2329	2.43750000	0.75000000	-0.13646139
2330	1.50000000	1.68750000	-0.19824047
2331	1.50000000	1.81250000	-0.19863148
2332	1.56250000	1.75000000	-0.19843597
2333	1.56250000	1.81250000	-0.19863148
2334	1.43750000	1.68750000	-0.19824047
2335	1.43750000	1.75000000	-0.19824047
2336	1.68750000	1.50000000	-0.19824047
2337	1.81250000	1.50000000	-0.19863148
2338	1.75000000	1.56250000	-0.19843597
2339	1.81250000	1.56250000	-0.19863148
2340	1.68750000	1.43750000	-0.19824047
2341	1.75000000	1.43750000	-0.19824047
2342	1.31250000	1.50000000	-0.19726295
2343	1.18750000	1.50000000	-0.19726295
2344	1.25000000	1.43750000	-0.19706745
2345	1.18750000	1.43750000	-0.19687195
2346	1.31250000	1.56250000	-0.19765396
2347	1.25000000	1.56250000	-0.19745846
2348	1.50000000	1.31250000	-0.19726295
2349	1.50000000	1.18750000	-0.19726295
2350	1.43750000	1.25000000	-0.19706745
2351	1.43750000	1.18750000	-0.19687195
2352	1.56250000	1.31250000	-0.19765396
2353	1.56250000	1.25000000	-0.19745846
2354	2.31250000	2.50000000	-0.12766373
2355	2.18750000	2.50000000	-0.14721408
2356	2.25000000	2.43750000	-0.14037146
2357	2.18750000	2.43750000	-0.15014663

2358	2.31250000	2.56250000	-0.12082111
2359	2.25000000	2.56250000	-0.13059629
2360	2.50000000	2.31250000	-0.13607038
2361	2.50000000	2.18750000	-0.16148583
2362	2.43750000	2.25000000	-0.14877810
2363	2.43750000	2.18750000	-0.16148583
2364	2.56250000	2.31250000	-0.12062561
2365	2.56250000	2.25000000	-0.13333333
2366	2.50000000	2.68750000	-0.07781036
2367	2.50000000	2.81250000	-0.06432063
2368	2.56250000	2.75000000	-0.05298143
2369	2.56250000	2.81250000	-0.04613881
2370	2.43750000	2.68750000	-0.08758553
2371	2.43750000	2.75000000	-0.08074291
2372	2.68750000	2.50000000	-0.05200391
2373	2.81250000	2.50000000	-0.02111437
2374	2.75000000	2.56250000	-0.02717498
2375	2.81250000	2.56250000	-0.01173021
2376	2.68750000	2.43750000	-0.06451613
2377	2.75000000	2.43750000	-0.04907136
2378	1.68750000	2.18750000	-0.19061584
2379	1.81250000	2.31250000	-0.18514174
2380	1.75000000	2.18750000	-0.19061584
2381	1.81250000	2.25000000	-0.18807429
2382	1.68750000	2.25000000	-0.18768328
2383	1.75000000	2.31250000	-0.18494624
2384	1.31250000	1.81250000	-0.19824047
2385	1.18750000	1.68750000	-0.19784946
2386	1.25000000	1.81250000	-0.19824047
2387	1.18750000	1.75000000	-0.19804497
2388	1.31250000	1.75000000	-0.19804497
2389	1.25000000	1.68750000	-0.19784946
2390	1.50000000	2.18750000	-0.19042033
2391	1.50000000	2.31250000	-0.18455523
2392	1.43750000	2.18750000	-0.19042033
2393	1.43750000	2.25000000	-0.18748778
2394	1.56250000	2.25000000	-0.18748778
2395	1.56250000	2.31250000	-0.18475073
2396	2.18750000	1.68750000	-0.19921799
2397	2.31250000	1.81250000	-0.19921799
2398	2.18750000	1.75000000	-0.19921799
2399	2.25000000	1.81250000	-0.19921799
2400	2.25000000	1.68750000	-0.19296188
2401	2.31250000	1.75000000	-0.19296188
2402	1.81250000	1.31250000	-0.19824047
2403	1.68750000	1.18750000	-0.19784946
2404	1.81250000	1.25000000	-0.19824047
2405	1.75000000	1.18750000	-0.19804497
2406	1.75000000	1.31250000	-0.19804497
2407	1.68750000	1.25000000	-0.19784946
2408	2.18750000	1.50000000	-0.18044966
2409	2.31250000	1.50000000	-0.16793744
2410	2.18750000	1.43750000	-0.18044966
2411	2.25000000	1.43750000	-0.17419355
2412	2.25000000	1.56250000	-0.18044966
2413	2.31250000	1.56250000	-0.17419355
2414	2.18750000	2.68750000	-0.12707722
2415	2.31250000	2.81250000	-0.09384164
2416	2.18750000	2.75000000	-0.12414467

2417	2.25000000	2.81250000	-0.10752688
2418	2.25000000	2.68750000	-0.11710655
2419	2.31250000	2.75000000	-0.10048876
2420	1.81250000	2.50000000	-0.17673509
2421	1.68750000	2.50000000	-0.17634409
2422	1.81250000	2.56250000	-0.17380254
2423	1.75000000	2.56250000	-0.17360704
2424	1.75000000	2.43750000	-0.17927664
2425	1.68750000	2.43750000	-0.17927664
2426	2.68750000	2.18750000	-0.11515152
2427	2.81250000	2.31250000	-0.05884653
2428	2.75000000	2.18750000	-0.10263930
2429	2.81250000	2.25000000	-0.07448680
2430	2.68750000	2.25000000	-0.10244379
2431	2.75000000	2.31250000	-0.07429130
2432	2.50000000	1.81250000	-0.18044966
2433	2.50000000	1.68750000	-0.16793744
2434	2.56250000	1.81250000	-0.16793744
2435	2.56250000	1.75000000	-0.16168133
2436	2.43750000	1.75000000	-0.18044966
2437	2.43750000	1.68750000	-0.17419355
2438	1.50000000	3.81250000	0.12785924
2439	1.50000000	3.68750000	0.08211144
2440	1.56250000	3.81250000	0.11769306
2441	1.56250000	3.75000000	0.09481916
2442	1.43750000	3.75000000	0.11006843
2443	1.43750000	3.68750000	0.08719453
2444	1.31250000	3.81250000	0.14310850
2445	1.18750000	3.68750000	0.10752688
2446	1.25000000	3.81250000	0.14193548
2447	1.18750000	3.75000000	0.12414467
2448	1.31250000	3.75000000	0.12023460
2449	1.25000000	3.68750000	0.10244379
2450	2.31250000	3.81250000	0.09775171
2451	2.18750000	3.68750000	0.05845552
2452	2.25000000	3.81250000	0.09266862
2453	2.18750000	3.75000000	0.07311828
2454	2.31250000	3.75000000	0.09051808
2455	2.25000000	3.68750000	0.07096774
2456	2.50000000	3.81250000	0.13528837
2457	2.50000000	3.68750000	0.12101662
2458	2.56250000	3.81250000	0.14056696
2459	2.56250000	3.75000000	0.13352884
2460	2.43750000	3.75000000	0.11554252
2461	2.43750000	3.68750000	0.10850440
2462	1.68750000	3.50000000	0.00821114
2463	1.81250000	3.50000000	0.00488759
2464	1.75000000	3.56250000	0.02111437
2465	1.81250000	3.56250000	0.01935484
2466	1.68750000	3.43750000	-0.00801564
2467	1.75000000	3.43750000	-0.00957967
2468	1.31250000	3.50000000	0.02873900
2469	1.18750000	3.50000000	0.03890518
2470	1.25000000	3.43750000	0.01739980
2471	1.18750000	3.43750000	0.02248289
2472	1.31250000	3.56250000	0.05161290
2473	1.25000000	3.56250000	0.05669599
2474	1.50000000	3.31250000	-0.03519062
2475	1.50000000	3.18750000	-0.06744868

2476	1.43750000	3.25000000	-0.04652981
2477	1.43750000	3.18750000	-0.06275660
2478	1.56250000	3.31250000	-0.03695015
2479	1.56250000	3.25000000	-0.05298143
2480	2.18750000	3.50000000	0.03734115
2481	2.31250000	3.50000000	0.06236559
2482	2.18750000	3.43750000	0.03069404
2483	2.25000000	3.43750000	0.03069404
2484	2.25000000	3.56250000	0.05689150
2485	2.31250000	3.56250000	0.06940371
2486	1.81250000	3.31250000	-0.04359726
2487	1.68750000	3.18750000	-0.07253177
2488	1.81250000	3.25000000	-0.05024438
2489	1.75000000	3.18750000	-0.07722385
2490	1.75000000	3.31250000	-0.04183773
2491	1.68750000	3.25000000	-0.05630499
2492	3.81250000	1.50000000	0.08465298
2493	3.68750000	1.50000000	0.06490714
2494	3.81250000	1.56250000	0.08817204
2495	3.75000000	1.56250000	0.07820137
2496	3.75000000	1.43750000	0.07585533
2497	3.68750000	1.43750000	0.06608016
2498	3.81250000	1.31250000	0.08778104
2499	3.68750000	1.18750000	0.06999022
2500	3.81250000	1.25000000	0.09638319
2501	3.75000000	1.18750000	0.08739003
2502	3.75000000	1.31250000	0.07781036
2503	3.68750000	1.25000000	0.06901271
2504	3.81250000	2.31250000	0.11867058
2505	3.68750000	2.18750000	0.07487781
2506	3.81250000	2.25000000	0.11339198
2507	3.75000000	2.18750000	0.09130010
2508	3.75000000	2.31250000	0.10048876
2509	3.68750000	2.25000000	0.07859238
2510	3.81250000	2.50000000	0.12961877
2511	3.68750000	2.50000000	0.09305963
2512	3.81250000	2.56250000	0.12844575
2513	3.75000000	2.56250000	0.11026393
2514	3.75000000	2.43750000	0.10772239
2515	3.68750000	2.43750000	0.08934506
2516	3.50000000	1.68750000	0.02521994
2517	3.50000000	1.81250000	0.01876833
2518	3.56250000	1.75000000	0.03870968
2519	3.56250000	1.81250000	0.03519062
2520	3.43750000	1.68750000	0.00879765
2521	3.43750000	1.75000000	0.00527859
2522	3.50000000	1.31250000	0.03831867
2523	3.50000000	1.18750000	0.04027370
2524	3.43750000	1.25000000	0.02170088
2525	3.43750000	1.18750000	0.02287390
2526	3.56250000	1.31250000	0.04828935
2527	3.56250000	1.25000000	0.04926686
2528	3.31250000	1.50000000	-0.01485826
2529	3.18750000	1.50000000	-0.04809384
2530	3.25000000	1.43750000	-0.03128055
2531	3.18750000	1.43750000	-0.06666667
2532	3.31250000	1.56250000	-0.01798631
2533	3.25000000	1.56250000	-0.03479961
2534	3.50000000	2.18750000	0.01994135

2535	3.50000000	2.31250000	0.02717498
2536	3.43750000	2.18750000	0.00645161
2537	3.43750000	2.25000000	0.00997067
2538	3.56250000	2.25000000	0.04203324
2539	3.56250000	2.31250000	0.04555230
2540	3.31250000	1.81250000	-0.03128055
2541	3.18750000	1.68750000	-0.05806452
2542	3.25000000	1.81250000	-0.04477028
2543	3.18750000	1.75000000	-0.05826002
2544	3.31250000	1.75000000	-0.02815249
2545	3.25000000	1.68750000	-0.04144673
2546	0.50000000	1.18750000	-0.17908113
2547	0.50000000	1.31250000	-0.17947214
2548	0.43750000	1.18750000	-0.17204301
2549	0.43750000	1.25000000	-0.17223851
2550	0.56250000	1.25000000	-0.18142717
2551	0.56250000	1.31250000	-0.18142717
2552	0.68750000	1.18750000	-0.18514174
2553	0.81250000	1.31250000	-0.18983382
2554	0.75000000	1.18750000	-0.18729228
2555	0.81250000	1.25000000	-0.18963832
2556	0.68750000	1.25000000	-0.18553275
2557	0.75000000	1.31250000	-0.18768328
2558	0.68750000	1.50000000	-0.18611926
2559	0.81250000	1.50000000	-0.19042033
2560	0.75000000	1.56250000	-0.18866080
2561	0.81250000	1.56250000	-0.19061584
2562	0.68750000	1.43750000	-0.18592375
2563	0.75000000	1.43750000	-0.18807429
2564	1.18750000	0.50000000	-0.17673509
2565	1.31250000	0.50000000	-0.17712610
2566	1.18750000	0.43750000	-0.16187683
2567	1.25000000	0.43750000	-0.16207234
2568	1.25000000	0.56250000	-0.17927664
2569	1.31250000	0.56250000	-0.17947214
2570	1.18750000	0.68750000	-0.18396872
2571	1.31250000	0.81250000	-0.18924731
2572	1.18750000	0.75000000	-0.18631476
2573	1.25000000	0.81250000	-0.18905181
2574	1.25000000	0.68750000	-0.18416422
2575	1.31250000	0.75000000	-0.18690127
2576	1.50000000	0.68750000	-0.18514174
2577	1.50000000	0.81250000	-0.18983382
2578	1.56250000	0.75000000	-0.18768328
2579	1.56250000	0.81250000	-0.19022483
2580	1.43750000	0.68750000	-0.18475073
2581	1.43750000	0.75000000	-0.18729228
2582	0.50000000	3.31250000	0.04203324
2583	0.50000000	3.18750000	0.00918866
2584	0.43750000	3.25000000	0.03734115
2585	0.43750000	3.18750000	0.01388074
2586	0.56250000	3.31250000	0.03753666
2587	0.56250000	3.25000000	0.02091887
2588	0.68750000	3.50000000	0.07741935
2589	0.81250000	3.50000000	0.06823069
2590	0.75000000	3.56250000	0.10185728
2591	0.81250000	3.56250000	0.08465298
2592	0.68750000	3.43750000	0.06119257
2593	0.75000000	3.43750000	0.05630499

2594	0.68750000	3.18750000	-0.00488759
2595	0.81250000	3.31250000	0.01857283
2596	0.75000000	3.18750000	-0.00957967
2597	0.81250000	3.25000000	0.00215054
2598	0.68750000	3.25000000	0.01153470
2599	0.75000000	3.31250000	0.02326491
2600	3.31250000	0.50000000	0.06647116
2601	3.18750000	0.50000000	0.03147605
2602	3.25000000	0.43750000	0.05865103
2603	3.18750000	0.43750000	0.04086022
2604	3.31250000	0.56250000	0.05708700
2605	3.25000000	0.56250000	0.03949169
2606	3.50000000	0.68750000	0.09071359
2607	3.50000000	0.81250000	0.07174976
2608	3.56250000	0.75000000	0.09872923
2609	3.56250000	0.81250000	0.08914956
2610	3.43750000	0.68750000	0.07311828
2611	3.43750000	0.75000000	0.06373412
2612	3.18750000	0.68750000	0.00312805
2613	3.31250000	0.81250000	0.01915934
2614	3.18750000	0.75000000	-0.00645161
2615	3.25000000	0.81250000	0.00175953
2616	3.25000000	0.68750000	0.02052786
2617	3.31250000	0.75000000	0.02873900
2618	2.81250000	3.31250000	0.07722385
2619	2.68750000	3.18750000	0.02873900
2620	2.81250000	3.25000000	0.07096774
2621	2.75000000	3.18750000	0.04672532
2622	2.75000000	3.31250000	0.07077224
2623	2.68750000	3.25000000	0.04672532
2624	2.81250000	3.50000000	0.13079179
2625	2.68750000	3.50000000	0.11827957
2626	2.81250000	3.56250000	0.13704790
2627	2.75000000	3.56250000	0.13079179
2628	2.75000000	3.43750000	0.10654936
2629	2.68750000	3.43750000	0.10029326
2630	3.31250000	2.81250000	0.09560117
2631	3.18750000	2.68750000	0.06744868
2632	3.25000000	2.81250000	0.09090909
2633	3.18750000	2.75000000	0.07683284
2634	3.31250000	2.75000000	0.08328446
2635	3.25000000	2.68750000	0.06920821
2636	3.50000000	2.81250000	0.10068426
2637	3.50000000	2.68750000	0.07565982
2638	3.56250000	2.81250000	0.10537634
2639	3.56250000	2.75000000	0.09286413
2640	3.43750000	2.75000000	0.08660802
2641	3.43750000	2.68750000	0.07409580
2642	2.50000000	3.18750000	0.00977517
2643	2.50000000	3.31250000	0.04555230
2644	2.43750000	3.18750000	-0.00391007
2645	2.43750000	3.25000000	0.01388074
2646	2.56250000	3.25000000	0.03401760
2647	2.56250000	3.31250000	0.05180841
2648	3.18750000	2.50000000	0.03030303
2649	3.31250000	2.50000000	0.03343109
2650	3.18750000	2.43750000	0.01466276
2651	3.25000000	2.43750000	0.01622678
2652	3.25000000	2.56250000	0.04437928

2653	3.31250000	2.56250000	0.04594330
2654	1.18750000	2.50000000	-0.15210166
2655	1.31250000	2.50000000	-0.16168133
2656	1.18750000	2.43750000	-0.15972630
2657	1.25000000	2.43750000	-0.16461388
2658	1.25000000	2.56250000	-0.14936461
2659	1.31250000	2.56250000	-0.15425220
2660	1.18750000	2.68750000	-0.12961877
2661	1.31250000	2.81250000	-0.12414467
2662	1.18750000	2.75000000	-0.12199413
2663	1.25000000	2.81250000	-0.11945259
2664	1.25000000	2.68750000	-0.13431085
2665	1.31250000	2.75000000	-0.13157380
2666	1.50000000	2.68750000	-0.15347019
2667	1.50000000	2.81250000	-0.13841642
2668	1.56250000	2.75000000	-0.15073314
2669	1.56250000	2.81250000	-0.14310850
2670	1.43750000	2.68750000	-0.14877810
2671	1.43750000	2.75000000	-0.14115347
2672	2.50000000	1.18750000	-0.14819159
2673	2.50000000	1.31250000	-0.14858260
2674	2.43750000	1.18750000	-0.15464321
2675	2.43750000	1.25000000	-0.15483871
2676	2.56250000	1.25000000	-0.14193548
2677	2.56250000	1.31250000	-0.14213099
2678	2.68750000	1.18750000	-0.12922776
2679	2.81250000	1.31250000	-0.11671554
2680	2.75000000	1.18750000	-0.12277615
2681	2.81250000	1.25000000	-0.13528837
2682	2.68750000	1.25000000	-0.12922776
2683	2.75000000	1.31250000	-0.12316716
2684	2.68750000	1.50000000	-0.13000978
2685	2.81250000	1.50000000	-0.11730205
2686	2.75000000	1.56250000	-0.12375367
2687	2.81250000	1.56250000	-0.11749756
2688	2.68750000	1.43750000	-0.12981427
2689	2.75000000	1.43750000	-0.12355816
2690	0.06250000	0.12500000	0.00332356
2691	0.06250000	0.18750000	-0.00703812
2692	0.12500000	0.06250000	0.00391007
2693	0.18750000	0.06250000	-0.00606061
2694	0.12500000	0.18750000	-0.00899316
2695	0.18750000	0.12500000	-0.00860215
2696	0.06250000	3.93750000	0.17478006
2697	0.06250000	3.87500000	0.16304985
2698	0.12500000	3.93750000	0.17458456
2699	3.93750000	0.06250000	0.18963832
2700	3.87500000	0.06250000	0.18064516
2701	3.93750000	0.12500000	0.18866080
2702	3.87500000	3.93750000	0.14076246
2703	3.93750000	3.87500000	0.17771261
2704	3.81250000	3.87500000	0.14330401
2705	3.87500000	3.81250000	0.18005865
2706	3.81250000	3.93750000	0.10400782
2707	3.93750000	3.81250000	0.17771261
2708	0.06250000	1.93750000	-0.13919844
2709	0.06250000	1.87500000	-0.14056696
2710	0.06250000	2.12500000	-0.09794721
2711	0.06250000	2.18750000	-0.06432063

2712	0.12500000	2.06250000	-0.13548387
2713	0.12500000	1.93750000	-0.14310850
2714	0.18750000	2.06250000	-0.13939394
2715	0.12500000	2.18750000	-0.09560117
2716	0.18750000	2.12500000	-0.13313783
2717	1.93750000	0.06250000	-0.07370479
2718	1.87500000	0.06250000	-0.02287390
2719	2.12500000	0.06250000	-0.10166178
2720	2.18750000	0.06250000	-0.08719453
2721	2.06250000	0.12500000	-0.12336266
2722	1.93750000	0.12500000	-0.08074291
2723	2.06250000	0.18750000	-0.13040078
2724	2.18750000	0.12500000	-0.10068426
2725	2.12500000	0.18750000	-0.12218964
2726	1.87500000	1.93750000	-0.19921799
2727	1.93750000	1.87500000	-0.19921799
2728	1.81250000	1.87500000	-0.19902248
2729	1.87500000	1.81250000	-0.19902248
2730	2.06250000	2.12500000	-0.18416422
2731	2.12500000	2.06250000	-0.18690127
2732	2.12500000	2.18750000	-0.17145650
2733	2.18750000	2.12500000	-0.17419355
2734	1.93750000	2.06250000	-0.19667644
2735	1.81250000	1.93750000	-0.19902248
2736	1.87500000	2.06250000	-0.19648094
2737	2.06250000	1.93750000	-0.19941349
2738	1.93750000	1.81250000	-0.19902248
2739	2.06250000	1.87500000	-0.19921799
2740	2.06250000	2.18750000	-0.18142717
2741	1.93750000	2.12500000	-0.19393939
2742	2.18750000	2.06250000	-0.18690127
2743	2.12500000	1.93750000	-0.19941349
2744	1.87500000	3.93750000	0.11300098
2745	1.81250000	3.93750000	0.12316716
2746	2.06250000	3.93750000	0.10654936
2747	2.12500000	3.93750000	0.11163245
2748	1.93750000	3.87500000	0.08856305
2749	1.81250000	3.87500000	0.10029326
2750	1.87500000	3.81250000	0.07565982
2751	2.06250000	3.87500000	0.09208211
2752	1.93750000	3.81250000	0.07409580
2753	3.93750000	1.87500000	0.12473118
2754	3.93750000	1.81250000	0.12140762
2755	3.93750000	2.06250000	0.13020528
2756	3.93750000	2.12500000	0.13567937
2757	3.87500000	1.93750000	0.11143695
2758	3.87500000	1.81250000	0.11143695
2759	3.81250000	1.87500000	0.09814272
2760	3.87500000	2.06250000	0.11358749
2761	3.81250000	1.93750000	0.09481916
2762	0.06250000	0.93750000	-0.12258065
2763	0.06250000	0.87500000	-0.11593353
2764	0.06250000	1.12500000	-0.13274682
2765	0.06250000	1.18750000	-0.13607038
2766	0.12500000	1.06250000	-0.13646139
2767	0.12500000	0.93750000	-0.12961877
2768	0.18750000	1.06250000	-0.14349951
2769	0.12500000	1.18750000	-0.13998045
2770	0.18750000	1.12500000	-0.14369501

2771	0.93750000	0.06250000	-0.07311828
2772	0.87500000	0.06250000	-0.07409580
2773	1.12500000	0.06250000	-0.07898338
2774	1.18750000	0.06250000	-0.08563050
2775	1.06250000	0.12500000	-0.08719453
2776	0.93750000	0.12500000	-0.08797654
2777	1.06250000	0.18750000	-0.10205279
2778	1.18750000	0.12500000	-0.09403715
2779	1.12500000	0.18750000	-0.10224829
2780	0.87500000	0.93750000	-0.17947214
2781	0.93750000	0.87500000	-0.17908113
2782	0.81250000	0.87500000	-0.16578690
2783	0.87500000	0.81250000	-0.16539589
2784	1.06250000	1.12500000	-0.19589443
2785	1.12500000	1.06250000	-0.19589443
2786	1.12500000	1.18750000	-0.19608993
2787	1.18750000	1.12500000	-0.19608993
2788	0.93750000	1.06250000	-0.19335288
2789	0.81250000	0.93750000	-0.17732160
2790	0.87500000	1.06250000	-0.19120235
2791	1.06250000	0.93750000	-0.19296188
2792	0.93750000	0.81250000	-0.17653959
2793	1.06250000	0.87500000	-0.19061584
2794	1.06250000	1.18750000	-0.19589443
2795	0.93750000	1.12500000	-0.19354839
2796	1.18750000	1.06250000	-0.19589443
2797	1.12500000	0.93750000	-0.19335288
2798	0.06250000	3.12500000	0.02052786
2799	0.06250000	3.18750000	0.03245357
2800	0.06250000	2.93750000	-0.01564027
2801	0.06250000	2.87500000	-0.02306940
2802	0.12500000	3.06250000	0.00410557
2803	0.12500000	3.18750000	0.03245357
2804	0.18750000	3.12500000	0.06510264
2805	0.12500000	2.93750000	-0.01974585
2806	0.18750000	3.06250000	0.04868035
2807	0.87500000	3.93750000	0.18670577
2808	0.81250000	3.93750000	0.18651026
2809	1.06250000	3.93750000	0.17086999
2810	1.12500000	3.93750000	0.17223851
2811	0.93750000	3.87500000	0.17028348
2812	0.81250000	3.87500000	0.18729228
2813	0.87500000	3.81250000	0.17106549
2814	1.06250000	3.87500000	0.15444770
2815	0.93750000	3.81250000	0.15386119
2816	3.12500000	0.06250000	0.07194526
2817	3.18750000	0.06250000	0.08093842
2818	2.93750000	0.06250000	0.03440860
2819	2.87500000	0.06250000	0.02365591
2820	3.06250000	0.12500000	0.05337243
2821	3.18750000	0.12500000	0.07996090
2822	3.12500000	0.18750000	0.06138807
2823	2.93750000	0.12500000	0.02502444
2824	3.06250000	0.18750000	0.04379277
2825	3.93750000	0.87500000	0.17634409
2826	3.93750000	0.81250000	0.17751711
2827	3.93750000	1.06250000	0.15718475
2828	3.93750000	1.12500000	0.14858260
2829	3.87500000	0.93750000	0.15777126

2830	3.87500000	0.81250000	0.16832845
2831	3.81250000	0.87500000	0.14956012
2832	3.87500000	1.06250000	0.13978495
2833	3.81250000	0.93750000	0.14017595
2834	3.06250000	3.12500000	0.11827957
2835	3.12500000	3.06250000	0.11671554
2836	3.12500000	3.18750000	0.12981427
2837	3.18750000	3.12500000	0.12844575
2838	2.87500000	2.93750000	0.05747801
2839	2.93750000	2.87500000	0.06608016
2840	2.81250000	2.87500000	0.03264907
2841	2.87500000	2.81250000	0.04125122
2842	2.93750000	3.06250000	0.08856305
2843	3.06250000	3.18750000	0.12434018
2844	2.93750000	3.12500000	0.09462366
2845	3.06250000	2.93750000	0.09560117
2846	3.18750000	3.06250000	0.12140762
2847	3.12500000	2.93750000	0.10029326
2848	2.81250000	2.93750000	0.03949169
2849	2.87500000	3.06250000	0.07038123
2850	2.93750000	2.81250000	0.05669599
2851	3.06250000	2.87500000	0.08621701
2852	3.06250000	3.93750000	0.15327468
2853	3.12500000	3.93750000	0.11358749
2854	2.87500000	3.93750000	0.18162268
2855	2.81250000	3.93750000	0.17634409
2856	2.93750000	3.87500000	0.18064516
2857	3.06250000	3.87500000	0.14701857
2858	2.93750000	3.81250000	0.17438905
2859	2.81250000	3.87500000	0.16930596
2860	2.87500000	3.81250000	0.16832845
2861	3.93750000	3.06250000	0.17067449
2862	3.93750000	3.12500000	0.17067449
2863	3.93750000	2.87500000	0.15953079
2864	3.93750000	2.81250000	0.16070381
2865	3.87500000	2.93750000	0.15366569
2866	3.87500000	3.06250000	0.16598240
2867	3.81250000	2.93750000	0.14897361
2868	3.87500000	2.81250000	0.14252199
2869	3.81250000	2.87500000	0.13665689
2870	0.87500000	1.93750000	-0.19217986
2871	0.93750000	2.06250000	-0.18690127
2872	0.81250000	1.93750000	-0.18807429
2873	0.87500000	2.06250000	-0.18279570
2874	1.06250000	1.93750000	-0.19843597
2875	1.12500000	2.06250000	-0.19589443
2876	1.12500000	1.93750000	-0.19843597
2877	1.18750000	2.06250000	-0.19608993
2878	0.93750000	1.87500000	-0.19608993
2879	0.81250000	1.87500000	-0.19002933
2880	0.87500000	1.81250000	-0.19374389
2881	1.06250000	1.87500000	-0.19804497
2882	0.93750000	1.81250000	-0.19569892
2883	1.06250000	2.12500000	-0.18826979
2884	0.93750000	2.12500000	-0.17947214
2885	1.06250000	2.18750000	-0.18064516
2886	1.18750000	2.12500000	-0.19315738
2887	1.12500000	2.18750000	-0.18533724
2888	0.87500000	2.93750000	-0.08074291

2889	0.93750000	2.87500000	-0.09286413
2890	0.81250000	2.87500000	-0.08875855
2891	0.87500000	2.81250000	-0.10068426
2892	1.06250000	3.12500000	-0.04985337
2893	1.12500000	3.06250000	-0.07116325
2894	1.12500000	3.18750000	-0.03851417
2895	1.18750000	3.12500000	-0.05962854
2896	0.93750000	3.06250000	-0.05650049
2897	0.81250000	2.93750000	-0.07624633
2898	0.87500000	3.06250000	-0.05180841
2899	1.06250000	3.18750000	-0.03343109
2900	0.93750000	3.12500000	-0.04007820
2901	1.06250000	2.93750000	-0.08993157
2902	0.93750000	2.81250000	-0.10029326
2903	1.06250000	2.87500000	-0.09736070
2904	1.18750000	3.06250000	-0.07585533
2905	1.12500000	2.93750000	-0.09481916
2906	1.93750000	0.87500000	-0.19335288
2907	2.06250000	0.93750000	-0.18963832
2908	1.93750000	0.81250000	-0.19022483
2909	2.06250000	0.87500000	-0.18651026
2910	1.93750000	1.06250000	-0.19882698
2911	2.06250000	1.12500000	-0.19296188
2912	1.93750000	1.12500000	-0.19882698
2913	2.06250000	1.18750000	-0.19296188
2914	1.87500000	0.93750000	-0.19608993
2915	1.87500000	0.81250000	-0.19061584
2916	1.81250000	0.87500000	-0.19354839
2917	1.87500000	1.06250000	-0.19843597
2918	1.81250000	0.93750000	-0.19589443
2919	2.12500000	1.06250000	-0.18651026
2920	2.12500000	0.93750000	-0.18318671
2921	2.18750000	1.06250000	-0.18005865
2922	2.12500000	1.18750000	-0.18670577
2923	2.18750000	1.12500000	-0.18025415
2924	2.93750000	0.87500000	-0.10146628
2925	2.87500000	0.93750000	-0.11730205
2926	2.87500000	0.81250000	-0.11554252
2927	2.81250000	0.87500000	-0.13137830
2928	3.12500000	1.06250000	-0.06295210
2929	3.06250000	1.12500000	-0.09931574
2930	3.18750000	1.12500000	-0.04633431
2931	3.12500000	1.18750000	-0.08269795
2932	3.06250000	0.93750000	-0.06999022
2933	2.93750000	0.81250000	-0.09208211
2934	3.06250000	0.87500000	-0.06041056
2935	3.18750000	1.06250000	-0.04535679
2936	3.12500000	0.93750000	-0.05239492
2937	2.93750000	1.06250000	-0.12218964
2938	2.81250000	0.93750000	-0.12375367
2939	2.87500000	1.06250000	-0.12844575
2940	3.06250000	1.18750000	-0.11808407
2941	2.93750000	1.12500000	-0.14095797
2942	2.06250000	2.93750000	-0.14291300
2943	1.93750000	2.87500000	-0.15933529
2944	2.06250000	2.87500000	-0.14565005
2945	1.93750000	2.81250000	-0.16226784
2946	2.06250000	3.12500000	-0.09032258
2947	1.93750000	3.06250000	-0.11710655

2948	2.06250000	3.18750000	-0.05845552
2949	1.93750000	3.12500000	-0.08543500
2950	2.12500000	3.06250000	-0.10850440
2951	2.12500000	2.93750000	-0.12922776
2952	2.18750000	3.06250000	-0.09462366
2953	2.12500000	3.18750000	-0.05865103
2954	2.18750000	3.12500000	-0.07663734
2955	1.87500000	2.93750000	-0.15171065
2956	1.87500000	2.81250000	-0.16226784
2957	1.81250000	2.87500000	-0.15464321
2958	1.87500000	3.06250000	-0.11241447
2959	1.81250000	2.93750000	-0.14701857
2960	2.93750000	2.06250000	-0.09638319
2961	2.87500000	1.93750000	-0.11808407
2962	2.87500000	2.06250000	-0.10889541
2963	2.81250000	1.93750000	-0.13059629
2964	3.12500000	2.06250000	-0.06862170
2965	3.06250000	1.93750000	-0.08563050
2966	3.18750000	2.06250000	-0.05493646
2967	3.12500000	1.93750000	-0.07194526
2968	3.06250000	2.12500000	-0.06666667
2969	2.93750000	2.12500000	-0.08074291
2970	3.06250000	2.18750000	-0.05102639
2971	3.18750000	2.12500000	-0.05141740
2972	3.12500000	2.18750000	-0.04946237
2973	2.93750000	1.87500000	-0.10537634
2974	2.81250000	1.87500000	-0.12414467
2975	2.87500000	1.81250000	-0.11143695
2976	3.06250000	1.87500000	-0.08543500
2977	2.93750000	1.81250000	-0.10518084
2978	0.06250000	0.43750000	-0.05747801
2979	0.06250000	0.37500000	-0.04418377
2980	0.06250000	0.62500000	-0.08934506
2981	0.06250000	0.68750000	-0.09599218
2982	0.12500000	0.56250000	-0.08465298
2983	0.12500000	0.43750000	-0.05962854
2984	0.18750000	0.56250000	-0.08660802
2985	0.12500000	0.68750000	-0.10303030
2986	0.18750000	0.62500000	-0.09833822
2987	0.43750000	0.06250000	-0.05474096
2988	0.37500000	0.06250000	-0.04183773
2989	0.62500000	0.06250000	-0.07800587
2990	0.68750000	0.06250000	-0.07702835
2991	0.56250000	0.12500000	-0.08152493
2992	0.43750000	0.12500000	-0.05728250
2993	0.56250000	0.18750000	-0.08406647
2994	0.68750000	0.12500000	-0.09188661
2995	0.62500000	0.18750000	-0.09540567
2996	0.37500000	0.43750000	-0.06803519
2997	0.43750000	0.37500000	-0.06744868
2998	0.31250000	0.37500000	-0.05239492
2999	0.37500000	0.31250000	-0.05200391
3000	0.56250000	0.62500000	-0.11104594
3001	0.62500000	0.56250000	-0.11065494
3002	0.62500000	0.68750000	-0.12473118
3003	0.68750000	0.62500000	-0.12434018
3004	0.43750000	0.56250000	-0.09521017
3005	0.31250000	0.43750000	-0.06588465
3006	0.37500000	0.56250000	-0.09305963

3007	0.56250000	0.43750000	-0.09423265
3008	0.43750000	0.31250000	-0.06490714
3009	0.56250000	0.37500000	-0.09169110
3010	0.56250000	0.68750000	-0.12258065
3011	0.43750000	0.62500000	-0.10674487
3012	0.68750000	0.56250000	-0.12179863
3013	0.62500000	0.43750000	-0.10557185
3014	0.06250000	3.62500000	0.11534702
3015	0.06250000	3.68750000	0.12727273
3016	0.06250000	3.43750000	0.07996090
3017	0.06250000	3.37500000	0.06823069
3018	0.12500000	3.56250000	0.10342131
3019	0.12500000	3.68750000	0.12707722
3020	0.18750000	3.62500000	0.11515152
3021	0.12500000	3.43750000	0.07996090
3022	0.18750000	3.56250000	0.10342131
3023	0.37500000	3.93750000	0.17360704
3024	0.31250000	3.93750000	0.17380254
3025	0.56250000	3.93750000	0.17321603
3026	0.62500000	3.93750000	0.17341153
3027	0.43750000	3.87500000	0.16148583
3028	0.31250000	3.87500000	0.16187683
3029	0.37500000	3.81250000	0.14995112
3030	0.56250000	3.87500000	0.16148583
3031	0.43750000	3.81250000	0.14975562
3032	3.62500000	0.06250000	0.14447703
3033	3.68750000	0.06250000	0.15366569
3034	3.43750000	0.06250000	0.11730205
3035	3.37500000	0.06250000	0.10830890
3036	3.56250000	0.12500000	0.13431085
3037	3.68750000	0.12500000	0.15249267
3038	3.62500000	0.18750000	0.14232649
3039	3.43750000	0.12500000	0.11632454
3040	3.56250000	0.18750000	0.13333333
3041	3.93750000	0.37500000	0.18455523
3042	3.93750000	0.31250000	0.18572825
3043	3.93750000	0.56250000	0.18142717
3044	3.93750000	0.62500000	0.18044966
3045	3.87500000	0.43750000	0.17438905
3046	3.87500000	0.31250000	0.17653959
3047	3.81250000	0.37500000	0.16637341
3048	3.87500000	0.56250000	0.17243402
3049	3.81250000	0.43750000	0.16539589
3050	3.56250000	3.62500000	0.15268817
3051	3.62500000	3.56250000	0.18944282
3052	3.62500000	3.68750000	0.15034213
3053	3.68750000	3.62500000	0.18709677
3054	3.37500000	3.43750000	0.17673509
3055	3.43750000	3.37500000	0.17536657
3056	3.31250000	3.37500000	0.16500489
3057	3.37500000	3.31250000	0.16363636
3058	3.43750000	3.56250000	0.14936461
3059	3.56250000	3.68750000	0.11358749
3060	3.43750000	3.62500000	0.11026393
3061	3.56250000	3.43750000	0.18475073
3062	3.68750000	3.56250000	0.18709677
3063	3.62500000	3.43750000	0.18240469
3064	3.31250000	3.43750000	0.17106549
3065	3.37500000	3.56250000	0.14369501

3066	3.43750000	3.31250000	0.16832845
3067	3.56250000	3.37500000	0.17771261
3068	3.56250000	3.93750000	-0.04320626
3069	3.62500000	3.93750000	-0.00645161
3070	3.37500000	3.93750000	-0.04594330
3071	3.31250000	3.93750000	-0.00606061
3072	3.43750000	3.87500000	-0.04652981
3073	3.56250000	3.87500000	-0.00410557
3074	3.43750000	3.81250000	-0.00742913
3075	3.31250000	3.87500000	-0.01231672
3076	3.37500000	3.81250000	-0.01309873
3077	3.93750000	3.56250000	0.17771261
3078	3.93750000	3.62500000	0.17771261
3079	3.93750000	3.37500000	0.17067449
3080	3.93750000	3.31250000	0.17067449
3081	3.87500000	3.43750000	0.17302053
3082	3.87500000	3.56250000	0.18005865
3083	3.81250000	3.43750000	0.17536657
3084	3.87500000	3.31250000	0.16598240
3085	3.81250000	3.37500000	0.16832845
3086	0.06250000	1.62500000	-0.14799609
3087	0.06250000	1.68750000	-0.14604106
3088	0.06250000	1.43750000	-0.14858260
3089	0.06250000	1.37500000	-0.14525904
3090	0.12500000	1.56250000	-0.15405670
3091	0.12500000	1.68750000	-0.15034213
3092	0.18750000	1.62500000	-0.15620723
3093	0.12500000	1.43750000	-0.15249267
3094	0.18750000	1.56250000	-0.15796676
3095	0.06250000	2.43750000	-0.05845552
3096	0.06250000	2.37500000	-0.04183773
3097	0.06250000	2.62500000	-0.05454545
3098	0.06250000	2.68750000	-0.09208211
3099	0.12500000	2.56250000	-0.07174976
3100	0.12500000	2.43750000	-0.06764418
3101	0.18750000	2.56250000	-0.08113392
3102	0.12500000	2.68750000	-0.09677419
3103	0.18750000	2.62500000	-0.06862170
3104	0.37500000	1.93750000	-0.15972630
3105	0.43750000	2.06250000	-0.15874878
3106	0.31250000	1.93750000	-0.15542522
3107	0.37500000	2.06250000	-0.15464321
3108	0.56250000	1.93750000	-0.17184751
3109	0.62500000	2.06250000	-0.16656891
3110	0.62500000	1.93750000	-0.17595308
3111	0.68750000	2.06250000	-0.17067449
3112	0.43750000	1.87500000	-0.16559140
3113	0.31250000	1.87500000	-0.15738025
3114	0.37500000	1.81250000	-0.16324536
3115	0.56250000	2.12500000	-0.15933529
3116	0.43750000	2.12500000	-0.15562072
3117	0.56250000	2.18750000	-0.15620723
3118	0.56250000	1.87500000	-0.17360704
3119	0.43750000	1.81250000	-0.16715543
3120	0.68750000	2.12500000	-0.16304985
3121	0.62500000	2.18750000	-0.15581623
3122	0.37500000	2.43750000	-0.12707722
3123	0.43750000	2.37500000	-0.13958944
3124	0.31250000	2.37500000	-0.12297165

3125	0.37500000	2.31250000	-0.13567937
3126	0.56250000	2.62500000	-0.12003910
3127	0.62500000	2.56250000	-0.12551320
3128	0.62500000	2.68750000	-0.11202346
3129	0.68750000	2.62500000	-0.12434018
3130	0.43750000	2.56250000	-0.11867058
3131	0.31250000	2.43750000	-0.11769306
3132	0.37500000	2.56250000	-0.10928641
3133	0.56250000	2.43750000	-0.13665689
3134	0.43750000	2.31250000	-0.14291300
3135	0.56250000	2.37500000	-0.14662757
3136	0.56250000	2.68750000	-0.10752688
3137	0.43750000	2.62500000	-0.10615836
3138	0.68750000	2.56250000	-0.12531769
3139	0.62500000	2.43750000	-0.13646139
3140	1.62500000	0.06250000	-0.09286413
3141	1.68750000	0.06250000	-0.04379277
3142	1.43750000	0.06250000	-0.02502444
3143	1.37500000	0.06250000	-0.01837732
3144	1.56250000	0.12500000	-0.12727273
3145	1.68750000	0.12500000	-0.07781036
3146	1.62500000	0.18750000	-0.13528837
3147	1.43750000	0.12500000	-0.12101662
3148	1.56250000	0.18750000	-0.13567937
3149	2.43750000	0.06250000	-0.04789834
3150	2.37500000	0.06250000	-0.05474096
3151	2.62500000	0.06250000	-0.01935484
3152	2.68750000	0.06250000	-0.00879765
3153	2.56250000	0.12500000	-0.03968719
3154	2.43750000	0.12500000	-0.05747801
3155	2.56250000	0.18750000	-0.04946237
3156	2.68750000	0.12500000	-0.01818182
3157	2.62500000	0.18750000	-0.03851417
3158	1.93750000	0.37500000	-0.15913978
3159	2.06250000	0.43750000	-0.15874878
3160	1.93750000	0.31250000	-0.15190616
3161	2.06250000	0.37500000	-0.15151515
3162	1.93750000	0.56250000	-0.17771261
3163	2.06250000	0.62500000	-0.17399804
3164	1.93750000	0.62500000	-0.18084066
3165	2.06250000	0.68750000	-0.17712610
3166	1.87500000	0.43750000	-0.16676442
3167	1.87500000	0.31250000	-0.15131965
3168	1.81250000	0.37500000	-0.15894428
3169	2.12500000	0.56250000	-0.16265885
3170	2.12500000	0.43750000	-0.15053763
3171	2.18750000	0.56250000	-0.15444770
3172	1.87500000	0.56250000	-0.17810362
3173	1.81250000	0.43750000	-0.16715543
3174	2.12500000	0.68750000	-0.17067449
3175	2.18750000	0.62500000	-0.15933529
3176	2.43750000	0.37500000	-0.10009775
3177	2.37500000	0.43750000	-0.11788856
3178	2.37500000	0.31250000	-0.10127077
3179	2.31250000	0.37500000	-0.11906158
3180	2.62500000	0.56250000	-0.10889541
3181	2.56250000	0.62500000	-0.12473118
3182	2.68750000	0.62500000	-0.11984360
3183	2.62500000	0.68750000	-0.13567937

3184	2.56250000	0.43750000	-0.08817204
3185	2.43750000	0.31250000	-0.09051808
3186	2.56250000	0.37500000	-0.07839687
3187	2.43750000	0.56250000	-0.12179863
3188	2.31250000	0.43750000	-0.12609971
3189	2.37500000	0.56250000	-0.12981427
3190	2.68750000	0.56250000	-0.09794721
3191	2.62500000	0.43750000	-0.07722385
3192	2.56250000	0.68750000	-0.12961877
3193	2.43750000	0.62500000	-0.12668622
3194	1.56250000	1.62500000	-0.19804497
3195	1.62500000	1.56250000	-0.19804497
3196	1.62500000	1.68750000	-0.19824047
3197	1.68750000	1.62500000	-0.19824047
3198	1.37500000	1.43750000	-0.19726295
3199	1.43750000	1.37500000	-0.19726295
3200	1.31250000	1.37500000	-0.19706745
3201	1.37500000	1.31250000	-0.19706745
3202	1.43750000	1.56250000	-0.19765396
3203	1.56250000	1.68750000	-0.19824047
3204	1.43750000	1.62500000	-0.19804497
3205	1.56250000	1.43750000	-0.19765396
3206	1.68750000	1.56250000	-0.19824047
3207	1.62500000	1.43750000	-0.19804497
3208	1.31250000	1.43750000	-0.19706745
3209	1.37500000	1.56250000	-0.19765396
3210	1.43750000	1.31250000	-0.19706745
3211	1.56250000	1.37500000	-0.19765396
3212	2.37500000	2.43750000	-0.12062561
3213	2.43750000	2.37500000	-0.12336266
3214	2.31250000	2.37500000	-0.13333333
3215	2.37500000	2.31250000	-0.13607038
3216	2.56250000	2.62500000	-0.06666667
3217	2.62500000	2.56250000	-0.05806452
3218	2.62500000	2.68750000	-0.04183773
3219	2.68750000	2.62500000	-0.03323558
3220	2.43750000	2.56250000	-0.10127077
3221	2.31250000	2.43750000	-0.13059629
3222	2.37500000	2.56250000	-0.11104594
3223	2.56250000	2.43750000	-0.09540567
3224	2.43750000	2.31250000	-0.13607038
3225	2.56250000	2.37500000	-0.10791789
3226	2.56250000	2.68750000	-0.05982405
3227	2.43750000	2.62500000	-0.09442815
3228	2.68750000	2.56250000	-0.04261975
3229	2.62500000	2.43750000	-0.07996090
3230	1.56250000	1.93750000	-0.19902248
3231	1.62500000	2.06250000	-0.19628543
3232	1.62500000	1.93750000	-0.19902248
3233	1.68750000	2.06250000	-0.19628543
3234	1.37500000	1.93750000	-0.19882698
3235	1.43750000	2.06250000	-0.19628543
3236	1.31250000	1.93750000	-0.19882698
3237	1.37500000	2.06250000	-0.19628543
3238	1.43750000	1.87500000	-0.19863148
3239	1.56250000	1.87500000	-0.19882698
3240	1.43750000	1.81250000	-0.19843597
3241	1.56250000	2.12500000	-0.19335288
3242	1.68750000	2.12500000	-0.19335288

3243	1.62500000	2.18750000	-0.19042033
3244	1.31250000	1.87500000	-0.19843597
3245	1.37500000	1.81250000	-0.19824047
3246	1.43750000	2.12500000	-0.19335288
3247	1.56250000	2.18750000	-0.19042033
3248	1.93750000	1.56250000	-0.19902248
3249	2.06250000	1.62500000	-0.19921799
3250	1.93750000	1.62500000	-0.19902248
3251	2.06250000	1.68750000	-0.19921799
3252	1.93750000	1.37500000	-0.19882698
3253	2.06250000	1.43750000	-0.19296188
3254	1.93750000	1.31250000	-0.19882698
3255	2.06250000	1.37500000	-0.19296188
3256	1.87500000	1.43750000	-0.19863148
3257	1.87500000	1.56250000	-0.19882698
3258	1.81250000	1.43750000	-0.19843597
3259	2.12500000	1.56250000	-0.19296188
3260	2.12500000	1.68750000	-0.19921799
3261	2.18750000	1.62500000	-0.19296188
3262	1.87500000	1.31250000	-0.19843597
3263	1.81250000	1.37500000	-0.19824047
3264	2.12500000	1.43750000	-0.18670577
3265	2.18750000	1.56250000	-0.18670577
3266	2.06250000	2.43750000	-0.16989247
3267	1.93750000	2.37500000	-0.18260020
3268	2.06250000	2.37500000	-0.17282502
3269	1.93750000	2.31250000	-0.18553275
3270	2.06250000	2.62500000	-0.15738025
3271	1.93750000	2.56250000	-0.17399804
3272	2.06250000	2.68750000	-0.15444770
3273	1.93750000	2.62500000	-0.17106549
3274	2.12500000	2.56250000	-0.15034213
3275	2.12500000	2.43750000	-0.15992180
3276	2.18750000	2.56250000	-0.14037146
3277	1.87500000	2.43750000	-0.17966764
3278	1.87500000	2.31250000	-0.18533724
3279	1.81250000	2.37500000	-0.18220919
3280	2.12500000	2.68750000	-0.14076246
3281	2.18750000	2.62500000	-0.13372434
3282	1.87500000	2.56250000	-0.17399804
3283	1.81250000	2.43750000	-0.17947214
3284	2.43750000	2.06250000	-0.18690127
3285	2.37500000	1.93750000	-0.19941349
3286	2.37500000	2.06250000	-0.18690127
3287	2.31250000	1.93750000	-0.19941349
3288	2.62500000	2.06250000	-0.15894428
3289	2.56250000	1.93750000	-0.18064516
3290	2.68750000	2.06250000	-0.14643206
3291	2.62500000	1.93750000	-0.16813294
3292	2.56250000	2.12500000	-0.15874878
3293	2.43750000	2.12500000	-0.17419355
3294	2.56250000	2.18750000	-0.14604106
3295	2.43750000	1.87500000	-0.19296188
3296	2.31250000	1.87500000	-0.19921799
3297	2.37500000	1.81250000	-0.19296188
3298	2.68750000	2.12500000	-0.13079179
3299	2.62500000	2.18750000	-0.13059629
3300	2.56250000	1.87500000	-0.17419355
3301	2.43750000	1.81250000	-0.18670577

3302	1.56250000	3.93750000	0.16344086
3303	1.62500000	3.93750000	0.15327468
3304	1.37500000	3.93750000	0.17732160
3305	1.31250000	3.93750000	0.17614858
3306	1.43750000	3.87500000	0.15581623
3307	1.56250000	3.87500000	0.14056696
3308	1.43750000	3.81250000	0.13294233
3309	1.31250000	3.87500000	0.15972630
3310	1.37500000	3.81250000	0.13802542
3311	2.37500000	3.93750000	0.13196481
3312	2.31250000	3.93750000	0.12688172
3313	2.56250000	3.93750000	0.15503421
3314	2.62500000	3.93750000	0.16031281
3315	2.43750000	3.87500000	0.13000978
3316	2.31250000	3.87500000	0.11241447
3317	2.37500000	3.81250000	0.11026393
3318	2.56250000	3.87500000	0.14780059
3319	2.43750000	3.81250000	0.12277615
3320	1.56250000	3.62500000	0.04907136
3321	1.62500000	3.56250000	0.02443793
3322	1.62500000	3.68750000	0.06177908
3323	1.68750000	3.62500000	0.03734115
3324	1.37500000	3.43750000	0.00703812
3325	1.43750000	3.37500000	-0.01427175
3326	1.31250000	3.37500000	-0.00430108
3327	1.37500000	3.31250000	-0.02561095
3328	1.43750000	3.56250000	0.04144673
3329	1.56250000	3.68750000	0.07194526
3330	1.43750000	3.62500000	0.06432063
3331	1.56250000	3.43750000	-0.00449658
3332	1.68750000	3.56250000	0.02287390
3333	1.62500000	3.43750000	-0.00625611
3334	1.31250000	3.43750000	0.01231672
3335	1.37500000	3.56250000	0.04652981
3336	1.43750000	3.31250000	-0.03030303
3337	1.56250000	3.37500000	-0.02072336
3338	2.06250000	3.62500000	0.03382209
3339	1.93750000	3.56250000	0.01603128
3340	2.06250000	3.68750000	0.04848485
3341	1.93750000	3.62500000	0.03049853
3342	2.06250000	3.43750000	0.00566960
3343	1.93750000	3.37500000	-0.02130987
3344	2.06250000	3.37500000	-0.00097752
3345	1.93750000	3.31250000	-0.02795699
3346	2.12500000	3.56250000	0.03186706
3347	2.12500000	3.68750000	0.05356794
3348	2.18750000	3.62500000	0.05141740
3349	1.87500000	3.43750000	-0.01290323
3350	1.87500000	3.56250000	0.01779081
3351	1.81250000	3.43750000	-0.01114370
3352	2.12500000	3.43750000	0.01818182
3353	2.18750000	3.56250000	0.04437928
3354	1.87500000	3.31250000	-0.03577713
3355	1.81250000	3.37500000	-0.02737048
3356	3.93750000	1.56250000	0.10791789
3357	3.93750000	1.62500000	0.11143695
3358	3.93750000	1.37500000	0.11417400
3359	3.93750000	1.31250000	0.12277615
3360	3.87500000	1.43750000	0.09560117

3361	3.87500000	1.56250000	0.09794721
3362	3.81250000	1.43750000	0.08563050
3363	3.87500000	1.31250000	0.10537634
3364	3.81250000	1.37500000	0.08680352
3365	3.93750000	2.37500000	0.15718475
3366	3.93750000	2.31250000	0.15190616
3367	3.93750000	2.56250000	0.16520039
3368	3.93750000	2.62500000	0.16402737
3369	3.87500000	2.43750000	0.14428152
3370	3.87500000	2.31250000	0.13528837
3371	3.81250000	2.37500000	0.12238514
3372	3.87500000	2.56250000	0.14682307
3373	3.81250000	2.43750000	0.12609971
3374	3.62500000	1.56250000	0.05826002
3375	3.56250000	1.62500000	0.04496579
3376	3.68750000	1.62500000	0.07155425
3377	3.62500000	1.68750000	0.05845552
3378	3.43750000	1.37500000	0.01974585
3379	3.37500000	1.43750000	0.00195503
3380	3.37500000	1.31250000	0.00312805
3381	3.31250000	1.37500000	-0.01446725
3382	3.56250000	1.43750000	0.04613881
3383	3.68750000	1.56250000	0.06823069
3384	3.62500000	1.43750000	0.05610948
3385	3.43750000	1.56250000	0.01505376
3386	3.56250000	1.68750000	0.04183773
3387	3.43750000	1.62500000	0.01192571
3388	3.43750000	1.31250000	0.02072336
3389	3.56250000	1.37500000	0.04731183
3390	3.31250000	1.43750000	-0.01466276
3391	3.37500000	1.56250000	-0.00136852
3392	3.62500000	2.06250000	0.04731183
3393	3.56250000	1.93750000	0.02854350
3394	3.68750000	2.06250000	0.06373412
3395	3.62500000	1.93750000	0.04496579
3396	3.43750000	2.06250000	-0.00097752
3397	3.37500000	1.93750000	-0.01798631
3398	3.37500000	2.06250000	-0.01446725
3399	3.31250000	1.93750000	-0.03147605
3400	3.56250000	2.12500000	0.03460411
3401	3.68750000	2.12500000	0.06940371
3402	3.62500000	2.18750000	0.05669599
3403	3.43750000	1.87500000	-0.00117302
3404	3.56250000	1.87500000	0.03186706
3405	3.43750000	1.81250000	0.00195503
3406	3.43750000	2.12500000	0.00273705
3407	3.56250000	2.18750000	0.03831867
3408	3.31250000	1.87500000	-0.03128055
3409	3.37500000	1.81250000	-0.01466276
3410	0.37500000	0.93750000	-0.15777126
3411	0.43750000	1.06250000	-0.17165200
3412	0.31250000	0.93750000	-0.15073314
3413	0.37500000	1.06250000	-0.16461388
3414	0.56250000	0.93750000	-0.16891496
3415	0.62500000	1.06250000	-0.18279570
3416	0.62500000	0.93750000	-0.17106549
3417	0.68750000	1.06250000	-0.18494624
3418	0.43750000	0.87500000	-0.15307918
3419	0.31250000	0.87500000	-0.14408602

3420	0.37500000	0.81250000	-0.13939394
3421	0.56250000	0.87500000	-0.15718475
3422	0.43750000	0.81250000	-0.14134897
3423	0.56250000	1.12500000	-0.18084066
3424	0.43750000	1.12500000	-0.17184751
3425	0.56250000	1.18750000	-0.18123167
3426	0.68750000	1.12500000	-0.18494624
3427	0.62500000	1.18750000	-0.18318671
3428	0.37500000	1.43750000	-0.16871945
3429	0.43750000	1.37500000	-0.17262952
3430	0.31250000	1.37500000	-0.16148583
3431	0.37500000	1.31250000	-0.16539589
3432	0.56250000	1.62500000	-0.18064516
3433	0.62500000	1.56250000	-0.18435973
3434	0.62500000	1.68750000	-0.18299120
3435	0.68750000	1.62500000	-0.18690127
3436	0.43750000	1.56250000	-0.17419355
3437	0.31250000	1.43750000	-0.16461388
3438	0.37500000	1.56250000	-0.17008798
3439	0.56250000	1.68750000	-0.17888563
3440	0.43750000	1.62500000	-0.17243402
3441	0.56250000	1.43750000	-0.18181818
3442	0.43750000	1.31250000	-0.17243402
3443	0.56250000	1.37500000	-0.18162268
3444	0.68750000	1.56250000	-0.18651026
3445	0.62500000	1.43750000	-0.18377322
3446	0.93750000	0.37500000	-0.14740958
3447	1.06250000	0.43750000	-0.16148583
3448	0.93750000	0.31250000	-0.13255132
3449	1.06250000	0.37500000	-0.14662757
3450	0.93750000	0.56250000	-0.16715543
3451	1.06250000	0.62500000	-0.18103617
3452	0.93750000	0.62500000	-0.16950147
3453	1.06250000	0.68750000	-0.18338221
3454	0.87500000	0.43750000	-0.15092864
3455	0.87500000	0.31250000	-0.13352884
3456	0.81250000	0.37500000	-0.13704790
3457	0.87500000	0.56250000	-0.15581623
3458	0.81250000	0.43750000	-0.13958944
3459	1.12500000	0.56250000	-0.17888563
3460	1.12500000	0.43750000	-0.16168133
3461	1.18750000	0.56250000	-0.17908113
3462	1.12500000	0.68750000	-0.18357771
3463	1.18750000	0.62500000	-0.18142717
3464	1.43750000	0.37500000	-0.15444770
3465	1.37500000	0.43750000	-0.16246334
3466	1.37500000	0.31250000	-0.13919844
3467	1.31250000	0.37500000	-0.14740958
3468	1.62500000	0.56250000	-0.18005865
3469	1.56250000	0.62500000	-0.18299120
3470	1.68750000	0.62500000	-0.18279570
3471	1.62500000	0.68750000	-0.18553275
3472	1.56250000	0.43750000	-0.16911046
3473	1.43750000	0.31250000	-0.14604106
3474	1.56250000	0.37500000	-0.16089932
3475	1.68750000	0.56250000	-0.17966764
3476	1.62500000	0.43750000	-0.16871945
3477	1.43750000	0.56250000	-0.18005865
3478	1.31250000	0.43750000	-0.16226784

3479	1.37500000	0.56250000	-0.17966764
3480	1.56250000	0.68750000	-0.18533724
3481	1.43750000	0.62500000	-0.18240469
3482	1.06250000	1.43750000	-0.19687195
3483	0.93750000	1.37500000	-0.19433040
3484	1.06250000	1.37500000	-0.19648094
3485	0.93750000	1.31250000	-0.19413490
3486	1.06250000	1.62500000	-0.19745846
3487	0.93750000	1.56250000	-0.19491691
3488	1.06250000	1.68750000	-0.19765396
3489	0.93750000	1.62500000	-0.19511241
3490	1.12500000	1.56250000	-0.19745846
3491	1.12500000	1.43750000	-0.19687195
3492	1.18750000	1.56250000	-0.19745846
3493	1.12500000	1.68750000	-0.19784946
3494	1.18750000	1.62500000	-0.19765396
3495	0.87500000	1.43750000	-0.19237537
3496	0.87500000	1.31250000	-0.19198436
3497	0.81250000	1.37500000	-0.19002933
3498	0.87500000	1.56250000	-0.19276637
3499	0.81250000	1.43750000	-0.19022483
3500	1.43750000	1.06250000	-0.19687195
3501	1.37500000	0.93750000	-0.19413490
3502	1.37500000	1.06250000	-0.19648094
3503	1.31250000	0.93750000	-0.19393939
3504	1.62500000	1.06250000	-0.19765396
3505	1.56250000	0.93750000	-0.19491691
3506	1.68750000	1.06250000	-0.19784946
3507	1.62500000	0.93750000	-0.19530792
3508	1.56250000	1.12500000	-0.19745846
3509	1.43750000	1.12500000	-0.19687195
3510	1.56250000	1.18750000	-0.19745846
3511	1.68750000	1.12500000	-0.19784946
3512	1.62500000	1.18750000	-0.19765396
3513	1.43750000	0.87500000	-0.19198436
3514	1.31250000	0.87500000	-0.19159335
3515	1.37500000	0.81250000	-0.18944282
3516	1.56250000	0.87500000	-0.19257087
3517	1.43750000	0.81250000	-0.18963832
3518	0.37500000	3.43750000	0.07976540
3519	0.43750000	3.37500000	0.06314761
3520	0.31250000	3.37500000	0.06940371
3521	0.37500000	3.31250000	0.05845552
3522	0.56250000	3.62500000	0.12727273
3523	0.62500000	3.56250000	0.11085044
3524	0.62500000	3.68750000	0.15151515
3525	0.68750000	3.62500000	0.13528837
3526	0.43750000	3.56250000	0.10322581
3527	0.31250000	3.43750000	0.08132942
3528	0.37500000	3.56250000	0.10322581
3529	0.56250000	3.68750000	0.13900293
3530	0.43750000	3.62500000	0.11476051
3531	0.56250000	3.43750000	0.07018573
3532	0.43750000	3.31250000	0.05376344
3533	0.56250000	3.37500000	0.05395894
3534	0.68750000	3.56250000	0.10635386
3535	0.62500000	3.43750000	0.06568915
3536	0.37500000	2.93750000	-0.03890518
3537	0.43750000	3.06250000	-0.01915934

3538	0.31250000	2.93750000	-0.02052786
3539	0.37500000	3.06250000	-0.01446725
3540	0.56250000	2.93750000	-0.05767351
3541	0.62500000	3.06250000	-0.03323558
3542	0.62500000	2.93750000	-0.06236559
3543	0.68750000	3.06250000	-0.03792766
3544	0.43750000	2.87500000	-0.05610948
3545	0.31250000	2.87500000	-0.02834800
3546	0.37500000	2.81250000	-0.05923754
3547	0.56250000	2.87500000	-0.07018573
3548	0.43750000	2.81250000	-0.06862170
3549	0.56250000	3.12500000	-0.01192571
3550	0.43750000	3.12500000	-0.00254154
3551	0.56250000	3.18750000	0.00449658
3552	0.68750000	3.12500000	-0.02130987
3553	0.62500000	3.18750000	-0.00019550
3554	1.06250000	3.62500000	0.08856305
3555	0.93750000	3.56250000	0.07546432
3556	1.06250000	3.68750000	0.10518084
3557	0.93750000	3.62500000	0.09188661
3558	1.06250000	3.43750000	0.03264907
3559	0.93750000	3.37500000	0.02580645
3560	1.06250000	3.37500000	0.01622678
3561	0.93750000	3.31250000	0.00938416
3562	1.12500000	3.56250000	0.06686217
3563	1.12500000	3.68750000	0.10635386
3564	1.18750000	3.62500000	0.08465298
3565	1.12500000	3.43750000	0.02756598
3566	1.18750000	3.56250000	0.06177908
3567	0.87500000	3.43750000	0.04692082
3568	0.87500000	3.56250000	0.07996090
3569	0.81250000	3.43750000	0.05161290
3570	0.87500000	3.31250000	0.01388074
3571	0.81250000	3.37500000	0.03499511
3572	3.43750000	0.37500000	0.11221896
3573	3.37500000	0.43750000	0.09345064
3574	3.37500000	0.31250000	0.10420332
3575	3.31250000	0.37500000	0.08563050
3576	3.62500000	0.56250000	0.13626588
3577	3.56250000	0.62500000	0.11769306
3578	3.68750000	0.62500000	0.14428152
3579	3.62500000	0.68750000	0.12570870
3580	3.56250000	0.43750000	0.12942326
3581	3.43750000	0.31250000	0.11339198
3582	3.56250000	0.37500000	0.13040078
3583	3.68750000	0.56250000	0.14545455
3584	3.62500000	0.43750000	0.13841642
3585	3.43750000	0.56250000	0.09208211
3586	3.31250000	0.43750000	0.07605083
3587	3.37500000	0.56250000	0.07448680
3588	3.56250000	0.68750000	0.10830890
3589	3.43750000	0.62500000	0.08269795
3590	2.93750000	0.37500000	-0.01290323
3591	3.06250000	0.43750000	0.00586510
3592	2.93750000	0.31250000	-0.00351906
3593	3.06250000	0.37500000	0.01524927
3594	2.93750000	0.56250000	-0.04164223
3595	3.06250000	0.62500000	-0.02248289
3596	2.93750000	0.62500000	-0.05122190

3597	3.06250000	0.68750000	-0.03206256
3598	2.87500000	0.43750000	-0.03343109
3599	2.87500000	0.31250000	-0.01427175
3600	2.81250000	0.37500000	-0.03479961
3601	2.87500000	0.56250000	-0.05278592
3602	2.81250000	0.43750000	-0.04457478
3603	3.12500000	0.56250000	0.00449658
3604	3.12500000	0.43750000	0.02326491
3605	3.18750000	0.56250000	0.02189638
3606	3.12500000	0.68750000	-0.01446725
3607	3.18750000	0.62500000	0.01251222
3608	3.62500000	1.06250000	0.06959922
3609	3.56250000	0.93750000	0.07018573
3610	3.68750000	1.06250000	0.08699902
3611	3.62500000	0.93750000	0.08758553
3612	3.43750000	1.06250000	0.02482893
3613	3.37500000	0.93750000	0.01779081
3614	3.37500000	1.06250000	0.00742913
3615	3.31250000	0.93750000	0.00039101
3616	3.56250000	1.12500000	0.05122190
3617	3.68750000	1.12500000	0.07859238
3618	3.62500000	1.18750000	0.06021505
3619	3.43750000	1.12500000	0.02385142
3620	3.56250000	1.18750000	0.05024438
3621	3.43750000	0.87500000	0.04477028
3622	3.56250000	0.87500000	0.07976540
3623	3.43750000	0.81250000	0.05434995
3624	3.31250000	0.87500000	0.00977517
3625	3.37500000	0.81250000	0.03675464
3626	3.06250000	3.43750000	0.14916911
3627	2.93750000	3.37500000	0.11945259
3628	3.06250000	3.37500000	0.14291300
3629	2.93750000	3.31250000	0.11319648
3630	3.06250000	3.62500000	0.12218964
3631	2.93750000	3.56250000	0.14956012
3632	3.06250000	3.68750000	0.12844575
3633	2.93750000	3.62500000	0.15581623
3634	3.12500000	3.56250000	0.12160313
3635	3.12500000	3.43750000	0.15464321
3636	3.18750000	3.56250000	0.12727273
3637	3.12500000	3.68750000	0.08856305
3638	3.18750000	3.62500000	0.08797654
3639	2.87500000	3.43750000	0.11925709
3640	2.87500000	3.31250000	0.09521017
3641	2.81250000	3.37500000	0.09501466
3642	2.87500000	3.56250000	0.14330401
3643	2.81250000	3.43750000	0.11280547
3644	3.43750000	3.06250000	0.14017595
3645	3.37500000	2.93750000	0.11906158
3646	3.37500000	3.06250000	0.13548387
3647	3.31250000	2.93750000	0.11436950
3648	3.62500000	3.06250000	0.14721408
3649	3.56250000	2.93750000	0.13020528
3650	3.68750000	3.06250000	0.15190616
3651	3.62500000	2.93750000	0.13489736
3652	3.56250000	3.12500000	0.14956012
3653	3.43750000	3.12500000	0.14721408
3654	3.56250000	3.18750000	0.15659824
3655	3.68750000	3.12500000	0.15190616

3656	3.62500000	3.18750000	0.15425220
3657	3.43750000	2.87500000	0.11143695
3658	3.31250000	2.87500000	0.10498534
3659	3.37500000	2.81250000	0.09736070
3660	3.56250000	2.87500000	0.11788856
3661	3.43750000	2.81250000	0.09912023
3662	2.56250000	2.93750000	-0.03284457
3663	2.62500000	3.06250000	-0.00175953
3664	2.62500000	2.93750000	-0.01466276
3665	2.68750000	3.06250000	0.01622678
3666	2.37500000	2.93750000	-0.07429130
3667	2.43750000	3.06250000	-0.03968719
3668	2.31250000	2.93750000	-0.08797654
3669	2.37500000	3.06250000	-0.05337243
3670	2.43750000	2.87500000	-0.06744868
3671	2.56250000	2.87500000	-0.03949169
3672	2.43750000	2.81250000	-0.07409580
3673	2.31250000	2.87500000	-0.09090909
3674	2.37500000	2.81250000	-0.08387097
3675	2.56250000	3.12500000	-0.00175953
3676	2.68750000	3.12500000	0.02248289
3677	2.62500000	3.18750000	0.02248289
3678	2.43750000	3.12500000	-0.02170088
3679	2.56250000	3.18750000	0.01622678
3680	2.93750000	2.56250000	0.01915934
3681	3.06250000	2.62500000	0.04868035
3682	2.93750000	2.62500000	0.02854350
3683	3.06250000	2.68750000	0.05806452
3684	2.93750000	2.37500000	-0.01818182
3685	3.06250000	2.43750000	0.01153470
3686	2.93750000	2.31250000	-0.03382209
3687	3.06250000	2.37500000	-0.00410557
3688	2.87500000	2.43750000	-0.01818182
3689	2.87500000	2.56250000	0.00371457
3690	2.81250000	2.43750000	-0.03362659
3691	2.87500000	2.31250000	-0.04633431
3692	2.81250000	2.37500000	-0.04613881
3693	3.12500000	2.56250000	0.04105572
3694	3.12500000	2.68750000	0.06275660
3695	3.18750000	2.62500000	0.05513196
3696	3.12500000	2.43750000	0.01309873
3697	3.18750000	2.56250000	0.04281525
3698	2.56250000	3.62500000	0.11925709
3699	2.62500000	3.56250000	0.11827957
3700	2.62500000	3.68750000	0.13176931
3701	2.68750000	3.62500000	0.13079179
3702	2.37500000	3.43750000	0.05552297
3703	2.43750000	3.37500000	0.04985337
3704	2.31250000	3.37500000	0.02385142
3705	2.37500000	3.31250000	0.01818182
3706	2.43750000	3.56250000	0.09442815
3707	2.56250000	3.68750000	0.12649071
3708	2.43750000	3.62500000	0.10146628
3709	2.31250000	3.43750000	0.04320626
3710	2.37500000	3.56250000	0.08191593
3711	2.56250000	3.43750000	0.08778104
3712	2.68750000	3.56250000	0.12453568
3713	2.62500000	3.43750000	0.09403715
3714	2.43750000	3.31250000	0.03186706

3715	2.56250000	3.37500000	0.06979472
3716	3.62500000	2.56250000	0.07370479
3717	3.56250000	2.62500000	0.06783969
3718	3.68750000	2.62500000	0.09090909
3719	3.62500000	2.68750000	0.08504399
3720	3.43750000	2.37500000	0.01720430
3721	3.37500000	2.43750000	0.01935484
3722	3.37500000	2.31250000	0.00000000
3723	3.31250000	2.37500000	0.00215054
3724	3.56250000	2.43750000	0.05278592
3725	3.68750000	2.56250000	0.09208211
3726	3.62500000	2.43750000	0.07116325
3727	3.43750000	2.31250000	0.01348974
3728	3.56250000	2.37500000	0.04907136
3729	3.43750000	2.56250000	0.04907136
3730	3.56250000	2.68750000	0.08035191
3731	3.43750000	2.62500000	0.06158358
3732	3.31250000	2.43750000	0.01779081
3733	3.37500000	2.56250000	0.04750733
3734	0.93750000	2.37500000	-0.14897361
3735	1.06250000	2.43750000	-0.15014663
3736	0.93750000	2.31250000	-0.15659824
3737	1.06250000	2.37500000	-0.15777126
3738	0.93750000	2.56250000	-0.13059629
3739	1.06250000	2.62500000	-0.12746823
3740	0.93750000	2.62500000	-0.12297165
3741	1.06250000	2.68750000	-0.11984360
3742	0.87500000	2.43750000	-0.14173998
3743	0.87500000	2.31250000	-0.15249267
3744	0.81250000	2.37500000	-0.14525904
3745	0.87500000	2.56250000	-0.13098729
3746	0.81250000	2.43750000	-0.14213099
3747	1.12500000	2.56250000	-0.13978495
3748	1.12500000	2.43750000	-0.15483871
3749	1.18750000	2.56250000	-0.14447703
3750	1.12500000	2.68750000	-0.12473118
3751	1.18750000	2.62500000	-0.13704790
3752	1.43750000	2.37500000	-0.18162268
3753	1.37500000	2.43750000	-0.17399804
3754	1.37500000	2.31250000	-0.18455523
3755	1.31250000	2.37500000	-0.17693060
3756	1.62500000	2.56250000	-0.17321603
3757	1.56250000	2.62500000	-0.16559140
3758	1.68750000	2.62500000	-0.17047898
3759	1.62500000	2.68750000	-0.16285435
3760	1.56250000	2.43750000	-0.17888563
3761	1.43750000	2.31250000	-0.18455523
3762	1.56250000	2.37500000	-0.18181818
3763	1.68750000	2.56250000	-0.17341153
3764	1.62500000	2.43750000	-0.17908113
3765	1.43750000	2.56250000	-0.16363636
3766	1.31250000	2.43750000	-0.16930596
3767	1.37500000	2.56250000	-0.15894428
3768	1.56250000	2.68750000	-0.15816227
3769	1.43750000	2.62500000	-0.15620723
3770	1.43750000	3.06250000	-0.09481916
3771	1.37500000	2.93750000	-0.11378299
3772	1.37500000	3.06250000	-0.09012708
3773	1.31250000	2.93750000	-0.10909091

3774	1.62500000	3.06250000	-0.10615836
3775	1.56250000	2.93750000	-0.12805474
3776	1.68750000	3.06250000	-0.11085044
3777	1.62500000	2.93750000	-0.13274682
3778	1.56250000	3.12500000	-0.08523949
3779	1.43750000	3.12500000	-0.07878788
3780	1.56250000	3.18750000	-0.06901271
3781	1.68750000	3.12500000	-0.09169110
3782	1.62500000	3.18750000	-0.07077224
3783	1.43750000	2.87500000	-0.12609971
3784	1.31250000	2.87500000	-0.11671554
3785	1.37500000	2.81250000	-0.12883675
3786	1.56250000	2.87500000	-0.13548387
3787	1.43750000	2.81250000	-0.13352884
3788	2.37500000	0.93750000	-0.15757576
3789	2.43750000	1.06250000	-0.15425220
3790	2.31250000	0.93750000	-0.17634409
3791	2.37500000	1.06250000	-0.16070381
3792	2.56250000	0.93750000	-0.13685239
3793	2.62500000	1.06250000	-0.13548387
3794	2.62500000	0.93750000	-0.13040078
3795	2.68750000	1.06250000	-0.12903226
3796	2.43750000	0.87500000	-0.14643206
3797	2.31250000	0.87500000	-0.17341153
3798	2.37500000	0.81250000	-0.14956012
3799	2.56250000	0.87500000	-0.13176931
3800	2.43750000	0.81250000	-0.14134897
3801	2.56250000	1.12500000	-0.14173998
3802	2.43750000	1.12500000	-0.15444770
3803	2.56250000	1.18750000	-0.14173998
3804	2.68750000	1.12500000	-0.12903226
3805	2.62500000	1.18750000	-0.13548387
3806	2.37500000	1.43750000	-0.16168133
3807	2.43750000	1.37500000	-0.15522972
3808	2.31250000	1.37500000	-0.16793744
3809	2.37500000	1.31250000	-0.16148583
3810	2.56250000	1.62500000	-0.14916911
3811	2.62500000	1.56250000	-0.13646139
3812	2.62500000	1.68750000	-0.14291300
3813	2.68750000	1.62500000	-0.13020528
3814	2.43750000	1.56250000	-0.16168133
3815	2.31250000	1.43750000	-0.16793744
3816	2.37500000	1.56250000	-0.16793744
3817	2.56250000	1.68750000	-0.15542522
3818	2.43750000	1.62500000	-0.16793744
3819	2.56250000	1.43750000	-0.14252199
3820	2.43750000	1.31250000	-0.15503421
3821	2.56250000	1.37500000	-0.14232649
3822	2.68750000	1.56250000	-0.13000978
3823	2.62500000	1.43750000	-0.13607038
3824	3.06250000	1.43750000	-0.09990225
3825	2.93750000	1.37500000	-0.12277615
3826	3.06250000	1.37500000	-0.11847507
3827	2.93750000	1.31250000	-0.14134897
3828	3.06250000	1.62500000	-0.08484848
3829	2.93750000	1.56250000	-0.10459433
3830	3.06250000	1.68750000	-0.08504399
3831	2.93750000	1.62500000	-0.10478983
3832	3.12500000	1.56250000	-0.06803519

3833	3.12500000	1.43750000	-0.08328446
3834	3.18750000	1.56250000	-0.05141740
3835	3.12500000	1.68750000	-0.07155425
3836	3.18750000	1.62500000	-0.05474096
3837	2.87500000	1.43750000	-0.11065494
3838	2.87500000	1.31250000	-0.12903226
3839	2.81250000	1.37500000	-0.11691105
3840	2.87500000	1.56250000	-0.11104594
3841	2.81250000	1.43750000	-0.11710655
3842	0.12500000	0.31250000	-0.03284457
3843	0.18750000	0.31250000	-0.03479961
3844	0.18750000	0.37500000	-0.04828935
3845	0.31250000	0.12500000	-0.03147605
3846	0.31250000	0.18750000	-0.03401760
3847	0.37500000	0.18750000	-0.04692082
3848	0.12500000	3.81250000	0.15092864
3849	0.18750000	3.87500000	0.16246334
3850	0.18750000	3.81250000	0.15053763
3851	3.81250000	0.12500000	0.17067449
3852	3.87500000	0.18750000	0.17869013
3853	3.81250000	0.18750000	0.16969697
3854	3.68750000	3.81250000	0.10889541
3855	3.68750000	3.87500000	0.06959922
3856	3.81250000	3.68750000	0.18240469
3857	3.87500000	3.68750000	0.18005865
3858	3.62500000	3.81250000	0.07194526
3859	3.81250000	3.62500000	0.18240469
3860	0.12500000	1.81250000	-0.14643206
3861	0.18750000	1.87500000	-0.14877810
3862	0.18750000	1.81250000	-0.15073314
3863	0.12500000	2.31250000	-0.05650049
3864	0.18750000	2.31250000	-0.08778104
3865	0.18750000	2.37500000	-0.08230694
3866	0.31250000	2.12500000	-0.14428152
3867	0.31250000	2.18750000	-0.13802542
3868	0.37500000	2.18750000	-0.14525904
3869	1.81250000	0.12500000	-0.02932551
3870	1.87500000	0.18750000	-0.08699902
3871	1.81250000	0.18750000	-0.08641251
3872	2.31250000	0.12500000	-0.07526882
3873	2.31250000	0.18750000	-0.08895406
3874	2.37500000	0.18750000	-0.07800587
3875	2.12500000	0.31250000	-0.13626588
3876	2.18750000	0.31250000	-0.12825024
3877	2.18750000	0.37500000	-0.13528837
3878	1.68750000	1.81250000	-0.19863148
3879	1.68750000	1.87500000	-0.19882698
3880	1.81250000	1.68750000	-0.19863148
3881	1.87500000	1.68750000	-0.19882698
3882	1.62500000	1.81250000	-0.19863148
3883	1.81250000	1.62500000	-0.19863148
3884	2.18750000	2.31250000	-0.15601173
3885	2.12500000	2.31250000	-0.16578690
3886	2.31250000	2.18750000	-0.16148583
3887	2.31250000	2.12500000	-0.17419355
3888	2.18750000	2.37500000	-0.15307918
3889	2.37500000	2.18750000	-0.16148583
3890	1.81250000	2.12500000	-0.19354839
3891	1.87500000	2.18750000	-0.19100684

3892	1.81250000	2.18750000	-0.19081134
3893	2.12500000	1.81250000	-0.19921799
3894	2.18750000	1.87500000	-0.19921799
3895	2.18750000	1.81250000	-0.19921799
3896	1.68750000	3.87500000	0.12043011
3897	1.68750000	3.81250000	0.09755621
3898	1.62500000	3.81250000	0.10752688
3899	2.18750000	3.87500000	0.10224829
3900	2.12500000	3.81250000	0.08269795
3901	2.18750000	3.81250000	0.08778104
3902	1.81250000	3.68750000	0.04848485
3903	1.87500000	3.68750000	0.04672532
3904	1.81250000	3.62500000	0.03401760
3905	3.87500000	1.68750000	0.10478983
3906	3.81250000	1.68750000	0.09481916
3907	3.81250000	1.62500000	0.09149560
3908	3.87500000	2.18750000	0.12453568
3909	3.81250000	2.12500000	0.10244379
3910	3.81250000	2.18750000	0.10791789
3911	3.68750000	1.81250000	0.06842620
3912	3.68750000	1.87500000	0.06490714
3913	3.62500000	1.81250000	0.05180841
3914	0.12500000	0.81250000	-0.11632454
3915	0.18750000	0.87500000	-0.13000978
3916	0.18750000	0.81250000	-0.12336266
3917	0.12500000	1.31250000	-0.14623656
3918	0.18750000	1.31250000	-0.15034213
3919	0.18750000	1.37500000	-0.15327468
3920	0.31250000	1.12500000	-0.15777126
3921	0.31250000	1.18750000	-0.15796676
3922	0.37500000	1.18750000	-0.16500489
3923	0.81250000	0.12500000	-0.08993157
3924	0.87500000	0.18750000	-0.10381232
3925	0.81250000	0.18750000	-0.10478983
3926	1.31250000	0.12500000	-0.10752688
3927	1.31250000	0.18750000	-0.11573803
3928	1.37500000	0.18750000	-0.12258065
3929	1.12500000	0.31250000	-0.13196481
3930	1.18750000	0.31250000	-0.13216031
3931	1.18750000	0.37500000	-0.14701857
3932	0.68750000	0.81250000	-0.14995112
3933	0.68750000	0.87500000	-0.16148583
3934	0.81250000	0.68750000	-0.14916911
3935	0.87500000	0.68750000	-0.16050831
3936	0.62500000	0.81250000	-0.14780059
3937	0.81250000	0.62500000	-0.14682307
3938	1.18750000	1.31250000	-0.19648094
3939	1.12500000	1.31250000	-0.19628543
3940	1.31250000	1.18750000	-0.19648094
3941	1.31250000	1.12500000	-0.19628543
3942	1.18750000	1.37500000	-0.19667644
3943	1.37500000	1.18750000	-0.19667644
3944	0.81250000	1.12500000	-0.18924731
3945	0.87500000	1.18750000	-0.19159335
3946	0.81250000	1.18750000	-0.18944282
3947	1.12500000	0.81250000	-0.18846530
3948	1.18750000	0.87500000	-0.19120235
3949	1.18750000	0.81250000	-0.18866080
3950	0.12500000	3.31250000	0.05630499

3951	0.18750000	3.31250000	0.05767351
3952	0.18750000	3.37500000	0.06959922
3953	0.12500000	2.81250000	-0.08074291
3954	0.18750000	2.87500000	-0.01857283
3955	0.18750000	2.81250000	-0.07214076
3956	0.31250000	3.18750000	0.07937439
3957	0.31250000	3.12500000	0.05591398
3958	0.37500000	3.18750000	0.02561095
3959	0.68750000	3.87500000	0.17438905
3960	0.68750000	3.81250000	0.17517107
3961	0.62500000	3.81250000	0.16246334
3962	1.18750000	3.87500000	0.15718475
3963	1.12500000	3.81250000	0.13939394
3964	1.18750000	3.81250000	0.14076246
3965	0.81250000	3.68750000	0.14271750
3966	0.87500000	3.68750000	0.12551320
3967	0.81250000	3.62500000	0.11358749
3968	3.31250000	0.12500000	0.09814272
3969	3.31250000	0.18750000	0.09716520
3970	3.37500000	0.18750000	0.10635386
3971	2.81250000	0.12500000	0.00332356
3972	2.87500000	0.18750000	0.00469208
3973	2.81250000	0.18750000	-0.00625611
3974	3.18750000	0.31250000	0.06001955
3975	3.12500000	0.31250000	0.04222874
3976	3.18750000	0.37500000	0.05043988
3977	3.87500000	0.68750000	0.17047898
3978	3.81250000	0.68750000	0.16148583
3979	3.81250000	0.62500000	0.16246334
3980	3.87500000	1.18750000	0.12258065
3981	3.81250000	1.12500000	0.11358749
3982	3.81250000	1.18750000	0.10498534
3983	3.68750000	0.81250000	0.12414467
3984	3.68750000	0.87500000	0.11456500
3985	3.62500000	0.81250000	0.10674487
3986	3.18750000	3.31250000	0.14760508
3987	3.12500000	3.31250000	0.14213099
3988	3.31250000	3.18750000	0.14486804
3989	3.31250000	3.12500000	0.13782991
3990	3.18750000	3.37500000	0.15386119
3991	3.37500000	3.18750000	0.14956012
3992	2.68750000	2.81250000	-0.01016618
3993	2.68750000	2.87500000	-0.00332356
3994	2.81250000	2.68750000	0.00703812
3995	2.87500000	2.68750000	0.02248289
3996	2.62500000	2.81250000	-0.02815249
3997	2.81250000	2.62500000	-0.00234604
3998	2.87500000	3.18750000	0.08269795
3999	2.81250000	3.12500000	0.05845552
4000	2.81250000	3.18750000	0.06471163
4001	3.18750000	2.87500000	0.09560117
4002	3.12500000	2.81250000	0.08152493
4003	3.18750000	2.81250000	0.08621701
4004	3.18750000	3.87500000	0.06744868
4005	3.12500000	3.81250000	0.10107527
4006	3.18750000	3.81250000	0.06119257
4007	2.68750000	3.87500000	0.15855327
4008	2.68750000	3.81250000	0.15151515
4009	2.62500000	3.81250000	0.14604106

4010	2.87500000	3.68750000	0.15581623
4011	2.81250000	3.68750000	0.14956012
4012	2.81250000	3.62500000	0.14330401
4013	3.87500000	3.18750000	0.16598240
4014	3.81250000	3.12500000	0.16129032
4015	3.81250000	3.18750000	0.16129032
4016	3.87500000	2.68750000	0.14467253
4017	3.81250000	2.68750000	0.12649071
4018	3.81250000	2.62500000	0.12746823
4019	3.68750000	2.87500000	0.12727273
4020	3.68750000	2.81250000	0.11476051
4021	3.62500000	2.81250000	0.11006843
4022	0.68750000	1.87500000	-0.18181818
4023	0.68750000	1.81250000	-0.18357771
4024	0.81250000	2.12500000	-0.17126100
4025	0.87500000	2.18750000	-0.16774194
4026	0.62500000	1.81250000	-0.17947214
4027	0.81250000	2.18750000	-0.16363636
4028	1.18750000	1.87500000	-0.19824047
4029	1.12500000	1.81250000	-0.19804497
4030	1.31250000	2.12500000	-0.19335288
4031	1.31250000	2.18750000	-0.19042033
4032	1.18750000	1.81250000	-0.19824047
4033	1.37500000	2.18750000	-0.19042033
4034	0.81250000	1.68750000	-0.19120235
4035	0.87500000	1.68750000	-0.19315738
4036	0.81250000	1.62500000	-0.19100684
4037	1.12500000	2.31250000	-0.17008798
4038	1.18750000	2.31250000	-0.17497556
4039	1.18750000	2.37500000	-0.16735093
4040	0.68750000	2.81250000	-0.09188661
4041	0.68750000	2.87500000	-0.07956989
4042	0.81250000	2.68750000	-0.11612903
4043	0.87500000	2.68750000	-0.11573803
4044	0.62500000	2.81250000	-0.08719453
4045	0.81250000	2.62500000	-0.12375367
4046	1.18750000	3.31250000	-0.01055718
4047	1.12500000	3.31250000	-0.00547410
4048	1.31250000	3.18750000	-0.05298143
4049	1.31250000	3.12500000	-0.06920821
4050	1.18750000	3.37500000	0.00606061
4051	1.37500000	3.18750000	-0.05786901
4052	0.81250000	3.12500000	-0.03069404
4053	0.87500000	3.18750000	-0.01896383
4054	0.81250000	3.18750000	-0.01427175
4055	1.12500000	2.81250000	-0.10967742
4056	1.18750000	2.87500000	-0.10713587
4057	1.18750000	2.81250000	-0.11456500
4058	1.87500000	0.68750000	-0.18435973
4059	1.81250000	0.68750000	-0.18494624
4060	2.12500000	0.81250000	-0.17693060
4061	2.18750000	0.87500000	-0.18611926
4062	1.81250000	0.62500000	-0.18181818
4063	2.18750000	0.81250000	-0.18299120
4064	1.87500000	1.18750000	-0.19843597
4065	1.81250000	1.12500000	-0.19824047
4066	2.12500000	1.31250000	-0.18670577
4067	2.18750000	1.31250000	-0.18044966
4068	1.81250000	1.18750000	-0.19824047

4069	2.18750000	1.37500000	-0.18044966
4070	1.68750000	0.81250000	-0.19061584
4071	1.68750000	0.87500000	-0.19315738
4072	1.62500000	0.81250000	-0.19042033
4073	2.31250000	1.12500000	-0.16735093
4074	2.31250000	1.18750000	-0.16754643
4075	2.37500000	1.18750000	-0.16109482
4076	2.81250000	0.68750000	-0.10752688
4077	2.87500000	0.68750000	-0.08426197
4078	2.68750000	0.81250000	-0.13919844
4079	2.68750000	0.87500000	-0.13157380
4080	2.81250000	0.62500000	-0.08563050
4081	2.62500000	0.81250000	-0.13294233
4082	3.31250000	1.18750000	-0.01231672
4083	3.31250000	1.12500000	-0.01114370
4084	3.18750000	1.31250000	-0.06627566
4085	3.12500000	1.31250000	-0.10166178
4086	3.37500000	1.18750000	0.00527859
4087	3.18750000	1.37500000	-0.06647116
4088	3.12500000	0.81250000	-0.03343109
4089	3.18750000	0.87500000	-0.02521994
4090	3.18750000	0.81250000	-0.01583578
4091	2.81250000	1.12500000	-0.13489736
4092	2.87500000	1.18750000	-0.14740958
4093	2.81250000	1.18750000	-0.13509286
4094	2.12500000	2.81250000	-0.13489736
4095	2.18750000	2.87500000	-0.11827957
4096	1.87500000	2.68750000	-0.16813294
4097	1.81250000	2.68750000	-0.16793744
4098	2.18750000	2.81250000	-0.12121212
4099	1.81250000	2.62500000	-0.17086999
4100	2.12500000	3.31250000	-0.00762463
4101	2.18750000	3.31250000	-0.00762463
4102	1.87500000	3.18750000	-0.06158358
4103	1.81250000	3.12500000	-0.08856305
4104	2.18750000	3.37500000	0.01153470
4105	1.81250000	3.18750000	-0.06940371
4106	2.31250000	3.12500000	-0.04907136
4107	2.31250000	3.18750000	-0.03128055
4108	2.37500000	3.18750000	-0.01759531
4109	1.68750000	2.81250000	-0.15268817
4110	1.68750000	2.87500000	-0.14506354
4111	1.62500000	2.81250000	-0.14780059
4112	2.81250000	2.12500000	-0.10576735
4113	2.87500000	2.18750000	-0.07761486
4114	2.68750000	1.87500000	-0.14916911
4115	2.68750000	1.81250000	-0.14291300
4116	2.81250000	2.18750000	-0.09012708
4117	2.62500000	1.81250000	-0.15542522
4118	3.31250000	2.12500000	-0.02424242
4119	3.31250000	2.18750000	-0.02072336
4120	3.18750000	1.87500000	-0.05826002
4121	3.12500000	1.81250000	-0.07174976
4122	3.37500000	2.18750000	-0.00723363
4123	3.18750000	1.81250000	-0.05826002
4124	3.12500000	2.31250000	-0.01818182
4125	3.18750000	2.31250000	-0.01661779
4126	3.18750000	2.37500000	-0.00097752
4127	2.81250000	1.68750000	-0.11749756

4128	2.87500000	1.68750000	-0.11124145
4129	2.81250000	1.62500000	-0.11749756
4130	0.31250000	0.62500000	-0.10244379
4131	0.31250000	0.68750000	-0.11397849
4132	0.37500000	0.68750000	-0.11612903
4133	0.62500000	0.31250000	-0.10048876
4134	0.68750000	0.31250000	-0.11182796
4135	0.68750000	0.37500000	-0.11436950
4136	0.31250000	3.68750000	0.12668622
4137	0.31250000	3.62500000	0.11495601
4138	0.37500000	3.68750000	0.12649071
4139	3.68750000	0.31250000	0.14936461
4140	3.62500000	0.31250000	0.14037146
4141	3.68750000	0.37500000	0.14838710
4142	3.37500000	3.68750000	0.06529814
4143	3.31250000	3.62500000	0.09912023
4144	3.31250000	3.68750000	0.05982405
4145	3.68750000	3.37500000	0.17302053
4146	3.62500000	3.31250000	0.16832845
4147	3.68750000	3.31250000	0.16598240
4148	0.31250000	1.68750000	-0.16246334
4149	0.31250000	1.62500000	-0.16422287
4150	0.37500000	1.68750000	-0.16656891
4151	0.31250000	2.62500000	-0.08739003
4152	0.31250000	2.68750000	-0.07487781
4153	0.37500000	2.68750000	-0.08426197
4154	0.62500000	2.31250000	-0.14956012
4155	0.68750000	2.31250000	-0.14916911
4156	0.68750000	2.37500000	-0.14604106
4157	1.68750000	0.31250000	-0.15151515
4158	1.62500000	0.31250000	-0.15210166
4159	1.68750000	0.37500000	-0.15992180
4160	2.62500000	0.31250000	-0.05786901
4161	2.68750000	0.31250000	-0.04711632
4162	2.68750000	0.37500000	-0.05669599
4163	2.31250000	0.62500000	-0.14291300
4164	2.31250000	0.68750000	-0.14780059
4165	2.37500000	0.68750000	-0.13958944
4166	1.37500000	1.68750000	-0.19804497
4167	1.31250000	1.62500000	-0.19784946
4168	1.31250000	1.68750000	-0.19784946
4169	1.68750000	1.37500000	-0.19804497
4170	1.62500000	1.31250000	-0.19784946
4171	1.68750000	1.31250000	-0.19784946
4172	2.31250000	2.62500000	-0.11397849
4173	2.37500000	2.68750000	-0.09736070
4174	2.31250000	2.68750000	-0.10713587
4175	2.62500000	2.31250000	-0.10518084
4176	2.68750000	2.37500000	-0.07702835
4177	2.68750000	2.31250000	-0.08973607
4178	1.68750000	2.31250000	-0.18475073
4179	1.62500000	2.31250000	-0.18475073
4180	1.68750000	2.37500000	-0.18201369
4181	2.31250000	1.68750000	-0.18670577
4182	2.31250000	1.62500000	-0.18044966
4183	2.37500000	1.68750000	-0.18044966
4184	1.37500000	3.68750000	0.09227761
4185	1.31250000	3.68750000	0.09736070
4186	1.31250000	3.62500000	0.07448680

4187	2.31250000	3.68750000	0.08347996
4188	2.37500000	3.68750000	0.09599218
4189	2.31250000	3.62500000	0.07644184
4190	1.68750000	3.37500000	-0.02404692
4191	1.62500000	3.31250000	-0.03851417
4192	1.68750000	3.31250000	-0.04007820
4193	3.68750000	1.37500000	0.06705767
4194	3.68750000	1.31250000	0.06803519
4195	3.62500000	1.31250000	0.05826002
4196	3.68750000	2.31250000	0.08230694
4197	3.68750000	2.37500000	0.08582600
4198	3.62500000	2.31250000	0.06392962
4199	3.37500000	1.68750000	-0.00801564
4200	3.31250000	1.62500000	-0.02130987
4201	3.31250000	1.68750000	-0.02482893
4202	0.62500000	1.31250000	-0.18357771
4203	0.68750000	1.31250000	-0.18572825
4204	0.68750000	1.37500000	-0.18572825
4205	1.31250000	0.62500000	-0.18181818
4206	1.31250000	0.68750000	-0.18435973
4207	1.37500000	0.68750000	-0.18455523
4208	0.62500000	3.31250000	0.03284457
4209	0.68750000	3.37500000	0.04457478
4210	0.68750000	3.31250000	0.02795699
4211	3.31250000	0.62500000	0.04750733
4212	3.37500000	0.68750000	0.05571848
4213	3.31250000	0.68750000	0.03812317
4214	2.68750000	3.31250000	0.06451613
4215	2.68750000	3.37500000	0.08230694
4216	2.62500000	3.31250000	0.05826002
4217	3.31250000	2.68750000	0.07096774
4218	3.37500000	2.68750000	0.07253177
4219	3.31250000	2.62500000	0.05845552
4220	1.31250000	2.62500000	-0.14662757
4221	1.31250000	2.68750000	-0.13900293
4222	1.37500000	2.68750000	-0.14389052
4223	2.62500000	1.31250000	-0.13567937
4224	2.68750000	1.31250000	-0.12942326
4225	2.68750000	1.37500000	-0.12961877
4226	1.46875000	0.00000000	-0.06402737
4227	1.78125000	0.03125000	0.05689150
4228	1.96875000	0.21875000	-0.11671554
4229	1.28125000	0.00000000	-0.04379277
4230	1.90625000	0.03125000	-0.04477028
4231	1.96875000	0.09375000	-0.10263930
4232	0.15625000	3.03125000	0.01818182
4233	1.40625000	0.00000000	0.03040078
4234	1.34375000	0.00000000	0.03714565
4235	1.40625000	0.03125000	0.02629521
4236	1.37500000	0.03125000	0.02961877
4237	1.46875000	0.09375000	-0.07634409
4238	1.84375000	0.09375000	-0.00097752
4239	1.90625000	0.15625000	-0.05884653
4240	1.78125000	0.15625000	-0.05757576
4241	1.84375000	0.21875000	-0.11524927
4242	1.37500000	0.09375000	-0.06637341
4243	0.15625000	2.78125000	-0.10322581
4244	0.21875000	3.09375000	0.07917889
4245	0.21875000	2.84375000	-0.04105572

4246	1.96875000	0.03125000	-0.09560117
4247	1.46875000	0.03125000	-0.06813294
4248	1.46875000	0.06250000	-0.07223851
4249	1.84375000	0.03125000	0.00606061
4250	1.78125000	0.09375000	-0.00039101
4251	1.96875000	0.15625000	-0.10967742
4252	1.90625000	0.21875000	-0.11593353
4253	1.31250000	0.03125000	-0.05131965
4254	1.34375000	0.06250000	-0.05884653
4255	0.21875000	3.03125000	0.01348974
4256	0.21875000	2.78125000	-0.06256109
4257	1.78125000	0.21875000	-0.11466276
4258	1.90625000	0.09375000	-0.05180841
4259	0.15625000	3.09375000	0.03460411
4260	1.43750000	0.09375000	-0.07302053
4261	1.40625000	0.09375000	-0.06969697
4262	1.84375000	0.15625000	-0.05816227
4263	0.15625000	2.84375000	-0.04965787

End of Node Coordinates

Connectivity Matrix of Nodes

Triangular Index	Node 1	Node 2	Node 3
1	1	1091	1092
2	291	2692	1091
3	292	1092	2692
4	1091	2692	1092
5	83	1512	1510
6	708	2693	1512
7	291	1510	2693
8	1512	2693	1510
9	84	1514	1517
10	292	2695	1514
11	708	1517	2695
12	1514	2695	1517
13	291	2693	2692
14	708	2695	2693
15	292	2692	2695
16	2693	2695	2692
17	27	1208	1206
18	408	2987	1208
19	406	1206	2987
20	1208	2987	1206
21	196	2128	2126
22	709	2992	2128
23	408	2126	2992
24	2128	2992	2126
25	83	1511	1513
26	406	2988	1511
27	709	1513	2988
28	1511	2988	1513
29	408	2992	2987
30	709	2988	2992
31	406	2987	2988
32	2992	2988	2987
33	28	1210	1213
34	410	2997	1210

35	413	1213	2997
36	1210	2997	1213
37	84	1519	1515
38	711	2999	1519
39	410	1515	2999
40	1519	2999	1515
41	196	2127	2129
42	413	3008	2127
43	711	2129	3008
44	2127	3008	2129
45	410	2999	2997
46	711	3008	2999
47	413	2997	3008
48	2999	3008	2997
49	83	1513	1512
50	709	3845	1513
51	708	1512	3845
52	1513	3845	1512
53	196	2129	2128
54	711	3847	2129
55	709	2128	3847
56	2129	3847	2128
57	84	1517	1519
58	708	3846	1517
59	711	1519	3846
60	1517	3846	1519
61	709	3847	3845
62	711	3846	3847
63	708	3845	3846
64	3847	3846	3845
65	11	1128	1126
66	328	2771	1128
67	326	1126	2771
68	1128	2771	1126
69	120	1696	1694
70	787	2776	1696
71	328	1694	2776
72	1696	2776	1694
73	118	1686	1688
74	326	2772	1686
75	787	1688	2772
76	1686	2772	1688
77	328	2776	2771
78	787	2772	2776
79	326	2771	2772
80	2776	2772	2771
81	60	1376	1374
82	576	3446	1376
83	574	1374	3446
84	1376	3446	1374
85	197	2135	2133
86	792	3455	2135
87	576	2133	3455
88	2135	3455	2133
89	120	1695	1698
90	574	3448	1695
91	792	1698	3448
92	1695	3448	1698
93	576	3455	3446

94	792	3448	3455
95	574	3446	3448
96	3455	3448	3446
97	27	1207	1209
98	407	2989	1207
99	409	1209	2989
100	1207	2989	1209
101	118	1689	1687
102	788	2990	1689
103	407	1687	2990
104	1689	2990	1687
105	197	2132	2134
106	409	2994	2132
107	788	2134	2994
108	2132	2994	2134
109	407	2990	2989
110	788	2994	2990
111	409	2989	2994
112	2990	2994	2989
113	120	1698	1696
114	792	3924	1698
115	787	1696	3924
116	1698	3924	1696
117	197	2134	2135
118	788	3925	2134
119	792	2135	3925
120	2134	3925	2135
121	118	1688	1689
122	787	3923	1688
123	788	1689	3923
124	1688	3923	1689
125	792	3925	3924
126	788	3923	3925
127	787	3924	3923
128	3925	3923	3924
129	12	1130	1133
130	330	2781	1130
131	333	1133	2781
132	1130	2781	1133
133	122	1709	1706
134	797	2783	1709
135	330	1706	2783
136	1709	2783	1706
137	125	1724	1726
138	333	2792	1724
139	797	1726	2792
140	1724	2792	1726
141	330	2783	2781
142	797	2792	2783
143	333	2781	2792
144	2783	2792	2781
145	28	1215	1211
146	415	3001	1215
147	411	1211	3001
148	1215	3001	1211
149	199	2146	2144
150	799	3012	2146
151	415	2144	3012
152	2146	3012	2144

153	122	1707	1711
154	411	3003	1707
155	799	1711	3003
156	1707	3003	1711
157	415	3012	3001
158	799	3003	3012
159	411	3001	3003
160	3012	3003	3001
161	60	1375	1377
162	575	3450	1375
163	577	1377	3450
164	1375	3450	1377
165	125	1728	1725
166	808	3452	1728
167	575	1725	3452
168	1728	3452	1725
169	199	2145	2147
170	577	3457	2145
171	808	2147	3457
172	2145	3457	2147
173	575	3452	3450
174	808	3457	3452
175	577	3450	3457
176	3452	3457	3450
177	122	1711	1709
178	799	3934	1711
179	797	1709	3934
180	1711	3934	1709
181	199	2147	2146
182	808	3937	2147
183	799	2146	3937
184	2147	3937	2146
185	125	1726	1728
186	797	3935	1726
187	808	1728	3935
188	1726	3935	1728
189	799	3937	3934
190	808	3935	3937
191	797	3934	3935
192	3937	3935	3934
193	27	1209	1208
194	409	2991	1209
195	408	1208	2991
196	1209	2991	1208
197	197	2136	2132
198	997	2995	2136
199	409	2132	2995
200	2136	2995	2132
201	196	2126	2130
202	408	2993	2126
203	997	2130	2993
204	2126	2993	2130
205	409	2995	2991
206	997	2993	2995
207	408	2991	2993
208	2995	2993	2991
209	60	1377	1376
210	577	3454	1377
211	576	1376	3454

212	1377	3454	1376
213	199	2149	2145
214	999	3458	2149
215	577	2145	3458
216	2149	3458	2145
217	197	2133	2137
218	576	3456	2133
219	999	2137	3456
220	2133	3456	2137
221	577	3458	3454
222	999	3456	3458
223	576	3454	3456
224	3458	3456	3454
225	28	1213	1215
226	413	3007	1213
227	415	1215	3007
228	1213	3007	1215
229	196	2131	2127
230	998	3009	2131
231	413	2127	3009
232	2131	3009	2127
233	199	2144	2148
234	415	3013	2144
235	998	2148	3013
236	2144	3013	2148
237	413	3009	3007
238	998	3013	3009
239	415	3007	3013
240	3009	3013	3007
241	197	2137	2136
242	999	4134	2137
243	997	2136	4134
244	2137	4134	2136
245	199	2148	2149
246	998	4135	2148
247	999	2149	4135
248	2148	4135	2149
249	196	2130	2131
250	997	4133	2130
251	998	2131	4133
252	2130	4133	2131
253	999	4135	4134
254	998	4133	4135
255	997	4134	4133
256	4135	4133	4134
257	42	1276	1274
258	476	3158	1276
259	474	1274	3158
260	1276	3158	1274
261	221	2279	2277
262	738	3167	2279
263	476	2277	3167
264	2279	3167	2277
265	98	1579	1582
266	474	3160	1579
267	738	1582	3160
268	1579	3160	1582
269	476	3167	3158
270	738	3160	3167

271	474	3158	3160
272	3167	3160	3158
273	40	1266	1268
274	466	3140	1266
275	468	1268	3140
276	1266	3140	1268
277	96	1573	1571
278	734	3141	1573
279	466	1571	3141
280	1573	3141	1571
281	221	2276	2278
282	468	3145	2276
283	734	2278	3145
284	2276	3145	2278
285	466	3141	3140
286	734	3145	3141
287	468	3140	3145
288	3141	3145	3140
289	22	1180	1178
290	380	2906	1180
291	378	1178	2906
292	1180	2906	1178
293	172	1984	1982
294	922	2915	1984
295	380	1982	2915
296	1984	2915	1982
297	170	1970	1972
298	378	2908	1970
299	922	1972	2908
300	1970	2908	1972
301	380	2915	2906
302	922	2908	2915
303	378	2906	2908
304	2915	2908	2906
305	61	1383	1381
306	583	3468	1383
307	581	1381	3468
308	1383	3468	1381
309	226	2309	2307
310	931	3475	2309
311	583	2307	3475
312	2309	3475	2307
313	172	1983	1986
314	581	3470	1983
315	931	1986	3470
316	1983	3470	1986
317	583	3475	3468
318	931	3470	3475
319	581	3468	3470
320	3475	3470	3468
321	42	1275	1278
322	475	3162	1275
323	478	1278	3162
324	1275	3162	1278
325	170	1974	1971
326	924	3164	1974
327	475	1971	3164
328	1974	3164	1971
329	226	2306	2308

330	478	3172	2306
331	924	2308	3172
332	2306	3172	2308
333	475	3164	3162
334	924	3172	3164
335	478	3162	3172
336	3164	3172	3162
337	172	1986	1984
338	931	4059	1986
339	922	1984	4059
340	1986	4059	1984
341	226	2308	2309
342	924	4062	2308
343	931	2309	4062
344	2308	4062	2309
345	170	1972	1974
346	922	4058	1972
347	924	1974	4058
348	1972	4058	1974
349	931	4062	4059
350	924	4058	4062
351	922	4059	4058
352	4062	4058	4059
353	11	1127	1129
354	327	2773	1127
355	329	1129	2773
356	1127	2773	1129
357	119	1692	1690
358	789	2774	1692
359	327	1690	2774
360	1692	2774	1690
361	121	1700	1702
362	329	2778	1700
363	789	1702	2778
364	1700	2778	1702
365	327	2774	2773
366	789	2778	2774
367	329	2773	2778
368	2774	2778	2773
369	222	2284	2282
370	790	3147	2284
371	469	2282	3147
372	2284	3147	2282
373	61	1380	1382
374	580	3464	1380
375	582	1382	3464
376	1380	3464	1382
377	121	1704	1701
378	794	3466	1704
379	580	1701	3466
380	1704	3466	1701
381	222	2283	2285
382	582	3473	2283
383	794	2285	3473
384	2283	3473	2285
385	580	3466	3464
386	794	3473	3466
387	582	3464	3473
388	3466	3473	3464

389	119	1693	1692
390	790	3926	1693
391	789	1692	3926
392	1693	3926	1692
393	222	2285	2284
394	794	3928	2285
395	790	2284	3928
396	2285	3928	2284
397	121	1702	1704
398	789	3927	1702
399	794	1704	3927
400	1702	3927	1704
401	790	3928	3926
402	794	3927	3928
403	789	3926	3927
404	3928	3927	3926
405	42	1278	1276
406	478	3166	1278
407	476	1276	3166
408	1278	3166	1276
409	226	2310	2306
410	1022	3173	2310
411	478	2306	3173
412	2310	3173	2306
413	221	2277	2281
414	476	3168	2277
415	1022	2281	3168
416	2277	3168	2281
417	478	3173	3166
418	1022	3168	3173
419	476	3166	3168
420	3173	3168	3166
421	61	1382	1383
422	582	3472	1382
423	583	1383	3472
424	1382	3472	1383
425	222	2287	2283
426	1023	3474	2287
427	582	2283	3474
428	2287	3474	2283
429	226	2307	2311
430	583	3476	2307
431	1023	2311	3476
432	2307	3476	2311
433	582	3474	3472
434	1023	3476	3474
435	583	3472	3476
436	3474	3476	3472
437	40	1268	1269
438	468	3144	1268
439	469	1269	3144
440	1268	3144	1269
441	221	2280	2276
442	1021	3146	2280
443	468	2276	3146
444	2280	3146	2276
445	222	2282	2286
446	469	3148	2282
447	1021	2286	3148

448	2282	3148	2286
449	468	3146	3144
450	1021	3148	3146
451	469	3144	3148
452	3146	3148	3144
453	226	2311	2310
454	1023	4159	2311
455	1022	2310	4159
456	2311	4159	2310
457	222	2286	2287
458	1021	4158	2286
459	1023	2287	4158
460	2286	4158	2287
461	221	2281	2280
462	1022	4157	2281
463	1021	2280	4157
464	2281	4157	2280
465	1023	4158	4159
466	1021	4157	4158
467	1022	4159	4157
468	4158	4157	4159
469	7	1108	1111
470	308	2727	1108
471	311	1111	2727
472	1108	2727	1111
473	100	1593	1590
474	743	2729	1593
475	308	1590	2729
476	1593	2729	1590
477	103	1608	1610
478	311	2738	1608
479	743	1610	2738
480	1608	2738	1610
481	308	2729	2727
482	743	2738	2729
483	311	2727	2738
484	2729	2738	2727
485	44	1289	1286
486	489	3195	1289
487	486	1286	3195
488	1289	3195	1286
489	231	2338	2336
490	745	3206	2338
491	489	2336	3206
492	2338	3206	2336
493	100	1591	1595
494	486	3197	1591
495	745	1595	3197
496	1591	3197	1595
497	489	3206	3195
498	745	3197	3206
499	486	3195	3197
500	3206	3197	3195
501	47	1304	1306
502	504	3248	1304
503	506	1306	3248
504	1304	3248	1306
505	103	1612	1609
506	754	3250	1612

507	504	1609	3250
508	1612	3250	1609
509	231	2337	2339
510	506	3257	2337
511	754	2339	3257
512	2337	3257	2339
513	504	3250	3248
514	754	3257	3250
515	506	3248	3257
516	3250	3257	3248
517	100	1595	1593
518	745	3880	1595
519	743	1593	3880
520	1595	3880	1593
521	231	2339	2338
522	754	3883	2339
523	745	2338	3883
524	2339	3883	2338
525	103	1610	1612
526	743	3881	1610
527	754	1612	3881
528	1610	3881	1612
529	745	3883	3880
530	754	3881	3883
531	743	3880	3881
532	3883	3881	3880
533	12	1135	1131
534	335	2785	1135
535	331	1131	2785
536	1135	2785	1131
537	127	1738	1736
538	801	2796	1738
539	335	1736	2796
540	1738	2796	1736
541	123	1712	1715
542	331	2787	1712
543	801	1715	2787
544	1712	2787	1715
545	335	2796	2785
546	801	2787	2796
547	331	2785	2787
548	2796	2787	2785
549	63	1394	1392
550	594	3500	1394
551	592	1392	3500
552	1394	3500	1392
553	233	2351	2349
554	812	3509	2351
555	594	2349	3509
556	2351	3509	2349
557	127	1737	1740
558	592	3502	1737
559	812	1740	3502
560	1737	3502	1740
561	594	3509	3500
562	812	3502	3509
563	592	3500	3502
564	3509	3502	3500
565	44	1287	1291

566	487	3199	1287
567	491	1291	3199
568	1287	3199	1291
569	123	1717	1713
570	803	3201	1717
571	487	1713	3201
572	1717	3201	1713
573	233	2348	2350
574	491	3210	2348
575	803	2350	3210
576	2348	3210	2350
577	487	3201	3199
578	803	3210	3201
579	491	3199	3210
580	3201	3210	3199
581	127	1740	1738
582	812	3941	1740
583	801	1738	3941
584	1740	3941	1738
585	233	2350	2351
586	803	3943	2350
587	812	2351	3943
588	2350	3943	2351
589	123	1715	1717
590	801	3940	1715
591	803	1717	3940
592	1715	3940	1717
593	812	3943	3941
594	803	3940	3943
595	801	3941	3940
596	3943	3940	3941
597	22	1179	1181
598	379	2910	1179
599	381	1181	2910
600	1179	2910	1181
601	171	1978	1976
602	926	2912	1978
603	379	1976	2912
604	1978	2912	1976
605	173	1988	1990
606	381	2917	1988
607	926	1990	2917
608	1988	2917	1990
609	379	2912	2910
610	926	2917	2912
611	381	2910	2917
612	2912	2917	2910
613	47	1308	1305
614	508	3252	1308
615	505	1305	3252
616	1308	3252	1305
617	242	2404	2402
618	928	3262	2404
619	508	2402	3262
620	2404	3262	2402
621	171	1977	1980
622	505	3254	1977
623	928	1980	3254
624	1977	3254	1980

625	508	3262	3252
626	928	3254	3262
627	505	3252	3254
628	3262	3254	3252
629	63	1393	1395
630	593	3504	1393
631	595	1395	3504
632	1393	3504	1395
633	173	1992	1989
634	933	3506	1992
635	593	1989	3506
636	1992	3506	1989
637	242	2403	2405
638	595	3511	2403
639	933	2405	3511
640	2403	3511	2405
641	593	3506	3504
642	933	3511	3506
643	595	3504	3511
644	3506	3511	3504
645	171	1980	1978
646	928	4064	1980
647	926	1978	4064
648	1980	4064	1978
649	242	2405	2404
650	933	4068	2405
651	928	2404	4068
652	2405	4068	2404
653	173	1990	1992
654	926	4065	1990
655	933	1992	4065
656	1990	4065	1992
657	928	4068	4064
658	933	4065	4068
659	926	4064	4065
660	4068	4065	4064
661	44	1291	1289
662	491	3205	1291
663	489	1289	3205
664	1291	3205	1289
665	233	2352	2348
666	1032	3211	2352
667	491	2348	3211
668	2352	3211	2348
669	231	2336	2340
670	489	3207	2336
671	1032	2340	3207
672	2336	3207	2340
673	491	3211	3205
674	1032	3207	3211
675	489	3205	3207
676	3211	3207	3205
677	63	1395	1394
678	595	3508	1395
679	594	1394	3508
680	1395	3508	1394
681	242	2407	2403
682	1035	3512	2407
683	595	2403	3512

684	2407	3512	2403
685	233	2349	2353
686	594	3510	2349
687	1035	2353	3510
688	2349	3510	2353
689	595	3512	3508
690	1035	3510	3512
691	594	3508	3510
692	3512	3510	3508
693	47	1306	1308
694	506	3256	1306
695	508	1308	3256
696	1306	3256	1308
697	231	2341	2337
698	1033	3258	2341
699	506	2337	3258
700	2341	3258	2337
701	242	2402	2406
702	508	3263	2402
703	1033	2406	3263
704	2402	3263	2406
705	506	3258	3256
706	1033	3263	3258
707	508	3256	3263
708	3258	3263	3256
709	233	2353	2352
710	1035	4170	2353
711	1032	2352	4170
712	2353	4170	2352
713	242	2406	2407
714	1033	4171	2406
715	1035	2407	4171
716	2406	4171	2407
717	231	2340	2341
718	1032	4169	2340
719	1033	2341	4169
720	2340	4169	2341
721	1035	4171	4170
722	1033	4169	4171
723	1032	4170	4169
724	4171	4169	4170
725	11	1129	1128
726	329	2775	1129
727	328	1128	2775
728	1129	2775	1128
729	121	1703	1700
730	791	2779	1703
731	329	1700	2779
732	1703	2779	1700
733	120	1694	1697
734	328	2777	1694
735	791	1697	2777
736	1694	2777	1697
737	329	2779	2775
738	791	2777	2779
739	328	2775	2777
740	2779	2777	2775
741	61	1384	1380
742	584	3465	1384

743	580	1380	3465
744	1384	3465	1380
745	269	2567	2565
746	795	3478	2567
747	584	2565	3478
748	2567	3478	2565
749	121	1701	1705
750	580	3467	1701
751	795	1705	3467
752	1701	3467	1705
753	584	3478	3465
754	795	3467	3478
755	580	3465	3467
756	3478	3467	3465
757	60	1374	1378
758	574	3447	1374
759	578	1378	3447
760	1374	3447	1378
761	120	1699	1695
762	793	3449	1699
763	574	1695	3449
764	1699	3449	1695
765	269	2564	2566
766	578	3460	2564
767	793	2566	3460
768	2564	3460	2566
769	574	3449	3447
770	793	3460	3449
771	578	3447	3460
772	3449	3460	3447
773	121	1705	1703
774	795	3930	1705
775	791	1703	3930
776	1705	3930	1703
777	269	2566	2567
778	793	3931	2566
779	795	2567	3931
780	2566	3931	2567
781	120	1697	1699
782	791	3929	1697
783	793	1699	3929
784	1697	3929	1699
785	795	3931	3930
786	793	3929	3931
787	791	3930	3929
788	3931	3929	3930
789	22	1181	1180
790	381	2914	1181
791	380	1180	2914
792	1181	2914	1180
793	173	1991	1988
794	930	2918	1991
795	381	1988	2918
796	1991	2918	1988
797	172	1982	1985
798	380	2916	1982
799	930	1985	2916
800	1982	2916	1985
801	381	2918	2914

802	930	2916	2918
803	380	2914	2916
804	2918	2916	2914
805	63	1397	1393
806	597	3505	1397
807	593	1393	3505
808	1397	3505	1393
809	271	2579	2577
810	934	3516	2579
811	597	2577	3516
812	2579	3516	2577
813	173	1989	1993
814	593	3507	1989
815	934	1993	3507
816	1989	3507	1993
817	597	3516	3505
818	934	3507	3516
819	593	3505	3507
820	3516	3507	3505
821	61	1381	1385
822	581	3469	1381
823	585	1385	3469
824	1381	3469	1385
825	172	1987	1983
826	932	3471	1987
827	581	1983	3471
828	1987	3471	1983
829	271	2576	2578
830	585	3480	2576
831	932	2578	3480
832	2576	3480	2578
833	581	3471	3469
834	932	3480	3471
835	585	3469	3480
836	3471	3480	3469
837	173	1993	1991
838	934	4071	1993
839	930	1991	4071
840	1993	4071	1991
841	271	2578	2579
842	932	4072	2578
843	934	2579	4072
844	2578	4072	2579
845	172	1985	1987
846	930	4070	1985
847	932	1987	4070
848	1985	4070	1987
849	934	4072	4071
850	932	4070	4072
851	930	4071	4070
852	4072	4070	4071
853	12	1133	1135
854	333	2791	1133
855	335	1135	2791
856	1133	2791	1135
857	125	1727	1724
858	807	2793	1727
859	333	1724	2793
860	1727	2793	1724

861	127	1736	1739
862	335	2797	1736
863	807	1739	2797
864	1736	2797	1739
865	333	2793	2791
866	807	2797	2793
867	335	2791	2797
868	2793	2797	2791
869	60	1379	1375
870	579	3451	1379
871	575	1375	3451
872	1379	3451	1375
873	270	2572	2570
874	809	3462	2572
875	579	2570	3462
876	2572	3462	2570
877	125	1725	1729
878	575	3453	1725
879	809	1729	3453
880	1725	3453	1729
881	579	3462	3451
882	809	3453	3462
883	575	3451	3453
884	3462	3453	3451
885	63	1392	1396
886	592	3501	1392
887	596	1396	3501
888	1392	3501	1396
889	127	1741	1737
890	813	3503	1741
891	592	1737	3503
892	1741	3503	1737
893	270	2571	2573
894	596	3514	2571
895	813	2573	3514
896	2571	3514	2573
897	592	3503	3501
898	813	3514	3503
899	596	3501	3514
900	3503	3514	3501
901	125	1729	1727
902	809	3947	1729
903	807	1727	3947
904	1729	3947	1727
905	270	2573	2572
906	813	3949	2573
907	809	2572	3949
908	2573	3949	2572
909	127	1739	1741
910	807	3948	1739
911	813	1741	3948
912	1739	3948	1741
913	809	3949	3947
914	813	3948	3949
915	807	3947	3948
916	3949	3948	3947
917	61	1385	1384
918	585	3477	1385
919	584	1384	3477

920	1385	3477	1384
921	271	2580	2576
922	1070	3481	2580
923	585	2576	3481
924	2580	3481	2576
925	269	2565	2569
926	584	3479	2565
927	1070	2569	3479
928	2565	3479	2569
929	585	3481	3477
930	1070	3479	3481
931	584	3477	3479
932	3481	3479	3477
933	63	1396	1397
934	596	3513	1396
935	597	1397	3513
936	1396	3513	1397
937	270	2575	2571
938	1071	3515	2575
939	596	2571	3515
940	2575	3515	2571
941	271	2577	2581
942	597	3517	2577
943	1071	2581	3517
944	2577	3517	2581
945	596	3515	3513
946	1071	3517	3515
947	597	3513	3517
948	3515	3517	3513
949	60	1378	1379
950	578	3459	1378
951	579	1379	3459
952	1378	3459	1379
953	269	2568	2564
954	1069	3461	2568
955	578	2564	3461
956	2568	3461	2564
957	270	2570	2574
958	579	3463	2570
959	1069	2574	3463
960	2570	3463	2574
961	578	3461	3459
962	1069	3463	3461
963	579	3459	3463
964	3461	3463	3459
965	271	2581	2580
966	1071	4207	2581
967	1070	2580	4207
968	2581	4207	2580
969	270	2574	2575
970	1069	4206	2574
971	1071	2575	4206
972	2574	4206	2575
973	269	2569	2568
974	1070	4205	2569
975	1069	2568	4205
976	2569	4205	2568
977	1071	4206	4207
978	1069	4205	4206

979	1070	4207	4205
980	4206	4205	4207
981	3	1096	1095
982	296	2699	1096
983	295	1095	2699
984	1096	2699	1095
985	88	1534	1532
986	715	2701	1534
987	296	1532	2701
988	1534	2701	1532
989	87	1528	1530
990	295	2700	1528
991	715	1530	2700
992	1528	2700	1530
993	296	2701	2699
994	715	2700	2701
995	295	2699	2700
996	2701	2700	2699
997	32	1230	1228
998	430	3041	1230
999	428	1228	3041
1000	1230	3041	1228
1001	203	2171	2169
1002	717	3046	2171
1003	430	2169	3046
1004	2171	3046	2169
1005	88	1533	1535
1006	428	3042	1533
1007	717	1535	3042
1008	1533	3042	1535
1009	430	3046	3041
1010	717	3042	3046
1011	428	3041	3042
1012	3046	3042	3041
1013	31	1224	1226
1014	424	3032	1224
1015	426	1226	3032
1016	1224	3032	1226
1017	87	1531	1529
1018	716	3033	1531
1019	424	1529	3033
1020	1531	3033	1529
1021	203	2168	2170
1022	426	3037	2168
1023	716	2170	3037
1024	2168	3037	2170
1025	424	3033	3032
1026	716	3037	3033
1027	426	3032	3037
1028	3033	3037	3032
1029	88	1535	1534
1030	717	3852	1535
1031	715	1534	3852
1032	1535	3852	1534
1033	203	2170	2171
1034	716	3853	2170
1035	717	2171	3853
1036	2170	3853	2171
1037	87	1530	1531

1038	715	3851	1530
1039	716	1531	3851
1040	1530	3851	1531
1041	717	3853	3852
1042	716	3851	3853
1043	715	3852	3851
1044	3853	3851	3852
1045	16	1150	1148
1046	350	2825	1150
1047	348	1148	2825
1048	1150	2825	1148
1049	142	1812	1810
1050	841	2830	1812
1051	350	1810	2830
1052	1812	2830	1810
1053	140	1802	1804
1054	348	2826	1802
1055	841	1804	2826
1056	1802	2826	1804
1057	350	2830	2825
1058	841	2826	2830
1059	348	2825	2826
1060	2830	2826	2825
1061	67	1419	1417
1062	619	3576	1419
1063	617	1417	3576
1064	1419	3576	1417
1065	205	2183	2181
1066	846	3583	2183
1067	619	2181	3583
1068	2183	3583	2181
1069	142	1811	1814
1070	617	3578	1811
1071	846	1814	3578
1072	1811	3578	1814
1073	619	3583	3576
1074	846	3578	3583
1075	617	3576	3578
1076	3583	3578	3576
1077	32	1229	1231
1078	429	3043	1229
1079	431	1231	3043
1080	1229	3043	1231
1081	140	1805	1803
1082	842	3044	1805
1083	429	1803	3044
1084	1805	3044	1803
1085	205	2180	2182
1086	431	3048	2180
1087	842	2182	3048
1088	2180	3048	2182
1089	429	3044	3043
1090	842	3048	3044
1091	431	3043	3048
1092	3044	3048	3043
1093	142	1814	1812
1094	846	3978	1814
1095	841	1812	3978
1096	1814	3978	1812

1097	205	2182	2183
1098	842	3979	2182
1099	846	2183	3979
1100	2182	3979	2183
1101	140	1804	1805
1102	841	3977	1804
1103	842	1805	3977
1104	1804	3977	1805
1105	846	3979	3978
1106	842	3977	3979
1107	841	3978	3977
1108	3979	3977	3978
1109	15	1144	1146
1110	344	2816	1144
1111	346	1146	2816
1112	1144	2816	1146
1113	136	1784	1782
1114	832	2817	1784
1115	344	1782	2817
1116	1784	2817	1782
1117	138	1790	1792
1118	346	2821	1790
1119	832	1792	2821
1120	1790	2821	1792
1121	344	2817	2816
1122	832	2821	2817
1123	346	2816	2821
1124	2817	2821	2816
1125	31	1227	1225
1126	427	3034	1227
1127	425	1225	3034
1128	1227	3034	1225
1129	204	2176	2174
1130	833	3039	2176
1131	427	2174	3039
1132	2176	3039	2174
1133	136	1783	1785
1134	425	3035	1783
1135	833	1785	3035
1136	1783	3035	1785
1137	427	3039	3034
1138	833	3035	3039
1139	425	3034	3035
1140	3039	3035	3034
1141	67	1416	1418
1142	616	3572	1416
1143	618	1418	3572
1144	1416	3572	1418
1145	138	1794	1791
1146	837	3574	1794
1147	616	1791	3574
1148	1794	3574	1791
1149	204	2175	2177
1150	618	3581	2175
1151	837	2177	3581
1152	2175	3581	2177
1153	616	3574	3572
1154	837	3581	3574
1155	618	3572	3581

1156	3574	3581	3572
1157	136	1785	1784
1158	833	3968	1785
1159	832	1784	3968
1160	1785	3968	1784
1161	204	2177	2176
1162	837	3970	2177
1163	833	2176	3970
1164	2177	3970	2176
1165	138	1792	1794
1166	832	3969	1792
1167	837	1794	3969
1168	1792	3969	1794
1169	833	3970	3968
1170	837	3969	3970
1171	832	3968	3969
1172	3970	3969	3968
1173	32	1231	1230
1174	431	3045	1231
1175	430	1230	3045
1176	1231	3045	1230
1177	205	2184	2180
1178	1004	3049	2184
1179	431	2180	3049
1180	2184	3049	2180
1181	203	2169	2173
1182	430	3047	2169
1183	1004	2173	3047
1184	2169	3047	2173
1185	431	3049	3045
1186	1004	3047	3049
1187	430	3045	3047
1188	3049	3047	3045
1189	67	1418	1419
1190	618	3580	1418
1191	619	1419	3580
1192	1418	3580	1419
1193	204	2179	2175
1194	1005	3582	2179
1195	618	2175	3582
1196	2179	3582	2175
1197	205	2181	2185
1198	619	3584	2181
1199	1005	2185	3584
1200	2181	3584	2185
1201	618	3582	3580
1202	1005	3584	3582
1203	619	3580	3584
1204	3582	3584	3580
1205	31	1226	1227
1206	426	3036	1226
1207	427	1227	3036
1208	1226	3036	1227
1209	203	2172	2168
1210	1003	3038	2172
1211	426	2168	3038
1212	2172	3038	2168
1213	204	2174	2178
1214	427	3040	2174

1215	1003	2178	3040
1216	2174	3040	2178
1217	426	3038	3036
1218	1003	3040	3038
1219	427	3036	3040
1220	3038	3040	3036
1221	205	2185	2184
1222	1005	4141	2185
1223	1004	2184	4141
1224	2185	4141	2184
1225	204	2178	2179
1226	1003	4140	2178
1227	1005	2179	4140
1228	2178	4140	2179
1229	203	2173	2172
1230	1004	4139	2173
1231	1003	2172	4139
1232	2173	4139	2172
1233	1005	4140	4141
1234	1003	4139	4140
1235	1004	4141	4139
1236	4140	4139	4141
1237	9	1120	1118
1238	320	2753	1120
1239	318	1118	2753
1240	1120	2753	1118
1241	112	1656	1654
1242	769	2758	1656
1243	320	1654	2758
1244	1656	2758	1654
1245	110	1646	1648
1246	318	2754	1646
1247	769	1648	2754
1248	1646	2754	1648
1249	320	2758	2753
1250	769	2754	2758
1251	318	2753	2754
1252	2758	2754	2753
1253	56	1352	1350
1254	552	3374	1352
1255	550	1350	3374
1256	1352	3374	1350
1257	257	2495	2493
1258	774	3383	2495
1259	552	2493	3383
1260	2495	3383	2493
1261	112	1655	1658
1262	550	3376	1655
1263	774	1658	3376
1264	1655	3376	1658
1265	552	3383	3374
1266	774	3376	3383
1267	550	3374	3376
1268	3383	3376	3374
1269	54	1342	1344
1270	542	3356	1342
1271	544	1344	3356
1272	1342	3356	1344
1273	110	1649	1647

1274	770	3357	1649
1275	542	1647	3357
1276	1649	3357	1647
1277	257	2492	2494
1278	544	3361	2492
1279	770	2494	3361
1280	2492	3361	2494
1281	542	3357	3356
1282	770	3361	3357
1283	544	3356	3361
1284	3357	3361	3356
1285	112	1658	1656
1286	774	3906	1658
1287	769	1656	3906
1288	1658	3906	1656
1289	257	2494	2495
1290	770	3907	2494
1291	774	2495	3907
1292	2494	3907	2495
1293	110	1648	1649
1294	769	3905	1648
1295	770	1649	3905
1296	1648	3905	1649
1297	774	3907	3906
1298	770	3905	3907
1299	769	3906	3905
1300	3907	3905	3906
1301	23	1187	1185
1302	387	2928	1187
1303	385	1185	2928
1304	1187	2928	1185
1305	179	2026	2024
1306	944	2935	2026
1307	387	2024	2935
1308	2026	2935	2024
1309	177	2012	2014
1310	385	2930	2012
1311	944	2014	2930
1312	2012	2930	2014
1313	387	2935	2928
1314	944	2930	2935
1315	385	2928	2930
1316	2935	2930	2928
1317	69	1431	1429
1318	631	3612	1431
1319	629	1429	3612
1320	1431	3612	1429
1321	262	2525	2523
1322	951	3619	2525
1323	631	2523	3619
1324	2525	3619	2523
1325	179	2025	2028
1326	629	3614	2025
1327	951	2028	3614
1328	2025	3614	2028
1329	631	3619	3612
1330	951	3614	3619
1331	629	3612	3614
1332	3619	3614	3612

1333	56	1351	1354
1334	551	3378	1351
1335	554	1354	3378
1336	1351	3378	1354
1337	177	2016	2013
1338	946	3380	2016
1339	551	2013	3380
1340	2016	3380	2013
1341	262	2522	2524
1342	554	3388	2522
1343	946	2524	3388
1344	2522	3388	2524
1345	551	3380	3378
1346	946	3388	3380
1347	554	3378	3388
1348	3380	3388	3378
1349	179	2028	2026
1350	951	4083	2028
1351	944	2026	4083
1352	2028	4083	2026
1353	262	2524	2525
1354	946	4086	2524
1355	951	2525	4086
1356	2524	4086	2525
1357	177	2014	2016
1358	944	4082	2014
1359	946	2016	4082
1360	2014	4082	2016
1361	951	4086	4083
1362	946	4082	4086
1363	944	4083	4082
1364	4086	4082	4083
1365	16	1149	1151
1366	349	2827	1149
1367	351	1151	2827
1368	1149	2827	1151
1369	141	1808	1806
1370	843	2828	1808
1371	349	1806	2828
1372	1808	2828	1806
1373	143	1816	1818
1374	351	2832	1816
1375	843	1818	2832
1376	1816	2832	1818
1377	349	2828	2827
1378	843	2832	2828
1379	351	2827	2832
1380	2828	2832	2827
1381	54	1345	1343
1382	545	3358	1345
1383	543	1343	3358
1384	1345	3358	1343
1385	258	2500	2498
1386	844	3363	2500
1387	545	2498	3363
1388	2500	3363	2498
1389	141	1807	1809
1390	543	3359	1807
1391	844	1809	3359

1392	1807	3359	1809
1393	545	3363	3358
1394	844	3359	3363
1395	543	3358	3359
1396	3363	3359	3358
1397	69	1428	1430
1398	628	3608	1428
1399	630	1430	3608
1400	1428	3608	1430
1401	143	1820	1817
1402	848	3610	1820
1403	628	1817	3610
1404	1820	3610	1817
1405	258	2499	2501
1406	630	3617	2499
1407	848	2501	3617
1408	2499	3617	2501
1409	628	3610	3608
1410	848	3617	3610
1411	630	3608	3617
1412	3610	3617	3608
1413	141	1809	1808
1414	844	3980	1809
1415	843	1808	3980
1416	1809	3980	1808
1417	258	2501	2500
1418	848	3982	2501
1419	844	2500	3982
1420	2501	3982	2500
1421	143	1818	1820
1422	843	3981	1818
1423	848	1820	3981
1424	1818	3981	1820
1425	844	3982	3980
1426	848	3981	3982
1427	843	3980	3981
1428	3982	3981	3980
1429	56	1354	1352
1430	554	3382	1354
1431	552	1352	3382
1432	1354	3382	1352
1433	262	2526	2522
1434	1058	3389	2526
1435	554	2522	3389
1436	2526	3389	2522
1437	257	2493	2497
1438	552	3384	2493
1439	1058	2497	3384
1440	2493	3384	2497
1441	554	3389	3382
1442	1058	3384	3389
1443	552	3382	3384
1444	3389	3384	3382
1445	69	1430	1431
1446	630	3616	1430
1447	631	1431	3616
1448	1430	3616	1431
1449	258	2503	2499
1450	1059	3618	2503

1451	630	2499	3618
1452	2503	3618	2499
1453	262	2523	2527
1454	631	3620	2523
1455	1059	2527	3620
1456	2523	3620	2527
1457	630	3618	3616
1458	1059	3620	3618
1459	631	3616	3620
1460	3618	3620	3616
1461	54	1344	1345
1462	544	3360	1344
1463	545	1345	3360
1464	1344	3360	1345
1465	257	2496	2492
1466	1057	3362	2496
1467	544	2492	3362
1468	2496	3362	2492
1469	258	2498	2502
1470	545	3364	2498
1471	1057	2502	3364
1472	2498	3364	2502
1473	544	3362	3360
1474	1057	3364	3362
1475	545	3360	3364
1476	3362	3364	3360
1477	262	2527	2526
1478	1059	4195	2527
1479	1058	2526	4195
1480	2527	4195	2526
1481	258	2502	2503
1482	1057	4194	2502
1483	1059	2503	4194
1484	2502	4194	2503
1485	257	2497	2496
1486	1058	4193	2497
1487	1057	2496	4193
1488	2497	4193	2496
1489	1059	4194	4195
1490	1057	4193	4194
1491	1058	4195	4193
1492	4194	4193	4195
1493	6	1105	1107
1494	305	2719	1105
1495	307	1107	2719
1496	1105	2719	1107
1497	97	1576	1574
1498	735	2720	1576
1499	305	1574	2720
1500	1576	2720	1574
1501	99	1584	1586
1502	307	2724	1584
1503	735	1586	2724
1504	1584	2724	1586
1505	305	2720	2719
1506	735	2724	2720
1507	307	2719	2724
1508	2720	2724	2719
1509	41	1272	1270

1510	472	3149	1272
1511	470	1270	3149
1512	1272	3149	1270
1513	223	2290	2288
1514	736	3154	2290
1515	472	2288	3154
1516	2290	3154	2288
1517	97	1575	1577
1518	470	3150	1575
1519	736	1577	3150
1520	1575	3150	1577
1521	472	3154	3149
1522	736	3150	3154
1523	470	3149	3150
1524	3154	3150	3149
1525	43	1280	1282
1526	480	3176	1280
1527	482	1282	3176
1528	1280	3176	1282
1529	99	1588	1585
1530	740	3178	1588
1531	480	1585	3178
1532	1588	3178	1585
1533	223	2289	2291
1534	482	3185	2289
1535	740	2291	3185
1536	2289	3185	2291
1537	480	3178	3176
1538	740	3185	3178
1539	482	3176	3185
1540	3178	3185	3176
1541	97	1577	1576
1542	736	3872	1577
1543	735	1576	3872
1544	1577	3872	1576
1545	223	2291	2290
1546	740	3874	2291
1547	736	2290	3874
1548	2291	3874	2290
1549	99	1586	1588
1550	735	3873	1586
1551	740	1588	3873
1552	1586	3873	1588
1553	736	3874	3872
1554	740	3873	3874
1555	735	3872	3873
1556	3874	3873	3872
1557	15	1147	1145
1558	347	2818	1147
1559	345	1145	2818
1560	1147	2818	1145
1561	139	1798	1796
1562	834	2823	1798
1563	347	1796	2823
1564	1798	2823	1796
1565	137	1786	1788
1566	345	2819	1786
1567	834	1788	2819
1568	1786	2819	1788

1569	347	2823	2818
1570	834	2819	2823
1571	345	2818	2819
1572	2823	2819	2818
1573	68	1424	1422
1574	624	3590	1424
1575	622	1422	3590
1576	1424	3590	1422
1577	224	2297	2295
1578	839	3599	2297
1579	624	2295	3599
1580	2297	3599	2295
1581	139	1797	1800
1582	622	3592	1797
1583	839	1800	3592
1584	1797	3592	1800
1585	624	3599	3590
1586	839	3592	3599
1587	622	3590	3592
1588	3599	3592	3590
1589	41	1271	1273
1590	471	3151	1271
1591	473	1273	3151
1592	1271	3151	1273
1593	137	1789	1787
1594	835	3152	1789
1595	471	1787	3152
1596	1789	3152	1787
1597	224	2294	2296
1598	473	3156	2294
1599	835	2296	3156
1600	2294	3156	2296
1601	471	3152	3151
1602	835	3156	3152
1603	473	3151	3156
1604	3152	3156	3151
1605	139	1800	1798
1606	839	3972	1800
1607	834	1798	3972
1608	1800	3972	1798
1609	224	2296	2297
1610	835	3973	2296
1611	839	2297	3973
1612	2296	3973	2297
1613	137	1788	1789
1614	834	3971	1788
1615	835	1789	3971
1616	1788	3971	1789
1617	839	3973	3972
1618	835	3971	3973
1619	834	3972	3971
1620	3973	3971	3972
1621	23	1184	1186
1622	384	2924	1184
1623	386	1186	2924
1624	1184	2924	1186
1625	176	2008	2006
1626	940	2926	2008
1627	384	2006	2926

1628	2008	2926	2006
1629	178	2018	2020
1630	386	2933	2018
1631	940	2020	2933
1632	2018	2933	2020
1633	384	2926	2924
1634	940	2933	2926
1635	386	2924	2933
1636	2926	2933	2924
1637	43	1284	1281
1638	484	3180	1284
1639	481	1281	3180
1640	1284	3180	1281
1641	228	2320	2318
1642	942	3190	2320
1643	484	2318	3190
1644	2320	3190	2318
1645	176	2007	2010
1646	481	3182	2007
1647	942	2010	3182
1648	2007	3182	2010
1649	484	3190	3180
1650	942	3182	3190
1651	481	3180	3182
1652	3190	3182	3180
1653	68	1423	1425
1654	623	3594	1423
1655	625	1425	3594
1656	1423	3594	1425
1657	178	2022	2019
1658	949	3596	2022
1659	623	2019	3596
1660	2022	3596	2019
1661	228	2319	2321
1662	625	3601	2319
1663	949	2321	3601
1664	2319	3601	2321
1665	623	3596	3594
1666	949	3601	3596
1667	625	3594	3601
1668	3596	3601	3594
1669	176	2010	2008
1670	942	4076	2010
1671	940	2008	4076
1672	2010	4076	2008
1673	228	2321	2320
1674	949	4080	2321
1675	942	2320	4080
1676	2321	4080	2320
1677	178	2020	2022
1678	940	4077	2020
1679	949	2022	4077
1680	2020	4077	2022
1681	942	4080	4076
1682	949	4077	4080
1683	940	4076	4077
1684	4080	4077	4076
1685	41	1273	1272
1686	473	3153	1273

1687	472	1272	3153
1688	1273	3153	1272
1689	224	2298	2294
1690	1024	3157	2298
1691	473	2294	3157
1692	2298	3157	2294
1693	223	2288	2292
1694	472	3155	2288
1695	1024	2292	3155
1696	2288	3155	2292
1697	473	3157	3153
1698	1024	3155	3157
1699	472	3153	3155
1700	3157	3155	3153
1701	68	1425	1424
1702	625	3598	1425
1703	624	1424	3598
1704	1425	3598	1424
1705	228	2323	2319
1706	1026	3602	2323
1707	625	2319	3602
1708	2323	3602	2319
1709	224	2295	2299
1710	624	3600	2295
1711	1026	2299	3600
1712	2295	3600	2299
1713	625	3602	3598
1714	1026	3600	3602
1715	624	3598	3600
1716	3602	3600	3598
1717	43	1282	1284
1718	482	3184	1282
1719	484	1284	3184
1720	1282	3184	1284
1721	223	2293	2289
1722	1025	3186	2293
1723	482	2289	3186
1724	2293	3186	2289
1725	228	2318	2322
1726	484	3191	2318
1727	1025	2322	3191
1728	2318	3191	2322
1729	482	3186	3184
1730	1025	3191	3186
1731	484	3184	3191
1732	3186	3191	3184
1733	224	2299	2298
1734	1026	4161	2299
1735	1024	2298	4161
1736	2299	4161	2298
1737	228	2322	2323
1738	1025	4162	2322
1739	1026	2323	4162
1740	2322	4162	2323
1741	223	2292	2293
1742	1024	4160	2292
1743	1025	2293	4160
1744	2292	4160	2293
1745	1026	4162	4161

1746	1025	4160	4162
1747	1024	4161	4160
1748	4162	4160	4161
1749	16	1151	1150
1750	351	2829	1151
1751	350	1150	2829
1752	1151	2829	1150
1753	143	1819	1816
1754	845	2833	1819
1755	351	1816	2833
1756	1819	2833	1816
1757	142	1810	1813
1758	350	2831	1810
1759	845	1813	2831
1760	1810	2831	1813
1761	351	2833	2829
1762	845	2831	2833
1763	350	2829	2831
1764	2833	2831	2829
1765	69	1432	1428
1766	632	3609	1432
1767	628	1428	3609
1768	1432	3609	1428
1769	276	2609	2607
1770	849	3622	2609
1771	632	2607	3622
1772	2609	3622	2607
1773	143	1817	1821
1774	628	3611	1817
1775	849	1821	3611
1776	1817	3611	1821
1777	632	3622	3609
1778	849	3611	3622
1779	628	3609	3611
1780	3622	3611	3609
1781	67	1417	1421
1782	617	3577	1417
1783	621	1421	3577
1784	1417	3577	1421
1785	142	1815	1811
1786	847	3579	1815
1787	617	1811	3579
1788	1815	3579	1811
1789	276	2606	2608
1790	621	3588	2606
1791	847	2608	3588
1792	2606	3588	2608
1793	617	3579	3577
1794	847	3588	3579
1795	621	3577	3588
1796	3579	3588	3577
1797	143	1821	1819
1798	849	3984	1821
1799	845	1819	3984
1800	1821	3984	1819
1801	276	2608	2609
1802	847	3985	2608
1803	849	2609	3985
1804	2608	3985	2609

1805	142	1813	1815
1806	845	3983	1813
1807	847	1815	3983
1808	1813	3983	1815
1809	849	3985	3984
1810	847	3983	3985
1811	845	3984	3983
1812	3985	3983	3984
1813	23	1186	1187
1814	386	2932	1186
1815	387	1187	2932
1816	1186	2932	1187
1817	178	2021	2018
1818	948	2934	2021
1819	386	2018	2934
1820	2021	2934	2018
1821	179	2024	2027
1822	387	2936	2024
1823	948	2027	2936
1824	2024	2936	2027
1825	386	2934	2932
1826	948	2936	2934
1827	387	2932	2936
1828	2934	2936	2932
1829	68	1427	1423
1830	627	3595	1427
1831	623	1423	3595
1832	1427	3595	1423
1833	277	2614	2612
1834	950	3606	2614
1835	627	2612	3606
1836	2614	3606	2612
1837	178	2019	2023
1838	623	3597	2019
1839	950	2023	3597
1840	2019	3597	2023
1841	627	3606	3595
1842	950	3597	3606
1843	623	3595	3597
1844	3606	3597	3595
1845	69	1429	1433
1846	629	3613	1429
1847	633	1433	3613
1848	1429	3613	1433
1849	179	2029	2025
1850	952	3615	2029
1851	629	2025	3615
1852	2029	3615	2025
1853	277	2613	2615
1854	633	3624	2613
1855	952	2615	3624
1856	2613	3624	2615
1857	629	3615	3613
1858	952	3624	3615
1859	633	3613	3624
1860	3615	3624	3613
1861	178	2023	2021
1862	950	4088	2023
1863	948	2021	4088

1864	2023	4088	2021
1865	277	2615	2614
1866	952	4090	2615
1867	950	2614	4090
1868	2615	4090	2614
1869	179	2027	2029
1870	948	4089	2027
1871	952	2029	4089
1872	2027	4089	2029
1873	950	4090	4088
1874	952	4089	4090
1875	948	4088	4089
1876	4090	4089	4088
1877	15	1146	1147
1878	346	2820	1146
1879	347	1147	2820
1880	1146	2820	1147
1881	138	1793	1790
1882	836	2822	1793
1883	346	1790	2822
1884	1793	2822	1790
1885	139	1796	1799
1886	347	2824	1796
1887	836	1799	2824
1888	1796	2824	1799
1889	346	2822	2820
1890	836	2824	2822
1891	347	2820	2824
1892	2822	2824	2820
1893	67	1420	1416
1894	620	3573	1420
1895	616	1416	3573
1896	1420	3573	1416
1897	275	2602	2600
1898	838	3586	2602
1899	620	2600	3586
1900	2602	3586	2600
1901	138	1791	1795
1902	616	3575	1791
1903	838	1795	3575
1904	1791	3575	1795
1905	620	3586	3573
1906	838	3575	3586
1907	616	3573	3575
1908	3586	3575	3573
1909	68	1422	1426
1910	622	3591	1422
1911	626	1426	3591
1912	1422	3591	1426
1913	139	1801	1797
1914	840	3593	1801
1915	622	1797	3593
1916	1801	3593	1797
1917	275	2601	2603
1918	626	3604	2601
1919	840	2603	3604
1920	2601	3604	2603
1921	622	3593	3591
1922	840	3604	3593

1923	626	3591	3604
1924	3593	3604	3591
1925	138	1795	1793
1926	838	3974	1795
1927	836	1793	3974
1928	1795	3974	1793
1929	275	2603	2602
1930	840	3976	2603
1931	838	2602	3976
1932	2603	3976	2602
1933	139	1799	1801
1934	836	3975	1799
1935	840	1801	3975
1936	1799	3975	1801
1937	838	3976	3974
1938	840	3975	3976
1939	836	3974	3975
1940	3976	3975	3974
1941	69	1433	1432
1942	633	3621	1433
1943	632	1432	3621
1944	1433	3621	1432
1945	277	2617	2613
1946	1077	3625	2617
1947	633	2613	3625
1948	2617	3625	2613
1949	276	2607	2611
1950	632	3623	2607
1951	1077	2611	3623
1952	2607	3623	2611
1953	633	3625	3621
1954	1077	3623	3625
1955	632	3621	3623
1956	3625	3623	3621
1957	68	1426	1427
1958	626	3603	1426
1959	627	1427	3603
1960	1426	3603	1427
1961	275	2605	2601
1962	1076	3605	2605
1963	626	2601	3605
1964	2605	3605	2601
1965	277	2612	2616
1966	627	3607	2612
1967	1076	2616	3607
1968	2612	3607	2616
1969	626	3605	3603
1970	1076	3607	3605
1971	627	3603	3607
1972	3605	3607	3603
1973	67	1421	1420
1974	621	3585	1421
1975	620	1420	3585
1976	1421	3585	1420
1977	276	2610	2606
1978	1075	3589	2610
1979	621	2606	3589
1980	2610	3589	2606
1981	275	2600	2604

1982	620	3587	2600
1983	1075	2604	3587
1984	2600	3587	2604
1985	621	3589	3585
1986	1075	3587	3589
1987	620	3585	3587
1988	3589	3587	3585
1989	277	2616	2617
1990	1076	4213	2616
1991	1077	2617	4213
1992	2616	4213	2617
1993	275	2604	2605
1994	1075	4211	2604
1995	1076	2605	4211
1996	2604	4211	2605
1997	276	2611	2610
1998	1077	4212	2611
1999	1075	2610	4212
2000	2611	4212	2610
2001	1076	4211	4213
2002	1075	4212	4211
2003	1077	4213	4212
2004	4211	4212	4213
2005	4	1097	1099
2006	297	2703	1097
2007	299	1099	2703
2008	1097	2703	1099
2009	89	1539	1536
2010	719	2705	1539
2011	297	1536	2705
2012	1539	2705	1536
2013	91	1546	1548
2014	299	2707	1546
2015	719	1548	2707
2016	1546	2707	1548
2017	297	2705	2703
2018	719	2707	2705
2019	299	2703	2707
2020	2705	2707	2703
2021	33	1235	1232
2022	435	3051	1235
2023	432	1232	3051
2024	1235	3051	1232
2025	207	2194	2192
2026	721	3062	2194
2027	435	2192	3062
2028	2194	3062	2192
2029	89	1537	1541
2030	432	3053	1537
2031	721	1541	3053
2032	1537	3053	1541
2033	435	3062	3051
2034	721	3053	3062
2035	432	3051	3053
2036	3062	3053	3051
2037	35	1242	1244
2038	442	3077	1242
2039	444	1244	3077
2040	1242	3077	1244

2041	91	1549	1547
2042	723	3078	1549
2043	442	1547	3078
2044	1549	3078	1547
2045	207	2193	2195
2046	444	3082	2193
2047	723	2195	3082
2048	2193	3082	2195
2049	442	3078	3077
2050	723	3082	3078
2051	444	3077	3082
2052	3078	3082	3077
2053	89	1541	1539
2054	721	3856	1541
2055	719	1539	3856
2056	1541	3856	1539
2057	207	2195	2194
2058	723	3859	2195
2059	721	2194	3859
2060	2195	3859	2194
2061	91	1548	1549
2062	719	3857	1548
2063	723	1549	3857
2064	1548	3857	1549
2065	721	3859	3856
2066	723	3857	3859
2067	719	3856	3857
2068	3859	3857	3856
2069	17	1155	1152
2070	355	2835	1155
2071	352	1152	2835
2072	1155	2835	1152
2073	147	1842	1840
2074	851	2846	1842
2075	355	1840	2846
2076	1842	2846	1840
2077	144	1822	1825
2078	352	2837	1822
2079	851	1825	2837
2080	1822	2837	1825
2081	355	2846	2835
2082	851	2837	2846
2083	352	2835	2837
2084	2846	2837	2835
2085	71	1442	1440
2086	642	3644	1442
2087	640	1440	3644
2088	1442	3644	1440
2089	209	2207	2205
2090	862	3653	2207
2091	642	2205	3653
2092	2207	3653	2205
2093	147	1841	1844
2094	640	3646	1841
2095	862	1844	3646
2096	1841	3646	1844
2097	642	3653	3644
2098	862	3646	3653
2099	640	3644	3646

2100	3653	3646	3644
2101	33	1233	1237
2102	433	3055	1233
2103	437	1237	3055
2104	1233	3055	1237
2105	144	1827	1823
2106	853	3057	1827
2107	433	1823	3057
2108	1827	3057	1823
2109	209	2204	2206
2110	437	3066	2204
2111	853	2206	3066
2112	2204	3066	2206
2113	433	3057	3055
2114	853	3066	3057
2115	437	3055	3066
2116	3057	3066	3055
2117	147	1844	1842
2118	862	3989	1844
2119	851	1842	3989
2120	1844	3989	1842
2121	209	2206	2207
2122	853	3991	2206
2123	862	2207	3991
2124	2206	3991	2207
2125	144	1825	1827
2126	851	3988	1825
2127	853	1827	3988
2128	1825	3988	1827
2129	862	3991	3989
2130	853	3988	3991
2131	851	3989	3988
2132	3991	3988	3989
2133	19	1162	1164
2134	362	2861	1162
2135	364	1164	2861
2136	1162	2861	1164
2137	154	1880	1878
2138	877	2862	1880
2139	362	1878	2862
2140	1880	2862	1878
2141	156	1886	1888
2142	364	2866	1886
2143	877	1888	2866
2144	1886	2866	1888
2145	362	2862	2861
2146	877	2866	2862
2147	364	2861	2866
2148	2862	2866	2861
2149	35	1245	1243
2150	445	3079	1245
2151	443	1243	3079
2152	1245	3079	1243
2153	211	2218	2216
2154	878	3084	2218
2155	445	2216	3084
2156	2218	3084	2216
2157	154	1879	1881
2158	443	3080	1879

2159	878	1881	3080
2160	1879	3080	1881
2161	445	3084	3079
2162	878	3080	3084
2163	443	3079	3080
2164	3084	3080	3079
2165	71	1441	1443
2166	641	3648	1441
2167	643	1443	3648
2168	1441	3648	1443
2169	156	1890	1887
2170	882	3650	1890
2171	641	1887	3650
2172	1890	3650	1887
2173	211	2217	2219
2174	643	3655	2217
2175	882	2219	3655
2176	2217	3655	2219
2177	641	3650	3648
2178	882	3655	3650
2179	643	3648	3655
2180	3650	3655	3648
2181	154	1881	1880
2182	878	4013	1881
2183	877	1880	4013
2184	1881	4013	1880
2185	211	2219	2218
2186	882	4015	2219
2187	878	2218	4015
2188	2219	4015	2218
2189	156	1888	1890
2190	877	4014	1888
2191	882	1890	4014
2192	1888	4014	1890
2193	878	4015	4013
2194	882	4014	4015
2195	877	4013	4014
2196	4015	4014	4013
2197	33	1237	1235
2198	437	3061	1237
2199	435	1235	3061
2200	1237	3061	1235
2201	209	2208	2204
2202	1008	3067	2208
2203	437	2204	3067
2204	2208	3067	2204
2205	207	2192	2196
2206	435	3063	2192
2207	1008	2196	3063
2208	2192	3063	2196
2209	437	3067	3061
2210	1008	3063	3067
2211	435	3061	3063
2212	3067	3063	3061
2213	71	1443	1442
2214	643	3652	1443
2215	642	1442	3652
2216	1443	3652	1442
2217	211	2221	2217

2218	1011	3656	2221
2219	643	2217	3656
2220	2221	3656	2217
2221	209	2205	2209
2222	642	3654	2205
2223	1011	2209	3654
2224	2205	3654	2209
2225	643	3656	3652
2226	1011	3654	3656
2227	642	3652	3654
2228	3656	3654	3652
2229	35	1244	1245
2230	444	3081	1244
2231	445	1245	3081
2232	1244	3081	1245
2233	207	2197	2193
2234	1009	3083	2197
2235	444	2193	3083
2236	2197	3083	2193
2237	211	2216	2220
2238	445	3085	2216
2239	1009	2220	3085
2240	2216	3085	2220
2241	444	3083	3081
2242	1009	3085	3083
2243	445	3081	3085
2244	3083	3085	3081
2245	209	2209	2208
2246	1011	4146	2209
2247	1008	2208	4146
2248	2209	4146	2208
2249	211	2220	2221
2250	1009	4147	2220
2251	1011	2221	4147
2252	2220	4147	2221
2253	207	2196	2197
2254	1008	4145	2196
2255	1009	2197	4145
2256	2196	4145	2197
2257	1011	4147	4146
2258	1009	4145	4147
2259	1008	4146	4145
2260	4147	4145	4146
2261	7	1113	1109
2262	313	2731	1113
2263	309	1109	2731
2264	1113	2731	1109
2265	105	1622	1620
2266	747	2742	1622
2267	313	1620	2742
2268	1622	2742	1620
2269	101	1596	1599
2270	309	2733	1596
2271	747	1599	2733
2272	1596	2733	1599
2273	313	2742	2731
2274	747	2733	2742
2275	309	2731	2733
2276	2742	2733	2731

2277	49	1318	1316
2278	518	3284	1318
2279	516	1316	3284
2280	1318	3284	1316
2281	235	2363	2361
2282	758	3293	2363
2283	518	2361	3293
2284	2363	3293	2361
2285	105	1621	1624
2286	516	3286	1621
2287	758	1624	3286
2288	1621	3286	1624
2289	518	3293	3284
2290	758	3286	3293
2291	516	3284	3286
2292	3293	3286	3284
2293	45	1292	1295
2294	492	3213	1292
2295	495	1295	3213
2296	1292	3213	1295
2297	101	1601	1597
2298	749	3215	1601
2299	492	1597	3215
2300	1601	3215	1597
2301	235	2360	2362
2302	495	3224	2360
2303	749	2362	3224
2304	2360	3224	2362
2305	492	3215	3213
2306	749	3224	3215
2307	495	3213	3224
2308	3215	3224	3213
2309	105	1624	1622
2310	758	3887	1624
2311	747	1622	3887
2312	1624	3887	1622
2313	235	2362	2363
2314	749	3889	2362
2315	758	2363	3889
2316	2362	3889	2363
2317	101	1599	1601
2318	747	3886	1599
2319	749	1601	3886
2320	1599	3886	1601
2321	758	3889	3887
2322	749	3886	3889
2323	747	3887	3886
2324	3889	3886	3887
2325	25	1198	1196
2326	398	2960	1198
2327	396	1196	2960
2328	1198	2960	1196
2329	190	2092	2090
2330	976	2969	2092
2331	398	2090	2969
2332	2092	2969	2090
2333	188	2078	2080
2334	396	2962	2078
2335	976	2080	2962

2336	2078	2962	2080
2337	398	2969	2960
2338	976	2962	2969
2339	396	2960	2962
2340	2969	2962	2960
2341	73	1455	1453
2342	655	3684	1455
2343	653	1453	3684
2344	1455	3684	1453
2345	246	2429	2427
2346	985	3691	2429
2347	655	2427	3691
2348	2429	3691	2427
2349	190	2091	2094
2350	653	3686	2091
2351	985	2094	3686
2352	2091	3686	2094
2353	655	3691	3684
2354	985	3686	3691
2355	653	3684	3686
2356	3691	3686	3684
2357	49	1317	1320
2358	517	3288	1317
2359	520	1320	3288
2360	1317	3288	1320
2361	188	2082	2079
2362	978	3290	2082
2363	517	2079	3290
2364	2082	3290	2079
2365	246	2426	2428
2366	520	3298	2426
2367	978	2428	3298
2368	2426	3298	2428
2369	517	3290	3288
2370	978	3298	3290
2371	520	3288	3298
2372	3290	3298	3288
2373	190	2094	2092
2374	985	4113	2094
2375	976	2092	4113
2376	2094	4113	2092
2377	246	2428	2429
2378	978	4116	2428
2379	985	2429	4116
2380	2428	4116	2429
2381	188	2080	2082
2382	976	4112	2080
2383	978	2082	4112
2384	2080	4112	2082
2385	985	4116	4113
2386	978	4112	4116
2387	976	4113	4112
2388	4116	4112	4113
2389	17	1153	1157
2390	353	2839	1153
2391	357	1157	2839
2392	1153	2839	1157
2393	145	1831	1828
2394	855	2841	1831

2395	353	1828	2841
2396	1831	2841	1828
2397	149	1852	1854
2398	357	2850	1852
2399	855	1854	2850
2400	1852	2850	1854
2401	353	2841	2839
2402	855	2850	2841
2403	357	2839	2850
2404	2841	2850	2839
2405	45	1297	1293
2406	497	3217	1297
2407	493	1293	3217
2408	1297	3217	1293
2409	237	2374	2372
2410	857	3228	2374
2411	497	2372	3228
2412	2374	3228	2372
2413	145	1829	1833
2414	493	3219	1829
2415	857	1833	3219
2416	1829	3219	1833
2417	497	3228	3217
2418	857	3219	3228
2419	493	3217	3219
2420	3228	3219	3217
2421	73	1452	1454
2422	652	3680	1452
2423	654	1454	3680
2424	1452	3680	1454
2425	149	1856	1853
2426	866	3682	1856
2427	652	1853	3682
2428	1856	3682	1853
2429	237	2373	2375
2430	654	3689	2373
2431	866	2375	3689
2432	2373	3689	2375
2433	652	3682	3680
2434	866	3689	3682
2435	654	3680	3689
2436	3682	3689	3680
2437	145	1833	1831
2438	857	3994	1833
2439	855	1831	3994
2440	1833	3994	1831
2441	237	2375	2374
2442	866	3997	2375
2443	857	2374	3997
2444	2375	3997	2374
2445	149	1854	1856
2446	855	3995	1854
2447	866	1856	3995
2448	1854	3995	1856
2449	857	3997	3994
2450	866	3995	3997
2451	855	3994	3995
2452	3997	3995	3994
2453	49	1320	1318

2454	520	3292	1320
2455	518	1318	3292
2456	1320	3292	1318
2457	246	2430	2426
2458	1039	3299	2430
2459	520	2426	3299
2460	2430	3299	2426
2461	235	2361	2365
2462	518	3294	2361
2463	1039	2365	3294
2464	2361	3294	2365
2465	520	3299	3292
2466	1039	3294	3299
2467	518	3292	3294
2468	3299	3294	3292
2469	73	1454	1455
2470	654	3688	1454
2471	655	1455	3688
2472	1454	3688	1455
2473	237	2377	2373
2474	1041	3690	2377
2475	654	2373	3690
2476	2377	3690	2373
2477	246	2427	2431
2478	655	3692	2427
2479	1041	2431	3692
2480	2427	3692	2431
2481	654	3690	3688
2482	1041	3692	3690
2483	655	3688	3692
2484	3690	3692	3688
2485	45	1295	1297
2486	495	3223	1295
2487	497	1297	3223
2488	1295	3223	1297
2489	235	2364	2360
2490	1038	3225	2364
2491	495	2360	3225
2492	2364	3225	2360
2493	237	2372	2376
2494	497	3229	2372
2495	1038	2376	3229
2496	2372	3229	2376
2497	495	3225	3223
2498	1038	3229	3225
2499	497	3223	3229
2500	3225	3229	3223
2501	246	2431	2430
2502	1041	4177	2431
2503	1039	2430	4177
2504	2431	4177	2430
2505	237	2376	2377
2506	1038	4176	2376
2507	1041	2377	4176
2508	2376	4176	2377
2509	235	2365	2364
2510	1039	4175	2365
2511	1038	2364	4175
2512	2365	4175	2364

2513	1041	4176	4177
2514	1038	4175	4176
2515	1039	4177	4175
2516	4176	4175	4177
2517	9	1119	1121
2518	319	2755	1119
2519	321	1121	2755
2520	1119	2755	1121
2521	111	1652	1650
2522	771	2756	1652
2523	319	1650	2756
2524	1652	2756	1650
2525	113	1660	1662
2526	321	2760	1660
2527	771	1662	2760
2528	1660	2760	1662
2529	319	2756	2755
2530	771	2760	2756
2531	321	2755	2760
2532	2756	2760	2755
2533	55	1348	1346
2534	548	3365	1348
2535	546	1346	3365
2536	1348	3365	1346
2537	259	2506	2504
2538	772	3370	2506
2539	548	2504	3370
2540	2506	3370	2504
2541	111	1651	1653
2542	546	3366	1651
2543	772	1653	3366
2544	1651	3366	1653
2545	548	3370	3365
2546	772	3366	3370
2547	546	3365	3366
2548	3370	3366	3365
2549	57	1356	1358
2550	556	3392	1356
2551	558	1358	3392
2552	1356	3392	1358
2553	113	1664	1661
2554	776	3394	1664
2555	556	1661	3394
2556	1664	3394	1661
2557	259	2505	2507
2558	558	3401	2505
2559	776	2507	3401
2560	2505	3401	2507
2561	556	3394	3392
2562	776	3401	3394
2563	558	3392	3401
2564	3394	3401	3392
2565	111	1653	1652
2566	772	3908	1653
2567	771	1652	3908
2568	1653	3908	1652
2569	259	2507	2506
2570	776	3910	2507
2571	772	2506	3910

2572	2507	3910	2506
2573	113	1662	1664
2574	771	3909	1662
2575	776	1664	3909
2576	1662	3909	1664
2577	772	3910	3908
2578	776	3909	3910
2579	771	3908	3909
2580	3910	3909	3908
2581	19	1165	1163
2582	365	2863	1165
2583	363	1163	2863
2584	1165	2863	1163
2585	157	1894	1892
2586	879	2868	1894
2587	365	1892	2868
2588	1894	2868	1892
2589	155	1882	1884
2590	363	2864	1882
2591	879	1884	2864
2592	1882	2864	1884
2593	365	2868	2863
2594	879	2864	2868
2595	363	2863	2864
2596	2868	2864	2863
2597	75	1466	1464
2598	666	3716	1466
2599	664	1464	3716
2600	1466	3716	1464
2601	260	2513	2511
2602	884	3725	2513
2603	666	2511	3725
2604	2513	3725	2511
2605	157	1893	1896
2606	664	3718	1893
2607	884	1896	3718
2608	1893	3718	1896
2609	666	3725	3716
2610	884	3718	3725
2611	664	3716	3718
2612	3725	3718	3716
2613	55	1347	1349
2614	547	3367	1347
2615	549	1349	3367
2616	1347	3367	1349
2617	155	1885	1883
2618	880	3368	1885
2619	547	1883	3368
2620	1885	3368	1883
2621	260	2510	2512
2622	549	3372	2510
2623	880	2512	3372
2624	2510	3372	2512
2625	547	3368	3367
2626	880	3372	3368
2627	549	3367	3372
2628	3368	3372	3367
2629	157	1896	1894
2630	884	4017	1896

2631	879	1894	4017
2632	1896	4017	1894
2633	260	2512	2513
2634	880	4018	2512
2635	884	2513	4018
2636	2512	4018	2513
2637	155	1884	1885
2638	879	4016	1884
2639	880	1885	4016
2640	1884	4016	1885
2641	884	4018	4017
2642	880	4016	4018
2643	879	4017	4016
2644	4018	4016	4017
2645	25	1197	1199
2646	397	2964	1197
2647	399	1199	2964
2648	1197	2964	1199
2649	189	2086	2084
2650	980	2966	2086
2651	397	2084	2966
2652	2086	2966	2084
2653	191	2096	2098
2654	399	2971	2096
2655	980	2098	2971
2656	2096	2971	2098
2657	397	2966	2964
2658	980	2971	2966
2659	399	2964	2971
2660	2966	2971	2964
2661	57	1360	1357
2662	560	3396	1360
2663	557	1357	3396
2664	1360	3396	1357
2665	264	2536	2534
2666	982	3406	2536
2667	560	2534	3406
2668	2536	3406	2534
2669	189	2085	2088
2670	557	3398	2085
2671	982	2088	3398
2672	2085	3398	2088
2673	560	3406	3396
2674	982	3398	3406
2675	557	3396	3398
2676	3406	3398	3396
2677	75	1465	1467
2678	665	3720	1465
2679	667	1467	3720
2680	1465	3720	1467
2681	191	2100	2097
2682	987	3722	2100
2683	665	2097	3722
2684	2100	3722	2097
2685	264	2535	2537
2686	667	3727	2535
2687	987	2537	3727
2688	2535	3727	2537
2689	665	3722	3720

2690	987	3727	3722
2691	667	3720	3727
2692	3722	3727	3720
2693	189	2088	2086
2694	982	4118	2088
2695	980	2086	4118
2696	2088	4118	2086
2697	264	2537	2536
2698	987	4122	2537
2699	982	2536	4122
2700	2537	4122	2536
2701	191	2098	2100
2702	980	4119	2098
2703	987	2100	4119
2704	2098	4119	2100
2705	982	4122	4118
2706	987	4119	4122
2707	980	4118	4119
2708	4122	4119	4118
2709	55	1349	1348
2710	549	3369	1349
2711	548	1348	3369
2712	1349	3369	1348
2713	260	2514	2510
2714	1060	3373	2514
2715	549	2510	3373
2716	2514	3373	2510
2717	259	2504	2508
2718	548	3371	2504
2719	1060	2508	3371
2720	2504	3371	2508
2721	549	3373	3369
2722	1060	3371	3373
2723	548	3369	3371
2724	3373	3371	3369
2725	75	1467	1466
2726	667	3724	1467
2727	666	1466	3724
2728	1467	3724	1466
2729	264	2539	2535
2730	1062	3728	2539
2731	667	2535	3728
2732	2539	3728	2535
2733	260	2511	2515
2734	666	3726	2511
2735	1062	2515	3726
2736	2511	3726	2515
2737	667	3728	3724
2738	1062	3726	3728
2739	666	3724	3726
2740	3728	3726	3724
2741	57	1358	1360
2742	558	3400	1358
2743	560	1360	3400
2744	1358	3400	1360
2745	259	2509	2505
2746	1061	3402	2509
2747	558	2505	3402
2748	2509	3402	2505

2749	264	2534	2538
2750	560	3407	2534
2751	1061	2538	3407
2752	2534	3407	2538
2753	558	3402	3400
2754	1061	3407	3402
2755	560	3400	3407
2756	3402	3407	3400
2757	260	2515	2514
2758	1062	4197	2515
2759	1060	2514	4197
2760	2515	4197	2514
2761	264	2538	2539
2762	1061	4198	2538
2763	1062	2539	4198
2764	2538	4198	2539
2765	259	2508	2509
2766	1060	4196	2508
2767	1061	2509	4196
2768	2508	4196	2509
2769	1062	4198	4197
2770	1061	4196	4198
2771	1060	4197	4196
2772	4198	4196	4197
2773	17	1157	1155
2774	357	2845	1157
2775	355	1155	2845
2776	1157	2845	1155
2777	149	1855	1852
2778	861	2851	1855
2779	357	1852	2851
2780	1855	2851	1852
2781	147	1840	1843
2782	355	2847	1840
2783	861	1843	2847
2784	1840	2847	1843
2785	357	2851	2845
2786	861	2847	2851
2787	355	2845	2847
2788	2851	2847	2845
2789	73	1456	1452
2790	656	3681	1456
2791	652	1452	3681
2792	1456	3681	1452
2793	280	2633	2631
2794	867	3694	2633
2795	656	2631	3694
2796	2633	3694	2631
2797	149	1853	1857
2798	652	3683	1853
2799	867	1857	3683
2800	1853	3683	1857
2801	656	3694	3681
2802	867	3683	3694
2803	652	3681	3683
2804	3694	3683	3681
2805	71	1440	1444
2806	640	3645	1440
2807	644	1444	3645

2808	1440	3645	1444
2809	147	1845	1841
2810	863	3647	1845
2811	640	1841	3647
2812	1845	3647	1841
2813	280	2630	2632
2814	644	3658	2630
2815	863	2632	3658
2816	2630	3658	2632
2817	640	3647	3645
2818	863	3658	3647
2819	644	3645	3658
2820	3647	3658	3645
2821	149	1857	1855
2822	867	4002	1857
2823	861	1855	4002
2824	1857	4002	1855
2825	280	2632	2633
2826	863	4003	2632
2827	867	2633	4003
2828	2632	4003	2633
2829	147	1843	1845
2830	861	4001	1843
2831	863	1845	4001
2832	1843	4001	1845
2833	867	4003	4002
2834	863	4001	4003
2835	861	4002	4001
2836	4003	4001	4002
2837	25	1199	1198
2838	399	2968	1199
2839	398	1198	2968
2840	1199	2968	1198
2841	191	2099	2096
2842	984	2972	2099
2843	399	2096	2972
2844	2099	2972	2096
2845	190	2090	2093
2846	398	2970	2090
2847	984	2093	2970
2848	2090	2970	2093
2849	399	2972	2968
2850	984	2970	2972
2851	398	2968	2970
2852	2972	2970	2968
2853	75	1469	1465
2854	669	3721	1469
2855	665	1465	3721
2856	1469	3721	1465
2857	283	2651	2649
2858	988	3732	2651
2859	669	2649	3732
2860	2651	3732	2649
2861	191	2097	2101
2862	665	3723	2097
2863	988	2101	3723
2864	2097	3723	2101
2865	669	3732	3721
2866	988	3723	3732

2867	665	3721	3723
2868	3732	3723	3721
2869	73	1453	1457
2870	653	3685	1453
2871	657	1457	3685
2872	1453	3685	1457
2873	190	2095	2091
2874	986	3687	2095
2875	653	2091	3687
2876	2095	3687	2091
2877	283	2648	2650
2878	657	3696	2648
2879	986	2650	3696
2880	2648	3696	2650
2881	653	3687	3685
2882	986	3696	3687
2883	657	3685	3696
2884	3687	3696	3685
2885	191	2101	2099
2886	988	4125	2101
2887	984	2099	4125
2888	2101	4125	2099
2889	283	2650	2651
2890	986	4126	2650
2891	988	2651	4126
2892	2650	4126	2651
2893	190	2093	2095
2894	984	4124	2093
2895	986	2095	4124
2896	2093	4124	2095
2897	988	4126	4125
2898	986	4124	4126
2899	984	4125	4124
2900	4126	4124	4125
2901	19	1164	1165
2902	364	2865	1164
2903	365	1165	2865
2904	1164	2865	1165
2905	156	1889	1886
2906	881	2867	1889
2907	364	1886	2867
2908	1889	2867	1886
2909	157	1892	1895
2910	365	2869	1892
2911	881	1895	2869
2912	1892	2869	1895
2913	364	2867	2865
2914	881	2869	2867
2915	365	2865	2869
2916	2867	2869	2865
2917	71	1445	1441
2918	645	3649	1445
2919	641	1441	3649
2920	1445	3649	1441
2921	281	2638	2636
2922	883	3660	2638
2923	645	2636	3660
2924	2638	3660	2636
2925	156	1887	1891

2926	641	3651	1887
2927	883	1891	3651
2928	1887	3651	1891
2929	645	3660	3649
2930	883	3651	3660
2931	641	3649	3651
2932	3660	3651	3649
2933	75	1464	1468
2934	664	3717	1464
2935	668	1468	3717
2936	1464	3717	1468
2937	157	1897	1893
2938	885	3719	1897
2939	664	1893	3719
2940	1897	3719	1893
2941	281	2637	2639
2942	668	3730	2637
2943	885	2639	3730
2944	2637	3730	2639
2945	664	3719	3717
2946	885	3730	3719
2947	668	3717	3730
2948	3719	3730	3717
2949	156	1891	1889
2950	883	4019	1891
2951	881	1889	4019
2952	1891	4019	1889
2953	281	2639	2638
2954	885	4021	2639
2955	883	2638	4021
2956	2639	4021	2638
2957	157	1895	1897
2958	881	4020	1895
2959	885	1897	4020
2960	1895	4020	1897
2961	883	4021	4019
2962	885	4020	4021
2963	881	4019	4020
2964	4021	4020	4019
2965	73	1457	1456
2966	657	3693	1457
2967	656	1456	3693
2968	1457	3693	1456
2969	283	2652	2648
2970	1082	3697	2652
2971	657	2648	3697
2972	2652	3697	2648
2973	280	2631	2635
2974	656	3695	2631
2975	1082	2635	3695
2976	2631	3695	2635
2977	657	3697	3693
2978	1082	3695	3697
2979	656	3693	3695
2980	3697	3695	3693
2981	75	1468	1469
2982	668	3729	1468
2983	669	1469	3729
2984	1468	3729	1469

2985	281	2641	2637
2986	1083	3731	2641
2987	668	2637	3731
2988	2641	3731	2637
2989	283	2649	2653
2990	669	3733	2649
2991	1083	2653	3733
2992	2649	3733	2653
2993	668	3731	3729
2994	1083	3733	3731
2995	669	3729	3733
2996	3731	3733	3729
2997	71	1444	1445
2998	644	3657	1444
2999	645	1445	3657
3000	1444	3657	1445
3001	280	2634	2630
3002	1081	3659	2634
3003	644	2630	3659
3004	2634	3659	2630
3005	281	2636	2640
3006	645	3661	2636
3007	1081	2640	3661
3008	2636	3661	2640
3009	644	3659	3657
3010	1081	3661	3659
3011	645	3657	3661
3012	3659	3661	3657
3013	283	2653	2652
3014	1083	4219	2653
3015	1082	2652	4219
3016	2653	4219	2652
3017	281	2640	2641
3018	1081	4218	2640
3019	1083	2641	4218
3020	2640	4218	2641
3021	280	2635	2634
3022	1082	4217	2635
3023	1081	2634	4217
3024	2635	4217	2634
3025	1083	4218	4219
3026	1081	4217	4218
3027	1082	4219	4217
3028	4218	4217	4219
3029	6	1107	1106
3030	307	2721	1107
3031	306	1106	2721
3032	1107	2721	1106
3033	99	1587	1584
3034	737	2725	1587
3035	307	1584	2725
3036	1587	2725	1584
3037	98	1578	1581
3038	306	2723	1578
3039	737	1581	2723
3040	1578	2723	1581
3041	307	2725	2721
3042	737	2723	2725
3043	306	2721	2723

3044	2725	2723	2721
3045	43	1283	1280
3046	483	3177	1283
3047	480	1280	3177
3048	1283	3177	1280
3049	225	2303	2301
3050	741	3188	2303
3051	483	2301	3188
3052	2303	3188	2301
3053	99	1585	1589
3054	480	3179	1585
3055	741	1589	3179
3056	1585	3179	1589
3057	483	3188	3177
3058	741	3179	3188
3059	480	3177	3179
3060	3188	3179	3177
3061	42	1274	1277
3062	474	3159	1274
3063	477	1277	3159
3064	1274	3159	1277
3065	98	1583	1579
3066	739	3161	1583
3067	474	1579	3161
3068	1583	3161	1579
3069	225	2300	2302
3070	477	3170	2300
3071	739	2302	3170
3072	2300	3170	2302
3073	474	3161	3159
3074	739	3170	3161
3075	477	3159	3170
3076	3161	3170	3159
3077	99	1589	1587
3078	741	3876	1589
3079	737	1587	3876
3080	1589	3876	1587
3081	225	2302	2303
3082	739	3877	2302
3083	741	2303	3877
3084	2302	3877	2303
3085	98	1581	1583
3086	737	3875	1581
3087	739	1583	3875
3088	1581	3875	1583
3089	741	3877	3876
3090	739	3875	3877
3091	737	3876	3875
3092	3877	3875	3876
3093	23	1188	1184
3094	388	2925	1188
3095	384	1184	2925
3096	1188	2925	1184
3097	180	2032	2030
3098	941	2938	2032
3099	388	2030	2938
3100	2032	2938	2030
3101	176	2006	2009
3102	384	2927	2006

3103	941	2009	2927
3104	2006	2927	2009
3105	388	2938	2925
3106	941	2927	2938
3107	384	2925	2927
3108	2938	2927	2925
3109	79	1491	1489
3110	691	3792	1491
3111	689	1489	3792
3112	1491	3792	1489
3113	229	2327	2325
3114	954	3799	2327
3115	691	2325	3799
3116	2327	3799	2325
3117	180	2031	2034
3118	689	3794	2031
3119	954	2034	3794
3120	2031	3794	2034
3121	691	3799	3792
3122	954	3794	3799
3123	689	3792	3794
3124	3799	3794	3792
3125	43	1281	1285
3126	481	3181	1281
3127	485	1285	3181
3128	1281	3181	1285
3129	176	2011	2007
3130	943	3183	2011
3131	481	2007	3183
3132	2011	3183	2007
3133	229	2324	2326
3134	485	3192	2324
3135	943	2326	3192
3136	2324	3192	2326
3137	481	3183	3181
3138	943	3192	3183
3139	485	3181	3192
3140	3183	3192	3181
3141	180	2034	2032
3142	954	4079	2034
3143	941	2032	4079
3144	2034	4079	2032
3145	229	2326	2327
3146	943	4081	2326
3147	954	2327	4081
3148	2326	4081	2327
3149	176	2009	2011
3150	941	4078	2009
3151	943	2011	4078
3152	2009	4078	2011
3153	954	4081	4079
3154	943	4078	4081
3155	941	4079	4078
3156	4081	4078	4079
3157	22	1178	1182
3158	378	2907	1178
3159	382	1182	2907
3160	1178	2907	1182
3161	170	1973	1970

3162	923	2909	1973
3163	378	1970	2909
3164	1973	2909	1970
3165	174	1994	1996
3166	382	2920	1994
3167	923	1996	2920
3168	1994	2920	1996
3169	378	2909	2907
3170	923	2920	2909
3171	382	2907	2920
3172	2909	2920	2907
3173	42	1279	1275
3174	479	3163	1279
3175	475	1275	3163
3176	1279	3163	1275
3177	227	2314	2312
3178	925	3174	2314
3179	479	2312	3174
3180	2314	3174	2312
3181	170	1971	1975
3182	475	3165	1971
3183	925	1975	3165
3184	1971	3165	1975
3185	479	3174	3163
3186	925	3165	3174
3187	475	3163	3165
3188	3174	3165	3163
3189	79	1488	1490
3190	688	3788	1488
3191	690	1490	3788
3192	1488	3788	1490
3193	174	1998	1995
3194	936	3790	1998
3195	688	1995	3790
3196	1998	3790	1995
3197	227	2313	2315
3198	690	3797	2313
3199	936	2315	3797
3200	2313	3797	2315
3201	688	3790	3788
3202	936	3797	3790
3203	690	3788	3797
3204	3790	3797	3788
3205	170	1975	1973
3206	925	4060	1975
3207	923	1973	4060
3208	1975	4060	1973
3209	227	2315	2314
3210	936	4063	2315
3211	925	2314	4063
3212	2315	4063	2314
3213	174	1996	1998
3214	923	4061	1996
3215	936	1998	4061
3216	1996	4061	1998
3217	925	4063	4060
3218	936	4061	4063
3219	923	4060	4061
3220	4063	4061	4060

3221	43	1285	1283
3222	485	3187	1285
3223	483	1283	3187
3224	1285	3187	1283
3225	229	2328	2324
3226	1028	3193	2328
3227	485	2324	3193
3228	2328	3193	2324
3229	225	2301	2305
3230	483	3189	2301
3231	1028	2305	3189
3232	2301	3189	2305
3233	485	3193	3187
3234	1028	3189	3193
3235	483	3187	3189
3236	3193	3189	3187
3237	79	1490	1491
3238	690	3796	1490
3239	691	1491	3796
3240	1490	3796	1491
3241	227	2317	2313
3242	1029	3798	2317
3243	690	2313	3798
3244	2317	3798	2313
3245	229	2325	2329
3246	691	3800	2325
3247	1029	2329	3800
3248	2325	3800	2329
3249	690	3798	3796
3250	1029	3800	3798
3251	691	3796	3800
3252	3798	3800	3796
3253	42	1277	1279
3254	477	3169	1277
3255	479	1279	3169
3256	1277	3169	1279
3257	225	2304	2300
3258	1027	3171	2304
3259	477	2300	3171
3260	2304	3171	2300
3261	227	2312	2316
3262	479	3175	2312
3263	1027	2316	3175
3264	2312	3175	2316
3265	477	3171	3169
3266	1027	3175	3171
3267	479	3169	3175
3268	3171	3175	3169
3269	229	2329	2328
3270	1029	4165	2329
3271	1028	2328	4165
3272	2329	4165	2328
3273	227	2316	2317
3274	1027	4164	2316
3275	1029	2317	4164
3276	2316	4164	2317
3277	225	2305	2304
3278	1028	4163	2305
3279	1027	2304	4163

3280	2305	4163	2304
3281	1029	4164	4165
3282	1027	4163	4164
3283	1028	4165	4163
3284	4164	4163	4165
3285	9	1121	1120
3286	321	2757	1121
3287	320	1120	2757
3288	1121	2757	1120
3289	113	1663	1660
3290	773	2761	1663
3291	321	1660	2761
3292	1663	2761	1660
3293	112	1654	1657
3294	320	2759	1654
3295	773	1657	2759
3296	1654	2759	1657
3297	321	2761	2757
3298	773	2759	2761
3299	320	2757	2759
3300	2761	2759	2757
3301	57	1359	1356
3302	559	3393	1359
3303	556	1356	3393
3304	1359	3393	1356
3305	261	2519	2517
3306	777	3404	2519
3307	559	2517	3404
3308	2519	3404	2517
3309	113	1661	1665
3310	556	3395	1661
3311	777	1665	3395
3312	1661	3395	1665
3313	559	3404	3393
3314	777	3395	3404
3315	556	3393	3395
3316	3404	3395	3393
3317	56	1350	1353
3318	550	3375	1350
3319	553	1353	3375
3320	1350	3375	1353
3321	112	1659	1655
3322	775	3377	1659
3323	550	1655	3377
3324	1659	3377	1655
3325	261	2516	2518
3326	553	3386	2516
3327	775	2518	3386
3328	2516	3386	2518
3329	550	3377	3375
3330	775	3386	3377
3331	553	3375	3386
3332	3377	3386	3375
3333	113	1665	1663
3334	777	3912	1665
3335	773	1663	3912
3336	1665	3912	1663
3337	261	2518	2519
3338	775	3913	2518

3339	777	2519	3913
3340	2518	3913	2519
3341	112	1657	1659
3342	773	3911	1657
3343	775	1659	3911
3344	1657	3911	1659
3345	777	3913	3912
3346	775	3911	3913
3347	773	3912	3911
3348	3913	3911	3912
3349	25	1201	1197
3350	401	2965	1201
3351	397	1197	2965
3352	1201	2965	1197
3353	193	2110	2108
3354	981	2976	2110
3355	401	2108	2976
3356	2110	2976	2108
3357	189	2084	2087
3358	397	2967	2084
3359	981	2087	2967
3360	2084	2967	2087
3361	401	2976	2965
3362	981	2967	2976
3363	397	2965	2967
3364	2976	2967	2965
3365	81	1503	1501
3366	703	3828	1503
3367	701	1501	3828
3368	1503	3828	1501
3369	265	2543	2541
3370	992	3835	2543
3371	703	2541	3835
3372	2543	3835	2541
3373	193	2109	2112
3374	701	3830	2109
3375	992	2112	3830
3376	2109	3830	2112
3377	703	3835	3828
3378	992	3830	3835
3379	701	3828	3830
3380	3835	3830	3828
3381	57	1357	1361
3382	557	3397	1357
3383	561	1361	3397
3384	1357	3397	1361
3385	189	2089	2085
3386	983	3399	2089
3387	557	2085	3399
3388	2089	3399	2085
3389	265	2540	2542
3390	561	3408	2540
3391	983	2542	3408
3392	2540	3408	2542
3393	557	3399	3397
3394	983	3408	3399
3395	561	3397	3408
3396	3399	3408	3397
3397	193	2112	2110

3398	992	4121	2112
3399	981	2110	4121
3400	2112	4121	2110
3401	265	2542	2543
3402	983	4123	2542
3403	992	2543	4123
3404	2542	4123	2543
3405	189	2087	2089
3406	981	4120	2087
3407	983	2089	4120
3408	2087	4120	2089
3409	992	4123	4121
3410	983	4120	4123
3411	981	4121	4120
3412	4123	4120	4121
3413	23	1185	1189
3414	385	2929	1185
3415	389	1189	2929
3416	1185	2929	1189
3417	177	2015	2012
3418	945	2931	2015
3419	385	2012	2931
3420	2015	2931	2012
3421	181	2036	2038
3422	389	2940	2036
3423	945	2038	2940
3424	2036	2940	2038
3425	385	2931	2929
3426	945	2940	2931
3427	389	2929	2940
3428	2931	2940	2929
3429	56	1355	1351
3430	555	3379	1355
3431	551	1351	3379
3432	1355	3379	1351
3433	263	2530	2528
3434	947	3390	2530
3435	555	2528	3390
3436	2530	3390	2528
3437	177	2013	2017
3438	551	3381	2013
3439	947	2017	3381
3440	2013	3381	2017
3441	555	3390	3379
3442	947	3381	3390
3443	551	3379	3381
3444	3390	3381	3379
3445	81	1500	1502
3446	700	3824	1500
3447	702	1502	3824
3448	1500	3824	1502
3449	181	2040	2037
3450	956	3826	2040
3451	700	2037	3826
3452	2040	3826	2037
3453	263	2529	2531
3454	702	3833	2529
3455	956	2531	3833
3456	2529	3833	2531

3457	700	3826	3824
3458	956	3833	3826
3459	702	3824	3833
3460	3826	3833	3824
3461	177	2017	2015
3462	947	4084	2017
3463	945	2015	4084
3464	2017	4084	2015
3465	263	2531	2530
3466	956	4087	2531
3467	947	2530	4087
3468	2531	4087	2530
3469	181	2038	2040
3470	945	4085	2038
3471	956	2040	4085
3472	2038	4085	2040
3473	947	4087	4084
3474	956	4085	4087
3475	945	4084	4085
3476	4087	4085	4084
3477	57	1361	1359
3478	561	3403	1361
3479	559	1359	3403
3480	1361	3403	1359
3481	265	2544	2540
3482	1064	3409	2544
3483	561	2540	3409
3484	2544	3409	2540
3485	261	2517	2521
3486	559	3405	2517
3487	1064	2521	3405
3488	2517	3405	2521
3489	561	3409	3403
3490	1064	3405	3409
3491	559	3403	3405
3492	3409	3405	3403
3493	81	1502	1503
3494	702	3832	1502
3495	703	1503	3832
3496	1502	3832	1503
3497	263	2533	2529
3498	1065	3834	2533
3499	702	2529	3834
3500	2533	3834	2529
3501	265	2541	2545
3502	703	3836	2541
3503	1065	2545	3836
3504	2541	3836	2545
3505	702	3834	3832
3506	1065	3836	3834
3507	703	3832	3836
3508	3834	3836	3832
3509	56	1353	1355
3510	553	3385	1353
3511	555	1355	3385
3512	1353	3385	1355
3513	261	2520	2516
3514	1063	3387	2520
3515	553	2516	3387

3516	2520	3387	2516
3517	263	2528	2532
3518	555	3391	2528
3519	1063	2532	3391
3520	2528	3391	2532
3521	553	3387	3385
3522	1063	3391	3387
3523	555	3385	3391
3524	3387	3391	3385
3525	265	2545	2544
3526	1065	4201	2545
3527	1064	2544	4201
3528	2545	4201	2544
3529	263	2532	2533
3530	1063	4200	2532
3531	1065	2533	4200
3532	2532	4200	2533
3533	261	2521	2520
3534	1064	4199	2521
3535	1063	2520	4199
3536	2521	4199	2520
3537	1065	4200	4201
3538	1063	4199	4200
3539	1064	4201	4199
3540	4200	4199	4201
3541	7	1111	1113
3542	311	2737	1111
3543	313	1113	2737
3544	1111	2737	1113
3545	103	1611	1608
3546	753	2739	1611
3547	311	1608	2739
3548	1611	2739	1608
3549	105	1620	1623
3550	313	2743	1620
3551	753	1623	2743
3552	1620	2743	1623
3553	311	2739	2737
3554	753	2743	2739
3555	313	2737	2743
3556	2739	2743	2737
3557	47	1307	1304
3558	507	3249	1307
3559	504	1304	3249
3560	1307	3249	1304
3561	241	2398	2396
3562	755	3260	2398
3563	507	2396	3260
3564	2398	3260	2396
3565	103	1609	1613
3566	504	3251	1609
3567	755	1613	3251
3568	1609	3251	1613
3569	507	3260	3249
3570	755	3251	3260
3571	504	3249	3251
3572	3260	3251	3249
3573	49	1316	1319
3574	516	3285	1316

3575	519	1319	3285
3576	1316	3285	1319
3577	105	1625	1621
3578	759	3287	1625
3579	516	1621	3287
3580	1625	3287	1621
3581	241	2397	2399
3582	519	3296	2397
3583	759	2399	3296
3584	2397	3296	2399
3585	516	3287	3285
3586	759	3296	3287
3587	519	3285	3296
3588	3287	3296	3285
3589	103	1613	1611
3590	755	3893	1613
3591	753	1611	3893
3592	1613	3893	1611
3593	241	2399	2398
3594	759	3895	2399
3595	755	2398	3895
3596	2399	3895	2398
3597	105	1623	1625
3598	753	3894	1623
3599	759	1625	3894
3600	1623	3894	1625
3601	755	3895	3893
3602	759	3894	3895
3603	753	3893	3894
3604	3895	3894	3893
3605	22	1183	1179
3606	383	2911	1183
3607	379	1179	2911
3608	1183	2911	1179
3609	175	2002	2000
3610	927	2922	2002
3611	383	2000	2922
3612	2002	2922	2000
3613	171	1976	1979
3614	379	2913	1976
3615	927	1979	2913
3616	1976	2913	1979
3617	383	2922	2911
3618	927	2913	2922
3619	379	2911	2913
3620	2922	2913	2911
3621	80	1496	1494
3622	696	3806	1496
3623	694	1494	3806
3624	1496	3806	1494
3625	243	2411	2409
3626	938	3815	2411
3627	696	2409	3815
3628	2411	3815	2409
3629	175	2001	2004
3630	694	3808	2001
3631	938	2004	3808
3632	2001	3808	2004
3633	696	3815	3806

3634	938	3808	3815
3635	694	3806	3808
3636	3815	3808	3806
3637	47	1305	1309
3638	505	3253	1305
3639	509	1309	3253
3640	1305	3253	1309
3641	171	1981	1977
3642	929	3255	1981
3643	505	1977	3255
3644	1981	3255	1977
3645	243	2408	2410
3646	509	3264	2408
3647	929	2410	3264
3648	2408	3264	2410
3649	505	3255	3253
3650	929	3264	3255
3651	509	3253	3264
3652	3255	3264	3253
3653	175	2004	2002
3654	938	4067	2004
3655	927	2002	4067
3656	2004	4067	2002
3657	243	2410	2411
3658	929	4069	2410
3659	938	2411	4069
3660	2410	4069	2411
3661	171	1979	1981
3662	927	4066	1979
3663	929	1981	4066
3664	1979	4066	1981
3665	938	4069	4067
3666	929	4066	4069
3667	927	4067	4066
3668	4069	4066	4067
3669	25	1196	1200
3670	396	2961	1196
3671	400	1200	2961
3672	1196	2961	1200
3673	188	2081	2078
3674	977	2963	2081
3675	396	2078	2963
3676	2081	2963	2078
3677	192	2102	2104
3678	400	2974	2102
3679	977	2104	2974
3680	2102	2974	2104
3681	396	2963	2961
3682	977	2974	2963
3683	400	2961	2974
3684	2963	2974	2961
3685	49	1321	1317
3686	521	3289	1321
3687	517	1317	3289
3688	1321	3289	1317
3689	247	2434	2432
3690	979	3300	2434
3691	521	2432	3300
3692	2434	3300	2432

3693	188	2079	2083
3694	517	3291	2079
3695	979	2083	3291
3696	2079	3291	2083
3697	521	3300	3289
3698	979	3291	3300
3699	517	3289	3291
3700	3300	3291	3289
3701	80	1495	1497
3702	695	3810	1495
3703	697	1497	3810
3704	1495	3810	1497
3705	192	2106	2103
3706	990	3812	2106
3707	695	2103	3812
3708	2106	3812	2103
3709	247	2433	2435
3710	697	3817	2433
3711	990	2435	3817
3712	2433	3817	2435
3713	695	3812	3810
3714	990	3817	3812
3715	697	3810	3817
3716	3812	3817	3810
3717	188	2083	2081
3718	979	4114	2083
3719	977	2081	4114
3720	2083	4114	2081
3721	247	2435	2434
3722	990	4117	2435
3723	979	2434	4117
3724	2435	4117	2434
3725	192	2104	2106
3726	977	4115	2104
3727	990	2106	4115
3728	2104	4115	2106
3729	979	4117	4114
3730	990	4115	4117
3731	977	4114	4115
3732	4117	4115	4114
3733	47	1309	1307
3734	509	3259	1309
3735	507	1307	3259
3736	1309	3259	1307
3737	243	2412	2408
3738	1045	3265	2412
3739	509	2408	3265
3740	2412	3265	2408
3741	241	2396	2400
3742	507	3261	2396
3743	1045	2400	3261
3744	2396	3261	2400
3745	509	3265	3259
3746	1045	3261	3265
3747	507	3259	3261
3748	3265	3261	3259
3749	80	1497	1496
3750	697	3814	1497
3751	696	1496	3814

3752	1497	3814	1496
3753	247	2437	2433
3754	1047	3818	2437
3755	697	2433	3818
3756	2437	3818	2433
3757	243	2409	2413
3758	696	3816	2409
3759	1047	2413	3816
3760	2409	3816	2413
3761	697	3818	3814
3762	1047	3816	3818
3763	696	3814	3816
3764	3818	3816	3814
3765	49	1319	1321
3766	519	3295	1319
3767	521	1321	3295
3768	1319	3295	1321
3769	241	2401	2397
3770	1046	3297	2401
3771	519	2397	3297
3772	2401	3297	2397
3773	247	2432	2436
3774	521	3301	2432
3775	1046	2436	3301
3776	2432	3301	2436
3777	519	3297	3295
3778	1046	3301	3297
3779	521	3295	3301
3780	3297	3301	3295
3781	243	2413	2412
3782	1047	4182	2413
3783	1045	2412	4182
3784	2413	4182	2412
3785	247	2436	2437
3786	1046	4183	2436
3787	1047	2437	4183
3788	2436	4183	2437
3789	241	2400	2401
3790	1045	4181	2400
3791	1046	2401	4181
3792	2400	4181	2401
3793	1047	4183	4182
3794	1046	4181	4183
3795	1045	4182	4181
3796	4183	4181	4182
3797	23	1189	1188
3798	389	2937	1189
3799	388	1188	2937
3800	1189	2937	1188
3801	181	2039	2036
3802	953	2941	2039
3803	389	2036	2941
3804	2039	2941	2036
3805	180	2030	2033
3806	388	2939	2030
3807	953	2033	2939
3808	2030	2939	2033
3809	389	2941	2937
3810	953	2939	2941

3811	388	2937	2939
3812	2941	2939	2937
3813	81	1504	1500
3814	704	3825	1504
3815	700	1500	3825
3816	1504	3825	1500
3817	288	2681	2679
3818	957	3838	2681
3819	704	2679	3838
3820	2681	3838	2679
3821	181	2037	2041
3822	700	3827	2037
3823	957	2041	3827
3824	2037	3827	2041
3825	704	3838	3825
3826	957	3827	3838
3827	700	3825	3827
3828	3838	3827	3825
3829	79	1489	1493
3830	689	3793	1489
3831	693	1493	3793
3832	1489	3793	1493
3833	180	2035	2031
3834	955	3795	2035
3835	689	2031	3795
3836	2035	3795	2031
3837	288	2678	2680
3838	693	3804	2678
3839	955	2680	3804
3840	2678	3804	2680
3841	689	3795	3793
3842	955	3804	3795
3843	693	3793	3804
3844	3795	3804	3793
3845	181	2041	2039
3846	957	4092	2041
3847	953	2039	4092
3848	2041	4092	2039
3849	288	2680	2681
3850	955	4093	2680
3851	957	2681	4093
3852	2680	4093	2681
3853	180	2033	2035
3854	953	4091	2033
3855	955	2035	4091
3856	2033	4091	2035
3857	957	4093	4092
3858	955	4091	4093
3859	953	4092	4091
3860	4093	4091	4092
3861	25	1200	1201
3862	400	2973	1200
3863	401	1201	2973
3864	1200	2973	1201
3865	192	2105	2102
3866	989	2975	2105
3867	400	2102	2975
3868	2105	2975	2102
3869	193	2108	2111

3870	401	2977	2108
3871	989	2111	2977
3872	2108	2977	2111
3873	400	2975	2973
3874	989	2977	2975
3875	401	2973	2977
3876	2975	2977	2973
3877	80	1499	1495
3878	699	3811	1499
3879	695	1495	3811
3880	1499	3811	1495
3881	289	2686	2684
3882	991	3822	2686
3883	699	2684	3822
3884	2686	3822	2684
3885	192	2103	2107
3886	695	3813	2103
3887	991	2107	3813
3888	2103	3813	2107
3889	699	3822	3811
3890	991	3813	3822
3891	695	3811	3813
3892	3822	3813	3811
3893	81	1501	1505
3894	701	3829	1501
3895	705	1505	3829
3896	1501	3829	1505
3897	193	2113	2109
3898	993	3831	2113
3899	701	2109	3831
3900	2113	3831	2109
3901	289	2685	2687
3902	705	3840	2685
3903	993	2687	3840
3904	2685	3840	2687
3905	701	3831	3829
3906	993	3840	3831
3907	705	3829	3840
3908	3831	3840	3829
3909	192	2107	2105
3910	991	4127	2107
3911	989	2105	4127
3912	2107	4127	2105
3913	289	2687	2686
3914	993	4129	2687
3915	991	2686	4129
3916	2687	4129	2686
3917	193	2111	2113
3918	989	4128	2111
3919	993	2113	4128
3920	2111	4128	2113
3921	991	4129	4127
3922	993	4128	4129
3923	989	4127	4128
3924	4129	4128	4127
3925	22	1182	1183
3926	382	2919	1182
3927	383	1183	2919
3928	1182	2919	1183

3929	174	1997	1994
3930	935	2921	1997
3931	382	1994	2921
3932	1997	2921	1994
3933	175	2000	2003
3934	383	2923	2000
3935	935	2003	2923
3936	2000	2923	2003
3937	382	2921	2919
3938	935	2923	2921
3939	383	2919	2923
3940	2921	2923	2919
3941	79	1492	1488
3942	692	3789	1492
3943	688	1488	3789
3944	1492	3789	1488
3945	287	2674	2672
3946	937	3802	2674
3947	692	2672	3802
3948	2674	3802	2672
3949	174	1995	1999
3950	688	3791	1995
3951	937	1999	3791
3952	1995	3791	1999
3953	692	3802	3789
3954	937	3791	3802
3955	688	3789	3791
3956	3802	3791	3789
3957	80	1494	1498
3958	694	3807	1494
3959	698	1498	3807
3960	1494	3807	1498
3961	175	2005	2001
3962	939	3809	2005
3963	694	2001	3809
3964	2005	3809	2001
3965	287	2673	2675
3966	698	3820	2673
3967	939	2675	3820
3968	2673	3820	2675
3969	694	3809	3807
3970	939	3820	3809
3971	698	3807	3820
3972	3809	3820	3807
3973	174	1999	1997
3974	937	4073	1999
3975	935	1997	4073
3976	1999	4073	1997
3977	287	2675	2674
3978	939	4075	2675
3979	937	2674	4075
3980	2675	4075	2674
3981	175	2003	2005
3982	935	4074	2003
3983	939	2005	4074
3984	2003	4074	2005
3985	937	4075	4073
3986	939	4074	4075
3987	935	4073	4074

3988	4075	4074	4073
3989	81	1505	1504
3990	705	3837	1505
3991	704	1504	3837
3992	1505	3837	1504
3993	289	2689	2685
3994	1089	3841	2689
3995	705	2685	3841
3996	2689	3841	2685
3997	288	2679	2683
3998	704	3839	2679
3999	1089	2683	3839
4000	2679	3839	2683
4001	705	3841	3837
4002	1089	3839	3841
4003	704	3837	3839
4004	3841	3839	3837
4005	80	1498	1499
4006	698	3819	1498
4007	699	1499	3819
4008	1498	3819	1499
4009	287	2677	2673
4010	1088	3821	2677
4011	698	2673	3821
4012	2677	3821	2673
4013	289	2684	2688
4014	699	3823	2684
4015	1088	2688	3823
4016	2684	3823	2688
4017	698	3821	3819
4018	1088	3823	3821
4019	699	3819	3823
4020	3821	3823	3819
4021	79	1493	1492
4022	693	3801	1493
4023	692	1492	3801
4024	1493	3801	1492
4025	288	2682	2678
4026	1087	3805	2682
4027	693	2678	3805
4028	2682	3805	2678
4029	287	2672	2676
4030	692	3803	2672
4031	1087	2676	3803
4032	2672	3803	2676
4033	693	3805	3801
4034	1087	3803	3805
4035	692	3801	3803
4036	3805	3803	3801
4037	289	2688	2689
4038	1088	4225	2688
4039	1089	2689	4225
4040	2688	4225	2689
4041	287	2676	2677
4042	1087	4223	2676
4043	1088	2677	4223
4044	2676	4223	2677
4045	288	2683	2682
4046	1089	4224	2683

4047	1087	2682	4224
4048	2683	4224	2682
4049	1088	4223	4225
4050	1087	4224	4223
4051	1089	4225	4224
4052	4223	4224	4225
4053	1	1092	1090
4054	292	2690	1092
4055	290	1090	2690
4056	1092	2690	1090
4057	84	1516	1514
4058	706	2694	1516
4059	292	1514	2694
4060	1516	2694	1514
4061	82	1506	1508
4062	290	2691	1506
4063	706	1508	2691
4064	1506	2691	1508
4065	292	2694	2690
4066	706	2691	2694
4067	290	2690	2691
4068	2694	2691	2690
4069	28	1212	1210
4070	412	2996	1212
4071	410	1210	2996
4072	1212	2996	1210
4073	194	2117	2115
4074	710	3005	2117
4075	412	2115	3005
4076	2117	3005	2115
4077	84	1515	1518
4078	410	2998	1515
4079	710	1518	2998
4080	1515	2998	1518
4081	412	3005	2996
4082	710	2998	3005
4083	410	2996	2998
4084	3005	2998	2996
4085	26	1202	1204
4086	402	2978	1202
4087	404	1204	2978
4088	1202	2978	1204
4089	82	1509	1507
4090	707	2979	1509
4091	402	1507	2979
4092	1509	2979	1507
4093	194	2114	2116
4094	404	2983	2114
4095	707	2116	2983
4096	2114	2983	2116
4097	402	2979	2978
4098	707	2983	2979
4099	404	2978	2983
4100	2979	2983	2978
4101	84	1518	1516
4102	710	3843	1518
4103	706	1516	3843
4104	1518	3843	1516
4105	194	2116	2117

4106	707	3844	2116
4107	710	2117	3844
4108	2116	3844	2117
4109	82	1508	1509
4110	706	3842	1508
4111	707	1509	3842
4112	1508	3842	1509
4113	710	3844	3843
4114	707	3842	3844
4115	706	3843	3842
4116	3844	3842	3843
4117	12	1132	1130
4118	332	2780	1132
4119	330	1130	2780
4120	1132	2780	1130
4121	124	1720	1718
4122	796	2789	1720
4123	332	1718	2789
4124	1720	2789	1718
4125	122	1706	1708
4126	330	2782	1706
4127	796	1708	2782
4128	1706	2782	1708
4129	332	2789	2780
4130	796	2782	2789
4131	330	2780	2782
4132	2789	2782	2780
4133	58	1365	1363
4134	565	3414	1365
4135	563	1363	3414
4136	1365	3414	1363
4137	198	2141	2139
4138	805	3421	2141
4139	565	2139	3421
4140	2141	3421	2139
4141	124	1719	1722
4142	563	3416	1719
4143	805	1722	3416
4144	1719	3416	1722
4145	565	3421	3414
4146	805	3416	3421
4147	563	3414	3416
4148	3421	3416	3414
4149	28	1211	1214
4150	411	3000	1211
4151	414	1214	3000
4152	1211	3000	1214
4153	122	1710	1707
4154	798	3002	1710
4155	411	1707	3002
4156	1710	3002	1707
4157	198	2138	2140
4158	414	3010	2138
4159	798	2140	3010
4160	2138	3010	2140
4161	411	3002	3000
4162	798	3010	3002
4163	414	3000	3010
4164	3002	3010	3000

4165	124	1722	1720
4166	805	3933	1722
4167	796	1720	3933
4168	1722	3933	1720
4169	198	2140	2141
4170	798	3936	2140
4171	805	2141	3936
4172	2140	3936	2141
4173	122	1708	1710
4174	796	3932	1708
4175	798	1710	3932
4176	1708	3932	1710
4177	805	3936	3933
4178	798	3932	3936
4179	796	3933	3932
4180	3936	3932	3933
4181	10	1122	1124
4182	322	2762	1122
4183	324	1124	2762
4184	1122	2762	1124
4185	114	1668	1666
4186	778	2763	1668
4187	322	1666	2763
4188	1668	2763	1666
4189	116	1674	1676
4190	324	2767	1674
4191	778	1676	2767
4192	1674	2767	1676
4193	322	2763	2762
4194	778	2767	2763
4195	324	2762	2767
4196	2763	2767	2762
4197	26	1205	1203
4198	405	2980	1205
4199	403	1203	2980
4200	1205	2980	1203
4201	195	2122	2120
4202	779	2985	2122
4203	405	2120	2985
4204	2122	2985	2120
4205	114	1667	1669
4206	403	2981	1667
4207	779	1669	2981
4208	1667	2981	1669
4209	405	2985	2980
4210	779	2981	2985
4211	403	2980	2981
4212	2985	2981	2980
4213	58	1362	1364
4214	562	3410	1362
4215	564	1364	3410
4216	1362	3410	1364
4217	116	1678	1675
4218	783	3412	1678
4219	562	1675	3412
4220	1678	3412	1675
4221	195	2121	2123
4222	564	3419	2121
4223	783	2123	3419

4224	2121	3419	2123
4225	562	3412	3410
4226	783	3419	3412
4227	564	3410	3419
4228	3412	3419	3410
4229	114	1669	1668
4230	779	3914	1669
4231	778	1668	3914
4232	1669	3914	1668
4233	195	2123	2122
4234	783	3916	2123
4235	779	2122	3916
4236	2123	3916	2122
4237	116	1676	1678
4238	778	3915	1676
4239	783	1678	3915
4240	1676	3915	1678
4241	779	3916	3914
4242	783	3915	3916
4243	778	3914	3915
4244	3916	3915	3914
4245	28	1214	1212
4246	414	3004	1214
4247	412	1212	3004
4248	1214	3004	1212
4249	198	2142	2138
4250	995	3011	2142
4251	414	2138	3011
4252	2142	3011	2138
4253	194	2115	2119
4254	412	3006	2115
4255	995	2119	3006
4256	2115	3006	2119
4257	414	3011	3004
4258	995	3006	3011
4259	412	3004	3006
4260	3011	3006	3004
4261	58	1364	1365
4262	564	3418	1364
4263	565	1365	3418
4264	1364	3418	1365
4265	195	2125	2121
4266	996	3420	2125
4267	564	2121	3420
4268	2125	3420	2121
4269	198	2139	2143
4270	565	3422	2139
4271	996	2143	3422
4272	2139	3422	2143
4273	564	3420	3418
4274	996	3422	3420
4275	565	3418	3422
4276	3420	3422	3418
4277	26	1204	1205
4278	404	2982	1204
4279	405	1205	2982
4280	1204	2982	1205
4281	194	2118	2114
4282	994	2984	2118

4283	404	2114	2984
4284	2118	2984	2114
4285	195	2120	2124
4286	405	2986	2120
4287	994	2124	2986
4288	2120	2986	2124
4289	404	2984	2982
4290	994	2986	2984
4291	405	2982	2986
4292	2984	2986	2982
4293	198	2143	2142
4294	996	4132	2143
4295	995	2142	4132
4296	2143	4132	2142
4297	195	2124	2125
4298	994	4131	2124
4299	996	2125	4131
4300	2124	4131	2125
4301	194	2119	2118
4302	995	4130	2119
4303	994	2118	4130
4304	2119	4130	2118
4305	996	4131	4132
4306	994	4130	4131
4307	995	4132	4130
4308	4131	4130	4132
4309	7	1110	1108
4310	310	2726	1110
4311	308	1108	2726
4312	1110	2726	1108
4313	102	1604	1602
4314	742	2735	1604
4315	310	1602	2735
4316	1604	2735	1602
4317	100	1590	1592
4318	308	2728	1590
4319	742	1592	2728
4320	1590	2728	1592
4321	310	2735	2726
4322	742	2728	2735
4323	308	2726	2728
4324	2735	2728	2726
4325	46	1300	1298
4326	500	3230	1300
4327	498	1298	3230
4328	1300	3230	1298
4329	230	2333	2331
4330	751	3239	2333
4331	500	2331	3239
4332	2333	3239	2331
4333	102	1603	1606
4334	498	3232	1603
4335	751	1606	3232
4336	1603	3232	1606
4337	500	3239	3230
4338	751	3232	3239
4339	498	3230	3232
4340	3239	3232	3230
4341	44	1286	1288

4342	486	3194	1286
4343	488	1288	3194
4344	1286	3194	1288
4345	100	1594	1591
4346	744	3196	1594
4347	486	1591	3196
4348	1594	3196	1591
4349	230	2330	2332
4350	488	3203	2330
4351	744	2332	3203
4352	2330	3203	2332
4353	486	3196	3194
4354	744	3203	3196
4355	488	3194	3203
4356	3196	3203	3194
4357	102	1606	1604
4358	751	3879	1606
4359	742	1604	3879
4360	1606	3879	1604
4361	230	2332	2333
4362	744	3882	2332
4363	751	2333	3882
4364	2332	3882	2333
4365	100	1592	1594
4366	742	3878	1592
4367	744	1594	3878
4368	1592	3878	1594
4369	751	3882	3879
4370	744	3878	3882
4371	742	3879	3878
4372	3882	3878	3879
4373	20	1169	1167
4374	369	2874	1169
4375	367	1167	2874
4376	1169	2874	1167
4377	161	1918	1916
4378	890	2881	1918
4379	369	1916	2881
4380	1918	2881	1916
4381	159	1904	1906
4382	367	2876	1904
4383	890	1906	2876
4384	1904	2876	1906
4385	369	2881	2874
4386	890	2876	2881
4387	367	2874	2876
4388	2881	2876	2874
4389	62	1389	1387
4390	589	3486	1389
4391	587	1387	3486
4392	1389	3486	1387
4393	239	2387	2385
4394	897	3493	2387
4395	589	2385	3493
4396	2387	3493	2385
4397	161	1917	1920
4398	587	3488	1917
4399	897	1920	3488
4400	1917	3488	1920

4401	589	3493	3486
4402	897	3488	3493
4403	587	3486	3488
4404	3493	3488	3486
4405	46	1299	1302
4406	499	3234	1299
4407	502	1302	3234
4408	1299	3234	1302
4409	159	1908	1905
4410	892	3236	1908
4411	499	1905	3236
4412	1908	3236	1905
4413	239	2384	2386
4414	502	3244	2384
4415	892	2386	3244
4416	2384	3244	2386
4417	499	3236	3234
4418	892	3244	3236
4419	502	3234	3244
4420	3236	3244	3234
4421	161	1920	1918
4422	897	4029	1920
4423	890	1918	4029
4424	1920	4029	1918
4425	239	2386	2387
4426	892	4032	2386
4427	897	2387	4032
4428	2386	4032	2387
4429	159	1906	1908
4430	890	4028	1906
4431	892	1908	4028
4432	1906	4028	1908
4433	897	4032	4029
4434	892	4028	4032
4435	890	4029	4028
4436	4032	4028	4029
4437	12	1131	1134
4438	331	2784	1131
4439	334	1134	2784
4440	1131	2784	1134
4441	123	1714	1712
4442	800	2786	1714
4443	331	1712	2786
4444	1714	2786	1712
4445	126	1730	1732
4446	334	2794	1730
4447	800	1732	2794
4448	1730	2794	1732
4449	331	2786	2784
4450	800	2794	2786
4451	334	2784	2794
4452	2786	2794	2784
4453	44	1290	1287
4454	490	3198	1290
4455	487	1287	3198
4456	1290	3198	1287
4457	232	2344	2342
4458	802	3208	2344
4459	490	2342	3208

4460	2344	3208	2342
4461	123	1713	1716
4462	487	3200	1713
4463	802	1716	3200
4464	1713	3200	1716
4465	490	3208	3198
4466	802	3200	3208
4467	487	3198	3200
4468	3208	3200	3198
4469	62	1386	1388
4470	586	3482	1386
4471	588	1388	3482
4472	1386	3482	1388
4473	126	1734	1731
4474	810	3484	1734
4475	586	1731	3484
4476	1734	3484	1731
4477	232	2343	2345
4478	588	3491	2343
4479	810	2345	3491
4480	2343	3491	2345
4481	586	3484	3482
4482	810	3491	3484
4483	588	3482	3491
4484	3484	3491	3482
4485	123	1716	1714
4486	802	3938	1716
4487	800	1714	3938
4488	1716	3938	1714
4489	232	2345	2344
4490	810	3942	2345
4491	802	2344	3942
4492	2345	3942	2344
4493	126	1732	1734
4494	800	3939	1732
4495	810	1734	3939
4496	1732	3939	1734
4497	802	3942	3938
4498	810	3939	3942
4499	800	3938	3939
4500	3942	3939	3938
4501	46	1302	1300
4502	502	3238	1302
4503	500	1300	3238
4504	1302	3238	1300
4505	239	2388	2384
4506	1031	3245	2388
4507	502	2384	3245
4508	2388	3245	2384
4509	230	2331	2335
4510	500	3240	2331
4511	1031	2335	3240
4512	2331	3240	2335
4513	502	3245	3238
4514	1031	3240	3245
4515	500	3238	3240
4516	3245	3240	3238
4517	62	1388	1389
4518	588	3490	1388

4519	589	1389	3490
4520	1388	3490	1389
4521	232	2347	2343
4522	1034	3492	2347
4523	588	2343	3492
4524	2347	3492	2343
4525	239	2385	2389
4526	589	3494	2385
4527	1034	2389	3494
4528	2385	3494	2389
4529	588	3492	3490
4530	1034	3494	3492
4531	589	3490	3494
4532	3492	3494	3490
4533	44	1288	1290
4534	488	3202	1288
4535	490	1290	3202
4536	1288	3202	1290
4537	230	2334	2330
4538	1030	3204	2334
4539	488	2330	3204
4540	2334	3204	2330
4541	232	2342	2346
4542	490	3209	2342
4543	1030	2346	3209
4544	2342	3209	2346
4545	488	3204	3202
4546	1030	3209	3204
4547	490	3202	3209
4548	3204	3209	3202
4549	239	2389	2388
4550	1034	4168	2389
4551	1031	2388	4168
4552	2389	4168	2388
4553	232	2346	2347
4554	1030	4167	2346
4555	1034	2347	4167
4556	2346	4167	2347
4557	230	2335	2334
4558	1031	4166	2335
4559	1030	2334	4166
4560	2335	4166	2334
4561	1034	4167	4168
4562	1030	4166	4167
4563	1031	4168	4166
4564	4167	4166	4168
4565	5	1100	1102
4566	300	2708	1100
4567	302	1102	2708
4568	1100	2708	1102
4569	92	1552	1550
4570	724	2709	1552
4571	300	1550	2709
4572	1552	2709	1550
4573	94	1558	1560
4574	302	2713	1558
4575	724	1560	2713
4576	1558	2713	1560
4577	300	2709	2708

4578	724	2713	2709
4579	302	2708	2713
4580	2709	2713	2708
4581	36	1248	1246
4582	448	3086	1248
4583	446	1246	3086
4584	1248	3086	1246
4585	212	2224	2222
4586	725	3091	2224
4587	448	2222	3091
4588	2224	3091	2222
4589	92	1551	1553
4590	446	3087	1551
4591	725	1553	3087
4592	1551	3087	1553
4593	448	3091	3086
4594	725	3087	3091
4595	446	3086	3087
4596	3091	3087	3086
4597	38	1254	1256
4598	454	3104	1254
4599	456	1256	3104
4600	1254	3104	1256
4601	94	1562	1559
4602	729	3106	1562
4603	454	1559	3106
4604	1562	3106	1559
4605	212	2223	2225
4606	456	3113	2223
4607	729	2225	3113
4608	2223	3113	2225
4609	454	3106	3104
4610	729	3113	3106
4611	456	3104	3113
4612	3106	3113	3104
4613	92	1553	1552
4614	725	3860	1553
4615	724	1552	3860
4616	1553	3860	1552
4617	212	2225	2224
4618	729	3862	2225
4619	725	2224	3862
4620	2225	3862	2224
4621	94	1560	1562
4622	724	3861	1560
4623	729	1562	3861
4624	1560	3861	1562
4625	725	3862	3860
4626	729	3861	3862
4627	724	3860	3861
4628	3862	3861	3860
4629	10	1125	1123
4630	325	2764	1125
4631	323	1123	2764
4632	1125	2764	1123
4633	117	1682	1680
4634	780	2769	1682
4635	325	1680	2769
4636	1682	2769	1680

4637	115	1670	1672
4638	323	2765	1670
4639	780	1672	2765
4640	1670	2765	1672
4641	325	2769	2764
4642	780	2765	2769
4643	323	2764	2765
4644	2769	2765	2764
4645	59	1370	1368
4646	570	3428	1370
4647	568	1368	3428
4648	1370	3428	1368
4649	213	2231	2229
4650	785	3437	2231
4651	570	2229	3437
4652	2231	3437	2229
4653	117	1681	1684
4654	568	3430	1681
4655	785	1684	3430
4656	1681	3430	1684
4657	570	3437	3428
4658	785	3430	3437
4659	568	3428	3430
4660	3437	3430	3428
4661	36	1247	1249
4662	447	3088	1247
4663	449	1249	3088
4664	1247	3088	1249
4665	115	1673	1671
4666	781	3089	1673
4667	447	1671	3089
4668	1673	3089	1671
4669	213	2228	2230
4670	449	3093	2228
4671	781	2230	3093
4672	2228	3093	2230
4673	447	3089	3088
4674	781	3093	3089
4675	449	3088	3093
4676	3089	3093	3088
4677	117	1684	1682
4678	785	3918	1684
4679	780	1682	3918
4680	1684	3918	1682
4681	213	2230	2231
4682	781	3919	2230
4683	785	2231	3919
4684	2230	3919	2231
4685	115	1672	1673
4686	780	3917	1672
4687	781	1673	3917
4688	1672	3917	1673
4689	785	3919	3918
4690	781	3917	3919
4691	780	3918	3917
4692	3919	3917	3918
4693	20	1166	1168
4694	366	2870	1166
4695	368	1168	2870

4696	1166	2870	1168
4697	158	1900	1898
4698	886	2872	1900
4699	366	1898	2872
4700	1900	2872	1898
4701	160	1910	1912
4702	368	2879	1910
4703	886	1912	2879
4704	1910	2879	1912
4705	366	2872	2870
4706	886	2879	2872
4707	368	2870	2879
4708	2872	2879	2870
4709	38	1258	1255
4710	458	3108	1258
4711	455	1255	3108
4712	1258	3108	1255
4713	217	2254	2252
4714	888	3118	2254
4715	458	2252	3118
4716	2254	3118	2252
4717	158	1899	1902
4718	455	3110	1899
4719	888	1902	3110
4720	1899	3110	1902
4721	458	3118	3108
4722	888	3110	3118
4723	455	3108	3110
4724	3118	3110	3108
4725	59	1369	1371
4726	569	3432	1369
4727	571	1371	3432
4728	1369	3432	1371
4729	160	1914	1911
4730	895	3434	1914
4731	569	1911	3434
4732	1914	3434	1911
4733	217	2253	2255
4734	571	3439	2253
4735	895	2255	3439
4736	2253	3439	2255
4737	569	3434	3432
4738	895	3439	3434
4739	571	3432	3439
4740	3434	3439	3432
4741	158	1902	1900
4742	888	4022	1902
4743	886	1900	4022
4744	1902	4022	1900
4745	217	2255	2254
4746	895	4026	2255
4747	888	2254	4026
4748	2255	4026	2254
4749	160	1912	1914
4750	886	4023	1912
4751	895	1914	4023
4752	1912	4023	1914
4753	888	4026	4022
4754	895	4023	4026

4755	886	4022	4023
4756	4026	4023	4022
4757	36	1249	1248
4758	449	3090	1249
4759	448	1248	3090
4760	1249	3090	1248
4761	213	2232	2228
4762	1012	3094	2232
4763	449	2228	3094
4764	2232	3094	2228
4765	212	2222	2226
4766	448	3092	2222
4767	1012	2226	3092
4768	2222	3092	2226
4769	449	3094	3090
4770	1012	3092	3094
4771	448	3090	3092
4772	3094	3092	3090
4773	59	1371	1370
4774	571	3436	1371
4775	570	1370	3436
4776	1371	3436	1370
4777	217	2257	2253
4778	1014	3440	2257
4779	571	2253	3440
4780	2257	3440	2253
4781	213	2229	2233
4782	570	3438	2229
4783	1014	2233	3438
4784	2229	3438	2233
4785	571	3440	3436
4786	1014	3438	3440
4787	570	3436	3438
4788	3440	3438	3436
4789	38	1256	1258
4790	456	3112	1256
4791	458	1258	3112
4792	1256	3112	1258
4793	212	2227	2223
4794	1013	3114	2227
4795	456	2223	3114
4796	2227	3114	2223
4797	217	2252	2256
4798	458	3119	2252
4799	1013	2256	3119
4800	2252	3119	2256
4801	456	3114	3112
4802	1013	3119	3114
4803	458	3112	3119
4804	3114	3119	3112
4805	213	2233	2232
4806	1014	4149	2233
4807	1012	2232	4149
4808	2233	4149	2232
4809	217	2256	2257
4810	1013	4150	2256
4811	1014	2257	4150
4812	2256	4150	2257
4813	212	2226	2227

4814	1012	4148	2226
4815	1013	2227	4148
4816	2226	4148	2227
4817	1014	4150	4149
4818	1013	4148	4150
4819	1012	4149	4148
4820	4150	4148	4149
4821	12	1134	1132
4822	334	2788	1134
4823	332	1132	2788
4824	1134	2788	1132
4825	126	1733	1730
4826	804	2795	1733
4827	334	1730	2795
4828	1733	2795	1730
4829	124	1718	1721
4830	332	2790	1718
4831	804	1721	2790
4832	1718	2790	1721
4833	334	2795	2788
4834	804	2790	2795
4835	332	2788	2790
4836	2795	2790	2788
4837	62	1390	1386
4838	590	3483	1390
4839	586	1386	3483
4840	1390	3483	1386
4841	267	2555	2553
4842	811	3496	2555
4843	590	2553	3496
4844	2555	3496	2553
4845	126	1731	1735
4846	586	3485	1731
4847	811	1735	3485
4848	1731	3485	1735
4849	590	3496	3483
4850	811	3485	3496
4851	586	3483	3485
4852	3496	3485	3483
4853	58	1363	1367
4854	563	3415	1363
4855	567	1367	3415
4856	1363	3415	1367
4857	124	1723	1719
4858	806	3417	1723
4859	563	1719	3417
4860	1723	3417	1719
4861	267	2552	2554
4862	567	3426	2552
4863	806	2554	3426
4864	2552	3426	2554
4865	563	3417	3415
4866	806	3426	3417
4867	567	3415	3426
4868	3417	3426	3415
4869	126	1735	1733
4870	811	3945	1735
4871	804	1733	3945
4872	1735	3945	1733

4873	267	2554	2555
4874	806	3946	2554
4875	811	2555	3946
4876	2554	3946	2555
4877	124	1721	1723
4878	804	3944	1721
4879	806	1723	3944
4880	1721	3944	1723
4881	811	3946	3945
4882	806	3944	3946
4883	804	3945	3944
4884	3946	3944	3945
4885	20	1168	1169
4886	368	2878	1168
4887	369	1169	2878
4888	1168	2878	1169
4889	160	1913	1910
4890	894	2880	1913
4891	368	1910	2880
4892	1913	2880	1910
4893	161	1916	1919
4894	369	2882	1916
4895	894	1919	2882
4896	1916	2882	1919
4897	368	2880	2878
4898	894	2882	2880
4899	369	2878	2882
4900	2880	2882	2878
4901	59	1373	1369
4902	573	3433	1373
4903	569	1369	3433
4904	1373	3433	1369
4905	268	2560	2558
4906	896	3444	2560
4907	573	2558	3444
4908	2560	3444	2558
4909	160	1911	1915
4910	569	3435	1911
4911	896	1915	3435
4912	1911	3435	1915
4913	573	3444	3433
4914	896	3435	3444
4915	569	3433	3435
4916	3444	3435	3433
4917	62	1387	1391
4918	587	3487	1387
4919	591	1391	3487
4920	1387	3487	1391
4921	161	1921	1917
4922	898	3489	1921
4923	587	1917	3489
4924	1921	3489	1917
4925	268	2559	2561
4926	591	3498	2559
4927	898	2561	3498
4928	2559	3498	2561
4929	587	3489	3487
4930	898	3498	3489
4931	591	3487	3498

4932	3489	3498	3487
4933	160	1915	1913
4934	896	4034	1915
4935	894	1913	4034
4936	1915	4034	1913
4937	268	2561	2560
4938	898	4036	2561
4939	896	2560	4036
4940	2561	4036	2560
4941	161	1919	1921
4942	894	4035	1919
4943	898	1921	4035
4944	1919	4035	1921
4945	896	4036	4034
4946	898	4035	4036
4947	894	4034	4035
4948	4036	4035	4034
4949	10	1124	1125
4950	324	2766	1124
4951	325	1125	2766
4952	1124	2766	1125
4953	116	1677	1674
4954	782	2768	1677
4955	324	1674	2768
4956	1677	2768	1674
4957	117	1680	1683
4958	325	2770	1680
4959	782	1683	2770
4960	1680	2770	1683
4961	324	2768	2766
4962	782	2770	2768
4963	325	2766	2770
4964	2768	2770	2766
4965	58	1366	1362
4966	566	3411	1366
4967	562	1362	3411
4968	1366	3411	1362
4969	266	2548	2546
4970	784	3424	2548
4971	566	2546	3424
4972	2548	3424	2546
4973	116	1675	1679
4974	562	3413	1675
4975	784	1679	3413
4976	1675	3413	1679
4977	566	3424	3411
4978	784	3413	3424
4979	562	3411	3413
4980	3424	3413	3411
4981	59	1368	1372
4982	568	3429	1368
4983	572	1372	3429
4984	1368	3429	1372
4985	117	1685	1681
4986	786	3431	1685
4987	568	1681	3431
4988	1685	3431	1681
4989	266	2547	2549
4990	572	3442	2547

4991	786	2549	3442
4992	2547	3442	2549
4993	568	3431	3429
4994	786	3442	3431
4995	572	3429	3442
4996	3431	3442	3429
4997	116	1679	1677
4998	784	3920	1679
4999	782	1677	3920
5000	1679	3920	1677
5001	266	2549	2548
5002	786	3922	2549
5003	784	2548	3922
5004	2549	3922	2548
5005	117	1683	1685
5006	782	3921	1683
5007	786	1685	3921
5008	1683	3921	1685
5009	784	3922	3920
5010	786	3921	3922
5011	782	3920	3921
5012	3922	3921	3920
5013	62	1391	1390
5014	591	3495	1391
5015	590	1390	3495
5016	1391	3495	1390
5017	268	2563	2559
5018	1068	3499	2563
5019	591	2559	3499
5020	2563	3499	2559
5021	267	2553	2557
5022	590	3497	2553
5023	1068	2557	3497
5024	2553	3497	2557
5025	591	3499	3495
5026	1068	3497	3499
5027	590	3495	3497
5028	3499	3497	3495
5029	59	1372	1373
5030	572	3441	1372
5031	573	1373	3441
5032	1372	3441	1373
5033	266	2551	2547
5034	1067	3443	2551
5035	572	2547	3443
5036	2551	3443	2547
5037	268	2558	2562
5038	573	3445	2558
5039	1067	2562	3445
5040	2558	3445	2562
5041	572	3443	3441
5042	1067	3445	3443
5043	573	3441	3445
5044	3443	3445	3441
5045	58	1367	1366
5046	567	3423	1367
5047	566	1366	3423
5048	1367	3423	1366
5049	267	2556	2552

5050	1066	3427	2556
5051	567	2552	3427
5052	2556	3427	2552
5053	266	2546	2550
5054	566	3425	2546
5055	1066	2550	3425
5056	2546	3425	2550
5057	567	3427	3423
5058	1066	3425	3427
5059	566	3423	3425
5060	3427	3425	3423
5061	268	2562	2563
5062	1067	4204	2562
5063	1068	2563	4204
5064	2562	4204	2563
5065	266	2550	2551
5066	1066	4202	2550
5067	1067	2551	4202
5068	2550	4202	2551
5069	267	2557	2556
5070	1068	4203	2557
5071	1066	2556	4203
5072	2557	4203	2556
5073	1067	4202	4204
5074	1066	4203	4202
5075	1068	4204	4203
5076	4202	4203	4204
5077	4	1098	1097
5078	298	2702	1098
5079	297	1097	2702
5080	1098	2702	1097
5081	90	1544	1542
5082	718	2706	1544
5083	298	1542	2706
5084	1544	2706	1542
5085	89	1536	1538
5086	297	2704	1536
5087	718	1538	2704
5088	1536	2704	1538
5089	298	2706	2702
5090	718	2704	2706
5091	297	2702	2704
5092	2706	2704	2702
5093	34	1240	1238
5094	440	3068	1240
5095	438	1238	3068
5096	1240	3068	1238
5097	206	2189	2187
5098	722	3073	2189
5099	440	2187	3073
5100	2189	3073	2187
5101	90	1543	1545
5102	438	3069	1543
5103	722	1545	3069
5104	1543	3069	1545
5105	440	3073	3068
5106	722	3069	3073
5107	438	3068	3069
5108	3073	3069	3068

5109	33	1232	1234
5110	432	3050	1232
5111	434	1234	3050
5112	1232	3050	1234
5113	89	1540	1537
5114	720	3052	1540
5115	432	1537	3052
5116	1540	3052	1537
5117	206	2186	2188
5118	434	3059	2186
5119	720	2188	3059
5120	2186	3059	2188
5121	432	3052	3050
5122	720	3059	3052
5123	434	3050	3059
5124	3052	3059	3050
5125	90	1545	1544
5126	722	3855	1545
5127	718	1544	3855
5128	1545	3855	1544
5129	206	2188	2189
5130	720	3858	2188
5131	722	2189	3858
5132	2188	3858	2189
5133	89	1538	1540
5134	718	3854	1538
5135	720	1540	3854
5136	1538	3854	1540
5137	722	3858	3855
5138	720	3854	3858
5139	718	3855	3854
5140	3858	3854	3855
5141	18	1160	1158
5142	360	2852	1160
5143	358	1158	2852
5144	1160	2852	1158
5145	152	1868	1866
5146	868	2857	1868
5147	360	1866	2857
5148	1868	2857	1866
5149	150	1858	1860
5150	358	2853	1858
5151	868	1860	2853
5152	1858	2853	1860
5153	360	2857	2852
5154	868	2853	2857
5155	358	2852	2853
5156	2857	2853	2852
5157	70	1437	1435
5158	637	3630	1437
5159	635	1435	3630
5160	1437	3630	1435
5161	210	2213	2211
5162	873	3637	2213
5163	637	2211	3637
5164	2213	3637	2211
5165	152	1867	1870
5166	635	3632	1867
5167	873	1870	3632

5168	1867	3632	1870
5169	637	3637	3630
5170	873	3632	3637
5171	635	3630	3632
5172	3637	3632	3630
5173	34	1239	1241
5174	439	3070	1239
5175	441	1241	3070
5176	1239	3070	1241
5177	150	1861	1859
5178	869	3071	1861
5179	439	1859	3071
5180	1861	3071	1859
5181	210	2210	2212
5182	441	3075	2210
5183	869	2212	3075
5184	2210	3075	2212
5185	439	3071	3070
5186	869	3075	3071
5187	441	3070	3075
5188	3071	3075	3070
5189	152	1870	1868
5190	873	4005	1870
5191	868	1868	4005
5192	1870	4005	1868
5193	210	2212	2213
5194	869	4006	2212
5195	873	2213	4006
5196	2212	4006	2213
5197	150	1860	1861
5198	868	4004	1860
5199	869	1861	4004
5200	1860	4004	1861
5201	873	4006	4005
5202	869	4004	4006
5203	868	4005	4004
5204	4006	4004	4005
5205	17	1152	1154
5206	352	2834	1152
5207	354	1154	2834
5208	1152	2834	1154
5209	144	1824	1822
5210	850	2836	1824
5211	352	1822	2836
5212	1824	2836	1822
5213	146	1834	1836
5214	354	2843	1834
5215	850	1836	2843
5216	1834	2843	1836
5217	352	2836	2834
5218	850	2843	2836
5219	354	2834	2843
5220	2836	2843	2834
5221	33	1236	1233
5222	436	3054	1236
5223	433	1233	3054
5224	1236	3054	1233
5225	208	2200	2198
5226	852	3064	2200

5227	436	2198	3064
5228	2200	3064	2198
5229	144	1823	1826
5230	433	3056	1823
5231	852	1826	3056
5232	1823	3056	1826
5233	436	3064	3054
5234	852	3056	3064
5235	433	3054	3056
5236	3064	3056	3054
5237	70	1434	1436
5238	634	3626	1434
5239	636	1436	3626
5240	1434	3626	1436
5241	146	1838	1835
5242	859	3628	1838
5243	634	1835	3628
5244	1838	3628	1835
5245	208	2199	2201
5246	636	3635	2199
5247	859	2201	3635
5248	2199	3635	2201
5249	634	3628	3626
5250	859	3635	3628
5251	636	3626	3635
5252	3628	3635	3626
5253	144	1826	1824
5254	852	3986	1826
5255	850	1824	3986
5256	1826	3986	1824
5257	208	2201	2200
5258	859	3990	2201
5259	852	2200	3990
5260	2201	3990	2200
5261	146	1836	1838
5262	850	3987	1836
5263	859	1838	3987
5264	1836	3987	1838
5265	852	3990	3986
5266	859	3987	3990
5267	850	3986	3987
5268	3990	3987	3986
5269	34	1241	1240
5270	441	3072	1241
5271	440	1240	3072
5272	1241	3072	1240
5273	210	2214	2210
5274	1007	3076	2214
5275	441	2210	3076
5276	2214	3076	2210
5277	206	2187	2191
5278	440	3074	2187
5279	1007	2191	3074
5280	2187	3074	2191
5281	441	3076	3072
5282	1007	3074	3076
5283	440	3072	3074
5284	3076	3074	3072
5285	70	1436	1437

5286	636	3634	1436
5287	637	1437	3634
5288	1436	3634	1437
5289	208	2203	2199
5290	1010	3636	2203
5291	636	2199	3636
5292	2203	3636	2199
5293	210	2211	2215
5294	637	3638	2211
5295	1010	2215	3638
5296	2211	3638	2215
5297	636	3636	3634
5298	1010	3638	3636
5299	637	3634	3638
5300	3636	3638	3634
5301	33	1234	1236
5302	434	3058	1234
5303	436	1236	3058
5304	1234	3058	1236
5305	206	2190	2186
5306	1006	3060	2190
5307	434	2186	3060
5308	2190	3060	2186
5309	208	2198	2202
5310	436	3065	2198
5311	1006	2202	3065
5312	2198	3065	2202
5313	434	3060	3058
5314	1006	3065	3060
5315	436	3058	3065
5316	3060	3065	3058
5317	210	2215	2214
5318	1010	4144	2215
5319	1007	2214	4144
5320	2215	4144	2214
5321	208	2202	2203
5322	1006	4143	2202
5323	1010	2203	4143
5324	2202	4143	2203
5325	206	2191	2190
5326	1007	4142	2191
5327	1006	2190	4142
5328	2191	4142	2190
5329	1010	4143	4144
5330	1006	4142	4143
5331	1007	4144	4142
5332	4143	4142	4144
5333	8	1117	1115
5334	317	2746	1117
5335	315	1115	2746
5336	1117	2746	1115
5337	109	1642	1640
5338	762	2751	1642
5339	317	1640	2751
5340	1642	2751	1640
5341	107	1630	1632
5342	315	2747	1630
5343	762	1632	2747
5344	1630	2747	1632

5345	317	2751	2746
5346	762	2747	2751
5347	315	2746	2747
5348	2751	2747	2746
5349	53	1338	1336
5350	538	3338	1338
5351	536	1336	3338
5352	1338	3338	1336
5353	250	2453	2451
5354	767	3347	2453
5355	538	2451	3347
5356	2453	3347	2451
5357	109	1641	1644
5358	536	3340	1641
5359	767	1644	3340
5360	1641	3340	1644
5361	538	3347	3338
5362	767	3340	3347
5363	536	3338	3340
5364	3347	3340	3338
5365	51	1326	1328
5366	526	3311	1326
5367	528	1328	3311
5368	1326	3311	1328
5369	107	1633	1631
5370	763	3312	1633
5371	526	1631	3312
5372	1633	3312	1631
5373	250	2450	2452
5374	528	3316	2450
5375	763	2452	3316
5376	2450	3316	2452
5377	526	3312	3311
5378	763	3316	3312
5379	528	3311	3316
5380	3312	3316	3311
5381	109	1644	1642
5382	767	3900	1644
5383	762	1642	3900
5384	1644	3900	1642
5385	250	2452	2453
5386	763	3901	2452
5387	767	2453	3901
5388	2452	3901	2453
5389	107	1632	1633
5390	762	3899	1632
5391	763	1633	3899
5392	1632	3899	1633
5393	767	3901	3900
5394	763	3899	3901
5395	762	3900	3899
5396	3901	3899	3900
5397	24	1193	1191
5398	393	2946	1193
5399	391	1191	2946
5400	1193	2946	1191
5401	185	2062	2060
5402	962	2953	2062
5403	393	2060	2953

5404	2062	2953	2060
5405	183	2048	2050
5406	391	2948	2048
5407	962	2050	2948
5408	2048	2948	2050
5409	393	2953	2946
5410	962	2948	2953
5411	391	2946	2948
5412	2953	2948	2946
5413	74	1461	1459
5414	661	3702	1461
5415	659	1459	3702
5416	1461	3702	1459
5417	255	2483	2481
5418	969	3709	2483
5419	661	2481	3709
5420	2483	3709	2481
5421	185	2061	2064
5422	659	3704	2061
5423	969	2064	3704
5424	2061	3704	2064
5425	661	3709	3702
5426	969	3704	3709
5427	659	3702	3704
5428	3709	3704	3702
5429	53	1337	1340
5430	537	3342	1337
5431	540	1340	3342
5432	1337	3342	1340
5433	183	2052	2049
5434	964	3344	2052
5435	537	2049	3344
5436	2052	3344	2049
5437	255	2480	2482
5438	540	3352	2480
5439	964	2482	3352
5440	2480	3352	2482
5441	537	3344	3342
5442	964	3352	3344
5443	540	3342	3352
5444	3344	3352	3342
5445	185	2064	2062
5446	969	4101	2064
5447	962	2062	4101
5448	2064	4101	2062
5449	255	2482	2483
5450	964	4104	2482
5451	969	2483	4104
5452	2482	4104	2483
5453	183	2050	2052
5454	962	4100	2050
5455	964	2052	4100
5456	2050	4100	2052
5457	969	4104	4101
5458	964	4100	4104
5459	962	4101	4100
5460	4104	4100	4101
5461	18	1159	1161
5462	359	2854	1159

5463	361	1161	2854
5464	1159	2854	1161
5465	151	1864	1862
5466	870	2855	1864
5467	359	1862	2855
5468	1864	2855	1862
5469	153	1872	1874
5470	361	2859	1872
5471	870	1874	2859
5472	1872	2859	1874
5473	359	2855	2854
5474	870	2859	2855
5475	361	2854	2859
5476	2855	2859	2854
5477	51	1329	1327
5478	529	3313	1329
5479	527	1327	3313
5480	1329	3313	1327
5481	251	2458	2456
5482	871	3318	2458
5483	529	2456	3318
5484	2458	3318	2456
5485	151	1863	1865
5486	527	3314	1863
5487	871	1865	3314
5488	1863	3314	1865
5489	529	3318	3313
5490	871	3314	3318
5491	527	3313	3314
5492	3318	3314	3313
5493	74	1458	1460
5494	658	3698	1458
5495	660	1460	3698
5496	1458	3698	1460
5497	153	1876	1873
5498	875	3700	1876
5499	658	1873	3700
5500	1876	3700	1873
5501	251	2457	2459
5502	660	3707	2457
5503	875	2459	3707
5504	2457	3707	2459
5505	658	3700	3698
5506	875	3707	3700
5507	660	3698	3707
5508	3700	3707	3698
5509	151	1865	1864
5510	871	4007	1865
5511	870	1864	4007
5512	1865	4007	1864
5513	251	2459	2458
5514	875	4009	2459
5515	871	2458	4009
5516	2459	4009	2458
5517	153	1874	1876
5518	870	4008	1874
5519	875	1876	4008
5520	1874	4008	1876
5521	871	4009	4007

5522	875	4008	4009
5523	870	4007	4008
5524	4009	4008	4007
5525	53	1340	1338
5526	540	3346	1340
5527	538	1338	3346
5528	1340	3346	1338
5529	255	2484	2480
5530	1052	3353	2484
5531	540	2480	3353
5532	2484	3353	2480
5533	250	2451	2455
5534	538	3348	2451
5535	1052	2455	3348
5536	2451	3348	2455
5537	540	3353	3346
5538	1052	3348	3353
5539	538	3346	3348
5540	3353	3348	3346
5541	74	1460	1461
5542	660	3706	1460
5543	661	1461	3706
5544	1460	3706	1461
5545	251	2461	2457
5546	1053	3708	2461
5547	660	2457	3708
5548	2461	3708	2457
5549	255	2481	2485
5550	661	3710	2481
5551	1053	2485	3710
5552	2481	3710	2485
5553	660	3708	3706
5554	1053	3710	3708
5555	661	3706	3710
5556	3708	3710	3706
5557	51	1328	1329
5558	528	3315	1328
5559	529	1329	3315
5560	1328	3315	1329
5561	250	2454	2450
5562	1051	3317	2454
5563	528	2450	3317
5564	2454	3317	2450
5565	251	2456	2460
5566	529	3319	2456
5567	1051	2460	3319
5568	2456	3319	2460
5569	528	3317	3315
5570	1051	3319	3317
5571	529	3315	3319
5572	3317	3319	3315
5573	255	2485	2484
5574	1053	4189	2485
5575	1052	2484	4189
5576	2485	4189	2484
5577	251	2460	2461
5578	1051	4188	2460
5579	1053	2461	4188
5580	2460	4188	2461

5581	250	2455	2454
5582	1052	4187	2455
5583	1051	2454	4187
5584	2455	4187	2454
5585	1053	4188	4189
5586	1051	4187	4188
5587	1052	4189	4187
5588	4188	4187	4189
5589	7	1109	1112
5590	309	2730	1109
5591	312	1112	2730
5592	1109	2730	1112
5593	101	1598	1596
5594	746	2732	1598
5595	309	1596	2732
5596	1598	2732	1596
5597	104	1614	1616
5598	312	2740	1614
5599	746	1616	2740
5600	1614	2740	1616
5601	309	2732	2730
5602	746	2740	2732
5603	312	2730	2740
5604	2732	2740	2730
5605	45	1294	1292
5606	494	3212	1294
5607	492	1292	3212
5608	1294	3212	1292
5609	234	2356	2354
5610	748	3221	2356
5611	494	2354	3221
5612	2356	3221	2354
5613	101	1597	1600
5614	492	3214	1597
5615	748	1600	3214
5616	1597	3214	1600
5617	494	3221	3212
5618	748	3214	3221
5619	492	3212	3214
5620	3221	3214	3212
5621	48	1310	1312
5622	510	3266	1310
5623	512	1312	3266
5624	1310	3266	1312
5625	104	1618	1615
5626	756	3268	1618
5627	510	1615	3268
5628	1618	3268	1615
5629	234	2355	2357
5630	512	3275	2355
5631	756	2357	3275
5632	2355	3275	2357
5633	510	3268	3266
5634	756	3275	3268
5635	512	3266	3275
5636	3268	3275	3266
5637	101	1600	1598
5638	748	3884	1600
5639	746	1598	3884

5640	1600	3884	1598
5641	234	2357	2356
5642	756	3888	2357
5643	748	2356	3888
5644	2357	3888	2356
5645	104	1616	1618
5646	746	3885	1616
5647	756	1618	3885
5648	1616	3885	1618
5649	748	3888	3884
5650	756	3885	3888
5651	746	3884	3885
5652	3888	3885	3884
5653	17	1156	1153
5654	356	2838	1156
5655	353	1153	2838
5656	1156	2838	1153
5657	148	1848	1846
5658	854	2848	1848
5659	356	1846	2848
5660	1848	2848	1846
5661	145	1828	1830
5662	353	2840	1828
5663	854	1830	2840
5664	1828	2840	1830
5665	356	2848	2838
5666	854	2840	2848
5667	353	2838	2840
5668	2848	2840	2838
5669	72	1448	1446
5670	648	3662	1448
5671	646	1446	3662
5672	1448	3662	1446
5673	236	2369	2367
5674	864	3671	2369
5675	648	2367	3671
5676	2369	3671	2367
5677	148	1847	1850
5678	646	3664	1847
5679	864	1850	3664
5680	1847	3664	1850
5681	648	3671	3662
5682	864	3664	3671
5683	646	3662	3664
5684	3671	3664	3662
5685	45	1293	1296
5686	493	3216	1293
5687	496	1296	3216
5688	1293	3216	1296
5689	145	1832	1829
5690	856	3218	1832
5691	493	1829	3218
5692	1832	3218	1829
5693	236	2366	2368
5694	496	3226	2366
5695	856	2368	3226
5696	2366	3226	2368
5697	493	3218	3216
5698	856	3226	3218

5699	496	3216	3226
5700	3218	3226	3216
5701	148	1850	1848
5702	864	3993	1850
5703	854	1848	3993
5704	1850	3993	1848
5705	236	2368	2369
5706	856	3996	2368
5707	864	2369	3996
5708	2368	3996	2369
5709	145	1830	1832
5710	854	3992	1830
5711	856	1832	3992
5712	1830	3992	1832
5713	864	3996	3993
5714	856	3992	3996
5715	854	3993	3992
5716	3996	3992	3993
5717	24	1190	1192
5718	390	2942	1190
5719	392	1192	2942
5720	1190	2942	1192
5721	182	2044	2042
5722	958	2944	2044
5723	390	2042	2944
5724	2044	2944	2042
5725	184	2054	2056
5726	392	2951	2054
5727	958	2056	2951
5728	2054	2951	2056
5729	390	2944	2942
5730	958	2951	2944
5731	392	2942	2951
5732	2944	2951	2942
5733	48	1314	1311
5734	514	3270	1314
5735	511	1311	3270
5736	1314	3270	1311
5737	244	2416	2414
5738	960	3280	2416
5739	514	2414	3280
5740	2416	3280	2414
5741	182	2043	2046
5742	511	3272	2043
5743	960	2046	3272
5744	2043	3272	2046
5745	514	3280	3270
5746	960	3272	3280
5747	511	3270	3272
5748	3280	3272	3270
5749	72	1447	1449
5750	647	3666	1447
5751	649	1449	3666
5752	1447	3666	1449
5753	184	2058	2055
5754	967	3668	2058
5755	647	2055	3668
5756	2058	3668	2055
5757	244	2415	2417

5758	649	3673	2415
5759	967	2417	3673
5760	2415	3673	2417
5761	647	3668	3666
5762	967	3673	3668
5763	649	3666	3673
5764	3668	3673	3666
5765	182	2046	2044
5766	960	4094	2046
5767	958	2044	4094
5768	2046	4094	2044
5769	244	2417	2416
5770	967	4098	2417
5771	960	2416	4098
5772	2417	4098	2416
5773	184	2056	2058
5774	958	4095	2056
5775	967	2058	4095
5776	2056	4095	2058
5777	960	4098	4094
5778	967	4095	4098
5779	958	4094	4095
5780	4098	4095	4094
5781	45	1296	1294
5782	496	3220	1296
5783	494	1294	3220
5784	1296	3220	1294
5785	236	2370	2366
5786	1036	3227	2370
5787	496	2366	3227
5788	2370	3227	2366
5789	234	2354	2358
5790	494	3222	2354
5791	1036	2358	3222
5792	2354	3222	2358
5793	496	3227	3220
5794	1036	3222	3227
5795	494	3220	3222
5796	3227	3222	3220
5797	72	1449	1448
5798	649	3670	1449
5799	648	1448	3670
5800	1449	3670	1448
5801	244	2419	2415
5802	1040	3674	2419
5803	649	2415	3674
5804	2419	3674	2415
5805	236	2367	2371
5806	648	3672	2367
5807	1040	2371	3672
5808	2367	3672	2371
5809	649	3674	3670
5810	1040	3672	3674
5811	648	3670	3672
5812	3674	3672	3670
5813	48	1312	1314
5814	512	3274	1312
5815	514	1314	3274
5816	1312	3274	1314

5817	234	2359	2355
5818	1037	3276	2359
5819	512	2355	3276
5820	2359	3276	2355
5821	244	2414	2418
5822	514	3281	2414
5823	1037	2418	3281
5824	2414	3281	2418
5825	512	3276	3274
5826	1037	3281	3276
5827	514	3274	3281
5828	3276	3281	3274
5829	236	2371	2370
5830	1040	4173	2371
5831	1036	2370	4173
5832	2371	4173	2370
5833	244	2418	2419
5834	1037	4174	2418
5835	1040	2419	4174
5836	2418	4174	2419
5837	234	2358	2359
5838	1036	4172	2358
5839	1037	2359	4172
5840	2358	4172	2359
5841	1040	4174	4173
5842	1037	4172	4174
5843	1036	4173	4172
5844	4174	4172	4173
5845	18	1161	1160
5846	361	2856	1161
5847	360	1160	2856
5848	1161	2856	1160
5849	153	1875	1872
5850	872	2860	1875
5851	361	1872	2860
5852	1875	2860	1872
5853	152	1866	1869
5854	360	2858	1866
5855	872	1869	2858
5856	1866	2858	1869
5857	361	2860	2856
5858	872	2858	2860
5859	360	2856	2858
5860	2860	2858	2856
5861	74	1462	1458
5862	662	3699	1462
5863	658	1458	3699
5864	1462	3699	1458
5865	279	2627	2625
5866	876	3712	2627
5867	662	2625	3712
5868	2627	3712	2625
5869	153	1873	1877
5870	658	3701	1873
5871	876	1877	3701
5872	1873	3701	1877
5873	662	3712	3699
5874	876	3701	3712
5875	658	3699	3701

5876	3712	3701	3699
5877	70	1435	1439
5878	635	3631	1435
5879	639	1439	3631
5880	1435	3631	1439
5881	152	1871	1867
5882	874	3633	1871
5883	635	1867	3633
5884	1871	3633	1867
5885	279	2624	2626
5886	639	3642	2624
5887	874	2626	3642
5888	2624	3642	2626
5889	635	3633	3631
5890	874	3642	3633
5891	639	3631	3642
5892	3633	3642	3631
5893	153	1877	1875
5894	876	4011	1877
5895	872	1875	4011
5896	1877	4011	1875
5897	279	2626	2627
5898	874	4012	2626
5899	876	2627	4012
5900	2626	4012	2627
5901	152	1869	1871
5902	872	4010	1869
5903	874	1871	4010
5904	1869	4010	1871
5905	876	4012	4011
5906	874	4010	4012
5907	872	4011	4010
5908	4012	4010	4011
5909	24	1192	1193
5910	392	2950	1192
5911	393	1193	2950
5912	1192	2950	1193
5913	184	2057	2054
5914	966	2952	2057
5915	392	2054	2952
5916	2057	2952	2054
5917	185	2060	2063
5918	393	2954	2060
5919	966	2063	2954
5920	2060	2954	2063
5921	392	2952	2950
5922	966	2954	2952
5923	393	2950	2954
5924	2952	2954	2950
5925	72	1451	1447
5926	651	3667	1451
5927	647	1447	3667
5928	1451	3667	1447
5929	282	2644	2642
5930	968	3678	2644
5931	651	2642	3678
5932	2644	3678	2642
5933	184	2055	2059
5934	647	3669	2055

5935	968	2059	3669
5936	2055	3669	2059
5937	651	3678	3667
5938	968	3669	3678
5939	647	3667	3669
5940	3678	3669	3667
5941	74	1459	1463
5942	659	3703	1459
5943	663	1463	3703
5944	1459	3703	1463
5945	185	2065	2061
5946	970	3705	2065
5947	659	2061	3705
5948	2065	3705	2061
5949	282	2643	2645
5950	663	3714	2643
5951	970	2645	3714
5952	2643	3714	2645
5953	659	3705	3703
5954	970	3714	3705
5955	663	3703	3714
5956	3705	3714	3703
5957	184	2059	2057
5958	968	4106	2059
5959	966	2057	4106
5960	2059	4106	2057
5961	282	2645	2644
5962	970	4108	2645
5963	968	2644	4108
5964	2645	4108	2644
5965	185	2063	2065
5966	966	4107	2063
5967	970	2065	4107
5968	2063	4107	2065
5969	968	4108	4106
5970	970	4107	4108
5971	966	4106	4107
5972	4108	4107	4106
5973	17	1154	1156
5974	354	2842	1154
5975	356	1156	2842
5976	1154	2842	1156
5977	146	1837	1834
5978	858	2844	1837
5979	354	1834	2844
5980	1837	2844	1834
5981	148	1846	1849
5982	356	2849	1846
5983	858	1849	2849
5984	1846	2849	1849
5985	354	2844	2842
5986	858	2849	2844
5987	356	2842	2849
5988	2844	2849	2842
5989	70	1438	1434
5990	638	3627	1438
5991	634	1434	3627
5992	1438	3627	1434
5993	278	2620	2618

5994	860	3640	2620
5995	638	2618	3640
5996	2620	3640	2618
5997	146	1835	1839
5998	634	3629	1835
5999	860	1839	3629
6000	1835	3629	1839
6001	638	3640	3627
6002	860	3629	3640
6003	634	3627	3629
6004	3640	3629	3627
6005	72	1446	1450
6006	646	3663	1446
6007	650	1450	3663
6008	1446	3663	1450
6009	148	1851	1847
6010	865	3665	1851
6011	646	1847	3665
6012	1851	3665	1847
6013	278	2619	2621
6014	650	3676	2619
6015	865	2621	3676
6016	2619	3676	2621
6017	646	3665	3663
6018	865	3676	3665
6019	650	3663	3676
6020	3665	3676	3663
6021	146	1839	1837
6022	860	3998	1839
6023	858	1837	3998
6024	1839	3998	1837
6025	278	2621	2620
6026	865	4000	2621
6027	860	2620	4000
6028	2621	4000	2620
6029	148	1849	1851
6030	858	3999	1849
6031	865	1851	3999
6032	1849	3999	1851
6033	860	4000	3998
6034	865	3999	4000
6035	858	3998	3999
6036	4000	3999	3998
6037	74	1463	1462
6038	663	3711	1463
6039	662	1462	3711
6040	1463	3711	1462
6041	282	2647	2643
6042	1080	3715	2647
6043	663	2643	3715
6044	2647	3715	2643
6045	279	2625	2629
6046	662	3713	2625
6047	1080	2629	3713
6048	2625	3713	2629
6049	663	3715	3711
6050	1080	3713	3715
6051	662	3711	3713
6052	3715	3713	3711

6053	72	1450	1451
6054	650	3675	1450
6055	651	1451	3675
6056	1450	3675	1451
6057	278	2623	2619
6058	1079	3677	2623
6059	650	2619	3677
6060	2623	3677	2619
6061	282	2642	2646
6062	651	3679	2642
6063	1079	2646	3679
6064	2642	3679	2646
6065	650	3677	3675
6066	1079	3679	3677
6067	651	3675	3679
6068	3677	3679	3675
6069	70	1439	1438
6070	639	3639	1439
6071	638	1438	3639
6072	1439	3639	1438
6073	279	2628	2624
6074	1078	3643	2628
6075	639	2624	3643
6076	2628	3643	2624
6077	278	2618	2622
6078	638	3641	2618
6079	1078	2622	3641
6080	2618	3641	2622
6081	639	3643	3639
6082	1078	3641	3643
6083	638	3639	3641
6084	3643	3641	3639
6085	282	2646	2647
6086	1079	4216	2646
6087	1080	2647	4216
6088	2646	4216	2647
6089	278	2622	2623
6090	1078	4214	2622
6091	1079	2623	4214
6092	2622	4214	2623
6093	279	2629	2628
6094	1080	4215	2629
6095	1078	2628	4215
6096	2629	4215	2628
6097	1079	4214	4216
6098	1078	4215	4214
6099	1080	4216	4215
6100	4214	4215	4216
6101	2	1093	1094
6102	293	2696	1093
6103	294	1094	2696
6104	1093	2696	1094
6105	85	1522	1520
6106	712	2697	1522
6107	293	1520	2697
6108	1522	2697	1520
6109	86	1524	1526
6110	294	2698	1524
6111	712	1526	2698

6112	1524	2698	1526
6113	293	2697	2696
6114	712	2698	2697
6115	294	2696	2698
6116	2697	2698	2696
6117	29	1218	1216
6118	418	3014	1218
6119	416	1216	3014
6120	1218	3014	1216
6121	200	2152	2150
6122	713	3019	2152
6123	418	2150	3019
6124	2152	3019	2150
6125	85	1521	1523
6126	416	3015	1521
6127	713	1523	3015
6128	1521	3015	1523
6129	418	3019	3014
6130	713	3015	3019
6131	416	3014	3015
6132	3019	3015	3014
6133	30	1220	1222
6134	420	3023	1220
6135	422	1222	3023
6136	1220	3023	1222
6137	86	1527	1525
6138	714	3024	1527
6139	420	1525	3024
6140	1527	3024	1525
6141	200	2151	2153
6142	422	3028	2151
6143	714	2153	3028
6144	2151	3028	2153
6145	420	3024	3023
6146	714	3028	3024
6147	422	3023	3028
6148	3024	3028	3023
6149	85	1523	1522
6150	713	3848	1523
6151	712	1522	3848
6152	1523	3848	1522
6153	200	2153	2152
6154	714	3850	2153
6155	713	2152	3850
6156	2153	3850	2152
6157	86	1526	1527
6158	712	3849	1526
6159	714	1527	3849
6160	1526	3849	1527
6161	713	3850	3848
6162	714	3849	3850
6163	712	3848	3849
6164	3850	3849	3848
6165	13	1138	1136
6166	338	2798	1138
6167	336	1136	2798
6168	1138	2798	1136
6169	130	1752	1750
6170	814	2803	1752

6171	338	1750	2803
6172	1752	2803	1750
6173	128	1742	1744
6174	336	2799	1742
6175	814	1744	2799
6176	1742	2799	1744
6177	338	2803	2798
6178	814	2799	2803
6179	336	2798	2799
6180	2803	2799	2798
6181	64	1400	1398
6182	600	3518	1400
6183	598	1398	3518
6184	1400	3518	1398
6185	201	2159	2157
6186	819	3527	2159
6187	600	2157	3527
6188	2159	3527	2157
6189	130	1751	1754
6190	598	3520	1751
6191	819	1754	3520
6192	1751	3520	1754
6193	600	3527	3518
6194	819	3520	3527
6195	598	3518	3520
6196	3527	3520	3518
6197	29	1217	1219
6198	417	3016	1217
6199	419	1219	3016
6200	1217	3016	1219
6201	128	1745	1743
6202	815	3017	1745
6203	417	1743	3017
6204	1745	3017	1743
6205	201	2156	2158
6206	419	3021	2156
6207	815	2158	3021
6208	2156	3021	2158
6209	417	3017	3016
6210	815	3021	3017
6211	419	3016	3021
6212	3017	3021	3016
6213	130	1754	1752
6214	819	3951	1754
6215	814	1752	3951
6216	1754	3951	1752
6217	201	2158	2159
6218	815	3952	2158
6219	819	2159	3952
6220	2158	3952	2159
6221	128	1744	1745
6222	814	3950	1744
6223	815	1745	3950
6224	1744	3950	1745
6225	819	3952	3951
6226	815	3950	3952
6227	814	3951	3950
6228	3952	3950	3951
6229	14	1140	1142

6230	340	2807	1140
6231	342	1142	2807
6232	1140	2807	1142
6233	132	1764	1762
6234	823	2808	1764
6235	340	1762	2808
6236	1764	2808	1762
6237	134	1770	1772
6238	342	2812	1770
6239	823	1772	2812
6240	1770	2812	1772
6241	340	2808	2807
6242	823	2812	2808
6243	342	2807	2812
6244	2808	2812	2807
6245	30	1223	1221
6246	423	3025	1223
6247	421	1221	3025
6248	1223	3025	1221
6249	202	2164	2162
6250	824	3030	2164
6251	423	2162	3030
6252	2164	3030	2162
6253	132	1763	1765
6254	421	3026	1763
6255	824	1765	3026
6256	1763	3026	1765
6257	423	3030	3025
6258	824	3026	3030
6259	421	3025	3026
6260	3030	3026	3025
6261	64	1399	1401
6262	599	3522	1399
6263	601	1401	3522
6264	1399	3522	1401
6265	134	1774	1771
6266	828	3524	1774
6267	599	1771	3524
6268	1774	3524	1771
6269	202	2163	2165
6270	601	3529	2163
6271	828	2165	3529
6272	2163	3529	2165
6273	599	3524	3522
6274	828	3529	3524
6275	601	3522	3529
6276	3524	3529	3522
6277	132	1765	1764
6278	824	3959	1765
6279	823	1764	3959
6280	1765	3959	1764
6281	202	2165	2164
6282	828	3961	2165
6283	824	2164	3961
6284	2165	3961	2164
6285	134	1772	1774
6286	823	3960	1772
6287	828	1774	3960
6288	1772	3960	1774

6289	824	3961	3959
6290	828	3960	3961
6291	823	3959	3960
6292	3961	3960	3959
6293	29	1219	1218
6294	419	3018	1219
6295	418	1218	3018
6296	1219	3018	1218
6297	201	2160	2156
6298	1000	3022	2160
6299	419	2156	3022
6300	2160	3022	2156
6301	200	2150	2154
6302	418	3020	2150
6303	1000	2154	3020
6304	2150	3020	2154
6305	419	3022	3018
6306	1000	3020	3022
6307	418	3018	3020
6308	3022	3020	3018
6309	64	1401	1400
6310	601	3526	1401
6311	600	1400	3526
6312	1401	3526	1400
6313	202	2167	2163
6314	1002	3530	2167
6315	601	2163	3530
6316	2167	3530	2163
6317	201	2157	2161
6318	600	3528	2157
6319	1002	2161	3528
6320	2157	3528	2161
6321	601	3530	3526
6322	1002	3528	3530
6323	600	3526	3528
6324	3530	3528	3526
6325	30	1222	1223
6326	422	3027	1222
6327	423	1223	3027
6328	1222	3027	1223
6329	200	2155	2151
6330	1001	3029	2155
6331	422	2151	3029
6332	2155	3029	2151
6333	202	2162	2166
6334	423	3031	2162
6335	1001	2166	3031
6336	2162	3031	2166
6337	422	3029	3027
6338	1001	3031	3029
6339	423	3027	3031
6340	3029	3031	3027
6341	201	2161	2160
6342	1002	4137	2161
6343	1000	2160	4137
6344	2161	4137	2160
6345	202	2166	2167
6346	1001	4138	2166
6347	1002	2167	4138

6348	2166	4138	2167
6349	200	2154	2155
6350	1000	4136	2154
6351	1001	2155	4136
6352	2154	4136	2155
6353	1002	4138	4137
6354	1001	4136	4138
6355	1000	4137	4136
6356	4138	4136	4137
6357	5	1103	1101
6358	303	2710	1103
6359	301	1101	2710
6360	1103	2710	1101
6361	95	1566	1564
6362	726	2715	1566
6363	303	1564	2715
6364	1566	2715	1564
6365	93	1554	1556
6366	301	2711	1554
6367	726	1556	2711
6368	1554	2711	1556
6369	303	2715	2710
6370	726	2711	2715
6371	301	2710	2711
6372	2715	2711	2710
6373	39	1262	1260
6374	462	3122	1262
6375	460	1260	3122
6376	1262	3122	1260
6377	214	2237	2235
6378	731	3131	2237
6379	462	2235	3131
6380	2237	3131	2235
6381	95	1565	1568
6382	460	3124	1565
6383	731	1568	3124
6384	1565	3124	1568
6385	462	3131	3122
6386	731	3124	3131
6387	460	3122	3124
6388	3131	3124	3122
6389	37	1250	1252
6390	450	3095	1250
6391	452	1252	3095
6392	1250	3095	1252
6393	93	1557	1555
6394	727	3096	1557
6395	450	1555	3096
6396	1557	3096	1555
6397	214	2234	2236
6398	452	3100	2234
6399	727	2236	3100
6400	2234	3100	2236
6401	450	3096	3095
6402	727	3100	3096
6403	452	3095	3100
6404	3096	3100	3095
6405	95	1568	1566
6406	731	3864	1568

6407	726	1566	3864
6408	1568	3864	1566
6409	214	2236	2237
6410	727	3865	2236
6411	731	2237	3865
6412	2236	3865	2237
6413	93	1556	1557
6414	726	3863	1556
6415	727	1557	3863
6416	1556	3863	1557
6417	731	3865	3864
6418	727	3863	3865
6419	726	3864	3863
6420	3865	3863	3864
6421	21	1174	1172
6422	374	2888	1174
6423	372	1172	2888
6424	1174	2888	1172
6425	166	1948	1946
6426	904	2897	1948
6427	374	1946	2897
6428	1948	2897	1946
6429	164	1934	1936
6430	372	2890	1934
6431	904	1936	2890
6432	1934	2890	1936
6433	374	2897	2888
6434	904	2890	2897
6435	372	2888	2890
6436	2897	2890	2888
6437	65	1407	1405
6438	607	3540	1407
6439	605	1405	3540
6440	1407	3540	1405
6441	219	2267	2265
6442	913	3547	2267
6443	607	2265	3547
6444	2267	3547	2265
6445	166	1947	1950
6446	605	3542	1947
6447	913	1950	3542
6448	1947	3542	1950
6449	607	3547	3540
6450	913	3542	3547
6451	605	3540	3542
6452	3547	3542	3540
6453	39	1261	1264
6454	461	3126	1261
6455	464	1264	3126
6456	1261	3126	1264
6457	164	1938	1935
6458	906	3128	1938
6459	461	1935	3128
6460	1938	3128	1935
6461	219	2264	2266
6462	464	3136	2264
6463	906	2266	3136
6464	2264	3136	2266
6465	461	3128	3126

6466	906	3136	3128
6467	464	3126	3136
6468	3128	3136	3126
6469	166	1950	1948
6470	913	4041	1950
6471	904	1948	4041
6472	1950	4041	1948
6473	219	2266	2267
6474	906	4044	2266
6475	913	2267	4044
6476	2266	4044	2267
6477	164	1936	1938
6478	904	4040	1936
6479	906	1938	4040
6480	1936	4040	1938
6481	913	4044	4041
6482	906	4040	4044
6483	904	4041	4040
6484	4044	4040	4041
6485	13	1137	1139
6486	337	2800	1137
6487	339	1139	2800
6488	1137	2800	1139
6489	129	1748	1746
6490	816	2801	1748
6491	337	1746	2801
6492	1748	2801	1746
6493	131	1756	1758
6494	339	2805	1756
6495	816	1758	2805
6496	1756	2805	1758
6497	337	2801	2800
6498	816	2805	2801
6499	339	2800	2805
6500	2801	2805	2800
6501	37	1253	1251
6502	453	3097	1253
6503	451	1251	3097
6504	1253	3097	1251
6505	215	2242	2240
6506	817	3102	2242
6507	453	2240	3102
6508	2242	3102	2240
6509	129	1747	1749
6510	451	3098	1747
6511	817	1749	3098
6512	1747	3098	1749
6513	453	3102	3097
6514	817	3098	3102
6515	451	3097	3098
6516	3102	3098	3097
6517	65	1404	1406
6518	604	3536	1404
6519	606	1406	3536
6520	1404	3536	1406
6521	131	1760	1757
6522	821	3538	1760
6523	604	1757	3538
6524	1760	3538	1757

6525	215	2241	2243
6526	606	3545	2241
6527	821	2243	3545
6528	2241	3545	2243
6529	604	3538	3536
6530	821	3545	3538
6531	606	3536	3545
6532	3538	3545	3536
6533	129	1749	1748
6534	817	3953	1749
6535	816	1748	3953
6536	1749	3953	1748
6537	131	1758	1760
6538	816	3954	1758
6539	821	1760	3954
6540	1758	3954	1760
6541	39	1264	1262
6542	464	3130	1264
6543	462	1262	3130
6544	1264	3130	1262
6545	219	2268	2264
6546	1016	3137	2268
6547	464	2264	3137
6548	2268	3137	2264
6549	214	2235	2239
6550	462	3132	2235
6551	1016	2239	3132
6552	2235	3132	2239
6553	464	3137	3130
6554	1016	3132	3137
6555	462	3130	3132
6556	3137	3132	3130
6557	65	1406	1407
6558	606	3544	1406
6559	607	1407	3544
6560	1406	3544	1407
6561	215	2245	2241
6562	1017	3546	2245
6563	606	2241	3546
6564	2245	3546	2241
6565	219	2265	2269
6566	607	3548	2265
6567	1017	2269	3548
6568	2265	3548	2269
6569	606	3546	3544
6570	1017	3548	3546
6571	607	3544	3548
6572	3546	3548	3544
6573	37	1252	1253
6574	452	3099	1252
6575	453	1253	3099
6576	1252	3099	1253
6577	214	2238	2234
6578	1015	3101	2238
6579	452	2234	3101
6580	2238	3101	2234
6581	215	2240	2244
6582	453	3103	2240
6583	1015	2244	3103

6584	2240	3103	2244
6585	452	3101	3099
6586	1015	3103	3101
6587	453	3099	3103
6588	3101	3103	3099
6589	219	2269	2268
6590	1017	4153	2269
6591	1016	2268	4153
6592	2269	4153	2268
6593	215	2244	2245
6594	1015	4152	2244
6595	1017	2245	4152
6596	2244	4152	2245
6597	214	2239	2238
6598	1016	4151	2239
6599	1015	2238	4151
6600	2239	4151	2238
6601	1017	4152	4153
6602	1015	4151	4152
6603	1016	4153	4151
6604	4152	4151	4153
6605	8	1114	1116
6606	314	2744	1114
6607	316	1116	2744
6608	1114	2744	1116
6609	106	1628	1626
6610	760	2745	1628
6611	314	1626	2745
6612	1628	2745	1626
6613	108	1634	1636
6614	316	2749	1634
6615	760	1636	2749
6616	1634	2749	1636
6617	314	2745	2744
6618	760	2749	2745
6619	316	2744	2749
6620	2745	2749	2744
6621	50	1324	1322
6622	524	3302	1324
6623	522	1322	3302
6624	1324	3302	1322
6625	248	2440	2438
6626	761	3307	2440
6627	524	2438	3307
6628	2440	3307	2438
6629	106	1627	1629
6630	522	3303	1627
6631	761	1629	3303
6632	1627	3303	1629
6633	524	3307	3302
6634	761	3303	3307
6635	522	3302	3303
6636	3307	3303	3302
6637	52	1330	1332
6638	530	3320	1330
6639	532	1332	3320
6640	1330	3320	1332
6641	108	1638	1635
6642	765	3322	1638

6643	530	1635	3322
6644	1638	3322	1635
6645	248	2439	2441
6646	532	3329	2439
6647	765	2441	3329
6648	2439	3329	2441
6649	530	3322	3320
6650	765	3329	3322
6651	532	3320	3329
6652	3322	3329	3320
6653	106	1629	1628
6654	761	3896	1629
6655	760	1628	3896
6656	1629	3896	1628
6657	248	2441	2440
6658	765	3898	2441
6659	761	2440	3898
6660	2441	3898	2440
6661	108	1636	1638
6662	760	3897	1636
6663	765	1638	3897
6664	1636	3897	1638
6665	761	3898	3896
6666	765	3897	3898
6667	760	3896	3897
6668	3898	3897	3896
6669	14	1143	1141
6670	343	2809	1143
6671	341	1141	2809
6672	1143	2809	1141
6673	135	1778	1776
6674	825	2814	1778
6675	343	1776	2814
6676	1778	2814	1776
6677	133	1766	1768
6678	341	2810	1766
6679	825	1768	2810
6680	1766	2810	1768
6681	343	2814	2809
6682	825	2810	2814
6683	341	2809	2810
6684	2814	2810	2809
6685	66	1412	1410
6686	612	3554	1412
6687	610	1410	3554
6688	1412	3554	1410
6689	249	2447	2445
6690	830	3563	2447
6691	612	2445	3563
6692	2447	3563	2445
6693	135	1777	1780
6694	610	3556	1777
6695	830	1780	3556
6696	1777	3556	1780
6697	612	3563	3554
6698	830	3556	3563
6699	610	3554	3556
6700	3563	3556	3554
6701	50	1323	1325

6702	523	3304	1323
6703	525	1325	3304
6704	1323	3304	1325
6705	133	1769	1767
6706	826	3305	1769
6707	523	1767	3305
6708	1769	3305	1767
6709	249	2444	2446
6710	525	3309	2444
6711	826	2446	3309
6712	2444	3309	2446
6713	523	3305	3304
6714	826	3309	3305
6715	525	3304	3309
6716	3305	3309	3304
6717	135	1780	1778
6718	830	3963	1780
6719	825	1778	3963
6720	1780	3963	1778
6721	249	2446	2447
6722	826	3964	2446
6723	830	2447	3964
6724	2446	3964	2447
6725	133	1768	1769
6726	825	3962	1768
6727	826	1769	3962
6728	1768	3962	1769
6729	830	3964	3963
6730	826	3962	3964
6731	825	3963	3962
6732	3964	3962	3963
6733	21	1173	1175
6734	373	2892	1173
6735	375	1175	2892
6736	1173	2892	1175
6737	165	1942	1940
6738	908	2894	1942
6739	373	1940	2894
6740	1942	2894	1940
6741	167	1952	1954
6742	375	2899	1952
6743	908	1954	2899
6744	1952	2899	1954
6745	373	2894	2892
6746	908	2899	2894
6747	375	2892	2899
6748	2894	2899	2892
6749	52	1334	1331
6750	534	3324	1334
6751	531	1331	3324
6752	1334	3324	1331
6753	253	2470	2468
6754	910	3334	2470
6755	534	2468	3334
6756	2470	3334	2468
6757	165	1941	1944
6758	531	3326	1941
6759	910	1944	3326
6760	1941	3326	1944

6761	534	3334	3324
6762	910	3326	3334
6763	531	3324	3326
6764	3334	3326	3324
6765	66	1411	1413
6766	611	3558	1411
6767	613	1413	3558
6768	1411	3558	1413
6769	167	1956	1953
6770	915	3560	1956
6771	611	1953	3560
6772	1956	3560	1953
6773	253	2469	2471
6774	613	3565	2469
6775	915	2471	3565
6776	2469	3565	2471
6777	611	3560	3558
6778	915	3565	3560
6779	613	3558	3565
6780	3560	3565	3558
6781	165	1944	1942
6782	910	4046	1944
6783	908	1942	4046
6784	1944	4046	1942
6785	253	2471	2470
6786	915	4050	2471
6787	910	2470	4050
6788	2471	4050	2470
6789	167	1954	1956
6790	908	4047	1954
6791	915	1956	4047
6792	1954	4047	1956
6793	910	4050	4046
6794	915	4047	4050
6795	908	4046	4047
6796	4050	4047	4046
6797	50	1325	1324
6798	525	3306	1325
6799	524	1324	3306
6800	1325	3306	1324
6801	249	2448	2444
6802	1048	3310	2448
6803	525	2444	3310
6804	2448	3310	2444
6805	248	2438	2442
6806	524	3308	2438
6807	1048	2442	3308
6808	2438	3308	2442
6809	525	3310	3306
6810	1048	3308	3310
6811	524	3306	3308
6812	3310	3308	3306
6813	66	1413	1412
6814	613	3562	1413
6815	612	1412	3562
6816	1413	3562	1412
6817	253	2473	2469
6818	1050	3566	2473
6819	613	2469	3566

6820	2473	3566	2469
6821	249	2445	2449
6822	612	3564	2445
6823	1050	2449	3564
6824	2445	3564	2449
6825	613	3566	3562
6826	1050	3564	3566
6827	612	3562	3564
6828	3566	3564	3562
6829	52	1332	1334
6830	532	3328	1332
6831	534	1334	3328
6832	1332	3328	1334
6833	248	2443	2439
6834	1049	3330	2443
6835	532	2439	3330
6836	2443	3330	2439
6837	253	2468	2472
6838	534	3335	2468
6839	1049	2472	3335
6840	2468	3335	2472
6841	532	3330	3328
6842	1049	3335	3330
6843	534	3328	3335
6844	3330	3335	3328
6845	249	2449	2448
6846	1050	4185	2449
6847	1048	2448	4185
6848	2449	4185	2448
6849	253	2472	2473
6850	1049	4186	2472
6851	1050	2473	4186
6852	2472	4186	2473
6853	248	2442	2443
6854	1048	4184	2442
6855	1049	2443	4184
6856	2442	4184	2443
6857	1050	4186	4185
6858	1049	4184	4186
6859	1048	4185	4184
6860	4186	4184	4185
6861	13	1139	1138
6862	339	2802	1139
6863	338	1138	2802
6864	1139	2802	1138
6865	130	1750	1753
6866	338	2804	1750
6867	818	1753	2804
6868	1750	2804	1753
6869	65	1408	1404
6870	608	3537	1408
6871	604	1404	3537
6872	1408	3537	1404
6873	272	2585	2583
6874	822	3550	2585
6875	608	2583	3550
6876	2585	3550	2583
6877	131	1757	1761
6878	604	3539	1757

6879	822	1761	3539
6880	1757	3539	1761
6881	608	3550	3537
6882	822	3539	3550
6883	604	3537	3539
6884	3550	3539	3537
6885	64	1398	1402
6886	598	3519	1398
6887	602	1402	3519
6888	1398	3519	1402
6889	130	1755	1751
6890	820	3521	1755
6891	598	1751	3521
6892	1755	3521	1751
6893	272	2582	2584
6894	602	3532	2582
6895	820	2584	3532
6896	2582	3532	2584
6897	598	3521	3519
6898	820	3532	3521
6899	602	3519	3532
6900	3521	3532	3519
6901	131	1761	1759
6902	822	3957	1761
6903	818	1759	3957
6904	1761	3957	1759
6905	272	2584	2585
6906	820	3958	2584
6907	822	2585	3958
6908	2584	3958	2585
6909	130	1753	1755
6910	818	3956	1753
6911	820	1755	3956
6912	1753	3956	1755
6913	822	3958	3957
6914	820	3956	3958
6915	818	3957	3956
6916	3958	3956	3957
6917	21	1175	1174
6918	375	2896	1175
6919	374	1174	2896
6920	1175	2896	1174
6921	167	1955	1952
6922	912	2900	1955
6923	375	1952	2900
6924	1955	2900	1952
6925	166	1946	1949
6926	374	2898	1946
6927	912	1949	2898
6928	1946	2898	1949
6929	375	2900	2896
6930	912	2898	2900
6931	374	2896	2898
6932	2900	2898	2896
6933	66	1415	1411
6934	615	3559	1415
6935	611	1411	3559
6936	1415	3559	1411
6937	274	2597	2595

6938	916	3570	2597
6939	615	2595	3570
6940	2597	3570	2595
6941	167	1953	1957
6942	611	3561	1953
6943	916	1957	3561
6944	1953	3561	1957
6945	615	3570	3559
6946	916	3561	3570
6947	611	3559	3561
6948	3570	3561	3559
6949	65	1405	1409
6950	605	3541	1405
6951	609	1409	3541
6952	1405	3541	1409
6953	166	1951	1947
6954	914	3543	1951
6955	605	1947	3543
6956	1951	3543	1947
6957	274	2594	2596
6958	609	3552	2594
6959	914	2596	3552
6960	2594	3552	2596
6961	605	3543	3541
6962	914	3552	3543
6963	609	3541	3552
6964	3543	3552	3541
6965	167	1957	1955
6966	916	4053	1957
6967	912	1955	4053
6968	1957	4053	1955
6969	274	2596	2597
6970	914	4054	2596
6971	916	2597	4054
6972	2596	4054	2597
6973	166	1949	1951
6974	912	4052	1949
6975	914	1951	4052
6976	1949	4052	1951
6977	916	4054	4053
6978	914	4052	4054
6979	912	4053	4052
6980	4054	4052	4053
6981	14	1142	1143
6982	342	2811	1142
6983	343	1143	2811
6984	1142	2811	1143
6985	134	1773	1770
6986	827	2813	1773
6987	342	1770	2813
6988	1773	2813	1770
6989	135	1776	1779
6990	343	2815	1776
6991	827	1779	2815
6992	1776	2815	1779
6993	342	2813	2811
6994	827	2815	2813
6995	343	2811	2815
6996	2813	2815	2811

6997	64	1403	1399
6998	603	3523	1403
6999	599	1399	3523
7000	1403	3523	1399
7001	273	2590	2588
7002	829	3534	2590
7003	603	2588	3534
7004	2590	3534	2588
7005	134	1771	1775
7006	599	3525	1771
7007	829	1775	3525
7008	1771	3525	1775
7009	603	3534	3523
7010	829	3525	3534
7011	599	3523	3525
7012	3534	3525	3523
7013	66	1410	1414
7014	610	3555	1410
7015	614	1414	3555
7016	1410	3555	1414
7017	135	1781	1777
7018	831	3557	1781
7019	610	1777	3557
7020	1781	3557	1777
7021	273	2589	2591
7022	614	3568	2589
7023	831	2591	3568
7024	2589	3568	2591
7025	610	3557	3555
7026	831	3568	3557
7027	614	3555	3568
7028	3557	3568	3555
7029	134	1775	1773
7030	829	3965	1775
7031	827	1773	3965
7032	1775	3965	1773
7033	273	2591	2590
7034	831	3967	2591
7035	829	2590	3967
7036	2591	3967	2590
7037	135	1779	1781
7038	827	3966	1779
7039	831	1781	3966
7040	1779	3966	1781
7041	829	3967	3965
7042	831	3966	3967
7043	827	3965	3966
7044	3967	3966	3965
7045	65	1409	1408
7046	609	3549	1409
7047	608	1408	3549
7048	1409	3549	1408
7049	274	2598	2594
7050	1073	3553	2598
7051	609	2594	3553
7052	2598	3553	2594
7053	272	2583	2587
7054	608	3551	2583
7055	1073	2587	3551

7056	2583	3551	2587
7057	609	3553	3549
7058	1073	3551	3553
7059	608	3549	3551
7060	3553	3551	3549
7061	66	1414	1415
7062	614	3567	1414
7063	615	1415	3567
7064	1414	3567	1415
7065	273	2593	2589
7066	1074	3569	2593
7067	614	2589	3569
7068	2593	3569	2589
7069	274	2595	2599
7070	615	3571	2595
7071	1074	2599	3571
7072	2595	3571	2599
7073	614	3569	3567
7074	1074	3571	3569
7075	615	3567	3571
7076	3569	3571	3567
7077	64	1402	1403
7078	602	3531	1402
7079	603	1403	3531
7080	1402	3531	1403
7081	272	2586	2582
7082	1072	3533	2586
7083	602	2582	3533
7084	2586	3533	2582
7085	273	2588	2592
7086	603	3535	2588
7087	1072	2592	3535
7088	2588	3535	2592
7089	602	3533	3531
7090	1072	3535	3533
7091	603	3531	3535
7092	3533	3535	3531
7093	274	2599	2598
7094	1074	4210	2599
7095	1073	2598	4210
7096	2599	4210	2598
7097	273	2592	2593
7098	1072	4209	2592
7099	1074	2593	4209
7100	2592	4209	2593
7101	272	2587	2586
7102	1073	4208	2587
7103	1072	2586	4208
7104	2587	4208	2586
7105	1074	4209	4210
7106	1072	4208	4209
7107	1073	4210	4208
7108	4209	4208	4210
7109	7	1112	1110
7110	312	2734	1112
7111	310	1110	2734
7112	1112	2734	1110
7113	104	1617	1614
7114	750	2741	1617

7115	312	1614	2741
7116	1617	2741	1614
7117	102	1602	1605
7118	310	2736	1602
7119	750	1605	2736
7120	1602	2736	1605
7121	312	2741	2734
7122	750	2736	2741
7123	310	2734	2736
7124	2741	2736	2734
7125	48	1313	1310
7126	513	3267	1313
7127	510	1310	3267
7128	1313	3267	1310
7129	238	2381	2379
7130	757	3278	2381
7131	513	2379	3278
7132	2381	3278	2379
7133	104	1615	1619
7134	510	3269	1615
7135	757	1619	3269
7136	1615	3269	1619
7137	513	3278	3267
7138	757	3269	3278
7139	510	3267	3269
7140	3278	3269	3267
7141	46	1298	1301
7142	498	3231	1298
7143	501	1301	3231
7144	1298	3231	1301
7145	102	1607	1603
7146	752	3233	1607
7147	498	1603	3233
7148	1607	3233	1603
7149	238	2378	2380
7150	501	3242	2378
7151	752	2380	3242
7152	2378	3242	2380
7153	498	3233	3231
7154	752	3242	3233
7155	501	3231	3242
7156	3233	3242	3231
7157	104	1619	1617
7158	757	3891	1619
7159	750	1617	3891
7160	1619	3891	1617
7161	238	2380	2381
7162	752	3892	2380
7163	757	2381	3892
7164	2380	3892	2381
7165	102	1605	1607
7166	750	3890	1605
7167	752	1607	3890
7168	1605	3890	1607
7169	757	3892	3891
7170	752	3890	3892
7171	750	3891	3890
7172	3892	3890	3891
7173	24	1194	1190

7174	394	2943	1194
7175	390	1190	2943
7176	1194	2943	1190
7177	186	2068	2066
7178	959	2956	2068
7179	394	2066	2956
7180	2068	2956	2066
7181	182	2042	2045
7182	390	2945	2042
7183	959	2045	2945
7184	2042	2945	2045
7185	394	2956	2943
7186	959	2945	2956
7187	390	2943	2945
7188	2956	2945	2943
7189	77	1479	1477
7190	679	3756	1479
7191	677	1477	3756
7192	1479	3756	1477
7193	245	2423	2421
7194	972	3763	2423
7195	679	2421	3763
7196	2423	3763	2421
7197	186	2067	2070
7198	677	3758	2067
7199	972	2070	3758
7200	2067	3758	2070
7201	679	3763	3756
7202	972	3758	3763
7203	677	3756	3758
7204	3763	3758	3756
7205	48	1311	1315
7206	511	3271	1311
7207	515	1315	3271
7208	1311	3271	1315
7209	182	2047	2043
7210	961	3273	2047
7211	511	2043	3273
7212	2047	3273	2043
7213	245	2420	2422
7214	515	3282	2420
7215	961	2422	3282
7216	2420	3282	2422
7217	511	3273	3271
7218	961	3282	3273
7219	515	3271	3282
7220	3273	3282	3271
7221	186	2070	2068
7222	972	4097	2070
7223	959	2068	4097
7224	2070	4097	2068
7225	245	2422	2423
7226	961	4099	2422
7227	972	2423	4099
7228	2422	4099	2423
7229	182	2045	2047
7230	959	4096	2045
7231	961	2047	4096
7232	2045	4096	2047

7233	972	4099	4097
7234	961	4096	4099
7235	959	4097	4096
7236	4099	4096	4097
7237	20	1167	1171
7238	367	2875	1167
7239	371	1171	2875
7240	1167	2875	1171
7241	159	1907	1904
7242	891	2877	1907
7243	367	1904	2877
7244	1907	2877	1904
7245	163	1928	1930
7246	371	2886	1928
7247	891	1930	2886
7248	1928	2886	1930
7249	367	2877	2875
7250	891	2886	2877
7251	371	2875	2886
7252	2877	2886	2875
7253	46	1303	1299
7254	503	3235	1303
7255	499	1299	3235
7256	1303	3235	1299
7257	240	2392	2390
7258	893	3246	2392
7259	503	2390	3246
7260	2392	3246	2390
7261	159	1905	1909
7262	499	3237	1905
7263	893	1909	3237
7264	1905	3237	1909
7265	503	3246	3235
7266	893	3237	3246
7267	499	3235	3237
7268	3246	3237	3235
7269	77	1476	1478
7270	676	3752	1476
7271	678	1478	3752
7272	1476	3752	1478
7273	163	1932	1929
7274	902	3754	1932
7275	676	1929	3754
7276	1932	3754	1929
7277	240	2391	2393
7278	678	3761	2391
7279	902	2393	3761
7280	2391	3761	2393
7281	676	3754	3752
7282	902	3761	3754
7283	678	3752	3761
7284	3754	3761	3752
7285	159	1909	1907
7286	893	4030	1909
7287	891	1907	4030
7288	1909	4030	1907
7289	240	2393	2392
7290	902	4033	2393
7291	893	2392	4033

7292	2393	4033	2392
7293	163	1930	1932
7294	891	4031	1930
7295	902	1932	4031
7296	1930	4031	1932
7297	893	4033	4030
7298	902	4031	4033
7299	891	4030	4031
7300	4033	4031	4030
7301	48	1315	1313
7302	515	3277	1315
7303	513	1313	3277
7304	1315	3277	1313
7305	245	2424	2420
7306	1043	3283	2424
7307	515	2420	3283
7308	2424	3283	2420
7309	238	2379	2383
7310	513	3279	2379
7311	1043	2383	3279
7312	2379	3279	2383
7313	515	3283	3277
7314	1043	3279	3283
7315	513	3277	3279
7316	3283	3279	3277
7317	77	1478	1479
7318	678	3760	1478
7319	679	1479	3760
7320	1478	3760	1479
7321	240	2395	2391
7322	1044	3762	2395
7323	678	2391	3762
7324	2395	3762	2391
7325	245	2421	2425
7326	679	3764	2421
7327	1044	2425	3764
7328	2421	3764	2425
7329	678	3762	3760
7330	1044	3764	3762
7331	679	3760	3764
7332	3762	3764	3760
7333	46	1301	1303
7334	501	3241	1301
7335	503	1303	3241
7336	1301	3241	1303
7337	238	2382	2378
7338	1042	3243	2382
7339	501	2378	3243
7340	2382	3243	2378
7341	240	2390	2394
7342	503	3247	2390
7343	1042	2394	3247
7344	2390	3247	2394
7345	501	3243	3241
7346	1042	3247	3243
7347	503	3241	3247
7348	3243	3247	3241
7349	245	2425	2424
7350	1044	4180	2425

7351	1043	2424	4180
7352	2425	4180	2424
7353	240	2394	2395
7354	1042	4179	2394
7355	1044	2395	4179
7356	2394	4179	2395
7357	238	2383	2382
7358	1043	4178	2383
7359	1042	2382	4178
7360	2383	4178	2382
7361	1044	4179	4180
7362	1042	4178	4179
7363	1043	4180	4178
7364	4179	4178	4180
7365	8	1116	1117
7366	316	2748	1116
7367	317	1117	2748
7368	1116	2748	1117
7369	108	1637	1634
7370	764	2750	1637
7371	316	1634	2750
7372	1637	2750	1634
7373	109	1640	1643
7374	317	2752	1640
7375	764	1643	2752
7376	1640	2752	1643
7377	316	2750	2748
7378	764	2752	2750
7379	317	2748	2752
7380	2750	2752	2748
7381	52	1333	1330
7382	533	3321	1333
7383	530	1330	3321
7384	1333	3321	1330
7385	252	2464	2462
7386	766	3332	2464
7387	533	2462	3332
7388	2464	3332	2462
7389	108	1635	1639
7390	530	3323	1635
7391	766	1639	3323
7392	1635	3323	1639
7393	533	3332	3321
7394	766	3323	3332
7395	530	3321	3323
7396	3332	3323	3321
7397	53	1336	1339
7398	536	3339	1336
7399	539	1339	3339
7400	1336	3339	1339
7401	109	1645	1641
7402	768	3341	1645
7403	536	1641	3341
7404	1645	3341	1641
7405	252	2463	2465
7406	539	3350	2463
7407	768	2465	3350
7408	2463	3350	2465
7409	536	3341	3339

7410	768	3350	3341
7411	539	3339	3350
7412	3341	3350	3339
7413	108	1639	1637
7414	766	3902	1639
7415	764	1637	3902
7416	1639	3902	1637
7417	252	2465	2464
7418	768	3904	2465
7419	766	2464	3904
7420	2465	3904	2464
7421	109	1643	1645
7422	764	3903	1643
7423	768	1645	3903
7424	1643	3903	1645
7425	766	3904	3902
7426	768	3903	3904
7427	764	3902	3903
7428	3904	3903	3902
7429	21	1177	1173
7430	377	2893	1177
7431	373	1173	2893
7432	1177	2893	1173
7433	169	1966	1964
7434	909	2904	1966
7435	377	1964	2904
7436	1966	2904	1964
7437	165	1940	1943
7438	373	2895	1940
7439	909	1943	2895
7440	1940	2895	1943
7441	377	2904	2893
7442	909	2895	2904
7443	373	2893	2895
7444	2904	2895	2893
7445	78	1484	1482
7446	684	3770	1484
7447	682	1482	3770
7448	1484	3770	1482
7449	254	2477	2475
7450	920	3779	2477
7451	684	2475	3779
7452	2477	3779	2475
7453	169	1965	1968
7454	682	3772	1965
7455	920	1968	3772
7456	1965	3772	1968
7457	684	3779	3770
7458	920	3772	3779
7459	682	3770	3772
7460	3779	3772	3770
7461	52	1331	1335
7462	531	3325	1331
7463	535	1335	3325
7464	1331	3325	1335
7465	165	1945	1941
7466	911	3327	1945
7467	531	1941	3327
7468	1945	3327	1941

7469	254	2474	2476
7470	535	3336	2474
7471	911	2476	3336
7472	2474	3336	2476
7473	531	3327	3325
7474	911	3336	3327
7475	535	3325	3336
7476	3327	3336	3325
7477	169	1968	1966
7478	920	4049	1968
7479	909	1966	4049
7480	1968	4049	1966
7481	254	2476	2477
7482	911	4051	2476
7483	920	2477	4051
7484	2476	4051	2477
7485	165	1943	1945
7486	909	4048	1943
7487	911	1945	4048
7488	1943	4048	1945
7489	920	4051	4049
7490	911	4048	4051
7491	909	4049	4048
7492	4051	4048	4049
7493	24	1191	1195
7494	391	2947	1191
7495	395	1195	2947
7496	1191	2947	1195
7497	183	2051	2048
7498	963	2949	2051
7499	391	2048	2949
7500	2051	2949	2048
7501	187	2072	2074
7502	395	2958	2072
7503	963	2074	2958
7504	2072	2958	2074
7505	391	2949	2947
7506	963	2958	2949
7507	395	2947	2958
7508	2949	2958	2947
7509	53	1341	1337
7510	541	3343	1341
7511	537	1337	3343
7512	1341	3343	1337
7513	256	2488	2486
7514	965	3354	2488
7515	541	2486	3354
7516	2488	3354	2486
7517	183	2049	2053
7518	537	3345	2049
7519	965	2053	3345
7520	2049	3345	2053
7521	541	3354	3343
7522	965	3345	3354
7523	537	3343	3345
7524	3354	3345	3343
7525	78	1483	1485
7526	683	3774	1483
7527	685	1485	3774

7528	1483	3774	1485
7529	187	2076	2073
7530	974	3776	2076
7531	683	2073	3776
7532	2076	3776	2073
7533	256	2487	2489
7534	685	3781	2487
7535	974	2489	3781
7536	2487	3781	2489
7537	683	3776	3774
7538	974	3781	3776
7539	685	3774	3781
7540	3776	3781	3774
7541	183	2053	2051
7542	965	4102	2053
7543	963	2051	4102
7544	2053	4102	2051
7545	256	2489	2488
7546	974	4105	2489
7547	965	2488	4105
7548	2489	4105	2488
7549	187	2074	2076
7550	963	4103	2074
7551	974	2076	4103
7552	2074	4103	2076
7553	965	4105	4102
7554	974	4103	4105
7555	963	4102	4103
7556	4105	4103	4102
7557	52	1335	1333
7558	535	3331	1335
7559	533	1333	3331
7560	1335	3331	1333
7561	254	2478	2474
7562	1054	3337	2478
7563	535	2474	3337
7564	2478	3337	2474
7565	252	2462	2466
7566	533	3333	2462
7567	1054	2466	3333
7568	2462	3333	2466
7569	535	3337	3331
7570	1054	3333	3337
7571	533	3331	3333
7572	3337	3333	3331
7573	78	1485	1484
7574	685	3778	1485
7575	684	1484	3778
7576	1485	3778	1484
7577	256	2491	2487
7578	1056	3782	2491
7579	685	2487	3782
7580	2491	3782	2487
7581	254	2475	2479
7582	684	3780	2475
7583	1056	2479	3780
7584	2475	3780	2479
7585	685	3782	3778
7586	1056	3780	3782

7587	684	3778	3780
7588	3782	3780	3778
7589	53	1339	1341
7590	539	3349	1339
7591	541	1341	3349
7592	1339	3349	1341
7593	252	2467	2463
7594	1055	3351	2467
7595	539	2463	3351
7596	2467	3351	2463
7597	256	2486	2490
7598	541	3355	2486
7599	1055	2490	3355
7600	2486	3355	2490
7601	539	3351	3349
7602	1055	3355	3351
7603	541	3349	3355
7604	3351	3355	3349
7605	254	2479	2478
7606	1056	4191	2479
7607	1054	2478	4191
7608	2479	4191	2478
7609	256	2490	2491
7610	1055	4192	2490
7611	1056	2491	4192
7612	2490	4192	2491
7613	252	2466	2467
7614	1054	4190	2466
7615	1055	2467	4190
7616	2466	4190	2467
7617	1056	4192	4191
7618	1055	4190	4192
7619	1054	4191	4190
7620	4192	4190	4191
7621	5	1102	1103
7622	302	2712	1102
7623	303	1103	2712
7624	1102	2712	1103
7625	94	1561	1558
7626	728	2714	1561
7627	302	1558	2714
7628	1561	2714	1558
7629	95	1564	1567
7630	303	2716	1564
7631	728	1567	2716
7632	1564	2716	1567
7633	302	2714	2712
7634	728	2716	2714
7635	303	2712	2716
7636	2714	2716	2712
7637	38	1257	1254
7638	457	3105	1257
7639	454	1254	3105
7640	1257	3105	1254
7641	216	2248	2246
7642	730	3116	2248
7643	457	2246	3116
7644	2248	3116	2246
7645	94	1559	1563

7646	454	3107	1559
7647	730	1563	3107
7648	1559	3107	1563
7649	457	3116	3105
7650	730	3107	3116
7651	454	3105	3107
7652	3116	3107	3105
7653	39	1260	1263
7654	460	3123	1260
7655	463	1263	3123
7656	1260	3123	1263
7657	95	1569	1565
7658	732	3125	1569
7659	460	1565	3125
7660	1569	3125	1565
7661	216	2247	2249
7662	463	3134	2247
7663	732	2249	3134
7664	2247	3134	2249
7665	460	3125	3123
7666	732	3134	3125
7667	463	3123	3134
7668	3125	3134	3123
7669	94	1563	1561
7670	730	3866	1563
7671	728	1561	3866
7672	1563	3866	1561
7673	216	2249	2248
7674	732	3868	2249
7675	730	2248	3868
7676	2249	3868	2248
7677	95	1567	1569
7678	728	3867	1567
7679	732	1569	3867
7680	1567	3867	1569
7681	730	3868	3866
7682	732	3867	3868
7683	728	3866	3867
7684	3868	3867	3866
7685	20	1170	1166
7686	370	2871	1170
7687	366	1166	2871
7688	1170	2871	1166
7689	162	1924	1922
7690	887	2884	1924
7691	370	1922	2884
7692	1924	2884	1922
7693	158	1898	1901
7694	366	2873	1898
7695	887	1901	2873
7696	1898	2873	1901
7697	370	2884	2871
7698	887	2873	2884
7699	366	2871	2873
7700	2884	2873	2871
7701	76	1472	1470
7702	672	3734	1472
7703	670	1470	3734
7704	1472	3734	1470

7705	218	2261	2259
7706	900	3743	2261
7707	672	2259	3743
7708	2261	3743	2259
7709	162	1923	1926
7710	670	3736	1923
7711	900	1926	3736
7712	1923	3736	1926
7713	672	3743	3734
7714	900	3736	3743
7715	670	3734	3736
7716	3743	3736	3734
7717	38	1255	1259
7718	455	3109	1255
7719	459	1259	3109
7720	1255	3109	1259
7721	158	1903	1899
7722	889	3111	1903
7723	455	1899	3111
7724	1903	3111	1899
7725	218	2258	2260
7726	459	3120	2258
7727	889	2260	3120
7728	2258	3120	2260
7729	455	3111	3109
7730	889	3120	3111
7731	459	3109	3120
7732	3111	3120	3109
7733	162	1926	1924
7734	900	4025	1926
7735	887	1924	4025
7736	1926	4025	1924
7737	218	2260	2261
7738	889	4027	2260
7739	900	2261	4027
7740	2260	4027	2261
7741	158	1901	1903
7742	887	4024	1901
7743	889	1903	4024
7744	1901	4024	1903
7745	900	4027	4025
7746	889	4024	4027
7747	887	4025	4024
7748	4027	4024	4025
7749	21	1172	1176
7750	372	2889	1172
7751	376	1176	2889
7752	1172	2889	1176
7753	164	1937	1934
7754	905	2891	1937
7755	372	1934	2891
7756	1937	2891	1934
7757	168	1958	1960
7758	376	2902	1958
7759	905	1960	2902
7760	1958	2902	1960
7761	372	2891	2889
7762	905	2902	2891
7763	376	2889	2902

7764	2891	2902	2889
7765	39	1265	1261
7766	465	3127	1265
7767	461	1261	3127
7768	1265	3127	1261
7769	220	2272	2270
7770	907	3138	2272
7771	465	2270	3138
7772	2272	3138	2270
7773	164	1935	1939
7774	461	3129	1935
7775	907	1939	3129
7776	1935	3129	1939
7777	465	3138	3127
7778	907	3129	3138
7779	461	3127	3129
7780	3138	3129	3127
7781	76	1471	1473
7782	671	3738	1471
7783	673	1473	3738
7784	1471	3738	1473
7785	168	1962	1959
7786	918	3740	1962
7787	671	1959	3740
7788	1962	3740	1959
7789	220	2271	2273
7790	673	3745	2271
7791	918	2273	3745
7792	2271	3745	2273
7793	671	3740	3738
7794	918	3745	3740
7795	673	3738	3745
7796	3740	3745	3738
7797	164	1939	1937
7798	907	4042	1939
7799	905	1937	4042
7800	1939	4042	1937
7801	220	2273	2272
7802	918	4045	2273
7803	907	2272	4045
7804	2273	4045	2272
7805	168	1960	1962
7806	905	4043	1960
7807	918	1962	4043
7808	1960	4043	1962
7809	907	4045	4042
7810	918	4043	4045
7811	905	4042	4043
7812	4045	4043	4042
7813	38	1259	1257
7814	459	3115	1259
7815	457	1257	3115
7816	1259	3115	1257
7817	218	2262	2258
7818	1018	3121	2262
7819	459	2258	3121
7820	2262	3121	2258
7821	216	2246	2250
7822	457	3117	2246

7823	1018	2250	3117
7824	2246	3117	2250
7825	459	3121	3115
7826	1018	3117	3121
7827	457	3115	3117
7828	3121	3117	3115
7829	76	1473	1472
7830	673	3742	1473
7831	672	1472	3742
7832	1473	3742	1472
7833	220	2275	2271
7834	1020	3746	2275
7835	673	2271	3746
7836	2275	3746	2271
7837	218	2259	2263
7838	672	3744	2259
7839	1020	2263	3744
7840	2259	3744	2263
7841	673	3746	3742
7842	1020	3744	3746
7843	672	3742	3744
7844	3746	3744	3742
7845	39	1263	1265
7846	463	3133	1263
7847	465	1265	3133
7848	1263	3133	1265
7849	216	2251	2247
7850	1019	3135	2251
7851	463	2247	3135
7852	2251	3135	2247
7853	220	2270	2274
7854	465	3139	2270
7855	1019	2274	3139
7856	2270	3139	2274
7857	463	3135	3133
7858	1019	3139	3135
7859	465	3133	3139
7860	3135	3139	3133
7861	218	2263	2262
7862	1020	4155	2263
7863	1018	2262	4155
7864	2263	4155	2262
7865	220	2274	2275
7866	1019	4156	2274
7867	1020	2275	4156
7868	2274	4156	2275
7869	216	2250	2251
7870	1018	4154	2250
7871	1019	2251	4154
7872	2250	4154	2251
7873	1020	4156	4155
7874	1019	4154	4156
7875	1018	4155	4154
7876	4156	4154	4155
7877	24	1195	1194
7878	395	2955	1195
7879	394	1194	2955
7880	1195	2955	1194
7881	187	2075	2072

7882	971	2959	2075
7883	395	2072	2959
7884	2075	2959	2072
7885	186	2066	2069
7886	394	2957	2066
7887	971	2069	2957
7888	2066	2957	2069
7889	395	2959	2955
7890	971	2957	2959
7891	394	2955	2957
7892	2959	2957	2955
7893	78	1487	1483
7894	687	3775	1487
7895	683	1483	3775
7896	1487	3775	1483
7897	286	2669	2667
7898	975	3786	2669
7899	687	2667	3786
7900	2669	3786	2667
7901	187	2073	2077
7902	683	3777	2073
7903	975	2077	3777
7904	2073	3777	2077
7905	687	3786	3775
7906	975	3777	3786
7907	683	3775	3777
7908	3786	3777	3775
7909	77	1477	1481
7910	677	3757	1477
7911	681	1481	3757
7912	1477	3757	1481
7913	186	2071	2067
7914	973	3759	2071
7915	677	2067	3759
7916	2071	3759	2067
7917	286	2666	2668
7918	681	3768	2666
7919	973	2668	3768
7920	2666	3768	2668
7921	677	3759	3757
7922	973	3768	3759
7923	681	3757	3768
7924	3759	3768	3757
7925	187	2077	2075
7926	975	4110	2077
7927	971	2075	4110
7928	2077	4110	2075
7929	286	2668	2669
7930	973	4111	2668
7931	975	2669	4111
7932	2668	4111	2669
7933	186	2069	2071
7934	971	4109	2069
7935	973	2071	4109
7936	2069	4109	2071
7937	975	4111	4110
7938	973	4109	4111
7939	971	4110	4109
7940	4111	4109	4110

7941	21	1176	1177
7942	376	2901	1176
7943	377	1177	2901
7944	1176	2901	1177
7945	168	1961	1958
7946	917	2903	1961
7947	376	1958	2903
7948	1961	2903	1958
7949	169	1964	1967
7950	377	2905	1964
7951	917	1967	2905
7952	1964	2905	1967
7953	376	2903	2901
7954	917	2905	2903
7955	377	2901	2905
7956	2903	2905	2901
7957	76	1475	1471
7958	675	3739	1475
7959	671	1471	3739
7960	1475	3739	1471
7961	285	2662	2660
7962	919	3750	2662
7963	675	2660	3750
7964	2662	3750	2660
7965	168	1959	1963
7966	671	3741	1959
7967	919	1963	3741
7968	1959	3741	1963
7969	675	3750	3739
7970	919	3741	3750
7971	671	3739	3741
7972	3750	3741	3739
7973	78	1482	1486
7974	682	3771	1482
7975	686	1486	3771
7976	1482	3771	1486
7977	169	1969	1965
7978	921	3773	1969
7979	682	1965	3773
7980	1969	3773	1965
7981	285	2661	2663
7982	686	3784	2661
7983	921	2663	3784
7984	2661	3784	2663
7985	682	3773	3771
7986	921	3784	3773
7987	686	3771	3784
7988	3773	3784	3771
7989	168	1963	1961
7990	919	4055	1963
7991	917	1961	4055
7992	1963	4055	1961
7993	285	2663	2662
7994	921	4057	2663
7995	919	2662	4057
7996	2663	4057	2662
7997	169	1967	1969
7998	917	4056	1967
7999	921	1969	4056

8000	1967	4056	1969
8001	919	4057	4055
8002	921	4056	4057
8003	917	4055	4056
8004	4057	4056	4055
8005	20	1171	1170
8006	371	2883	1171
8007	370	1170	2883
8008	1171	2883	1170
8009	163	1931	1928
8010	899	2887	1931
8011	371	1928	2887
8012	1931	2887	1928
8013	162	1922	1925
8014	370	2885	1922
8015	899	1925	2885
8016	1922	2885	1925
8017	371	2887	2883
8018	899	2885	2887
8019	370	2883	2885
8020	2887	2885	2883
8021	77	1480	1476
8022	680	3753	1480
8023	676	1476	3753
8024	1480	3753	1476
8025	284	2657	2655
8026	903	3766	2657
8027	680	2655	3766
8028	2657	3766	2655
8029	163	1929	1933
8030	676	3755	1929
8031	903	1933	3755
8032	1929	3755	1933
8033	680	3766	3753
8034	903	3755	3766
8035	676	3753	3755
8036	3766	3755	3753
8037	76	1470	1474
8038	670	3735	1470
8039	674	1474	3735
8040	1470	3735	1474
8041	162	1927	1923
8042	901	3737	1927
8043	670	1923	3737
8044	1927	3737	1923
8045	284	2654	2656
8046	674	3748	2654
8047	901	2656	3748
8048	2654	3748	2656
8049	670	3737	3735
8050	901	3748	3737
8051	674	3735	3748
8052	3737	3748	3735
8053	163	1933	1931
8054	903	4038	1933
8055	899	1931	4038
8056	1933	4038	1931
8057	284	2656	2657
8058	901	4039	2656

8059	903	2657	4039
8060	2656	4039	2657
8061	162	1925	1927
8062	899	4037	1925
8063	901	1927	4037
8064	1925	4037	1927
8065	903	4039	4038
8066	901	4037	4039
8067	899	4038	4037
8068	4039	4037	4038
8069	78	1486	1487
8070	686	3783	1486
8071	687	1487	3783
8072	1486	3783	1487
8073	285	2665	2661
8074	1086	3785	2665
8075	686	2661	3785
8076	2665	3785	2661
8077	286	2667	2671
8078	687	3787	2667
8079	1086	2671	3787
8080	2667	3787	2671
8081	686	3785	3783
8082	1086	3787	3785
8083	687	3783	3787
8084	3785	3787	3783
8085	76	1474	1475
8086	674	3747	1474
8087	675	1475	3747
8088	1474	3747	1475
8089	284	2658	2654
8090	1084	3749	2658
8091	674	2654	3749
8092	2658	3749	2654
8093	285	2660	2664
8094	675	3751	2660
8095	1084	2664	3751
8096	2660	3751	2664
8097	674	3749	3747
8098	1084	3751	3749
8099	675	3747	3751
8100	3749	3751	3747
8101	77	1481	1480
8102	681	3765	1481
8103	680	1480	3765
8104	1481	3765	1480
8105	286	2670	2666
8106	1085	3769	2670
8107	681	2666	3769
8108	2670	3769	2666
8109	284	2655	2659
8110	680	3767	2655
8111	1085	2659	3767
8112	2655	3767	2659
8113	681	3769	3765
8114	1085	3767	3769
8115	680	3765	3767
8116	3769	3767	3765
8117	285	2664	2665

8118	1084	4221	2664
8119	1086	2665	4221
8120	2664	4221	2665
8121	284	2659	2658
8122	1085	4220	2659
8123	1084	2658	4220
8124	2659	4220	2658
8125	286	2671	2670
8126	1086	4222	2671
8127	1085	2670	4222
8128	2671	4222	2670
8129	1084	4220	4221
8130	1085	4222	4220
8131	1086	4221	4222
8132	4220	4222	4221
8133	6	1106	4246
8134	6	4246	1104
8135	1106	4231	2717
8136	1106	306	4231
8137	1104	2717	4230
8138	1104	4230	304
8139	2717	4246	1106
8140	2717	1104	4246
8141	1578	4228	1580
8142	1578	98	4228
8143	2722	1580	4239
8144	2722	4239	733
8145	306	4251	2722
8146	306	1578	4251
8147	1580	2722	4251
8148	1580	4251	1578
8149	1570	1572	4227
8150	1570	4227	96
8151	304	2718	4249
8152	304	4249	1570
8153	2718	4238	1572
8154	2718	733	4238
8155	1572	4249	2718
8156	1572	1570	4249
8157	2722	2717	4231
8158	2722	4231	306
8159	733	4258	2722
8160	733	2718	4258
8161	2718	4230	2717
8162	2718	304	4230
8163	2717	2722	4258
8164	2717	4258	2718
8165	1582	1580	4228
8166	1582	4228	98
8167	738	3870	4252
8168	738	4252	1582
8169	3870	4239	1580
8170	3870	733	4239
8171	1580	4252	3870
8172	1580	1582	4252
8173	221	4257	2279
8174	221	2278	4257
8175	2278	4240	3871
8176	2278	734	4240

8177	2279	3871	4241
8178	2279	4241	738
8179	3871	2279	4257
8180	3871	4257	2278
8181	1573	4227	1572
8182	1573	96	4227
8183	3869	1572	4238
8184	3869	4238	733
8185	734	4250	3869
8186	734	1573	4250
8187	1572	3869	4250
8188	1572	4250	1573
8189	3870	4241	3871
8190	3870	738	4241
8191	3869	3871	4240
8192	3869	4240	734
8193	733	3870	4262
8194	733	4262	3869
8195	3871	4262	3870
8196	3871	3869	4262
8197	1267	4226	4247
8198	4226	40	4247
8199	4247	40	1269
8200	3142	4248	4237
8201	4237	4248	1269
8202	4237	1269	469
8203	467	4233	4235
8204	4233	1267	4235
8205	4235	1267	3142
8206	1269	4248	4247
8207	4247	4248	3142
8208	4247	3142	1267
8209	1691	4253	4229
8210	4229	4253	119
8211	4253	1693	119
8212	467	4236	4234
8213	4234	4236	1691
8214	4236	3143	1691
8215	3143	4242	4254
8216	4242	1693	4254
8217	4242	790	1693
8218	1693	4253	4254
8219	4253	3143	4254
8220	4253	1691	3143
8221	3142	4237	4260
8222	4237	3147	4260
8223	4237	469	3147
8224	3143	4261	4242
8225	4242	4261	790
8226	4261	3147	790
8227	467	4235	4236
8228	4235	3143	4236
8229	4235	3142	3143
8230	3147	4261	4260
8231	4260	4261	3142
8232	4261	3143	3142
8233	215	2243	4256
8234	215	4256	2242
8235	2243	4245	3955

8236	2243	821	4245
8237	2242	3955	4243
8238	2242	4243	817
8239	3955	4256	2243
8240	3955	2242	4256
8241	3953	4243	3955
8242	3953	817	4243
8243	3954	3955	4245
8244	3954	4245	821
8245	816	4263	3954
8246	816	3953	4263
8247	3955	3954	4263
8248	3955	4263	3953
8249	131	1759	4255
8250	131	4255	1756
8251	1759	4244	2806
8252	1759	818	4244
8253	1756	2806	4232
8254	1756	4232	339
8255	2806	4255	1759
8256	2806	1756	4255
8257	2802	4232	2806
8258	2802	339	4232
8259	2804	2806	4244
8260	2804	4244	818
8261	338	4259	2804
8262	338	2802	4259
8263	2806	2804	4259
8264	2806	4259	2802

End of Connectivity Matrix of Nodes

DESIGN 3

	Node Coordinates		
Node Index	X(m)	Y(m)	Z(m)
1	0.00000000	0.00000000	0.19765396
2	0.00000000	4.00000000	0.10107527
3	4.00000000	0.00000000	0.12844575
4	4.00000000	4.00000000	0.12179863
5	0.00000000	2.00000000	-0.20000000
6	2.00000000	0.00000000	0.19882698
7	2.00000000	2.00000000	0.05728250
8	2.00000000	4.00000000	-0.00254154
9	4.00000000	2.00000000	-0.19804497
10	0.00000000	1.00000000	-0.19687195
11	1.00000000	0.00000000	0.11515152
12	1.00000000	1.00000000	-0.19569892
13	0.00000000	3.00000000	0.17341153
14	1.00000000	4.00000000	0.08152493
15	3.00000000	0.00000000	0.09286413
16	4.00000000	1.00000000	-0.19804497
17	3.00000000	3.00000000	0.07956989
18	3.00000000	4.00000000	-0.11827957
19	4.00000000	3.00000000	0.05024438
20	1.00000000	2.00000000	-0.19569892
21	1.00000000	3.00000000	0.14916911
22	2.00000000	1.00000000	-0.00801564
23	3.00000000	1.00000000	-0.19882698
24	2.00000000	3.00000000	0.19804497
25	3.00000000	2.00000000	-0.19413490
26	0.00000000	0.50000000	-0.03773216
27	0.50000000	0.00000000	0.18084066
28	0.50000000	0.50000000	-0.03929619
29	0.00000000	3.50000000	0.13743891
30	0.50000000	4.00000000	0.04125122
31	3.50000000	0.00000000	0.18435973
32	4.00000000	0.50000000	0.16480938
33	3.50000000	3.50000000	0.17966764
34	3.50000000	4.00000000	-0.07018573
35	4.00000000	3.50000000	0.15816227
36	0.00000000	1.50000000	-0.19452590
37	0.00000000	2.50000000	-0.11436950
38	0.50000000	2.00000000	-0.14330401
39	0.50000000	2.50000000	-0.12531769
40	1.50000000	0.00000000	0.16950147
41	2.50000000	0.00000000	0.18826979
42	2.00000000	0.50000000	0.19569892
43	2.50000000	0.50000000	0.00645161
44	1.50000000	1.50000000	-0.06979472
45	2.50000000	2.50000000	0.09051808
46	1.50000000	2.00000000	-0.06901271
47	2.00000000	1.50000000	0.02482893
48	2.00000000	2.50000000	0.13391984
49	2.50000000	2.00000000	-0.07487781
50	1.50000000	4.00000000	-0.00957967
51	2.50000000	4.00000000	0.13939394
52	1.50000000	3.50000000	-0.00527859
53	2.00000000	3.50000000	-0.10224829
54	4.00000000	1.50000000	-0.17380254
55	4.00000000	2.50000000	-0.09912023
56	3.50000000	1.50000000	-0.19178886

57	3.50000000	2.00000000	-0.13118280
58	0.50000000	1.00000000	-0.19374389
59	0.50000000	1.50000000	-0.19608993
60	1.00000000	0.50000000	-0.12805474
61	1.50000000	0.50000000	0.06314761
62	1.00000000	1.50000000	-0.19608993
63	1.50000000	1.00000000	-0.10537634
64	0.50000000	3.50000000	0.19022483
65	0.50000000	3.00000000	0.18631476
66	1.00000000	3.50000000	0.16559140
67	3.50000000	0.50000000	-0.00488759
68	3.00000000	0.50000000	0.00332356
69	3.50000000	1.00000000	-0.14877810
70	3.00000000	3.50000000	0.19413490
71	3.50000000	3.00000000	0.13978495
72	2.50000000	3.00000000	0.16402737
73	3.00000000	2.50000000	-0.13235582
74	2.50000000	3.50000000	0.03890518
75	3.50000000	2.50000000	0.17849462
76	1.00000000	2.50000000	-0.06080156
77	1.50000000	2.50000000	0.00645161
78	1.50000000	3.00000000	-0.14213099
79	2.50000000	1.00000000	-0.09716520
80	2.50000000	1.50000000	-0.12609971
81	3.00000000	1.50000000	-0.19022483
82	0.00000000	0.25000000	0.01348974
83	0.25000000	0.00000000	0.12297165
84	0.25000000	0.25000000	0.03773216
85	0.00000000	3.75000000	0.11945259
86	0.25000000	4.00000000	0.07135875
87	3.75000000	0.00000000	0.15659824
88	4.00000000	0.25000000	0.04672532
89	3.75000000	3.75000000	0.15073314
90	3.75000000	4.00000000	0.02561095
91	4.00000000	3.75000000	0.13978495
92	0.00000000	1.75000000	-0.19765396
93	0.00000000	2.25000000	0.04320626
94	0.25000000	2.00000000	-0.17106549
95	0.25000000	2.25000000	-0.16246334
96	1.75000000	0.00000000	0.18396872
97	2.25000000	0.00000000	0.19687195
98	2.00000000	0.25000000	0.19726295
99	2.25000000	0.25000000	0.12766373
100	1.75000000	1.75000000	-0.00645161
101	2.25000000	2.25000000	0.07409580
102	1.75000000	2.00000000	-0.00606061
103	2.00000000	1.75000000	0.04086022
104	2.00000000	2.25000000	0.09560117
105	2.25000000	2.00000000	-0.00879765
106	1.75000000	4.00000000	0.09403715
107	2.25000000	4.00000000	0.01857283
108	1.75000000	3.75000000	0.09599218
109	2.00000000	3.75000000	-0.07800587
110	4.00000000	1.75000000	-0.18592375
111	4.00000000	2.25000000	-0.12336266
112	3.75000000	1.75000000	-0.19491691
113	3.75000000	2.00000000	-0.16285435
114	0.00000000	0.75000000	-0.11749756
115	0.00000000	1.25000000	-0.19608993

116	0.25000000	1.00000000	-0.19374389
117	0.25000000	1.25000000	-0.19648094
118	0.75000000	0.00000000	0.11827957
119	1.25000000	0.00000000	0.04242424
120	1.00000000	0.25000000	-0.00645161
121	1.25000000	0.25000000	0.09560117
122	0.75000000	0.75000000	-0.11749756
123	1.25000000	1.25000000	-0.13274682
124	0.75000000	1.00000000	-0.19648094
125	1.00000000	0.75000000	-0.16168133
126	1.00000000	1.25000000	-0.20000000
127	1.25000000	1.00000000	-0.15034213
128	0.00000000	3.25000000	0.15542522
129	0.00000000	2.75000000	0.02952102
130	0.25000000	3.25000000	0.08191593
131	0.25000000	3.00000000	0.18084066
132	0.75000000	4.00000000	0.06158358
133	1.25000000	4.00000000	0.12961877
134	0.75000000	3.75000000	0.13509286
135	1.00000000	3.75000000	0.14877810
136	3.25000000	0.00000000	0.13861193
137	2.75000000	0.00000000	0.14056696
138	3.25000000	0.25000000	0.09481916
139	3.00000000	0.25000000	0.04828935
140	4.00000000	0.75000000	-0.18787879
141	4.00000000	1.25000000	-0.19882698
142	3.75000000	0.75000000	-0.10694037
143	3.75000000	1.00000000	-0.20000000
144	3.25000000	3.25000000	0.14369501
145	2.75000000	2.75000000	0.03577713
146	3.00000000	3.25000000	0.13704790
147	3.25000000	3.00000000	0.10967742
148	2.75000000	3.00000000	0.14643206
149	3.00000000	2.75000000	0.01114370
150	3.25000000	4.00000000	-0.04555230
151	2.75000000	4.00000000	0.01075269
152	3.00000000	3.75000000	0.03812317
153	2.75000000	3.75000000	-0.03890518
154	4.00000000	3.25000000	0.10420332
155	4.00000000	2.75000000	0.00371457
156	3.75000000	3.00000000	0.02013685
157	3.75000000	2.75000000	-0.18592375
158	0.75000000	2.00000000	0.02521994
159	1.25000000	2.00000000	-0.13235582
160	0.75000000	1.75000000	-0.19608993
161	1.00000000	1.75000000	-0.19608993
162	1.00000000	2.25000000	-0.10928641
163	1.25000000	2.25000000	-0.09521017
164	0.75000000	2.75000000	0.01192571
165	1.25000000	3.25000000	0.17497556
166	0.75000000	3.00000000	0.16793744
167	1.00000000	3.25000000	0.15738025
168	1.00000000	2.75000000	-0.13626588
169	1.25000000	3.00000000	0.16129032
170	2.00000000	0.75000000	0.08152493
171	2.00000000	1.25000000	0.00840665
172	1.75000000	0.75000000	0.02756598
173	1.75000000	1.00000000	0.14056696
174	2.25000000	1.00000000	-0.05259042

175	2.25000000	1.25000000	0.14565005
176	2.75000000	0.75000000	-0.09599218
177	3.25000000	1.25000000	-0.19530792
178	3.00000000	0.75000000	-0.09755621
179	3.25000000	1.00000000	-0.17106549
180	2.75000000	1.00000000	0.05063539
181	3.00000000	1.25000000	-0.19452590
182	2.00000000	2.75000000	0.16598240
183	2.00000000	3.25000000	0.02287390
184	2.25000000	3.00000000	0.19335288
185	2.25000000	3.25000000	0.06901271
186	1.75000000	2.75000000	0.15229717
187	1.75000000	3.00000000	0.18592375
188	2.75000000	2.00000000	-0.18435973
189	3.25000000	2.00000000	-0.16246334
190	3.00000000	2.25000000	-0.16950147
191	3.25000000	2.25000000	-0.12023460
192	2.75000000	1.75000000	-0.14760508
193	3.00000000	1.75000000	-0.19374389
194	0.25000000	0.50000000	-0.13861193
195	0.25000000	0.75000000	-0.10068426
196	0.50000000	0.25000000	0.07096774
197	0.75000000	0.25000000	0.03264907
198	0.50000000	0.75000000	-0.10185728
199	0.75000000	0.50000000	-0.07722385
200	0.25000000	3.75000000	0.09247312
201	0.25000000	3.50000000	0.16324536
202	0.50000000	3.75000000	-0.08426197
203	3.75000000	0.25000000	-0.17536657
204	3.50000000	0.25000000	0.06549365
205	3.75000000	0.50000000	-0.10850440
206	3.50000000	3.75000000	0.05337243
207	3.75000000	3.50000000	0.16637341
208	3.25000000	3.50000000	0.19960899
209	3.50000000	3.25000000	0.15659824
210	3.25000000	3.75000000	0.05259042
211	3.75000000	3.25000000	0.14916911
212	0.25000000	1.75000000	-0.19335288
213	0.25000000	1.50000000	-0.16950147
214	0.25000000	2.50000000	-0.11280547
215	0.25000000	2.75000000	-0.16402737
216	0.50000000	2.25000000	-0.13352884
217	0.50000000	1.75000000	-0.16989247
218	0.75000000	2.25000000	0.14134897
219	0.50000000	2.75000000	0.03069404
220	0.75000000	2.50000000	-0.07409580
221	1.75000000	0.25000000	0.18279570
222	1.50000000	0.25000000	0.11593353
223	2.50000000	0.25000000	0.10029326
224	2.75000000	0.25000000	0.09912023
225	2.25000000	0.50000000	0.10420332
226	1.75000000	0.50000000	0.11710655
227	2.25000000	0.75000000	0.09990225
228	2.75000000	0.50000000	-0.19530792
229	2.50000000	0.75000000	-0.04516129
230	1.50000000	1.75000000	-0.06940371
231	1.75000000	1.50000000	-0.02248289
232	1.25000000	1.50000000	-0.13431085
233	1.50000000	1.25000000	-0.08739003

234	2.25000000	2.50000000	0.11241447
235	2.50000000	2.25000000	0.00879765
236	2.50000000	2.75000000	0.12727273
237	2.75000000	2.50000000	0.04164223
238	1.75000000	2.25000000	0.03264907
239	1.25000000	1.75000000	-0.13235582
240	1.50000000	2.25000000	-0.13157380
241	2.25000000	1.75000000	-0.02482893
242	1.75000000	1.25000000	-0.04007820
243	2.25000000	1.50000000	-0.12570870
244	2.25000000	2.75000000	0.19687195
245	1.75000000	2.50000000	0.17028348
246	2.75000000	2.25000000	-0.05376344
247	2.50000000	1.75000000	-0.18944282
248	1.50000000	3.75000000	-0.03186706
249	1.25000000	3.75000000	0.17771261
250	2.25000000	3.75000000	-0.00606061
251	2.50000000	3.75000000	0.09012708
252	1.75000000	3.50000000	-0.17927664
253	1.25000000	3.50000000	0.13000978
254	1.50000000	3.25000000	0.19648094
255	2.25000000	3.50000000	-0.09325513
256	1.75000000	3.25000000	-0.18318671
257	3.75000000	1.50000000	-0.18201369
258	3.75000000	1.25000000	-0.16129032
259	3.75000000	2.25000000	-0.09951124
260	3.75000000	2.50000000	-0.08543500
261	3.50000000	1.75000000	-0.16129032
262	3.50000000	1.25000000	-0.17028348
263	3.25000000	1.50000000	-0.18787879
264	3.50000000	2.25000000	-0.07644184
265	3.25000000	1.75000000	-0.15738025
266	0.50000000	1.25000000	-0.19335288
267	0.75000000	1.25000000	-0.19335288
268	0.75000000	1.50000000	-0.19569892
269	1.25000000	0.50000000	-0.03264907
270	1.25000000	0.75000000	-0.11671554
271	1.50000000	0.75000000	-0.02091887
272	0.50000000	3.25000000	0.18826979
273	0.75000000	3.50000000	0.17810362
274	0.75000000	3.25000000	0.17653959
275	3.25000000	0.50000000	0.00019550
276	3.50000000	0.75000000	-0.07605083
277	3.25000000	0.75000000	-0.17302053
278	2.75000000	3.25000000	0.17888563
279	2.75000000	3.50000000	0.11710655
280	3.25000000	2.75000000	0.05376344
281	3.50000000	2.75000000	0.05884653
282	2.50000000	3.25000000	0.10224829
283	3.25000000	2.50000000	-0.02678397
284	1.25000000	2.50000000	-0.00840665
285	1.25000000	2.75000000	0.07526882
286	1.50000000	2.75000000	0.09012708
287	2.50000000	1.25000000	-0.09912023
288	2.75000000	1.25000000	-0.14369501
289	2.75000000	1.50000000	-0.14565005
290	0.00000000	0.12500000	0.05376344
291	0.12500000	0.00000000	0.10224829
292	0.12500000	0.12500000	0.05962854

293	0.00000000	3.87500000	0.11045943
294	0.12500000	4.00000000	0.08621701
295	3.87500000	0.00000000	0.04242424
296	4.00000000	0.12500000	0.08778104
297	3.87500000	3.87500000	0.13626588
298	3.87500000	4.00000000	0.07370479
299	4.00000000	3.87500000	0.13079179
300	0.00000000	1.87500000	0.00136852
301	0.00000000	2.12500000	-0.07839687
302	0.12500000	2.00000000	-0.18553275
303	0.12500000	2.12500000	-0.18123167
304	1.87500000	0.00000000	0.19139785
305	2.12500000	0.00000000	0.19804497
306	2.00000000	0.12500000	0.19804497
307	2.12500000	0.12500000	0.16324536
308	1.87500000	1.87500000	0.02521994
309	2.12500000	2.12500000	0.06588465
310	1.87500000	2.00000000	0.02561095
311	2.00000000	1.87500000	0.04907136
312	2.00000000	2.12500000	0.07644184
313	2.12500000	2.00000000	0.02443793
314	1.87500000	4.00000000	0.04594330
315	2.12500000	4.00000000	0.00801564
316	1.87500000	3.87500000	0.04672532
317	2.00000000	3.87500000	-0.04320626
318	4.00000000	1.87500000	-0.19217986
319	4.00000000	2.12500000	-0.16050831
320	3.87500000	1.87500000	-0.19491691
321	3.87500000	2.00000000	-0.18123167
322	0.00000000	0.87500000	-0.15698925
323	0.00000000	1.12500000	-0.19648094
324	0.12500000	1.00000000	-0.19530792
325	0.12500000	1.12500000	-0.19687195
326	0.87500000	0.00000000	0.12297165
327	1.12500000	0.00000000	0.07878788
328	1.00000000	0.12500000	0.00449658
329	1.12500000	0.12500000	0.10537634
330	0.87500000	0.87500000	-0.15659824
331	1.12500000	1.12500000	-0.16441838
332	0.87500000	1.00000000	-0.19530792
333	1.00000000	0.87500000	-0.17888563
334	1.00000000	1.12500000	-0.19960899
335	1.12500000	1.00000000	-0.17302053
336	0.00000000	3.12500000	0.06432063
337	0.00000000	2.87500000	0.10146628
338	0.12500000	3.12500000	0.12766373
339	0.12500000	3.00000000	0.17771261
340	0.87500000	4.00000000	0.07174976
341	1.12500000	4.00000000	0.10185728
342	0.87500000	3.87500000	0.10850440
343	1.00000000	3.87500000	0.11515152
344	3.12500000	0.00000000	0.11593353
345	2.87500000	0.00000000	0.11593353
346	3.12500000	0.12500000	0.03147605
347	3.00000000	0.12500000	0.07057674
348	4.00000000	0.87500000	-0.19296188
349	4.00000000	1.12500000	-0.19843597
350	3.87500000	0.87500000	-0.15229717
351	3.87500000	1.00000000	-0.19921799

352	3.12500000	3.12500000	0.10537634
353	2.87500000	2.87500000	0.05767351
354	3.00000000	3.12500000	0.10850440
355	3.12500000	3.00000000	0.09481916
356	2.87500000	3.00000000	0.11319648
357	3.00000000	2.87500000	0.04555230
358	3.12500000	4.00000000	-0.08191593
359	2.87500000	4.00000000	-0.15386119
360	3.00000000	3.87500000	-0.04007820
361	2.87500000	3.87500000	-0.07839687
362	4.00000000	3.12500000	0.07722385
363	4.00000000	2.87500000	0.02678397
364	3.87500000	3.00000000	0.03499511
365	3.87500000	2.87500000	0.13235582
366	0.87500000	2.00000000	-0.08504399
367	1.12500000	2.00000000	-0.16402737
368	0.87500000	1.87500000	-0.19608993
369	1.00000000	1.87500000	-0.19608993
370	1.00000000	2.12500000	-0.15229717
371	1.12500000	2.12500000	-0.14525904
372	0.87500000	2.87500000	0.08035191
373	1.12500000	3.12500000	0.16207234
374	0.87500000	3.00000000	0.15855327
375	1.00000000	3.12500000	0.15972630
376	1.00000000	2.87500000	0.00645161
377	1.12500000	3.00000000	0.15620723
378	2.00000000	0.87500000	0.04320626
379	2.00000000	1.12500000	0.00019550
380	1.87500000	0.87500000	0.00997067
381	1.87500000	1.00000000	0.06627566
382	2.12500000	1.00000000	-0.03030303
383	2.12500000	1.12500000	0.06901271
384	2.87500000	0.87500000	-0.14447703
385	3.12500000	1.12500000	-0.09716520
386	3.00000000	0.87500000	-0.14799609
387	3.12500000	1.00000000	-0.18670577
388	2.87500000	1.00000000	-0.07409580
389	3.00000000	1.12500000	-0.19687195
390	2.00000000	2.87500000	0.17575758
391	2.00000000	3.12500000	0.11045943
392	2.12500000	3.00000000	0.18944282
393	2.12500000	3.12500000	0.13352884
394	1.87500000	2.87500000	0.16246334
395	1.87500000	3.00000000	0.19217986
396	2.87500000	2.00000000	-0.18944282
397	3.12500000	2.00000000	-0.17849462
398	3.00000000	2.12500000	-0.17888563
399	3.12500000	2.12500000	-0.15698925
400	2.87500000	1.87500000	-0.17106549
401	3.00000000	1.87500000	-0.19491691
402	0.00000000	0.37500000	-0.00840665
403	0.00000000	0.62500000	-0.07761486
404	0.12500000	0.50000000	-0.08817204
405	0.12500000	0.62500000	-0.06901271
406	0.37500000	0.00000000	0.15190616
407	0.62500000	0.00000000	0.14682307
408	0.50000000	0.12500000	0.12609971
409	0.62500000	0.12500000	0.10694037
410	0.37500000	0.37500000	-0.00058651

411	0.62500000	0.62500000	-0.07839687
412	0.37500000	0.50000000	-0.08895406
413	0.50000000	0.37500000	0.01583578
414	0.50000000	0.62500000	-0.07761486
415	0.62500000	0.50000000	-0.08308895
416	0.00000000	3.62500000	0.12844575
417	0.00000000	3.37500000	0.14643206
418	0.12500000	3.62500000	0.11515152
419	0.12500000	3.50000000	0.15034213
420	0.37500000	4.00000000	0.05728250
421	0.62500000	4.00000000	0.05141740
422	0.37500000	3.87500000	0.07331378
423	0.50000000	3.87500000	-0.11984360
424	3.62500000	0.00000000	0.17145650
425	3.37500000	0.00000000	0.16168133
426	3.62500000	0.12500000	0.10615836
427	3.50000000	0.12500000	0.12492669
428	4.00000000	0.37500000	0.10576735
429	4.00000000	0.62500000	-0.01153470
430	3.87500000	0.37500000	-0.05532747
431	3.87500000	0.50000000	0.02756598
432	3.62500000	3.62500000	0.16520039
433	3.37500000	3.37500000	0.16168133
434	3.50000000	3.62500000	0.11671554
435	3.62500000	3.50000000	0.17302053
436	3.37500000	3.50000000	0.18357771
437	3.50000000	3.37500000	0.16832845
438	3.62500000	4.00000000	-0.12258065
439	3.37500000	4.00000000	-0.05767351
440	3.50000000	3.87500000	-0.03343109
441	3.37500000	3.87500000	-0.00723363
442	4.00000000	3.62500000	0.14916911
443	4.00000000	3.37500000	0.13118280
444	3.87500000	3.50000000	0.16246334
445	3.87500000	3.37500000	0.15347019
446	0.00000000	1.62500000	-0.09599218
447	0.00000000	1.37500000	-0.19530792
448	0.12500000	1.62500000	-0.19413490
449	0.12500000	1.50000000	-0.18201369
450	0.00000000	2.37500000	-0.03538612
451	0.00000000	2.62500000	-0.04242424
452	0.12500000	2.50000000	-0.11397849
453	0.12500000	2.62500000	-0.13939394
454	0.37500000	2.00000000	-0.15698925
455	0.62500000	2.00000000	-0.05884653
456	0.37500000	1.87500000	-0.16832845
457	0.50000000	2.12500000	-0.13822092
458	0.50000000	1.87500000	-0.15659824
459	0.62500000	2.12500000	-0.00019550
460	0.37500000	2.37500000	-0.14369501
461	0.62500000	2.62500000	-0.05650049
462	0.37500000	2.50000000	-0.12258065
463	0.50000000	2.37500000	-0.12922776
464	0.50000000	2.62500000	-0.04711632
465	0.62500000	2.50000000	-0.09951124
466	1.62500000	0.00000000	0.17693060
467	1.37500000	0.00000000	0.10615836
468	1.62500000	0.12500000	0.17614858
469	1.50000000	0.12500000	0.14252199

470	2.37500000	0.00000000	0.19257087
471	2.62500000	0.00000000	0.16441838
472	2.50000000	0.12500000	0.14447703
473	2.62500000	0.12500000	0.14369501
474	2.00000000	0.37500000	0.19648094
475	2.00000000	0.62500000	0.13861193
476	1.87500000	0.37500000	0.18944282
477	2.12500000	0.50000000	0.14995112
478	1.87500000	0.50000000	0.15659824
479	2.12500000	0.62500000	0.14799609
480	2.37500000	0.37500000	0.06705767
481	2.62500000	0.62500000	-0.04516129
482	2.50000000	0.37500000	0.05337243
483	2.37500000	0.50000000	0.05532747
484	2.62500000	0.50000000	-0.09442815
485	2.50000000	0.62500000	-0.01935484
486	1.62500000	1.62500000	-0.03812317
487	1.37500000	1.37500000	-0.12609971
488	1.50000000	1.62500000	-0.06940371
489	1.62500000	1.50000000	-0.04594330
490	1.37500000	1.50000000	-0.10224829
491	1.50000000	1.37500000	-0.07839687
492	2.37500000	2.37500000	0.08230694
493	2.62500000	2.62500000	0.06314761
494	2.37500000	2.50000000	0.10146628
495	2.50000000	2.37500000	0.03734115
496	2.50000000	2.62500000	0.10889541
497	2.62500000	2.50000000	0.06627566
498	1.62500000	2.00000000	-0.03734115
499	1.37500000	2.00000000	-0.10068426
500	1.50000000	1.87500000	-0.06901271
501	1.62500000	2.12500000	-0.01818182
502	1.37500000	1.87500000	-0.10068426
503	1.50000000	2.12500000	-0.10029326
504	2.00000000	1.62500000	0.03264907
505	2.00000000	1.37500000	0.01661779
506	1.87500000	1.50000000	0.00136852
507	2.12500000	1.62500000	0.02521994
508	1.87500000	1.37500000	-0.01075269
509	2.12500000	1.50000000	0.14369501
510	2.00000000	2.37500000	0.13978495
511	2.00000000	2.62500000	0.14682307
512	2.12500000	2.50000000	0.12336266
513	1.87500000	2.37500000	0.08347996
514	2.12500000	2.62500000	0.16871945
515	1.87500000	2.50000000	0.18357771
516	2.37500000	2.00000000	-0.04164223
517	2.62500000	2.00000000	-0.13118280
518	2.50000000	2.12500000	-0.03304008
519	2.37500000	1.87500000	0.15034213
520	2.62500000	2.12500000	-0.07174976
521	2.50000000	1.87500000	-0.03147605
522	1.62500000	4.00000000	0.09247312
523	1.37500000	4.00000000	0.07253177
524	1.50000000	3.87500000	0.07878788
525	1.37500000	3.87500000	0.08426197
526	2.37500000	4.00000000	0.07565982
527	2.62500000	4.00000000	0.07409580
528	2.37500000	3.87500000	0.09169110

529	2.50000000	3.87500000	0.14291300
530	1.62500000	3.62500000	0.00879765
531	1.37500000	3.37500000	0.08152493
532	1.50000000	3.62500000	0.08113392
533	1.62500000	3.50000000	0.00801564
534	1.37500000	3.50000000	0.06236559
535	1.50000000	3.37500000	0.03968719
536	2.00000000	3.62500000	-0.08856305
537	2.00000000	3.37500000	-0.03968719
538	2.12500000	3.62500000	-0.06041056
539	1.87500000	3.50000000	-0.04046921
540	2.12500000	3.50000000	-0.09794721
541	1.87500000	3.37500000	-0.06744868
542	4.00000000	1.62500000	-0.17966764
543	4.00000000	1.37500000	0.06705767
544	3.87500000	1.50000000	-0.15425220
545	3.87500000	1.37500000	-0.16441838
546	4.00000000	2.37500000	-0.01114370
547	4.00000000	2.62500000	-0.04750733
548	3.87500000	2.37500000	-0.07370479
549	3.87500000	2.50000000	-0.09208211
550	3.62500000	1.62500000	-0.14330401
551	3.37500000	1.37500000	-0.12453568
552	3.62500000	1.50000000	-0.18748778
553	3.50000000	1.62500000	0.07448680
554	3.50000000	1.37500000	-0.05571848
555	3.37500000	1.50000000	-0.19139785
556	3.62500000	2.00000000	-0.18279570
557	3.37500000	2.00000000	-0.04672532
558	3.62500000	2.12500000	-0.01466276
559	3.50000000	1.87500000	-0.04594330
560	3.50000000	2.12500000	0.09638319
561	3.37500000	1.87500000	-0.14408602
562	0.37500000	1.00000000	-0.19687195
563	0.62500000	1.00000000	-0.19491691
564	0.37500000	0.87500000	-0.14760508
565	0.50000000	0.87500000	-0.05376344
566	0.50000000	1.12500000	0.08504399
567	0.62500000	1.12500000	-0.19178886
568	0.37500000	1.37500000	-0.18944282
569	0.62500000	1.62500000	0.01661779
570	0.37500000	1.50000000	-0.19530792
571	0.50000000	1.62500000	-0.18318671
572	0.50000000	1.37500000	-0.19452590
573	0.62500000	1.50000000	-0.19608993
574	1.00000000	0.37500000	-0.06705767
575	1.00000000	0.62500000	-0.14486804
576	0.87500000	0.37500000	-0.04437928
577	0.87500000	0.50000000	-0.10263930
578	1.12500000	0.50000000	-0.07996090
579	1.12500000	0.62500000	-0.12375367
580	1.37500000	0.37500000	0.09325513
581	1.62500000	0.62500000	0.04555230
582	1.50000000	0.37500000	0.08973607
583	1.62500000	0.50000000	-0.11006843
584	1.37500000	0.50000000	0.01544477
585	1.50000000	0.62500000	-0.17888563
586	1.00000000	1.37500000	-0.19491691
587	1.00000000	1.62500000	-0.19608993

588	1.12500000	1.50000000	-0.16520039
589	1.12500000	1.62500000	-0.16285435
590	0.87500000	1.37500000	-0.19452590
591	0.87500000	1.50000000	-0.18983382
592	1.37500000	1.00000000	-0.10263930
593	1.62500000	1.00000000	0.11788856
594	1.50000000	1.12500000	-0.09638319
595	1.62500000	1.12500000	-0.07292278
596	1.37500000	0.87500000	-0.11085044
597	1.50000000	0.87500000	-0.06314761
598	0.37500000	3.37500000	0.13587488
599	0.62500000	3.62500000	0.16285435
600	0.37500000	3.50000000	0.17693060
601	0.50000000	3.62500000	-0.14721408
602	0.50000000	3.37500000	0.18944282
603	0.62500000	3.50000000	0.18435973
604	0.37500000	3.00000000	0.13196481
605	0.62500000	3.00000000	0.17693060
606	0.37500000	2.87500000	0.01114370
607	0.50000000	2.87500000	0.10224829
608	0.50000000	3.12500000	0.18592375
609	0.62500000	3.12500000	0.18084066
610	1.00000000	3.62500000	0.15738025
611	1.00000000	3.37500000	0.16168133
612	1.12500000	3.62500000	0.16246334
613	1.12500000	3.50000000	0.14799609
614	0.87500000	3.50000000	0.17184751
615	0.87500000	3.37500000	0.17106549
616	3.37500000	0.37500000	0.04516129
617	3.62500000	0.62500000	-0.05493646
618	3.50000000	0.37500000	-0.06353861
619	3.62500000	0.50000000	-0.05650049
620	3.37500000	0.50000000	-0.00840665
621	3.50000000	0.62500000	-0.04046921
622	3.00000000	0.37500000	0.02521994
623	3.00000000	0.62500000	-0.04711632
624	2.87500000	0.37500000	0.06705767
625	2.87500000	0.50000000	-0.09599218
626	3.12500000	0.50000000	0.02678397
627	3.12500000	0.62500000	-0.04164223
628	3.62500000	1.00000000	-0.17184751
629	3.37500000	1.00000000	0.01505376
630	3.62500000	1.12500000	-0.15503421
631	3.50000000	1.12500000	-0.15933529
632	3.50000000	0.87500000	-0.11241447
633	3.37500000	0.87500000	-0.11085044
634	3.00000000	3.37500000	0.16559140
635	3.00000000	3.62500000	0.16637341
636	3.12500000	3.50000000	0.19608993
637	3.12500000	3.62500000	-0.07996090
638	2.87500000	3.37500000	0.19921799
639	2.87500000	3.50000000	0.05415445
640	3.37500000	3.00000000	0.11006843
641	3.62500000	3.00000000	0.08934506
642	3.50000000	3.12500000	0.14838710
643	3.62500000	3.12500000	0.19530792
644	3.37500000	2.87500000	0.08426197
645	3.50000000	2.87500000	0.09951124
646	2.62500000	3.00000000	0.15542522

647	2.37500000	3.00000000	0.17849462
648	2.50000000	2.87500000	0.14565005
649	2.37500000	2.87500000	0.18044966
650	2.62500000	3.12500000	0.17145650
651	2.50000000	3.12500000	0.13313783
652	3.00000000	2.62500000	-0.06041056
653	3.00000000	2.37500000	-0.14799609
654	2.87500000	2.50000000	-0.04516129
655	2.87500000	2.37500000	-0.09286413
656	3.12500000	2.62500000	-0.03929619
657	3.12500000	2.50000000	-0.07956989
658	2.62500000	3.62500000	0.00019550
659	2.37500000	3.37500000	0.05415445
660	2.50000000	3.62500000	0.06471163
661	2.37500000	3.50000000	-0.01466276
662	2.62500000	3.50000000	0.07800587
663	2.50000000	3.37500000	0.07057674
664	3.62500000	2.62500000	0.19648094
665	3.37500000	2.37500000	0.02913001
666	3.62500000	2.50000000	0.04672532
667	3.50000000	2.37500000	0.05102639
668	3.50000000	2.62500000	0.11906158
669	3.37500000	2.50000000	0.07605083
670	1.00000000	2.37500000	-0.08504399
671	1.00000000	2.62500000	-0.09833822
672	0.87500000	2.37500000	0.04125122
673	0.87500000	2.50000000	-0.06744868
674	1.12500000	2.50000000	-0.03460411
675	1.12500000	2.62500000	0.00723363
676	1.37500000	2.37500000	-0.04437928
677	1.62500000	2.62500000	0.07917889
678	1.50000000	2.37500000	-0.06236559
679	1.62500000	2.50000000	0.08856305
680	1.37500000	2.50000000	-0.00097752
681	1.50000000	2.62500000	0.04828935
682	1.37500000	3.00000000	0.00957967
683	1.62500000	3.00000000	0.02209189
684	1.50000000	3.12500000	0.02091887
685	1.62500000	3.12500000	-0.06236559
686	1.37500000	2.87500000	-0.03343109
687	1.50000000	2.87500000	-0.02600196
688	2.37500000	1.00000000	-0.07487781
689	2.62500000	1.00000000	-0.02326491
690	2.37500000	0.87500000	0.00136852
691	2.50000000	0.87500000	-0.07096774
692	2.50000000	1.12500000	-0.09794721
693	2.62500000	1.12500000	-0.12023460
694	2.37500000	1.37500000	0.00997067
695	2.62500000	1.62500000	-0.13665689
696	2.37500000	1.50000000	-0.13196481
697	2.50000000	1.62500000	-0.15698925
698	2.50000000	1.37500000	-0.11241447
699	2.62500000	1.50000000	-0.13587488
700	3.00000000	1.37500000	-0.19217986
701	3.00000000	1.62500000	-0.19217986
702	3.12500000	1.50000000	-0.19061584
703	3.12500000	1.62500000	-0.17380254
704	2.87500000	1.37500000	-0.16715543
705	2.87500000	1.50000000	-0.16793744

706	0.12500000	0.25000000	0.02873900
707	0.12500000	0.37500000	-0.06158358
708	0.25000000	0.12500000	0.08035191
709	0.37500000	0.12500000	0.09716520
710	0.25000000	0.37500000	-0.06275660
711	0.37500000	0.25000000	0.05415445
712	0.12500000	3.87500000	0.09560117
713	0.12500000	3.75000000	0.10615836
714	0.25000000	3.87500000	0.08191593
715	3.87500000	0.12500000	0.10185728
716	3.75000000	0.12500000	-0.00957967
717	3.87500000	0.25000000	-0.06432063
718	3.75000000	3.87500000	0.08817204
719	3.87500000	3.75000000	0.14525904
720	3.62500000	3.75000000	0.10224829
721	3.75000000	3.62500000	0.15855327
722	3.62500000	3.87500000	0.03968719
723	3.87500000	3.62500000	0.15307918
724	0.12500000	1.87500000	-0.18435973
725	0.12500000	1.75000000	-0.19530792
726	0.12500000	2.25000000	-0.05962854
727	0.12500000	2.37500000	-0.03499511
728	0.25000000	2.12500000	-0.16676442
729	0.25000000	1.87500000	-0.18201369
730	0.37500000	2.12500000	-0.15229717
731	0.25000000	2.37500000	-0.13782991
732	0.37500000	2.25000000	-0.14799609
733	1.87500000	0.12500000	0.19061584
734	1.75000000	0.12500000	0.18357771
735	2.25000000	0.12500000	0.16246334
736	2.37500000	0.12500000	0.14877810
737	2.12500000	0.25000000	0.16246334
738	1.87500000	0.25000000	0.18983382
739	2.12500000	0.37500000	0.15073314
740	2.37500000	0.25000000	0.11397849
741	2.25000000	0.37500000	0.11593353
742	1.75000000	1.87500000	-0.00606061
743	1.87500000	1.75000000	0.01739980
744	1.62500000	1.75000000	-0.03773216
745	1.75000000	1.62500000	-0.01427175
746	2.12500000	2.25000000	0.08465298
747	2.25000000	2.12500000	0.03264907
748	2.25000000	2.37500000	0.09325513
749	2.37500000	2.25000000	0.04164223
750	1.87500000	2.12500000	0.04477028
751	1.62500000	1.87500000	-0.03773216
752	1.75000000	2.12500000	0.01348974
753	2.12500000	1.87500000	0.01622678
754	1.87500000	1.62500000	0.00918866
755	2.12500000	1.75000000	0.00840665
756	2.12500000	2.37500000	0.10420332
757	1.87500000	2.25000000	0.06432063
758	2.37500000	2.12500000	0.00019550
759	2.25000000	1.87500000	-0.01661779
760	1.75000000	3.87500000	0.09521017
761	1.62500000	3.87500000	0.13079179
762	2.12500000	3.87500000	-0.02873900
763	2.25000000	3.87500000	0.00645161
764	1.87500000	3.75000000	0.00918866

765	1.62500000	3.75000000	0.13196481
766	1.75000000	3.62500000	0.05845552
767	2.12500000	3.75000000	-0.04203324
768	1.87500000	3.62500000	-0.02873900
769	3.87500000	1.75000000	-0.19022483
770	3.87500000	1.62500000	-0.18396872
771	3.87500000	2.12500000	-0.14369501
772	3.87500000	2.25000000	-0.11124145
773	3.75000000	1.87500000	-0.17966764
774	3.75000000	1.62500000	-0.18826979
775	3.62500000	1.75000000	-0.17810362
776	3.75000000	2.12500000	-0.13040078
777	3.62500000	1.87500000	-0.16050831
778	0.12500000	0.87500000	-0.15581623
779	0.12500000	0.75000000	-0.10889541
780	0.12500000	1.25000000	-0.19608993
781	0.12500000	1.37500000	-0.18279570
782	0.25000000	1.12500000	-0.19530792
783	0.25000000	0.87500000	-0.14721408
784	0.37500000	1.12500000	-0.19335288
785	0.25000000	1.37500000	-0.18279570
786	0.37500000	1.25000000	-0.19491691
787	0.87500000	0.12500000	0.05298143
788	0.75000000	0.12500000	0.07526882
789	1.25000000	0.12500000	0.16911046
790	1.37500000	0.12500000	0.07917889
791	1.12500000	0.25000000	0.04437928
792	0.87500000	0.25000000	0.01309873
793	1.12500000	0.37500000	-0.01935484
794	1.37500000	0.25000000	0.10576735
795	1.25000000	0.37500000	0.03147605
796	0.75000000	0.87500000	-0.15698925
797	0.87500000	0.75000000	-0.13939394
798	0.62500000	0.75000000	-0.11593353
799	0.75000000	0.62500000	-0.09716520
800	1.12500000	1.25000000	-0.16324536
801	1.25000000	1.12500000	-0.14134897
802	1.25000000	1.37500000	-0.13352884
803	1.37500000	1.25000000	-0.11006843
804	0.87500000	1.12500000	-0.19804497
805	0.62500000	0.87500000	-0.15542522
806	0.75000000	1.12500000	-0.19491691
807	1.12500000	0.87500000	-0.15581623
808	0.87500000	0.62500000	-0.11945259
809	1.12500000	0.75000000	-0.13900293
810	1.12500000	1.37500000	-0.16871945
811	0.87500000	1.25000000	-0.19648094
812	1.37500000	1.12500000	-0.11867058
813	1.25000000	0.87500000	-0.13352884
814	0.12500000	3.25000000	0.11867058
815	0.12500000	3.37500000	0.15933529
816	0.12500000	2.87500000	0.10537634
817	0.12500000	2.75000000	-0.06705767
818	0.25000000	3.12500000	0.12649071
819	0.25000000	3.37500000	0.12258065
820	0.37500000	3.25000000	0.13509286
821	0.25000000	2.87500000	0.00840665
822	0.37500000	3.12500000	0.18553275
823	0.75000000	3.87500000	0.09833822

824	0.62500000	3.87500000	-0.01153470
825	1.12500000	3.87500000	0.13939394
826	1.25000000	3.87500000	0.15386119
827	0.87500000	3.75000000	0.14213099
828	0.62500000	3.75000000	0.02561095
829	0.75000000	3.62500000	0.15659824
830	1.12500000	3.75000000	0.16363636
831	0.87500000	3.62500000	0.16363636
832	3.25000000	0.12500000	0.11671554
833	3.37500000	0.12500000	0.10224829
834	2.87500000	0.12500000	0.09442815
835	2.75000000	0.12500000	0.11984360
836	3.12500000	0.25000000	0.07174976
837	3.37500000	0.25000000	0.07996090
838	3.25000000	0.37500000	0.04750733
839	2.87500000	0.25000000	0.07370479
840	3.12500000	0.37500000	0.02443793
841	3.87500000	0.75000000	-0.14760508
842	3.87500000	0.62500000	-0.14799609
843	3.87500000	1.12500000	-0.19960899
844	3.87500000	1.25000000	-0.18005865
845	3.75000000	0.87500000	-0.15347019
846	3.75000000	0.62500000	-0.10772239
847	3.62500000	0.75000000	-0.09130010
848	3.75000000	1.12500000	-0.18084066
849	3.62500000	0.87500000	-0.13782991
850	3.12500000	3.25000000	0.14682307
851	3.25000000	3.12500000	0.13939394
852	3.25000000	3.37500000	0.15933529
853	3.37500000	3.25000000	0.18787879
854	2.75000000	2.87500000	0.09130010
855	2.87500000	2.75000000	0.02365591
856	2.62500000	2.75000000	0.08152493
857	2.75000000	2.62500000	0.03890518
858	2.87500000	3.12500000	0.14173998
859	3.12500000	3.37500000	0.16207234
860	2.87500000	3.25000000	0.15816227
861	3.12500000	2.87500000	0.06041056
862	3.37500000	3.12500000	0.13313783
863	3.25000000	2.87500000	0.08191593
864	2.62500000	2.87500000	0.13704790
865	2.75000000	3.12500000	0.16285435
866	2.87500000	2.62500000	0.02639296
867	3.12500000	2.75000000	0.03264907
868	3.12500000	3.87500000	-0.00371457
869	3.25000000	3.87500000	0.00527859
870	2.75000000	3.87500000	-0.01388074
871	2.62500000	3.87500000	0.05063539
872	2.87500000	3.75000000	-0.00019550
873	3.12500000	3.75000000	0.04711632
874	2.87500000	3.62500000	0.07761486
875	2.62500000	3.75000000	0.02561095
876	2.75000000	3.62500000	0.03929619
877	3.87500000	3.12500000	0.08699902
878	3.87500000	3.25000000	0.12649071
879	3.87500000	2.75000000	0.10928641
880	3.87500000	2.62500000	-0.14095797
881	3.75000000	2.87500000	-0.08308895
882	3.75000000	3.12500000	0.18475073

883	3.62500000	2.87500000	0.08973607
884	3.75000000	2.62500000	-0.13548387
885	3.62500000	2.75000000	-0.16324536
886	0.75000000	1.87500000	-0.08543500
887	0.87500000	2.12500000	-0.04203324
888	0.62500000	1.87500000	-0.07214076
889	0.75000000	2.12500000	0.08426197
890	1.12500000	1.87500000	-0.16441838
891	1.25000000	2.12500000	-0.11397849
892	1.25000000	1.87500000	-0.13235582
893	1.37500000	2.12500000	-0.13196481
894	0.87500000	1.75000000	-0.19608993
895	0.62500000	1.75000000	-0.18279570
896	0.75000000	1.62500000	-0.19608993
897	1.12500000	1.75000000	-0.16441838
898	0.87500000	1.62500000	-0.19608993
899	1.12500000	2.25000000	-0.10224829
900	0.87500000	2.25000000	0.01700880
901	1.12500000	2.37500000	-0.05884653
902	1.37500000	2.25000000	-0.11319648
903	1.25000000	2.37500000	-0.05180841
904	0.75000000	2.87500000	0.08973607
905	0.87500000	2.75000000	-0.06236559
906	0.62500000	2.75000000	0.02130987
907	0.75000000	2.62500000	-0.03108504
908	1.12500000	3.25000000	0.16637341
909	1.25000000	3.12500000	0.16676442
910	1.25000000	3.37500000	0.15268817
911	1.37500000	3.25000000	0.17966764
912	0.87500000	3.12500000	0.16285435
913	0.62500000	2.87500000	0.09951124
914	0.75000000	3.12500000	0.17223851
915	1.12500000	3.37500000	0.14369501
916	0.87500000	3.25000000	0.16676442
917	1.12500000	2.87500000	0.00019550
918	0.87500000	2.62500000	-0.10537634
919	1.12500000	2.75000000	-0.03030303
920	1.37500000	3.12500000	0.17419355
921	1.25000000	2.87500000	0.11827957
922	1.87500000	0.75000000	0.05454545
923	2.12500000	0.87500000	0.02091887
924	1.87500000	0.62500000	0.06823069
925	2.12500000	0.75000000	0.09090909
926	1.87500000	1.12500000	0.07448680
927	2.12500000	1.25000000	0.07683284
928	1.87500000	1.25000000	-0.01583578
929	2.12500000	1.37500000	-0.06510264
930	1.75000000	0.87500000	0.08387097
931	1.75000000	0.62500000	0.07253177
932	1.62500000	0.75000000	0.00332356
933	1.75000000	1.12500000	0.05024438
934	1.62500000	0.87500000	0.06001955
935	2.25000000	1.12500000	0.04672532
936	2.25000000	0.87500000	0.02365591
937	2.37500000	1.12500000	-0.07565982
938	2.25000000	1.37500000	0.00371457
939	2.37500000	1.25000000	0.02326491
940	2.87500000	0.75000000	-0.09677419
941	2.75000000	0.87500000	-0.02248289

942	2.75000000	0.62500000	-0.14565005
943	2.62500000	0.75000000	-0.07057674
944	3.25000000	1.12500000	-0.18318671
945	3.12500000	1.25000000	-0.19491691
946	3.37500000	1.25000000	-0.18279570
947	3.25000000	1.37500000	-0.19335288
948	3.12500000	0.87500000	-0.13431085
949	2.87500000	0.62500000	-0.14643206
950	3.12500000	0.75000000	-0.08543500
951	3.37500000	1.12500000	-0.17067449
952	3.25000000	0.87500000	-0.12179863
953	2.87500000	1.12500000	-0.07174976
954	2.62500000	0.87500000	0.00293255
955	2.75000000	1.12500000	-0.04633431
956	3.12500000	1.37500000	-0.19296188
957	2.87500000	1.25000000	-0.16911046
958	2.12500000	2.87500000	0.17966764
959	1.87500000	2.75000000	0.15933529
960	2.12500000	2.75000000	0.18162268
961	1.87500000	2.62500000	0.16832845
962	2.12500000	3.25000000	0.04594330
963	1.87500000	3.12500000	0.10459433
964	2.12500000	3.37500000	-0.02287390
965	1.87500000	3.25000000	0.02013685
966	2.25000000	3.12500000	0.13743891
967	2.25000000	2.87500000	0.19530792
968	2.37500000	3.12500000	0.14760508
969	2.25000000	3.37500000	0.00019550
970	2.37500000	3.25000000	0.08582600
971	1.75000000	2.87500000	0.16911046
972	1.75000000	2.62500000	0.16129032
973	1.62500000	2.75000000	0.12140762
974	1.75000000	3.12500000	0.10146628
975	1.62500000	2.87500000	0.13822092
976	2.87500000	2.12500000	-0.17380254
977	2.75000000	1.87500000	-0.16598240
978	2.75000000	2.12500000	-0.11906158
979	2.62500000	1.87500000	-0.18631476
980	3.25000000	2.12500000	-0.14134897
981	3.12500000	1.87500000	-0.17810362
982	3.37500000	2.12500000	-0.11945259
983	3.25000000	1.87500000	-0.15972630
984	3.12500000	2.25000000	-0.14173998
985	2.87500000	2.25000000	-0.10850440
986	3.12500000	2.37500000	-0.09481916
987	3.37500000	2.25000000	-0.09833822
988	3.25000000	2.37500000	-0.07331378
989	2.87500000	1.75000000	-0.17067449
990	2.62500000	1.75000000	-0.16754643
991	2.75000000	1.62500000	-0.14643206
992	3.12500000	1.75000000	-0.17536657
993	2.87500000	1.62500000	-0.16950147
994	0.25000000	0.62500000	-0.11945259
995	0.37500000	0.62500000	-0.12649071
996	0.37500000	0.75000000	-0.10733138
997	0.62500000	0.25000000	0.05180841
998	0.62500000	0.37500000	-0.00332356
999	0.75000000	0.37500000	-0.02209189
1000	0.25000000	3.62500000	0.12805474

1001	0.37500000	3.75000000	0.00410557
1002	0.37500000	3.62500000	0.03968719
1003	3.62500000	0.25000000	-0.05493646
1004	3.75000000	0.37500000	-0.14213099
1005	3.62500000	0.37500000	-0.02130987
1006	3.37500000	3.62500000	0.02013685
1007	3.37500000	3.75000000	0.05454545
1008	3.62500000	3.37500000	0.16168133
1009	3.75000000	3.37500000	0.15777126
1010	3.25000000	3.62500000	0.12140762
1011	3.62500000	3.25000000	0.15307918
1012	0.25000000	1.62500000	-0.18123167
1013	0.37500000	1.75000000	-0.18162268
1014	0.37500000	1.62500000	-0.16950147
1015	0.25000000	2.62500000	-0.13861193
1016	0.37500000	2.62500000	-0.04750733
1017	0.37500000	2.75000000	-0.16676442
1018	0.62500000	2.25000000	0.00488759
1019	0.62500000	2.37500000	-0.10381232
1020	0.75000000	2.37500000	0.13470186
1021	1.62500000	0.25000000	0.14956012
1022	1.75000000	0.37500000	0.14995112
1023	1.62500000	0.37500000	0.11671554
1024	2.62500000	0.25000000	0.09951124
1025	2.62500000	0.37500000	-0.04750733
1026	2.75000000	0.37500000	-0.04789834
1027	2.25000000	0.62500000	0.10224829
1028	2.37500000	0.62500000	0.02952102
1029	2.37500000	0.75000000	0.02756598
1030	1.37500000	1.62500000	-0.10185728
1031	1.37500000	1.75000000	-0.10107527
1032	1.62500000	1.37500000	-0.05493646
1033	1.75000000	1.37500000	-0.03147605
1034	1.25000000	1.62500000	-0.13352884
1035	1.62500000	1.25000000	-0.06353861
1036	2.37500000	2.62500000	0.11984360
1037	2.25000000	2.62500000	0.15464321
1038	2.62500000	2.37500000	-0.17497556
1039	2.62500000	2.25000000	-0.02248289
1040	2.37500000	2.75000000	0.16207234
1041	2.75000000	2.37500000	-0.00606061
1042	1.62500000	2.25000000	-0.04946237
1043	1.75000000	2.37500000	0.10146628
1044	1.62500000	2.37500000	0.01935484
1045	2.25000000	1.62500000	-0.08152493
1046	2.37500000	1.75000000	-0.10615836
1047	2.37500000	1.62500000	-0.16324536
1048	1.37500000	3.75000000	0.17302053
1049	1.37500000	3.62500000	0.14877810
1050	1.25000000	3.62500000	0.15425220
1051	2.37500000	3.75000000	0.04203324
1052	2.25000000	3.62500000	-0.03734115
1053	2.37500000	3.62500000	0.01075269
1054	1.62500000	3.37500000	0.10263930
1055	1.75000000	3.37500000	0.01896383
1056	1.62500000	3.25000000	0.10068426
1057	3.75000000	1.37500000	-0.19648094
1058	3.62500000	1.37500000	-0.15112414
1059	3.62500000	1.25000000	-0.16559140

1060	3.75000000	2.37500000	-0.09247312
1061	3.62500000	2.25000000	-0.08778104
1062	3.62500000	2.37500000	-0.08074291
1063	3.37500000	1.62500000	-0.17614858
1064	3.37500000	1.75000000	-0.15933529
1065	3.25000000	1.62500000	-0.17419355
1066	0.62500000	1.25000000	-0.19335288
1067	0.62500000	1.37500000	-0.19452590
1068	0.75000000	1.37500000	-0.19452590
1069	1.25000000	0.62500000	-0.07448680
1070	1.37500000	0.62500000	-0.02678397
1071	1.37500000	0.75000000	-0.06862170
1072	0.62500000	3.37500000	0.18318671
1073	0.62500000	3.25000000	0.18240469
1074	0.75000000	3.37500000	0.17732160
1075	3.37500000	0.62500000	-0.03773216
1076	3.25000000	0.62500000	-0.03655914
1077	3.37500000	0.75000000	-0.07448680
1078	2.75000000	3.37500000	0.14799609
1079	2.62500000	3.25000000	0.14056696
1080	2.62500000	3.37500000	0.10967742
1081	3.37500000	2.75000000	0.05650049
1082	3.25000000	2.62500000	0.01348974
1083	3.37500000	2.62500000	0.01622678
1084	1.25000000	2.62500000	0.03343109
1085	1.37500000	2.62500000	0.04086022
1086	1.37500000	2.75000000	0.08269795
1087	2.62500000	1.25000000	-0.12140762
1088	2.62500000	1.37500000	-0.12218964
1089	2.75000000	1.37500000	-0.14447703
1090	0.00000000	0.06250000	0.12570870
1091	0.06250000	0.00000000	0.14995112
1092	0.06250000	0.06250000	0.12864125
1093	0.00000000	3.93750000	0.10576735
1094	0.06250000	4.00000000	0.09364614
1095	3.93750000	0.00000000	0.08543500
1096	4.00000000	0.06250000	0.10811339
1097	3.93750000	3.93750000	0.12903226
1098	3.93750000	4.00000000	0.09775171
1099	4.00000000	3.93750000	0.12629521
1100	0.00000000	1.93750000	-0.09931574
1101	0.00000000	2.06250000	-0.13919844
1102	0.06250000	2.00000000	-0.19276637
1103	0.06250000	2.06250000	-0.19061584
1104	1.93750000	0.00000000	0.19511241
1105	2.06250000	0.00000000	0.19843597
1106	2.00000000	0.06250000	0.19843597
1107	2.06250000	0.06250000	0.18103617
1108	1.93750000	1.93750000	0.04125122
1109	2.06250000	2.06250000	0.06158358
1110	1.93750000	2.00000000	0.04144673
1111	2.00000000	1.93750000	0.05317693
1112	2.00000000	2.06250000	0.06686217
1113	2.06250000	2.00000000	0.04086022
1114	1.93750000	4.00000000	0.02170088
1115	2.06250000	4.00000000	0.00273705
1116	1.93750000	3.93750000	0.02209189
1117	2.00000000	3.93750000	-0.02287390
1118	4.00000000	1.93750000	-0.19511241

1119	4.00000000	2.06250000	-0.17927664
1120	3.93750000	1.93750000	-0.19648094
1121	3.93750000	2.00000000	-0.18963832
1122	0.00000000	0.93750000	-0.17693060
1123	0.00000000	1.06250000	-0.19667644
1124	0.06250000	1.00000000	-0.19608993
1125	0.06250000	1.06250000	-0.19687195
1126	0.93750000	0.00000000	0.11906158
1127	1.06250000	0.00000000	0.09696970
1128	1.00000000	0.06250000	0.05982405
1129	1.06250000	0.06250000	0.11026393
1130	0.93750000	0.93750000	-0.17614858
1131	1.06250000	1.06250000	-0.18005865
1132	0.93750000	1.00000000	-0.19550342
1133	1.00000000	0.93750000	-0.18729228
1134	1.00000000	1.06250000	-0.19765396
1135	1.06250000	1.00000000	-0.18435973
1136	0.00000000	3.06250000	0.11886608
1137	0.00000000	2.93750000	0.13743891
1138	0.06250000	3.06250000	0.15053763
1139	0.06250000	3.00000000	0.17556207
1140	0.93750000	4.00000000	0.07663734
1141	1.06250000	4.00000000	0.09169110
1142	0.93750000	3.93750000	0.09501466
1143	1.00000000	3.93750000	0.09833822
1144	3.06250000	0.00000000	0.10439883
1145	2.93750000	0.00000000	0.10439883
1146	3.06250000	0.06250000	0.06217009
1147	3.00000000	0.06250000	0.08172043
1148	4.00000000	0.93750000	-0.19550342
1149	4.00000000	1.06250000	-0.19824047
1150	3.93750000	0.93750000	-0.17517107
1151	3.93750000	1.00000000	-0.19863148
1152	3.06250000	3.06250000	0.09247312
1153	2.93750000	2.93750000	0.06862170
1154	3.00000000	3.06250000	0.09403715
1155	3.06250000	3.00000000	0.08719453
1156	2.93750000	3.00000000	0.09638319
1157	3.00000000	2.93750000	0.06256109
1158	3.06250000	4.00000000	-0.10009775
1159	2.93750000	4.00000000	-0.13607038
1160	3.00000000	3.93750000	-0.07917889
1161	2.93750000	3.93750000	-0.09833822
1162	4.00000000	3.06250000	0.06373412
1163	4.00000000	2.93750000	0.03851417
1164	3.93750000	3.00000000	0.04261975
1165	3.93750000	2.93750000	0.09130010
1166	0.93750000	2.00000000	-0.14037146
1167	1.06250000	2.00000000	-0.17986315
1168	0.93750000	1.93750000	-0.19589443
1169	1.00000000	1.93750000	-0.19589443
1170	1.00000000	2.06250000	-0.17399804
1171	1.06250000	2.06250000	-0.17047898
1172	0.93750000	2.93750000	0.11476051
1173	1.06250000	3.06250000	0.15562072
1174	0.93750000	3.00000000	0.15386119
1175	1.00000000	3.06250000	0.15444770
1176	1.00000000	2.93750000	0.07781036
1177	1.06250000	3.00000000	0.15268817

1178	2.00000000	0.93750000	0.01759531
1179	2.00000000	1.06250000	-0.00391007
1180	1.93750000	0.93750000	0.00097752
1181	1.93750000	1.00000000	0.02913001
1182	2.06250000	1.00000000	-0.01915934
1183	2.06250000	1.06250000	0.03049853
1184	2.93750000	0.93750000	-0.17165200
1185	3.06250000	1.06250000	-0.14799609
1186	3.00000000	0.93750000	-0.17341153
1187	3.06250000	1.00000000	-0.19276637
1188	2.93750000	1.00000000	-0.13646139
1189	3.00000000	1.06250000	-0.19784946
1190	2.00000000	2.93750000	0.18690127
1191	2.00000000	3.06250000	0.15425220
1192	2.06250000	3.00000000	0.19374389
1193	2.06250000	3.06250000	0.16578690
1194	1.93750000	2.93750000	0.18025415
1195	1.93750000	3.00000000	0.19511241
1196	2.93750000	2.00000000	-0.19178886
1197	3.06250000	2.00000000	-0.18631476
1198	3.00000000	2.06250000	-0.18651026
1199	3.06250000	2.06250000	-0.17556207
1200	2.93750000	1.93750000	-0.18260020
1201	3.00000000	1.93750000	-0.19452590
1202	0.00000000	0.43750000	-0.02306940
1203	0.00000000	0.56250000	-0.05767351
1204	0.06250000	0.50000000	-0.06295210
1205	0.06250000	0.56250000	-0.05337243
1206	0.43750000	0.00000000	0.16637341
1207	0.56250000	0.00000000	0.16383187
1208	0.50000000	0.06250000	0.15347019
1209	0.56250000	0.06250000	0.14389052
1210	0.43750000	0.43750000	-0.01994135
1211	0.56250000	0.56250000	-0.05884653
1212	0.43750000	0.50000000	-0.06412512
1213	0.50000000	0.43750000	-0.01173021
1214	0.50000000	0.56250000	-0.05845552
1215	0.56250000	0.50000000	-0.06119257
1216	0.00000000	3.56250000	0.13294233
1217	0.00000000	3.43750000	0.14193548
1218	0.06250000	3.56250000	0.12629521
1219	0.06250000	3.50000000	0.14389052
1220	0.43750000	4.00000000	0.04926686
1221	0.56250000	4.00000000	0.04633431
1222	0.43750000	3.93750000	0.05728250
1223	0.50000000	3.93750000	-0.03929619
1224	3.56250000	0.00000000	0.17790811
1225	3.43750000	0.00000000	0.17302053
1226	3.56250000	0.06250000	0.14525904
1227	3.50000000	0.06250000	0.15464321
1228	4.00000000	0.43750000	0.13528837
1229	4.00000000	0.56250000	0.07663734
1230	3.93750000	0.43750000	0.05474096
1231	3.93750000	0.50000000	0.09618768
1232	3.56250000	3.56250000	0.17243402
1233	3.43750000	3.43750000	0.17067449
1234	3.50000000	3.56250000	0.14819159
1235	3.56250000	3.50000000	0.17634409
1236	3.43750000	3.50000000	0.18162268

1237	3.50000000	3.43750000	0.17399804
1238	3.56250000	4.00000000	-0.09638319
1239	3.43750000	4.00000000	-0.06392962
1240	3.50000000	3.93750000	-0.05180841
1241	3.43750000	3.93750000	-0.03870968
1242	4.00000000	3.56250000	0.15366569
1243	4.00000000	3.43750000	0.14467253
1244	3.93750000	3.50000000	0.16031281
1245	3.93750000	3.43750000	0.15581623
1246	0.00000000	1.56250000	-0.14525904
1247	0.00000000	1.43750000	-0.19491691
1248	0.06250000	1.56250000	-0.19433040
1249	0.06250000	1.50000000	-0.18826979
1250	0.00000000	2.43750000	-0.07487781
1251	0.00000000	2.56250000	-0.07839687
1252	0.06250000	2.50000000	-0.11417400
1253	0.06250000	2.56250000	-0.12688172
1254	0.43750000	2.00000000	-0.15014663
1255	0.56250000	2.00000000	-0.10107527
1256	0.43750000	1.93750000	-0.15581623
1257	0.50000000	2.06250000	-0.14076246
1258	0.50000000	1.93750000	-0.14995112
1259	0.56250000	2.06250000	-0.07174976
1260	0.43750000	2.43750000	-0.13450635
1261	0.56250000	2.56250000	-0.09090909
1262	0.43750000	2.50000000	-0.12394917
1263	0.50000000	2.43750000	-0.12727273
1264	0.50000000	2.56250000	-0.08621701
1265	0.56250000	2.50000000	-0.11241447
1266	1.56250000	0.00000000	0.17321603
1267	1.43750000	0.00000000	0.13782991
1268	1.56250000	0.06250000	0.17282502
1269	1.50000000	0.06250000	0.15601173
1270	2.43750000	0.00000000	0.19042033
1271	2.56250000	0.00000000	0.17634409
1272	2.50000000	0.06250000	0.16637341
1273	2.56250000	0.06250000	0.16598240
1274	2.00000000	0.43750000	0.19608993
1275	2.00000000	0.56250000	0.16715543
1276	1.93750000	0.43750000	0.19257087
1277	2.06250000	0.50000000	0.17282502
1278	1.93750000	0.50000000	0.17614858
1279	2.06250000	0.56250000	0.17184751
1280	2.43750000	0.43750000	0.03675464
1281	2.56250000	0.56250000	-0.01935484
1282	2.50000000	0.43750000	0.02991202
1283	2.43750000	0.50000000	0.03088954
1284	2.56250000	0.50000000	-0.04398827
1285	2.50000000	0.56250000	-0.00645161
1286	1.56250000	1.56250000	-0.05395894
1287	1.43750000	1.43750000	-0.09794721
1288	1.50000000	1.56250000	-0.06959922
1289	1.56250000	1.50000000	-0.05786901
1290	1.43750000	1.50000000	-0.08602151
1291	1.50000000	1.43750000	-0.07409580
1292	2.43750000	2.43750000	0.08641251
1293	2.56250000	2.56250000	0.07683284
1294	2.43750000	2.50000000	0.09599218
1295	2.50000000	2.43750000	0.06392962

1296	2.50000000	2.56250000	0.09970674
1297	2.56250000	2.50000000	0.07839687
1298	1.56250000	2.00000000	-0.05317693
1299	1.43750000	2.00000000	-0.08484848
1300	1.50000000	1.93750000	-0.06901271
1301	1.56250000	2.06250000	-0.04359726
1302	1.43750000	1.93750000	-0.08484848
1303	1.50000000	2.06250000	-0.08465298
1304	2.00000000	1.56250000	0.02873900
1305	2.00000000	1.43750000	0.02072336
1306	1.93750000	1.50000000	0.01309873
1307	2.06250000	1.56250000	0.02502444
1308	1.93750000	1.43750000	0.00703812
1309	2.06250000	1.50000000	0.08426197
1310	2.00000000	2.43750000	0.13685239
1311	2.00000000	2.56250000	0.14037146
1312	2.06250000	2.50000000	0.12864125
1313	1.93750000	2.43750000	0.10869990
1314	2.06250000	2.56250000	0.15131965
1315	1.93750000	2.50000000	0.15874878
1316	2.43750000	2.00000000	-0.05826002
1317	2.56250000	2.00000000	-0.10303030
1318	2.50000000	2.06250000	-0.05395894
1319	2.43750000	1.93750000	0.03773216
1320	2.56250000	2.06250000	-0.07331378
1321	2.50000000	1.93750000	-0.05317693
1322	1.56250000	4.00000000	0.04144673
1323	1.43750000	4.00000000	0.03147605
1324	1.50000000	3.93750000	0.03460411
1325	1.43750000	3.93750000	0.03734115
1326	2.43750000	4.00000000	0.10752688
1327	2.56250000	4.00000000	0.10674487
1328	2.43750000	3.93750000	0.11554252
1329	2.50000000	3.93750000	0.14115347
1330	1.56250000	3.56250000	0.00175953
1331	1.43750000	3.43750000	0.03812317
1332	1.50000000	3.56250000	0.03792766
1333	1.56250000	3.50000000	0.00136852
1334	1.43750000	3.50000000	0.02854350
1335	1.50000000	3.43750000	0.01720430
1336	2.00000000	3.56250000	-0.09540567
1337	2.00000000	3.43750000	-0.07096774
1338	2.06250000	3.56250000	-0.08132942
1339	1.93750000	3.50000000	-0.07135875
1340	2.06250000	3.50000000	-0.10009775
1341	1.93750000	3.43750000	-0.08484848
1342	4.00000000	1.56250000	-0.17673509
1343	4.00000000	1.43750000	-0.05337243
1344	3.93750000	1.50000000	-0.16402737
1345	3.93750000	1.43750000	-0.16911046
1346	4.00000000	2.43750000	-0.05513196
1347	4.00000000	2.56250000	-0.07331378
1348	3.93750000	2.43750000	-0.08641251
1349	3.93750000	2.50000000	-0.09560117
1350	3.56250000	1.56250000	-0.16754643
1351	3.43750000	1.43750000	-0.15816227
1352	3.56250000	1.50000000	-0.18963832
1353	3.50000000	1.56250000	-0.05865103
1354	3.50000000	1.43750000	-0.12375367

1355	3.43750000	1.50000000	-0.19159335
1356	3.56250000	2.00000000	-0.15698925
1357	3.43750000	2.00000000	-0.08895406
1358	3.56250000	2.06250000	-0.07292278
1359	3.50000000	1.93750000	-0.08856305
1360	3.50000000	2.06250000	-0.01739980
1361	3.43750000	1.93750000	-0.13763441
1362	0.43750000	1.00000000	-0.19530792
1363	0.56250000	1.00000000	-0.19433040
1364	0.43750000	0.93750000	-0.17067449
1365	0.50000000	0.93750000	-0.12375367
1366	0.50000000	1.06250000	-0.05434995
1367	0.56250000	1.06250000	-0.19276637
1368	0.43750000	1.43750000	-0.19276637
1369	0.56250000	1.56250000	-0.08973607
1370	0.43750000	1.50000000	-0.19569892
1371	0.50000000	1.56250000	-0.18963832
1372	0.50000000	1.43750000	-0.19530792
1373	0.56250000	1.50000000	-0.19608993
1374	1.00000000	0.43750000	-0.09755621
1375	1.00000000	0.56250000	-0.13646139
1376	0.93750000	0.43750000	-0.08621701
1377	0.93750000	0.50000000	-0.11534702
1378	1.06250000	0.50000000	-0.10400782
1379	1.06250000	0.56250000	-0.12590420
1380	1.43750000	0.43750000	0.07820137
1381	1.56250000	0.56250000	0.05434995
1382	1.50000000	0.43750000	0.07644184
1383	1.56250000	0.50000000	-0.02346041
1384	1.43750000	0.50000000	0.03929619
1385	1.50000000	0.56250000	-0.05786901
1386	1.00000000	1.43750000	-0.19550342
1387	1.00000000	1.56250000	-0.19608993
1388	1.06250000	1.50000000	-0.18064516
1389	1.06250000	1.56250000	-0.17947214
1390	0.93750000	1.43750000	-0.19530792
1391	0.93750000	1.50000000	-0.19296188
1392	1.43750000	1.00000000	-0.10400782
1393	1.56250000	1.00000000	0.00625611
1394	1.50000000	1.06250000	-0.10087977
1395	1.56250000	1.06250000	-0.08914956
1396	1.43750000	0.93750000	-0.10811339
1397	1.50000000	0.93750000	-0.08426197
1398	0.43750000	3.43750000	0.16304985
1399	0.56250000	3.56250000	0.17653959
1400	0.43750000	3.50000000	0.18357771
1401	0.50000000	3.56250000	0.02150538
1402	0.50000000	3.43750000	0.18983382
1403	0.56250000	3.50000000	0.18729228
1404	0.43750000	3.00000000	0.15913978
1405	0.56250000	3.00000000	0.18162268
1406	0.43750000	2.93750000	0.09872923
1407	0.50000000	2.93750000	0.14428152
1408	0.50000000	3.06250000	0.18611926
1409	0.56250000	3.06250000	0.18357771
1410	1.00000000	3.56250000	0.16148583
1411	1.00000000	3.43750000	0.16363636
1412	1.06250000	3.56250000	0.16402737
1413	1.06250000	3.50000000	0.15679374

1414	0.93750000	3.50000000	0.16871945
1415	0.93750000	3.43750000	0.16832845
1416	3.43750000	0.43750000	0.02013685
1417	3.56250000	0.56250000	-0.02991202
1418	3.50000000	0.43750000	-0.03421310
1419	3.56250000	0.50000000	-0.03069404
1420	3.43750000	0.50000000	-0.00664712
1421	3.50000000	0.56250000	-0.02267840
1422	3.00000000	0.43750000	0.01427175
1423	3.00000000	0.56250000	-0.02189638
1424	2.93750000	0.43750000	0.03519062
1425	2.93750000	0.50000000	-0.04633431
1426	3.06250000	0.50000000	0.01505376
1427	3.06250000	0.56250000	-0.01915934
1428	3.56250000	1.00000000	-0.16031281
1429	3.43750000	1.00000000	-0.06686217
1430	3.56250000	1.06250000	-0.15190616
1431	3.50000000	1.06250000	-0.15405670
1432	3.50000000	0.93750000	-0.13059629
1433	3.43750000	0.93750000	-0.12981427
1434	3.00000000	3.43750000	0.17986315
1435	3.00000000	3.56250000	0.18025415
1436	3.06250000	3.50000000	0.19511241
1437	3.06250000	3.56250000	0.05708700
1438	2.93750000	3.43750000	0.19667644
1439	2.93750000	3.50000000	0.12414467
1440	3.43750000	3.00000000	0.12492669
1441	3.56250000	3.00000000	0.11456500
1442	3.50000000	3.06250000	0.14408602
1443	3.56250000	3.06250000	0.16754643
1444	3.43750000	2.93750000	0.11202346
1445	3.50000000	2.93750000	0.11964809
1446	2.56250000	3.00000000	0.15972630
1447	2.43750000	3.00000000	0.17126100
1448	2.50000000	2.93750000	0.15483871
1449	2.43750000	2.93750000	0.17223851
1450	2.56250000	3.06250000	0.16774194
1451	2.50000000	3.06250000	0.14858260
1452	3.00000000	2.56250000	-0.09638319
1453	3.00000000	2.43750000	-0.14017595
1454	2.93750000	2.50000000	-0.08875855
1455	2.93750000	2.43750000	-0.11260997
1456	3.06250000	2.56250000	-0.08582600
1457	3.06250000	2.50000000	-0.10596285
1458	2.56250000	3.56250000	0.01955034
1459	2.43750000	3.43750000	0.04652981
1460	2.50000000	3.56250000	0.05180841
1461	2.43750000	3.50000000	0.01212121
1462	2.56250000	3.50000000	0.05845552
1463	2.50000000	3.43750000	0.05474096
1464	3.56250000	2.56250000	0.18748778
1465	3.43750000	2.43750000	0.10381232
1466	3.56250000	2.50000000	0.11260997
1467	3.50000000	2.43750000	0.11476051
1468	3.50000000	2.56250000	0.14877810
1469	3.43750000	2.50000000	0.12727273
1470	1.00000000	2.43750000	-0.07292278
1471	1.00000000	2.56250000	-0.07956989
1472	0.93750000	2.43750000	-0.00977517

1473	0.93750000	2.50000000	-0.06412512
1474	1.06250000	2.50000000	-0.04770283
1475	1.06250000	2.56250000	-0.02678397
1476	1.43750000	2.43750000	-0.01896383
1477	1.56250000	2.56250000	0.04281525
1478	1.50000000	2.43750000	-0.02795699
1479	1.56250000	2.50000000	0.04750733
1480	1.43750000	2.50000000	0.00273705
1481	1.50000000	2.56250000	0.02737048
1482	1.43750000	3.00000000	-0.06627566
1483	1.56250000	3.00000000	-0.06001955
1484	1.50000000	3.06250000	-0.06060606
1485	1.56250000	3.06250000	-0.10224829
1486	1.43750000	2.93750000	-0.08778104
1487	1.50000000	2.93750000	-0.08406647
1488	2.43750000	1.00000000	-0.08602151
1489	2.56250000	1.00000000	-0.06021505
1490	2.43750000	0.93750000	-0.04789834
1491	2.50000000	0.93750000	-0.08406647
1492	2.50000000	1.06250000	-0.09755621
1493	2.56250000	1.06250000	-0.10869990
1494	2.43750000	1.43750000	-0.05806452
1495	2.56250000	1.56250000	-0.13137830
1496	2.43750000	1.50000000	-0.12903226
1497	2.50000000	1.56250000	-0.14154448
1498	2.50000000	1.43750000	-0.11925709
1499	2.56250000	1.50000000	-0.13098729
1500	3.00000000	1.43750000	-0.19120235
1501	3.00000000	1.56250000	-0.19120235
1502	3.06250000	1.50000000	-0.19042033
1503	3.06250000	1.56250000	-0.18201369
1504	2.93750000	1.43750000	-0.17869013
1505	2.93750000	1.50000000	-0.17908113
1506	0.00000000	0.18750000	0.03362659
1507	0.00000000	0.31250000	0.00254154
1508	0.06250000	0.25000000	0.02111437
1509	0.06250000	0.31250000	-0.02404692
1510	0.18750000	0.00000000	0.11260997
1511	0.31250000	0.00000000	0.13743891
1512	0.25000000	0.06250000	0.10166178
1513	0.31250000	0.06250000	0.11006843
1514	0.18750000	0.18750000	0.04868035
1515	0.31250000	0.31250000	0.01857283
1516	0.18750000	0.25000000	0.03323558
1517	0.25000000	0.18750000	0.05904203
1518	0.25000000	0.31250000	-0.01251222
1519	0.31250000	0.25000000	0.04594330
1520	0.00000000	3.81250000	0.11495601
1521	0.00000000	3.68750000	0.12394917
1522	0.06250000	3.81250000	0.10752688
1523	0.06250000	3.75000000	0.11280547
1524	0.18750000	4.00000000	0.07878788
1525	0.31250000	4.00000000	0.06432063
1526	0.18750000	3.93750000	0.08347996
1527	0.25000000	3.93750000	0.07663734
1528	3.81250000	0.00000000	0.09951124
1529	3.68750000	0.00000000	0.16402737
1530	3.81250000	0.06250000	0.12922776
1531	3.75000000	0.06250000	0.07350929

1532	4.00000000	0.18750000	0.06725318
1533	4.00000000	0.31250000	0.07624633
1534	3.93750000	0.18750000	0.07429130
1535	3.93750000	0.25000000	-0.00879765
1536	3.81250000	3.81250000	0.14349951
1537	3.68750000	3.68750000	0.15796676
1538	3.75000000	3.81250000	0.11945259
1539	3.81250000	3.75000000	0.14799609
1540	3.68750000	3.75000000	0.12649071
1541	3.75000000	3.68750000	0.15464321
1542	3.81250000	4.00000000	0.04965787
1543	3.68750000	4.00000000	-0.04848485
1544	3.75000000	3.93750000	0.05689150
1545	3.68750000	3.93750000	0.03264907
1546	4.00000000	3.81250000	0.13528837
1547	4.00000000	3.68750000	0.14447703
1548	3.93750000	3.75000000	0.14252199
1549	3.93750000	3.68750000	0.14643206
1550	0.00000000	1.81250000	-0.09814272
1551	0.00000000	1.68750000	-0.14682307
1552	0.06250000	1.81250000	-0.19100684
1553	0.06250000	1.75000000	-0.19648094
1554	0.00000000	2.18750000	-0.01759531
1555	0.00000000	2.31250000	0.00391007
1556	0.06250000	2.25000000	-0.00821114
1557	0.06250000	2.31250000	0.00410557
1558	0.18750000	2.00000000	-0.17829912
1559	0.31250000	2.00000000	-0.16402737
1560	0.18750000	1.93750000	-0.17771261
1561	0.25000000	2.06250000	-0.16891496
1562	0.25000000	1.93750000	-0.17653959
1563	0.31250000	2.06250000	-0.16168133
1564	0.18750000	2.18750000	-0.17184751
1565	0.31250000	2.31250000	-0.15307918
1566	0.18750000	2.25000000	-0.11104594
1567	0.25000000	2.18750000	-0.16461388
1568	0.25000000	2.31250000	-0.15014663
1569	0.31250000	2.25000000	-0.15522972
1570	1.81250000	0.00000000	0.18768328
1571	1.68750000	0.00000000	0.18044966
1572	1.81250000	0.06250000	0.18729228
1573	1.75000000	0.06250000	0.18377322
1574	2.18750000	0.00000000	0.19745846
1575	2.31250000	0.00000000	0.19472141
1576	2.25000000	0.06250000	0.17966764
1577	2.31250000	0.06250000	0.17282502
1578	2.00000000	0.18750000	0.19765396
1579	2.00000000	0.31250000	0.19687195
1580	1.93750000	0.18750000	0.19393939
1581	2.06250000	0.25000000	0.17986315
1582	1.93750000	0.25000000	0.19354839
1583	2.06250000	0.31250000	0.17399804
1584	2.18750000	0.18750000	0.14545455
1585	2.31250000	0.31250000	0.09736070
1586	2.25000000	0.18750000	0.14506354
1587	2.18750000	0.25000000	0.14506354
1588	2.31250000	0.25000000	0.12082111
1589	2.25000000	0.31250000	0.12179863
1590	1.81250000	1.81250000	0.00938416

1591	1.68750000	1.68750000	-0.02228739
1592	1.75000000	1.81250000	-0.00625611
1593	1.81250000	1.75000000	0.00547410
1594	1.68750000	1.75000000	-0.02209189
1595	1.75000000	1.68750000	-0.01036168
1596	2.18750000	2.18750000	0.06999022
1597	2.31250000	2.31250000	0.07820137
1598	2.18750000	2.25000000	0.07937439
1599	2.25000000	2.18750000	0.05337243
1600	2.25000000	2.31250000	0.08367546
1601	2.31250000	2.25000000	0.05786901
1602	1.81250000	2.00000000	0.00977517
1603	1.68750000	2.00000000	-0.02170088
1604	1.75000000	1.93750000	-0.00606061
1605	1.81250000	2.06250000	0.01935484
1606	1.68750000	1.93750000	-0.02189638
1607	1.75000000	2.06250000	0.00371457
1608	2.00000000	1.81250000	0.04496579
1609	2.00000000	1.68750000	0.03675464
1610	1.93750000	1.75000000	0.02913001
1611	2.06250000	1.81250000	0.02854350
1612	1.93750000	1.68750000	0.02502444
1613	2.06250000	1.75000000	0.02463343
1614	2.00000000	2.18750000	0.08602151
1615	2.00000000	2.31250000	0.11769306
1616	2.06250000	2.25000000	0.09012708
1617	1.93750000	2.18750000	0.07018573
1618	2.06250000	2.31250000	0.09990225
1619	1.93750000	2.25000000	0.07996090
1620	2.18750000	2.00000000	0.00782014
1621	2.31250000	2.00000000	-0.02521994
1622	2.25000000	2.06250000	0.01192571
1623	2.18750000	1.93750000	0.00371457
1624	2.31250000	2.06250000	-0.00430108
1625	2.25000000	1.93750000	-0.01270772
1626	1.81250000	4.00000000	0.06999022
1627	1.68750000	4.00000000	0.09325513
1628	1.75000000	3.93750000	0.09462366
1629	1.68750000	3.93750000	0.11241447
1630	2.18750000	4.00000000	0.01329423
1631	2.31250000	4.00000000	0.04711632
1632	2.18750000	3.93750000	-0.00508309
1633	2.25000000	3.93750000	0.01251222
1634	1.81250000	3.81250000	0.07135875
1635	1.68750000	3.68750000	0.05239492
1636	1.75000000	3.81250000	0.09560117
1637	1.81250000	3.75000000	0.05259042
1638	1.68750000	3.75000000	0.11397849
1639	1.75000000	3.68750000	0.07722385
1640	2.00000000	3.81250000	-0.06060606
1641	2.00000000	3.68750000	-0.08328446
1642	2.06250000	3.81250000	-0.05337243
1643	1.93750000	3.75000000	-0.03440860
1644	2.06250000	3.75000000	-0.06001955
1645	1.93750000	3.68750000	-0.05337243
1646	4.00000000	1.81250000	-0.18905181
1647	4.00000000	1.68750000	-0.18279570
1648	3.93750000	1.75000000	-0.18807429
1649	3.93750000	1.68750000	-0.18494624

1650	4.00000000	2.18750000	-0.14193548
1651	4.00000000	2.31250000	-0.06725318
1652	3.93750000	2.18750000	-0.13352884
1653	3.93750000	2.25000000	-0.11730205
1654	3.81250000	1.81250000	-0.19491691
1655	3.68750000	1.68750000	-0.16911046
1656	3.81250000	1.75000000	-0.19257087
1657	3.75000000	1.81250000	-0.18729228
1658	3.75000000	1.68750000	-0.19159335
1659	3.68750000	1.75000000	-0.18651026
1660	3.81250000	2.00000000	-0.17204301
1661	3.68750000	2.00000000	-0.17282502
1662	3.81250000	2.06250000	-0.15327468
1663	3.75000000	1.93750000	-0.17126100
1664	3.75000000	2.06250000	-0.14662757
1665	3.68750000	1.93750000	-0.16168133
1666	0.00000000	0.81250000	-0.13724340
1667	0.00000000	0.68750000	-0.09755621
1668	0.06250000	0.81250000	-0.13665689
1669	0.06250000	0.75000000	-0.11319648
1670	0.00000000	1.18750000	-0.19628543
1671	0.00000000	1.31250000	-0.19569892
1672	0.06250000	1.25000000	-0.19608993
1673	0.06250000	1.31250000	-0.18944282
1674	0.18750000	1.00000000	-0.19452590
1675	0.31250000	1.00000000	-0.19530792
1676	0.18750000	0.93750000	-0.17478006
1677	0.25000000	1.06250000	-0.19452590
1678	0.25000000	0.93750000	-0.17047898
1679	0.31250000	1.06250000	-0.19354839
1680	0.18750000	1.18750000	-0.19667644
1681	0.31250000	1.31250000	-0.19296188
1682	0.18750000	1.25000000	-0.19628543
1683	0.25000000	1.18750000	-0.19589443
1684	0.25000000	1.31250000	-0.18963832
1685	0.31250000	1.25000000	-0.19569892
1686	0.81250000	0.00000000	0.12062561
1687	0.68750000	0.00000000	0.13255132
1688	0.81250000	0.06250000	0.08563050
1689	0.75000000	0.06250000	0.09677419
1690	1.18750000	0.00000000	0.06060606
1691	1.31250000	0.00000000	0.07429130
1692	1.25000000	0.06250000	0.10576735
1693	1.31250000	0.06250000	0.06080156
1694	1.00000000	0.18750000	-0.00097752
1695	1.00000000	0.31250000	-0.03675464
1696	0.93750000	0.18750000	0.02326491
1697	1.06250000	0.25000000	0.01896383
1698	0.93750000	0.25000000	0.00332356
1699	1.06250000	0.31250000	-0.01290323
1700	1.18750000	0.18750000	0.10048876
1701	1.31250000	0.31250000	0.09442815
1702	1.25000000	0.18750000	0.13235582
1703	1.18750000	0.25000000	0.06999022
1704	1.31250000	0.25000000	0.10068426
1705	1.25000000	0.31250000	0.06353861
1706	0.81250000	0.81250000	-0.13704790
1707	0.68750000	0.68750000	-0.09794721
1708	0.75000000	0.81250000	-0.13724340

1709	0.81250000	0.75000000	-0.12844575
1710	0.68750000	0.75000000	-0.11671554
1711	0.75000000	0.68750000	-0.10733138
1712	1.18750000	1.18750000	-0.14858260
1713	1.31250000	1.31250000	-0.12942326
1714	1.18750000	1.25000000	-0.14799609
1715	1.25000000	1.18750000	-0.13704790
1716	1.25000000	1.31250000	-0.13313783
1717	1.31250000	1.25000000	-0.12140762
1718	0.81250000	1.00000000	-0.19589443
1719	0.68750000	1.00000000	-0.19569892
1720	0.75000000	0.93750000	-0.17673509
1721	0.81250000	1.06250000	-0.19726295
1722	0.68750000	0.93750000	-0.17595308
1723	0.75000000	1.06250000	-0.19569892
1724	1.00000000	0.81250000	-0.17028348
1725	1.00000000	0.68750000	-0.15327468
1726	0.93750000	0.75000000	-0.15053763
1727	1.06250000	0.81250000	-0.15874878
1728	0.93750000	0.68750000	-0.14056696
1729	1.06250000	0.75000000	-0.15034213
1730	1.00000000	1.18750000	-0.19980450
1731	1.00000000	1.31250000	-0.19745846
1732	1.06250000	1.25000000	-0.18162268
1733	0.93750000	1.18750000	-0.19902248
1734	1.06250000	1.31250000	-0.18435973
1735	0.93750000	1.25000000	-0.19824047
1736	1.18750000	1.00000000	-0.16168133
1737	1.31250000	1.00000000	-0.12649071
1738	1.25000000	1.06250000	-0.14584555
1739	1.18750000	0.93750000	-0.15307918
1740	1.31250000	1.06250000	-0.13450635
1741	1.25000000	0.93750000	-0.14193548
1742	0.00000000	3.18750000	0.10987292
1743	0.00000000	3.31250000	0.15092864
1744	0.06250000	3.25000000	0.13704790
1745	0.06250000	3.31250000	0.15738025
1746	0.00000000	2.81250000	0.06549365
1747	0.00000000	2.68750000	-0.00645161
1748	0.06250000	2.81250000	0.06744868
1749	0.06250000	2.75000000	-0.01876833
1750	0.18750000	3.18750000	0.10478983
1751	0.31250000	3.31250000	0.10889541
1752	0.18750000	3.25000000	0.10029326
1753	0.25000000	3.18750000	0.10420332
1754	0.25000000	3.31250000	0.10224829
1755	0.31250000	3.25000000	0.10850440
1756	0.18750000	3.00000000	0.17927664
1757	0.31250000	3.00000000	0.15640274
1758	0.18750000	2.93750000	0.14310850
1759	0.25000000	3.06250000	0.15366569
1760	0.25000000	2.93750000	0.09462366
1761	0.31250000	3.06250000	0.18318671
1762	0.81250000	4.00000000	0.06666667
1763	0.68750000	4.00000000	0.05650049
1764	0.75000000	3.93750000	0.07996090
1765	0.68750000	3.93750000	0.02502444
1766	1.18750000	4.00000000	0.11573803
1767	1.31250000	4.00000000	0.10107527

1768	1.18750000	3.93750000	0.13450635
1769	1.25000000	3.93750000	0.14173998
1770	0.81250000	3.81250000	0.12179863
1771	0.68750000	3.68750000	0.14897361
1772	0.75000000	3.81250000	0.11671554
1773	0.81250000	3.75000000	0.13861193
1774	0.68750000	3.75000000	0.08035191
1775	0.75000000	3.68750000	0.14584555
1776	1.00000000	3.81250000	0.13196481
1777	1.00000000	3.68750000	0.15307918
1778	1.06250000	3.81250000	0.14408602
1779	0.93750000	3.75000000	0.14545455
1780	1.06250000	3.75000000	0.15620723
1781	0.93750000	3.68750000	0.15620723
1782	3.18750000	0.00000000	0.12727273
1783	3.31250000	0.00000000	0.15014663
1784	3.25000000	0.06250000	0.12766373
1785	3.31250000	0.06250000	0.12043011
1786	2.81250000	0.00000000	0.12825024
1787	2.68750000	0.00000000	0.15249267
1788	2.81250000	0.06250000	0.11749756
1789	2.75000000	0.06250000	0.13020528
1790	3.18750000	0.18750000	0.06314761
1791	3.31250000	0.31250000	0.06999022
1792	3.25000000	0.18750000	0.10576735
1793	3.18750000	0.25000000	0.08328446
1794	3.31250000	0.25000000	0.08739003
1795	3.25000000	0.31250000	0.07116325
1796	3.00000000	0.18750000	0.05943304
1797	3.00000000	0.31250000	0.03675464
1798	2.93750000	0.18750000	0.07135875
1799	3.06250000	0.25000000	0.06001955
1800	2.93750000	0.25000000	0.06099707
1801	3.06250000	0.31250000	0.03636364
1802	4.00000000	0.81250000	-0.19042033
1803	4.00000000	0.68750000	-0.09970674
1804	3.93750000	0.75000000	-0.16774194
1805	3.93750000	0.68750000	-0.16793744
1806	4.00000000	1.18750000	-0.19863148
1807	4.00000000	1.31250000	-0.06588465
1808	3.93750000	1.18750000	-0.19921799
1809	3.93750000	1.25000000	-0.18944282
1810	3.81250000	0.81250000	-0.12961877
1811	3.68750000	0.68750000	-0.08093842
1812	3.81250000	0.75000000	-0.12727273
1813	3.75000000	0.81250000	-0.13020528
1814	3.75000000	0.68750000	-0.10733138
1815	3.68750000	0.75000000	-0.09912023
1816	3.81250000	1.00000000	-0.19960899
1817	3.68750000	1.00000000	-0.18592375
1818	3.81250000	1.06250000	-0.19980450
1819	3.75000000	0.93750000	-0.17673509
1820	3.75000000	1.06250000	-0.19042033
1821	3.68750000	0.93750000	-0.16891496
1822	3.18750000	3.18750000	0.12453568
1823	3.31250000	3.31250000	0.15268817
1824	3.18750000	3.25000000	0.14525904
1825	3.25000000	3.18750000	0.14154448
1826	3.25000000	3.31250000	0.15151515

1827	3.31250000	3.25000000	0.16578690
1828	2.81250000	2.81250000	0.04672532
1829	2.68750000	2.68750000	0.04946237
1830	2.75000000	2.81250000	0.06353861
1831	2.81250000	2.75000000	0.02971652
1832	2.68750000	2.75000000	0.05865103
1833	2.75000000	2.68750000	0.03734115
1834	3.00000000	3.18750000	0.12277615
1835	3.00000000	3.31250000	0.15131965
1836	3.06250000	3.25000000	0.14193548
1837	2.93750000	3.18750000	0.13939394
1838	3.06250000	3.31250000	0.14956012
1839	2.93750000	3.25000000	0.14760508
1840	3.18750000	3.00000000	0.10224829
1841	3.31250000	3.00000000	0.10987292
1842	3.25000000	3.06250000	0.12453568
1843	3.18750000	2.93750000	0.08504399
1844	3.31250000	3.06250000	0.12140762
1845	3.25000000	2.93750000	0.09579668
1846	2.81250000	3.00000000	0.12981427
1847	2.68750000	3.00000000	0.15092864
1848	2.75000000	2.93750000	0.11886608
1849	2.81250000	3.06250000	0.14408602
1850	2.68750000	2.93750000	0.14173998
1851	2.75000000	3.06250000	0.15464321
1852	3.00000000	2.81250000	0.02834800
1853	3.00000000	2.68750000	-0.02463343
1854	2.93750000	2.75000000	0.01739980
1855	3.06250000	2.81250000	0.03577713
1856	2.93750000	2.68750000	0.01876833
1857	3.06250000	2.75000000	0.02189638
1858	3.18750000	4.00000000	-0.06373412
1859	3.31250000	4.00000000	-0.05161290
1860	3.18750000	3.93750000	-0.02463343
1861	3.25000000	3.93750000	-0.02013685
1862	2.81250000	4.00000000	-0.07155425
1863	2.68750000	4.00000000	0.04242424
1864	2.75000000	3.93750000	-0.00156403
1865	2.68750000	3.93750000	0.03069404
1866	3.00000000	3.81250000	-0.00097752
1867	3.00000000	3.68750000	0.10224829
1868	3.06250000	3.81250000	0.01720430
1869	2.93750000	3.75000000	0.01896383
1870	3.06250000	3.75000000	0.04261975
1871	2.93750000	3.68750000	0.05786901
1872	2.81250000	3.81250000	-0.05865103
1873	2.68750000	3.68750000	-0.01935484
1874	2.75000000	3.81250000	-0.02639296
1875	2.81250000	3.75000000	-0.01955034
1876	2.68750000	3.75000000	-0.00664712
1877	2.75000000	3.68750000	0.00019550
1878	4.00000000	3.18750000	0.09071359
1879	4.00000000	3.31250000	0.11769306
1880	3.93750000	3.18750000	0.09560117
1881	3.93750000	3.25000000	0.11534702
1882	4.00000000	2.81250000	0.01524927
1883	4.00000000	2.68750000	-0.02189638
1884	3.93750000	2.75000000	0.05650049
1885	3.93750000	2.68750000	-0.06862170

1886	3.81250000	3.00000000	0.02756598
1887	3.68750000	3.00000000	0.05474096
1888	3.81250000	3.06250000	0.05356794
1889	3.75000000	2.93750000	-0.03147605
1890	3.75000000	3.06250000	0.10244379
1891	3.68750000	2.93750000	0.05493646
1892	3.81250000	2.81250000	-0.02678397
1893	3.68750000	2.68750000	0.00527859
1894	3.81250000	2.75000000	-0.03831867
1895	3.75000000	2.81250000	-0.13450635
1896	3.75000000	2.68750000	-0.16070381
1897	3.68750000	2.75000000	-0.17458456
1898	0.81250000	2.00000000	-0.02991202
1899	0.68750000	2.00000000	-0.01681329
1900	0.75000000	1.93750000	-0.03010753
1901	0.81250000	2.06250000	-0.00840665
1902	0.68750000	1.93750000	-0.02346041
1903	0.75000000	2.06250000	0.05474096
1904	1.18750000	2.00000000	-0.14819159
1905	1.31250000	2.00000000	-0.11652004
1906	1.18750000	1.93750000	-0.14838710
1907	1.25000000	2.06250000	-0.12316716
1908	1.25000000	1.93750000	-0.13235582
1909	1.31250000	2.06250000	-0.13216031
1910	0.81250000	1.81250000	-0.19608993
1911	0.68750000	1.68750000	-0.08973607
1912	0.75000000	1.81250000	-0.14076246
1913	0.81250000	1.75000000	-0.19608993
1914	0.68750000	1.75000000	-0.18944282
1915	0.75000000	1.68750000	-0.19608993
1916	1.00000000	1.81250000	-0.19608993
1917	1.00000000	1.68750000	-0.19608993
1918	1.06250000	1.81250000	-0.18025415
1919	0.93750000	1.75000000	-0.19608993
1920	1.06250000	1.75000000	-0.18025415
1921	0.93750000	1.68750000	-0.19608993
1922	1.00000000	2.18750000	-0.13079179
1923	1.00000000	2.31250000	-0.09716520
1924	0.93750000	2.18750000	-0.07565982
1925	1.06250000	2.25000000	-0.10576735
1926	0.93750000	2.25000000	-0.04613881
1927	1.06250000	2.31250000	-0.08406647
1928	1.18750000	2.18750000	-0.12023460
1929	1.31250000	2.31250000	-0.06979472
1930	1.25000000	2.18750000	-0.10459433
1931	1.18750000	2.25000000	-0.09872923
1932	1.31250000	2.25000000	-0.10420332
1933	1.25000000	2.31250000	-0.07350929
1934	0.81250000	2.81250000	0.04613881
1935	0.68750000	2.68750000	-0.02228739
1936	0.75000000	2.81250000	0.05083089
1937	0.81250000	2.75000000	-0.02521994
1938	0.68750000	2.75000000	0.01661779
1939	0.75000000	2.68750000	-0.00957967
1940	1.18750000	3.18750000	0.16852395
1941	1.31250000	3.31250000	0.12825024
1942	1.18750000	3.25000000	0.17067449
1943	1.25000000	3.18750000	0.17086999
1944	1.25000000	3.31250000	0.16383187

1945	1.31250000	3.25000000	0.17732160
1946	0.81250000	3.00000000	0.16324536
1947	0.68750000	3.00000000	0.17243402
1948	0.75000000	2.93750000	0.12883675
1949	0.81250000	3.06250000	0.16539589
1950	0.68750000	2.93750000	0.13372434
1951	0.75000000	3.06250000	0.17008798
1952	1.00000000	3.18750000	0.15855327
1953	1.00000000	3.31250000	0.15953079
1954	1.06250000	3.25000000	0.16187683
1955	0.93750000	3.18750000	0.16011730
1956	1.06250000	3.31250000	0.15053763
1957	0.93750000	3.25000000	0.16207234
1958	1.00000000	2.81250000	-0.06490714
1959	1.00000000	2.68750000	-0.11730205
1960	0.93750000	2.75000000	-0.09931574
1961	1.06250000	2.81250000	-0.06803519
1962	0.93750000	2.68750000	-0.12082111
1963	1.06250000	2.75000000	-0.08328446
1964	1.18750000	3.00000000	0.15874878
1965	1.31250000	3.00000000	0.08543500
1966	1.25000000	3.06250000	0.16402737
1967	1.18750000	2.93750000	0.08074291
1968	1.31250000	3.06250000	0.16774194
1969	1.25000000	2.93750000	0.13978495
1970	2.00000000	0.81250000	0.06236559
1971	2.00000000	0.68750000	0.11006843
1972	1.93750000	0.75000000	0.06803519
1973	2.06250000	0.81250000	0.05122190
1974	1.93750000	0.68750000	0.07487781
1975	2.06250000	0.75000000	0.08621701
1976	2.00000000	1.18750000	0.00430108
1977	2.00000000	1.31250000	0.01251222
1978	1.93750000	1.18750000	0.04144673
1979	2.06250000	1.25000000	0.04261975
1980	1.93750000	1.25000000	-0.00371457
1981	2.06250000	1.31250000	-0.02834800
1982	1.81250000	0.81250000	0.01876833
1983	1.68750000	0.68750000	0.03655914
1984	1.81250000	0.75000000	0.04105572
1985	1.75000000	0.81250000	0.05571848
1986	1.75000000	0.68750000	0.05004888
1987	1.68750000	0.75000000	0.01544477
1988	1.81250000	1.00000000	0.10342131
1989	1.68750000	1.00000000	0.12922776
1990	1.81250000	1.06250000	0.10752688
1991	1.75000000	0.93750000	0.11221896
1992	1.75000000	1.06250000	0.09540567
1993	1.68750000	0.93750000	0.10029326
1994	2.18750000	1.00000000	-0.04144673
1995	2.31250000	1.00000000	-0.06373412
1996	2.18750000	0.93750000	-0.01583578
1997	2.25000000	1.06250000	-0.00293255
1998	2.25000000	0.93750000	-0.01446725
1999	2.31250000	1.06250000	-0.06412512
2000	2.18750000	1.18750000	0.10733138
2001	2.31250000	1.31250000	0.07781036
2002	2.18750000	1.25000000	0.11124145
2003	2.25000000	1.18750000	0.09618768

2004	2.25000000	1.31250000	0.07468231
2005	2.31250000	1.25000000	0.08445748
2006	2.81250000	0.81250000	-0.12023460
2007	2.68750000	0.68750000	-0.07057674
2008	2.81250000	0.75000000	-0.09638319
2009	2.75000000	0.81250000	-0.05923754
2010	2.75000000	0.68750000	-0.12082111
2011	2.68750000	0.75000000	-0.08328446
2012	3.18750000	1.18750000	-0.14623656
2013	3.31250000	1.31250000	-0.15992180
2014	3.25000000	1.18750000	-0.18924731
2015	3.18750000	1.25000000	-0.19511241
2016	3.31250000	1.25000000	-0.18905181
2017	3.25000000	1.31250000	-0.19433040
2018	3.00000000	0.81250000	-0.12277615
2019	3.00000000	0.68750000	-0.07233627
2020	2.93750000	0.75000000	-0.09716520
2021	3.06250000	0.81250000	-0.11593353
2022	2.93750000	0.68750000	-0.12199413
2023	3.06250000	0.75000000	-0.09149560
2024	3.18750000	1.00000000	-0.17888563
2025	3.31250000	1.00000000	-0.07800587
2026	3.25000000	1.06250000	-0.17712610
2027	3.18750000	0.93750000	-0.15268817
2028	3.31250000	1.06250000	-0.17086999
2029	3.25000000	0.93750000	-0.14643206
2030	2.81250000	1.00000000	-0.01173021
2031	2.68750000	1.00000000	0.01368524
2032	2.75000000	0.93750000	0.01407625
2033	2.81250000	1.06250000	-0.01055718
2034	2.68750000	0.93750000	0.02678397
2035	2.75000000	1.06250000	0.00215054
2036	3.00000000	1.18750000	-0.19569892
2037	3.00000000	1.31250000	-0.19335288
2038	3.06250000	1.25000000	-0.19472141
2039	2.93750000	1.18750000	-0.13313783
2040	3.06250000	1.31250000	-0.19374389
2041	2.93750000	1.25000000	-0.18181818
2042	2.00000000	2.81250000	0.17086999
2043	2.00000000	2.68750000	0.15640274
2044	2.06250000	2.81250000	0.17282502
2045	1.93750000	2.75000000	0.16265885
2046	2.06250000	2.75000000	0.17380254
2047	1.93750000	2.68750000	0.16715543
2048	2.00000000	3.18750000	0.06666667
2049	2.00000000	3.31250000	-0.00840665
2050	2.06250000	3.25000000	0.03440860
2051	1.93750000	3.18750000	0.06373412
2052	2.06250000	3.31250000	-0.00000000
2053	1.93750000	3.25000000	0.02150538
2054	2.18750000	3.00000000	0.19139785
2055	2.31250000	3.00000000	0.18592375
2056	2.18750000	2.93750000	0.18651026
2057	2.25000000	3.06250000	0.16539589
2058	2.25000000	2.93750000	0.19433040
2059	2.31250000	3.06250000	0.17047898
2060	2.18750000	3.18750000	0.10127077
2061	2.31250000	3.31250000	0.06158358
2062	2.18750000	3.25000000	0.05747801

2063	2.25000000	3.18750000	0.10322581
2064	2.25000000	3.31250000	0.03460411
2065	2.31250000	3.25000000	0.07741935
2066	1.81250000	2.81250000	0.15738025
2067	1.68750000	2.68750000	0.11573803
2068	1.81250000	2.75000000	0.15581623
2069	1.75000000	2.81250000	0.16070381
2070	1.75000000	2.68750000	0.15679374
2071	1.68750000	2.75000000	0.13685239
2072	1.81250000	3.00000000	0.18905181
2073	1.68750000	3.00000000	0.10400782
2074	1.81250000	3.06250000	0.14525904
2075	1.75000000	2.93750000	0.17751711
2076	1.75000000	3.06250000	0.14369501
2077	1.68750000	2.93750000	0.16207234
2078	2.81250000	2.00000000	-0.18690127
2079	2.68750000	2.00000000	-0.15777126
2080	2.81250000	2.06250000	-0.17908113
2081	2.75000000	1.93750000	-0.17517107
2082	2.75000000	2.06250000	-0.15171065
2083	2.68750000	1.93750000	-0.18533724
2084	3.18750000	2.00000000	-0.17047898
2085	3.31250000	2.00000000	-0.10459433
2086	3.25000000	2.06250000	-0.15190616
2087	3.18750000	1.93750000	-0.17028348
2088	3.31250000	2.06250000	-0.14095797
2089	3.25000000	1.93750000	-0.16109482
2090	3.00000000	2.18750000	-0.17419355
2091	3.00000000	2.31250000	-0.15874878
2092	2.93750000	2.18750000	-0.17165200
2093	3.06250000	2.25000000	-0.15562072
2094	2.93750000	2.25000000	-0.13900293
2095	3.06250000	2.31250000	-0.13216031
2096	3.18750000	2.18750000	-0.13861193
2097	3.31250000	2.31250000	-0.04555230
2098	3.25000000	2.18750000	-0.13079179
2099	3.18750000	2.25000000	-0.13098729
2100	3.31250000	2.25000000	-0.10928641
2101	3.25000000	2.31250000	-0.09677419
2102	2.81250000	1.81250000	-0.15933529
2103	2.68750000	1.68750000	-0.14213099
2104	2.75000000	1.81250000	-0.15679374
2105	2.81250000	1.75000000	-0.15913978
2106	2.68750000	1.75000000	-0.15757576
2107	2.75000000	1.68750000	-0.14701857
2108	3.00000000	1.81250000	-0.19433040
2109	3.00000000	1.68750000	-0.19296188
2110	3.06250000	1.81250000	-0.18592375
2111	2.93750000	1.75000000	-0.18220919
2112	3.06250000	1.75000000	-0.18455523
2113	2.93750000	1.68750000	-0.18162268
2114	0.18750000	0.50000000	-0.11339198
2115	0.31250000	0.50000000	-0.11378299
2116	0.18750000	0.43750000	-0.10009775
2117	0.25000000	0.43750000	-0.10068426
2118	0.25000000	0.56250000	-0.12903226
2119	0.31250000	0.56250000	-0.13255132
2120	0.18750000	0.68750000	-0.08484848
2121	0.31250000	0.81250000	-0.12414467

2122	0.18750000	0.75000000	-0.10478983
2123	0.25000000	0.81250000	-0.12394917
2124	0.25000000	0.68750000	-0.11006843
2125	0.31250000	0.75000000	-0.10400782
2126	0.50000000	0.18750000	0.09853372
2127	0.50000000	0.31250000	0.04340176
2128	0.43750000	0.18750000	0.08406647
2129	0.43750000	0.25000000	0.06256109
2130	0.56250000	0.25000000	0.06138807
2131	0.56250000	0.31250000	0.03382209
2132	0.68750000	0.18750000	0.06979472
2133	0.81250000	0.31250000	-0.00586510
2134	0.75000000	0.18750000	0.05395894
2135	0.81250000	0.25000000	0.02287390
2136	0.68750000	0.25000000	0.04222874
2137	0.75000000	0.31250000	0.00527859
2138	0.50000000	0.68750000	-0.08973607
2139	0.50000000	0.81250000	-0.07781036
2140	0.56250000	0.75000000	-0.10889541
2141	0.56250000	0.81250000	-0.12864125
2142	0.43750000	0.68750000	-0.11417400
2143	0.43750000	0.75000000	-0.10459433
2144	0.68750000	0.50000000	-0.08015640
2145	0.81250000	0.50000000	-0.08993157
2146	0.75000000	0.56250000	-0.08719453
2147	0.81250000	0.56250000	-0.09833822
2148	0.68750000	0.43750000	-0.04027370
2149	0.75000000	0.43750000	-0.04965787
2150	0.18750000	3.68750000	0.10381232
2151	0.31250000	3.81250000	0.08289345
2152	0.18750000	3.75000000	0.09931574
2153	0.25000000	3.81250000	0.08719453
2154	0.25000000	3.68750000	0.11026393
2155	0.31250000	3.75000000	0.04828935
2156	0.18750000	3.50000000	0.15679374
2157	0.31250000	3.50000000	0.17008798
2158	0.18750000	3.43750000	0.16129032
2159	0.25000000	3.43750000	0.14291300
2160	0.25000000	3.56250000	0.14565005
2161	0.31250000	3.56250000	0.10146628
2162	0.50000000	3.81250000	-0.10205279
2163	0.50000000	3.68750000	-0.11573803
2164	0.56250000	3.81250000	-0.04789834
2165	0.56250000	3.75000000	-0.02932551
2166	0.43750000	3.75000000	-0.04007820
2167	0.43750000	3.68750000	-0.02228739
2168	3.68750000	0.18750000	-0.03460411
2169	3.81250000	0.31250000	-0.11534702
2170	3.75000000	0.18750000	-0.09247312
2171	3.81250000	0.25000000	-0.11984360
2172	3.68750000	0.25000000	-0.11515152
2173	3.75000000	0.31250000	-0.15874878
2174	3.50000000	0.18750000	0.09521017
2175	3.50000000	0.31250000	0.00097752
2176	3.43750000	0.18750000	0.08387097
2177	3.43750000	0.25000000	0.07272727
2178	3.56250000	0.25000000	0.00527859
2179	3.56250000	0.31250000	0.02209189
2180	3.81250000	0.50000000	-0.04046921

2181	3.68750000	0.50000000	-0.08250244
2182	3.81250000	0.56250000	-0.12825024
2183	3.75000000	0.56250000	-0.10811339
2184	3.75000000	0.43750000	-0.12531769
2185	3.68750000	0.43750000	-0.06490714
2186	3.50000000	3.68750000	0.08504399
2187	3.50000000	3.81250000	0.00997067
2188	3.56250000	3.75000000	0.07781036
2189	3.56250000	3.81250000	0.04652981
2190	3.43750000	3.68750000	0.03675464
2191	3.43750000	3.75000000	0.05395894
2192	3.68750000	3.50000000	0.16969697
2193	3.81250000	3.50000000	0.16441838
2194	3.75000000	3.56250000	0.16246334
2195	3.81250000	3.56250000	0.15972630
2196	3.68750000	3.43750000	0.16402737
2197	3.75000000	3.43750000	0.16207234
2198	3.31250000	3.50000000	0.19159335
2199	3.18750000	3.50000000	0.19784946
2200	3.25000000	3.43750000	0.17947214
2201	3.18750000	3.43750000	0.18084066
2202	3.31250000	3.56250000	0.10987292
2203	3.25000000	3.56250000	0.16050831
2204	3.50000000	3.31250000	0.16246334
2205	3.50000000	3.18750000	0.15249267
2206	3.43750000	3.25000000	0.17223851
2207	3.43750000	3.18750000	0.14486804
2208	3.56250000	3.31250000	0.15913978
2209	3.56250000	3.25000000	0.15483871
2210	3.31250000	3.81250000	0.02267840
2211	3.18750000	3.68750000	-0.01368524
2212	3.25000000	3.81250000	0.02893451
2213	3.18750000	3.75000000	0.04985337
2214	3.31250000	3.75000000	0.05356794
2215	3.25000000	3.68750000	0.08699902
2216	3.81250000	3.31250000	0.15131965
2217	3.68750000	3.18750000	0.17223851
2218	3.81250000	3.25000000	0.13782991
2219	3.75000000	3.18750000	0.16695992
2220	3.75000000	3.31250000	0.15347019
2221	3.68750000	3.25000000	0.15112414
2222	0.18750000	1.68750000	-0.19374389
2223	0.31250000	1.81250000	-0.18084066
2224	0.18750000	1.75000000	-0.19433040
2225	0.25000000	1.81250000	-0.18768328
2226	0.25000000	1.68750000	-0.18729228
2227	0.31250000	1.75000000	-0.18748778
2228	0.18750000	1.50000000	-0.17575758
2229	0.31250000	1.50000000	-0.18240469
2230	0.18750000	1.43750000	-0.17614858
2231	0.25000000	1.43750000	-0.17614858
2232	0.25000000	1.56250000	-0.17536657
2233	0.31250000	1.56250000	-0.16950147
2234	0.18750000	2.50000000	-0.11339198
2235	0.31250000	2.50000000	-0.11769306
2236	0.18750000	2.43750000	-0.07390029
2237	0.25000000	2.43750000	-0.12531769
2238	0.25000000	2.56250000	-0.12570870
2239	0.31250000	2.56250000	-0.08015640

2240	0.18750000	2.68750000	-0.15171065
2241	0.31250000	2.81250000	-0.07644184
2242	0.18750000	2.75000000	-0.11554252
2243	0.25000000	2.81250000	-0.07781036
2244	0.25000000	2.68750000	-0.15131965
2245	0.31250000	2.75000000	-0.16539589
2246	0.50000000	2.18750000	-0.13587488
2247	0.50000000	2.31250000	-0.13137830
2248	0.43750000	2.18750000	-0.14291300
2249	0.43750000	2.25000000	-0.14076246
2250	0.56250000	2.25000000	-0.06432063
2251	0.56250000	2.31250000	-0.11867058
2252	0.50000000	1.81250000	-0.16324536
2253	0.50000000	1.68750000	-0.17653959
2254	0.56250000	1.81250000	-0.12101662
2255	0.56250000	1.75000000	-0.17634409
2256	0.43750000	1.75000000	-0.17575758
2257	0.43750000	1.68750000	-0.16969697
2258	0.68750000	2.18750000	0.07057674
2259	0.81250000	2.31250000	0.09130010
2260	0.75000000	2.18750000	0.11280547
2261	0.81250000	2.25000000	0.07917889
2262	0.68750000	2.25000000	0.07311828
2263	0.75000000	2.31250000	0.13802542
2264	0.50000000	2.68750000	-0.00821114
2265	0.50000000	2.81250000	0.06647116
2266	0.56250000	2.75000000	0.02600196
2267	0.56250000	2.81250000	0.06510264
2268	0.43750000	2.68750000	-0.00840665
2269	0.43750000	2.75000000	-0.06803519
2270	0.68750000	2.50000000	-0.08680352
2271	0.81250000	2.50000000	-0.07077224
2272	0.75000000	2.56250000	-0.05259042
2273	0.81250000	2.56250000	-0.08973607
2274	0.68750000	2.43750000	-0.08895406
2275	0.75000000	2.43750000	0.03030303
2276	1.68750000	0.18750000	0.17947214
2277	1.81250000	0.31250000	0.18611926
2278	1.75000000	0.18750000	0.18318671
2279	1.81250000	0.25000000	0.18631476
2280	1.68750000	0.25000000	0.16617791
2281	1.75000000	0.31250000	0.16637341
2282	1.50000000	0.18750000	0.12922776
2283	1.50000000	0.31250000	0.10283480
2284	1.43750000	0.18750000	0.09755621
2285	1.43750000	0.25000000	0.11085044
2286	1.56250000	0.25000000	0.13274682
2287	1.56250000	0.31250000	0.11632454
2288	2.50000000	0.18750000	0.12238514
2289	2.50000000	0.31250000	0.07683284
2290	2.43750000	0.18750000	0.12453568
2291	2.43750000	0.25000000	0.10713587
2292	2.56250000	0.25000000	0.09990225
2293	2.56250000	0.31250000	0.02639296
2294	2.68750000	0.18750000	0.12140762
2295	2.81250000	0.31250000	0.08308895
2296	2.75000000	0.18750000	0.10948192
2297	2.81250000	0.25000000	0.08641251
2298	2.68750000	0.25000000	0.09931574

2299	2.75000000	0.31250000	0.02561095
2300	2.18750000	0.50000000	0.12707722
2301	2.31250000	0.50000000	0.07976540
2302	2.18750000	0.43750000	0.12746823
2303	2.25000000	0.43750000	0.11006843
2304	2.25000000	0.56250000	0.10322581
2305	2.31250000	0.56250000	0.06686217
2306	1.81250000	0.50000000	0.13685239
2307	1.68750000	0.50000000	0.00351906
2308	1.81250000	0.56250000	0.09266862
2309	1.75000000	0.56250000	0.09481916
2310	1.75000000	0.43750000	0.13352884
2311	1.68750000	0.43750000	0.11691105
2312	2.18750000	0.68750000	0.12394917
2313	2.31250000	0.81250000	0.05063539
2314	2.18750000	0.75000000	0.09540567
2315	2.25000000	0.81250000	0.06177908
2316	2.25000000	0.68750000	0.10107527
2317	2.31250000	0.75000000	0.06373412
2318	2.68750000	0.50000000	-0.14486804
2319	2.81250000	0.50000000	-0.14565005
2320	2.75000000	0.56250000	-0.17047898
2321	2.81250000	0.56250000	-0.17086999
2322	2.68750000	0.43750000	-0.12140762
2323	2.75000000	0.43750000	-0.12160313
2324	2.50000000	0.68750000	-0.03225806
2325	2.50000000	0.81250000	-0.05806452
2326	2.56250000	0.75000000	-0.05786901
2327	2.56250000	0.81250000	-0.02111437
2328	2.43750000	0.68750000	-0.00782014
2329	2.43750000	0.75000000	-0.00879765
2330	1.50000000	1.68750000	-0.06940371
2331	1.50000000	1.81250000	-0.06920821
2332	1.56250000	1.75000000	-0.05356794
2333	1.56250000	1.81250000	-0.05356794
2334	1.43750000	1.68750000	-0.08563050
2335	1.43750000	1.75000000	-0.08523949
2336	1.68750000	1.50000000	-0.03421310
2337	1.81250000	1.50000000	-0.01055718
2338	1.75000000	1.56250000	-0.01837732
2339	1.81250000	1.56250000	-0.00664712
2340	1.68750000	1.43750000	-0.03870968
2341	1.75000000	1.43750000	-0.02697947
2342	1.31250000	1.50000000	-0.11827957
2343	1.18750000	1.50000000	-0.14975562
2344	1.25000000	1.43750000	-0.13391984
2345	1.18750000	1.43750000	-0.15151515
2346	1.31250000	1.56250000	-0.11808407
2347	1.25000000	1.56250000	-0.13391984
2348	1.50000000	1.31250000	-0.08289345
2349	1.50000000	1.18750000	-0.09188661
2350	1.43750000	1.25000000	-0.09872923
2351	1.43750000	1.18750000	-0.10303030
2352	1.56250000	1.31250000	-0.07116325
2353	1.56250000	1.25000000	-0.07546432
2354	2.31250000	2.50000000	0.10694037
2355	2.18750000	2.50000000	0.11788856
2356	2.25000000	2.43750000	0.10283480
2357	2.18750000	2.43750000	0.10830890

2358	2.31250000	2.56250000	0.11612903
2359	2.25000000	2.56250000	0.13352884
2360	2.50000000	2.31250000	0.02306940
2361	2.50000000	2.18750000	-0.01212121
2362	2.43750000	2.25000000	0.02521994
2363	2.43750000	2.18750000	0.00449658
2364	2.56250000	2.31250000	-0.08308895
2365	2.56250000	2.25000000	-0.00684262
2366	2.50000000	2.68750000	0.11808407
2367	2.50000000	2.81250000	0.13646139
2368	2.56250000	2.75000000	0.10439883
2369	2.56250000	2.81250000	0.13216031
2370	2.43750000	2.68750000	0.12355816
2371	2.43750000	2.75000000	0.14467253
2372	2.68750000	2.50000000	0.05395894
2373	2.81250000	2.50000000	-0.00175953
2374	2.75000000	2.56250000	0.04027370
2375	2.81250000	2.56250000	0.03401760
2376	2.68750000	2.43750000	-0.06666667
2377	2.75000000	2.43750000	0.01779081
2378	1.68750000	2.18750000	0.00723363
2379	1.81250000	2.31250000	0.05806452
2380	1.75000000	2.18750000	0.02306940
2381	1.81250000	2.25000000	0.04848485
2382	1.68750000	2.25000000	-0.00840665
2383	1.75000000	2.31250000	0.06705767
2384	1.31250000	1.81250000	-0.11652004
2385	1.18750000	1.68750000	-0.14760508
2386	1.25000000	1.81250000	-0.13235582
2387	1.18750000	1.75000000	-0.14838710
2388	1.31250000	1.75000000	-0.11671554
2389	1.25000000	1.68750000	-0.13294233
2390	1.50000000	2.18750000	-0.11593353
2391	1.50000000	2.31250000	-0.09696970
2392	1.43750000	2.18750000	-0.13176931
2393	1.43750000	2.25000000	-0.12238514
2394	1.56250000	2.25000000	-0.09051808
2395	1.56250000	2.31250000	-0.05610948
2396	2.18750000	1.68750000	0.00019550
2397	2.31250000	1.81250000	0.06275660
2398	2.18750000	1.75000000	-0.00821114
2399	2.25000000	1.81250000	-0.02072336
2400	2.25000000	1.68750000	-0.05317693
2401	2.31250000	1.75000000	-0.06549365
2402	1.81250000	1.31250000	-0.02541544
2403	1.68750000	1.18750000	-0.05650049
2404	1.81250000	1.25000000	-0.02795699
2405	1.75000000	1.18750000	0.00508309
2406	1.75000000	1.31250000	-0.03577713
2407	1.68750000	1.25000000	-0.05180841
2408	2.18750000	1.50000000	0.00899316
2409	2.31250000	1.50000000	-0.12883675
2410	2.18750000	1.43750000	-0.09540567
2411	2.25000000	1.43750000	-0.06099707
2412	2.25000000	1.56250000	-0.10361681
2413	2.31250000	1.56250000	-0.14447703
2414	2.18750000	2.68750000	0.18279570
2415	2.31250000	2.81250000	0.18866080
2416	2.18750000	2.75000000	0.18924731

2417	2.25000000	2.81250000	0.19608993
2418	2.25000000	2.68750000	0.17575758
2419	2.31250000	2.75000000	0.17947214
2420	1.81250000	2.50000000	0.17693060
2421	1.68750000	2.50000000	0.12942326
2422	1.81250000	2.56250000	0.16930596
2423	1.75000000	2.56250000	0.16578690
2424	1.75000000	2.43750000	0.13587488
2425	1.68750000	2.43750000	0.09481916
2426	2.68750000	2.18750000	-0.06275660
2427	2.81250000	2.31250000	-0.07331378
2428	2.75000000	2.18750000	-0.08641251
2429	2.81250000	2.25000000	-0.08113392
2430	2.68750000	2.25000000	-0.03812317
2431	2.75000000	2.31250000	-0.02991202
2432	2.50000000	1.81250000	-0.11045943
2433	2.50000000	1.68750000	-0.17321603
2434	2.56250000	1.81250000	-0.18787879
2435	2.56250000	1.75000000	-0.17849462
2436	2.43750000	1.75000000	-0.14780059
2437	2.43750000	1.68750000	-0.17634409
2438	1.50000000	3.81250000	0.02346041
2439	1.50000000	3.68750000	0.02463343
2440	1.56250000	3.81250000	0.04946237
2441	1.56250000	3.75000000	0.05004888
2442	1.43750000	3.75000000	0.07057674
2443	1.43750000	3.68750000	0.05845552
2444	1.31250000	3.81250000	0.13098729
2445	1.18750000	3.68750000	0.17008798
2446	1.25000000	3.81250000	0.16578690
2447	1.18750000	3.75000000	0.17067449
2448	1.31250000	3.75000000	0.17536657
2449	1.25000000	3.68750000	0.16598240
2450	2.31250000	3.81250000	0.04281525
2451	2.18750000	3.68750000	-0.03323558
2452	2.25000000	3.81250000	0.00019550
2453	2.18750000	3.75000000	-0.02404692
2454	2.31250000	3.75000000	0.01798631
2455	2.25000000	3.68750000	-0.02170088
2456	2.50000000	3.81250000	0.11652004
2457	2.50000000	3.68750000	0.07741935
2458	2.56250000	3.81250000	0.07038123
2459	2.56250000	3.75000000	0.05786901
2460	2.43750000	3.75000000	0.06608016
2461	2.43750000	3.68750000	0.05043988
2462	1.68750000	3.50000000	-0.08563050
2463	1.81250000	3.50000000	-0.10987292
2464	1.75000000	3.56250000	-0.06041056
2465	1.81250000	3.56250000	-0.10400782
2466	1.68750000	3.43750000	-0.03831867
2467	1.75000000	3.43750000	-0.08015640
2468	1.31250000	3.50000000	0.09618768
2469	1.18750000	3.50000000	0.13900293
2470	1.25000000	3.43750000	0.14134897
2471	1.18750000	3.43750000	0.13685239
2472	1.31250000	3.56250000	0.13939394
2473	1.25000000	3.56250000	0.14213099
2474	1.50000000	3.31250000	0.11808407
2475	1.50000000	3.18750000	0.10869990

2476	1.43750000	3.25000000	0.18807429
2477	1.43750000	3.18750000	0.18533724
2478	1.56250000	3.31250000	0.14956012
2479	1.56250000	3.25000000	0.14858260
2480	2.18750000	3.50000000	-0.09560117
2481	2.31250000	3.50000000	-0.05395894
2482	2.18750000	3.43750000	-0.05806452
2483	2.25000000	3.43750000	-0.04652981
2484	2.25000000	3.56250000	-0.06529814
2485	2.31250000	3.56250000	-0.04125122
2486	1.81250000	3.31250000	-0.12531769
2487	1.68750000	3.18750000	-0.12277615
2488	1.81250000	3.25000000	-0.08152493
2489	1.75000000	3.18750000	-0.04086022
2490	1.75000000	3.31250000	-0.08211144
2491	1.68750000	3.25000000	-0.04125122
2492	3.81250000	1.50000000	-0.16813294
2493	3.68750000	1.50000000	-0.18475073
2494	3.81250000	1.56250000	-0.18299120
2495	3.75000000	1.56250000	-0.18514174
2496	3.75000000	1.43750000	-0.18924731
2497	3.68750000	1.43750000	-0.16656891
2498	3.81250000	1.31250000	-0.16285435
2499	3.68750000	1.18750000	-0.15816227
2500	3.81250000	1.25000000	-0.17067449
2501	3.75000000	1.18750000	-0.17106549
2502	3.75000000	1.31250000	-0.17888563
2503	3.68750000	1.25000000	-0.16344086
2504	3.81250000	2.31250000	-0.08660802
2505	3.68750000	2.18750000	-0.05708700
2506	3.81250000	2.25000000	-0.10537634
2507	3.75000000	2.18750000	-0.11495601
2508	3.75000000	2.31250000	-0.09599218
2509	3.68750000	2.25000000	-0.09364614
2510	3.81250000	2.50000000	-0.08875855
2511	3.68750000	2.50000000	-0.01935484
2512	3.81250000	2.56250000	-0.11319648
2513	3.75000000	2.56250000	-0.11045943
2514	3.75000000	2.43750000	-0.08895406
2515	3.68750000	2.43750000	-0.08308895
2516	3.50000000	1.68750000	-0.04340176
2517	3.50000000	1.81250000	-0.10361681
2518	3.56250000	1.75000000	-0.16969697
2519	3.56250000	1.81250000	-0.16089932
2520	3.43750000	1.68750000	-0.16871945
2521	3.43750000	1.75000000	-0.16031281
2522	3.50000000	1.31250000	-0.11300098
2523	3.50000000	1.18750000	-0.16480938
2524	3.43750000	1.25000000	-0.17653959
2525	3.43750000	1.18750000	-0.17047898
2526	3.56250000	1.31250000	-0.16070381
2527	3.56250000	1.25000000	-0.16793744
2528	3.31250000	1.50000000	-0.18963832
2529	3.18750000	1.50000000	-0.18924731
2530	3.25000000	1.43750000	-0.19061584
2531	3.18750000	1.43750000	-0.19042033
2532	3.31250000	1.56250000	-0.18201369
2533	3.25000000	1.56250000	-0.18103617
2534	3.50000000	2.18750000	0.00997067

2535	3.50000000	2.31250000	-0.01270772
2536	3.43750000	2.18750000	-0.09794721
2537	3.43750000	2.25000000	-0.08739003
2538	3.56250000	2.25000000	-0.08211144
2539	3.56250000	2.31250000	-0.07859238
2540	3.31250000	1.81250000	-0.15073314
2541	3.18750000	1.68750000	-0.16559140
2542	3.25000000	1.81250000	-0.15855327
2543	3.18750000	1.75000000	-0.16637341
2544	3.31250000	1.75000000	-0.15835777
2545	3.25000000	1.68750000	-0.16578690
2546	0.50000000	1.18750000	-0.05415445
2547	0.50000000	1.31250000	-0.19393939
2548	0.43750000	1.18750000	-0.19335288
2549	0.43750000	1.25000000	-0.19413490
2550	0.56250000	1.25000000	-0.19335288
2551	0.56250000	1.31250000	-0.19393939
2552	0.68750000	1.18750000	-0.19257087
2553	0.81250000	1.31250000	-0.19393939
2554	0.75000000	1.18750000	-0.19413490
2555	0.81250000	1.25000000	-0.19491691
2556	0.68750000	1.25000000	-0.19335288
2557	0.75000000	1.31250000	-0.19393939
2558	0.68750000	1.50000000	-0.19589443
2559	0.81250000	1.50000000	-0.19276637
2560	0.75000000	1.56250000	-0.19589443
2561	0.81250000	1.56250000	-0.19589443
2562	0.68750000	1.43750000	-0.19511241
2563	0.75000000	1.43750000	-0.19511241
2564	1.18750000	0.50000000	-0.05630499
2565	1.31250000	0.50000000	-0.00860215
2566	1.18750000	0.43750000	-0.02600196
2567	1.25000000	0.43750000	-0.00058651
2568	1.25000000	0.56250000	-0.05356794
2569	1.31250000	0.56250000	-0.02971652
2570	1.18750000	0.68750000	-0.12023460
2571	1.31250000	0.81250000	-0.11378299
2572	1.18750000	0.75000000	-0.12785924
2573	1.25000000	0.81250000	-0.12512219
2574	1.25000000	0.68750000	-0.09560117
2575	1.31250000	0.75000000	-0.09266862
2576	1.50000000	0.68750000	-0.09990225
2577	1.50000000	0.81250000	-0.04203324
2578	1.56250000	0.75000000	-0.00879765
2579	1.56250000	0.81250000	0.01955034
2580	1.43750000	0.68750000	-0.02385142
2581	1.43750000	0.75000000	-0.04477028
2582	0.50000000	3.31250000	0.18885630
2583	0.50000000	3.18750000	0.18709677
2584	0.43750000	3.25000000	0.16168133
2585	0.43750000	3.18750000	0.18690127
2586	0.56250000	3.31250000	0.18572825
2587	0.56250000	3.25000000	0.18533724
2588	0.68750000	3.50000000	0.18123167
2589	0.81250000	3.50000000	0.17497556
2590	0.75000000	3.56250000	0.16735093
2591	0.81250000	3.56250000	0.17086999
2592	0.68750000	3.43750000	0.18064516
2593	0.75000000	3.43750000	0.17771261

2594	0.68750000	3.18750000	0.17869013
2595	0.81250000	3.31250000	0.17380254
2596	0.75000000	3.18750000	0.17438905
2597	0.81250000	3.25000000	0.17165200
2598	0.68750000	3.25000000	0.17947214
2599	0.75000000	3.31250000	0.17693060
2600	3.31250000	0.50000000	-0.00410557
2601	3.18750000	0.50000000	0.01348974
2602	3.25000000	0.43750000	0.02385142
2603	3.18750000	0.43750000	0.01231672
2604	3.31250000	0.56250000	-0.01876833
2605	3.25000000	0.56250000	-0.01818182
2606	3.50000000	0.68750000	-0.05826002
2607	3.50000000	0.81250000	-0.09423265
2608	3.56250000	0.75000000	-0.08367546
2609	3.56250000	0.81250000	-0.10694037
2610	3.43750000	0.68750000	-0.05689150
2611	3.43750000	0.75000000	-0.07526882
2612	3.18750000	0.68750000	-0.10733138
2613	3.31250000	0.81250000	-0.14193548
2614	3.18750000	0.75000000	-0.12922776
2615	3.25000000	0.81250000	-0.14740958
2616	3.25000000	0.68750000	-0.10478983
2617	3.31250000	0.75000000	-0.12375367
2618	2.81250000	3.31250000	0.18905181
2619	2.68750000	3.18750000	0.17517107
2620	2.81250000	3.25000000	0.16852395
2621	2.75000000	3.18750000	0.17086999
2622	2.75000000	3.31250000	0.16344086
2623	2.68750000	3.25000000	0.15972630
2624	2.81250000	3.50000000	0.08563050
2625	2.68750000	3.50000000	0.09755621
2626	2.81250000	3.56250000	0.09736070
2627	2.75000000	3.56250000	0.07820137
2628	2.75000000	3.43750000	0.13255132
2629	2.68750000	3.43750000	0.11339198
2630	3.31250000	2.81250000	0.06901271
2631	3.18750000	2.68750000	0.00723363
2632	3.25000000	2.81250000	0.06783969
2633	3.18750000	2.75000000	0.04320626
2634	3.31250000	2.75000000	0.05513196
2635	3.25000000	2.68750000	0.03362659
2636	3.50000000	2.81250000	0.07917889
2637	3.50000000	2.68750000	0.08895406
2638	3.56250000	2.81250000	0.07429130
2639	3.56250000	2.75000000	-0.05219941
2640	3.43750000	2.75000000	0.05767351
2641	3.43750000	2.68750000	0.03753666
2642	2.50000000	3.18750000	0.11769306
2643	2.50000000	3.31250000	0.08641251
2644	2.43750000	3.18750000	0.12492669
2645	2.43750000	3.25000000	0.09403715
2646	2.56250000	3.25000000	0.12140762
2647	2.56250000	3.31250000	0.10596285
2648	3.18750000	2.50000000	-0.05317693
2649	3.31250000	2.50000000	0.02463343
2650	3.18750000	2.43750000	-0.06080156
2651	3.25000000	2.43750000	-0.05004888
2652	3.25000000	2.56250000	-0.00664712

2653	3.31250000	2.56250000	-0.00527859
2654	1.18750000	2.50000000	-0.02150538
2655	1.31250000	2.50000000	-0.00469208
2656	1.18750000	2.43750000	-0.03362659
2657	1.25000000	2.43750000	-0.03010753
2658	1.25000000	2.56250000	0.01251222
2659	1.31250000	2.56250000	0.01622678
2660	1.18750000	2.68750000	0.04125122
2661	1.31250000	2.81250000	0.02091887
2662	1.18750000	2.75000000	0.02248289
2663	1.25000000	2.81250000	0.09677419
2664	1.25000000	2.68750000	0.05434995
2665	1.31250000	2.75000000	0.07898338
2666	1.50000000	2.68750000	0.06920821
2667	1.50000000	2.81250000	0.03206256
2668	1.56250000	2.75000000	0.10576735
2669	1.56250000	2.81250000	0.11417400
2670	1.43750000	2.68750000	0.06549365
2671	1.43750000	2.75000000	0.08641251
2672	2.50000000	1.18750000	-0.09853372
2673	2.50000000	1.31250000	-0.10576735
2674	2.43750000	1.18750000	-0.08739003
2675	2.43750000	1.25000000	-0.03792766
2676	2.56250000	1.25000000	-0.11026393
2677	2.56250000	1.31250000	-0.11065494
2678	2.68750000	1.18750000	-0.13196481
2679	2.81250000	1.31250000	-0.15542522
2680	2.75000000	1.18750000	-0.09501466
2681	2.81250000	1.25000000	-0.15640274
2682	2.68750000	1.25000000	-0.13255132
2683	2.75000000	1.31250000	-0.14408602
2684	2.68750000	1.50000000	-0.14076246
2685	2.81250000	1.50000000	-0.15679374
2686	2.75000000	1.56250000	-0.14604106
2687	2.81250000	1.56250000	-0.15757576
2688	2.68750000	1.43750000	-0.13391984
2689	2.75000000	1.43750000	-0.14506354
2690	0.06250000	0.12500000	0.05669599
2691	0.06250000	0.18750000	0.04125122
2692	0.12500000	0.06250000	0.08093842
2693	0.18750000	0.06250000	0.09130010
2694	0.12500000	0.18750000	0.04418377
2695	0.18750000	0.12500000	0.06999022
2696	0.06250000	3.93750000	0.09833822
2697	0.06250000	3.87500000	0.10303030
2698	0.12500000	3.93750000	0.09090909
2699	3.93750000	0.06250000	0.06510264
2700	3.87500000	0.06250000	0.07214076
2701	3.93750000	0.12500000	0.09481916
2702	3.87500000	3.93750000	0.10498534
2703	3.93750000	3.87500000	0.13352884
2704	3.81250000	3.87500000	0.11221896
2705	3.87500000	3.81250000	0.14076246
2706	3.81250000	3.93750000	0.08093842
2707	3.93750000	3.81250000	0.13802542
2708	0.06250000	1.93750000	-0.09208211
2709	0.06250000	1.87500000	-0.09149560
2710	0.06250000	2.12500000	-0.12981427
2711	0.06250000	2.18750000	-0.06901271

2712	0.12500000	2.06250000	-0.18338221
2713	0.12500000	1.93750000	-0.18494624
2714	0.18750000	2.06250000	-0.17614858
2715	0.12500000	2.18750000	-0.12043011
2716	0.18750000	2.12500000	-0.17399804
2717	1.93750000	0.06250000	0.19472141
2718	1.87500000	0.06250000	0.19100684
2719	2.12500000	0.06250000	0.18064516
2720	2.18750000	0.06250000	0.18025415
2721	2.06250000	0.12500000	0.18064516
2722	1.93750000	0.12500000	0.19433040
2723	2.06250000	0.18750000	0.18025415
2724	2.18750000	0.12500000	0.16285435
2725	2.12500000	0.18750000	0.16285435
2726	1.87500000	1.93750000	0.02541544
2727	1.93750000	1.87500000	0.03714565
2728	1.81250000	1.87500000	0.00957967
2729	1.87500000	1.81250000	0.02130987
2730	2.06250000	2.12500000	0.07116325
2731	2.12500000	2.06250000	0.04516129
2732	2.12500000	2.18750000	0.07526882
2733	2.18750000	2.12500000	0.04926686
2734	1.93750000	2.06250000	0.05102639
2735	1.81250000	1.93750000	0.00977517
2736	1.87500000	2.06250000	0.03519062
2737	2.06250000	1.93750000	0.03675464
2738	1.93750000	1.81250000	0.03323558
2739	2.06250000	1.87500000	0.03264907
2740	2.06250000	2.18750000	0.08054741
2741	1.93750000	2.12500000	0.06060606
2742	2.18750000	2.06250000	0.02854350
2743	2.12500000	1.93750000	0.02033236
2744	1.87500000	3.93750000	0.04633431
2745	1.81250000	3.93750000	0.07057674
2746	2.06250000	3.93750000	-0.01759531
2747	2.12500000	3.93750000	-0.01036168
2748	1.93750000	3.87500000	0.00175953
2749	1.81250000	3.87500000	0.07096774
2750	1.87500000	3.81250000	0.02795699
2751	2.06250000	3.87500000	-0.03597263
2752	1.93750000	3.81250000	-0.01700880
2753	3.93750000	1.87500000	-0.19354839
2754	3.93750000	1.81250000	-0.19120235
2755	3.93750000	2.06250000	-0.17086999
2756	3.93750000	2.12500000	-0.15210166
2757	3.87500000	1.93750000	-0.18807429
2758	3.87500000	1.81250000	-0.19257087
2759	3.81250000	1.87500000	-0.18729228
2760	3.87500000	2.06250000	-0.16246334
2761	3.81250000	1.93750000	-0.18044966
2762	0.06250000	0.93750000	-0.17614858
2763	0.06250000	0.87500000	-0.15640274
2764	0.06250000	1.12500000	-0.19667644
2765	0.06250000	1.18750000	-0.19628543
2766	0.12500000	1.06250000	-0.19608993
2767	0.12500000	0.93750000	-0.17556207
2768	0.18750000	1.06250000	-0.19530792
2769	0.12500000	1.18750000	-0.19648094
2770	0.18750000	1.12500000	-0.19608993

2771	0.93750000	0.06250000	0.06373412
2772	0.87500000	0.06250000	0.08797654
2773	1.12500000	0.06250000	0.09208211
2774	1.18750000	0.06250000	0.12394917
2775	1.06250000	0.12500000	0.05493646
2776	0.93750000	0.12500000	0.02873900
2777	1.06250000	0.18750000	0.02443793
2778	1.18750000	0.12500000	0.13724340
2779	1.12500000	0.18750000	0.07487781
2780	0.87500000	0.93750000	-0.17595308
2781	0.93750000	0.87500000	-0.16774194
2782	0.81250000	0.87500000	-0.15679374
2783	0.87500000	0.81250000	-0.14799609
2784	1.06250000	1.12500000	-0.18201369
2785	1.12500000	1.06250000	-0.16871945
2786	1.12500000	1.18750000	-0.16383187
2787	1.18750000	1.12500000	-0.15288368
2788	0.93750000	1.06250000	-0.19745846
2789	0.81250000	0.93750000	-0.17614858
2790	0.87500000	1.06250000	-0.19667644
2791	1.06250000	0.93750000	-0.17595308
2792	0.93750000	0.81250000	-0.15913978
2793	1.06250000	0.87500000	-0.16735093
2794	1.06250000	1.18750000	-0.18142717
2795	0.93750000	1.12500000	-0.19882698
2796	1.18750000	1.06250000	-0.15718475
2797	1.12500000	0.93750000	-0.16441838
2798	0.06250000	3.12500000	0.09599218
2799	0.06250000	3.18750000	0.09149560
2800	0.06250000	2.93750000	0.13958944
2801	0.06250000	2.87500000	0.10342131
2802	0.12500000	3.06250000	0.15268817
2803	0.12500000	3.18750000	0.12316716
2804	0.18750000	3.12500000	0.12707722
2805	0.12500000	2.93750000	0.14154448
2806	0.18750000	3.06250000	0.15210166
2807	0.87500000	3.93750000	0.09012708
2808	0.81250000	3.93750000	0.08504399
2809	1.06250000	3.93750000	0.10850440
2810	1.12500000	3.93750000	0.12062561
2811	0.93750000	3.87500000	0.11182796
2812	0.81250000	3.87500000	0.10342131
2813	0.87500000	3.81250000	0.12531769
2814	1.06250000	3.87500000	0.12727273
2815	0.93750000	3.81250000	0.12864125
2816	3.12500000	0.06250000	0.07370479
2817	3.18750000	0.06250000	0.11632454
2818	2.93750000	0.06250000	0.09325513
2819	2.87500000	0.06250000	0.10518084
2820	3.06250000	0.12500000	0.05102639
2821	3.18750000	0.12500000	0.07409580
2822	3.12500000	0.18750000	0.05161290
2823	2.93750000	0.12500000	0.08250244
2824	3.06250000	0.18750000	0.07116325
2825	3.93750000	0.87500000	-0.17262952
2826	3.93750000	0.81250000	-0.17028348
2827	3.93750000	1.06250000	-0.19882698
2828	3.93750000	1.12500000	-0.19902248
2829	3.87500000	0.93750000	-0.17575758

2830	3.87500000	0.81250000	-0.14995112
2831	3.81250000	0.87500000	-0.15288368
2832	3.87500000	1.06250000	-0.19941349
2833	3.81250000	0.93750000	-0.17634409
2834	3.06250000	3.12500000	0.10694037
2835	3.12500000	3.06250000	0.10009775
2836	3.12500000	3.18750000	0.12609971
2837	3.18750000	3.12500000	0.12238514
2838	2.87500000	2.93750000	0.08543500
2839	2.93750000	2.87500000	0.05161290
2840	2.81250000	2.87500000	0.07448680
2841	2.87500000	2.81250000	0.04066471
2842	2.93750000	3.06250000	0.11085044
2843	3.06250000	3.18750000	0.12766373
2844	2.93750000	3.12500000	0.12512219
2845	3.06250000	2.93750000	0.07018573
2846	3.18750000	3.06250000	0.11710655
2847	3.12500000	2.93750000	0.07761486
2848	2.81250000	2.93750000	0.10224829
2849	2.87500000	3.06250000	0.12746823
2850	2.93750000	2.81250000	0.03460411
2851	3.06250000	2.87500000	0.05298143
2852	3.06250000	3.93750000	-0.06099707
2853	3.12500000	3.93750000	-0.04281525
2854	2.87500000	3.93750000	-0.11612903
2855	2.81250000	3.93750000	-0.08387097
2856	2.93750000	3.87500000	-0.05923754
2857	3.06250000	3.87500000	-0.02189638
2858	2.93750000	3.81250000	-0.02013685
2859	2.81250000	3.87500000	-0.04613881
2860	2.87500000	3.81250000	-0.03929619
2861	3.93750000	3.06250000	0.05610948
2862	3.93750000	3.12500000	0.08211144
2863	3.93750000	2.87500000	0.07956989
2864	3.93750000	2.81250000	0.06803519
2865	3.87500000	2.93750000	0.08367546
2866	3.87500000	3.06250000	0.06099707
2867	3.81250000	2.93750000	-0.02404692
2868	3.87500000	2.81250000	0.12082111
2869	3.81250000	2.87500000	0.02463343
2870	0.87500000	1.93750000	-0.14056696
2871	0.93750000	2.06250000	-0.11867058
2872	0.81250000	1.93750000	-0.08523949
2873	0.87500000	2.06250000	-0.06353861
2874	1.06250000	1.93750000	-0.18005865
2875	1.12500000	2.06250000	-0.15464321
2876	1.12500000	1.93750000	-0.16422287
2877	1.18750000	2.06250000	-0.13900293
2878	0.93750000	1.87500000	-0.19608993
2879	0.81250000	1.87500000	-0.14076246
2880	0.87500000	1.81250000	-0.19608993
2881	1.06250000	1.87500000	-0.18025415
2882	0.93750000	1.81250000	-0.19608993
2883	1.06250000	2.12500000	-0.14877810
2884	0.93750000	2.12500000	-0.09716520
2885	1.06250000	2.18750000	-0.12727273
2886	1.18750000	2.12500000	-0.12961877
2887	1.12500000	2.18750000	-0.12375367
2888	0.87500000	2.93750000	0.11945259

2889	0.93750000	2.87500000	0.04340176
2890	0.81250000	2.87500000	0.08504399
2891	0.87500000	2.81250000	0.00899316
2892	1.06250000	3.12500000	0.16089932
2893	1.12500000	3.06250000	0.15913978
2894	1.12500000	3.18750000	0.16422287
2895	1.18750000	3.12500000	0.16441838
2896	0.93750000	3.06250000	0.15913978
2897	0.81250000	2.93750000	0.12414467
2898	0.87500000	3.06250000	0.16070381
2899	1.06250000	3.18750000	0.16304985
2900	0.93750000	3.12500000	0.16129032
2901	1.06250000	2.93750000	0.08132942
2902	0.93750000	2.81250000	-0.02795699
2903	1.06250000	2.87500000	0.00332356
2904	1.18750000	3.06250000	0.16148583
2905	1.12500000	2.93750000	0.07820137
2906	1.93750000	0.87500000	0.02658847
2907	2.06250000	0.93750000	0.00645161
2908	1.93750000	0.81250000	0.04887586
2909	2.06250000	0.87500000	0.03206256
2910	1.93750000	1.06250000	0.03323558
2911	2.06250000	1.12500000	0.03460411
2912	1.93750000	1.12500000	0.03734115
2913	2.06250000	1.18750000	0.03851417
2914	1.87500000	0.93750000	0.03812317
2915	1.87500000	0.81250000	0.03225806
2916	1.81250000	0.87500000	0.04692082
2917	1.87500000	1.06250000	0.07038123
2918	1.81250000	0.93750000	0.07507331
2919	2.12500000	1.06250000	0.01935484
2920	2.12500000	0.93750000	-0.00469208
2921	2.18750000	1.06250000	0.00821114
2922	2.12500000	1.18750000	0.07292278
2923	2.18750000	1.12500000	0.05786901
2924	2.93750000	0.87500000	-0.14623656
2925	2.87500000	0.93750000	-0.10928641
2926	2.87500000	0.81250000	-0.12062561
2927	2.81250000	0.87500000	-0.08347996
2928	3.12500000	1.06250000	-0.14193548
2929	3.06250000	1.12500000	-0.14701857
2930	3.18750000	1.12500000	-0.14017595
2931	3.12500000	1.18750000	-0.14604106
2932	3.06250000	0.93750000	-0.16735093
2933	2.93750000	0.81250000	-0.12238514
2934	3.06250000	0.87500000	-0.14115347
2935	3.18750000	1.06250000	-0.18494624
2936	3.12500000	0.93750000	-0.16050831
2937	2.93750000	1.06250000	-0.13548387
2938	2.81250000	0.93750000	-0.04828935
2939	2.87500000	1.06250000	-0.07292278
2940	3.06250000	1.18750000	-0.19589443
2941	2.93750000	1.12500000	-0.13431085
2942	2.06250000	2.93750000	0.18260020
2943	1.93750000	2.87500000	0.16911046
2944	2.06250000	2.87500000	0.17771261
2945	1.93750000	2.81250000	0.16754643
2946	2.06250000	3.12500000	0.12199413
2947	1.93750000	3.06250000	0.15131965

2948	2.06250000	3.18750000	0.07820137
2949	1.93750000	3.12500000	0.10752688
2950	2.12500000	3.06250000	0.16148583
2951	2.12500000	2.93750000	0.18455523
2952	2.18750000	3.06250000	0.16344086
2953	2.12500000	3.18750000	0.08973607
2954	2.18750000	3.12500000	0.13548387
2955	1.87500000	2.93750000	0.17732160
2956	1.87500000	2.81250000	0.16089932
2957	1.81250000	2.87500000	0.16578690
2958	1.87500000	3.06250000	0.14838710
2959	1.81250000	2.93750000	0.18064516
2960	2.93750000	2.06250000	-0.18416422
2961	2.87500000	1.93750000	-0.18025415
2962	2.87500000	2.06250000	-0.18162268
2963	2.81250000	1.93750000	-0.17771261
2964	3.12500000	2.06250000	-0.16774194
2965	3.06250000	1.93750000	-0.18670577
2966	3.18750000	2.06250000	-0.15992180
2967	3.12500000	1.93750000	-0.17829912
2968	3.06250000	2.12500000	-0.16793744
2969	2.93750000	2.12500000	-0.17634409
2970	3.06250000	2.18750000	-0.16031281
2971	3.18750000	2.12500000	-0.14916911
2972	3.12500000	2.18750000	-0.14936461
2973	2.93750000	1.87500000	-0.18299120
2974	2.81250000	1.87500000	-0.16852395
2975	2.87500000	1.81250000	-0.17086999
2976	3.06250000	1.87500000	-0.18651026
2977	2.93750000	1.81250000	-0.18279570
2978	0.06250000	0.43750000	-0.04828935
2979	0.06250000	0.37500000	-0.03499511
2980	0.06250000	0.62500000	-0.07331378
2981	0.06250000	0.68750000	-0.09325513
2982	0.12500000	0.56250000	-0.07859238
2983	0.12500000	0.43750000	-0.07487781
2984	0.18750000	0.56250000	-0.10381232
2985	0.12500000	0.68750000	-0.08895406
2986	0.18750000	0.62500000	-0.09423265
2987	0.43750000	0.06250000	0.13900293
2988	0.37500000	0.06250000	0.12453568
2989	0.62500000	0.06250000	0.12688172
2990	0.68750000	0.06250000	0.11104594
2991	0.56250000	0.12500000	0.11652004
2992	0.43750000	0.12500000	0.11163245
2993	0.56250000	0.18750000	0.08895406
2994	0.68750000	0.12500000	0.09110459
2995	0.62500000	0.18750000	0.07937439
2996	0.37500000	0.43750000	-0.04477028
2997	0.43750000	0.37500000	0.00762463
2998	0.31250000	0.37500000	-0.03167155
2999	0.37500000	0.31250000	0.02678397
3000	0.56250000	0.62500000	-0.07800587
3001	0.62500000	0.56250000	-0.08074291
3002	0.62500000	0.68750000	-0.09716520
3003	0.68750000	0.62500000	-0.08778104
3004	0.43750000	0.56250000	-0.08328446
3005	0.31250000	0.43750000	-0.07585533
3006	0.37500000	0.56250000	-0.10772239

3007	0.56250000	0.43750000	-0.03362659
3008	0.43750000	0.31250000	0.03499511
3009	0.56250000	0.37500000	0.00625611
3010	0.56250000	0.68750000	-0.09677419
3011	0.43750000	0.62500000	-0.10205279
3012	0.68750000	0.56250000	-0.09012708
3013	0.62500000	0.43750000	-0.04320626
3014	0.06250000	3.62500000	0.12179863
3015	0.06250000	3.68750000	0.11730205
3016	0.06250000	3.43750000	0.14838710
3017	0.06250000	3.37500000	0.15288368
3018	0.12500000	3.56250000	0.13274682
3019	0.12500000	3.68750000	0.11065494
3020	0.18750000	3.62500000	0.12160313
3021	0.12500000	3.43750000	0.15483871
3022	0.18750000	3.56250000	0.13919844
3023	0.37500000	3.93750000	0.06529814
3024	0.31250000	3.93750000	0.06959922
3025	0.56250000	3.93750000	-0.03421310
3026	0.62500000	3.93750000	0.01994135
3027	0.43750000	3.87500000	-0.02326491
3028	0.31250000	3.87500000	0.07761486
3029	0.37500000	3.81250000	0.03870968
3030	0.56250000	3.87500000	-0.06568915
3031	0.43750000	3.81250000	-0.05786901
3032	3.62500000	0.06250000	0.13880743
3033	3.68750000	0.06250000	0.08093842
3034	3.43750000	0.06250000	0.14330401
3035	3.37500000	0.06250000	0.13196481
3036	3.56250000	0.12500000	0.11554252
3037	3.68750000	0.12500000	0.04828935
3038	3.62500000	0.18750000	0.02561095
3039	3.43750000	0.12500000	0.11358749
3040	3.56250000	0.18750000	0.03499511
3041	3.93750000	0.37500000	0.02521994
3042	3.93750000	0.31250000	0.02072336
3043	3.93750000	0.56250000	0.00801564
3044	3.93750000	0.62500000	-0.07976540
3045	3.87500000	0.43750000	-0.01388074
3046	3.87500000	0.31250000	-0.05982405
3047	3.81250000	0.37500000	-0.09872923
3048	3.87500000	0.56250000	-0.06021505
3049	3.81250000	0.43750000	-0.05728250
3050	3.56250000	3.62500000	0.14095797
3051	3.62500000	3.56250000	0.16911046
3052	3.62500000	3.68750000	0.13372434
3053	3.68750000	3.62500000	0.16187683
3054	3.37500000	3.43750000	0.17262952
3055	3.43750000	3.37500000	0.16500489
3056	3.31250000	3.37500000	0.16050831
3057	3.37500000	3.31250000	0.17478006
3058	3.43750000	3.56250000	0.15014663
3059	3.56250000	3.68750000	0.10948192
3060	3.43750000	3.62500000	0.06842620
3061	3.56250000	3.43750000	0.17067449
3062	3.68750000	3.56250000	0.16578690
3063	3.62500000	3.43750000	0.16735093
3064	3.31250000	3.43750000	0.17145650
3065	3.37500000	3.56250000	0.10185728

3066	3.43750000	3.31250000	0.17810362
3067	3.56250000	3.37500000	0.16500489
3068	3.56250000	3.93750000	-0.07800587
3069	3.62500000	3.93750000	-0.04144673
3070	3.37500000	3.93750000	-0.03245357
3071	3.31250000	3.93750000	-0.02619746
3072	3.43750000	3.87500000	-0.02033236
3073	3.56250000	3.87500000	0.00312805
3074	3.43750000	3.81250000	0.01055718
3075	3.31250000	3.87500000	-0.00097752
3076	3.37500000	3.81250000	0.02365591
3077	3.93750000	3.56250000	0.15581623
3078	3.93750000	3.62500000	0.15112414
3079	3.93750000	3.37500000	0.14232649
3080	3.93750000	3.31250000	0.12883675
3081	3.87500000	3.43750000	0.15796676
3082	3.87500000	3.56250000	0.15777126
3083	3.81250000	3.43750000	0.16011730
3084	3.87500000	3.31250000	0.13998045
3085	3.81250000	3.37500000	0.15562072
3086	0.06250000	1.62500000	-0.14506354
3087	0.06250000	1.68750000	-0.14565005
3088	0.06250000	1.43750000	-0.18866080
3089	0.06250000	1.37500000	-0.18905181
3090	0.12500000	1.56250000	-0.18807429
3091	0.12500000	1.68750000	-0.19472141
3092	0.18750000	1.62500000	-0.18768328
3093	0.12500000	1.43750000	-0.18240469
3094	0.18750000	1.56250000	-0.18162268
3095	0.06250000	2.43750000	-0.07468231
3096	0.06250000	2.37500000	-0.03519062
3097	0.06250000	2.62500000	-0.09090909
3098	0.06250000	2.68750000	-0.05474096
3099	0.12500000	2.56250000	-0.12668622
3100	0.12500000	2.43750000	-0.07448680
3101	0.18750000	2.56250000	-0.12629521
3102	0.12500000	2.68750000	-0.10322581
3103	0.18750000	2.62500000	-0.13900293
3104	0.37500000	1.93750000	-0.16265885
3105	0.43750000	2.06250000	-0.14760508
3106	0.31250000	1.93750000	-0.16950147
3107	0.37500000	2.06250000	-0.15464321
3108	0.56250000	1.93750000	-0.10772239
3109	0.62500000	2.06250000	-0.02952102
3110	0.62500000	1.93750000	-0.06549365
3111	0.68750000	2.06250000	0.01270772
3112	0.43750000	1.87500000	-0.16246334
3113	0.31250000	1.87500000	-0.17517107
3114	0.37500000	1.81250000	-0.17497556
3115	0.56250000	2.12500000	-0.06920821
3116	0.43750000	2.12500000	-0.14525904
3117	0.56250000	2.18750000	-0.06666667
3118	0.56250000	1.87500000	-0.11436950
3119	0.43750000	1.81250000	-0.16911046
3120	0.68750000	2.12500000	0.04203324
3121	0.62500000	2.18750000	0.00234604
3122	0.37500000	2.43750000	-0.13313783
3123	0.43750000	2.37500000	-0.13646139
3124	0.31250000	2.37500000	-0.14076246

3125	0.37500000	2.31250000	-0.14584555
3126	0.56250000	2.62500000	-0.05180841
3127	0.62500000	2.56250000	-0.07800587
3128	0.62500000	2.68750000	-0.01759531
3129	0.68750000	2.62500000	-0.04379277
3130	0.43750000	2.56250000	-0.08484848
3131	0.31250000	2.43750000	-0.13020528
3132	0.37500000	2.56250000	-0.08504399
3133	0.56250000	2.43750000	-0.11436950
3134	0.43750000	2.31250000	-0.13861193
3135	0.56250000	2.37500000	-0.11652004
3136	0.56250000	2.68750000	-0.01290323
3137	0.43750000	2.62500000	-0.04731183
3138	0.68750000	2.56250000	-0.06529814
3139	0.62500000	2.43750000	-0.10166178
3140	1.62500000	0.06250000	0.17653959
3141	1.68750000	0.06250000	0.18025415
3142	1.43750000	0.06250000	0.12434018
3143	1.37500000	0.06250000	0.09266862
3144	1.56250000	0.12500000	0.15933529
3145	1.68750000	0.12500000	0.17986315
3146	1.62500000	0.18750000	0.16285435
3147	1.43750000	0.12500000	0.11085044
3148	1.56250000	0.18750000	0.14604106
3149	2.43750000	0.06250000	0.16852395
3150	2.37500000	0.06250000	0.17067449
3151	2.62500000	0.06250000	0.15405670
3152	2.68750000	0.06250000	0.14213099
3153	2.56250000	0.12500000	0.14408602
3154	2.43750000	0.12500000	0.14662757
3155	2.56250000	0.18750000	0.12199413
3156	2.68750000	0.12500000	0.13176931
3157	2.62500000	0.18750000	0.12160313
3158	1.93750000	0.37500000	0.19296188
3159	2.06250000	0.43750000	0.17321603
3160	1.93750000	0.31250000	0.19315738
3161	2.06250000	0.37500000	0.17360704
3162	1.93750000	0.56250000	0.14760508
3163	2.06250000	0.62500000	0.14330401
3164	1.93750000	0.62500000	0.10342131
3165	2.06250000	0.68750000	0.11476051
3166	1.87500000	0.43750000	0.17302053
3167	1.87500000	0.31250000	0.18963832
3168	1.81250000	0.37500000	0.16969697
3169	2.12500000	0.56250000	0.14897361
3170	2.12500000	0.43750000	0.15034213
3171	2.18750000	0.56250000	0.12609971
3172	1.87500000	0.56250000	0.11241447
3173	1.81250000	0.43750000	0.15327468
3174	2.12500000	0.68750000	0.11945259
3175	2.18750000	0.62500000	0.12512219
3176	2.43750000	0.37500000	0.06021505
3177	2.37500000	0.43750000	0.06119257
3178	2.37500000	0.31250000	0.09051808
3179	2.31250000	0.37500000	0.09149560
3180	2.62500000	0.56250000	-0.06979472
3181	2.56250000	0.62500000	-0.03225806
3182	2.68750000	0.62500000	-0.09540567
3183	2.62500000	0.68750000	-0.05786901

3184	2.56250000	0.43750000	-0.02052786
3185	2.43750000	0.31250000	0.08367546
3186	2.56250000	0.37500000	0.00293255
3187	2.43750000	0.56250000	0.01798631
3188	2.31250000	0.43750000	0.08563050
3189	2.37500000	0.56250000	0.04242424
3190	2.68750000	0.56250000	-0.12003910
3191	2.62500000	0.43750000	-0.07096774
3192	2.56250000	0.68750000	-0.04496579
3193	2.43750000	0.62500000	0.00508309
3194	1.56250000	1.62500000	-0.05376344
3195	1.62500000	1.56250000	-0.04203324
3196	1.62500000	1.68750000	-0.03792766
3197	1.68750000	1.62500000	-0.02619746
3198	1.37500000	1.43750000	-0.11417400
3199	1.43750000	1.37500000	-0.10224829
3200	1.31250000	1.37500000	-0.12981427
3201	1.37500000	1.31250000	-0.11808407
3202	1.43750000	1.56250000	-0.08582600
3203	1.56250000	1.68750000	-0.05356794
3204	1.43750000	1.62500000	-0.08563050
3205	1.56250000	1.43750000	-0.06217009
3206	1.68750000	1.56250000	-0.03010753
3207	1.62500000	1.43750000	-0.05043988
3208	1.31250000	1.43750000	-0.11788856
3209	1.37500000	1.56250000	-0.10205279
3210	1.43750000	1.31250000	-0.09423265
3211	1.56250000	1.37500000	-0.06666667
3212	2.37500000	2.43750000	0.09188661
3213	2.43750000	2.37500000	0.05982405
3214	2.31250000	2.37500000	0.08778104
3215	2.37500000	2.31250000	0.06197458
3216	2.56250000	2.62500000	0.08602151
3217	2.62500000	2.56250000	0.06471163
3218	2.62500000	2.68750000	0.07233627
3219	2.68750000	2.62500000	0.05102639
3220	2.43750000	2.56250000	0.10518084
3221	2.31250000	2.43750000	0.09736070
3222	2.37500000	2.56250000	0.11065494
3223	2.56250000	2.43750000	0.05180841
3224	2.43750000	2.31250000	0.03949169
3225	2.56250000	2.37500000	-0.06881720
3226	2.56250000	2.68750000	0.09521017
3227	2.43750000	2.62500000	0.11436950
3228	2.68750000	2.56250000	0.05259042
3229	2.62500000	2.43750000	-0.05434995
3230	1.56250000	1.93750000	-0.05317693
3231	1.62500000	2.06250000	-0.02776149
3232	1.62500000	1.93750000	-0.03753666
3233	1.68750000	2.06250000	-0.01192571
3234	1.37500000	1.93750000	-0.10068426
3235	1.43750000	2.06250000	-0.10048876
3236	1.31250000	1.93750000	-0.11652004
3237	1.37500000	2.06250000	-0.11632454
3238	1.43750000	1.87500000	-0.08484848
3239	1.56250000	1.87500000	-0.05337243
3240	1.43750000	1.81250000	-0.08504399
3241	1.56250000	2.12500000	-0.05923754
3242	1.68750000	2.12500000	-0.00234604

3243	1.62500000	2.18750000	-0.03382209
3244	1.31250000	1.87500000	-0.11652004
3245	1.37500000	1.81250000	-0.10087977
3246	1.43750000	2.12500000	-0.11612903
3247	1.56250000	2.18750000	-0.07487781
3248	1.93750000	1.56250000	0.01700880
3249	2.06250000	1.62500000	0.02893451
3250	1.93750000	1.62500000	0.02091887
3251	2.06250000	1.68750000	0.02052786
3252	1.93750000	1.37500000	0.00293255
3253	2.06250000	1.43750000	0.08015640
3254	1.93750000	1.31250000	0.00039101
3255	2.06250000	1.37500000	-0.02424242
3256	1.87500000	1.43750000	-0.00469208
3257	1.87500000	1.56250000	0.00527859
3258	1.81250000	1.43750000	-0.01505376
3259	2.12500000	1.56250000	0.08445748
3260	2.12500000	1.68750000	0.01681329
3261	2.18750000	1.62500000	-0.02815249
3262	1.87500000	1.31250000	-0.01329423
3263	1.81250000	1.37500000	-0.02111437
3264	2.12500000	1.43750000	0.03929619
3265	2.18750000	1.56250000	0.03108504
3266	2.06250000	2.43750000	0.13157380
3267	1.93750000	2.37500000	0.11163245
3268	2.06250000	2.37500000	0.12199413
3269	1.93750000	2.31250000	0.10205279
3270	2.06250000	2.62500000	0.15777126
3271	1.93750000	2.56250000	0.16520039
3272	2.06250000	2.68750000	0.16422287
3273	1.93750000	2.62500000	0.15757576
3274	2.12500000	2.56250000	0.14604106
3275	2.12500000	2.43750000	0.11378299
3276	2.18750000	2.56250000	0.13900293
3277	1.87500000	2.43750000	0.13352884
3278	1.87500000	2.31250000	0.07390029
3279	1.81250000	2.37500000	0.09247312
3280	2.12500000	2.68750000	0.17517107
3281	2.18750000	2.62500000	0.16168133
3282	1.87500000	2.56250000	0.17595308
3283	1.81250000	2.43750000	0.14252199
3284	2.43750000	2.06250000	-0.03734115
3285	2.37500000	1.93750000	0.05434995
3286	2.37500000	2.06250000	-0.02072336
3287	2.31250000	1.93750000	-0.02913001
3288	2.62500000	2.06250000	-0.10146628
3289	2.56250000	1.93750000	-0.08132942
3290	2.68750000	2.06250000	-0.12512219
3291	2.62500000	1.93750000	-0.15874878
3292	2.56250000	2.12500000	-0.05239492
3293	2.43750000	2.12500000	-0.01642229
3294	2.56250000	2.18750000	-0.02776149
3295	2.43750000	1.87500000	0.05943304
3296	2.31250000	1.87500000	0.06686217
3297	2.37500000	1.81250000	0.02209189
3298	2.68750000	2.12500000	-0.09540567
3299	2.62500000	2.18750000	-0.04711632
3300	2.56250000	1.87500000	-0.10889541
3301	2.43750000	1.81250000	-0.06881720

3302	1.56250000	3.93750000	0.08563050
3303	1.62500000	3.93750000	0.11163245
3304	1.37500000	3.93750000	0.07839687
3305	1.31250000	3.93750000	0.11319648
3306	1.43750000	3.87500000	0.08152493
3307	1.56250000	3.87500000	0.10478983
3308	1.43750000	3.81250000	0.12590420
3309	1.31250000	3.87500000	0.11906158
3310	1.37500000	3.81250000	0.12864125
3311	2.37500000	3.93750000	0.08367546
3312	2.31250000	3.93750000	0.04105572
3313	2.56250000	3.93750000	0.10850440
3314	2.62500000	3.93750000	0.06236559
3315	2.43750000	3.87500000	0.11730205
3316	2.31250000	3.87500000	0.04907136
3317	2.37500000	3.81250000	0.06686217
3318	2.56250000	3.87500000	0.09677419
3319	2.43750000	3.81250000	0.09247312
3320	1.56250000	3.62500000	0.04496579
3321	1.62500000	3.56250000	0.00840665
3322	1.62500000	3.68750000	0.07038123
3323	1.68750000	3.62500000	0.03362659
3324	1.37500000	3.43750000	0.07194526
3325	1.43750000	3.37500000	0.06060606
3326	1.31250000	3.37500000	0.11710655
3327	1.37500000	3.31250000	0.13059629
3328	1.43750000	3.56250000	0.07174976
3329	1.56250000	3.68750000	0.10654936
3330	1.43750000	3.62500000	0.11495601
3331	1.56250000	3.43750000	0.02385142
3332	1.68750000	3.56250000	0.03323558
3333	1.62500000	3.43750000	0.05532747
3334	1.31250000	3.43750000	0.10752688
3335	1.37500000	3.56250000	0.10557185
3336	1.43750000	3.31250000	0.10967742
3337	1.56250000	3.37500000	0.07116325
3338	2.06250000	3.62500000	-0.07448680
3339	1.93750000	3.56250000	-0.06451613
3340	2.06250000	3.68750000	-0.06529814
3341	1.93750000	3.62500000	-0.05865103
3342	2.06250000	3.43750000	-0.06881720
3343	1.93750000	3.37500000	-0.05356794
3344	2.06250000	3.37500000	-0.03128055
3345	1.93750000	3.31250000	-0.00977517
3346	2.12500000	3.56250000	-0.07917889
3347	2.12500000	3.68750000	-0.05122190
3348	2.18750000	3.62500000	-0.04887586
3349	1.87500000	3.43750000	-0.05395894
3350	1.87500000	3.56250000	-0.03460411
3351	1.81250000	3.43750000	-0.01075269
3352	2.12500000	3.43750000	-0.06041056
3353	2.18750000	3.56250000	-0.06764418
3354	1.87500000	3.31250000	-0.02365591
3355	1.81250000	3.37500000	-0.02424242
3356	3.93750000	1.56250000	-0.16695992
3357	3.93750000	1.62500000	-0.18181818
3358	3.93750000	1.37500000	-0.04868035
3359	3.93750000	1.31250000	-0.05650049
3360	3.87500000	1.43750000	-0.15933529

3361	3.87500000	1.56250000	-0.16911046
3362	3.81250000	1.43750000	-0.17536657
3363	3.87500000	1.31250000	-0.17223851
3364	3.81250000	1.37500000	-0.18044966
3365	3.93750000	2.37500000	-0.04242424
3366	3.93750000	2.31250000	-0.06119257
3367	3.93750000	2.56250000	-0.06979472
3368	3.93750000	2.62500000	-0.09423265
3369	3.87500000	2.43750000	-0.08289345
3370	3.87500000	2.31250000	-0.09247312
3371	3.81250000	2.37500000	-0.08308895
3372	3.87500000	2.56250000	-0.11652004
3373	3.81250000	2.43750000	-0.09227761
3374	3.62500000	1.56250000	-0.16539589
3375	3.56250000	1.62500000	-0.03440860
3376	3.68750000	1.62500000	-0.16578690
3377	3.62500000	1.68750000	-0.16070381
3378	3.43750000	1.37500000	-0.09012708
3379	3.37500000	1.43750000	-0.15796676
3380	3.37500000	1.31250000	-0.15366569
3381	3.31250000	1.37500000	-0.15894428
3382	3.56250000	1.43750000	-0.12160313
3383	3.68750000	1.56250000	-0.18787879
3384	3.62500000	1.43750000	-0.16930596
3385	3.43750000	1.56250000	-0.05845552
3386	3.56250000	1.68750000	-0.05180841
3387	3.43750000	1.62500000	-0.05083089
3388	3.43750000	1.31250000	-0.11925709
3389	3.56250000	1.37500000	-0.10342131
3390	3.31250000	1.43750000	-0.19237537
3391	3.37500000	1.56250000	-0.18377322
3392	3.62500000	2.06250000	-0.09872923
3393	3.56250000	1.93750000	-0.11436950
3394	3.68750000	2.06250000	-0.15659824
3395	3.62500000	1.93750000	-0.17165200
3396	3.43750000	2.06250000	0.02482893
3397	3.37500000	1.93750000	-0.09540567
3398	3.37500000	2.06250000	-0.08308895
3399	3.31250000	1.93750000	-0.10322581
3400	3.56250000	2.12500000	0.04086022
3401	3.68750000	2.12500000	-0.07253177
3402	3.62500000	2.18750000	-0.05122190
3403	3.43750000	1.87500000	-0.09501466
3404	3.56250000	1.87500000	-0.10322581
3405	3.43750000	1.81250000	-0.10263930
3406	3.43750000	2.12500000	-0.01153470
3407	3.56250000	2.18750000	0.00430108
3408	3.31250000	1.87500000	-0.15190616
3409	3.37500000	1.81250000	-0.15171065
3410	0.37500000	0.93750000	-0.17223851
3411	0.43750000	1.06250000	-0.05591398
3412	0.31250000	0.93750000	-0.17204301
3413	0.37500000	1.06250000	-0.19511241
3414	0.56250000	0.93750000	-0.12434018
3415	0.62500000	1.06250000	-0.19335288
3416	0.62500000	0.93750000	-0.17517107
3417	0.68750000	1.06250000	-0.19491691
3418	0.43750000	0.87500000	-0.10068426
3419	0.31250000	0.87500000	-0.14740958

3420	0.37500000	0.81250000	-0.12746823
3421	0.56250000	0.87500000	-0.10459433
3422	0.43750000	0.81250000	-0.08054741
3423	0.56250000	1.12500000	-0.05337243
3424	0.43750000	1.12500000	-0.05415445
3425	0.56250000	1.18750000	-0.05415445
3426	0.68750000	1.12500000	-0.19335288
3427	0.62500000	1.18750000	-0.19257087
3428	0.37500000	1.43750000	-0.19237537
3429	0.43750000	1.37500000	-0.19198436
3430	0.31250000	1.37500000	-0.18611926
3431	0.37500000	1.31250000	-0.19217986
3432	0.56250000	1.62500000	-0.08328446
3433	0.62500000	1.56250000	-0.08973607
3434	0.62500000	1.68750000	-0.08308895
3435	0.68750000	1.62500000	-0.08973607
3436	0.43750000	1.56250000	-0.18924731
3437	0.31250000	1.43750000	-0.18905181
3438	0.37500000	1.56250000	-0.18240469
3439	0.56250000	1.68750000	-0.18299120
3440	0.43750000	1.62500000	-0.17634409
3441	0.56250000	1.43750000	-0.19530792
3442	0.43750000	1.31250000	-0.19472141
3443	0.56250000	1.37500000	-0.19452590
3444	0.68750000	1.56250000	-0.19608993
3445	0.62500000	1.43750000	-0.19530792
3446	0.93750000	0.37500000	-0.05571848
3447	1.06250000	0.43750000	-0.07350929
3448	0.93750000	0.31250000	-0.02697947
3449	1.06250000	0.37500000	-0.04320626
3450	0.93750000	0.56250000	-0.12375367
3451	1.06250000	0.62500000	-0.13431085
3452	0.93750000	0.62500000	-0.13216031
3453	1.06250000	0.68750000	-0.14193548
3454	0.87500000	0.43750000	-0.07350929
3455	0.87500000	0.31250000	-0.01564027
3456	0.81250000	0.37500000	-0.03323558
3457	0.87500000	0.56250000	-0.11104594
3458	0.81250000	0.43750000	-0.06236559
3459	1.12500000	0.56250000	-0.10185728
3460	1.12500000	0.43750000	-0.04965787
3461	1.18750000	0.56250000	-0.07722385
3462	1.12500000	0.68750000	-0.13137830
3463	1.18750000	0.62500000	-0.09912023
3464	1.43750000	0.37500000	0.09149560
3465	1.37500000	0.43750000	0.05434995
3466	1.37500000	0.31250000	0.09951124
3467	1.31250000	0.37500000	0.06236559
3468	1.62500000	0.56250000	-0.03225806
3469	1.56250000	0.62500000	-0.06666667
3470	1.68750000	0.62500000	0.05904203
3471	1.62500000	0.68750000	0.02443793
3472	1.56250000	0.43750000	-0.01016618
3473	1.43750000	0.31250000	0.09775171
3474	1.56250000	0.37500000	0.10322581
3475	1.68750000	0.56250000	-0.01876833
3476	1.62500000	0.43750000	0.00332356
3477	1.43750000	0.56250000	-0.08172043
3478	1.31250000	0.43750000	0.02346041

3479	1.37500000	0.56250000	-0.00566960
3480	1.56250000	0.68750000	-0.08778104
3481	1.43750000	0.62500000	-0.10283480
3482	1.06250000	1.43750000	-0.18005865
3483	0.93750000	1.37500000	-0.19472141
3484	1.06250000	1.37500000	-0.18181818
3485	0.93750000	1.31250000	-0.19569892
3486	1.06250000	1.62500000	-0.17947214
3487	0.93750000	1.56250000	-0.19296188
3488	1.06250000	1.68750000	-0.18025415
3489	0.93750000	1.62500000	-0.19608993
3490	1.12500000	1.56250000	-0.16402737
3491	1.12500000	1.43750000	-0.16695992
3492	1.18750000	1.56250000	-0.14936461
3493	1.12500000	1.68750000	-0.16363636
3494	1.18750000	1.62500000	-0.14819159
3495	0.87500000	1.43750000	-0.19217986
3496	0.87500000	1.31250000	-0.19550342
3497	0.81250000	1.37500000	-0.19452590
3498	0.87500000	1.56250000	-0.19296188
3499	0.81250000	1.43750000	-0.19217986
3500	1.43750000	1.06250000	-0.09951124
3501	1.37500000	0.93750000	-0.10674487
3502	1.37500000	1.06250000	-0.11065494
3503	1.31250000	0.93750000	-0.11808407
3504	1.62500000	1.06250000	0.02248289
3505	1.56250000	0.93750000	0.02737048
3506	1.68750000	1.06250000	0.08406647
3507	1.62500000	0.93750000	0.08895406
3508	1.56250000	1.12500000	-0.08465298
3509	1.43750000	1.12500000	-0.10752688
3510	1.56250000	1.18750000	-0.07996090
3511	1.68750000	1.12500000	-0.01133920
3512	1.62500000	1.18750000	-0.06823069
3513	1.43750000	0.87500000	-0.08699902
3514	1.31250000	0.87500000	-0.12218964
3515	1.37500000	0.81250000	-0.08973607
3516	1.56250000	0.87500000	-0.00156403
3517	1.43750000	0.81250000	-0.06588465
3518	0.37500000	3.43750000	0.15640274
3519	0.43750000	3.37500000	0.16265885
3520	0.31250000	3.37500000	0.12922776
3521	0.37500000	3.31250000	0.13548387
3522	0.56250000	3.62500000	0.00782014
3523	0.62500000	3.56250000	0.17360704
3524	0.62500000	3.68750000	0.09423265
3525	0.68750000	3.62500000	0.15972630
3526	0.43750000	3.56250000	0.01485826
3527	0.31250000	3.43750000	0.14975562
3528	0.37500000	3.56250000	0.10830890
3529	0.56250000	3.68750000	-0.06080156
3530	0.43750000	3.62500000	-0.05376344
3531	0.56250000	3.43750000	0.18690127
3532	0.43750000	3.31250000	0.16226784
3533	0.56250000	3.37500000	0.18631476
3534	0.68750000	3.56250000	0.17047898
3535	0.62500000	3.43750000	0.18377322
3536	0.37500000	2.93750000	0.07155425
3537	0.43750000	3.06250000	0.15894428

3538	0.31250000	2.93750000	0.07018573
3539	0.37500000	3.06250000	0.15874878
3540	0.56250000	2.93750000	0.13958944
3541	0.62500000	3.06250000	0.17888563
3542	0.62500000	2.93750000	0.13822092
3543	0.68750000	3.06250000	0.17458456
3544	0.43750000	2.87500000	0.05669599
3545	0.31250000	2.87500000	0.00977517
3546	0.37500000	2.81250000	-0.07781036
3547	0.56250000	2.87500000	0.10087977
3548	0.43750000	2.81250000	-0.03225806
3549	0.56250000	3.12500000	0.18338221
3550	0.43750000	3.12500000	0.18572825
3551	0.56250000	3.18750000	0.18416422
3552	0.68750000	3.12500000	0.17653959
3553	0.62500000	3.18750000	0.18162268
3554	1.06250000	3.62500000	0.15992180
3555	0.93750000	3.56250000	0.16461388
3556	1.06250000	3.68750000	0.16050831
3557	0.93750000	3.62500000	0.16050831
3558	1.06250000	3.43750000	0.15483871
3559	0.93750000	3.37500000	0.16637341
3560	1.06250000	3.37500000	0.15268817
3561	0.93750000	3.31250000	0.16422287
3562	1.12500000	3.56250000	0.15522972
3563	1.12500000	3.68750000	0.16304985
3564	1.18750000	3.62500000	0.15835777
3565	1.12500000	3.43750000	0.14584555
3566	1.18750000	3.56250000	0.15112414
3567	0.87500000	3.43750000	0.17145650
3568	0.87500000	3.56250000	0.16774194
3569	0.81250000	3.43750000	0.17458456
3570	0.87500000	3.31250000	0.16891496
3571	0.81250000	3.37500000	0.17419355
3572	3.43750000	0.37500000	-0.00918866
3573	3.37500000	0.43750000	0.01837732
3574	3.37500000	0.31250000	0.06256109
3575	3.31250000	0.37500000	0.04633431
3576	3.62500000	0.56250000	-0.05571848
3577	3.56250000	0.62500000	-0.04770283
3578	3.68750000	0.62500000	-0.08132942
3579	3.62500000	0.68750000	-0.07311828
3580	3.56250000	0.43750000	-0.06001955
3581	3.43750000	0.31250000	0.00821114
3582	3.56250000	0.37500000	-0.04242424
3583	3.68750000	0.56250000	-0.08211144
3584	3.62500000	0.43750000	-0.03890518
3585	3.43750000	0.56250000	-0.02443793
3586	3.31250000	0.43750000	0.01955034
3587	3.37500000	0.56250000	-0.02306940
3588	3.56250000	0.68750000	-0.06588465
3589	3.43750000	0.62500000	-0.03910068
3590	2.93750000	0.37500000	0.04613881
3591	3.06250000	0.43750000	0.02600196
3592	2.93750000	0.31250000	0.04946237
3593	3.06250000	0.37500000	0.02482893
3594	2.93750000	0.56250000	-0.07155425
3595	3.06250000	0.62500000	-0.04437928
3596	2.93750000	0.62500000	-0.09677419

3597	3.06250000	0.68750000	-0.06627566
3598	2.87500000	0.43750000	-0.01446725
3599	2.87500000	0.31250000	0.07038123
3600	2.81250000	0.37500000	0.00957967
3601	2.87500000	0.56250000	-0.12121212
3602	2.81250000	0.43750000	-0.07194526
3603	3.12500000	0.56250000	-0.00742913
3604	3.12500000	0.43750000	0.02561095
3605	3.18750000	0.56250000	-0.00488759
3606	3.12500000	0.68750000	-0.06353861
3607	3.18750000	0.62500000	-0.03910068
3608	3.62500000	1.06250000	-0.16344086
3609	3.56250000	0.93750000	-0.14213099
3610	3.68750000	1.06250000	-0.17634409
3611	3.62500000	0.93750000	-0.15483871
3612	3.43750000	1.06250000	-0.07214076
3613	3.37500000	0.93750000	-0.04789834
3614	3.37500000	1.06250000	-0.07781036
3615	3.31250000	0.93750000	-0.05337243
3616	3.56250000	1.12500000	-0.15718475
3617	3.68750000	1.12500000	-0.16793744
3618	3.62500000	1.18750000	-0.16031281
3619	3.43750000	1.12500000	-0.16500489
3620	3.56250000	1.18750000	-0.16246334
3621	3.43750000	0.87500000	-0.11163245
3622	3.56250000	0.87500000	-0.12512219
3623	3.43750000	0.81250000	-0.09345064
3624	3.31250000	0.87500000	-0.11632454
3625	3.37500000	0.81250000	-0.09266862
3626	3.06250000	3.43750000	0.18084066
3627	2.93750000	3.37500000	0.18240469
3628	3.06250000	3.37500000	0.16383187
3629	2.93750000	3.31250000	0.16187683
3630	3.06250000	3.62500000	0.04320626
3631	2.93750000	3.56250000	0.11026393
3632	3.06250000	3.68750000	0.10674487
3633	2.93750000	3.62500000	0.12199413
3634	3.12500000	3.56250000	0.05806452
3635	3.12500000	3.43750000	0.17908113
3636	3.18750000	3.56250000	0.15874878
3637	3.12500000	3.68750000	-0.01642229
3638	3.18750000	3.62500000	0.02072336
3639	2.87500000	3.43750000	0.12668622
3640	2.87500000	3.31250000	0.17869013
3641	2.81250000	3.37500000	0.17360704
3642	2.87500000	3.56250000	0.06588465
3643	2.81250000	3.43750000	0.10107527
3644	3.43750000	3.06250000	0.12922776
3645	3.37500000	2.93750000	0.09716520
3646	3.37500000	3.06250000	0.12160313
3647	3.31250000	2.93750000	0.09599218
3648	3.62500000	3.06250000	0.14232649
3649	3.56250000	2.93750000	0.09442815
3650	3.68750000	3.06250000	0.13704790
3651	3.62500000	2.93750000	0.08954057
3652	3.56250000	3.12500000	0.17184751
3653	3.43750000	3.12500000	0.14076246
3654	3.56250000	3.18750000	0.15073314
3655	3.68750000	3.12500000	0.19002933

3656	3.62500000	3.18750000	0.17419355
3657	3.43750000	2.87500000	0.09188661
3658	3.31250000	2.87500000	0.08308895
3659	3.37500000	2.81250000	0.07038123
3660	3.56250000	2.87500000	0.09462366
3661	3.43750000	2.81250000	0.07800587
3662	2.56250000	2.93750000	0.15053763
3663	2.62500000	3.06250000	0.16344086
3664	2.62500000	2.93750000	0.14623656
3665	2.68750000	3.06250000	0.15913978
3666	2.37500000	2.93750000	0.17947214
3667	2.43750000	3.06250000	0.15581623
3668	2.31250000	2.93750000	0.18690127
3669	2.37500000	3.06250000	0.16304985
3670	2.43750000	2.87500000	0.16304985
3671	2.56250000	2.87500000	0.14134897
3672	2.43750000	2.81250000	0.15386119
3673	2.31250000	2.87500000	0.18787879
3674	2.37500000	2.81250000	0.17126100
3675	2.56250000	3.12500000	0.15229717
3676	2.68750000	3.12500000	0.16715543
3677	2.62500000	3.18750000	0.15601173
3678	2.43750000	3.12500000	0.14037146
3679	2.56250000	3.18750000	0.13685239
3680	2.93750000	2.56250000	-0.05278592
3681	3.06250000	2.62500000	-0.04985337
3682	2.93750000	2.62500000	-0.01700880
3683	3.06250000	2.68750000	-0.01388074
3684	2.93750000	2.37500000	-0.12043011
3685	3.06250000	2.43750000	-0.11378299
3686	2.93750000	2.31250000	-0.12825024
3687	3.06250000	2.37500000	-0.12140762
3688	2.87500000	2.43750000	-0.06901271
3689	2.87500000	2.56250000	-0.00938416
3690	2.81250000	2.43750000	-0.02561095
3691	2.87500000	2.31250000	-0.10068426
3692	2.81250000	2.37500000	-0.04946237
3693	3.12500000	2.56250000	-0.05943304
3694	3.12500000	2.68750000	-0.00332356
3695	3.18750000	2.62500000	-0.01290323
3696	3.12500000	2.43750000	-0.08719453
3697	3.18750000	2.56250000	-0.03304008
3698	2.56250000	3.62500000	0.03245357
3699	2.62500000	3.56250000	0.03910068
3700	2.62500000	3.68750000	0.01290323
3701	2.68750000	3.62500000	0.01974585
3702	2.37500000	3.43750000	0.01974585
3703	2.43750000	3.37500000	0.06236559
3704	2.31250000	3.37500000	0.02717498
3705	2.37500000	3.31250000	0.06999022
3706	2.43750000	3.56250000	0.02502444
3707	2.56250000	3.68750000	0.04516129
3708	2.43750000	3.62500000	0.03773216
3709	2.31250000	3.43750000	-0.00723363
3710	2.37500000	3.56250000	-0.00195503
3711	2.56250000	3.43750000	0.07429130
3712	2.68750000	3.56250000	0.05865103
3713	2.62500000	3.43750000	0.09384164
3714	2.43750000	3.31250000	0.07820137

3715	2.56250000	3.37500000	0.09012708
3716	3.62500000	2.56250000	0.12160313
3717	3.56250000	2.62500000	0.15777126
3718	3.68750000	2.62500000	0.03049853
3719	3.62500000	2.68750000	0.01661779
3720	3.43750000	2.37500000	0.04007820
3721	3.37500000	2.43750000	0.05259042
3722	3.37500000	2.31250000	-0.03460411
3723	3.31250000	2.37500000	-0.02209189
3724	3.56250000	2.43750000	0.04887586
3725	3.68750000	2.56250000	-0.04437928
3726	3.62500000	2.43750000	-0.01700880
3727	3.43750000	2.31250000	-0.02365591
3728	3.56250000	2.37500000	-0.01485826
3729	3.43750000	2.56250000	0.09755621
3730	3.56250000	2.68750000	-0.02209189
3731	3.43750000	2.62500000	0.06764418
3732	3.31250000	2.43750000	0.00136852
3733	3.37500000	2.56250000	0.04613881
3734	0.93750000	2.37500000	-0.02189638
3735	1.06250000	2.43750000	-0.05982405
3736	0.93750000	2.31250000	-0.03401760
3737	1.06250000	2.37500000	-0.07194526
3738	0.93750000	2.56250000	-0.08289345
3739	1.06250000	2.62500000	-0.04555230
3740	0.93750000	2.62500000	-0.10185728
3741	1.06250000	2.68750000	-0.06432063
3742	0.87500000	2.43750000	-0.01309873
3743	0.87500000	2.31250000	0.02913001
3744	0.81250000	2.37500000	0.08797654
3745	0.87500000	2.56250000	-0.08641251
3746	0.81250000	2.43750000	0.03362659
3747	1.12500000	2.56250000	-0.01368524
3748	1.12500000	2.43750000	-0.04672532
3749	1.18750000	2.56250000	-0.00058651
3750	1.12500000	2.68750000	-0.01153470
3751	1.18750000	2.62500000	0.02033236
3752	1.43750000	2.37500000	-0.05337243
3753	1.37500000	2.43750000	-0.02267840
3754	1.37500000	2.31250000	-0.07878788
3755	1.31250000	2.37500000	-0.04809384
3756	1.62500000	2.56250000	0.08387097
3757	1.56250000	2.62500000	0.06373412
3758	1.68750000	2.62500000	0.12023460
3759	1.62500000	2.68750000	0.10029326
3760	1.56250000	2.43750000	0.01309873
3761	1.43750000	2.31250000	-0.08778104
3762	1.56250000	2.37500000	-0.02150538
3763	1.68750000	2.56250000	0.12492669
3764	1.62500000	2.43750000	0.05395894
3765	1.43750000	2.56250000	0.02365591
3766	1.31250000	2.43750000	-0.02639296
3767	1.37500000	2.56250000	0.01994135
3768	1.56250000	2.68750000	0.08484848
3769	1.43750000	2.62500000	0.04457478
3770	1.43750000	3.06250000	0.01524927
3771	1.37500000	2.93750000	-0.01192571
3772	1.37500000	3.06250000	0.09188661
3773	1.31250000	2.93750000	0.06392962

3774	1.62500000	3.06250000	-0.02013685
3775	1.56250000	2.93750000	-0.00195503
3776	1.68750000	3.06250000	0.06177908
3777	1.62500000	2.93750000	0.08015640
3778	1.56250000	3.12500000	-0.02072336
3779	1.43750000	3.12500000	0.09755621
3780	1.56250000	3.18750000	0.06080156
3781	1.68750000	3.12500000	0.01955034
3782	1.62500000	3.18750000	0.01915934
3783	1.43750000	2.87500000	-0.02971652
3784	1.31250000	2.87500000	0.04242424
3785	1.37500000	2.81250000	0.02463343
3786	1.56250000	2.87500000	0.05610948
3787	1.43750000	2.81250000	0.02834800
3788	2.37500000	0.93750000	-0.03675464
3789	2.43750000	1.06250000	-0.08641251
3790	2.31250000	0.93750000	-0.02561095
3791	2.37500000	1.06250000	-0.07526882
3792	2.56250000	0.93750000	-0.04711632
3793	2.62500000	1.06250000	-0.07174976
3794	2.62500000	0.93750000	-0.01016618
3795	2.68750000	1.06250000	-0.03479961
3796	2.43750000	0.87500000	-0.03479961
3797	2.31250000	0.87500000	0.01251222
3798	2.37500000	0.81250000	0.01446725
3799	2.56250000	0.87500000	-0.03401760
3800	2.43750000	0.81250000	-0.02170088
3801	2.56250000	1.12500000	-0.10909091
3802	2.43750000	1.12500000	-0.08680352
3803	2.56250000	1.18750000	-0.10967742
3804	2.68750000	1.12500000	-0.08328446
3805	2.62500000	1.18750000	-0.12082111
3806	2.37500000	1.43750000	-0.06099707
3807	2.43750000	1.37500000	-0.05122190
3808	2.31250000	1.37500000	0.00684262
3809	2.37500000	1.31250000	0.01661779
3810	2.56250000	1.62500000	-0.14682307
3811	2.62500000	1.56250000	-0.13626588
3812	2.62500000	1.68750000	-0.15210166
3813	2.68750000	1.62500000	-0.14154448
3814	2.43750000	1.56250000	-0.14447703
3815	2.31250000	1.43750000	-0.06412512
3816	2.37500000	1.56250000	-0.14760508
3817	2.56250000	1.68750000	-0.16226784
3818	2.43750000	1.62500000	-0.16011730
3819	2.56250000	1.43750000	-0.12414467
3820	2.43750000	1.31250000	-0.04457478
3821	2.56250000	1.37500000	-0.11730205
3822	2.68750000	1.56250000	-0.14115347
3823	2.62500000	1.43750000	-0.12903226
3824	3.06250000	1.43750000	-0.19139785
3825	2.93750000	1.37500000	-0.17966764
3826	3.06250000	1.37500000	-0.19257087
3827	2.93750000	1.31250000	-0.18064516
3828	3.06250000	1.62500000	-0.18299120
3829	2.93750000	1.56250000	-0.18005865
3830	3.06250000	1.68750000	-0.18377322
3831	2.93750000	1.62500000	-0.18084066
3832	3.12500000	1.56250000	-0.18220919

3833	3.12500000	1.43750000	-0.19178886
3834	3.18750000	1.56250000	-0.18240469
3835	3.12500000	1.68750000	-0.17458456
3836	3.18750000	1.62500000	-0.17399804
3837	2.87500000	1.43750000	-0.16754643
3838	2.87500000	1.31250000	-0.16813294
3839	2.81250000	1.37500000	-0.15581623
3840	2.87500000	1.56250000	-0.16871945
3841	2.81250000	1.43750000	-0.15620723
3842	0.12500000	0.31250000	-0.01642229
3843	0.18750000	0.31250000	-0.01700880
3844	0.18750000	0.37500000	-0.06217009
3845	0.31250000	0.12500000	0.08875855
3846	0.31250000	0.18750000	0.06725318
3847	0.37500000	0.18750000	0.07565982
3848	0.12500000	3.81250000	0.10087977
3849	0.18750000	3.87500000	0.08875855
3850	0.18750000	3.81250000	0.09403715
3851	3.81250000	0.12500000	0.04613881
3852	3.87500000	0.18750000	0.01876833
3853	3.81250000	0.18750000	-0.03695015
3854	3.68750000	3.81250000	0.09521017
3855	3.68750000	3.87500000	0.06392962
3856	3.81250000	3.68750000	0.15190616
3857	3.87500000	3.68750000	0.14916911
3858	3.62500000	3.81250000	0.07096774
3859	3.81250000	3.62500000	0.15581623
3860	0.12500000	1.81250000	-0.18983382
3861	0.18750000	1.87500000	-0.18318671
3862	0.18750000	1.81250000	-0.18866080
3863	0.12500000	2.31250000	-0.04731183
3864	0.18750000	2.31250000	-0.09872923
3865	0.18750000	2.37500000	-0.08641251
3866	0.31250000	2.12500000	-0.15953079
3867	0.31250000	2.18750000	-0.15738025
3868	0.37500000	2.18750000	-0.15014663
3869	1.81250000	0.12500000	0.18709677
3870	1.87500000	0.18750000	0.19022483
3871	1.81250000	0.18750000	0.18670577
3872	2.31250000	0.12500000	0.15562072
3873	2.31250000	0.18750000	0.13822092
3874	2.37500000	0.18750000	0.13137830
3875	2.12500000	0.31250000	0.15659824
3876	2.18750000	0.31250000	0.13919844
3877	2.18750000	0.37500000	0.13333333
3878	1.68750000	1.81250000	-0.02189638
3879	1.68750000	1.87500000	-0.02189638
3880	1.81250000	1.68750000	0.00156403
3881	1.87500000	1.68750000	0.01329423
3882	1.62500000	1.81250000	-0.03773216
3883	1.81250000	1.62500000	-0.00254154
3884	2.18750000	2.31250000	0.08895406
3885	2.12500000	2.31250000	0.09442815
3886	2.31250000	2.18750000	0.03714565
3887	2.31250000	2.12500000	0.01642229
3888	2.18750000	2.37500000	0.09872923
3889	2.37500000	2.18750000	0.02091887
3890	1.81250000	2.12500000	0.02913001
3891	1.87500000	2.18750000	0.05454545

3892	1.81250000	2.18750000	0.03890518
3893	2.12500000	1.81250000	0.01231672
3894	2.18750000	1.87500000	-0.00019550
3895	2.18750000	1.81250000	-0.00410557
3896	1.68750000	3.87500000	0.11300098
3897	1.68750000	3.81250000	0.11358749
3898	1.62500000	3.81250000	0.13137830
3899	2.18750000	3.87500000	-0.01114370
3900	2.12500000	3.81250000	-0.03538612
3901	2.18750000	3.81250000	-0.01779081
3902	1.81250000	3.68750000	0.03382209
3903	1.87500000	3.68750000	-0.00977517
3904	1.81250000	3.62500000	0.01485826
3905	3.87500000	1.68750000	-0.18709677
3906	3.81250000	1.68750000	-0.18924731
3907	3.81250000	1.62500000	-0.18611926
3908	3.87500000	2.18750000	-0.12746823
3909	3.81250000	2.12500000	-0.13704790
3910	3.81250000	2.18750000	-0.12082111
3911	3.68750000	1.81250000	-0.17888563
3912	3.68750000	1.87500000	-0.17008798
3913	3.62500000	1.81250000	-0.16930596
3914	0.12500000	0.81250000	-0.13235582
3915	0.18750000	0.87500000	-0.15151515
3916	0.18750000	0.81250000	-0.12805474
3917	0.12500000	1.31250000	-0.18944282
3918	0.18750000	1.31250000	-0.18944282
3919	0.18750000	1.37500000	-0.18279570
3920	0.31250000	1.12500000	-0.19433040
3921	0.31250000	1.18750000	-0.19511241
3922	0.37500000	1.18750000	-0.19413490
3923	0.81250000	0.12500000	0.06412512
3924	0.87500000	0.18750000	0.03304008
3925	0.81250000	0.18750000	0.04418377
3926	1.31250000	0.12500000	0.12414467
3927	1.31250000	0.18750000	0.13743891
3928	1.37500000	0.18750000	0.09247312
3929	1.12500000	0.31250000	0.01251222
3930	1.18750000	0.31250000	0.03792766
3931	1.18750000	0.37500000	0.00606061
3932	0.68750000	0.81250000	-0.13646139
3933	0.68750000	0.87500000	-0.15620723
3934	0.81250000	0.68750000	-0.11827957
3935	0.87500000	0.68750000	-0.12942326
3936	0.62500000	0.81250000	-0.13567937
3937	0.81250000	0.62500000	-0.10830890
3938	1.18750000	1.31250000	-0.14838710
3939	1.12500000	1.31250000	-0.16598240
3940	1.31250000	1.18750000	-0.12570870
3941	1.31250000	1.12500000	-0.13000978
3942	1.18750000	1.37500000	-0.15112414
3943	1.37500000	1.18750000	-0.11436950
3944	0.81250000	1.12500000	-0.19648094
3945	0.87500000	1.18750000	-0.19726295
3946	0.81250000	1.18750000	-0.19569892
3947	1.12500000	0.81250000	-0.14740958
3948	1.18750000	0.87500000	-0.14467253
3949	1.18750000	0.81250000	-0.13626588
3950	0.12500000	3.31250000	0.13900293

3951	0.18750000	3.31250000	0.12062561
3952	0.18750000	3.37500000	0.14095797
3953	0.12500000	2.81250000	0.01915934
3954	0.18750000	2.87500000	0.05689150
3955	0.18750000	2.81250000	-0.02932551
3956	0.31250000	3.18750000	0.13079179
3957	0.31250000	3.12500000	0.15601173
3958	0.37500000	3.18750000	0.16031281
3959	0.68750000	3.87500000	0.04340176
3960	0.68750000	3.81250000	0.06197458
3961	0.62500000	3.81250000	0.00703812
3962	1.18750000	3.87500000	0.14662757
3963	1.12500000	3.81250000	0.15151515
3964	1.18750000	3.81250000	0.15874878
3965	0.81250000	3.68750000	0.14936461
3966	0.87500000	3.68750000	0.15288368
3967	0.81250000	3.62500000	0.16011730
3968	3.31250000	0.12500000	0.10948192
3969	3.31250000	0.18750000	0.09833822
3970	3.37500000	0.18750000	0.09110459
3971	2.81250000	0.12500000	0.10713587
3972	2.87500000	0.18750000	0.08406647
3973	2.81250000	0.18750000	0.09677419
3974	3.18750000	0.31250000	0.05962854
3975	3.12500000	0.31250000	0.04809384
3976	3.18750000	0.37500000	0.03597263
3977	3.87500000	0.68750000	-0.14780059
3978	3.81250000	0.68750000	-0.12766373
3979	3.81250000	0.62500000	-0.12785924
3980	3.87500000	1.18750000	-0.18983382
3981	3.81250000	1.12500000	-0.19022483
3982	3.81250000	1.18750000	-0.18044966
3983	3.68750000	0.81250000	-0.12238514
3984	3.68750000	0.87500000	-0.14565005
3985	3.62500000	0.81250000	-0.11456500
3986	3.18750000	3.31250000	0.15307918
3987	3.12500000	3.31250000	0.15444770
3988	3.31250000	3.18750000	0.16363636
3989	3.31250000	3.12500000	0.13626588
3990	3.18750000	3.37500000	0.16070381
3991	3.37500000	3.18750000	0.16050831
3992	2.68750000	2.81250000	0.08641251
3993	2.68750000	2.87500000	0.11417400
3994	2.81250000	2.68750000	0.03128055
3995	2.87500000	2.68750000	0.02502444
3996	2.62500000	2.81250000	0.10928641
3997	2.81250000	2.62500000	0.03264907
3998	2.87500000	3.18750000	0.14995112
3999	2.81250000	3.12500000	0.15229717
4000	2.81250000	3.18750000	0.16050831
4001	3.18750000	2.87500000	0.07116325
4002	3.12500000	2.81250000	0.04652981
4003	3.18750000	2.81250000	0.05728250
4004	3.18750000	3.87500000	0.00078201
4005	3.12500000	3.81250000	0.02170088
4006	3.18750000	3.81250000	0.02619746
4007	2.68750000	3.87500000	0.01837732
4008	2.68750000	3.81250000	0.00586510
4009	2.62500000	3.81250000	0.03812317

4010	2.87500000	3.68750000	0.03870968
4011	2.81250000	3.68750000	0.01955034
4012	2.81250000	3.62500000	0.05845552
4013	3.87500000	3.18750000	0.10674487
4014	3.81250000	3.12500000	0.13587488
4015	3.81250000	3.18750000	0.15562072
4016	3.87500000	2.68750000	-0.01583578
4017	3.81250000	2.68750000	-0.01309873
4018	3.81250000	2.62500000	-0.13822092
4019	3.68750000	2.87500000	0.00332356
4020	3.68750000	2.81250000	-0.12316716
4021	3.62500000	2.81250000	-0.03675464
4022	0.68750000	1.87500000	-0.07878788
4023	0.68750000	1.81250000	-0.13411535
4024	0.81250000	2.12500000	0.02111437
4025	0.87500000	2.18750000	-0.01251222
4026	0.62500000	1.81250000	-0.12746823
4027	0.81250000	2.18750000	0.05063539
4028	1.18750000	1.87500000	-0.14838710
4029	1.12500000	1.81250000	-0.16441838
4030	1.31250000	2.12500000	-0.12297165
4031	1.31250000	2.18750000	-0.11358749
4032	1.18750000	1.81250000	-0.14838710
4033	1.37500000	2.18750000	-0.12258065
4034	0.81250000	1.68750000	-0.19608993
4035	0.87500000	1.68750000	-0.19608993
4036	0.81250000	1.62500000	-0.19608993
4037	1.12500000	2.31250000	-0.08054741
4038	1.18750000	2.31250000	-0.07702835
4039	1.18750000	2.37500000	-0.05532747
4040	0.68750000	2.81250000	0.05552297
4041	0.68750000	2.87500000	0.09462366
4042	0.81250000	2.68750000	-0.04672532
4043	0.87500000	2.68750000	-0.08387097
4044	0.62500000	2.81250000	0.06041056
4045	0.81250000	2.62500000	-0.06823069
4046	1.18750000	3.31250000	0.15953079
4047	1.12500000	3.31250000	0.15503421
4048	1.31250000	3.18750000	0.17321603
4049	1.31250000	3.12500000	0.17047898
4050	1.18750000	3.37500000	0.14819159
4051	1.37500000	3.18750000	0.17693060
4052	0.81250000	3.12500000	0.16754643
4053	0.87500000	3.18750000	0.16480938
4054	0.81250000	3.18750000	0.16950147
4055	1.12500000	2.81250000	-0.01505376
4056	1.18750000	2.87500000	0.05923754
4057	1.18750000	2.81250000	0.04398827
4058	1.87500000	0.68750000	0.06138807
4059	1.81250000	0.68750000	0.06353861
4060	2.12500000	0.81250000	0.05591398
4061	2.18750000	0.87500000	0.02228739
4062	1.81250000	0.62500000	0.07038123
4063	2.18750000	0.81250000	0.05728250
4064	1.87500000	1.18750000	0.02932551
4065	1.81250000	1.12500000	0.06236559
4066	2.12500000	1.31250000	0.00586510
4067	2.18750000	1.31250000	0.04027370
4068	1.81250000	1.18750000	0.01720430

4069	2.18750000	1.37500000	-0.03069404
4070	1.68750000	0.81250000	0.04359726
4071	1.68750000	0.87500000	0.07194526
4072	1.62500000	0.81250000	0.03167155
4073	2.31250000	1.12500000	-0.01446725
4074	2.31250000	1.18750000	0.03499511
4075	2.37500000	1.18750000	-0.02619746
4076	2.81250000	0.68750000	-0.12121212
4077	2.87500000	0.68750000	-0.12160313
4078	2.68750000	0.81250000	-0.04652981
4079	2.68750000	0.87500000	-0.00977517
4080	2.81250000	0.62500000	-0.14604106
4081	2.62500000	0.81250000	-0.03382209
4082	3.31250000	1.18750000	-0.18299120
4083	3.31250000	1.12500000	-0.17693060
4084	3.18750000	1.31250000	-0.19413490
4085	3.12500000	1.31250000	-0.19393939
4086	3.37500000	1.18750000	-0.17673509
4087	3.18750000	1.37500000	-0.19315738
4088	3.12500000	0.81250000	-0.10987292
4089	3.18750000	0.87500000	-0.12805474
4090	3.18750000	0.81250000	-0.10361681
4091	2.81250000	1.12500000	-0.05904203
4092	2.87500000	1.18750000	-0.12043011
4093	2.81250000	1.18750000	-0.10772239
4094	2.12500000	2.81250000	0.18064516
4095	2.18750000	2.87500000	0.18748778
4096	1.87500000	2.68750000	0.16383187
4097	1.81250000	2.68750000	0.16031281
4098	2.18750000	2.81250000	0.18846530
4099	1.81250000	2.62500000	0.16480938
4100	2.12500000	3.31250000	0.01153470
4101	2.18750000	3.31250000	0.02306940
4102	1.87500000	3.18750000	0.06236559
4103	1.81250000	3.12500000	0.10303030
4104	2.18750000	3.37500000	-0.01133920
4105	1.81250000	3.18750000	0.06080156
4106	2.31250000	3.12500000	0.14252199
4107	2.31250000	3.18750000	0.11163245
4108	2.37500000	3.18750000	0.11671554
4109	1.68750000	2.81250000	0.14525904
4110	1.68750000	2.87500000	0.15366569
4111	1.62500000	2.81250000	0.12981427
4112	2.81250000	2.12500000	-0.14643206
4113	2.87500000	2.18750000	-0.14115347
4114	2.68750000	1.87500000	-0.17614858
4115	2.68750000	1.81250000	-0.16676442
4116	2.81250000	2.18750000	-0.11378299
4117	2.62500000	1.81250000	-0.17693060
4118	3.31250000	2.12500000	-0.13040078
4119	3.31250000	2.18750000	-0.11984360
4120	3.18750000	1.87500000	-0.16891496
4121	3.12500000	1.81250000	-0.17673509
4122	3.37500000	2.18750000	-0.10889541
4123	3.18750000	1.81250000	-0.16754643
4124	3.12500000	2.31250000	-0.11827957
4125	3.18750000	2.31250000	-0.10752688
4126	3.18750000	2.37500000	-0.08406647
4127	2.81250000	1.68750000	-0.15855327

4128	2.87500000	1.68750000	-0.17008798
4129	2.81250000	1.62500000	-0.15796676
4130	0.31250000	0.62500000	-0.12297165
4131	0.31250000	0.68750000	-0.11339198
4132	0.37500000	0.68750000	-0.11691105
4133	0.62500000	0.31250000	0.02424242
4134	0.68750000	0.31250000	0.01485826
4135	0.68750000	0.37500000	-0.01270772
4136	0.31250000	3.68750000	0.06608016
4137	0.31250000	3.62500000	0.08387097
4138	0.37500000	3.68750000	0.02189638
4139	3.68750000	0.31250000	-0.09853372
4140	3.62500000	0.31250000	-0.03812317
4141	3.68750000	0.37500000	-0.08172043
4142	3.37500000	3.68750000	0.03734115
4143	3.31250000	3.62500000	0.07077224
4144	3.31250000	3.68750000	0.08797654
4145	3.68750000	3.37500000	0.15972630
4146	3.62500000	3.31250000	0.15738025
4147	3.68750000	3.31250000	0.15542522
4148	0.31250000	1.68750000	-0.18142717
4149	0.31250000	1.62500000	-0.17536657
4150	0.37500000	1.68750000	-0.17556207
4151	0.31250000	2.62500000	-0.09305963
4152	0.31250000	2.68750000	-0.15268817
4153	0.37500000	2.68750000	-0.10713587
4154	0.62500000	2.31250000	-0.04946237
4155	0.68750000	2.31250000	0.06979472
4156	0.68750000	2.37500000	0.01544477
4157	1.68750000	0.31250000	0.14975562
4158	1.62500000	0.31250000	0.13313783
4159	1.68750000	0.37500000	0.13333333
4160	2.62500000	0.31250000	0.02600196
4161	2.68750000	0.31250000	0.02580645
4162	2.68750000	0.37500000	-0.04770283
4163	2.31250000	0.62500000	0.06588465
4164	2.31250000	0.68750000	0.06490714
4165	2.37500000	0.68750000	0.02854350
4166	1.37500000	1.68750000	-0.10146628
4167	1.31250000	1.62500000	-0.11769306
4168	1.31250000	1.68750000	-0.11730205
4169	1.68750000	1.37500000	-0.04320626
4170	1.62500000	1.31250000	-0.05923754
4171	1.68750000	1.31250000	-0.04750733
4172	2.31250000	2.62500000	0.13724340
4173	2.37500000	2.68750000	0.14095797
4174	2.31250000	2.68750000	0.15835777
4175	2.62500000	2.31250000	-0.09872923
4176	2.68750000	2.37500000	-0.09051808
4177	2.68750000	2.31250000	-0.01427175
4178	1.68750000	2.31250000	0.02600196
4179	1.62500000	2.31250000	-0.01505376
4180	1.68750000	2.37500000	0.06041056
4181	2.31250000	1.68750000	-0.09384164
4182	2.31250000	1.62500000	-0.12238514
4183	2.37500000	1.68750000	-0.13470186
4184	1.37500000	3.68750000	0.16089932
4185	1.31250000	3.68750000	0.16363636
4186	1.31250000	3.62500000	0.15151515

4187	2.31250000	3.68750000	0.00234604
4188	2.37500000	3.68750000	0.02639296
4189	2.31250000	3.62500000	-0.01329423
4190	1.68750000	3.37500000	0.06080156
4191	1.62500000	3.31250000	0.10166178
4192	1.68750000	3.31250000	0.05982405
4193	3.68750000	1.37500000	-0.17380254
4194	3.68750000	1.31250000	-0.18103617
4195	3.62500000	1.31250000	-0.15835777
4196	3.68750000	2.31250000	-0.09012708
4197	3.68750000	2.37500000	-0.08660802
4198	3.62500000	2.31250000	-0.08426197
4199	3.37500000	1.68750000	-0.16774194
4200	3.31250000	1.62500000	-0.17517107
4201	3.31250000	1.68750000	-0.16676442
4202	0.62500000	1.31250000	-0.19393939
4203	0.68750000	1.31250000	-0.19393939
4204	0.68750000	1.37500000	-0.19452590
4205	1.31250000	0.62500000	-0.05063539
4206	1.31250000	0.68750000	-0.07155425
4207	1.37500000	0.68750000	-0.04770283
4208	0.62500000	3.31250000	0.18279570
4209	0.68750000	3.37500000	0.18025415
4210	0.68750000	3.31250000	0.17986315
4211	3.31250000	0.62500000	-0.03714565
4212	3.37500000	0.68750000	-0.05610948
4213	3.31250000	0.68750000	-0.05552297
4214	2.68750000	3.31250000	0.14428152
4215	2.68750000	3.37500000	0.12883675
4216	2.62500000	3.31250000	0.12512219
4217	3.31250000	2.68750000	0.03499511
4218	3.37500000	2.68750000	0.03636364
4219	3.31250000	2.62500000	0.01485826
4220	1.31250000	2.62500000	0.03714565
4221	1.31250000	2.68750000	0.05806452
4222	1.37500000	2.68750000	0.06177908
4223	2.62500000	1.31250000	-0.12179863
4224	2.68750000	1.31250000	-0.13294233
4225	2.68750000	1.37500000	-0.13333333
4226	0.03125000	0.03125000	0.16314761
4227	0.00000000	1.96875000	-0.14965787
4228	3.03125000	1.03125000	-0.17341153
4229	0.50000000	3.96875000	0.00097752
4230	4.00000000	0.53125000	0.12072336
4231	3.96875000	0.46875000	0.10977517
4232	0.53125000	2.03125000	-0.10752688
4233	2.46875000	1.96875000	-0.01857283
4234	1.46875000	3.96875000	0.01388074
4235	4.00000000	1.46875000	-0.11358749
4236	3.50000000	1.53125000	-0.12521994
4237	3.53125000	2.03125000	-0.10205279
4238	3.50000000	2.03125000	-0.07429130
4239	0.50000000	1.03125000	-0.12404692
4240	0.53125000	1.53125000	-0.14291300
4241	1.53125000	0.50000000	0.01984360
4242	1.50000000	0.53125000	0.00263930
4243	1.53125000	1.00000000	-0.04956012
4244	0.50000000	3.53125000	0.10586510
4245	0.46875000	2.96875000	0.14252199

4246	3.46875000	1.00000000	-0.10782014
4247	3.03125000	3.53125000	0.12561095
4248	3.46875000	2.46875000	0.14115347
4249	0.96875000	2.46875000	-0.03528837
4250	1.53125000	3.00000000	-0.10107527
4251	1.50000000	3.03125000	-0.10136852
4252	1.46875000	2.96875000	-0.11495601
4253	2.46875000	0.96875000	-0.07253177
4254	2.46875000	1.46875000	-0.09208211
4255	3.75000000	0.03125000	0.11505376
4256	0.00000000	1.78125000	-0.14789834
4257	0.25000000	2.96875000	0.13773216
4258	4.00000000	0.71875000	-0.14379277
4259	4.00000000	1.28125000	-0.13235582
4260	2.78125000	4.00000000	-0.03040078
4261	3.96875000	2.71875000	-0.03245357
4262	3.75000000	3.03125000	0.06129032
4263	3.78125000	2.78125000	-0.10635386
4264	3.71875000	2.71875000	-0.09032258
4265	3.78125000	2.75000000	-0.11212121
4266	0.71875000	1.96875000	0.00087977
4267	0.71875000	1.71875000	-0.14291300
4268	1.03125000	2.78125000	-0.10215054
4269	1.21875000	2.96875000	0.12101662
4270	2.28125000	1.28125000	0.11173021
4271	3.21875000	1.21875000	-0.17077224
4272	3.28125000	1.00000000	-0.12453568
4273	2.78125000	1.03125000	0.02003910
4274	2.96875000	1.21875000	-0.16383187
4275	1.71875000	3.00000000	0.14496579
4276	3.28125000	2.28125000	-0.08289345
4277	0.28125000	3.53125000	0.13235582
4278	0.46875000	3.71875000	-0.05327468
4279	3.71875000	0.21875000	-0.10498534
4280	3.78125000	0.28125000	-0.14535679
4281	3.75000000	0.21875000	-0.13391984
4282	3.28125000	3.53125000	0.15474096
4283	3.21875000	3.71875000	0.01945259
4284	0.28125000	2.78125000	-0.12023460
4285	0.25000000	2.78125000	-0.12091887
4286	0.53125000	1.78125000	-0.14545455
4287	0.71875000	2.21875000	0.10596285
4288	0.78125000	2.28125000	0.11632454
4289	0.46875000	2.75000000	-0.01867058
4290	0.75000000	2.46875000	-0.02189638
4291	2.53125000	0.28125000	0.06334311
4292	1.71875000	0.50000000	0.06031281
4293	2.28125000	0.78125000	0.07526882
4294	2.71875000	0.46875000	-0.15835777
4295	2.53125000	2.28125000	-0.03714565
4296	2.71875000	2.46875000	-0.01251222
4297	1.53125000	2.28125000	-0.09384164
4298	2.28125000	1.78125000	0.01896383
4299	2.21875000	1.50000000	-0.05835777
4300	1.71875000	2.46875000	0.13255132
4301	2.50000000	1.78125000	-0.14995112
4302	1.53125000	3.78125000	0.00879765
4303	1.53125000	3.75000000	0.00909091
4304	1.46875000	3.75000000	0.01935484

4305	1.46875000	3.71875000	0.01329423
4306	2.28125000	3.78125000	0.01837732
4307	1.71875000	3.50000000	-0.13245357
4308	1.75000000	3.53125000	-0.11984360
4309	1.78125000	3.53125000	-0.14164223
4310	1.71875000	3.46875000	-0.10879765
4311	1.75000000	3.46875000	-0.12971652
4312	1.50000000	3.28125000	0.15728250
4313	1.50000000	3.21875000	0.15259042
4314	1.53125000	3.28125000	0.17302053
4315	2.28125000	3.53125000	-0.06725318
4316	1.78125000	3.28125000	-0.15425220
4317	1.71875000	3.21875000	-0.15298143
4318	1.78125000	3.25000000	-0.13235582
4319	1.75000000	3.21875000	-0.11202346
4320	1.75000000	3.28125000	-0.13264907
4321	1.71875000	3.25000000	-0.11221896
4322	3.50000000	1.71875000	-0.10234604
4323	3.50000000	2.21875000	-0.03323558
4324	0.50000000	1.21875000	-0.12375367
4325	1.50000000	0.71875000	-0.06041056
4326	3.21875000	0.71875000	-0.14017595
4327	3.53125000	2.75000000	0.00332356
4328	1.28125000	2.78125000	0.04809384
4329	0.09375000	0.09375000	0.09413490
4330	0.00000000	1.90625000	-0.04897361
4331	0.00000000	1.84375000	-0.04838710
4332	0.03125000	1.90625000	-0.04535679
4333	0.03125000	1.87500000	-0.04506354
4334	0.09375000	1.96875000	-0.13880743
4335	0.90625000	0.03125000	0.09335288
4336	0.96875000	0.09375000	0.03411535
4337	2.84375000	4.00000000	-0.11270772
4338	2.84375000	3.96875000	-0.11886608
4339	3.84375000	2.96875000	0.00547410
4340	3.84375000	2.84375000	0.05278592
4341	3.84375000	2.87500000	0.07849462
4342	1.03125000	2.90625000	0.04389052
4343	1.09375000	2.96875000	0.11876833
4344	3.09375000	1.09375000	-0.12258065
4345	3.15625000	1.15625000	-0.12170088
4346	2.90625000	1.03125000	-0.10478983
4347	2.96875000	1.09375000	-0.16617791
4348	0.53125000	0.40625000	-0.00889541
4349	0.59375000	0.46875000	-0.05835777
4350	0.59375000	3.96875000	0.00860215
4351	0.40625000	3.87500000	0.02502444
4352	0.50000000	3.90625000	-0.07956989
4353	0.53125000	3.90625000	-0.07702835
4354	0.46875000	3.87500000	-0.07155425
4355	0.46875000	3.84375000	-0.08885630
4356	3.65625000	0.03125000	0.12619746
4357	3.65625000	0.15625000	0.03577713
4358	3.62500000	0.15625000	0.06588465
4359	3.53125000	0.15625000	0.07996090
4360	3.96875000	0.37500000	0.06549365
4361	3.96875000	0.34375000	0.06324536
4362	4.00000000	0.59375000	0.03255132
4363	4.00000000	0.65625000	-0.05562072

4364	3.90625000	0.40625000	-0.00029326
4365	3.84375000	0.34375000	-0.08533724
4366	3.90625000	0.37500000	-0.01505376
4367	3.87500000	0.53125000	-0.01632454
4368	3.84375000	0.46875000	-0.01485826
4369	3.37500000	3.53125000	0.14271750
4370	3.62500000	3.96875000	-0.08201369
4371	0.03125000	1.65625000	-0.12082111
4372	0.59375000	1.96875000	-0.08328446
4373	0.65625000	2.03125000	-0.02306940
4374	0.53125000	2.15625000	-0.10244379
4375	0.53125000	1.90625000	-0.13216031
4376	0.59375000	2.09375000	-0.03597263
4377	0.65625000	2.15625000	0.03519062
4378	2.53125000	0.40625000	0.01642229
4379	2.59375000	0.46875000	-0.05747801
4380	2.53125000	2.37500000	-0.01573803
4381	2.62500000	2.46875000	0.00596285
4382	2.03125000	1.40625000	0.04838710
4383	2.15625000	1.50000000	0.07634409
4384	2.09375000	1.46875000	0.11192571
4385	2.12500000	1.46875000	0.09149560
4386	2.15625000	1.53125000	0.08739003
4387	2.37500000	1.96875000	0.00635386
4388	2.59375000	1.96875000	-0.10625611
4389	2.40625000	1.90625000	0.09403715
4390	2.34375000	1.84375000	0.10654936
4391	2.37500000	1.90625000	0.10234604
4392	2.40625000	1.87500000	0.10488759
4393	2.34375000	1.87500000	0.10860215
4394	2.37500000	1.84375000	0.08621701
4395	2.50000000	1.84375000	-0.07096774
4396	2.53125000	1.90625000	-0.05640274
4397	2.46875000	1.87500000	0.01397849
4398	1.46875000	3.84375000	0.10234604
4399	1.40625000	3.90625000	0.06080156
4400	2.34375000	3.84375000	0.06725318
4401	2.46875000	3.84375000	0.11769306
4402	1.65625000	3.50000000	-0.03880743
4403	1.50000000	3.34375000	0.07888563
4404	1.46875000	3.34375000	0.07468231
4405	1.84375000	3.34375000	-0.09638319
4406	4.00000000	1.40625000	0.00684262
4407	4.00000000	1.34375000	0.00058651
4408	3.96875000	1.37500000	0.00918866
4409	3.96875000	1.34375000	0.00527859
4410	3.90625000	1.37500000	-0.10654936
4411	3.96875000	2.34375000	-0.03616813
4412	3.59375000	1.62500000	-0.08885630
4413	3.59375000	1.46875000	-0.15454545
4414	3.50000000	1.59375000	0.00791789
4415	3.50000000	1.65625000	0.01554252
4416	3.53125000	1.62500000	0.02003910
4417	3.46875000	1.59375000	0.00801564
4418	3.53125000	1.65625000	0.01133920
4419	3.46875000	1.62500000	0.01182796
4420	3.53125000	1.40625000	-0.08866080
4421	3.46875000	1.34375000	-0.08748778
4422	3.40625000	1.53125000	-0.12492669

4423	3.62500000	2.03125000	-0.14076246
4424	3.59375000	1.96875000	-0.14858260
4425	3.40625000	2.03125000	-0.01094819
4426	3.34375000	1.96875000	-0.07497556
4427	3.59375000	2.09375000	-0.04379277
4428	3.62500000	2.09375000	-0.05669599
4429	3.53125000	1.90625000	-0.08015640
4430	3.46875000	1.84375000	-0.07429130
4431	3.50000000	2.09375000	0.03949169
4432	3.50000000	2.15625000	0.05317693
4433	3.46875000	2.09375000	0.06060606
4434	3.46875000	2.12500000	0.04242424
4435	3.53125000	2.15625000	0.05034213
4436	0.40625000	1.03125000	-0.12639296
4437	0.59375000	0.96875000	-0.15962854
4438	0.53125000	0.90625000	-0.08905181
4439	0.50000000	1.09375000	0.01534702
4440	0.50000000	1.15625000	0.01544477
4441	0.46875000	1.09375000	0.01456500
4442	0.53125000	1.12500000	0.01583578
4443	0.46875000	1.12500000	0.01544477
4444	0.53125000	1.15625000	0.01544477
4445	0.59375000	1.12500000	-0.12258065
4446	0.59375000	1.59375000	-0.03655914
4447	0.65625000	1.65625000	-0.03655914
4448	0.59375000	1.62500000	-0.03333333
4449	0.62500000	1.59375000	-0.03655914
4450	0.62500000	1.65625000	-0.03323558
4451	0.65625000	1.62500000	-0.03655914
4452	0.53125000	1.62500000	-0.13323558
4453	0.62500000	1.53125000	-0.14291300
4454	1.59375000	0.62500000	-0.01055718
4455	1.53125000	0.40625000	0.03978495
4456	1.59375000	0.50000000	-0.06676442
4457	1.65625000	0.50000000	-0.05327468
4458	1.59375000	0.46875000	-0.06011730
4459	1.65625000	0.53125000	-0.06441838
4460	1.62500000	0.46875000	-0.05337243
4461	1.40625000	0.53125000	-0.03313783
4462	1.50000000	0.59375000	-0.11837732
4463	1.50000000	0.65625000	-0.13939394
4464	1.53125000	0.62500000	-0.12277615
4465	1.46875000	0.59375000	-0.13030303
4466	1.53125000	0.65625000	-0.13333333
4467	1.59375000	1.00000000	0.06207234
4468	1.62500000	1.03125000	0.07018573
4469	1.59375000	0.96875000	0.07262952
4470	1.62500000	1.09375000	-0.02521994
4471	1.53125000	0.90625000	-0.01788856
4472	0.59375000	3.62500000	0.08533724
4473	0.40625000	3.53125000	0.09589443
4474	0.50000000	3.59375000	-0.06285435
4475	0.53125000	3.62500000	-0.06969697
4476	0.46875000	3.59375000	-0.06617791
4477	0.53125000	3.65625000	-0.10400782
4478	0.46875000	3.62500000	-0.10048876
4479	0.34375000	2.96875000	0.10107527
4480	0.40625000	2.90625000	0.05493646
4481	0.34375000	2.84375000	-0.03264907

4482	0.37500000	2.84375000	-0.03333333
4483	0.46875000	2.84375000	0.03499511
4484	3.46875000	0.34375000	-0.02766373
4485	2.87500000	0.40625000	0.02629521
4486	2.87500000	0.46875000	-0.05522972
4487	3.15625000	0.65625000	-0.07448680
4488	3.40625000	1.00000000	-0.02590420
4489	3.34375000	1.00000000	-0.03147605
4490	3.40625000	1.03125000	-0.02854350
4491	3.37500000	1.03125000	-0.03137830
4492	3.34375000	0.96875000	-0.01915934
4493	3.46875000	1.09375000	-0.11573803
4494	3.03125000	3.62500000	0.10478983
4495	2.96875000	3.59375000	0.13831867
4496	3.03125000	3.65625000	0.13655914
4497	3.12500000	3.53125000	0.12707722
4498	3.09375000	3.59375000	-0.01143695
4499	3.15625000	3.65625000	-0.04682307
4500	3.09375000	3.62500000	-0.01837732
4501	3.12500000	3.59375000	-0.01094819
4502	3.15625000	3.62500000	-0.02961877
4503	2.90625000	3.53125000	0.08220919
4504	2.84375000	3.46875000	0.07761486
4505	3.65625000	3.03125000	0.11319648
4506	3.65625000	2.65625000	0.10087977
4507	3.65625000	2.62500000	0.11348974
4508	3.62500000	2.65625000	0.10654936
4509	3.40625000	2.40625000	0.06647116
4510	3.34375000	2.34375000	-0.00821114
4511	3.65625000	2.53125000	0.00117302
4512	3.46875000	2.34375000	0.01368524
4513	3.53125000	2.65625000	0.04848485
4514	3.34375000	2.46875000	0.03870968
4515	0.96875000	2.34375000	-0.05953079
4516	0.90625000	2.40625000	0.01573803
4517	0.84375000	2.34375000	0.06627566
4518	0.84375000	2.46875000	-0.01691105
4519	1.53125000	2.40625000	-0.02463343
4520	1.59375000	2.46875000	0.05083089
4521	1.37500000	3.03125000	0.05073314
4522	1.34375000	2.96875000	0.03675464
4523	1.59375000	3.00000000	-0.01896383
4524	1.65625000	3.00000000	0.06304985
4525	1.50000000	3.09375000	-0.01984360
4526	1.50000000	3.15625000	0.06480938
4527	1.65625000	3.15625000	-0.09257087
4528	1.65625000	3.12500000	-0.02140762
4529	1.62500000	3.15625000	-0.02160313
4530	1.40625000	2.90625000	-0.06060606
4531	1.34375000	2.84375000	-0.00625611
4532	1.53125000	2.87500000	0.01505376
4533	1.46875000	2.84375000	0.00117302
4534	2.34375000	0.96875000	-0.05024438
4535	2.40625000	0.90625000	-0.02326491
4536	2.34375000	0.84375000	0.02600196
4537	2.46875000	0.84375000	-0.04633431
4538	2.40625000	1.40625000	-0.02404692
4539	2.34375000	1.34375000	0.04389052
4540	2.34375000	1.46875000	-0.09804497

4541	2.46875000	1.34375000	-0.07849462
4542	3.87500000	0.15625000	0.06031281
4543	3.75000000	0.09375000	0.03196481
4544	3.75000000	0.15625000	-0.05102639
4545	3.71875000	0.09375000	0.03567937
4546	3.90625000	0.28125000	-0.02179863
4547	3.87500000	0.21875000	-0.02277615
4548	3.62500000	3.90625000	-0.00087977
4549	0.09375000	1.87500000	-0.13792766
4550	0.09375000	1.71875000	-0.17047898
4551	2.28125000	1.87500000	0.02512219
4552	1.59375000	3.84375000	0.09012708
4553	1.59375000	3.75000000	0.09100684
4554	1.75000000	3.59375000	-0.00097752
4555	1.84375000	3.59375000	-0.06637341
4556	3.90625000	2.28125000	-0.08621701
4557	3.59375000	1.71875000	-0.11495601
4558	0.40625000	1.12500000	-0.12375367
4559	0.12500000	2.84375000	0.06226784
4560	0.12500000	2.78125000	-0.02394917
4561	0.25000000	2.90625000	0.05151515
4562	0.25000000	2.84375000	-0.03470186
4563	0.28125000	2.90625000	0.03929619
4564	0.59375000	3.71875000	-0.01759531
4565	3.40625000	0.28125000	0.04408602
4566	3.87500000	0.59375000	-0.10410557
4567	3.90625000	1.28125000	-0.11827957
4568	2.78125000	3.90625000	-0.04887586
4569	3.09375000	3.71875000	0.07693060
4570	3.84375000	2.75000000	0.03548387
4571	3.87500000	2.71875000	0.04672532
4572	3.84375000	2.71875000	0.04809384
4573	3.90625000	2.65625000	-0.10478983
4574	3.87500000	2.65625000	-0.07839687
4575	3.78125000	2.90625000	-0.05356794
4576	3.78125000	2.87500000	-0.02922776
4577	3.71875000	2.87500000	-0.03988270
4578	3.75000000	3.09375000	0.14359726
4579	3.71875000	3.09375000	0.16089932
4580	3.65625000	2.87500000	0.04652981
4581	3.62500000	2.84375000	0.02649071
4582	3.71875000	2.62500000	-0.05249267
4583	3.71875000	2.59375000	-0.08993157
4584	3.78125000	2.65625000	-0.07429130
4585	3.59375000	2.75000000	-0.10772239
4586	3.62500000	2.71875000	-0.07331378
4587	3.59375000	2.71875000	-0.09266862
4588	3.62500000	2.78125000	-0.10000000
4589	0.71875000	1.84375000	-0.10977517
4590	0.65625000	1.90625000	-0.04780059
4591	0.59375000	1.84375000	-0.09657869
4592	0.71875000	2.09375000	0.04848485
4593	0.62500000	1.71875000	-0.13294233
4594	0.65625000	1.78125000	-0.15845552
4595	0.71875000	1.62500000	-0.14291300
4596	0.90625000	2.28125000	-0.00850440
4597	1.40625000	3.28125000	0.14467253
4598	1.09375000	2.84375000	-0.03391984
4599	1.15625000	2.90625000	0.04046921

4600	1.15625000	2.78125000	0.00684262
4601	1.37500000	3.09375000	0.13304008
4602	1.28125000	2.90625000	0.09110459
4603	1.21875000	2.84375000	0.08113392
4604	2.12500000	1.40625000	-0.01290323
4605	1.71875000	0.59375000	0.02688172
4606	1.59375000	0.71875000	-0.04222874
4607	2.28125000	0.90625000	-0.00097752
4608	2.28125000	1.40625000	-0.03020528
4609	2.40625000	1.28125000	-0.01065494
4610	3.40625000	1.28125000	-0.15102639
4611	3.37500000	1.09375000	-0.12424242
4612	3.28125000	0.90625000	-0.08758553
4613	2.84375000	1.09375000	-0.04115347
4614	2.90625000	1.15625000	-0.10244379
4615	2.78125000	1.15625000	-0.07702835
4616	2.84375000	1.21875000	-0.13841642
4617	1.84375000	3.25000000	-0.03069404
4618	1.75000000	3.15625000	0.03030303
4619	1.71875000	3.12500000	0.06050831
4620	1.59375000	2.87500000	0.09716520
4621	3.40625000	2.12500000	-0.06549365
4622	3.28125000	1.90625000	-0.13147605
4623	3.40625000	2.28125000	-0.06099707
4624	3.28125000	2.40625000	-0.03597263
4625	0.28125000	3.65625000	0.09706745
4626	0.40625000	3.78125000	-0.02688172
4627	0.34375000	3.71875000	0.03509286
4628	0.34375000	3.59375000	0.07057674
4629	0.40625000	3.65625000	0.00869990
4630	0.40625000	3.62500000	-0.00703812
4631	3.62500000	0.21875000	-0.01466276
4632	3.59375000	0.21875000	-0.00997067
4633	3.78125000	0.40625000	-0.09970674
4634	3.34375000	3.59375000	0.06500489
4635	3.37500000	3.59375000	0.06099707
4636	3.21875000	3.62500000	0.07106549
4637	0.40625000	2.75000000	-0.11739980
4638	0.37500000	2.78125000	-0.12228739
4639	0.40625000	2.78125000	-0.09951124
4640	0.59375000	2.21875000	-0.03088954
4641	0.65625000	2.28125000	0.03734115
4642	0.65625000	2.37500000	-0.04418377
4643	0.75000000	2.40625000	0.08250244
4644	0.78125000	2.40625000	0.08416422
4645	0.71875000	2.34375000	0.10224829
4646	0.71875000	2.37500000	0.07507331
4647	1.62500000	0.40625000	0.06001955
4648	2.65625000	0.28125000	0.06265885
4649	2.59375000	0.34375000	-0.01055718
4650	2.65625000	0.40625000	-0.08445748
4651	2.71875000	0.34375000	-0.01104594
4652	2.40625000	0.78125000	0.00293255
4653	2.59375000	2.34375000	-0.12903226
4654	2.65625000	2.40625000	-0.12082111
4655	2.59375000	2.37500000	-0.12189638
4656	2.62500000	2.40625000	-0.11466276
4657	2.65625000	2.37500000	-0.13274682
4658	2.71875000	2.37500000	-0.04828935

4659	1.65625000	2.28125000	-0.01173021
4660	1.71875000	2.34375000	0.06373412
4661	1.59375000	2.34375000	-0.01837732
4662	1.65625000	2.40625000	0.05708700
4663	2.21875000	1.59375000	-0.02521994
4664	2.37500000	1.78125000	-0.04203324
4665	1.40625000	3.75000000	0.12179863
4666	1.40625000	3.78125000	0.14946237
4667	1.40625000	3.65625000	0.10361681
4668	2.40625000	3.78125000	0.06725318
4669	2.34375000	3.59375000	-0.01524927
4670	1.65625000	3.40625000	0.03216031
4671	1.59375000	3.34375000	0.12609971
4672	1.75000000	3.40625000	-0.03059629
4673	1.75000000	3.34375000	-0.03157380
4674	1.65625000	3.25000000	0.02971652
4675	1.62500000	3.21875000	0.05992180
4676	3.59375000	2.21875000	-0.04173998
4677	3.40625000	1.62500000	-0.11348974
4678	3.40625000	1.78125000	-0.13098729
4679	0.59375000	1.21875000	-0.12375367
4680	2.78125000	3.40625000	0.12453568
4681	1.40625000	2.78125000	0.05552297
4682	0.03125000	0.09375000	0.09120235
4683	0.09375000	0.03125000	0.11544477
4684	0.03125000	1.96875000	-0.14604106
4685	0.06250000	1.96875000	-0.14242424
4686	0.96875000	0.03125000	0.08944282
4687	1.03125000	2.96875000	0.11524927
4688	3.09375000	1.03125000	-0.16735093
4689	2.96875000	1.03125000	-0.16715543
4690	3.03125000	1.09375000	-0.17243402
4691	0.53125000	0.46875000	-0.03646139
4692	0.53125000	3.96875000	0.00351906
4693	0.56250000	3.96875000	0.00606061
4694	0.46875000	3.93750000	0.00899316
4695	0.43750000	3.90625000	0.01700880
4696	3.96875000	0.43750000	0.09501466
4697	3.96875000	0.40625000	0.08025415
4698	3.93750000	0.53125000	0.05210166
4699	3.90625000	0.46875000	0.04115347
4700	0.53125000	1.96875000	-0.12551320
4701	0.59375000	2.03125000	-0.06529814
4702	0.53125000	2.09375000	-0.10498534
4703	2.53125000	0.46875000	-0.00703812
4704	2.03125000	1.46875000	0.05249267
4705	2.43750000	1.96875000	-0.01026393
4706	2.40625000	1.96875000	-0.00195503
4707	2.53125000	1.96875000	-0.07810362
4708	2.46875000	1.93750000	-0.00772239
4709	2.46875000	1.90625000	0.00312805
4710	1.40625000	3.96875000	0.05493646
4711	1.46875000	3.90625000	0.05806452
4712	3.96875000	1.43750000	-0.11124145
4713	3.93750000	1.40625000	-0.10889541
4714	3.53125000	1.56250000	-0.11309873
4715	3.56250000	1.59375000	-0.10097752
4716	3.53125000	1.46875000	-0.15669599
4717	3.46875000	1.53125000	-0.12512219

4718	3.43750000	1.53125000	-0.12502444
4719	3.56250000	2.03125000	-0.11495601
4720	3.53125000	1.96875000	-0.12277615
4721	3.59375000	2.03125000	-0.12785924
4722	3.46875000	2.03125000	-0.05317693
4723	3.43750000	2.03125000	-0.03206256
4724	3.56250000	2.09375000	-0.01603128
4725	3.53125000	2.09375000	0.01173021
4726	0.46875000	1.03125000	-0.12482893
4727	0.43750000	1.03125000	-0.12561095
4728	0.53125000	0.96875000	-0.15904203
4729	0.53125000	1.06250000	-0.12355816
4730	0.56250000	1.09375000	-0.12306940
4731	0.53125000	1.56250000	-0.13968719
4732	0.56250000	1.53125000	-0.14291300
4733	0.53125000	1.59375000	-0.13646139
4734	0.59375000	1.53125000	-0.14291300
4735	1.53125000	0.56250000	-0.00175953
4736	1.59375000	0.56250000	0.01104594
4737	1.56250000	0.59375000	-0.00615836
4738	1.53125000	0.46875000	0.02649071
4739	1.53125000	0.43750000	0.03313783
4740	1.46875000	0.53125000	-0.00928641
4741	1.43750000	0.53125000	-0.02121212
4742	1.56250000	1.03125000	-0.04144673
4743	1.53125000	0.96875000	-0.03900293
4744	1.59375000	1.06250000	-0.03333333
4745	1.53125000	0.93750000	-0.02844575
4746	0.53125000	3.56250000	0.09902248
4747	0.56250000	3.59375000	0.09217986
4748	0.46875000	3.53125000	0.10254154
4749	0.43750000	3.53125000	0.09921799
4750	0.40625000	2.96875000	0.11534702
4751	0.46875000	2.90625000	0.10048876
4752	2.93750000	0.46875000	-0.00557185
4753	3.46875000	1.03125000	-0.11045943
4754	3.46875000	1.06250000	-0.11309873
4755	3.40625000	0.93750000	-0.08885630
4756	3.03125000	3.56250000	0.11867058
4757	2.96875000	3.53125000	0.15219941
4758	3.03125000	3.59375000	0.11173021
4759	3.06250000	3.53125000	0.12609971
4760	3.09375000	3.53125000	0.12658847
4761	3.46875000	2.40625000	0.07741935
4762	3.40625000	2.46875000	0.08993157
4763	0.96875000	2.40625000	-0.04740958
4764	0.90625000	2.46875000	-0.03861193
4765	1.53125000	2.46875000	0.00977517
4766	1.43750000	3.03125000	-0.02551320
4767	1.40625000	2.96875000	-0.03910068
4768	1.59375000	3.06250000	-0.06119257
4769	1.56250000	3.09375000	-0.06148583
4770	1.53125000	2.93750000	-0.04301075
4771	1.46875000	2.90625000	-0.05689150
4772	2.40625000	0.96875000	-0.06138807
4773	2.46875000	0.90625000	-0.05943304
4774	2.40625000	1.46875000	-0.09501466
4775	2.46875000	1.40625000	-0.08523949
4776	3.71875000	0.03125000	0.11876833

4777	3.68750000	0.03125000	0.12248289
4778	3.81250000	0.09375000	0.08768328
4779	3.96875000	0.28125000	0.03372434
4780	3.93750000	0.21875000	0.03274682
4781	3.68750000	3.96875000	-0.00791789
4782	0.03125000	1.81250000	-0.14457478
4783	0.03125000	1.71875000	-0.17165200
4784	0.06250000	1.84375000	-0.14125122
4785	3.96875000	2.28125000	-0.09227761
4786	0.06250000	2.78125000	0.02434018
4787	0.28125000	2.96875000	0.12551320
4788	0.31250000	2.96875000	0.11329423
4789	0.18750000	2.90625000	0.10000000
4790	3.93750000	0.65625000	-0.12385142
4791	3.96875000	1.28125000	-0.12766373
4792	3.93750000	1.28125000	-0.12297165
4793	2.78125000	3.96875000	-0.03655914
4794	2.78125000	3.93750000	-0.04271750
4795	3.03125000	3.71875000	0.07243402
4796	3.96875000	2.65625000	-0.05806452
4797	3.93750000	2.71875000	-0.00606061
4798	3.90625000	2.71875000	0.02033236
4799	3.78125000	2.96875000	-0.00195503
4800	3.71875000	3.03125000	0.07859238
4801	3.68750000	3.03125000	0.09589443
4802	3.81250000	3.09375000	0.09472141
4803	3.71875000	2.93750000	0.01173021
4804	3.78125000	2.81250000	-0.08064516
4805	3.84375000	2.81250000	0.04701857
4806	3.71875000	2.68750000	-0.07771261
4807	3.68750000	2.71875000	-0.08465298
4808	3.78125000	2.71875000	-0.09951124
4809	3.84375000	2.78125000	0.04125122
4810	3.78125000	2.84375000	-0.05493646
4811	3.71875000	2.65625000	-0.06510264
4812	3.78125000	2.68750000	-0.08690127
4813	3.65625000	2.71875000	-0.07898338
4814	0.71875000	2.03125000	0.01896383
4815	0.65625000	1.96875000	-0.04115347
4816	0.71875000	1.90625000	-0.05444770
4817	0.68750000	1.71875000	-0.13958944
4818	0.71875000	1.68750000	-0.14291300
4819	0.71875000	1.78125000	-0.16510264
4820	0.65625000	1.71875000	-0.13626588
4821	0.71875000	1.65625000	-0.14291300
4822	0.96875000	2.28125000	-0.07165200
4823	1.03125000	2.84375000	-0.03079179
4824	1.09375000	2.78125000	-0.04916911
4825	1.15625000	2.96875000	0.11847507
4826	1.31250000	3.03125000	0.12658847
4827	1.28125000	2.96875000	0.11260997
4828	1.21875000	2.90625000	0.09951124
4829	2.28125000	0.96875000	-0.03910068
4830	2.28125000	1.34375000	0.04076246
4831	2.34375000	1.28125000	0.05053763
4832	3.21875000	1.15625000	-0.16471163
4833	3.15625000	1.21875000	-0.17057674
4834	3.31250000	1.03125000	-0.12443793
4835	3.28125000	0.96875000	-0.11221896

4836	3.34375000	1.06250000	-0.12434018
4837	3.28125000	0.93750000	-0.09990225
4838	2.84375000	1.03125000	-0.04232649
4839	2.78125000	1.09375000	-0.02844575
4840	2.96875000	1.15625000	-0.16500489
4841	2.90625000	1.21875000	-0.15112414
4842	1.71875000	3.06250000	0.10273705
4843	1.65625000	2.93750000	0.12111437
4844	3.28125000	1.96875000	-0.13284457
4845	3.34375000	2.28125000	-0.07194526
4846	3.28125000	2.34375000	-0.05943304
4847	0.28125000	3.71875000	0.07927664
4848	0.34375000	3.53125000	0.13919844
4849	0.28125000	3.59375000	0.11476051
4850	0.46875000	3.78125000	-0.07106549
4851	0.53125000	3.71875000	-0.07253177
4852	0.46875000	3.68750000	-0.06901271
4853	0.46875000	3.65625000	-0.08475073
4854	0.40625000	3.71875000	-0.00909091
4855	3.68750000	0.21875000	-0.07487781
4856	3.68750000	0.15625000	0.00684262
4857	3.71875000	0.15625000	-0.02209189
4858	3.84375000	0.28125000	-0.08983382
4859	3.81250000	0.21875000	-0.07839687
4860	3.65625000	0.21875000	-0.04477028
4861	3.78125000	0.34375000	-0.12873900
4862	3.53125000	0.21875000	0.05024438
4863	3.46875000	0.28125000	0.03685239
4864	3.81250000	0.53125000	-0.08435973
4865	3.78125000	0.46875000	-0.08289345
4866	3.31250000	3.53125000	0.15073314
4867	3.34375000	3.53125000	0.14672532
4868	3.28125000	3.59375000	0.11564027
4869	3.21875000	3.68750000	0.03665689
4870	3.15625000	3.71875000	0.01671554
4871	3.21875000	3.65625000	0.05386119
4872	0.31250000	2.78125000	-0.12091887
4873	0.31250000	2.84375000	-0.03333333
4874	0.18750000	2.78125000	-0.07243402
4875	0.28125000	2.84375000	-0.03401760
4876	0.34375000	2.78125000	-0.12160313
4877	0.53125000	2.21875000	-0.10009775
4878	0.53125000	1.84375000	-0.13880743
4879	0.59375000	1.78125000	-0.15190616
4880	0.71875000	2.15625000	0.07741935
4881	0.84375000	2.28125000	0.05415445
4882	0.71875000	2.28125000	0.10557185
4883	0.65625000	2.21875000	0.03773216
4884	0.78125000	2.34375000	0.11300098
4885	0.46875000	2.78125000	-0.00078201
4886	0.46875000	2.81250000	0.01710655
4887	0.40625000	2.68750000	-0.05777126
4888	0.78125000	2.46875000	-0.02023460
4889	0.81250000	2.46875000	-0.01857283
4890	0.71875000	2.43750000	-0.02932551
4891	0.68750000	2.40625000	-0.03675464
4892	2.53125000	0.34375000	0.03988270
4893	2.59375000	0.28125000	0.06295210
4894	2.71875000	0.28125000	0.06246334

4895	1.71875000	0.53125000	0.04916911
4896	1.68750000	0.46875000	0.06021505
4897	1.71875000	0.56250000	0.03802542
4898	1.65625000	0.43750000	0.06011730
4899	2.28125000	0.84375000	0.03714565
4900	2.34375000	0.78125000	0.03910068
4901	2.65625000	0.46875000	-0.10791789
4902	2.71875000	0.40625000	-0.08465298
4903	2.46875000	0.78125000	-0.03343109
4904	2.53125000	2.31250000	-0.03000978
4905	2.53125000	2.34375000	-0.02287390
4906	2.59375000	2.28125000	-0.05278592
4907	2.68750000	2.46875000	-0.00635386
4908	2.65625000	2.46875000	-0.00019550
4909	2.71875000	2.43750000	-0.02443793
4910	2.71875000	2.40625000	-0.03636364
4911	1.71875000	2.28125000	0.02932551
4912	1.53125000	2.34375000	-0.05923754
4913	1.59375000	2.28125000	-0.05278592
4914	2.28125000	1.81250000	0.02101662
4915	2.31250000	1.78125000	-0.00136852
4916	2.28125000	1.84375000	0.02306940
4917	2.34375000	1.78125000	-0.02170088
4918	2.18750000	1.46875000	-0.04320626
4919	2.21875000	1.53125000	-0.04731183
4920	2.28125000	1.46875000	-0.09491691
4921	2.15625000	1.43750000	-0.02805474
4922	2.21875000	1.56250000	-0.03626588
4923	1.65625000	2.46875000	0.09169110
4924	1.71875000	2.40625000	0.09814272
4925	2.56250000	1.84375000	-0.14838710
4926	2.43750000	1.78125000	-0.10830890
4927	1.46875000	3.78125000	0.04701857
4928	1.53125000	3.84375000	0.06412512
4929	1.46875000	3.81250000	0.07468231
4930	1.53125000	3.68750000	0.06559140
4931	1.46875000	3.65625000	0.06979472
4932	1.59375000	3.81250000	0.09042033
4933	1.59375000	3.78125000	0.09071359
4934	1.40625000	3.71875000	0.11573803
4935	1.40625000	3.68750000	0.10967742
4936	2.28125000	3.84375000	0.02463343
4937	2.34375000	3.78125000	0.04242424
4938	2.46875000	3.78125000	0.09130010
4939	1.68750000	3.53125000	-0.02619746
4940	1.65625000	3.46875000	-0.01515152
4941	1.84375000	3.53125000	-0.07223851
4942	1.81250000	3.46875000	-0.06031281
4943	1.71875000	3.56250000	-0.01358749
4944	1.78125000	3.59375000	-0.02277615
4945	1.81250000	3.59375000	-0.04457478
4946	1.65625000	3.43750000	0.00850440
4947	1.68750000	3.40625000	0.01124145
4948	1.71875000	3.40625000	-0.00967742
4949	1.46875000	3.28125000	0.15307918
4950	1.53125000	3.34375000	0.09462366
4951	1.43750000	3.28125000	0.14887586
4952	1.43750000	3.15625000	0.14144673
4953	1.56250000	3.34375000	0.11036168

4954	1.56250000	3.21875000	0.10469208
4955	1.59375000	3.28125000	0.12512219
4956	2.34375000	3.53125000	-0.02795699
4957	2.28125000	3.59375000	-0.03929619
4958	1.84375000	3.31250000	-0.07448680
4959	1.81250000	3.34375000	-0.07478006
4960	1.71875000	3.18750000	-0.08181818
4961	1.68750000	3.21875000	-0.08201369
4962	1.68750000	3.15625000	-0.05161290
4963	1.65625000	3.18750000	-0.05180841
4964	1.84375000	3.28125000	-0.05259042
4965	1.81250000	3.21875000	-0.01036168
4966	1.71875000	3.15625000	-0.01065494
4967	1.78125000	3.18750000	0.00997067
4968	1.78125000	3.34375000	-0.05317693
4969	1.71875000	3.31250000	-0.01114370
4970	1.65625000	3.21875000	-0.01104594
4971	1.68750000	3.28125000	0.00928641
4972	3.71875000	2.53125000	-0.06490714
4973	3.53125000	1.71875000	-0.10654936
4974	3.46875000	1.68750000	-0.10606061
4975	3.46875000	1.78125000	-0.13196481
4976	3.56250000	1.71875000	-0.11075269
4977	3.43750000	1.65625000	-0.10977517
4978	3.46875000	1.28125000	-0.14477028
4979	3.46875000	2.18750000	-0.04398827
4980	3.53125000	2.21875000	-0.03607038
4981	3.46875000	2.28125000	-0.05004888
4982	3.43750000	2.15625000	-0.05474096
4983	3.56250000	2.21875000	-0.03890518
4984	0.46875000	1.18750000	-0.12375367
4985	0.53125000	1.21875000	-0.12375367
4986	0.43750000	1.15625000	-0.12375367
4987	0.56250000	1.21875000	-0.12375367
4988	1.53125000	0.71875000	-0.05434995
4989	1.56250000	0.71875000	-0.04828935
4990	1.43750000	0.65625000	-0.06334311
4991	3.15625000	0.71875000	-0.09638319
4992	3.21875000	0.65625000	-0.07194526
4993	2.78125000	3.46875000	0.10909091
4994	3.53125000	2.71875000	0.01837732
4995	3.53125000	2.68750000	0.03343109
4996	3.56250000	2.78125000	0.01104594
4997	3.59375000	2.81250000	0.01876833
4998	3.28125000	2.46875000	-0.01270772
4999	1.21875000	2.78125000	0.05962854
5000	1.28125000	2.84375000	0.06959922
5001	1.34375000	2.78125000	0.05180841
5002	1.53125000	2.81250000	0.07311828
5003	1.46875000	2.78125000	0.05923754
5004	2.46875000	1.28125000	-0.07184751
5005	2.78125000	1.21875000	-0.12570870
5006	0.09375000	1.93750000	-0.13851417
5007	0.09375000	1.90625000	-0.13822092
5008	0.90625000	0.09375000	0.05835777
5009	2.84375000	3.90625000	-0.08113392
5010	3.84375000	2.93750000	0.02981427
5011	3.84375000	2.90625000	0.05415445
5012	1.09375000	2.90625000	0.04076246

5013	2.90625000	1.09375000	-0.10361681
5014	0.59375000	0.40625000	-0.01847507
5015	0.59375000	3.90625000	-0.02287390
5016	0.40625000	3.84375000	0.00772239
5017	0.40625000	3.81250000	-0.00957967
5018	3.65625000	0.09375000	0.09354839
5019	3.59375000	0.15625000	0.07057674
5020	3.56250000	0.15625000	0.07526882
5021	3.90625000	0.34375000	-0.01730205
5022	3.90625000	0.31250000	-0.01955034
5023	3.93750000	0.59375000	-0.03587488
5024	3.84375000	0.40625000	-0.05630499
5025	3.43750000	3.59375000	0.10928641
5026	3.56250000	3.90625000	-0.03743891
5027	0.09375000	1.65625000	-0.16989247
5028	0.65625000	2.09375000	0.00625611
5029	0.59375000	1.90625000	-0.08993157
5030	0.59375000	2.15625000	-0.03343109
5031	2.59375000	0.40625000	-0.03401760
5032	2.56250000	2.40625000	-0.00850440
5033	2.59375000	2.43750000	-0.00127077
5034	2.06250000	1.40625000	0.02795699
5035	2.09375000	1.40625000	0.00752688
5036	2.15625000	1.59375000	0.02815249
5037	2.34375000	1.93750000	0.01260997
5038	2.31250000	1.90625000	0.01886608
5039	2.59375000	1.90625000	-0.13382209
5040	2.43750000	1.84375000	-0.00469208
5041	2.40625000	1.81250000	-0.02336266
5042	1.40625000	3.84375000	0.10508309
5043	2.40625000	3.84375000	0.09208211
5044	1.40625000	3.34375000	0.09560117
5045	3.90625000	1.34375000	-0.11045943
5046	3.90625000	1.31250000	-0.11436950
5047	3.90625000	2.34375000	-0.06744868
5048	3.59375000	1.65625000	-0.09755621
5049	3.59375000	1.68750000	-0.10625611
5050	3.40625000	1.34375000	-0.12189638
5051	3.59375000	1.40625000	-0.13636364
5052	3.40625000	1.56250000	-0.12111437
5053	3.40625000	1.59375000	-0.11730205
5054	3.68750000	2.09375000	-0.11456500
5055	3.59375000	1.90625000	-0.13743891
5056	3.40625000	2.06250000	-0.02913001
5057	3.34375000	1.90625000	-0.12365591
5058	3.40625000	2.09375000	-0.04731183
5059	3.59375000	2.15625000	-0.00518084
5060	3.40625000	1.84375000	-0.12336266
5061	0.40625000	1.06250000	-0.12551320
5062	0.40625000	1.09375000	-0.12463343
5063	0.59375000	0.90625000	-0.13988270
5064	0.59375000	1.15625000	-0.12297165
5065	0.59375000	1.18750000	-0.12336266
5066	0.56250000	1.65625000	-0.13313783
5067	0.65625000	1.56250000	-0.14291300
5068	0.59375000	1.68750000	-0.13304008
5069	0.68750000	1.59375000	-0.14291300
5070	1.65625000	0.59375000	0.01339198
5071	1.59375000	0.65625000	-0.02111437

5072	1.59375000	0.68750000	-0.03167155
5073	1.56250000	0.40625000	0.04652981
5074	1.59375000	0.40625000	0.05327468
5075	1.40625000	0.59375000	-0.05425220
5076	1.68750000	1.09375000	0.03636364
5077	1.59375000	0.90625000	0.04369501
5078	0.59375000	3.65625000	0.05102639
5079	0.59375000	3.68750000	0.01671554
5080	0.40625000	3.56250000	0.06158358
5081	0.40625000	3.59375000	0.02727273
5082	0.34375000	2.90625000	0.04066471
5083	0.40625000	2.84375000	-0.01055718
5084	0.43750000	2.84375000	0.01221896
5085	3.40625000	0.34375000	0.02668622
5086	2.81250000	0.40625000	-0.03118280
5087	3.43750000	1.09375000	-0.11857283
5088	3.34375000	0.90625000	-0.08211144
5089	3.40625000	1.09375000	-0.12140762
5090	3.09375000	3.65625000	0.01339198
5091	3.09375000	3.68750000	0.04516129
5092	2.90625000	3.59375000	0.09393939
5093	3.15625000	3.56250000	0.10840665
5094	3.18750000	3.59375000	0.08973607
5095	2.84375000	3.40625000	0.15014663
5096	3.65625000	3.09375000	0.16617791
5097	3.65625000	2.59375000	0.07605083
5098	3.65625000	2.56250000	0.03861193
5099	3.59375000	2.65625000	0.08719453
5100	3.56250000	2.65625000	0.06783969
5101	3.40625000	2.34375000	0.00273705
5102	3.34375000	2.40625000	0.01524927
5103	0.90625000	2.34375000	0.00361681
5104	0.84375000	2.40625000	0.03743891
5105	1.59375000	2.40625000	0.01622678
5106	1.43750000	3.09375000	0.05640274
5107	1.34375000	2.90625000	0.01524927
5108	1.65625000	3.06250000	0.02082111
5109	1.59375000	2.93750000	0.03910068
5110	1.56250000	3.15625000	0.02003910
5111	1.40625000	2.84375000	-0.00254154
5112	2.34375000	0.90625000	-0.01212121
5113	2.40625000	0.84375000	-0.01016618
5114	2.34375000	1.40625000	-0.02707722
5115	2.40625000	1.34375000	-0.01730205
5116	3.81250000	0.15625000	0.00459433
5117	0.18750000	2.84375000	0.01378299
5118	3.84375000	2.65625000	-0.07702835
5119	3.81250000	2.65625000	-0.07565982
5120	3.68750000	2.84375000	-0.05992180
5121	3.65625000	2.81250000	-0.07996090
5122	0.65625000	1.84375000	-0.10312805
5123	1.15625000	2.84375000	0.02209189
5124	2.84375000	1.15625000	-0.08973607
5125	0.34375000	3.65625000	0.05288368
5126	0.65625000	2.31250000	0.01016618
5127	0.65625000	2.34375000	-0.01700880
5128	2.65625000	0.34375000	-0.01085044
5129	2.65625000	2.31250000	-0.05650049
5130	1.65625000	2.34375000	0.02267840

5131	3.73437500	2.73437500	-0.13812317
5132	3.64062500	2.64062500	0.14868035
5133	3.70312500	2.70312500	-0.04252199
5134	3.67187500	2.67187500	0.05307918
5135	3.73437500	2.67187500	-0.11290323
5136	3.67187500	2.73437500	-0.12678397
5137	3.70312500	2.64062500	-0.01730205
5138	3.64062500	2.70312500	-0.03118280

End of Node Coordinates

Connectivity Matrix of Nodes

Triangular Index	Node 1	Node 2	Node 3
1	83	1512	1510
2	708	2693	1512
3	291	1510	2693
4	1512	2693	1510
5	84	1514	1517
6	292	2695	1514
7	708	1517	2695
8	1514	2695	1517
9	291	2693	2692
10	708	2695	2693
11	292	2692	2695
12	2693	2695	2692
13	27	1208	1206
14	408	2987	1208
15	406	1206	2987
16	1208	2987	1206
17	196	2128	2126
18	709	2992	2128
19	408	2126	2992
20	2128	2992	2126
21	83	1511	1513
22	406	2988	1511
23	709	1513	2988
24	1511	2988	1513
25	408	2992	2987
26	709	2988	2992
27	406	2987	2988
28	2992	2988	2987
29	28	1210	1213
30	410	2997	1210
31	413	1213	2997
32	1210	2997	1213
33	84	1519	1515
34	711	2999	1519
35	410	1515	2999
36	1519	2999	1515
37	196	2127	2129
38	413	3008	2127
39	711	2129	3008
40	2127	3008	2129
41	410	2999	2997
42	711	3008	2999
43	413	2997	3008
44	2999	3008	2997

45	83	1513	1512
46	709	3845	1513
47	708	1512	3845
48	1513	3845	1512
49	196	2129	2128
50	711	3847	2129
51	709	2128	3847
52	2129	3847	2128
53	84	1517	1519
54	708	3846	1517
55	711	1519	3846
56	1517	3846	1519
57	709	3847	3845
58	711	3846	3847
59	708	3845	3846
60	3847	3846	3845
61	120	1696	1694
62	787	2776	1696
63	328	1694	2776
64	1696	2776	1694
65	118	1686	1688
66	326	2772	1686
67	787	1688	2772
68	1686	2772	1688
69	60	1376	1374
70	576	3446	1376
71	574	1374	3446
72	1376	3446	1374
73	197	2135	2133
74	792	3455	2135
75	576	2133	3455
76	2135	3455	2133
77	120	1695	1698
78	574	3448	1695
79	792	1698	3448
80	1695	3448	1698
81	576	3455	3446
82	792	3448	3455
83	574	3446	3448
84	3455	3448	3446
85	27	1207	1209
86	407	2989	1207
87	409	1209	2989
88	1207	2989	1209
89	118	1689	1687
90	788	2990	1689
91	407	1687	2990
92	1689	2990	1687
93	197	2132	2134
94	409	2994	2132
95	788	2134	2994
96	2132	2994	2134
97	407	2990	2989
98	788	2994	2990
99	409	2989	2994
100	2990	2994	2989
101	120	1698	1696
102	792	3924	1698
103	787	1696	3924

104	1698	3924	1696
105	197	2134	2135
106	788	3925	2134
107	792	2135	3925
108	2134	3925	2135
109	118	1688	1689
110	787	3923	1688
111	788	1689	3923
112	1688	3923	1689
113	792	3925	3924
114	788	3923	3925
115	787	3924	3923
116	3925	3923	3924
117	12	1130	1133
118	330	2781	1130
119	333	1133	2781
120	1130	2781	1133
121	122	1709	1706
122	797	2783	1709
123	330	1706	2783
124	1709	2783	1706
125	125	1724	1726
126	333	2792	1724
127	797	1726	2792
128	1724	2792	1726
129	330	2783	2781
130	797	2792	2783
131	333	2781	2792
132	2783	2792	2781
133	28	1215	1211
134	415	3001	1215
135	411	1211	3001
136	1215	3001	1211
137	199	2146	2144
138	799	3012	2146
139	415	2144	3012
140	2146	3012	2144
141	122	1707	1711
142	411	3003	1707
143	799	1711	3003
144	1707	3003	1711
145	415	3012	3001
146	799	3003	3012
147	411	3001	3003
148	3012	3003	3001
149	60	1375	1377
150	575	3450	1375
151	577	1377	3450
152	1375	3450	1377
153	125	1728	1725
154	808	3452	1728
155	575	1725	3452
156	1728	3452	1725
157	199	2145	2147
158	577	3457	2145
159	808	2147	3457
160	2145	3457	2147
161	575	3452	3450
162	808	3457	3452

163	577	3450	3457
164	3452	3457	3450
165	122	1711	1709
166	799	3934	1711
167	797	1709	3934
168	1711	3934	1709
169	199	2147	2146
170	808	3937	2147
171	799	2146	3937
172	2147	3937	2146
173	125	1726	1728
174	797	3935	1726
175	808	1728	3935
176	1726	3935	1728
177	799	3937	3934
178	808	3935	3937
179	797	3934	3935
180	3937	3935	3934
181	27	1209	1208
182	409	2991	1209
183	408	1208	2991
184	1209	2991	1208
185	197	2136	2132
186	997	2995	2136
187	409	2132	2995
188	2136	2995	2132
189	196	2126	2130
190	408	2993	2126
191	997	2130	2993
192	2126	2993	2130
193	409	2995	2991
194	997	2993	2995
195	408	2991	2993
196	2995	2993	2991
197	60	1377	1376
198	577	3454	1377
199	576	1376	3454
200	1377	3454	1376
201	199	2149	2145
202	999	3458	2149
203	577	2145	3458
204	2149	3458	2145
205	197	2133	2137
206	576	3456	2133
207	999	2137	3456
208	2133	3456	2137
209	577	3458	3454
210	999	3456	3458
211	576	3454	3456
212	3458	3456	3454
213	196	2131	2127
214	998	3009	2131
215	413	2127	3009
216	2131	3009	2127
217	199	2144	2148
218	415	3013	2144
219	998	2148	3013
220	2144	3013	2148
221	197	2137	2136

222	999	4134	2137
223	997	2136	4134
224	2137	4134	2136
225	199	2148	2149
226	998	4135	2148
227	999	2149	4135
228	2148	4135	2149
229	196	2130	2131
230	997	4133	2130
231	998	2131	4133
232	2130	4133	2131
233	999	4135	4134
234	998	4133	4135
235	997	4134	4133
236	4135	4133	4134
237	6	1106	1104
238	306	2717	1106
239	304	1104	2717
240	1106	2717	1104
241	98	1580	1578
242	733	2722	1580
243	306	1578	2722
244	1580	2722	1578
245	96	1570	1572
246	304	2718	1570
247	733	1572	2718
248	1570	2718	1572
249	306	2722	2717
250	733	2718	2722
251	304	2717	2718
252	2722	2718	2717
253	42	1276	1274
254	476	3158	1276
255	474	1274	3158
256	1276	3158	1274
257	221	2279	2277
258	738	3167	2279
259	476	2277	3167
260	2279	3167	2277
261	98	1579	1582
262	474	3160	1579
263	738	1582	3160
264	1579	3160	1582
265	476	3167	3158
266	738	3160	3167
267	474	3158	3160
268	3167	3160	3158
269	40	1266	1268
270	466	3140	1266
271	468	1268	3140
272	1266	3140	1268
273	96	1573	1571
274	734	3141	1573
275	466	1571	3141
276	1573	3141	1571
277	221	2276	2278
278	468	3145	2276
279	734	2278	3145
280	2276	3145	2278

281	466	3141	3140
282	734	3145	3141
283	468	3140	3145
284	3141	3145	3140
285	98	1582	1580
286	738	3870	1582
287	733	1580	3870
288	1582	3870	1580
289	221	2278	2279
290	734	3871	2278
291	738	2279	3871
292	2278	3871	2279
293	96	1572	1573
294	733	3869	1572
295	734	1573	3869
296	1572	3869	1573
297	738	3871	3870
298	734	3869	3871
299	733	3870	3869
300	3871	3869	3870
301	22	1180	1178
302	380	2906	1180
303	378	1178	2906
304	1180	2906	1178
305	172	1984	1982
306	922	2915	1984
307	380	1982	2915
308	1984	2915	1982
309	170	1970	1972
310	378	2908	1970
311	922	1972	2908
312	1970	2908	1972
313	380	2915	2906
314	922	2908	2915
315	378	2906	2908
316	2915	2908	2906
317	172	1983	1986
318	581	3470	1983
319	931	1986	3470
320	1983	3470	1986
321	42	1275	1278
322	475	3162	1275
323	478	1278	3162
324	1275	3162	1278
325	170	1974	1971
326	924	3164	1974
327	475	1971	3164
328	1974	3164	1971
329	226	2306	2308
330	478	3172	2306
331	924	2308	3172
332	2306	3172	2308
333	475	3164	3162
334	924	3172	3164
335	478	3162	3172
336	3164	3172	3162
337	172	1986	1984
338	931	4059	1986
339	922	1984	4059

340	1986	4059	1984
341	226	2308	2309
342	924	4062	2308
343	931	2309	4062
344	2308	4062	2309
345	170	1972	1974
346	922	4058	1972
347	924	1974	4058
348	1972	4058	1974
349	931	4062	4059
350	924	4058	4062
351	922	4059	4058
352	4062	4058	4059
353	11	1127	1129
354	327	2773	1127
355	329	1129	2773
356	1127	2773	1129
357	119	1692	1690
358	789	2774	1692
359	327	1690	2774
360	1692	2774	1690
361	121	1700	1702
362	329	2778	1700
363	789	1702	2778
364	1700	2778	1702
365	327	2774	2773
366	789	2778	2774
367	329	2773	2778
368	2774	2778	2773
369	40	1269	1267
370	469	3142	1269
371	467	1267	3142
372	1269	3142	1267
373	222	2284	2282
374	790	3147	2284
375	469	2282	3147
376	2284	3147	2282
377	119	1691	1693
378	467	3143	1691
379	790	1693	3143
380	1691	3143	1693
381	469	3147	3142
382	790	3143	3147
383	467	3142	3143
384	3147	3143	3142
385	61	1380	1382
386	580	3464	1380
387	582	1382	3464
388	1380	3464	1382
389	121	1704	1701
390	794	3466	1704
391	580	1701	3466
392	1704	3466	1701
393	222	2283	2285
394	582	3473	2283
395	794	2285	3473
396	2283	3473	2285
397	580	3466	3464
398	794	3473	3466

399	582	3464	3473
400	3466	3473	3464
401	119	1693	1692
402	790	3926	1693
403	789	1692	3926
404	1693	3926	1692
405	222	2285	2284
406	794	3928	2285
407	790	2284	3928
408	2285	3928	2284
409	121	1702	1704
410	789	3927	1702
411	794	1704	3927
412	1702	3927	1704
413	790	3928	3926
414	794	3927	3928
415	789	3926	3927
416	3928	3927	3926
417	42	1278	1276
418	478	3166	1278
419	476	1276	3166
420	1278	3166	1276
421	226	2310	2306
422	1022	3173	2310
423	478	2306	3173
424	2310	3173	2306
425	221	2277	2281
426	476	3168	2277
427	1022	2281	3168
428	2277	3168	2281
429	478	3173	3166
430	1022	3168	3173
431	476	3166	3168
432	3173	3168	3166
433	222	2287	2283
434	1023	3474	2287
435	582	2283	3474
436	2287	3474	2283
437	40	1268	1269
438	468	3144	1268
439	469	1269	3144
440	1268	3144	1269
441	221	2280	2276
442	1021	3146	2280
443	468	2276	3146
444	2280	3146	2276
445	222	2282	2286
446	469	3148	2282
447	1021	2286	3148
448	2282	3148	2286
449	468	3146	3144
450	1021	3148	3146
451	469	3144	3148
452	3146	3148	3144
453	226	2311	2310
454	1023	4159	2311
455	1022	2310	4159
456	2311	4159	2310
457	222	2286	2287

458	1021	4158	2286
459	1023	2287	4158
460	2286	4158	2287
461	221	2281	2280
462	1022	4157	2281
463	1021	2280	4157
464	2281	4157	2280
465	1023	4158	4159
466	1021	4157	4158
467	1022	4159	4157
468	4158	4157	4159
469	7	1108	1111
470	308	2727	1108
471	311	1111	2727
472	1108	2727	1111
473	100	1593	1590
474	743	2729	1593
475	308	1590	2729
476	1593	2729	1590
477	103	1608	1610
478	311	2738	1608
479	743	1610	2738
480	1608	2738	1610
481	308	2729	2727
482	743	2738	2729
483	311	2727	2738
484	2729	2738	2727
485	44	1289	1286
486	489	3195	1289
487	486	1286	3195
488	1289	3195	1286
489	231	2338	2336
490	745	3206	2338
491	489	2336	3206
492	2338	3206	2336
493	100	1591	1595
494	486	3197	1591
495	745	1595	3197
496	1591	3197	1595
497	489	3206	3195
498	745	3197	3206
499	486	3195	3197
500	3206	3197	3195
501	47	1304	1306
502	504	3248	1304
503	506	1306	3248
504	1304	3248	1306
505	103	1612	1609
506	754	3250	1612
507	504	1609	3250
508	1612	3250	1609
509	231	2337	2339
510	506	3257	2337
511	754	2339	3257
512	2337	3257	2339
513	504	3250	3248
514	754	3257	3250
515	506	3248	3257
516	3250	3257	3248

517	100	1595	1593
518	745	3880	1595
519	743	1593	3880
520	1595	3880	1593
521	231	2339	2338
522	754	3883	2339
523	745	2338	3883
524	2339	3883	2338
525	103	1610	1612
526	743	3881	1610
527	754	1612	3881
528	1610	3881	1612
529	745	3883	3880
530	754	3881	3883
531	743	3880	3881
532	3883	3881	3880
533	12	1135	1131
534	335	2785	1135
535	331	1131	2785
536	1135	2785	1131
537	127	1738	1736
538	801	2796	1738
539	335	1736	2796
540	1738	2796	1736
541	123	1712	1715
542	331	2787	1712
543	801	1715	2787
544	1712	2787	1715
545	335	2796	2785
546	801	2787	2796
547	331	2785	2787
548	2796	2787	2785
549	63	1394	1392
550	594	3500	1394
551	592	1392	3500
552	1394	3500	1392
553	233	2351	2349
554	812	3509	2351
555	594	2349	3509
556	2351	3509	2349
557	127	1737	1740
558	592	3502	1737
559	812	1740	3502
560	1737	3502	1740
561	594	3509	3500
562	812	3502	3509
563	592	3500	3502
564	3509	3502	3500
565	44	1287	1291
566	487	3199	1287
567	491	1291	3199
568	1287	3199	1291
569	123	1717	1713
570	803	3201	1717
571	487	1713	3201
572	1717	3201	1713
573	233	2348	2350
574	491	3210	2348
575	803	2350	3210

576	2348	3210	2350
577	487	3201	3199
578	803	3210	3201
579	491	3199	3210
580	3201	3210	3199
581	127	1740	1738
582	812	3941	1740
583	801	1738	3941
584	1740	3941	1738
585	233	2350	2351
586	803	3943	2350
587	812	2351	3943
588	2350	3943	2351
589	123	1715	1717
590	801	3940	1715
591	803	1717	3940
592	1715	3940	1717
593	812	3943	3941
594	803	3940	3943
595	801	3941	3940
596	3943	3940	3941
597	22	1179	1181
598	379	2910	1179
599	381	1181	2910
600	1179	2910	1181
601	171	1978	1976
602	926	2912	1978
603	379	1976	2912
604	1978	2912	1976
605	173	1988	1990
606	381	2917	1988
607	926	1990	2917
608	1988	2917	1990
609	379	2912	2910
610	926	2917	2912
611	381	2910	2917
612	2912	2917	2910
613	47	1308	1305
614	508	3252	1308
615	505	1305	3252
616	1308	3252	1305
617	242	2404	2402
618	928	3262	2404
619	508	2402	3262
620	2404	3262	2402
621	171	1977	1980
622	505	3254	1977
623	928	1980	3254
624	1977	3254	1980
625	508	3262	3252
626	928	3254	3262
627	505	3252	3254
628	3262	3254	3252
629	173	1992	1989
630	933	3506	1992
631	593	1989	3506
632	1992	3506	1989
633	242	2403	2405
634	595	3511	2403

635	933	2405	3511
636	2403	3511	2405
637	171	1980	1978
638	928	4064	1980
639	926	1978	4064
640	1980	4064	1978
641	242	2405	2404
642	933	4068	2405
643	928	2404	4068
644	2405	4068	2404
645	173	1990	1992
646	926	4065	1990
647	933	1992	4065
648	1990	4065	1992
649	928	4068	4064
650	933	4065	4068
651	926	4064	4065
652	4068	4065	4064
653	44	1291	1289
654	491	3205	1291
655	489	1289	3205
656	1291	3205	1289
657	233	2352	2348
658	1032	3211	2352
659	491	2348	3211
660	2352	3211	2348
661	231	2336	2340
662	489	3207	2336
663	1032	2340	3207
664	2336	3207	2340
665	491	3211	3205
666	1032	3207	3211
667	489	3205	3207
668	3211	3207	3205
669	63	1395	1394
670	595	3508	1395
671	594	1394	3508
672	1395	3508	1394
673	242	2407	2403
674	1035	3512	2407
675	595	2403	3512
676	2407	3512	2403
677	233	2349	2353
678	594	3510	2349
679	1035	2353	3510
680	2349	3510	2353
681	595	3512	3508
682	1035	3510	3512
683	594	3508	3510
684	3512	3510	3508
685	47	1306	1308
686	506	3256	1306
687	508	1308	3256
688	1306	3256	1308
689	231	2341	2337
690	1033	3258	2341
691	506	2337	3258
692	2341	3258	2337
693	242	2402	2406

694	508	3263	2402
695	1033	2406	3263
696	2402	3263	2406
697	506	3258	3256
698	1033	3263	3258
699	508	3256	3263
700	3258	3263	3256
701	233	2353	2352
702	1035	4170	2353
703	1032	2352	4170
704	2353	4170	2352
705	242	2406	2407
706	1033	4171	2406
707	1035	2407	4171
708	2406	4171	2407
709	231	2340	2341
710	1032	4169	2340
711	1033	2341	4169
712	2340	4169	2341
713	1035	4171	4170
714	1033	4169	4171
715	1032	4170	4169
716	4171	4169	4170
717	11	1129	1128
718	329	2775	1129
719	328	1128	2775
720	1129	2775	1128
721	121	1703	1700
722	791	2779	1703
723	329	1700	2779
724	1703	2779	1700
725	120	1694	1697
726	328	2777	1694
727	791	1697	2777
728	1694	2777	1697
729	329	2779	2775
730	791	2777	2779
731	328	2775	2777
732	2779	2777	2775
733	61	1384	1380
734	584	3465	1384
735	580	1380	3465
736	1384	3465	1380
737	269	2567	2565
738	795	3478	2567
739	584	2565	3478
740	2567	3478	2565
741	121	1701	1705
742	580	3467	1701
743	795	1705	3467
744	1701	3467	1705
745	584	3478	3465
746	795	3467	3478
747	580	3465	3467
748	3478	3467	3465
749	60	1374	1378
750	574	3447	1374
751	578	1378	3447
752	1374	3447	1378

753	120	1699	1695
754	793	3449	1699
755	574	1695	3449
756	1699	3449	1695
757	269	2564	2566
758	578	3460	2564
759	793	2566	3460
760	2564	3460	2566
761	574	3449	3447
762	793	3460	3449
763	578	3447	3460
764	3449	3460	3447
765	121	1705	1703
766	795	3930	1705
767	791	1703	3930
768	1705	3930	1703
769	269	2566	2567
770	793	3931	2566
771	795	2567	3931
772	2566	3931	2567
773	120	1697	1699
774	791	3929	1697
775	793	1699	3929
776	1697	3929	1699
777	795	3931	3930
778	793	3929	3931
779	791	3930	3929
780	3931	3929	3930
781	22	1181	1180
782	381	2914	1181
783	380	1180	2914
784	1181	2914	1180
785	173	1991	1988
786	930	2918	1991
787	381	1988	2918
788	1991	2918	1988
789	172	1982	1985
790	380	2916	1982
791	930	1985	2916
792	1982	2916	1985
793	381	2918	2914
794	930	2916	2918
795	380	2914	2916
796	2918	2916	2914
797	271	2579	2577
798	934	3516	2579
799	597	2577	3516
800	2579	3516	2577
801	173	1989	1993
802	593	3507	1989
803	934	1993	3507
804	1989	3507	1993
805	172	1987	1983
806	932	3471	1987
807	581	1983	3471
808	1987	3471	1983
809	173	1993	1991
810	934	4071	1993
811	930	1991	4071

812	1993	4071	1991
813	271	2578	2579
814	932	4072	2578
815	934	2579	4072
816	2578	4072	2579
817	172	1985	1987
818	930	4070	1985
819	932	1987	4070
820	1985	4070	1987
821	934	4072	4071
822	932	4070	4072
823	930	4071	4070
824	4072	4070	4071
825	12	1133	1135
826	333	2791	1133
827	335	1135	2791
828	1133	2791	1135
829	125	1727	1724
830	807	2793	1727
831	333	1724	2793
832	1727	2793	1724
833	127	1736	1739
834	335	2797	1736
835	807	1739	2797
836	1736	2797	1739
837	333	2793	2791
838	807	2797	2793
839	335	2791	2797
840	2793	2797	2791
841	60	1379	1375
842	579	3451	1379
843	575	1375	3451
844	1379	3451	1375
845	270	2572	2570
846	809	3462	2572
847	579	2570	3462
848	2572	3462	2570
849	125	1725	1729
850	575	3453	1725
851	809	1729	3453
852	1725	3453	1729
853	579	3462	3451
854	809	3453	3462
855	575	3451	3453
856	3462	3453	3451
857	63	1392	1396
858	592	3501	1392
859	596	1396	3501
860	1392	3501	1396
861	127	1741	1737
862	813	3503	1741
863	592	1737	3503
864	1741	3503	1737
865	270	2571	2573
866	596	3514	2571
867	813	2573	3514
868	2571	3514	2573
869	592	3503	3501
870	813	3514	3503

871	596	3501	3514
872	3503	3514	3501
873	125	1729	1727
874	809	3947	1729
875	807	1727	3947
876	1729	3947	1727
877	270	2573	2572
878	813	3949	2573
879	809	2572	3949
880	2573	3949	2572
881	127	1739	1741
882	807	3948	1739
883	813	1741	3948
884	1739	3948	1741
885	809	3949	3947
886	813	3948	3949
887	807	3947	3948
888	3949	3948	3947
889	269	2565	2569
890	584	3479	2565
891	1070	2569	3479
892	2565	3479	2569
893	63	1396	1397
894	596	3513	1396
895	597	1397	3513
896	1396	3513	1397
897	270	2575	2571
898	1071	3515	2575
899	596	2571	3515
900	2575	3515	2571
901	271	2577	2581
902	597	3517	2577
903	1071	2581	3517
904	2577	3517	2581
905	596	3515	3513
906	1071	3517	3515
907	597	3513	3517
908	3515	3517	3513
909	60	1378	1379
910	578	3459	1378
911	579	1379	3459
912	1378	3459	1379
913	269	2568	2564
914	1069	3461	2568
915	578	2564	3461
916	2568	3461	2564
917	270	2570	2574
918	579	3463	2570
919	1069	2574	3463
920	2570	3463	2574
921	578	3461	3459
922	1069	3463	3461
923	579	3459	3463
924	3461	3463	3459
925	271	2581	2580
926	1071	4207	2581
927	1070	2580	4207
928	2581	4207	2580
929	270	2574	2575

930	1069	4206	2574
931	1071	2575	4206
932	2574	4206	2575
933	269	2569	2568
934	1070	4205	2569
935	1069	2568	4205
936	2569	4205	2568
937	1071	4206	4207
938	1069	4205	4206
939	1070	4207	4205
940	4206	4205	4207
941	3	1096	1095
942	296	2699	1096
943	295	1095	2699
944	1096	2699	1095
945	88	1534	1532
946	715	2701	1534
947	296	1532	2701
948	1534	2701	1532
949	87	1528	1530
950	295	2700	1528
951	715	1530	2700
952	1528	2700	1530
953	296	2701	2699
954	715	2700	2701
955	295	2699	2700
956	2701	2700	2699
957	31	1224	1226
958	424	3032	1224
959	426	1226	3032
960	1224	3032	1226
961	16	1150	1148
962	350	2825	1150
963	348	1148	2825
964	1150	2825	1148
965	142	1812	1810
966	841	2830	1812
967	350	1810	2830
968	1812	2830	1810
969	140	1802	1804
970	348	2826	1802
971	841	1804	2826
972	1802	2826	1804
973	350	2830	2825
974	841	2826	2830
975	348	2825	2826
976	2830	2826	2825
977	67	1419	1417
978	619	3576	1419
979	617	1417	3576
980	1419	3576	1417
981	205	2183	2181
982	846	3583	2183
983	619	2181	3583
984	2183	3583	2181
985	142	1811	1814
986	617	3578	1811
987	846	1814	3578
988	1811	3578	1814

989	619	3583	3576
990	846	3578	3583
991	617	3576	3578
992	3583	3578	3576
993	142	1814	1812
994	846	3978	1814
995	841	1812	3978
996	1814	3978	1812
997	205	2182	2183
998	842	3979	2182
999	846	2183	3979
1000	2182	3979	2183
1001	140	1804	1805
1002	841	3977	1804
1003	842	1805	3977
1004	1804	3977	1805
1005	846	3979	3978
1006	842	3977	3979
1007	841	3978	3977
1008	3979	3977	3978
1009	15	1144	1146
1010	344	2816	1144
1011	346	1146	2816
1012	1144	2816	1146
1013	136	1784	1782
1014	832	2817	1784
1015	344	1782	2817
1016	1784	2817	1782
1017	138	1790	1792
1018	346	2821	1790
1019	832	1792	2821
1020	1790	2821	1792
1021	344	2817	2816
1022	832	2821	2817
1023	346	2816	2821
1024	2817	2821	2816
1025	31	1227	1225
1026	427	3034	1227
1027	425	1225	3034
1028	1227	3034	1225
1029	204	2176	2174
1030	833	3039	2176
1031	427	2174	3039
1032	2176	3039	2174
1033	136	1783	1785
1034	425	3035	1783
1035	833	1785	3035
1036	1783	3035	1785
1037	427	3039	3034
1038	833	3035	3039
1039	425	3034	3035
1040	3039	3035	3034
1041	67	1416	1418
1042	616	3572	1416
1043	618	1418	3572
1044	1416	3572	1418
1045	138	1794	1791
1046	837	3574	1794
1047	616	1791	3574

1048	1794	3574	1791
1049	136	1785	1784
1050	833	3968	1785
1051	832	1784	3968
1052	1785	3968	1784
1053	204	2177	2176
1054	837	3970	2177
1055	833	2176	3970
1056	2177	3970	2176
1057	138	1792	1794
1058	832	3969	1792
1059	837	1794	3969
1060	1792	3969	1794
1061	833	3970	3968
1062	837	3969	3970
1063	832	3968	3969
1064	3970	3969	3968
1065	67	1418	1419
1066	618	3580	1418
1067	619	1419	3580
1068	1418	3580	1419
1069	204	2179	2175
1070	1005	3582	2179
1071	618	2175	3582
1072	2179	3582	2175
1073	205	2181	2185
1074	619	3584	2181
1075	1005	2185	3584
1076	2181	3584	2185
1077	618	3582	3580
1078	1005	3584	3582
1079	619	3580	3584
1080	3582	3584	3580
1081	31	1226	1227
1082	426	3036	1226
1083	427	1227	3036
1084	1226	3036	1227
1085	205	2185	2184
1086	1005	4141	2185
1087	1004	2184	4141
1088	2185	4141	2184
1089	204	2178	2179
1090	1003	4140	2178
1091	1005	2179	4140
1092	2178	4140	2179
1093	203	2173	2172
1094	1004	4139	2173
1095	1003	2172	4139
1096	2173	4139	2172
1097	1005	4140	4141
1098	1003	4139	4140
1099	1004	4141	4139
1100	4140	4139	4141
1101	9	1120	1118
1102	320	2753	1120
1103	318	1118	2753
1104	1120	2753	1118
1105	112	1656	1654
1106	769	2758	1656

1107	320	1654	2758
1108	1656	2758	1654
1109	110	1646	1648
1110	318	2754	1646
1111	769	1648	2754
1112	1646	2754	1648
1113	320	2758	2753
1114	769	2754	2758
1115	318	2753	2754
1116	2758	2754	2753
1117	56	1352	1350
1118	552	3374	1352
1119	550	1350	3374
1120	1352	3374	1350
1121	257	2495	2493
1122	774	3383	2495
1123	552	2493	3383
1124	2495	3383	2493
1125	112	1655	1658
1126	550	3376	1655
1127	774	1658	3376
1128	1655	3376	1658
1129	552	3383	3374
1130	774	3376	3383
1131	550	3374	3376
1132	3383	3376	3374
1133	54	1342	1344
1134	542	3356	1342
1135	544	1344	3356
1136	1342	3356	1344
1137	110	1649	1647
1138	770	3357	1649
1139	542	1647	3357
1140	1649	3357	1647
1141	257	2492	2494
1142	544	3361	2492
1143	770	2494	3361
1144	2492	3361	2494
1145	542	3357	3356
1146	770	3361	3357
1147	544	3356	3361
1148	3357	3361	3356
1149	112	1658	1656
1150	774	3906	1658
1151	769	1656	3906
1152	1658	3906	1656
1153	257	2494	2495
1154	770	3907	2494
1155	774	2495	3907
1156	2494	3907	2495
1157	110	1648	1649
1158	769	3905	1648
1159	770	1649	3905
1160	1648	3905	1649
1161	774	3907	3906
1162	770	3905	3907
1163	769	3906	3905
1164	3907	3905	3906
1165	179	2026	2024

1166	944	2935	2026
1167	387	2024	2935
1168	2026	2935	2024
1169	387	2935	2928
1170	944	2930	2935
1171	385	2928	2930
1172	2935	2930	2928
1173	262	2525	2523
1174	951	3619	2525
1175	631	2523	3619
1176	2525	3619	2523
1177	56	1351	1354
1178	551	3378	1351
1179	554	1354	3378
1180	1351	3378	1354
1181	177	2016	2013
1182	946	3380	2016
1183	551	2013	3380
1184	2016	3380	2013
1185	179	2028	2026
1186	951	4083	2028
1187	944	2026	4083
1188	2028	4083	2026
1189	262	2524	2525
1190	946	4086	2524
1191	951	2525	4086
1192	2524	4086	2525
1193	177	2014	2016
1194	944	4082	2014
1195	946	2016	4082
1196	2014	4082	2016
1197	951	4086	4083
1198	946	4082	4086
1199	944	4083	4082
1200	4086	4082	4083
1201	16	1149	1151
1202	349	2827	1149
1203	351	1151	2827
1204	1149	2827	1151
1205	141	1808	1806
1206	843	2828	1808
1207	349	1806	2828
1208	1808	2828	1806
1209	143	1816	1818
1210	351	2832	1816
1211	843	1818	2832
1212	1816	2832	1818
1213	349	2828	2827
1214	843	2832	2828
1215	351	2827	2832
1216	2828	2832	2827
1217	258	2500	2498
1218	844	3363	2500
1219	545	2498	3363
1220	2500	3363	2498
1221	69	1428	1430
1222	628	3608	1428
1223	630	1430	3608
1224	1428	3608	1430

1225	143	1820	1817
1226	848	3610	1820
1227	628	1817	3610
1228	1820	3610	1817
1229	258	2499	2501
1230	630	3617	2499
1231	848	2501	3617
1232	2499	3617	2501
1233	628	3610	3608
1234	848	3617	3610
1235	630	3608	3617
1236	3610	3617	3608
1237	141	1809	1808
1238	844	3980	1809
1239	843	1808	3980
1240	1809	3980	1808
1241	258	2501	2500
1242	848	3982	2501
1243	844	2500	3982
1244	2501	3982	2500
1245	143	1818	1820
1246	843	3981	1818
1247	848	1820	3981
1248	1818	3981	1820
1249	844	3982	3980
1250	848	3981	3982
1251	843	3980	3981
1252	3982	3981	3980
1253	262	2526	2522
1254	1058	3389	2526
1255	554	2522	3389
1256	2526	3389	2522
1257	257	2493	2497
1258	552	3384	2493
1259	1058	2497	3384
1260	2493	3384	2497
1261	69	1430	1431
1262	630	3616	1430
1263	631	1431	3616
1264	1430	3616	1431
1265	258	2503	2499
1266	1059	3618	2503
1267	630	2499	3618
1268	2503	3618	2499
1269	262	2523	2527
1270	631	3620	2523
1271	1059	2527	3620
1272	2523	3620	2527
1273	630	3618	3616
1274	1059	3620	3618
1275	631	3616	3620
1276	3618	3620	3616
1277	54	1344	1345
1278	544	3360	1344
1279	545	1345	3360
1280	1344	3360	1345
1281	257	2496	2492
1282	1057	3362	2496
1283	544	2492	3362

1284	2496	3362	2492
1285	258	2498	2502
1286	545	3364	2498
1287	1057	2502	3364
1288	2498	3364	2502
1289	544	3362	3360
1290	1057	3364	3362
1291	545	3360	3364
1292	3362	3364	3360
1293	262	2527	2526
1294	1059	4195	2527
1295	1058	2526	4195
1296	2527	4195	2526
1297	258	2502	2503
1298	1057	4194	2502
1299	1059	2503	4194
1300	2502	4194	2503
1301	257	2497	2496
1302	1058	4193	2497
1303	1057	2496	4193
1304	2497	4193	2496
1305	1059	4194	4195
1306	1057	4193	4194
1307	1058	4195	4193
1308	4194	4193	4195
1309	6	1105	1107
1310	305	2719	1105
1311	307	1107	2719
1312	1105	2719	1107
1313	97	1576	1574
1314	735	2720	1576
1315	305	1574	2720
1316	1576	2720	1574
1317	99	1584	1586
1318	307	2724	1584
1319	735	1586	2724
1320	1584	2724	1586
1321	305	2720	2719
1322	735	2724	2720
1323	307	2719	2724
1324	2720	2724	2719
1325	41	1272	1270
1326	472	3149	1272
1327	470	1270	3149
1328	1272	3149	1270
1329	223	2290	2288
1330	736	3154	2290
1331	472	2288	3154
1332	2290	3154	2288
1333	97	1575	1577
1334	470	3150	1575
1335	736	1577	3150
1336	1575	3150	1577
1337	472	3154	3149
1338	736	3150	3154
1339	470	3149	3150
1340	3154	3150	3149
1341	43	1280	1282
1342	480	3176	1280

1343	482	1282	3176
1344	1280	3176	1282
1345	99	1588	1585
1346	740	3178	1588
1347	480	1585	3178
1348	1588	3178	1585
1349	223	2289	2291
1350	482	3185	2289
1351	740	2291	3185
1352	2289	3185	2291
1353	480	3178	3176
1354	740	3185	3178
1355	482	3176	3185
1356	3178	3185	3176
1357	97	1577	1576
1358	736	3872	1577
1359	735	1576	3872
1360	1577	3872	1576
1361	223	2291	2290
1362	740	3874	2291
1363	736	2290	3874
1364	2291	3874	2290
1365	99	1586	1588
1366	735	3873	1586
1367	740	1588	3873
1368	1586	3873	1588
1369	736	3874	3872
1370	740	3873	3874
1371	735	3872	3873
1372	3874	3873	3872
1373	15	1147	1145
1374	347	2818	1147
1375	345	1145	2818
1376	1147	2818	1145
1377	139	1798	1796
1378	834	2823	1798
1379	347	1796	2823
1380	1798	2823	1796
1381	137	1786	1788
1382	345	2819	1786
1383	834	1788	2819
1384	1786	2819	1788
1385	347	2823	2818
1386	834	2819	2823
1387	345	2818	2819
1388	2823	2819	2818
1389	68	1424	1422
1390	624	3590	1424
1391	622	1422	3590
1392	1424	3590	1422
1393	224	2297	2295
1394	839	3599	2297
1395	624	2295	3599
1396	2297	3599	2295
1397	139	1797	1800
1398	622	3592	1797
1399	839	1800	3592
1400	1797	3592	1800
1401	624	3599	3590

1402	839	3592	3599
1403	622	3590	3592
1404	3599	3592	3590
1405	41	1271	1273
1406	471	3151	1271
1407	473	1273	3151
1408	1271	3151	1273
1409	137	1789	1787
1410	835	3152	1789
1411	471	1787	3152
1412	1789	3152	1787
1413	224	2294	2296
1414	473	3156	2294
1415	835	2296	3156
1416	2294	3156	2296
1417	471	3152	3151
1418	835	3156	3152
1419	473	3151	3156
1420	3152	3156	3151
1421	139	1800	1798
1422	839	3972	1800
1423	834	1798	3972
1424	1800	3972	1798
1425	224	2296	2297
1426	835	3973	2296
1427	839	2297	3973
1428	2296	3973	2297
1429	137	1788	1789
1430	834	3971	1788
1431	835	1789	3971
1432	1788	3971	1789
1433	839	3973	3972
1434	835	3971	3973
1435	834	3972	3971
1436	3973	3971	3972
1437	23	1184	1186
1438	384	2924	1184
1439	386	1186	2924
1440	1184	2924	1186
1441	176	2008	2006
1442	940	2926	2008
1443	384	2006	2926
1444	2008	2926	2006
1445	178	2018	2020
1446	386	2933	2018
1447	940	2020	2933
1448	2018	2933	2020
1449	384	2926	2924
1450	940	2933	2926
1451	386	2924	2933
1452	2926	2933	2924
1453	43	1284	1281
1454	484	3180	1284
1455	481	1281	3180
1456	1284	3180	1281
1457	228	2320	2318
1458	942	3190	2320
1459	484	2318	3190
1460	2320	3190	2318

1461	176	2007	2010
1462	481	3182	2007
1463	942	2010	3182
1464	2007	3182	2010
1465	484	3190	3180
1466	942	3182	3190
1467	481	3180	3182
1468	3190	3182	3180
1469	68	1423	1425
1470	623	3594	1423
1471	625	1425	3594
1472	1423	3594	1425
1473	178	2022	2019
1474	949	3596	2022
1475	623	2019	3596
1476	2022	3596	2019
1477	228	2319	2321
1478	625	3601	2319
1479	949	2321	3601
1480	2319	3601	2321
1481	623	3596	3594
1482	949	3601	3596
1483	625	3594	3601
1484	3596	3601	3594
1485	176	2010	2008
1486	942	4076	2010
1487	940	2008	4076
1488	2010	4076	2008
1489	228	2321	2320
1490	949	4080	2321
1491	942	2320	4080
1492	2321	4080	2320
1493	178	2020	2022
1494	940	4077	2020
1495	949	2022	4077
1496	2020	4077	2022
1497	942	4080	4076
1498	949	4077	4080
1499	940	4076	4077
1500	4080	4077	4076
1501	41	1273	1272
1502	473	3153	1273
1503	472	1272	3153
1504	1273	3153	1272
1505	224	2298	2294
1506	1024	3157	2298
1507	473	2294	3157
1508	2298	3157	2294
1509	223	2288	2292
1510	472	3155	2288
1511	1024	2292	3155
1512	2288	3155	2292
1513	473	3157	3153
1514	1024	3155	3157
1515	472	3153	3155
1516	3157	3155	3153
1517	228	2323	2319
1518	1026	3602	2323
1519	625	2319	3602

1520	2323	3602	2319
1521	224	2295	2299
1522	624	3600	2295
1523	1026	2299	3600
1524	2295	3600	2299
1525	16	1151	1150
1526	351	2829	1151
1527	350	1150	2829
1528	1151	2829	1150
1529	143	1819	1816
1530	845	2833	1819
1531	351	1816	2833
1532	1819	2833	1816
1533	142	1810	1813
1534	350	2831	1810
1535	845	1813	2831
1536	1810	2831	1813
1537	351	2833	2829
1538	845	2831	2833
1539	350	2829	2831
1540	2833	2831	2829
1541	69	1432	1428
1542	632	3609	1432
1543	628	1428	3609
1544	1432	3609	1428
1545	276	2609	2607
1546	849	3622	2609
1547	632	2607	3622
1548	2609	3622	2607
1549	143	1817	1821
1550	628	3611	1817
1551	849	1821	3611
1552	1817	3611	1821
1553	632	3622	3609
1554	849	3611	3622
1555	628	3609	3611
1556	3622	3611	3609
1557	67	1417	1421
1558	617	3577	1417
1559	621	1421	3577
1560	1417	3577	1421
1561	142	1815	1811
1562	847	3579	1815
1563	617	1811	3579
1564	1815	3579	1811
1565	276	2606	2608
1566	621	3588	2606
1567	847	2608	3588
1568	2606	3588	2608
1569	617	3579	3577
1570	847	3588	3579
1571	621	3577	3588
1572	3579	3588	3577
1573	143	1821	1819
1574	849	3984	1821
1575	845	1819	3984
1576	1821	3984	1819
1577	276	2608	2609
1578	847	3985	2608

1579	849	2609	3985
1580	2608	3985	2609
1581	142	1813	1815
1582	845	3983	1813
1583	847	1815	3983
1584	1813	3983	1815
1585	849	3985	3984
1586	847	3983	3985
1587	845	3984	3983
1588	3985	3983	3984
1589	23	1186	1187
1590	386	2932	1186
1591	387	1187	2932
1592	1186	2932	1187
1593	178	2021	2018
1594	948	2934	2021
1595	386	2018	2934
1596	2021	2934	2018
1597	179	2024	2027
1598	387	2936	2024
1599	948	2027	2936
1600	2024	2936	2027
1601	386	2934	2932
1602	948	2936	2934
1603	387	2932	2936
1604	2934	2936	2932
1605	68	1427	1423
1606	627	3595	1427
1607	623	1423	3595
1608	1427	3595	1423
1609	178	2019	2023
1610	623	3597	2019
1611	950	2023	3597
1612	2019	3597	2023
1613	627	3606	3595
1614	950	3597	3606
1615	623	3595	3597
1616	3606	3597	3595
1617	277	2613	2615
1618	633	3624	2613
1619	952	2615	3624
1620	2613	3624	2615
1621	178	2023	2021
1622	950	4088	2023
1623	948	2021	4088
1624	2023	4088	2021
1625	277	2615	2614
1626	952	4090	2615
1627	950	2614	4090
1628	2615	4090	2614
1629	179	2027	2029
1630	948	4089	2027
1631	952	2029	4089
1632	2027	4089	2029
1633	950	4090	4088
1634	952	4089	4090
1635	948	4088	4089
1636	4090	4089	4088
1637	15	1146	1147

1638	346	2820	1146
1639	347	1147	2820
1640	1146	2820	1147
1641	138	1793	1790
1642	836	2822	1793
1643	346	1790	2822
1644	1793	2822	1790
1645	139	1796	1799
1646	347	2824	1796
1647	836	1799	2824
1648	1796	2824	1799
1649	346	2822	2820
1650	836	2824	2822
1651	347	2820	2824
1652	2822	2824	2820
1653	67	1420	1416
1654	620	3573	1420
1655	616	1416	3573
1656	1420	3573	1416
1657	275	2602	2600
1658	838	3586	2602
1659	620	2600	3586
1660	2602	3586	2600
1661	138	1791	1795
1662	616	3575	1791
1663	838	1795	3575
1664	1791	3575	1795
1665	620	3586	3573
1666	838	3575	3586
1667	616	3573	3575
1668	3586	3575	3573
1669	68	1422	1426
1670	622	3591	1422
1671	626	1426	3591
1672	1422	3591	1426
1673	139	1801	1797
1674	840	3593	1801
1675	622	1797	3593
1676	1801	3593	1797
1677	275	2601	2603
1678	626	3604	2601
1679	840	2603	3604
1680	2601	3604	2603
1681	622	3593	3591
1682	840	3604	3593
1683	626	3591	3604
1684	3593	3604	3591
1685	138	1795	1793
1686	838	3974	1795
1687	836	1793	3974
1688	1795	3974	1793
1689	275	2603	2602
1690	840	3976	2603
1691	838	2602	3976
1692	2603	3976	2602
1693	139	1799	1801
1694	836	3975	1799
1695	840	1801	3975
1696	1799	3975	1801

1697	838	3976	3974
1698	840	3975	3976
1699	836	3974	3975
1700	3976	3975	3974
1701	69	1433	1432
1702	633	3621	1433
1703	632	1432	3621
1704	1433	3621	1432
1705	277	2617	2613
1706	1077	3625	2617
1707	633	2613	3625
1708	2617	3625	2613
1709	276	2607	2611
1710	632	3623	2607
1711	1077	2611	3623
1712	2607	3623	2611
1713	633	3625	3621
1714	1077	3623	3625
1715	632	3621	3623
1716	3625	3623	3621
1717	68	1426	1427
1718	626	3603	1426
1719	627	1427	3603
1720	1426	3603	1427
1721	275	2605	2601
1722	1076	3605	2605
1723	626	2601	3605
1724	2605	3605	2601
1725	626	3605	3603
1726	1076	3607	3605
1727	627	3603	3607
1728	3605	3607	3603
1729	67	1421	1420
1730	621	3585	1421
1731	620	1420	3585
1732	1421	3585	1420
1733	276	2610	2606
1734	1075	3589	2610
1735	621	2606	3589
1736	2610	3589	2606
1737	275	2600	2604
1738	620	3587	2600
1739	1075	2604	3587
1740	2600	3587	2604
1741	621	3589	3585
1742	1075	3587	3589
1743	620	3585	3587
1744	3589	3587	3585
1745	277	2616	2617
1746	1076	4213	2616
1747	1077	2617	4213
1748	2616	4213	2617
1749	275	2604	2605
1750	1075	4211	2604
1751	1076	2605	4211
1752	2604	4211	2605
1753	276	2611	2610
1754	1077	4212	2611
1755	1075	2610	4212

1756	2611	4212	2610
1757	1076	4211	4213
1758	1075	4212	4211
1759	1077	4213	4212
1760	4211	4212	4213
1761	4	1097	1099
1762	297	2703	1097
1763	299	1099	2703
1764	1097	2703	1099
1765	89	1539	1536
1766	719	2705	1539
1767	297	1536	2705
1768	1539	2705	1536
1769	91	1546	1548
1770	299	2707	1546
1771	719	1548	2707
1772	1546	2707	1548
1773	297	2705	2703
1774	719	2707	2705
1775	299	2703	2707
1776	2705	2707	2703
1777	33	1235	1232
1778	435	3051	1235
1779	432	1232	3051
1780	1235	3051	1232
1781	207	2194	2192
1782	721	3062	2194
1783	435	2192	3062
1784	2194	3062	2192
1785	89	1537	1541
1786	432	3053	1537
1787	721	1541	3053
1788	1537	3053	1541
1789	435	3062	3051
1790	721	3053	3062
1791	432	3051	3053
1792	3062	3053	3051
1793	35	1242	1244
1794	442	3077	1242
1795	444	1244	3077
1796	1242	3077	1244
1797	91	1549	1547
1798	723	3078	1549
1799	442	1547	3078
1800	1549	3078	1547
1801	207	2193	2195
1802	444	3082	2193
1803	723	2195	3082
1804	2193	3082	2195
1805	442	3078	3077
1806	723	3082	3078
1807	444	3077	3082
1808	3078	3082	3077
1809	89	1541	1539
1810	721	3856	1541
1811	719	1539	3856
1812	1541	3856	1539
1813	207	2195	2194
1814	723	3859	2195

1815	721	2194	3859
1816	2195	3859	2194
1817	91	1548	1549
1818	719	3857	1548
1819	723	1549	3857
1820	1548	3857	1549
1821	721	3859	3856
1822	723	3857	3859
1823	719	3856	3857
1824	3859	3857	3856
1825	17	1155	1152
1826	355	2835	1155
1827	352	1152	2835
1828	1155	2835	1152
1829	147	1842	1840
1830	851	2846	1842
1831	355	1840	2846
1832	1842	2846	1840
1833	144	1822	1825
1834	352	2837	1822
1835	851	1825	2837
1836	1822	2837	1825
1837	355	2846	2835
1838	851	2837	2846
1839	352	2835	2837
1840	2846	2837	2835
1841	71	1442	1440
1842	642	3644	1442
1843	640	1440	3644
1844	1442	3644	1440
1845	209	2207	2205
1846	862	3653	2207
1847	642	2205	3653
1848	2207	3653	2205
1849	147	1841	1844
1850	640	3646	1841
1851	862	1844	3646
1852	1841	3646	1844
1853	642	3653	3644
1854	862	3646	3653
1855	640	3644	3646
1856	3653	3646	3644
1857	33	1233	1237
1858	433	3055	1233
1859	437	1237	3055
1860	1233	3055	1237
1861	144	1827	1823
1862	853	3057	1827
1863	433	1823	3057
1864	1827	3057	1823
1865	209	2204	2206
1866	437	3066	2204
1867	853	2206	3066
1868	2204	3066	2206
1869	433	3057	3055
1870	853	3066	3057
1871	437	3055	3066
1872	3057	3066	3055
1873	147	1844	1842

1874	862	3989	1844
1875	851	1842	3989
1876	1844	3989	1842
1877	209	2206	2207
1878	853	3991	2206
1879	862	2207	3991
1880	2206	3991	2207
1881	144	1825	1827
1882	851	3988	1825
1883	853	1827	3988
1884	1825	3988	1827
1885	862	3991	3989
1886	853	3988	3991
1887	851	3989	3988
1888	3991	3988	3989
1889	19	1162	1164
1890	362	2861	1162
1891	364	1164	2861
1892	1162	2861	1164
1893	154	1880	1878
1894	877	2862	1880
1895	362	1878	2862
1896	1880	2862	1878
1897	156	1886	1888
1898	364	2866	1886
1899	877	1888	2866
1900	1886	2866	1888
1901	362	2862	2861
1902	877	2866	2862
1903	364	2861	2866
1904	2862	2866	2861
1905	35	1245	1243
1906	445	3079	1245
1907	443	1243	3079
1908	1245	3079	1243
1909	211	2218	2216
1910	878	3084	2218
1911	445	2216	3084
1912	2218	3084	2216
1913	154	1879	1881
1914	443	3080	1879
1915	878	1881	3080
1916	1879	3080	1881
1917	445	3084	3079
1918	878	3080	3084
1919	443	3079	3080
1920	3084	3080	3079
1921	71	1441	1443
1922	641	3648	1441
1923	643	1443	3648
1924	1441	3648	1443
1925	211	2217	2219
1926	643	3655	2217
1927	882	2219	3655
1928	2217	3655	2219
1929	154	1881	1880
1930	878	4013	1881
1931	877	1880	4013
1932	1881	4013	1880

1933	211	2219	2218
1934	882	4015	2219
1935	878	2218	4015
1936	2219	4015	2218
1937	878	4015	4013
1938	882	4014	4015
1939	877	4013	4014
1940	4015	4014	4013
1941	33	1237	1235
1942	437	3061	1237
1943	435	1235	3061
1944	1237	3061	1235
1945	209	2208	2204
1946	1008	3067	2208
1947	437	2204	3067
1948	2208	3067	2204
1949	207	2192	2196
1950	435	3063	2192
1951	1008	2196	3063
1952	2192	3063	2196
1953	437	3067	3061
1954	1008	3063	3067
1955	435	3061	3063
1956	3067	3063	3061
1957	71	1443	1442
1958	643	3652	1443
1959	642	1442	3652
1960	1443	3652	1442
1961	211	2221	2217
1962	1011	3656	2221
1963	643	2217	3656
1964	2221	3656	2217
1965	209	2205	2209
1966	642	3654	2205
1967	1011	2209	3654
1968	2205	3654	2209
1969	643	3656	3652
1970	1011	3654	3656
1971	642	3652	3654
1972	3656	3654	3652
1973	35	1244	1245
1974	444	3081	1244
1975	445	1245	3081
1976	1244	3081	1245
1977	207	2197	2193
1978	1009	3083	2197
1979	444	2193	3083
1980	2197	3083	2193
1981	211	2216	2220
1982	445	3085	2216
1983	1009	2220	3085
1984	2216	3085	2220
1985	444	3083	3081
1986	1009	3085	3083
1987	445	3081	3085
1988	3083	3085	3081
1989	209	2209	2208
1990	1011	4146	2209
1991	1008	2208	4146

1992	2209	4146	2208
1993	211	2220	2221
1994	1009	4147	2220
1995	1011	2221	4147
1996	2220	4147	2221
1997	207	2196	2197
1998	1008	4145	2196
1999	1009	2197	4145
2000	2196	4145	2197
2001	1011	4147	4146
2002	1009	4145	4147
2003	1008	4146	4145
2004	4147	4145	4146
2005	7	1113	1109
2006	313	2731	1113
2007	309	1109	2731
2008	1113	2731	1109
2009	105	1622	1620
2010	747	2742	1622
2011	313	1620	2742
2012	1622	2742	1620
2013	101	1596	1599
2014	309	2733	1596
2015	747	1599	2733
2016	1596	2733	1599
2017	313	2742	2731
2018	747	2733	2742
2019	309	2731	2733
2020	2742	2733	2731
2021	49	1318	1316
2022	518	3284	1318
2023	516	1316	3284
2024	1318	3284	1316
2025	235	2363	2361
2026	758	3293	2363
2027	518	2361	3293
2028	2363	3293	2361
2029	105	1621	1624
2030	516	3286	1621
2031	758	1624	3286
2032	1621	3286	1624
2033	518	3293	3284
2034	758	3286	3293
2035	516	3284	3286
2036	3293	3286	3284
2037	45	1292	1295
2038	492	3213	1292
2039	495	1295	3213
2040	1292	3213	1295
2041	101	1601	1597
2042	749	3215	1601
2043	492	1597	3215
2044	1601	3215	1597
2045	235	2360	2362
2046	495	3224	2360
2047	749	2362	3224
2048	2360	3224	2362
2049	492	3215	3213
2050	749	3224	3215

2051	495	3213	3224
2052	3215	3224	3213
2053	105	1624	1622
2054	758	3887	1624
2055	747	1622	3887
2056	1624	3887	1622
2057	235	2362	2363
2058	749	3889	2362
2059	758	2363	3889
2060	2362	3889	2363
2061	101	1599	1601
2062	747	3886	1599
2063	749	1601	3886
2064	1599	3886	1601
2065	758	3889	3887
2066	749	3886	3889
2067	747	3887	3886
2068	3889	3886	3887
2069	25	1198	1196
2070	398	2960	1198
2071	396	1196	2960
2072	1198	2960	1196
2073	190	2092	2090
2074	976	2969	2092
2075	398	2090	2969
2076	2092	2969	2090
2077	188	2078	2080
2078	396	2962	2078
2079	976	2080	2962
2080	2078	2962	2080
2081	398	2969	2960
2082	976	2962	2969
2083	396	2960	2962
2084	2969	2962	2960
2085	73	1455	1453
2086	655	3684	1455
2087	653	1453	3684
2088	1455	3684	1453
2089	246	2429	2427
2090	985	3691	2429
2091	655	2427	3691
2092	2429	3691	2427
2093	190	2091	2094
2094	653	3686	2091
2095	985	2094	3686
2096	2091	3686	2094
2097	655	3691	3684
2098	985	3686	3691
2099	653	3684	3686
2100	3691	3686	3684
2101	49	1317	1320
2102	517	3288	1317
2103	520	1320	3288
2104	1317	3288	1320
2105	188	2082	2079
2106	978	3290	2082
2107	517	2079	3290
2108	2082	3290	2079
2109	246	2426	2428

2110	520	3298	2426
2111	978	2428	3298
2112	2426	3298	2428
2113	517	3290	3288
2114	978	3298	3290
2115	520	3288	3298
2116	3290	3298	3288
2117	190	2094	2092
2118	985	4113	2094
2119	976	2092	4113
2120	2094	4113	2092
2121	246	2428	2429
2122	978	4116	2428
2123	985	2429	4116
2124	2428	4116	2429
2125	188	2080	2082
2126	976	4112	2080
2127	978	2082	4112
2128	2080	4112	2082
2129	985	4116	4113
2130	978	4112	4116
2131	976	4113	4112
2132	4116	4112	4113
2133	17	1153	1157
2134	353	2839	1153
2135	357	1157	2839
2136	1153	2839	1157
2137	145	1831	1828
2138	855	2841	1831
2139	353	1828	2841
2140	1831	2841	1828
2141	149	1852	1854
2142	357	2850	1852
2143	855	1854	2850
2144	1852	2850	1854
2145	353	2841	2839
2146	855	2850	2841
2147	357	2839	2850
2148	2841	2850	2839
2149	45	1297	1293
2150	497	3217	1297
2151	493	1293	3217
2152	1297	3217	1293
2153	237	2374	2372
2154	857	3228	2374
2155	497	2372	3228
2156	2374	3228	2372
2157	145	1829	1833
2158	493	3219	1829
2159	857	1833	3219
2160	1829	3219	1833
2161	497	3228	3217
2162	857	3219	3228
2163	493	3217	3219
2164	3228	3219	3217
2165	73	1452	1454
2166	652	3680	1452
2167	654	1454	3680
2168	1452	3680	1454

2169	149	1856	1853
2170	866	3682	1856
2171	652	1853	3682
2172	1856	3682	1853
2173	237	2373	2375
2174	654	3689	2373
2175	866	2375	3689
2176	2373	3689	2375
2177	652	3682	3680
2178	866	3689	3682
2179	654	3680	3689
2180	3682	3689	3680
2181	145	1833	1831
2182	857	3994	1833
2183	855	1831	3994
2184	1833	3994	1831
2185	237	2375	2374
2186	866	3997	2375
2187	857	2374	3997
2188	2375	3997	2374
2189	149	1854	1856
2190	855	3995	1854
2191	866	1856	3995
2192	1854	3995	1856
2193	857	3997	3994
2194	866	3995	3997
2195	855	3994	3995
2196	3997	3995	3994
2197	49	1320	1318
2198	520	3292	1320
2199	518	1318	3292
2200	1320	3292	1318
2201	246	2430	2426
2202	1039	3299	2430
2203	520	2426	3299
2204	2430	3299	2426
2205	235	2361	2365
2206	518	3294	2361
2207	1039	2365	3294
2208	2361	3294	2365
2209	520	3299	3292
2210	1039	3294	3299
2211	518	3292	3294
2212	3299	3294	3292
2213	73	1454	1455
2214	654	3688	1454
2215	655	1455	3688
2216	1454	3688	1455
2217	237	2377	2373
2218	1041	3690	2377
2219	654	2373	3690
2220	2377	3690	2373
2221	246	2427	2431
2222	655	3692	2427
2223	1041	2431	3692
2224	2427	3692	2431
2225	654	3690	3688
2226	1041	3692	3690
2227	655	3688	3692

2228	3690	3692	3688
2229	45	1295	1297
2230	495	3223	1295
2231	497	1297	3223
2232	1295	3223	1297
2233	246	2431	2430
2234	1041	4177	2431
2235	1039	2430	4177
2236	2431	4177	2430
2237	9	1119	1121
2238	319	2755	1119
2239	321	1121	2755
2240	1119	2755	1121
2241	111	1652	1650
2242	771	2756	1652
2243	319	1650	2756
2244	1652	2756	1650
2245	113	1660	1662
2246	321	2760	1660
2247	771	1662	2760
2248	1660	2760	1662
2249	319	2756	2755
2250	771	2760	2756
2251	321	2755	2760
2252	2756	2760	2755
2253	55	1348	1346
2254	548	3365	1348
2255	546	1346	3365
2256	1348	3365	1346
2257	259	2506	2504
2258	772	3370	2506
2259	548	2504	3370
2260	2506	3370	2504
2261	113	1664	1661
2262	776	3394	1664
2263	556	1661	3394
2264	1664	3394	1661
2265	259	2505	2507
2266	558	3401	2505
2267	776	2507	3401
2268	2505	3401	2507
2269	111	1653	1652
2270	772	3908	1653
2271	771	1652	3908
2272	1653	3908	1652
2273	259	2507	2506
2274	776	3910	2507
2275	772	2506	3910
2276	2507	3910	2506
2277	113	1662	1664
2278	771	3909	1662
2279	776	1664	3909
2280	1662	3909	1664
2281	772	3910	3908
2282	776	3909	3910
2283	771	3908	3909
2284	3910	3909	3908
2285	19	1165	1163
2286	365	2863	1165

2287	363	1163	2863
2288	1165	2863	1163
2289	155	1882	1884
2290	363	2864	1882
2291	879	1884	2864
2292	1882	2864	1884
2293	365	2868	2863
2294	879	2864	2868
2295	363	2863	2864
2296	2868	2864	2863
2297	75	1466	1464
2298	666	3716	1466
2299	664	1464	3716
2300	1466	3716	1464
2301	55	1347	1349
2302	547	3367	1347
2303	549	1349	3367
2304	1347	3367	1349
2305	260	2510	2512
2306	549	3372	2510
2307	880	2512	3372
2308	2510	3372	2512
2309	547	3368	3367
2310	880	3372	3368
2311	549	3367	3372
2312	3368	3372	3367
2313	260	2512	2513
2314	880	4018	2512
2315	884	2513	4018
2316	2512	4018	2513
2317	25	1197	1199
2318	397	2964	1197
2319	399	1199	2964
2320	1197	2964	1199
2321	189	2086	2084
2322	980	2966	2086
2323	397	2084	2966
2324	2086	2966	2084
2325	191	2096	2098
2326	399	2971	2096
2327	980	2098	2971
2328	2096	2971	2098
2329	397	2966	2964
2330	980	2971	2966
2331	399	2964	2971
2332	2966	2971	2964
2333	189	2085	2088
2334	557	3398	2085
2335	982	2088	3398
2336	2085	3398	2088
2337	189	2088	2086
2338	982	4118	2088
2339	980	2086	4118
2340	2088	4118	2086
2341	264	2537	2536
2342	987	4122	2537
2343	982	2536	4122
2344	2537	4122	2536
2345	191	2098	2100

2346	980	4119	2098
2347	987	2100	4119
2348	2098	4119	2100
2349	982	4122	4118
2350	987	4119	4122
2351	980	4118	4119
2352	4122	4119	4118
2353	55	1349	1348
2354	549	3369	1349
2355	548	1348	3369
2356	1349	3369	1348
2357	260	2514	2510
2358	1060	3373	2514
2359	549	2510	3373
2360	2514	3373	2510
2361	259	2504	2508
2362	548	3371	2504
2363	1060	2508	3371
2364	2504	3371	2508
2365	549	3373	3369
2366	1060	3371	3373
2367	548	3369	3371
2368	3373	3371	3369
2369	75	1467	1466
2370	667	3724	1467
2371	666	1466	3724
2372	1467	3724	1466
2373	264	2539	2535
2374	1062	3728	2539
2375	667	2535	3728
2376	2539	3728	2535
2377	260	2511	2515
2378	666	3726	2511
2379	1062	2515	3726
2380	2511	3726	2515
2381	667	3728	3724
2382	1062	3726	3728
2383	666	3724	3726
2384	3728	3726	3724
2385	259	2509	2505
2386	1061	3402	2509
2387	558	2505	3402
2388	2509	3402	2505
2389	260	2515	2514
2390	1062	4197	2515
2391	1060	2514	4197
2392	2515	4197	2514
2393	264	2538	2539
2394	1061	4198	2538
2395	1062	2539	4198
2396	2538	4198	2539
2397	259	2508	2509
2398	1060	4196	2508
2399	1061	2509	4196
2400	2508	4196	2509
2401	1062	4198	4197
2402	1061	4196	4198
2403	1060	4197	4196
2404	4198	4196	4197

2405	17	1157	1155
2406	357	2845	1157
2407	355	1155	2845
2408	1157	2845	1155
2409	149	1855	1852
2410	861	2851	1855
2411	357	1852	2851
2412	1855	2851	1852
2413	147	1840	1843
2414	355	2847	1840
2415	861	1843	2847
2416	1840	2847	1843
2417	357	2851	2845
2418	861	2847	2851
2419	355	2845	2847
2420	2851	2847	2845
2421	73	1456	1452
2422	656	3681	1456
2423	652	1452	3681
2424	1456	3681	1452
2425	280	2633	2631
2426	867	3694	2633
2427	656	2631	3694
2428	2633	3694	2631
2429	149	1853	1857
2430	652	3683	1853
2431	867	1857	3683
2432	1853	3683	1857
2433	656	3694	3681
2434	867	3683	3694
2435	652	3681	3683
2436	3694	3683	3681
2437	71	1440	1444
2438	640	3645	1440
2439	644	1444	3645
2440	1440	3645	1444
2441	147	1845	1841
2442	863	3647	1845
2443	640	1841	3647
2444	1845	3647	1841
2445	280	2630	2632
2446	644	3658	2630
2447	863	2632	3658
2448	2630	3658	2632
2449	640	3647	3645
2450	863	3658	3647
2451	644	3645	3658
2452	3647	3658	3645
2453	149	1857	1855
2454	867	4002	1857
2455	861	1855	4002
2456	1857	4002	1855
2457	280	2632	2633
2458	863	4003	2632
2459	867	2633	4003
2460	2632	4003	2633
2461	147	1843	1845
2462	861	4001	1843
2463	863	1845	4001

2464	1843	4001	1845
2465	867	4003	4002
2466	863	4001	4003
2467	861	4002	4001
2468	4003	4001	4002
2469	25	1199	1198
2470	399	2968	1199
2471	398	1198	2968
2472	1199	2968	1198
2473	191	2099	2096
2474	984	2972	2099
2475	399	2096	2972
2476	2099	2972	2096
2477	190	2090	2093
2478	398	2970	2090
2479	984	2093	2970
2480	2090	2970	2093
2481	399	2972	2968
2482	984	2970	2972
2483	398	2968	2970
2484	2972	2970	2968
2485	73	1453	1457
2486	653	3685	1453
2487	657	1457	3685
2488	1453	3685	1457
2489	190	2095	2091
2490	986	3687	2095
2491	653	2091	3687
2492	2095	3687	2091
2493	283	2648	2650
2494	657	3696	2648
2495	986	2650	3696
2496	2648	3696	2650
2497	653	3687	3685
2498	986	3696	3687
2499	657	3685	3696
2500	3687	3696	3685
2501	191	2101	2099
2502	988	4125	2101
2503	984	2099	4125
2504	2101	4125	2099
2505	283	2650	2651
2506	986	4126	2650
2507	988	2651	4126
2508	2650	4126	2651
2509	190	2093	2095
2510	984	4124	2093
2511	986	2095	4124
2512	2093	4124	2095
2513	988	4126	4125
2514	986	4124	4126
2515	984	4125	4124
2516	4126	4124	4125
2517	19	1164	1165
2518	364	2865	1164
2519	365	1165	2865
2520	1164	2865	1165
2521	71	1445	1441
2522	645	3649	1445

2523	641	1441	3649
2524	1445	3649	1441
2525	281	2638	2636
2526	883	3660	2638
2527	645	2636	3660
2528	2638	3660	2636
2529	156	1887	1891
2530	641	3651	1887
2531	883	1891	3651
2532	1887	3651	1891
2533	645	3660	3649
2534	883	3651	3660
2535	641	3649	3651
2536	3660	3651	3649
2537	75	1464	1468
2538	664	3717	1464
2539	668	1468	3717
2540	1464	3717	1468
2541	157	1895	1897
2542	881	4020	1895
2543	885	1897	4020
2544	1895	4020	1897
2545	73	1457	1456
2546	657	3693	1457
2547	656	1456	3693
2548	1457	3693	1456
2549	283	2652	2648
2550	1082	3697	2652
2551	657	2648	3697
2552	2652	3697	2648
2553	280	2631	2635
2554	656	3695	2631
2555	1082	2635	3695
2556	2631	3695	2635
2557	657	3697	3693
2558	1082	3695	3697
2559	656	3693	3695
2560	3697	3695	3693
2561	75	1468	1469
2562	668	3729	1468
2563	669	1469	3729
2564	1468	3729	1469
2565	281	2641	2637
2566	1083	3731	2641
2567	668	2637	3731
2568	2641	3731	2637
2569	283	2649	2653
2570	669	3733	2649
2571	1083	2653	3733
2572	2649	3733	2653
2573	668	3731	3729
2574	1083	3733	3731
2575	669	3729	3733
2576	3731	3733	3729
2577	71	1444	1445
2578	644	3657	1444
2579	645	1445	3657
2580	1444	3657	1445
2581	280	2634	2630

2582	1081	3659	2634
2583	644	2630	3659
2584	2634	3659	2630
2585	281	2636	2640
2586	645	3661	2636
2587	1081	2640	3661
2588	2636	3661	2640
2589	644	3659	3657
2590	1081	3661	3659
2591	645	3657	3661
2592	3659	3661	3657
2593	283	2653	2652
2594	1083	4219	2653
2595	1082	2652	4219
2596	2653	4219	2652
2597	281	2640	2641
2598	1081	4218	2640
2599	1083	2641	4218
2600	2640	4218	2641
2601	280	2635	2634
2602	1082	4217	2635
2603	1081	2634	4217
2604	2635	4217	2634
2605	1083	4218	4219
2606	1081	4217	4218
2607	1082	4219	4217
2608	4218	4217	4219
2609	6	1107	1106
2610	307	2721	1107
2611	306	1106	2721
2612	1107	2721	1106
2613	99	1587	1584
2614	737	2725	1587
2615	307	1584	2725
2616	1587	2725	1584
2617	98	1578	1581
2618	306	2723	1578
2619	737	1581	2723
2620	1578	2723	1581
2621	307	2725	2721
2622	737	2723	2725
2623	306	2721	2723
2624	2725	2723	2721
2625	43	1283	1280
2626	483	3177	1283
2627	480	1280	3177
2628	1283	3177	1280
2629	225	2303	2301
2630	741	3188	2303
2631	483	2301	3188
2632	2303	3188	2301
2633	99	1585	1589
2634	480	3179	1585
2635	741	1589	3179
2636	1585	3179	1589
2637	483	3188	3177
2638	741	3179	3188
2639	480	3177	3179
2640	3188	3179	3177

2641	42	1274	1277
2642	474	3159	1274
2643	477	1277	3159
2644	1274	3159	1277
2645	98	1583	1579
2646	739	3161	1583
2647	474	1579	3161
2648	1583	3161	1579
2649	225	2300	2302
2650	477	3170	2300
2651	739	2302	3170
2652	2300	3170	2302
2653	474	3161	3159
2654	739	3170	3161
2655	477	3159	3170
2656	3161	3170	3159
2657	99	1589	1587
2658	741	3876	1589
2659	737	1587	3876
2660	1589	3876	1587
2661	225	2302	2303
2662	739	3877	2302
2663	741	2303	3877
2664	2302	3877	2303
2665	98	1581	1583
2666	737	3875	1581
2667	739	1583	3875
2668	1581	3875	1583
2669	741	3877	3876
2670	739	3875	3877
2671	737	3876	3875
2672	3877	3875	3876
2673	23	1188	1184
2674	388	2925	1188
2675	384	1184	2925
2676	1188	2925	1184
2677	180	2032	2030
2678	941	2938	2032
2679	388	2030	2938
2680	2032	2938	2030
2681	176	2006	2009
2682	384	2927	2006
2683	941	2009	2927
2684	2006	2927	2009
2685	388	2938	2925
2686	941	2927	2938
2687	384	2925	2927
2688	2938	2927	2925
2689	79	1491	1489
2690	691	3792	1491
2691	689	1489	3792
2692	1491	3792	1489
2693	229	2327	2325
2694	954	3799	2327
2695	691	2325	3799
2696	2327	3799	2325
2697	180	2031	2034
2698	689	3794	2031
2699	954	2034	3794

2700	2031	3794	2034
2701	691	3799	3792
2702	954	3794	3799
2703	689	3792	3794
2704	3799	3794	3792
2705	43	1281	1285
2706	481	3181	1281
2707	485	1285	3181
2708	1281	3181	1285
2709	176	2011	2007
2710	943	3183	2011
2711	481	2007	3183
2712	2011	3183	2007
2713	229	2324	2326
2714	485	3192	2324
2715	943	2326	3192
2716	2324	3192	2326
2717	481	3183	3181
2718	943	3192	3183
2719	485	3181	3192
2720	3183	3192	3181
2721	180	2034	2032
2722	954	4079	2034
2723	941	2032	4079
2724	2034	4079	2032
2725	229	2326	2327
2726	943	4081	2326
2727	954	2327	4081
2728	2326	4081	2327
2729	176	2009	2011
2730	941	4078	2009
2731	943	2011	4078
2732	2009	4078	2011
2733	954	4081	4079
2734	943	4078	4081
2735	941	4079	4078
2736	4081	4078	4079
2737	22	1178	1182
2738	378	2907	1178
2739	382	1182	2907
2740	1178	2907	1182
2741	170	1973	1970
2742	923	2909	1973
2743	378	1970	2909
2744	1973	2909	1970
2745	174	1994	1996
2746	382	2920	1994
2747	923	1996	2920
2748	1994	2920	1996
2749	378	2909	2907
2750	923	2920	2909
2751	382	2907	2920
2752	2909	2920	2907
2753	42	1279	1275
2754	479	3163	1279
2755	475	1275	3163
2756	1279	3163	1275
2757	227	2314	2312
2758	925	3174	2314

2759	479	2312	3174
2760	2314	3174	2312
2761	170	1971	1975
2762	475	3165	1971
2763	925	1975	3165
2764	1971	3165	1975
2765	479	3174	3163
2766	925	3165	3174
2767	475	3163	3165
2768	3174	3165	3163
2769	170	1975	1973
2770	925	4060	1975
2771	923	1973	4060
2772	1975	4060	1973
2773	227	2315	2314
2774	936	4063	2315
2775	925	2314	4063
2776	2315	4063	2314
2777	174	1996	1998
2778	923	4061	1996
2779	936	1998	4061
2780	1996	4061	1998
2781	925	4063	4060
2782	936	4061	4063
2783	923	4060	4061
2784	4063	4061	4060
2785	43	1285	1283
2786	485	3187	1285
2787	483	1283	3187
2788	1285	3187	1283
2789	229	2328	2324
2790	1028	3193	2328
2791	485	2324	3193
2792	2328	3193	2324
2793	225	2301	2305
2794	483	3189	2301
2795	1028	2305	3189
2796	2301	3189	2305
2797	485	3193	3187
2798	1028	3189	3193
2799	483	3187	3189
2800	3193	3189	3187
2801	42	1277	1279
2802	477	3169	1277
2803	479	1279	3169
2804	1277	3169	1279
2805	225	2304	2300
2806	1027	3171	2304
2807	477	2300	3171
2808	2304	3171	2300
2809	227	2312	2316
2810	479	3175	2312
2811	1027	2316	3175
2812	2312	3175	2316
2813	477	3171	3169
2814	1027	3175	3171
2815	479	3169	3175
2816	3171	3175	3169
2817	229	2329	2328

2818	1029	4165	2329
2819	1028	2328	4165
2820	2329	4165	2328
2821	227	2316	2317
2822	1027	4164	2316
2823	1029	2317	4164
2824	2316	4164	2317
2825	225	2305	2304
2826	1028	4163	2305
2827	1027	2304	4163
2828	2305	4163	2304
2829	1029	4164	4165
2830	1027	4163	4164
2831	1028	4165	4163
2832	4164	4163	4165
2833	9	1121	1120
2834	321	2757	1121
2835	320	1120	2757
2836	1121	2757	1120
2837	113	1663	1660
2838	773	2761	1663
2839	321	1660	2761
2840	1663	2761	1660
2841	112	1654	1657
2842	320	2759	1654
2843	773	1657	2759
2844	1654	2759	1657
2845	321	2761	2757
2846	773	2759	2761
2847	320	2757	2759
2848	2761	2759	2757
2849	261	2519	2517
2850	777	3404	2519
2851	559	2517	3404
2852	2519	3404	2517
2853	113	1661	1665
2854	556	3395	1661
2855	777	1665	3395
2856	1661	3395	1665
2857	112	1659	1655
2858	775	3377	1659
2859	550	1655	3377
2860	1659	3377	1655
2861	113	1665	1663
2862	777	3912	1665
2863	773	1663	3912
2864	1665	3912	1663
2865	261	2518	2519
2866	775	3913	2518
2867	777	2519	3913
2868	2518	3913	2519
2869	112	1657	1659
2870	773	3911	1657
2871	775	1659	3911
2872	1657	3911	1659
2873	777	3913	3912
2874	775	3911	3913
2875	773	3912	3911
2876	3913	3911	3912

2877	25	1201	1197
2878	401	2965	1201
2879	397	1197	2965
2880	1201	2965	1197
2881	193	2110	2108
2882	981	2976	2110
2883	401	2108	2976
2884	2110	2976	2108
2885	189	2084	2087
2886	397	2967	2084
2887	981	2087	2967
2888	2084	2967	2087
2889	401	2976	2965
2890	981	2967	2976
2891	397	2965	2967
2892	2976	2967	2965
2893	81	1503	1501
2894	703	3828	1503
2895	701	1501	3828
2896	1503	3828	1501
2897	265	2543	2541
2898	992	3835	2543
2899	703	2541	3835
2900	2543	3835	2541
2901	193	2109	2112
2902	701	3830	2109
2903	992	2112	3830
2904	2109	3830	2112
2905	703	3835	3828
2906	992	3830	3835
2907	701	3828	3830
2908	3835	3830	3828
2909	57	1357	1361
2910	557	3397	1357
2911	561	1361	3397
2912	1357	3397	1361
2913	265	2540	2542
2914	561	3408	2540
2915	983	2542	3408
2916	2540	3408	2542
2917	193	2112	2110
2918	992	4121	2112
2919	981	2110	4121
2920	2112	4121	2110
2921	265	2542	2543
2922	983	4123	2542
2923	992	2543	4123
2924	2542	4123	2543
2925	189	2087	2089
2926	981	4120	2087
2927	983	2089	4120
2928	2087	4120	2089
2929	992	4123	4121
2930	983	4120	4123
2931	981	4121	4120
2932	4123	4120	4121
2933	181	2036	2038
2934	389	2940	2036
2935	945	2038	2940

2936	2036	2940	2038
2937	385	2931	2929
2938	945	2940	2931
2939	389	2929	2940
2940	2931	2940	2929
2941	56	1355	1351
2942	555	3379	1355
2943	551	1351	3379
2944	1355	3379	1351
2945	263	2530	2528
2946	947	3390	2530
2947	555	2528	3390
2948	2530	3390	2528
2949	177	2013	2017
2950	551	3381	2013
2951	947	2017	3381
2952	2013	3381	2017
2953	555	3390	3379
2954	947	3381	3390
2955	551	3379	3381
2956	3390	3381	3379
2957	81	1500	1502
2958	700	3824	1500
2959	702	1502	3824
2960	1500	3824	1502
2961	181	2040	2037
2962	956	3826	2040
2963	700	2037	3826
2964	2040	3826	2037
2965	263	2529	2531
2966	702	3833	2529
2967	956	2531	3833
2968	2529	3833	2531
2969	700	3826	3824
2970	956	3833	3826
2971	702	3824	3833
2972	3826	3833	3824
2973	177	2017	2015
2974	947	4084	2017
2975	945	2015	4084
2976	2017	4084	2015
2977	263	2531	2530
2978	956	4087	2531
2979	947	2530	4087
2980	2531	4087	2530
2981	181	2038	2040
2982	945	4085	2038
2983	956	2040	4085
2984	2038	4085	2040
2985	947	4087	4084
2986	956	4085	4087
2987	945	4084	4085
2988	4087	4085	4084
2989	57	1361	1359
2990	561	3403	1361
2991	559	1359	3403
2992	1361	3403	1359
2993	265	2544	2540
2994	1064	3409	2544

2995	561	2540	3409
2996	2544	3409	2540
2997	81	1502	1503
2998	702	3832	1502
2999	703	1503	3832
3000	1502	3832	1503
3001	263	2533	2529
3002	1065	3834	2533
3003	702	2529	3834
3004	2533	3834	2529
3005	265	2541	2545
3006	703	3836	2541
3007	1065	2545	3836
3008	2541	3836	2545
3009	702	3834	3832
3010	1065	3836	3834
3011	703	3832	3836
3012	3834	3836	3832
3013	263	2528	2532
3014	555	3391	2528
3015	1063	2532	3391
3016	2528	3391	2532
3017	265	2545	2544
3018	1065	4201	2545
3019	1064	2544	4201
3020	2545	4201	2544
3021	263	2532	2533
3022	1063	4200	2532
3023	1065	2533	4200
3024	2532	4200	2533
3025	261	2521	2520
3026	1064	4199	2521
3027	1063	2520	4199
3028	2521	4199	2520
3029	1065	4200	4201
3030	1063	4199	4200
3031	1064	4201	4199
3032	4200	4199	4201
3033	7	1111	1113
3034	311	2737	1111
3035	313	1113	2737
3036	1111	2737	1113
3037	103	1611	1608
3038	753	2739	1611
3039	311	1608	2739
3040	1611	2739	1608
3041	105	1620	1623
3042	313	2743	1620
3043	753	1623	2743
3044	1620	2743	1623
3045	311	2739	2737
3046	753	2743	2739
3047	313	2737	2743
3048	2739	2743	2737
3049	47	1307	1304
3050	507	3249	1307
3051	504	1304	3249
3052	1307	3249	1304
3053	241	2398	2396

3054	755	3260	2398
3055	507	2396	3260
3056	2398	3260	2396
3057	103	1609	1613
3058	504	3251	1609
3059	755	1613	3251
3060	1609	3251	1613
3061	507	3260	3249
3062	755	3251	3260
3063	504	3249	3251
3064	3260	3251	3249
3065	105	1625	1621
3066	759	3287	1625
3067	516	1621	3287
3068	1625	3287	1621
3069	103	1613	1611
3070	755	3893	1613
3071	753	1611	3893
3072	1613	3893	1611
3073	241	2399	2398
3074	759	3895	2399
3075	755	2398	3895
3076	2399	3895	2398
3077	105	1623	1625
3078	753	3894	1623
3079	759	1625	3894
3080	1623	3894	1625
3081	755	3895	3893
3082	759	3894	3895
3083	753	3893	3894
3084	3895	3894	3893
3085	22	1183	1179
3086	383	2911	1183
3087	379	1179	2911
3088	1183	2911	1179
3089	175	2002	2000
3090	927	2922	2002
3091	383	2000	2922
3092	2002	2922	2000
3093	171	1976	1979
3094	379	2913	1976
3095	927	1979	2913
3096	1976	2913	1979
3097	383	2922	2911
3098	927	2913	2922
3099	379	2911	2913
3100	2922	2913	2911
3101	171	1981	1977
3102	929	3255	1981
3103	505	1977	3255
3104	1981	3255	1977
3105	175	2004	2002
3106	938	4067	2004
3107	927	2002	4067
3108	2004	4067	2002
3109	243	2410	2411
3110	929	4069	2410
3111	938	2411	4069
3112	2410	4069	2411

3113	171	1979	1981
3114	927	4066	1979
3115	929	1981	4066
3116	1979	4066	1981
3117	938	4069	4067
3118	929	4066	4069
3119	927	4067	4066
3120	4069	4066	4067
3121	25	1196	1200
3122	396	2961	1196
3123	400	1200	2961
3124	1196	2961	1200
3125	188	2081	2078
3126	977	2963	2081
3127	396	2078	2963
3128	2081	2963	2078
3129	192	2102	2104
3130	400	2974	2102
3131	977	2104	2974
3132	2102	2974	2104
3133	396	2963	2961
3134	977	2974	2963
3135	400	2961	2974
3136	2963	2974	2961
3137	188	2079	2083
3138	517	3291	2079
3139	979	2083	3291
3140	2079	3291	2083
3141	80	1495	1497
3142	695	3810	1495
3143	697	1497	3810
3144	1495	3810	1497
3145	192	2106	2103
3146	990	3812	2106
3147	695	2103	3812
3148	2106	3812	2103
3149	247	2433	2435
3150	697	3817	2433
3151	990	2435	3817
3152	2433	3817	2435
3153	695	3812	3810
3154	990	3817	3812
3155	697	3810	3817
3156	3812	3817	3810
3157	188	2083	2081
3158	979	4114	2083
3159	977	2081	4114
3160	2083	4114	2081
3161	247	2435	2434
3162	990	4117	2435
3163	979	2434	4117
3164	2435	4117	2434
3165	192	2104	2106
3166	977	4115	2104
3167	990	2106	4115
3168	2104	4115	2106
3169	979	4117	4114
3170	990	4115	4117
3171	977	4114	4115

3172	4117	4115	4114
3173	47	1309	1307
3174	509	3259	1309
3175	507	1307	3259
3176	1309	3259	1307
3177	241	2396	2400
3178	507	3261	2396
3179	1045	2400	3261
3180	2396	3261	2400
3181	80	1497	1496
3182	697	3814	1497
3183	696	1496	3814
3184	1497	3814	1496
3185	247	2437	2433
3186	1047	3818	2437
3187	697	2433	3818
3188	2437	3818	2433
3189	243	2409	2413
3190	696	3816	2409
3191	1047	2413	3816
3192	2409	3816	2413
3193	697	3818	3814
3194	1047	3816	3818
3195	696	3814	3816
3196	3818	3816	3814
3197	243	2413	2412
3198	1047	4182	2413
3199	1045	2412	4182
3200	2413	4182	2412
3201	247	2436	2437
3202	1046	4183	2436
3203	1047	2437	4183
3204	2436	4183	2437
3205	241	2400	2401
3206	1045	4181	2400
3207	1046	2401	4181
3208	2400	4181	2401
3209	1047	4183	4182
3210	1046	4181	4183
3211	1045	4182	4181
3212	4183	4181	4182
3213	81	1504	1500
3214	704	3825	1504
3215	700	1500	3825
3216	1504	3825	1500
3217	288	2681	2679
3218	957	3838	2681
3219	704	2679	3838
3220	2681	3838	2679
3221	181	2037	2041
3222	700	3827	2037
3223	957	2041	3827
3224	2037	3827	2041
3225	704	3838	3825
3226	957	3827	3838
3227	700	3825	3827
3228	3838	3827	3825
3229	79	1489	1493
3230	689	3793	1489

3231	693	1493	3793
3232	1489	3793	1493
3233	180	2035	2031
3234	955	3795	2035
3235	689	2031	3795
3236	2035	3795	2031
3237	288	2678	2680
3238	693	3804	2678
3239	955	2680	3804
3240	2678	3804	2680
3241	689	3795	3793
3242	955	3804	3795
3243	693	3793	3804
3244	3795	3804	3793
3245	25	1200	1201
3246	400	2973	1200
3247	401	1201	2973
3248	1200	2973	1201
3249	192	2105	2102
3250	989	2975	2105
3251	400	2102	2975
3252	2105	2975	2102
3253	193	2108	2111
3254	401	2977	2108
3255	989	2111	2977
3256	2108	2977	2111
3257	400	2975	2973
3258	989	2977	2975
3259	401	2973	2977
3260	2975	2977	2973
3261	80	1499	1495
3262	699	3811	1499
3263	695	1495	3811
3264	1499	3811	1495
3265	289	2686	2684
3266	991	3822	2686
3267	699	2684	3822
3268	2686	3822	2684
3269	192	2103	2107
3270	695	3813	2103
3271	991	2107	3813
3272	2103	3813	2107
3273	699	3822	3811
3274	991	3813	3822
3275	695	3811	3813
3276	3822	3813	3811
3277	81	1501	1505
3278	701	3829	1501
3279	705	1505	3829
3280	1501	3829	1505
3281	193	2113	2109
3282	993	3831	2113
3283	701	2109	3831
3284	2113	3831	2109
3285	289	2685	2687
3286	705	3840	2685
3287	993	2687	3840
3288	2685	3840	2687
3289	701	3831	3829

3290	993	3840	3831
3291	705	3829	3840
3292	3831	3840	3829
3293	192	2107	2105
3294	991	4127	2107
3295	989	2105	4127
3296	2107	4127	2105
3297	289	2687	2686
3298	993	4129	2687
3299	991	2686	4129
3300	2687	4129	2686
3301	193	2111	2113
3302	989	4128	2111
3303	993	2113	4128
3304	2111	4128	2113
3305	991	4129	4127
3306	993	4128	4129
3307	989	4127	4128
3308	4129	4128	4127
3309	22	1182	1183
3310	382	2919	1182
3311	383	1183	2919
3312	1182	2919	1183
3313	174	1997	1994
3314	935	2921	1997
3315	382	1994	2921
3316	1997	2921	1994
3317	175	2000	2003
3318	383	2923	2000
3319	935	2003	2923
3320	2000	2923	2003
3321	382	2921	2919
3322	935	2923	2921
3323	383	2919	2923
3324	2921	2923	2919
3325	79	1492	1488
3326	692	3789	1492
3327	688	1488	3789
3328	1492	3789	1488
3329	287	2674	2672
3330	937	3802	2674
3331	692	2672	3802
3332	2674	3802	2672
3333	174	1995	1999
3334	688	3791	1995
3335	937	1999	3791
3336	1995	3791	1999
3337	692	3802	3789
3338	937	3791	3802
3339	688	3789	3791
3340	3802	3791	3789
3341	174	1999	1997
3342	937	4073	1999
3343	935	1997	4073
3344	1999	4073	1997
3345	287	2675	2674
3346	939	4075	2675
3347	937	2674	4075
3348	2675	4075	2674

3349	175	2003	2005
3350	935	4074	2003
3351	939	2005	4074
3352	2003	4074	2005
3353	937	4075	4073
3354	939	4074	4075
3355	935	4073	4074
3356	4075	4074	4073
3357	81	1505	1504
3358	705	3837	1505
3359	704	1504	3837
3360	1505	3837	1504
3361	289	2689	2685
3362	1089	3841	2689
3363	705	2685	3841
3364	2689	3841	2685
3365	288	2679	2683
3366	704	3839	2679
3367	1089	2683	3839
3368	2679	3839	2683
3369	705	3841	3837
3370	1089	3839	3841
3371	704	3837	3839
3372	3841	3839	3837
3373	80	1498	1499
3374	698	3819	1498
3375	699	1499	3819
3376	1498	3819	1499
3377	287	2677	2673
3378	1088	3821	2677
3379	698	2673	3821
3380	2677	3821	2673
3381	289	2684	2688
3382	699	3823	2684
3383	1088	2688	3823
3384	2684	3823	2688
3385	698	3821	3819
3386	1088	3823	3821
3387	699	3819	3823
3388	3821	3823	3819
3389	79	1493	1492
3390	693	3801	1493
3391	692	1492	3801
3392	1493	3801	1492
3393	288	2682	2678
3394	1087	3805	2682
3395	693	2678	3805
3396	2682	3805	2678
3397	287	2672	2676
3398	692	3803	2672
3399	1087	2676	3803
3400	2672	3803	2676
3401	693	3805	3801
3402	1087	3803	3805
3403	692	3801	3803
3404	3805	3803	3801
3405	289	2688	2689
3406	1088	4225	2688
3407	1089	2689	4225

3408	2688	4225	2689
3409	287	2676	2677
3410	1087	4223	2676
3411	1088	2677	4223
3412	2676	4223	2677
3413	288	2683	2682
3414	1089	4224	2683
3415	1087	2682	4224
3416	2683	4224	2682
3417	1088	4223	4225
3418	1087	4224	4223
3419	1089	4225	4224
3420	4223	4224	4225
3421	84	1516	1514
3422	706	2694	1516
3423	292	1514	2694
3424	1516	2694	1514
3425	82	1506	1508
3426	290	2691	1506
3427	706	1508	2691
3428	1506	2691	1508
3429	292	2694	2690
3430	706	2691	2694
3431	290	2690	2691
3432	2694	2691	2690
3433	28	1212	1210
3434	412	2996	1212
3435	410	1210	2996
3436	1212	2996	1210
3437	194	2117	2115
3438	710	3005	2117
3439	412	2115	3005
3440	2117	3005	2115
3441	84	1515	1518
3442	410	2998	1515
3443	710	1518	2998
3444	1515	2998	1518
3445	412	3005	2996
3446	710	2998	3005
3447	410	2996	2998
3448	3005	2998	2996
3449	26	1202	1204
3450	402	2978	1202
3451	404	1204	2978
3452	1202	2978	1204
3453	82	1509	1507
3454	707	2979	1509
3455	402	1507	2979
3456	1509	2979	1507
3457	194	2114	2116
3458	404	2983	2114
3459	707	2116	2983
3460	2114	2983	2116
3461	402	2979	2978
3462	707	2983	2979
3463	404	2978	2983
3464	2979	2983	2978
3465	84	1518	1516
3466	710	3843	1518

3467	706	1516	3843
3468	1518	3843	1516
3469	194	2116	2117
3470	707	3844	2116
3471	710	2117	3844
3472	2116	3844	2117
3473	82	1508	1509
3474	706	3842	1508
3475	707	1509	3842
3476	1508	3842	1509
3477	710	3844	3843
3478	707	3842	3844
3479	706	3843	3842
3480	3844	3842	3843
3481	12	1132	1130
3482	332	2780	1132
3483	330	1130	2780
3484	1132	2780	1130
3485	124	1720	1718
3486	796	2789	1720
3487	332	1718	2789
3488	1720	2789	1718
3489	122	1706	1708
3490	330	2782	1706
3491	796	1708	2782
3492	1706	2782	1708
3493	332	2789	2780
3494	796	2782	2789
3495	330	2780	2782
3496	2789	2782	2780
3497	198	2141	2139
3498	805	3421	2141
3499	565	2139	3421
3500	2141	3421	2139
3501	124	1719	1722
3502	563	3416	1719
3503	805	1722	3416
3504	1719	3416	1722
3505	28	1211	1214
3506	411	3000	1211
3507	414	1214	3000
3508	1211	3000	1214
3509	122	1710	1707
3510	798	3002	1710
3511	411	1707	3002
3512	1710	3002	1707
3513	198	2138	2140
3514	414	3010	2138
3515	798	2140	3010
3516	2138	3010	2140
3517	411	3002	3000
3518	798	3010	3002
3519	414	3000	3010
3520	3002	3010	3000
3521	124	1722	1720
3522	805	3933	1722
3523	796	1720	3933
3524	1722	3933	1720
3525	198	2140	2141

3526	798	3936	2140
3527	805	2141	3936
3528	2140	3936	2141
3529	122	1708	1710
3530	796	3932	1708
3531	798	1710	3932
3532	1708	3932	1710
3533	805	3936	3933
3534	798	3932	3936
3535	796	3933	3932
3536	3936	3932	3933
3537	10	1122	1124
3538	322	2762	1122
3539	324	1124	2762
3540	1122	2762	1124
3541	114	1668	1666
3542	778	2763	1668
3543	322	1666	2763
3544	1668	2763	1666
3545	116	1674	1676
3546	324	2767	1674
3547	778	1676	2767
3548	1674	2767	1676
3549	322	2763	2762
3550	778	2767	2763
3551	324	2762	2767
3552	2763	2767	2762
3553	26	1205	1203
3554	405	2980	1205
3555	403	1203	2980
3556	1205	2980	1203
3557	195	2122	2120
3558	779	2985	2122
3559	405	2120	2985
3560	2122	2985	2120
3561	114	1667	1669
3562	403	2981	1667
3563	779	1669	2981
3564	1667	2981	1669
3565	405	2985	2980
3566	779	2981	2985
3567	403	2980	2981
3568	2985	2981	2980
3569	58	1362	1364
3570	562	3410	1362
3571	564	1364	3410
3572	1362	3410	1364
3573	116	1678	1675
3574	783	3412	1678
3575	562	1675	3412
3576	1678	3412	1675
3577	195	2121	2123
3578	564	3419	2121
3579	783	2123	3419
3580	2121	3419	2123
3581	562	3412	3410
3582	783	3419	3412
3583	564	3410	3419
3584	3412	3419	3410

3585	114	1669	1668
3586	779	3914	1669
3587	778	1668	3914
3588	1669	3914	1668
3589	195	2123	2122
3590	783	3916	2123
3591	779	2122	3916
3592	2123	3916	2122
3593	116	1676	1678
3594	778	3915	1676
3595	783	1678	3915
3596	1676	3915	1678
3597	779	3916	3914
3598	783	3915	3916
3599	778	3914	3915
3600	3916	3915	3914
3601	28	1214	1212
3602	414	3004	1214
3603	412	1212	3004
3604	1214	3004	1212
3605	198	2142	2138
3606	995	3011	2142
3607	414	2138	3011
3608	2142	3011	2138
3609	194	2115	2119
3610	412	3006	2115
3611	995	2119	3006
3612	2115	3006	2119
3613	414	3011	3004
3614	995	3006	3011
3615	412	3004	3006
3616	3011	3006	3004
3617	58	1364	1365
3618	564	3418	1364
3619	565	1365	3418
3620	1364	3418	1365
3621	195	2125	2121
3622	996	3420	2125
3623	564	2121	3420
3624	2125	3420	2121
3625	198	2139	2143
3626	565	3422	2139
3627	996	2143	3422
3628	2139	3422	2143
3629	564	3420	3418
3630	996	3422	3420
3631	565	3418	3422
3632	3420	3422	3418
3633	26	1204	1205
3634	404	2982	1204
3635	405	1205	2982
3636	1204	2982	1205
3637	194	2118	2114
3638	994	2984	2118
3639	404	2114	2984
3640	2118	2984	2114
3641	195	2120	2124
3642	405	2986	2120
3643	994	2124	2986

3644	2120	2986	2124
3645	404	2984	2982
3646	994	2986	2984
3647	405	2982	2986
3648	2984	2986	2982
3649	198	2143	2142
3650	996	4132	2143
3651	995	2142	4132
3652	2143	4132	2142
3653	195	2124	2125
3654	994	4131	2124
3655	996	2125	4131
3656	2124	4131	2125
3657	194	2119	2118
3658	995	4130	2119
3659	994	2118	4130
3660	2119	4130	2118
3661	996	4131	4132
3662	994	4130	4131
3663	995	4132	4130
3664	4131	4130	4132
3665	7	1110	1108
3666	310	2726	1110
3667	308	1108	2726
3668	1110	2726	1108
3669	102	1604	1602
3670	742	2735	1604
3671	310	1602	2735
3672	1604	2735	1602
3673	100	1590	1592
3674	308	2728	1590
3675	742	1592	2728
3676	1590	2728	1592
3677	310	2735	2726
3678	742	2728	2735
3679	308	2726	2728
3680	2735	2728	2726
3681	46	1300	1298
3682	500	3230	1300
3683	498	1298	3230
3684	1300	3230	1298
3685	230	2333	2331
3686	751	3239	2333
3687	500	2331	3239
3688	2333	3239	2331
3689	102	1603	1606
3690	498	3232	1603
3691	751	1606	3232
3692	1603	3232	1606
3693	500	3239	3230
3694	751	3232	3239
3695	498	3230	3232
3696	3239	3232	3230
3697	44	1286	1288
3698	486	3194	1286
3699	488	1288	3194
3700	1286	3194	1288
3701	100	1594	1591
3702	744	3196	1594

3703	486	1591	3196
3704	1594	3196	1591
3705	230	2330	2332
3706	488	3203	2330
3707	744	2332	3203
3708	2330	3203	2332
3709	486	3196	3194
3710	744	3203	3196
3711	488	3194	3203
3712	3196	3203	3194
3713	102	1606	1604
3714	751	3879	1606
3715	742	1604	3879
3716	1606	3879	1604
3717	230	2332	2333
3718	744	3882	2332
3719	751	2333	3882
3720	2332	3882	2333
3721	100	1592	1594
3722	742	3878	1592
3723	744	1594	3878
3724	1592	3878	1594
3725	751	3882	3879
3726	744	3878	3882
3727	742	3879	3878
3728	3882	3878	3879
3729	20	1169	1167
3730	369	2874	1169
3731	367	1167	2874
3732	1169	2874	1167
3733	161	1918	1916
3734	890	2881	1918
3735	369	1916	2881
3736	1918	2881	1916
3737	159	1904	1906
3738	367	2876	1904
3739	890	1906	2876
3740	1904	2876	1906
3741	369	2881	2874
3742	890	2876	2881
3743	367	2874	2876
3744	2881	2876	2874
3745	62	1389	1387
3746	589	3486	1389
3747	587	1387	3486
3748	1389	3486	1387
3749	239	2387	2385
3750	897	3493	2387
3751	589	2385	3493
3752	2387	3493	2385
3753	161	1917	1920
3754	587	3488	1917
3755	897	1920	3488
3756	1917	3488	1920
3757	589	3493	3486
3758	897	3488	3493
3759	587	3486	3488
3760	3493	3488	3486
3761	46	1299	1302

3762	499	3234	1299
3763	502	1302	3234
3764	1299	3234	1302
3765	159	1908	1905
3766	892	3236	1908
3767	499	1905	3236
3768	1908	3236	1905
3769	239	2384	2386
3770	502	3244	2384
3771	892	2386	3244
3772	2384	3244	2386
3773	499	3236	3234
3774	892	3244	3236
3775	502	3234	3244
3776	3236	3244	3234
3777	161	1920	1918
3778	897	4029	1920
3779	890	1918	4029
3780	1920	4029	1918
3781	239	2386	2387
3782	892	4032	2386
3783	897	2387	4032
3784	2386	4032	2387
3785	159	1906	1908
3786	890	4028	1906
3787	892	1908	4028
3788	1906	4028	1908
3789	897	4032	4029
3790	892	4028	4032
3791	890	4029	4028
3792	4032	4028	4029
3793	12	1131	1134
3794	331	2784	1131
3795	334	1134	2784
3796	1131	2784	1134
3797	123	1714	1712
3798	800	2786	1714
3799	331	1712	2786
3800	1714	2786	1712
3801	126	1730	1732
3802	334	2794	1730
3803	800	1732	2794
3804	1730	2794	1732
3805	331	2786	2784
3806	800	2794	2786
3807	334	2784	2794
3808	2786	2794	2784
3809	44	1290	1287
3810	490	3198	1290
3811	487	1287	3198
3812	1290	3198	1287
3813	232	2344	2342
3814	802	3208	2344
3815	490	2342	3208
3816	2344	3208	2342
3817	123	1713	1716
3818	487	3200	1713
3819	802	1716	3200
3820	1713	3200	1716

3821	490	3208	3198
3822	802	3200	3208
3823	487	3198	3200
3824	3208	3200	3198
3825	62	1386	1388
3826	586	3482	1386
3827	588	1388	3482
3828	1386	3482	1388
3829	126	1734	1731
3830	810	3484	1734
3831	586	1731	3484
3832	1734	3484	1731
3833	232	2343	2345
3834	588	3491	2343
3835	810	2345	3491
3836	2343	3491	2345
3837	586	3484	3482
3838	810	3491	3484
3839	588	3482	3491
3840	3484	3491	3482
3841	123	1716	1714
3842	802	3938	1716
3843	800	1714	3938
3844	1716	3938	1714
3845	232	2345	2344
3846	810	3942	2345
3847	802	2344	3942
3848	2345	3942	2344
3849	126	1732	1734
3850	800	3939	1732
3851	810	1734	3939
3852	1732	3939	1734
3853	802	3942	3938
3854	810	3939	3942
3855	800	3938	3939
3856	3942	3939	3938
3857	46	1302	1300
3858	502	3238	1302
3859	500	1300	3238
3860	1302	3238	1300
3861	239	2388	2384
3862	1031	3245	2388
3863	502	2384	3245
3864	2388	3245	2384
3865	230	2331	2335
3866	500	3240	2331
3867	1031	2335	3240
3868	2331	3240	2335
3869	502	3245	3238
3870	1031	3240	3245
3871	500	3238	3240
3872	3245	3240	3238
3873	62	1388	1389
3874	588	3490	1388
3875	589	1389	3490
3876	1388	3490	1389
3877	232	2347	2343
3878	1034	3492	2347
3879	588	2343	3492

3880	2347	3492	2343
3881	239	2385	2389
3882	589	3494	2385
3883	1034	2389	3494
3884	2385	3494	2389
3885	588	3492	3490
3886	1034	3494	3492
3887	589	3490	3494
3888	3492	3494	3490
3889	44	1288	1290
3890	488	3202	1288
3891	490	1290	3202
3892	1288	3202	1290
3893	230	2334	2330
3894	1030	3204	2334
3895	488	2330	3204
3896	2334	3204	2330
3897	232	2342	2346
3898	490	3209	2342
3899	1030	2346	3209
3900	2342	3209	2346
3901	488	3204	3202
3902	1030	3209	3204
3903	490	3202	3209
3904	3204	3209	3202
3905	239	2389	2388
3906	1034	4168	2389
3907	1031	2388	4168
3908	2389	4168	2388
3909	232	2346	2347
3910	1030	4167	2346
3911	1034	2347	4167
3912	2346	4167	2347
3913	230	2335	2334
3914	1031	4166	2335
3915	1030	2334	4166
3916	2335	4166	2334
3917	1034	4167	4168
3918	1030	4166	4167
3919	1031	4168	4166
3920	4167	4166	4168
3921	94	1558	1560
3922	302	2713	1558
3923	724	1560	2713
3924	1558	2713	1560
3925	36	1248	1246
3926	448	3086	1248
3927	446	1246	3086
3928	1248	3086	1246
3929	212	2224	2222
3930	725	3091	2224
3931	448	2222	3091
3932	2224	3091	2222
3933	38	1254	1256
3934	454	3104	1254
3935	456	1256	3104
3936	1254	3104	1256
3937	94	1562	1559
3938	729	3106	1562

3939	454	1559	3106
3940	1562	3106	1559
3941	212	2223	2225
3942	456	3113	2223
3943	729	2225	3113
3944	2223	3113	2225
3945	454	3106	3104
3946	729	3113	3106
3947	456	3104	3113
3948	3106	3113	3104
3949	92	1553	1552
3950	725	3860	1553
3951	724	1552	3860
3952	1553	3860	1552
3953	212	2225	2224
3954	729	3862	2225
3955	725	2224	3862
3956	2225	3862	2224
3957	94	1560	1562
3958	724	3861	1560
3959	729	1562	3861
3960	1560	3861	1562
3961	725	3862	3860
3962	729	3861	3862
3963	724	3860	3861
3964	3862	3861	3860
3965	10	1125	1123
3966	325	2764	1125
3967	323	1123	2764
3968	1125	2764	1123
3969	117	1682	1680
3970	780	2769	1682
3971	325	1680	2769
3972	1682	2769	1680
3973	115	1670	1672
3974	323	2765	1670
3975	780	1672	2765
3976	1670	2765	1672
3977	325	2769	2764
3978	780	2765	2769
3979	323	2764	2765
3980	2769	2765	2764
3981	59	1370	1368
3982	570	3428	1370
3983	568	1368	3428
3984	1370	3428	1368
3985	213	2231	2229
3986	785	3437	2231
3987	570	2229	3437
3988	2231	3437	2229
3989	117	1681	1684
3990	568	3430	1681
3991	785	1684	3430
3992	1681	3430	1684
3993	570	3437	3428
3994	785	3430	3437
3995	568	3428	3430
3996	3437	3430	3428
3997	36	1247	1249

3998	447	3088	1247
3999	449	1249	3088
4000	1247	3088	1249
4001	115	1673	1671
4002	781	3089	1673
4003	447	1671	3089
4004	1673	3089	1671
4005	213	2228	2230
4006	449	3093	2228
4007	781	2230	3093
4008	2228	3093	2230
4009	447	3089	3088
4010	781	3093	3089
4011	449	3088	3093
4012	3089	3093	3088
4013	117	1684	1682
4014	785	3918	1684
4015	780	1682	3918
4016	1684	3918	1682
4017	213	2230	2231
4018	781	3919	2230
4019	785	2231	3919
4020	2230	3919	2231
4021	115	1672	1673
4022	780	3917	1672
4023	781	1673	3917
4024	1672	3917	1673
4025	785	3919	3918
4026	781	3917	3919
4027	780	3918	3917
4028	3919	3917	3918
4029	20	1166	1168
4030	366	2870	1166
4031	368	1168	2870
4032	1166	2870	1168
4033	158	1900	1898
4034	886	2872	1900
4035	366	1898	2872
4036	1900	2872	1898
4037	160	1910	1912
4038	368	2879	1910
4039	886	1912	2879
4040	1910	2879	1912
4041	366	2872	2870
4042	886	2879	2872
4043	368	2870	2879
4044	2872	2879	2870
4045	217	2253	2255
4046	571	3439	2253
4047	895	2255	3439
4048	2253	3439	2255
4049	36	1249	1248
4050	449	3090	1249
4051	448	1248	3090
4052	1249	3090	1248
4053	213	2232	2228
4054	1012	3094	2232
4055	449	2228	3094
4056	2232	3094	2228

4057	212	2222	2226
4058	448	3092	2222
4059	1012	2226	3092
4060	2222	3092	2226
4061	449	3094	3090
4062	1012	3092	3094
4063	448	3090	3092
4064	3094	3092	3090
4065	59	1371	1370
4066	571	3436	1371
4067	570	1370	3436
4068	1371	3436	1370
4069	217	2257	2253
4070	1014	3440	2257
4071	571	2253	3440
4072	2257	3440	2253
4073	213	2229	2233
4074	570	3438	2229
4075	1014	2233	3438
4076	2229	3438	2233
4077	571	3440	3436
4078	1014	3438	3440
4079	570	3436	3438
4080	3440	3438	3436
4081	38	1256	1258
4082	456	3112	1256
4083	458	1258	3112
4084	1256	3112	1258
4085	212	2227	2223
4086	1013	3114	2227
4087	456	2223	3114
4088	2227	3114	2223
4089	217	2252	2256
4090	458	3119	2252
4091	1013	2256	3119
4092	2252	3119	2256
4093	456	3114	3112
4094	1013	3119	3114
4095	458	3112	3119
4096	3114	3119	3112
4097	213	2233	2232
4098	1014	4149	2233
4099	1012	2232	4149
4100	2233	4149	2232
4101	217	2256	2257
4102	1013	4150	2256
4103	1014	2257	4150
4104	2256	4150	2257
4105	212	2226	2227
4106	1012	4148	2226
4107	1013	2227	4148
4108	2226	4148	2227
4109	1014	4150	4149
4110	1013	4148	4150
4111	1012	4149	4148
4112	4150	4148	4149
4113	12	1134	1132
4114	334	2788	1134
4115	332	1132	2788

4116	1134	2788	1132
4117	126	1733	1730
4118	804	2795	1733
4119	334	1730	2795
4120	1733	2795	1730
4121	124	1718	1721
4122	332	2790	1718
4123	804	1721	2790
4124	1718	2790	1721
4125	334	2795	2788
4126	804	2790	2795
4127	332	2788	2790
4128	2795	2790	2788
4129	62	1390	1386
4130	590	3483	1390
4131	586	1386	3483
4132	1390	3483	1386
4133	267	2555	2553
4134	811	3496	2555
4135	590	2553	3496
4136	2555	3496	2553
4137	126	1731	1735
4138	586	3485	1731
4139	811	1735	3485
4140	1731	3485	1735
4141	590	3496	3483
4142	811	3485	3496
4143	586	3483	3485
4144	3496	3485	3483
4145	58	1363	1367
4146	563	3415	1363
4147	567	1367	3415
4148	1363	3415	1367
4149	124	1723	1719
4150	806	3417	1723
4151	563	1719	3417
4152	1723	3417	1719
4153	267	2552	2554
4154	567	3426	2552
4155	806	2554	3426
4156	2552	3426	2554
4157	563	3417	3415
4158	806	3426	3417
4159	567	3415	3426
4160	3417	3426	3415
4161	126	1735	1733
4162	811	3945	1735
4163	804	1733	3945
4164	1735	3945	1733
4165	267	2554	2555
4166	806	3946	2554
4167	811	2555	3946
4168	2554	3946	2555
4169	124	1721	1723
4170	804	3944	1721
4171	806	1723	3944
4172	1721	3944	1723
4173	811	3946	3945
4174	806	3944	3946

4175	804	3945	3944
4176	3946	3944	3945
4177	20	1168	1169
4178	368	2878	1168
4179	369	1169	2878
4180	1168	2878	1169
4181	160	1913	1910
4182	894	2880	1913
4183	368	1910	2880
4184	1913	2880	1910
4185	161	1916	1919
4186	369	2882	1916
4187	894	1919	2882
4188	1916	2882	1919
4189	368	2880	2878
4190	894	2882	2880
4191	369	2878	2882
4192	2880	2882	2878
4193	268	2560	2558
4194	896	3444	2560
4195	573	2558	3444
4196	2560	3444	2558
4197	62	1387	1391
4198	587	3487	1387
4199	591	1391	3487
4200	1387	3487	1391
4201	161	1921	1917
4202	898	3489	1921
4203	587	1917	3489
4204	1921	3489	1917
4205	268	2559	2561
4206	591	3498	2559
4207	898	2561	3498
4208	2559	3498	2561
4209	587	3489	3487
4210	898	3498	3489
4211	591	3487	3498
4212	3489	3498	3487
4213	160	1915	1913
4214	896	4034	1915
4215	894	1913	4034
4216	1915	4034	1913
4217	268	2561	2560
4218	898	4036	2561
4219	896	2560	4036
4220	2561	4036	2560
4221	161	1919	1921
4222	894	4035	1919
4223	898	1921	4035
4224	1919	4035	1921
4225	896	4036	4034
4226	898	4035	4036
4227	894	4034	4035
4228	4036	4035	4034
4229	10	1124	1125
4230	324	2766	1124
4231	325	1125	2766
4232	1124	2766	1125
4233	116	1677	1674

4234	782	2768	1677
4235	324	1674	2768
4236	1677	2768	1674
4237	117	1680	1683
4238	325	2770	1680
4239	782	1683	2770
4240	1680	2770	1683
4241	324	2768	2766
4242	782	2770	2768
4243	325	2766	2770
4244	2768	2770	2766
4245	116	1675	1679
4246	562	3413	1675
4247	784	1679	3413
4248	1675	3413	1679
4249	59	1368	1372
4250	568	3429	1368
4251	572	1372	3429
4252	1368	3429	1372
4253	117	1685	1681
4254	786	3431	1685
4255	568	1681	3431
4256	1685	3431	1681
4257	266	2547	2549
4258	572	3442	2547
4259	786	2549	3442
4260	2547	3442	2549
4261	568	3431	3429
4262	786	3442	3431
4263	572	3429	3442
4264	3431	3442	3429
4265	116	1679	1677
4266	784	3920	1679
4267	782	1677	3920
4268	1679	3920	1677
4269	266	2549	2548
4270	786	3922	2549
4271	784	2548	3922
4272	2549	3922	2548
4273	117	1683	1685
4274	782	3921	1683
4275	786	1685	3921
4276	1683	3921	1685
4277	784	3922	3920
4278	786	3921	3922
4279	782	3920	3921
4280	3922	3921	3920
4281	62	1391	1390
4282	591	3495	1391
4283	590	1390	3495
4284	1391	3495	1390
4285	268	2563	2559
4286	1068	3499	2563
4287	591	2559	3499
4288	2563	3499	2559
4289	267	2553	2557
4290	590	3497	2553
4291	1068	2557	3497
4292	2553	3497	2557

4293	591	3499	3495
4294	1068	3497	3499
4295	590	3495	3497
4296	3499	3497	3495
4297	59	1372	1373
4298	572	3441	1372
4299	573	1373	3441
4300	1372	3441	1373
4301	266	2551	2547
4302	1067	3443	2551
4303	572	2547	3443
4304	2551	3443	2547
4305	268	2558	2562
4306	573	3445	2558
4307	1067	2562	3445
4308	2558	3445	2562
4309	572	3443	3441
4310	1067	3445	3443
4311	573	3441	3445
4312	3443	3445	3441
4313	267	2556	2552
4314	1066	3427	2556
4315	567	2552	3427
4316	2556	3427	2552
4317	268	2562	2563
4318	1067	4204	2562
4319	1068	2563	4204
4320	2562	4204	2563
4321	266	2550	2551
4322	1066	4202	2550
4323	1067	2551	4202
4324	2550	4202	2551
4325	267	2557	2556
4326	1068	4203	2557
4327	1066	2556	4203
4328	2557	4203	2556
4329	1067	4202	4204
4330	1066	4203	4202
4331	1068	4204	4203
4332	4202	4203	4204
4333	4	1098	1097
4334	298	2702	1098
4335	297	1097	2702
4336	1098	2702	1097
4337	90	1544	1542
4338	718	2706	1544
4339	298	1542	2706
4340	1544	2706	1542
4341	89	1536	1538
4342	297	2704	1536
4343	718	1538	2704
4344	1536	2704	1538
4345	298	2706	2702
4346	718	2704	2706
4347	297	2702	2704
4348	2706	2704	2702
4349	34	1240	1238
4350	440	3068	1240
4351	438	1238	3068

4352	1240	3068	1238
4353	206	2189	2187
4354	722	3073	2189
4355	440	2187	3073
4356	2189	3073	2187
4357	33	1232	1234
4358	432	3050	1232
4359	434	1234	3050
4360	1232	3050	1234
4361	89	1540	1537
4362	720	3052	1540
4363	432	1537	3052
4364	1540	3052	1537
4365	206	2186	2188
4366	434	3059	2186
4367	720	2188	3059
4368	2186	3059	2188
4369	432	3052	3050
4370	720	3059	3052
4371	434	3050	3059
4372	3052	3059	3050
4373	90	1545	1544
4374	722	3855	1545
4375	718	1544	3855
4376	1545	3855	1544
4377	206	2188	2189
4378	720	3858	2188
4379	722	2189	3858
4380	2188	3858	2189
4381	89	1538	1540
4382	718	3854	1538
4383	720	1540	3854
4384	1538	3854	1540
4385	722	3858	3855
4386	720	3854	3858
4387	718	3855	3854
4388	3858	3854	3855
4389	18	1160	1158
4390	360	2852	1160
4391	358	1158	2852
4392	1160	2852	1158
4393	152	1868	1866
4394	868	2857	1868
4395	360	1866	2857
4396	1868	2857	1866
4397	150	1858	1860
4398	358	2853	1858
4399	868	1860	2853
4400	1858	2853	1860
4401	360	2857	2852
4402	868	2853	2857
4403	358	2852	2853
4404	2857	2853	2852
4405	34	1239	1241
4406	439	3070	1239
4407	441	1241	3070
4408	1239	3070	1241
4409	150	1861	1859
4410	869	3071	1861

4411	439	1859	3071
4412	1861	3071	1859
4413	210	2210	2212
4414	441	3075	2210
4415	869	2212	3075
4416	2210	3075	2212
4417	439	3071	3070
4418	869	3075	3071
4419	441	3070	3075
4420	3071	3075	3070
4421	152	1870	1868
4422	873	4005	1870
4423	868	1868	4005
4424	1870	4005	1868
4425	210	2212	2213
4426	869	4006	2212
4427	873	2213	4006
4428	2212	4006	2213
4429	150	1860	1861
4430	868	4004	1860
4431	869	1861	4004
4432	1860	4004	1861
4433	873	4006	4005
4434	869	4004	4006
4435	868	4005	4004
4436	4006	4004	4005
4437	17	1152	1154
4438	352	2834	1152
4439	354	1154	2834
4440	1152	2834	1154
4441	144	1824	1822
4442	850	2836	1824
4443	352	1822	2836
4444	1824	2836	1822
4445	146	1834	1836
4446	354	2843	1834
4447	850	1836	2843
4448	1834	2843	1836
4449	352	2836	2834
4450	850	2843	2836
4451	354	2834	2843
4452	2836	2843	2834
4453	33	1236	1233
4454	436	3054	1236
4455	433	1233	3054
4456	1236	3054	1233
4457	208	2200	2198
4458	852	3064	2200
4459	436	2198	3064
4460	2200	3064	2198
4461	144	1823	1826
4462	433	3056	1823
4463	852	1826	3056
4464	1823	3056	1826
4465	436	3064	3054
4466	852	3056	3064
4467	433	3054	3056
4468	3064	3056	3054
4469	70	1434	1436

4470	634	3626	1434
4471	636	1436	3626
4472	1434	3626	1436
4473	146	1838	1835
4474	859	3628	1838
4475	634	1835	3628
4476	1838	3628	1835
4477	208	2199	2201
4478	636	3635	2199
4479	859	2201	3635
4480	2199	3635	2201
4481	634	3628	3626
4482	859	3635	3628
4483	636	3626	3635
4484	3628	3635	3626
4485	144	1826	1824
4486	852	3986	1826
4487	850	1824	3986
4488	1826	3986	1824
4489	208	2201	2200
4490	859	3990	2201
4491	852	2200	3990
4492	2201	3990	2200
4493	146	1836	1838
4494	850	3987	1836
4495	859	1838	3987
4496	1836	3987	1838
4497	852	3990	3986
4498	859	3987	3990
4499	850	3986	3987
4500	3990	3987	3986
4501	34	1241	1240
4502	441	3072	1241
4503	440	1240	3072
4504	1241	3072	1240
4505	210	2214	2210
4506	1007	3076	2214
4507	441	2210	3076
4508	2214	3076	2210
4509	206	2187	2191
4510	440	3074	2187
4511	1007	2191	3074
4512	2187	3074	2191
4513	441	3076	3072
4514	1007	3074	3076
4515	440	3072	3074
4516	3076	3074	3072
4517	208	2203	2199
4518	1010	3636	2203
4519	636	2199	3636
4520	2203	3636	2199
4521	33	1234	1236
4522	434	3058	1234
4523	436	1236	3058
4524	1234	3058	1236
4525	206	2190	2186
4526	1006	3060	2190
4527	434	2186	3060
4528	2190	3060	2186

4529	210	2215	2214
4530	1010	4144	2215
4531	1007	2214	4144
4532	2215	4144	2214
4533	206	2191	2190
4534	1007	4142	2191
4535	1006	2190	4142
4536	2191	4142	2190
4537	1010	4143	4144
4538	1006	4142	4143
4539	1007	4144	4142
4540	4143	4142	4144
4541	8	1117	1115
4542	317	2746	1117
4543	315	1115	2746
4544	1117	2746	1115
4545	109	1642	1640
4546	762	2751	1642
4547	317	1640	2751
4548	1642	2751	1640
4549	107	1630	1632
4550	315	2747	1630
4551	762	1632	2747
4552	1630	2747	1632
4553	317	2751	2746
4554	762	2747	2751
4555	315	2746	2747
4556	2751	2747	2746
4557	53	1338	1336
4558	538	3338	1338
4559	536	1336	3338
4560	1338	3338	1336
4561	250	2453	2451
4562	767	3347	2453
4563	538	2451	3347
4564	2453	3347	2451
4565	109	1641	1644
4566	536	3340	1641
4567	767	1644	3340
4568	1641	3340	1644
4569	538	3347	3338
4570	767	3340	3347
4571	536	3338	3340
4572	3347	3340	3338
4573	51	1326	1328
4574	526	3311	1326
4575	528	1328	3311
4576	1326	3311	1328
4577	107	1633	1631
4578	763	3312	1633
4579	526	1631	3312
4580	1633	3312	1631
4581	526	3312	3311
4582	763	3316	3312
4583	528	3311	3316
4584	3312	3316	3311
4585	109	1644	1642
4586	767	3900	1644
4587	762	1642	3900

4588	1644	3900	1642
4589	250	2452	2453
4590	763	3901	2452
4591	767	2453	3901
4592	2452	3901	2453
4593	107	1632	1633
4594	762	3899	1632
4595	763	1633	3899
4596	1632	3899	1633
4597	767	3901	3900
4598	763	3899	3901
4599	762	3900	3899
4600	3901	3899	3900
4601	24	1193	1191
4602	393	2946	1193
4603	391	1191	2946
4604	1193	2946	1191
4605	185	2062	2060
4606	962	2953	2062
4607	393	2060	2953
4608	2062	2953	2060
4609	183	2048	2050
4610	391	2948	2048
4611	962	2050	2948
4612	2048	2948	2050
4613	393	2953	2946
4614	962	2948	2953
4615	391	2946	2948
4616	2953	2948	2946
4617	74	1461	1459
4618	661	3702	1461
4619	659	1459	3702
4620	1461	3702	1459
4621	255	2483	2481
4622	969	3709	2483
4623	661	2481	3709
4624	2483	3709	2481
4625	185	2061	2064
4626	659	3704	2061
4627	969	2064	3704
4628	2061	3704	2064
4629	661	3709	3702
4630	969	3704	3709
4631	659	3702	3704
4632	3709	3704	3702
4633	53	1337	1340
4634	537	3342	1337
4635	540	1340	3342
4636	1337	3342	1340
4637	183	2052	2049
4638	964	3344	2052
4639	537	2049	3344
4640	2052	3344	2049
4641	255	2480	2482
4642	540	3352	2480
4643	964	2482	3352
4644	2480	3352	2482
4645	537	3344	3342
4646	964	3352	3344

4647	540	3342	3352
4648	3344	3352	3342
4649	185	2064	2062
4650	969	4101	2064
4651	962	2062	4101
4652	2064	4101	2062
4653	255	2482	2483
4654	964	4104	2482
4655	969	2483	4104
4656	2482	4104	2483
4657	183	2050	2052
4658	962	4100	2050
4659	964	2052	4100
4660	2050	4100	2052
4661	969	4104	4101
4662	964	4100	4104
4663	962	4101	4100
4664	4104	4100	4101
4665	18	1159	1161
4666	359	2854	1159
4667	361	1161	2854
4668	1159	2854	1161
4669	153	1872	1874
4670	361	2859	1872
4671	870	1874	2859
4672	1872	2859	1874
4673	51	1329	1327
4674	529	3313	1329
4675	527	1327	3313
4676	1329	3313	1327
4677	251	2458	2456
4678	871	3318	2458
4679	529	2456	3318
4680	2458	3318	2456
4681	151	1863	1865
4682	527	3314	1863
4683	871	1865	3314
4684	1863	3314	1865
4685	529	3318	3313
4686	871	3314	3318
4687	527	3313	3314
4688	3318	3314	3313
4689	74	1458	1460
4690	658	3698	1458
4691	660	1460	3698
4692	1458	3698	1460
4693	153	1876	1873
4694	875	3700	1876
4695	658	1873	3700
4696	1876	3700	1873
4697	251	2457	2459
4698	660	3707	2457
4699	875	2459	3707
4700	2457	3707	2459
4701	658	3700	3698
4702	875	3707	3700
4703	660	3698	3707
4704	3700	3707	3698
4705	151	1865	1864

4706	871	4007	1865
4707	870	1864	4007
4708	1865	4007	1864
4709	251	2459	2458
4710	875	4009	2459
4711	871	2458	4009
4712	2459	4009	2458
4713	153	1874	1876
4714	870	4008	1874
4715	875	1876	4008
4716	1874	4008	1876
4717	871	4009	4007
4718	875	4008	4009
4719	870	4007	4008
4720	4009	4008	4007
4721	53	1340	1338
4722	540	3346	1340
4723	538	1338	3346
4724	1340	3346	1338
4725	255	2484	2480
4726	1052	3353	2484
4727	540	2480	3353
4728	2484	3353	2480
4729	250	2451	2455
4730	538	3348	2451
4731	1052	2455	3348
4732	2451	3348	2455
4733	540	3353	3346
4734	1052	3348	3353
4735	538	3346	3348
4736	3353	3348	3346
4737	74	1460	1461
4738	660	3706	1460
4739	661	1461	3706
4740	1460	3706	1461
4741	251	2461	2457
4742	1053	3708	2461
4743	660	2457	3708
4744	2461	3708	2457
4745	660	3708	3706
4746	1053	3710	3708
4747	661	3706	3710
4748	3708	3710	3706
4749	51	1328	1329
4750	528	3315	1328
4751	529	1329	3315
4752	1328	3315	1329
4753	251	2460	2461
4754	1051	4188	2460
4755	1053	2461	4188
4756	2460	4188	2461
4757	250	2455	2454
4758	1052	4187	2455
4759	1051	2454	4187
4760	2455	4187	2454
4761	1053	4188	4189
4762	1051	4187	4188
4763	1052	4189	4187
4764	4188	4187	4189

4765	7	1109	1112
4766	309	2730	1109
4767	312	1112	2730
4768	1109	2730	1112
4769	101	1598	1596
4770	746	2732	1598
4771	309	1596	2732
4772	1598	2732	1596
4773	104	1614	1616
4774	312	2740	1614
4775	746	1616	2740
4776	1614	2740	1616
4777	309	2732	2730
4778	746	2740	2732
4779	312	2730	2740
4780	2732	2740	2730
4781	45	1294	1292
4782	494	3212	1294
4783	492	1292	3212
4784	1294	3212	1292
4785	234	2356	2354
4786	748	3221	2356
4787	494	2354	3221
4788	2356	3221	2354
4789	101	1597	1600
4790	492	3214	1597
4791	748	1600	3214
4792	1597	3214	1600
4793	494	3221	3212
4794	748	3214	3221
4795	492	3212	3214
4796	3221	3214	3212
4797	48	1310	1312
4798	510	3266	1310
4799	512	1312	3266
4800	1310	3266	1312
4801	104	1618	1615
4802	756	3268	1618
4803	510	1615	3268
4804	1618	3268	1615
4805	234	2355	2357
4806	512	3275	2355
4807	756	2357	3275
4808	2355	3275	2357
4809	510	3268	3266
4810	756	3275	3268
4811	512	3266	3275
4812	3268	3275	3266
4813	101	1600	1598
4814	748	3884	1600
4815	746	1598	3884
4816	1600	3884	1598
4817	234	2357	2356
4818	756	3888	2357
4819	748	2356	3888
4820	2357	3888	2356
4821	104	1616	1618
4822	746	3885	1616
4823	756	1618	3885

4824	1616	3885	1618
4825	748	3888	3884
4826	756	3885	3888
4827	746	3884	3885
4828	3888	3885	3884
4829	17	1156	1153
4830	356	2838	1156
4831	353	1153	2838
4832	1156	2838	1153
4833	148	1848	1846
4834	854	2848	1848
4835	356	1846	2848
4836	1848	2848	1846
4837	145	1828	1830
4838	353	2840	1828
4839	854	1830	2840
4840	1828	2840	1830
4841	356	2848	2838
4842	854	2840	2848
4843	353	2838	2840
4844	2848	2840	2838
4845	72	1448	1446
4846	648	3662	1448
4847	646	1446	3662
4848	1448	3662	1446
4849	236	2369	2367
4850	864	3671	2369
4851	648	2367	3671
4852	2369	3671	2367
4853	148	1847	1850
4854	646	3664	1847
4855	864	1850	3664
4856	1847	3664	1850
4857	648	3671	3662
4858	864	3664	3671
4859	646	3662	3664
4860	3671	3664	3662
4861	45	1293	1296
4862	493	3216	1293
4863	496	1296	3216
4864	1293	3216	1296
4865	145	1832	1829
4866	856	3218	1832
4867	493	1829	3218
4868	1832	3218	1829
4869	236	2366	2368
4870	496	3226	2366
4871	856	2368	3226
4872	2366	3226	2368
4873	493	3218	3216
4874	856	3226	3218
4875	496	3216	3226
4876	3218	3226	3216
4877	148	1850	1848
4878	864	3993	1850
4879	854	1848	3993
4880	1850	3993	1848
4881	236	2368	2369
4882	856	3996	2368

4883	864	2369	3996
4884	2368	3996	2369
4885	145	1830	1832
4886	854	3992	1830
4887	856	1832	3992
4888	1830	3992	1832
4889	864	3996	3993
4890	856	3992	3996
4891	854	3993	3992
4892	3996	3992	3993
4893	24	1190	1192
4894	390	2942	1190
4895	392	1192	2942
4896	1190	2942	1192
4897	182	2044	2042
4898	958	2944	2044
4899	390	2042	2944
4900	2044	2944	2042
4901	184	2054	2056
4902	392	2951	2054
4903	958	2056	2951
4904	2054	2951	2056
4905	390	2944	2942
4906	958	2951	2944
4907	392	2942	2951
4908	2944	2951	2942
4909	48	1314	1311
4910	514	3270	1314
4911	511	1311	3270
4912	1314	3270	1311
4913	244	2416	2414
4914	960	3280	2416
4915	514	2414	3280
4916	2416	3280	2414
4917	182	2043	2046
4918	511	3272	2043
4919	960	2046	3272
4920	2043	3272	2046
4921	514	3280	3270
4922	960	3272	3280
4923	511	3270	3272
4924	3280	3272	3270
4925	72	1447	1449
4926	647	3666	1447
4927	649	1449	3666
4928	1447	3666	1449
4929	184	2058	2055
4930	967	3668	2058
4931	647	2055	3668
4932	2058	3668	2055
4933	244	2415	2417
4934	649	3673	2415
4935	967	2417	3673
4936	2415	3673	2417
4937	647	3668	3666
4938	967	3673	3668
4939	649	3666	3673
4940	3668	3673	3666
4941	182	2046	2044

4942	960	4094	2046
4943	958	2044	4094
4944	2046	4094	2044
4945	244	2417	2416
4946	967	4098	2417
4947	960	2416	4098
4948	2417	4098	2416
4949	184	2056	2058
4950	958	4095	2056
4951	967	2058	4095
4952	2056	4095	2058
4953	960	4098	4094
4954	967	4095	4098
4955	958	4094	4095
4956	4098	4095	4094
4957	45	1296	1294
4958	496	3220	1296
4959	494	1294	3220
4960	1296	3220	1294
4961	236	2370	2366
4962	1036	3227	2370
4963	496	2366	3227
4964	2370	3227	2366
4965	234	2354	2358
4966	494	3222	2354
4967	1036	2358	3222
4968	2354	3222	2358
4969	496	3227	3220
4970	1036	3222	3227
4971	494	3220	3222
4972	3227	3222	3220
4973	72	1449	1448
4974	649	3670	1449
4975	648	1448	3670
4976	1449	3670	1448
4977	244	2419	2415
4978	1040	3674	2419
4979	649	2415	3674
4980	2419	3674	2415
4981	236	2367	2371
4982	648	3672	2367
4983	1040	2371	3672
4984	2367	3672	2371
4985	649	3674	3670
4986	1040	3672	3674
4987	648	3670	3672
4988	3674	3672	3670
4989	48	1312	1314
4990	512	3274	1312
4991	514	1314	3274
4992	1312	3274	1314
4993	234	2359	2355
4994	1037	3276	2359
4995	512	2355	3276
4996	2359	3276	2355
4997	244	2414	2418
4998	514	3281	2414
4999	1037	2418	3281
5000	2414	3281	2418

5001	512	3276	3274
5002	1037	3281	3276
5003	514	3274	3281
5004	3276	3281	3274
5005	236	2371	2370
5006	1040	4173	2371
5007	1036	2370	4173
5008	2371	4173	2370
5009	244	2418	2419
5010	1037	4174	2418
5011	1040	2419	4174
5012	2418	4174	2419
5013	234	2358	2359
5014	1036	4172	2358
5015	1037	2359	4172
5016	2358	4172	2359
5017	1040	4174	4173
5018	1037	4172	4174
5019	1036	4173	4172
5020	4174	4172	4173
5021	18	1161	1160
5022	361	2856	1161
5023	360	1160	2856
5024	1161	2856	1160
5025	153	1875	1872
5026	872	2860	1875
5027	361	1872	2860
5028	1875	2860	1872
5029	152	1866	1869
5030	360	2858	1866
5031	872	1869	2858
5032	1866	2858	1869
5033	361	2860	2856
5034	872	2858	2860
5035	360	2856	2858
5036	2860	2858	2856
5037	74	1462	1458
5038	662	3699	1462
5039	658	1458	3699
5040	1462	3699	1458
5041	279	2627	2625
5042	876	3712	2627
5043	662	2625	3712
5044	2627	3712	2625
5045	153	1873	1877
5046	658	3701	1873
5047	876	1877	3701
5048	1873	3701	1877
5049	662	3712	3699
5050	876	3701	3712
5051	658	3699	3701
5052	3712	3701	3699
5053	152	1871	1867
5054	874	3633	1871
5055	635	1867	3633
5056	1871	3633	1867
5057	279	2624	2626
5058	639	3642	2624
5059	874	2626	3642

5060	2624	3642	2626
5061	153	1877	1875
5062	876	4011	1877
5063	872	1875	4011
5064	1877	4011	1875
5065	279	2626	2627
5066	874	4012	2626
5067	876	2627	4012
5068	2626	4012	2627
5069	152	1869	1871
5070	872	4010	1869
5071	874	1871	4010
5072	1869	4010	1871
5073	876	4012	4011
5074	874	4010	4012
5075	872	4011	4010
5076	4012	4010	4011
5077	24	1192	1193
5078	392	2950	1192
5079	393	1193	2950
5080	1192	2950	1193
5081	184	2057	2054
5082	966	2952	2057
5083	392	2054	2952
5084	2057	2952	2054
5085	185	2060	2063
5086	393	2954	2060
5087	966	2063	2954
5088	2060	2954	2063
5089	392	2952	2950
5090	966	2954	2952
5091	393	2950	2954
5092	2952	2954	2950
5093	72	1451	1447
5094	651	3667	1451
5095	647	1447	3667
5096	1451	3667	1447
5097	282	2644	2642
5098	968	3678	2644
5099	651	2642	3678
5100	2644	3678	2642
5101	184	2055	2059
5102	647	3669	2055
5103	968	2059	3669
5104	2055	3669	2059
5105	651	3678	3667
5106	968	3669	3678
5107	647	3667	3669
5108	3678	3669	3667
5109	74	1459	1463
5110	659	3703	1459
5111	663	1463	3703
5112	1459	3703	1463
5113	185	2065	2061
5114	970	3705	2065
5115	659	2061	3705
5116	2065	3705	2061
5117	282	2643	2645
5118	663	3714	2643

5119	970	2645	3714
5120	2643	3714	2645
5121	659	3705	3703
5122	970	3714	3705
5123	663	3703	3714
5124	3705	3714	3703
5125	184	2059	2057
5126	968	4106	2059
5127	966	2057	4106
5128	2059	4106	2057
5129	282	2645	2644
5130	970	4108	2645
5131	968	2644	4108
5132	2645	4108	2644
5133	185	2063	2065
5134	966	4107	2063
5135	970	2065	4107
5136	2063	4107	2065
5137	968	4108	4106
5138	970	4107	4108
5139	966	4106	4107
5140	4108	4107	4106
5141	17	1154	1156
5142	354	2842	1154
5143	356	1156	2842
5144	1154	2842	1156
5145	146	1837	1834
5146	858	2844	1837
5147	354	1834	2844
5148	1837	2844	1834
5149	148	1846	1849
5150	356	2849	1846
5151	858	1849	2849
5152	1846	2849	1849
5153	354	2844	2842
5154	858	2849	2844
5155	356	2842	2849
5156	2844	2849	2842
5157	70	1438	1434
5158	638	3627	1438
5159	634	1434	3627
5160	1438	3627	1434
5161	278	2620	2618
5162	860	3640	2620
5163	638	2618	3640
5164	2620	3640	2618
5165	146	1835	1839
5166	634	3629	1835
5167	860	1839	3629
5168	1835	3629	1839
5169	638	3640	3627
5170	860	3629	3640
5171	634	3627	3629
5172	3640	3629	3627
5173	72	1446	1450
5174	646	3663	1446
5175	650	1450	3663
5176	1446	3663	1450
5177	148	1851	1847

5178	865	3665	1851
5179	646	1847	3665
5180	1851	3665	1847
5181	278	2619	2621
5182	650	3676	2619
5183	865	2621	3676
5184	2619	3676	2621
5185	646	3665	3663
5186	865	3676	3665
5187	650	3663	3676
5188	3665	3676	3663
5189	146	1839	1837
5190	860	3998	1839
5191	858	1837	3998
5192	1839	3998	1837
5193	278	2621	2620
5194	865	4000	2621
5195	860	2620	4000
5196	2621	4000	2620
5197	148	1849	1851
5198	858	3999	1849
5199	865	1851	3999
5200	1849	3999	1851
5201	860	4000	3998
5202	865	3999	4000
5203	858	3998	3999
5204	4000	3999	3998
5205	74	1463	1462
5206	663	3711	1463
5207	662	1462	3711
5208	1463	3711	1462
5209	282	2647	2643
5210	1080	3715	2647
5211	663	2643	3715
5212	2647	3715	2643
5213	279	2625	2629
5214	662	3713	2625
5215	1080	2629	3713
5216	2625	3713	2629
5217	663	3715	3711
5218	1080	3713	3715
5219	662	3711	3713
5220	3715	3713	3711
5221	72	1450	1451
5222	650	3675	1450
5223	651	1451	3675
5224	1450	3675	1451
5225	278	2623	2619
5226	1079	3677	2623
5227	650	2619	3677
5228	2623	3677	2619
5229	282	2642	2646
5230	651	3679	2642
5231	1079	2646	3679
5232	2642	3679	2646
5233	650	3677	3675
5234	1079	3679	3677
5235	651	3675	3679
5236	3677	3679	3675

5237	70	1439	1438
5238	639	3639	1439
5239	638	1438	3639
5240	1439	3639	1438
5241	278	2618	2622
5242	638	3641	2618
5243	1078	2622	3641
5244	2618	3641	2622
5245	282	2646	2647
5246	1079	4216	2646
5247	1080	2647	4216
5248	2646	4216	2647
5249	278	2622	2623
5250	1078	4214	2622
5251	1079	2623	4214
5252	2622	4214	2623
5253	279	2629	2628
5254	1080	4215	2629
5255	1078	2628	4215
5256	2629	4215	2628
5257	1079	4214	4216
5258	1078	4215	4214
5259	1080	4216	4215
5260	4214	4215	4216
5261	2	1093	1094
5262	293	2696	1093
5263	294	1094	2696
5264	1093	2696	1094
5265	85	1522	1520
5266	712	2697	1522
5267	293	1520	2697
5268	1522	2697	1520
5269	86	1524	1526
5270	294	2698	1524
5271	712	1526	2698
5272	1524	2698	1526
5273	293	2697	2696
5274	712	2698	2697
5275	294	2696	2698
5276	2697	2698	2696
5277	29	1218	1216
5278	418	3014	1218
5279	416	1216	3014
5280	1218	3014	1216
5281	200	2152	2150
5282	713	3019	2152
5283	418	2150	3019
5284	2152	3019	2150
5285	85	1521	1523
5286	416	3015	1521
5287	713	1523	3015
5288	1521	3015	1523
5289	418	3019	3014
5290	713	3015	3019
5291	416	3014	3015
5292	3019	3015	3014
5293	30	1220	1222
5294	420	3023	1220
5295	422	1222	3023

5296	1220	3023	1222
5297	86	1527	1525
5298	714	3024	1527
5299	420	1525	3024
5300	1527	3024	1525
5301	200	2151	2153
5302	422	3028	2151
5303	714	2153	3028
5304	2151	3028	2153
5305	420	3024	3023
5306	714	3028	3024
5307	422	3023	3028
5308	3024	3028	3023
5309	85	1523	1522
5310	713	3848	1523
5311	712	1522	3848
5312	1523	3848	1522
5313	200	2153	2152
5314	714	3850	2153
5315	713	2152	3850
5316	2153	3850	2152
5317	86	1526	1527
5318	712	3849	1526
5319	714	1527	3849
5320	1526	3849	1527
5321	713	3850	3848
5322	714	3849	3850
5323	712	3848	3849
5324	3850	3849	3848
5325	13	1138	1136
5326	338	2798	1138
5327	336	1136	2798
5328	1138	2798	1136
5329	130	1752	1750
5330	814	2803	1752
5331	338	1750	2803
5332	1752	2803	1750
5333	128	1742	1744
5334	336	2799	1742
5335	814	1744	2799
5336	1742	2799	1744
5337	338	2803	2798
5338	814	2799	2803
5339	336	2798	2799
5340	2803	2799	2798
5341	64	1400	1398
5342	600	3518	1400
5343	598	1398	3518
5344	1400	3518	1398
5345	201	2159	2157
5346	819	3527	2159
5347	600	2157	3527
5348	2159	3527	2157
5349	130	1751	1754
5350	598	3520	1751
5351	819	1754	3520
5352	1751	3520	1754
5353	600	3527	3518
5354	819	3520	3527

5355	598	3518	3520
5356	3527	3520	3518
5357	29	1217	1219
5358	417	3016	1217
5359	419	1219	3016
5360	1217	3016	1219
5361	128	1745	1743
5362	815	3017	1745
5363	417	1743	3017
5364	1745	3017	1743
5365	201	2156	2158
5366	419	3021	2156
5367	815	2158	3021
5368	2156	3021	2158
5369	417	3017	3016
5370	815	3021	3017
5371	419	3016	3021
5372	3017	3021	3016
5373	130	1754	1752
5374	819	3951	1754
5375	814	1752	3951
5376	1754	3951	1752
5377	201	2158	2159
5378	815	3952	2158
5379	819	2159	3952
5380	2158	3952	2159
5381	128	1744	1745
5382	814	3950	1744
5383	815	1745	3950
5384	1744	3950	1745
5385	819	3952	3951
5386	815	3950	3952
5387	814	3951	3950
5388	3952	3950	3951
5389	14	1140	1142
5390	340	2807	1140
5391	342	1142	2807
5392	1140	2807	1142
5393	132	1764	1762
5394	823	2808	1764
5395	340	1762	2808
5396	1764	2808	1762
5397	134	1770	1772
5398	342	2812	1770
5399	823	1772	2812
5400	1770	2812	1772
5401	340	2808	2807
5402	823	2812	2808
5403	342	2807	2812
5404	2808	2812	2807
5405	202	2164	2162
5406	824	3030	2164
5407	423	2162	3030
5408	2164	3030	2162
5409	132	1763	1765
5410	421	3026	1763
5411	824	1765	3026
5412	1763	3026	1765
5413	134	1774	1771

5414	828	3524	1774
5415	599	1771	3524
5416	1774	3524	1771
5417	132	1765	1764
5418	824	3959	1765
5419	823	1764	3959
5420	1765	3959	1764
5421	202	2165	2164
5422	828	3961	2165
5423	824	2164	3961
5424	2165	3961	2164
5425	134	1772	1774
5426	823	3960	1772
5427	828	1774	3960
5428	1772	3960	1774
5429	824	3961	3959
5430	828	3960	3961
5431	823	3959	3960
5432	3961	3960	3959
5433	29	1219	1218
5434	419	3018	1219
5435	418	1218	3018
5436	1219	3018	1218
5437	201	2160	2156
5438	1000	3022	2160
5439	419	2156	3022
5440	2160	3022	2156
5441	200	2150	2154
5442	418	3020	2150
5443	1000	2154	3020
5444	2150	3020	2154
5445	419	3022	3018
5446	1000	3020	3022
5447	418	3018	3020
5448	3022	3020	3018
5449	200	2155	2151
5450	1001	3029	2155
5451	422	2151	3029
5452	2155	3029	2151
5453	5	1103	1101
5454	303	2710	1103
5455	301	1101	2710
5456	1103	2710	1101
5457	95	1566	1564
5458	726	2715	1566
5459	303	1564	2715
5460	1566	2715	1564
5461	93	1554	1556
5462	301	2711	1554
5463	726	1556	2711
5464	1554	2711	1556
5465	303	2715	2710
5466	726	2711	2715
5467	301	2710	2711
5468	2715	2711	2710
5469	39	1262	1260
5470	462	3122	1262
5471	460	1260	3122
5472	1262	3122	1260

5473	214	2237	2235
5474	731	3131	2237
5475	462	2235	3131
5476	2237	3131	2235
5477	95	1565	1568
5478	460	3124	1565
5479	731	1568	3124
5480	1565	3124	1568
5481	462	3131	3122
5482	731	3124	3131
5483	460	3122	3124
5484	3131	3124	3122
5485	37	1250	1252
5486	450	3095	1250
5487	452	1252	3095
5488	1250	3095	1252
5489	93	1557	1555
5490	727	3096	1557
5491	450	1555	3096
5492	1557	3096	1555
5493	214	2234	2236
5494	452	3100	2234
5495	727	2236	3100
5496	2234	3100	2236
5497	450	3096	3095
5498	727	3100	3096
5499	452	3095	3100
5500	3096	3100	3095
5501	95	1568	1566
5502	731	3864	1568
5503	726	1566	3864
5504	1568	3864	1566
5505	214	2236	2237
5506	727	3865	2236
5507	731	2237	3865
5508	2236	3865	2237
5509	93	1556	1557
5510	726	3863	1556
5511	727	1557	3863
5512	1556	3863	1557
5513	731	3865	3864
5514	727	3863	3865
5515	726	3864	3863
5516	3865	3863	3864
5517	21	1174	1172
5518	374	2888	1174
5519	372	1172	2888
5520	1174	2888	1172
5521	166	1948	1946
5522	904	2897	1948
5523	374	1946	2897
5524	1948	2897	1946
5525	164	1934	1936
5526	372	2890	1934
5527	904	1936	2890
5528	1934	2890	1936
5529	374	2897	2888
5530	904	2890	2897
5531	372	2888	2890

5532	2897	2890	2888
5533	65	1407	1405
5534	607	3540	1407
5535	605	1405	3540
5536	1407	3540	1405
5537	219	2267	2265
5538	913	3547	2267
5539	607	2265	3547
5540	2267	3547	2265
5541	166	1947	1950
5542	605	3542	1947
5543	913	1950	3542
5544	1947	3542	1950
5545	607	3547	3540
5546	913	3542	3547
5547	605	3540	3542
5548	3547	3542	3540
5549	39	1261	1264
5550	461	3126	1261
5551	464	1264	3126
5552	1261	3126	1264
5553	164	1938	1935
5554	906	3128	1938
5555	461	1935	3128
5556	1938	3128	1935
5557	219	2264	2266
5558	464	3136	2264
5559	906	2266	3136
5560	2264	3136	2266
5561	461	3128	3126
5562	906	3136	3128
5563	464	3126	3136
5564	3128	3136	3126
5565	166	1950	1948
5566	913	4041	1950
5567	904	1948	4041
5568	1950	4041	1948
5569	219	2266	2267
5570	906	4044	2266
5571	913	2267	4044
5572	2266	4044	2267
5573	164	1936	1938
5574	904	4040	1936
5575	906	1938	4040
5576	1936	4040	1938
5577	913	4044	4041
5578	906	4040	4044
5579	904	4041	4040
5580	4044	4040	4041
5581	13	1137	1139
5582	337	2800	1137
5583	339	1139	2800
5584	1137	2800	1139
5585	129	1748	1746
5586	816	2801	1748
5587	337	1746	2801
5588	1748	2801	1746
5589	131	1756	1758
5590	339	2805	1756

5591	816	1758	2805
5592	1756	2805	1758
5593	337	2801	2800
5594	816	2805	2801
5595	339	2800	2805
5596	2801	2805	2800
5597	37	1253	1251
5598	453	3097	1253
5599	451	1251	3097
5600	1253	3097	1251
5601	215	2242	2240
5602	817	3102	2242
5603	453	2240	3102
5604	2242	3102	2240
5605	129	1747	1749
5606	451	3098	1747
5607	817	1749	3098
5608	1747	3098	1749
5609	453	3102	3097
5610	817	3098	3102
5611	451	3097	3098
5612	3102	3098	3097
5613	39	1264	1262
5614	464	3130	1264
5615	462	1262	3130
5616	1264	3130	1262
5617	219	2268	2264
5618	1016	3137	2268
5619	464	2264	3137
5620	2268	3137	2264
5621	214	2235	2239
5622	462	3132	2235
5623	1016	2239	3132
5624	2235	3132	2239
5625	464	3137	3130
5626	1016	3132	3137
5627	462	3130	3132
5628	3137	3132	3130
5629	37	1252	1253
5630	452	3099	1252
5631	453	1253	3099
5632	1252	3099	1253
5633	214	2238	2234
5634	1015	3101	2238
5635	452	2234	3101
5636	2238	3101	2234
5637	215	2240	2244
5638	453	3103	2240
5639	1015	2244	3103
5640	2240	3103	2244
5641	452	3101	3099
5642	1015	3103	3101
5643	453	3099	3103
5644	3101	3103	3099
5645	215	2244	2245
5646	1015	4152	2244
5647	1017	2245	4152
5648	2244	4152	2245
5649	214	2239	2238

5650	1016	4151	2239
5651	1015	2238	4151
5652	2239	4151	2238
5653	1017	4152	4153
5654	1015	4151	4152
5655	1016	4153	4151
5656	4152	4151	4153
5657	8	1114	1116
5658	314	2744	1114
5659	316	1116	2744
5660	1114	2744	1116
5661	106	1628	1626
5662	760	2745	1628
5663	314	1626	2745
5664	1628	2745	1626
5665	108	1634	1636
5666	316	2749	1634
5667	760	1636	2749
5668	1634	2749	1636
5669	314	2745	2744
5670	760	2749	2745
5671	316	2744	2749
5672	2745	2749	2744
5673	50	1324	1322
5674	524	3302	1324
5675	522	1322	3302
5676	1324	3302	1322
5677	106	1627	1629
5678	522	3303	1627
5679	761	1629	3303
5680	1627	3303	1629
5681	524	3307	3302
5682	761	3303	3307
5683	522	3302	3303
5684	3307	3303	3302
5685	52	1330	1332
5686	530	3320	1330
5687	532	1332	3320
5688	1330	3320	1332
5689	108	1638	1635
5690	765	3322	1638
5691	530	1635	3322
5692	1638	3322	1635
5693	530	3322	3320
5694	765	3329	3322
5695	532	3320	3329
5696	3322	3329	3320
5697	106	1629	1628
5698	761	3896	1629
5699	760	1628	3896
5700	1629	3896	1628
5701	108	1636	1638
5702	760	3897	1636
5703	765	1638	3897
5704	1636	3897	1638
5705	761	3898	3896
5706	765	3897	3898
5707	760	3896	3897
5708	3898	3897	3896

5709	14	1143	1141
5710	343	2809	1143
5711	341	1141	2809
5712	1143	2809	1141
5713	135	1778	1776
5714	825	2814	1778
5715	343	1776	2814
5716	1778	2814	1776
5717	133	1766	1768
5718	341	2810	1766
5719	825	1768	2810
5720	1766	2810	1768
5721	343	2814	2809
5722	825	2810	2814
5723	341	2809	2810
5724	2814	2810	2809
5725	66	1412	1410
5726	612	3554	1412
5727	610	1410	3554
5728	1412	3554	1410
5729	249	2447	2445
5730	830	3563	2447
5731	612	2445	3563
5732	2447	3563	2445
5733	135	1777	1780
5734	610	3556	1777
5735	830	1780	3556
5736	1777	3556	1780
5737	612	3563	3554
5738	830	3556	3563
5739	610	3554	3556
5740	3563	3556	3554
5741	133	1769	1767
5742	826	3305	1769
5743	523	1767	3305
5744	1769	3305	1767
5745	249	2444	2446
5746	525	3309	2444
5747	826	2446	3309
5748	2444	3309	2446
5749	523	3305	3304
5750	826	3309	3305
5751	525	3304	3309
5752	3305	3309	3304
5753	135	1780	1778
5754	830	3963	1780
5755	825	1778	3963
5756	1780	3963	1778
5757	249	2446	2447
5758	826	3964	2446
5759	830	2447	3964
5760	2446	3964	2447
5761	133	1768	1769
5762	825	3962	1768
5763	826	1769	3962
5764	1768	3962	1769
5765	830	3964	3963
5766	826	3962	3964
5767	825	3963	3962

5768	3964	3962	3963
5769	21	1173	1175
5770	373	2892	1173
5771	375	1175	2892
5772	1173	2892	1175
5773	165	1942	1940
5774	908	2894	1942
5775	373	1940	2894
5776	1942	2894	1940
5777	167	1952	1954
5778	375	2899	1952
5779	908	1954	2899
5780	1952	2899	1954
5781	373	2894	2892
5782	908	2899	2894
5783	375	2892	2899
5784	2894	2899	2892
5785	52	1334	1331
5786	534	3324	1334
5787	531	1331	3324
5788	1334	3324	1331
5789	253	2470	2468
5790	910	3334	2470
5791	534	2468	3334
5792	2470	3334	2468
5793	165	1941	1944
5794	531	3326	1941
5795	910	1944	3326
5796	1941	3326	1944
5797	534	3334	3324
5798	910	3326	3334
5799	531	3324	3326
5800	3334	3326	3324
5801	66	1411	1413
5802	611	3558	1411
5803	613	1413	3558
5804	1411	3558	1413
5805	167	1956	1953
5806	915	3560	1956
5807	611	1953	3560
5808	1956	3560	1953
5809	253	2469	2471
5810	613	3565	2469
5811	915	2471	3565
5812	2469	3565	2471
5813	611	3560	3558
5814	915	3565	3560
5815	613	3558	3565
5816	3560	3565	3558
5817	165	1944	1942
5818	910	4046	1944
5819	908	1942	4046
5820	1944	4046	1942
5821	253	2471	2470
5822	915	4050	2471
5823	910	2470	4050
5824	2471	4050	2470
5825	167	1954	1956
5826	908	4047	1954

5827	915	1956	4047
5828	1954	4047	1956
5829	910	4050	4046
5830	915	4047	4050
5831	908	4046	4047
5832	4050	4047	4046
5833	249	2448	2444
5834	1048	3310	2448
5835	525	2444	3310
5836	2448	3310	2444
5837	66	1413	1412
5838	613	3562	1413
5839	612	1412	3562
5840	1413	3562	1412
5841	253	2473	2469
5842	1050	3566	2473
5843	613	2469	3566
5844	2473	3566	2469
5845	249	2445	2449
5846	612	3564	2445
5847	1050	2449	3564
5848	2445	3564	2449
5849	613	3566	3562
5850	1050	3564	3566
5851	612	3562	3564
5852	3566	3564	3562
5853	52	1332	1334
5854	532	3328	1332
5855	534	1334	3328
5856	1332	3328	1334
5857	253	2468	2472
5858	534	3335	2468
5859	1049	2472	3335
5860	2468	3335	2472
5861	532	3330	3328
5862	1049	3335	3330
5863	534	3328	3335
5864	3330	3335	3328
5865	249	2449	2448
5866	1050	4185	2449
5867	1048	2448	4185
5868	2449	4185	2448
5869	253	2472	2473
5870	1049	4186	2472
5871	1050	2473	4186
5872	2472	4186	2473
5873	1050	4186	4185
5874	1049	4184	4186
5875	1048	4185	4184
5876	4186	4184	4185
5877	13	1139	1138
5878	339	2802	1139
5879	338	1138	2802
5880	1139	2802	1138
5881	131	1759	1756
5882	818	2806	1759
5883	339	1756	2806
5884	1759	2806	1756
5885	130	1750	1753

5886	338	2804	1750
5887	818	1753	2804
5888	1750	2804	1753
5889	339	2806	2802
5890	818	2804	2806
5891	338	2802	2804
5892	2806	2804	2802
5893	65	1408	1404
5894	608	3537	1408
5895	604	1404	3537
5896	1408	3537	1404
5897	272	2585	2583
5898	822	3550	2585
5899	608	2583	3550
5900	2585	3550	2583
5901	131	1757	1761
5902	604	3539	1757
5903	822	1761	3539
5904	1757	3539	1761
5905	608	3550	3537
5906	822	3539	3550
5907	604	3537	3539
5908	3550	3539	3537
5909	64	1398	1402
5910	598	3519	1398
5911	602	1402	3519
5912	1398	3519	1402
5913	130	1755	1751
5914	820	3521	1755
5915	598	1751	3521
5916	1755	3521	1751
5917	272	2582	2584
5918	602	3532	2582
5919	820	2584	3532
5920	2582	3532	2584
5921	598	3521	3519
5922	820	3532	3521
5923	602	3519	3532
5924	3521	3532	3519
5925	131	1761	1759
5926	822	3957	1761
5927	818	1759	3957
5928	1761	3957	1759
5929	272	2584	2585
5930	820	3958	2584
5931	822	2585	3958
5932	2584	3958	2585
5933	130	1753	1755
5934	818	3956	1753
5935	820	1755	3956
5936	1753	3956	1755
5937	822	3958	3957
5938	820	3956	3958
5939	818	3957	3956
5940	3958	3956	3957
5941	21	1175	1174
5942	375	2896	1175
5943	374	1174	2896
5944	1175	2896	1174

5945	167	1955	1952
5946	912	2900	1955
5947	375	1952	2900
5948	1955	2900	1952
5949	166	1946	1949
5950	374	2898	1946
5951	912	1949	2898
5952	1946	2898	1949
5953	375	2900	2896
5954	912	2898	2900
5955	374	2896	2898
5956	2900	2898	2896
5957	66	1415	1411
5958	615	3559	1415
5959	611	1411	3559
5960	1415	3559	1411
5961	274	2597	2595
5962	916	3570	2597
5963	615	2595	3570
5964	2597	3570	2595
5965	167	1953	1957
5966	611	3561	1953
5967	916	1957	3561
5968	1953	3561	1957
5969	615	3570	3559
5970	916	3561	3570
5971	611	3559	3561
5972	3570	3561	3559
5973	65	1405	1409
5974	605	3541	1405
5975	609	1409	3541
5976	1405	3541	1409
5977	166	1951	1947
5978	914	3543	1951
5979	605	1947	3543
5980	1951	3543	1947
5981	274	2594	2596
5982	609	3552	2594
5983	914	2596	3552
5984	2594	3552	2596
5985	605	3543	3541
5986	914	3552	3543
5987	609	3541	3552
5988	3543	3552	3541
5989	167	1957	1955
5990	916	4053	1957
5991	912	1955	4053
5992	1957	4053	1955
5993	274	2596	2597
5994	914	4054	2596
5995	916	2597	4054
5996	2596	4054	2597
5997	166	1949	1951
5998	912	4052	1949
5999	914	1951	4052
6000	1949	4052	1951
6001	916	4054	4053
6002	914	4052	4054
6003	912	4053	4052

6004	4054	4052	4053
6005	14	1142	1143
6006	342	2811	1142
6007	343	1143	2811
6008	1142	2811	1143
6009	134	1773	1770
6010	827	2813	1773
6011	342	1770	2813
6012	1773	2813	1770
6013	135	1776	1779
6014	343	2815	1776
6015	827	1779	2815
6016	1776	2815	1779
6017	342	2813	2811
6018	827	2815	2813
6019	343	2811	2815
6020	2813	2815	2811
6021	64	1403	1399
6022	603	3523	1403
6023	599	1399	3523
6024	1403	3523	1399
6025	273	2590	2588
6026	829	3534	2590
6027	603	2588	3534
6028	2590	3534	2588
6029	134	1771	1775
6030	599	3525	1771
6031	829	1775	3525
6032	1771	3525	1775
6033	603	3534	3523
6034	829	3525	3534
6035	599	3523	3525
6036	3534	3525	3523
6037	66	1410	1414
6038	610	3555	1410
6039	614	1414	3555
6040	1410	3555	1414
6041	135	1781	1777
6042	831	3557	1781
6043	610	1777	3557
6044	1781	3557	1777
6045	273	2589	2591
6046	614	3568	2589
6047	831	2591	3568
6048	2589	3568	2591
6049	610	3557	3555
6050	831	3568	3557
6051	614	3555	3568
6052	3557	3568	3555
6053	134	1775	1773
6054	829	3965	1775
6055	827	1773	3965
6056	1775	3965	1773
6057	273	2591	2590
6058	831	3967	2591
6059	829	2590	3967
6060	2591	3967	2590
6061	135	1779	1781
6062	827	3966	1779

6063	831	1781	3966
6064	1779	3966	1781
6065	829	3967	3965
6066	831	3966	3967
6067	827	3965	3966
6068	3967	3966	3965
6069	65	1409	1408
6070	609	3549	1409
6071	608	1408	3549
6072	1409	3549	1408
6073	274	2598	2594
6074	1073	3553	2598
6075	609	2594	3553
6076	2598	3553	2594
6077	272	2583	2587
6078	608	3551	2583
6079	1073	2587	3551
6080	2583	3551	2587
6081	609	3553	3549
6082	1073	3551	3553
6083	608	3549	3551
6084	3553	3551	3549
6085	66	1414	1415
6086	614	3567	1414
6087	615	1415	3567
6088	1414	3567	1415
6089	273	2593	2589
6090	1074	3569	2593
6091	614	2589	3569
6092	2593	3569	2589
6093	274	2595	2599
6094	615	3571	2595
6095	1074	2599	3571
6096	2595	3571	2599
6097	614	3569	3567
6098	1074	3571	3569
6099	615	3567	3571
6100	3569	3571	3567
6101	64	1402	1403
6102	602	3531	1402
6103	603	1403	3531
6104	1402	3531	1403
6105	272	2586	2582
6106	1072	3533	2586
6107	602	2582	3533
6108	2586	3533	2582
6109	273	2588	2592
6110	603	3535	2588
6111	1072	2592	3535
6112	2588	3535	2592
6113	602	3533	3531
6114	1072	3535	3533
6115	603	3531	3535
6116	3533	3535	3531
6117	274	2599	2598
6118	1074	4210	2599
6119	1073	2598	4210
6120	2599	4210	2598
6121	273	2592	2593

6122	1072	4209	2592
6123	1074	2593	4209
6124	2592	4209	2593
6125	272	2587	2586
6126	1073	4208	2587
6127	1072	2586	4208
6128	2587	4208	2586
6129	1074	4209	4210
6130	1072	4208	4209
6131	1073	4210	4208
6132	4209	4208	4210
6133	7	1112	1110
6134	312	2734	1112
6135	310	1110	2734
6136	1112	2734	1110
6137	104	1617	1614
6138	750	2741	1617
6139	312	1614	2741
6140	1617	2741	1614
6141	102	1602	1605
6142	310	2736	1602
6143	750	1605	2736
6144	1602	2736	1605
6145	312	2741	2734
6146	750	2736	2741
6147	310	2734	2736
6148	2741	2736	2734
6149	48	1313	1310
6150	513	3267	1313
6151	510	1310	3267
6152	1313	3267	1310
6153	238	2381	2379
6154	757	3278	2381
6155	513	2379	3278
6156	2381	3278	2379
6157	104	1615	1619
6158	510	3269	1615
6159	757	1619	3269
6160	1615	3269	1619
6161	513	3278	3267
6162	757	3269	3278
6163	510	3267	3269
6164	3278	3269	3267
6165	46	1298	1301
6166	498	3231	1298
6167	501	1301	3231
6168	1298	3231	1301
6169	102	1607	1603
6170	752	3233	1607
6171	498	1603	3233
6172	1607	3233	1603
6173	238	2378	2380
6174	501	3242	2378
6175	752	2380	3242
6176	2378	3242	2380
6177	498	3233	3231
6178	752	3242	3233
6179	501	3231	3242
6180	3233	3242	3231

6181	104	1619	1617
6182	757	3891	1619
6183	750	1617	3891
6184	1619	3891	1617
6185	238	2380	2381
6186	752	3892	2380
6187	757	2381	3892
6188	2380	3892	2381
6189	102	1605	1607
6190	750	3890	1605
6191	752	1607	3890
6192	1605	3890	1607
6193	757	3892	3891
6194	752	3890	3892
6195	750	3891	3890
6196	3892	3890	3891
6197	24	1194	1190
6198	394	2943	1194
6199	390	1190	2943
6200	1194	2943	1190
6201	186	2068	2066
6202	959	2956	2068
6203	394	2066	2956
6204	2068	2956	2066
6205	182	2042	2045
6206	390	2945	2042
6207	959	2045	2945
6208	2042	2945	2045
6209	394	2956	2943
6210	959	2945	2956
6211	390	2943	2945
6212	2956	2945	2943
6213	77	1479	1477
6214	679	3756	1479
6215	677	1477	3756
6216	1479	3756	1477
6217	245	2423	2421
6218	972	3763	2423
6219	679	2421	3763
6220	2423	3763	2421
6221	186	2067	2070
6222	677	3758	2067
6223	972	2070	3758
6224	2067	3758	2070
6225	679	3763	3756
6226	972	3758	3763
6227	677	3756	3758
6228	3763	3758	3756
6229	48	1311	1315
6230	511	3271	1311
6231	515	1315	3271
6232	1311	3271	1315
6233	182	2047	2043
6234	961	3273	2047
6235	511	2043	3273
6236	2047	3273	2043
6237	245	2420	2422
6238	515	3282	2420
6239	961	2422	3282

6240	2420	3282	2422
6241	511	3273	3271
6242	961	3282	3273
6243	515	3271	3282
6244	3273	3282	3271
6245	186	2070	2068
6246	972	4097	2070
6247	959	2068	4097
6248	2070	4097	2068
6249	245	2422	2423
6250	961	4099	2422
6251	972	2423	4099
6252	2422	4099	2423
6253	182	2045	2047
6254	959	4096	2045
6255	961	2047	4096
6256	2045	4096	2047
6257	972	4099	4097
6258	961	4096	4099
6259	959	4097	4096
6260	4099	4096	4097
6261	20	1167	1171
6262	367	2875	1167
6263	371	1171	2875
6264	1167	2875	1171
6265	159	1907	1904
6266	891	2877	1907
6267	367	1904	2877
6268	1907	2877	1904
6269	163	1928	1930
6270	371	2886	1928
6271	891	1930	2886
6272	1928	2886	1930
6273	367	2877	2875
6274	891	2886	2877
6275	371	2875	2886
6276	2877	2886	2875
6277	46	1303	1299
6278	503	3235	1303
6279	499	1299	3235
6280	1303	3235	1299
6281	240	2392	2390
6282	893	3246	2392
6283	503	2390	3246
6284	2392	3246	2390
6285	159	1905	1909
6286	499	3237	1905
6287	893	1909	3237
6288	1905	3237	1909
6289	503	3246	3235
6290	893	3237	3246
6291	499	3235	3237
6292	3246	3237	3235
6293	77	1476	1478
6294	676	3752	1476
6295	678	1478	3752
6296	1476	3752	1478
6297	163	1932	1929
6298	902	3754	1932

6299	676	1929	3754
6300	1932	3754	1929
6301	240	2391	2393
6302	678	3761	2391
6303	902	2393	3761
6304	2391	3761	2393
6305	676	3754	3752
6306	902	3761	3754
6307	678	3752	3761
6308	3754	3761	3752
6309	159	1909	1907
6310	893	4030	1909
6311	891	1907	4030
6312	1909	4030	1907
6313	240	2393	2392
6314	902	4033	2393
6315	893	2392	4033
6316	2393	4033	2392
6317	163	1930	1932
6318	891	4031	1930
6319	902	1932	4031
6320	1930	4031	1932
6321	893	4033	4030
6322	902	4031	4033
6323	891	4030	4031
6324	4033	4031	4030
6325	48	1315	1313
6326	515	3277	1315
6327	513	1313	3277
6328	1315	3277	1313
6329	245	2424	2420
6330	1043	3283	2424
6331	515	2420	3283
6332	2424	3283	2420
6333	238	2379	2383
6334	513	3279	2379
6335	1043	2383	3279
6336	2379	3279	2383
6337	515	3283	3277
6338	1043	3279	3283
6339	513	3277	3279
6340	3283	3279	3277
6341	46	1301	1303
6342	501	3241	1301
6343	503	1303	3241
6344	1301	3241	1303
6345	238	2382	2378
6346	1042	3243	2382
6347	501	2378	3243
6348	2382	3243	2378
6349	240	2390	2394
6350	503	3247	2390
6351	1042	2394	3247
6352	2390	3247	2394
6353	501	3243	3241
6354	1042	3247	3243
6355	503	3241	3247
6356	3243	3247	3241
6357	8	1116	1117

6358	316	2748	1116
6359	317	1117	2748
6360	1116	2748	1117
6361	108	1637	1634
6362	764	2750	1637
6363	316	1634	2750
6364	1637	2750	1634
6365	109	1640	1643
6366	317	2752	1640
6367	764	1643	2752
6368	1640	2752	1643
6369	316	2750	2748
6370	764	2752	2750
6371	317	2748	2752
6372	2750	2752	2748
6373	52	1333	1330
6374	533	3321	1333
6375	530	1330	3321
6376	1333	3321	1330
6377	108	1635	1639
6378	530	3323	1635
6379	766	1639	3323
6380	1635	3323	1639
6381	533	3332	3321
6382	766	3323	3332
6383	530	3321	3323
6384	3332	3323	3321
6385	53	1336	1339
6386	536	3339	1336
6387	539	1339	3339
6388	1336	3339	1339
6389	109	1645	1641
6390	768	3341	1645
6391	536	1641	3341
6392	1645	3341	1641
6393	536	3341	3339
6394	768	3350	3341
6395	539	3339	3350
6396	3341	3350	3339
6397	108	1639	1637
6398	766	3902	1639
6399	764	1637	3902
6400	1639	3902	1637
6401	109	1643	1645
6402	764	3903	1643
6403	768	1645	3903
6404	1643	3903	1645
6405	766	3904	3902
6406	768	3903	3904
6407	764	3902	3903
6408	3904	3903	3902
6409	21	1177	1173
6410	377	2893	1177
6411	373	1173	2893
6412	1177	2893	1173
6413	169	1966	1964
6414	909	2904	1966
6415	377	1964	2904
6416	1966	2904	1964

6417	165	1940	1943
6418	373	2895	1940
6419	909	1943	2895
6420	1940	2895	1943
6421	377	2904	2893
6422	909	2895	2904
6423	373	2893	2895
6424	2904	2895	2893
6425	52	1331	1335
6426	531	3325	1331
6427	535	1335	3325
6428	1331	3325	1335
6429	165	1945	1941
6430	911	3327	1945
6431	531	1941	3327
6432	1945	3327	1941
6433	169	1968	1966
6434	920	4049	1968
6435	909	1966	4049
6436	1968	4049	1966
6437	254	2476	2477
6438	911	4051	2476
6439	920	2477	4051
6440	2476	4051	2477
6441	165	1943	1945
6442	909	4048	1943
6443	911	1945	4048
6444	1943	4048	1945
6445	920	4051	4049
6446	911	4048	4051
6447	909	4049	4048
6448	4051	4048	4049
6449	24	1191	1195
6450	391	2947	1191
6451	395	1195	2947
6452	1191	2947	1195
6453	183	2051	2048
6454	963	2949	2051
6455	391	2048	2949
6456	2051	2949	2048
6457	187	2072	2074
6458	395	2958	2072
6459	963	2074	2958
6460	2072	2958	2074
6461	391	2949	2947
6462	963	2958	2949
6463	395	2947	2958
6464	2949	2958	2947
6465	53	1341	1337
6466	541	3343	1341
6467	537	1337	3343
6468	1341	3343	1337
6469	183	2049	2053
6470	537	3345	2049
6471	965	2053	3345
6472	2049	3345	2053
6473	541	3354	3343
6474	965	3345	3354
6475	537	3343	3345

6476	3354	3345	3343
6477	183	2053	2051
6478	965	4102	2053
6479	963	2051	4102
6480	2053	4102	2051
6481	187	2074	2076
6482	963	4103	2074
6483	974	2076	4103
6484	2074	4103	2076
6485	965	4105	4102
6486	974	4103	4105
6487	963	4102	4103
6488	4105	4103	4102
6489	52	1335	1333
6490	535	3331	1335
6491	533	1333	3331
6492	1335	3331	1333
6493	535	3337	3331
6494	1054	3333	3337
6495	533	3331	3333
6496	3337	3333	3331
6497	53	1339	1341
6498	539	3349	1339
6499	541	1341	3349
6500	1339	3349	1341
6501	539	3351	3349
6502	1055	3355	3351
6503	541	3349	3355
6504	3351	3355	3349
6505	1056	4192	4191
6506	1055	4190	4192
6507	1054	4191	4190
6508	4192	4190	4191
6509	5	1102	1103
6510	302	2712	1102
6511	303	1103	2712
6512	1102	2712	1103
6513	94	1561	1558
6514	728	2714	1561
6515	302	1558	2714
6516	1561	2714	1558
6517	95	1564	1567
6518	303	2716	1564
6519	728	1567	2716
6520	1564	2716	1567
6521	302	2714	2712
6522	728	2716	2714
6523	303	2712	2716
6524	2714	2716	2712
6525	38	1257	1254
6526	457	3105	1257
6527	454	1254	3105
6528	1257	3105	1254
6529	216	2248	2246
6530	730	3116	2248
6531	457	2246	3116
6532	2248	3116	2246
6533	94	1559	1563
6534	454	3107	1559

6535	730	1563	3107
6536	1559	3107	1563
6537	457	3116	3105
6538	730	3107	3116
6539	454	3105	3107
6540	3116	3107	3105
6541	39	1260	1263
6542	460	3123	1260
6543	463	1263	3123
6544	1260	3123	1263
6545	95	1569	1565
6546	732	3125	1569
6547	460	1565	3125
6548	1569	3125	1565
6549	216	2247	2249
6550	463	3134	2247
6551	732	2249	3134
6552	2247	3134	2249
6553	460	3125	3123
6554	732	3134	3125
6555	463	3123	3134
6556	3125	3134	3123
6557	94	1563	1561
6558	730	3866	1563
6559	728	1561	3866
6560	1563	3866	1561
6561	216	2249	2248
6562	732	3868	2249
6563	730	2248	3868
6564	2249	3868	2248
6565	95	1567	1569
6566	728	3867	1567
6567	732	1569	3867
6568	1567	3867	1569
6569	730	3868	3866
6570	732	3867	3868
6571	728	3866	3867
6572	3868	3867	3866
6573	20	1170	1166
6574	370	2871	1170
6575	366	1166	2871
6576	1170	2871	1166
6577	162	1924	1922
6578	887	2884	1924
6579	370	1922	2884
6580	1924	2884	1922
6581	158	1898	1901
6582	366	2873	1898
6583	887	1901	2873
6584	1898	2873	1901
6585	370	2884	2871
6586	887	2873	2884
6587	366	2871	2873
6588	2884	2873	2871
6589	162	1926	1924
6590	900	4025	1926
6591	887	1924	4025
6592	1926	4025	1924
6593	218	2260	2261

6594	889	4027	2260
6595	900	2261	4027
6596	2260	4027	2261
6597	158	1901	1903
6598	887	4024	1901
6599	889	1903	4024
6600	1901	4024	1903
6601	900	4027	4025
6602	889	4024	4027
6603	887	4025	4024
6604	4027	4024	4025
6605	21	1172	1176
6606	372	2889	1172
6607	376	1176	2889
6608	1172	2889	1176
6609	164	1937	1934
6610	905	2891	1937
6611	372	1934	2891
6612	1937	2891	1934
6613	168	1958	1960
6614	376	2902	1958
6615	905	1960	2902
6616	1958	2902	1960
6617	372	2891	2889
6618	905	2902	2891
6619	376	2889	2902
6620	2891	2902	2889
6621	39	1265	1261
6622	465	3127	1265
6623	461	1261	3127
6624	1265	3127	1261
6625	220	2272	2270
6626	907	3138	2272
6627	465	2270	3138
6628	2272	3138	2270
6629	164	1935	1939
6630	461	3129	1935
6631	907	1939	3129
6632	1935	3129	1939
6633	465	3138	3127
6634	907	3129	3138
6635	461	3127	3129
6636	3138	3129	3127
6637	76	1471	1473
6638	671	3738	1471
6639	673	1473	3738
6640	1471	3738	1473
6641	168	1962	1959
6642	918	3740	1962
6643	671	1959	3740
6644	1962	3740	1959
6645	220	2271	2273
6646	673	3745	2271
6647	918	2273	3745
6648	2271	3745	2273
6649	671	3740	3738
6650	918	3745	3740
6651	673	3738	3745
6652	3740	3745	3738

6653	164	1939	1937
6654	907	4042	1939
6655	905	1937	4042
6656	1939	4042	1937
6657	220	2273	2272
6658	918	4045	2273
6659	907	2272	4045
6660	2273	4045	2272
6661	168	1960	1962
6662	905	4043	1960
6663	918	1962	4043
6664	1960	4043	1962
6665	907	4045	4042
6666	918	4043	4045
6667	905	4042	4043
6668	4045	4043	4042
6669	39	1263	1265
6670	463	3133	1263
6671	465	1265	3133
6672	1263	3133	1265
6673	216	2251	2247
6674	1019	3135	2251
6675	463	2247	3135
6676	2251	3135	2247
6677	220	2270	2274
6678	465	3139	2270
6679	1019	2274	3139
6680	2270	3139	2274
6681	463	3135	3133
6682	1019	3139	3135
6683	465	3133	3139
6684	3135	3139	3133
6685	216	2250	2251
6686	1018	4154	2250
6687	1019	2251	4154
6688	2250	4154	2251
6689	24	1195	1194
6690	395	2955	1195
6691	394	1194	2955
6692	1195	2955	1194
6693	187	2075	2072
6694	971	2959	2075
6695	395	2072	2959
6696	2075	2959	2072
6697	186	2066	2069
6698	394	2957	2066
6699	971	2069	2957
6700	2066	2957	2069
6701	395	2959	2955
6702	971	2957	2959
6703	394	2955	2957
6704	2959	2957	2955
6705	77	1477	1481
6706	677	3757	1477
6707	681	1481	3757
6708	1477	3757	1481
6709	186	2071	2067
6710	973	3759	2071
6711	677	2067	3759

6712	2071	3759	2067
6713	286	2666	2668
6714	681	3768	2666
6715	973	2668	3768
6716	2666	3768	2668
6717	677	3759	3757
6718	973	3768	3759
6719	681	3757	3768
6720	3759	3768	3757
6721	187	2077	2075
6722	975	4110	2077
6723	971	2075	4110
6724	2077	4110	2075
6725	286	2668	2669
6726	973	4111	2668
6727	975	2669	4111
6728	2668	4111	2669
6729	186	2069	2071
6730	971	4109	2069
6731	973	2071	4109
6732	2069	4109	2071
6733	975	4111	4110
6734	973	4109	4111
6735	971	4110	4109
6736	4111	4109	4110
6737	76	1475	1471
6738	675	3739	1475
6739	671	1471	3739
6740	1475	3739	1471
6741	285	2662	2660
6742	919	3750	2662
6743	675	2660	3750
6744	2662	3750	2660
6745	168	1959	1963
6746	671	3741	1959
6747	919	1963	3741
6748	1959	3741	1963
6749	675	3750	3739
6750	919	3741	3750
6751	671	3739	3741
6752	3750	3741	3739
6753	20	1171	1170
6754	371	2883	1171
6755	370	1170	2883
6756	1171	2883	1170
6757	163	1931	1928
6758	899	2887	1931
6759	371	1928	2887
6760	1931	2887	1928
6761	162	1922	1925
6762	370	2885	1922
6763	899	1925	2885
6764	1922	2885	1925
6765	371	2887	2883
6766	899	2885	2887
6767	370	2883	2885
6768	2887	2885	2883
6769	77	1480	1476
6770	680	3753	1480

6771	676	1476	3753
6772	1480	3753	1476
6773	284	2657	2655
6774	903	3766	2657
6775	680	2655	3766
6776	2657	3766	2655
6777	163	1929	1933
6778	676	3755	1929
6779	903	1933	3755
6780	1929	3755	1933
6781	680	3766	3753
6782	903	3755	3766
6783	676	3753	3755
6784	3766	3755	3753
6785	76	1470	1474
6786	670	3735	1470
6787	674	1474	3735
6788	1470	3735	1474
6789	162	1927	1923
6790	901	3737	1927
6791	670	1923	3737
6792	1927	3737	1923
6793	284	2654	2656
6794	674	3748	2654
6795	901	2656	3748
6796	2654	3748	2656
6797	670	3737	3735
6798	901	3748	3737
6799	674	3735	3748
6800	3737	3748	3735
6801	163	1933	1931
6802	903	4038	1933
6803	899	1931	4038
6804	1933	4038	1931
6805	284	2656	2657
6806	901	4039	2656
6807	903	2657	4039
6808	2656	4039	2657
6809	162	1925	1927
6810	899	4037	1925
6811	901	1927	4037
6812	1925	4037	1927
6813	903	4039	4038
6814	901	4037	4039
6815	899	4038	4037
6816	4039	4037	4038
6817	76	1474	1475
6818	674	3747	1474
6819	675	1475	3747
6820	1474	3747	1475
6821	284	2658	2654
6822	1084	3749	2658
6823	674	2654	3749
6824	2658	3749	2654
6825	285	2660	2664
6826	675	3751	2660
6827	1084	2664	3751
6828	2660	3751	2664
6829	674	3749	3747

6830	1084	3751	3749
6831	675	3747	3751
6832	3749	3751	3747
6833	77	1481	1480
6834	681	3765	1481
6835	680	1480	3765
6836	1481	3765	1480
6837	286	2670	2666
6838	1085	3769	2670
6839	681	2666	3769
6840	2670	3769	2666
6841	284	2655	2659
6842	680	3767	2655
6843	1085	2659	3767
6844	2655	3767	2659
6845	681	3769	3765
6846	1085	3767	3769
6847	680	3765	3767
6848	3769	3767	3765
6849	285	2664	2665
6850	1084	4221	2664
6851	1086	2665	4221
6852	2664	4221	2665
6853	284	2659	2658
6854	1085	4220	2659
6855	1084	2658	4220
6856	2659	4220	2658
6857	286	2671	2670
6858	1086	4222	2671
6859	1085	2670	4222
6860	2671	4222	2670
6861	1084	4220	4221
6862	1085	4222	4220
6863	1086	4221	4222
6864	4220	4222	4221
6865	1091	1092	4226
6866	1091	4226	1
6867	291	2692	4683
6868	291	4683	1091
6869	2692	4329	1092
6870	2692	292	4329
6871	1092	4683	2692
6872	1092	1091	4683
6873	11	1128	4686
6874	11	4686	1126
6875	1128	4336	2771
6876	1128	328	4336
6877	1126	2771	4335
6878	1126	4335	326
6879	2771	4686	1128
6880	2771	1126	4686
6881	2776	2771	4336
6882	2776	4336	328
6883	787	5008	2776
6884	787	2772	5008
6885	2772	4335	2771
6886	2772	326	4335
6887	2771	2776	5008
6888	2771	5008	2772

6889	28	4691	1215
6890	28	1213	4691
6891	1213	4348	3007
6892	1213	413	4348
6893	1215	3007	4349
6894	1215	4349	415
6895	3007	1215	4691
6896	3007	4691	1213
6897	3009	3007	4348
6898	3009	4348	413
6899	998	3013	5014
6900	998	5014	3009
6901	3013	4349	3007
6902	3013	415	4349
6903	3007	5014	3013
6904	3007	3009	5014
6905	1381	4241	1383
6906	1381	61	4241
6907	3468	1383	4456
6908	3468	4456	583
6909	581	4736	3468
6910	581	1381	4736
6911	1383	3468	4736
6912	1383	4736	1381
6913	2307	4292	4895
6914	4292	2309	4895
6915	4292	226	2309
6916	3475	4897	4605
6917	4605	4897	931
6918	4897	2309	931
6919	583	4457	4459
6920	4457	3475	4459
6921	4457	2307	3475
6922	2309	4897	4895
6923	4895	4897	2307
6924	4897	3475	2307
6925	3468	4459	3475
6926	3468	583	4459
6927	3470	3475	4605
6928	3470	4605	931
6929	581	5070	3470
6930	581	3468	5070
6931	3475	3470	5070
6932	3475	5070	3468
6933	1383	4241	4738
6934	4241	61	4738
6935	4738	61	1382
6936	3472	4739	4455
6937	4455	4739	1382
6938	4455	1382	582
6939	583	4456	4458
6940	4456	1383	4458
6941	4458	1383	3472
6942	1382	4739	4738
6943	4738	4739	3472
6944	4738	3472	1383
6945	2307	4896	4292
6946	4292	4896	2311
6947	4292	2311	226

6948	583	4460	4457
6949	4457	4460	3476
6950	4457	3476	2307
6951	3476	4647	4898
6952	4647	1023	4898
6953	4898	1023	2311
6954	2311	4896	4898
6955	4896	2307	4898
6956	4898	2307	3476
6957	3472	4455	5073
6958	4455	3474	5073
6959	4455	582	3474
6960	3476	5074	4647
6961	4647	5074	1023
6962	5074	3474	1023
6963	583	4458	4460
6964	4458	3476	4460
6965	4458	3472	3476
6966	3474	5074	5073
6967	5073	5074	3472
6968	5074	3476	3472
6969	1393	4742	4243
6970	4243	4742	1395
6971	4243	1395	63
6972	593	4468	4467
6973	4467	4468	3504
6974	4467	3504	1393
6975	3504	4470	4744
6976	4470	595	4744
6977	4744	595	1395
6978	1395	4742	4744
6979	4742	1393	4744
6980	4744	1393	3504
6981	3506	3504	4468
6982	3506	4468	593
6983	933	3511	5076
6984	933	5076	3506
6985	3511	4470	3504
6986	3511	595	4470
6987	3504	5076	3511
6988	3504	3506	5076
6989	1393	4243	4743
6990	4243	1397	4743
6991	4243	63	1397
6992	3505	4745	4471
6993	4471	4745	597
6994	4745	1397	597
6995	593	4467	4469
6996	4467	3505	4469
6997	4467	1393	3505
6998	1397	4745	4743
6999	4743	4745	1393
7000	4745	3505	1393
7001	3516	3505	4471
7002	3516	4471	597
7003	934	5077	3516
7004	934	3507	5077
7005	3507	4469	3505
7006	3507	593	4469

7007	3505	3516	5077
7008	3505	5077	3507
7009	1385	4242	4735
7010	4242	1381	4735
7011	4242	61	1381
7012	3469	4737	4454
7013	4454	4737	581
7014	4737	1381	581
7015	585	4462	4464
7016	4462	3469	4464
7017	4462	1385	3469
7018	1381	4737	4735
7019	4735	4737	1385
7020	4737	3469	1385
7021	2576	4988	4325
7022	4325	4988	271
7023	4988	2578	271
7024	585	4466	4463
7025	4463	4466	2576
7026	4466	3480	2576
7027	3480	4606	4989
7028	4606	2578	4989
7029	4606	932	2578
7030	2578	4988	4989
7031	4988	3480	4989
7032	4988	2576	3480
7033	3469	4454	5071
7034	4454	3471	5071
7035	4454	581	3471
7036	3480	5072	4606
7037	4606	5072	932
7038	5072	3471	932
7039	585	4464	4466
7040	4464	3480	4466
7041	4464	3469	3480
7042	3471	5072	5071
7043	5071	5072	3469
7044	5072	3480	3469
7045	1385	4740	4242
7046	4242	4740	1384
7047	4242	1384	61
7048	585	4465	4462
7049	4462	4465	3477
7050	4462	3477	1385
7051	3477	4461	4741
7052	4461	584	4741
7053	4741	584	1384
7054	1384	4740	4741
7055	4740	1385	4741
7056	4741	1385	3477
7057	2580	2576	4325
7058	2580	4325	271
7059	1070	3481	4990
7060	1070	4990	2580
7061	3481	4463	2576
7062	3481	585	4463
7063	2576	4990	3481
7064	2576	2580	4990
7065	3481	3477	4465

7066	3481	4465	585
7067	1070	5075	3481
7068	1070	3479	5075
7069	3479	4461	3477
7070	3479	584	4461
7071	3477	3481	5075
7072	3477	5075	3479
7073	1230	4696	4231
7074	4231	4696	1228
7075	4231	1228	32
7076	430	4366	4364
7077	4364	4366	3041
7078	4364	3041	1230
7079	3041	4360	4697
7080	4360	428	4697
7081	4697	428	1228
7082	1228	4696	4697
7083	4696	1230	4697
7084	4697	1230	3041
7085	2171	2169	4280
7086	2171	4280	203
7087	717	3046	4858
7088	717	4858	2171
7089	3046	4365	2169
7090	3046	430	4365
7091	2169	4858	3046
7092	2169	2171	4858
7093	88	4779	1535
7094	88	1533	4779
7095	1533	4361	3042
7096	1533	428	4361
7097	1535	3042	4546
7098	1535	4546	717
7099	3042	1535	4779
7100	3042	4779	1533
7101	3041	4366	5021
7102	4366	430	5021
7103	5021	430	3046
7104	3042	5022	4546
7105	4546	5022	3046
7106	4546	3046	717
7107	428	4360	4361
7108	4360	3041	4361
7109	4361	3041	3042
7110	3046	5022	5021
7111	5021	5022	3042
7112	5021	3042	3041
7113	1531	4776	4255
7114	4255	4776	87
7115	4776	1529	87
7116	716	4545	4543
7117	4543	4545	1531
7118	4545	3033	1531
7119	3033	4356	4777
7120	4356	1529	4777
7121	4356	424	1529
7122	1529	4776	4777
7123	4776	3033	4777
7124	4776	1531	3033

7125	203	4279	4281
7126	4279	2170	4281
7127	4279	2168	2170
7128	2168	4357	4856
7129	4357	3037	4856
7130	4357	426	3037
7131	2170	4857	4544
7132	4544	4857	716
7133	4857	3037	716
7134	3037	4857	4856
7135	4856	4857	2168
7136	4857	2170	2168
7137	3032	4356	3033
7138	3032	424	4356
7139	3037	3033	4545
7140	3037	4545	716
7141	426	5018	3037
7142	426	3032	5018
7143	3033	3037	5018
7144	3033	5018	3032
7145	88	1535	4780
7146	88	4780	1534
7147	1535	4547	3852
7148	1535	717	4547
7149	1534	3852	4542
7150	1534	4542	715
7151	3852	4780	1535
7152	3852	1534	4780
7153	2171	4281	2170
7154	2171	203	4281
7155	3853	2170	4544
7156	3853	4544	716
7157	717	4859	3853
7158	717	2171	4859
7159	2170	3853	4859
7160	2170	4859	2171
7161	1530	1531	4255
7162	1530	4255	87
7163	715	3851	4778
7164	715	4778	1530
7165	3851	4543	1531
7166	3851	716	4543
7167	1531	4778	3851
7168	1531	1530	4778
7169	3853	3852	4547
7170	3853	4547	717
7171	716	5116	3853
7172	716	3851	5116
7173	3851	4542	3852
7174	3851	715	4542
7175	3852	3853	5116
7176	3852	5116	3851
7177	1231	4230	1229
7178	1231	32	4230
7179	3043	1229	4362
7180	3043	4362	429
7181	431	4698	3043
7182	431	1231	4698
7183	1229	3043	4698

7184	1229	4698	1231
7185	1805	1803	4258
7186	1805	4258	140
7187	842	3044	4790
7188	842	4790	1805
7189	3044	4363	1803
7190	3044	429	4363
7191	1803	4790	3044
7192	1803	1805	4790
7193	205	4864	2182
7194	205	2180	4864
7195	2180	4367	3048
7196	2180	431	4367
7197	2182	3048	4566
7198	2182	4566	842
7199	3048	2182	4864
7200	3048	4864	2180
7201	429	3044	5023
7202	429	5023	3043
7203	3044	4566	3048
7204	3044	842	4566
7205	3043	3048	4367
7206	3043	4367	431
7207	3048	5023	3044
7208	3048	3043	5023
7209	204	4863	2177
7210	204	2175	4863
7211	2175	4484	3581
7212	2175	618	4484
7213	2177	3581	4565
7214	2177	4565	837
7215	3581	2177	4863
7216	3581	4863	2175
7217	616	3574	5085
7218	616	5085	3572
7219	3574	4565	3581
7220	3574	837	4565
7221	3572	3581	4484
7222	3572	4484	618
7223	3581	5085	3574
7224	3581	3572	5085
7225	1231	1230	4231
7226	1231	4231	32
7227	431	3045	4699
7228	431	4699	1231
7229	3045	4364	1230
7230	3045	430	4364
7231	1230	4699	3045
7232	1230	1231	4699
7233	205	2184	4865
7234	205	4865	2180
7235	2184	4633	3049
7236	2184	1004	4633
7237	2180	3049	4368
7238	2180	4368	431
7239	3049	4865	2184
7240	3049	2180	4865
7241	2173	4280	2169
7242	2173	203	4280

7243	3047	2169	4365
7244	3047	4365	430
7245	1004	4861	3047
7246	1004	2173	4861
7247	2169	3047	4861
7248	2169	4861	2173
7249	3045	4368	3049
7250	3045	431	4368
7251	3047	3049	4633
7252	3047	4633	1004
7253	430	5024	3047
7254	430	3045	5024
7255	3049	3047	5024
7256	3049	5024	3045
7257	2168	4279	4855
7258	4279	2172	4855
7259	4279	203	2172
7260	3038	4860	4631
7261	4631	4860	1003
7262	4860	2172	1003
7263	426	4357	4358
7264	4357	3038	4358
7265	4357	2168	3038
7266	2172	4860	4855
7267	4855	4860	2168
7268	4860	3038	2168
7269	204	4862	2178
7270	204	2174	4862
7271	2174	4359	3040
7272	2174	427	4359
7273	2178	3040	4632
7274	2178	4632	1003
7275	3040	2178	4862
7276	3040	4862	2174
7277	3038	5019	4358
7278	4358	5019	426
7279	5019	3036	426
7280	1003	4632	4631
7281	4631	4632	3038
7282	4632	3040	3038
7283	3040	4359	5020
7284	4359	3036	5020
7285	4359	427	3036
7286	3036	5019	5020
7287	5019	3040	5020
7288	5019	3038	3040
7289	1187	1185	4228
7290	1187	4228	23
7291	387	2928	4688
7292	387	4688	1187
7293	2928	4344	1185
7294	2928	385	4344
7295	1185	4688	2928
7296	1185	1187	4688
7297	2014	4271	2012
7298	2014	177	4271
7299	2930	2012	4345
7300	2930	4345	385
7301	944	4832	2930

7302	944	2014	4832
7303	2012	2930	4832
7304	2012	4832	2014
7305	1429	4246	4753
7306	4246	69	4753
7307	4753	69	1431
7308	3612	4754	4493
7309	4493	4754	1431
7310	4493	1431	631
7311	629	4488	4490
7312	4488	1429	4490
7313	4490	1429	3612
7314	1431	4754	4753
7315	4753	4754	3612
7316	4753	3612	1429
7317	2025	4834	4272
7318	4272	4834	2028
7319	4272	2028	179
7320	629	4491	4489
7321	4489	4491	3614
7322	4489	3614	2025
7323	3614	4611	4836
7324	4611	951	4836
7325	4836	951	2028
7326	2028	4834	4836
7327	4834	2025	4836
7328	4836	2025	3614
7329	3612	4493	5087
7330	4493	3619	5087
7331	4493	631	3619
7332	3614	5089	4611
7333	4611	5089	951
7334	5089	3619	951
7335	629	4490	4491
7336	4490	3614	4491
7337	4490	3612	3614
7338	3619	5089	5087
7339	5087	5089	3612
7340	5089	3614	3612
7341	262	4978	2524
7342	262	2522	4978
7343	2522	4421	3388
7344	2522	554	4421
7345	2524	3388	4610
7346	2524	4610	946
7347	3388	2524	4978
7348	3388	4978	2522
7349	551	3380	5050
7350	551	5050	3378
7351	3380	4610	3388
7352	3380	946	4610
7353	3378	3388	4421
7354	3378	4421	554
7355	3388	5050	3380
7356	3388	3378	5050
7357	1343	4235	4712
7358	4235	1345	4712
7359	4235	54	1345
7360	3358	4713	4410

7361	4410	4713	545
7362	4713	1345	545
7363	543	4406	4408
7364	4406	3358	4408
7365	4406	1343	3358
7366	1345	4713	4712
7367	4712	4713	1343
7368	4713	3358	1343
7369	1807	4791	4259
7370	4259	4791	141
7371	4791	1809	141
7372	543	4409	4407
7373	4407	4409	1807
7374	4409	3359	1807
7375	3359	4567	4792
7376	4567	1809	4792
7377	4567	844	1809
7378	1809	4791	4792
7379	4791	3359	4792
7380	4791	1807	3359
7381	3358	4410	5045
7382	4410	545	5045
7383	5045	545	3363
7384	3359	5046	4567
7385	4567	5046	3363
7386	4567	3363	844
7387	543	4408	4409
7388	4408	3358	4409
7389	4409	3358	3359
7390	3363	5046	5045
7391	5045	5046	3359
7392	5045	3359	3358
7393	56	1354	4716
7394	56	4716	1352
7395	1354	4420	3382
7396	1354	554	4420
7397	1352	3382	4413
7398	1352	4413	552
7399	3382	4716	1354
7400	3382	1352	4716
7401	3389	3382	4420
7402	3389	4420	554
7403	1058	5051	3389
7404	1058	3384	5051
7405	3384	4413	3382
7406	3384	552	4413
7407	3382	3389	5051
7408	3382	5051	3384
7409	68	1425	4752
7410	68	4752	1424
7411	1425	4486	3598
7412	1425	625	4486
7413	1424	3598	4485
7414	1424	4485	624
7415	3598	4752	1425
7416	3598	1424	4752
7417	3602	3598	4486
7418	3602	4486	625
7419	1026	5086	3602

7420	1026	3600	5086
7421	3600	4485	3598
7422	3600	624	4485
7423	3598	3602	5086
7424	3598	5086	3600
7425	43	4703	1284
7426	43	1282	4703
7427	1282	4378	3184
7428	1282	482	4378
7429	1284	3184	4379
7430	1284	4379	484
7431	3184	1284	4703
7432	3184	4703	1282
7433	2289	4291	2293
7434	2289	223	4291
7435	3186	2293	4649
7436	3186	4649	1025
7437	482	4892	3186
7438	482	2289	4892
7439	2293	3186	4892
7440	2293	4892	2289
7441	2318	2322	4294
7442	2318	4294	228
7443	484	3191	4901
7444	484	4901	2318
7445	3191	4650	2322
7446	3191	1025	4650
7447	2322	4901	3191
7448	2322	2318	4901
7449	3186	3184	4378
7450	3186	4378	482
7451	1025	3191	5031
7452	1025	5031	3186
7453	3191	4379	3184
7454	3191	484	4379
7455	3184	5031	3191
7456	3184	3186	5031
7457	224	2299	4894
7458	224	4894	2298
7459	2299	4651	4161
7460	2299	1026	4651
7461	2298	4161	4648
7462	2298	4648	1024
7463	4161	4894	2299
7464	4161	2298	4894
7465	2323	4294	2322
7466	2323	228	4294
7467	4162	2322	4650
7468	4162	4650	1025
7469	1026	4902	4162
7470	1026	2323	4902
7471	2322	4162	4902
7472	2322	4902	2323
7473	2292	2293	4291
7474	2292	4291	223
7475	1024	4160	4893
7476	1024	4893	2292
7477	4160	4649	2293
7478	4160	1025	4649

7479	2293	4893	4160
7480	2293	2292	4893
7481	4162	4161	4651
7482	4162	4651	1026
7483	1025	5128	4162
7484	1025	4160	5128
7485	4160	4648	4161
7486	4160	1024	4648
7487	4161	4162	5128
7488	4161	5128	4160
7489	2614	2612	4326
7490	2614	4326	277
7491	950	3606	4991
7492	950	4991	2614
7493	3606	4487	2612
7494	3606	627	4487
7495	2612	4991	3606
7496	2612	2614	4991
7497	1433	4246	1429
7498	1433	69	4246
7499	3613	1429	4488
7500	3613	4488	629
7501	633	4755	3613
7502	633	1433	4755
7503	1429	3613	4755
7504	1429	4755	1433
7505	2025	4272	4835
7506	4272	2029	4835
7507	4272	179	2029
7508	3615	4837	4612
7509	4612	4837	952
7510	4837	2029	952
7511	629	4489	4492
7512	4489	3615	4492
7513	4489	2025	3615
7514	2029	4837	4835
7515	4835	4837	2025
7516	4837	3615	2025
7517	3613	4492	3615
7518	3613	629	4492
7519	3624	3615	4612
7520	3624	4612	952
7521	633	5088	3624
7522	633	3613	5088
7523	3615	3624	5088
7524	3615	5088	3613
7525	2616	4326	2612
7526	2616	277	4326
7527	3607	2612	4487
7528	3607	4487	627
7529	1076	4992	3607
7530	1076	2616	4992
7531	2612	3607	4992
7532	2612	4992	2616
7533	1890	4800	4262
7534	4262	4800	1887
7535	4262	1887	156
7536	882	4579	4578
7537	4578	4579	3650

7538	4578	3650	1890
7539	3650	4505	4801
7540	4505	641	4801
7541	4801	641	1887
7542	1887	4800	4801
7543	4800	1890	4801
7544	4801	1890	3650
7545	3648	4505	3650
7546	3648	641	4505
7547	3655	3650	4579
7548	3655	4579	882
7549	643	5096	3655
7550	643	3648	5096
7551	3650	3655	5096
7552	3650	5096	3648
7553	1888	1890	4262
7554	1888	4262	156
7555	877	4014	4802
7556	877	4802	1888
7557	4014	4578	1890
7558	4014	882	4578
7559	1890	4802	4014
7560	1890	1888	4802
7561	2364	4904	4295
7562	4295	4904	2360
7563	4295	2360	235
7564	1038	4655	4653
7565	4653	4655	3225
7566	4653	3225	2364
7567	3225	4380	4905
7568	4380	495	4905
7569	4905	495	2360
7570	2360	4904	4905
7571	4904	2364	4905
7572	4905	2364	3225
7573	2376	4296	4907
7574	4296	2372	4907
7575	4296	237	2372
7576	3229	4908	4381
7577	4381	4908	497
7578	4908	2372	497
7579	1038	4654	4656
7580	4654	3229	4656
7581	4654	2376	3229
7582	2372	4908	4907
7583	4907	4908	2376
7584	4908	3229	2376
7585	3225	5032	4380
7586	4380	5032	495
7587	5032	3223	495
7588	1038	4656	4655
7589	4655	4656	3225
7590	4656	3229	3225
7591	3229	4381	5033
7592	4381	3223	5033
7593	4381	497	3223
7594	3223	5032	5033
7595	5032	3229	5033
7596	5032	3225	3229

7597	2376	4909	4296
7598	4296	4909	2377
7599	4296	2377	237
7600	1038	4657	4654
7601	4654	4657	4176
7602	4654	4176	2376
7603	4176	4658	4910
7604	4658	1041	4910
7605	4910	1041	2377
7606	2377	4909	4910
7607	4909	2376	4910
7608	4910	2376	4176
7609	2365	2364	4295
7610	2365	4295	235
7611	1039	4175	4906
7612	1039	4906	2365
7613	4175	4653	2364
7614	4175	1038	4653
7615	2364	4906	4175
7616	2364	2365	4906
7617	4177	4658	4176
7618	4177	1041	4658
7619	4175	4176	4657
7620	4175	4657	1038
7621	1039	4177	5129
7622	1039	5129	4175
7623	4176	5129	4177
7624	4176	4175	5129
7625	111	4785	1653
7626	111	1651	4785
7627	1651	4411	3366
7628	1651	546	4411
7629	1653	3366	4556
7630	1653	4556	772
7631	3366	1653	4785
7632	3366	4785	1651
7633	548	3370	5047
7634	548	5047	3365
7635	3370	4556	3366
7636	3370	772	4556
7637	3365	3366	4411
7638	3365	4411	546
7639	3366	5047	3370
7640	3366	3365	5047
7641	1358	4237	4719
7642	4237	1356	4719
7643	4237	57	1356
7644	3392	4721	4423
7645	4423	4721	556
7646	4721	1356	556
7647	558	4427	4428
7648	4427	3392	4428
7649	4427	1358	3392
7650	1356	4721	4719
7651	4719	4721	1358
7652	4721	3392	1358
7653	3394	3392	4423
7654	3394	4423	556
7655	776	3401	5054

7656	776	5054	3394
7657	3401	4428	3392
7658	3401	558	4428
7659	3392	5054	3401
7660	3392	3394	5054
7661	157	4265	4263
7662	4265	1894	4263
7663	4263	1894	1892
7664	1894	4570	4809
7665	4570	879	4809
7666	4809	879	2868
7667	1892	4805	4340
7668	4340	4805	2868
7669	4340	2868	365
7670	2868	4805	4809
7671	4809	4805	1892
7672	4809	1892	1894
7673	260	2513	4972
7674	260	4972	2511
7675	2513	4583	3725
7676	2513	884	4583
7677	2511	3725	4511
7678	2511	4511	666
7679	3725	4972	2513
7680	3725	2511	4972
7681	4264	4806	1896
7682	4506	4507	3718
7683	4582	884	4811
7684	4806	1893	4811
7685	3725	5098	4511
7686	4511	5098	666
7687	5098	3716	666
7688	884	4582	4583
7689	4583	4582	3725
7690	4582	3718	3725
7691	3718	4507	5097
7692	4507	3716	5097
7693	4507	664	3716
7694	3716	5098	5097
7695	5098	3718	5097
7696	5098	3725	3718
7697	1883	4261	1885
7698	1883	155	4261
7699	3368	1885	4573
7700	3368	4573	880
7701	547	4796	3368
7702	547	1883	4796
7703	1885	3368	4796
7704	1885	4796	1883
7705	1894	4265	4808
7706	4265	1896	4808
7707	4265	157	1896
7708	4017	4812	4584
7709	4584	4812	884
7710	4812	1896	884
7711	879	4570	4572
7712	4570	4017	4572
7713	4570	1894	4017
7714	1896	4812	4808

7715	4808	4812	1894
7716	4812	4017	1894
7717	1885	4261	4797
7718	4261	155	4797
7719	4797	155	1884
7720	4016	4798	4571
7721	4571	4798	1884
7722	4571	1884	879
7723	880	4573	4574
7724	4573	1885	4574
7725	4574	1885	4016
7726	1884	4798	4797
7727	4797	4798	4016
7728	4797	4016	1885
7729	4017	4584	5119
7730	4584	4018	5119
7731	4584	884	4018
7732	4016	5118	4574
7733	4574	5118	880
7734	5118	4018	880
7735	879	4572	4571
7736	4572	4016	4571
7737	4572	4017	4016
7738	4018	5118	5119
7739	5119	5118	4017
7740	5118	4016	4017
7741	1360	4722	4238
7742	4238	4722	1357
7743	4238	1357	57
7744	560	4433	4431
7745	4431	4433	3396
7746	4431	3396	1360
7747	3396	4425	4723
7748	4425	557	4723
7749	4723	557	1357
7750	1357	4722	4723
7751	4722	1360	4723
7752	4723	1360	3396
7753	2534	4323	4979
7754	4323	264	4979
7755	4979	264	2536
7756	3406	4982	4621
7757	4621	4982	2536
7758	4621	2536	982
7759	560	4432	4434
7760	4432	2534	4434
7761	4434	2534	3406
7762	2536	4982	4979
7763	4979	4982	3406
7764	4979	3406	2534
7765	560	4434	4433
7766	4434	3396	4433
7767	4434	3406	3396
7768	3406	4621	5058
7769	4621	3398	5058
7770	4621	982	3398
7771	3396	5056	4425
7772	4425	5056	557
7773	5056	3398	557

7774	3398	5056	5058
7775	5058	5056	3406
7776	5056	3396	3406
7777	1467	4248	1465
7778	1467	75	4248
7779	3720	1465	4509
7780	3720	4509	665
7781	667	4761	3720
7782	667	1467	4761
7783	1465	3720	4761
7784	1465	4761	1467
7785	2100	2097	4276
7786	2100	4276	191
7787	987	3722	4845
7788	987	4845	2100
7789	3722	4510	2097
7790	3722	665	4510
7791	2097	4845	3722
7792	2097	2100	4845
7793	264	4981	2537
7794	264	2535	4981
7795	2535	4512	3727
7796	2535	667	4512
7797	2537	3727	4623
7798	2537	4623	987
7799	3727	2537	4981
7800	3727	4981	2535
7801	665	3722	5101
7802	665	5101	3720
7803	3722	4623	3727
7804	3722	987	4623
7805	3720	3727	4512
7806	3720	4512	667
7807	3727	5101	3722
7808	3727	3720	5101
7809	57	4237	4238
7810	4237	1358	4238
7811	4238	1358	1360
7812	1358	4427	4724
7813	4427	558	4724
7814	4724	558	3400
7815	1360	4725	4431
7816	4431	4725	3400
7817	4431	3400	560
7818	3400	4725	4724
7819	4724	4725	1360
7820	4724	1360	1358
7821	2534	4980	4323
7822	4323	4980	264
7823	4980	2538	264
7824	560	4435	4432
7825	4432	4435	2534
7826	4435	3407	2534
7827	3407	4676	4983
7828	4676	2538	4983
7829	4676	1061	2538
7830	2538	4980	4983
7831	4980	3407	4983
7832	4980	2534	3407

7833	558	3402	5059
7834	558	5059	3400
7835	3402	4676	3407
7836	3402	1061	4676
7837	3400	3407	4435
7838	3400	4435	560
7839	3407	5059	3402
7840	3407	3400	5059
7841	1469	1465	4248
7842	1469	4248	75
7843	669	3721	4762
7844	669	4762	1469
7845	3721	4509	1465
7846	3721	665	4509
7847	1465	4762	3721
7848	1465	1469	4762
7849	283	2651	4998
7850	283	4998	2649
7851	2651	4624	3732
7852	2651	988	4624
7853	2649	3732	4514
7854	2649	4514	669
7855	3732	4998	2651
7856	3732	2649	4998
7857	2101	4276	2097
7858	2101	191	4276
7859	3723	2097	4510
7860	3723	4510	665
7861	988	4846	3723
7862	988	2101	4846
7863	2097	3723	4846
7864	2097	4846	2101
7865	3721	4514	3732
7866	3721	669	4514
7867	3723	3732	4624
7868	3723	4624	988
7869	665	5102	3723
7870	665	3721	5102
7871	3732	3723	5102
7872	3732	5102	3721
7873	156	1889	4799
7874	156	4799	1886
7875	1889	4575	2867
7876	1889	881	4575
7877	1886	2867	4339
7878	1886	4339	364
7879	2867	4799	1889
7880	2867	1886	4799
7881	1892	4804	4263
7882	4263	4804	1895
7883	4263	1895	157
7884	365	4341	4340
7885	4340	4341	2869
7886	4340	2869	1892
7887	2869	4576	4810
7888	4576	881	4810
7889	4810	881	1895
7890	1895	4804	4810
7891	4804	1892	4810

7892	4810	1892	2869
7893	2867	5010	4339
7894	4339	5010	364
7895	5010	2865	364
7896	881	4576	4575
7897	4575	4576	2867
7898	4576	2869	2867
7899	2869	4341	5011
7900	4341	2865	5011
7901	4341	365	2865
7902	2865	5010	5011
7903	5010	2869	5011
7904	5010	2867	2869
7905	4264	1897	4807
7906	4586	4813	885
7907	4506	3719	4508
7908	4807	4813	1893
7909	2639	4327	4994
7910	4327	281	4994
7911	4994	281	2637
7912	3730	4995	4513
7913	4513	4995	2637
7914	4513	2637	668
7915	885	4585	4587
7916	4585	2639	4587
7917	4587	2639	3730
7918	2637	4995	4994
7919	4994	4995	3730
7920	4994	3730	2639
7921	3719	5099	4508
7922	4508	5099	3717
7923	4508	3717	664
7924	885	4587	4586
7925	4586	4587	3730
7926	4586	3730	3719
7927	3730	4513	5100
7928	4513	668	5100
7929	5100	668	3717
7930	3717	5099	5100
7931	5099	3719	5100
7932	5100	3719	3730
7933	156	1891	4803
7934	156	4803	1889
7935	1891	4580	4019
7936	1891	883	4580
7937	1889	4019	4577
7938	1889	4577	881
7939	4019	4803	1891
7940	4019	1889	4803
7941	2639	4996	4327
7942	4327	4996	281
7943	4996	2638	281
7944	885	4588	4585
7945	4585	4588	2639
7946	4588	4021	2639
7947	4021	4581	4997
7948	4581	2638	4997
7949	4581	883	2638
7950	2638	4996	4997

7951	4996	4021	4997
7952	4996	2639	4021
7953	883	4581	4580
7954	4581	4019	4580
7955	4581	4021	4019
7956	4021	4588	5121
7957	4588	4020	5121
7958	4588	885	4020
7959	4019	5120	4577
7960	4577	5120	881
7961	5120	4020	881
7962	4020	5120	5121
7963	5121	5120	4021
7964	5120	4019	4021
7965	1488	1490	4253
7966	1488	4253	79
7967	688	3788	4772
7968	688	4772	1488
7969	3788	4535	1490
7970	3788	690	4535
7971	1490	4772	3788
7972	1490	1488	4772
7973	174	1998	4829
7974	174	4829	1995
7975	1998	4607	3790
7976	1998	936	4607
7977	1995	3790	4534
7978	1995	4534	688
7979	3790	4829	1998
7980	3790	1995	4829
7981	2315	4293	2313
7982	2315	227	4293
7983	3797	2313	4536
7984	3797	4536	690
7985	936	4899	3797
7986	936	2315	4899
7987	2313	3797	4899
7988	2313	4899	2315
7989	3788	4534	3790
7990	3788	688	4534
7991	3797	3790	4607
7992	3797	4607	936
7993	690	5112	3797
7994	690	3788	5112
7995	3790	3797	5112
7996	3790	5112	3788
7997	1491	4253	1490
7998	1491	79	4253
7999	3796	1490	4535
8000	3796	4535	690
8001	691	4773	3796
8002	691	1491	4773
8003	1490	3796	4773
8004	1490	4773	1491
8005	2317	2313	4293
8006	2317	4293	227
8007	1029	3798	4900
8008	1029	4900	2317
8009	3798	4536	2313

8010	3798	690	4536
8011	2313	4900	3798
8012	2313	2317	4900
8013	229	4903	2329
8014	229	2325	4903
8015	2325	4537	3800
8016	2325	691	4537
8017	2329	3800	4652
8018	2329	4652	1029
8019	3800	2329	4903
8020	3800	4903	2325
8021	690	3798	5113
8022	690	5113	3796
8023	3798	4652	3800
8024	3798	1029	4652
8025	3796	3800	4537
8026	3796	4537	691
8027	3800	5113	3798
8028	3800	3796	5113
8029	57	1359	4720
8030	57	4720	1356
8031	1359	4429	3393
8032	1359	559	4429
8033	1356	3393	4424
8034	1356	4424	556
8035	3393	4720	1359
8036	3393	1356	4720
8037	3404	3393	4429
8038	3404	4429	559
8039	777	5055	3404
8040	777	3395	5055
8041	3395	4424	3393
8042	3395	556	4424
8043	3393	3404	5055
8044	3393	5055	3395
8045	1353	4236	4714
8046	4236	1350	4714
8047	4236	56	1350
8048	3375	4715	4412
8049	4412	4715	550
8050	4715	1350	550
8051	553	4414	4416
8052	4414	3375	4416
8053	4414	1353	3375
8054	1350	4715	4714
8055	4714	4715	1353
8056	4715	3375	1353
8057	2516	4973	4322
8058	4322	4973	261
8059	4973	2518	261
8060	553	4418	4415
8061	4415	4418	2516
8062	4418	3386	2516
8063	3386	4557	4976
8064	4557	2518	4976
8065	4557	775	2518
8066	2518	4973	4976
8067	4973	3386	4976
8068	4973	2516	3386

8069	3375	4412	5048
8070	4412	550	5048
8071	5048	550	3377
8072	3386	5049	4557
8073	4557	5049	3377
8074	4557	3377	775
8075	553	4416	4418
8076	4416	3375	4418
8077	4418	3375	3386
8078	3377	5049	5048
8079	5048	5049	3386
8080	5048	3386	3375
8081	189	2089	4844
8082	189	4844	2085
8083	2089	4622	3399
8084	2089	983	4622
8085	2085	3399	4426
8086	2085	4426	557
8087	3399	4844	2089
8088	3399	2085	4844
8089	3397	4426	3399
8090	3397	557	4426
8091	3408	3399	4622
8092	3408	4622	983
8093	561	5057	3408
8094	561	3397	5057
8095	3399	3408	5057
8096	3399	5057	3397
8097	1189	4228	1185
8098	1189	23	4228
8099	2929	1185	4344
8100	2929	4344	385
8101	389	4690	2929
8102	389	1189	4690
8103	1185	2929	4690
8104	1185	4690	1189
8105	2015	2012	4271
8106	2015	4271	177
8107	945	2931	4833
8108	945	4833	2015
8109	2931	4345	2012
8110	2931	385	4345
8111	2012	4833	2931
8112	2012	2015	4833
8113	261	4975	2521
8114	261	2517	4975
8115	2517	4430	3405
8116	2517	559	4430
8117	2521	3405	4678
8118	2521	4678	1064
8119	3405	2521	4975
8120	3405	4975	2517
8121	561	3409	5060
8122	561	5060	3403
8123	3409	4678	3405
8124	3409	1064	4678
8125	3403	3405	4430
8126	3403	4430	559
8127	3405	5060	3409

8128	3405	3403	5060
8129	1353	4717	4236
8130	4236	4717	56
8131	4717	1355	56
8132	553	4417	4414
8133	4414	4417	1353
8134	4417	3385	1353
8135	3385	4422	4718
8136	4422	1355	4718
8137	4422	555	1355
8138	1355	4717	4718
8139	4717	3385	4718
8140	4717	1353	3385
8141	2516	4322	4974
8142	4322	261	4974
8143	4974	261	2520
8144	3387	4977	4677
8145	4677	4977	2520
8146	4677	2520	1063
8147	553	4415	4419
8148	4415	2516	4419
8149	4419	2516	3387
8150	2520	4977	4974
8151	4974	4977	3387
8152	4974	3387	2516
8153	553	4419	4417
8154	4419	3387	4417
8155	4417	3387	3385
8156	3387	4677	5053
8157	4677	1063	5053
8158	5053	1063	3391
8159	3385	5052	4422
8160	4422	5052	3391
8161	4422	3391	555
8162	3391	5052	5053
8163	5053	5052	3385
8164	5053	3385	3387
8165	1319	4233	4705
8166	4233	1316	4705
8167	4233	49	1316
8168	3285	4706	4387
8169	4387	4706	516
8170	4706	1316	516
8171	519	4389	4391
8172	4389	3285	4391
8173	4389	1319	3285
8174	1316	4706	4705
8175	4705	4706	1319
8176	4706	3285	1319
8177	2397	4914	4298
8178	4298	4914	2399
8179	4298	2399	241
8180	519	4393	4390
8181	4390	4393	3296
8182	4390	3296	2397
8183	3296	4551	4916
8184	4551	759	4916
8185	4916	759	2399
8186	2399	4914	4916

8187	4914	2397	4916
8188	4916	2397	3296
8189	3285	4387	5037
8190	4387	3287	5037
8191	4387	516	3287
8192	3296	5038	4551
8193	4551	5038	759
8194	5038	3287	759
8195	519	4391	4393
8196	4391	3296	4393
8197	4391	3285	3296
8198	3287	5038	5037
8199	5037	5038	3285
8200	5038	3296	3285
8201	1496	1494	4254
8202	1496	4254	80
8203	696	3806	4774
8204	696	4774	1496
8205	3806	4538	1494
8206	3806	694	4538
8207	1494	4774	3806
8208	1494	1496	4774
8209	243	2411	4920
8210	243	4920	2409
8211	2411	4608	3815
8212	2411	938	4608
8213	2409	3815	4540
8214	2409	4540	696
8215	3815	4920	2411
8216	3815	2409	4920
8217	2004	4270	2001
8218	2004	175	4270
8219	3808	2001	4539
8220	3808	4539	694
8221	938	4830	3808
8222	938	2004	4830
8223	2001	3808	4830
8224	2001	4830	2004
8225	3806	4540	3815
8226	3806	696	4540
8227	3808	3815	4608
8228	3808	4608	938
8229	694	5114	3808
8230	694	3806	5114
8231	3815	3808	5114
8232	3815	5114	3806
8233	47	4704	1309
8234	47	1305	4704
8235	1305	4382	3253
8236	1305	505	4382
8237	1309	3253	4384
8238	1309	4384	509
8239	3253	1309	4704
8240	3253	4704	1305
8241	2408	4918	4299
8242	4299	4918	2410
8243	4299	2410	243
8244	509	4385	4383
8245	4383	4385	3264

8246	4383	3264	2408
8247	3264	4604	4921
8248	4604	929	4921
8249	4921	929	2410
8250	2410	4918	4921
8251	4918	2408	4921
8252	4921	2408	3264
8253	3253	4382	5034
8254	4382	505	5034
8255	5034	505	3255
8256	3264	5035	4604
8257	4604	5035	3255
8258	4604	3255	929
8259	509	4384	4385
8260	4384	3253	4385
8261	4385	3253	3264
8262	3255	5035	5034
8263	5034	5035	3264
8264	5034	3264	3253
8265	49	1321	4707
8266	49	4707	1317
8267	1321	4396	3289
8268	1321	521	4396
8269	1317	3289	4388
8270	1317	4388	517
8271	3289	4707	1321
8272	3289	1317	4707
8273	2434	2432	4301
8274	2434	4301	247
8275	979	3300	4925
8276	979	4925	2434
8277	3300	4395	2432
8278	3300	521	4395
8279	2432	4925	3300
8280	2432	2434	4925
8281	3300	3289	4396
8282	3300	4396	521
8283	979	5039	3300
8284	979	3291	5039
8285	3291	4388	3289
8286	3291	517	4388
8287	3289	3300	5039
8288	3289	5039	3291
8289	2408	4299	4919
8290	4299	2412	4919
8291	4299	243	2412
8292	3265	4922	4663
8293	4663	4922	1045
8294	4922	2412	1045
8295	509	4383	4386
8296	4383	3265	4386
8297	4383	2408	3265
8298	2412	4922	4919
8299	4919	4922	2408
8300	4922	3265	2408
8301	3259	4386	3265
8302	3259	509	4386
8303	3261	3265	4663
8304	3261	4663	1045

8305	507	5036	3261
8306	507	3259	5036
8307	3265	3261	5036
8308	3265	5036	3259
8309	1319	4708	4233
8310	4233	4708	1321
8311	4233	1321	49
8312	519	4392	4389
8313	4389	4392	3295
8314	4389	3295	1319
8315	3295	4397	4709
8316	4397	521	4709
8317	4709	521	1321
8318	1321	4708	4709
8319	4708	1319	4709
8320	4709	1319	3295
8321	2397	4298	4915
8322	4298	241	4915
8323	4915	241	2401
8324	3297	4917	4664
8325	4664	4917	2401
8326	4664	2401	1046
8327	519	4390	4394
8328	4390	2397	4394
8329	4394	2397	3297
8330	2401	4917	4915
8331	4915	4917	3297
8332	4915	3297	2397
8333	2436	4301	2432
8334	2436	247	4301
8335	3301	2432	4395
8336	3301	4395	521
8337	1046	4926	3301
8338	1046	2436	4926
8339	2432	3301	4926
8340	2432	4926	2436
8341	519	4394	4392
8342	4394	3295	4392
8343	4394	3297	3295
8344	3297	4664	5041
8345	4664	3301	5041
8346	4664	1046	3301
8347	3295	5040	4397
8348	4397	5040	521
8349	5040	3301	521
8350	3301	5040	5041
8351	5041	5040	3297
8352	5040	3295	3297
8353	23	1189	4689
8354	23	4689	1188
8355	1189	4347	2937
8356	1189	389	4347
8357	1188	2937	4346
8358	1188	4346	388
8359	2937	4689	1189
8360	2937	1188	4689
8361	2036	4274	2039
8362	2036	181	4274
8363	2941	2039	4614

8364	2941	4614	953
8365	389	4840	2941
8366	389	2036	4840
8367	2039	2941	4840
8368	2039	4840	2036
8369	2030	2033	4273
8370	2030	4273	180
8371	388	2939	4838
8372	388	4838	2030
8373	2939	4613	2033
8374	2939	953	4613
8375	2033	4838	2939
8376	2033	2030	4838
8377	2941	2937	4347
8378	2941	4347	389
8379	953	5013	2941
8380	953	2939	5013
8381	2939	4346	2937
8382	2939	388	4346
8383	2937	2941	5013
8384	2937	5013	2939
8385	2041	2039	4274
8386	2041	4274	181
8387	957	4092	4841
8388	957	4841	2041
8389	4092	4614	2039
8390	4092	953	4614
8391	2039	4841	4092
8392	2039	2041	4841
8393	288	5005	2681
8394	288	2680	5005
8395	2680	4615	4093
8396	2680	955	4615
8397	2681	4093	4616
8398	2681	4616	957
8399	4093	2681	5005
8400	4093	5005	2680
8401	2035	4273	2033
8402	2035	180	4273
8403	4091	2033	4613
8404	4091	4613	953
8405	955	4839	4091
8406	955	2035	4839
8407	2033	4091	4839
8408	2033	4839	2035
8409	4092	4616	4093
8410	4092	957	4616
8411	4091	4093	4615
8412	4091	4615	955
8413	953	4092	5124
8414	953	5124	4091
8415	4093	5124	4092
8416	4093	4091	5124
8417	1498	4254	1494
8418	1498	80	4254
8419	3807	1494	4538
8420	3807	4538	694
8421	698	4775	3807
8422	698	1498	4775

8423	1494	3807	4775
8424	1494	4775	1498
8425	2005	2001	4270
8426	2005	4270	175
8427	939	3809	4831
8428	939	4831	2005
8429	3809	4539	2001
8430	3809	694	4539
8431	2001	4831	3809
8432	2001	2005	4831
8433	287	5004	2675
8434	287	2673	5004
8435	2673	4541	3820
8436	2673	698	4541
8437	2675	3820	4609
8438	2675	4609	939
8439	3820	2675	5004
8440	3820	5004	2673
8441	694	3809	5115
8442	694	5115	3807
8443	3809	4609	3820
8444	3809	939	4609
8445	3807	3820	4541
8446	3807	4541	698
8447	3820	5115	3809
8448	3820	3807	5115
8449	1090	4226	1092
8450	1090	1	4226
8451	2690	1092	4329
8452	2690	4329	292
8453	290	4682	2690
8454	290	1090	4682
8455	1092	2690	4682
8456	1092	4682	1090
8457	58	1365	4728
8458	58	4728	1363
8459	1365	4438	3414
8460	1365	565	4438
8461	1363	3414	4437
8462	1363	4437	563
8463	3414	4728	1365
8464	3414	1363	4728
8465	3421	3414	4438
8466	3421	4438	565
8467	805	5063	3421
8468	805	3416	5063
8469	3416	4437	3414
8470	3416	563	4437
8471	3414	3421	5063
8472	3414	5063	3416
8473	1100	4684	4227
8474	4227	4684	5
8475	4684	1102	5
8476	300	4332	4330
8477	4330	4332	1100
8478	4332	2708	1100
8479	2708	4334	4685
8480	4334	1102	4685
8481	4334	302	1102

8482	1102	4684	4685
8483	4684	2708	4685
8484	4684	1100	2708
8485	1550	4256	4782
8486	4256	1552	4782
8487	4256	92	1552
8488	2709	4784	4549
8489	4549	4784	724
8490	4784	1552	724
8491	300	4331	4333
8492	4331	2709	4333
8493	4331	1550	2709
8494	1552	4784	4782
8495	4782	4784	1550
8496	4784	2709	1550
8497	300	4333	4332
8498	4333	2709	4332
8499	4332	2709	2708
8500	2709	4549	5007
8501	4549	724	5007
8502	5007	724	2713
8503	2708	5006	4334
8504	4334	5006	2713
8505	4334	2713	302
8506	2713	5006	5007
8507	5007	5006	2708
8508	5007	2708	2709
8509	92	4783	1553
8510	92	1551	4783
8511	1551	4371	3087
8512	1551	446	4371
8513	1553	3087	4550
8514	1553	4550	725
8515	3087	1553	4783
8516	3087	4783	1551
8517	448	3091	5027
8518	448	5027	3086
8519	3091	4550	3087
8520	3091	725	4550
8521	3086	3087	4371
8522	3086	4371	446
8523	3087	5027	3091
8524	3087	3086	5027
8525	38	1258	4700
8526	38	4700	1255
8527	1258	4375	3108
8528	1258	458	4375
8529	1255	3108	4372
8530	1255	4372	455
8531	3108	4700	1258
8532	3108	1255	4700
8533	2252	4286	2254
8534	2252	217	4286
8535	3118	2254	4591
8536	3118	4591	888
8537	458	4878	3118
8538	458	2252	4878
8539	2254	3118	4878
8540	2254	4878	2252

8541	1899	1902	4266
8542	1899	4266	158
8543	455	3110	4815
8544	455	4815	1899
8545	3110	4590	1902
8546	3110	888	4590
8547	1902	4815	3110
8548	1902	1899	4815
8549	3118	3108	4375
8550	3118	4375	458
8551	888	5029	3118
8552	888	3110	5029
8553	3110	4372	3108
8554	3110	455	4372
8555	3108	3118	5029
8556	3108	5029	3110
8557	1369	4731	4240
8558	4240	4731	1371
8559	4240	1371	59
8560	569	4448	4446
8561	4446	4448	3432
8562	4446	3432	1369
8563	3432	4452	4733
8564	4452	571	4733
8565	4733	571	1371
8566	1371	4731	4733
8567	4731	1369	4733
8568	4733	1369	3432
8569	1911	4267	4817
8570	4267	1914	4817
8571	4267	160	1914
8572	3434	4820	4593
8573	4593	4820	895
8574	4820	1914	895
8575	569	4447	4450
8576	4447	3434	4450
8577	4447	1911	3434
8578	1914	4820	4817
8579	4817	4820	1911
8580	4820	3434	1911
8581	569	4450	4448
8582	4450	3434	4448
8583	4448	3434	3432
8584	3434	4593	5068
8585	4593	895	5068
8586	5068	895	3439
8587	3432	5066	4452
8588	4452	5066	3439
8589	4452	3439	571
8590	3439	5066	5068
8591	5068	5066	3432
8592	5068	3432	3434
8593	1900	4266	1902
8594	1900	158	4266
8595	4022	1902	4590
8596	4022	4590	888
8597	886	4816	4022
8598	886	1900	4816
8599	1902	4022	4816

8600	1902	4816	1900
8601	2255	2254	4286
8602	2255	4286	217
8603	895	4026	4879
8604	895	4879	2255
8605	4026	4591	2254
8606	4026	888	4591
8607	2254	4879	4026
8608	2254	2255	4879
8609	160	4819	1914
8610	160	1912	4819
8611	1912	4589	4023
8612	1912	886	4589
8613	1914	4023	4594
8614	1914	4594	895
8615	4023	1914	4819
8616	4023	4819	1912
8617	888	4026	5122
8618	888	5122	4022
8619	4026	4594	4023
8620	4026	895	4594
8621	4022	4023	4589
8622	4022	4589	886
8623	4023	5122	4026
8624	4023	4022	5122
8625	1369	4240	4732
8626	4240	1373	4732
8627	4240	59	1373
8628	3433	4734	4453
8629	4453	4734	573
8630	4734	1373	573
8631	569	4446	4449
8632	4446	3433	4449
8633	4446	1369	3433
8634	1373	4734	4732
8635	4732	4734	1369
8636	4734	3433	1369
8637	1911	4818	4267
8638	4267	4818	1915
8639	4267	1915	160
8640	569	4451	4447
8641	4447	4451	3435
8642	4447	3435	1911
8643	3435	4595	4821
8644	4595	896	4821
8645	4821	896	1915
8646	1915	4818	4821
8647	4818	1911	4821
8648	4821	1911	3435
8649	3433	4453	5067
8650	4453	3444	5067
8651	4453	573	3444
8652	3435	5069	4595
8653	4595	5069	3444
8654	4595	3444	896
8655	569	4449	4451
8656	4449	3435	4451
8657	4449	3433	3435
8658	3444	5069	5067

8659	5067	5069	3435
8660	5067	3435	3433
8661	1366	4726	4239
8662	4239	4726	58
8663	4726	1362	58
8664	566	4441	4439
8665	4439	4441	1366
8666	4441	3411	1366
8667	3411	4436	4727
8668	4436	1362	4727
8669	4436	562	1362
8670	1362	4726	4727
8671	4726	3411	4727
8672	4726	1366	3411
8673	2546	4324	4984
8674	4324	2548	4984
8675	4324	266	2548
8676	3424	4986	4558
8677	4558	4986	2548
8678	4558	2548	784
8679	566	4440	4443
8680	4440	3424	4443
8681	4440	2546	3424
8682	2548	4986	4984
8683	4984	4986	3424
8684	4984	3424	2546
8685	566	4443	4441
8686	4443	3424	4441
8687	4441	3424	3411
8688	3424	4558	5062
8689	4558	784	5062
8690	5062	784	3413
8691	3411	5061	4436
8692	4436	5061	3413
8693	4436	3413	562
8694	3413	5061	5062
8695	5062	5061	3411
8696	5062	3411	3424
8697	1366	4239	4729
8698	4239	1367	4729
8699	4239	58	1367
8700	3423	4730	4445
8701	4445	4730	567
8702	4730	1367	567
8703	566	4439	4442
8704	4439	3423	4442
8705	4439	1366	3423
8706	1367	4730	4729
8707	4729	4730	1366
8708	4730	3423	1366
8709	2546	4985	4324
8710	4324	4985	266
8711	4985	2550	266
8712	566	4444	4440
8713	4440	4444	2546
8714	4444	3425	2546
8715	3425	4679	4987
8716	4679	2550	4987
8717	4679	1066	2550

8718	2550	4985	4987
8719	4985	3425	4987
8720	4985	2546	3425
8721	3423	4445	5064
8722	4445	567	5064
8723	5064	567	3427
8724	3425	5065	4679
8725	4679	5065	3427
8726	4679	3427	1066
8727	566	4442	4444
8728	4442	3423	4444
8729	4444	3423	3425
8730	3427	5065	5064
8731	5064	5065	3425
8732	5064	3425	3423
8733	90	4781	1545
8734	90	1543	4781
8735	1543	4370	3069
8736	1543	438	4370
8737	1545	3069	4548
8738	1545	4548	722
8739	3069	1545	4781
8740	3069	4781	1543
8741	440	3073	5026
8742	440	5026	3068
8743	3073	4548	3069
8744	3073	722	4548
8745	3068	3069	4370
8746	3068	4370	438
8747	3069	5026	3073
8748	3069	3068	5026
8749	1437	4756	4247
8750	4247	4756	1435
8751	4247	1435	70
8752	637	4500	4498
8753	4498	4500	3630
8754	4498	3630	1437
8755	3630	4494	4758
8756	4494	635	4758
8757	4758	635	1435
8758	1435	4756	4758
8759	4756	1437	4758
8760	4758	1437	3630
8761	2213	2211	4283
8762	2213	4283	210
8763	873	3637	4870
8764	873	4870	2213
8765	3637	4499	2211
8766	3637	637	4499
8767	2211	4870	3637
8768	2211	2213	4870
8769	152	4795	1870
8770	152	1867	4795
8771	1867	4496	3632
8772	1867	635	4496
8773	1870	3632	4569
8774	1870	4569	873
8775	3632	1870	4795
8776	3632	4795	1867

8777	3630	4500	5090
8778	4500	3637	5090
8779	4500	637	3637
8780	3632	5091	4569
8781	4569	5091	873
8782	5091	3637	873
8783	635	4494	4496
8784	4494	3632	4496
8785	4494	3630	3632
8786	3637	5091	5090
8787	5090	5091	3630
8788	5091	3632	3630
8789	1437	4247	4759
8790	4247	1436	4759
8791	4247	70	1436
8792	3634	4760	4497
8793	4497	4760	636
8794	4760	1436	636
8795	637	4498	4501
8796	4498	3634	4501
8797	4498	1437	3634
8798	1436	4760	4759
8799	4759	4760	1437
8800	4760	3634	1437
8801	2211	4869	4283
8802	4283	4869	210
8803	4869	2215	210
8804	637	4502	4499
8805	4499	4502	2211
8806	4502	3638	2211
8807	3638	4636	4871
8808	4636	2215	4871
8809	4636	1010	2215
8810	2215	4869	4871
8811	4869	3638	4871
8812	4869	2211	3638
8813	3634	4497	5093
8814	4497	3636	5093
8815	4497	636	3636
8816	3638	5094	4636
8817	4636	5094	1010
8818	5094	3636	1010
8819	637	4501	4502
8820	4501	3638	4502
8821	4501	3634	3638
8822	3636	5094	5093
8823	5093	5094	3634
8824	5094	3638	3634
8825	2202	4282	4866
8826	4282	2198	4866
8827	4282	208	2198
8828	3065	4867	4369
8829	4369	4867	436
8830	4867	2198	436
8831	1006	4634	4635
8832	4634	3065	4635
8833	4634	2202	3065
8834	2198	4867	4866
8835	4866	4867	2202

8836	4867	3065	2202
8837	434	3060	5025
8838	434	5025	3058
8839	3060	4635	3065
8840	3060	1006	4635
8841	3058	3065	4369
8842	3058	4369	436
8843	3065	5025	3060
8844	3065	3058	5025
8845	2203	4282	2202
8846	2203	208	4282
8847	4143	2202	4634
8848	4143	4634	1006
8849	1010	4868	4143
8850	1010	2203	4868
8851	2202	4143	4868
8852	2202	4868	2203
8853	2452	4306	2450
8854	2452	250	4306
8855	3316	2450	4400
8856	3316	4400	528
8857	763	4936	3316
8858	763	2452	4936
8859	2450	3316	4936
8860	2450	4936	2452
8861	1862	4260	4793
8862	4260	151	4793
8863	4793	151	1864
8864	2855	4794	4568
8865	4568	4794	1864
8866	4568	1864	870
8867	359	4337	4338
8868	4337	1862	4338
8869	4338	1862	2855
8870	1864	4794	4793
8871	4793	4794	2855
8872	4793	2855	1862
8873	2854	4338	2855
8874	2854	359	4338
8875	2859	2855	4568
8876	2859	4568	870
8877	361	5009	2859
8878	361	2854	5009
8879	2855	2859	5009
8880	2855	5009	2854
8881	2481	2485	4315
8882	2481	4315	255
8883	661	3710	4956
8884	661	4956	2481
8885	3710	4669	2485
8886	3710	1053	4669
8887	2485	4956	3710
8888	2485	2481	4956
8889	2454	2450	4306
8890	2454	4306	250
8891	1051	3317	4937
8892	1051	4937	2454
8893	3317	4400	2450
8894	3317	528	4400

8895	2450	4937	3317
8896	2450	2454	4937
8897	251	4938	2460
8898	251	2456	4938
8899	2456	4401	3319
8900	2456	529	4401
8901	2460	3319	4668
8902	2460	4668	1051
8903	3319	2460	4938
8904	3319	4938	2456
8905	528	3317	5043
8906	528	5043	3315
8907	3317	4668	3319
8908	3317	1051	4668
8909	3315	3319	4401
8910	3315	4401	529
8911	3319	5043	3317
8912	3319	3315	5043
8913	2484	4315	2485
8914	2484	255	4315
8915	4189	2485	4669
8916	4189	4669	1053
8917	1052	4957	4189
8918	1052	2484	4957
8919	2485	4189	4957
8920	2485	4957	2484
8921	70	4757	1439
8922	70	1435	4757
8923	1435	4495	3631
8924	1435	635	4495
8925	1439	3631	4503
8926	1439	4503	639
8927	3631	1439	4757
8928	3631	4757	1435
8929	3633	3631	4495
8930	3633	4495	635
8931	874	3642	5092
8932	874	5092	3633
8933	3642	4503	3631
8934	3642	639	4503
8935	3631	5092	3642
8936	3631	3633	5092
8937	279	2628	4993
8938	279	4993	2624
8939	2628	4680	3643
8940	2628	1078	4680
8941	2624	3643	4504
8942	2624	4504	639
8943	3643	4993	2628
8944	3643	2624	4993
8945	3639	4504	3643
8946	3639	639	4504
8947	3641	3643	4680
8948	3641	4680	1078
8949	638	5095	3641
8950	638	3639	5095
8951	3643	3641	5095
8952	3643	5095	3639
8953	1223	4692	4229

8954	4229	4692	30
8955	4692	1221	30
8956	423	4353	4352
8957	4352	4353	1223
8958	4353	3025	1223
8959	3025	4350	4693
8960	4350	1221	4693
8961	4350	421	1221
8962	1221	4692	4693
8963	4692	3025	4693
8964	4692	1223	3025
8965	3030	3025	4353
8966	3030	4353	423
8967	824	5015	3030
8968	824	3026	5015
8969	3026	4350	3025
8970	3026	421	4350
8971	3025	3030	5015
8972	3025	5015	3026
8973	1401	4244	4746
8974	4244	1399	4746
8975	4244	64	1399
8976	3522	4747	4472
8977	4472	4747	599
8978	4747	1399	599
8979	601	4474	4475
8980	4474	3522	4475
8981	4474	1401	3522
8982	1399	4747	4746
8983	4746	4747	1401
8984	4747	3522	1401
8985	202	4851	2165
8986	202	2163	4851
8987	2163	4477	3529
8988	2163	601	4477
8989	2165	3529	4564
8990	2165	4564	828
8991	3529	2165	4851
8992	3529	4851	2163
8993	3522	4472	5078
8994	4472	3524	5078
8995	4472	599	3524
8996	3529	5079	4564
8997	4564	5079	828
8998	5079	3524	828
8999	601	4475	4477
9000	4475	3529	4477
9001	4475	3522	3529
9002	3524	5079	5078
9003	5078	5079	3522
9004	5079	3529	3522
9005	1401	4748	4244
9006	4244	4748	64
9007	4748	1400	64
9008	601	4476	4474
9009	4474	4476	1401
9010	4476	3526	1401
9011	3526	4473	4749
9012	4473	1400	4749

9013	4473	600	1400
9014	1400	4748	4749
9015	4748	3526	4749
9016	4748	1401	3526
9017	2167	4852	4278
9018	4278	4852	202
9019	4852	2163	202
9020	1002	4630	4629
9021	4629	4630	2167
9022	4630	3530	2167
9023	3530	4478	4853
9024	4478	2163	4853
9025	4478	601	2163
9026	2163	4852	4853
9027	4852	3530	4853
9028	4852	2167	3530
9029	2157	2161	4277
9030	2157	4277	201
9031	600	3528	4848
9032	600	4848	2157
9033	3528	4628	2161
9034	3528	1002	4628
9035	2161	4848	3528
9036	2161	2157	4848
9037	601	4478	4476
9038	4478	3530	4476
9039	4476	3530	3526
9040	3530	4630	5081
9041	4630	1002	5081
9042	5081	1002	3528
9043	3526	5080	4473
9044	4473	5080	3528
9045	4473	3528	600
9046	3528	5080	5081
9047	5081	5080	3526
9048	5081	3526	3530
9049	1223	4229	4694
9050	4229	30	4694
9051	4694	30	1222
9052	3027	4695	4351
9053	4351	4695	1222
9054	4351	1222	422
9055	423	4352	4354
9056	4352	1223	4354
9057	4354	1223	3027
9058	1222	4695	4694
9059	4694	4695	3027
9060	4694	3027	1223
9061	202	4850	2166
9062	202	2162	4850
9063	2162	4355	3031
9064	2162	423	4355
9065	2166	3031	4626
9066	2166	4626	1001
9067	3031	2166	4850
9068	3031	4850	2162
9069	3027	4351	5016
9070	4351	3029	5016
9071	4351	422	3029

9072	3031	5017	4626
9073	4626	5017	1001
9074	5017	3029	1001
9075	423	4354	4355
9076	4354	3031	4355
9077	4354	3027	3031
9078	3029	5017	5016
9079	5016	5017	3027
9080	5017	3031	3027
9081	2160	4277	2161
9082	2160	201	4277
9083	4137	2161	4628
9084	4137	4628	1002
9085	1000	4849	4137
9086	1000	2160	4849
9087	2161	4137	4849
9088	2161	4849	2160
9089	2166	2167	4278
9090	2166	4278	202
9091	1001	4138	4854
9092	1001	4854	2166
9093	4138	4629	2167
9094	4138	1002	4629
9095	2167	4854	4138
9096	2167	2166	4854
9097	200	4847	2155
9098	200	2154	4847
9099	2154	4625	4136
9100	2154	1000	4625
9101	2155	4136	4627
9102	2155	4627	1001
9103	4136	2155	4847
9104	4136	4847	2154
9105	1002	4138	5125
9106	1002	5125	4137
9107	4138	4627	4136
9108	4138	1001	4627
9109	4137	4136	4625
9110	4137	4625	1000
9111	4136	5125	4138
9112	4136	4137	5125
9113	1404	1406	4245
9114	1404	4245	65
9115	604	3536	4750
9116	604	4750	1404
9117	3536	4480	1406
9118	3536	606	4480
9119	1406	4750	3536
9120	1406	1404	4750
9121	1760	4787	4257
9122	4257	4787	131
9123	4787	1757	131
9124	821	4563	4561
9125	4561	4563	1760
9126	4563	3538	1760
9127	3538	4479	4788
9128	4479	1757	4788
9129	4479	604	1757
9130	1757	4787	4788

9131	4787	3538	4788
9132	4787	1760	3538
9133	215	4284	4285
9134	4284	2243	4285
9135	4284	2241	2243
9136	2241	4481	4873
9137	4481	3545	4873
9138	4481	606	3545
9139	2243	4875	4562
9140	4562	4875	821
9141	4875	3545	821
9142	3545	4875	4873
9143	4873	4875	2241
9144	4875	2243	2241
9145	3536	4479	3538
9146	3536	604	4479
9147	3545	3538	4563
9148	3545	4563	821
9149	606	5082	3545
9150	606	3536	5082
9151	3538	3545	5082
9152	3538	5082	3536
9153	129	1749	4786
9154	129	4786	1748
9155	1749	4560	3953
9156	1749	817	4560
9157	1748	3953	4559
9158	1748	4559	816
9159	3953	4786	1749
9160	3953	1748	4786
9161	2242	4285	2243
9162	2242	215	4285
9163	3955	2243	4562
9164	3955	4562	821
9165	817	4874	3955
9166	817	2242	4874
9167	2243	3955	4874
9168	2243	4874	2242
9169	1758	1760	4257
9170	1758	4257	131
9171	816	3954	4789
9172	816	4789	1758
9173	3954	4561	1760
9174	3954	821	4561
9175	1760	4789	3954
9176	1760	1758	4789
9177	3955	3953	4560
9178	3955	4560	817
9179	821	5117	3955
9180	821	3954	5117
9181	3954	4559	3953
9182	3954	816	4559
9183	3953	3955	5117
9184	3953	5117	3954
9185	1407	4245	1406
9186	1407	65	4245
9187	3544	1406	4480
9188	3544	4480	606
9189	607	4751	3544

9190	607	1407	4751
9191	1406	3544	4751
9192	1406	4751	1407
9193	2241	4284	4872
9194	4284	2245	4872
9195	4284	215	2245
9196	3546	4876	4638
9197	4638	4876	1017
9198	4876	2245	1017
9199	606	4481	4482
9200	4481	3546	4482
9201	4481	2241	3546
9202	2245	4876	4872
9203	4872	4876	2241
9204	4876	3546	2241
9205	2269	4289	4885
9206	4289	219	4885
9207	4885	219	2265
9208	3548	4886	4483
9209	4483	4886	2265
9210	4483	2265	607
9211	1017	4637	4639
9212	4637	2269	4639
9213	4639	2269	3548
9214	2265	4886	4885
9215	4885	4886	3548
9216	4885	3548	2269
9217	3546	5083	4482
9218	4482	5083	606
9219	5083	3544	606
9220	1017	4639	4638
9221	4638	4639	3546
9222	4639	3548	3546
9223	3548	4483	5084
9224	4483	3544	5084
9225	4483	607	3544
9226	3544	5083	5084
9227	5083	3548	5084
9228	5083	3546	3548
9229	2268	4289	2269
9230	2268	219	4289
9231	4153	2269	4637
9232	4153	4637	1017
9233	1016	4887	4153
9234	1016	2268	4887
9235	2269	4153	4887
9236	2269	4887	2268
9237	2438	4302	2440
9238	2438	248	4302
9239	3307	2440	4552
9240	3307	4552	761
9241	524	4928	3307
9242	524	2438	4928
9243	2440	3307	4928
9244	2440	4928	2438
9245	2439	2441	4303
9246	2439	4303	248
9247	532	3329	4930
9248	532	4930	2439

9249	3329	4553	2441
9250	3329	765	4553
9251	2441	4930	3329
9252	2441	2439	4930
9253	248	4303	4302
9254	4303	2441	4302
9255	4302	2441	2440
9256	2441	4553	4933
9257	4553	765	4933
9258	4933	765	3898
9259	2440	4932	4552
9260	4552	4932	3898
9261	4552	3898	761
9262	3898	4932	4933
9263	4933	4932	2440
9264	4933	2440	2441
9265	1323	1325	4234
9266	1323	4234	50
9267	523	3304	4710
9268	523	4710	1323
9269	3304	4399	1325
9270	3304	525	4399
9271	1325	4710	3304
9272	1325	1323	4710
9273	1324	4234	1325
9274	1324	50	4234
9275	3306	1325	4399
9276	3306	4399	525
9277	524	4711	3306
9278	524	1324	4711
9279	1325	3306	4711
9280	1325	4711	1324
9281	2442	4304	4927
9282	4304	2438	4927
9283	4304	248	2438
9284	3308	4929	4398
9285	4398	4929	524
9286	4929	2438	524
9287	1048	4665	4666
9288	4665	3308	4666
9289	4665	2442	3308
9290	2438	4929	4927
9291	4927	4929	2442
9292	4929	3308	2442
9293	525	3310	5042
9294	525	5042	3306
9295	3310	4666	3308
9296	3310	1048	4666
9297	3306	3308	4398
9298	3306	4398	524
9299	3308	5042	3310
9300	3308	3306	5042
9301	2439	4305	2443
9302	2439	248	4305
9303	3330	2443	4667
9304	3330	4667	1049
9305	532	4931	3330
9306	532	2439	4931
9307	2443	3330	4931

9308	2443	4931	2439
9309	248	4304	4305
9310	4304	2442	4305
9311	4305	2442	2443
9312	2442	4665	4934
9313	4665	1048	4934
9314	4934	1048	4184
9315	2443	4935	4667
9316	4667	4935	4184
9317	4667	4184	1049
9318	4184	4935	4934
9319	4934	4935	2443
9320	4934	2443	2442
9321	77	4765	1479
9322	77	1478	4765
9323	1478	4519	3760
9324	1478	678	4519
9325	1479	3760	4520
9326	1479	4520	679
9327	3760	1479	4765
9328	3760	4765	1478
9329	2391	4297	2395
9330	2391	240	4297
9331	3762	2395	4661
9332	3762	4661	1044
9333	678	4912	3762
9334	678	2391	4912
9335	2395	3762	4912
9336	2395	4912	2391
9337	2421	2425	4300
9338	2421	4300	245
9339	679	3764	4923
9340	679	4923	2421
9341	3764	4662	2425
9342	3764	1044	4662
9343	2425	4923	3764
9344	2425	2421	4923
9345	3762	3760	4519
9346	3762	4519	678
9347	1044	3764	5105
9348	1044	5105	3762
9349	3764	4520	3760
9350	3764	679	4520
9351	3760	5105	3764
9352	3760	3762	5105
9353	2424	4300	2425
9354	2424	245	4300
9355	4180	2425	4662
9356	4180	4662	1044
9357	1043	4924	4180
9358	1043	2424	4924
9359	2425	4180	4924
9360	2425	4924	2424
9361	2394	2395	4297
9362	2394	4297	240
9363	1042	4179	4913
9364	1042	4913	2394
9365	4179	4661	2395
9366	4179	1044	4661

9367	2395	4913	4179
9368	2395	2394	4913
9369	238	2383	4911
9370	238	4911	2382
9371	2383	4660	4178
9372	2383	1043	4660
9373	2382	4178	4659
9374	2382	4659	1042
9375	4178	4911	2383
9376	4178	2382	4911
9377	1044	5130	4180
9378	1044	4179	5130
9379	4179	4659	4178
9380	4179	1042	4659
9381	4180	4178	4660
9382	4180	4660	1043
9383	4178	4180	5130
9384	4178	5130	4179
9385	252	4308	4307
9386	4308	2462	4307
9387	4308	2464	2462
9388	2464	4554	4943
9389	4554	3332	4943
9390	4554	766	3332
9391	2462	4939	4402
9392	4402	4939	533
9393	4939	3332	533
9394	3332	4939	4943
9395	4943	4939	2464
9396	4939	2462	2464
9397	2463	2465	4309
9398	2463	4309	252
9399	539	3350	4941
9400	539	4941	2463
9401	3350	4555	2465
9402	3350	768	4555
9403	2465	4941	3350
9404	2465	2463	4941
9405	252	4309	4308
9406	4309	2465	4308
9407	4308	2465	2464
9408	2465	4555	4945
9409	4555	768	4945
9410	4945	768	3904
9411	2464	4944	4554
9412	4554	4944	3904
9413	4554	3904	766
9414	3904	4944	4945
9415	4945	4944	2464
9416	4945	2464	2465
9417	1482	4251	1484
9418	1482	78	4251
9419	3770	1484	4525
9420	3770	4525	684
9421	682	4766	3770
9422	682	1482	4766
9423	1484	3770	4766
9424	1484	4766	1482
9425	2477	2475	4313

9426	2477	4313	254
9427	920	3779	4952
9428	920	4952	2477
9429	3779	4526	2475
9430	3779	684	4526
9431	2475	4952	3779
9432	2475	2477	4952
9433	169	4826	1968
9434	169	1965	4826
9435	1965	4521	3772
9436	1965	682	4521
9437	1968	3772	4601
9438	1968	4601	920
9439	3772	1968	4826
9440	3772	4826	1965
9441	684	3779	5106
9442	684	5106	3770
9443	3779	4601	3772
9444	3779	920	4601
9445	3770	3772	4521
9446	3770	4521	682
9447	3772	5106	3779
9448	3772	3770	5106
9449	2474	4949	4312
9450	4312	4949	254
9451	4949	2476	254
9452	535	4404	4403
9453	4403	4404	2474
9454	4404	3336	2474
9455	3336	4597	4951
9456	4597	2476	4951
9457	4597	911	2476
9458	2476	4949	4951
9459	4949	3336	4951
9460	4949	2474	3336
9461	531	3327	5044
9462	531	5044	3325
9463	3327	4597	3336
9464	3327	911	4597
9465	3325	3336	4404
9466	3325	4404	535
9467	3336	5044	3327
9468	3336	3325	5044
9469	256	4318	4316
9470	4318	2486	4316
9471	4318	2488	2486
9472	2488	4617	4964
9473	4617	3354	4964
9474	4617	965	3354
9475	2486	4958	4405
9476	4405	4958	541
9477	4958	3354	541
9478	3354	4958	4964
9479	4964	4958	2488
9480	4958	2486	2488
9481	1485	4250	1483
9482	1485	78	4250
9483	3774	1483	4523
9484	3774	4523	683

9485	685	4768	3774
9486	685	1485	4768
9487	1483	3774	4768
9488	1483	4768	1485
9489	2076	2073	4275
9490	2076	4275	187
9491	974	3776	4842
9492	974	4842	2076
9493	3776	4524	2073
9494	3776	683	4524
9495	2073	4842	3776
9496	2073	2076	4842
9497	256	4317	4319
9498	2487	4960	4317
9499	2489	4319	4960
9500	4317	4960	4319
9501	685	4528	4527
9502	2487	4527	4962
9503	3781	4962	4528
9504	4527	4528	4962
9505	974	4618	4619
9506	2489	4966	4618
9507	3781	4619	4966
9508	4618	4966	4619
9509	2487	4962	4960
9510	2489	4960	4966
9511	3781	4966	4962
9512	4960	4962	4966
9513	683	3776	5108
9514	683	5108	3774
9515	3776	4619	3781
9516	3776	974	4619
9517	3774	3781	4528
9518	3774	4528	685
9519	3781	5108	3776
9520	3781	3774	5108
9521	256	4319	4318
9522	4319	2488	4318
9523	4319	2489	2488
9524	2489	4618	4967
9525	4618	4105	4967
9526	4618	974	4105
9527	2488	4965	4617
9528	4617	4965	965
9529	4965	4105	965
9530	4105	4965	4967
9531	4967	4965	2489
9532	4965	2488	2489
9533	254	4314	4312
9534	4314	2478	4312
9535	4312	2478	2474
9536	2478	4671	4953
9537	4671	1054	4953
9538	4953	1054	3337
9539	2474	4950	4403
9540	4403	4950	3337
9541	4403	3337	535
9542	3337	4950	4953
9543	4953	4950	2474

9544	4953	2474	2478
9545	252	4307	4310
9546	4307	2462	4310
9547	4310	2462	2466
9548	2462	4402	4940
9549	4402	533	4940
9550	4940	533	3333
9551	2466	4946	4670
9552	4670	4946	3333
9553	4670	3333	1054
9554	3333	4946	4940
9555	4940	4946	2466
9556	4940	2466	2462
9557	1485	1484	4251
9558	1485	4251	78
9559	685	3778	4769
9560	685	4769	1485
9561	3778	4525	1484
9562	3778	684	4525
9563	1484	4769	3778
9564	1484	1485	4769
9565	256	4321	4317
9566	2491	4961	4321
9567	2487	4317	4961
9568	4321	4961	4317
9569	1056	4675	4674
9570	2491	4674	4970
9571	3782	4970	4675
9572	4674	4675	4970
9573	685	4527	4529
9574	2487	4963	4527
9575	3782	4529	4963
9576	4527	4963	4529
9577	2491	4970	4961
9578	2487	4961	4963
9579	3782	4963	4970
9580	4961	4970	4963
9581	2479	4313	2475
9582	2479	254	4313
9583	3780	2475	4526
9584	3780	4526	684
9585	1056	4954	3780
9586	1056	2479	4954
9587	2475	3780	4954
9588	2475	4954	2479
9589	3778	4529	3782
9590	3778	685	4529
9591	3780	3782	4675
9592	3780	4675	1056
9593	684	5110	3780
9594	684	3778	5110
9595	3782	3780	5110
9596	3782	5110	3778
9597	2463	4311	2467
9598	2463	252	4311
9599	3351	2467	4672
9600	3351	4672	1055
9601	539	4942	3351
9602	539	2463	4942

9603	2467	3351	4942
9604	2467	4942	2463
9605	256	4316	4320
9606	4316	2486	4320
9607	4320	2486	2490
9608	2486	4405	4959
9609	4405	541	4959
9610	4959	541	3355
9611	2490	4968	4673
9612	4673	4968	3355
9613	4673	3355	1055
9614	3355	4968	4959
9615	4959	4968	2490
9616	4959	2490	2486
9617	2479	2478	4314
9618	2479	4314	254
9619	1056	4191	4955
9620	1056	4955	2479
9621	4191	4671	2478
9622	4191	1054	4671
9623	2478	4955	4191
9624	2478	2479	4955
9625	256	4320	4321
9626	4320	2490	4321
9627	4321	2490	2491
9628	2490	4673	4969
9629	4673	1055	4969
9630	4969	1055	4192
9631	2491	4971	4674
9632	4674	4971	4192
9633	4674	4192	1056
9634	4192	4971	4969
9635	4969	4971	2491
9636	4969	2491	2490
9637	252	4310	4311
9638	4310	2467	4311
9639	4310	2466	2467
9640	2466	4670	4947
9641	4670	4190	4947
9642	4670	1054	4190
9643	2467	4948	4672
9644	4672	4948	1055
9645	4948	4190	1055
9646	4190	4948	4947
9647	4947	4948	2466
9648	4948	2467	2466
9649	1470	4249	1472
9650	1470	76	4249
9651	3734	1472	4516
9652	3734	4516	672
9653	670	4763	3734
9654	670	1470	4763
9655	1472	3734	4763
9656	1472	4763	1470
9657	2261	2259	4288
9658	2261	4288	218
9659	900	3743	4881
9660	900	4881	2261
9661	3743	4517	2259

9662	3743	672	4517
9663	2259	4881	3743
9664	2259	2261	4881
9665	162	4822	1926
9666	162	1923	4822
9667	1923	4515	3736
9668	1923	670	4515
9669	1926	3736	4596
9670	1926	4596	900
9671	3736	1926	4822
9672	3736	4822	1923
9673	672	3743	5103
9674	672	5103	3734
9675	3743	4596	3736
9676	3743	900	4596
9677	3734	3736	4515
9678	3734	4515	670
9679	3736	5103	3743
9680	3736	3734	5103
9681	1255	1259	4232
9682	1255	4232	38
9683	455	3109	4701
9684	455	4701	1255
9685	3109	4376	1259
9686	3109	459	4376
9687	1259	4701	3109
9688	1259	1255	4701
9689	158	1903	4814
9690	158	4814	1899
9691	1903	4592	3111
9692	1903	889	4592
9693	1899	3111	4373
9694	1899	4373	455
9695	3111	4814	1903
9696	3111	1899	4814
9697	2260	4287	2258
9698	2260	218	4287
9699	3120	2258	4377
9700	3120	4377	459
9701	889	4880	3120
9702	889	2260	4880
9703	2258	3120	4880
9704	2258	4880	2260
9705	3109	4373	3111
9706	3109	455	4373
9707	3120	3111	4592
9708	3120	4592	889
9709	459	5028	3120
9710	459	3109	5028
9711	3111	3120	5028
9712	3111	5028	3109
9713	1257	4232	1259
9714	1257	38	4232
9715	3115	1259	4376
9716	3115	4376	459
9717	457	4702	3115
9718	457	1257	4702
9719	1259	3115	4702
9720	1259	4702	1257

9721	2262	2258	4287
9722	2262	4287	218
9723	1018	3121	4883
9724	1018	4883	2262
9725	3121	4377	2258
9726	3121	459	4377
9727	2258	4883	3121
9728	2258	2262	4883
9729	216	4877	2250
9730	216	2246	4877
9731	2246	4374	3117
9732	2246	457	4374
9733	2250	3117	4640
9734	2250	4640	1018
9735	3117	2250	4877
9736	3117	4877	2246
9737	459	3121	5030
9738	459	5030	3115
9739	3121	4640	3117
9740	3121	1018	4640
9741	3115	3117	4374
9742	3115	4374	457
9743	3117	5030	3121
9744	3117	3115	5030
9745	1473	1472	4249
9746	1473	4249	76
9747	673	3742	4764
9748	673	4764	1473
9749	3742	4516	1472
9750	3742	672	4516
9751	1472	4764	3742
9752	1472	1473	4764
9753	2275	4888	4290
9754	4290	4888	220
9755	4888	2271	220
9756	1020	4644	4643
9757	4643	4644	2275
9758	4644	3746	2275
9759	3746	4518	4889
9760	4518	2271	4889
9761	4518	673	2271
9762	2271	4888	4889
9763	4888	3746	4889
9764	4888	2275	3746
9765	2263	4288	2259
9766	2263	218	4288
9767	3744	2259	4517
9768	3744	4517	672
9769	1020	4884	3744
9770	1020	2263	4884
9771	2259	3744	4884
9772	2259	4884	2263
9773	3742	4518	3746
9774	3742	673	4518
9775	3744	3746	4644
9776	3744	4644	1020
9777	672	5104	3744
9778	672	3742	5104
9779	3746	3744	5104

9780	3746	5104	3742
9781	218	2263	4882
9782	218	4882	2262
9783	2263	4645	4155
9784	2263	1020	4645
9785	2262	4155	4641
9786	2262	4641	1018
9787	4155	4882	2263
9788	4155	2262	4882
9789	2275	4290	4890
9790	4290	220	4890
9791	4890	220	2274
9792	4156	4891	4642
9793	4642	4891	2274
9794	4642	2274	1019
9795	1020	4643	4646
9796	4643	2275	4646
9797	4646	2275	4156
9798	2274	4891	4890
9799	4890	4891	4156
9800	4890	4156	2275
9801	1020	4646	4645
9802	4646	4155	4645
9803	4646	4156	4155
9804	4156	4642	5127
9805	4642	4154	5127
9806	4642	1019	4154
9807	4155	5126	4641
9808	4641	5126	1018
9809	5126	4154	1018
9810	4154	5126	5127
9811	5127	5126	4156
9812	5126	4155	4156
9813	1487	1483	4250
9814	1487	4250	78
9815	687	3775	4770
9816	687	4770	1487
9817	3775	4523	1483
9818	3775	683	4523
9819	1483	4770	3775
9820	1483	1487	4770
9821	286	2669	5002
9822	286	5002	2667
9823	2669	4620	3786
9824	2669	975	4620
9825	2667	3786	4532
9826	2667	4532	687
9827	3786	5002	2669
9828	3786	2667	5002
9829	2077	4275	2073
9830	2077	187	4275
9831	3777	2073	4524
9832	3777	4524	683
9833	975	4843	3777
9834	975	2077	4843
9835	2073	3777	4843
9836	2073	4843	2077
9837	3775	4532	3786
9838	3775	687	4532

9839	3777	3786	4620
9840	3777	4620	975
9841	683	5109	3777
9842	683	3775	5109
9843	3786	3777	5109
9844	3786	5109	3775
9845	21	4687	1177
9846	21	1176	4687
9847	1176	4342	2901
9848	1176	376	4342
9849	1177	2901	4343
9850	1177	4343	377
9851	2901	1177	4687
9852	2901	4687	1176
9853	1958	4268	1961
9854	1958	168	4268
9855	2903	1961	4598
9856	2903	4598	917
9857	376	4823	2903
9858	376	1958	4823
9859	1961	2903	4823
9860	1961	4823	1958
9861	1964	1967	4269
9862	1964	4269	169
9863	377	2905	4825
9864	377	4825	1964
9865	2905	4599	1967
9866	2905	917	4599
9867	1967	4825	2905
9868	1967	1964	4825
9869	2903	2901	4342
9870	2903	4342	376
9871	917	2905	5012
9872	917	5012	2903
9873	2905	4343	2901
9874	2905	377	4343
9875	2901	5012	2905
9876	2901	2903	5012
9877	1482	1486	4252
9878	1482	4252	78
9879	682	3771	4767
9880	682	4767	1482
9881	3771	4530	1486
9882	3771	686	4530
9883	1486	4767	3771
9884	1486	1482	4767
9885	169	1969	4827
9886	169	4827	1965
9887	1969	4602	3773
9888	1969	921	4602
9889	1965	3773	4522
9890	1965	4522	682
9891	3773	4827	1969
9892	3773	1965	4827
9893	2663	4328	2661
9894	2663	285	4328
9895	3784	2661	4531
9896	3784	4531	686
9897	921	5000	3784

9898	921	2663	5000
9899	2661	3784	5000
9900	2661	5000	2663
9901	3771	4522	3773
9902	3771	682	4522
9903	3784	3773	4602
9904	3784	4602	921
9905	686	5107	3784
9906	686	3771	5107
9907	3773	3784	5107
9908	3773	5107	3771
9909	1963	1961	4268
9910	1963	4268	168
9911	919	4055	4824
9912	919	4824	1963
9913	4055	4598	1961
9914	4055	917	4598
9915	1961	4824	4055
9916	1961	1963	4824
9917	285	2663	4999
9918	285	4999	2662
9919	2663	4603	4057
9920	2663	921	4603
9921	2662	4057	4600
9922	2662	4600	919
9923	4057	4999	2663
9924	4057	2662	4999
9925	1969	4269	1967
9926	1969	169	4269
9927	4056	1967	4599
9928	4056	4599	917
9929	921	4828	4056
9930	921	1969	4828
9931	1967	4056	4828
9932	1967	4828	1969
9933	4055	4600	4057
9934	4055	919	4600
9935	4056	4057	4603
9936	4056	4603	921
9937	917	5123	4056
9938	917	4055	5123
9939	4057	4056	5123
9940	4057	5123	4055
9941	1487	4252	1486
9942	1487	78	4252
9943	3783	1486	4530
9944	3783	4530	686
9945	687	4771	3783
9946	687	1487	4771
9947	1486	3783	4771
9948	1486	4771	1487
9949	2665	2661	4328
9950	2665	4328	285
9951	1086	3785	5001
9952	1086	5001	2665
9953	3785	4531	2661
9954	3785	686	4531
9955	2661	5001	3785
9956	2661	2665	5001

9957	286	5003	2671
9958	286	2667	5003
9959	2667	4533	3787
9960	2667	687	4533
9961	2671	3787	4681
9962	2671	4681	1086
9963	3787	2671	5003
9964	3787	5003	2667
9965	686	3785	5111
9966	686	5111	3783
9967	3785	4681	3787
9968	3785	1086	4681
9969	3783	3787	4533
9970	3783	4533	687
9971	3787	5111	3785
9972	3787	3783	5111
9973	4806	4264	5133
9974	4806	5133	1893
9975	1896	5131	4264
9976	1896	157	5131
9977	4507	4506	5132
9978	4507	5132	664
9979	3718	5134	4506
9980	3718	1893	5134
9981	4582	4811	5137
9982	4582	5137	3718
9983	884	5135	4811
9984	884	1896	5135
9985	4806	4811	5135
9986	4806	5135	1896
9987	1893	5137	4811
9988	1893	3718	5137
9989	4807	5133	4264
9990	4807	1893	5133
9991	1897	4264	5131
9992	1897	5131	157
9993	4586	5138	4813
9994	4586	3719	5138
9995	885	4813	5136
9996	885	5136	1897
9997	4508	5132	4506
9998	4508	664	5132
9999	3719	4506	5134
10000	3719	5134	1893
10001	4807	5136	4813
10002	4807	1897	5136
10003	1893	4813	5138
10004	1893	5138	3719

End of Connectivity Matrix of Nodes

DESIGN 4

Node Coordinates

Node Index	X(m)	Y(m)	Z(m)
1	0.00000000	0.00000000	0.16715543
2	0.00000000	4.00000000	0.19296188
3	4.00000000	0.00000000	0.17966764
4	4.00000000	4.00000000	0.20000000
5	0.00000000	2.00000000	-0.14760508
6	2.00000000	0.00000000	0.17067449
7	2.00000000	2.00000000	-0.04477028
8	2.00000000	4.00000000	0.08895406
9	4.00000000	2.00000000	-0.12140762
10	0.00000000	1.00000000	-0.06471163
11	1.00000000	0.00000000	-0.05650049
12	1.00000000	1.00000000	-0.20000000
13	0.00000000	3.00000000	0.06901271
14	1.00000000	4.00000000	0.19960899
15	3.00000000	0.00000000	-0.02991202
16	4.00000000	1.00000000	-0.10694037
17	3.00000000	3.00000000	-0.17497556
18	3.00000000	4.00000000	0.19765396
19	4.00000000	3.00000000	0.03773216
20	1.00000000	2.00000000	-0.19569892
21	1.00000000	3.00000000	-0.05063539
22	2.00000000	1.00000000	-0.07057674
23	3.00000000	1.00000000	-0.19648094
24	2.00000000	3.00000000	0.16754643
25	3.00000000	2.00000000	-0.20000000
26	0.00000000	0.50000000	-0.04555230
27	0.50000000	0.00000000	0.06627566
28	0.50000000	0.50000000	-0.13196481
29	0.00000000	3.50000000	0.17732160
30	0.50000000	4.00000000	0.19648094
31	3.50000000	0.00000000	0.04946237
32	4.00000000	0.50000000	0.02170088
33	3.50000000	3.50000000	0.17458456
34	3.50000000	4.00000000	0.19882698
35	4.00000000	3.50000000	0.14486804
36	0.00000000	1.50000000	-0.16715543
37	0.00000000	2.50000000	0.01075269
38	0.50000000	2.00000000	-0.17028348
39	0.50000000	2.50000000	-0.09130010
40	1.50000000	0.00000000	0.15151515
41	2.50000000	0.00000000	0.15816227
42	2.00000000	0.50000000	0.01348974
43	2.50000000	0.50000000	-0.00097752
44	1.50000000	1.50000000	-0.14095797
45	2.50000000	2.50000000	-0.00488759
46	1.50000000	2.00000000	-0.01348974
47	2.00000000	1.50000000	-0.05884653
48	2.00000000	2.50000000	0.06275660
49	2.50000000	2.00000000	-0.10029326
50	1.50000000	4.00000000	-0.10029326
51	2.50000000	4.00000000	-0.19687195
52	1.50000000	3.50000000	0.17497556
53	2.00000000	3.50000000	0.18709677
54	4.00000000	1.50000000	-0.04046921
55	4.00000000	2.50000000	-0.05571848
56	3.50000000	1.50000000	-0.17341153

57	3.50000000	2.00000000	-0.16246334
58	0.50000000	1.00000000	-0.19530792
59	0.50000000	1.50000000	-0.19296188
60	1.00000000	0.50000000	-0.13431085
61	1.50000000	0.50000000	0.13040078
62	1.00000000	1.50000000	-0.19726295
63	1.50000000	1.00000000	-0.13587488
64	0.50000000	3.50000000	0.18123167
65	0.50000000	3.00000000	0.05610948
66	1.00000000	3.50000000	0.07448680
67	3.50000000	0.50000000	-0.10576735
68	3.00000000	0.50000000	-0.13822092
69	3.50000000	1.00000000	-0.16441838
70	3.00000000	3.50000000	0.12101662
71	3.50000000	3.00000000	0.03460411
72	2.50000000	3.00000000	0.09951124
73	3.00000000	2.50000000	-0.09833822
74	2.50000000	3.50000000	0.20000000
75	3.50000000	2.50000000	-0.09325513
76	1.00000000	2.50000000	-0.17458456
77	1.50000000	2.50000000	-0.07448680
78	1.50000000	3.00000000	0.15855327
79	2.50000000	1.00000000	-0.10850440
80	2.50000000	1.50000000	-0.12258065
81	3.00000000	1.50000000	-0.18553275
82	0.00000000	0.25000000	0.04672532
83	0.25000000	0.00000000	0.09012708
84	0.25000000	0.25000000	-0.01192571
85	0.00000000	3.75000000	0.18553275
86	0.25000000	4.00000000	0.19491691
87	3.75000000	0.00000000	0.11476051
88	4.00000000	0.25000000	0.09599218
89	3.75000000	3.75000000	0.16129032
90	3.75000000	4.00000000	0.19960899
91	4.00000000	3.75000000	0.17341153
92	0.00000000	1.75000000	-0.15816227
93	0.00000000	2.25000000	-0.06979472
94	0.25000000	2.00000000	-0.15894428
95	0.25000000	2.25000000	-0.12258065
96	1.75000000	0.00000000	0.16089932
97	2.25000000	0.00000000	0.16441838
98	2.00000000	0.25000000	0.11710655
99	2.25000000	0.25000000	0.08465298
100	1.75000000	1.75000000	-0.09364614
101	2.25000000	2.25000000	-0.02482893
102	1.75000000	2.00000000	-0.07996090
103	2.00000000	1.75000000	-0.05180841
104	2.00000000	2.25000000	0.00918866
105	2.25000000	2.00000000	-0.07409580
106	1.75000000	4.00000000	-0.00566960
107	2.25000000	4.00000000	-0.05376344
108	1.75000000	3.75000000	0.13196481
109	2.00000000	3.75000000	0.13822092
110	4.00000000	1.75000000	-0.08074291
111	4.00000000	2.25000000	-0.08856305
112	3.75000000	1.75000000	-0.14525904
113	3.75000000	2.00000000	-0.14173998
114	0.00000000	0.75000000	-0.05454545
115	0.00000000	1.25000000	-0.11671554

116	0.25000000	1.00000000	-0.13000978
117	0.25000000	1.25000000	-0.12883675
118	0.75000000	0.00000000	0.00488759
119	1.25000000	0.00000000	0.04750733
120	1.00000000	0.25000000	-0.09521017
121	1.25000000	0.25000000	-0.06314761
122	0.75000000	0.75000000	-0.16832845
123	1.25000000	1.25000000	-0.17106549
124	0.75000000	1.00000000	-0.19765396
125	1.00000000	0.75000000	-0.16715543
126	1.00000000	1.25000000	-0.19843597
127	1.25000000	1.00000000	-0.16793744
128	0.00000000	3.25000000	0.12414467
129	0.00000000	2.75000000	0.03812317
130	0.25000000	3.25000000	0.12492669
131	0.25000000	3.00000000	0.06275660
132	0.75000000	4.00000000	0.19804497
133	1.25000000	4.00000000	0.04985337
134	0.75000000	3.75000000	0.19061584
135	1.00000000	3.75000000	0.13704790
136	3.25000000	0.00000000	0.00997067
137	2.75000000	0.00000000	0.06432063
138	3.25000000	0.25000000	-0.06783969
139	3.00000000	0.25000000	-0.09677419
140	4.00000000	0.75000000	-0.04750733
141	4.00000000	1.25000000	-0.07370479
142	3.75000000	0.75000000	-0.10615836
143	3.75000000	1.00000000	-0.13548387
144	3.25000000	3.25000000	-0.02639296
145	2.75000000	2.75000000	-0.08973607
146	3.00000000	3.25000000	-0.02678397
147	3.25000000	3.00000000	-0.07018573
148	2.75000000	3.00000000	-0.03773216
149	3.00000000	2.75000000	-0.13665689
150	3.25000000	4.00000000	0.19843597
151	2.75000000	4.00000000	0.00058651
152	3.00000000	3.75000000	0.15933529
153	2.75000000	3.75000000	0.19882698
154	4.00000000	3.25000000	0.09208211
155	4.00000000	2.75000000	-0.00879765
156	3.75000000	3.00000000	0.03616813
157	3.75000000	2.75000000	-0.02756598
158	0.75000000	2.00000000	-0.18279570
159	1.25000000	2.00000000	-0.15542522
160	0.75000000	1.75000000	-0.19413490
161	1.00000000	1.75000000	-0.19648094
162	1.00000000	2.25000000	-0.18514174
163	1.25000000	2.25000000	-0.13509286
164	0.75000000	2.75000000	-0.07370479
165	1.25000000	3.25000000	0.06236559
166	0.75000000	3.00000000	0.00293255
167	1.00000000	3.25000000	0.01192571
168	1.00000000	2.75000000	-0.11241447
169	1.25000000	3.00000000	0.06041056
170	2.00000000	0.75000000	-0.00371457
171	2.00000000	1.25000000	-0.07096774
172	1.75000000	0.75000000	-0.07018573
173	1.75000000	1.00000000	-0.10303030
174	2.25000000	1.00000000	-0.08934506

175	2.25000000	1.25000000	0.10381232
176	2.75000000	0.75000000	-0.09951124
177	3.25000000	1.25000000	-0.18475073
178	3.00000000	0.75000000	-0.16715543
179	3.25000000	1.00000000	-0.18044966
180	2.75000000	1.00000000	-0.15268817
181	3.00000000	1.25000000	-0.19100684
182	2.00000000	2.75000000	0.11827957
183	2.00000000	3.25000000	0.18044966
184	2.25000000	3.00000000	0.13665689
185	2.25000000	3.25000000	0.18670577
186	1.75000000	2.75000000	0.04946237
187	1.75000000	3.00000000	0.16637341
188	2.75000000	2.00000000	-0.19960899
189	3.25000000	2.00000000	-0.18123167
190	3.00000000	2.25000000	-0.14995112
191	3.25000000	2.25000000	-0.14643206
192	2.75000000	1.75000000	-0.16129032
193	3.00000000	1.75000000	-0.19296188
194	0.25000000	0.50000000	-0.06549365
195	0.25000000	0.75000000	-0.11906158
196	0.50000000	0.25000000	-0.03499511
197	0.75000000	0.25000000	-0.03421310
198	0.50000000	0.75000000	-0.16598240
199	0.75000000	0.50000000	-0.13548387
200	0.25000000	3.75000000	0.13743891
201	0.25000000	3.50000000	0.17966764
202	0.50000000	3.75000000	0.18905181
203	3.75000000	0.25000000	0.03108504
204	3.50000000	0.25000000	-0.02795699
205	3.75000000	0.50000000	-0.04672532
206	3.50000000	3.75000000	0.16050831
207	3.75000000	3.50000000	0.13431085
208	3.25000000	3.50000000	0.12179863
209	3.50000000	3.25000000	0.07839687
210	3.25000000	3.75000000	0.18514174
211	3.75000000	3.25000000	0.09051808
212	0.25000000	1.75000000	-0.16950147
213	0.25000000	1.50000000	-0.18084066
214	0.25000000	2.50000000	-0.09481916
215	0.25000000	2.75000000	0.03343109
216	0.50000000	2.25000000	-0.13391984
217	0.50000000	1.75000000	-0.18162268
218	0.75000000	2.25000000	-0.17223851
219	0.50000000	2.75000000	-0.02091887
220	0.75000000	2.50000000	-0.13587488
221	1.75000000	0.25000000	0.10889541
222	1.50000000	0.25000000	0.04046921
223	2.50000000	0.25000000	0.07839687
224	2.75000000	0.25000000	0.00997067
225	2.25000000	0.50000000	0.03147605
226	1.75000000	0.50000000	-0.00293255
227	2.25000000	0.75000000	-0.04750733
228	2.75000000	0.50000000	-0.06940371
229	2.50000000	0.75000000	-0.05454545
230	1.50000000	1.75000000	-0.12883675
231	1.75000000	1.50000000	-0.10068426
232	1.25000000	1.50000000	-0.16989247
233	1.50000000	1.25000000	0.06119257

234	2.25000000	2.50000000	0.02873900
235	2.50000000	2.25000000	-0.05259042
236	2.50000000	2.75000000	0.04750733
237	2.75000000	2.50000000	-0.05141740
238	1.75000000	2.25000000	-0.02639296
239	1.25000000	1.75000000	-0.16871945
240	1.50000000	2.25000000	-0.09481916
241	2.25000000	1.75000000	-0.09364614
242	1.75000000	1.25000000	-0.09716520
243	2.25000000	1.50000000	-0.09051808
244	2.25000000	2.75000000	0.08113392
245	1.75000000	2.50000000	-0.00566960
246	2.75000000	2.25000000	-0.19921799
247	2.50000000	1.75000000	-0.10654936
248	1.50000000	3.75000000	0.03734115
249	1.25000000	3.75000000	-0.01270772
250	2.25000000	3.75000000	-0.00488759
251	2.50000000	3.75000000	0.00175953
252	1.75000000	3.50000000	0.18123167
253	1.25000000	3.50000000	0.12492669
254	1.50000000	3.25000000	0.16676442
255	2.25000000	3.50000000	0.19374389
256	1.75000000	3.25000000	0.17302053
257	3.75000000	1.50000000	-0.10694037
258	3.75000000	1.25000000	-0.10224829
259	3.75000000	2.25000000	-0.10889541
260	3.75000000	2.50000000	-0.07448680
261	3.50000000	1.75000000	-0.16793744
262	3.50000000	1.25000000	-0.17380254
263	3.25000000	1.50000000	-0.17966764
264	3.50000000	2.25000000	-0.12609971
265	3.25000000	1.75000000	-0.17380254
266	0.50000000	1.25000000	-0.19413490
267	0.75000000	1.25000000	-0.19608993
268	0.75000000	1.50000000	-0.19530792
269	1.25000000	0.50000000	-0.10224829
270	1.25000000	0.75000000	-0.13509286
271	1.50000000	0.75000000	-0.10263930
272	0.50000000	3.25000000	0.11867058
273	0.75000000	3.50000000	0.12805474
274	0.75000000	3.25000000	0.06510264
275	3.25000000	0.50000000	-0.12179863
276	3.50000000	0.75000000	-0.13509286
277	3.25000000	0.75000000	-0.15151515
278	2.75000000	3.25000000	0.11045943
279	2.75000000	3.50000000	0.16050831
280	3.25000000	2.75000000	-0.03186706
281	3.50000000	2.75000000	-0.02913001
282	2.50000000	3.25000000	0.14956012
283	3.25000000	2.50000000	-0.09560117
284	1.25000000	2.50000000	-0.12453568
285	1.25000000	2.75000000	-0.00801564
286	1.50000000	2.75000000	0.04203324
287	2.50000000	1.25000000	-0.11554252
288	2.75000000	1.25000000	-0.14682307
289	2.75000000	1.50000000	-0.15386119
290	0.00000000	0.12500000	0.09638319
291	0.12500000	0.00000000	0.11827957
292	0.12500000	0.12500000	0.06744868

293	0.00000000	3.87500000	0.18944282
294	0.12500000	4.00000000	0.19413490
295	3.87500000	0.00000000	0.14721408
296	4.00000000	0.12500000	0.13782991
297	3.87500000	3.87500000	0.18084066
298	3.87500000	4.00000000	0.20000000
299	4.00000000	3.87500000	0.18670577
300	0.00000000	1.87500000	-0.15268817
301	0.00000000	2.12500000	-0.10850440
302	0.12500000	2.00000000	-0.15307918
303	0.12500000	2.12500000	-0.13509286
304	1.87500000	0.00000000	0.16598240
305	2.12500000	0.00000000	0.16832845
306	2.00000000	0.12500000	0.14369501
307	2.12500000	0.12500000	0.12766373
308	1.87500000	1.87500000	-0.06901271
309	2.12500000	2.12500000	-0.03460411
310	1.87500000	2.00000000	-0.06236559
311	2.00000000	1.87500000	-0.04828935
312	2.00000000	2.12500000	-0.01779081
313	2.12500000	2.00000000	-0.05962854
314	1.87500000	4.00000000	0.04164223
315	2.12500000	4.00000000	0.01779081
316	1.87500000	3.87500000	0.11045943
317	2.00000000	3.87500000	0.11358749
318	4.00000000	1.87500000	-0.10107527
319	4.00000000	2.12500000	-0.10498534
320	3.87500000	1.87500000	-0.13313783
321	3.87500000	2.00000000	-0.13157380
322	0.00000000	0.87500000	-0.05962854
323	0.00000000	1.12500000	-0.09051808
324	0.12500000	1.00000000	-0.09716520
325	0.12500000	1.12500000	-0.09677419
326	0.87500000	0.00000000	-0.02561095
327	1.12500000	0.00000000	-0.00449658
328	1.00000000	0.12500000	-0.07565982
329	1.12500000	0.12500000	-0.05962854
330	0.87500000	0.87500000	-0.18396872
331	1.12500000	1.12500000	-0.18553275
332	0.87500000	1.00000000	-0.19882698
333	1.00000000	0.87500000	-0.18357771
334	1.00000000	1.12500000	-0.19921799
335	1.12500000	1.00000000	-0.18396872
336	0.00000000	3.12500000	0.09638319
337	0.00000000	2.87500000	0.05337243
338	0.12500000	3.12500000	0.09716520
339	0.12500000	3.00000000	0.06588465
340	0.87500000	4.00000000	0.19882698
341	1.12500000	4.00000000	0.12492669
342	0.87500000	3.87500000	0.19530792
343	1.00000000	3.87500000	0.16832845
344	3.12500000	0.00000000	-0.00997067
345	2.87500000	0.00000000	0.01739980
346	3.12500000	0.12500000	-0.04868035
347	3.00000000	0.12500000	-0.06314761
348	4.00000000	0.87500000	-0.07722385
349	4.00000000	1.12500000	-0.09012708
350	3.87500000	0.87500000	-0.10654936
351	3.87500000	1.00000000	-0.12101662

352	3.12500000	3.12500000	-0.10068426
353	2.87500000	2.87500000	-0.13235582
354	3.00000000	3.12500000	-0.10068426
355	3.12500000	3.00000000	-0.12258065
356	2.87500000	3.00000000	-0.10615836
357	3.00000000	2.87500000	-0.15581623
358	3.12500000	4.00000000	0.19804497
359	2.87500000	4.00000000	0.09912023
360	3.00000000	3.87500000	0.17849462
361	2.87500000	3.87500000	0.19843597
362	4.00000000	3.12500000	0.09012708
363	4.00000000	2.87500000	0.01309873
364	3.87500000	3.00000000	0.03538612
365	3.87500000	2.87500000	0.00527859
366	0.87500000	2.00000000	-0.18944282
367	1.12500000	2.00000000	-0.17536657
368	0.87500000	1.87500000	-0.19491691
369	1.00000000	1.87500000	-0.19608993
370	1.00000000	2.12500000	-0.19022483
371	1.12500000	2.12500000	-0.16520039
372	0.87500000	2.87500000	-0.06197458
373	1.12500000	3.12500000	0.00606061
374	0.87500000	3.00000000	-0.02365591
375	1.00000000	3.12500000	-0.01935484
376	1.00000000	2.87500000	-0.08152493
377	1.12500000	3.00000000	0.00488759
378	2.00000000	0.87500000	-0.03734115
379	2.00000000	1.12500000	-0.07096774
380	1.87500000	0.87500000	-0.07018573
381	1.87500000	1.00000000	-0.08660802
382	2.12500000	1.00000000	-0.07996090
383	2.12500000	1.12500000	0.01661779
384	2.87500000	0.87500000	-0.14799609
385	3.12500000	1.12500000	-0.19061584
386	3.00000000	0.87500000	-0.18162268
387	3.12500000	1.00000000	-0.18826979
388	2.87500000	1.00000000	-0.17458456
389	3.00000000	1.12500000	-0.19374389
390	2.00000000	2.87500000	0.14291300
391	2.00000000	3.12500000	0.17419355
392	2.12500000	3.00000000	0.15229717
393	2.12500000	3.12500000	0.17693060
394	1.87500000	2.87500000	0.10850440
395	1.87500000	3.00000000	0.16715543
396	2.87500000	2.00000000	-0.20000000
397	3.12500000	2.00000000	-0.19061584
398	3.00000000	2.12500000	-0.17497556
399	3.12500000	2.12500000	-0.17302053
400	2.87500000	1.87500000	-0.18084066
401	3.00000000	1.87500000	-0.19648094
402	0.00000000	0.37500000	0.00058651
403	0.00000000	0.62500000	-0.04985337
404	0.12500000	0.50000000	-0.05571848
405	0.12500000	0.62500000	-0.08230694
406	0.37500000	0.00000000	0.07800587
407	0.62500000	0.00000000	0.03577713
408	0.50000000	0.12500000	0.01583578
409	0.62500000	0.12500000	0.01583578
410	0.37500000	0.37500000	-0.07174976

411	0.62500000	0.62500000	-0.14682307
412	0.37500000	0.50000000	-0.09872923
413	0.50000000	0.37500000	-0.08347996
414	0.50000000	0.62500000	-0.14877810
415	0.62500000	0.50000000	-0.13431085
416	0.00000000	3.62500000	0.18162268
417	0.00000000	3.37500000	0.15073314
418	0.12500000	3.62500000	0.18240469
419	0.12500000	3.50000000	0.17849462
420	0.37500000	4.00000000	0.19569892
421	0.62500000	4.00000000	0.19726295
422	0.37500000	3.87500000	0.19178886
423	0.50000000	3.87500000	0.19296188
424	3.62500000	0.00000000	0.08191593
425	3.37500000	0.00000000	0.02991202
426	3.62500000	0.12500000	0.04046921
427	3.50000000	0.12500000	0.00449658
428	4.00000000	0.37500000	0.05884653
429	4.00000000	0.62500000	-0.01309873
430	3.87500000	0.37500000	0.02952102
431	3.87500000	0.50000000	-0.01231672
432	3.62500000	3.62500000	0.16793744
433	3.37500000	3.37500000	0.07409580
434	3.50000000	3.62500000	0.16832845
435	3.62500000	3.50000000	0.15464321
436	3.37500000	3.50000000	0.14799609
437	3.50000000	3.37500000	0.12649071
438	3.62500000	4.00000000	0.19921799
439	3.37500000	4.00000000	0.19882698
440	3.50000000	3.87500000	0.17966764
441	3.37500000	3.87500000	0.19217986
442	4.00000000	3.62500000	0.15933529
443	4.00000000	3.37500000	0.14369501
444	3.87500000	3.50000000	0.13978495
445	3.87500000	3.37500000	0.11945259
446	0.00000000	1.62500000	-0.16246334
447	0.00000000	1.37500000	-0.14173998
448	0.12500000	1.62500000	-0.16832845
449	0.12500000	1.50000000	-0.07370479
450	0.00000000	2.37500000	-0.02952102
451	0.00000000	2.62500000	0.07448680
452	0.12500000	2.50000000	-0.01700880
453	0.12500000	2.62500000	0.02130987
454	0.37500000	2.00000000	-0.16441838
455	0.62500000	2.00000000	-0.17653959
456	0.37500000	1.87500000	-0.11984360
457	0.50000000	2.12500000	-0.16441838
458	0.50000000	1.87500000	-0.12570870
459	0.62500000	2.12500000	-0.17106549
460	0.37500000	2.37500000	-0.10694037
461	0.62500000	2.62500000	-0.08230694
462	0.37500000	2.50000000	-0.06783969
463	0.50000000	2.37500000	-0.12492669
464	0.50000000	2.62500000	-0.05610948
465	0.62500000	2.50000000	-0.11358749
466	1.62500000	0.00000000	0.15620723
467	1.37500000	0.00000000	0.09951124
468	1.62500000	0.12500000	0.13000978
469	1.50000000	0.12500000	0.09599218

470	2.37500000	0.00000000	0.16129032
471	2.62500000	0.00000000	0.11124145
472	2.50000000	0.12500000	0.11827957
473	2.62500000	0.12500000	0.08387097
474	2.00000000	0.37500000	-0.13470186
475	2.00000000	0.62500000	0.00488759
476	1.87500000	0.37500000	0.06119257
477	2.12500000	0.50000000	0.02248289
478	1.87500000	0.50000000	0.00527859
479	2.12500000	0.62500000	-0.06705767
480	2.37500000	0.37500000	0.04203324
481	2.62500000	0.62500000	-0.05024438
482	2.50000000	0.37500000	0.03890518
483	2.37500000	0.50000000	0.01544477
484	2.62500000	0.50000000	-0.03538612
485	2.50000000	0.62500000	-0.02756598
486	1.62500000	1.62500000	0.08308895
487	1.37500000	1.37500000	-0.15581623
488	1.50000000	1.62500000	-0.13470186
489	1.62500000	1.50000000	-0.12062561
490	1.37500000	1.50000000	-0.15542522
491	1.50000000	1.37500000	-0.04007820
492	2.37500000	2.37500000	-0.01466276
493	2.62500000	2.62500000	-0.04711632
494	2.37500000	2.50000000	0.01192571
495	2.50000000	2.37500000	-0.02873900
496	2.50000000	2.62500000	0.02130987
497	2.62500000	2.50000000	-0.02795699
498	1.62500000	2.00000000	-0.04672532
499	1.37500000	2.00000000	-0.08426197
500	1.50000000	1.87500000	-0.07096774
501	1.62500000	2.12500000	-0.01974585
502	1.37500000	1.87500000	-0.09090909
503	1.50000000	2.12500000	-0.05415445
504	2.00000000	1.62500000	-0.05532747
505	2.00000000	1.37500000	-0.06471163
506	1.87500000	1.50000000	-0.07956989
507	2.12500000	1.62500000	-0.07605083
508	1.87500000	1.37500000	-0.07800587
509	2.12500000	1.50000000	-0.07448680
510	2.00000000	2.37500000	0.03577713
511	2.00000000	2.62500000	0.09051808
512	2.12500000	2.50000000	0.02091887
513	1.87500000	2.37500000	0.01818182
514	2.12500000	2.62500000	0.07214076
515	1.87500000	2.50000000	0.02834800
516	2.37500000	2.00000000	-0.03695015
517	2.62500000	2.00000000	-0.14995112
518	2.50000000	2.12500000	-0.07644184
519	2.37500000	1.87500000	-0.09677419
520	2.62500000	2.12500000	-0.14643206
521	2.50000000	1.87500000	-0.00332356
522	1.62500000	4.00000000	-0.05298143
523	1.37500000	4.00000000	-0.02521994
524	1.50000000	3.87500000	-0.03147605
525	1.37500000	3.87500000	-0.05650049
526	2.37500000	4.00000000	-0.12688172
527	2.62500000	4.00000000	-0.08543500
528	2.37500000	3.87500000	-0.10068426

529	2.50000000	3.87500000	-0.09755621
530	1.62500000	3.62500000	-0.04672532
531	1.37500000	3.37500000	0.11867058
532	1.50000000	3.62500000	0.10615836
533	1.62500000	3.50000000	0.17810362
534	1.37500000	3.50000000	0.14995112
535	1.50000000	3.37500000	0.17419355
536	2.00000000	3.62500000	0.16285435
537	2.00000000	3.37500000	0.18396872
538	2.12500000	3.62500000	0.09130010
539	1.87500000	3.50000000	0.18435973
540	2.12500000	3.50000000	0.19061584
541	1.87500000	3.37500000	0.18005865
542	4.00000000	1.62500000	-0.06041056
543	4.00000000	1.37500000	-0.05689150
544	3.87500000	1.50000000	-0.07370479
545	3.87500000	1.37500000	-0.07135875
546	4.00000000	2.37500000	-0.07214076
547	4.00000000	2.62500000	-0.03225806
548	3.87500000	2.37500000	-0.08230694
549	3.87500000	2.50000000	-0.06510264
550	3.62500000	1.62500000	-0.15933529
551	3.37500000	1.37500000	-0.17888563
552	3.62500000	1.50000000	-0.14017595
553	3.50000000	1.62500000	-0.17067449
554	3.50000000	1.37500000	-0.17380254
555	3.37500000	1.50000000	-0.17653959
556	3.62500000	2.00000000	-0.15190616
557	3.37500000	2.00000000	-0.17497556
558	3.62500000	2.12500000	-0.13548387
559	3.50000000	1.87500000	-0.16520039
560	3.50000000	2.12500000	-0.13861193
561	3.37500000	1.87500000	-0.16832845
562	0.37500000	1.00000000	-0.16246334
563	0.62500000	1.00000000	-0.19648094
564	0.37500000	0.87500000	-0.10694037
565	0.50000000	0.87500000	-0.18084066
566	0.50000000	1.12500000	-0.19452590
567	0.62500000	1.12500000	-0.19569892
568	0.37500000	1.37500000	-0.16089932
569	0.62500000	1.62500000	-0.16832845
570	0.37500000	1.50000000	-0.18670577
571	0.50000000	1.62500000	-0.18748778
572	0.50000000	1.37500000	-0.19335288
573	0.62500000	1.50000000	-0.19413490
574	1.00000000	0.37500000	-0.11476051
575	1.00000000	0.62500000	-0.15073314
576	0.87500000	0.37500000	-0.08426197
577	0.87500000	0.50000000	-0.13509286
578	1.12500000	0.50000000	-0.11827957
579	1.12500000	0.62500000	-0.13470186
580	1.37500000	0.37500000	0.03382209
581	1.62500000	0.62500000	0.03030303
582	1.50000000	0.37500000	0.08543500
583	1.62500000	0.50000000	0.06392962
584	1.37500000	0.50000000	0.03929619
585	1.50000000	0.62500000	0.01388074
586	1.00000000	1.37500000	-0.19178886
587	1.00000000	1.62500000	-0.19687195

588	1.12500000	1.50000000	-0.18357771
589	1.12500000	1.62500000	-0.18279570
590	0.87500000	1.37500000	-0.19648094
591	0.87500000	1.50000000	-0.19608993
592	1.37500000	1.00000000	-0.15190616
593	1.62500000	1.00000000	-0.11945259
594	1.50000000	1.12500000	-0.03734115
595	1.62500000	1.12500000	-0.11397849
596	1.37500000	0.87500000	-0.13548387
597	1.50000000	0.87500000	-0.11280547
598	0.37500000	3.37500000	0.15268817
599	0.62500000	3.62500000	0.18592375
600	0.37500000	3.50000000	0.18044966
601	0.50000000	3.62500000	0.18514174
602	0.50000000	3.37500000	0.14995112
603	0.62500000	3.50000000	0.15464321
604	0.37500000	3.00000000	0.05923754
605	0.62500000	3.00000000	0.02952102
606	0.37500000	2.87500000	0.04398827
607	0.50000000	2.87500000	0.01779081
608	0.50000000	3.12500000	0.08739003
609	0.62500000	3.12500000	0.06080156
610	1.00000000	3.62500000	0.10576735
611	1.00000000	3.37500000	0.04320626
612	1.12500000	3.62500000	0.03069404
613	1.12500000	3.50000000	0.09951124
614	0.87500000	3.50000000	0.10146628
615	0.87500000	3.37500000	0.06979472
616	3.37500000	0.37500000	0.11358749
617	3.62500000	0.62500000	-0.10576735
618	3.50000000	0.37500000	-0.05415445
619	3.62500000	0.50000000	-0.07526882
620	3.37500000	0.50000000	-0.11397849
621	3.50000000	0.62500000	-0.12023460
622	3.00000000	0.37500000	-0.11749756
623	3.00000000	0.62500000	-0.15268817
624	2.87500000	0.37500000	-0.06392962
625	2.87500000	0.50000000	-0.10381232
626	3.12500000	0.50000000	-0.13000978
627	3.12500000	0.62500000	-0.14486804
628	3.62500000	1.00000000	-0.14995112
629	3.37500000	1.00000000	-0.17223851
630	3.62500000	1.12500000	-0.13313783
631	3.50000000	1.12500000	-0.16911046
632	3.50000000	0.87500000	-0.14995112
633	3.37500000	0.87500000	-0.18279570
634	3.00000000	3.37500000	0.04711632
635	3.00000000	3.62500000	0.14017595
636	3.12500000	3.50000000	0.12140762
637	3.12500000	3.62500000	0.17810362
638	2.87500000	3.37500000	0.11593353
639	2.87500000	3.50000000	0.14095797
640	3.37500000	3.00000000	-0.01779081
641	3.62500000	3.00000000	0.03538612
642	3.50000000	3.12500000	0.05650049
643	3.62500000	3.12500000	-0.13587488
644	3.37500000	2.87500000	0.00136852
645	3.50000000	2.87500000	0.00293255
646	2.62500000	3.00000000	0.03069404

647	2.37500000	3.00000000	0.11827957
648	2.50000000	2.87500000	0.07370479
649	2.37500000	2.87500000	0.09051808
650	2.62500000	3.12500000	0.10498534
651	2.50000000	3.12500000	0.12453568
652	3.00000000	2.62500000	-0.11749756
653	3.00000000	2.37500000	-0.12414467
654	2.87500000	2.50000000	-0.07487781
655	2.87500000	2.37500000	-0.14877810
656	3.12500000	2.62500000	-0.09012708
657	3.12500000	2.50000000	-0.09521017
658	2.62500000	3.62500000	0.19960899
659	2.37500000	3.37500000	0.19335288
660	2.50000000	3.62500000	0.10107527
661	2.37500000	3.50000000	0.19687195
662	2.62500000	3.50000000	0.18005865
663	2.50000000	3.37500000	0.17497556
664	3.62500000	2.62500000	-0.06041056
665	3.37500000	2.37500000	-0.12297165
666	3.62500000	2.50000000	-0.08387097
667	3.50000000	2.37500000	-0.11045943
668	3.50000000	2.62500000	-0.06119257
669	3.37500000	2.50000000	-0.09442815
670	1.00000000	2.37500000	-0.17966764
671	1.00000000	2.62500000	-0.14330401
672	0.87500000	2.37500000	-0.17341153
673	0.87500000	2.50000000	-0.15503421
674	1.12500000	2.50000000	-0.14956012
675	1.12500000	2.62500000	-0.09130010
676	1.37500000	2.37500000	-0.10459433
677	1.62500000	2.62500000	-0.01231672
678	1.50000000	2.37500000	-0.08465298
679	1.62500000	2.50000000	-0.04007820
680	1.37500000	2.50000000	-0.09951124
681	1.50000000	2.62500000	-0.01622678
682	1.37500000	3.00000000	0.10967742
683	1.62500000	3.00000000	0.16246334
684	1.50000000	3.12500000	0.16285435
685	1.62500000	3.12500000	0.16598240
686	1.37500000	2.87500000	0.07526882
687	1.50000000	2.87500000	0.10029326
688	2.37500000	1.00000000	-0.09872923
689	2.62500000	1.00000000	-0.13079179
690	2.37500000	0.87500000	-0.07800587
691	2.50000000	0.87500000	-0.08152493
692	2.50000000	1.12500000	-0.11202346
693	2.62500000	1.12500000	-0.12766373
694	2.37500000	1.37500000	-0.00957967
695	2.62500000	1.62500000	-0.14213099
696	2.37500000	1.50000000	-0.10654936
697	2.50000000	1.62500000	-0.11476051
698	2.50000000	1.37500000	-0.11906158
699	2.62500000	1.50000000	-0.13822092
700	3.00000000	1.37500000	-0.18826979
701	3.00000000	1.62500000	-0.18944282
702	3.12500000	1.50000000	-0.18279570
703	3.12500000	1.62500000	-0.17966764
704	2.87500000	1.37500000	-0.16637341
705	2.87500000	1.50000000	-0.16950147

706	0.12500000	0.25000000	0.01739980
707	0.12500000	0.37500000	-0.00957967
708	0.25000000	0.12500000	0.03929619
709	0.37500000	0.12500000	0.02756598
710	0.25000000	0.37500000	-0.03851417
711	0.37500000	0.25000000	-0.02326491
712	0.12500000	3.87500000	0.19022483
713	0.12500000	3.75000000	0.18670577
714	0.25000000	3.87500000	0.19139785
715	3.87500000	0.12500000	0.10537634
716	3.75000000	0.12500000	0.07292278
717	3.87500000	0.25000000	0.06353861
718	3.75000000	3.87500000	0.18044966
719	3.87500000	3.75000000	0.16754643
720	3.62500000	3.75000000	0.16089932
721	3.75000000	3.62500000	0.14799609
722	3.62500000	3.87500000	0.18005865
723	3.87500000	3.62500000	0.15386119
724	0.12500000	1.87500000	-0.15855327
725	0.12500000	1.75000000	-0.11358749
726	0.12500000	2.25000000	-0.09599218
727	0.12500000	2.37500000	-0.05102639
728	0.25000000	2.12500000	-0.14056696
729	0.25000000	1.87500000	-0.16402737
730	0.37500000	2.12500000	-0.13391984
731	0.25000000	2.37500000	-0.08347996
732	0.37500000	2.25000000	-0.11671554
733	1.87500000	0.12500000	0.13900293
734	1.75000000	0.12500000	0.13470186
735	2.25000000	0.12500000	0.12453568
736	2.37500000	0.12500000	0.12140762
737	2.12500000	0.25000000	0.10107527
738	1.87500000	0.25000000	0.11319648
739	2.12500000	0.37500000	0.07448680
740	2.37500000	0.25000000	0.08152493
741	2.25000000	0.37500000	0.05806452
742	1.75000000	1.87500000	-0.08660802
743	1.87500000	1.75000000	-0.07292278
744	1.62500000	1.75000000	-0.11124145
745	1.75000000	1.62500000	-0.09716520
746	2.12500000	2.25000000	-0.00762463
747	2.25000000	2.12500000	-0.04946237
748	2.25000000	2.37500000	0.00215054
749	2.37500000	2.25000000	-0.03851417
750	1.87500000	2.12500000	-0.03538612
751	1.62500000	1.87500000	-0.10420332
752	1.75000000	2.12500000	-0.05298143
753	2.12500000	1.87500000	-0.06275660
754	1.87500000	1.62500000	-0.07644184
755	2.12500000	1.75000000	-0.07292278
756	2.12500000	2.37500000	0.01896383
757	1.87500000	2.25000000	-0.00840665
758	2.37500000	2.12500000	-0.06314761
759	2.25000000	1.87500000	-0.08387097
760	1.75000000	3.87500000	0.06314761
761	1.62500000	3.87500000	0.01583578
762	2.12500000	3.87500000	0.04242424
763	2.25000000	3.87500000	-0.02913001
764	1.87500000	3.75000000	0.13509286

765	1.62500000	3.75000000	0.08465298
766	1.75000000	3.62500000	0.15659824
767	2.12500000	3.75000000	0.06666667
768	1.87500000	3.62500000	0.15972630
769	3.87500000	1.75000000	-0.11280547
770	3.87500000	1.62500000	-0.09364614
771	3.87500000	2.12500000	-0.11515152
772	3.87500000	2.25000000	-0.09872923
773	3.75000000	1.87500000	-0.14330401
774	3.75000000	1.62500000	-0.12609971
775	3.62500000	1.75000000	-0.15659824
776	3.75000000	2.12500000	-0.12531769
777	3.62500000	1.87500000	-0.15464321
778	0.12500000	0.87500000	-0.09208211
779	0.12500000	0.75000000	-0.08660802
780	0.12500000	1.25000000	-0.12258065
781	0.12500000	1.37500000	-0.14877810
782	0.25000000	1.12500000	-0.12922776
783	0.25000000	0.87500000	-0.12453568
784	0.37500000	1.12500000	-0.16207234
785	0.25000000	1.37500000	-0.15464321
786	0.37500000	1.25000000	-0.16129032
787	0.87500000	0.12500000	-0.04516129
788	0.75000000	0.12500000	-0.01466276
789	1.25000000	0.12500000	-0.00762463
790	1.37500000	0.12500000	0.04398827
791	1.12500000	0.25000000	-0.07917889
792	0.87500000	0.25000000	-0.06471163
793	1.12500000	0.37500000	-0.09872923
794	1.37500000	0.25000000	-0.01153470
795	1.25000000	0.37500000	-0.08269795
796	0.75000000	0.87500000	-0.18279570
797	0.87500000	0.75000000	-0.16754643
798	0.62500000	0.75000000	-0.16715543
799	0.75000000	0.62500000	-0.15190616
800	1.12500000	1.25000000	-0.18475073
801	1.25000000	1.12500000	-0.16950147
802	1.25000000	1.37500000	-0.17028348
803	1.37500000	1.25000000	-0.07996090
804	0.87500000	1.12500000	-0.19804497
805	0.62500000	0.87500000	-0.18162268
806	0.75000000	1.12500000	-0.19687195
807	1.12500000	0.87500000	-0.16754643
808	0.87500000	0.62500000	-0.15151515
809	1.12500000	0.75000000	-0.15112414
810	1.12500000	1.37500000	-0.18396872
811	0.87500000	1.25000000	-0.19726295
812	1.37500000	1.12500000	-0.05337243
813	1.25000000	0.87500000	-0.15151515
814	0.12500000	3.25000000	0.12453568
815	0.12500000	3.37500000	0.15190616
816	0.12500000	2.87500000	0.05063539
817	0.12500000	2.75000000	0.03499511
818	0.25000000	3.12500000	0.09364614
819	0.25000000	3.37500000	0.15229717
820	0.37500000	3.25000000	0.12179863
821	0.25000000	2.87500000	0.04828935
822	0.37500000	3.12500000	0.09051808
823	0.75000000	3.87500000	0.19452590

824	0.62500000	3.87500000	0.19374389
825	1.12500000	3.87500000	0.09364614
826	1.25000000	3.87500000	0.01857283
827	0.87500000	3.75000000	0.16402737
828	0.62500000	3.75000000	0.18983382
829	0.75000000	3.62500000	0.15933529
830	1.12500000	3.75000000	0.06236559
831	0.87500000	3.62500000	0.13274682
832	3.25000000	0.12500000	-0.02873900
833	3.37500000	0.12500000	-0.00918866
834	2.87500000	0.12500000	-0.01622678
835	2.75000000	0.12500000	0.03734115
836	3.12500000	0.25000000	-0.08230694
837	3.37500000	0.25000000	-0.04789834
838	3.25000000	0.37500000	-0.09481916
839	2.87500000	0.25000000	-0.04359726
840	3.12500000	0.37500000	-0.10928641
841	3.87500000	0.75000000	-0.07683284
842	3.87500000	0.62500000	-0.04711632
843	3.87500000	1.12500000	-0.10459433
844	3.87500000	1.25000000	-0.08778104
845	3.75000000	0.87500000	-0.12062561
846	3.75000000	0.62500000	-0.07644184
847	3.62500000	0.75000000	-0.12062561
848	3.75000000	1.12500000	-0.11867058
849	3.62500000	0.87500000	-0.13548387
850	3.12500000	3.25000000	-0.02678397
851	3.25000000	3.12500000	-0.04828935
852	3.25000000	3.37500000	0.04750733
853	3.37500000	3.25000000	0.02600196
854	2.75000000	2.87500000	-0.06353861
855	2.87500000	2.75000000	-0.11319648
856	2.62500000	2.75000000	-0.02091887
857	2.75000000	2.62500000	-0.07057674
858	2.87500000	3.12500000	-0.03225806
859	3.12500000	3.37500000	0.04750733
860	2.87500000	3.25000000	0.04203324
861	3.12500000	2.87500000	-0.10342131
862	3.37500000	3.12500000	0.00410557
863	3.25000000	2.87500000	-0.05102639
864	2.62500000	2.87500000	0.00488759
865	2.75000000	3.12500000	0.03655914
866	2.87500000	2.62500000	-0.09403715
867	3.12500000	2.75000000	-0.08426197
868	3.12500000	3.87500000	0.17888563
869	3.25000000	3.87500000	0.19178886
870	2.75000000	3.87500000	0.09990225
871	2.62500000	3.87500000	0.00136852
872	2.87500000	3.75000000	0.17927664
873	3.12500000	3.75000000	0.17223851
874	2.87500000	3.62500000	0.16011730
875	2.62500000	3.75000000	0.10029326
876	2.75000000	3.62500000	0.17966764
877	3.87500000	3.12500000	0.06432063
878	3.87500000	3.25000000	0.09286413
879	3.87500000	2.75000000	-0.01818182
880	3.87500000	2.62500000	-0.04164223
881	3.75000000	2.87500000	0.00449658
882	3.75000000	3.12500000	0.06353861

883	3.62500000	2.87500000	0.00371457
884	3.75000000	2.62500000	-0.05102639
885	3.62500000	2.75000000	-0.02834800
886	0.75000000	1.87500000	-0.18826979
887	0.87500000	2.12500000	-0.18396872
888	0.62500000	1.87500000	-0.18201369
889	0.75000000	2.12500000	-0.17732160
890	1.12500000	1.87500000	-0.17575758
891	1.25000000	2.12500000	-0.14525904
892	1.25000000	1.87500000	-0.16207234
893	1.37500000	2.12500000	-0.12492669
894	0.87500000	1.75000000	-0.19530792
895	0.62500000	1.75000000	-0.18787879
896	0.75000000	1.62500000	-0.19452590
897	1.12500000	1.75000000	-0.18279570
898	0.87500000	1.62500000	-0.19608993
899	1.12500000	2.25000000	-0.16011730
900	0.87500000	2.25000000	-0.17888563
901	1.12500000	2.37500000	-0.15464321
902	1.37500000	2.25000000	-0.11476051
903	1.25000000	2.37500000	-0.12961877
904	0.75000000	2.87500000	-0.03538612
905	0.87500000	2.75000000	-0.09286413
906	0.62500000	2.75000000	-0.04711632
907	0.75000000	2.62500000	-0.10459433
908	1.12500000	3.25000000	0.03734115
909	1.25000000	3.12500000	0.06158358
910	1.25000000	3.37500000	0.09364614
911	1.37500000	3.25000000	0.11476051
912	0.87500000	3.12500000	0.00762463
913	0.62500000	2.87500000	-0.00918866
914	0.75000000	3.12500000	0.03382209
915	1.12500000	3.37500000	0.06862170
916	0.87500000	3.25000000	0.03851417
917	1.12500000	2.87500000	-0.02600196
918	0.87500000	2.62500000	-0.12414467
919	1.12500000	2.75000000	-0.06001955
920	1.37500000	3.12500000	0.11358749
921	1.25000000	2.87500000	0.02639296
922	1.87500000	0.75000000	-0.03695015
923	2.12500000	0.87500000	-0.04633431
924	1.87500000	0.62500000	-0.00332356
925	2.12500000	0.75000000	-0.02561095
926	1.87500000	1.12500000	-0.08699902
927	2.12500000	1.25000000	0.01661779
928	1.87500000	1.25000000	-0.08426197
929	2.12500000	1.37500000	-0.08074291
930	1.75000000	0.87500000	-0.08660802
931	1.75000000	0.62500000	-0.03655914
932	1.62500000	0.75000000	-0.08660802
933	1.75000000	1.12500000	-0.09990225
934	1.62500000	0.87500000	-0.10263930
935	2.25000000	1.12500000	0.00723363
936	2.25000000	0.87500000	-0.06823069
937	2.37500000	1.12500000	-0.10224829
938	2.25000000	1.37500000	0.00684262
939	2.37500000	1.25000000	-0.00566960
940	2.87500000	0.75000000	-0.13352884
941	2.75000000	0.87500000	-0.12609971

942	2.75000000	0.62500000	-0.08465298
943	2.62500000	0.75000000	-0.07683284
944	3.25000000	1.12500000	-0.18279570
945	3.12500000	1.25000000	-0.18787879
946	3.37500000	1.25000000	-0.17927664
947	3.25000000	1.37500000	-0.18201369
948	3.12500000	0.87500000	-0.17380254
949	2.87500000	0.62500000	-0.11827957
950	3.12500000	0.75000000	-0.15933529
951	3.37500000	1.12500000	-0.17693060
952	3.25000000	0.87500000	-0.16598240
953	2.87500000	1.12500000	-0.17184751
954	2.62500000	0.87500000	-0.10342131
955	2.75000000	1.12500000	-0.14995112
956	3.12500000	1.37500000	-0.18553275
957	2.87500000	1.25000000	-0.16911046
958	2.12500000	2.87500000	0.12766373
959	1.87500000	2.75000000	0.08387097
960	2.12500000	2.75000000	0.09990225
961	1.87500000	2.62500000	0.05650049
962	2.12500000	3.25000000	0.18357771
963	1.87500000	3.12500000	0.17341153
964	2.12500000	3.37500000	0.18709677
965	1.87500000	3.25000000	0.17693060
966	2.25000000	3.12500000	0.16168133
967	2.25000000	2.87500000	0.10889541
968	2.37500000	3.12500000	0.14330401
969	2.25000000	3.37500000	0.19022483
970	2.37500000	3.25000000	0.16832845
971	1.75000000	2.87500000	0.10811339
972	1.75000000	2.62500000	0.02209189
973	1.62500000	2.75000000	0.04594330
974	1.75000000	3.12500000	0.16989247
975	1.62500000	2.87500000	0.10420332
976	2.87500000	2.12500000	-0.17497556
977	2.75000000	1.87500000	-0.18044966
978	2.75000000	2.12500000	-0.19921799
979	2.62500000	1.87500000	-0.15307918
980	3.25000000	2.12500000	-0.16402737
981	3.12500000	1.87500000	-0.18709677
982	3.37500000	2.12500000	-0.15464321
983	3.25000000	1.87500000	-0.17771261
984	3.12500000	2.25000000	-0.14799609
985	2.87500000	2.25000000	-0.17458456
986	3.12500000	2.37500000	-0.12258065
987	3.37500000	2.25000000	-0.13704790
988	3.25000000	2.37500000	-0.12101662
989	2.87500000	1.75000000	-0.17693060
990	2.62500000	1.75000000	-0.13391984
991	2.75000000	1.62500000	-0.15777126
992	3.12500000	1.75000000	-0.18357771
993	2.87500000	1.62500000	-0.17341153
994	0.25000000	0.62500000	-0.09208211
995	0.37500000	0.62500000	-0.11593353
996	0.37500000	0.75000000	-0.14252199
997	0.62500000	0.25000000	-0.03460411
998	0.62500000	0.37500000	-0.08504399
999	0.75000000	0.37500000	-0.08465298
1000	0.25000000	3.62500000	0.18357771

1001	0.37500000	3.75000000	0.18826979
1002	0.37500000	3.62500000	0.18435973
1003	3.62500000	0.25000000	0.00175953
1004	3.75000000	0.37500000	-0.00762463
1005	3.62500000	0.37500000	-0.03734115
1006	3.37500000	3.62500000	0.14134897
1007	3.37500000	3.75000000	0.17302053
1008	3.62500000	3.37500000	0.10654936
1009	3.75000000	3.37500000	0.12023460
1010	3.25000000	3.62500000	0.17849462
1011	3.62500000	3.25000000	0.08543500
1012	0.25000000	1.62500000	-0.14995112
1013	0.37500000	1.75000000	-0.15034213
1014	0.37500000	1.62500000	-0.18123167
1015	0.25000000	2.62500000	-0.02052786
1016	0.37500000	2.62500000	-0.08152493
1017	0.37500000	2.75000000	0.00566960
1018	0.62500000	2.25000000	-0.10303030
1019	0.62500000	2.37500000	-0.12218964
1020	0.75000000	2.37500000	-0.15386119
1021	1.62500000	0.25000000	0.07487781
1022	1.75000000	0.37500000	0.05298143
1023	1.62500000	0.37500000	0.01896383
1024	2.62500000	0.25000000	0.04437928
1025	2.62500000	0.37500000	0.00449658
1026	2.75000000	0.37500000	-0.02952102
1027	2.25000000	0.62500000	-0.00801564
1028	2.37500000	0.62500000	-0.01153470
1029	2.37500000	0.75000000	-0.05102639
1030	1.37500000	1.62500000	-0.14916911
1031	1.37500000	1.75000000	-0.14877810
1032	1.62500000	1.37500000	-0.01974585
1033	1.75000000	1.37500000	-0.09872923
1034	1.25000000	1.62500000	-0.16911046
1035	1.62500000	1.25000000	-0.01779081
1036	2.37500000	2.62500000	0.03812317
1037	2.25000000	2.62500000	0.05493646
1038	2.62500000	2.37500000	-0.05180841
1039	2.62500000	2.25000000	-0.12570870
1040	2.37500000	2.75000000	0.06432063
1041	2.75000000	2.37500000	-0.12531769
1042	1.62500000	2.25000000	-0.06041056
1043	1.75000000	2.37500000	-0.01583578
1044	1.62500000	2.37500000	-0.05024438
1045	2.25000000	1.62500000	-0.09208211
1046	2.37500000	1.75000000	-0.10029326
1047	2.37500000	1.62500000	-0.09833822
1048	1.37500000	3.75000000	0.01231672
1049	1.37500000	3.62500000	0.08113392
1050	1.25000000	3.62500000	0.05610948
1051	2.37500000	3.75000000	-0.00136852
1052	2.25000000	3.62500000	0.09442815
1053	2.37500000	3.62500000	0.09794721
1054	1.62500000	3.37500000	0.17419355
1055	1.75000000	3.37500000	0.17732160
1056	1.62500000	3.25000000	0.16989247
1057	3.75000000	1.37500000	-0.10459433
1058	3.62500000	1.37500000	-0.14956012
1059	3.62500000	1.25000000	-0.13861193

1060	3.75000000	2.37500000	-0.19335288
1061	3.62500000	2.25000000	-0.10576735
1062	3.62500000	2.37500000	0.18670577
1063	3.37500000	1.62500000	-0.17380254
1064	3.37500000	1.75000000	-0.17106549
1065	3.25000000	1.62500000	-0.17693060
1066	0.62500000	1.25000000	-0.19530792
1067	0.62500000	1.37500000	-0.19452590
1068	0.75000000	1.37500000	-0.19569892
1069	1.25000000	0.62500000	-0.11867058
1070	1.37500000	0.62500000	-0.10224829
1071	1.37500000	0.75000000	-0.11867058
1072	0.62500000	3.37500000	0.12336266
1073	0.62500000	3.25000000	0.09169110
1074	0.75000000	3.37500000	0.09677419
1075	3.37500000	0.62500000	-0.12844575
1076	3.25000000	0.62500000	-0.13665689
1077	3.37500000	0.75000000	-0.14330401
1078	2.75000000	3.37500000	0.13548387
1079	2.62500000	3.25000000	0.13000978
1080	2.62500000	3.37500000	0.15503421
1081	3.37500000	2.75000000	-0.03030303
1082	3.25000000	2.62500000	-0.06353861
1083	3.37500000	2.62500000	-0.06236559
1084	1.25000000	2.62500000	-0.06627566
1085	1.37500000	2.62500000	-0.04125122
1086	1.37500000	2.75000000	0.01700880
1087	2.62500000	1.25000000	-0.13118280
1088	2.62500000	1.37500000	-0.13470186
1089	2.75000000	1.37500000	-0.15034213
1090	0.00000000	0.06250000	0.13176931
1091	0.06250000	0.00000000	0.14271750
1092	0.06250000	0.06250000	0.11730205
1093	0.00000000	3.93750000	0.19120235
1094	0.06250000	4.00000000	0.19354839
1095	3.93750000	0.00000000	0.16344086
1096	4.00000000	0.06250000	0.15874878
1097	3.93750000	3.93750000	0.19042033
1098	3.93750000	4.00000000	0.20000000
1099	4.00000000	3.93750000	0.19335288
1100	0.00000000	1.93750000	-0.15014663
1101	0.00000000	2.06250000	-0.12805474
1102	0.06250000	2.00000000	-0.15034213
1103	0.06250000	2.06250000	-0.14134897
1104	1.93750000	0.00000000	0.16832845
1105	2.06250000	0.00000000	0.16950147
1106	2.00000000	0.06250000	0.15718475
1107	2.06250000	0.06250000	0.14916911
1108	1.93750000	1.93750000	-0.05689150
1109	2.06250000	2.06250000	-0.03968719
1110	1.93750000	2.00000000	-0.05356794
1111	2.00000000	1.93750000	-0.04652981
1112	2.00000000	2.06250000	-0.03128055
1113	2.06250000	2.00000000	-0.05219941
1114	1.93750000	4.00000000	0.06529814
1115	2.06250000	4.00000000	0.05337243
1116	1.93750000	3.93750000	0.09970674
1117	2.00000000	3.93750000	0.10127077
1118	4.00000000	1.93750000	-0.11124145

1119	4.00000000	2.06250000	-0.11319648
1120	3.93750000	1.93750000	-0.12727273
1121	3.93750000	2.00000000	-0.12649071
1122	0.00000000	0.93750000	-0.06217009
1123	0.00000000	1.06250000	-0.07761486
1124	0.06250000	1.00000000	-0.08093842
1125	0.06250000	1.06250000	-0.08074291
1126	0.93750000	0.00000000	-0.04105572
1127	1.06250000	0.00000000	-0.03049853
1128	1.00000000	0.06250000	-0.06608016
1129	1.06250000	0.06250000	-0.05806452
1130	0.93750000	0.93750000	-0.19198436
1131	1.06250000	1.06250000	-0.19276637
1132	0.93750000	1.00000000	-0.19941349
1133	1.00000000	0.93750000	-0.19178886
1134	1.00000000	1.06250000	-0.19960899
1135	1.06250000	1.00000000	-0.19198436
1136	0.00000000	3.06250000	0.08269795
1137	0.00000000	2.93750000	0.06119257
1138	0.06250000	3.06250000	0.08308895
1139	0.06250000	3.00000000	0.06744868
1140	0.93750000	4.00000000	0.19921799
1141	1.06250000	4.00000000	0.16226784
1142	0.93750000	3.93750000	0.19745846
1143	1.00000000	3.93750000	0.18396872
1144	3.06250000	0.00000000	-0.01994135
1145	2.93750000	0.00000000	-0.00625611
1146	3.06250000	0.06250000	-0.03929619
1147	3.00000000	0.06250000	-0.04652981
1148	4.00000000	0.93750000	-0.09208211
1149	4.00000000	1.06250000	-0.09853372
1150	3.93750000	0.93750000	-0.10674487
1151	3.93750000	1.00000000	-0.11397849
1152	3.06250000	3.06250000	-0.13782991
1153	2.93750000	2.93750000	-0.15366569
1154	3.00000000	3.06250000	-0.13782991
1155	3.06250000	3.00000000	-0.14877810
1156	2.93750000	3.00000000	-0.14056696
1157	3.00000000	2.93750000	-0.16539589
1158	3.06250000	4.00000000	0.19784946
1159	2.93750000	4.00000000	0.14838710
1160	3.00000000	3.93750000	0.18807429
1161	2.93750000	3.93750000	0.19804497
1162	4.00000000	3.06250000	0.06392962
1163	4.00000000	2.93750000	0.02541544
1164	3.93750000	3.00000000	0.03655914
1165	3.93750000	2.93750000	0.02150538
1166	0.93750000	2.00000000	-0.19257087
1167	1.06250000	2.00000000	-0.18553275
1168	0.93750000	1.93750000	-0.19530792
1169	1.00000000	1.93750000	-0.19589443
1170	1.00000000	2.06250000	-0.19296188
1171	1.06250000	2.06250000	-0.18044966
1172	0.93750000	2.93750000	-0.05630499
1173	1.06250000	3.06250000	-0.02228739
1174	0.93750000	3.00000000	-0.03714565
1175	1.00000000	3.06250000	-0.03499511
1176	1.00000000	2.93750000	-0.06608016
1177	1.06250000	3.00000000	-0.02287390

1178	2.00000000	0.93750000	-0.05395894
1179	2.00000000	1.06250000	-0.07077224
1180	1.93750000	0.93750000	-0.07038123
1181	1.93750000	1.00000000	-0.07859238
1182	2.06250000	1.00000000	-0.07526882
1183	2.06250000	1.06250000	-0.02697947
1184	2.93750000	0.93750000	-0.17223851
1185	3.06250000	1.06250000	-0.19354839
1186	3.00000000	0.93750000	-0.18905181
1187	3.06250000	1.00000000	-0.19237537
1188	2.93750000	1.00000000	-0.18553275
1189	3.00000000	1.06250000	-0.19511241
1190	2.00000000	2.93750000	0.15522972
1191	2.00000000	3.06250000	0.17086999
1192	2.06250000	3.00000000	0.15992180
1193	2.06250000	3.06250000	0.17223851
1194	1.93750000	2.93750000	0.13802542
1195	1.93750000	3.00000000	0.16735093
1196	2.93750000	2.00000000	-0.20000000
1197	3.06250000	2.00000000	-0.19530792
1198	3.00000000	2.06250000	-0.18748778
1199	3.06250000	2.06250000	-0.18651026
1200	2.93750000	1.93750000	-0.19042033
1201	3.00000000	1.93750000	-0.19824047
1202	0.00000000	0.43750000	-0.02248289
1203	0.00000000	0.56250000	-0.04770283
1204	0.06250000	0.50000000	-0.05063539
1205	0.06250000	0.56250000	-0.06392962
1206	0.43750000	0.00000000	0.07214076
1207	0.56250000	0.00000000	0.05102639
1208	0.50000000	0.06250000	0.04105572
1209	0.56250000	0.06250000	0.04105572
1210	0.43750000	0.43750000	-0.10185728
1211	0.56250000	0.56250000	-0.13939394
1212	0.43750000	0.50000000	-0.11534702
1213	0.50000000	0.43750000	-0.10772239
1214	0.50000000	0.56250000	-0.14037146
1215	0.56250000	0.50000000	-0.13313783
1216	0.00000000	3.56250000	0.17947214
1217	0.00000000	3.43750000	0.16402737
1218	0.06250000	3.56250000	0.17986315
1219	0.06250000	3.50000000	0.17790811
1220	0.43750000	4.00000000	0.19608993
1221	0.56250000	4.00000000	0.19687195
1222	0.43750000	3.93750000	0.19413490
1223	0.50000000	3.93750000	0.19472141
1224	3.56250000	0.00000000	0.06568915
1225	3.43750000	0.00000000	0.03968719
1226	3.56250000	0.06250000	0.04496579
1227	3.50000000	0.06250000	0.02697947
1228	4.00000000	0.43750000	0.04027370
1229	4.00000000	0.56250000	0.00430108
1230	3.93750000	0.43750000	0.02561095
1231	3.93750000	0.50000000	0.00469208
1232	3.56250000	3.56250000	0.17126100
1233	3.43750000	3.43750000	0.12434018
1234	3.50000000	3.56250000	0.17145650
1235	3.56250000	3.50000000	0.16461388
1236	3.43750000	3.50000000	0.16129032

1237	3.50000000	3.43750000	0.15053763
1238	3.56250000	4.00000000	0.19902248
1239	3.43750000	4.00000000	0.19882698
1240	3.50000000	3.93750000	0.18924731
1241	3.43750000	3.93750000	0.19550342
1242	4.00000000	3.56250000	0.15210166
1243	4.00000000	3.43750000	0.14428152
1244	3.93750000	3.50000000	0.14232649
1245	3.93750000	3.43750000	0.13216031
1246	0.00000000	1.56250000	-0.16480938
1247	0.00000000	1.43750000	-0.15444770
1248	0.06250000	1.56250000	-0.16774194
1249	0.06250000	1.50000000	-0.12043011
1250	0.00000000	2.43750000	-0.00938416
1251	0.00000000	2.56250000	0.04261975
1252	0.06250000	2.50000000	-0.00312805
1253	0.06250000	2.56250000	0.01603128
1254	0.43750000	2.00000000	-0.16735093
1255	0.56250000	2.00000000	-0.17341153
1256	0.43750000	1.93750000	-0.14506354
1257	0.50000000	2.06250000	-0.16735093
1258	0.50000000	1.93750000	-0.14799609
1259	0.56250000	2.06250000	-0.17067449
1260	0.43750000	2.43750000	-0.09912023
1261	0.56250000	2.56250000	-0.08680352
1262	0.43750000	2.50000000	-0.07956989
1263	0.50000000	2.43750000	-0.10811339
1264	0.50000000	2.56250000	-0.07370479
1265	0.56250000	2.50000000	-0.10244379
1266	1.56250000	0.00000000	0.15386119
1267	1.43750000	0.00000000	0.12551320
1268	1.56250000	0.06250000	0.14076246
1269	1.50000000	0.06250000	0.12375367
1270	2.43750000	0.00000000	0.15972630
1271	2.56250000	0.00000000	0.13470186
1272	2.50000000	0.06250000	0.13822092
1273	2.56250000	0.06250000	0.12101662
1274	2.00000000	0.43750000	-0.06060606
1275	2.00000000	0.56250000	0.00918866
1276	1.93750000	0.43750000	0.03734115
1277	2.06250000	0.50000000	0.01798631
1278	1.93750000	0.50000000	0.00938416
1279	2.06250000	0.56250000	-0.02678397
1280	2.43750000	0.43750000	0.02052786
1281	2.56250000	0.56250000	-0.02561095
1282	2.50000000	0.43750000	0.01896383
1283	2.43750000	0.50000000	0.00723363
1284	2.56250000	0.50000000	-0.01818182
1285	2.50000000	0.56250000	-0.01427175
1286	1.56250000	1.56250000	-0.02893451
1287	1.43750000	1.43750000	-0.14838710
1288	1.50000000	1.56250000	-0.13782991
1289	1.56250000	1.50000000	-0.13079179
1290	1.43750000	1.50000000	-0.14819159
1291	1.50000000	1.43750000	-0.09051808
1292	2.43750000	2.43750000	-0.00977517
1293	2.56250000	2.56250000	-0.02600196
1294	2.43750000	2.50000000	0.00351906
1295	2.50000000	2.43750000	-0.01681329

1296	2.50000000	2.56250000	0.00821114
1297	2.56250000	2.50000000	-0.01642229
1298	1.56250000	2.00000000	-0.03010753
1299	1.43750000	2.00000000	-0.04887586
1300	1.50000000	1.93750000	-0.04222874
1301	1.56250000	2.06250000	-0.01661779
1302	1.43750000	1.93750000	-0.05219941
1303	1.50000000	2.06250000	-0.03382209
1304	2.00000000	1.56250000	-0.05708700
1305	2.00000000	1.43750000	-0.06177908
1306	1.93750000	1.50000000	-0.06920821
1307	2.06250000	1.56250000	-0.06744868
1308	1.93750000	1.43750000	-0.06842620
1309	2.06250000	1.50000000	-0.06666667
1310	2.00000000	2.43750000	0.04926686
1311	2.00000000	2.56250000	0.07663734
1312	2.06250000	2.50000000	0.04183773
1313	1.93750000	2.43750000	0.04046921
1314	2.06250000	2.56250000	0.06744868
1315	1.93750000	2.50000000	0.04555230
1316	2.43750000	2.00000000	-0.06862170
1317	2.56250000	2.00000000	-0.12512219
1318	2.50000000	2.06250000	-0.08836755
1319	2.43750000	1.93750000	-0.09853372
1320	2.56250000	2.06250000	-0.12336266
1321	2.50000000	1.93750000	-0.05180841
1322	1.56250000	4.00000000	-0.07663734
1323	1.43750000	4.00000000	-0.06275660
1324	1.50000000	3.93750000	-0.06588465
1325	1.43750000	3.93750000	-0.07839687
1326	2.43750000	4.00000000	-0.16187683
1327	2.56250000	4.00000000	-0.14115347
1328	2.43750000	3.93750000	-0.14877810
1329	2.50000000	3.93750000	-0.14721408
1330	1.56250000	3.56250000	0.06412512
1331	1.43750000	3.43750000	0.14682307
1332	1.50000000	3.56250000	0.14056696
1333	1.56250000	3.50000000	0.17653959
1334	1.43750000	3.50000000	0.16246334
1335	1.50000000	3.43750000	0.17458456
1336	2.00000000	3.56250000	0.17497556
1337	2.00000000	3.43750000	0.18553275
1338	2.06250000	3.56250000	0.13919844
1339	1.93750000	3.50000000	0.18572825
1340	2.06250000	3.50000000	0.18885630
1341	1.93750000	3.43750000	0.18357771
1342	4.00000000	1.56250000	-0.05043988
1343	4.00000000	1.43750000	-0.04868035
1344	3.93750000	1.50000000	-0.05708700
1345	3.93750000	1.43750000	-0.05591398
1346	4.00000000	2.43750000	-0.06392962
1347	4.00000000	2.56250000	-0.04398827
1348	3.93750000	2.43750000	-0.06901271
1349	3.93750000	2.50000000	-0.06041056
1350	3.56250000	1.56250000	-0.16637341
1351	3.43750000	1.43750000	-0.17614858
1352	3.56250000	1.50000000	-0.15679374
1353	3.50000000	1.56250000	-0.17204301
1354	3.50000000	1.43750000	-0.17360704

1355	3.43750000	1.50000000	-0.17497556
1356	3.56250000	2.00000000	-0.15718475
1357	3.43750000	2.00000000	-0.16871945
1358	3.56250000	2.06250000	-0.14897361
1359	3.50000000	1.93750000	-0.16383187
1360	3.50000000	2.06250000	-0.15053763
1361	3.43750000	1.93750000	-0.16539589
1362	0.43750000	1.00000000	-0.17888563
1363	0.56250000	1.00000000	-0.19589443
1364	0.43750000	0.93750000	-0.15112414
1365	0.50000000	0.93750000	-0.18807429
1366	0.50000000	1.06250000	-0.19491691
1367	0.56250000	1.06250000	-0.19550342
1368	0.43750000	1.43750000	-0.17693060
1369	0.56250000	1.56250000	-0.18064516
1370	0.43750000	1.50000000	-0.18983382
1371	0.50000000	1.56250000	-0.19022483
1372	0.50000000	1.43750000	-0.19315738
1373	0.56250000	1.50000000	-0.19354839
1374	1.00000000	0.43750000	-0.12453568
1375	1.00000000	0.56250000	-0.14252199
1376	0.93750000	0.43750000	-0.10928641
1377	0.93750000	0.50000000	-0.13470186
1378	1.06250000	0.50000000	-0.12629521
1379	1.06250000	0.56250000	-0.13450635
1380	1.43750000	0.43750000	0.08211144
1381	1.56250000	0.56250000	0.08035191
1382	1.50000000	0.43750000	0.10791789
1383	1.56250000	0.50000000	0.09716520
1384	1.43750000	0.50000000	0.08484848
1385	1.50000000	0.56250000	0.07214076
1386	1.00000000	1.43750000	-0.19452590
1387	1.00000000	1.56250000	-0.19706745
1388	1.06250000	1.50000000	-0.19042033
1389	1.06250000	1.56250000	-0.19002933
1390	0.93750000	1.43750000	-0.19687195
1391	0.93750000	1.50000000	-0.19667644
1392	1.43750000	1.00000000	-0.14389052
1393	1.56250000	1.00000000	-0.12766373
1394	1.50000000	1.06250000	-0.08660802
1395	1.56250000	1.06250000	-0.12492669
1396	1.43750000	0.93750000	-0.13567937
1397	1.50000000	0.93750000	-0.12434018
1398	0.43750000	3.43750000	0.16695992
1399	0.56250000	3.56250000	0.18357771
1400	0.43750000	3.50000000	0.18084066
1401	0.50000000	3.56250000	0.18318671
1402	0.50000000	3.43750000	0.16559140
1403	0.56250000	3.50000000	0.16793744
1404	0.43750000	3.00000000	0.05767351
1405	0.56250000	3.00000000	0.04281525
1406	0.43750000	2.93750000	0.05004888
1407	0.50000000	2.93750000	0.03695015
1408	0.50000000	3.06250000	0.07174976
1409	0.56250000	3.06250000	0.05845552
1410	1.00000000	3.56250000	0.09012708
1411	1.00000000	3.43750000	0.05884653
1412	1.06250000	3.56250000	0.05259042
1413	1.06250000	3.50000000	0.08699902

1414	0.93750000	3.50000000	0.08797654
1415	0.93750000	3.43750000	0.07214076
1416	3.43750000	0.43750000	0.00391007
1417	3.56250000	0.56250000	-0.10576735
1418	3.50000000	0.43750000	-0.07996090
1419	3.56250000	0.50000000	-0.09051808
1420	3.43750000	0.50000000	-0.10987292
1421	3.50000000	0.56250000	-0.11300098
1422	3.00000000	0.43750000	-0.12785924
1423	3.00000000	0.56250000	-0.14545455
1424	2.93750000	0.43750000	-0.10107527
1425	2.93750000	0.50000000	-0.12101662
1426	3.06250000	0.50000000	-0.13411535
1427	3.06250000	0.56250000	-0.14154448
1428	3.56250000	1.00000000	-0.15718475
1429	3.43750000	1.00000000	-0.16832845
1430	3.56250000	1.06250000	-0.14877810
1431	3.50000000	1.06250000	-0.16676442
1432	3.50000000	0.93750000	-0.15718475
1433	3.43750000	0.93750000	-0.17360704
1434	3.00000000	3.43750000	0.08406647
1435	3.00000000	3.56250000	0.13059629
1436	3.06250000	3.50000000	0.12121212
1437	3.06250000	3.56250000	0.14956012
1438	2.93750000	3.43750000	0.11847507
1439	2.93750000	3.50000000	0.13098729
1440	3.43750000	3.00000000	0.00840665
1441	3.56250000	3.00000000	0.03499511
1442	3.50000000	3.06250000	0.04555230
1443	3.56250000	3.06250000	-0.05063539
1444	3.43750000	2.93750000	0.01798631
1445	3.50000000	2.93750000	0.01876833
1446	2.56250000	3.00000000	0.06510264
1447	2.43750000	3.00000000	0.10889541
1448	2.50000000	2.93750000	0.08660802
1449	2.43750000	2.93750000	0.09501466
1450	2.56250000	3.06250000	0.10224829
1451	2.50000000	3.06250000	0.11202346
1452	3.00000000	2.56250000	-0.10791789
1453	3.00000000	2.43750000	-0.11124145
1454	2.93750000	2.50000000	-0.08660802
1455	2.93750000	2.43750000	-0.12355816
1456	3.06250000	2.56250000	-0.09423265
1457	3.06250000	2.50000000	-0.09677419
1458	2.56250000	3.56250000	0.19980450
1459	2.43750000	3.43750000	0.19667644
1460	2.50000000	3.56250000	0.15053763
1461	2.43750000	3.50000000	0.19843597
1462	2.56250000	3.50000000	0.19002933
1463	2.50000000	3.43750000	0.18748778
1464	3.56250000	2.56250000	-0.07683284
1465	3.43750000	2.43750000	-0.10811339
1466	3.56250000	2.50000000	-0.08856305
1467	3.50000000	2.43750000	-0.10185728
1468	3.50000000	2.56250000	-0.07722385
1469	3.43750000	2.50000000	-0.09384164
1470	1.00000000	2.43750000	-0.17712610
1471	1.00000000	2.56250000	-0.15894428
1472	0.93750000	2.43750000	-0.17399804

1473	0.93750000	2.50000000	-0.16480938
1474	1.06250000	2.50000000	-0.16207234
1475	1.06250000	2.56250000	-0.13294233
1476	1.43750000	2.43750000	-0.08954057
1477	1.56250000	2.56250000	-0.04340176
1478	1.50000000	2.43750000	-0.07956989
1479	1.56250000	2.50000000	-0.05728250
1480	1.43750000	2.50000000	-0.08699902
1481	1.50000000	2.56250000	-0.04535679
1482	1.43750000	3.00000000	0.13411535
1483	1.56250000	3.00000000	0.16050831
1484	1.50000000	3.06250000	0.16070381
1485	1.56250000	3.06250000	0.16226784
1486	1.43750000	2.93750000	0.11691105
1487	1.50000000	2.93750000	0.12942326
1488	2.43750000	1.00000000	-0.10361681
1489	2.56250000	1.00000000	-0.11964809
1490	2.43750000	0.93750000	-0.09325513
1491	2.50000000	0.93750000	-0.09501466
1492	2.50000000	1.06250000	-0.11026393
1493	2.56250000	1.06250000	-0.11808407
1494	2.43750000	1.43750000	-0.06608016
1495	2.56250000	1.56250000	-0.13235582
1496	2.43750000	1.50000000	-0.11456500
1497	2.50000000	1.56250000	-0.11867058
1498	2.50000000	1.43750000	-0.12082111
1499	2.56250000	1.50000000	-0.13040078
1500	3.00000000	1.43750000	-0.18690127
1501	3.00000000	1.56250000	-0.18748778
1502	3.06250000	1.50000000	-0.18416422
1503	3.06250000	1.56250000	-0.18260020
1504	2.93750000	1.43750000	-0.17595308
1505	2.93750000	1.50000000	-0.17751711
1506	0.00000000	0.18750000	0.07155425
1507	0.00000000	0.31250000	0.02365591
1508	0.06250000	0.25000000	0.03206256
1509	0.06250000	0.31250000	0.01857283
1510	0.18750000	0.00000000	0.10420332
1511	0.31250000	0.00000000	0.08406647
1512	0.25000000	0.06250000	0.06471163
1513	0.31250000	0.06250000	0.05884653
1514	0.18750000	0.18750000	0.02776149
1515	0.31250000	0.31250000	-0.04183773
1516	0.18750000	0.25000000	0.00273705
1517	0.25000000	0.18750000	0.01368524
1518	0.25000000	0.31250000	-0.02521994
1519	0.31250000	0.25000000	-0.01759531
1520	0.00000000	3.81250000	0.18748778
1521	0.00000000	3.68750000	0.18357771
1522	0.06250000	3.81250000	0.18787879
1523	0.06250000	3.75000000	0.18611926
1524	0.18750000	4.00000000	0.19452590
1525	0.31250000	4.00000000	0.19530792
1526	0.18750000	3.93750000	0.19257087
1527	0.25000000	3.93750000	0.19315738
1528	3.81250000	0.00000000	0.13098729
1529	3.68750000	0.00000000	0.09833822
1530	3.81250000	0.06250000	0.11006843
1531	3.75000000	0.06250000	0.09384164

1532	4.00000000	0.18750000	0.11691105
1533	4.00000000	0.31250000	0.07741935
1534	3.93750000	0.18750000	0.10068426
1535	3.93750000	0.25000000	0.07976540
1536	3.81250000	3.81250000	0.17106549
1537	3.68750000	3.68750000	0.16461388
1538	3.75000000	3.81250000	0.17086999
1539	3.81250000	3.75000000	0.16441838
1540	3.68750000	3.75000000	0.16109482
1541	3.75000000	3.68750000	0.15464321
1542	3.81250000	4.00000000	0.19980450
1543	3.68750000	4.00000000	0.19941349
1544	3.75000000	3.93750000	0.19002933
1545	3.68750000	3.93750000	0.18983382
1546	4.00000000	3.81250000	0.18005865
1547	4.00000000	3.68750000	0.16637341
1548	3.93750000	3.75000000	0.17047898
1549	3.93750000	3.68750000	0.16363636
1550	0.00000000	1.81250000	-0.15542522
1551	0.00000000	1.68750000	-0.16031281
1552	0.06250000	1.81250000	-0.15835777
1553	0.06250000	1.75000000	-0.13587488
1554	0.00000000	2.18750000	-0.08914956
1555	0.00000000	2.31250000	-0.04965787
1556	0.06250000	2.25000000	-0.08289345
1557	0.06250000	2.31250000	-0.06041056
1558	0.18750000	2.00000000	-0.15601173
1559	0.31250000	2.00000000	-0.16168133
1560	0.18750000	1.93750000	-0.15874878
1561	0.25000000	2.06250000	-0.14975562
1562	0.25000000	1.93750000	-0.16148583
1563	0.31250000	2.06250000	-0.14643206
1564	0.18750000	2.18750000	-0.12883675
1565	0.31250000	2.31250000	-0.11476051
1566	0.18750000	2.25000000	-0.10928641
1567	0.25000000	2.18750000	-0.13157380
1568	0.25000000	2.31250000	-0.10303030
1569	0.31250000	2.25000000	-0.11964809
1570	1.81250000	0.00000000	0.16344086
1571	1.68750000	0.00000000	0.15855327
1572	1.81250000	0.06250000	0.14995112
1573	1.75000000	0.06250000	0.14780059
1574	2.18750000	0.00000000	0.16637341
1575	2.31250000	0.00000000	0.16285435
1576	2.25000000	0.06250000	0.14447703
1577	2.31250000	0.06250000	0.14291300
1578	2.00000000	0.18750000	0.13040078
1579	2.00000000	0.31250000	-0.00879765
1580	1.93750000	0.18750000	0.12805474
1581	2.06250000	0.25000000	0.10909091
1582	1.93750000	0.25000000	0.11515152
1583	2.06250000	0.31250000	0.09579668
1584	2.18750000	0.18750000	0.10615836
1585	2.31250000	0.31250000	0.06334311
1586	2.25000000	0.18750000	0.10459433
1587	2.18750000	0.25000000	0.09286413
1588	2.31250000	0.25000000	0.08308895
1589	2.25000000	0.31250000	0.07135875
1590	1.81250000	1.81250000	-0.08132942

1591	1.68750000	1.68750000	-0.00527859
1592	1.75000000	1.81250000	-0.09012708
1593	1.81250000	1.75000000	-0.08328446
1594	1.68750000	1.75000000	-0.10244379
1595	1.75000000	1.68750000	-0.09540567
1596	2.18750000	2.18750000	-0.02971652
1597	2.31250000	2.31250000	-0.01974585
1598	2.18750000	2.25000000	-0.01622678
1599	2.25000000	2.18750000	-0.03714565
1600	2.25000000	2.31250000	-0.01133920
1601	2.31250000	2.25000000	-0.03167155
1602	1.81250000	2.00000000	-0.07116325
1603	1.68750000	2.00000000	-0.06334311
1604	1.75000000	1.93750000	-0.08328446
1605	1.81250000	2.06250000	-0.05767351
1606	1.68750000	1.93750000	-0.09208211
1607	1.75000000	2.06250000	-0.06647116
1608	2.00000000	1.81250000	-0.05004888
1609	2.00000000	1.68750000	-0.05356794
1610	1.93750000	1.75000000	-0.06236559
1611	2.06250000	1.81250000	-0.05728250
1612	1.93750000	1.68750000	-0.06412512
1613	2.06250000	1.75000000	-0.06236559
1614	2.00000000	2.18750000	-0.00430108
1615	2.00000000	2.31250000	0.02248289
1616	2.06250000	2.25000000	0.00078201
1617	1.93750000	2.18750000	-0.01309873
1618	2.06250000	2.31250000	0.01407625
1619	1.93750000	2.25000000	0.00039101
1620	2.18750000	2.00000000	-0.06686217
1621	2.31250000	2.00000000	-0.05552297
1622	2.25000000	2.06250000	-0.06177908
1623	2.18750000	1.93750000	-0.06842620
1624	2.31250000	2.06250000	-0.06862170
1625	2.25000000	1.93750000	-0.07898338
1626	1.81250000	4.00000000	0.01798631
1627	1.68750000	4.00000000	-0.02932551
1628	1.75000000	3.93750000	0.02873900
1629	1.68750000	3.93750000	0.00508309
1630	2.18750000	4.00000000	-0.01798631
1631	2.31250000	4.00000000	-0.09032258
1632	2.18750000	3.93750000	-0.00566960
1633	2.25000000	3.93750000	-0.04144673
1634	1.81250000	3.81250000	0.12121212
1635	1.68750000	3.68750000	0.04261975
1636	1.75000000	3.81250000	0.09755621
1637	1.81250000	3.75000000	0.13352884
1638	1.68750000	3.75000000	0.10830890
1639	1.75000000	3.68750000	0.14428152
1640	2.00000000	3.81250000	0.12590420
1641	2.00000000	3.68750000	0.15053763
1642	2.06250000	3.81250000	0.09032258
1643	1.93750000	3.75000000	0.13665689
1644	2.06250000	3.75000000	0.10244379
1645	1.93750000	3.68750000	0.14897361
1646	4.00000000	1.81250000	-0.09090909
1647	4.00000000	1.68750000	-0.07057674
1648	3.93750000	1.75000000	-0.09677419
1649	3.93750000	1.68750000	-0.08719453

1650	4.00000000	2.18750000	-0.09677419
1651	4.00000000	2.31250000	-0.08035191
1652	3.93750000	2.18750000	-0.10185728
1653	3.93750000	2.25000000	-0.09364614
1654	3.81250000	1.81250000	-0.13919844
1655	3.68750000	1.68750000	-0.15229717
1656	3.81250000	1.75000000	-0.12903226
1657	3.75000000	1.81250000	-0.14428152
1658	3.75000000	1.68750000	-0.13567937
1659	3.68750000	1.75000000	-0.15092864
1660	3.81250000	2.00000000	-0.13665689
1661	3.68750000	2.00000000	-0.14682307
1662	3.81250000	2.06250000	-0.12844575
1663	3.75000000	1.93750000	-0.14252199
1664	3.75000000	2.06250000	-0.13352884
1665	3.68750000	1.93750000	-0.14819159
1666	0.00000000	0.81250000	-0.05708700
1667	0.00000000	0.68750000	-0.05219941
1668	0.06250000	0.81250000	-0.07331378
1669	0.06250000	0.75000000	-0.07057674
1670	0.00000000	1.18750000	-0.10361681
1671	0.00000000	1.31250000	-0.12922776
1672	0.06250000	1.25000000	-0.11964809
1673	0.06250000	1.31250000	-0.13274682
1674	0.18750000	1.00000000	-0.11358749
1675	0.31250000	1.00000000	-0.14623656
1676	0.18750000	0.93750000	-0.11104594
1677	0.25000000	1.06250000	-0.12961877
1678	0.25000000	0.93750000	-0.12727273
1679	0.31250000	1.06250000	-0.14604106
1680	0.18750000	1.18750000	-0.11280547
1681	0.31250000	1.31250000	-0.14486804
1682	0.18750000	1.25000000	-0.12570870
1683	0.25000000	1.18750000	-0.12903226
1684	0.25000000	1.31250000	-0.14173998
1685	0.31250000	1.25000000	-0.14506354
1686	0.81250000	0.00000000	-0.01036168
1687	0.68750000	0.00000000	0.02033236
1688	0.81250000	0.06250000	-0.02013685
1689	0.75000000	0.06250000	-0.00488759
1690	1.18750000	0.00000000	0.02150538
1691	1.31250000	0.00000000	0.07350929
1692	1.25000000	0.06250000	0.01994135
1693	1.31250000	0.06250000	0.04574780
1694	1.00000000	0.18750000	-0.08543500
1695	1.00000000	0.31250000	-0.10498534
1696	0.93750000	0.18750000	-0.07018573
1697	1.06250000	0.25000000	-0.08719453
1698	0.93750000	0.25000000	-0.07996090
1699	1.06250000	0.31250000	-0.09696970
1700	1.18750000	0.18750000	-0.06138807
1701	1.31250000	0.31250000	-0.01466276
1702	1.25000000	0.18750000	-0.03538612
1703	1.18750000	0.25000000	-0.07116325
1704	1.31250000	0.25000000	-0.03734115
1705	1.25000000	0.31250000	-0.07292278
1706	0.81250000	0.81250000	-0.17614858
1707	0.68750000	0.68750000	-0.15757576
1708	0.75000000	0.81250000	-0.17556207

1709	0.81250000	0.75000000	-0.16793744
1710	0.68750000	0.75000000	-0.16774194
1711	0.75000000	0.68750000	-0.16011730
1712	1.18750000	1.18750000	-0.17829912
1713	1.31250000	1.31250000	-0.16344086
1714	1.18750000	1.25000000	-0.17790811
1715	1.25000000	1.18750000	-0.17028348
1716	1.25000000	1.31250000	-0.17067449
1717	1.31250000	1.25000000	-0.12551320
1718	0.81250000	1.00000000	-0.19824047
1719	0.68750000	1.00000000	-0.19706745
1720	0.75000000	0.93750000	-0.19022483
1721	0.81250000	1.06250000	-0.19784946
1722	0.68750000	0.93750000	-0.18963832
1723	0.75000000	1.06250000	-0.19726295
1724	1.00000000	0.81250000	-0.17536657
1725	1.00000000	0.68750000	-0.15894428
1726	0.93750000	0.75000000	-0.16735093
1727	1.06250000	0.81250000	-0.16735093
1728	0.93750000	0.68750000	-0.15933529
1729	1.06250000	0.75000000	-0.15913978
1730	1.00000000	1.18750000	-0.19882698
1731	1.00000000	1.31250000	-0.19511241
1732	1.06250000	1.25000000	-0.19159335
1733	0.93750000	1.18750000	-0.19824047
1734	1.06250000	1.31250000	-0.19120235
1735	0.93750000	1.25000000	-0.19784946
1736	1.18750000	1.00000000	-0.17595308
1737	1.31250000	1.00000000	-0.15992180
1738	1.25000000	1.06250000	-0.16871945
1739	1.18750000	0.93750000	-0.16774194
1740	1.31250000	1.06250000	-0.11065494
1741	1.25000000	0.93750000	-0.15972630
1742	0.00000000	3.18750000	0.11026393
1743	0.00000000	3.31250000	0.13743891
1744	0.06250000	3.25000000	0.12434018
1745	0.06250000	3.31250000	0.13802542
1746	0.00000000	2.81250000	0.04574780
1747	0.00000000	2.68750000	0.05630499
1748	0.06250000	2.81250000	0.04437928
1749	0.06250000	2.75000000	0.03655914
1750	0.18750000	3.18750000	0.11104594
1751	0.31250000	3.31250000	0.13880743
1752	0.18750000	3.25000000	0.12473118
1753	0.25000000	3.18750000	0.10928641
1754	0.25000000	3.31250000	0.13861193
1755	0.31250000	3.25000000	0.12336266
1756	0.18750000	3.00000000	0.06432063
1757	0.31250000	3.00000000	0.06099707
1758	0.18750000	2.93750000	0.05669599
1759	0.25000000	3.06250000	0.07820137
1760	0.25000000	2.93750000	0.05552297
1761	0.31250000	3.06250000	0.07663734
1762	0.81250000	4.00000000	0.19843597
1763	0.68750000	4.00000000	0.19765396
1764	0.75000000	3.93750000	0.19628543
1765	0.68750000	3.93750000	0.19589443
1766	1.18750000	4.00000000	0.08739003
1767	1.31250000	4.00000000	0.01231672

1768	1.18750000	3.93750000	0.07174976
1769	1.25000000	3.93750000	0.03421310
1770	0.81250000	3.81250000	0.19296188
1771	0.68750000	3.68750000	0.18826979
1772	0.75000000	3.81250000	0.19257087
1773	0.81250000	3.75000000	0.17732160
1774	0.68750000	3.75000000	0.19022483
1775	0.75000000	3.68750000	0.17497556
1776	1.00000000	3.81250000	0.15268817
1777	1.00000000	3.68750000	0.12140762
1778	1.06250000	3.81250000	0.11534702
1779	0.93750000	3.75000000	0.15053763
1780	1.06250000	3.75000000	0.09970674
1781	0.93750000	3.68750000	0.13489736
1782	3.18750000	0.00000000	0.00000000
1783	3.31250000	0.00000000	0.01994135
1784	3.25000000	0.06250000	-0.00938416
1785	3.31250000	0.06250000	0.00039101
1786	2.81250000	0.00000000	0.04086022
1787	2.68750000	0.00000000	0.08778104
1788	2.81250000	0.06250000	0.02404692
1789	2.75000000	0.06250000	0.05083089
1790	3.18750000	0.18750000	-0.05826002
1791	3.31250000	0.31250000	0.02287390
1792	3.25000000	0.18750000	-0.04828935
1793	3.18750000	0.25000000	-0.07507331
1794	3.31250000	0.25000000	-0.05786901
1795	3.25000000	0.31250000	-0.08132942
1796	3.00000000	0.18750000	-0.07996090
1797	3.00000000	0.31250000	-0.10713587
1798	2.93750000	0.18750000	-0.05650049
1799	3.06250000	0.25000000	-0.08954057
1800	2.93750000	0.25000000	-0.07018573
1801	3.06250000	0.31250000	-0.10303030
1802	4.00000000	0.81250000	-0.06236559
1803	4.00000000	0.68750000	-0.03030303
1804	3.93750000	0.75000000	-0.06217009
1805	3.93750000	0.68750000	-0.04731183
1806	4.00000000	1.18750000	-0.08191593
1807	4.00000000	1.31250000	-0.06529814
1808	3.93750000	1.18750000	-0.08914956
1809	3.93750000	1.25000000	-0.08074291
1810	3.81250000	0.81250000	-0.10635386
1811	3.68750000	0.68750000	-0.10596285
1812	3.81250000	0.75000000	-0.09149560
1813	3.75000000	0.81250000	-0.11339198
1814	3.75000000	0.68750000	-0.09130010
1815	3.68750000	0.75000000	-0.11339198
1816	3.81250000	1.00000000	-0.12825024
1817	3.68750000	1.00000000	-0.14271750
1818	3.81250000	1.06250000	-0.12003910
1819	3.75000000	0.93750000	-0.12805474
1820	3.75000000	1.06250000	-0.12707722
1821	3.68750000	0.93750000	-0.13548387
1822	3.18750000	3.18750000	-0.06353861
1823	3.31250000	3.31250000	0.02385142
1824	3.18750000	3.25000000	-0.02658847
1825	3.25000000	3.18750000	-0.03734115
1826	3.25000000	3.31250000	0.01055718

1827	3.31250000	3.25000000	-0.00019550
1828	2.81250000	2.81250000	-0.11104594
1829	2.68750000	2.68750000	-0.06842620
1830	2.75000000	2.81250000	-0.07663734
1831	2.81250000	2.75000000	-0.10146628
1832	2.68750000	2.75000000	-0.05532747
1833	2.75000000	2.68750000	-0.08015640
1834	3.00000000	3.18750000	-0.06373412
1835	3.00000000	3.31250000	0.01016618
1836	3.06250000	3.25000000	-0.02678397
1837	2.93750000	3.18750000	-0.02952102
1838	3.06250000	3.31250000	0.01036168
1839	2.93750000	3.25000000	0.00762463
1840	3.18750000	3.00000000	-0.09638319
1841	3.31250000	3.00000000	-0.04398827
1842	3.25000000	3.06250000	-0.05923754
1843	3.18750000	2.93750000	-0.08680352
1844	3.31250000	3.06250000	-0.03304008
1845	3.25000000	2.93750000	-0.06060606
1846	2.81250000	3.00000000	-0.07194526
1847	2.68750000	3.00000000	-0.00351906
1848	2.75000000	2.93750000	-0.05063539
1849	2.81250000	3.06250000	-0.03499511
1850	2.68750000	2.93750000	-0.01642229
1851	2.75000000	3.06250000	-0.00058651
1852	3.00000000	2.81250000	-0.14623656
1853	3.00000000	2.68750000	-0.12707722
1854	2.93750000	2.75000000	-0.12492669
1855	3.06250000	2.81250000	-0.12003910
1856	2.93750000	2.68750000	-0.11534702
1857	3.06250000	2.75000000	-0.11045943
1858	3.18750000	4.00000000	0.19824047
1859	3.31250000	4.00000000	0.19863148
1860	3.18750000	3.93750000	0.18866080
1861	3.25000000	3.93750000	0.19511241
1862	2.81250000	4.00000000	0.04985337
1863	2.68750000	4.00000000	-0.04242424
1864	2.75000000	3.93750000	0.05024438
1865	2.68750000	3.93750000	0.00097752
1866	3.00000000	3.81250000	0.16891496
1867	3.00000000	3.68750000	0.14975562
1868	3.06250000	3.81250000	0.16911046
1869	2.93750000	3.75000000	0.16930596
1870	3.06250000	3.75000000	0.16578690
1871	2.93750000	3.68750000	0.15972630
1872	2.81250000	3.81250000	0.19863148
1873	2.68750000	3.68750000	0.19921799
1874	2.75000000	3.81250000	0.14936461
1875	2.81250000	3.75000000	0.18905181
1876	2.68750000	3.75000000	0.14956012
1877	2.75000000	3.68750000	0.18924731
1878	4.00000000	3.18750000	0.09110459
1879	4.00000000	3.31250000	0.11788856
1880	3.93750000	3.18750000	0.07820137
1881	3.93750000	3.25000000	0.09247312
1882	4.00000000	2.81250000	0.00215054
1883	4.00000000	2.68750000	-0.02052786
1884	3.93750000	2.75000000	-0.01348974
1885	3.93750000	2.68750000	-0.02521994

1886	3.81250000	3.00000000	0.03577713
1887	3.68750000	3.00000000	0.03577713
1888	3.81250000	3.06250000	0.05024438
1889	3.75000000	2.93750000	0.02033236
1890	3.75000000	3.06250000	0.04985337
1891	3.68750000	2.93750000	0.01994135
1892	3.81250000	2.81250000	-0.01114370
1893	3.68750000	2.68750000	-0.04398827
1894	3.81250000	2.75000000	-0.02287390
1895	3.75000000	2.81250000	-0.01153470
1896	3.75000000	2.68750000	-0.03929619
1897	3.68750000	2.75000000	-0.02795699
1898	0.81250000	2.00000000	-0.18611926
1899	0.68750000	2.00000000	-0.17966764
1900	0.75000000	1.93750000	-0.18553275
1901	0.81250000	2.06250000	-0.18338221
1902	0.68750000	1.93750000	-0.18240469
1903	0.75000000	2.06250000	-0.18005865
1904	1.18750000	2.00000000	-0.16539589
1905	1.31250000	2.00000000	-0.11984360
1906	1.18750000	1.93750000	-0.16559140
1907	1.25000000	2.06250000	-0.15034213
1908	1.25000000	1.93750000	-0.15874878
1909	1.31250000	2.06250000	-0.14017595
1910	0.81250000	1.81250000	-0.19452590
1911	0.68750000	1.68750000	-0.18123167
1912	0.75000000	1.81250000	-0.19120235
1913	0.81250000	1.75000000	-0.19472141
1914	0.68750000	1.75000000	-0.19100684
1915	0.75000000	1.68750000	-0.19433040
1916	1.00000000	1.81250000	-0.19628543
1917	1.00000000	1.68750000	-0.19667644
1918	1.06250000	1.81250000	-0.18611926
1919	0.93750000	1.75000000	-0.19589443
1920	1.06250000	1.75000000	-0.18963832
1921	0.93750000	1.68750000	-0.19628543
1922	1.00000000	2.18750000	-0.18768328
1923	1.00000000	2.31250000	-0.18240469
1924	0.93750000	2.18750000	-0.18455523
1925	1.06250000	2.25000000	-0.17262952
1926	0.93750000	2.25000000	-0.18201369
1927	1.06250000	2.31250000	-0.16989247
1928	1.18750000	2.18750000	-0.15014663
1929	1.31250000	2.31250000	-0.11984360
1930	1.25000000	2.18750000	-0.14017595
1931	1.18750000	2.25000000	-0.14760508
1932	1.31250000	2.25000000	-0.12492669
1933	1.25000000	2.31250000	-0.13235582
1934	0.81250000	2.81250000	-0.06783969
1935	0.68750000	2.68750000	-0.07800587
1936	0.75000000	2.81250000	-0.05454545
1937	0.81250000	2.75000000	-0.08328446
1938	0.68750000	2.75000000	-0.06041056
1939	0.75000000	2.68750000	-0.08914956
1940	1.18750000	3.18750000	0.03421310
1941	1.31250000	3.31250000	0.09051808
1942	1.18750000	3.25000000	0.04985337
1943	1.25000000	3.18750000	0.06197458
1944	1.25000000	3.31250000	0.07800587

1945	1.31250000	3.25000000	0.08856305
1946	0.81250000	3.00000000	-0.01036168
1947	0.68750000	3.00000000	0.01622678
1948	0.75000000	2.93750000	-0.01622678
1949	0.81250000	3.06250000	0.00527859
1950	0.68750000	2.93750000	-0.00312805
1951	0.75000000	3.06250000	0.01837732
1952	1.00000000	3.18750000	-0.00371457
1953	1.00000000	3.31250000	0.02756598
1954	1.06250000	3.25000000	0.02463343
1955	0.93750000	3.18750000	0.00977517
1956	1.06250000	3.31250000	0.04027370
1957	0.93750000	3.25000000	0.02521994
1958	1.00000000	2.81250000	-0.09696970
1959	1.00000000	2.68750000	-0.12785924
1960	0.93750000	2.75000000	-0.10263930
1961	1.06250000	2.81250000	-0.06920821
1962	0.93750000	2.68750000	-0.11827957
1963	1.06250000	2.75000000	-0.08621701
1964	1.18750000	3.00000000	0.03264907
1965	1.31250000	3.00000000	0.08504399
1966	1.25000000	3.06250000	0.06099707
1967	1.18750000	2.93750000	0.01720430
1968	1.31250000	3.06250000	0.08699902
1969	1.25000000	2.93750000	0.04340176
1970	2.00000000	0.81250000	-0.02052786
1971	2.00000000	0.68750000	0.00058651
1972	1.93750000	0.75000000	-0.02033236
1973	2.06250000	0.81250000	-0.02502444
1974	1.93750000	0.68750000	-0.00351906
1975	2.06250000	0.75000000	-0.01466276
1976	2.00000000	1.18750000	-0.07096774
1977	2.00000000	1.31250000	-0.06783969
1978	1.93750000	1.18750000	-0.07898338
1979	2.06250000	1.25000000	-0.02717498
1980	1.93750000	1.25000000	-0.07761486
1981	2.06250000	1.31250000	-0.07585533
1982	1.81250000	0.81250000	-0.07018573
1983	1.68750000	0.68750000	-0.01994135
1984	1.81250000	0.75000000	-0.05356794
1985	1.75000000	0.81250000	-0.07839687
1986	1.75000000	0.68750000	-0.05337243
1987	1.68750000	0.75000000	-0.07839687
1988	1.81250000	1.00000000	-0.09481916
1989	1.68750000	1.00000000	-0.11124145
1990	1.81250000	1.06250000	-0.09501466
1991	1.75000000	0.93750000	-0.09481916
1992	1.75000000	1.06250000	-0.10146628
1993	1.68750000	0.93750000	-0.10283480
1994	2.18750000	1.00000000	-0.08465298
1995	2.31250000	1.00000000	-0.09403715
1996	2.18750000	0.93750000	-0.06783969
1997	2.25000000	1.06250000	-0.04105572
1998	2.25000000	0.93750000	-0.07878788
1999	2.31250000	1.06250000	-0.09579668
2000	2.18750000	1.18750000	0.06021505
2001	2.31250000	1.31250000	0.04711632
2002	2.18750000	1.25000000	0.06021505
2003	2.25000000	1.18750000	0.05552297

2004	2.25000000	1.31250000	0.05532747
2005	2.31250000	1.25000000	0.04907136
2006	2.81250000	0.81250000	-0.12375367
2007	2.68750000	0.68750000	-0.07487781
2008	2.81250000	0.75000000	-0.11652004
2009	2.75000000	0.81250000	-0.11280547
2010	2.75000000	0.68750000	-0.09208211
2011	2.68750000	0.75000000	-0.08817204
2012	3.18750000	1.18750000	-0.18768328
2013	3.31250000	1.31250000	-0.18181818
2014	3.25000000	1.18750000	-0.18377322
2015	3.18750000	1.25000000	-0.18631476
2016	3.31250000	1.25000000	-0.18201369
2017	3.25000000	1.31250000	-0.18338221
2018	3.00000000	0.81250000	-0.17438905
2019	3.00000000	0.68750000	-0.15992180
2020	2.93750000	0.75000000	-0.15034213
2021	3.06250000	0.81250000	-0.17047898
2022	2.93750000	0.68750000	-0.14271750
2023	3.06250000	0.75000000	-0.16324536
2024	3.18750000	1.00000000	-0.18435973
2025	3.31250000	1.00000000	-0.17634409
2026	3.25000000	1.06250000	-0.18162268
2027	3.18750000	0.93750000	-0.17712610
2028	3.31250000	1.06250000	-0.17869013
2029	3.25000000	0.93750000	-0.17321603
2030	2.81250000	1.00000000	-0.16363636
2031	2.68750000	1.00000000	-0.14173998
2032	2.75000000	0.93750000	-0.13939394
2033	2.81250000	1.06250000	-0.16226784
2034	2.68750000	0.93750000	-0.12805474
2035	2.75000000	1.06250000	-0.15131965
2036	3.00000000	1.18750000	-0.19237537
2037	3.00000000	1.31250000	-0.18963832
2038	3.06250000	1.25000000	-0.18944282
2039	2.93750000	1.18750000	-0.18142717
2040	3.06250000	1.31250000	-0.18826979
2041	2.93750000	1.25000000	-0.18005865
2042	2.00000000	2.81250000	0.13059629
2043	2.00000000	2.68750000	0.10439883
2044	2.06250000	2.81250000	0.12297165
2045	1.93750000	2.75000000	0.10107527
2046	2.06250000	2.75000000	0.10909091
2047	1.93750000	2.68750000	0.08739003
2048	2.00000000	3.18750000	0.17732160
2049	2.00000000	3.31250000	0.18220919
2050	2.06250000	3.25000000	0.18201369
2051	1.93750000	3.18750000	0.17693060
2052	2.06250000	3.31250000	0.18377322
2053	1.93750000	3.25000000	0.17869013
2054	2.18750000	3.00000000	0.14447703
2055	2.31250000	3.00000000	0.12746823
2056	2.18750000	2.93750000	0.13216031
2057	2.25000000	3.06250000	0.14916911
2058	2.25000000	2.93750000	0.12277615
2059	2.31250000	3.06250000	0.13998045
2060	2.18750000	3.18750000	0.18181818
2061	2.31250000	3.31250000	0.19002933
2062	2.18750000	3.25000000	0.18514174

2063	2.25000000	3.18750000	0.17419355
2064	2.25000000	3.31250000	0.18846530
2065	2.31250000	3.25000000	0.17751711
2066	1.81250000	2.81250000	0.07898338
2067	1.68750000	2.68750000	0.01857283
2068	1.81250000	2.75000000	0.06666667
2069	1.75000000	2.81250000	0.07878788
2070	1.75000000	2.68750000	0.03577713
2071	1.68750000	2.75000000	0.04770283
2072	1.81250000	3.00000000	0.16676442
2073	1.68750000	3.00000000	0.16441838
2074	1.81250000	3.06250000	0.16989247
2075	1.75000000	2.93750000	0.13724340
2076	1.75000000	3.06250000	0.16813294
2077	1.68750000	2.93750000	0.13528837
2078	2.81250000	2.00000000	-0.19980450
2079	2.68750000	2.00000000	-0.17478006
2080	2.81250000	2.06250000	-0.18729228
2081	2.75000000	1.93750000	-0.19002933
2082	2.75000000	2.06250000	-0.19941349
2083	2.68750000	1.93750000	-0.17634409
2084	3.18750000	2.00000000	-0.18592375
2085	3.31250000	2.00000000	-0.17810362
2086	3.25000000	2.06250000	-0.17262952
2087	3.18750000	1.93750000	-0.18416422
2088	3.31250000	2.06250000	-0.16793744
2089	3.25000000	1.93750000	-0.17947214
2090	3.00000000	2.18750000	-0.16246334
2091	3.00000000	2.31250000	-0.13704790
2092	2.93750000	2.18750000	-0.16246334
2093	3.06250000	2.25000000	-0.14897361
2094	2.93750000	2.25000000	-0.16226784
2095	3.06250000	2.31250000	-0.13626588
2096	3.18750000	2.18750000	-0.15972630
2097	3.31250000	2.31250000	-0.13470186
2098	3.25000000	2.18750000	-0.15522972
2099	3.18750000	2.25000000	-0.14721408
2100	3.31250000	2.25000000	-0.14173998
2101	3.25000000	2.31250000	-0.13372434
2102	2.81250000	1.81250000	-0.17106549
2103	2.68750000	1.68750000	-0.15171065
2104	2.75000000	1.81250000	-0.17086999
2105	2.81250000	1.75000000	-0.16911046
2106	2.68750000	1.75000000	-0.14760508
2107	2.75000000	1.68750000	-0.15953079
2108	3.00000000	1.81250000	-0.19472141
2109	3.00000000	1.68750000	-0.19120235
2110	3.06250000	1.81250000	-0.19002933
2111	2.93750000	1.75000000	-0.18494624
2112	3.06250000	1.75000000	-0.18826979
2113	2.93750000	1.68750000	-0.18318671
2114	0.18750000	0.50000000	-0.06060606
2115	0.31250000	0.50000000	-0.08211144
2116	0.18750000	0.43750000	-0.03753666
2117	0.25000000	0.43750000	-0.05200391
2118	0.25000000	0.56250000	-0.07878788
2119	0.31250000	0.56250000	-0.09071359
2120	0.18750000	0.68750000	-0.10068426
2121	0.31250000	0.81250000	-0.11300098

2122	0.18750000	0.75000000	-0.10283480
2123	0.25000000	0.81250000	-0.12179863
2124	0.25000000	0.68750000	-0.10557185
2125	0.31250000	0.75000000	-0.13079179
2126	0.50000000	0.18750000	-0.00957967
2127	0.50000000	0.31250000	-0.05923754
2128	0.43750000	0.18750000	-0.00371457
2129	0.43750000	0.25000000	-0.02913001
2130	0.56250000	0.25000000	-0.03479961
2131	0.56250000	0.31250000	-0.06001955
2132	0.68750000	0.18750000	-0.00918866
2133	0.81250000	0.31250000	-0.05923754
2134	0.75000000	0.18750000	-0.02443793
2135	0.81250000	0.25000000	-0.04946237
2136	0.68750000	0.25000000	-0.03440860
2137	0.75000000	0.31250000	-0.05943304
2138	0.50000000	0.68750000	-0.15738025
2139	0.50000000	0.81250000	-0.17341153
2140	0.56250000	0.75000000	-0.16656891
2141	0.56250000	0.81250000	-0.17380254
2142	0.43750000	0.68750000	-0.14095797
2143	0.43750000	0.75000000	-0.15425220
2144	0.68750000	0.50000000	-0.13489736
2145	0.81250000	0.50000000	-0.13528837
2146	0.75000000	0.56250000	-0.14369501
2147	0.81250000	0.56250000	-0.14349951
2148	0.68750000	0.43750000	-0.11026393
2149	0.75000000	0.43750000	-0.11006843
2150	0.18750000	3.68750000	0.15992180
2151	0.31250000	3.81250000	0.16461388
2152	0.18750000	3.75000000	0.16207234
2153	0.25000000	3.81250000	0.16441838
2154	0.25000000	3.68750000	0.16050831
2155	0.31250000	3.75000000	0.16285435
2156	0.18750000	3.50000000	0.17908113
2157	0.31250000	3.50000000	0.18005865
2158	0.18750000	3.43750000	0.16578690
2159	0.25000000	3.43750000	0.16598240
2160	0.25000000	3.56250000	0.18162268
2161	0.31250000	3.56250000	0.18201369
2162	0.50000000	3.81250000	0.19100684
2163	0.50000000	3.68750000	0.18709677
2164	0.56250000	3.81250000	0.19139785
2165	0.56250000	3.75000000	0.18944282
2166	0.43750000	3.75000000	0.18866080
2167	0.43750000	3.68750000	0.18670577
2168	3.68750000	0.18750000	0.03577713
2169	3.81250000	0.31250000	0.03030303
2170	3.75000000	0.18750000	0.05200391
2171	3.81250000	0.25000000	0.04731183
2172	3.68750000	0.25000000	0.01642229
2173	3.75000000	0.31250000	0.01173021
2174	3.50000000	0.18750000	-0.01173021
2175	3.50000000	0.31250000	-0.04105572
2176	3.43750000	0.18750000	-0.01857283
2177	3.43750000	0.25000000	-0.03792766
2178	3.56250000	0.25000000	-0.01309873
2179	3.56250000	0.31250000	-0.03264907
2180	3.81250000	0.50000000	-0.02952102

2181	3.68750000	0.50000000	-0.06099707
2182	3.81250000	0.56250000	-0.04692082
2183	3.75000000	0.56250000	-0.06158358
2184	3.75000000	0.43750000	-0.02717498
2185	3.68750000	0.43750000	-0.04203324
2186	3.50000000	3.68750000	0.16441838
2187	3.50000000	3.81250000	0.17008798
2188	3.56250000	3.75000000	0.16070381
2189	3.56250000	3.81250000	0.17028348
2190	3.43750000	3.68750000	0.15092864
2191	3.43750000	3.75000000	0.16676442
2192	3.68750000	3.50000000	0.14447703
2193	3.81250000	3.50000000	0.13704790
2194	3.75000000	3.56250000	0.14115347
2195	3.81250000	3.56250000	0.14408602
2196	3.68750000	3.43750000	0.12043011
2197	3.75000000	3.43750000	0.12727273
2198	3.31250000	3.50000000	0.13489736
2199	3.18750000	3.50000000	0.12160313
2200	3.25000000	3.43750000	0.08465298
2201	3.18750000	3.43750000	0.08465298
2202	3.31250000	3.56250000	0.13157380
2203	3.25000000	3.56250000	0.15014663
2204	3.50000000	3.31250000	0.10244379
2205	3.50000000	3.18750000	0.06744868
2206	3.43750000	3.25000000	0.05219941
2207	3.43750000	3.18750000	0.04125122
2208	3.56250000	3.31250000	0.09247312
2209	3.56250000	3.25000000	0.08191593
2210	3.31250000	3.81250000	0.18866080
2211	3.18750000	3.68750000	0.18162268
2212	3.25000000	3.81250000	0.18846530
2213	3.18750000	3.75000000	0.17869013
2214	3.31250000	3.75000000	0.17908113
2215	3.25000000	3.68750000	0.18181818
2216	3.81250000	3.31250000	0.10498534
2217	3.68750000	3.18750000	-0.02267840
2218	3.81250000	3.25000000	0.09169110
2219	3.75000000	3.18750000	0.07702835
2220	3.75000000	3.31250000	0.10537634
2221	3.68750000	3.25000000	0.08797654
2222	0.18750000	1.68750000	-0.16891496
2223	0.31250000	1.81250000	-0.14467253
2224	0.18750000	1.75000000	-0.14154448
2225	0.25000000	1.81250000	-0.16676442
2226	0.25000000	1.68750000	-0.15972630
2227	0.31250000	1.75000000	-0.15992180
2228	0.18750000	1.50000000	-0.12727273
2229	0.31250000	1.50000000	-0.18377322
2230	0.18750000	1.43750000	-0.16480938
2231	0.25000000	1.43750000	-0.16774194
2232	0.25000000	1.56250000	-0.16539589
2233	0.31250000	1.56250000	-0.18103617
2234	0.18750000	2.50000000	-0.05591398
2235	0.31250000	2.50000000	-0.08132942
2236	0.18750000	2.43750000	-0.07292278
2237	0.25000000	2.43750000	-0.08914956
2238	0.25000000	2.56250000	-0.05767351
2239	0.31250000	2.56250000	-0.08817204

2240	0.18750000	2.68750000	0.02737048
2241	0.31250000	2.81250000	0.03870968
2242	0.18750000	2.75000000	0.03421310
2243	0.25000000	2.81250000	0.04086022
2244	0.25000000	2.68750000	0.00645161
2245	0.31250000	2.75000000	0.01955034
2246	0.50000000	2.18750000	-0.14916911
2247	0.50000000	2.31250000	-0.12942326
2248	0.43750000	2.18750000	-0.13391984
2249	0.43750000	2.25000000	-0.12531769
2250	0.56250000	2.25000000	-0.11847507
2251	0.56250000	2.31250000	-0.12805474
2252	0.50000000	1.81250000	-0.15366569
2253	0.50000000	1.68750000	-0.18455523
2254	0.56250000	1.81250000	-0.18181818
2255	0.56250000	1.75000000	-0.18475073
2256	0.43750000	1.75000000	-0.16598240
2257	0.43750000	1.68750000	-0.18142717
2258	0.68750000	2.18750000	-0.17165200
2259	0.81250000	2.31250000	-0.17282502
2260	0.75000000	2.18750000	-0.17478006
2261	0.81250000	2.25000000	-0.17556207
2262	0.68750000	2.25000000	-0.13763441
2263	0.75000000	2.31250000	-0.16304985
2264	0.50000000	2.68750000	-0.03851417
2265	0.50000000	2.81250000	-0.00156403
2266	0.56250000	2.75000000	-0.03401760
2267	0.56250000	2.81250000	-0.01505376
2268	0.43750000	2.68750000	-0.05122190
2269	0.43750000	2.75000000	-0.00762463
2270	0.68750000	2.50000000	-0.12473118
2271	0.81250000	2.50000000	-0.14545455
2272	0.75000000	2.56250000	-0.12023460
2273	0.81250000	2.56250000	-0.13000978
2274	0.68750000	2.43750000	-0.12903226
2275	0.75000000	2.43750000	-0.14486804
2276	1.68750000	0.18750000	0.11945259
2277	1.81250000	0.31250000	0.08504399
2278	1.75000000	0.18750000	0.12179863
2279	1.81250000	0.25000000	0.11104594
2280	1.68750000	0.25000000	0.09188661
2281	1.75000000	0.31250000	0.08093842
2282	1.50000000	0.18750000	0.06823069
2283	1.50000000	0.31250000	0.06295210
2284	1.43750000	0.18750000	0.04222874
2285	1.43750000	0.25000000	0.01446725
2286	1.56250000	0.25000000	0.05767351
2287	1.56250000	0.31250000	0.02971652
2288	2.50000000	0.18750000	0.09833822
2289	2.50000000	0.31250000	0.05865103
2290	2.43750000	0.18750000	0.09990225
2291	2.43750000	0.25000000	0.07996090
2292	2.56250000	0.25000000	0.06138807
2293	2.56250000	0.31250000	0.04144673
2294	2.68750000	0.18750000	0.04692082
2295	2.81250000	0.31250000	-0.02697947
2296	2.75000000	0.18750000	0.02365591
2297	2.81250000	0.25000000	-0.01681329
2298	2.68750000	0.25000000	0.02717498

2299	2.75000000	0.31250000	-0.00977517
2300	2.18750000	0.50000000	0.02697947
2301	2.31250000	0.50000000	0.02346041
2302	2.18750000	0.43750000	0.05298143
2303	2.25000000	0.43750000	0.04477028
2304	2.25000000	0.56250000	0.01173021
2305	2.31250000	0.56250000	0.00997067
2306	1.81250000	0.50000000	0.00117302
2307	1.68750000	0.50000000	0.03049853
2308	1.81250000	0.56250000	-0.00312805
2309	1.75000000	0.56250000	-0.01974585
2310	1.75000000	0.43750000	0.02502444
2311	1.68750000	0.43750000	0.00801564
2312	2.18750000	0.68750000	-0.05728250
2313	2.31250000	0.81250000	-0.06275660
2314	2.18750000	0.75000000	-0.03655914
2315	2.25000000	0.81250000	-0.05786901
2316	2.25000000	0.68750000	-0.02776149
2317	2.31250000	0.75000000	-0.04926686
2318	2.68750000	0.50000000	-0.05239492
2319	2.81250000	0.50000000	-0.08660802
2320	2.75000000	0.56250000	-0.07702835
2321	2.81250000	0.56250000	-0.09384164
2322	2.68750000	0.43750000	-0.03245357
2323	2.75000000	0.43750000	-0.04946237
2324	2.50000000	0.68750000	-0.04105572
2325	2.50000000	0.81250000	-0.06803519
2326	2.56250000	0.75000000	-0.06568915
2327	2.56250000	0.81250000	-0.07898338
2328	2.43750000	0.68750000	-0.03304008
2329	2.43750000	0.75000000	-0.05278592
2330	1.50000000	1.68750000	-0.13176931
2331	1.50000000	1.81250000	-0.09990225
2332	1.56250000	1.75000000	-0.12003910
2333	1.56250000	1.81250000	-0.11652004
2334	1.43750000	1.68750000	-0.13900293
2335	1.43750000	1.75000000	-0.13880743
2336	1.68750000	1.50000000	-0.11065494
2337	1.81250000	1.50000000	-0.09012708
2338	1.75000000	1.56250000	-0.09892473
2339	1.81250000	1.56250000	-0.08856305
2340	1.68750000	1.43750000	-0.06021505
2341	1.75000000	1.43750000	-0.09970674
2342	1.31250000	1.50000000	-0.16265885
2343	1.18750000	1.50000000	-0.17673509
2344	1.25000000	1.43750000	-0.17008798
2345	1.18750000	1.43750000	-0.17693060
2346	1.31250000	1.56250000	-0.15953079
2347	1.25000000	1.56250000	-0.16950147
2348	1.50000000	1.31250000	0.01055718
2349	1.50000000	1.18750000	0.01192571
2350	1.43750000	1.25000000	-0.00938416
2351	1.43750000	1.18750000	0.00391007
2352	1.56250000	1.31250000	0.02072336
2353	1.56250000	1.25000000	0.02170088
2354	2.31250000	2.50000000	0.02033236
2355	2.18750000	2.50000000	0.02482893
2356	2.25000000	2.43750000	0.01544477
2357	2.18750000	2.43750000	0.02385142

2358	2.31250000	2.56250000	0.03343109
2359	2.25000000	2.56250000	0.04183773
2360	2.50000000	2.31250000	-0.04066471
2361	2.50000000	2.18750000	-0.06451613
2362	2.43750000	2.25000000	-0.04555230
2363	2.43750000	2.18750000	-0.05786901
2364	2.56250000	2.31250000	-0.05219941
2365	2.56250000	2.25000000	-0.08914956
2366	2.50000000	2.68750000	0.03440860
2367	2.50000000	2.81250000	0.06060606
2368	2.56250000	2.75000000	0.01329423
2369	2.56250000	2.81250000	0.02619746
2370	2.43750000	2.68750000	0.04281525
2371	2.43750000	2.75000000	0.05591398
2372	2.68750000	2.50000000	-0.03968719
2373	2.81250000	2.50000000	-0.06314761
2374	2.75000000	2.56250000	-0.06099707
2375	2.81250000	2.56250000	-0.07272727
2376	2.68750000	2.43750000	-0.05161290
2377	2.75000000	2.43750000	-0.08836755
2378	1.68750000	2.18750000	-0.02306940
2379	1.81250000	2.31250000	-0.00410557
2380	1.75000000	2.18750000	-0.03968719
2381	1.81250000	2.25000000	-0.01739980
2382	1.68750000	2.25000000	-0.04340176
2383	1.75000000	2.31250000	-0.02111437
2384	1.31250000	1.81250000	-0.12981427
2385	1.18750000	1.68750000	-0.17575758
2386	1.25000000	1.81250000	-0.16539589
2387	1.18750000	1.75000000	-0.17575758
2388	1.31250000	1.75000000	-0.15874878
2389	1.25000000	1.68750000	-0.16891496
2390	1.50000000	2.18750000	-0.07448680
2391	1.50000000	2.31250000	-0.08973607
2392	1.43750000	2.18750000	-0.10987292
2393	1.43750000	2.25000000	-0.10478983
2394	1.56250000	2.25000000	-0.07761486
2395	1.56250000	2.31250000	-0.07253177
2396	2.18750000	1.68750000	-0.08484848
2397	2.31250000	1.81250000	-0.09521017
2398	2.18750000	1.75000000	-0.08328446
2399	2.25000000	1.81250000	-0.08875855
2400	2.25000000	1.68750000	-0.09286413
2401	2.31250000	1.75000000	-0.09696970
2402	1.81250000	1.31250000	-0.08758553
2403	1.68750000	1.18750000	-0.10557185
2404	1.81250000	1.25000000	-0.09071359
2405	1.75000000	1.18750000	-0.09853372
2406	1.75000000	1.31250000	-0.09794721
2407	1.68750000	1.25000000	-0.05747801
2408	2.18750000	1.50000000	-0.08250244
2409	2.31250000	1.50000000	-0.09853372
2410	2.18750000	1.43750000	-0.08563050
2411	2.25000000	1.43750000	-0.04183773
2412	2.25000000	1.56250000	-0.09130010
2413	2.31250000	1.56250000	-0.09442815
2414	2.18750000	2.68750000	0.07663734
2415	2.31250000	2.81250000	0.08582600
2416	2.18750000	2.75000000	0.09051808

2417	2.25000000	2.81250000	0.09501466
2418	2.25000000	2.68750000	0.06803519
2419	2.31250000	2.75000000	0.07272727
2420	1.81250000	2.50000000	0.01133920
2421	1.68750000	2.50000000	-0.02287390
2422	1.81250000	2.56250000	0.02541544
2423	1.75000000	2.56250000	0.00821114
2424	1.75000000	2.43750000	-0.01075269
2425	1.68750000	2.43750000	-0.02795699
2426	2.68750000	2.18750000	-0.17282502
2427	2.81250000	2.31250000	-0.17399804
2428	2.75000000	2.18750000	-0.19921799
2429	2.81250000	2.25000000	-0.18690127
2430	2.68750000	2.25000000	-0.16246334
2431	2.75000000	2.31250000	-0.16226784
2432	2.50000000	1.81250000	-0.05493646
2433	2.50000000	1.68750000	-0.11065494
2434	2.56250000	1.81250000	-0.12981427
2435	2.56250000	1.75000000	-0.12023460
2436	2.43750000	1.75000000	-0.10342131
2437	2.43750000	1.68750000	-0.10244379
2438	1.50000000	3.81250000	0.00293255
2439	1.50000000	3.68750000	0.07174976
2440	1.56250000	3.81250000	0.02658847
2441	1.56250000	3.75000000	0.06099707
2442	1.43750000	3.75000000	0.02482893
2443	1.43750000	3.68750000	0.05923754
2444	1.31250000	3.81250000	-0.03460411
2445	1.18750000	3.68750000	0.00899316
2446	1.25000000	3.81250000	0.00293255
2447	1.18750000	3.75000000	0.02482893
2448	1.31250000	3.75000000	-0.00019550
2449	1.25000000	3.68750000	0.02170088
2450	2.31250000	3.81250000	-0.05278592
2451	2.18750000	3.68750000	0.04320626
2452	2.25000000	3.81250000	-0.01700880
2453	2.18750000	3.75000000	0.03088954
2454	2.31250000	3.75000000	-0.00312805
2455	2.25000000	3.68750000	0.04477028
2456	2.50000000	3.81250000	-0.04789834
2457	2.50000000	3.68750000	0.05141740
2458	2.56250000	3.81250000	0.00156403
2459	2.56250000	3.75000000	0.05102639
2460	2.43750000	3.75000000	0.00019550
2461	2.43750000	3.68750000	0.04985337
2462	1.68750000	3.50000000	0.17966764
2463	1.81250000	3.50000000	0.18279570
2464	1.75000000	3.56250000	0.16891496
2465	1.81250000	3.56250000	0.17047898
2466	1.68750000	3.43750000	0.17771261
2467	1.75000000	3.43750000	0.17927664
2468	1.31250000	3.50000000	0.13743891
2469	1.18750000	3.50000000	0.11221896
2470	1.25000000	3.43750000	0.10928641
2471	1.18750000	3.43750000	0.09677419
2472	1.31250000	3.56250000	0.10303030
2473	1.25000000	3.56250000	0.09051808
2474	1.50000000	3.31250000	0.17047898
2475	1.50000000	3.18750000	0.16480938

2476	1.43750000	3.25000000	0.14076246
2477	1.43750000	3.18750000	0.14017595
2478	1.56250000	3.31250000	0.17047898
2479	1.56250000	3.25000000	0.16832845
2480	2.18750000	3.50000000	0.19217986
2481	2.31250000	3.50000000	0.19530792
2482	2.18750000	3.43750000	0.19042033
2483	2.25000000	3.43750000	0.19198436
2484	2.25000000	3.56250000	0.14408602
2485	2.31250000	3.56250000	0.14584555
2486	1.81250000	3.31250000	0.17653959
2487	1.68750000	3.18750000	0.16950147
2488	1.81250000	3.25000000	0.17497556
2489	1.75000000	3.18750000	0.17145650
2490	1.75000000	3.31250000	0.17517107
2491	1.68750000	3.25000000	0.17145650
2492	3.81250000	1.50000000	-0.09032258
2493	3.68750000	1.50000000	-0.12355816
2494	3.81250000	1.56250000	-0.10029326
2495	3.75000000	1.56250000	-0.11652004
2496	3.75000000	1.43750000	-0.10576735
2497	3.68750000	1.43750000	-0.12825024
2498	3.81250000	1.31250000	-0.08680352
2499	3.68750000	1.18750000	-0.11769306
2500	3.81250000	1.25000000	-0.09501466
2501	3.75000000	1.18750000	-0.11045943
2502	3.75000000	1.31250000	-0.10342131
2503	3.68750000	1.25000000	-0.12043011
2504	3.81250000	2.31250000	-0.09560117
2505	3.68750000	2.18750000	-0.12218964
2506	3.81250000	2.25000000	-0.10381232
2507	3.75000000	2.18750000	-0.11710655
2508	3.75000000	2.31250000	-0.15112414
2509	3.68750000	2.25000000	-0.10733138
2510	3.81250000	2.50000000	-0.06979472
2511	3.68750000	2.50000000	-0.07917889
2512	3.81250000	2.56250000	-0.05806452
2513	3.75000000	2.56250000	-0.06275660
2514	3.75000000	2.43750000	-0.13391984
2515	3.68750000	2.43750000	0.05610948
2516	3.50000000	1.68750000	-0.16930596
2517	3.50000000	1.81250000	-0.16656891
2518	3.56250000	1.75000000	-0.16226784
2519	3.56250000	1.81250000	-0.16129032
2520	3.43750000	1.68750000	-0.17086999
2521	3.43750000	1.75000000	-0.16950147
2522	3.50000000	1.31250000	-0.17380254
2523	3.50000000	1.18750000	-0.17145650
2524	3.43750000	1.25000000	-0.17653959
2525	3.43750000	1.18750000	-0.17536657
2526	3.56250000	1.31250000	-0.16168133
2527	3.56250000	1.25000000	-0.15620723
2528	3.31250000	1.50000000	-0.17810362
2529	3.18750000	1.50000000	-0.18123167
2530	3.25000000	1.43750000	-0.18084066
2531	3.18750000	1.43750000	-0.18260020
2532	3.31250000	1.56250000	-0.17673509
2533	3.25000000	1.56250000	-0.17829912
2534	3.50000000	2.18750000	-0.13235582

2535	3.50000000	2.31250000	-0.11827957
2536	3.43750000	2.18750000	-0.14037146
2537	3.43750000	2.25000000	-0.13157380
2538	3.56250000	2.25000000	-0.11593353
2539	3.56250000	2.31250000	0.03030303
2540	3.31250000	1.81250000	-0.17106549
2541	3.18750000	1.68750000	-0.17673509
2542	3.25000000	1.81250000	-0.17575758
2543	3.18750000	1.75000000	-0.17869013
2544	3.31250000	1.75000000	-0.17243402
2545	3.25000000	1.68750000	-0.17536657
2546	0.50000000	1.18750000	-0.19433040
2547	0.50000000	1.31250000	-0.19374389
2548	0.43750000	1.18750000	-0.17810362
2549	0.43750000	1.25000000	-0.17771261
2550	0.56250000	1.25000000	-0.19472141
2551	0.56250000	1.31250000	-0.19433040
2552	0.68750000	1.18750000	-0.19589443
2553	0.81250000	1.31250000	-0.19628543
2554	0.75000000	1.18750000	-0.19648094
2555	0.81250000	1.25000000	-0.19667644
2556	0.68750000	1.25000000	-0.19569892
2557	0.75000000	1.31250000	-0.19589443
2558	0.68750000	1.50000000	-0.19472141
2559	0.81250000	1.50000000	-0.19569892
2560	0.75000000	1.56250000	-0.19491691
2561	0.81250000	1.56250000	-0.19569892
2562	0.68750000	1.43750000	-0.19491691
2563	0.75000000	1.43750000	-0.19550342
2564	1.18750000	0.50000000	-0.11026393
2565	1.31250000	0.50000000	-0.03147605
2566	1.18750000	0.43750000	-0.10048876
2567	1.25000000	0.43750000	-0.09247312
2568	1.25000000	0.56250000	-0.11045943
2569	1.31250000	0.56250000	-0.10224829
2570	1.18750000	0.68750000	-0.13489736
2571	1.31250000	0.81250000	-0.13528837
2572	1.18750000	0.75000000	-0.14310850
2573	1.25000000	0.81250000	-0.14330401
2574	1.25000000	0.68750000	-0.12688172
2575	1.31250000	0.75000000	-0.12688172
2576	1.50000000	0.68750000	-0.04437928
2577	1.50000000	0.81250000	-0.10772239
2578	1.56250000	0.75000000	-0.09462366
2579	1.56250000	0.81250000	-0.10263930
2580	1.43750000	0.68750000	-0.10244379
2581	1.43750000	0.75000000	-0.11065494
2582	0.50000000	3.31250000	0.13431085
2583	0.50000000	3.18750000	0.10303030
2584	0.43750000	3.25000000	0.12023460
2585	0.43750000	3.18750000	0.10459433
2586	0.56250000	3.31250000	0.12101662
2587	0.56250000	3.25000000	0.10518084
2588	0.68750000	3.50000000	0.14134897
2589	0.81250000	3.50000000	0.11476051
2590	0.75000000	3.56250000	0.14369501
2591	0.81250000	3.56250000	0.13040078
2592	0.68750000	3.43750000	0.12570870
2593	0.75000000	3.43750000	0.11241447

2594	0.68750000	3.18750000	0.06295210
2595	0.81250000	3.31250000	0.06744868
2596	0.75000000	3.18750000	0.04946237
2597	0.81250000	3.25000000	0.05180841
2598	0.68750000	3.25000000	0.07839687
2599	0.75000000	3.31250000	0.08093842
2600	3.31250000	0.50000000	-0.11788856
2601	3.18750000	0.50000000	-0.12590420
2602	3.25000000	0.43750000	-0.10830890
2603	3.18750000	0.43750000	-0.11554252
2604	3.31250000	0.56250000	-0.12512219
2605	3.25000000	0.56250000	-0.12922776
2606	3.50000000	0.68750000	-0.12766373
2607	3.50000000	0.81250000	-0.14252199
2608	3.56250000	0.75000000	-0.12785924
2609	3.56250000	0.81250000	-0.13528837
2610	3.43750000	0.68750000	-0.13176931
2611	3.43750000	0.75000000	-0.13919844
2612	3.18750000	0.68750000	-0.14819159
2613	3.31250000	0.81250000	-0.16715543
2614	3.18750000	0.75000000	-0.15542522
2615	3.25000000	0.81250000	-0.15874878
2616	3.25000000	0.68750000	-0.14408602
2617	3.31250000	0.75000000	-0.14740958
2618	2.81250000	3.31250000	0.11319648
2619	2.68750000	3.18750000	0.10772239
2620	2.81250000	3.25000000	0.07624633
2621	2.75000000	3.18750000	0.07350929
2622	2.75000000	3.31250000	0.12297165
2623	2.68750000	3.25000000	0.12023460
2624	2.81250000	3.50000000	0.15073314
2625	2.68750000	3.50000000	0.17028348
2626	2.81250000	3.56250000	0.16031281
2627	2.75000000	3.56250000	0.17008798
2628	2.75000000	3.43750000	0.14799609
2629	2.68750000	3.43750000	0.15777126
2630	3.31250000	2.81250000	-0.01524927
2631	3.18750000	2.68750000	-0.06099707
2632	3.25000000	2.81250000	-0.04144673
2633	3.18750000	2.75000000	-0.05806452
2634	3.31250000	2.75000000	-0.03108504
2635	3.25000000	2.68750000	-0.04770283
2636	3.50000000	2.81250000	-0.01309873
2637	3.50000000	2.68750000	-0.04516129
2638	3.56250000	2.81250000	-0.01270772
2639	3.56250000	2.75000000	-0.02873900
2640	3.43750000	2.75000000	-0.02971652
2641	3.43750000	2.68750000	-0.04574780
2642	2.50000000	3.18750000	0.13704790
2643	2.50000000	3.31250000	0.16226784
2644	2.43750000	3.18750000	0.14643206
2645	2.43750000	3.25000000	0.15894428
2646	2.56250000	3.25000000	0.13978495
2647	2.56250000	3.31250000	0.15229717
2648	3.18750000	2.50000000	-0.09540567
2649	3.31250000	2.50000000	-0.09501466
2650	3.18750000	2.43750000	-0.10909091
2651	3.25000000	2.43750000	-0.10830890
2652	3.25000000	2.56250000	-0.07956989

2653	3.31250000	2.56250000	-0.07898338
2654	1.18750000	2.50000000	-0.13704790
2655	1.31250000	2.50000000	-0.11202346
2656	1.18750000	2.43750000	-0.13958944
2657	1.25000000	2.43750000	-0.12707722
2658	1.25000000	2.56250000	-0.09540567
2659	1.31250000	2.56250000	-0.08289345
2660	1.18750000	2.68750000	-0.04965787
2661	1.31250000	2.81250000	0.03362659
2662	1.18750000	2.75000000	-0.03401760
2663	1.25000000	2.81250000	0.00918866
2664	1.25000000	2.68750000	-0.03714565
2665	1.31250000	2.75000000	0.00449658
2666	1.50000000	2.68750000	0.01290323
2667	1.50000000	2.81250000	0.07116325
2668	1.56250000	2.75000000	0.04398827
2669	1.56250000	2.81250000	0.07311828
2670	1.43750000	2.68750000	0.00039101
2671	1.43750000	2.75000000	0.02952102
2672	2.50000000	1.18750000	-0.11378299
2673	2.50000000	1.31250000	-0.11730205
2674	2.43750000	1.18750000	-0.10889541
2675	2.43750000	1.25000000	-0.06060606
2676	2.56250000	1.25000000	-0.12336266
2677	2.56250000	1.31250000	-0.12512219
2678	2.68750000	1.18750000	-0.13724340
2679	2.81250000	1.31250000	-0.15659824
2680	2.75000000	1.18750000	-0.14838710
2681	2.81250000	1.25000000	-0.15796676
2682	2.68750000	1.25000000	-0.13900293
2683	2.75000000	1.31250000	-0.14858260
2684	2.68750000	1.50000000	-0.14604106
2685	2.81250000	1.50000000	-0.16168133
2686	2.75000000	1.56250000	-0.15581623
2687	2.81250000	1.56250000	-0.16363636
2688	2.68750000	1.43750000	-0.14428152
2689	2.75000000	1.43750000	-0.15210166
2690	0.06250000	0.12500000	0.08191593
2691	0.06250000	0.18750000	0.05689150
2692	0.12500000	0.06250000	0.09286413
2693	0.18750000	0.06250000	0.07878788
2694	0.12500000	0.18750000	0.04242424
2695	0.18750000	0.12500000	0.05337243
2696	0.06250000	3.93750000	0.19178886
2697	0.06250000	3.87500000	0.18983382
2698	0.12500000	3.93750000	0.19217986
2699	3.93750000	0.06250000	0.14252199
2700	3.87500000	0.06250000	0.12629521
2701	3.93750000	0.12500000	0.12160313
2702	3.87500000	3.93750000	0.19042033
2703	3.93750000	3.87500000	0.18377322
2704	3.81250000	3.87500000	0.18064516
2705	3.87500000	3.81250000	0.17419355
2706	3.81250000	3.93750000	0.19022483
2707	3.93750000	3.81250000	0.17712610
2708	0.06250000	1.93750000	-0.15288368
2709	0.06250000	1.87500000	-0.15562072
2710	0.06250000	2.12500000	-0.12179863
2711	0.06250000	2.18750000	-0.10224829

2712	0.12500000	2.06250000	-0.14408602
2713	0.12500000	1.93750000	-0.15581623
2714	0.18750000	2.06250000	-0.14682307
2715	0.12500000	2.18750000	-0.11554252
2716	0.18750000	2.12500000	-0.13782991
2717	1.93750000	0.06250000	0.15483871
2718	1.87500000	0.06250000	0.15249267
2719	2.12500000	0.06250000	0.14799609
2720	2.18750000	0.06250000	0.14643206
2721	2.06250000	0.12500000	0.13567937
2722	1.93750000	0.12500000	0.14134897
2723	2.06250000	0.18750000	0.12238514
2724	2.18750000	0.12500000	0.12609971
2725	2.12500000	0.18750000	0.11436950
2726	1.87500000	1.93750000	-0.06568915
2727	1.93750000	1.87500000	-0.05865103
2728	1.81250000	1.87500000	-0.07781036
2729	1.87500000	1.81250000	-0.07096774
2730	2.06250000	2.12500000	-0.02619746
2731	2.12500000	2.06250000	-0.04711632
2732	2.12500000	2.18750000	-0.02111437
2733	2.18750000	2.12500000	-0.04203324
2734	1.93750000	2.06250000	-0.04007820
2735	1.81250000	1.93750000	-0.07448680
2736	1.87500000	2.06250000	-0.04887586
2737	2.06250000	1.93750000	-0.05395894
2738	1.93750000	1.81250000	-0.06060606
2739	2.06250000	1.87500000	-0.05552297
2740	2.06250000	2.18750000	-0.01270772
2741	1.93750000	2.12500000	-0.02658847
2742	2.18750000	2.06250000	-0.05454545
2743	2.12500000	1.93750000	-0.06119257
2744	1.87500000	3.93750000	0.07605083
2745	1.81250000	3.93750000	0.05239492
2746	2.06250000	3.93750000	0.06568915
2747	2.12500000	3.93750000	0.03010753
2748	1.93750000	3.87500000	0.11202346
2749	1.81250000	3.87500000	0.08680352
2750	1.87500000	3.81250000	0.12277615
2751	2.06250000	3.87500000	0.07800587
2752	1.93750000	3.81250000	0.12434018
2753	3.93750000	1.87500000	-0.11710655
2754	3.93750000	1.81250000	-0.10694037
2755	3.93750000	2.06250000	-0.11827957
2756	3.93750000	2.12500000	-0.11006843
2757	3.87500000	1.93750000	-0.13235582
2758	3.87500000	1.81250000	-0.12297165
2759	3.81250000	1.87500000	-0.13822092
2760	3.87500000	2.06250000	-0.12336266
2761	3.81250000	1.93750000	-0.13743891
2762	0.06250000	0.93750000	-0.07839687
2763	0.06250000	0.87500000	-0.07585533
2764	0.06250000	1.12500000	-0.09364614
2765	0.06250000	1.18750000	-0.10654936
2766	0.12500000	1.06250000	-0.09696970
2767	0.12500000	0.93750000	-0.09462366
2768	0.18750000	1.06250000	-0.11319648
2769	0.12500000	1.18750000	-0.10967742
2770	0.18750000	1.12500000	-0.11300098

2771	0.93750000	0.06250000	-0.05063539
2772	0.87500000	0.06250000	-0.03538612
2773	1.12500000	0.06250000	-0.03206256
2774	1.18750000	0.06250000	-0.00606061
2775	1.06250000	0.12500000	-0.06764418
2776	0.93750000	0.12500000	-0.06041056
2777	1.06250000	0.18750000	-0.07741935
2778	1.18750000	0.12500000	-0.03362659
2779	1.12500000	0.18750000	-0.06940371
2780	0.87500000	0.93750000	-0.19139785
2781	0.93750000	0.87500000	-0.18377322
2782	0.81250000	0.87500000	-0.18338221
2783	0.87500000	0.81250000	-0.17575758
2784	1.06250000	1.12500000	-0.19237537
2785	1.12500000	1.06250000	-0.18475073
2786	1.12500000	1.18750000	-0.18514174
2787	1.18750000	1.12500000	-0.17751711
2788	0.93750000	1.06250000	-0.19902248
2789	0.81250000	0.93750000	-0.19081134
2790	0.87500000	1.06250000	-0.19843597
2791	1.06250000	0.93750000	-0.18377322
2792	0.93750000	0.81250000	-0.17556207
2793	1.06250000	0.87500000	-0.17556207
2794	1.06250000	1.18750000	-0.19198436
2795	0.93750000	1.12500000	-0.19863148
2796	1.18750000	1.06250000	-0.17673509
2797	1.12500000	0.93750000	-0.17575758
2798	0.06250000	3.12500000	0.09677419
2799	0.06250000	3.18750000	0.11045943
2800	0.06250000	2.93750000	0.05962854
2801	0.06250000	2.87500000	0.05200391
2802	0.12500000	3.06250000	0.08152493
2803	0.12500000	3.18750000	0.11085044
2804	0.18750000	3.12500000	0.09540567
2805	0.12500000	2.93750000	0.05826002
2806	0.18750000	3.06250000	0.07976540
2807	0.87500000	3.93750000	0.19706745
2808	0.81250000	3.93750000	0.19667644
2809	1.06250000	3.93750000	0.14662757
2810	1.12500000	3.93750000	0.10928641
2811	0.93750000	3.87500000	0.18181818
2812	0.81250000	3.87500000	0.19491691
2813	0.87500000	3.81250000	0.17966764
2814	1.06250000	3.87500000	0.13098729
2815	0.93750000	3.81250000	0.16617791
2816	3.12500000	0.06250000	-0.02932551
2817	3.18750000	0.06250000	-0.01935484
2818	2.93750000	0.06250000	-0.02287390
2819	2.87500000	0.06250000	0.00058651
2820	3.06250000	0.12500000	-0.05591398
2821	3.18750000	0.12500000	-0.03870968
2822	3.12500000	0.18750000	-0.06549365
2823	2.93750000	0.12500000	-0.03968719
2824	3.06250000	0.18750000	-0.07272727
2825	3.93750000	0.87500000	-0.09188661
2826	3.93750000	0.81250000	-0.07702835
2827	3.93750000	1.06250000	-0.10557185
2828	3.93750000	1.12500000	-0.09736070
2829	3.87500000	0.93750000	-0.11378299

2830	3.87500000	0.81250000	-0.09169110
2831	3.81250000	0.87500000	-0.11358749
2832	3.87500000	1.06250000	-0.11280547
2833	3.81250000	0.93750000	-0.12082111
2834	3.06250000	3.12500000	-0.10068426
2835	3.12500000	3.06250000	-0.11163245
2836	3.12500000	3.18750000	-0.06373412
2837	3.18750000	3.12500000	-0.07448680
2838	2.87500000	2.93750000	-0.11925709
2839	2.93750000	2.87500000	-0.14408602
2840	2.81250000	2.87500000	-0.09794721
2841	2.87500000	2.81250000	-0.12277615
2842	2.93750000	3.06250000	-0.10342131
2843	3.06250000	3.18750000	-0.06373412
2844	2.93750000	3.12500000	-0.06647116
2845	3.06250000	2.93750000	-0.13919844
2846	3.18750000	3.06250000	-0.08543500
2847	3.12500000	2.93750000	-0.11300098
2848	2.81250000	2.93750000	-0.08484848
2849	2.87500000	3.06250000	-0.06920821
2850	2.93750000	2.81250000	-0.13450635
2851	3.06250000	2.87500000	-0.12961877
2852	3.06250000	3.93750000	0.18826979
2853	3.12500000	3.93750000	0.18846530
2854	2.87500000	3.93750000	0.14877810
2855	2.81250000	3.93750000	0.09951124
2856	2.93750000	3.87500000	0.18846530
2857	3.06250000	3.87500000	0.17869013
2858	2.93750000	3.81250000	0.17888563
2859	2.81250000	3.87500000	0.14916911
2860	2.87500000	3.81250000	0.18885630
2861	3.93750000	3.06250000	0.06275660
2862	3.93750000	3.12500000	0.07722385
2863	3.93750000	2.87500000	0.00918866
2864	3.93750000	2.81250000	-0.00254154
2865	3.87500000	2.93750000	0.02033236
2866	3.87500000	3.06250000	0.04985337
2867	3.81250000	2.93750000	0.01994135
2868	3.87500000	2.81250000	-0.00645161
2869	3.81250000	2.87500000	0.00488759
2870	0.87500000	1.93750000	-0.19217986
2871	0.93750000	2.06250000	-0.18983382
2872	0.81250000	1.93750000	-0.18885630
2873	0.87500000	2.06250000	-0.18670577
2874	1.06250000	1.93750000	-0.18572825
2875	1.12500000	2.06250000	-0.17028348
2876	1.12500000	1.93750000	-0.17556207
2877	1.18750000	2.06250000	-0.16031281
2878	0.93750000	1.87500000	-0.19550342
2879	0.81250000	1.87500000	-0.19159335
2880	0.87500000	1.81250000	-0.19511241
2881	1.06250000	1.87500000	-0.18592375
2882	0.93750000	1.81250000	-0.19569892
2883	1.06250000	2.12500000	-0.17771261
2884	0.93750000	2.12500000	-0.18709677
2885	1.06250000	2.18750000	-0.17517107
2886	1.18750000	2.12500000	-0.15522972
2887	1.12500000	2.18750000	-0.16265885
2888	0.87500000	2.93750000	-0.04281525

2889	0.93750000	2.87500000	-0.07174976
2890	0.81250000	2.87500000	-0.04868035
2891	0.87500000	2.81250000	-0.07741935
2892	1.06250000	3.12500000	-0.00664712
2893	1.12500000	3.06250000	0.00547410
2894	1.12500000	3.18750000	0.02170088
2895	1.18750000	3.12500000	0.03382209
2896	0.93750000	3.06250000	-0.02150538
2897	0.81250000	2.93750000	-0.02952102
2898	0.87500000	3.06250000	-0.00801564
2899	1.06250000	3.18750000	0.00899316
2900	0.93750000	3.12500000	-0.00586510
2901	1.06250000	2.93750000	-0.03831867
2902	0.93750000	2.81250000	-0.08719453
2903	1.06250000	2.87500000	-0.05376344
2904	1.18750000	3.06250000	0.03323558
2905	1.12500000	2.93750000	-0.01055718
2906	1.93750000	0.87500000	-0.05376344
2907	2.06250000	0.93750000	-0.05865103
2908	1.93750000	0.81250000	-0.03714565
2909	2.06250000	0.87500000	-0.04183773
2910	1.93750000	1.06250000	-0.07878788
2911	2.06250000	1.12500000	-0.02717498
2912	1.93750000	1.12500000	-0.07898338
2913	2.06250000	1.18750000	-0.02717498
2914	1.87500000	0.93750000	-0.07839687
2915	1.87500000	0.81250000	-0.05356794
2916	1.81250000	0.87500000	-0.07839687
2917	1.87500000	1.06250000	-0.08680352
2918	1.81250000	0.93750000	-0.08660802
2919	2.12500000	1.06250000	-0.03167155
2920	2.12500000	0.93750000	-0.06314761
2921	2.18750000	1.06250000	-0.03636364
2922	2.12500000	1.18750000	0.01661779
2923	2.18750000	1.12500000	0.01192571
2924	2.93750000	0.87500000	-0.16480938
2925	2.87500000	0.93750000	-0.16129032
2926	2.87500000	0.81250000	-0.14076246
2927	2.81250000	0.87500000	-0.13704790
2928	3.12500000	1.06250000	-0.18944282
2929	3.06250000	1.12500000	-0.19217986
2930	3.18750000	1.12500000	-0.18670577
2931	3.12500000	1.18750000	-0.18924731
2932	3.06250000	0.93750000	-0.18494624
2933	2.93750000	0.81250000	-0.15757576
2934	3.06250000	0.87500000	-0.17771261
2935	3.18750000	1.06250000	-0.18553275
2936	3.12500000	0.93750000	-0.18103617
2937	2.93750000	1.06250000	-0.18416422
2938	2.81250000	0.93750000	-0.15034213
2939	2.87500000	1.06250000	-0.17321603
2940	3.06250000	1.18750000	-0.19081134
2941	2.93750000	1.12500000	-0.18279570
2942	2.06250000	2.93750000	0.14760508
2943	1.93750000	2.87500000	0.12570870
2944	2.06250000	2.87500000	0.13528837
2945	1.93750000	2.81250000	0.11339198
2946	2.06250000	3.12500000	0.17556207
2947	1.93750000	3.06250000	0.17067449

2948	2.06250000	3.18750000	0.17888563
2949	1.93750000	3.12500000	0.17380254
2950	2.12500000	3.06250000	0.16461388
2951	2.12500000	2.93750000	0.13998045
2952	2.18750000	3.06250000	0.15698925
2953	2.12500000	3.18750000	0.18025415
2954	2.18750000	3.12500000	0.16930596
2955	1.87500000	2.93750000	0.13782991
2956	1.87500000	2.81250000	0.09618768
2957	1.81250000	2.87500000	0.10830890
2958	1.87500000	3.06250000	0.17028348
2959	1.81250000	2.93750000	0.13763441
2960	2.93750000	2.06250000	-0.18748778
2961	2.87500000	1.93750000	-0.19042033
2962	2.87500000	2.06250000	-0.18748778
2963	2.81250000	1.93750000	-0.19022483
2964	3.12500000	2.06250000	-0.18181818
2965	3.06250000	1.93750000	-0.19354839
2966	3.18750000	2.06250000	-0.17732160
2967	3.12500000	1.93750000	-0.18885630
2968	3.06250000	2.12500000	-0.17399804
2969	2.93750000	2.12500000	-0.17497556
2970	3.06250000	2.18750000	-0.16148583
2971	3.18750000	2.12500000	-0.16852395
2972	3.12500000	2.18750000	-0.16050831
2973	2.93750000	1.87500000	-0.18866080
2974	2.81250000	1.87500000	-0.18064516
2975	2.87500000	1.81250000	-0.17888563
2976	3.06250000	1.87500000	-0.19178886
2977	2.93750000	1.81250000	-0.18670577
2978	0.06250000	0.43750000	-0.02756598
2979	0.06250000	0.37500000	-0.00449658
2980	0.06250000	0.62500000	-0.06608016
2981	0.06250000	0.68750000	-0.06823069
2982	0.12500000	0.56250000	-0.06901271
2983	0.12500000	0.43750000	-0.03264907
2984	0.18750000	0.56250000	-0.07390029
2985	0.12500000	0.68750000	-0.08445748
2986	0.18750000	0.62500000	-0.08719453
2987	0.43750000	0.06250000	0.04692082
2988	0.37500000	0.06250000	0.05278592
2989	0.62500000	0.06250000	0.02580645
2990	0.68750000	0.06250000	0.01055718
2991	0.56250000	0.12500000	0.01583578
2992	0.43750000	0.12500000	0.02170088
2993	0.56250000	0.18750000	-0.00938416
2994	0.68750000	0.12500000	0.00058651
2995	0.62500000	0.18750000	-0.00938416
2996	0.37500000	0.43750000	-0.08523949
2997	0.43750000	0.37500000	-0.07761486
2998	0.31250000	0.37500000	-0.05513196
2999	0.37500000	0.31250000	-0.04750733
3000	0.56250000	0.62500000	-0.14780059
3001	0.62500000	0.56250000	-0.14056696
3002	0.62500000	0.68750000	-0.15698925
3003	0.68750000	0.62500000	-0.14936461
3004	0.43750000	0.56250000	-0.12375367
3005	0.31250000	0.43750000	-0.06862170
3006	0.37500000	0.56250000	-0.10733138

3007	0.56250000	0.43750000	-0.10889541
3008	0.43750000	0.31250000	-0.05337243
3009	0.56250000	0.37500000	-0.08426197
3010	0.56250000	0.68750000	-0.15796676
3011	0.43750000	0.62500000	-0.13235582
3012	0.68750000	0.56250000	-0.14310850
3013	0.62500000	0.43750000	-0.10967742
3014	0.06250000	3.62500000	0.18201369
3015	0.06250000	3.68750000	0.18416422
3016	0.06250000	3.43750000	0.16461388
3017	0.06250000	3.37500000	0.15131965
3018	0.12500000	3.56250000	0.18044966
3019	0.12500000	3.68750000	0.18455523
3020	0.18750000	3.62500000	0.18299120
3021	0.12500000	3.43750000	0.16520039
3022	0.18750000	3.56250000	0.18103617
3023	0.37500000	3.93750000	0.19374389
3024	0.31250000	3.93750000	0.19354839
3025	0.56250000	3.93750000	0.19511241
3026	0.62500000	3.93750000	0.19550342
3027	0.43750000	3.87500000	0.19237537
3028	0.31250000	3.87500000	0.19159335
3029	0.37500000	3.81250000	0.19002933
3030	0.56250000	3.87500000	0.19335288
3031	0.43750000	3.81250000	0.19061584
3032	3.62500000	0.06250000	0.06119257
3033	3.68750000	0.06250000	0.07741935
3034	3.43750000	0.06250000	0.01720430
3035	3.37500000	0.06250000	0.01036168
3036	3.56250000	0.12500000	0.02248289
3037	3.68750000	0.12500000	0.05669599
3038	3.62500000	0.18750000	0.02111437
3039	3.43750000	0.12500000	-0.00234604
3040	3.56250000	0.18750000	0.00312805
3041	3.93750000	0.37500000	0.04418377
3042	3.93750000	0.31250000	0.06119257
3043	3.93750000	0.56250000	-0.01270772
3044	3.93750000	0.62500000	-0.03010753
3045	3.87500000	0.43750000	0.00860215
3046	3.87500000	0.31250000	0.04652981
3047	3.81250000	0.37500000	0.01094819
3048	3.87500000	0.56250000	-0.02971652
3049	3.81250000	0.43750000	-0.00997067
3050	3.56250000	3.62500000	0.16813294
3051	3.62500000	3.56250000	0.16129032
3052	3.62500000	3.68750000	0.16441838
3053	3.68750000	3.62500000	0.15796676
3054	3.37500000	3.43750000	0.11104594
3055	3.43750000	3.37500000	0.10029326
3056	3.31250000	3.37500000	0.06080156
3057	3.37500000	3.31250000	0.05004888
3058	3.43750000	3.56250000	0.15816227
3059	3.56250000	3.68750000	0.16461388
3060	3.43750000	3.62500000	0.15483871
3061	3.56250000	3.43750000	0.14056696
3062	3.68750000	3.56250000	0.15131965
3063	3.62500000	3.43750000	0.13059629
3064	3.31250000	3.43750000	0.09775171
3065	3.37500000	3.56250000	0.14467253

3066	3.43750000	3.31250000	0.07624633
3067	3.56250000	3.37500000	0.11652004
3068	3.56250000	3.93750000	0.18944282
3069	3.62500000	3.93750000	0.18963832
3070	3.37500000	3.93750000	0.19550342
3071	3.31250000	3.93750000	0.19530792
3072	3.43750000	3.87500000	0.18592375
3073	3.56250000	3.87500000	0.17986315
3074	3.43750000	3.81250000	0.17634409
3075	3.31250000	3.87500000	0.19198436
3076	3.37500000	3.81250000	0.18260020
3077	3.93750000	3.56250000	0.14956012
3078	3.93750000	3.62500000	0.15659824
3079	3.93750000	3.37500000	0.13157380
3080	3.93750000	3.31250000	0.11827957
3081	3.87500000	3.43750000	0.12961877
3082	3.87500000	3.56250000	0.14682307
3083	3.81250000	3.43750000	0.13000978
3084	3.87500000	3.31250000	0.10615836
3085	3.81250000	3.37500000	0.11984360
3086	0.06250000	1.62500000	-0.16539589
3087	0.06250000	1.68750000	-0.13802542
3088	0.06250000	1.43750000	-0.10772239
3089	0.06250000	1.37500000	-0.14525904
3090	0.12500000	1.56250000	-0.12101662
3091	0.12500000	1.68750000	-0.14095797
3092	0.18750000	1.62500000	-0.15913978
3093	0.12500000	1.43750000	-0.11124145
3094	0.18750000	1.56250000	-0.11182796
3095	0.06250000	2.43750000	-0.02326491
3096	0.06250000	2.37500000	-0.04027370
3097	0.06250000	2.62500000	0.04789834
3098	0.06250000	2.68750000	0.05474096
3099	0.12500000	2.56250000	0.00215054
3100	0.12500000	2.43750000	-0.03401760
3101	0.18750000	2.56250000	-0.01876833
3102	0.12500000	2.68750000	0.02815249
3103	0.18750000	2.62500000	0.00039101
3104	0.37500000	1.93750000	-0.14213099
3105	0.43750000	2.06250000	-0.16441838
3106	0.31250000	1.93750000	-0.16422287
3107	0.37500000	2.06250000	-0.14916911
3108	0.56250000	1.93750000	-0.15112414
3109	0.62500000	2.06250000	-0.17380254
3110	0.62500000	1.93750000	-0.17927664
3111	0.68750000	2.06250000	-0.17693060
3112	0.43750000	1.87500000	-0.12277615
3113	0.31250000	1.87500000	-0.14193548
3114	0.37500000	1.81250000	-0.13509286
3115	0.56250000	2.12500000	-0.16774194
3116	0.43750000	2.12500000	-0.14916911
3117	0.56250000	2.18750000	-0.13372434
3118	0.56250000	1.87500000	-0.15386119
3119	0.43750000	1.81250000	-0.13802542
3120	0.68750000	2.12500000	-0.17419355
3121	0.62500000	2.18750000	-0.13704790
3122	0.37500000	2.43750000	-0.08739003
3123	0.43750000	2.37500000	-0.11593353
3124	0.31250000	2.37500000	-0.09521017

3125	0.37500000	2.31250000	-0.11182796
3126	0.56250000	2.62500000	-0.06920821
3127	0.62500000	2.56250000	-0.09794721
3128	0.62500000	2.68750000	-0.06471163
3129	0.68750000	2.62500000	-0.09345064
3130	0.43750000	2.56250000	-0.06197458
3131	0.31250000	2.43750000	-0.07565982
3132	0.37500000	2.56250000	-0.07468231
3133	0.56250000	2.43750000	-0.11925709
3134	0.43750000	2.31250000	-0.12082111
3135	0.56250000	2.37500000	-0.12355816
3136	0.56250000	2.68750000	-0.05161290
3137	0.43750000	2.62500000	-0.06881720
3138	0.68750000	2.56250000	-0.10909091
3139	0.62500000	2.43750000	-0.11788856
3140	1.62500000	0.06250000	0.14310850
3141	1.68750000	0.06250000	0.14545455
3142	1.43750000	0.06250000	0.09775171
3143	1.37500000	0.06250000	0.07174976
3144	1.56250000	0.12500000	0.11300098
3145	1.68750000	0.12500000	0.13235582
3146	1.62500000	0.18750000	0.10244379
3147	1.43750000	0.12500000	0.06999022
3148	1.56250000	0.18750000	0.08543500
3149	2.43750000	0.06250000	0.13978495
3150	2.37500000	0.06250000	0.14134897
3151	2.62500000	0.06250000	0.09755621
3152	2.68750000	0.06250000	0.07429130
3153	2.56250000	0.12500000	0.10107527
3154	2.43750000	0.12500000	0.11984360
3155	2.56250000	0.18750000	0.08132942
3156	2.68750000	0.12500000	0.06060606
3157	2.62500000	0.18750000	0.06412512
3158	1.93750000	0.37500000	-0.03675464
3159	2.06250000	0.43750000	-0.05610948
3160	1.93750000	0.31250000	-0.01075269
3161	2.06250000	0.37500000	-0.03010753
3162	1.93750000	0.56250000	0.00508309
3163	2.06250000	0.62500000	-0.03108504
3164	1.93750000	0.62500000	0.00078201
3165	2.06250000	0.68750000	-0.01036168
3166	1.87500000	0.43750000	0.03323558
3167	1.87500000	0.31250000	0.08719453
3168	1.81250000	0.37500000	0.05708700
3169	2.12500000	0.56250000	-0.02228739
3170	2.12500000	0.43750000	0.04848485
3171	2.18750000	0.56250000	0.00723363
3172	1.87500000	0.56250000	0.00097752
3173	1.81250000	0.43750000	0.02913001
3174	2.12500000	0.68750000	-0.04633431
3175	2.18750000	0.62500000	-0.03753666
3176	2.43750000	0.37500000	0.04046921
3177	2.37500000	0.43750000	0.02873900
3178	2.37500000	0.31250000	0.06177908
3179	2.31250000	0.37500000	0.05004888
3180	2.62500000	0.56250000	-0.04281525
3181	2.56250000	0.62500000	-0.03890518
3182	2.68750000	0.62500000	-0.06744868
3183	2.62500000	0.68750000	-0.06353861

3184	2.56250000	0.43750000	0.00175953
3185	2.43750000	0.31250000	0.06021505
3186	2.56250000	0.37500000	0.02170088
3187	2.43750000	0.56250000	-0.00606061
3188	2.31250000	0.43750000	0.03675464
3189	2.37500000	0.56250000	0.00195503
3190	2.68750000	0.56250000	-0.06001955
3191	2.62500000	0.43750000	-0.01544477
3192	2.56250000	0.68750000	-0.05219941
3193	2.43750000	0.62500000	-0.01955034
3194	1.56250000	1.62500000	-0.02580645
3195	1.62500000	1.56250000	-0.01876833
3196	1.62500000	1.68750000	-0.01407625
3197	1.68750000	1.62500000	-0.00703812
3198	1.37500000	1.43750000	-0.15562072
3199	1.43750000	1.37500000	-0.09794721
3200	1.31250000	1.37500000	-0.16304985
3201	1.37500000	1.31250000	-0.11788856
3202	1.43750000	1.56250000	-0.14506354
3203	1.56250000	1.68750000	-0.12297165
3204	1.43750000	1.62500000	-0.14193548
3205	1.56250000	1.43750000	-0.08035191
3206	1.68750000	1.56250000	-0.10889541
3207	1.62500000	1.43750000	-0.07018573
3208	1.31250000	1.43750000	-0.16285435
3209	1.37500000	1.56250000	-0.15229717
3210	1.43750000	1.31250000	-0.06001955
3211	1.56250000	1.37500000	-0.02991202
3212	2.37500000	2.43750000	-0.00136852
3213	2.43750000	2.37500000	-0.02170088
3214	2.31250000	2.37500000	-0.00625611
3215	2.37500000	2.31250000	-0.02658847
3216	2.56250000	2.62500000	-0.01290323
3217	2.62500000	2.56250000	-0.03753666
3218	2.62500000	2.68750000	-0.03401760
3219	2.68750000	2.62500000	-0.05884653
3220	2.43750000	2.56250000	0.01661779
3221	2.31250000	2.43750000	0.00703812
3222	2.37500000	2.56250000	0.02502444
3223	2.56250000	2.43750000	-0.02834800
3224	2.43750000	2.31250000	-0.03362659
3225	2.56250000	2.37500000	-0.04027370
3226	2.56250000	2.68750000	0.00019550
3227	2.43750000	2.62500000	0.02971652
3228	2.68750000	2.56250000	-0.04926686
3229	2.62500000	2.43750000	-0.03988270
3230	1.56250000	1.93750000	-0.05884653
3231	1.62500000	2.06250000	-0.03323558
3232	1.62500000	1.93750000	-0.07546432
3233	1.68750000	2.06250000	-0.04985337
3234	1.37500000	1.93750000	-0.08758553
3235	1.43750000	2.06250000	-0.06920821
3236	1.31250000	1.93750000	-0.12316716
3237	1.37500000	2.06250000	-0.10459433
3238	1.43750000	1.87500000	-0.08093842
3239	1.56250000	1.87500000	-0.08758553
3240	1.43750000	1.81250000	-0.10987292
3241	1.56250000	2.12500000	-0.03695015
3242	1.68750000	2.12500000	-0.03636364

3243	1.62500000	2.18750000	-0.04007820
3244	1.31250000	1.87500000	-0.12649071
3245	1.37500000	1.81250000	-0.11984360
3246	1.43750000	2.12500000	-0.08954057
3247	1.56250000	2.18750000	-0.05728250
3248	1.93750000	1.56250000	-0.06744868
3249	2.06250000	1.62500000	-0.06568915
3250	1.93750000	1.62500000	-0.06588465
3251	2.06250000	1.68750000	-0.06412512
3252	1.93750000	1.37500000	-0.07135875
3253	2.06250000	1.43750000	-0.06959922
3254	1.93750000	1.31250000	-0.07448680
3255	2.06250000	1.37500000	-0.07272727
3256	1.87500000	1.43750000	-0.07878788
3257	1.87500000	1.56250000	-0.07800587
3258	1.81250000	1.43750000	-0.08914956
3259	2.12500000	1.56250000	-0.07526882
3260	2.12500000	1.68750000	-0.07448680
3261	2.18750000	1.62500000	-0.08406647
3262	1.87500000	1.31250000	-0.08113392
3263	1.81250000	1.37500000	-0.08836755
3264	2.12500000	1.43750000	-0.07761486
3265	2.18750000	1.56250000	-0.08328446
3266	2.06250000	2.43750000	0.02834800
3267	1.93750000	2.37500000	0.02697947
3268	2.06250000	2.37500000	0.02737048
3269	1.93750000	2.31250000	0.01368524
3270	2.06250000	2.62500000	0.08132942
3271	1.93750000	2.56250000	0.05943304
3272	2.06250000	2.68750000	0.09521017
3273	1.93750000	2.62500000	0.07350929
3274	2.12500000	2.56250000	0.04652981
3275	2.12500000	2.43750000	0.01994135
3276	2.18750000	2.56250000	0.03792766
3277	1.87500000	2.43750000	0.02326491
3278	1.87500000	2.31250000	0.00488759
3279	1.81250000	2.37500000	0.00117302
3280	2.12500000	2.68750000	0.08602151
3281	2.18750000	2.62500000	0.06353861
3282	1.87500000	2.56250000	0.04242424
3283	1.81250000	2.43750000	0.00625611
3284	2.43750000	2.06250000	-0.05669599
3285	2.37500000	1.93750000	-0.06686217
3286	2.37500000	2.06250000	-0.05004888
3287	2.31250000	1.93750000	-0.06041056
3288	2.62500000	2.06250000	-0.14819159
3289	2.56250000	1.93750000	-0.07663734
3290	2.68750000	2.06250000	-0.17458456
3291	2.62500000	1.93750000	-0.15151515
3292	2.56250000	2.12500000	-0.11143695
3293	2.43750000	2.12500000	-0.06979472
3294	2.56250000	2.18750000	-0.10107527
3295	2.43750000	1.87500000	-0.05004888
3296	2.31250000	1.87500000	-0.09032258
3297	2.37500000	1.81250000	-0.09853372
3298	2.68750000	2.12500000	-0.17282502
3299	2.62500000	2.18750000	-0.13607038
3300	2.56250000	1.87500000	-0.07820137
3301	2.43750000	1.81250000	-0.05180841

3302	1.56250000	3.93750000	-0.04222874
3303	1.62500000	3.93750000	-0.01857283
3304	1.37500000	3.93750000	-0.04086022
3305	1.31250000	3.93750000	-0.00332356
3306	1.43750000	3.87500000	-0.04398827
3307	1.56250000	3.87500000	-0.00782014
3308	1.43750000	3.81250000	-0.00957967
3309	1.31250000	3.87500000	-0.01896383
3310	1.37500000	3.81250000	-0.02209189
3311	2.37500000	3.93750000	-0.11378299
3312	2.31250000	3.93750000	-0.07800587
3313	2.56250000	3.93750000	-0.09149560
3314	2.62500000	3.93750000	-0.04203324
3315	2.43750000	3.87500000	-0.09912023
3316	2.31250000	3.87500000	-0.06490714
3317	2.37500000	3.81250000	-0.05102639
3318	2.56250000	3.87500000	-0.04809384
3319	2.43750000	3.81250000	-0.04946237
3320	1.56250000	3.62500000	0.02971652
3321	1.62500000	3.56250000	0.06568915
3322	1.62500000	3.68750000	0.01896383
3323	1.68750000	3.62500000	0.05493646
3324	1.37500000	3.43750000	0.13431085
3325	1.43750000	3.37500000	0.14643206
3326	1.31250000	3.37500000	0.10615836
3327	1.37500000	3.31250000	0.11671554
3328	1.43750000	3.56250000	0.12805474
3329	1.56250000	3.68750000	0.09540567
3330	1.43750000	3.62500000	0.09364614
3331	1.56250000	3.43750000	0.17614858
3332	1.68750000	3.56250000	0.16735093
3333	1.62500000	3.43750000	0.17614858
3334	1.31250000	3.43750000	0.12179863
3335	1.37500000	3.56250000	0.11554252
3336	1.43750000	3.31250000	0.14447703
3337	1.56250000	3.37500000	0.17419355
3338	2.06250000	3.62500000	0.12707722
3339	1.93750000	3.56250000	0.17360704
3340	2.06250000	3.68750000	0.11476051
3341	1.93750000	3.62500000	0.16129032
3342	2.06250000	3.43750000	0.18729228
3343	1.93750000	3.37500000	0.18201369
3344	2.06250000	3.37500000	0.18553275
3345	1.93750000	3.31250000	0.18044966
3346	2.12500000	3.56250000	0.14095797
3347	2.12500000	3.68750000	0.07898338
3348	2.18750000	3.62500000	0.09286413
3349	1.87500000	3.43750000	0.18220919
3350	1.87500000	3.56250000	0.17204301
3351	1.81250000	3.43750000	0.18084066
3352	2.12500000	3.43750000	0.18885630
3353	2.18750000	3.56250000	0.14252199
3354	1.87500000	3.31250000	0.17849462
3355	1.81250000	3.37500000	0.17869013
3356	3.93750000	1.56250000	-0.06705767
3357	3.93750000	1.62500000	-0.07702835
3358	3.93750000	1.37500000	-0.06412512
3359	3.93750000	1.31250000	-0.07233627
3360	3.87500000	1.43750000	-0.07253177

3361	3.87500000	1.56250000	-0.08367546
3362	3.81250000	1.43750000	-0.08914956
3363	3.87500000	1.31250000	-0.07956989
3364	3.81250000	1.37500000	-0.08797654
3365	3.93750000	2.37500000	-0.07722385
3366	3.93750000	2.31250000	-0.08543500
3367	3.93750000	2.56250000	-0.04868035
3368	3.93750000	2.62500000	-0.03695015
3369	3.87500000	2.43750000	-0.07370479
3370	3.87500000	2.31250000	-0.09051808
3371	3.81250000	2.37500000	-0.13782991
3372	3.87500000	2.56250000	-0.05337243
3373	3.81250000	2.43750000	-0.12922776
3374	3.62500000	1.56250000	-0.14975562
3375	3.56250000	1.62500000	-0.16500489
3376	3.68750000	1.62500000	-0.14271750
3377	3.62500000	1.68750000	-0.15796676
3378	3.43750000	1.37500000	-0.17634409
3379	3.37500000	1.43750000	-0.17771261
3380	3.37500000	1.31250000	-0.17908113
3381	3.31250000	1.37500000	-0.18044966
3382	3.56250000	1.43750000	-0.15698925
3383	3.68750000	1.56250000	-0.13313783
3384	3.62500000	1.43750000	-0.14486804
3385	3.43750000	1.56250000	-0.17360704
3386	3.56250000	1.68750000	-0.16363636
3387	3.43750000	1.62500000	-0.17223851
3388	3.43750000	1.31250000	-0.17653959
3389	3.56250000	1.37500000	-0.16168133
3390	3.31250000	1.43750000	-0.17927664
3391	3.37500000	1.56250000	-0.17517107
3392	3.62500000	2.06250000	-0.14369501
3393	3.56250000	1.93750000	-0.15855327
3394	3.68750000	2.06250000	-0.13861193
3395	3.62500000	1.93750000	-0.15327468
3396	3.43750000	2.06250000	-0.15679374
3397	3.37500000	1.93750000	-0.17165200
3398	3.37500000	2.06250000	-0.16480938
3399	3.31250000	1.93750000	-0.17634409
3400	3.56250000	2.12500000	-0.13704790
3401	3.68750000	2.12500000	-0.13040078
3402	3.62500000	2.18750000	-0.12062561
3403	3.43750000	1.87500000	-0.16676442
3404	3.56250000	1.87500000	-0.15992180
3405	3.43750000	1.81250000	-0.16813294
3406	3.43750000	2.12500000	-0.14662757
3407	3.56250000	2.18750000	-0.12218964
3408	3.31250000	1.87500000	-0.17302053
3409	3.37500000	1.81250000	-0.16969697
3410	0.37500000	0.93750000	-0.13470186
3411	0.43750000	1.06250000	-0.17849462
3412	0.31250000	0.93750000	-0.14349951
3413	0.37500000	1.06250000	-0.16226784
3414	0.56250000	0.93750000	-0.18866080
3415	0.62500000	1.06250000	-0.19608993
3416	0.62500000	0.93750000	-0.18905181
3417	0.68750000	1.06250000	-0.19667644
3418	0.43750000	0.87500000	-0.14389052
3419	0.31250000	0.87500000	-0.11573803

3420	0.37500000	0.81250000	-0.12473118
3421	0.56250000	0.87500000	-0.18123167
3422	0.43750000	0.81250000	-0.16168133
3423	0.56250000	1.12500000	-0.19511241
3424	0.43750000	1.12500000	-0.17829912
3425	0.56250000	1.18750000	-0.19491691
3426	0.68750000	1.12500000	-0.19628543
3427	0.62500000	1.18750000	-0.19550342
3428	0.37500000	1.43750000	-0.17380254
3429	0.43750000	1.37500000	-0.17712610
3430	0.31250000	1.37500000	-0.15777126
3431	0.37500000	1.31250000	-0.16109482
3432	0.56250000	1.62500000	-0.17790811
3433	0.62500000	1.56250000	-0.18123167
3434	0.62500000	1.68750000	-0.17810362
3435	0.68750000	1.62500000	-0.18142717
3436	0.43750000	1.56250000	-0.18709677
3437	0.31250000	1.43750000	-0.17067449
3438	0.37500000	1.56250000	-0.18396872
3439	0.56250000	1.68750000	-0.18768328
3440	0.43750000	1.62500000	-0.18435973
3441	0.56250000	1.43750000	-0.19374389
3442	0.43750000	1.31250000	-0.17732160
3443	0.56250000	1.37500000	-0.19393939
3444	0.68750000	1.56250000	-0.19433040
3445	0.62500000	1.43750000	-0.19433040
3446	0.93750000	0.37500000	-0.09951124
3447	1.06250000	0.43750000	-0.11652004
3448	0.93750000	0.31250000	-0.08973607
3449	1.06250000	0.37500000	-0.10674487
3450	0.93750000	0.56250000	-0.14291300
3451	1.06250000	0.62500000	-0.14271750
3452	0.93750000	0.62500000	-0.15112414
3453	1.06250000	0.68750000	-0.15092864
3454	0.87500000	0.43750000	-0.10967742
3455	0.87500000	0.31250000	-0.07448680
3456	0.81250000	0.37500000	-0.08445748
3457	0.87500000	0.56250000	-0.14330401
3458	0.81250000	0.43750000	-0.10987292
3459	1.12500000	0.56250000	-0.12649071
3460	1.12500000	0.43750000	-0.10850440
3461	1.18750000	0.56250000	-0.11847507
3462	1.12500000	0.68750000	-0.14291300
3463	1.18750000	0.62500000	-0.12668622
3464	1.43750000	0.37500000	0.05962854
3465	1.37500000	0.43750000	0.03655914
3466	1.37500000	0.31250000	0.01114370
3467	1.31250000	0.37500000	-0.02443793
3468	1.62500000	0.56250000	0.04711632
3469	1.56250000	0.62500000	0.02209189
3470	1.68750000	0.62500000	-0.00312805
3471	1.62500000	0.68750000	-0.02815249
3472	1.56250000	0.43750000	0.07468231
3473	1.43750000	0.31250000	0.03695015
3474	1.56250000	0.37500000	0.05219941
3475	1.68750000	0.56250000	0.01368524
3476	1.62500000	0.43750000	0.04144673
3477	1.43750000	0.56250000	0.02658847
3478	1.31250000	0.43750000	-0.02170088

3479	1.37500000	0.56250000	-0.03147605
3480	1.56250000	0.68750000	-0.03636364
3481	1.43750000	0.62500000	-0.04418377
3482	1.06250000	1.43750000	-0.18768328
3483	0.93750000	1.37500000	-0.19413490
3484	1.06250000	1.37500000	-0.18787879
3485	0.93750000	1.31250000	-0.19452590
3486	1.06250000	1.62500000	-0.18983382
3487	0.93750000	1.56250000	-0.19648094
3488	1.06250000	1.68750000	-0.18983382
3489	0.93750000	1.62500000	-0.19648094
3490	1.12500000	1.56250000	-0.18318671
3491	1.12500000	1.43750000	-0.18377322
3492	1.18750000	1.56250000	-0.17634409
3493	1.12500000	1.68750000	-0.18279570
3494	1.18750000	1.62500000	-0.17595308
3495	0.87500000	1.43750000	-0.19628543
3496	0.87500000	1.31250000	-0.19687195
3497	0.81250000	1.37500000	-0.19608993
3498	0.87500000	1.56250000	-0.19608993
3499	0.81250000	1.43750000	-0.19589443
3500	1.43750000	1.06250000	-0.09462366
3501	1.37500000	0.93750000	-0.14369501
3502	1.37500000	1.06250000	-0.10263930
3503	1.31250000	0.93750000	-0.15171065
3504	1.62500000	1.06250000	-0.11671554
3505	1.56250000	0.93750000	-0.11612903
3506	1.68750000	1.06250000	-0.10967742
3507	1.62500000	0.93750000	-0.11104594
3508	1.56250000	1.12500000	-0.07565982
3509	1.43750000	1.12500000	-0.04535679
3510	1.56250000	1.18750000	-0.02756598
3511	1.68750000	1.12500000	-0.10694037
3512	1.62500000	1.18750000	-0.06588465
3513	1.43750000	0.87500000	-0.12414467
3514	1.31250000	0.87500000	-0.14349951
3515	1.37500000	0.81250000	-0.12707722
3516	1.56250000	0.87500000	-0.10772239
3517	1.43750000	0.81250000	-0.11573803
3518	0.37500000	3.43750000	0.16656891
3519	0.43750000	3.37500000	0.15131965
3520	0.31250000	3.37500000	0.15249267
3521	0.37500000	3.31250000	0.13724340
3522	0.56250000	3.62500000	0.18553275
3523	0.62500000	3.56250000	0.17028348
3524	0.62500000	3.68750000	0.18787879
3525	0.68750000	3.62500000	0.17262952
3526	0.43750000	3.56250000	0.18279570
3527	0.31250000	3.43750000	0.16637341
3528	0.37500000	3.56250000	0.18240469
3529	0.56250000	3.68750000	0.18748778
3530	0.43750000	3.62500000	0.18475073
3531	0.56250000	3.43750000	0.15229717
3532	0.43750000	3.31250000	0.13587488
3533	0.56250000	3.37500000	0.13665689
3534	0.68750000	3.56250000	0.15698925
3535	0.62500000	3.43750000	0.13900293
3536	0.37500000	2.93750000	0.05161290
3537	0.43750000	3.06250000	0.07331378

3538	0.31250000	2.93750000	0.05376344
3539	0.37500000	3.06250000	0.07487781
3540	0.56250000	2.93750000	0.02365591
3541	0.62500000	3.06250000	0.04516129
3542	0.62500000	2.93750000	0.01016618
3543	0.68750000	3.06250000	0.03167155
3544	0.43750000	2.87500000	0.03088954
3545	0.31250000	2.87500000	0.04613881
3546	0.37500000	2.81250000	0.02482893
3547	0.56250000	2.87500000	0.00430108
3548	0.43750000	2.81250000	0.01173021
3549	0.56250000	3.12500000	0.07409580
3550	0.43750000	3.12500000	0.08895406
3551	0.56250000	3.18750000	0.08954057
3552	0.68750000	3.12500000	0.04731183
3553	0.62500000	3.18750000	0.07624633
3554	1.06250000	3.62500000	0.06823069
3555	0.93750000	3.56250000	0.10361681
3556	1.06250000	3.68750000	0.08406647
3557	0.93750000	3.62500000	0.11925709
3558	1.06250000	3.43750000	0.07135875
3559	0.93750000	3.37500000	0.05650049
3560	1.06250000	3.37500000	0.05591398
3561	0.93750000	3.31250000	0.04086022
3562	1.12500000	3.56250000	0.06510264
3563	1.12500000	3.68750000	0.04652981
3564	1.18750000	3.62500000	0.04340176
3565	1.12500000	3.43750000	0.08406647
3566	1.18750000	3.56250000	0.07781036
3567	0.87500000	3.43750000	0.08563050
3568	0.87500000	3.56250000	0.11710655
3569	0.81250000	3.43750000	0.09912023
3570	0.87500000	3.31250000	0.05415445
3571	0.81250000	3.37500000	0.08328446
3572	3.43750000	0.37500000	0.02971652
3573	3.37500000	0.43750000	-0.00019550
3574	3.37500000	0.31250000	0.03284457
3575	3.31250000	0.37500000	0.00938416
3576	3.62500000	0.56250000	-0.09051808
3577	3.56250000	0.62500000	-0.11300098
3578	3.68750000	0.62500000	-0.09110459
3579	3.62500000	0.68750000	-0.11319648
3580	3.56250000	0.43750000	-0.06471163
3581	3.43750000	0.31250000	-0.05102639
3582	3.56250000	0.37500000	-0.04574780
3583	3.68750000	0.56250000	-0.07585533
3584	3.62500000	0.43750000	-0.05630499
3585	3.43750000	0.56250000	-0.11710655
3586	3.31250000	0.43750000	-0.10439883
3587	3.37500000	0.56250000	-0.12121212
3588	3.56250000	0.68750000	-0.12043011
3589	3.43750000	0.62500000	-0.12434018
3590	2.93750000	0.37500000	-0.09071359
3591	3.06250000	0.43750000	-0.12375367
3592	2.93750000	0.31250000	-0.08054741
3593	3.06250000	0.37500000	-0.11339198
3594	2.93750000	0.56250000	-0.12825024
3595	3.06250000	0.62500000	-0.14877810
3596	2.93750000	0.62500000	-0.13548387

3597	3.06250000	0.68750000	-0.15601173
3598	2.87500000	0.43750000	-0.08387097
3599	2.87500000	0.31250000	-0.05376344
3600	2.81250000	0.37500000	-0.04672532
3601	2.87500000	0.56250000	-0.11104594
3602	2.81250000	0.43750000	-0.06666667
3603	3.12500000	0.56250000	-0.13743891
3604	3.12500000	0.43750000	-0.11964809
3605	3.18750000	0.56250000	-0.13333333
3606	3.12500000	0.68750000	-0.15210166
3607	3.18750000	0.62500000	-0.14076246
3608	3.62500000	1.06250000	-0.14154448
3609	3.56250000	0.93750000	-0.14995112
3610	3.68750000	1.06250000	-0.13431085
3611	3.62500000	0.93750000	-0.14271750
3612	3.43750000	1.06250000	-0.17067449
3613	3.37500000	0.93750000	-0.17751711
3614	3.37500000	1.06250000	-0.17458456
3615	3.31250000	0.93750000	-0.16911046
3616	3.56250000	1.12500000	-0.15112414
3617	3.68750000	1.12500000	-0.12590420
3618	3.62500000	1.18750000	-0.13587488
3619	3.43750000	1.12500000	-0.17302053
3620	3.56250000	1.18750000	-0.15386119
3621	3.43750000	0.87500000	-0.16637341
3622	3.56250000	0.87500000	-0.14271750
3623	3.43750000	0.81250000	-0.14662757
3624	3.31250000	0.87500000	-0.17438905
3625	3.37500000	0.81250000	-0.16304985
3626	3.06250000	3.43750000	0.08426197
3627	2.93750000	3.37500000	0.08152493
3628	3.06250000	3.37500000	0.04731183
3629	2.93750000	3.31250000	0.04457478
3630	3.06250000	3.62500000	0.15913978
3631	2.93750000	3.56250000	0.14056696
3632	3.06250000	3.68750000	0.15620723
3633	2.93750000	3.62500000	0.15014663
3634	3.12500000	3.56250000	0.14975562
3635	3.12500000	3.43750000	0.08445748
3636	3.18750000	3.56250000	0.14995112
3637	3.12500000	3.68750000	0.17517107
3638	3.18750000	3.62500000	0.17829912
3639	2.87500000	3.43750000	0.12844575
3640	2.87500000	3.31250000	0.07898338
3641	2.81250000	3.37500000	0.12570870
3642	2.87500000	3.56250000	0.15053763
3643	2.81250000	3.43750000	0.13822092
3644	3.43750000	3.06250000	0.01935484
3645	3.37500000	2.93750000	-0.00821114
3646	3.37500000	3.06250000	-0.00684262
3647	3.31250000	2.93750000	-0.03440860
3648	3.62500000	3.06250000	-0.05024438
3649	3.56250000	2.93750000	0.01915934
3650	3.68750000	3.06250000	0.04946237
3651	3.62500000	2.93750000	0.01955034
3652	3.56250000	3.12500000	-0.03968719
3653	3.43750000	3.12500000	0.03030303
3654	3.56250000	3.18750000	0.07096774
3655	3.68750000	3.12500000	-0.03616813

3656	3.62500000	3.18750000	-0.02521994
3657	3.43750000	2.87500000	0.00215054
3658	3.31250000	2.87500000	-0.02482893
3659	3.37500000	2.81250000	-0.01446725
3660	3.56250000	2.87500000	0.00332356
3661	3.43750000	2.81250000	-0.01368524
3662	2.56250000	2.93750000	0.05219941
3663	2.62500000	3.06250000	0.06783969
3664	2.62500000	2.93750000	0.01779081
3665	2.68750000	3.06250000	0.03362659
3666	2.37500000	2.93750000	0.10439883
3667	2.43750000	3.06250000	0.12140762
3668	2.31250000	2.93750000	0.11358749
3669	2.37500000	3.06250000	0.13079179
3670	2.43750000	2.87500000	0.08211144
3671	2.56250000	2.87500000	0.03929619
3672	2.43750000	2.81250000	0.06901271
3673	2.31250000	2.87500000	0.09970674
3674	2.37500000	2.81250000	0.07741935
3675	2.56250000	3.12500000	0.11476051
3676	2.68750000	3.12500000	0.07077224
3677	2.62500000	3.18750000	0.11749756
3678	2.43750000	3.12500000	0.13391984
3679	2.56250000	3.18750000	0.12727273
3680	2.93750000	2.56250000	-0.09618768
3681	3.06250000	2.62500000	-0.10381232
3682	2.93750000	2.62500000	-0.10576735
3683	3.06250000	2.68750000	-0.10087977
3684	2.93750000	2.37500000	-0.13646139
3685	3.06250000	2.43750000	-0.10967742
3686	2.93750000	2.31250000	-0.14936461
3687	3.06250000	2.37500000	-0.12336266
3688	2.87500000	2.43750000	-0.11182796
3689	2.87500000	2.56250000	-0.08445748
3690	2.81250000	2.43750000	-0.10009775
3691	2.87500000	2.31250000	-0.16168133
3692	2.81250000	2.37500000	-0.13704790
3693	3.12500000	2.56250000	-0.09266862
3694	3.12500000	2.68750000	-0.08719453
3695	3.18750000	2.62500000	-0.07683284
3696	3.12500000	2.43750000	-0.10889541
3697	3.18750000	2.56250000	-0.07937439
3698	2.56250000	3.62500000	0.15034213
3699	2.62500000	3.56250000	0.18983382
3700	2.62500000	3.68750000	0.14995112
3701	2.68750000	3.62500000	0.18963832
3702	2.37500000	3.43750000	0.19511241
3703	2.43750000	3.37500000	0.18416422
3704	2.31250000	3.37500000	0.19178886
3705	2.37500000	3.31250000	0.18084066
3706	2.43750000	3.56250000	0.14897361
3707	2.56250000	3.68750000	0.10068426
3708	2.43750000	3.62500000	0.09951124
3709	2.31250000	3.43750000	0.19354839
3710	2.37500000	3.56250000	0.14740958
3711	2.56250000	3.43750000	0.17751711
3712	2.68750000	3.56250000	0.17986315
3713	2.62500000	3.43750000	0.16754643
3714	2.43750000	3.31250000	0.17165200

3715	2.56250000	3.37500000	0.16500489
3716	3.62500000	2.56250000	-0.07214076
3717	3.56250000	2.62500000	-0.06080156
3718	3.68750000	2.62500000	-0.05571848
3719	3.62500000	2.68750000	-0.04437928
3720	3.43750000	2.37500000	-0.11671554
3721	3.37500000	2.43750000	-0.10869990
3722	3.37500000	2.31250000	-0.13000978
3723	3.31250000	2.37500000	-0.12199413
3724	3.56250000	2.43750000	-0.09716520
3725	3.68750000	2.56250000	-0.06744868
3726	3.62500000	2.43750000	0.05141740
3727	3.43750000	2.31250000	-0.12375367
3728	3.56250000	2.37500000	0.03812317
3729	3.43750000	2.56250000	-0.07781036
3730	3.56250000	2.68750000	-0.04477028
3731	3.43750000	2.62500000	-0.06177908
3732	3.31250000	2.43750000	-0.10772239
3733	3.37500000	2.56250000	-0.07839687
3734	0.93750000	2.37500000	-0.17653959
3735	1.06250000	2.43750000	-0.16461388
3736	0.93750000	2.31250000	-0.17927664
3737	1.06250000	2.37500000	-0.16715543
3738	0.93750000	2.56250000	-0.14916911
3739	1.06250000	2.62500000	-0.11730205
3740	0.93750000	2.62500000	-0.13372434
3741	1.06250000	2.68750000	-0.10166178
3742	0.87500000	2.43750000	-0.16422287
3743	0.87500000	2.31250000	-0.17614858
3744	0.81250000	2.37500000	-0.16363636
3745	0.87500000	2.56250000	-0.13958944
3746	0.81250000	2.43750000	-0.15444770
3747	1.12500000	2.56250000	-0.12043011
3748	1.12500000	2.43750000	-0.15210166
3749	1.18750000	2.56250000	-0.10791789
3750	1.12500000	2.68750000	-0.07565982
3751	1.18750000	2.62500000	-0.07878788
3752	1.43750000	2.37500000	-0.09462366
3753	1.37500000	2.43750000	-0.10205279
3754	1.37500000	2.31250000	-0.10967742
3755	1.31250000	2.37500000	-0.11710655
3756	1.62500000	2.56250000	-0.02619746
3757	1.56250000	2.62500000	-0.01427175
3758	1.68750000	2.62500000	0.00488759
3759	1.62500000	2.68750000	0.01681329
3760	1.56250000	2.43750000	-0.06236559
3761	1.43750000	2.31250000	-0.09970674
3762	1.56250000	2.37500000	-0.06744868
3763	1.68750000	2.56250000	-0.00899316
3764	1.62500000	2.43750000	-0.04516129
3765	1.43750000	2.56250000	-0.05786901
3766	1.31250000	2.43750000	-0.11456500
3767	1.37500000	2.56250000	-0.07038123
3768	1.56250000	2.68750000	0.01485826
3769	1.43750000	2.62500000	-0.02873900
3770	1.43750000	3.06250000	0.13626588
3771	1.37500000	2.93750000	0.09247312
3772	1.37500000	3.06250000	0.11163245
3773	1.31250000	2.93750000	0.06803519

3774	1.62500000	3.06250000	0.16422287
3775	1.56250000	2.93750000	0.13137830
3776	1.68750000	3.06250000	0.16617791
3777	1.62500000	2.93750000	0.13333333
3778	1.56250000	3.12500000	0.16441838
3779	1.43750000	3.12500000	0.13822092
3780	1.56250000	3.18750000	0.16637341
3781	1.68750000	3.12500000	0.16793744
3782	1.62500000	3.18750000	0.16793744
3783	1.43750000	2.87500000	0.08778104
3784	1.31250000	2.87500000	0.05083089
3785	1.37500000	2.81250000	0.04613881
3786	1.56250000	2.87500000	0.10224829
3787	1.43750000	2.81250000	0.05865103
3788	2.37500000	0.93750000	-0.08836755
3789	2.43750000	1.06250000	-0.10537634
3790	2.31250000	0.93750000	-0.08347996
3791	2.37500000	1.06250000	-0.10048876
3792	2.56250000	0.93750000	-0.10615836
3793	2.62500000	1.06250000	-0.12922776
3794	2.62500000	0.93750000	-0.11710655
3795	2.68750000	1.06250000	-0.14037146
3796	2.43750000	0.87500000	-0.07976540
3797	2.31250000	0.87500000	-0.07311828
3798	2.37500000	0.81250000	-0.06451613
3799	2.56250000	0.87500000	-0.09247312
3800	2.43750000	0.81250000	-0.06627566
3801	2.56250000	1.12500000	-0.11984360
3802	2.43750000	1.12500000	-0.10713587
3803	2.56250000	1.18750000	-0.12160313
3804	2.68750000	1.12500000	-0.13880743
3805	2.62500000	1.18750000	-0.12942326
3806	2.37500000	1.43750000	-0.05806452
3807	2.43750000	1.37500000	-0.06432063
3808	2.31250000	1.37500000	-0.00136852
3809	2.37500000	1.31250000	-0.00762463
3810	2.56250000	1.62500000	-0.12844575
3811	2.62500000	1.56250000	-0.14017595
3812	2.62500000	1.68750000	-0.13802542
3813	2.68750000	1.62500000	-0.14995112
3814	2.43750000	1.56250000	-0.11065494
3815	2.31250000	1.43750000	-0.04985337
3816	2.37500000	1.56250000	-0.10244379
3817	2.56250000	1.68750000	-0.12434018
3818	2.43750000	1.62500000	-0.10654936
3819	2.56250000	1.43750000	-0.12864125
3820	2.43750000	1.31250000	-0.06236559
3821	2.56250000	1.37500000	-0.12688172
3822	2.68750000	1.56250000	-0.14799609
3823	2.62500000	1.43750000	-0.13646139
3824	3.06250000	1.43750000	-0.18553275
3825	2.93750000	1.37500000	-0.17732160
3826	3.06250000	1.37500000	-0.18690127
3827	2.93750000	1.31250000	-0.17869013
3828	3.06250000	1.62500000	-0.18455523
3829	2.93750000	1.56250000	-0.17947214
3830	3.06250000	1.68750000	-0.18651026
3831	2.93750000	1.62500000	-0.18142717
3832	3.12500000	1.56250000	-0.18123167

3833	3.12500000	1.43750000	-0.18416422
3834	3.18750000	1.56250000	-0.17986315
3835	3.12500000	1.68750000	-0.18162268
3836	3.18750000	1.62500000	-0.17829912
3837	2.87500000	1.43750000	-0.16793744
3838	2.87500000	1.31250000	-0.16774194
3839	2.81250000	1.37500000	-0.15835777
3840	2.87500000	1.56250000	-0.17145650
3841	2.81250000	1.43750000	-0.15992180
3842	0.12500000	0.31250000	0.00391007
3843	0.18750000	0.31250000	-0.01055718
3844	0.18750000	0.37500000	-0.02404692
3845	0.31250000	0.12500000	0.03343109
3846	0.31250000	0.18750000	0.00801564
3847	0.37500000	0.18750000	0.00215054
3848	0.12500000	3.81250000	0.18846530
3849	0.18750000	3.87500000	0.19081134
3850	0.18750000	3.81250000	0.18905181
3851	3.81250000	0.12500000	0.08914956
3852	3.87500000	0.18750000	0.08445748
3853	3.81250000	0.18750000	0.06823069
3854	3.68750000	3.81250000	0.17067449
3855	3.68750000	3.87500000	0.18025415
3856	3.81250000	3.68750000	0.15777126
3857	3.87500000	3.68750000	0.16070381
3858	3.62500000	3.81250000	0.17047898
3859	3.81250000	3.62500000	0.15092864
3860	0.12500000	1.81250000	-0.13607038
3861	0.18750000	1.87500000	-0.16129032
3862	0.18750000	1.81250000	-0.13880743
3863	0.12500000	2.31250000	-0.07350929
3864	0.18750000	2.31250000	-0.08973607
3865	0.18750000	2.37500000	-0.06725318
3866	0.31250000	2.12500000	-0.13724340
3867	0.31250000	2.18750000	-0.12864125
3868	0.37500000	2.18750000	-0.12531769
3869	1.81250000	0.12500000	0.13685239
3870	1.87500000	0.18750000	0.12609971
3871	1.81250000	0.18750000	0.12394917
3872	2.31250000	0.12500000	0.12297165
3873	2.31250000	0.18750000	0.10303030
3874	2.37500000	0.18750000	0.10146628
3875	2.12500000	0.31250000	0.08778104
3876	2.18750000	0.31250000	0.07956989
3877	2.18750000	0.37500000	0.06627566
3878	1.68750000	1.81250000	-0.09892473
3879	1.68750000	1.87500000	-0.09540567
3880	1.81250000	1.68750000	-0.08504399
3881	1.87500000	1.68750000	-0.07468231
3882	1.62500000	1.81250000	-0.10772239
3883	1.81250000	1.62500000	-0.08680352
3884	2.18750000	2.31250000	-0.00273705
3885	2.12500000	2.31250000	0.00566960
3886	2.31250000	2.18750000	-0.04398827
3887	2.31250000	2.12500000	-0.05630499
3888	2.18750000	2.37500000	0.01055718
3889	2.37500000	2.18750000	-0.05083089
3890	1.81250000	2.12500000	-0.04418377
3891	1.87500000	2.18750000	-0.02189638

3892	1.81250000	2.18750000	-0.03069404
3893	2.12500000	1.81250000	-0.06783969
3894	2.18750000	1.87500000	-0.07331378
3895	2.18750000	1.81250000	-0.07839687
3896	1.68750000	3.87500000	0.03949169
3897	1.68750000	3.81250000	0.07390029
3898	1.62500000	3.81250000	0.05024438
3899	2.18750000	3.87500000	0.00664712
3900	2.12500000	3.81250000	0.05454545
3901	2.18750000	3.81250000	0.01876833
3902	1.81250000	3.68750000	0.14584555
3903	1.87500000	3.68750000	0.14740958
3904	1.81250000	3.62500000	0.15816227
3905	3.87500000	1.68750000	-0.10322581
3906	3.81250000	1.68750000	-0.11945259
3907	3.81250000	1.62500000	-0.10987292
3908	3.87500000	2.18750000	-0.10694037
3909	3.81250000	2.12500000	-0.12023460
3910	3.81250000	2.18750000	-0.11202346
3911	3.68750000	1.81250000	-0.14995112
3912	3.68750000	1.87500000	-0.14897361
3913	3.62500000	1.81250000	-0.15562072
3914	0.12500000	0.81250000	-0.08934506
3915	0.18750000	0.87500000	-0.10830890
3916	0.18750000	0.81250000	-0.10557185
3917	0.12500000	1.31250000	-0.13567937
3918	0.18750000	1.31250000	-0.13861193
3919	0.18750000	1.37500000	-0.15171065
3920	0.31250000	1.12500000	-0.14565005
3921	0.31250000	1.18750000	-0.14525904
3922	0.37500000	1.18750000	-0.16168133
3923	0.81250000	0.12500000	-0.02991202
3924	0.87500000	0.18750000	-0.05493646
3925	0.81250000	0.18750000	-0.03968719
3926	1.31250000	0.12500000	0.01818182
3927	1.31250000	0.18750000	-0.00957967
3928	1.37500000	0.18750000	0.01622678
3929	1.12500000	0.31250000	-0.08895406
3930	1.18750000	0.31250000	-0.08093842
3931	1.18750000	0.37500000	-0.09071359
3932	0.68750000	0.81250000	-0.17497556
3933	0.68750000	0.87500000	-0.18220919
3934	0.81250000	0.68750000	-0.15972630
3935	0.87500000	0.68750000	-0.15953079
3936	0.62500000	0.81250000	-0.17438905
3937	0.81250000	0.62500000	-0.15171065
3938	1.18750000	1.31250000	-0.17751711
3939	1.12500000	1.31250000	-0.18435973
3940	1.31250000	1.18750000	-0.12473118
3941	1.31250000	1.12500000	-0.11143695
3942	1.18750000	1.37500000	-0.17712610
3943	1.37500000	1.18750000	-0.06666667
3944	0.81250000	1.12500000	-0.19745846
3945	0.87500000	1.18750000	-0.19765396
3946	0.81250000	1.18750000	-0.19706745
3947	1.12500000	0.81250000	-0.15933529
3948	1.18750000	0.87500000	-0.15953079
3949	1.18750000	0.81250000	-0.15131965
3950	0.12500000	3.31250000	0.13822092

3951	0.18750000	3.31250000	0.13841642
3952	0.18750000	3.37500000	0.15210166
3953	0.12500000	2.81250000	0.04281525
3954	0.18750000	2.87500000	0.04946237
3955	0.18750000	2.81250000	0.04164223
3956	0.31250000	3.18750000	0.10772239
3957	0.31250000	3.12500000	0.09208211
3958	0.37500000	3.18750000	0.10615836
3959	0.68750000	3.87500000	0.19413490
3960	0.68750000	3.81250000	0.19217986
3961	0.62500000	3.81250000	0.19178886
3962	1.18750000	3.87500000	0.05610948
3963	1.12500000	3.81250000	0.07800587
3964	1.18750000	3.81250000	0.04046921
3965	0.81250000	3.68750000	0.16168133
3966	0.87500000	3.68750000	0.14838710
3967	0.81250000	3.62500000	0.14604106
3968	3.31250000	0.12500000	-0.01896383
3969	3.31250000	0.18750000	-0.03831867
3970	3.37500000	0.18750000	-0.02854350
3971	2.81250000	0.12500000	0.01055718
3972	2.87500000	0.18750000	-0.02991202
3973	2.81250000	0.18750000	-0.00312805
3974	3.18750000	0.31250000	-0.08856305
3975	3.12500000	0.31250000	-0.09579668
3976	3.18750000	0.37500000	-0.10205279
3977	3.87500000	0.68750000	-0.06197458
3978	3.81250000	0.68750000	-0.07663734
3979	3.81250000	0.62500000	-0.06177908
3980	3.87500000	1.18750000	-0.09618768
3981	3.81250000	1.12500000	-0.11163245
3982	3.81250000	1.18750000	-0.10322581
3983	3.68750000	0.81250000	-0.12062561
3984	3.68750000	0.87500000	-0.12805474
3985	3.62500000	0.81250000	-0.12805474
3986	3.18750000	3.31250000	0.01036168
3987	3.12500000	3.31250000	0.01036168
3988	3.31250000	3.18750000	-0.01114370
3989	3.31250000	3.12500000	-0.02209189
3990	3.18750000	3.37500000	0.04750733
3991	3.37500000	3.18750000	0.01505376
3992	2.68750000	2.81250000	-0.04222874
3993	2.68750000	2.87500000	-0.02932551
3994	2.81250000	2.68750000	-0.09188661
3995	2.87500000	2.68750000	-0.10361681
3996	2.62500000	2.81250000	-0.00801564
3997	2.81250000	2.62500000	-0.08230694
3998	2.87500000	3.18750000	0.00488759
3999	2.81250000	3.12500000	0.00215054
4000	2.81250000	3.18750000	0.03929619
4001	3.18750000	2.87500000	-0.07722385
4002	3.12500000	2.81250000	-0.09384164
4003	3.18750000	2.81250000	-0.06764418
4004	3.18750000	3.87500000	0.18533724
4005	3.12500000	3.81250000	0.17556207
4006	3.18750000	3.81250000	0.18201369
4007	2.68750000	3.87500000	0.05063539
4008	2.68750000	3.81250000	0.10009775
4009	2.62500000	3.81250000	0.05083089

4010	2.87500000	3.68750000	0.16969697
4011	2.81250000	3.68750000	0.17947214
4012	2.81250000	3.62500000	0.16989247
4013	3.87500000	3.18750000	0.07859238
4014	3.81250000	3.12500000	0.06392962
4015	3.81250000	3.18750000	0.07820137
4016	3.87500000	2.68750000	-0.02991202
4017	3.81250000	2.68750000	-0.03460411
4018	3.81250000	2.62500000	-0.04633431
4019	3.68750000	2.87500000	0.00410557
4020	3.68750000	2.81250000	-0.01192571
4021	3.62500000	2.81250000	-0.01231672
4022	0.68750000	1.87500000	-0.18514174
4023	0.68750000	1.81250000	-0.18807429
4024	0.81250000	2.12500000	-0.18064516
4025	0.87500000	2.18750000	-0.18142717
4026	0.62500000	1.81250000	-0.18494624
4027	0.81250000	2.18750000	-0.17810362
4028	1.18750000	1.87500000	-0.16891496
4029	1.12500000	1.81250000	-0.17927664
4030	1.31250000	2.12500000	-0.13509286
4031	1.31250000	2.18750000	-0.13000978
4032	1.18750000	1.81250000	-0.17243402
4033	1.37500000	2.18750000	-0.11984360
4034	0.81250000	1.68750000	-0.19491691
4035	0.87500000	1.68750000	-0.19569892
4036	0.81250000	1.62500000	-0.19530792
4037	1.12500000	2.31250000	-0.15738025
4038	1.18750000	2.31250000	-0.14486804
4039	1.18750000	2.37500000	-0.14213099
4040	0.68750000	2.81250000	-0.04125122
4041	0.68750000	2.87500000	-0.02228739
4042	0.81250000	2.68750000	-0.09872923
4043	0.87500000	2.68750000	-0.10850440
4044	0.62500000	2.81250000	-0.02815249
4045	0.81250000	2.62500000	-0.11436950
4046	1.18750000	3.31250000	0.06549365
4047	1.12500000	3.31250000	0.05298143
4048	1.31250000	3.18750000	0.08817204
4049	1.31250000	3.12500000	0.08758553
4050	1.18750000	3.37500000	0.08113392
4051	1.37500000	3.18750000	0.11417400
4052	0.81250000	3.12500000	0.02072336
4053	0.87500000	3.18750000	0.02306940
4054	0.81250000	3.18750000	0.03616813
4055	1.12500000	2.81250000	-0.04301075
4056	1.18750000	2.87500000	0.00019550
4057	1.18750000	2.81250000	-0.01681329
4058	1.87500000	0.68750000	-0.02013685
4059	1.81250000	0.68750000	-0.03675464
4060	2.12500000	0.81250000	-0.03597263
4061	2.18750000	0.87500000	-0.05728250
4062	1.81250000	0.62500000	-0.01994135
4063	2.18750000	0.81250000	-0.04692082
4064	1.87500000	1.18750000	-0.08563050
4065	1.81250000	1.12500000	-0.09345064
4066	2.12500000	1.31250000	-0.03206256
4067	2.18750000	1.31250000	0.01173021
4068	1.81250000	1.18750000	-0.09208211

4069	2.18750000	1.37500000	-0.03695015
4070	1.68750000	0.81250000	-0.08660802
4071	1.68750000	0.87500000	-0.09462366
4072	1.62500000	0.81250000	-0.09462366
4073	2.31250000	1.12500000	-0.04750733
4074	2.31250000	1.18750000	0.00078201
4075	2.37500000	1.18750000	-0.05395894
4076	2.81250000	0.68750000	-0.10909091
4077	2.87500000	0.68750000	-0.12590420
4078	2.68750000	0.81250000	-0.10146628
4079	2.68750000	0.87500000	-0.11476051
4080	2.81250000	0.62500000	-0.10146628
4081	2.62500000	0.81250000	-0.09012708
4082	3.31250000	1.18750000	-0.18103617
4083	3.31250000	1.12500000	-0.17986315
4084	3.18750000	1.31250000	-0.18494624
4085	3.12500000	1.31250000	-0.18670577
4086	3.37500000	1.18750000	-0.17810362
4087	3.18750000	1.37500000	-0.18377322
4088	3.12500000	0.81250000	-0.16656891
4089	3.18750000	0.87500000	-0.16989247
4090	3.18750000	0.81250000	-0.16265885
4091	2.81250000	1.12500000	-0.16089932
4092	2.87500000	1.18750000	-0.17047898
4093	2.81250000	1.18750000	-0.15953079
4094	2.12500000	2.81250000	0.11378299
4095	2.18750000	2.87500000	0.11827957
4096	1.87500000	2.68750000	0.07018573
4097	1.81250000	2.68750000	0.05298143
4098	2.18750000	2.81250000	0.10439883
4099	1.81250000	2.62500000	0.03929619
4100	2.12500000	3.31250000	0.18533724
4101	2.18750000	3.31250000	0.18690127
4102	1.87500000	3.18750000	0.17517107
4103	1.81250000	3.12500000	0.17165200
4104	2.18750000	3.37500000	0.18866080
4105	1.81250000	3.18750000	0.17341153
4106	2.31250000	3.12500000	0.15249267
4107	2.31250000	3.18750000	0.16500489
4108	2.37500000	3.18750000	0.15581623
4109	1.68750000	2.81250000	0.07702835
4110	1.68750000	2.87500000	0.10615836
4111	1.62500000	2.81250000	0.07507331
4112	2.81250000	2.12500000	-0.18709677
4113	2.87500000	2.18750000	-0.17478006
4114	2.68750000	1.87500000	-0.16676442
4115	2.68750000	1.81250000	-0.15718475
4116	2.81250000	2.18750000	-0.18690127
4117	2.62500000	1.81250000	-0.14349951
4118	3.31250000	2.12500000	-0.15933529
4119	3.31250000	2.18750000	-0.15053763
4120	3.18750000	1.87500000	-0.18240469
4121	3.12500000	1.81250000	-0.18533724
4122	3.37500000	2.18750000	-0.14584555
4123	3.18750000	1.81250000	-0.18064516
4124	3.12500000	2.31250000	-0.13528837
4125	3.18750000	2.31250000	-0.13450635
4126	3.18750000	2.37500000	-0.12179863
4127	2.81250000	1.68750000	-0.16735093

4128	2.87500000	1.68750000	-0.17517107
4129	2.81250000	1.62500000	-0.16559140
4130	0.31250000	0.62500000	-0.10400782
4131	0.31250000	0.68750000	-0.11730205
4132	0.37500000	0.68750000	-0.12922776
4133	0.62500000	0.31250000	-0.05982405
4134	0.68750000	0.31250000	-0.05962854
4135	0.68750000	0.37500000	-0.08484848
4136	0.31250000	3.68750000	0.18592375
4137	0.31250000	3.62500000	0.18396872
4138	0.37500000	3.68750000	0.18631476
4139	3.68750000	0.31250000	-0.00293255
4140	3.62500000	0.31250000	-0.01779081
4141	3.68750000	0.37500000	-0.02248289
4142	3.37500000	3.68750000	0.15718475
4143	3.31250000	3.62500000	0.15992180
4144	3.31250000	3.68750000	0.17575758
4145	3.68750000	3.37500000	0.11339198
4146	3.62500000	3.31250000	0.09599218
4147	3.68750000	3.31250000	0.10283480
4148	0.31250000	1.68750000	-0.15014663
4149	0.31250000	1.62500000	-0.16559140
4150	0.37500000	1.68750000	-0.16578690
4151	0.31250000	2.62500000	-0.05102639
4152	0.31250000	2.68750000	-0.00742913
4153	0.37500000	2.68750000	-0.03792766
4154	0.62500000	2.31250000	-0.11260997
4155	0.68750000	2.31250000	-0.12844575
4156	0.68750000	2.37500000	-0.13802542
4157	1.68750000	0.31250000	0.06392962
4158	1.62500000	0.31250000	0.04692082
4159	1.68750000	0.37500000	0.03597263
4160	2.62500000	0.31250000	0.02443793
4161	2.68750000	0.31250000	0.00742913
4162	2.68750000	0.37500000	-0.01251222
4163	2.31250000	0.62500000	-0.00977517
4164	2.31250000	0.68750000	-0.02952102
4165	2.37500000	0.68750000	-0.03128055
4166	1.37500000	1.68750000	-0.14897361
4167	1.31250000	1.62500000	-0.15913978
4168	1.31250000	1.68750000	-0.15894428
4169	1.68750000	1.37500000	-0.05923754
4170	1.62500000	1.31250000	-0.01876833
4171	1.68750000	1.31250000	-0.05826002
4172	2.31250000	2.62500000	0.04652981
4173	2.37500000	2.68750000	0.05122190
4174	2.31250000	2.68750000	0.05962854
4175	2.62500000	2.31250000	-0.08875855
4176	2.68750000	2.37500000	-0.08856305
4177	2.68750000	2.31250000	-0.12551320
4178	1.68750000	2.31250000	-0.03812317
4179	1.62500000	2.31250000	-0.05532747
4180	1.68750000	2.37500000	-0.03304008
4181	2.31250000	1.68750000	-0.09618768
4182	2.31250000	1.62500000	-0.09521017
4183	2.37500000	1.68750000	-0.09931574
4184	1.37500000	3.68750000	0.04672532
4185	1.31250000	3.68750000	0.03421310
4186	1.31250000	3.62500000	0.06862170

4187	2.31250000	3.68750000	0.04652981
4188	2.37500000	3.68750000	0.04828935
4189	2.31250000	3.62500000	0.09618768
4190	1.68750000	3.37500000	0.17575758
4191	1.62500000	3.31250000	0.17204301
4192	1.68750000	3.31250000	0.17360704
4193	3.68750000	1.37500000	-0.12707722
4194	3.68750000	1.31250000	-0.12160313
4195	3.62500000	1.31250000	-0.14408602
4196	3.68750000	2.31250000	-0.14956012
4197	3.68750000	2.37500000	-0.00332356
4198	3.62500000	2.31250000	0.04046921
4199	3.37500000	1.68750000	-0.17243402
4200	3.31250000	1.62500000	-0.17536657
4201	3.31250000	1.68750000	-0.17399804
4202	0.62500000	1.31250000	-0.19491691
4203	0.68750000	1.31250000	-0.19550342
4204	0.68750000	1.37500000	-0.19511241
4205	1.31250000	0.62500000	-0.11045943
4206	1.31250000	0.68750000	-0.11867058
4207	1.37500000	0.68750000	-0.11045943
4208	0.62500000	3.31250000	0.10752688
4209	0.68750000	3.37500000	0.11006843
4210	0.68750000	3.31250000	0.09423265
4211	3.31250000	0.62500000	-0.13255132
4212	3.37500000	0.68750000	-0.13587488
4213	3.31250000	0.68750000	-0.13998045
4214	2.68750000	3.31250000	0.13274682
4215	2.68750000	3.37500000	0.14525904
4216	2.62500000	3.31250000	0.14252199
4217	3.31250000	2.68750000	-0.04692082
4218	3.37500000	2.68750000	-0.04633431
4219	3.31250000	2.62500000	-0.06295210
4220	1.31250000	2.62500000	-0.05376344
4221	1.31250000	2.68750000	-0.02463343
4222	1.37500000	2.68750000	-0.01212121
4223	2.62500000	1.31250000	-0.13294233
4224	2.68750000	1.31250000	-0.14076246
4225	2.68750000	1.37500000	-0.14252199
4226	0.03125000	0.03125000	0.14222874
4227	3.46875000	3.46875000	0.14946237
4228	1.53125000	1.53125000	-0.08494624
4229	2.46875000	3.96875000	-0.17282502
4230	1.53125000	3.53125000	0.11955034
4231	2.03125000	3.53125000	0.16314761
4232	1.46875000	0.46875000	0.10625611
4233	1.53125000	0.53125000	0.10537634
4234	3.46875000	0.46875000	-0.05092864
4235	3.53125000	3.03125000	-0.00801564
4236	2.46875000	1.46875000	-0.09433040
4237	2.00000000	0.28125000	0.05415445
4238	1.71875000	1.71875000	-0.04946237
4239	2.21875000	3.96875000	-0.02971652
4240	1.71875000	3.71875000	0.08729228
4241	2.03125000	3.78125000	0.11427175
4242	1.28125000	0.28125000	-0.03890518
4243	1.28125000	1.03125000	-0.13929619
4244	3.28125000	0.28125000	-0.02248289
4245	3.28125000	3.28125000	-0.00127077

4246	1.71875000	0.71875000	-0.04506354
4247	2.28125000	1.28125000	0.07546432
4248	3.71875000	3.21875000	0.03391984
4249	1.46875000	1.21875000	0.03255132
4250	2.28125000	3.78125000	-0.02883675
4251	2.21875000	3.71875000	0.01915934
4252	2.46875000	3.71875000	0.02580645
4253	2.28125000	3.53125000	0.16979472
4254	3.71875000	2.46875000	-0.00918866
4255	3.53125000	2.28125000	-0.04789834
4256	0.09375000	0.09375000	0.09237537
4257	2.09375000	3.96875000	0.04173998
4258	2.03125000	3.90625000	0.08963832
4259	3.40625000	3.40625000	0.09921799
4260	3.34375000	3.34375000	0.04897361
4261	3.34375000	3.46875000	0.12287390
4262	3.46875000	3.34375000	0.10136852
4263	2.00000000	0.34375000	-0.07174976
4264	1.96875000	0.37500000	-0.08572825
4265	2.03125000	0.40625000	-0.09540567
4266	1.96875000	0.34375000	-0.07272727
4267	2.03125000	0.37500000	-0.08240469
4268	1.90625000	0.37500000	0.01221896
4269	2.09375000	0.46875000	-0.01681329
4270	1.59375000	1.59375000	0.02707722
4271	1.65625000	1.65625000	0.03890518
4272	1.59375000	1.62500000	0.02864125
4273	1.62500000	1.59375000	0.03216031
4274	1.62500000	1.65625000	0.03450635
4275	1.65625000	1.62500000	0.03802542
4276	1.53125000	1.62500000	-0.08025415
4277	1.62500000	1.53125000	-0.06969697
4278	2.59375000	1.96875000	-0.11329423
4279	2.53125000	1.90625000	-0.03998045
4280	2.46875000	1.84375000	-0.02756598
4281	2.34375000	3.96875000	-0.10244379
4282	2.40625000	3.90625000	-0.12473118
4283	2.34375000	3.84375000	-0.07673509
4284	2.46875000	3.84375000	-0.07350929
4285	1.59375000	3.59375000	0.00869990
4286	1.65625000	3.65625000	-0.00205279
4287	1.62500000	3.59375000	0.00948192
4288	1.65625000	3.62500000	0.00410557
4289	1.62500000	3.53125000	0.12189638
4290	2.03125000	3.65625000	0.13880743
4291	2.09375000	3.59375000	0.11524927
4292	2.15625000	3.65625000	0.06725318
4293	2.15625000	3.53125000	0.16656891
4294	3.84375000	2.46875000	-0.09716520
4295	1.40625000	0.40625000	0.05796676
4296	1.34375000	0.34375000	0.00957967
4297	1.59375000	0.59375000	0.05532747
4298	1.65625000	0.65625000	0.00518084
4299	1.46875000	0.34375000	0.06119257
4300	1.65625000	0.53125000	0.03880743
4301	1.34375000	0.46875000	0.00879765
4302	1.53125000	0.65625000	-0.01124145
4303	1.40625000	1.03125000	-0.12326491
4304	1.46875000	1.09375000	-0.06598240

4305	3.40625000	0.40625000	0.05874878
4306	3.34375000	0.34375000	0.06823069
4307	3.40625000	0.37500000	0.07165200
4308	3.37500000	0.40625000	0.05669599
4309	3.37500000	0.34375000	0.07321603
4310	3.34375000	0.37500000	0.06148583
4311	3.46875000	0.37500000	-0.01221896
4312	3.37500000	0.46875000	-0.05708700
4313	3.62500000	3.03125000	-0.00742913
4314	3.53125000	3.12500000	0.00840665
4315	3.59375000	3.09375000	-0.09325513
4316	3.65625000	3.15625000	-0.07927664
4317	3.62500000	3.09375000	-0.09305963
4318	3.59375000	3.12500000	-0.08778104
4319	3.65625000	3.12500000	-0.08602151
4320	3.62500000	3.15625000	-0.08054741
4321	2.46875000	3.59375000	0.12502444
4322	2.40625000	3.53125000	0.17292278
4323	3.62500000	2.46875000	-0.01622678
4324	3.53125000	2.37500000	-0.03616813
4325	2.40625000	1.40625000	-0.03782991
4326	2.34375000	1.34375000	0.01876833
4327	2.34375000	1.46875000	-0.07820137
4328	2.46875000	1.34375000	-0.09071359
4329	1.90625000	0.28125000	0.05122190
4330	2.09375000	0.37500000	0.02218964
4331	1.62500000	1.71875000	-0.06265885
4332	1.71875000	1.62500000	-0.05210166
4333	2.15625000	3.90625000	0.01837732
4334	2.09375000	3.84375000	0.06637341
4335	2.28125000	3.90625000	-0.05356794
4336	2.21875000	3.84375000	-0.00518084
4337	1.71875000	3.62500000	0.10576735
4338	2.09375000	3.71875000	0.09071359
4339	2.15625000	3.78125000	0.04271750
4340	1.40625000	0.28125000	0.01270772
4341	1.28125000	0.40625000	-0.05219941
4342	1.34375000	1.09375000	-0.08201369
4343	1.40625000	1.15625000	-0.02473118
4344	3.37500000	0.28125000	-0.00752688
4345	3.28125000	0.37500000	-0.04271750
4346	3.28125000	3.40625000	0.07262952
4347	3.40625000	3.28125000	0.05112414
4348	3.71875000	3.12500000	0.01368524
4349	1.71875000	0.59375000	-0.01143695
4350	1.59375000	0.71875000	-0.06148583
4351	2.28125000	1.40625000	-0.02150538
4352	2.40625000	1.28125000	-0.03401760
4353	3.62500000	3.21875000	0.03010753
4354	2.40625000	1.78125000	-0.07605083
4355	2.40625000	3.78125000	-0.02541544
4356	2.34375000	3.71875000	0.02258065
4357	2.21875000	3.59375000	0.11847507
4358	2.28125000	3.65625000	0.07047898
4359	2.40625000	3.65625000	0.07390029
4360	2.34375000	3.59375000	0.12189638
4361	3.78125000	2.40625000	-0.16129032
4362	3.71875000	2.37500000	-0.09833822
4363	3.62500000	2.28125000	-0.03264907

4364	3.65625000	2.40625000	0.12140762
4365	3.59375000	2.34375000	0.10850440
4366	3.62500000	2.40625000	0.11906158
4367	3.59375000	2.37500000	0.11241447
4368	3.65625000	2.37500000	0.09169110
4369	3.62500000	2.34375000	0.11358749
4370	0.03125000	0.09375000	0.10684262
4371	0.09375000	0.03125000	0.11779081
4372	2.03125000	3.96875000	0.07732160
4373	3.40625000	3.46875000	0.13616813
4374	3.46875000	3.40625000	0.12541544
4375	1.96875000	0.43750000	-0.01163245
4376	2.03125000	0.46875000	-0.02130987
4377	1.53125000	1.56250000	-0.08338221
4378	1.56250000	1.53125000	-0.07986315
4379	1.53125000	1.59375000	-0.08181818
4380	1.59375000	1.53125000	-0.07478006
4381	2.53125000	1.96875000	-0.08846530
4382	2.40625000	3.96875000	-0.13782991
4383	2.46875000	3.90625000	-0.12316716
4384	1.56250000	3.53125000	0.12033236
4385	1.53125000	3.59375000	0.08514174
4386	1.59375000	3.53125000	0.12111437
4387	2.03125000	3.59375000	0.15102639
4388	2.09375000	3.53125000	0.16490714
4389	1.46875000	0.40625000	0.08377322
4390	1.59375000	0.53125000	0.07214076
4391	1.40625000	0.46875000	0.06070381
4392	1.53125000	0.59375000	0.04711632
4393	1.46875000	1.03125000	-0.11524927
4394	3.46875000	0.43750000	-0.03802542
4395	3.43750000	0.46875000	-0.05298143
4396	3.46875000	0.40625000	-0.02512219
4397	3.40625000	0.46875000	-0.05503421
4398	3.56250000	3.03125000	-0.00782014
4399	3.59375000	3.03125000	-0.00762463
4400	3.53125000	3.06250000	-0.00254154
4401	3.53125000	3.09375000	0.00293255
4402	2.46875000	3.53125000	0.17448680
4403	2.40625000	1.46875000	-0.08631476
4404	2.46875000	1.40625000	-0.09257087
4405	1.96875000	0.28125000	0.05317693
4406	2.03125000	0.31250000	0.04349951
4407	1.93750000	0.28125000	0.05219941
4408	2.06250000	0.34375000	0.03284457
4409	1.68750000	1.71875000	-0.05386119
4410	1.71875000	1.68750000	-0.05034213
4411	1.65625000	1.71875000	-0.05826002
4412	1.71875000	1.65625000	-0.05122190
4413	2.15625000	3.96875000	0.00606061
4414	2.28125000	3.96875000	-0.06588465
4415	2.21875000	3.90625000	-0.01739980
4416	1.71875000	3.68750000	0.09345064
4417	1.65625000	3.71875000	0.06363636
4418	1.71875000	3.65625000	0.09960899
4419	2.03125000	3.84375000	0.10195503
4420	2.03125000	3.71875000	0.12649071
4421	2.09375000	3.78125000	0.07849462
4422	1.34375000	0.28125000	-0.01309873

4423	1.28125000	0.34375000	-0.04868035
4424	1.34375000	1.03125000	-0.13128055
4425	1.28125000	1.09375000	-0.14007820
4426	3.31250000	0.28125000	-0.01749756
4427	3.28125000	0.31250000	-0.02922776
4428	3.34375000	0.28125000	-0.01251222
4429	3.28125000	0.34375000	-0.03597263
4430	3.28125000	3.34375000	0.03567937
4431	3.34375000	3.28125000	0.02492669
4432	1.71875000	0.65625000	-0.02825024
4433	1.65625000	0.71875000	-0.05327468
4434	2.28125000	1.34375000	0.02697947
4435	2.34375000	1.28125000	0.02072336
4436	3.28125000	3.46875000	0.10977517
4437	3.46875000	3.28125000	0.07732160
4438	3.71875000	3.18750000	0.02717498
4439	3.68750000	3.21875000	0.03264907
4440	3.71875000	3.15625000	0.02043011
4441	3.65625000	3.21875000	0.03137830
4442	1.46875000	0.28125000	0.03870968
4443	1.71875000	0.53125000	0.00537634
4444	1.46875000	1.15625000	-0.01671554
4445	1.40625000	1.21875000	-0.03802542
4446	2.28125000	1.46875000	-0.07018573
4447	2.46875000	1.78125000	-0.07917889
4448	2.21875000	3.78125000	0.00694037
4449	2.28125000	3.84375000	-0.04095797
4450	2.15625000	3.71875000	0.05493646
4451	2.28125000	3.71875000	0.02082111
4452	2.34375000	3.78125000	-0.02707722
4453	2.21875000	3.65625000	0.06881720
4454	2.46875000	3.78125000	-0.02385142
4455	2.46875000	3.65625000	0.07546432
4456	2.40625000	3.71875000	0.02424242
4457	2.21875000	3.53125000	0.16813294
4458	2.34375000	3.53125000	0.17135875
4459	2.28125000	3.59375000	0.12013685
4460	3.78125000	2.46875000	-0.10185728
4461	3.68750000	2.46875000	-0.01153470
4462	3.65625000	2.46875000	-0.01388074
4463	3.71875000	2.43750000	-0.03890518
4464	3.71875000	2.40625000	-0.06862170
4465	3.53125000	2.31250000	-0.04398827
4466	3.53125000	2.34375000	-0.04007820
4467	3.56250000	2.28125000	-0.04281525
4468	3.59375000	2.28125000	-0.03773216
4469	1.28125000	0.46875000	-0.06197458
4470	1.53125000	0.71875000	-0.06950147
4471	2.46875000	1.28125000	-0.08895406
4472	2.09375000	3.90625000	0.05405670
4473	3.34375000	3.40625000	0.08592375
4474	3.40625000	3.34375000	0.07517107
4475	1.90625000	0.34375000	0.02521994
4476	2.09375000	0.43750000	-0.00381232
4477	1.90625000	0.31250000	0.03822092
4478	2.09375000	0.40625000	0.00918866
4479	1.56250000	1.65625000	-0.07438905
4480	1.65625000	1.56250000	-0.06383187
4481	1.59375000	1.68750000	-0.06852395

4482	1.68750000	1.59375000	-0.05796676
4483	2.59375000	1.90625000	-0.11485826
4484	2.40625000	1.84375000	-0.07429130
4485	2.34375000	3.90625000	-0.08934506
4486	2.40625000	3.84375000	-0.07507331
4487	1.65625000	3.56250000	0.11652004
4488	1.68750000	3.59375000	0.11114370
4489	2.09375000	3.65625000	0.10303030
4490	2.15625000	3.59375000	0.11691105
4491	3.84375000	2.40625000	-0.10576735
4492	1.40625000	0.34375000	0.03538612
4493	1.34375000	0.40625000	0.00606061
4494	1.65625000	0.59375000	0.02199413
4495	1.59375000	0.65625000	-0.00303030
4496	1.40625000	1.09375000	-0.07399804
4497	3.43750000	0.34375000	-0.01065494
4498	3.34375000	0.43750000	-0.05229717
4499	3.40625000	0.31250000	-0.00909091
4500	3.31250000	0.40625000	-0.04750733
4501	3.65625000	3.06250000	-0.00039101
4502	3.68750000	3.09375000	0.00664712
4503	3.56250000	3.15625000	0.01564027
4504	3.59375000	3.18750000	0.02287390
4505	2.40625000	3.59375000	0.12346041
4506	3.59375000	2.43750000	-0.02287390
4507	3.56250000	2.40625000	-0.02952102
4508	2.34375000	1.40625000	-0.02971652
4509	2.40625000	1.34375000	-0.03597263
4510	2.15625000	3.84375000	0.03059629
4511	2.34375000	3.65625000	0.07223851
4512	3.68750000	2.34375000	-0.07644184
4513	3.65625000	2.31250000	-0.05454545
4514	3.73437500	2.37500000	-0.14584555
4515	3.64062500	2.37500000	0.13919844
4516	3.73437500	2.43750000	-0.08641251
4517	3.70312500	2.43750000	0.00860215
4518	3.67187500	2.31250000	-0.10205279
4519	3.70312500	2.37500000	-0.05083089
4520	3.67187500	2.37500000	0.04418377
4521	3.64062500	2.31250000	-0.00703812

End of Node Coordinates

Connectivity Matrix of Nodes

Triangular Index	Node 1	Node 2	Node 3
1	83	1512	1510
2	708	2693	1512
3	291	1510	2693
4	1512	2693	1510
5	84	1514	1517
6	292	2695	1514
7	708	1517	2695
8	1514	2695	1517
9	291	2693	2692
10	708	2695	2693
11	292	2692	2695
12	2693	2695	2692

13	27	1208	1206
14	408	2987	1208
15	406	1206	2987
16	1208	2987	1206
17	196	2128	2126
18	709	2992	2128
19	408	2126	2992
20	2128	2992	2126
21	83	1511	1513
22	406	2988	1511
23	709	1513	2988
24	1511	2988	1513
25	408	2992	2987
26	709	2988	2992
27	406	2987	2988
28	2992	2988	2987
29	28	1210	1213
30	410	2997	1210
31	413	1213	2997
32	1210	2997	1213
33	84	1519	1515
34	711	2999	1519
35	410	1515	2999
36	1519	2999	1515
37	196	2127	2129
38	413	3008	2127
39	711	2129	3008
40	2127	3008	2129
41	410	2999	2997
42	711	3008	2999
43	413	2997	3008
44	2999	3008	2997
45	83	1513	1512
46	709	3845	1513
47	708	1512	3845
48	1513	3845	1512
49	196	2129	2128
50	711	3847	2129
51	709	2128	3847
52	2129	3847	2128
53	84	1517	1519
54	708	3846	1517
55	711	1519	3846
56	1517	3846	1519
57	709	3847	3845
58	711	3846	3847
59	708	3845	3846
60	3847	3846	3845
61	11	1128	1126
62	328	2771	1128
63	326	1126	2771
64	1128	2771	1126
65	120	1696	1694
66	787	2776	1696
67	328	1694	2776
68	1696	2776	1694
69	118	1686	1688
70	326	2772	1686
71	787	1688	2772

72	1686	2772	1688
73	328	2776	2771
74	787	2772	2776
75	326	2771	2772
76	2776	2772	2771
77	60	1376	1374
78	576	3446	1376
79	574	1374	3446
80	1376	3446	1374
81	197	2135	2133
82	792	3455	2135
83	576	2133	3455
84	2135	3455	2133
85	120	1695	1698
86	574	3448	1695
87	792	1698	3448
88	1695	3448	1698
89	576	3455	3446
90	792	3448	3455
91	574	3446	3448
92	3455	3448	3446
93	27	1207	1209
94	407	2989	1207
95	409	1209	2989
96	1207	2989	1209
97	118	1689	1687
98	788	2990	1689
99	407	1687	2990
100	1689	2990	1687
101	197	2132	2134
102	409	2994	2132
103	788	2134	2994
104	2132	2994	2134
105	407	2990	2989
106	788	2994	2990
107	409	2989	2994
108	2990	2994	2989
109	120	1698	1696
110	792	3924	1698
111	787	1696	3924
112	1698	3924	1696
113	197	2134	2135
114	788	3925	2134
115	792	2135	3925
116	2134	3925	2135
117	118	1688	1689
118	787	3923	1688
119	788	1689	3923
120	1688	3923	1689
121	792	3925	3924
122	788	3923	3925
123	787	3924	3923
124	3925	3923	3924
125	12	1130	1133
126	330	2781	1130
127	333	1133	2781
128	1130	2781	1133
129	122	1709	1706
130	797	2783	1709

131	330	1706	2783
132	1709	2783	1706
133	125	1724	1726
134	333	2792	1724
135	797	1726	2792
136	1724	2792	1726
137	330	2783	2781
138	797	2792	2783
139	333	2781	2792
140	2783	2792	2781
141	28	1215	1211
142	415	3001	1215
143	411	1211	3001
144	1215	3001	1211
145	199	2146	2144
146	799	3012	2146
147	415	2144	3012
148	2146	3012	2144
149	122	1707	1711
150	411	3003	1707
151	799	1711	3003
152	1707	3003	1711
153	415	3012	3001
154	799	3003	3012
155	411	3001	3003
156	3012	3003	3001
157	60	1375	1377
158	575	3450	1375
159	577	1377	3450
160	1375	3450	1377
161	125	1728	1725
162	808	3452	1728
163	575	1725	3452
164	1728	3452	1725
165	199	2145	2147
166	577	3457	2145
167	808	2147	3457
168	2145	3457	2147
169	575	3452	3450
170	808	3457	3452
171	577	3450	3457
172	3452	3457	3450
173	122	1711	1709
174	799	3934	1711
175	797	1709	3934
176	1711	3934	1709
177	199	2147	2146
178	808	3937	2147
179	799	2146	3937
180	2147	3937	2146
181	125	1726	1728
182	797	3935	1726
183	808	1728	3935
184	1726	3935	1728
185	799	3937	3934
186	808	3935	3937
187	797	3934	3935
188	3937	3935	3934
189	27	1209	1208

190	409	2991	1209
191	408	1208	2991
192	1209	2991	1208
193	197	2136	2132
194	997	2995	2136
195	409	2132	2995
196	2136	2995	2132
197	196	2126	2130
198	408	2993	2126
199	997	2130	2993
200	2126	2993	2130
201	409	2995	2991
202	997	2993	2995
203	408	2991	2993
204	2995	2993	2991
205	60	1377	1376
206	577	3454	1377
207	576	1376	3454
208	1377	3454	1376
209	199	2149	2145
210	999	3458	2149
211	577	2145	3458
212	2149	3458	2145
213	197	2133	2137
214	576	3456	2133
215	999	2137	3456
216	2133	3456	2137
217	577	3458	3454
218	999	3456	3458
219	576	3454	3456
220	3458	3456	3454
221	28	1213	1215
222	413	3007	1213
223	415	1215	3007
224	1213	3007	1215
225	196	2131	2127
226	998	3009	2131
227	413	2127	3009
228	2131	3009	2127
229	199	2144	2148
230	415	3013	2144
231	998	2148	3013
232	2144	3013	2148
233	413	3009	3007
234	998	3013	3009
235	415	3007	3013
236	3009	3013	3007
237	197	2137	2136
238	999	4134	2137
239	997	2136	4134
240	2137	4134	2136
241	199	2148	2149
242	998	4135	2148
243	999	2149	4135
244	2148	4135	2149
245	196	2130	2131
246	997	4133	2130
247	998	2131	4133
248	2130	4133	2131

249	999	4135	4134
250	998	4133	4135
251	997	4134	4133
252	4135	4133	4134
253	6	1106	1104
254	306	2717	1106
255	304	1104	2717
256	1106	2717	1104
257	98	1580	1578
258	733	2722	1580
259	306	1578	2722
260	1580	2722	1578
261	96	1570	1572
262	304	2718	1570
263	733	1572	2718
264	1570	2718	1572
265	306	2722	2717
266	733	2718	2722
267	304	2717	2718
268	2722	2718	2717
269	221	2279	2277
270	738	3167	2279
271	476	2277	3167
272	2279	3167	2277
273	40	1266	1268
274	466	3140	1266
275	468	1268	3140
276	1266	3140	1268
277	96	1573	1571
278	734	3141	1573
279	466	1571	3141
280	1573	3141	1571
281	221	2276	2278
282	468	3145	2276
283	734	2278	3145
284	2276	3145	2278
285	466	3141	3140
286	734	3145	3141
287	468	3140	3145
288	3141	3145	3140
289	98	1582	1580
290	738	3870	1582
291	733	1580	3870
292	1582	3870	1580
293	221	2278	2279
294	734	3871	2278
295	738	2279	3871
296	2278	3871	2279
297	96	1572	1573
298	733	3869	1572
299	734	1573	3869
300	1572	3869	1573
301	738	3871	3870
302	734	3869	3871
303	733	3870	3869
304	3871	3869	3870
305	22	1180	1178
306	380	2906	1180
307	378	1178	2906

308	1180	2906	1178
309	172	1984	1982
310	922	2915	1984
311	380	1982	2915
312	1984	2915	1982
313	170	1970	1972
314	378	2908	1970
315	922	1972	2908
316	1970	2908	1972
317	380	2915	2906
318	922	2908	2915
319	378	2906	2908
320	2915	2908	2906
321	42	1275	1278
322	475	3162	1275
323	478	1278	3162
324	1275	3162	1278
325	170	1974	1971
326	924	3164	1974
327	475	1971	3164
328	1974	3164	1971
329	226	2306	2308
330	478	3172	2306
331	924	2308	3172
332	2306	3172	2308
333	475	3164	3162
334	924	3172	3164
335	478	3162	3172
336	3164	3172	3162
337	172	1986	1984
338	931	4059	1986
339	922	1984	4059
340	1986	4059	1984
341	226	2308	2309
342	924	4062	2308
343	931	2309	4062
344	2308	4062	2309
345	170	1972	1974
346	922	4058	1972
347	924	1974	4058
348	1972	4058	1974
349	931	4062	4059
350	924	4058	4062
351	922	4059	4058
352	4062	4058	4059
353	11	1127	1129
354	327	2773	1127
355	329	1129	2773
356	1127	2773	1129
357	119	1692	1690
358	789	2774	1692
359	327	1690	2774
360	1692	2774	1690
361	121	1700	1702
362	329	2778	1700
363	789	1702	2778
364	1700	2778	1702
365	327	2774	2773
366	789	2778	2774

367	329	2773	2778
368	2774	2778	2773
369	40	1269	1267
370	469	3142	1269
371	467	1267	3142
372	1269	3142	1267
373	222	2284	2282
374	790	3147	2284
375	469	2282	3147
376	2284	3147	2282
377	119	1691	1693
378	467	3143	1691
379	790	1693	3143
380	1691	3143	1693
381	469	3147	3142
382	790	3143	3147
383	467	3142	3143
384	3147	3143	3142
385	119	1693	1692
386	790	3926	1693
387	789	1692	3926
388	1693	3926	1692
389	222	2285	2284
390	794	3928	2285
391	790	2284	3928
392	2285	3928	2284
393	121	1702	1704
394	789	3927	1702
395	794	1704	3927
396	1702	3927	1704
397	790	3928	3926
398	794	3927	3928
399	789	3926	3927
400	3928	3927	3926
401	42	1278	1276
402	478	3166	1278
403	476	1276	3166
404	1278	3166	1276
405	226	2310	2306
406	1022	3173	2310
407	478	2306	3173
408	2310	3173	2306
409	221	2277	2281
410	476	3168	2277
411	1022	2281	3168
412	2277	3168	2281
413	478	3173	3166
414	1022	3168	3173
415	476	3166	3168
416	3173	3168	3166
417	61	1382	1383
418	582	3472	1382
419	583	1383	3472
420	1382	3472	1383
421	222	2287	2283
422	1023	3474	2287
423	582	2283	3474
424	2287	3474	2283
425	226	2307	2311

426	583	3476	2307
427	1023	2311	3476
428	2307	3476	2311
429	582	3474	3472
430	1023	3476	3474
431	583	3472	3476
432	3474	3476	3472
433	40	1268	1269
434	468	3144	1268
435	469	1269	3144
436	1268	3144	1269
437	221	2280	2276
438	1021	3146	2280
439	468	2276	3146
440	2280	3146	2276
441	222	2282	2286
442	469	3148	2282
443	1021	2286	3148
444	2282	3148	2286
445	468	3146	3144
446	1021	3148	3146
447	469	3144	3148
448	3146	3148	3144
449	226	2311	2310
450	1023	4159	2311
451	1022	2310	4159
452	2311	4159	2310
453	222	2286	2287
454	1021	4158	2286
455	1023	2287	4158
456	2286	4158	2287
457	221	2281	2280
458	1022	4157	2281
459	1021	2280	4157
460	2281	4157	2280
461	1023	4158	4159
462	1021	4157	4158
463	1022	4159	4157
464	4158	4157	4159
465	7	1108	1111
466	308	2727	1108
467	311	1111	2727
468	1108	2727	1111
469	100	1593	1590
470	743	2729	1593
471	308	1590	2729
472	1593	2729	1590
473	103	1608	1610
474	311	2738	1608
475	743	1610	2738
476	1608	2738	1610
477	308	2729	2727
478	743	2738	2729
479	311	2727	2738
480	2729	2738	2727
481	231	2338	2336
482	745	3206	2338
483	489	2336	3206
484	2338	3206	2336

485	47	1304	1306
486	504	3248	1304
487	506	1306	3248
488	1304	3248	1306
489	103	1612	1609
490	754	3250	1612
491	504	1609	3250
492	1612	3250	1609
493	231	2337	2339
494	506	3257	2337
495	754	2339	3257
496	2337	3257	2339
497	504	3250	3248
498	754	3257	3250
499	506	3248	3257
500	3250	3257	3248
501	100	1595	1593
502	745	3880	1595
503	743	1593	3880
504	1595	3880	1593
505	231	2339	2338
506	754	3883	2339
507	745	2338	3883
508	2339	3883	2338
509	103	1610	1612
510	743	3881	1610
511	754	1612	3881
512	1610	3881	1612
513	745	3883	3880
514	754	3881	3883
515	743	3880	3881
516	3883	3881	3880
517	12	1135	1131
518	335	2785	1135
519	331	1131	2785
520	1135	2785	1131
521	127	1738	1736
522	801	2796	1738
523	335	1736	2796
524	1738	2796	1736
525	123	1712	1715
526	331	2787	1712
527	801	1715	2787
528	1712	2787	1715
529	335	2796	2785
530	801	2787	2796
531	331	2785	2787
532	2796	2787	2785
533	44	1287	1291
534	487	3199	1287
535	491	1291	3199
536	1287	3199	1291
537	123	1717	1713
538	803	3201	1717
539	487	1713	3201
540	1717	3201	1713
541	233	2348	2350
542	491	3210	2348
543	803	2350	3210

544	2348	3210	2350
545	487	3201	3199
546	803	3210	3201
547	491	3199	3210
548	3201	3210	3199
549	123	1715	1717
550	801	3940	1715
551	803	1717	3940
552	1715	3940	1717
553	812	3943	3941
554	803	3940	3943
555	801	3941	3940
556	3943	3940	3941
557	22	1179	1181
558	379	2910	1179
559	381	1181	2910
560	1179	2910	1181
561	171	1978	1976
562	926	2912	1978
563	379	1976	2912
564	1978	2912	1976
565	173	1988	1990
566	381	2917	1988
567	926	1990	2917
568	1988	2917	1990
569	379	2912	2910
570	926	2917	2912
571	381	2910	2917
572	2912	2917	2910
573	47	1308	1305
574	508	3252	1308
575	505	1305	3252
576	1308	3252	1305
577	242	2404	2402
578	928	3262	2404
579	508	2402	3262
580	2404	3262	2402
581	171	1977	1980
582	505	3254	1977
583	928	1980	3254
584	1977	3254	1980
585	508	3262	3252
586	928	3254	3262
587	505	3252	3254
588	3262	3254	3252
589	63	1393	1395
590	593	3504	1393
591	595	1395	3504
592	1393	3504	1395
593	173	1992	1989
594	933	3506	1992
595	593	1989	3506
596	1992	3506	1989
597	242	2403	2405
598	595	3511	2403
599	933	2405	3511
600	2403	3511	2405
601	593	3506	3504
602	933	3511	3506

603	595	3504	3511
604	3506	3511	3504
605	171	1980	1978
606	928	4064	1980
607	926	1978	4064
608	1980	4064	1978
609	242	2405	2404
610	933	4068	2405
611	928	2404	4068
612	2405	4068	2404
613	173	1990	1992
614	926	4065	1990
615	933	1992	4065
616	1990	4065	1992
617	928	4068	4064
618	933	4065	4068
619	926	4064	4065
620	4068	4065	4064
621	44	1291	1289
622	491	3205	1291
623	489	1289	3205
624	1291	3205	1289
625	233	2352	2348
626	1032	3211	2352
627	491	2348	3211
628	2352	3211	2348
629	231	2336	2340
630	489	3207	2336
631	1032	2340	3207
632	2336	3207	2340
633	491	3211	3205
634	1032	3207	3211
635	489	3205	3207
636	3211	3207	3205
637	63	1395	1394
638	595	3508	1395
639	594	1394	3508
640	1395	3508	1394
641	242	2407	2403
642	1035	3512	2407
643	595	2403	3512
644	2407	3512	2403
645	233	2349	2353
646	594	3510	2349
647	1035	2353	3510
648	2349	3510	2353
649	595	3512	3508
650	1035	3510	3512
651	594	3508	3510
652	3512	3510	3508
653	47	1306	1308
654	506	3256	1306
655	508	1308	3256
656	1306	3256	1308
657	231	2341	2337
658	1033	3258	2341
659	506	2337	3258
660	2341	3258	2337
661	242	2402	2406

662	508	3263	2402
663	1033	2406	3263
664	2402	3263	2406
665	506	3258	3256
666	1033	3263	3258
667	508	3256	3263
668	3258	3263	3256
669	233	2353	2352
670	1035	4170	2353
671	1032	2352	4170
672	2353	4170	2352
673	242	2406	2407
674	1033	4171	2406
675	1035	2407	4171
676	2406	4171	2407
677	231	2340	2341
678	1032	4169	2340
679	1033	2341	4169
680	2340	4169	2341
681	1035	4171	4170
682	1033	4169	4171
683	1032	4170	4169
684	4171	4169	4170
685	11	1129	1128
686	329	2775	1129
687	328	1128	2775
688	1129	2775	1128
689	121	1703	1700
690	791	2779	1703
691	329	1700	2779
692	1703	2779	1700
693	120	1694	1697
694	328	2777	1694
695	791	1697	2777
696	1694	2777	1697
697	329	2779	2775
698	791	2777	2779
699	328	2775	2777
700	2779	2777	2775
701	60	1374	1378
702	574	3447	1374
703	578	1378	3447
704	1374	3447	1378
705	120	1699	1695
706	793	3449	1699
707	574	1695	3449
708	1699	3449	1695
709	269	2564	2566
710	578	3460	2564
711	793	2566	3460
712	2564	3460	2566
713	574	3449	3447
714	793	3460	3449
715	578	3447	3460
716	3449	3460	3447
717	121	1705	1703
718	795	3930	1705
719	791	1703	3930
720	1705	3930	1703

721	269	2566	2567
722	793	3931	2566
723	795	2567	3931
724	2566	3931	2567
725	120	1697	1699
726	791	3929	1697
727	793	1699	3929
728	1697	3929	1699
729	795	3931	3930
730	793	3929	3931
731	791	3930	3929
732	3931	3929	3930
733	22	1181	1180
734	381	2914	1181
735	380	1180	2914
736	1181	2914	1180
737	173	1991	1988
738	930	2918	1991
739	381	1988	2918
740	1991	2918	1988
741	172	1982	1985
742	380	2916	1982
743	930	1985	2916
744	1982	2916	1985
745	381	2918	2914
746	930	2916	2918
747	380	2914	2916
748	2918	2916	2914
749	63	1397	1393
750	597	3505	1397
751	593	1393	3505
752	1397	3505	1393
753	271	2579	2577
754	934	3516	2579
755	597	2577	3516
756	2579	3516	2577
757	173	1989	1993
758	593	3507	1989
759	934	1993	3507
760	1989	3507	1993
761	597	3516	3505
762	934	3507	3516
763	593	3505	3507
764	3516	3507	3505
765	173	1993	1991
766	934	4071	1993
767	930	1991	4071
768	1993	4071	1991
769	271	2578	2579
770	932	4072	2578
771	934	2579	4072
772	2578	4072	2579
773	172	1985	1987
774	930	4070	1985
775	932	1987	4070
776	1985	4070	1987
777	934	4072	4071
778	932	4070	4072
779	930	4071	4070

780	4072	4070	4071
781	12	1133	1135
782	333	2791	1133
783	335	1135	2791
784	1133	2791	1135
785	125	1727	1724
786	807	2793	1727
787	333	1724	2793
788	1727	2793	1724
789	127	1736	1739
790	335	2797	1736
791	807	1739	2797
792	1736	2797	1739
793	333	2793	2791
794	807	2797	2793
795	335	2791	2797
796	2793	2797	2791
797	60	1379	1375
798	579	3451	1379
799	575	1375	3451
800	1379	3451	1375
801	270	2572	2570
802	809	3462	2572
803	579	2570	3462
804	2572	3462	2570
805	125	1725	1729
806	575	3453	1725
807	809	1729	3453
808	1725	3453	1729
809	579	3462	3451
810	809	3453	3462
811	575	3451	3453
812	3462	3453	3451
813	63	1392	1396
814	592	3501	1392
815	596	1396	3501
816	1392	3501	1396
817	127	1741	1737
818	813	3503	1741
819	592	1737	3503
820	1741	3503	1737
821	270	2571	2573
822	596	3514	2571
823	813	2573	3514
824	2571	3514	2573
825	592	3503	3501
826	813	3514	3503
827	596	3501	3514
828	3503	3514	3501
829	125	1729	1727
830	809	3947	1729
831	807	1727	3947
832	1729	3947	1727
833	270	2573	2572
834	813	3949	2573
835	809	2572	3949
836	2573	3949	2572
837	127	1739	1741
838	807	3948	1739

839	813	1741	3948
840	1739	3948	1741
841	809	3949	3947
842	813	3948	3949
843	807	3947	3948
844	3949	3948	3947
845	61	1385	1384
846	585	3477	1385
847	584	1384	3477
848	1385	3477	1384
849	271	2580	2576
850	1070	3481	2580
851	585	2576	3481
852	2580	3481	2576
853	269	2565	2569
854	584	3479	2565
855	1070	2569	3479
856	2565	3479	2569
857	585	3481	3477
858	1070	3479	3481
859	584	3477	3479
860	3481	3479	3477
861	63	1396	1397
862	596	3513	1396
863	597	1397	3513
864	1396	3513	1397
865	270	2575	2571
866	1071	3515	2575
867	596	2571	3515
868	2575	3515	2571
869	271	2577	2581
870	597	3517	2577
871	1071	2581	3517
872	2577	3517	2581
873	596	3515	3513
874	1071	3517	3515
875	597	3513	3517
876	3515	3517	3513
877	60	1378	1379
878	578	3459	1378
879	579	1379	3459
880	1378	3459	1379
881	269	2568	2564
882	1069	3461	2568
883	578	2564	3461
884	2568	3461	2564
885	270	2570	2574
886	579	3463	2570
887	1069	2574	3463
888	2570	3463	2574
889	578	3461	3459
890	1069	3463	3461
891	579	3459	3463
892	3461	3463	3459
893	271	2581	2580
894	1071	4207	2581
895	1070	2580	4207
896	2581	4207	2580
897	270	2574	2575

898	1069	4206	2574
899	1071	2575	4206
900	2574	4206	2575
901	269	2569	2568
902	1070	4205	2569
903	1069	2568	4205
904	2569	4205	2568
905	1071	4206	4207
906	1069	4205	4206
907	1070	4207	4205
908	4206	4205	4207
909	3	1096	1095
910	296	2699	1096
911	295	1095	2699
912	1096	2699	1095
913	88	1534	1532
914	715	2701	1534
915	296	1532	2701
916	1534	2701	1532
917	87	1528	1530
918	295	2700	1528
919	715	1530	2700
920	1528	2700	1530
921	296	2701	2699
922	715	2700	2701
923	295	2699	2700
924	2701	2700	2699
925	32	1230	1228
926	430	3041	1230
927	428	1228	3041
928	1230	3041	1228
929	203	2171	2169
930	717	3046	2171
931	430	2169	3046
932	2171	3046	2169
933	88	1533	1535
934	428	3042	1533
935	717	1535	3042
936	1533	3042	1535
937	430	3046	3041
938	717	3042	3046
939	428	3041	3042
940	3046	3042	3041
941	31	1224	1226
942	424	3032	1224
943	426	1226	3032
944	1224	3032	1226
945	87	1531	1529
946	716	3033	1531
947	424	1529	3033
948	1531	3033	1529
949	203	2168	2170
950	426	3037	2168
951	716	2170	3037
952	2168	3037	2170
953	424	3033	3032
954	716	3037	3033
955	426	3032	3037
956	3033	3037	3032

957	88	1535	1534
958	717	3852	1535
959	715	1534	3852
960	1535	3852	1534
961	203	2170	2171
962	716	3853	2170
963	717	2171	3853
964	2170	3853	2171
965	87	1530	1531
966	715	3851	1530
967	716	1531	3851
968	1530	3851	1531
969	717	3853	3852
970	716	3851	3853
971	715	3852	3851
972	3853	3851	3852
973	16	1150	1148
974	350	2825	1150
975	348	1148	2825
976	1150	2825	1148
977	142	1812	1810
978	841	2830	1812
979	350	1810	2830
980	1812	2830	1810
981	140	1802	1804
982	348	2826	1802
983	841	1804	2826
984	1802	2826	1804
985	350	2830	2825
986	841	2826	2830
987	348	2825	2826
988	2830	2826	2825
989	67	1419	1417
990	619	3576	1419
991	617	1417	3576
992	1419	3576	1417
993	205	2183	2181
994	846	3583	2183
995	619	2181	3583
996	2183	3583	2181
997	142	1811	1814
998	617	3578	1811
999	846	1814	3578
1000	1811	3578	1814
1001	619	3583	3576
1002	846	3578	3583
1003	617	3576	3578
1004	3583	3578	3576
1005	32	1229	1231
1006	429	3043	1229
1007	431	1231	3043
1008	1229	3043	1231
1009	140	1805	1803
1010	842	3044	1805
1011	429	1803	3044
1012	1805	3044	1803
1013	205	2180	2182
1014	431	3048	2180
1015	842	2182	3048

1016	2180	3048	2182
1017	429	3044	3043
1018	842	3048	3044
1019	431	3043	3048
1020	3044	3048	3043
1021	142	1814	1812
1022	846	3978	1814
1023	841	1812	3978
1024	1814	3978	1812
1025	205	2182	2183
1026	842	3979	2182
1027	846	2183	3979
1028	2182	3979	2183
1029	140	1804	1805
1030	841	3977	1804
1031	842	1805	3977
1032	1804	3977	1805
1033	846	3979	3978
1034	842	3977	3979
1035	841	3978	3977
1036	3979	3977	3978
1037	15	1144	1146
1038	344	2816	1144
1039	346	1146	2816
1040	1144	2816	1146
1041	136	1784	1782
1042	832	2817	1784
1043	344	1782	2817
1044	1784	2817	1782
1045	138	1790	1792
1046	346	2821	1790
1047	832	1792	2821
1048	1790	2821	1792
1049	344	2817	2816
1050	832	2821	2817
1051	346	2816	2821
1052	2817	2821	2816
1053	31	1227	1225
1054	427	3034	1227
1055	425	1225	3034
1056	1227	3034	1225
1057	204	2176	2174
1058	833	3039	2176
1059	427	2174	3039
1060	2176	3039	2174
1061	136	1783	1785
1062	425	3035	1783
1063	833	1785	3035
1064	1783	3035	1785
1065	427	3039	3034
1066	833	3035	3039
1067	425	3034	3035
1068	3039	3035	3034
1069	204	2175	2177
1070	618	3581	2175
1071	837	2177	3581
1072	2175	3581	2177
1073	136	1785	1784
1074	833	3968	1785

1075	832	1784	3968
1076	1785	3968	1784
1077	204	2177	2176
1078	837	3970	2177
1079	833	2176	3970
1080	2177	3970	2176
1081	138	1792	1794
1082	832	3969	1792
1083	837	1794	3969
1084	1792	3969	1794
1085	833	3970	3968
1086	837	3969	3970
1087	832	3968	3969
1088	3970	3969	3968
1089	32	1231	1230
1090	431	3045	1231
1091	430	1230	3045
1092	1231	3045	1230
1093	205	2184	2180
1094	1004	3049	2184
1095	431	2180	3049
1096	2184	3049	2180
1097	203	2169	2173
1098	430	3047	2169
1099	1004	2173	3047
1100	2169	3047	2173
1101	431	3049	3045
1102	1004	3047	3049
1103	430	3045	3047
1104	3049	3047	3045
1105	67	1418	1419
1106	618	3580	1418
1107	619	1419	3580
1108	1418	3580	1419
1109	204	2179	2175
1110	1005	3582	2179
1111	618	2175	3582
1112	2179	3582	2175
1113	205	2181	2185
1114	619	3584	2181
1115	1005	2185	3584
1116	2181	3584	2185
1117	618	3582	3580
1118	1005	3584	3582
1119	619	3580	3584
1120	3582	3584	3580
1121	31	1226	1227
1122	426	3036	1226
1123	427	1227	3036
1124	1226	3036	1227
1125	203	2172	2168
1126	1003	3038	2172
1127	426	2168	3038
1128	2172	3038	2168
1129	204	2174	2178
1130	427	3040	2174
1131	1003	2178	3040
1132	2174	3040	2178
1133	426	3038	3036

1134	1003	3040	3038
1135	427	3036	3040
1136	3038	3040	3036
1137	205	2185	2184
1138	1005	4141	2185
1139	1004	2184	4141
1140	2185	4141	2184
1141	204	2178	2179
1142	1003	4140	2178
1143	1005	2179	4140
1144	2178	4140	2179
1145	203	2173	2172
1146	1004	4139	2173
1147	1003	2172	4139
1148	2173	4139	2172
1149	1005	4140	4141
1150	1003	4139	4140
1151	1004	4141	4139
1152	4140	4139	4141
1153	9	1120	1118
1154	320	2753	1120
1155	318	1118	2753
1156	1120	2753	1118
1157	112	1656	1654
1158	769	2758	1656
1159	320	1654	2758
1160	1656	2758	1654
1161	110	1646	1648
1162	318	2754	1646
1163	769	1648	2754
1164	1646	2754	1648
1165	320	2758	2753
1166	769	2754	2758
1167	318	2753	2754
1168	2758	2754	2753
1169	56	1352	1350
1170	552	3374	1352
1171	550	1350	3374
1172	1352	3374	1350
1173	257	2495	2493
1174	774	3383	2495
1175	552	2493	3383
1176	2495	3383	2493
1177	112	1655	1658
1178	550	3376	1655
1179	774	1658	3376
1180	1655	3376	1658
1181	552	3383	3374
1182	774	3376	3383
1183	550	3374	3376
1184	3383	3376	3374
1185	54	1342	1344
1186	542	3356	1342
1187	544	1344	3356
1188	1342	3356	1344
1189	110	1649	1647
1190	770	3357	1649
1191	542	1647	3357
1192	1649	3357	1647

1193	257	2492	2494
1194	544	3361	2492
1195	770	2494	3361
1196	2492	3361	2494
1197	542	3357	3356
1198	770	3361	3357
1199	544	3356	3361
1200	3357	3361	3356
1201	112	1658	1656
1202	774	3906	1658
1203	769	1656	3906
1204	1658	3906	1656
1205	257	2494	2495
1206	770	3907	2494
1207	774	2495	3907
1208	2494	3907	2495
1209	110	1648	1649
1210	769	3905	1648
1211	770	1649	3905
1212	1648	3905	1649
1213	774	3907	3906
1214	770	3905	3907
1215	769	3906	3905
1216	3907	3905	3906
1217	23	1187	1185
1218	387	2928	1187
1219	385	1185	2928
1220	1187	2928	1185
1221	179	2026	2024
1222	944	2935	2026
1223	387	2024	2935
1224	2026	2935	2024
1225	177	2012	2014
1226	385	2930	2012
1227	944	2014	2930
1228	2012	2930	2014
1229	387	2935	2928
1230	944	2930	2935
1231	385	2928	2930
1232	2935	2930	2928
1233	69	1431	1429
1234	631	3612	1431
1235	629	1429	3612
1236	1431	3612	1429
1237	262	2525	2523
1238	951	3619	2525
1239	631	2523	3619
1240	2525	3619	2523
1241	179	2025	2028
1242	629	3614	2025
1243	951	2028	3614
1244	2025	3614	2028
1245	631	3619	3612
1246	951	3614	3619
1247	629	3612	3614
1248	3619	3614	3612
1249	56	1351	1354
1250	551	3378	1351
1251	554	1354	3378

1252	1351	3378	1354
1253	177	2016	2013
1254	946	3380	2016
1255	551	2013	3380
1256	2016	3380	2013
1257	262	2522	2524
1258	554	3388	2522
1259	946	2524	3388
1260	2522	3388	2524
1261	551	3380	3378
1262	946	3388	3380
1263	554	3378	3388
1264	3380	3388	3378
1265	179	2028	2026
1266	951	4083	2028
1267	944	2026	4083
1268	2028	4083	2026
1269	262	2524	2525
1270	946	4086	2524
1271	951	2525	4086
1272	2524	4086	2525
1273	177	2014	2016
1274	944	4082	2014
1275	946	2016	4082
1276	2014	4082	2016
1277	951	4086	4083
1278	946	4082	4086
1279	944	4083	4082
1280	4086	4082	4083
1281	16	1149	1151
1282	349	2827	1149
1283	351	1151	2827
1284	1149	2827	1151
1285	141	1808	1806
1286	843	2828	1808
1287	349	1806	2828
1288	1808	2828	1806
1289	143	1816	1818
1290	351	2832	1816
1291	843	1818	2832
1292	1816	2832	1818
1293	349	2828	2827
1294	843	2832	2828
1295	351	2827	2832
1296	2828	2832	2827
1297	54	1345	1343
1298	545	3358	1345
1299	543	1343	3358
1300	1345	3358	1343
1301	258	2500	2498
1302	844	3363	2500
1303	545	2498	3363
1304	2500	3363	2498
1305	141	1807	1809
1306	543	3359	1807
1307	844	1809	3359
1308	1807	3359	1809
1309	545	3363	3358
1310	844	3359	3363

1311	543	3358	3359
1312	3363	3359	3358
1313	69	1428	1430
1314	628	3608	1428
1315	630	1430	3608
1316	1428	3608	1430
1317	143	1820	1817
1318	848	3610	1820
1319	628	1817	3610
1320	1820	3610	1817
1321	258	2499	2501
1322	630	3617	2499
1323	848	2501	3617
1324	2499	3617	2501
1325	628	3610	3608
1326	848	3617	3610
1327	630	3608	3617
1328	3610	3617	3608
1329	141	1809	1808
1330	844	3980	1809
1331	843	1808	3980
1332	1809	3980	1808
1333	258	2501	2500
1334	848	3982	2501
1335	844	2500	3982
1336	2501	3982	2500
1337	143	1818	1820
1338	843	3981	1818
1339	848	1820	3981
1340	1818	3981	1820
1341	844	3982	3980
1342	848	3981	3982
1343	843	3980	3981
1344	3982	3981	3980
1345	56	1354	1352
1346	554	3382	1354
1347	552	1352	3382
1348	1354	3382	1352
1349	262	2526	2522
1350	1058	3389	2526
1351	554	2522	3389
1352	2526	3389	2522
1353	257	2493	2497
1354	552	3384	2493
1355	1058	2497	3384
1356	2493	3384	2497
1357	554	3389	3382
1358	1058	3384	3389
1359	552	3382	3384
1360	3389	3384	3382
1361	69	1430	1431
1362	630	3616	1430
1363	631	1431	3616
1364	1430	3616	1431
1365	258	2503	2499
1366	1059	3618	2503
1367	630	2499	3618
1368	2503	3618	2499
1369	262	2523	2527

1370	631	3620	2523
1371	1059	2527	3620
1372	2523	3620	2527
1373	630	3618	3616
1374	1059	3620	3618
1375	631	3616	3620
1376	3618	3620	3616
1377	54	1344	1345
1378	544	3360	1344
1379	545	1345	3360
1380	1344	3360	1345
1381	257	2496	2492
1382	1057	3362	2496
1383	544	2492	3362
1384	2496	3362	2492
1385	258	2498	2502
1386	545	3364	2498
1387	1057	2502	3364
1388	2498	3364	2502
1389	544	3362	3360
1390	1057	3364	3362
1391	545	3360	3364
1392	3362	3364	3360
1393	262	2527	2526
1394	1059	4195	2527
1395	1058	2526	4195
1396	2527	4195	2526
1397	258	2502	2503
1398	1057	4194	2502
1399	1059	2503	4194
1400	2502	4194	2503
1401	257	2497	2496
1402	1058	4193	2497
1403	1057	2496	4193
1404	2497	4193	2496
1405	1059	4194	4195
1406	1057	4193	4194
1407	1058	4195	4193
1408	4194	4193	4195
1409	6	1105	1107
1410	305	2719	1105
1411	307	1107	2719
1412	1105	2719	1107
1413	97	1576	1574
1414	735	2720	1576
1415	305	1574	2720
1416	1576	2720	1574
1417	99	1584	1586
1418	307	2724	1584
1419	735	1586	2724
1420	1584	2724	1586
1421	305	2720	2719
1422	735	2724	2720
1423	307	2719	2724
1424	2720	2724	2719
1425	41	1272	1270
1426	472	3149	1272
1427	470	1270	3149
1428	1272	3149	1270

1429	223	2290	2288
1430	736	3154	2290
1431	472	2288	3154
1432	2290	3154	2288
1433	97	1575	1577
1434	470	3150	1575
1435	736	1577	3150
1436	1575	3150	1577
1437	472	3154	3149
1438	736	3150	3154
1439	470	3149	3150
1440	3154	3150	3149
1441	43	1280	1282
1442	480	3176	1280
1443	482	1282	3176
1444	1280	3176	1282
1445	99	1588	1585
1446	740	3178	1588
1447	480	1585	3178
1448	1588	3178	1585
1449	223	2289	2291
1450	482	3185	2289
1451	740	2291	3185
1452	2289	3185	2291
1453	480	3178	3176
1454	740	3185	3178
1455	482	3176	3185
1456	3178	3185	3176
1457	97	1577	1576
1458	736	3872	1577
1459	735	1576	3872
1460	1577	3872	1576
1461	223	2291	2290
1462	740	3874	2291
1463	736	2290	3874
1464	2291	3874	2290
1465	99	1586	1588
1466	735	3873	1586
1467	740	1588	3873
1468	1586	3873	1588
1469	736	3874	3872
1470	740	3873	3874
1471	735	3872	3873
1472	3874	3873	3872
1473	15	1147	1145
1474	347	2818	1147
1475	345	1145	2818
1476	1147	2818	1145
1477	139	1798	1796
1478	834	2823	1798
1479	347	1796	2823
1480	1798	2823	1796
1481	137	1786	1788
1482	345	2819	1786
1483	834	1788	2819
1484	1786	2819	1788
1485	347	2823	2818
1486	834	2819	2823
1487	345	2818	2819

1488	2823	2819	2818
1489	68	1424	1422
1490	624	3590	1424
1491	622	1422	3590
1492	1424	3590	1422
1493	224	2297	2295
1494	839	3599	2297
1495	624	2295	3599
1496	2297	3599	2295
1497	139	1797	1800
1498	622	3592	1797
1499	839	1800	3592
1500	1797	3592	1800
1501	624	3599	3590
1502	839	3592	3599
1503	622	3590	3592
1504	3599	3592	3590
1505	41	1271	1273
1506	471	3151	1271
1507	473	1273	3151
1508	1271	3151	1273
1509	137	1789	1787
1510	835	3152	1789
1511	471	1787	3152
1512	1789	3152	1787
1513	224	2294	2296
1514	473	3156	2294
1515	835	2296	3156
1516	2294	3156	2296
1517	471	3152	3151
1518	835	3156	3152
1519	473	3151	3156
1520	3152	3156	3151
1521	139	1800	1798
1522	839	3972	1800
1523	834	1798	3972
1524	1800	3972	1798
1525	224	2296	2297
1526	835	3973	2296
1527	839	2297	3973
1528	2296	3973	2297
1529	137	1788	1789
1530	834	3971	1788
1531	835	1789	3971
1532	1788	3971	1789
1533	839	3973	3972
1534	835	3971	3973
1535	834	3972	3971
1536	3973	3971	3972
1537	23	1184	1186
1538	384	2924	1184
1539	386	1186	2924
1540	1184	2924	1186
1541	176	2008	2006
1542	940	2926	2008
1543	384	2006	2926
1544	2008	2926	2006
1545	178	2018	2020
1546	386	2933	2018

1547	940	2020	2933
1548	2018	2933	2020
1549	384	2926	2924
1550	940	2933	2926
1551	386	2924	2933
1552	2926	2933	2924
1553	43	1284	1281
1554	484	3180	1284
1555	481	1281	3180
1556	1284	3180	1281
1557	228	2320	2318
1558	942	3190	2320
1559	484	2318	3190
1560	2320	3190	2318
1561	176	2007	2010
1562	481	3182	2007
1563	942	2010	3182
1564	2007	3182	2010
1565	484	3190	3180
1566	942	3182	3190
1567	481	3180	3182
1568	3190	3182	3180
1569	68	1423	1425
1570	623	3594	1423
1571	625	1425	3594
1572	1423	3594	1425
1573	178	2022	2019
1574	949	3596	2022
1575	623	2019	3596
1576	2022	3596	2019
1577	228	2319	2321
1578	625	3601	2319
1579	949	2321	3601
1580	2319	3601	2321
1581	623	3596	3594
1582	949	3601	3596
1583	625	3594	3601
1584	3596	3601	3594
1585	176	2010	2008
1586	942	4076	2010
1587	940	2008	4076
1588	2010	4076	2008
1589	228	2321	2320
1590	949	4080	2321
1591	942	2320	4080
1592	2321	4080	2320
1593	178	2020	2022
1594	940	4077	2020
1595	949	2022	4077
1596	2020	4077	2022
1597	942	4080	4076
1598	949	4077	4080
1599	940	4076	4077
1600	4080	4077	4076
1601	41	1273	1272
1602	473	3153	1273
1603	472	1272	3153
1604	1273	3153	1272
1605	224	2298	2294

1606	1024	3157	2298
1607	473	2294	3157
1608	2298	3157	2294
1609	223	2288	2292
1610	472	3155	2288
1611	1024	2292	3155
1612	2288	3155	2292
1613	473	3157	3153
1614	1024	3155	3157
1615	472	3153	3155
1616	3157	3155	3153
1617	68	1425	1424
1618	625	3598	1425
1619	624	1424	3598
1620	1425	3598	1424
1621	228	2323	2319
1622	1026	3602	2323
1623	625	2319	3602
1624	2323	3602	2319
1625	224	2295	2299
1626	624	3600	2295
1627	1026	2299	3600
1628	2295	3600	2299
1629	625	3602	3598
1630	1026	3600	3602
1631	624	3598	3600
1632	3602	3600	3598
1633	43	1282	1284
1634	482	3184	1282
1635	484	1284	3184
1636	1282	3184	1284
1637	223	2293	2289
1638	1025	3186	2293
1639	482	2289	3186
1640	2293	3186	2289
1641	228	2318	2322
1642	484	3191	2318
1643	1025	2322	3191
1644	2318	3191	2322
1645	482	3186	3184
1646	1025	3191	3186
1647	484	3184	3191
1648	3186	3191	3184
1649	224	2299	2298
1650	1026	4161	2299
1651	1024	2298	4161
1652	2299	4161	2298
1653	228	2322	2323
1654	1025	4162	2322
1655	1026	2323	4162
1656	2322	4162	2323
1657	223	2292	2293
1658	1024	4160	2292
1659	1025	2293	4160
1660	2292	4160	2293
1661	1026	4162	4161
1662	1025	4160	4162
1663	1024	4161	4160
1664	4162	4160	4161

1665	16	1151	1150
1666	351	2829	1151
1667	350	1150	2829
1668	1151	2829	1150
1669	143	1819	1816
1670	845	2833	1819
1671	351	1816	2833
1672	1819	2833	1816
1673	142	1810	1813
1674	350	2831	1810
1675	845	1813	2831
1676	1810	2831	1813
1677	351	2833	2829
1678	845	2831	2833
1679	350	2829	2831
1680	2833	2831	2829
1681	69	1432	1428
1682	632	3609	1432
1683	628	1428	3609
1684	1432	3609	1428
1685	276	2609	2607
1686	849	3622	2609
1687	632	2607	3622
1688	2609	3622	2607
1689	143	1817	1821
1690	628	3611	1817
1691	849	1821	3611
1692	1817	3611	1821
1693	632	3622	3609
1694	849	3611	3622
1695	628	3609	3611
1696	3622	3611	3609
1697	67	1417	1421
1698	617	3577	1417
1699	621	1421	3577
1700	1417	3577	1421
1701	142	1815	1811
1702	847	3579	1815
1703	617	1811	3579
1704	1815	3579	1811
1705	276	2606	2608
1706	621	3588	2606
1707	847	2608	3588
1708	2606	3588	2608
1709	617	3579	3577
1710	847	3588	3579
1711	621	3577	3588
1712	3579	3588	3577
1713	143	1821	1819
1714	849	3984	1821
1715	845	1819	3984
1716	1821	3984	1819
1717	276	2608	2609
1718	847	3985	2608
1719	849	2609	3985
1720	2608	3985	2609
1721	142	1813	1815
1722	845	3983	1813
1723	847	1815	3983

1724	1813	3983	1815
1725	849	3985	3984
1726	847	3983	3985
1727	845	3984	3983
1728	3985	3983	3984
1729	23	1186	1187
1730	386	2932	1186
1731	387	1187	2932
1732	1186	2932	1187
1733	178	2021	2018
1734	948	2934	2021
1735	386	2018	2934
1736	2021	2934	2018
1737	179	2024	2027
1738	387	2936	2024
1739	948	2027	2936
1740	2024	2936	2027
1741	386	2934	2932
1742	948	2936	2934
1743	387	2932	2936
1744	2934	2936	2932
1745	68	1427	1423
1746	627	3595	1427
1747	623	1423	3595
1748	1427	3595	1423
1749	277	2614	2612
1750	950	3606	2614
1751	627	2612	3606
1752	2614	3606	2612
1753	178	2019	2023
1754	623	3597	2019
1755	950	2023	3597
1756	2019	3597	2023
1757	627	3606	3595
1758	950	3597	3606
1759	623	3595	3597
1760	3606	3597	3595
1761	69	1429	1433
1762	629	3613	1429
1763	633	1433	3613
1764	1429	3613	1433
1765	179	2029	2025
1766	952	3615	2029
1767	629	2025	3615
1768	2029	3615	2025
1769	277	2613	2615
1770	633	3624	2613
1771	952	2615	3624
1772	2613	3624	2615
1773	629	3615	3613
1774	952	3624	3615
1775	633	3613	3624
1776	3615	3624	3613
1777	178	2023	2021
1778	950	4088	2023
1779	948	2021	4088
1780	2023	4088	2021
1781	277	2615	2614
1782	952	4090	2615

1783	950	2614	4090
1784	2615	4090	2614
1785	179	2027	2029
1786	948	4089	2027
1787	952	2029	4089
1788	2027	4089	2029
1789	950	4090	4088
1790	952	4089	4090
1791	948	4088	4089
1792	4090	4089	4088
1793	15	1146	1147
1794	346	2820	1146
1795	347	1147	2820
1796	1146	2820	1147
1797	138	1793	1790
1798	836	2822	1793
1799	346	1790	2822
1800	1793	2822	1790
1801	139	1796	1799
1802	347	2824	1796
1803	836	1799	2824
1804	1796	2824	1799
1805	346	2822	2820
1806	836	2824	2822
1807	347	2820	2824
1808	2822	2824	2820
1809	275	2602	2600
1810	838	3586	2602
1811	620	2600	3586
1812	2602	3586	2600
1813	68	1422	1426
1814	622	3591	1422
1815	626	1426	3591
1816	1422	3591	1426
1817	139	1801	1797
1818	840	3593	1801
1819	622	1797	3593
1820	1801	3593	1797
1821	275	2601	2603
1822	626	3604	2601
1823	840	2603	3604
1824	2601	3604	2603
1825	622	3593	3591
1826	840	3604	3593
1827	626	3591	3604
1828	3593	3604	3591
1829	138	1795	1793
1830	838	3974	1795
1831	836	1793	3974
1832	1795	3974	1793
1833	275	2603	2602
1834	840	3976	2603
1835	838	2602	3976
1836	2603	3976	2602
1837	139	1799	1801
1838	836	3975	1799
1839	840	1801	3975
1840	1799	3975	1801
1841	838	3976	3974

1842	840	3975	3976
1843	836	3974	3975
1844	3976	3975	3974
1845	69	1433	1432
1846	633	3621	1433
1847	632	1432	3621
1848	1433	3621	1432
1849	277	2617	2613
1850	1077	3625	2617
1851	633	2613	3625
1852	2617	3625	2613
1853	276	2607	2611
1854	632	3623	2607
1855	1077	2611	3623
1856	2607	3623	2611
1857	633	3625	3621
1858	1077	3623	3625
1859	632	3621	3623
1860	3625	3623	3621
1861	68	1426	1427
1862	626	3603	1426
1863	627	1427	3603
1864	1426	3603	1427
1865	275	2605	2601
1866	1076	3605	2605
1867	626	2601	3605
1868	2605	3605	2601
1869	277	2612	2616
1870	627	3607	2612
1871	1076	2616	3607
1872	2612	3607	2616
1873	626	3605	3603
1874	1076	3607	3605
1875	627	3603	3607
1876	3605	3607	3603
1877	67	1421	1420
1878	621	3585	1421
1879	620	1420	3585
1880	1421	3585	1420
1881	276	2610	2606
1882	1075	3589	2610
1883	621	2606	3589
1884	2610	3589	2606
1885	275	2600	2604
1886	620	3587	2600
1887	1075	2604	3587
1888	2600	3587	2604
1889	621	3589	3585
1890	1075	3587	3589
1891	620	3585	3587
1892	3589	3587	3585
1893	277	2616	2617
1894	1076	4213	2616
1895	1077	2617	4213
1896	2616	4213	2617
1897	275	2604	2605
1898	1075	4211	2604
1899	1076	2605	4211
1900	2604	4211	2605

1901	276	2611	2610
1902	1077	4212	2611
1903	1075	2610	4212
1904	2611	4212	2610
1905	1076	4211	4213
1906	1075	4212	4211
1907	1077	4213	4212
1908	4211	4212	4213
1909	4	1097	1099
1910	297	2703	1097
1911	299	1099	2703
1912	1097	2703	1099
1913	89	1539	1536
1914	719	2705	1539
1915	297	1536	2705
1916	1539	2705	1536
1917	91	1546	1548
1918	299	2707	1546
1919	719	1548	2707
1920	1546	2707	1548
1921	297	2705	2703
1922	719	2707	2705
1923	299	2703	2707
1924	2705	2707	2703
1925	33	1235	1232
1926	435	3051	1235
1927	432	1232	3051
1928	1235	3051	1232
1929	207	2194	2192
1930	721	3062	2194
1931	435	2192	3062
1932	2194	3062	2192
1933	89	1537	1541
1934	432	3053	1537
1935	721	1541	3053
1936	1537	3053	1541
1937	435	3062	3051
1938	721	3053	3062
1939	432	3051	3053
1940	3062	3053	3051
1941	35	1242	1244
1942	442	3077	1242
1943	444	1244	3077
1944	1242	3077	1244
1945	91	1549	1547
1946	723	3078	1549
1947	442	1547	3078
1948	1549	3078	1547
1949	207	2193	2195
1950	444	3082	2193
1951	723	2195	3082
1952	2193	3082	2195
1953	442	3078	3077
1954	723	3082	3078
1955	444	3077	3082
1956	3078	3082	3077
1957	89	1541	1539
1958	721	3856	1541
1959	719	1539	3856

1960	1541	3856	1539
1961	207	2195	2194
1962	723	3859	2195
1963	721	2194	3859
1964	2195	3859	2194
1965	91	1548	1549
1966	719	3857	1548
1967	723	1549	3857
1968	1548	3857	1549
1969	721	3859	3856
1970	723	3857	3859
1971	719	3856	3857
1972	3859	3857	3856
1973	17	1155	1152
1974	355	2835	1155
1975	352	1152	2835
1976	1155	2835	1152
1977	147	1842	1840
1978	851	2846	1842
1979	355	1840	2846
1980	1842	2846	1840
1981	144	1822	1825
1982	352	2837	1822
1983	851	1825	2837
1984	1822	2837	1825
1985	355	2846	2835
1986	851	2837	2846
1987	352	2835	2837
1988	2846	2837	2835
1989	71	1442	1440
1990	642	3644	1442
1991	640	1440	3644
1992	1442	3644	1440
1993	209	2207	2205
1994	862	3653	2207
1995	642	2205	3653
1996	2207	3653	2205
1997	147	1841	1844
1998	640	3646	1841
1999	862	1844	3646
2000	1841	3646	1844
2001	642	3653	3644
2002	862	3646	3653
2003	640	3644	3646
2004	3653	3646	3644
2005	147	1844	1842
2006	862	3989	1844
2007	851	1842	3989
2008	1844	3989	1842
2009	209	2206	2207
2010	853	3991	2206
2011	862	2207	3991
2012	2206	3991	2207
2013	144	1825	1827
2014	851	3988	1825
2015	853	1827	3988
2016	1825	3988	1827
2017	862	3991	3989
2018	853	3988	3991

2019	851	3989	3988
2020	3991	3988	3989
2021	19	1162	1164
2022	362	2861	1162
2023	364	1164	2861
2024	1162	2861	1164
2025	154	1880	1878
2026	877	2862	1880
2027	362	1878	2862
2028	1880	2862	1878
2029	156	1886	1888
2030	364	2866	1886
2031	877	1888	2866
2032	1886	2866	1888
2033	362	2862	2861
2034	877	2866	2862
2035	364	2861	2866
2036	2862	2866	2861
2037	35	1245	1243
2038	445	3079	1245
2039	443	1243	3079
2040	1245	3079	1243
2041	211	2218	2216
2042	878	3084	2218
2043	445	2216	3084
2044	2218	3084	2216
2045	154	1879	1881
2046	443	3080	1879
2047	878	1881	3080
2048	1879	3080	1881
2049	445	3084	3079
2050	878	3080	3084
2051	443	3079	3080
2052	3084	3080	3079
2053	156	1890	1887
2054	882	3650	1890
2055	641	1887	3650
2056	1890	3650	1887
2057	154	1881	1880
2058	878	4013	1881
2059	877	1880	4013
2060	1881	4013	1880
2061	211	2219	2218
2062	882	4015	2219
2063	878	2218	4015
2064	2219	4015	2218
2065	156	1888	1890
2066	877	4014	1888
2067	882	1890	4014
2068	1888	4014	1890
2069	878	4015	4013
2070	882	4014	4015
2071	877	4013	4014
2072	4015	4014	4013
2073	33	1237	1235
2074	437	3061	1237
2075	435	1235	3061
2076	1237	3061	1235
2077	209	2208	2204

2078	1008	3067	2208
2079	437	2204	3067
2080	2208	3067	2204
2081	207	2192	2196
2082	435	3063	2192
2083	1008	2196	3063
2084	2192	3063	2196
2085	437	3067	3061
2086	1008	3063	3067
2087	435	3061	3063
2088	3067	3063	3061
2089	209	2205	2209
2090	642	3654	2205
2091	1011	2209	3654
2092	2205	3654	2209
2093	35	1244	1245
2094	444	3081	1244
2095	445	1245	3081
2096	1244	3081	1245
2097	207	2197	2193
2098	1009	3083	2197
2099	444	2193	3083
2100	2197	3083	2193
2101	211	2216	2220
2102	445	3085	2216
2103	1009	2220	3085
2104	2216	3085	2220
2105	444	3083	3081
2106	1009	3085	3083
2107	445	3081	3085
2108	3083	3085	3081
2109	209	2209	2208
2110	1011	4146	2209
2111	1008	2208	4146
2112	2209	4146	2208
2113	211	2220	2221
2114	1009	4147	2220
2115	1011	2221	4147
2116	2220	4147	2221
2117	207	2196	2197
2118	1008	4145	2196
2119	1009	2197	4145
2120	2196	4145	2197
2121	1011	4147	4146
2122	1009	4145	4147
2123	1008	4146	4145
2124	4147	4145	4146
2125	7	1113	1109
2126	313	2731	1113
2127	309	1109	2731
2128	1113	2731	1109
2129	105	1622	1620
2130	747	2742	1622
2131	313	1620	2742
2132	1622	2742	1620
2133	101	1596	1599
2134	309	2733	1596
2135	747	1599	2733
2136	1596	2733	1599

2137	313	2742	2731
2138	747	2733	2742
2139	309	2731	2733
2140	2742	2733	2731
2141	49	1318	1316
2142	518	3284	1318
2143	516	1316	3284
2144	1318	3284	1316
2145	235	2363	2361
2146	758	3293	2363
2147	518	2361	3293
2148	2363	3293	2361
2149	105	1621	1624
2150	516	3286	1621
2151	758	1624	3286
2152	1621	3286	1624
2153	518	3293	3284
2154	758	3286	3293
2155	516	3284	3286
2156	3293	3286	3284
2157	45	1292	1295
2158	492	3213	1292
2159	495	1295	3213
2160	1292	3213	1295
2161	101	1601	1597
2162	749	3215	1601
2163	492	1597	3215
2164	1601	3215	1597
2165	235	2360	2362
2166	495	3224	2360
2167	749	2362	3224
2168	2360	3224	2362
2169	492	3215	3213
2170	749	3224	3215
2171	495	3213	3224
2172	3215	3224	3213
2173	105	1624	1622
2174	758	3887	1624
2175	747	1622	3887
2176	1624	3887	1622
2177	235	2362	2363
2178	749	3889	2362
2179	758	2363	3889
2180	2362	3889	2363
2181	101	1599	1601
2182	747	3886	1599
2183	749	1601	3886
2184	1599	3886	1601
2185	758	3889	3887
2186	749	3886	3889
2187	747	3887	3886
2188	3889	3886	3887
2189	25	1198	1196
2190	398	2960	1198
2191	396	1196	2960
2192	1198	2960	1196
2193	190	2092	2090
2194	976	2969	2092
2195	398	2090	2969

2196	2092	2969	2090
2197	188	2078	2080
2198	396	2962	2078
2199	976	2080	2962
2200	2078	2962	2080
2201	398	2969	2960
2202	976	2962	2969
2203	396	2960	2962
2204	2969	2962	2960
2205	73	1455	1453
2206	655	3684	1455
2207	653	1453	3684
2208	1455	3684	1453
2209	246	2429	2427
2210	985	3691	2429
2211	655	2427	3691
2212	2429	3691	2427
2213	190	2091	2094
2214	653	3686	2091
2215	985	2094	3686
2216	2091	3686	2094
2217	655	3691	3684
2218	985	3686	3691
2219	653	3684	3686
2220	3691	3686	3684
2221	49	1317	1320
2222	517	3288	1317
2223	520	1320	3288
2224	1317	3288	1320
2225	188	2082	2079
2226	978	3290	2082
2227	517	2079	3290
2228	2082	3290	2079
2229	246	2426	2428
2230	520	3298	2426
2231	978	2428	3298
2232	2426	3298	2428
2233	517	3290	3288
2234	978	3298	3290
2235	520	3288	3298
2236	3290	3298	3288
2237	190	2094	2092
2238	985	4113	2094
2239	976	2092	4113
2240	2094	4113	2092
2241	246	2428	2429
2242	978	4116	2428
2243	985	2429	4116
2244	2428	4116	2429
2245	188	2080	2082
2246	976	4112	2080
2247	978	2082	4112
2248	2080	4112	2082
2249	985	4116	4113
2250	978	4112	4116
2251	976	4113	4112
2252	4116	4112	4113
2253	17	1153	1157
2254	353	2839	1153

2255	357	1157	2839
2256	1153	2839	1157
2257	145	1831	1828
2258	855	2841	1831
2259	353	1828	2841
2260	1831	2841	1828
2261	149	1852	1854
2262	357	2850	1852
2263	855	1854	2850
2264	1852	2850	1854
2265	353	2841	2839
2266	855	2850	2841
2267	357	2839	2850
2268	2841	2850	2839
2269	45	1297	1293
2270	497	3217	1297
2271	493	1293	3217
2272	1297	3217	1293
2273	237	2374	2372
2274	857	3228	2374
2275	497	2372	3228
2276	2374	3228	2372
2277	145	1829	1833
2278	493	3219	1829
2279	857	1833	3219
2280	1829	3219	1833
2281	497	3228	3217
2282	857	3219	3228
2283	493	3217	3219
2284	3228	3219	3217
2285	73	1452	1454
2286	652	3680	1452
2287	654	1454	3680
2288	1452	3680	1454
2289	149	1856	1853
2290	866	3682	1856
2291	652	1853	3682
2292	1856	3682	1853
2293	237	2373	2375
2294	654	3689	2373
2295	866	2375	3689
2296	2373	3689	2375
2297	652	3682	3680
2298	866	3689	3682
2299	654	3680	3689
2300	3682	3689	3680
2301	145	1833	1831
2302	857	3994	1833
2303	855	1831	3994
2304	1833	3994	1831
2305	237	2375	2374
2306	866	3997	2375
2307	857	2374	3997
2308	2375	3997	2374
2309	149	1854	1856
2310	855	3995	1854
2311	866	1856	3995
2312	1854	3995	1856
2313	857	3997	3994

2314	866	3995	3997
2315	855	3994	3995
2316	3997	3995	3994
2317	49	1320	1318
2318	520	3292	1320
2319	518	1318	3292
2320	1320	3292	1318
2321	246	2430	2426
2322	1039	3299	2430
2323	520	2426	3299
2324	2430	3299	2426
2325	235	2361	2365
2326	518	3294	2361
2327	1039	2365	3294
2328	2361	3294	2365
2329	520	3299	3292
2330	1039	3294	3299
2331	518	3292	3294
2332	3299	3294	3292
2333	73	1454	1455
2334	654	3688	1454
2335	655	1455	3688
2336	1454	3688	1455
2337	237	2377	2373
2338	1041	3690	2377
2339	654	2373	3690
2340	2377	3690	2373
2341	246	2427	2431
2342	655	3692	2427
2343	1041	2431	3692
2344	2427	3692	2431
2345	654	3690	3688
2346	1041	3692	3690
2347	655	3688	3692
2348	3690	3692	3688
2349	45	1295	1297
2350	495	3223	1295
2351	497	1297	3223
2352	1295	3223	1297
2353	235	2364	2360
2354	1038	3225	2364
2355	495	2360	3225
2356	2364	3225	2360
2357	237	2372	2376
2358	497	3229	2372
2359	1038	2376	3229
2360	2372	3229	2376
2361	495	3225	3223
2362	1038	3229	3225
2363	497	3223	3229
2364	3225	3229	3223
2365	246	2431	2430
2366	1041	4177	2431
2367	1039	2430	4177
2368	2431	4177	2430
2369	237	2376	2377
2370	1038	4176	2376
2371	1041	2377	4176
2372	2376	4176	2377

2373	235	2365	2364
2374	1039	4175	2365
2375	1038	2364	4175
2376	2365	4175	2364
2377	1041	4176	4177
2378	1038	4175	4176
2379	1039	4177	4175
2380	4176	4175	4177
2381	9	1119	1121
2382	319	2755	1119
2383	321	1121	2755
2384	1119	2755	1121
2385	111	1652	1650
2386	771	2756	1652
2387	319	1650	2756
2388	1652	2756	1650
2389	113	1660	1662
2390	321	2760	1660
2391	771	1662	2760
2392	1660	2760	1662
2393	319	2756	2755
2394	771	2760	2756
2395	321	2755	2760
2396	2756	2760	2755
2397	55	1348	1346
2398	548	3365	1348
2399	546	1346	3365
2400	1348	3365	1346
2401	259	2506	2504
2402	772	3370	2506
2403	548	2504	3370
2404	2506	3370	2504
2405	111	1651	1653
2406	546	3366	1651
2407	772	1653	3366
2408	1651	3366	1653
2409	548	3370	3365
2410	772	3366	3370
2411	546	3365	3366
2412	3370	3366	3365
2413	57	1356	1358
2414	556	3392	1356
2415	558	1358	3392
2416	1356	3392	1358
2417	113	1664	1661
2418	776	3394	1664
2419	556	1661	3394
2420	1664	3394	1661
2421	259	2505	2507
2422	558	3401	2505
2423	776	2507	3401
2424	2505	3401	2507
2425	556	3394	3392
2426	776	3401	3394
2427	558	3392	3401
2428	3394	3401	3392
2429	111	1653	1652
2430	772	3908	1653
2431	771	1652	3908

2432	1653	3908	1652
2433	259	2507	2506
2434	776	3910	2507
2435	772	2506	3910
2436	2507	3910	2506
2437	113	1662	1664
2438	771	3909	1662
2439	776	1664	3909
2440	1662	3909	1664
2441	772	3910	3908
2442	776	3909	3910
2443	771	3908	3909
2444	3910	3909	3908
2445	19	1165	1163
2446	365	2863	1165
2447	363	1163	2863
2448	1165	2863	1163
2449	157	1894	1892
2450	879	2868	1894
2451	365	1892	2868
2452	1894	2868	1892
2453	155	1882	1884
2454	363	2864	1882
2455	879	1884	2864
2456	1882	2864	1884
2457	365	2868	2863
2458	879	2864	2868
2459	363	2863	2864
2460	2868	2864	2863
2461	75	1466	1464
2462	666	3716	1466
2463	664	1464	3716
2464	1466	3716	1464
2465	260	2513	2511
2466	884	3725	2513
2467	666	2511	3725
2468	2513	3725	2511
2469	157	1893	1896
2470	664	3718	1893
2471	884	1896	3718
2472	1893	3718	1896
2473	666	3725	3716
2474	884	3718	3725
2475	664	3716	3718
2476	3725	3718	3716
2477	55	1347	1349
2478	547	3367	1347
2479	549	1349	3367
2480	1347	3367	1349
2481	155	1885	1883
2482	880	3368	1885
2483	547	1883	3368
2484	1885	3368	1883
2485	260	2510	2512
2486	549	3372	2510
2487	880	2512	3372
2488	2510	3372	2512
2489	547	3368	3367
2490	880	3372	3368

2491	549	3367	3372
2492	3368	3372	3367
2493	157	1896	1894
2494	884	4017	1896
2495	879	1894	4017
2496	1896	4017	1894
2497	260	2512	2513
2498	880	4018	2512
2499	884	2513	4018
2500	2512	4018	2513
2501	155	1884	1885
2502	879	4016	1884
2503	880	1885	4016
2504	1884	4016	1885
2505	884	4018	4017
2506	880	4016	4018
2507	879	4017	4016
2508	4018	4016	4017
2509	25	1197	1199
2510	397	2964	1197
2511	399	1199	2964
2512	1197	2964	1199
2513	189	2086	2084
2514	980	2966	2086
2515	397	2084	2966
2516	2086	2966	2084
2517	191	2096	2098
2518	399	2971	2096
2519	980	2098	2971
2520	2096	2971	2098
2521	397	2966	2964
2522	980	2971	2966
2523	399	2964	2971
2524	2966	2971	2964
2525	57	1360	1357
2526	560	3396	1360
2527	557	1357	3396
2528	1360	3396	1357
2529	264	2536	2534
2530	982	3406	2536
2531	560	2534	3406
2532	2536	3406	2534
2533	189	2085	2088
2534	557	3398	2085
2535	982	2088	3398
2536	2085	3398	2088
2537	560	3406	3396
2538	982	3398	3406
2539	557	3396	3398
2540	3406	3398	3396
2541	75	1465	1467
2542	665	3720	1465
2543	667	1467	3720
2544	1465	3720	1467
2545	191	2100	2097
2546	987	3722	2100
2547	665	2097	3722
2548	2100	3722	2097
2549	264	2535	2537

2550	667	3727	2535
2551	987	2537	3727
2552	2535	3727	2537
2553	665	3722	3720
2554	987	3727	3722
2555	667	3720	3727
2556	3722	3727	3720
2557	189	2088	2086
2558	982	4118	2088
2559	980	2086	4118
2560	2088	4118	2086
2561	264	2537	2536
2562	987	4122	2537
2563	982	2536	4122
2564	2537	4122	2536
2565	191	2098	2100
2566	980	4119	2098
2567	987	2100	4119
2568	2098	4119	2100
2569	982	4122	4118
2570	987	4119	4122
2571	980	4118	4119
2572	4122	4119	4118
2573	55	1349	1348
2574	549	3369	1349
2575	548	1348	3369
2576	1349	3369	1348
2577	259	2504	2508
2578	548	3371	2504
2579	1060	2508	3371
2580	2504	3371	2508
2581	75	1467	1466
2582	667	3724	1467
2583	666	1466	3724
2584	1467	3724	1466
2585	57	1358	1360
2586	558	3400	1358
2587	560	1360	3400
2588	1358	3400	1360
2589	259	2509	2505
2590	1061	3402	2509
2591	558	2505	3402
2592	2509	3402	2505
2593	264	2534	2538
2594	560	3407	2534
2595	1061	2538	3407
2596	2534	3407	2538
2597	558	3402	3400
2598	1061	3407	3402
2599	560	3400	3407
2600	3402	3407	3400
2601	259	2508	2509
2602	1060	4196	2508
2603	1061	2509	4196
2604	2508	4196	2509
2605	17	1157	1155
2606	357	2845	1157
2607	355	1155	2845
2608	1157	2845	1155

2609	149	1855	1852
2610	861	2851	1855
2611	357	1852	2851
2612	1855	2851	1852
2613	147	1840	1843
2614	355	2847	1840
2615	861	1843	2847
2616	1840	2847	1843
2617	357	2851	2845
2618	861	2847	2851
2619	355	2845	2847
2620	2851	2847	2845
2621	73	1456	1452
2622	656	3681	1456
2623	652	1452	3681
2624	1456	3681	1452
2625	280	2633	2631
2626	867	3694	2633
2627	656	2631	3694
2628	2633	3694	2631
2629	149	1853	1857
2630	652	3683	1853
2631	867	1857	3683
2632	1853	3683	1857
2633	656	3694	3681
2634	867	3683	3694
2635	652	3681	3683
2636	3694	3683	3681
2637	71	1440	1444
2638	640	3645	1440
2639	644	1444	3645
2640	1440	3645	1444
2641	147	1845	1841
2642	863	3647	1845
2643	640	1841	3647
2644	1845	3647	1841
2645	280	2630	2632
2646	644	3658	2630
2647	863	2632	3658
2648	2630	3658	2632
2649	640	3647	3645
2650	863	3658	3647
2651	644	3645	3658
2652	3647	3658	3645
2653	149	1857	1855
2654	867	4002	1857
2655	861	1855	4002
2656	1857	4002	1855
2657	280	2632	2633
2658	863	4003	2632
2659	867	2633	4003
2660	2632	4003	2633
2661	147	1843	1845
2662	861	4001	1843
2663	863	1845	4001
2664	1843	4001	1845
2665	867	4003	4002
2666	863	4001	4003
2667	861	4002	4001

2668	4003	4001	4002
2669	25	1199	1198
2670	399	2968	1199
2671	398	1198	2968
2672	1199	2968	1198
2673	191	2099	2096
2674	984	2972	2099
2675	399	2096	2972
2676	2099	2972	2096
2677	190	2090	2093
2678	398	2970	2090
2679	984	2093	2970
2680	2090	2970	2093
2681	399	2972	2968
2682	984	2970	2972
2683	398	2968	2970
2684	2972	2970	2968
2685	75	1469	1465
2686	669	3721	1469
2687	665	1465	3721
2688	1469	3721	1465
2689	283	2651	2649
2690	988	3732	2651
2691	669	2649	3732
2692	2651	3732	2649
2693	191	2097	2101
2694	665	3723	2097
2695	988	2101	3723
2696	2097	3723	2101
2697	669	3732	3721
2698	988	3723	3732
2699	665	3721	3723
2700	3732	3723	3721
2701	73	1453	1457
2702	653	3685	1453
2703	657	1457	3685
2704	1453	3685	1457
2705	190	2095	2091
2706	986	3687	2095
2707	653	2091	3687
2708	2095	3687	2091
2709	283	2648	2650
2710	657	3696	2648
2711	986	2650	3696
2712	2648	3696	2650
2713	653	3687	3685
2714	986	3696	3687
2715	657	3685	3696
2716	3687	3696	3685
2717	191	2101	2099
2718	988	4125	2101
2719	984	2099	4125
2720	2101	4125	2099
2721	283	2650	2651
2722	986	4126	2650
2723	988	2651	4126
2724	2650	4126	2651
2725	190	2093	2095
2726	984	4124	2093

2727	986	2095	4124
2728	2093	4124	2095
2729	988	4126	4125
2730	986	4124	4126
2731	984	4125	4124
2732	4126	4124	4125
2733	19	1164	1165
2734	364	2865	1164
2735	365	1165	2865
2736	1164	2865	1165
2737	156	1889	1886
2738	881	2867	1889
2739	364	1886	2867
2740	1889	2867	1886
2741	157	1892	1895
2742	365	2869	1892
2743	881	1895	2869
2744	1892	2869	1895
2745	364	2867	2865
2746	881	2869	2867
2747	365	2865	2869
2748	2867	2869	2865
2749	71	1445	1441
2750	645	3649	1445
2751	641	1441	3649
2752	1445	3649	1441
2753	281	2638	2636
2754	883	3660	2638
2755	645	2636	3660
2756	2638	3660	2636
2757	156	1887	1891
2758	641	3651	1887
2759	883	1891	3651
2760	1887	3651	1891
2761	645	3660	3649
2762	883	3651	3660
2763	641	3649	3651
2764	3660	3651	3649
2765	75	1464	1468
2766	664	3717	1464
2767	668	1468	3717
2768	1464	3717	1468
2769	157	1897	1893
2770	885	3719	1897
2771	664	1893	3719
2772	1897	3719	1893
2773	281	2637	2639
2774	668	3730	2637
2775	885	2639	3730
2776	2637	3730	2639
2777	664	3719	3717
2778	885	3730	3719
2779	668	3717	3730
2780	3719	3730	3717
2781	156	1891	1889
2782	883	4019	1891
2783	881	1889	4019
2784	1891	4019	1889
2785	281	2639	2638

2786	885	4021	2639
2787	883	2638	4021
2788	2639	4021	2638
2789	157	1895	1897
2790	881	4020	1895
2791	885	1897	4020
2792	1895	4020	1897
2793	883	4021	4019
2794	885	4020	4021
2795	881	4019	4020
2796	4021	4020	4019
2797	73	1457	1456
2798	657	3693	1457
2799	656	1456	3693
2800	1457	3693	1456
2801	283	2652	2648
2802	1082	3697	2652
2803	657	2648	3697
2804	2652	3697	2648
2805	280	2631	2635
2806	656	3695	2631
2807	1082	2635	3695
2808	2631	3695	2635
2809	657	3697	3693
2810	1082	3695	3697
2811	656	3693	3695
2812	3697	3695	3693
2813	75	1468	1469
2814	668	3729	1468
2815	669	1469	3729
2816	1468	3729	1469
2817	281	2641	2637
2818	1083	3731	2641
2819	668	2637	3731
2820	2641	3731	2637
2821	283	2649	2653
2822	669	3733	2649
2823	1083	2653	3733
2824	2649	3733	2653
2825	668	3731	3729
2826	1083	3733	3731
2827	669	3729	3733
2828	3731	3733	3729
2829	71	1444	1445
2830	644	3657	1444
2831	645	1445	3657
2832	1444	3657	1445
2833	280	2634	2630
2834	1081	3659	2634
2835	644	2630	3659
2836	2634	3659	2630
2837	281	2636	2640
2838	645	3661	2636
2839	1081	2640	3661
2840	2636	3661	2640
2841	644	3659	3657
2842	1081	3661	3659
2843	645	3657	3661
2844	3659	3661	3657

2845	283	2653	2652
2846	1083	4219	2653
2847	1082	2652	4219
2848	2653	4219	2652
2849	281	2640	2641
2850	1081	4218	2640
2851	1083	2641	4218
2852	2640	4218	2641
2853	280	2635	2634
2854	1082	4217	2635
2855	1081	2634	4217
2856	2635	4217	2634
2857	1083	4218	4219
2858	1081	4217	4218
2859	1082	4219	4217
2860	4218	4217	4219
2861	6	1107	1106
2862	307	2721	1107
2863	306	1106	2721
2864	1107	2721	1106
2865	99	1587	1584
2866	737	2725	1587
2867	307	1584	2725
2868	1587	2725	1584
2869	98	1578	1581
2870	306	2723	1578
2871	737	1581	2723
2872	1578	2723	1581
2873	307	2725	2721
2874	737	2723	2725
2875	306	2721	2723
2876	2725	2723	2721
2877	43	1283	1280
2878	483	3177	1283
2879	480	1280	3177
2880	1283	3177	1280
2881	225	2303	2301
2882	741	3188	2303
2883	483	2301	3188
2884	2303	3188	2301
2885	99	1585	1589
2886	480	3179	1585
2887	741	1589	3179
2888	1585	3179	1589
2889	483	3188	3177
2890	741	3179	3188
2891	480	3177	3179
2892	3188	3179	3177
2893	225	2300	2302
2894	477	3170	2300
2895	739	2302	3170
2896	2300	3170	2302
2897	99	1589	1587
2898	741	3876	1589
2899	737	1587	3876
2900	1589	3876	1587
2901	225	2302	2303
2902	739	3877	2302
2903	741	2303	3877

2904	2302	3877	2303
2905	98	1581	1583
2906	737	3875	1581
2907	739	1583	3875
2908	1581	3875	1583
2909	741	3877	3876
2910	739	3875	3877
2911	737	3876	3875
2912	3877	3875	3876
2913	23	1188	1184
2914	388	2925	1188
2915	384	1184	2925
2916	1188	2925	1184
2917	180	2032	2030
2918	941	2938	2032
2919	388	2030	2938
2920	2032	2938	2030
2921	176	2006	2009
2922	384	2927	2006
2923	941	2009	2927
2924	2006	2927	2009
2925	388	2938	2925
2926	941	2927	2938
2927	384	2925	2927
2928	2938	2927	2925
2929	79	1491	1489
2930	691	3792	1491
2931	689	1489	3792
2932	1491	3792	1489
2933	229	2327	2325
2934	954	3799	2327
2935	691	2325	3799
2936	2327	3799	2325
2937	180	2031	2034
2938	689	3794	2031
2939	954	2034	3794
2940	2031	3794	2034
2941	691	3799	3792
2942	954	3794	3799
2943	689	3792	3794
2944	3799	3794	3792
2945	43	1281	1285
2946	481	3181	1281
2947	485	1285	3181
2948	1281	3181	1285
2949	176	2011	2007
2950	943	3183	2011
2951	481	2007	3183
2952	2011	3183	2007
2953	229	2324	2326
2954	485	3192	2324
2955	943	2326	3192
2956	2324	3192	2326
2957	481	3183	3181
2958	943	3192	3183
2959	485	3181	3192
2960	3183	3192	3181
2961	180	2034	2032
2962	954	4079	2034

2963	941	2032	4079
2964	2034	4079	2032
2965	229	2326	2327
2966	943	4081	2326
2967	954	2327	4081
2968	2326	4081	2327
2969	176	2009	2011
2970	941	4078	2009
2971	943	2011	4078
2972	2009	4078	2011
2973	954	4081	4079
2974	943	4078	4081
2975	941	4079	4078
2976	4081	4078	4079
2977	22	1178	1182
2978	378	2907	1178
2979	382	1182	2907
2980	1178	2907	1182
2981	170	1973	1970
2982	923	2909	1973
2983	378	1970	2909
2984	1973	2909	1970
2985	174	1994	1996
2986	382	2920	1994
2987	923	1996	2920
2988	1994	2920	1996
2989	378	2909	2907
2990	923	2920	2909
2991	382	2907	2920
2992	2909	2920	2907
2993	42	1279	1275
2994	479	3163	1279
2995	475	1275	3163
2996	1279	3163	1275
2997	227	2314	2312
2998	925	3174	2314
2999	479	2312	3174
3000	2314	3174	2312
3001	170	1971	1975
3002	475	3165	1971
3003	925	1975	3165
3004	1971	3165	1975
3005	479	3174	3163
3006	925	3165	3174
3007	475	3163	3165
3008	3174	3165	3163
3009	79	1488	1490
3010	688	3788	1488
3011	690	1490	3788
3012	1488	3788	1490
3013	174	1998	1995
3014	936	3790	1998
3015	688	1995	3790
3016	1998	3790	1995
3017	227	2313	2315
3018	690	3797	2313
3019	936	2315	3797
3020	2313	3797	2315
3021	688	3790	3788

3022	936	3797	3790
3023	690	3788	3797
3024	3790	3797	3788
3025	170	1975	1973
3026	925	4060	1975
3027	923	1973	4060
3028	1975	4060	1973
3029	227	2315	2314
3030	936	4063	2315
3031	925	2314	4063
3032	2315	4063	2314
3033	174	1996	1998
3034	923	4061	1996
3035	936	1998	4061
3036	1996	4061	1998
3037	925	4063	4060
3038	936	4061	4063
3039	923	4060	4061
3040	4063	4061	4060
3041	43	1285	1283
3042	485	3187	1285
3043	483	1283	3187
3044	1285	3187	1283
3045	229	2328	2324
3046	1028	3193	2328
3047	485	2324	3193
3048	2328	3193	2324
3049	225	2301	2305
3050	483	3189	2301
3051	1028	2305	3189
3052	2301	3189	2305
3053	485	3193	3187
3054	1028	3189	3193
3055	483	3187	3189
3056	3193	3189	3187
3057	79	1490	1491
3058	690	3796	1490
3059	691	1491	3796
3060	1490	3796	1491
3061	227	2317	2313
3062	1029	3798	2317
3063	690	2313	3798
3064	2317	3798	2313
3065	229	2325	2329
3066	691	3800	2325
3067	1029	2329	3800
3068	2325	3800	2329
3069	690	3798	3796
3070	1029	3800	3798
3071	691	3796	3800
3072	3798	3800	3796
3073	42	1277	1279
3074	477	3169	1277
3075	479	1279	3169
3076	1277	3169	1279
3077	225	2304	2300
3078	1027	3171	2304
3079	477	2300	3171
3080	2304	3171	2300

3081	227	2312	2316
3082	479	3175	2312
3083	1027	2316	3175
3084	2312	3175	2316
3085	477	3171	3169
3086	1027	3175	3171
3087	479	3169	3175
3088	3171	3175	3169
3089	229	2329	2328
3090	1029	4165	2329
3091	1028	2328	4165
3092	2329	4165	2328
3093	227	2316	2317
3094	1027	4164	2316
3095	1029	2317	4164
3096	2316	4164	2317
3097	225	2305	2304
3098	1028	4163	2305
3099	1027	2304	4163
3100	2305	4163	2304
3101	1029	4164	4165
3102	1027	4163	4164
3103	1028	4165	4163
3104	4164	4163	4165
3105	9	1121	1120
3106	321	2757	1121
3107	320	1120	2757
3108	1121	2757	1120
3109	113	1663	1660
3110	773	2761	1663
3111	321	1660	2761
3112	1663	2761	1660
3113	112	1654	1657
3114	320	2759	1654
3115	773	1657	2759
3116	1654	2759	1657
3117	321	2761	2757
3118	773	2759	2761
3119	320	2757	2759
3120	2761	2759	2757
3121	57	1359	1356
3122	559	3393	1359
3123	556	1356	3393
3124	1359	3393	1356
3125	261	2519	2517
3126	777	3404	2519
3127	559	2517	3404
3128	2519	3404	2517
3129	113	1661	1665
3130	556	3395	1661
3131	777	1665	3395
3132	1661	3395	1665
3133	559	3404	3393
3134	777	3395	3404
3135	556	3393	3395
3136	3404	3395	3393
3137	56	1350	1353
3138	550	3375	1350
3139	553	1353	3375

3140	1350	3375	1353
3141	112	1659	1655
3142	775	3377	1659
3143	550	1655	3377
3144	1659	3377	1655
3145	261	2516	2518
3146	553	3386	2516
3147	775	2518	3386
3148	2516	3386	2518
3149	550	3377	3375
3150	775	3386	3377
3151	553	3375	3386
3152	3377	3386	3375
3153	113	1665	1663
3154	777	3912	1665
3155	773	1663	3912
3156	1665	3912	1663
3157	261	2518	2519
3158	775	3913	2518
3159	777	2519	3913
3160	2518	3913	2519
3161	112	1657	1659
3162	773	3911	1657
3163	775	1659	3911
3164	1657	3911	1659
3165	777	3913	3912
3166	775	3911	3913
3167	773	3912	3911
3168	3913	3911	3912
3169	25	1201	1197
3170	401	2965	1201
3171	397	1197	2965
3172	1201	2965	1197
3173	193	2110	2108
3174	981	2976	2110
3175	401	2108	2976
3176	2110	2976	2108
3177	189	2084	2087
3178	397	2967	2084
3179	981	2087	2967
3180	2084	2967	2087
3181	401	2976	2965
3182	981	2967	2976
3183	397	2965	2967
3184	2976	2967	2965
3185	81	1503	1501
3186	703	3828	1503
3187	701	1501	3828
3188	1503	3828	1501
3189	265	2543	2541
3190	992	3835	2543
3191	703	2541	3835
3192	2543	3835	2541
3193	193	2109	2112
3194	701	3830	2109
3195	992	2112	3830
3196	2109	3830	2112
3197	703	3835	3828
3198	992	3830	3835

3199	701	3828	3830
3200	3835	3830	3828
3201	57	1357	1361
3202	557	3397	1357
3203	561	1361	3397
3204	1357	3397	1361
3205	189	2089	2085
3206	983	3399	2089
3207	557	2085	3399
3208	2089	3399	2085
3209	265	2540	2542
3210	561	3408	2540
3211	983	2542	3408
3212	2540	3408	2542
3213	557	3399	3397
3214	983	3408	3399
3215	561	3397	3408
3216	3399	3408	3397
3217	193	2112	2110
3218	992	4121	2112
3219	981	2110	4121
3220	2112	4121	2110
3221	265	2542	2543
3222	983	4123	2542
3223	992	2543	4123
3224	2542	4123	2543
3225	189	2087	2089
3226	981	4120	2087
3227	983	2089	4120
3228	2087	4120	2089
3229	992	4123	4121
3230	983	4120	4123
3231	981	4121	4120
3232	4123	4120	4121
3233	23	1185	1189
3234	385	2929	1185
3235	389	1189	2929
3236	1185	2929	1189
3237	177	2015	2012
3238	945	2931	2015
3239	385	2012	2931
3240	2015	2931	2012
3241	181	2036	2038
3242	389	2940	2036
3243	945	2038	2940
3244	2036	2940	2038
3245	385	2931	2929
3246	945	2940	2931
3247	389	2929	2940
3248	2931	2940	2929
3249	56	1355	1351
3250	555	3379	1355
3251	551	1351	3379
3252	1355	3379	1351
3253	263	2530	2528
3254	947	3390	2530
3255	555	2528	3390
3256	2530	3390	2528
3257	177	2013	2017

3258	551	3381	2013
3259	947	2017	3381
3260	2013	3381	2017
3261	555	3390	3379
3262	947	3381	3390
3263	551	3379	3381
3264	3390	3381	3379
3265	81	1500	1502
3266	700	3824	1500
3267	702	1502	3824
3268	1500	3824	1502
3269	181	2040	2037
3270	956	3826	2040
3271	700	2037	3826
3272	2040	3826	2037
3273	263	2529	2531
3274	702	3833	2529
3275	956	2531	3833
3276	2529	3833	2531
3277	700	3826	3824
3278	956	3833	3826
3279	702	3824	3833
3280	3826	3833	3824
3281	177	2017	2015
3282	947	4084	2017
3283	945	2015	4084
3284	2017	4084	2015
3285	263	2531	2530
3286	956	4087	2531
3287	947	2530	4087
3288	2531	4087	2530
3289	181	2038	2040
3290	945	4085	2038
3291	956	2040	4085
3292	2038	4085	2040
3293	947	4087	4084
3294	956	4085	4087
3295	945	4084	4085
3296	4087	4085	4084
3297	57	1361	1359
3298	561	3403	1361
3299	559	1359	3403
3300	1361	3403	1359
3301	265	2544	2540
3302	1064	3409	2544
3303	561	2540	3409
3304	2544	3409	2540
3305	261	2517	2521
3306	559	3405	2517
3307	1064	2521	3405
3308	2517	3405	2521
3309	561	3409	3403
3310	1064	3405	3409
3311	559	3403	3405
3312	3409	3405	3403
3313	81	1502	1503
3314	702	3832	1502
3315	703	1503	3832
3316	1502	3832	1503

3317	263	2533	2529
3318	1065	3834	2533
3319	702	2529	3834
3320	2533	3834	2529
3321	265	2541	2545
3322	703	3836	2541
3323	1065	2545	3836
3324	2541	3836	2545
3325	702	3834	3832
3326	1065	3836	3834
3327	703	3832	3836
3328	3834	3836	3832
3329	56	1353	1355
3330	553	3385	1353
3331	555	1355	3385
3332	1353	3385	1355
3333	261	2520	2516
3334	1063	3387	2520
3335	553	2516	3387
3336	2520	3387	2516
3337	263	2528	2532
3338	555	3391	2528
3339	1063	2532	3391
3340	2528	3391	2532
3341	553	3387	3385
3342	1063	3391	3387
3343	555	3385	3391
3344	3387	3391	3385
3345	265	2545	2544
3346	1065	4201	2545
3347	1064	2544	4201
3348	2545	4201	2544
3349	263	2532	2533
3350	1063	4200	2532
3351	1065	2533	4200
3352	2532	4200	2533
3353	261	2521	2520
3354	1064	4199	2521
3355	1063	2520	4199
3356	2521	4199	2520
3357	1065	4200	4201
3358	1063	4199	4200
3359	1064	4201	4199
3360	4200	4199	4201
3361	7	1111	1113
3362	311	2737	1111
3363	313	1113	2737
3364	1111	2737	1113
3365	103	1611	1608
3366	753	2739	1611
3367	311	1608	2739
3368	1611	2739	1608
3369	105	1620	1623
3370	313	2743	1620
3371	753	1623	2743
3372	1620	2743	1623
3373	311	2739	2737
3374	753	2743	2739
3375	313	2737	2743

3376	2739	2743	2737
3377	47	1307	1304
3378	507	3249	1307
3379	504	1304	3249
3380	1307	3249	1304
3381	241	2398	2396
3382	755	3260	2398
3383	507	2396	3260
3384	2398	3260	2396
3385	103	1609	1613
3386	504	3251	1609
3387	755	1613	3251
3388	1609	3251	1613
3389	507	3260	3249
3390	755	3251	3260
3391	504	3249	3251
3392	3260	3251	3249
3393	49	1316	1319
3394	516	3285	1316
3395	519	1319	3285
3396	1316	3285	1319
3397	105	1625	1621
3398	759	3287	1625
3399	516	1621	3287
3400	1625	3287	1621
3401	241	2397	2399
3402	519	3296	2397
3403	759	2399	3296
3404	2397	3296	2399
3405	516	3287	3285
3406	759	3296	3287
3407	519	3285	3296
3408	3287	3296	3285
3409	103	1613	1611
3410	755	3893	1613
3411	753	1611	3893
3412	1613	3893	1611
3413	241	2399	2398
3414	759	3895	2399
3415	755	2398	3895
3416	2399	3895	2398
3417	105	1623	1625
3418	753	3894	1623
3419	759	1625	3894
3420	1623	3894	1625
3421	755	3895	3893
3422	759	3894	3895
3423	753	3893	3894
3424	3895	3894	3893
3425	22	1183	1179
3426	383	2911	1183
3427	379	1179	2911
3428	1183	2911	1179
3429	175	2002	2000
3430	927	2922	2002
3431	383	2000	2922
3432	2002	2922	2000
3433	171	1976	1979
3434	379	2913	1976

3435	927	1979	2913
3436	1976	2913	1979
3437	383	2922	2911
3438	927	2913	2922
3439	379	2911	2913
3440	2922	2913	2911
3441	47	1305	1309
3442	505	3253	1305
3443	509	1309	3253
3444	1305	3253	1309
3445	171	1981	1977
3446	929	3255	1981
3447	505	1977	3255
3448	1981	3255	1977
3449	243	2408	2410
3450	509	3264	2408
3451	929	2410	3264
3452	2408	3264	2410
3453	505	3255	3253
3454	929	3264	3255
3455	509	3253	3264
3456	3255	3264	3253
3457	175	2004	2002
3458	938	4067	2004
3459	927	2002	4067
3460	2004	4067	2002
3461	243	2410	2411
3462	929	4069	2410
3463	938	2411	4069
3464	2410	4069	2411
3465	171	1979	1981
3466	927	4066	1979
3467	929	1981	4066
3468	1979	4066	1981
3469	938	4069	4067
3470	929	4066	4069
3471	927	4067	4066
3472	4069	4066	4067
3473	25	1196	1200
3474	396	2961	1196
3475	400	1200	2961
3476	1196	2961	1200
3477	188	2081	2078
3478	977	2963	2081
3479	396	2078	2963
3480	2081	2963	2078
3481	192	2102	2104
3482	400	2974	2102
3483	977	2104	2974
3484	2102	2974	2104
3485	396	2963	2961
3486	977	2974	2963
3487	400	2961	2974
3488	2963	2974	2961
3489	247	2434	2432
3490	979	3300	2434
3491	521	2432	3300
3492	2434	3300	2432
3493	188	2079	2083

3494	517	3291	2079
3495	979	2083	3291
3496	2079	3291	2083
3497	80	1495	1497
3498	695	3810	1495
3499	697	1497	3810
3500	1495	3810	1497
3501	192	2106	2103
3502	990	3812	2106
3503	695	2103	3812
3504	2106	3812	2103
3505	247	2433	2435
3506	697	3817	2433
3507	990	2435	3817
3508	2433	3817	2435
3509	695	3812	3810
3510	990	3817	3812
3511	697	3810	3817
3512	3812	3817	3810
3513	188	2083	2081
3514	979	4114	2083
3515	977	2081	4114
3516	2083	4114	2081
3517	247	2435	2434
3518	990	4117	2435
3519	979	2434	4117
3520	2435	4117	2434
3521	192	2104	2106
3522	977	4115	2104
3523	990	2106	4115
3524	2104	4115	2106
3525	979	4117	4114
3526	990	4115	4117
3527	977	4114	4115
3528	4117	4115	4114
3529	47	1309	1307
3530	509	3259	1309
3531	507	1307	3259
3532	1309	3259	1307
3533	243	2412	2408
3534	1045	3265	2412
3535	509	2408	3265
3536	2412	3265	2408
3537	241	2396	2400
3538	507	3261	2396
3539	1045	2400	3261
3540	2396	3261	2400
3541	509	3265	3259
3542	1045	3261	3265
3543	507	3259	3261
3544	3265	3261	3259
3545	80	1497	1496
3546	697	3814	1497
3547	696	1496	3814
3548	1497	3814	1496
3549	247	2437	2433
3550	1047	3818	2437
3551	697	2433	3818
3552	2437	3818	2433

3553	243	2409	2413
3554	696	3816	2409
3555	1047	2413	3816
3556	2409	3816	2413
3557	697	3818	3814
3558	1047	3816	3818
3559	696	3814	3816
3560	3818	3816	3814
3561	49	1319	1321
3562	519	3295	1319
3563	521	1321	3295
3564	1319	3295	1321
3565	241	2401	2397
3566	1046	3297	2401
3567	519	2397	3297
3568	2401	3297	2397
3569	243	2413	2412
3570	1047	4182	2413
3571	1045	2412	4182
3572	2413	4182	2412
3573	247	2436	2437
3574	1046	4183	2436
3575	1047	2437	4183
3576	2436	4183	2437
3577	241	2400	2401
3578	1045	4181	2400
3579	1046	2401	4181
3580	2400	4181	2401
3581	1047	4183	4182
3582	1046	4181	4183
3583	1045	4182	4181
3584	4183	4181	4182
3585	23	1189	1188
3586	389	2937	1189
3587	388	1188	2937
3588	1189	2937	1188
3589	181	2039	2036
3590	953	2941	2039
3591	389	2036	2941
3592	2039	2941	2036
3593	180	2030	2033
3594	388	2939	2030
3595	953	2033	2939
3596	2030	2939	2033
3597	389	2941	2937
3598	953	2939	2941
3599	388	2937	2939
3600	2941	2939	2937
3601	81	1504	1500
3602	704	3825	1504
3603	700	1500	3825
3604	1504	3825	1500
3605	288	2681	2679
3606	957	3838	2681
3607	704	2679	3838
3608	2681	3838	2679
3609	181	2037	2041
3610	700	3827	2037
3611	957	2041	3827

3612	2037	3827	2041
3613	704	3838	3825
3614	957	3827	3838
3615	700	3825	3827
3616	3838	3827	3825
3617	79	1489	1493
3618	689	3793	1489
3619	693	1493	3793
3620	1489	3793	1493
3621	180	2035	2031
3622	955	3795	2035
3623	689	2031	3795
3624	2035	3795	2031
3625	288	2678	2680
3626	693	3804	2678
3627	955	2680	3804
3628	2678	3804	2680
3629	689	3795	3793
3630	955	3804	3795
3631	693	3793	3804
3632	3795	3804	3793
3633	181	2041	2039
3634	957	4092	2041
3635	953	2039	4092
3636	2041	4092	2039
3637	288	2680	2681
3638	955	4093	2680
3639	957	2681	4093
3640	2680	4093	2681
3641	180	2033	2035
3642	953	4091	2033
3643	955	2035	4091
3644	2033	4091	2035
3645	957	4093	4092
3646	955	4091	4093
3647	953	4092	4091
3648	4093	4091	4092
3649	25	1200	1201
3650	400	2973	1200
3651	401	1201	2973
3652	1200	2973	1201
3653	192	2105	2102
3654	989	2975	2105
3655	400	2102	2975
3656	2105	2975	2102
3657	193	2108	2111
3658	401	2977	2108
3659	989	2111	2977
3660	2108	2977	2111
3661	400	2975	2973
3662	989	2977	2975
3663	401	2973	2977
3664	2975	2977	2973
3665	80	1499	1495
3666	699	3811	1499
3667	695	1495	3811
3668	1499	3811	1495
3669	289	2686	2684
3670	991	3822	2686

3671	699	2684	3822
3672	2686	3822	2684
3673	192	2103	2107
3674	695	3813	2103
3675	991	2107	3813
3676	2103	3813	2107
3677	699	3822	3811
3678	991	3813	3822
3679	695	3811	3813
3680	3822	3813	3811
3681	81	1501	1505
3682	701	3829	1501
3683	705	1505	3829
3684	1501	3829	1505
3685	193	2113	2109
3686	993	3831	2113
3687	701	2109	3831
3688	2113	3831	2109
3689	289	2685	2687
3690	705	3840	2685
3691	993	2687	3840
3692	2685	3840	2687
3693	701	3831	3829
3694	993	3840	3831
3695	705	3829	3840
3696	3831	3840	3829
3697	192	2107	2105
3698	991	4127	2107
3699	989	2105	4127
3700	2107	4127	2105
3701	289	2687	2686
3702	993	4129	2687
3703	991	2686	4129
3704	2687	4129	2686
3705	193	2111	2113
3706	989	4128	2111
3707	993	2113	4128
3708	2111	4128	2113
3709	991	4129	4127
3710	993	4128	4129
3711	989	4127	4128
3712	4129	4128	4127
3713	22	1182	1183
3714	382	2919	1182
3715	383	1183	2919
3716	1182	2919	1183
3717	174	1997	1994
3718	935	2921	1997
3719	382	1994	2921
3720	1997	2921	1994
3721	175	2000	2003
3722	383	2923	2000
3723	935	2003	2923
3724	2000	2923	2003
3725	382	2921	2919
3726	935	2923	2921
3727	383	2919	2923
3728	2921	2923	2919
3729	79	1492	1488

3730	692	3789	1492
3731	688	1488	3789
3732	1492	3789	1488
3733	287	2674	2672
3734	937	3802	2674
3735	692	2672	3802
3736	2674	3802	2672
3737	174	1995	1999
3738	688	3791	1995
3739	937	1999	3791
3740	1995	3791	1999
3741	692	3802	3789
3742	937	3791	3802
3743	688	3789	3791
3744	3802	3791	3789
3745	174	1999	1997
3746	937	4073	1999
3747	935	1997	4073
3748	1999	4073	1997
3749	287	2675	2674
3750	939	4075	2675
3751	937	2674	4075
3752	2675	4075	2674
3753	175	2003	2005
3754	935	4074	2003
3755	939	2005	4074
3756	2003	4074	2005
3757	937	4075	4073
3758	939	4074	4075
3759	935	4073	4074
3760	4075	4074	4073
3761	81	1505	1504
3762	705	3837	1505
3763	704	1504	3837
3764	1505	3837	1504
3765	289	2689	2685
3766	1089	3841	2689
3767	705	2685	3841
3768	2689	3841	2685
3769	288	2679	2683
3770	704	3839	2679
3771	1089	2683	3839
3772	2679	3839	2683
3773	705	3841	3837
3774	1089	3839	3841
3775	704	3837	3839
3776	3841	3839	3837
3777	80	1498	1499
3778	698	3819	1498
3779	699	1499	3819
3780	1498	3819	1499
3781	287	2677	2673
3782	1088	3821	2677
3783	698	2673	3821
3784	2677	3821	2673
3785	289	2684	2688
3786	699	3823	2684
3787	1088	2688	3823
3788	2684	3823	2688

3789	698	3821	3819
3790	1088	3823	3821
3791	699	3819	3823
3792	3821	3823	3819
3793	79	1493	1492
3794	693	3801	1493
3795	692	1492	3801
3796	1493	3801	1492
3797	288	2682	2678
3798	1087	3805	2682
3799	693	2678	3805
3800	2682	3805	2678
3801	287	2672	2676
3802	692	3803	2672
3803	1087	2676	3803
3804	2672	3803	2676
3805	693	3805	3801
3806	1087	3803	3805
3807	692	3801	3803
3808	3805	3803	3801
3809	289	2688	2689
3810	1088	4225	2688
3811	1089	2689	4225
3812	2688	4225	2689
3813	287	2676	2677
3814	1087	4223	2676
3815	1088	2677	4223
3816	2676	4223	2677
3817	288	2683	2682
3818	1089	4224	2683
3819	1087	2682	4224
3820	2683	4224	2682
3821	1088	4223	4225
3822	1087	4224	4223
3823	1089	4225	4224
3824	4223	4224	4225
3825	84	1516	1514
3826	706	2694	1516
3827	292	1514	2694
3828	1516	2694	1514
3829	82	1506	1508
3830	290	2691	1506
3831	706	1508	2691
3832	1506	2691	1508
3833	292	2694	2690
3834	706	2691	2694
3835	290	2690	2691
3836	2694	2691	2690
3837	28	1212	1210
3838	412	2996	1212
3839	410	1210	2996
3840	1212	2996	1210
3841	194	2117	2115
3842	710	3005	2117
3843	412	2115	3005
3844	2117	3005	2115
3845	84	1515	1518
3846	410	2998	1515
3847	710	1518	2998

3848	1515	2998	1518
3849	412	3005	2996
3850	710	2998	3005
3851	410	2996	2998
3852	3005	2998	2996
3853	26	1202	1204
3854	402	2978	1202
3855	404	1204	2978
3856	1202	2978	1204
3857	82	1509	1507
3858	707	2979	1509
3859	402	1507	2979
3860	1509	2979	1507
3861	194	2114	2116
3862	404	2983	2114
3863	707	2116	2983
3864	2114	2983	2116
3865	402	2979	2978
3866	707	2983	2979
3867	404	2978	2983
3868	2979	2983	2978
3869	84	1518	1516
3870	710	3843	1518
3871	706	1516	3843
3872	1518	3843	1516
3873	194	2116	2117
3874	707	3844	2116
3875	710	2117	3844
3876	2116	3844	2117
3877	82	1508	1509
3878	706	3842	1508
3879	707	1509	3842
3880	1508	3842	1509
3881	710	3844	3843
3882	707	3842	3844
3883	706	3843	3842
3884	3844	3842	3843
3885	12	1132	1130
3886	332	2780	1132
3887	330	1130	2780
3888	1132	2780	1130
3889	124	1720	1718
3890	796	2789	1720
3891	332	1718	2789
3892	1720	2789	1718
3893	122	1706	1708
3894	330	2782	1706
3895	796	1708	2782
3896	1706	2782	1708
3897	332	2789	2780
3898	796	2782	2789
3899	330	2780	2782
3900	2789	2782	2780
3901	58	1365	1363
3902	565	3414	1365
3903	563	1363	3414
3904	1365	3414	1363
3905	198	2141	2139
3906	805	3421	2141

3907	565	2139	3421
3908	2141	3421	2139
3909	124	1719	1722
3910	563	3416	1719
3911	805	1722	3416
3912	1719	3416	1722
3913	565	3421	3414
3914	805	3416	3421
3915	563	3414	3416
3916	3421	3416	3414
3917	28	1211	1214
3918	411	3000	1211
3919	414	1214	3000
3920	1211	3000	1214
3921	122	1710	1707
3922	798	3002	1710
3923	411	1707	3002
3924	1710	3002	1707
3925	198	2138	2140
3926	414	3010	2138
3927	798	2140	3010
3928	2138	3010	2140
3929	411	3002	3000
3930	798	3010	3002
3931	414	3000	3010
3932	3002	3010	3000
3933	124	1722	1720
3934	805	3933	1722
3935	796	1720	3933
3936	1722	3933	1720
3937	198	2140	2141
3938	798	3936	2140
3939	805	2141	3936
3940	2140	3936	2141
3941	122	1708	1710
3942	796	3932	1708
3943	798	1710	3932
3944	1708	3932	1710
3945	805	3936	3933
3946	798	3932	3936
3947	796	3933	3932
3948	3936	3932	3933
3949	10	1122	1124
3950	322	2762	1122
3951	324	1124	2762
3952	1122	2762	1124
3953	114	1668	1666
3954	778	2763	1668
3955	322	1666	2763
3956	1668	2763	1666
3957	116	1674	1676
3958	324	2767	1674
3959	778	1676	2767
3960	1674	2767	1676
3961	322	2763	2762
3962	778	2767	2763
3963	324	2762	2767
3964	2763	2767	2762
3965	26	1205	1203

3966	405	2980	1205
3967	403	1203	2980
3968	1205	2980	1203
3969	195	2122	2120
3970	779	2985	2122
3971	405	2120	2985
3972	2122	2985	2120
3973	114	1667	1669
3974	403	2981	1667
3975	779	1669	2981
3976	1667	2981	1669
3977	405	2985	2980
3978	779	2981	2985
3979	403	2980	2981
3980	2985	2981	2980
3981	58	1362	1364
3982	562	3410	1362
3983	564	1364	3410
3984	1362	3410	1364
3985	116	1678	1675
3986	783	3412	1678
3987	562	1675	3412
3988	1678	3412	1675
3989	195	2121	2123
3990	564	3419	2121
3991	783	2123	3419
3992	2121	3419	2123
3993	562	3412	3410
3994	783	3419	3412
3995	564	3410	3419
3996	3412	3419	3410
3997	114	1669	1668
3998	779	3914	1669
3999	778	1668	3914
4000	1669	3914	1668
4001	195	2123	2122
4002	783	3916	2123
4003	779	2122	3916
4004	2123	3916	2122
4005	116	1676	1678
4006	778	3915	1676
4007	783	1678	3915
4008	1676	3915	1678
4009	779	3916	3914
4010	783	3915	3916
4011	778	3914	3915
4012	3916	3915	3914
4013	28	1214	1212
4014	414	3004	1214
4015	412	1212	3004
4016	1214	3004	1212
4017	198	2142	2138
4018	995	3011	2142
4019	414	2138	3011
4020	2142	3011	2138
4021	194	2115	2119
4022	412	3006	2115
4023	995	2119	3006
4024	2115	3006	2119

4025	414	3011	3004
4026	995	3006	3011
4027	412	3004	3006
4028	3011	3006	3004
4029	58	1364	1365
4030	564	3418	1364
4031	565	1365	3418
4032	1364	3418	1365
4033	195	2125	2121
4034	996	3420	2125
4035	564	2121	3420
4036	2125	3420	2121
4037	198	2139	2143
4038	565	3422	2139
4039	996	2143	3422
4040	2139	3422	2143
4041	564	3420	3418
4042	996	3422	3420
4043	565	3418	3422
4044	3420	3422	3418
4045	26	1204	1205
4046	404	2982	1204
4047	405	1205	2982
4048	1204	2982	1205
4049	194	2118	2114
4050	994	2984	2118
4051	404	2114	2984
4052	2118	2984	2114
4053	195	2120	2124
4054	405	2986	2120
4055	994	2124	2986
4056	2120	2986	2124
4057	404	2984	2982
4058	994	2986	2984
4059	405	2982	2986
4060	2984	2986	2982
4061	198	2143	2142
4062	996	4132	2143
4063	995	2142	4132
4064	2143	4132	2142
4065	195	2124	2125
4066	994	4131	2124
4067	996	2125	4131
4068	2124	4131	2125
4069	194	2119	2118
4070	995	4130	2119
4071	994	2118	4130
4072	2119	4130	2118
4073	996	4131	4132
4074	994	4130	4131
4075	995	4132	4130
4076	4131	4130	4132
4077	7	1110	1108
4078	310	2726	1110
4079	308	1108	2726
4080	1110	2726	1108
4081	102	1604	1602
4082	742	2735	1604
4083	310	1602	2735

4084	1604	2735	1602
4085	100	1590	1592
4086	308	2728	1590
4087	742	1592	2728
4088	1590	2728	1592
4089	310	2735	2726
4090	742	2728	2735
4091	308	2726	2728
4092	2735	2728	2726
4093	46	1300	1298
4094	500	3230	1300
4095	498	1298	3230
4096	1300	3230	1298
4097	230	2333	2331
4098	751	3239	2333
4099	500	2331	3239
4100	2333	3239	2331
4101	102	1603	1606
4102	498	3232	1603
4103	751	1606	3232
4104	1603	3232	1606
4105	500	3239	3230
4106	751	3232	3239
4107	498	3230	3232
4108	3239	3232	3230
4109	230	2330	2332
4110	488	3203	2330
4111	744	2332	3203
4112	2330	3203	2332
4113	102	1606	1604
4114	751	3879	1606
4115	742	1604	3879
4116	1606	3879	1604
4117	230	2332	2333
4118	744	3882	2332
4119	751	2333	3882
4120	2332	3882	2333
4121	100	1592	1594
4122	742	3878	1592
4123	744	1594	3878
4124	1592	3878	1594
4125	751	3882	3879
4126	744	3878	3882
4127	742	3879	3878
4128	3882	3878	3879
4129	20	1169	1167
4130	369	2874	1169
4131	367	1167	2874
4132	1169	2874	1167
4133	161	1918	1916
4134	890	2881	1918
4135	369	1916	2881
4136	1918	2881	1916
4137	159	1904	1906
4138	367	2876	1904
4139	890	1906	2876
4140	1904	2876	1906
4141	369	2881	2874
4142	890	2876	2881

4143	367	2874	2876
4144	2881	2876	2874
4145	62	1389	1387
4146	589	3486	1389
4147	587	1387	3486
4148	1389	3486	1387
4149	239	2387	2385
4150	897	3493	2387
4151	589	2385	3493
4152	2387	3493	2385
4153	161	1917	1920
4154	587	3488	1917
4155	897	1920	3488
4156	1917	3488	1920
4157	589	3493	3486
4158	897	3488	3493
4159	587	3486	3488
4160	3493	3488	3486
4161	46	1299	1302
4162	499	3234	1299
4163	502	1302	3234
4164	1299	3234	1302
4165	159	1908	1905
4166	892	3236	1908
4167	499	1905	3236
4168	1908	3236	1905
4169	239	2384	2386
4170	502	3244	2384
4171	892	2386	3244
4172	2384	3244	2386
4173	499	3236	3234
4174	892	3244	3236
4175	502	3234	3244
4176	3236	3244	3234
4177	161	1920	1918
4178	897	4029	1920
4179	890	1918	4029
4180	1920	4029	1918
4181	239	2386	2387
4182	892	4032	2386
4183	897	2387	4032
4184	2386	4032	2387
4185	159	1906	1908
4186	890	4028	1906
4187	892	1908	4028
4188	1906	4028	1908
4189	897	4032	4029
4190	892	4028	4032
4191	890	4029	4028
4192	4032	4028	4029
4193	12	1131	1134
4194	331	2784	1131
4195	334	1134	2784
4196	1131	2784	1134
4197	123	1714	1712
4198	800	2786	1714
4199	331	1712	2786
4200	1714	2786	1712
4201	126	1730	1732

4202	334	2794	1730
4203	800	1732	2794
4204	1730	2794	1732
4205	331	2786	2784
4206	800	2794	2786
4207	334	2784	2794
4208	2786	2794	2784
4209	44	1290	1287
4210	490	3198	1290
4211	487	1287	3198
4212	1290	3198	1287
4213	232	2344	2342
4214	802	3208	2344
4215	490	2342	3208
4216	2344	3208	2342
4217	123	1713	1716
4218	487	3200	1713
4219	802	1716	3200
4220	1713	3200	1716
4221	490	3208	3198
4222	802	3200	3208
4223	487	3198	3200
4224	3208	3200	3198
4225	62	1386	1388
4226	586	3482	1386
4227	588	1388	3482
4228	1386	3482	1388
4229	126	1734	1731
4230	810	3484	1734
4231	586	1731	3484
4232	1734	3484	1731
4233	232	2343	2345
4234	588	3491	2343
4235	810	2345	3491
4236	2343	3491	2345
4237	586	3484	3482
4238	810	3491	3484
4239	588	3482	3491
4240	3484	3491	3482
4241	123	1716	1714
4242	802	3938	1716
4243	800	1714	3938
4244	1716	3938	1714
4245	232	2345	2344
4246	810	3942	2345
4247	802	2344	3942
4248	2345	3942	2344
4249	126	1732	1734
4250	800	3939	1732
4251	810	1734	3939
4252	1732	3939	1734
4253	802	3942	3938
4254	810	3939	3942
4255	800	3938	3939
4256	3942	3939	3938
4257	46	1302	1300
4258	502	3238	1302
4259	500	1300	3238
4260	1302	3238	1300

4261	239	2388	2384
4262	1031	3245	2388
4263	502	2384	3245
4264	2388	3245	2384
4265	230	2331	2335
4266	500	3240	2331
4267	1031	2335	3240
4268	2331	3240	2335
4269	502	3245	3238
4270	1031	3240	3245
4271	500	3238	3240
4272	3245	3240	3238
4273	62	1388	1389
4274	588	3490	1388
4275	589	1389	3490
4276	1388	3490	1389
4277	232	2347	2343
4278	1034	3492	2347
4279	588	2343	3492
4280	2347	3492	2343
4281	239	2385	2389
4282	589	3494	2385
4283	1034	2389	3494
4284	2385	3494	2389
4285	588	3492	3490
4286	1034	3494	3492
4287	589	3490	3494
4288	3492	3494	3490
4289	44	1288	1290
4290	488	3202	1288
4291	490	1290	3202
4292	1288	3202	1290
4293	230	2334	2330
4294	1030	3204	2334
4295	488	2330	3204
4296	2334	3204	2330
4297	232	2342	2346
4298	490	3209	2342
4299	1030	2346	3209
4300	2342	3209	2346
4301	488	3204	3202
4302	1030	3209	3204
4303	490	3202	3209
4304	3204	3209	3202
4305	239	2389	2388
4306	1034	4168	2389
4307	1031	2388	4168
4308	2389	4168	2388
4309	232	2346	2347
4310	1030	4167	2346
4311	1034	2347	4167
4312	2346	4167	2347
4313	230	2335	2334
4314	1031	4166	2335
4315	1030	2334	4166
4316	2335	4166	2334
4317	1034	4167	4168
4318	1030	4166	4167
4319	1031	4168	4166

4320	4167	4166	4168
4321	5	1100	1102
4322	300	2708	1100
4323	302	1102	2708
4324	1100	2708	1102
4325	92	1552	1550
4326	724	2709	1552
4327	300	1550	2709
4328	1552	2709	1550
4329	94	1558	1560
4330	302	2713	1558
4331	724	1560	2713
4332	1558	2713	1560
4333	300	2709	2708
4334	724	2713	2709
4335	302	2708	2713
4336	2709	2713	2708
4337	36	1248	1246
4338	448	3086	1248
4339	446	1246	3086
4340	1248	3086	1246
4341	212	2224	2222
4342	725	3091	2224
4343	448	2222	3091
4344	2224	3091	2222
4345	92	1551	1553
4346	446	3087	1551
4347	725	1553	3087
4348	1551	3087	1553
4349	448	3091	3086
4350	725	3087	3091
4351	446	3086	3087
4352	3091	3087	3086
4353	38	1254	1256
4354	454	3104	1254
4355	456	1256	3104
4356	1254	3104	1256
4357	94	1562	1559
4358	729	3106	1562
4359	454	1559	3106
4360	1562	3106	1559
4361	212	2223	2225
4362	456	3113	2223
4363	729	2225	3113
4364	2223	3113	2225
4365	454	3106	3104
4366	729	3113	3106
4367	456	3104	3113
4368	3106	3113	3104
4369	92	1553	1552
4370	725	3860	1553
4371	724	1552	3860
4372	1553	3860	1552
4373	212	2225	2224
4374	729	3862	2225
4375	725	2224	3862
4376	2225	3862	2224
4377	94	1560	1562
4378	724	3861	1560

4379	729	1562	3861
4380	1560	3861	1562
4381	725	3862	3860
4382	729	3861	3862
4383	724	3860	3861
4384	3862	3861	3860
4385	10	1125	1123
4386	325	2764	1125
4387	323	1123	2764
4388	1125	2764	1123
4389	117	1682	1680
4390	780	2769	1682
4391	325	1680	2769
4392	1682	2769	1680
4393	115	1670	1672
4394	323	2765	1670
4395	780	1672	2765
4396	1670	2765	1672
4397	325	2769	2764
4398	780	2765	2769
4399	323	2764	2765
4400	2769	2765	2764
4401	59	1370	1368
4402	570	3428	1370
4403	568	1368	3428
4404	1370	3428	1368
4405	213	2231	2229
4406	785	3437	2231
4407	570	2229	3437
4408	2231	3437	2229
4409	117	1681	1684
4410	568	3430	1681
4411	785	1684	3430
4412	1681	3430	1684
4413	570	3437	3428
4414	785	3430	3437
4415	568	3428	3430
4416	3437	3430	3428
4417	36	1247	1249
4418	447	3088	1247
4419	449	1249	3088
4420	1247	3088	1249
4421	115	1673	1671
4422	781	3089	1673
4423	447	1671	3089
4424	1673	3089	1671
4425	213	2228	2230
4426	449	3093	2228
4427	781	2230	3093
4428	2228	3093	2230
4429	447	3089	3088
4430	781	3093	3089
4431	449	3088	3093
4432	3089	3093	3088
4433	117	1684	1682
4434	785	3918	1684
4435	780	1682	3918
4436	1684	3918	1682
4437	213	2230	2231

4438	781	3919	2230
4439	785	2231	3919
4440	2230	3919	2231
4441	115	1672	1673
4442	780	3917	1672
4443	781	1673	3917
4444	1672	3917	1673
4445	785	3919	3918
4446	781	3917	3919
4447	780	3918	3917
4448	3919	3917	3918
4449	20	1166	1168
4450	366	2870	1166
4451	368	1168	2870
4452	1166	2870	1168
4453	158	1900	1898
4454	886	2872	1900
4455	366	1898	2872
4456	1900	2872	1898
4457	160	1910	1912
4458	368	2879	1910
4459	886	1912	2879
4460	1910	2879	1912
4461	366	2872	2870
4462	886	2879	2872
4463	368	2870	2879
4464	2872	2879	2870
4465	38	1258	1255
4466	458	3108	1258
4467	455	1255	3108
4468	1258	3108	1255
4469	217	2254	2252
4470	888	3118	2254
4471	458	2252	3118
4472	2254	3118	2252
4473	158	1899	1902
4474	455	3110	1899
4475	888	1902	3110
4476	1899	3110	1902
4477	458	3118	3108
4478	888	3110	3118
4479	455	3108	3110
4480	3118	3110	3108
4481	59	1369	1371
4482	569	3432	1369
4483	571	1371	3432
4484	1369	3432	1371
4485	160	1914	1911
4486	895	3434	1914
4487	569	1911	3434
4488	1914	3434	1911
4489	217	2253	2255
4490	571	3439	2253
4491	895	2255	3439
4492	2253	3439	2255
4493	569	3434	3432
4494	895	3439	3434
4495	571	3432	3439
4496	3434	3439	3432

4497	158	1902	1900
4498	888	4022	1902
4499	886	1900	4022
4500	1902	4022	1900
4501	217	2255	2254
4502	895	4026	2255
4503	888	2254	4026
4504	2255	4026	2254
4505	160	1912	1914
4506	886	4023	1912
4507	895	1914	4023
4508	1912	4023	1914
4509	888	4026	4022
4510	895	4023	4026
4511	886	4022	4023
4512	4026	4023	4022
4513	36	1249	1248
4514	449	3090	1249
4515	448	1248	3090
4516	1249	3090	1248
4517	213	2232	2228
4518	1012	3094	2232
4519	449	2228	3094
4520	2232	3094	2228
4521	212	2222	2226
4522	448	3092	2222
4523	1012	2226	3092
4524	2222	3092	2226
4525	449	3094	3090
4526	1012	3092	3094
4527	448	3090	3092
4528	3094	3092	3090
4529	59	1371	1370
4530	571	3436	1371
4531	570	1370	3436
4532	1371	3436	1370
4533	217	2257	2253
4534	1014	3440	2257
4535	571	2253	3440
4536	2257	3440	2253
4537	213	2229	2233
4538	570	3438	2229
4539	1014	2233	3438
4540	2229	3438	2233
4541	571	3440	3436
4542	1014	3438	3440
4543	570	3436	3438
4544	3440	3438	3436
4545	38	1256	1258
4546	456	3112	1256
4547	458	1258	3112
4548	1256	3112	1258
4549	212	2227	2223
4550	1013	3114	2227
4551	456	2223	3114
4552	2227	3114	2223
4553	217	2252	2256
4554	458	3119	2252
4555	1013	2256	3119

4556	2252	3119	2256
4557	456	3114	3112
4558	1013	3119	3114
4559	458	3112	3119
4560	3114	3119	3112
4561	213	2233	2232
4562	1014	4149	2233
4563	1012	2232	4149
4564	2233	4149	2232
4565	217	2256	2257
4566	1013	4150	2256
4567	1014	2257	4150
4568	2256	4150	2257
4569	212	2226	2227
4570	1012	4148	2226
4571	1013	2227	4148
4572	2226	4148	2227
4573	1014	4150	4149
4574	1013	4148	4150
4575	1012	4149	4148
4576	4150	4148	4149
4577	12	1134	1132
4578	334	2788	1134
4579	332	1132	2788
4580	1134	2788	1132
4581	126	1733	1730
4582	804	2795	1733
4583	334	1730	2795
4584	1733	2795	1730
4585	124	1718	1721
4586	332	2790	1718
4587	804	1721	2790
4588	1718	2790	1721
4589	334	2795	2788
4590	804	2790	2795
4591	332	2788	2790
4592	2795	2790	2788
4593	62	1390	1386
4594	590	3483	1390
4595	586	1386	3483
4596	1390	3483	1386
4597	267	2555	2553
4598	811	3496	2555
4599	590	2553	3496
4600	2555	3496	2553
4601	126	1731	1735
4602	586	3485	1731
4603	811	1735	3485
4604	1731	3485	1735
4605	590	3496	3483
4606	811	3485	3496
4607	586	3483	3485
4608	3496	3485	3483
4609	58	1363	1367
4610	563	3415	1363
4611	567	1367	3415
4612	1363	3415	1367
4613	124	1723	1719
4614	806	3417	1723

4615	563	1719	3417
4616	1723	3417	1719
4617	267	2552	2554
4618	567	3426	2552
4619	806	2554	3426
4620	2552	3426	2554
4621	563	3417	3415
4622	806	3426	3417
4623	567	3415	3426
4624	3417	3426	3415
4625	126	1735	1733
4626	811	3945	1735
4627	804	1733	3945
4628	1735	3945	1733
4629	267	2554	2555
4630	806	3946	2554
4631	811	2555	3946
4632	2554	3946	2555
4633	124	1721	1723
4634	804	3944	1721
4635	806	1723	3944
4636	1721	3944	1723
4637	811	3946	3945
4638	806	3944	3946
4639	804	3945	3944
4640	3946	3944	3945
4641	20	1168	1169
4642	368	2878	1168
4643	369	1169	2878
4644	1168	2878	1169
4645	160	1913	1910
4646	894	2880	1913
4647	368	1910	2880
4648	1913	2880	1910
4649	161	1916	1919
4650	369	2882	1916
4651	894	1919	2882
4652	1916	2882	1919
4653	368	2880	2878
4654	894	2882	2880
4655	369	2878	2882
4656	2880	2882	2878
4657	59	1373	1369
4658	573	3433	1373
4659	569	1369	3433
4660	1373	3433	1369
4661	268	2560	2558
4662	896	3444	2560
4663	573	2558	3444
4664	2560	3444	2558
4665	160	1911	1915
4666	569	3435	1911
4667	896	1915	3435
4668	1911	3435	1915
4669	573	3444	3433
4670	896	3435	3444
4671	569	3433	3435
4672	3444	3435	3433
4673	62	1387	1391

4674	587	3487	1387
4675	591	1391	3487
4676	1387	3487	1391
4677	161	1921	1917
4678	898	3489	1921
4679	587	1917	3489
4680	1921	3489	1917
4681	268	2559	2561
4682	591	3498	2559
4683	898	2561	3498
4684	2559	3498	2561
4685	587	3489	3487
4686	898	3498	3489
4687	591	3487	3498
4688	3489	3498	3487
4689	160	1915	1913
4690	896	4034	1915
4691	894	1913	4034
4692	1915	4034	1913
4693	268	2561	2560
4694	898	4036	2561
4695	896	2560	4036
4696	2561	4036	2560
4697	161	1919	1921
4698	894	4035	1919
4699	898	1921	4035
4700	1919	4035	1921
4701	896	4036	4034
4702	898	4035	4036
4703	894	4034	4035
4704	4036	4035	4034
4705	10	1124	1125
4706	324	2766	1124
4707	325	1125	2766
4708	1124	2766	1125
4709	116	1677	1674
4710	782	2768	1677
4711	324	1674	2768
4712	1677	2768	1674
4713	117	1680	1683
4714	325	2770	1680
4715	782	1683	2770
4716	1680	2770	1683
4717	324	2768	2766
4718	782	2770	2768
4719	325	2766	2770
4720	2768	2770	2766
4721	58	1366	1362
4722	566	3411	1366
4723	562	1362	3411
4724	1366	3411	1362
4725	266	2548	2546
4726	784	3424	2548
4727	566	2546	3424
4728	2548	3424	2546
4729	116	1675	1679
4730	562	3413	1675
4731	784	1679	3413
4732	1675	3413	1679

4733	566	3424	3411
4734	784	3413	3424
4735	562	3411	3413
4736	3424	3413	3411
4737	59	1368	1372
4738	568	3429	1368
4739	572	1372	3429
4740	1368	3429	1372
4741	117	1685	1681
4742	786	3431	1685
4743	568	1681	3431
4744	1685	3431	1681
4745	266	2547	2549
4746	572	3442	2547
4747	786	2549	3442
4748	2547	3442	2549
4749	568	3431	3429
4750	786	3442	3431
4751	572	3429	3442
4752	3431	3442	3429
4753	116	1679	1677
4754	784	3920	1679
4755	782	1677	3920
4756	1679	3920	1677
4757	266	2549	2548
4758	786	3922	2549
4759	784	2548	3922
4760	2549	3922	2548
4761	117	1683	1685
4762	782	3921	1683
4763	786	1685	3921
4764	1683	3921	1685
4765	784	3922	3920
4766	786	3921	3922
4767	782	3920	3921
4768	3922	3921	3920
4769	62	1391	1390
4770	591	3495	1391
4771	590	1390	3495
4772	1391	3495	1390
4773	268	2563	2559
4774	1068	3499	2563
4775	591	2559	3499
4776	2563	3499	2559
4777	267	2553	2557
4778	590	3497	2553
4779	1068	2557	3497
4780	2553	3497	2557
4781	591	3499	3495
4782	1068	3497	3499
4783	590	3495	3497
4784	3499	3497	3495
4785	59	1372	1373
4786	572	3441	1372
4787	573	1373	3441
4788	1372	3441	1373
4789	266	2551	2547
4790	1067	3443	2551
4791	572	2547	3443

4792	2551	3443	2547
4793	268	2558	2562
4794	573	3445	2558
4795	1067	2562	3445
4796	2558	3445	2562
4797	572	3443	3441
4798	1067	3445	3443
4799	573	3441	3445
4800	3443	3445	3441
4801	58	1367	1366
4802	567	3423	1367
4803	566	1366	3423
4804	1367	3423	1366
4805	267	2556	2552
4806	1066	3427	2556
4807	567	2552	3427
4808	2556	3427	2552
4809	266	2546	2550
4810	566	3425	2546
4811	1066	2550	3425
4812	2546	3425	2550
4813	567	3427	3423
4814	1066	3425	3427
4815	566	3423	3425
4816	3427	3425	3423
4817	268	2562	2563
4818	1067	4204	2562
4819	1068	2563	4204
4820	2562	4204	2563
4821	266	2550	2551
4822	1066	4202	2550
4823	1067	2551	4202
4824	2550	4202	2551
4825	267	2557	2556
4826	1068	4203	2557
4827	1066	2556	4203
4828	2557	4203	2556
4829	1067	4202	4204
4830	1066	4203	4202
4831	1068	4204	4203
4832	4202	4203	4204
4833	4	1098	1097
4834	298	2702	1098
4835	297	1097	2702
4836	1098	2702	1097
4837	90	1544	1542
4838	718	2706	1544
4839	298	1542	2706
4840	1544	2706	1542
4841	89	1536	1538
4842	297	2704	1536
4843	718	1538	2704
4844	1536	2704	1538
4845	298	2706	2702
4846	718	2704	2706
4847	297	2702	2704
4848	2706	2704	2702
4849	34	1240	1238
4850	440	3068	1240

4851	438	1238	3068
4852	1240	3068	1238
4853	206	2189	2187
4854	722	3073	2189
4855	440	2187	3073
4856	2189	3073	2187
4857	90	1543	1545
4858	438	3069	1543
4859	722	1545	3069
4860	1543	3069	1545
4861	440	3073	3068
4862	722	3069	3073
4863	438	3068	3069
4864	3073	3069	3068
4865	33	1232	1234
4866	432	3050	1232
4867	434	1234	3050
4868	1232	3050	1234
4869	89	1540	1537
4870	720	3052	1540
4871	432	1537	3052
4872	1540	3052	1537
4873	206	2186	2188
4874	434	3059	2186
4875	720	2188	3059
4876	2186	3059	2188
4877	432	3052	3050
4878	720	3059	3052
4879	434	3050	3059
4880	3052	3059	3050
4881	90	1545	1544
4882	722	3855	1545
4883	718	1544	3855
4884	1545	3855	1544
4885	206	2188	2189
4886	720	3858	2188
4887	722	2189	3858
4888	2188	3858	2189
4889	89	1538	1540
4890	718	3854	1538
4891	720	1540	3854
4892	1538	3854	1540
4893	722	3858	3855
4894	720	3854	3858
4895	718	3855	3854
4896	3858	3854	3855
4897	18	1160	1158
4898	360	2852	1160
4899	358	1158	2852
4900	1160	2852	1158
4901	152	1868	1866
4902	868	2857	1868
4903	360	1866	2857
4904	1868	2857	1866
4905	150	1858	1860
4906	358	2853	1858
4907	868	1860	2853
4908	1858	2853	1860
4909	360	2857	2852

4910	868	2853	2857
4911	358	2852	2853
4912	2857	2853	2852
4913	70	1437	1435
4914	637	3630	1437
4915	635	1435	3630
4916	1437	3630	1435
4917	210	2213	2211
4918	873	3637	2213
4919	637	2211	3637
4920	2213	3637	2211
4921	152	1867	1870
4922	635	3632	1867
4923	873	1870	3632
4924	1867	3632	1870
4925	637	3637	3630
4926	873	3632	3637
4927	635	3630	3632
4928	3637	3632	3630
4929	34	1239	1241
4930	439	3070	1239
4931	441	1241	3070
4932	1239	3070	1241
4933	150	1861	1859
4934	869	3071	1861
4935	439	1859	3071
4936	1861	3071	1859
4937	210	2210	2212
4938	441	3075	2210
4939	869	2212	3075
4940	2210	3075	2212
4941	439	3071	3070
4942	869	3075	3071
4943	441	3070	3075
4944	3071	3075	3070
4945	152	1870	1868
4946	873	4005	1870
4947	868	1868	4005
4948	1870	4005	1868
4949	210	2212	2213
4950	869	4006	2212
4951	873	2213	4006
4952	2212	4006	2213
4953	150	1860	1861
4954	868	4004	1860
4955	869	1861	4004
4956	1860	4004	1861
4957	873	4006	4005
4958	869	4004	4006
4959	868	4005	4004
4960	4006	4004	4005
4961	17	1152	1154
4962	352	2834	1152
4963	354	1154	2834
4964	1152	2834	1154
4965	144	1824	1822
4966	850	2836	1824
4967	352	1822	2836
4968	1824	2836	1822

4969	146	1834	1836
4970	354	2843	1834
4971	850	1836	2843
4972	1834	2843	1836
4973	352	2836	2834
4974	850	2843	2836
4975	354	2834	2843
4976	2836	2843	2834
4977	70	1434	1436
4978	634	3626	1434
4979	636	1436	3626
4980	1434	3626	1436
4981	146	1838	1835
4982	859	3628	1838
4983	634	1835	3628
4984	1838	3628	1835
4985	208	2199	2201
4986	636	3635	2199
4987	859	2201	3635
4988	2199	3635	2201
4989	634	3628	3626
4990	859	3635	3628
4991	636	3626	3635
4992	3628	3635	3626
4993	144	1826	1824
4994	852	3986	1826
4995	850	1824	3986
4996	1826	3986	1824
4997	208	2201	2200
4998	859	3990	2201
4999	852	2200	3990
5000	2201	3990	2200
5001	146	1836	1838
5002	850	3987	1836
5003	859	1838	3987
5004	1836	3987	1838
5005	852	3990	3986
5006	859	3987	3990
5007	850	3986	3987
5008	3990	3987	3986
5009	34	1241	1240
5010	441	3072	1241
5011	440	1240	3072
5012	1241	3072	1240
5013	210	2214	2210
5014	1007	3076	2214
5015	441	2210	3076
5016	2214	3076	2210
5017	206	2187	2191
5018	440	3074	2187
5019	1007	2191	3074
5020	2187	3074	2191
5021	441	3076	3072
5022	1007	3074	3076
5023	440	3072	3074
5024	3076	3074	3072
5025	70	1436	1437
5026	636	3634	1436
5027	637	1437	3634

5028	1436	3634	1437
5029	208	2203	2199
5030	1010	3636	2203
5031	636	2199	3636
5032	2203	3636	2199
5033	210	2211	2215
5034	637	3638	2211
5035	1010	2215	3638
5036	2211	3638	2215
5037	636	3636	3634
5038	1010	3638	3636
5039	637	3634	3638
5040	3636	3638	3634
5041	33	1234	1236
5042	434	3058	1234
5043	436	1236	3058
5044	1234	3058	1236
5045	206	2190	2186
5046	1006	3060	2190
5047	434	2186	3060
5048	2190	3060	2186
5049	208	2198	2202
5050	436	3065	2198
5051	1006	2202	3065
5052	2198	3065	2202
5053	434	3060	3058
5054	1006	3065	3060
5055	436	3058	3065
5056	3060	3065	3058
5057	210	2215	2214
5058	1010	4144	2215
5059	1007	2214	4144
5060	2215	4144	2214
5061	208	2202	2203
5062	1006	4143	2202
5063	1010	2203	4143
5064	2202	4143	2203
5065	206	2191	2190
5066	1007	4142	2191
5067	1006	2190	4142
5068	2191	4142	2190
5069	1010	4143	4144
5070	1006	4142	4143
5071	1007	4144	4142
5072	4143	4142	4144
5073	24	1193	1191
5074	393	2946	1193
5075	391	1191	2946
5076	1193	2946	1191
5077	185	2062	2060
5078	962	2953	2062
5079	393	2060	2953
5080	2062	2953	2060
5081	183	2048	2050
5082	391	2948	2048
5083	962	2050	2948
5084	2048	2948	2050
5085	393	2953	2946
5086	962	2948	2953

5087	391	2946	2948
5088	2953	2948	2946
5089	74	1461	1459
5090	661	3702	1461
5091	659	1459	3702
5092	1461	3702	1459
5093	255	2483	2481
5094	969	3709	2483
5095	661	2481	3709
5096	2483	3709	2481
5097	185	2061	2064
5098	659	3704	2061
5099	969	2064	3704
5100	2061	3704	2064
5101	661	3709	3702
5102	969	3704	3709
5103	659	3702	3704
5104	3709	3704	3702
5105	53	1337	1340
5106	537	3342	1337
5107	540	1340	3342
5108	1337	3342	1340
5109	183	2052	2049
5110	964	3344	2052
5111	537	2049	3344
5112	2052	3344	2049
5113	255	2480	2482
5114	540	3352	2480
5115	964	2482	3352
5116	2480	3352	2482
5117	537	3344	3342
5118	964	3352	3344
5119	540	3342	3352
5120	3344	3352	3342
5121	185	2064	2062
5122	969	4101	2064
5123	962	2062	4101
5124	2064	4101	2062
5125	255	2482	2483
5126	964	4104	2482
5127	969	2483	4104
5128	2482	4104	2483
5129	183	2050	2052
5130	962	4100	2050
5131	964	2052	4100
5132	2050	4100	2052
5133	969	4104	4101
5134	964	4100	4104
5135	962	4101	4100
5136	4104	4100	4101
5137	18	1159	1161
5138	359	2854	1159
5139	361	1161	2854
5140	1159	2854	1161
5141	151	1864	1862
5142	870	2855	1864
5143	359	1862	2855
5144	1864	2855	1862
5145	153	1872	1874

5146	361	2859	1872
5147	870	1874	2859
5148	1872	2859	1874
5149	359	2855	2854
5150	870	2859	2855
5151	361	2854	2859
5152	2855	2859	2854
5153	51	1329	1327
5154	529	3313	1329
5155	527	1327	3313
5156	1329	3313	1327
5157	251	2458	2456
5158	871	3318	2458
5159	529	2456	3318
5160	2458	3318	2456
5161	151	1863	1865
5162	527	3314	1863
5163	871	1865	3314
5164	1863	3314	1865
5165	529	3318	3313
5166	871	3314	3318
5167	527	3313	3314
5168	3318	3314	3313
5169	74	1458	1460
5170	658	3698	1458
5171	660	1460	3698
5172	1458	3698	1460
5173	153	1876	1873
5174	875	3700	1876
5175	658	1873	3700
5176	1876	3700	1873
5177	251	2457	2459
5178	660	3707	2457
5179	875	2459	3707
5180	2457	3707	2459
5181	658	3700	3698
5182	875	3707	3700
5183	660	3698	3707
5184	3700	3707	3698
5185	151	1865	1864
5186	871	4007	1865
5187	870	1864	4007
5188	1865	4007	1864
5189	251	2459	2458
5190	875	4009	2459
5191	871	2458	4009
5192	2459	4009	2458
5193	153	1874	1876
5194	870	4008	1874
5195	875	1876	4008
5196	1874	4008	1876
5197	871	4009	4007
5198	875	4008	4009
5199	870	4007	4008
5200	4009	4008	4007
5201	7	1109	1112
5202	309	2730	1109
5203	312	1112	2730
5204	1109	2730	1112

5205	101	1598	1596
5206	746	2732	1598
5207	309	1596	2732
5208	1598	2732	1596
5209	104	1614	1616
5210	312	2740	1614
5211	746	1616	2740
5212	1614	2740	1616
5213	309	2732	2730
5214	746	2740	2732
5215	312	2730	2740
5216	2732	2740	2730
5217	45	1294	1292
5218	494	3212	1294
5219	492	1292	3212
5220	1294	3212	1292
5221	234	2356	2354
5222	748	3221	2356
5223	494	2354	3221
5224	2356	3221	2354
5225	101	1597	1600
5226	492	3214	1597
5227	748	1600	3214
5228	1597	3214	1600
5229	494	3221	3212
5230	748	3214	3221
5231	492	3212	3214
5232	3221	3214	3212
5233	48	1310	1312
5234	510	3266	1310
5235	512	1312	3266
5236	1310	3266	1312
5237	104	1618	1615
5238	756	3268	1618
5239	510	1615	3268
5240	1618	3268	1615
5241	234	2355	2357
5242	512	3275	2355
5243	756	2357	3275
5244	2355	3275	2357
5245	510	3268	3266
5246	756	3275	3268
5247	512	3266	3275
5248	3268	3275	3266
5249	101	1600	1598
5250	748	3884	1600
5251	746	1598	3884
5252	1600	3884	1598
5253	234	2357	2356
5254	756	3888	2357
5255	748	2356	3888
5256	2357	3888	2356
5257	104	1616	1618
5258	746	3885	1616
5259	756	1618	3885
5260	1616	3885	1618
5261	748	3888	3884
5262	756	3885	3888
5263	746	3884	3885

5264	3888	3885	3884
5265	17	1156	1153
5266	356	2838	1156
5267	353	1153	2838
5268	1156	2838	1153
5269	148	1848	1846
5270	854	2848	1848
5271	356	1846	2848
5272	1848	2848	1846
5273	145	1828	1830
5274	353	2840	1828
5275	854	1830	2840
5276	1828	2840	1830
5277	356	2848	2838
5278	854	2840	2848
5279	353	2838	2840
5280	2848	2840	2838
5281	72	1448	1446
5282	648	3662	1448
5283	646	1446	3662
5284	1448	3662	1446
5285	236	2369	2367
5286	864	3671	2369
5287	648	2367	3671
5288	2369	3671	2367
5289	148	1847	1850
5290	646	3664	1847
5291	864	1850	3664
5292	1847	3664	1850
5293	648	3671	3662
5294	864	3664	3671
5295	646	3662	3664
5296	3671	3664	3662
5297	45	1293	1296
5298	493	3216	1293
5299	496	1296	3216
5300	1293	3216	1296
5301	145	1832	1829
5302	856	3218	1832
5303	493	1829	3218
5304	1832	3218	1829
5305	236	2366	2368
5306	496	3226	2366
5307	856	2368	3226
5308	2366	3226	2368
5309	493	3218	3216
5310	856	3226	3218
5311	496	3216	3226
5312	3218	3226	3216
5313	148	1850	1848
5314	864	3993	1850
5315	854	1848	3993
5316	1850	3993	1848
5317	236	2368	2369
5318	856	3996	2368
5319	864	2369	3996
5320	2368	3996	2369
5321	145	1830	1832
5322	854	3992	1830

5323	856	1832	3992
5324	1830	3992	1832
5325	864	3996	3993
5326	856	3992	3996
5327	854	3993	3992
5328	3996	3992	3993
5329	24	1190	1192
5330	390	2942	1190
5331	392	1192	2942
5332	1190	2942	1192
5333	182	2044	2042
5334	958	2944	2044
5335	390	2042	2944
5336	2044	2944	2042
5337	184	2054	2056
5338	392	2951	2054
5339	958	2056	2951
5340	2054	2951	2056
5341	390	2944	2942
5342	958	2951	2944
5343	392	2942	2951
5344	2944	2951	2942
5345	48	1314	1311
5346	514	3270	1314
5347	511	1311	3270
5348	1314	3270	1311
5349	244	2416	2414
5350	960	3280	2416
5351	514	2414	3280
5352	2416	3280	2414
5353	182	2043	2046
5354	511	3272	2043
5355	960	2046	3272
5356	2043	3272	2046
5357	514	3280	3270
5358	960	3272	3280
5359	511	3270	3272
5360	3280	3272	3270
5361	72	1447	1449
5362	647	3666	1447
5363	649	1449	3666
5364	1447	3666	1449
5365	184	2058	2055
5366	967	3668	2058
5367	647	2055	3668
5368	2058	3668	2055
5369	244	2415	2417
5370	649	3673	2415
5371	967	2417	3673
5372	2415	3673	2417
5373	647	3668	3666
5374	967	3673	3668
5375	649	3666	3673
5376	3668	3673	3666
5377	182	2046	2044
5378	960	4094	2046
5379	958	2044	4094
5380	2046	4094	2044
5381	244	2417	2416

5382	967	4098	2417
5383	960	2416	4098
5384	2417	4098	2416
5385	184	2056	2058
5386	958	4095	2056
5387	967	2058	4095
5388	2056	4095	2058
5389	960	4098	4094
5390	967	4095	4098
5391	958	4094	4095
5392	4098	4095	4094
5393	45	1296	1294
5394	496	3220	1296
5395	494	1294	3220
5396	1296	3220	1294
5397	236	2370	2366
5398	1036	3227	2370
5399	496	2366	3227
5400	2370	3227	2366
5401	234	2354	2358
5402	494	3222	2354
5403	1036	2358	3222
5404	2354	3222	2358
5405	496	3227	3220
5406	1036	3222	3227
5407	494	3220	3222
5408	3227	3222	3220
5409	72	1449	1448
5410	649	3670	1449
5411	648	1448	3670
5412	1449	3670	1448
5413	244	2419	2415
5414	1040	3674	2419
5415	649	2415	3674
5416	2419	3674	2415
5417	236	2367	2371
5418	648	3672	2367
5419	1040	2371	3672
5420	2367	3672	2371
5421	649	3674	3670
5422	1040	3672	3674
5423	648	3670	3672
5424	3674	3672	3670
5425	48	1312	1314
5426	512	3274	1312
5427	514	1314	3274
5428	1312	3274	1314
5429	234	2359	2355
5430	1037	3276	2359
5431	512	2355	3276
5432	2359	3276	2355
5433	244	2414	2418
5434	514	3281	2414
5435	1037	2418	3281
5436	2414	3281	2418
5437	512	3276	3274
5438	1037	3281	3276
5439	514	3274	3281
5440	3276	3281	3274

5441	236	2371	2370
5442	1040	4173	2371
5443	1036	2370	4173
5444	2371	4173	2370
5445	244	2418	2419
5446	1037	4174	2418
5447	1040	2419	4174
5448	2418	4174	2419
5449	234	2358	2359
5450	1036	4172	2358
5451	1037	2359	4172
5452	2358	4172	2359
5453	1040	4174	4173
5454	1037	4172	4174
5455	1036	4173	4172
5456	4174	4172	4173
5457	18	1161	1160
5458	361	2856	1161
5459	360	1160	2856
5460	1161	2856	1160
5461	153	1875	1872
5462	872	2860	1875
5463	361	1872	2860
5464	1875	2860	1872
5465	152	1866	1869
5466	360	2858	1866
5467	872	1869	2858
5468	1866	2858	1869
5469	361	2860	2856
5470	872	2858	2860
5471	360	2856	2858
5472	2860	2858	2856
5473	74	1462	1458
5474	662	3699	1462
5475	658	1458	3699
5476	1462	3699	1458
5477	279	2627	2625
5478	876	3712	2627
5479	662	2625	3712
5480	2627	3712	2625
5481	153	1873	1877
5482	658	3701	1873
5483	876	1877	3701
5484	1873	3701	1877
5485	662	3712	3699
5486	876	3701	3712
5487	658	3699	3701
5488	3712	3701	3699
5489	70	1435	1439
5490	635	3631	1435
5491	639	1439	3631
5492	1435	3631	1439
5493	152	1871	1867
5494	874	3633	1871
5495	635	1867	3633
5496	1871	3633	1867
5497	279	2624	2626
5498	639	3642	2624
5499	874	2626	3642

5500	2624	3642	2626
5501	635	3633	3631
5502	874	3642	3633
5503	639	3631	3642
5504	3633	3642	3631
5505	153	1877	1875
5506	876	4011	1877
5507	872	1875	4011
5508	1877	4011	1875
5509	279	2626	2627
5510	874	4012	2626
5511	876	2627	4012
5512	2626	4012	2627
5513	152	1869	1871
5514	872	4010	1869
5515	874	1871	4010
5516	1869	4010	1871
5517	876	4012	4011
5518	874	4010	4012
5519	872	4011	4010
5520	4012	4010	4011
5521	24	1192	1193
5522	392	2950	1192
5523	393	1193	2950
5524	1192	2950	1193
5525	184	2057	2054
5526	966	2952	2057
5527	392	2054	2952
5528	2057	2952	2054
5529	185	2060	2063
5530	393	2954	2060
5531	966	2063	2954
5532	2060	2954	2063
5533	392	2952	2950
5534	966	2954	2952
5535	393	2950	2954
5536	2952	2954	2950
5537	72	1451	1447
5538	651	3667	1451
5539	647	1447	3667
5540	1451	3667	1447
5541	282	2644	2642
5542	968	3678	2644
5543	651	2642	3678
5544	2644	3678	2642
5545	184	2055	2059
5546	647	3669	2055
5547	968	2059	3669
5548	2055	3669	2059
5549	651	3678	3667
5550	968	3669	3678
5551	647	3667	3669
5552	3678	3669	3667
5553	74	1459	1463
5554	659	3703	1459
5555	663	1463	3703
5556	1459	3703	1463
5557	185	2065	2061
5558	970	3705	2065

5559	659	2061	3705
5560	2065	3705	2061
5561	282	2643	2645
5562	663	3714	2643
5563	970	2645	3714
5564	2643	3714	2645
5565	659	3705	3703
5566	970	3714	3705
5567	663	3703	3714
5568	3705	3714	3703
5569	184	2059	2057
5570	968	4106	2059
5571	966	2057	4106
5572	2059	4106	2057
5573	282	2645	2644
5574	970	4108	2645
5575	968	2644	4108
5576	2645	4108	2644
5577	185	2063	2065
5578	966	4107	2063
5579	970	2065	4107
5580	2063	4107	2065
5581	968	4108	4106
5582	970	4107	4108
5583	966	4106	4107
5584	4108	4107	4106
5585	17	1154	1156
5586	354	2842	1154
5587	356	1156	2842
5588	1154	2842	1156
5589	146	1837	1834
5590	858	2844	1837
5591	354	1834	2844
5592	1837	2844	1834
5593	148	1846	1849
5594	356	2849	1846
5595	858	1849	2849
5596	1846	2849	1849
5597	354	2844	2842
5598	858	2849	2844
5599	356	2842	2849
5600	2844	2849	2842
5601	70	1438	1434
5602	638	3627	1438
5603	634	1434	3627
5604	1438	3627	1434
5605	278	2620	2618
5606	860	3640	2620
5607	638	2618	3640
5608	2620	3640	2618
5609	146	1835	1839
5610	634	3629	1835
5611	860	1839	3629
5612	1835	3629	1839
5613	638	3640	3627
5614	860	3629	3640
5615	634	3627	3629
5616	3640	3629	3627
5617	72	1446	1450

5618	646	3663	1446
5619	650	1450	3663
5620	1446	3663	1450
5621	148	1851	1847
5622	865	3665	1851
5623	646	1847	3665
5624	1851	3665	1847
5625	278	2619	2621
5626	650	3676	2619
5627	865	2621	3676
5628	2619	3676	2621
5629	646	3665	3663
5630	865	3676	3665
5631	650	3663	3676
5632	3665	3676	3663
5633	146	1839	1837
5634	860	3998	1839
5635	858	1837	3998
5636	1839	3998	1837
5637	278	2621	2620
5638	865	4000	2621
5639	860	2620	4000
5640	2621	4000	2620
5641	148	1849	1851
5642	858	3999	1849
5643	865	1851	3999
5644	1849	3999	1851
5645	860	4000	3998
5646	865	3999	4000
5647	858	3998	3999
5648	4000	3999	3998
5649	74	1463	1462
5650	663	3711	1463
5651	662	1462	3711
5652	1463	3711	1462
5653	282	2647	2643
5654	1080	3715	2647
5655	663	2643	3715
5656	2647	3715	2643
5657	279	2625	2629
5658	662	3713	2625
5659	1080	2629	3713
5660	2625	3713	2629
5661	663	3715	3711
5662	1080	3713	3715
5663	662	3711	3713
5664	3715	3713	3711
5665	72	1450	1451
5666	650	3675	1450
5667	651	1451	3675
5668	1450	3675	1451
5669	278	2623	2619
5670	1079	3677	2623
5671	650	2619	3677
5672	2623	3677	2619
5673	282	2642	2646
5674	651	3679	2642
5675	1079	2646	3679
5676	2642	3679	2646

5677	650	3677	3675
5678	1079	3679	3677
5679	651	3675	3679
5680	3677	3679	3675
5681	70	1439	1438
5682	639	3639	1439
5683	638	1438	3639
5684	1439	3639	1438
5685	279	2628	2624
5686	1078	3643	2628
5687	639	2624	3643
5688	2628	3643	2624
5689	278	2618	2622
5690	638	3641	2618
5691	1078	2622	3641
5692	2618	3641	2622
5693	639	3643	3639
5694	1078	3641	3643
5695	638	3639	3641
5696	3643	3641	3639
5697	282	2646	2647
5698	1079	4216	2646
5699	1080	2647	4216
5700	2646	4216	2647
5701	278	2622	2623
5702	1078	4214	2622
5703	1079	2623	4214
5704	2622	4214	2623
5705	279	2629	2628
5706	1080	4215	2629
5707	1078	2628	4215
5708	2629	4215	2628
5709	1079	4214	4216
5710	1078	4215	4214
5711	1080	4216	4215
5712	4214	4215	4216
5713	2	1093	1094
5714	293	2696	1093
5715	294	1094	2696
5716	1093	2696	1094
5717	85	1522	1520
5718	712	2697	1522
5719	293	1520	2697
5720	1522	2697	1520
5721	86	1524	1526
5722	294	2698	1524
5723	712	1526	2698
5724	1524	2698	1526
5725	293	2697	2696
5726	712	2698	2697
5727	294	2696	2698
5728	2697	2698	2696
5729	29	1218	1216
5730	418	3014	1218
5731	416	1216	3014
5732	1218	3014	1216
5733	200	2152	2150
5734	713	3019	2152
5735	418	2150	3019

5736	2152	3019	2150
5737	85	1521	1523
5738	416	3015	1521
5739	713	1523	3015
5740	1521	3015	1523
5741	418	3019	3014
5742	713	3015	3019
5743	416	3014	3015
5744	3019	3015	3014
5745	30	1220	1222
5746	420	3023	1220
5747	422	1222	3023
5748	1220	3023	1222
5749	86	1527	1525
5750	714	3024	1527
5751	420	1525	3024
5752	1527	3024	1525
5753	200	2151	2153
5754	422	3028	2151
5755	714	2153	3028
5756	2151	3028	2153
5757	420	3024	3023
5758	714	3028	3024
5759	422	3023	3028
5760	3024	3028	3023
5761	85	1523	1522
5762	713	3848	1523
5763	712	1522	3848
5764	1523	3848	1522
5765	200	2153	2152
5766	714	3850	2153
5767	713	2152	3850
5768	2153	3850	2152
5769	86	1526	1527
5770	712	3849	1526
5771	714	1527	3849
5772	1526	3849	1527
5773	713	3850	3848
5774	714	3849	3850
5775	712	3848	3849
5776	3850	3849	3848
5777	13	1138	1136
5778	338	2798	1138
5779	336	1136	2798
5780	1138	2798	1136
5781	130	1752	1750
5782	814	2803	1752
5783	338	1750	2803
5784	1752	2803	1750
5785	128	1742	1744
5786	336	2799	1742
5787	814	1744	2799
5788	1742	2799	1744
5789	338	2803	2798
5790	814	2799	2803
5791	336	2798	2799
5792	2803	2799	2798
5793	64	1400	1398
5794	600	3518	1400

5795	598	1398	3518
5796	1400	3518	1398
5797	201	2159	2157
5798	819	3527	2159
5799	600	2157	3527
5800	2159	3527	2157
5801	130	1751	1754
5802	598	3520	1751
5803	819	1754	3520
5804	1751	3520	1754
5805	600	3527	3518
5806	819	3520	3527
5807	598	3518	3520
5808	3527	3520	3518
5809	29	1217	1219
5810	417	3016	1217
5811	419	1219	3016
5812	1217	3016	1219
5813	128	1745	1743
5814	815	3017	1745
5815	417	1743	3017
5816	1745	3017	1743
5817	201	2156	2158
5818	419	3021	2156
5819	815	2158	3021
5820	2156	3021	2158
5821	417	3017	3016
5822	815	3021	3017
5823	419	3016	3021
5824	3017	3021	3016
5825	130	1754	1752
5826	819	3951	1754
5827	814	1752	3951
5828	1754	3951	1752
5829	201	2158	2159
5830	815	3952	2158
5831	819	2159	3952
5832	2158	3952	2159
5833	128	1744	1745
5834	814	3950	1744
5835	815	1745	3950
5836	1744	3950	1745
5837	819	3952	3951
5838	815	3950	3952
5839	814	3951	3950
5840	3952	3950	3951
5841	14	1140	1142
5842	340	2807	1140
5843	342	1142	2807
5844	1140	2807	1142
5845	132	1764	1762
5846	823	2808	1764
5847	340	1762	2808
5848	1764	2808	1762
5849	134	1770	1772
5850	342	2812	1770
5851	823	1772	2812
5852	1770	2812	1772
5853	340	2808	2807

5854	823	2812	2808
5855	342	2807	2812
5856	2808	2812	2807
5857	30	1223	1221
5858	423	3025	1223
5859	421	1221	3025
5860	1223	3025	1221
5861	202	2164	2162
5862	824	3030	2164
5863	423	2162	3030
5864	2164	3030	2162
5865	132	1763	1765
5866	421	3026	1763
5867	824	1765	3026
5868	1763	3026	1765
5869	423	3030	3025
5870	824	3026	3030
5871	421	3025	3026
5872	3030	3026	3025
5873	64	1399	1401
5874	599	3522	1399
5875	601	1401	3522
5876	1399	3522	1401
5877	134	1774	1771
5878	828	3524	1774
5879	599	1771	3524
5880	1774	3524	1771
5881	202	2163	2165
5882	601	3529	2163
5883	828	2165	3529
5884	2163	3529	2165
5885	599	3524	3522
5886	828	3529	3524
5887	601	3522	3529
5888	3524	3529	3522
5889	132	1765	1764
5890	824	3959	1765
5891	823	1764	3959
5892	1765	3959	1764
5893	202	2165	2164
5894	828	3961	2165
5895	824	2164	3961
5896	2165	3961	2164
5897	134	1772	1774
5898	823	3960	1772
5899	828	1774	3960
5900	1772	3960	1774
5901	824	3961	3959
5902	828	3960	3961
5903	823	3959	3960
5904	3961	3960	3959
5905	29	1219	1218
5906	419	3018	1219
5907	418	1218	3018
5908	1219	3018	1218
5909	201	2160	2156
5910	1000	3022	2160
5911	419	2156	3022
5912	2160	3022	2156

5913	200	2150	2154
5914	418	3020	2150
5915	1000	2154	3020
5916	2150	3020	2154
5917	419	3022	3018
5918	1000	3020	3022
5919	418	3018	3020
5920	3022	3020	3018
5921	64	1401	1400
5922	601	3526	1401
5923	600	1400	3526
5924	1401	3526	1400
5925	202	2167	2163
5926	1002	3530	2167
5927	601	2163	3530
5928	2167	3530	2163
5929	201	2157	2161
5930	600	3528	2157
5931	1002	2161	3528
5932	2157	3528	2161
5933	601	3530	3526
5934	1002	3528	3530
5935	600	3526	3528
5936	3530	3528	3526
5937	30	1222	1223
5938	422	3027	1222
5939	423	1223	3027
5940	1222	3027	1223
5941	200	2155	2151
5942	1001	3029	2155
5943	422	2151	3029
5944	2155	3029	2151
5945	202	2162	2166
5946	423	3031	2162
5947	1001	2166	3031
5948	2162	3031	2166
5949	422	3029	3027
5950	1001	3031	3029
5951	423	3027	3031
5952	3029	3031	3027
5953	201	2161	2160
5954	1002	4137	2161
5955	1000	2160	4137
5956	2161	4137	2160
5957	202	2166	2167
5958	1001	4138	2166
5959	1002	2167	4138
5960	2166	4138	2167
5961	200	2154	2155
5962	1000	4136	2154
5963	1001	2155	4136
5964	2154	4136	2155
5965	1002	4138	4137
5966	1001	4136	4138
5967	1000	4137	4136
5968	4138	4136	4137
5969	5	1103	1101
5970	303	2710	1103
5971	301	1101	2710

5972	1103	2710	1101
5973	95	1566	1564
5974	726	2715	1566
5975	303	1564	2715
5976	1566	2715	1564
5977	93	1554	1556
5978	301	2711	1554
5979	726	1556	2711
5980	1554	2711	1556
5981	303	2715	2710
5982	726	2711	2715
5983	301	2710	2711
5984	2715	2711	2710
5985	39	1262	1260
5986	462	3122	1262
5987	460	1260	3122
5988	1262	3122	1260
5989	214	2237	2235
5990	731	3131	2237
5991	462	2235	3131
5992	2237	3131	2235
5993	95	1565	1568
5994	460	3124	1565
5995	731	1568	3124
5996	1565	3124	1568
5997	462	3131	3122
5998	731	3124	3131
5999	460	3122	3124
6000	3131	3124	3122
6001	37	1250	1252
6002	450	3095	1250
6003	452	1252	3095
6004	1250	3095	1252
6005	93	1557	1555
6006	727	3096	1557
6007	450	1555	3096
6008	1557	3096	1555
6009	214	2234	2236
6010	452	3100	2234
6011	727	2236	3100
6012	2234	3100	2236
6013	450	3096	3095
6014	727	3100	3096
6015	452	3095	3100
6016	3096	3100	3095
6017	95	1568	1566
6018	731	3864	1568
6019	726	1566	3864
6020	1568	3864	1566
6021	214	2236	2237
6022	727	3865	2236
6023	731	2237	3865
6024	2236	3865	2237
6025	93	1556	1557
6026	726	3863	1556
6027	727	1557	3863
6028	1556	3863	1557
6029	731	3865	3864
6030	727	3863	3865

6031	726	3864	3863
6032	3865	3863	3864
6033	21	1174	1172
6034	374	2888	1174
6035	372	1172	2888
6036	1174	2888	1172
6037	166	1948	1946
6038	904	2897	1948
6039	374	1946	2897
6040	1948	2897	1946
6041	164	1934	1936
6042	372	2890	1934
6043	904	1936	2890
6044	1934	2890	1936
6045	374	2897	2888
6046	904	2890	2897
6047	372	2888	2890
6048	2897	2890	2888
6049	65	1407	1405
6050	607	3540	1407
6051	605	1405	3540
6052	1407	3540	1405
6053	219	2267	2265
6054	913	3547	2267
6055	607	2265	3547
6056	2267	3547	2265
6057	166	1947	1950
6058	605	3542	1947
6059	913	1950	3542
6060	1947	3542	1950
6061	607	3547	3540
6062	913	3542	3547
6063	605	3540	3542
6064	3547	3542	3540
6065	39	1261	1264
6066	461	3126	1261
6067	464	1264	3126
6068	1261	3126	1264
6069	164	1938	1935
6070	906	3128	1938
6071	461	1935	3128
6072	1938	3128	1935
6073	219	2264	2266
6074	464	3136	2264
6075	906	2266	3136
6076	2264	3136	2266
6077	461	3128	3126
6078	906	3136	3128
6079	464	3126	3136
6080	3128	3136	3126
6081	166	1950	1948
6082	913	4041	1950
6083	904	1948	4041
6084	1950	4041	1948
6085	219	2266	2267
6086	906	4044	2266
6087	913	2267	4044
6088	2266	4044	2267
6089	164	1936	1938

6090	904	4040	1936
6091	906	1938	4040
6092	1936	4040	1938
6093	913	4044	4041
6094	906	4040	4044
6095	904	4041	4040
6096	4044	4040	4041
6097	13	1137	1139
6098	337	2800	1137
6099	339	1139	2800
6100	1137	2800	1139
6101	129	1748	1746
6102	816	2801	1748
6103	337	1746	2801
6104	1748	2801	1746
6105	131	1756	1758
6106	339	2805	1756
6107	816	1758	2805
6108	1756	2805	1758
6109	337	2801	2800
6110	816	2805	2801
6111	339	2800	2805
6112	2801	2805	2800
6113	37	1253	1251
6114	453	3097	1253
6115	451	1251	3097
6116	1253	3097	1251
6117	215	2242	2240
6118	817	3102	2242
6119	453	2240	3102
6120	2242	3102	2240
6121	129	1747	1749
6122	451	3098	1747
6123	817	1749	3098
6124	1747	3098	1749
6125	453	3102	3097
6126	817	3098	3102
6127	451	3097	3098
6128	3102	3098	3097
6129	65	1404	1406
6130	604	3536	1404
6131	606	1406	3536
6132	1404	3536	1406
6133	131	1760	1757
6134	821	3538	1760
6135	604	1757	3538
6136	1760	3538	1757
6137	215	2241	2243
6138	606	3545	2241
6139	821	2243	3545
6140	2241	3545	2243
6141	604	3538	3536
6142	821	3545	3538
6143	606	3536	3545
6144	3538	3545	3536
6145	129	1749	1748
6146	817	3953	1749
6147	816	1748	3953
6148	1749	3953	1748

6149	215	2243	2242
6150	821	3955	2243
6151	817	2242	3955
6152	2243	3955	2242
6153	131	1758	1760
6154	816	3954	1758
6155	821	1760	3954
6156	1758	3954	1760
6157	817	3955	3953
6158	821	3954	3955
6159	816	3953	3954
6160	3955	3954	3953
6161	39	1264	1262
6162	464	3130	1264
6163	462	1262	3130
6164	1264	3130	1262
6165	219	2268	2264
6166	1016	3137	2268
6167	464	2264	3137
6168	2268	3137	2264
6169	214	2235	2239
6170	462	3132	2235
6171	1016	2239	3132
6172	2235	3132	2239
6173	464	3137	3130
6174	1016	3132	3137
6175	462	3130	3132
6176	3137	3132	3130
6177	65	1406	1407
6178	606	3544	1406
6179	607	1407	3544
6180	1406	3544	1407
6181	215	2245	2241
6182	1017	3546	2245
6183	606	2241	3546
6184	2245	3546	2241
6185	219	2265	2269
6186	607	3548	2265
6187	1017	2269	3548
6188	2265	3548	2269
6189	606	3546	3544
6190	1017	3548	3546
6191	607	3544	3548
6192	3546	3548	3544
6193	37	1252	1253
6194	452	3099	1252
6195	453	1253	3099
6196	1252	3099	1253
6197	214	2238	2234
6198	1015	3101	2238
6199	452	2234	3101
6200	2238	3101	2234
6201	215	2240	2244
6202	453	3103	2240
6203	1015	2244	3103
6204	2240	3103	2244
6205	452	3101	3099
6206	1015	3103	3101
6207	453	3099	3103

6208	3101	3103	3099
6209	219	2269	2268
6210	1017	4153	2269
6211	1016	2268	4153
6212	2269	4153	2268
6213	215	2244	2245
6214	1015	4152	2244
6215	1017	2245	4152
6216	2244	4152	2245
6217	214	2239	2238
6218	1016	4151	2239
6219	1015	2238	4151
6220	2239	4151	2238
6221	1017	4152	4153
6222	1015	4151	4152
6223	1016	4153	4151
6224	4152	4151	4153
6225	8	1114	1116
6226	314	2744	1114
6227	316	1116	2744
6228	1114	2744	1116
6229	106	1628	1626
6230	760	2745	1628
6231	314	1626	2745
6232	1628	2745	1626
6233	108	1634	1636
6234	316	2749	1634
6235	760	1636	2749
6236	1634	2749	1636
6237	314	2745	2744
6238	760	2749	2745
6239	316	2744	2749
6240	2745	2749	2744
6241	50	1324	1322
6242	524	3302	1324
6243	522	1322	3302
6244	1324	3302	1322
6245	248	2440	2438
6246	761	3307	2440
6247	524	2438	3307
6248	2440	3307	2438
6249	106	1627	1629
6250	522	3303	1627
6251	761	1629	3303
6252	1627	3303	1629
6253	524	3307	3302
6254	761	3303	3307
6255	522	3302	3303
6256	3307	3303	3302
6257	248	2439	2441
6258	532	3329	2439
6259	765	2441	3329
6260	2439	3329	2441
6261	530	3322	3320
6262	765	3329	3322
6263	532	3320	3329
6264	3322	3329	3320
6265	106	1629	1628
6266	761	3896	1629

6267	760	1628	3896
6268	1629	3896	1628
6269	248	2441	2440
6270	765	3898	2441
6271	761	2440	3898
6272	2441	3898	2440
6273	108	1636	1638
6274	760	3897	1636
6275	765	1638	3897
6276	1636	3897	1638
6277	761	3898	3896
6278	765	3897	3898
6279	760	3896	3897
6280	3898	3897	3896
6281	14	1143	1141
6282	343	2809	1143
6283	341	1141	2809
6284	1143	2809	1141
6285	135	1778	1776
6286	825	2814	1778
6287	343	1776	2814
6288	1778	2814	1776
6289	133	1766	1768
6290	341	2810	1766
6291	825	1768	2810
6292	1766	2810	1768
6293	343	2814	2809
6294	825	2810	2814
6295	341	2809	2810
6296	2814	2810	2809
6297	66	1412	1410
6298	612	3554	1412
6299	610	1410	3554
6300	1412	3554	1410
6301	249	2447	2445
6302	830	3563	2447
6303	612	2445	3563
6304	2447	3563	2445
6305	135	1777	1780
6306	610	3556	1777
6307	830	1780	3556
6308	1777	3556	1780
6309	612	3563	3554
6310	830	3556	3563
6311	610	3554	3556
6312	3563	3556	3554
6313	50	1323	1325
6314	523	3304	1323
6315	525	1325	3304
6316	1323	3304	1325
6317	133	1769	1767
6318	826	3305	1769
6319	523	1767	3305
6320	1769	3305	1767
6321	249	2444	2446
6322	525	3309	2444
6323	826	2446	3309
6324	2444	3309	2446
6325	523	3305	3304

6326	826	3309	3305
6327	525	3304	3309
6328	3305	3309	3304
6329	135	1780	1778
6330	830	3963	1780
6331	825	1778	3963
6332	1780	3963	1778
6333	249	2446	2447
6334	826	3964	2446
6335	830	2447	3964
6336	2446	3964	2447
6337	133	1768	1769
6338	825	3962	1768
6339	826	1769	3962
6340	1768	3962	1769
6341	830	3964	3963
6342	826	3962	3964
6343	825	3963	3962
6344	3964	3962	3963
6345	21	1173	1175
6346	373	2892	1173
6347	375	1175	2892
6348	1173	2892	1175
6349	165	1942	1940
6350	908	2894	1942
6351	373	1940	2894
6352	1942	2894	1940
6353	167	1952	1954
6354	375	2899	1952
6355	908	1954	2899
6356	1952	2899	1954
6357	373	2894	2892
6358	908	2899	2894
6359	375	2892	2899
6360	2894	2899	2892
6361	52	1334	1331
6362	534	3324	1334
6363	531	1331	3324
6364	1334	3324	1331
6365	253	2470	2468
6366	910	3334	2470
6367	534	2468	3334
6368	2470	3334	2468
6369	165	1941	1944
6370	531	3326	1941
6371	910	1944	3326
6372	1941	3326	1944
6373	534	3334	3324
6374	910	3326	3334
6375	531	3324	3326
6376	3334	3326	3324
6377	66	1411	1413
6378	611	3558	1411
6379	613	1413	3558
6380	1411	3558	1413
6381	167	1956	1953
6382	915	3560	1956
6383	611	1953	3560
6384	1956	3560	1953

6385	253	2469	2471
6386	613	3565	2469
6387	915	2471	3565
6388	2469	3565	2471
6389	611	3560	3558
6390	915	3565	3560
6391	613	3558	3565
6392	3560	3565	3558
6393	165	1944	1942
6394	910	4046	1944
6395	908	1942	4046
6396	1944	4046	1942
6397	253	2471	2470
6398	915	4050	2471
6399	910	2470	4050
6400	2471	4050	2470
6401	167	1954	1956
6402	908	4047	1954
6403	915	1956	4047
6404	1954	4047	1956
6405	910	4050	4046
6406	915	4047	4050
6407	908	4046	4047
6408	4050	4047	4046
6409	50	1325	1324
6410	525	3306	1325
6411	524	1324	3306
6412	1325	3306	1324
6413	249	2448	2444
6414	1048	3310	2448
6415	525	2444	3310
6416	2448	3310	2444
6417	248	2438	2442
6418	524	3308	2438
6419	1048	2442	3308
6420	2438	3308	2442
6421	525	3310	3306
6422	1048	3308	3310
6423	524	3306	3308
6424	3310	3308	3306
6425	66	1413	1412
6426	613	3562	1413
6427	612	1412	3562
6428	1413	3562	1412
6429	253	2473	2469
6430	1050	3566	2473
6431	613	2469	3566
6432	2473	3566	2469
6433	249	2445	2449
6434	612	3564	2445
6435	1050	2449	3564
6436	2445	3564	2449
6437	613	3566	3562
6438	1050	3564	3566
6439	612	3562	3564
6440	3566	3564	3562
6441	52	1332	1334
6442	532	3328	1332
6443	534	1334	3328

6444	1332	3328	1334
6445	248	2443	2439
6446	1049	3330	2443
6447	532	2439	3330
6448	2443	3330	2439
6449	253	2468	2472
6450	534	3335	2468
6451	1049	2472	3335
6452	2468	3335	2472
6453	532	3330	3328
6454	1049	3335	3330
6455	534	3328	3335
6456	3330	3335	3328
6457	249	2449	2448
6458	1050	4185	2449
6459	1048	2448	4185
6460	2449	4185	2448
6461	253	2472	2473
6462	1049	4186	2472
6463	1050	2473	4186
6464	2472	4186	2473
6465	248	2442	2443
6466	1048	4184	2442
6467	1049	2443	4184
6468	2442	4184	2443
6469	1050	4186	4185
6470	1049	4184	4186
6471	1048	4185	4184
6472	4186	4184	4185
6473	13	1139	1138
6474	339	2802	1139
6475	338	1138	2802
6476	1139	2802	1138
6477	131	1759	1756
6478	818	2806	1759
6479	339	1756	2806
6480	1759	2806	1756
6481	130	1750	1753
6482	338	2804	1750
6483	818	1753	2804
6484	1750	2804	1753
6485	339	2806	2802
6486	818	2804	2806
6487	338	2802	2804
6488	2806	2804	2802
6489	65	1408	1404
6490	608	3537	1408
6491	604	1404	3537
6492	1408	3537	1404
6493	272	2585	2583
6494	822	3550	2585
6495	608	2583	3550
6496	2585	3550	2583
6497	131	1757	1761
6498	604	3539	1757
6499	822	1761	3539
6500	1757	3539	1761
6501	608	3550	3537
6502	822	3539	3550

6503	604	3537	3539
6504	3550	3539	3537
6505	64	1398	1402
6506	598	3519	1398
6507	602	1402	3519
6508	1398	3519	1402
6509	130	1755	1751
6510	820	3521	1755
6511	598	1751	3521
6512	1755	3521	1751
6513	272	2582	2584
6514	602	3532	2582
6515	820	2584	3532
6516	2582	3532	2584
6517	598	3521	3519
6518	820	3532	3521
6519	602	3519	3532
6520	3521	3532	3519
6521	131	1761	1759
6522	822	3957	1761
6523	818	1759	3957
6524	1761	3957	1759
6525	272	2584	2585
6526	820	3958	2584
6527	822	2585	3958
6528	2584	3958	2585
6529	130	1753	1755
6530	818	3956	1753
6531	820	1755	3956
6532	1753	3956	1755
6533	822	3958	3957
6534	820	3956	3958
6535	818	3957	3956
6536	3958	3956	3957
6537	21	1175	1174
6538	375	2896	1175
6539	374	1174	2896
6540	1175	2896	1174
6541	167	1955	1952
6542	912	2900	1955
6543	375	1952	2900
6544	1955	2900	1952
6545	166	1946	1949
6546	374	2898	1946
6547	912	1949	2898
6548	1946	2898	1949
6549	375	2900	2896
6550	912	2898	2900
6551	374	2896	2898
6552	2900	2898	2896
6553	66	1415	1411
6554	615	3559	1415
6555	611	1411	3559
6556	1415	3559	1411
6557	274	2597	2595
6558	916	3570	2597
6559	615	2595	3570
6560	2597	3570	2595
6561	167	1953	1957

6562	611	3561	1953
6563	916	1957	3561
6564	1953	3561	1957
6565	615	3570	3559
6566	916	3561	3570
6567	611	3559	3561
6568	3570	3561	3559
6569	65	1405	1409
6570	605	3541	1405
6571	609	1409	3541
6572	1405	3541	1409
6573	166	1951	1947
6574	914	3543	1951
6575	605	1947	3543
6576	1951	3543	1947
6577	274	2594	2596
6578	609	3552	2594
6579	914	2596	3552
6580	2594	3552	2596
6581	605	3543	3541
6582	914	3552	3543
6583	609	3541	3552
6584	3543	3552	3541
6585	167	1957	1955
6586	916	4053	1957
6587	912	1955	4053
6588	1957	4053	1955
6589	274	2596	2597
6590	914	4054	2596
6591	916	2597	4054
6592	2596	4054	2597
6593	166	1949	1951
6594	912	4052	1949
6595	914	1951	4052
6596	1949	4052	1951
6597	916	4054	4053
6598	914	4052	4054
6599	912	4053	4052
6600	4054	4052	4053
6601	14	1142	1143
6602	342	2811	1142
6603	343	1143	2811
6604	1142	2811	1143
6605	134	1773	1770
6606	827	2813	1773
6607	342	1770	2813
6608	1773	2813	1770
6609	135	1776	1779
6610	343	2815	1776
6611	827	1779	2815
6612	1776	2815	1779
6613	342	2813	2811
6614	827	2815	2813
6615	343	2811	2815
6616	2813	2815	2811
6617	64	1403	1399
6618	603	3523	1403
6619	599	1399	3523
6620	1403	3523	1399

6621	273	2590	2588
6622	829	3534	2590
6623	603	2588	3534
6624	2590	3534	2588
6625	134	1771	1775
6626	599	3525	1771
6627	829	1775	3525
6628	1771	3525	1775
6629	603	3534	3523
6630	829	3525	3534
6631	599	3523	3525
6632	3534	3525	3523
6633	66	1410	1414
6634	610	3555	1410
6635	614	1414	3555
6636	1410	3555	1414
6637	135	1781	1777
6638	831	3557	1781
6639	610	1777	3557
6640	1781	3557	1777
6641	273	2589	2591
6642	614	3568	2589
6643	831	2591	3568
6644	2589	3568	2591
6645	610	3557	3555
6646	831	3568	3557
6647	614	3555	3568
6648	3557	3568	3555
6649	134	1775	1773
6650	829	3965	1775
6651	827	1773	3965
6652	1775	3965	1773
6653	273	2591	2590
6654	831	3967	2591
6655	829	2590	3967
6656	2591	3967	2590
6657	135	1779	1781
6658	827	3966	1779
6659	831	1781	3966
6660	1779	3966	1781
6661	829	3967	3965
6662	831	3966	3967
6663	827	3965	3966
6664	3967	3966	3965
6665	65	1409	1408
6666	609	3549	1409
6667	608	1408	3549
6668	1409	3549	1408
6669	274	2598	2594
6670	1073	3553	2598
6671	609	2594	3553
6672	2598	3553	2594
6673	272	2583	2587
6674	608	3551	2583
6675	1073	2587	3551
6676	2583	3551	2587
6677	609	3553	3549
6678	1073	3551	3553
6679	608	3549	3551

6680	3553	3551	3549
6681	66	1414	1415
6682	614	3567	1414
6683	615	1415	3567
6684	1414	3567	1415
6685	273	2593	2589
6686	1074	3569	2593
6687	614	2589	3569
6688	2593	3569	2589
6689	274	2595	2599
6690	615	3571	2595
6691	1074	2599	3571
6692	2595	3571	2599
6693	614	3569	3567
6694	1074	3571	3569
6695	615	3567	3571
6696	3569	3571	3567
6697	64	1402	1403
6698	602	3531	1402
6699	603	1403	3531
6700	1402	3531	1403
6701	272	2586	2582
6702	1072	3533	2586
6703	602	2582	3533
6704	2586	3533	2582
6705	273	2588	2592
6706	603	3535	2588
6707	1072	2592	3535
6708	2588	3535	2592
6709	602	3533	3531
6710	1072	3535	3533
6711	603	3531	3535
6712	3533	3535	3531
6713	274	2599	2598
6714	1074	4210	2599
6715	1073	2598	4210
6716	2599	4210	2598
6717	273	2592	2593
6718	1072	4209	2592
6719	1074	2593	4209
6720	2592	4209	2593
6721	272	2587	2586
6722	1073	4208	2587
6723	1072	2586	4208
6724	2587	4208	2586
6725	1074	4209	4210
6726	1072	4208	4209
6727	1073	4210	4208
6728	4209	4208	4210
6729	7	1112	1110
6730	312	2734	1112
6731	310	1110	2734
6732	1112	2734	1110
6733	104	1617	1614
6734	750	2741	1617
6735	312	1614	2741
6736	1617	2741	1614
6737	102	1602	1605
6738	310	2736	1602

6739	750	1605	2736
6740	1602	2736	1605
6741	312	2741	2734
6742	750	2736	2741
6743	310	2734	2736
6744	2741	2736	2734
6745	48	1313	1310
6746	513	3267	1313
6747	510	1310	3267
6748	1313	3267	1310
6749	238	2381	2379
6750	757	3278	2381
6751	513	2379	3278
6752	2381	3278	2379
6753	104	1615	1619
6754	510	3269	1615
6755	757	1619	3269
6756	1615	3269	1619
6757	513	3278	3267
6758	757	3269	3278
6759	510	3267	3269
6760	3278	3269	3267
6761	46	1298	1301
6762	498	3231	1298
6763	501	1301	3231
6764	1298	3231	1301
6765	102	1607	1603
6766	752	3233	1607
6767	498	1603	3233
6768	1607	3233	1603
6769	238	2378	2380
6770	501	3242	2378
6771	752	2380	3242
6772	2378	3242	2380
6773	498	3233	3231
6774	752	3242	3233
6775	501	3231	3242
6776	3233	3242	3231
6777	104	1619	1617
6778	757	3891	1619
6779	750	1617	3891
6780	1619	3891	1617
6781	238	2380	2381
6782	752	3892	2380
6783	757	2381	3892
6784	2380	3892	2381
6785	102	1605	1607
6786	750	3890	1605
6787	752	1607	3890
6788	1605	3890	1607
6789	757	3892	3891
6790	752	3890	3892
6791	750	3891	3890
6792	3892	3890	3891
6793	24	1194	1190
6794	394	2943	1194
6795	390	1190	2943
6796	1194	2943	1190
6797	186	2068	2066

6798	959	2956	2068
6799	394	2066	2956
6800	2068	2956	2066
6801	182	2042	2045
6802	390	2945	2042
6803	959	2045	2945
6804	2042	2945	2045
6805	394	2956	2943
6806	959	2945	2956
6807	390	2943	2945
6808	2956	2945	2943
6809	77	1479	1477
6810	679	3756	1479
6811	677	1477	3756
6812	1479	3756	1477
6813	245	2423	2421
6814	972	3763	2423
6815	679	2421	3763
6816	2423	3763	2421
6817	186	2067	2070
6818	677	3758	2067
6819	972	2070	3758
6820	2067	3758	2070
6821	679	3763	3756
6822	972	3758	3763
6823	677	3756	3758
6824	3763	3758	3756
6825	48	1311	1315
6826	511	3271	1311
6827	515	1315	3271
6828	1311	3271	1315
6829	182	2047	2043
6830	961	3273	2047
6831	511	2043	3273
6832	2047	3273	2043
6833	245	2420	2422
6834	515	3282	2420
6835	961	2422	3282
6836	2420	3282	2422
6837	511	3273	3271
6838	961	3282	3273
6839	515	3271	3282
6840	3273	3282	3271
6841	186	2070	2068
6842	972	4097	2070
6843	959	2068	4097
6844	2070	4097	2068
6845	245	2422	2423
6846	961	4099	2422
6847	972	2423	4099
6848	2422	4099	2423
6849	182	2045	2047
6850	959	4096	2045
6851	961	2047	4096
6852	2045	4096	2047
6853	972	4099	4097
6854	961	4096	4099
6855	959	4097	4096
6856	4099	4096	4097

6857	20	1167	1171
6858	367	2875	1167
6859	371	1171	2875
6860	1167	2875	1171
6861	159	1907	1904
6862	891	2877	1907
6863	367	1904	2877
6864	1907	2877	1904
6865	163	1928	1930
6866	371	2886	1928
6867	891	1930	2886
6868	1928	2886	1930
6869	367	2877	2875
6870	891	2886	2877
6871	371	2875	2886
6872	2877	2886	2875
6873	46	1303	1299
6874	503	3235	1303
6875	499	1299	3235
6876	1303	3235	1299
6877	240	2392	2390
6878	893	3246	2392
6879	503	2390	3246
6880	2392	3246	2390
6881	159	1905	1909
6882	499	3237	1905
6883	893	1909	3237
6884	1905	3237	1909
6885	503	3246	3235
6886	893	3237	3246
6887	499	3235	3237
6888	3246	3237	3235
6889	77	1476	1478
6890	676	3752	1476
6891	678	1478	3752
6892	1476	3752	1478
6893	163	1932	1929
6894	902	3754	1932
6895	676	1929	3754
6896	1932	3754	1929
6897	240	2391	2393
6898	678	3761	2391
6899	902	2393	3761
6900	2391	3761	2393
6901	676	3754	3752
6902	902	3761	3754
6903	678	3752	3761
6904	3754	3761	3752
6905	159	1909	1907
6906	893	4030	1909
6907	891	1907	4030
6908	1909	4030	1907
6909	240	2393	2392
6910	902	4033	2393
6911	893	2392	4033
6912	2393	4033	2392
6913	163	1930	1932
6914	891	4031	1930
6915	902	1932	4031

6916	1930	4031	1932
6917	893	4033	4030
6918	902	4031	4033
6919	891	4030	4031
6920	4033	4031	4030
6921	48	1315	1313
6922	515	3277	1315
6923	513	1313	3277
6924	1315	3277	1313
6925	245	2424	2420
6926	1043	3283	2424
6927	515	2420	3283
6928	2424	3283	2420
6929	238	2379	2383
6930	513	3279	2379
6931	1043	2383	3279
6932	2379	3279	2383
6933	515	3283	3277
6934	1043	3279	3283
6935	513	3277	3279
6936	3283	3279	3277
6937	77	1478	1479
6938	678	3760	1478
6939	679	1479	3760
6940	1478	3760	1479
6941	240	2395	2391
6942	1044	3762	2395
6943	678	2391	3762
6944	2395	3762	2391
6945	245	2421	2425
6946	679	3764	2421
6947	1044	2425	3764
6948	2421	3764	2425
6949	678	3762	3760
6950	1044	3764	3762
6951	679	3760	3764
6952	3762	3764	3760
6953	46	1301	1303
6954	501	3241	1301
6955	503	1303	3241
6956	1301	3241	1303
6957	238	2382	2378
6958	1042	3243	2382
6959	501	2378	3243
6960	2382	3243	2378
6961	240	2390	2394
6962	503	3247	2390
6963	1042	2394	3247
6964	2390	3247	2394
6965	501	3243	3241
6966	1042	3247	3243
6967	503	3241	3247
6968	3243	3247	3241
6969	245	2425	2424
6970	1044	4180	2425
6971	1043	2424	4180
6972	2425	4180	2424
6973	240	2394	2395
6974	1042	4179	2394

6975	1044	2395	4179
6976	2394	4179	2395
6977	238	2383	2382
6978	1043	4178	2383
6979	1042	2382	4178
6980	2383	4178	2382
6981	1044	4179	4180
6982	1042	4178	4179
6983	1043	4180	4178
6984	4179	4178	4180
6985	8	1116	1117
6986	316	2748	1116
6987	317	1117	2748
6988	1116	2748	1117
6989	108	1637	1634
6990	764	2750	1637
6991	316	1634	2750
6992	1637	2750	1634
6993	109	1640	1643
6994	317	2752	1640
6995	764	1643	2752
6996	1640	2752	1643
6997	316	2750	2748
6998	764	2752	2750
6999	317	2748	2752
7000	2750	2752	2748
7001	252	2464	2462
7002	766	3332	2464
7003	533	2462	3332
7004	2464	3332	2462
7005	53	1336	1339
7006	536	3339	1336
7007	539	1339	3339
7008	1336	3339	1339
7009	109	1645	1641
7010	768	3341	1645
7011	536	1641	3341
7012	1645	3341	1641
7013	252	2463	2465
7014	539	3350	2463
7015	768	2465	3350
7016	2463	3350	2465
7017	536	3341	3339
7018	768	3350	3341
7019	539	3339	3350
7020	3341	3350	3339
7021	108	1639	1637
7022	766	3902	1639
7023	764	1637	3902
7024	1639	3902	1637
7025	252	2465	2464
7026	768	3904	2465
7027	766	2464	3904
7028	2465	3904	2464
7029	109	1643	1645
7030	764	3903	1643
7031	768	1645	3903
7032	1643	3903	1645
7033	766	3904	3902

7034	768	3903	3904
7035	764	3902	3903
7036	3904	3903	3902
7037	21	1177	1173
7038	377	2893	1177
7039	373	1173	2893
7040	1177	2893	1173
7041	169	1966	1964
7042	909	2904	1966
7043	377	1964	2904
7044	1966	2904	1964
7045	165	1940	1943
7046	373	2895	1940
7047	909	1943	2895
7048	1940	2895	1943
7049	377	2904	2893
7050	909	2895	2904
7051	373	2893	2895
7052	2904	2895	2893
7053	78	1484	1482
7054	684	3770	1484
7055	682	1482	3770
7056	1484	3770	1482
7057	254	2477	2475
7058	920	3779	2477
7059	684	2475	3779
7060	2477	3779	2475
7061	169	1965	1968
7062	682	3772	1965
7063	920	1968	3772
7064	1965	3772	1968
7065	684	3779	3770
7066	920	3772	3779
7067	682	3770	3772
7068	3779	3772	3770
7069	52	1331	1335
7070	531	3325	1331
7071	535	1335	3325
7072	1331	3325	1335
7073	165	1945	1941
7074	911	3327	1945
7075	531	1941	3327
7076	1945	3327	1941
7077	254	2474	2476
7078	535	3336	2474
7079	911	2476	3336
7080	2474	3336	2476
7081	531	3327	3325
7082	911	3336	3327
7083	535	3325	3336
7084	3327	3336	3325
7085	169	1968	1966
7086	920	4049	1968
7087	909	1966	4049
7088	1968	4049	1966
7089	254	2476	2477
7090	911	4051	2476
7091	920	2477	4051
7092	2476	4051	2477

7093	165	1943	1945
7094	909	4048	1943
7095	911	1945	4048
7096	1943	4048	1945
7097	920	4051	4049
7098	911	4048	4051
7099	909	4049	4048
7100	4051	4048	4049
7101	24	1191	1195
7102	391	2947	1191
7103	395	1195	2947
7104	1191	2947	1195
7105	183	2051	2048
7106	963	2949	2051
7107	391	2048	2949
7108	2051	2949	2048
7109	187	2072	2074
7110	395	2958	2072
7111	963	2074	2958
7112	2072	2958	2074
7113	391	2949	2947
7114	963	2958	2949
7115	395	2947	2958
7116	2949	2958	2947
7117	53	1341	1337
7118	541	3343	1341
7119	537	1337	3343
7120	1341	3343	1337
7121	256	2488	2486
7122	965	3354	2488
7123	541	2486	3354
7124	2488	3354	2486
7125	183	2049	2053
7126	537	3345	2049
7127	965	2053	3345
7128	2049	3345	2053
7129	541	3354	3343
7130	965	3345	3354
7131	537	3343	3345
7132	3354	3345	3343
7133	78	1483	1485
7134	683	3774	1483
7135	685	1485	3774
7136	1483	3774	1485
7137	187	2076	2073
7138	974	3776	2076
7139	683	2073	3776
7140	2076	3776	2073
7141	256	2487	2489
7142	685	3781	2487
7143	974	2489	3781
7144	2487	3781	2489
7145	683	3776	3774
7146	974	3781	3776
7147	685	3774	3781
7148	3776	3781	3774
7149	183	2053	2051
7150	965	4102	2053
7151	963	2051	4102

7152	2053	4102	2051
7153	256	2489	2488
7154	974	4105	2489
7155	965	2488	4105
7156	2489	4105	2488
7157	187	2074	2076
7158	963	4103	2074
7159	974	2076	4103
7160	2074	4103	2076
7161	965	4105	4102
7162	974	4103	4105
7163	963	4102	4103
7164	4105	4103	4102
7165	52	1335	1333
7166	535	3331	1335
7167	533	1333	3331
7168	1335	3331	1333
7169	254	2478	2474
7170	1054	3337	2478
7171	535	2474	3337
7172	2478	3337	2474
7173	252	2462	2466
7174	533	3333	2462
7175	1054	2466	3333
7176	2462	3333	2466
7177	535	3337	3331
7178	1054	3333	3337
7179	533	3331	3333
7180	3337	3333	3331
7181	78	1485	1484
7182	685	3778	1485
7183	684	1484	3778
7184	1485	3778	1484
7185	256	2491	2487
7186	1056	3782	2491
7187	685	2487	3782
7188	2491	3782	2487
7189	254	2475	2479
7190	684	3780	2475
7191	1056	2479	3780
7192	2475	3780	2479
7193	685	3782	3778
7194	1056	3780	3782
7195	684	3778	3780
7196	3782	3780	3778
7197	53	1339	1341
7198	539	3349	1339
7199	541	1341	3349
7200	1339	3349	1341
7201	252	2467	2463
7202	1055	3351	2467
7203	539	2463	3351
7204	2467	3351	2463
7205	256	2486	2490
7206	541	3355	2486
7207	1055	2490	3355
7208	2486	3355	2490
7209	539	3351	3349
7210	1055	3355	3351

7211	541	3349	3355
7212	3351	3355	3349
7213	254	2479	2478
7214	1056	4191	2479
7215	1054	2478	4191
7216	2479	4191	2478
7217	256	2490	2491
7218	1055	4192	2490
7219	1056	2491	4192
7220	2490	4192	2491
7221	252	2466	2467
7222	1054	4190	2466
7223	1055	2467	4190
7224	2466	4190	2467
7225	1056	4192	4191
7226	1055	4190	4192
7227	1054	4191	4190
7228	4192	4190	4191
7229	5	1102	1103
7230	302	2712	1102
7231	303	1103	2712
7232	1102	2712	1103
7233	94	1561	1558
7234	728	2714	1561
7235	302	1558	2714
7236	1561	2714	1558
7237	95	1564	1567
7238	303	2716	1564
7239	728	1567	2716
7240	1564	2716	1567
7241	302	2714	2712
7242	728	2716	2714
7243	303	2712	2716
7244	2714	2716	2712
7245	38	1257	1254
7246	457	3105	1257
7247	454	1254	3105
7248	1257	3105	1254
7249	216	2248	2246
7250	730	3116	2248
7251	457	2246	3116
7252	2248	3116	2246
7253	94	1559	1563
7254	454	3107	1559
7255	730	1563	3107
7256	1559	3107	1563
7257	457	3116	3105
7258	730	3107	3116
7259	454	3105	3107
7260	3116	3107	3105
7261	39	1260	1263
7262	460	3123	1260
7263	463	1263	3123
7264	1260	3123	1263
7265	95	1569	1565
7266	732	3125	1569
7267	460	1565	3125
7268	1569	3125	1565
7269	216	2247	2249

7270	463	3134	2247
7271	732	2249	3134
7272	2247	3134	2249
7273	460	3125	3123
7274	732	3134	3125
7275	463	3123	3134
7276	3125	3134	3123
7277	94	1563	1561
7278	730	3866	1563
7279	728	1561	3866
7280	1563	3866	1561
7281	216	2249	2248
7282	732	3868	2249
7283	730	2248	3868
7284	2249	3868	2248
7285	95	1567	1569
7286	728	3867	1567
7287	732	1569	3867
7288	1567	3867	1569
7289	730	3868	3866
7290	732	3867	3868
7291	728	3866	3867
7292	3868	3867	3866
7293	20	1170	1166
7294	370	2871	1170
7295	366	1166	2871
7296	1170	2871	1166
7297	162	1924	1922
7298	887	2884	1924
7299	370	1922	2884
7300	1924	2884	1922
7301	158	1898	1901
7302	366	2873	1898
7303	887	1901	2873
7304	1898	2873	1901
7305	370	2884	2871
7306	887	2873	2884
7307	366	2871	2873
7308	2884	2873	2871
7309	76	1472	1470
7310	672	3734	1472
7311	670	1470	3734
7312	1472	3734	1470
7313	218	2261	2259
7314	900	3743	2261
7315	672	2259	3743
7316	2261	3743	2259
7317	162	1923	1926
7318	670	3736	1923
7319	900	1926	3736
7320	1923	3736	1926
7321	672	3743	3734
7322	900	3736	3743
7323	670	3734	3736
7324	3743	3736	3734
7325	38	1255	1259
7326	455	3109	1255
7327	459	1259	3109
7328	1255	3109	1259

7329	158	1903	1899
7330	889	3111	1903
7331	455	1899	3111
7332	1903	3111	1899
7333	218	2258	2260
7334	459	3120	2258
7335	889	2260	3120
7336	2258	3120	2260
7337	455	3111	3109
7338	889	3120	3111
7339	459	3109	3120
7340	3111	3120	3109
7341	162	1926	1924
7342	900	4025	1926
7343	887	1924	4025
7344	1926	4025	1924
7345	218	2260	2261
7346	889	4027	2260
7347	900	2261	4027
7348	2260	4027	2261
7349	158	1901	1903
7350	887	4024	1901
7351	889	1903	4024
7352	1901	4024	1903
7353	900	4027	4025
7354	889	4024	4027
7355	887	4025	4024
7356	4027	4024	4025
7357	21	1172	1176
7358	372	2889	1172
7359	376	1176	2889
7360	1172	2889	1176
7361	164	1937	1934
7362	905	2891	1937
7363	372	1934	2891
7364	1937	2891	1934
7365	168	1958	1960
7366	376	2902	1958
7367	905	1960	2902
7368	1958	2902	1960
7369	372	2891	2889
7370	905	2902	2891
7371	376	2889	2902
7372	2891	2902	2889
7373	39	1265	1261
7374	465	3127	1265
7375	461	1261	3127
7376	1265	3127	1261
7377	220	2272	2270
7378	907	3138	2272
7379	465	2270	3138
7380	2272	3138	2270
7381	164	1935	1939
7382	461	3129	1935
7383	907	1939	3129
7384	1935	3129	1939
7385	465	3138	3127
7386	907	3129	3138
7387	461	3127	3129

7388	3138	3129	3127
7389	76	1471	1473
7390	671	3738	1471
7391	673	1473	3738
7392	1471	3738	1473
7393	168	1962	1959
7394	918	3740	1962
7395	671	1959	3740
7396	1962	3740	1959
7397	220	2271	2273
7398	673	3745	2271
7399	918	2273	3745
7400	2271	3745	2273
7401	671	3740	3738
7402	918	3745	3740
7403	673	3738	3745
7404	3740	3745	3738
7405	164	1939	1937
7406	907	4042	1939
7407	905	1937	4042
7408	1939	4042	1937
7409	220	2273	2272
7410	918	4045	2273
7411	907	2272	4045
7412	2273	4045	2272
7413	168	1960	1962
7414	905	4043	1960
7415	918	1962	4043
7416	1960	4043	1962
7417	907	4045	4042
7418	918	4043	4045
7419	905	4042	4043
7420	4045	4043	4042
7421	38	1259	1257
7422	459	3115	1259
7423	457	1257	3115
7424	1259	3115	1257
7425	218	2262	2258
7426	1018	3121	2262
7427	459	2258	3121
7428	2262	3121	2258
7429	216	2246	2250
7430	457	3117	2246
7431	1018	2250	3117
7432	2246	3117	2250
7433	459	3121	3115
7434	1018	3117	3121
7435	457	3115	3117
7436	3121	3117	3115
7437	76	1473	1472
7438	673	3742	1473
7439	672	1472	3742
7440	1473	3742	1472
7441	220	2275	2271
7442	1020	3746	2275
7443	673	2271	3746
7444	2275	3746	2271
7445	218	2259	2263
7446	672	3744	2259

7447	1020	2263	3744
7448	2259	3744	2263
7449	673	3746	3742
7450	1020	3744	3746
7451	672	3742	3744
7452	3746	3744	3742
7453	39	1263	1265
7454	463	3133	1263
7455	465	1265	3133
7456	1263	3133	1265
7457	216	2251	2247
7458	1019	3135	2251
7459	463	2247	3135
7460	2251	3135	2247
7461	220	2270	2274
7462	465	3139	2270
7463	1019	2274	3139
7464	2270	3139	2274
7465	463	3135	3133
7466	1019	3139	3135
7467	465	3133	3139
7468	3135	3139	3133
7469	218	2263	2262
7470	1020	4155	2263
7471	1018	2262	4155
7472	2263	4155	2262
7473	220	2274	2275
7474	1019	4156	2274
7475	1020	2275	4156
7476	2274	4156	2275
7477	216	2250	2251
7478	1018	4154	2250
7479	1019	2251	4154
7480	2250	4154	2251
7481	1020	4156	4155
7482	1019	4154	4156
7483	1018	4155	4154
7484	4156	4154	4155
7485	24	1195	1194
7486	395	2955	1195
7487	394	1194	2955
7488	1195	2955	1194
7489	187	2075	2072
7490	971	2959	2075
7491	395	2072	2959
7492	2075	2959	2072
7493	186	2066	2069
7494	394	2957	2066
7495	971	2069	2957
7496	2066	2957	2069
7497	395	2959	2955
7498	971	2957	2959
7499	394	2955	2957
7500	2959	2957	2955
7501	78	1487	1483
7502	687	3775	1487
7503	683	1483	3775
7504	1487	3775	1483
7505	286	2669	2667

7506	975	3786	2669
7507	687	2667	3786
7508	2669	3786	2667
7509	187	2073	2077
7510	683	3777	2073
7511	975	2077	3777
7512	2073	3777	2077
7513	687	3786	3775
7514	975	3777	3786
7515	683	3775	3777
7516	3786	3777	3775
7517	77	1477	1481
7518	677	3757	1477
7519	681	1481	3757
7520	1477	3757	1481
7521	186	2071	2067
7522	973	3759	2071
7523	677	2067	3759
7524	2071	3759	2067
7525	286	2666	2668
7526	681	3768	2666
7527	973	2668	3768
7528	2666	3768	2668
7529	677	3759	3757
7530	973	3768	3759
7531	681	3757	3768
7532	3759	3768	3757
7533	187	2077	2075
7534	975	4110	2077
7535	971	2075	4110
7536	2077	4110	2075
7537	286	2668	2669
7538	973	4111	2668
7539	975	2669	4111
7540	2668	4111	2669
7541	186	2069	2071
7542	971	4109	2069
7543	973	2071	4109
7544	2069	4109	2071
7545	975	4111	4110
7546	973	4109	4111
7547	971	4110	4109
7548	4111	4109	4110
7549	21	1176	1177
7550	376	2901	1176
7551	377	1177	2901
7552	1176	2901	1177
7553	168	1961	1958
7554	917	2903	1961
7555	376	1958	2903
7556	1961	2903	1958
7557	169	1964	1967
7558	377	2905	1964
7559	917	1967	2905
7560	1964	2905	1967
7561	376	2903	2901
7562	917	2905	2903
7563	377	2901	2905
7564	2903	2905	2901

7565	76	1475	1471
7566	675	3739	1475
7567	671	1471	3739
7568	1475	3739	1471
7569	285	2662	2660
7570	919	3750	2662
7571	675	2660	3750
7572	2662	3750	2660
7573	168	1959	1963
7574	671	3741	1959
7575	919	1963	3741
7576	1959	3741	1963
7577	675	3750	3739
7578	919	3741	3750
7579	671	3739	3741
7580	3750	3741	3739
7581	78	1482	1486
7582	682	3771	1482
7583	686	1486	3771
7584	1482	3771	1486
7585	169	1969	1965
7586	921	3773	1969
7587	682	1965	3773
7588	1969	3773	1965
7589	285	2661	2663
7590	686	3784	2661
7591	921	2663	3784
7592	2661	3784	2663
7593	682	3773	3771
7594	921	3784	3773
7595	686	3771	3784
7596	3773	3784	3771
7597	168	1963	1961
7598	919	4055	1963
7599	917	1961	4055
7600	1963	4055	1961
7601	285	2663	2662
7602	921	4057	2663
7603	919	2662	4057
7604	2663	4057	2662
7605	169	1967	1969
7606	917	4056	1967
7607	921	1969	4056
7608	1967	4056	1969
7609	919	4057	4055
7610	921	4056	4057
7611	917	4055	4056
7612	4057	4056	4055
7613	20	1171	1170
7614	371	2883	1171
7615	370	1170	2883
7616	1171	2883	1170
7617	163	1931	1928
7618	899	2887	1931
7619	371	1928	2887
7620	1931	2887	1928
7621	162	1922	1925
7622	370	2885	1922
7623	899	1925	2885

7624	1922	2885	1925
7625	371	2887	2883
7626	899	2885	2887
7627	370	2883	2885
7628	2887	2885	2883
7629	77	1480	1476
7630	680	3753	1480
7631	676	1476	3753
7632	1480	3753	1476
7633	284	2657	2655
7634	903	3766	2657
7635	680	2655	3766
7636	2657	3766	2655
7637	163	1929	1933
7638	676	3755	1929
7639	903	1933	3755
7640	1929	3755	1933
7641	680	3766	3753
7642	903	3755	3766
7643	676	3753	3755
7644	3766	3755	3753
7645	76	1470	1474
7646	670	3735	1470
7647	674	1474	3735
7648	1470	3735	1474
7649	162	1927	1923
7650	901	3737	1927
7651	670	1923	3737
7652	1927	3737	1923
7653	284	2654	2656
7654	674	3748	2654
7655	901	2656	3748
7656	2654	3748	2656
7657	670	3737	3735
7658	901	3748	3737
7659	674	3735	3748
7660	3737	3748	3735
7661	163	1933	1931
7662	903	4038	1933
7663	899	1931	4038
7664	1933	4038	1931
7665	284	2656	2657
7666	901	4039	2656
7667	903	2657	4039
7668	2656	4039	2657
7669	162	1925	1927
7670	899	4037	1925
7671	901	1927	4037
7672	1925	4037	1927
7673	903	4039	4038
7674	901	4037	4039
7675	899	4038	4037
7676	4039	4037	4038
7677	78	1486	1487
7678	686	3783	1486
7679	687	1487	3783
7680	1486	3783	1487
7681	285	2665	2661
7682	1086	3785	2665

7683	686	2661	3785
7684	2665	3785	2661
7685	286	2667	2671
7686	687	3787	2667
7687	1086	2671	3787
7688	2667	3787	2671
7689	686	3785	3783
7690	1086	3787	3785
7691	687	3783	3787
7692	3785	3787	3783
7693	76	1474	1475
7694	674	3747	1474
7695	675	1475	3747
7696	1474	3747	1475
7697	284	2658	2654
7698	1084	3749	2658
7699	674	2654	3749
7700	2658	3749	2654
7701	285	2660	2664
7702	675	3751	2660
7703	1084	2664	3751
7704	2660	3751	2664
7705	674	3749	3747
7706	1084	3751	3749
7707	675	3747	3751
7708	3749	3751	3747
7709	77	1481	1480
7710	681	3765	1481
7711	680	1480	3765
7712	1481	3765	1480
7713	286	2670	2666
7714	1085	3769	2670
7715	681	2666	3769
7716	2670	3769	2666
7717	284	2655	2659
7718	680	3767	2655
7719	1085	2659	3767
7720	2655	3767	2659
7721	681	3769	3765
7722	1085	3767	3769
7723	680	3765	3767
7724	3769	3767	3765
7725	285	2664	2665
7726	1084	4221	2664
7727	1086	2665	4221
7728	2664	4221	2665
7729	284	2659	2658
7730	1085	4220	2659
7731	1084	2658	4220
7732	2659	4220	2658
7733	286	2671	2670
7734	1086	4222	2671
7735	1085	2670	4222
7736	2671	4222	2670
7737	1084	4220	4221
7738	1085	4222	4220
7739	1086	4221	4222
7740	4220	4222	4221
7741	1091	1092	4226

7742	1091	4226	1
7743	291	2692	4371
7744	291	4371	1091
7745	2692	4256	1092
7746	2692	292	4256
7747	1092	4371	2692
7748	1092	1091	4371
7749	42	1276	4375
7750	42	4375	1274
7751	1276	4268	3158
7752	1276	476	4268
7753	1274	3158	4264
7754	1274	4264	474
7755	3158	4375	1276
7756	3158	1274	4375
7757	1579	4405	4237
7758	4237	4405	98
7759	4405	1582	98
7760	474	4266	4263
7761	4263	4266	1579
7762	4266	3160	1579
7763	3160	4329	4407
7764	4329	1582	4407
7765	4329	738	1582
7766	1582	4405	4407
7767	4405	3160	4407
7768	4405	1579	3160
7769	3158	4268	4475
7770	4268	476	4475
7771	4475	476	3167
7772	3160	4477	4329
7773	4329	4477	3167
7774	4329	3167	738
7775	474	4264	4266
7776	4264	3158	4266
7777	4266	3158	3160
7778	3167	4477	4475
7779	4475	4477	3160
7780	4475	3160	3158
7781	1383	1381	4233
7782	1383	4233	61
7783	583	3468	4390
7784	583	4390	1383
7785	3468	4297	1381
7786	3468	581	4297
7787	1381	4390	3468
7788	1381	1383	4390
7789	226	2309	4443
7790	226	4443	2307
7791	2309	4349	3475
7792	2309	931	4349
7793	2307	3475	4300
7794	2307	4300	583
7795	3475	4443	2309
7796	3475	2307	4443
7797	1986	4246	1983
7798	1986	172	4246
7799	3470	1983	4298
7800	3470	4298	581

7801	931	4432	3470
7802	931	1986	4432
7803	1983	3470	4432
7804	1983	4432	1986
7805	3468	4300	3475
7806	3468	583	4300
7807	3470	3475	4349
7808	3470	4349	931
7809	581	4494	3470
7810	581	3468	4494
7811	3475	3470	4494
7812	3475	4494	3468
7813	1382	4232	1380
7814	1382	61	4232
7815	3464	1380	4295
7816	3464	4295	580
7817	582	4389	3464
7818	582	1382	4389
7819	1380	3464	4389
7820	1380	4389	1382
7821	1704	1701	4242
7822	1704	4242	121
7823	794	3466	4422
7824	794	4422	1704
7825	3466	4296	1701
7826	3466	580	4296
7827	1701	4422	3466
7828	1701	1704	4422
7829	222	4442	2285
7830	222	2283	4442
7831	2283	4299	3473
7832	2283	582	4299
7833	2285	3473	4340
7834	2285	4340	794
7835	3473	2285	4442
7836	3473	4442	2283
7837	580	3466	4492
7838	580	4492	3464
7839	3466	4340	3473
7840	3466	794	4340
7841	3464	3473	4299
7842	3464	4299	582
7843	3473	4492	3466
7844	3473	3464	4492
7845	1286	4228	4378
7846	4228	1289	4378
7847	4228	44	1289
7848	3195	4380	4277
7849	4277	4380	489
7850	4380	1289	489
7851	486	4270	4273
7852	4270	3195	4273
7853	4270	1286	3195
7854	1289	4380	4378
7855	4378	4380	1286
7856	4380	3195	1286
7857	1591	4410	4238
7858	4238	4410	1595
7859	4238	1595	100

7860	486	4275	4271
7861	4271	4275	3197
7862	4271	3197	1591
7863	3197	4332	4412
7864	4332	745	4412
7865	4412	745	1595
7866	1595	4410	4412
7867	4410	1591	4412
7868	4412	1591	3197
7869	3195	4277	4480
7870	4277	3206	4480
7871	4277	489	3206
7872	3197	4482	4332
7873	4332	4482	745
7874	4482	3206	745
7875	486	4273	4275
7876	4273	3197	4275
7877	4273	3195	3197
7878	3206	4482	4480
7879	4480	4482	3195
7880	4482	3197	3195
7881	63	1394	4393
7882	63	4393	1392
7883	1394	4304	3500
7884	1394	594	4304
7885	1392	3500	4303
7886	1392	4303	592
7887	3500	4393	1394
7888	3500	1392	4393
7889	2349	4249	2351
7890	2349	233	4249
7891	3509	2351	4343
7892	3509	4343	812
7893	594	4444	3509
7894	594	2349	4444
7895	2351	3509	4444
7896	2351	4444	2349
7897	1737	1740	4243
7898	1737	4243	127
7899	592	3502	4424
7900	592	4424	1737
7901	3502	4342	1740
7902	3502	812	4342
7903	1740	4424	3502
7904	1740	1737	4424
7905	3509	3500	4304
7906	3509	4304	594
7907	812	4496	3509
7908	812	3502	4496
7909	3502	4303	3500
7910	3502	592	4303
7911	3500	3509	4496
7912	3500	4496	3502
7913	1738	4243	1740
7914	1738	127	4243
7915	3941	1740	4342
7916	3941	4342	812
7917	801	4425	3941
7918	801	1738	4425

7919	1740	3941	4425
7920	1740	4425	1738
7921	2350	2351	4249
7922	2350	4249	233
7923	803	3943	4445
7924	803	4445	2350
7925	3943	4343	2351
7926	3943	812	4343
7927	2351	4445	3943
7928	2351	2350	4445
7929	1384	1380	4232
7930	1384	4232	61
7931	584	3465	4391
7932	584	4391	1384
7933	3465	4295	1380
7934	3465	580	4295
7935	1380	4391	3465
7936	1380	1384	4391
7937	269	2567	4469
7938	269	4469	2565
7939	2567	4341	3478
7940	2567	795	4341
7941	2565	3478	4301
7942	2565	4301	584
7943	3478	4469	2567
7944	3478	2565	4469
7945	1705	4242	1701
7946	1705	121	4242
7947	3467	1701	4296
7948	3467	4296	580
7949	795	4423	3467
7950	795	1705	4423
7951	1701	3467	4423
7952	1701	4423	1705
7953	3465	4301	3478
7954	3465	584	4301
7955	3467	3478	4341
7956	3467	4341	795
7957	580	4493	3467
7958	580	3465	4493
7959	3478	3467	4493
7960	3478	4493	3465
7961	1385	4233	1381
7962	1385	61	4233
7963	3469	1381	4297
7964	3469	4297	581
7965	585	4392	3469
7966	585	1385	4392
7967	1381	3469	4392
7968	1381	4392	1385
7969	1987	1983	4246
7970	1987	4246	172
7971	932	3471	4433
7972	932	4433	1987
7973	3471	4298	1983
7974	3471	581	4298
7975	1983	4433	3471
7976	1983	1987	4433
7977	271	4470	2578

7978	271	2576	4470
7979	2576	4302	3480
7980	2576	585	4302
7981	2578	3480	4350
7982	2578	4350	932
7983	3480	2578	4470
7984	3480	4470	2576
7985	581	3471	4495
7986	581	4495	3469
7987	3471	4350	3480
7988	3471	932	4350
7989	3469	3480	4302
7990	3469	4302	585
7991	3480	4495	3471
7992	3480	3469	4495
7993	1416	4394	4234
7994	4234	4394	1418
7995	4234	1418	67
7996	616	4307	4305
7997	4305	4307	3572
7998	4305	3572	1416
7999	3572	4311	4396
8000	4311	618	4396
8001	4396	618	1418
8002	1418	4394	4396
8003	4394	1416	4396
8004	4396	1416	3572
8005	1791	4244	4426
8006	4244	1794	4426
8007	4244	138	1794
8008	3574	4428	4344
8009	4344	4428	837
8010	4428	1794	837
8011	616	4306	4309
8012	4306	3574	4309
8013	4306	1791	3574
8014	1794	4428	4426
8015	4426	4428	1791
8016	4428	3574	1791
8017	616	4309	4307
8018	4309	3574	4307
8019	4307	3574	3572
8020	3574	4344	4499
8021	4344	837	4499
8022	4499	837	3581
8023	3572	4497	4311
8024	4311	4497	3581
8025	4311	3581	618
8026	3581	4497	4499
8027	4499	4497	3572
8028	4499	3572	3574
8029	1416	4234	4395
8030	4234	1420	4395
8031	4234	67	1420
8032	3573	4397	4312
8033	4312	4397	620
8034	4397	1420	620
8035	616	4305	4308
8036	4305	3573	4308

8037	4305	1416	3573
8038	1420	4397	4395
8039	4395	4397	1416
8040	4397	3573	1416
8041	1791	4427	4244
8042	4244	4427	1795
8043	4244	1795	138
8044	616	4310	4306
8045	4306	4310	3575
8046	4306	3575	1791
8047	3575	4345	4429
8048	4345	838	4429
8049	4429	838	1795
8050	1795	4427	4429
8051	4427	1791	4429
8052	4429	1791	3575
8053	3573	4312	4498
8054	4312	3586	4498
8055	4312	620	3586
8056	3575	4500	4345
8057	4345	4500	838
8058	4500	3586	838
8059	616	4308	4310
8060	4308	3575	4310
8061	4308	3573	3575
8062	3586	4500	4498
8063	4498	4500	3573
8064	4500	3575	3573
8065	1237	4227	1233
8066	1237	33	4227
8067	3055	1233	4259
8068	3055	4259	433
8069	437	4374	3055
8070	437	1237	4374
8071	1233	3055	4374
8072	1233	4374	1237
8073	1827	1823	4245
8074	1827	4245	144
8075	853	3057	4431
8076	853	4431	1827
8077	3057	4260	1823
8078	3057	433	4260
8079	1823	4431	3057
8080	1823	1827	4431
8081	209	4437	2206
8082	209	2204	4437
8083	2204	4262	3066
8084	2204	437	4262
8085	2206	3066	4347
8086	2206	4347	853
8087	3066	2206	4437
8088	3066	4437	2204
8089	433	3057	4474
8090	433	4474	3055
8091	3057	4347	3066
8092	3057	853	4347
8093	3055	3066	4262
8094	3055	4262	437
8095	3066	4474	3057

8096	3066	3055	4474
8097	1443	4235	4398
8098	4235	1441	4398
8099	4235	71	1441
8100	3648	4399	4313
8101	4313	4399	641
8102	4399	1441	641
8103	643	4315	4317
8104	4315	3648	4317
8105	4315	1443	3648
8106	1441	4399	4398
8107	4398	4399	1443
8108	4399	3648	1443
8109	2217	4438	4248
8110	4248	4438	2219
8111	4248	2219	211
8112	643	4319	4316
8113	4316	4319	3655
8114	4316	3655	2217
8115	3655	4348	4440
8116	4348	882	4440
8117	4440	882	2219
8118	2219	4438	4440
8119	4438	2217	4440
8120	4440	2217	3655
8121	3648	4313	4501
8122	4313	641	4501
8123	4501	641	3650
8124	3655	4502	4348
8125	4348	4502	3650
8126	4348	3650	882
8127	643	4317	4319
8128	4317	3648	4319
8129	4319	3648	3655
8130	3650	4502	4501
8131	4501	4502	3655
8132	4501	3655	3648
8133	1443	4400	4235
8134	4235	4400	1442
8135	4235	1442	71
8136	643	4318	4315
8137	4315	4318	3652
8138	4315	3652	1443
8139	3652	4314	4401
8140	4314	642	4401
8141	4401	642	1442
8142	1442	4400	4401
8143	4400	1443	4401
8144	4401	1443	3652
8145	2217	4248	4439
8146	4248	2221	4439
8147	4248	211	2221
8148	3656	4441	4353
8149	4353	4441	1011
8150	4441	2221	1011
8151	643	4316	4320
8152	4316	3656	4320
8153	4316	2217	3656
8154	2221	4441	4439

8155	4439	4441	2217
8156	4441	3656	2217
8157	643	4320	4318
8158	4320	3652	4318
8159	4320	3656	3652
8160	3656	4353	4504
8161	4353	3654	4504
8162	4353	1011	3654
8163	3652	4503	4314
8164	4314	4503	642
8165	4503	3654	642
8166	3654	4503	4504
8167	4504	4503	3656
8168	4503	3652	3656
8169	260	2514	4460
8170	260	4460	2510
8171	2514	4361	3373
8172	2514	1060	4361
8173	2510	3373	4294
8174	2510	4294	549
8175	3373	4460	2514
8176	3373	2510	4460
8177	3369	4294	3373
8178	3369	549	4294
8179	3371	3373	4361
8180	3371	4361	1060
8181	548	4491	3371
8182	548	3369	4491
8183	3373	3371	4491
8184	3373	4491	3369
8185	2539	4465	4255
8186	4255	4465	2535
8187	4255	2535	264
8188	1062	4367	4365
8189	4365	4367	3728
8190	4365	3728	2539
8191	3728	4324	4466
8192	4324	667	4466
8193	4466	667	2535
8194	2535	4465	4466
8195	4465	2539	4466
8196	4466	2539	3728
8197	2515	4254	4461
8198	4254	2511	4461
8199	4254	260	2511
8200	3726	4462	4323
8201	4323	4462	666
8202	4462	2511	666
8203	1062	4364	4366
8204	4364	3726	4366
8205	4364	2515	3726
8206	2511	4462	4461
8207	4461	4462	2515
8208	4462	3726	2515
8209	3728	4507	4324
8210	4324	4507	3724
8211	4324	3724	667
8212	1062	4366	4367
8213	4367	4366	3726

8214	4367	3726	3728
8215	3726	4323	4506
8216	4323	666	4506
8217	4506	666	3724
8218	3724	4507	4506
8219	4507	3728	4506
8220	4506	3728	3726
8221	4254	4463	260
8222	4364	4368	2515
8223	4362	2514	4464
8224	4463	4197	4464
8225	2539	4255	4467
8226	4255	2538	4467
8227	4255	264	2538
8228	4198	4468	4363
8229	4363	4468	1061
8230	4468	2538	1061
8231	1062	4365	4369
8232	4365	4198	4369
8233	4365	2539	4198
8234	2538	4468	4467
8235	4467	4468	2539
8236	4468	4198	2539
8237	4369	4198	4368
8238	4363	1061	4513
8239	4362	4512	4196
8240	4513	4512	4197
8241	42	4376	1277
8242	42	1274	4376
8243	1274	4265	3159
8244	1274	474	4265
8245	1277	3159	4269
8246	1277	4269	477
8247	3159	1277	4376
8248	3159	4376	1274
8249	1579	4237	4406
8250	4237	1583	4406
8251	4237	98	1583
8252	3161	4408	4330
8253	4330	4408	739
8254	4408	1583	739
8255	474	4263	4267
8256	4263	3161	4267
8257	4263	1579	3161
8258	1583	4408	4406
8259	4406	4408	1579
8260	4408	3161	1579
8261	474	4267	4265
8262	4267	3159	4265
8263	4267	3161	3159
8264	3161	4330	4478
8265	4330	3170	4478
8266	4330	739	3170
8267	3159	4476	4269
8268	4269	4476	477
8269	4476	3170	477
8270	3170	4476	4478
8271	4478	4476	3161
8272	4476	3159	3161

8273	1496	1494	4236
8274	1496	4236	80
8275	696	3806	4403
8276	696	4403	1496
8277	3806	4325	1494
8278	3806	694	4325
8279	1494	4403	3806
8280	1494	1496	4403
8281	243	2411	4446
8282	243	4446	2409
8283	2411	4351	3815
8284	2411	938	4351
8285	2409	3815	4327
8286	2409	4327	696
8287	3815	4446	2411
8288	3815	2409	4446
8289	2004	4247	2001
8290	2004	175	4247
8291	3808	2001	4326
8292	3808	4326	694
8293	938	4434	3808
8294	938	2004	4434
8295	2001	3808	4434
8296	2001	4434	2004
8297	3806	4327	3815
8298	3806	696	4327
8299	3808	3815	4351
8300	3808	4351	938
8301	694	4508	3808
8302	694	3806	4508
8303	3815	3808	4508
8304	3815	4508	3806
8305	49	1321	4381
8306	49	4381	1317
8307	1321	4279	3289
8308	1321	521	4279
8309	1317	3289	4278
8310	1317	4278	517
8311	3289	4381	1321
8312	3289	1317	4381
8313	3300	3289	4279
8314	3300	4279	521
8315	979	4483	3300
8316	979	3291	4483
8317	3291	4278	3289
8318	3291	517	4278
8319	3289	3300	4483
8320	3289	4483	3291
8321	247	4447	2436
8322	247	2432	4447
8323	2432	4280	3301
8324	2432	521	4280
8325	2436	3301	4354
8326	2436	4354	1046
8327	3301	2436	4447
8328	3301	4447	2432
8329	519	3297	4484
8330	519	4484	3295
8331	3297	4354	3301

8332	3297	1046	4354
8333	3295	3301	4280
8334	3295	4280	521
8335	3301	4484	3297
8336	3301	3295	4484
8337	1498	4236	1494
8338	1498	80	4236
8339	3807	1494	4325
8340	3807	4325	694
8341	698	4404	3807
8342	698	1498	4404
8343	1494	3807	4404
8344	1494	4404	1498
8345	2005	2001	4247
8346	2005	4247	175
8347	939	3809	4435
8348	939	4435	2005
8349	3809	4326	2001
8350	3809	694	4326
8351	2001	4435	3809
8352	2001	2005	4435
8353	287	4471	2675
8354	287	2673	4471
8355	2673	4328	3820
8356	2673	698	4328
8357	2675	3820	4352
8358	2675	4352	939
8359	3820	2675	4471
8360	3820	4471	2673
8361	694	3809	4509
8362	694	4509	3807
8363	3809	4352	3820
8364	3809	939	4352
8365	3807	3820	4328
8366	3807	4328	698
8367	3820	4509	3809
8368	3820	3807	4509
8369	1090	4226	1092
8370	1090	1	4226
8371	2690	1092	4256
8372	2690	4256	292
8373	290	4370	2690
8374	290	1090	4370
8375	1092	2690	4370
8376	1092	4370	1090
8377	1286	4377	4228
8378	4228	4377	1288
8379	4228	1288	44
8380	486	4272	4270
8381	4270	4272	3194
8382	4270	3194	1286
8383	3194	4276	4379
8384	4276	488	4379
8385	4379	488	1288
8386	1288	4377	4379
8387	4377	1286	4379
8388	4379	1286	3194
8389	1591	4238	4409
8390	4238	1594	4409

8391	4238	100	1594
8392	3196	4411	4331
8393	4331	4411	744
8394	4411	1594	744
8395	486	4271	4274
8396	4271	3196	4274
8397	4271	1591	3196
8398	1594	4411	4409
8399	4409	4411	1591
8400	4411	3196	1591
8401	486	4274	4272
8402	4274	3196	4272
8403	4272	3196	3194
8404	3196	4331	4481
8405	4331	744	4481
8406	4481	744	3203
8407	3194	4479	4276
8408	4276	4479	3203
8409	4276	3203	488
8410	3203	4479	4481
8411	4481	4479	3194
8412	4481	3194	3196
8413	1236	1233	4227
8414	1236	4227	33
8415	436	3054	4373
8416	436	4373	1236
8417	3054	4259	1233
8418	3054	433	4259
8419	1233	4373	3054
8420	1233	1236	4373
8421	208	2200	4436
8422	208	4436	2198
8423	2200	4346	3064
8424	2200	852	4346
8425	2198	3064	4261
8426	2198	4261	436
8427	3064	4436	2200
8428	3064	2198	4436
8429	1826	4245	1823
8430	1826	144	4245
8431	3056	1823	4260
8432	3056	4260	433
8433	852	4430	3056
8434	852	1826	4430
8435	1823	3056	4430
8436	1823	4430	1826
8437	3054	4261	3064
8438	3054	436	4261
8439	3056	3064	4346
8440	3056	4346	852
8441	433	4473	3056
8442	433	3054	4473
8443	3064	3056	4473
8444	3064	4473	3054
8445	8	1117	4372
8446	8	4372	1115
8447	1117	4258	2746
8448	1117	317	4258
8449	1115	2746	4257

8450	1115	4257	315
8451	2746	4372	1117
8452	2746	1115	4372
8453	1640	4241	1642
8454	1640	109	4241
8455	2751	1642	4334
8456	2751	4334	762
8457	317	4419	2751
8458	317	1640	4419
8459	1642	2751	4419
8460	1642	4419	1640
8461	1630	1632	4239
8462	1630	4239	107
8463	315	2747	4413
8464	315	4413	1630
8465	2747	4333	1632
8466	2747	762	4333
8467	1632	4413	2747
8468	1632	1630	4413
8469	2751	2746	4258
8470	2751	4258	317
8471	762	4472	2751
8472	762	2747	4472
8473	2747	4257	2746
8474	2747	315	4257
8475	2746	2751	4472
8476	2746	4472	2747
8477	1336	4231	1338
8478	1336	53	4231
8479	3338	1338	4291
8480	3338	4291	538
8481	536	4387	3338
8482	536	1336	4387
8483	1338	3338	4387
8484	1338	4387	1336
8485	2453	2451	4251
8486	2453	4251	250
8487	767	3347	4450
8488	767	4450	2453
8489	3347	4292	2451
8490	3347	538	4292
8491	2451	4450	3347
8492	2451	2453	4450
8493	109	4420	1644
8494	109	1641	4420
8495	1641	4290	3340
8496	1641	536	4290
8497	1644	3340	4338
8498	1644	4338	767
8499	3340	1644	4420
8500	3340	4420	1641
8501	538	3347	4489
8502	538	4489	3338
8503	3347	4338	3340
8504	3347	767	4338
8505	3338	3340	4290
8506	3338	4290	536
8507	3340	4489	3347
8508	3340	3338	4489

8509	1326	1328	4229
8510	1326	4229	51
8511	526	3311	4382
8512	526	4382	1326
8513	3311	4282	1328
8514	3311	528	4282
8515	1328	4382	3311
8516	1328	1326	4382
8517	107	1633	4414
8518	107	4414	1631
8519	1633	4335	3312
8520	1633	763	4335
8521	1631	3312	4281
8522	1631	4281	526
8523	3312	4414	1633
8524	3312	1631	4414
8525	2452	4250	2450
8526	2452	250	4250
8527	3316	2450	4283
8528	3316	4283	528
8529	763	4449	3316
8530	763	2452	4449
8531	2450	3316	4449
8532	2450	4449	2452
8533	3311	4281	3312
8534	3311	526	4281
8535	3316	3312	4335
8536	3316	4335	763
8537	528	4485	3316
8538	528	3311	4485
8539	3312	3316	4485
8540	3312	4485	3311
8541	1644	1642	4241
8542	1644	4241	109
8543	767	3900	4421
8544	767	4421	1644
8545	3900	4334	1642
8546	3900	762	4334
8547	1642	4421	3900
8548	1642	1644	4421
8549	250	4448	2453
8550	250	2452	4448
8551	2452	4336	3901
8552	2452	763	4336
8553	2453	3901	4339
8554	2453	4339	767
8555	3901	2453	4448
8556	3901	4448	2452
8557	1633	4239	1632
8558	1633	107	4239
8559	3899	1632	4333
8560	3899	4333	762
8561	763	4415	3899
8562	763	1633	4415
8563	1632	3899	4415
8564	1632	4415	1633
8565	3900	4339	3901
8566	3900	767	4339
8567	3899	3901	4336

8568	3899	4336	763
8569	762	3900	4510
8570	762	4510	3899
8571	3901	4510	3900
8572	3901	3899	4510
8573	1340	1338	4231
8574	1340	4231	53
8575	540	3346	4388
8576	540	4388	1340
8577	3346	4291	1338
8578	3346	538	4291
8579	1338	4388	3346
8580	1338	1340	4388
8581	255	2484	4457
8582	255	4457	2480
8583	2484	4357	3353
8584	2484	1052	4357
8585	2480	3353	4293
8586	2480	4293	540
8587	3353	4457	2484
8588	3353	2480	4457
8589	2455	4251	2451
8590	2455	250	4251
8591	3348	2451	4292
8592	3348	4292	538
8593	1052	4453	3348
8594	1052	2455	4453
8595	2451	3348	4453
8596	2451	4453	2455
8597	3346	4293	3353
8598	3346	540	4293
8599	3348	3353	4357
8600	3348	4357	1052
8601	538	4490	3348
8602	538	3346	4490
8603	3353	3348	4490
8604	3353	4490	3346
8605	74	4402	1461
8606	74	1460	4402
8607	1460	4321	3706
8608	1460	660	4321
8609	1461	3706	4322
8610	1461	4322	661
8611	3706	1461	4402
8612	3706	4402	1460
8613	2457	4252	2461
8614	2457	251	4252
8615	3708	2461	4359
8616	3708	4359	1053
8617	660	4455	3708
8618	660	2457	4455
8619	2461	3708	4455
8620	2461	4455	2457
8621	2481	2485	4253
8622	2481	4253	255
8623	661	3710	4458
8624	661	4458	2481
8625	3710	4360	2485
8626	3710	1053	4360

8627	2485	4458	3710
8628	2485	2481	4458
8629	3708	3706	4321
8630	3708	4321	660
8631	1053	3710	4505
8632	1053	4505	3708
8633	3710	4322	3706
8634	3710	661	4322
8635	3706	4505	3710
8636	3706	3708	4505
8637	1329	4229	1328
8638	1329	51	4229
8639	3315	1328	4282
8640	3315	4282	528
8641	529	4383	3315
8642	529	1329	4383
8643	1328	3315	4383
8644	1328	4383	1329
8645	2454	2450	4250
8646	2454	4250	250
8647	1051	3317	4452
8648	1051	4452	2454
8649	3317	4283	2450
8650	3317	528	4283
8651	2450	4452	3317
8652	2450	2454	4452
8653	251	4454	2460
8654	251	2456	4454
8655	2456	4284	3319
8656	2456	529	4284
8657	2460	3319	4355
8658	2460	4355	1051
8659	3319	2460	4454
8660	3319	4454	2456
8661	528	3317	4486
8662	528	4486	3315
8663	3317	4355	3319
8664	3317	1051	4355
8665	3315	3319	4284
8666	3315	4284	529
8667	3319	4486	3317
8668	3319	3315	4486
8669	2484	4253	2485
8670	2484	255	4253
8671	4189	2485	4360
8672	4189	4360	1053
8673	1052	4459	4189
8674	1052	2484	4459
8675	2485	4189	4459
8676	2485	4459	2484
8677	2460	2461	4252
8678	2460	4252	251
8679	1051	4188	4456
8680	1051	4456	2460
8681	4188	4359	2461
8682	4188	1053	4359
8683	2461	4456	4188
8684	2461	2460	4456
8685	250	2455	4451

8686	250	4451	2454
8687	2455	4358	4187
8688	2455	1052	4358
8689	2454	4187	4356
8690	2454	4356	1051
8691	4187	4451	2455
8692	4187	2454	4451
8693	1053	4511	4189
8694	1053	4188	4511
8695	4188	4356	4187
8696	4188	1051	4356
8697	4189	4187	4358
8698	4189	4358	1052
8699	4187	4189	4511
8700	4187	4511	4188
8701	1332	4230	1330
8702	1332	52	4230
8703	3320	1330	4285
8704	3320	4285	530
8705	532	4385	3320
8706	532	1332	4385
8707	1330	3320	4385
8708	1330	4385	1332
8709	1638	1635	4240
8710	1638	4240	108
8711	765	3322	4417
8712	765	4417	1638
8713	3322	4286	1635
8714	3322	530	4286
8715	1635	4417	3322
8716	1635	1638	4417
8717	1330	4230	4384
8718	4230	1333	4384
8719	4230	52	1333
8720	3321	4386	4289
8721	4289	4386	533
8722	4386	1333	533
8723	530	4285	4287
8724	4285	3321	4287
8725	4285	1330	3321
8726	1333	4386	4384
8727	4384	4386	1330
8728	4386	3321	1330
8729	1635	4416	4240
8730	4240	4416	1639
8731	4240	1639	108
8732	530	4288	4286
8733	4286	4288	3323
8734	4286	3323	1635
8735	3323	4337	4418
8736	4337	766	4418
8737	4418	766	1639
8738	1639	4416	4418
8739	4416	1635	4418
8740	4418	1635	3323
8741	3321	4289	4487
8742	4289	3332	4487
8743	4289	533	3332
8744	3323	4488	4337

8745	4337	4488	766
8746	4488	3332	766
8747	530	4287	4288
8748	4287	3323	4288
8749	4287	3321	3323
8750	3332	4488	4487
8751	4487	4488	3321
8752	4488	3323	3321
8753	4254	4517	4463
8754	4254	2515	4517
8755	260	4463	4516
8756	260	4516	2514
8757	4364	4515	4368
8758	4364	1062	4515
8759	2515	4368	4520
8760	2515	4520	4197
8761	4464	4519	4362
8762	4464	4197	4519
8763	2514	4362	4514
8764	2514	4514	1060
8765	4464	4516	4463
8766	4464	2514	4516
8767	4197	4463	4517
8768	4197	4517	2515
8769	4369	4368	4515
8770	4369	4515	1062
8771	4198	4520	4368
8772	4198	4197	4520
8773	4363	4513	4521
8774	4363	4521	4198
8775	1061	4518	4513
8776	1061	4196	4518
8777	4512	4362	4519
8778	4512	4519	4197
8779	4196	4514	4362
8780	4196	1060	4514
8781	4512	4513	4518
8782	4512	4518	4196
8783	4197	4521	4513
8784	4197	4198	4521

End of Connectivity Matrix of Nodes
